# The 2HA line of *Medicago truncatula* has characteristics of an epigenetic mutant that is weakly ethylene insensitive

**DOI:** 10.1186/1471-2229-14-174

**Published:** 2014-06-21

**Authors:** Sergey Kurdyukov, Ulrike Mathesius, Kim E Nolan, Nicolas Goffard, Bernard J Carroll, Ray J Rose

**Affiliations:** 1 School of Environmental & Life Sciences, University of Newcastle, Callaghan, NSW, Australia; 2 Division of Plant Science, Research School of Biology, Australian National University, Canberra, Australia; 3 School of Chemistry & Molecular Biosciences, University of Queensland, Brisbane, Australia; 4 Kolling Institute of Medical Research, Royal North Shore Hospital, St Leonards, NSW, Australia; 5 Enterome Bioscience, Paris, France

**Keywords:** 2HA seed line, Somatic embryogenesis, Ethylene insensitivity, Nodulation, Root growth, *Medicago truncatula*, Epigenetics, DNA methylation, miRNA

## Abstract

**Background:**

The *Medicago truncatula* 2HA seed line is highly embryogenic while the parental line Jemalong rarely produces embryos. The 2HA line was developed from one of the rare Jemalong regenerates and this method for obtaining a highly regenerable genotype in *M. truncatula* is readily reproducible suggesting an epigenetic mechanism. Microarray transcriptomic analysis showed down regulation of an *ETHYLENE INSENSITIVE 3-like* gene in 2HA callus which provided an approach to investigating epigenetic regulation of genes related to ethylene signalling and the 2HA phenotype. Ethylene is involved in many developmental processes including somatic embryogenesis (SE) and is associated with stress responses.

**Results:**

Microarray transcriptomic analysis showed a significant number of up-regulated transcripts in 2HA tissue culture, including nodule and embryo specific genes and transposon-like genes, while only a few genes were down-regulated, including an *EIN3-like* gene we called *MtEIL1*. This reduced expression was associated with ethylene insensitivity of 2HA plants that was further investigated. The weak ethylene insensitivity affected root and nodule development. Sequencing of *MtEIL1* found no difference between 2HA and wild-type plants. DNA methylation analysis of *MtEIL1* revealed significant difference between 2HA and wild-type plants. Tiling arrays demonstrated an elevated level of miRNA in 2HA plants that hybridised to the antisense strand of the *MtEIL1* gene. AFLP-like methylation profiling revealed more differences in DNA methylation between 2HA and wild-type. Segregation analysis demonstrated the recessive nature of the *eil1* phenotype and the dominant nature of the SE trait.

**Conclusions:**

We have demonstrated that *EIL1* of *Medicago truncatula* (*MtEIL1*) is epigenetically silenced in the 2HA seed line. The possible cause is an elevated level of miRNA that targets its 3’UTR and is also associated with DNA methylation of *MtEIL1*. Down regulation of *MtEIL1* makes it possible to form nodules in the presence of ethylene and affects root growth under normal conditions. Segregation analysis showed no association between *MtEIL1* expression and SE in culture but the role and mechanism of ethylene signalling in the process of plant regeneration through SE requires further investigation. The work also suggests that epigenetic changes to a particular gene induced in culture can be fixed in regenerated plants.

## Background

Forward genetics is a way to gain new knowledge about gene function. However, weak phenotypes are often overlooked. The reverse genetics approach is a way to look more closely at possible phenotypes especially if data about mutated genes are available. The *Medicago truncatula* seed line, 2HA differs from its wild type (WT) progenitor line, Jemalong, in its ability to produce greatly increased numbers of somatic embryos in tissue culture [1]. Investigation of gene expression points toward candidate genes that are involved in the process of somatic embryogenesis. The ability to produce somatic embryos is often species- and even cultivar-dependent, and the search for key genes in this process has revealed several genes that stimulate or inhibit SE in different plant species [2-8].

Ethylene is a plant hormone that is involved in different developmental processes: root elongation [9], lateral root emergence [10], nodulation [11,12], senescence [13], fruit ripening [14] and SE [15,16]. The *ETHYLENE INSENSITIVE3* (*EIN3*)-*like* (*EIL*) gene family in Arabidopsis has been well-characterised using mutants and overexpression experiments. Mutations in *EIN3* and *EIL1* genes cause strong ethylene insensitivity, whereas mutations of other *EIL* family members are related to weaker phenotypes, supporting the idea that they are playing an accompanying role during ethylene responses [17]. The triple response test was developed in order to distinguish mutants impaired in response to ethylene. It involves germination of seedlings in the presence of exogenous ethylene or its precursor 1-aminocyclopropane-1-carboxylic acid (ACC). Under these conditions, wild type seedlings display the triple response phenotype of shorter, thicker, and overly-bent hypocotyls. The widely accepted model of ethylene signalling indicates that in the absence of ethylene, EIN3 and other EIL proteins are continuously degraded. The binding of ethylene to its receptor complex leads to stabilisation of these proteins and elicits the downstream ethylene response. EIN3 and EIL1 are transcription factors that bind to promoters of *ERF* genes and regulate their expression. ERFs are also transcription factors, and they in turn promote ethylene responses by binding to promoters of other genes [18].

In this study, genes showing differential expression in microarray studies of 2HA and Jemalong four week old cultures were analysed with about 117 genes up-regulated greater than 1.5 times in 2HA. Only a few genes were down-regulated in 2HA. One of these, an *EIN3-like* gene (*MtEIL1*) showed a large decrease in expression in 2HA plants. Different aspects of ethylene insensitivity of the 2HA line have been analysed, and weak ethylene insensitivity has been confirmed. Analysis of phenotypic segregation, absence of mutations in its DNA and presence of other phenotypes in 2HA prompted us to further investigate epigenetic differences between 2HA and wild type. Tissue culture is known to be able to induce a number of genomic perturbations and epigenetic mutations [19]. We found many examples of differential DNA methylation between 2HA and the WT plants using an arbitrarily-primed, methylation-sensitive PCR profiling method called Amplified Methylation Polymorphism (AMP) [20]. Differences in methylation of the *MtEIL1* gene between the 2HA and WT plants were also found. The methylation of *MtEIL1* in 2HA was associated with increased abundance of a small RNA with homology to the 3’ region of *MtEIL1*. Based on all the data accumulated, we propose that the 2HA seed line is an epigenetic variant of WT Jemalong, and some of its distinctive phenotypes are most likely due to down-regulation of *MtEIL1*. The proposed epigenetic nature of the phenotypes is discussed.

## Results

### Comparison of gene expression between embryogenic and non-embryogenic tissue cultures

**Figure 1:**
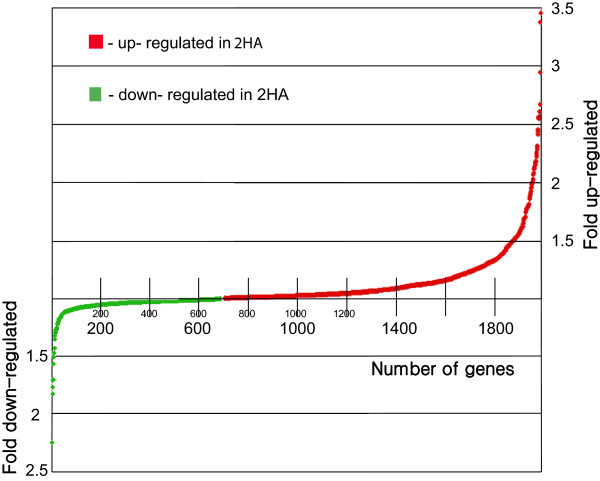
**Graphical presentation of microarray data for 4 week tissue culture of 2HA vs. WT Jemalong.** Transcripts with p < 0.05 were selected and plotted against their fold change value.

In order to characterise the 2HA line more fully, a comparison of gene expression was performed using the Affymetrix Gene Chip. Calli of 2HA and Jemalong were collected after four weeks of culture, shortly before somatic embryos are visible to the naked eye in 2HA. We have previously used transition stage cultures from single 2HA cells [15], and this stage had not been previously examined for 2HA and wild type gene expression comparisons [21]. There were 117 genes up-regulated >1.5 times in 2HA embryonic tissue cultures compared to Jemalong but only seven genes were down-regulated >1.5 times. Thirty four transcripts were up-regulated more than two times in 2HA cultures and only two transcripts down-regulated more than two times. All these differentially expressed genes (with t-test p-value ≤0.05) plotted against their fold change values are shown in Figure 1.

Transcripts that were up- or down-regulated at least 2 times were analysed in more detail (Additional file 1: Table S1 and Additional file 2: Table S2). The annotation and expression of each of these transcripts was checked against available data on the *Medicago truncatula* Gene Expression Atlas (http://mtgea.noble.org/v2/index.php) [22], which shows gene expression in different plant organs and under various experimental conditions. The corresponding TC or EST sequences were retrieved and predicted amino acid sequences from these were used in BlastP searches to find predicted protein domains and matches to other known proteins (Additional file 2: Table S2). Analysis using the *M. truncatula* Gene Expression Atlas indicated that of the 34 transcripts upregulated in 2HA, 18 were classed as legume-specific, 15 showed high expression in nodules with four of these classed as nodule-specific, ten showed high expression in developing seed and, three were predicted transcription factors. Seven predicted transcripts encoded transposase-like proteins containing either a hAT family dimerisation and/or a BED zinc finger domain. The hAT family dimerisation domain is found in transposases belonging to the hAT superfamily [23]. The BED zinc finger domain is a DNA binding domain also found in transposases and in chromatin-boundary-element binding proteins [24].

### Validation of microarray results by RT- qPCR

**Figure 2:**
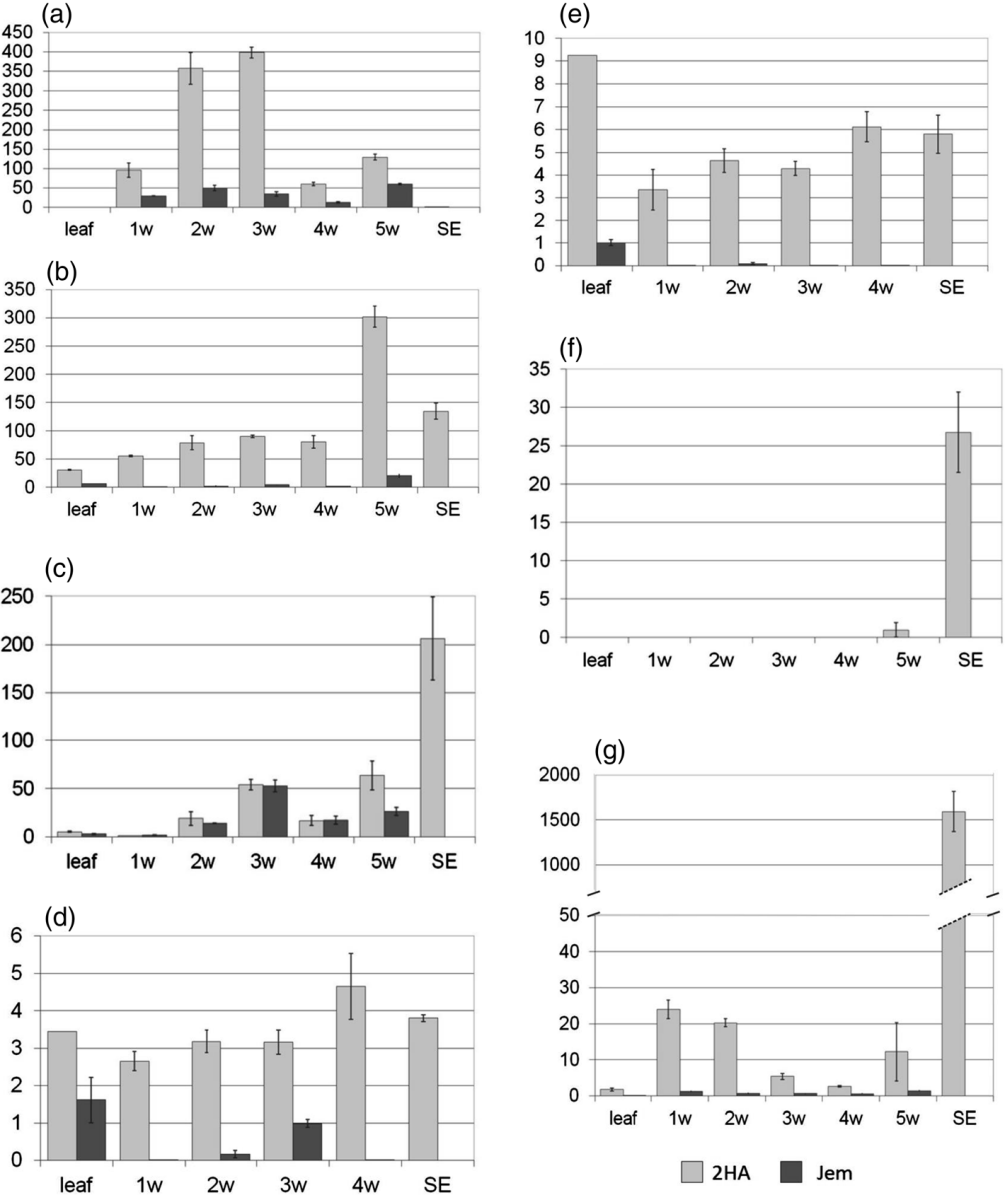
**Time course RT-qPCR data shown as relative expression on the Y axis, for weeks 1–5 for 2HA tissue cultures and for somatic embryos, for seven genes.** These genes were shown to be up-regulated in microarrays of 2HA compared to Jemalong (WT control with no somatic embryogenesis) after 4 week tissue culture. w = weeks, SE = somatic embryos. **(a)** Mtr. 49328.1.S1 **(b)** Mtr.47691.1.S1 **(c)** Mtr.37852.1.S1 **(d)***BH1* Mtr.47631.1 **(e)***BH2* Mtr.10847.1.S1 **(f)** Mtr.10482.1.S1 **(g)** Mtr.35660.1.S1. Results are mean ± SE of 3 biological repeats.

In order to validate the microarray results, we checked expression of seven up-regulated (Figure 2) and two down-regulated predicted transcripts in leaves, callus and SEs by RT- qPCR. Two of the up-regulated 2HA transcripts (Mtr.47631.1 Mtr.10847.1.S1) expressed throughout the culture period are putative transposases. The Mtr. 49328.1.S1 transcript is highly expressed in the 2HA callus phase and Mtr.47691.1.S1is highly expressed in the transition to embryogenesis phase. Both are expressed in nodulation (Additional file 2: Table S2).

Both of the predicted down-regulated transcripts demonstrated nearly identical results in RT- qPCR (probes Mtr.10439.1.S1_at and Mtr.1670.1.S1). PCR amplification with different combinations of primers (F1-F6/R1-R6, Additional file 2: Table S4) followed by sequencing revealed that the two transcripts were part of the same *EIN3-like* gene (Additional file 3: Figure S1) which we called *MtEIL1* and deposited as accession number GQ914771 in NCBI GenBank. This *EIN3-like* gene was also listed as down-regulated in 2 week 2HA embryogenic calli but was not studied in detail [21]. The 2 week stage is an early callus phase when the induction of SE is starting while week 4 is when the transition from callus to embryogenesis is occurring [25,26].

### Analysis of the *EIN3-like* gene *MtEIL1* and its protein

**Figure 3:**
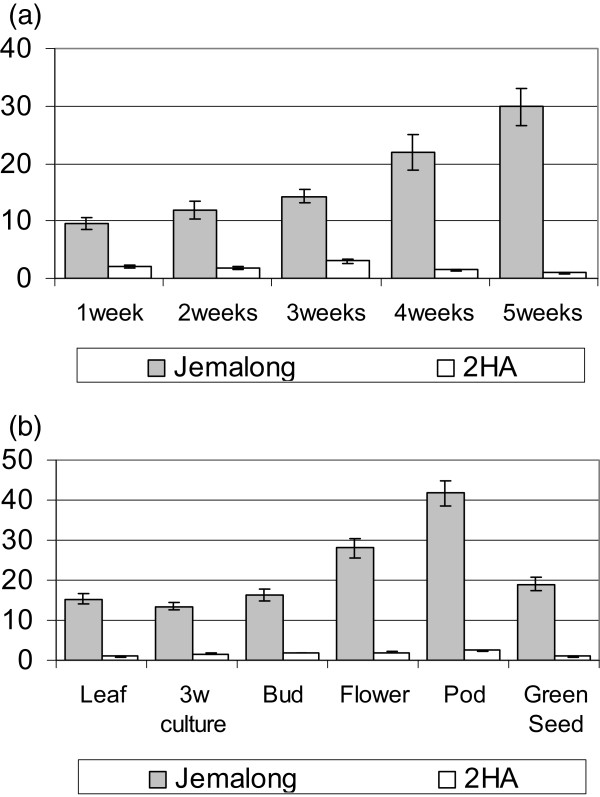
**Time course RT-qPCR analysis for *MtEIL1 *expression in tissue culture (a) and (b) different organs of 2HA and WT Jemalong.** The 3 weeks tissue culture is included in **(a)** and **(b)** for comparative purposes. Results are mean ± SE of 3 biological repeats.

It was of interest that only a single gene was down-regulated more than two times and this gene was involved in ethylene signalling. Ethylene has been shown to be a significant regulator of SE [15,16]. Expression analysis with RT- qPCR for the down-regulated *MtEIL1* transcript was also carried out using material from different organs of 2HA and WT plants (Figures 3a and 3b). Expression of this gene was increased during growth of Jemalong callus but remained low in the case of 2HA (Figure 3a). The difference in expression between these two lines in callus was up to 30 times.

**Figure 4:**
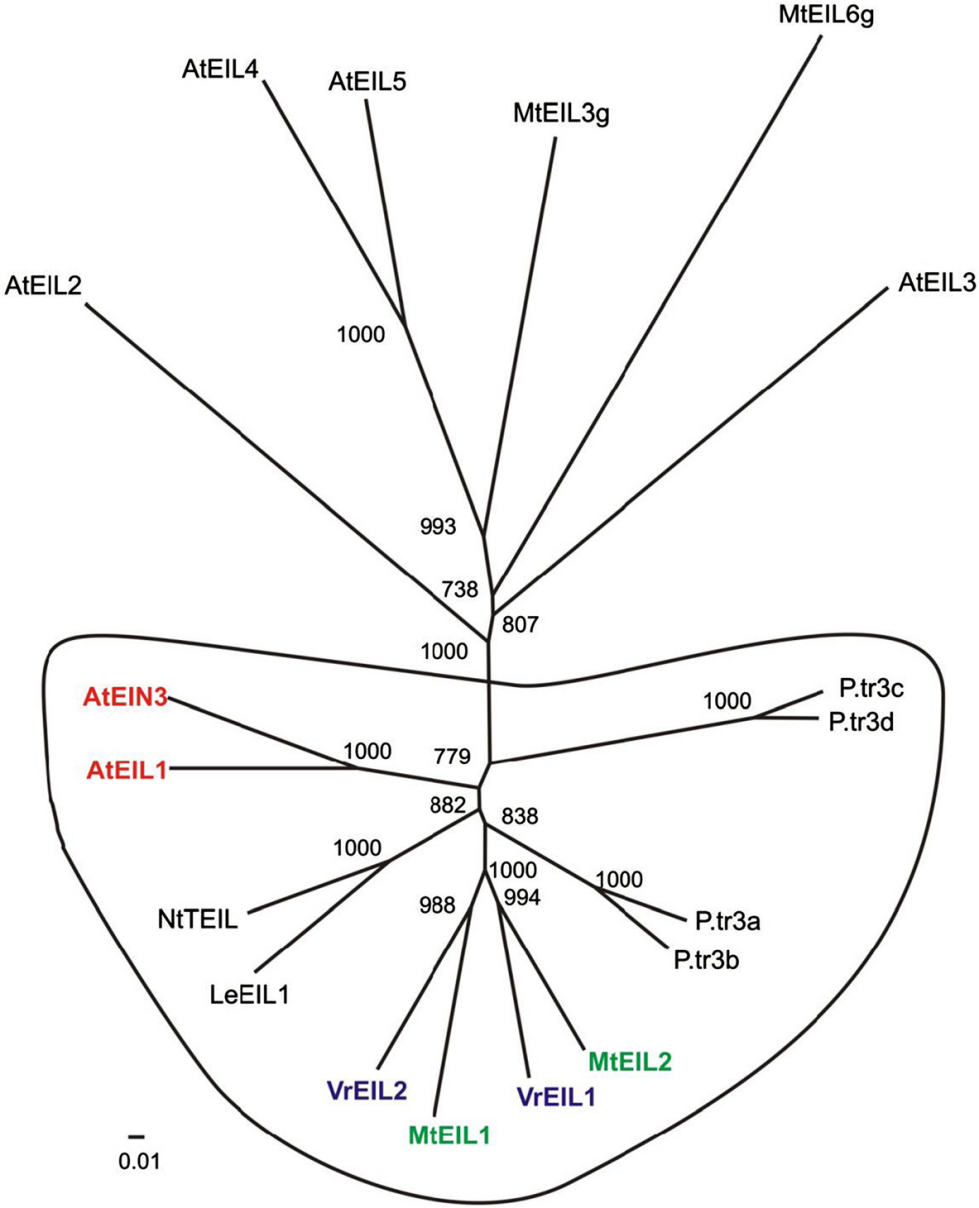
**Phylogenetic analysis of EIL proteins: Mt (*Medicago truncatula *); P.tr (*Populus trichocarpa *- poplar); Le (*Lycopersicum esculentum *- tomato); Nt (*Nicotiana tabacum *- tobacco), At – *Arabidopsis thaliana *, Vr (*Vigna radiata *- mung bean).** Bootstrap values are given (1000 rounds). NCBI protein accession numbers: AtEIN3 - NP_188173; AtEIL1 - NP_180273; AtEIL2 - NP_197611; AtEIL3- NP_177514; AtEIL4 - NP_201315; AtEIL5 - NP_196574; LeEIL1 - NP_001234541; MtEIL1 - ACX54782; MtEIL2 - XP_003617086; MtEIL3g - XP_003601983; MtEIL6g - XP_003619645; NtTEIL - BAA74714; P.tr3a - XP_002312841; P.tr3b - XP_002328098; P.tr3c - XP_002315400; P.tr3d - XP_002310961; VrEIL1 - AAL76272; VrEIL2 - AAL76271.

Additional EIN3-like proteins were predicted by the FGENESH program (http://www.softberry.com) from available *M. truncatula* genomic sequences. Protein alignment and phylogenetic analysis of EIN3-like proteins from *M. truncatula,* and additional proteins from *Arabidopsis thaliana* and some other plant species, was then performed (Additional file 3: Figures S2 and Figure 4). One of the *M. truncatula* EIN3-like proteins, designated MtEIL2 (Figure 4), showed close homology to MtEIL1 and to previously characterised EIL proteins from mung bean [27].

### Weak ethylene insensitivity of the 2HA line

Given the down-regulation of *MtEIL1,* we examined the 2HA line for phenotypes associated with ethylene insensitivity. We also examined *sickle* mutants of *M. truncatula* in parallel to WT and 2HA, as these have very strong ethylene-insensitive phenotypes. The *sickle* mutation in the *EIN2* gene, biochemically, is upstream of *EIL* genes in the ethylene response pathway in *M. truncatula*[28].

**Figure 5:**
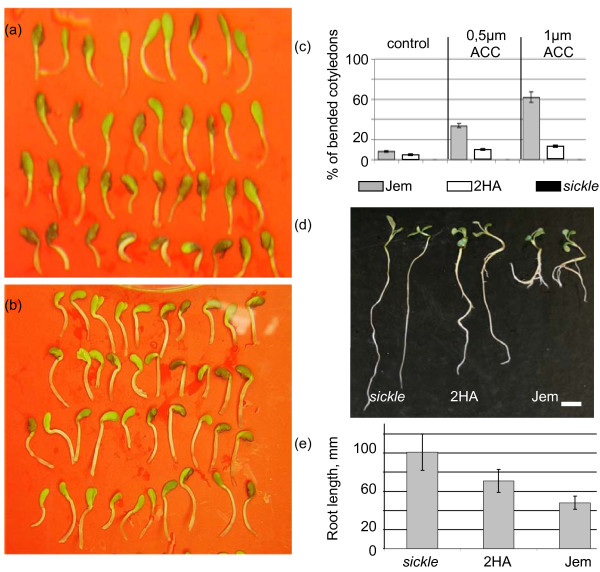
**Ethylene insensitive-related phenotypes of 2HA plants.** Bending of cotyledons on P40 media supplemented with ACC. 2HA plants **(a)** and Jemalong plants **(b)** germinated on P40 media with 0.5 μM ACC. Graph **(c)** represents % of seedlings with bent cotyledons. Cotyledons that formed an angle of < 90° in relation to the stalk are counted as bent. Results are mean ± SE of 3 biological repeats. Bars for *sickle* plants are not shown because they are not bent at any concentration of ACC. **(d)** Root growth on P4 medium. Seven days old seedlings of *sickle* mutant, 2HA line and WT Jemalong are presented in duplicate. Bar is 1 cm. **(e)** Root length (mean ± SE in mm) of seven day old seedlings grown on P4 medium (n = 90).

The ethylene triple response test revealed no significant differences between Jemalong and 2HA at 10 μM of the ethylene precursor ACC. However, at lower levels of ACC (0.5 and 1 μM), the bending of germinated cotyledons was distinctly different between Jemalong and 2HA (Figure 5a-c). Root growth was retarded in a similar way in 2HA and Jemalong even at 0.5 μM ACC. However, roots of seven-day old seedlings grown without addition of ACC were more elongated in 2HA than in WT. 2HA in this case demonstrated an intermediate phenotype between WT and the strong ethylene insensitive mutant *sickle* (Figure 5d-e).

**Figure 6:**
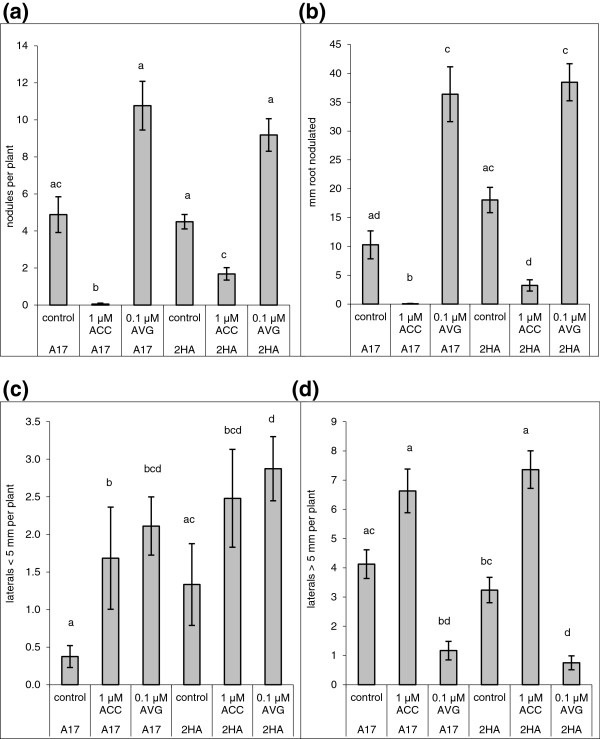
**2HA and WT (A17) plants in the presence of AVG (ethylene inhibitor) or ACC (ethylene precursor) were compared for nodulation (a and b), (c) number of short lateral roots (<5 mm) per plant and (d) number of long lateral roots (>5 mm) per plant.** Bars marked with different letters differ significantly at p < 0.05 (Kruskal-Wallis test). Results are mean ± SE (n = 25).

Because ethylene insensitivity strongly affects nodulation, and *sickle* forms more nodules than the wild type plants [11], we carried out nodulation tests on 2HA plants. 2HA formed similar number of nodules as WT plants in standard conditions. Addition of the ethylene inhibitor aminoethoxyvinylglycine (AVG) caused a two-fold increase in nodulation for both WT and 2HA plants (Figure 6a), and the nodulation zone widened in AVG-treated roots of both lines, although this difference was only significant for the wild type (Figure 6b). However, addition of ACC, which completely inhibited nodule formation in WT (A17 cultivar), did not abolish nodule formation in 2HA plants (Figure 6a). The nodules that formed on ACC-treated 2HA were in a narrow cluster on the root (Figure 6b). Lateral root number was inversely affected by ethylene compared to nodule number. While ACC stimulated the total numbers of lateral roots, AVG did not have a significant effect on total lateral root number (Additional file 3: Figure S3). However, we did observe a change in the proportion of short emerged compared to elongated lateral roots between wild type and 2HA plants. The AVG-treated roots were characterized by increased short lateral roots, whereas ACC increased the number of both short and elongated lateral roots (Figures 6c and 6d). Though the effects of ethylene on lateral root numbers were similar for both plant lines, ACC only caused a significant increase in short lateral root numbers in wild type plants, while the increased caused by ACC in the 2HA mutant was not significant (p < 0.05) (Figure 6c). In contrast, ACC did not cause a significant increase in the number of longer (>5 mm) lateral roots in the wild type, while in 2HA mutants the increase in longer lateral roots was significant (P < 0.05) (Figure 6d). These results suggest that 2HA has altered ethylene sensitivity for both nodule and lateral root formation. For lateral root development, the elongation step seemed to be the stage differentially affected in the 2HA mutant.

Other developmental phenotypes of the 2HA line were consistent with ethylene insensitivity. Delayed senescence of flowers, a very pronounced feature of *sickle*, was regularly observed for 2HA but never for WT Jemalong. It was also evident that calli of 2HA and *sickle* grow better on media supplemented with 1 μM ACC compared to WT calli growth which was negatively affected by this treatment. (Additional file 2: Table S3). All of the phenotypic differences between the 2HA and WT that could be explained by ethylene insensitivity are summarised in Table 1.

**Table 1:** **Ethylene insensitive phenotypes of 2HA line compared to WT Jemalong and strong ethylene insensitive mutant *sickle***

**Phenotype**	***Sickle***	**2HA**	**Jemalong**	**Figure, Table/Ref**
Ethylene triple response tests	**–**	**+/−**	**+**	5 (a,b,c)
Main root length is increased at 7 days after germination	**++**	**+**	**–**	5 (d,e)
Nodulation on 1 μM ACC	+++*	**+**	**–**	6 (a)*
Callus growth on 10 μM ACC	**+++**	**+++**	**++**	Additional file 2: Table S3
Increase of short lateral roots (<5 mm) on 1 μm ACC	**NA**	**-**	**+**	6 (c)
Increase of long lateral roots (>5 mm) on 1 μm ACC	**NA**	**+**	**-**	6 (d)
Recessive mutation	**+**	**+**	**NA**	7

*See Penmetsa and Cook [11] re *sickle* and nodulation.

### Segregation analysis of the 2HA phenotypes

**Figure 7:**
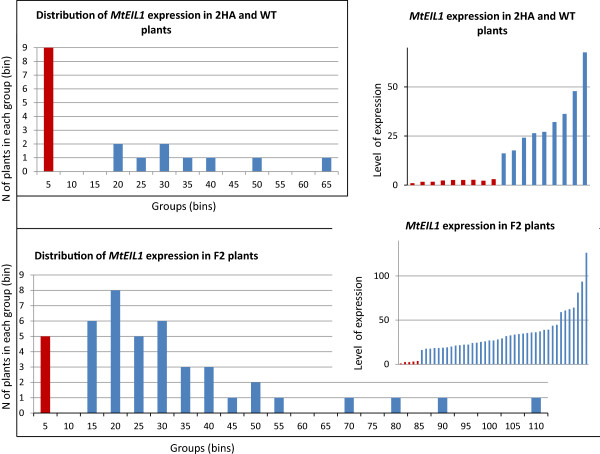
**Level of *MtEIL1 *expression in individual plants measured by RT- qPCR in 2HA (blue bars) and WT Jemalong (red bars) plants and in F2 plants (2HA × Jemalong).** In addition the data have been grouped into bins of 5.

**Figure 8:**
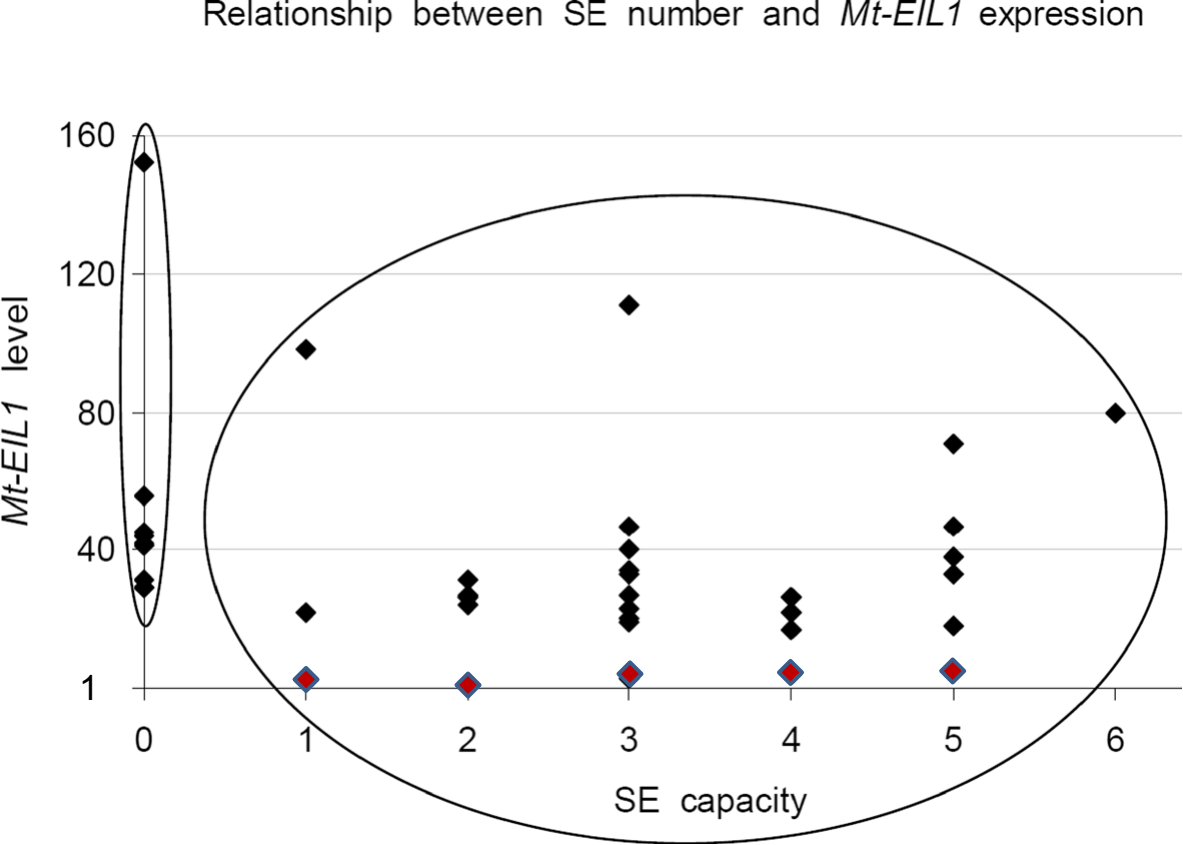
**Segregation analysis for two features of 2HA: SE capacity and level of *MtEIL1 *expression.** Each dot represents one F2 plant. Plants circled on the left have no SEs, plants circled on the right produce varying numbers of SEs with varying *MtEIL1* expression (including *eil1s* that are shown as coloured triangles).

In order to gain an insight into the molecular nature of the 2HA phenotype, we followed *MtEIL1* transcript levels in segregating populations of F2 and F3 plants from a cross between WT Jemalong and 2HA. Forty five and eighty five individual plants were analysed in the F2 and F3 generations, respectively (Figure 7 and Additional file 3: Figure S4). Also, nine plants were assessed for Jemalong and 2HA as controls (Figure 7). It was clear from progeny analysis that the 2HA phenotype was not inherited as a one or two Mendelian gene model, as the segregation in the selfed progeny of F1or F2 heterozygotes was about 1:8 (2HA:WT). At the same time the ability to produce SE is a dominant feature with a segregation ratio of 3:1 (Figure 8). SE segregation data confirms previous data from crosses between non-embryogenic Jemalong and the highly embryogenic 2HA seed line; F1 is dominant for SE and F2 gives a 3:1 ratio [29] using 2HA classes defined by Rose et al. 1999 [1].

### The molecular basis of the 2HA phenotypes

**Figure 9:**
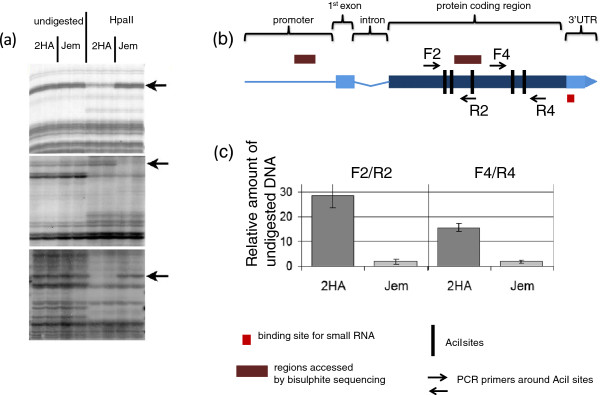
**Analysis of DNA methylation. (a)** Amplified methylation polymorphism (AMP) profiling. DNA from two biological replicates (lanes) was used for AMP before (on the left) or after (right) digestion with HpaII. Arrows point to the differences in methylation between 2HA and Jemalong. **(b, c)** Analysis of *MtEIL1* methylation. **(b)** The *MtEIL1* gene and promoter. Vertical bars depict positions of AciI cut sites, and bisulphate sequenced regions also indicated. After digestion of genomic DNA by AciI, qPCR was performed with primers F2/R2 and F4/R4 separately. **(c)** qPCR results show amount of undigested DNA due to methylation of digestion sites. Results are mean ± SE of 4 repeats.

Initially, we examined large-scale rearrangements in the 2HA karyotype compared to wild type, and no differences were found (Additional file 3: Figure S5). Next, we used AMP, an arbitrarily-primed, methylation-sensitive PCR [20] to assess genome-wide DNA methylation patterns in 2HA and WT (Figure 9a). It was clear that many DNA methylation changes had occurred in 2HA, without detectable genome sequence change. In order to further investigate the molecular basis of ethylene insensitivity, we sequenced the full-length *MtEIL1* gene from two 2HA and two Jemalong plants, and found no nucleotide differences between them. We also sequenced the promoter (2 kb) of this gene, which also revealed no differences between 2HA and Jemalong. As there were no nucleotide mutations in *EIL1* we checked expression of *EIN2, MtEIL2* and a predicted *EIN3*-like gene in cultured WT Jemalong and 2HA. No differences were found between WT Jemalong and 2HA plants (Additional file 3: Figure S6, primer sequences are in Additional file 2: Table S4).

Data from AMP PCR, together with the absence of a nucleotide mutation in *MtEIL1*, prompted a detailed examination of DNA methylation in *MtEIL1*. We compared methylation of *MtEIL1* and its promoter in leaves of 2HA and Jemalong. Genomic DNA was digested by methylation-sensitive endonucleases and used for qPCR with primers surrounding their sites. We found no differences between WT and 2HA in methylation of the promoter of *MtEIL1*. Methylation of 2HA and WT Jemalong coding regions was different, with strong methylation in the case of 2HA but not Jemalong (Figures 9b,c). Bisulphite sequencing using DNA from Jemalong and 2HA plants confirmed methylation profiling demonstrated in the digestion based assay and showed additional methylation in the 2HA coding region but not in Jemalong (Additional file 3: Figure S7).

**Figure 10:**
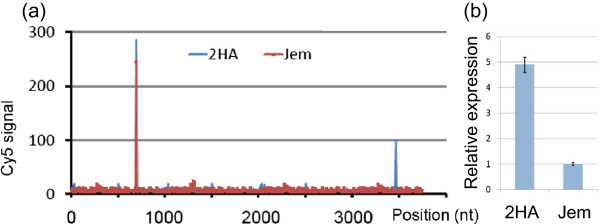
**Small RNA against *MtEIL1. *(a)** 40-nucleotide tiling array hybridised with RNA from two week 2HA and Jemalong cultures. Note increased small RNA Cy5 signal for 2HA at the 3’ end. **(b)** qPCR of a small RNA sequence within the 40 nt sequence showing difference in expression between 2HA and WT Jemalong.

The methylation of coding regions is commonly associated with post-transcriptional gene silencing [30,31], so we carried out investigations to identify any small RNA species that could contribute to the silencing. A 40-nucleotide *MtEIL1* tiling array with 20-nucleotide overlaps was hybridised with small RNA from 2HA and wild type. An antisense small regulatory RNA more abundant in 2HA was identified by the hybridization with the sequence “TTCCCTT*TGGGCCAAAAAGGTGTATTCA*ATTTTCTTCGAC” located at the 3’ end of *MtEIL1* (Figures 9b, Figure 10a, and Additional file 3: Figure S8). Using qPCR and a series of overlapping primers, a putative small regulatory RNA was narrowed down to a shorter region within the 40 nt sequence (in italics), and the difference in expression of the small antisense RNA between 2HA and Jemalong was confirmed (Figure 10b).

## Discussion

### Ethylene insensitivity of 2HA plants

Down-regulation of an *EIN3-like* transcript in 2HA plants was found in all organs checked and led to further investigation of this gene and possible phenotypes caused by the absence of its expression. This transcript, GQ914771, is a homologue of Arabidopsis *EIN3* and we called it *MtEIL1*. The family of *EIL* genes in Arabidopsis is involved in the ethylene response pathway. Among six *EIN3–like* genes in Arabidopsis, only two (*EIN3* and *EIL1)* are crucial for the ethylene response and double *ein3/eil1* mutants demonstrate complete ethylene insensitivity. At the same time, several other mutants of Arabidopsis were found that demonstrated weak ethylene insensitivity [17]. The *EIN3* and *EIN3*-like gene function in Arabidopsis is mainly regulated through the stability of the corresponding proteins [32].

The 2HA line demonstrated weak ethylene insensitivity compared to WT plants (Tables 1 and Additional file 2: Table S3, Figures 5 and 6), most probably due to down-regulation of the *MtEIL1* gene. Most interesting is the ability to form nodules in the presence of an exogenously added ethylene precursor (Figure 6a). The difference between 2HA and WT is not as pronounced in standard growth conditions except for the increased main root elongation of seven day old seedlings. Similar to Arabidopsis ethylene-insensitive mutant nomenclature, we propose that the 2HA seed line has characteristics of a *Medicago eil1* mutant.

Endogenous concentrations of ethylene are low and the whole plant system is well tuned, so small differences (Figure 6) can lead to complete inhibition of nodulation (in the case of 1 μM ACC for WT plants) or to a two times increase in the number of nodules (in the case of 0.1 μM AVG). Ethylene possibly works to control the number of infection sites, so mild attenuation of its action leads to an increased number of nodules without affecting the general system of nodule development [11,28]. The more pronounced widening of the nodulation zone by AVG in wild type compared to 2HA is reminiscent of the inability of AVG to widen the nodulation zone in the ethylene insensitive *sickle* mutant [33]. Weak ethylene-insensitivity could be an advantage for plants as they could form nodules even under stress conditions when the ethylene concentration is elevated. The effects of ethylene on lateral root initiation and emergence was less pronounced, but showed significant relative differences between wild type and 2HA. Differential effects of ethylene on lateral root initiation and emergence have been observed in *Arabidopsis*, where high concentrations of ACC inhibited the initiation of lateral root primordia but promoted their elongation. Low ACC concentration had opposite effects, suggesting a narrow window of optimum ethylene concentration [34]. We found that in wild type plants, a relatively high (1 μM) concentration of ACC caused a significant increase in short lateral roots, but a non-significant increase in longer lateral roots, opposite to the effect in Arabidopsis, suggesting different optimum levels of ethylene between *Medicago* and *Arabidopsis*. Surprisingly, AVG had a similar effect to ACC on short lateral numbers, underlining the importance of ethylene acting in a strict concentration window in the root. The 2HA line showed a different response to ACC compared to the wild type, with significant increase in elongated but not short lateral roots. This could indicate a different optimum window of ethylene in 2HA compared to wild type plants.

### Gene expression differences between embryogenic calli 2HA and non-embryogenic calli WT

Transcription profiling obtained through microarrays revealed a relatively small number of differentially expressed genes. At the same time there was general up-regulation of transcription in 2HA compared to WT (Figure 1) that could have a cumulative effect on cell fate and mediate further developmental changes [35]. Up-regulation of transposon-like genes with BED and hAT domains that we called *BEDHAT1* and *BEDHAT 2* (*BH1* deposited in NCBI GenBank as accession KF679497, putative transposase), found in 2HA culture (Figure 2), could be an indicator of the general “openness” of chromatin in 2HA tissue cultures [36]. Interestingly, *BH2* is active in zygotic embryogenesis and *BH1* is up-regulated during nodule formation and zygotic embryogenesis according to the Medicago Gene Expression Atlas [22]. Earlier in culture in the early callus phase (2 weeks) there were more up-regulated and down regulated genes at the 2 times cut-off, but over 99.5% of probe sets were similar in 2HA and Jemalong. However there were transposases and nodulins up-regulated and the *EIN3-like* gene was down-regulated [21]. It does seem that arrays over weekly periods (as in Figure 2 for individual genes) would provide additional insights.

Among 34 transcripts that showed ≥2 fold up-regulation in 2HA embryogenic 4-week calli we identified several embryo-specific genes, even though their expression is probably limited to a small number of cells. These genes could be good markers for further investigation of SE. Several embryo-specific genes and a number of genes that are preferentially expressed during zygotic embryogenesis as well as during nodule formation (Additional file 1: Tables S1 and Additional file 2: Table S2) were also up-regulated in 2HA tissue cultures or in SE. This suggests a commonality between these three processes: zygotic embryogenesis, somatic embryogenesis and nodule formation. In all these cases, re-differentiation is required and a particular level of pluripotency or “stemness” is necessary.

### Relationship between the SE phenotype of 2HA and *MtEIL1* expression

F2 plants from a cross between 2HA and WT with a high level of *MtEIL1* expression are still able to develop somatic embryos (Figure 8). Therefore, low *MtEIL1*expression does not appear to be an absolute requirement for SE*.* The development of the 2HA seedline involved regeneration of plants from somatic embryos in tissue culture while the segregation of SE and *eil1* traits were limited to tissue culture studies, so the success rate of plant regeneration from somatic embryos is not known. As the original 2HA line has a low level of *MtEIL1* expression, there is still a possibility that modified ethylene signalling is playing a role in the process of 2HA development from somatic embryos and further investigations are required in order to find why these two phenotypes co-exist in the 2HA seed line. An interesting observation in *B. napus* SE [37], is that from four plant lines which demonstrated SE, the most highly embryogenic was the line that also demonstrated delayed senescence of flowers – an ethylene insensitivity phenotype. We have observed that 2HA has delayed senescence of petals. It has also been shown in Arabidopsis that osmotic stress induces ethylene in leaves, without up-regulation of *EIN3*, and meristemoids are induced [38]. Ethylene has been shown to be an activator of the transcription factor MtSERF1 that is essential for SE in *M. truncatula*[15] as well as in soybean and Arabidopsis [16]. As the signalling through MtEIL1 is questionable in 2HA it could occur through the closely related MtEIL2 whose expression is not down-regulated.

### Toward molecular characterisation of the 2HA seed line - the *MtEIL1* gene

The MtEIL1 protein clusters together with MtEIL2 and their closest homologs are two EIL proteins from mung bean (Figure 4). These two proteins are probably equally involved in the ethylene signal transduction pathway as proposed in the original paper [27]. A similar pair is formed by EIN3 and EIL1 from Arabidopsis. In Arabidopsis, EIL1 works co-operatively with, but distinctly from EIN3 to regulate a myriad of ethylene responses [39]. Protein alignment shows that both MtEILs contain the same functional domains that have been previously found (Additional file 3: Figure S2) in mung bean and Arabidopsis [40]. The only difference found between MtEIL1 and MtEIL2 is in the terminal domain where they have nine and six Gln/Asn residues respectively.

The *MtEIL1* gene and its promoter were sequenced but no mutations were found. Digestion with methylation-sensitive restriction endonucleases revealed differences in methylation between 2HA and Jemalong plants for the *MtEIL1* gene. DNA methylation affected the coding region of *MtEIL1* in 2HA but not the promoter. This was associated with increased abundance of a small anti-sense RNA in 2HA as demonstrated by hybridisation to a tiled array of *MtEIL1,* and RT-qPCR confirmed the existence of this small RNA. It is possible then that the driver of *MtEIL1* down regulation is a miRNA that targets the 3’-UTR and causes the down-regulation. To be unequivocal with this latter conclusion an anti-miRNA approach would be required.

### DNA methylation analysis and the epigenetic nature of 2HA phenotypes

Differences in DNA methylation between Jemalong and 2HA genomes were obtained in the AMP experiment (Figure 9a). Even though AMP is able to sample only a small part of the genome, it was a good indicator of widespread differences in DNA methylation between WT and 2HA. At the same time, the AMP profiles were consistent with the isogenicity of 2HA and Jemalong genomes because no differences were found in profiles for undigested DNA. DNA methylation changes as a result of *in vitro* culture are well documented [41], but there is little work on changes in individual genes resulting in a phenotypic change. Though regions that are hypermethylated in 2HA (such as *MtEIL1*) are present, hypomethylated regions in 2HA may be responsible for the up-regulation of transposon-like genes (*BH1* and *BH2*) found in 2HA culture. Global hypomethylation changes in reprogramming to somatic embryogenesis in pollen have been documented [42]. Our observations are consistent with the origin of 2HA by selection from a regenerated plant that passed through a cycle of tissue culture [1,31] and suggests that epigenetic changes in culture directed at specific genes can be fixed in regenerated plants.

Transcriptional gene silencing (TGS) and posttranscriptional gene silencing (PTGS) can interconvert during tissue culture [31]. Their dominance and segregation in progeny are quite different from each other and not simply Mendelian in ratio. Such processes could be responsible for the appearance of the 2HA seed line.

## Conclusions

Microarray-based transcription profiling revealed only one gene that was down-regulated >2 times in the 2HA seed line compared to WT but significantly more (34 genes) were up-regulated. Most of them are embryo and nodule specific genes as well as some transposon-like genes. There were no large scale changes observed in the karyotype. The methylation profiling revealed some differences in DNA methylation between 2HA and WT. Furthermore, hypermethylation of the *MtEIL1* gene is associated with increased abundance of a small antisense RNA which targets the 3’- UTR region and likely acts to posttranscriptionally silence *MtEIL1* expression. *MtEIL1* down-regulation impacts on nodulation, root growth, root branching and other phenotypes, consistent with weak ethylene insensitivity. As signalling through MtEIL1 is questionable it could occur through the closely related MtEIL2 whose expression is not down-regulated. These observations are consistent with the origin of 2HA by selection from a regenerated plant that had passed through a cycle of tissue culture [1,28] suggesting that the epigenetic changes in culture can be fixed in regenerated plants. Finally, segregation analysis indicates that the *eil1* trait and SE are not directly linked.

## Methods

### Plant growth and tissue culture

Plant materials were obtained from glasshouse-grown plants. The glasshouse had a 14 h photoperiod and 23/19°C day/night temperature regime. Jemalong 2HA (2HA) was the highly regenerable seed line [1] and Jemalong and Jemalong A17 (a representative of WT cv Jemalong seed) were WT. The WT Jemalong rarely produce somatic embryos and no embryos were formed in this study. WT Jemalong and WT Jemalong A17 when tested in other studies were interchangeable e.g. Rose et al. [43]. The standard tissue culture procedure was as described [44]. Explants were cultured on P4 10:4 on agar plates for 3 weeks (10 μM NAA, 4 μM BAP) before transfer to P4 10:4:1 (10 μM NAA, 4 μM BAP, 1 μM ABA). Cultures were incubated in the dark. The time course of the developmental sequence of embryogenic calli has been previously documented [25,26]. Dedifferentiation occurs in the first week and callus is visible at the end of week one and in the second, third and fourth weeks callusing continues and the induction processes for SE occur. At week 4 there is the beginning of transition from calli to embryo formation. This is where morphologically the 2HA and WT start to diverge.

### Root growth and the ethylene triple response test

Seeds were soaked in concentrated H_2_SO_4_ for two min, washed in water and sterilised in diluted bleach (0.5% (w/v) of sodium hypochlorite) for 6 min, then washed three times in sterile water and spread on filter paper in Petri dishes with P40 media [45]. Sixty to eighty seeds were used for each genotype. Seven days after germination the root length was measured. Tukey-Kramer tests confirmed (p < 0.05) that all three genotypes (Jemalong, 2HA and *sickle*) were significantly different. For the ethylene triple response measurements were made 4 days after germination. Bending of cotyledons was observed on P40 media and on P40 with 0.5 μm and 1 μm ACC. Cotyledons that formed an angle of < 90° in relation to the stalk were counted as bent. This experiment was repeated three times.

### Nodulation and lateral root assays

Seeds of A17 (WT) and 2HA were scarified on sand paper, sterilized in 6% (w/v) sodium hypochlorite on a shaker for 10 min, washed five times in sterile water and plated on water agar plates. The seeds were left at 4°C for two days and then transferred to 25°C overnight for germination. Seeds were then transferred to Fåhreus medium [46] agar plates, five seedlings per plate, five plates per treatment (i.e. 25 seedlings per treatment). Seedlings were grown in a growth chamber for three days with a day/night cycle of 16 h/8 h, at 25°C with a light intensity of approximately 100 μE. A culture of *Sinorhizobium meliloti* strain 1021 was grown in Bergensen’s Modified Medium [47] in a shaking incubator at 28°C overnight on day 3 and adjusted to an OD_600_ of 0.1. Roots were transferred to Fåhreus medium agar plates containing either 1 μM ACC, 0.1 μM AVG or solvent (methanol at 1 μL/L) as a control on day 3. After a further 24 h incubation in the plant growth chamber to adapt to the altered hormone media, roots were inoculated with 10 μL of a culture of *S. meliloti* at the zone of emerging root hairs (i.e. on day 4 after germination). Plates were returned to the plant growth chamber (same conditions as above) and left to grow for four weeks.

Nodules were counted under a dissection microscope. Lateral root numbers were counted and classified as either of <5 mm or >5 mm length. The root length covered with nodules (first to last nodule along the root) was measured with a ruler. All data were statistically analysed using InStat version 3.06 for Windows (GraphPad Software). As most data were not normally distributed, comparisons were analysed by Kruskall Wallis tests with Dunn’s multiple comparison post tests.

### mRNA microarray analysis

Total RNA was isolated from four weeks culture for three 2HA and three Jemalong samples using the RNAqueous™-4PCR Kit (Ambion, http://www.ambion.com/). Hybridisations were performed according to the Affymetrix manual (Affymetrix, http://www.affymetrix.com/). Data processing was performed as described earlier [21]. Raw data were background corrected, normalized, summarized and log2 transformed using the GCRMA algorithm (ver. 2.2.0) using the *affy* package of the bioconductor software [48] with default parameters. Normalised data are provided in Additional file 1: Table S1. Differentially expressed genes were identified by evaluating the log2 ratio between embryogenic and non-embryogenic tissue culture in combination with a standard *t*-test.

### RT-qPCR

Total RNA was isolated as for microarray analysis. cDNA was synthesised with SuperscriptII (Invitrogen, http://www.invitrogen.com/). PCR master mixes used Platinum Taq polymerase (Invitrogen) with the provided buffer and1.5 μM SYTO9 dye, primers at 0.4 μm and 3 mM dNTPs. A Corbett Rotor-Gene 6000 (Qiagen, http://www.qiagen.com/) machine was used to carry out real-time PCR. The qPCR cycling conditions comprised an initial denaturation at 95°C for 2 min followed by 40 cycles of 95°C for 10 s, 60°C for 30 s and 72°C for 30 s. Dissociation analysis (0.5°C step) was performed in every run and products checked by gel electrophoresis to ensure product uniformity. Analysis of data was performed using Q-Gene software [49]. The *GAPDH* gene was used as a calibrator. *GAPDH* is a suitable reference gene for *M. truncatula* based on geNORM software [50] and our previous microarray and RT- qPCR studies on SE [15]. Three biological repeats were carried out in triplicate. Sequences of primers are listed in Additional file 2: Table S4.

### Amino acid alignment and phylograms

Full length amino acid sequences were aligned using ClustalX 2.0.10 [51]. The phylograms were constructed from aligned sequences using the protein maximum likelihood, proml, programme in PHYLIP [Phylogeny Inference Package Version 3.69; [52] and drawn with Dendroscope [53].

### Karyograms

Actively growing root tips (2 cm) from germinating seed were collected and placed on ice for 22 h. Root tips were fixed for 3 d in 3:1 ethanol:propionic acid in which 0.2% (w:v) ferric chloride hexahydrate was dissolved as a mordent. After fixation root tips were hydrolysed in 1 N HCl at 60°C for 6 min and stained in aceto orcein (1% (w:v) orcein in 45% (v:v) acetic acid) for 5 d. The 1 mm meristematic region was then treated with 1% (w:v) pectinase at 25°C for 30 min then 5 min in 45% (v:v) acetic acid. Squashes were carried out on a slide with 45% (v:v) acetic acid and the coverslip sealed with paraffin wax. Photgraphs were taken using a Zeiss Axiophot microscope and a Zeiss Axiocam HRc digital camera.

### Promoter isolation

Isolation of the *MtEIL1* promoter was performed using a modified gene walking technique (Clontech, http://www.clontech.com) where the first gene-specific primer was biotinylated. After the first round of PCR the resulting product was immobilised on Dynabeads M-280 (Invitrogen), washed with the buffer provided and used for the second round of PCR with nested primers. The sequence of the *MtEIL1* promoter was deposited in NCBI GenBank as accession EU499308 (Additional file 3: Figure S1).

### Methylation analysis

Genomic DNA was isolated from leaves of four Jemalong and four 2HA plants using the CTAB method [54]. The Amplified methylation polymorphism (AMP) protocol for arbitrarily detecting DNA methylation variation in genomic DNA was that described in [20]. To test for differential methylation within *MtEIL1* in 2HA and Jemalong e.g. [55], DNA (10 μg) was digested overnight by AciI (New England Biolabs, http://www.neb.com) and cleaned through a PCR clean up column (Promega, http://www.promega.com). The DNA concentration was measured, adjusted and used for subsequent qPCR with primers that surround the digestion site (primer pairs F2/R2 and F4/R4). Normalisation was carried out using part of the *MtEIL1* gene that does not have a site for these enzymes. Primers F5 and R5 were used and are closely adjacent to the investigated regions F2/R2 and F4/R4. The whole locus that contains all three fragments is shorter than 2 kb, is surrounded by AciI sites and is readily isolated. Undigested DNA was used in parallel and all products were checked on a gel plus melting curve analysis performed in order to demonstrate specificity of the product. Sequences of primers are listed in Additional file 2: Table S4.

For bisulphite sequencing, DNA was prepared using the MethylEasy™ Bisulphite Kit (Human Genetic Signatures, Australia, http://geneticsignatures.com/) from 3 week-old tissue cultures or from leaves in three biological repeats. Part of the *MtEIL1*coding region and promoter were amplified using several pairs of primers (Additional file 2: Table S4). PCR products were directly sequenced and methylation as detected by CpG > TpG conversion of CpG sites confirmed in 2HA, but no methylation was detected in Jemalong. The fragment amplified with bFor2 and bRev2 primers demonstrated the most differences in methylation.

### Analysis of small RNAs

A 40-nucleotide *MtEIL1* tiling array was custom built by LC Sciences (Houston, Texas, USA) with 20-nucleotide steps was hybridised with RNA from 2HA and wild-type callus culture for two weeks. RNA was extracted, labelled with the fluorophore Cy5 and hybridized to the array as previously described [56]. A putative small RNA identified by microarray analysis was amplified by qPCR using an miScript PCR System (Qiagen) with a set of primers (M1 to M7 in Additional file 2: Table S4) that spanned the potential miRNA sequence. Gene specific primers were used together with adaptor primers provided with the miScript PCR System (Qiagen). The M5 primer was used for comparative studies of 2HA and Jemalong RNA isolated from 2 weeks old tissue culture (Figure 10b).

### Availability of supporting data

All data sets supporting this article are available within the article and in additional files. The microarray data are deposited in the NCBI GEO repository with GEO accession number GSE58223 at http://www.ncbi.nlm.nih.gov/geo/query/acc.cgi?acc=GSE58223.

## Competing interests

The authors declare that they have no competing interests.

## Authors’ contributions

SK conducted the experimental work, database mining, and drafted the manuscript. UM conducted lateral root growth and nodulation assays and contributed to the manuscript. KEN conducted some qPCR experiments and analysis, and contributed to the manuscript. MBS performed phylogenetic analysis and discussed results. NG performed bioinformatics related to the microarray experiment. BJC supervised the AMP assay, tiling array, discussed results and contributed to the manuscript. RJR supervised the analysis, discussed the results and critically revised the manuscript. All authors have read and approved the final manuscript.

## Supplementary Material

Additional file 1: Table S1 Microarray analysis of 4 week 2HA tissue culture vs. Jemalong. Click here for file

Additional file 2: Table S2 Transcripts up-regulated more than two times in microarray analysis. **Table S3.** Callus growth in response to ACC and AVG. **Table S4.** Sequences of oligonucleotides used. Click here for file

Additional file 3: Figure S1 *MtEIL1* gene structure. **Figure S2.** Clustal W alignment of EIN3-like proteins. **Figure S3.** Effect of ACC and AVG on total lateral root numbers. **Figure S4.** Level of *MtEIL1* expression in F3 plants. **Figure S5.** Karyotype of 2HA and WT strains Jemalong and A17. **Figure S6.** Expression in leaves and culture of *MtEIN2, MtEIL1, MtEIL2, MtEIL-like*. **Figure S7.** Bisulphite sequencing of fragment of *MtEIL1* coding region. **Figure S8.** Location of predicted miRNA in the 3’ end sequence of the *MtEIL1* gene. Click here for file
