# Supplementary material for: The 2HA line of Medicago truncatula has characteristics of an epigenetic mutant that is weakly ethylene insensitive
Source: BMC Plant Biol. 2014 Jun 21;14:174. doi: 10.1186/1471-2229-14-174 (PMC4082419; doi:10.1186/1471-2229-14-174)
Supplement: Additional file 2: Table S2 — Transcripts up-regulated more than two times in microarray analysis. Table S3. Callus growth in response to ACC and AVG. Table S4. Sequences of oligonucleotides used. [file 1471-2229-14-174-S2.pdf]

SUPPLEMENTARY TABLES 2-4

Supplementary Table 2. Transcripts up-regulated more than 2 times in microarray analysis.

Supplementary Table 3. Callus growth in response to ACC and AVG

Supplementary Table 4. Sequences of oligonucleotides used.

Table S2. Transcripts that up-regulated more than 2 times in 4 weeks tissue culture of 2HA compared to Jemalong.

N	Probe ID	2HA/Jem fold change	Public ID	Annotation or hits on blastp	Expression profile from *Medicago truncatula* Gene Atlas
1	Mtr.47631.1.S1_s_at	3.46	TC108815	hAT dimerisation, putative transposase	Different organs. Increasing during seed development.
2	Mtr.49328.1.S1_at	3.38	AC138451, TC138521		Nodule specific.
3	Mtr.17084.1.S1_at	2.95	AC136840		Hairy roots and cell suspension culture only.
4	Mtr.7138.1.S1_at	2.67	CX549464, AC155100_22.4		Roots and nodules. Increasing during seed development. Cell suspension culture. 1 week 2HA culture on NAA. 2 weeks 2HA culture on NAA+BAP.
5	Mtr.35698.1.S1_at	2.61	TC111259, TC104705		Nodule specific.
6	Mtr.15107.1.S1_at	2.57			Cell suspension culture. 1 week 2HA culture on NAA. 1 week 2HA culture (no hormones). 2 weeks 2HA culture on NAA+BAP.
7	Mtr.25654.1.S1_at	2.57	AC146631, AC135415_17.5, TC134301		Cell suspension culture. 1 week 2HA culture on NAA. 2 weeks 2HA culture on NAA+BAP.
8	Mtr.1666.1.S1_at	2.56	AW694130, TC123760		Varied expression in different organs. Increasing during late stages of seed development. High in the root tip, cell suspension culture, vegetative bud, nodules.
9	Mtr.10847.1.S1_at	2.55	TC108112	BED zinc finger, hAT dimerisation, putative transposase	Varied expression in different organs. Increasing during seed development. Very low in 2weeks Jem tissue culture on NAA+BAP.
10	Mtr.38978.1.S1_s_at	2.46	TC103947	BED zinc finger, putative transposase	Cell suspension culture.
11	Mtr.12107.1.S1_at	2.44	TC112184	IPR000109 domain (TGF-beta receptor, type I/II extracellular region)	Cell suspension culture, hairy roots, 1 week 2HA culture on NAA.
12	Mtr.36518.1.S1_at	2.41	BQ135749	No	Increasing during seed development. High in roots.
13	Mtr.12941.1.S1_at	2.32	TC96464	No	High in roots and nodules. Increasing during seed development. High in cell suspension culture, 1 week 2HA culture on NAA. 2 weeks 2HA culture on NAA+BAP.
14	Mtr.41147.1.S1_at	2.30	TC108815	hAT dimerisation, putative transposase	Increasing during seed development. High in cell suspension culture, 1 week 2HA culture on NAA, 1 week 2HA culture (no hormones), 2 weeks

					2HA culture on NAA+BAP.
15	Mtr.47691.1.S1_at	2.28	NP7255184	No	High in nodules, during seed development and in cell suspension.
16	Mtr.38978.1.S1_at	2.26	TC103947	BED zinc finger, putative transposase	Varied.
17	Mtr.35660.1.S1_at	2.24	TC110477	Non specific lipid transfer protein.	Seed and nodule specific. Increasing during seed development.
18	Mtr.27117.1.S1_at	2.22	AW560293, TC131620	Phospholipase A2	High in nodules, stem and 1 week 2HA culture (no hormones).
19	Mtr.41525.1.S1_at	2.19	TC109573	No	High in nodules and stem
20	Mtr.2633.1.S1_s_at	2.18	BI311333, TC96464	No	Varied. High in nodule, roots, cell suspension.
21	Mtr.29680.1.S1_at	2.18	AJ846639	Peptidase aspartic, Retrotransposon gag protein	Varied. High in the root tip.
22	Mtr.50630.1.S1_x_at	2.16	No	No	High during early stages of seed development and in cell suspension.
23	Mtr.8585.1.S1_at	2.15	TC100726	MtN3/saliva family	High in flowers, leaves and seeds. Very low in 2weeks Jem tissue culture on NAA+BAP.
24	Mtr.2633.1.S1_at	2.13	BI311333	No	Increasing in seed development. High in roots, nodules and in cell suspension.
25	Mtr.37852.1.S1_at	2.12	TC101559	MtN13 protein. Homology to polyketide cyclase / dehydrase and lipid transport.	Nodule specific. High in 1 week 2HA culture on NAA and 2 weeks 2HA culture on NAA+BAP.
26	Mtr.10482.1.S1_at	2.12	TC106915, AC163324_20.4	BURP domain-containing protein/polygalacturonase	Seed specific. Increasing during seed development.
27	Mtr.29875.1.S1_at	2.12	TC134517, AW560633	No	High in nodules and in denodulated roots.
28	Mtr.35697.1.S1_s_at	2.12	TC111246	ubiquitin-protein ligase	High in roost and nodules.
29	Mtr.27780.1.S1_at	2.09	BE999268	unknown protein	High in nodules. Up-regulated in 12 days old seeds. High in 1 week 2HA culture on NAA and 2 weeks 2HA culture on NAA+BAP.
30	Mtr.20232.1.S1_at	2.07	TC121835	Ethylene Responsive Factor (ERF)(	High in stem, nodules and denodulated roots.
31	Mtr.32209.1.S1_at	2.07	AW684842	No	High in 10 days nodules.
32	Mtr.7307.1.S1_at	2.03	TC106108, AC202510_25.3	hAT dimerisation, putative transposase	High during early seed development (10,12 days) and in cell suspension. High in 2 weeks tissue culture.
33	Mtr.13412.1.S1_at	2.02	TC97935, AC146631_18.4	No	Nodule specific. High in 1 week 2HA culture on NAA and 2 weeks 2HA culture on NAA+BAP.

34	Mtr.50913.1.S1_at	2.01	AW586279	No		Flower, pod, root, nodules. High in 1 week 2HA culture on NAA and 2 weeks 2HA culture on NAA+BAP.

Table S3 Callus growth in response to ACC and AVG

Expt 1 - 11 weeks culture		
Callus type	Control	+ACC 10µM
Jem WT	180±10	120±2.5
2HA	140±4	150±8
Expt 2 - 11 weeks culture		
Callus type	Control	+AVG 10µM
Jem WT	230±10	180±7
2HA	160-±6	190±10
Expt 3 - 5 weeks culture		
Callus type	Control	+ACC 10µM
A17 WT	98±7	56±6
Sickle	54 ±3	51±2

Area in mm^2 as estimate of callus size, using the ImageJ program (http://rsb.info.nih.gov/ij). Expt 1 and 2 measurements of 22 calli and Expt 3 measurement of 6 calli. SE indicated.

MtEIL1 gene

QPCR primers for *MtEIL1* gene

Mtr.30770.1.S1_AT(CA922595)For	ATGGATCATCTTCAGAAACAGCCAGA
Mtr.30770.1.S1_AT(CA922595)Rev	ATGATATGATATGGGTGGCCAAAC
Mtr.10439.1.S1(TC106784)For	GGTTCCACCTTCCATTGACATAA
Mtr.10439.1.S1(TC106784)Rev	GTGATTTTCCGTTGCAGCTAAGT

Primers for *MtEIL1* promoter trapping

EIN3bio	TGAAAAAGGGAGTTTAGTTTAGAGTT
rev3PrTr	GGCTGTCCTAGGTGAATGTGAAGAG

Primers for amplification of *MtEIL1* promoter for sequencing

F1p	GCTTGACCACATTTTAAAGATACTAGACTA
F2p	CTAGACTAGAAGCGAAATAATCCATTAAC
F3p	GCCATGTAGATTAAATTAATACATCGTCA
F4p	CGTCAGAGTCAATTCTACTGTAAGAAC
F5p	TGTGGTTACTAATATAATATTAAATTAGT
F6p	GGGTCTATTCTTTAAGACCAACAC

Primers for amplification of *MtEIL1* gene for sequencing

F1	GGTTTACCCCACCAATAATAACC
F2	GAACTGGTGAACAGTGATAGTTG
F3	GAAGTACATGCTGAAAATGATGGAG
F4	AGAAGGCCTGGAAGGTTGGAGTTCT
F5	GGCGAGTTTAACATGATGGATCC
F6	GGTCCCACCTCCCATTGACATAA
R1	CGGTAATATCCCCTTCAACTTG
R2	CATTCGCGGAGATTATCCGATG
R3	TTTGTCTTGAAGGCATTTGGATTGC
R4	GAAGCCCTGTCTGGAAAACCGAT
R5	GTGATTTTCCGTTGCAACTAAGT
R6	ATGATATGATATGGGTGGCCAAAC

Primers for *MtEIL1* promoter methylation analysis

Met2for	CAATGATATGACGATTTACATGGGTACG
Met2rev	CGATTAAAGATTGATCGAGACCCTCAA

Primers for amplification of *MtEIL1* promoter after bisulfite treatment

FOR13	GTAAATATTTTGGAGAAATTTTAATTATGA
REV13	ATCAATTAATATTATCCATAAATACTTACT

Primers for amplification of *MtEIL1* gene after bisulfite treatment

biFor2_EIN3C	ATGATGATGATGTTTGATCATGAGATG
biFor2_EIN3T	ATGATGATGATGTTTGATTATGAGATG
biRev2_EIN3A	GCCTTACAAACCTCCATCATTTTCA
biRev2_EIN3G	GCCTTGCAAACCTCCATCATTTTCA
bFor2	TGGAGGTTTGTAAGGTTTAAGGGTTTGTTTA
bRev2	ACCAATAAAATTACCTCCATCATTCTTCCC

QPCR primers for *MtEIN2* gene

EIN2For	CTTGCTATCCTCTTGGACATTGAGA
EIN2Rev	ACTGGAACTTCGGATTGGATGA

QPCR primers for *MtEIL* genes Fig.S4

For (MtEIL2)	CAGAAAATGATCAGCGACCTTATGT
Rev (*MtEIL2*)	ACTACACAATTGGTGGAACTTGCAT

QPCRF (*MtEIL-like*)	AAATTATCGGAGGAGGCATGAATTA
QPCRR (*MtEIL-like*)	GTTACTCAACGGTGATCCAAACCTA

QPCR primers for genes upregulated in 2HA tissue culture compare to Jem tissue culture at four weeks

Mtr.49328.1.S1_at For	GAGTCTTATGAAGAAGAGGAGCCATTG
Mtr.49328.1.S1_at Rev	GTCACTGCTTTTGACTCCATCCTTC
Mtr.47691.1.S1_at For	ATTTGTTTAGGTTCCCTTATGATTGTT
Mtr.47691.1.S1_at Rev	GTCACAAATTCATCATCACTTGCAT
Mtr.35660.1.S1_at For	GCTGCTTACAGGAGAATCAACCAAG
Mtr.35660.1.S1_at Rev	CTAGCACAATTGAAGGAAGCACTGA
Mtr.37852.1.S1_at For	TCATCGAGAAAGCAGAAACTCTTGA
Mtr.37852.1.S1_at Rev	ATGTGTTGCTTCGTAGACCCAAAAT
Mtr.10482.1.S1_at For	CTCTGACCTATTGCTACCTTATGGCA
Mtr.10482.1.S1_at Rev	TCTTTCCAGGGTAGAGGTCATGTTC
Mtr.47631.1.S1_s_at For (*BH1*)	ATGGCGGAAGCTTTGATTTGTACT
Mtr.47631.1.S1_s_at Rev *(BH1*)	GACTTGTCCAATTTCTGCCACAAC
Mtr.10847.1.S1_at For (*BH2*)	GTTCCGAACAGAAATGCAAGGTTA
Mtr.10847.1.S1_at Rev (*BH2*)	TCCATCACCCTTCCACCATAGTTA

QPCR primers for miRNA against Mt-EIL1

M1	CATGTCGAAGAAAATTGAATACA
M2	GTCGAAGAAAATTGAATACACCTT
M3	GAAATTGAATACACCTTTTTGG
M4	AAATTGAATACACCTTTTTGGC
M5	TGAATACACCTTTTTGGCCCA
M6	TACACCTTTTTGGCCCAAAG
M7	ACCTTTTTGGCCCAAAGGGA
