# Supplementary material for: The 2HA line of Medicago truncatula has characteristics of an epigenetic mutant that is weakly ethylene insensitive
Source: BMC Plant Biol. 2014 Jun 21;14:174. doi: 10.1186/1471-2229-14-174 (PMC4082419; doi:10.1186/1471-2229-14-174)
Supplement: Additional file 3: Figure S1 — MtEIL1 gene structure. Figure S2. Clustal W alignment of EIN3-like proteins. Figure S3. Effect of ACC and AVG on total lateral root numbers. Figure S4. Level of MtEIL1 expression in F3 plants. Figure S5. Karyotype of 2HA and WT strains Jemalong and A17. Figure S6. Expression in leaves and culture of MtEIN2, MtEIL1, MtEIL2, MtEIL-like. Figure S7. Bisulphite sequencing of fragment of MtEIL1 coding region. Figure S8. Location of predicted miRNA in the 3’ end sequence of the MtEIL1 gene. [file 1471-2229-14-174-S3.pdf]

SUPPLEMENTARY FIGURES

Supplementary Figure 1. *MtEIL1* gene structure

Supplementary Figure 2. Clustal W alignment of EIN3-like proteins. P.tr – *Populus trichocarpa* (poplar), Le – *Lycopersicum esculentum* (tomato), Nt – *Nicotiana tabacum*, (tobacco), At – *Arabidopsis thaliana*, Vr – *Vigna radiata* (mung bean). TC128021 corresponds to the MtEIL1 protein (ACX54782), AC124972 corresponds to MtEIL2 (acc. XP_003617086) ; AC125389 (MtEIL3g XP_003601983) ; AC144619 (MtEIL6g XP_003619645) , AtEIN3 (NP_188173) ; AtEIL1 (NP_180273) ; AtEIL2 (NP_197611) ; AtEIL3 (NP_177514) ; AtEIL4 (NP_201315) ; AtEIL5 (NP_196574) ; LeEIL1 (NP_001234541) ; NtTEIL (BAA74714) ; P.tr3a (XP_002312841) ; P.tr3b (XP_002328098) ; P.tr3c (XP_002315400) ; P.tr3d (XP_002310961) ; VrEIL1 (AAL76272) ; VrEIL2 (AAL76271).

Supplementary Figure 3. Effect of ACC and AVG on total lateral root numbers per plant for WT (A17) and 2HA. Bars marked with different letters differ significantly at $p<0.05$ (Kruskal-Wallis test). Results are mean ± SE (n=25).

Supplementary Figure 4. Level of *MtEIL1* expression in F3 plants. (a) 85 plants were used for analysis: all descendants of one F2 (heterozygote) plant. RNAs were extracted from 4week old calli. (b) Graph is an illustration of distribution of the *MtEIL1* signal. Values from 85 plants were grouped with an interval of 5 units per group.

Supplementary Figure 5. Karyotype of 2HA and WT strains Jemalong and A17.

Supplementary Figure 6. Expression in leaves and cultured tissue of *MtEIN2, MtEIL2, MtEIL-like* (NCBI protein XM_003597428) in 2HA and WT Jemalong contrasting with *MtEIL1,* using RT-qPCR. SE indicated.

Supplementary Figure 7. Bisulphite sequencing in coding region between AciI sites using R2 and F2 primers (Fig 11), showing methylation in 2HA but not Jemalong *MtEil1*.

Supplementary Figure 8. The 3' end sequence for the *MtEIL1* gene. **TGA** – stop codon, **AATAAAA** – polyadenylation signal. Predicted binding site (from miRNA hybridisation array) is underlined. Precise location of predicted miRNA obtained after qPCR experiments is highlighted in blue.

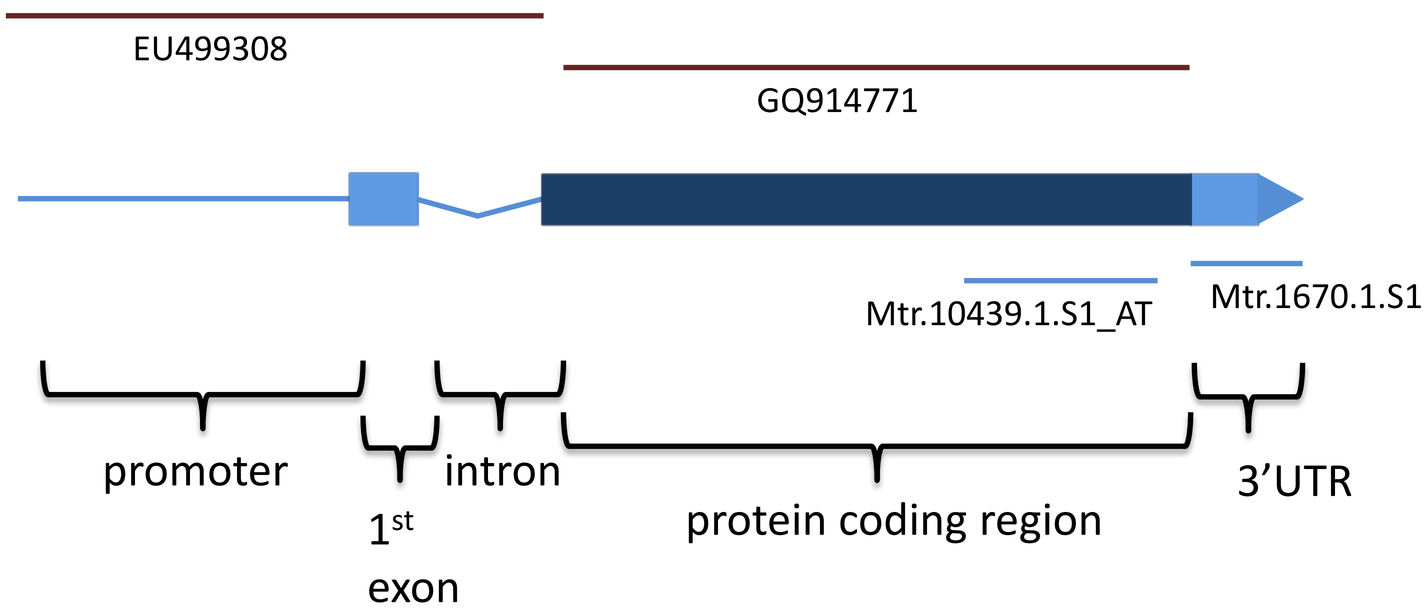
EU499308
GQ914771
Mtr.10439.1.S1_AT
Mtr.1670.1.S1
promoter
1st exon
intron
protein coding region
3'UTR

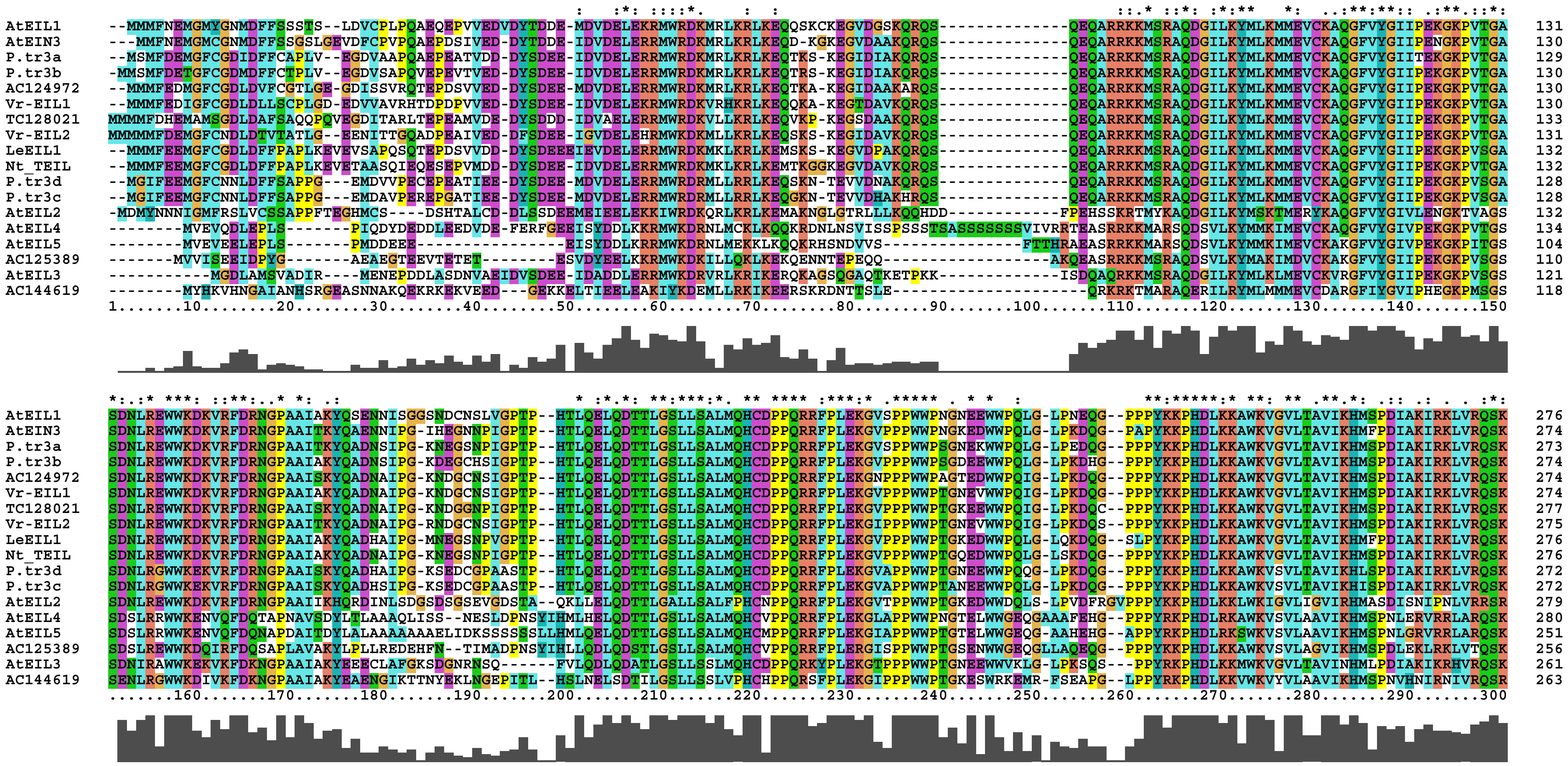
AtEIL1
AtEIN3
P.tr3a
P.tr3b
AC124972
Vr-EIL1
TC128021
Vr-EIL2
LeEIL1
Nt_TEIL
P.tr3d
P.tr3c
AtEIL2
AtEIL4
AtEIL5
AC125389
AtEIL3
AC144619

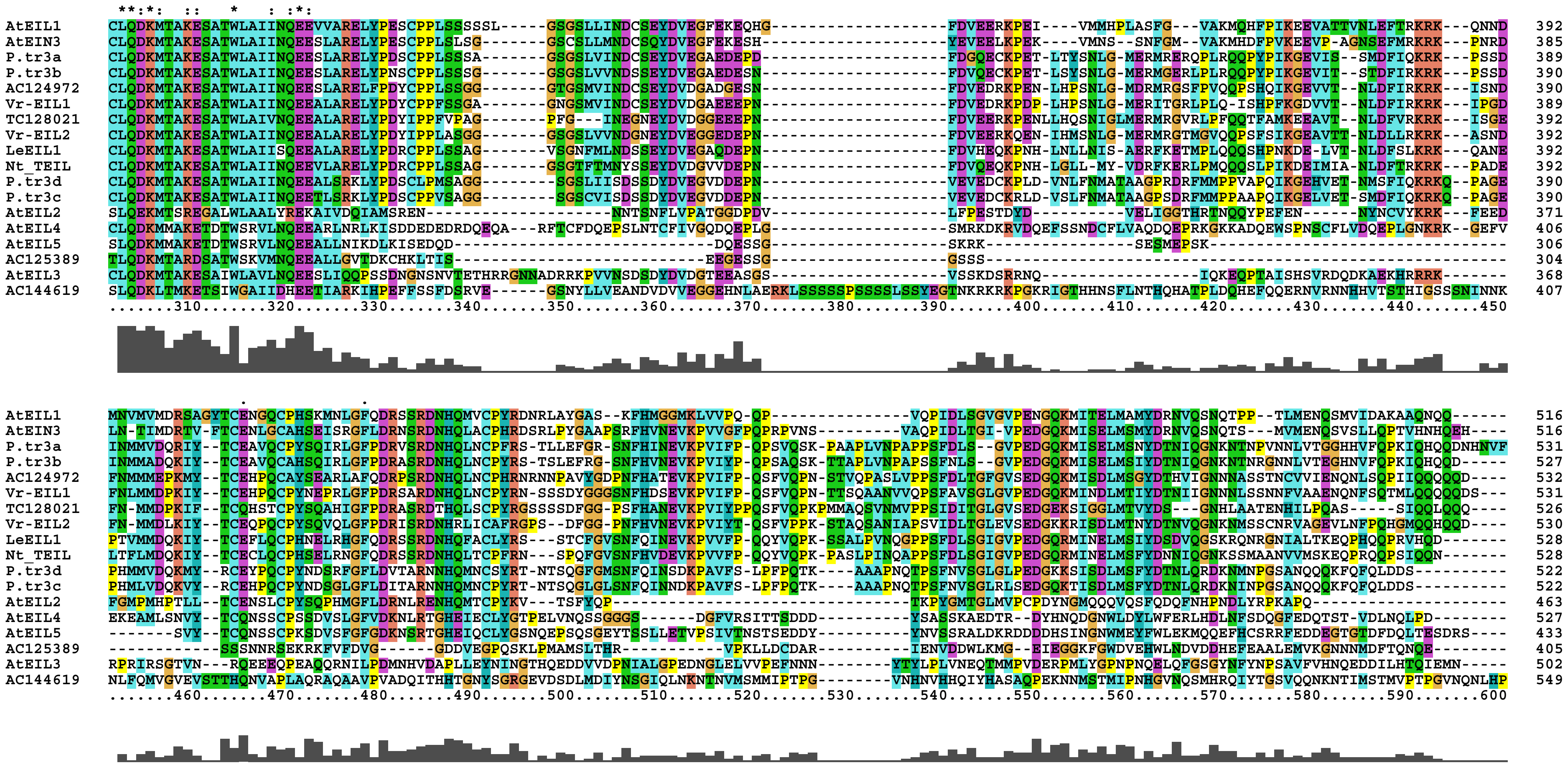
AtEIL1
AtEIN3
P.tr3a
P.tr3b
AC124972
Vr-EIL1
TC128021
Vr-EIL2
LeEIL1
Nt_TEIL
P.tr3d
P.tr3c
AtEIL2
AtEIL4
AtEIL5
AC125389
AtEIL3
AC144619

```
                                                                                                                                          :
AtEIL1    -----------LNFN----------------------SGNQMFMQQG-----------------------TNNGVNNR------FQMVFDS-TPFDMAAFDYRDDWQTGAMEGMGKQQQQQQQQQDVSIWF--   584
AtEIN3    -----------LQFPGNMVEGSFFEDLNIPNRANNNNSSNNQTFFQGNNNNNNVFKFDTADHNNFEAAHNNNNNSSGNR------FQLVFDS-TPFDMASFDYRDDMSMPGVVGT--MDGMQQKQQDVSIWF--   628
P.tr3a    QPKIQHQQGNHFRSQGNVIDGNVFKESNINSN--HQLFSQEGGQFDR----------------FKPLNSPFETSQNNSS-----FNLMFSS--PLDLSSFEYKED-----LQGLG-MDSLPKHQQDVSIWF--   631
P.tr3b    ---------NHFRSQSNMINGSIFEGSNINQN--HQMFSQEGGQFDR----------------FKPLNSPFETNQNNSG-----FNLMFGS--PFDLSSFDYKDD-----LQVLG-MDTLPKH-QDVSTWF--   617
AC124972  ---------NFFLNQGMVMEAN-------------FFTREENQFDR----------------FKAMNSPFEANLNNNN---NMHPMYGS--SCDLASFDFKEDL---QLQGGVGMDALYKQP-DVSIWNY-   615
Vr-EIL1   ------RRDNYFTGQGMMTEGN-------------FFAREEGQFER----------------FKAMN--MNAPFDTN-------HMLFSP--QCDLSSFDFKD------IQGG-GMDTGHRQQQEVSIWFE-   609
TC128021  ---------NYFRGQGMVMEGNMFEATNMSNNNHHHMFARDEGQFDQR----------------FKALNSPFENNHNHNQNHNNNFHLMFGSPPHCDLTSYEFKGD-----MHGVGIMDHLQKQPDISSVWYQ-   629
Vr-EIL2   ---------NFIRGRGITMEGNVFDEATMSNN--HHTFARDECQFDR----------------FKALNSPFETNHNN----NNNFHSMFGS--FCDLASFDFKED-----MQGVG-MDALQKQTDFS-VWYQ-   622
LeEIL1    ---------NYLLSQG-IMDGNIFKNTNISTT--QSMLPQVD-PFDQ----------------SK----AFNAGSNDN------FHFMFGS--PFNIQSTNYNGN-----LPSIG-YDTTPKQ--DAPIWY--   610
Nt_TEIL   ---------NYLHNQGIILDGNIFGDTNISAN--HSMFPQGD-RFDQ----------------SKVLTSPFNAGSNDN------FHFMFGS--PFNLQSTDYTEA-----LSGIT-QDNMPKQ--DVPVWY--   615
P.tr3d    -----------FYGQGAIMGNNITEVTSMPVN--SSAFPSTEMQFDH----------------CKAFDSAFDANVNDN-----VADFRFGS--PFTMPPVDYSMD------------PMPKQ--DAGMWYV-   603
P.tr3c    -----------FYGQGAMVGNNITEATSMPVN--NPVFSSTENQFDH----------------CKAFDSAFDTNVNDN-----ITDFRFGS--PFPSPPVDYSMD------------LIQKQ--DVGMWYV-   603
AtEIL2    ------------RGNDDLVEDLN--------------------------------------------PSPSTLNQN------LGLVLPT---------DFNGG-----EETVGTENNLHNQGQELPTSWIQ   518
AtEIL4    -------------------------------------------------------------------HSDSNQTMNED------------------DISLWDMGCED---------KDIYMSQD---------   557
AtEIL5    ---------------------------------------DNVNLNQL-------------------TKSDRSDNVNRS------------------AFSVWDMGCED---------KDIYMFD----------   471
AC125389  -----------------------------------------------------------------------HNLHGQEE-----------------EKSIWDFRYQ-------------YPSQD---------   428
AtEIL3    -----------------------------------TQAPPHNSGFEEAP----------------GGVLQPLGLLGNEDG--------VTGS------ELPQYQSG----ILSPLTDLDFDYGGFGDDFSWFGA   567
AC144619  QVYTSSVHQQQKSNTMMNTSMMSNSMPVMNMVATPVFNQNMQHQMDQN----------------FYAQQGGADSYYNYKVRNAEVANVPMQAN--VSTTSFDPTFEHLKAFNSQFHVDAYNNSLASSSYDWNQ-   664
          .......610.......620.......630.......640.......650.......660.......670.......680.......690.......700.......710.......720.......730....
```

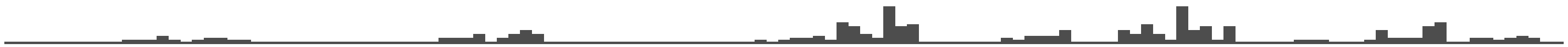

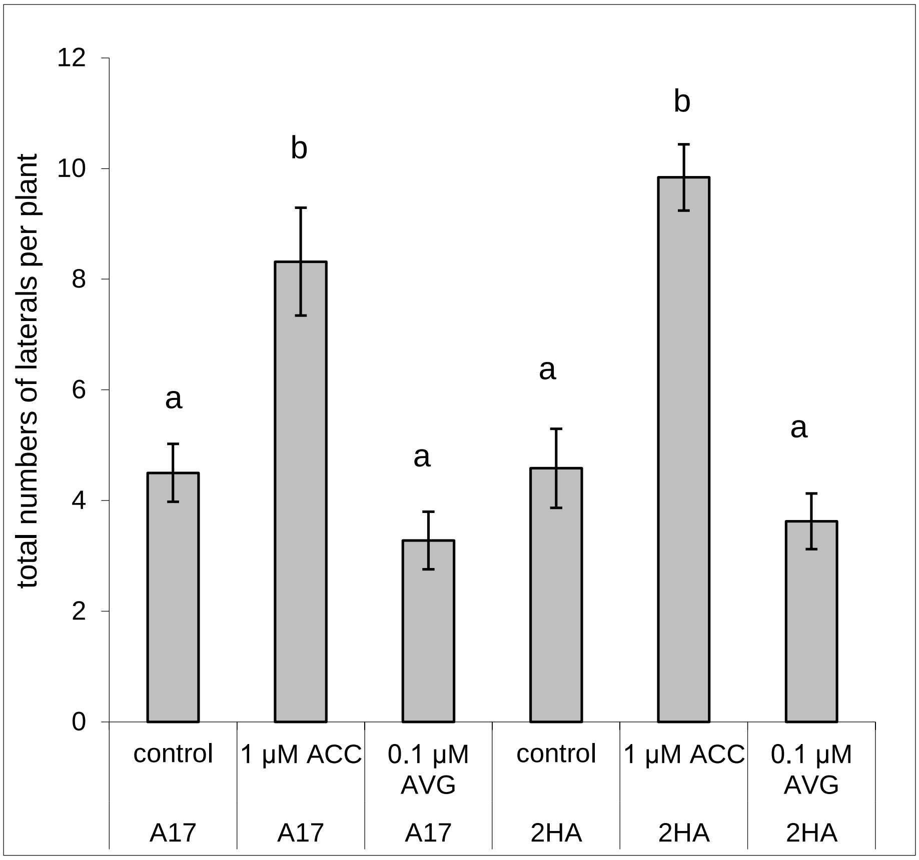

Supplementary Figure 3. Effect of ACC and AVG on total lateral root numbers per plant for WT (A17) and 2HA. Bars marked with different letters differ significantly at $p<0.05$ (Kruskal-Wallis test). Results are mean ± SE (n=25).

(a)

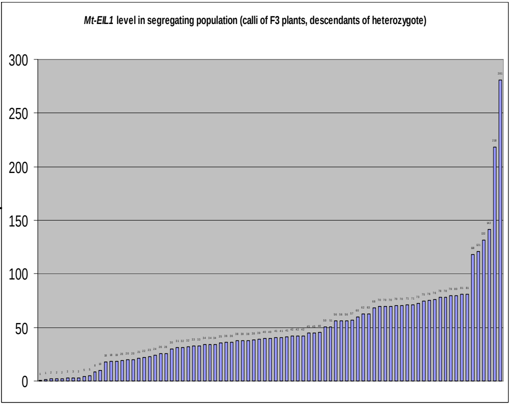
Mt-EIL1 level in segregating population (calli of F3 plants, descendants of heterozygote)
Level of expression
300
250
200
150
100
50
0

(b)

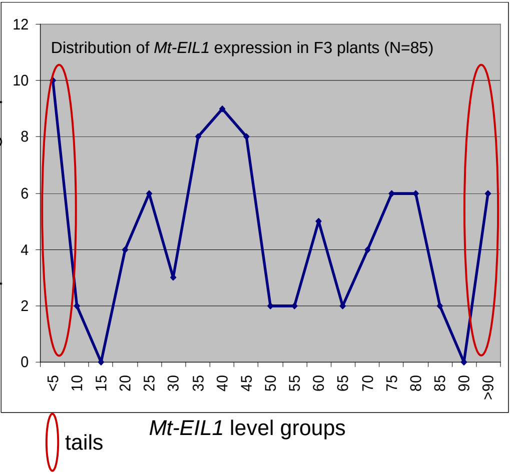
Distribution of *Mt-EIL1* expression in F3 plants (N=85)
Number of plants in each group
12
10
8
6
4
2
0
<5
10
15
20
25
30
35
40
45
50
55
60
65
70
75
80
85
90
>90
Mt-EIL1 level groups
tails

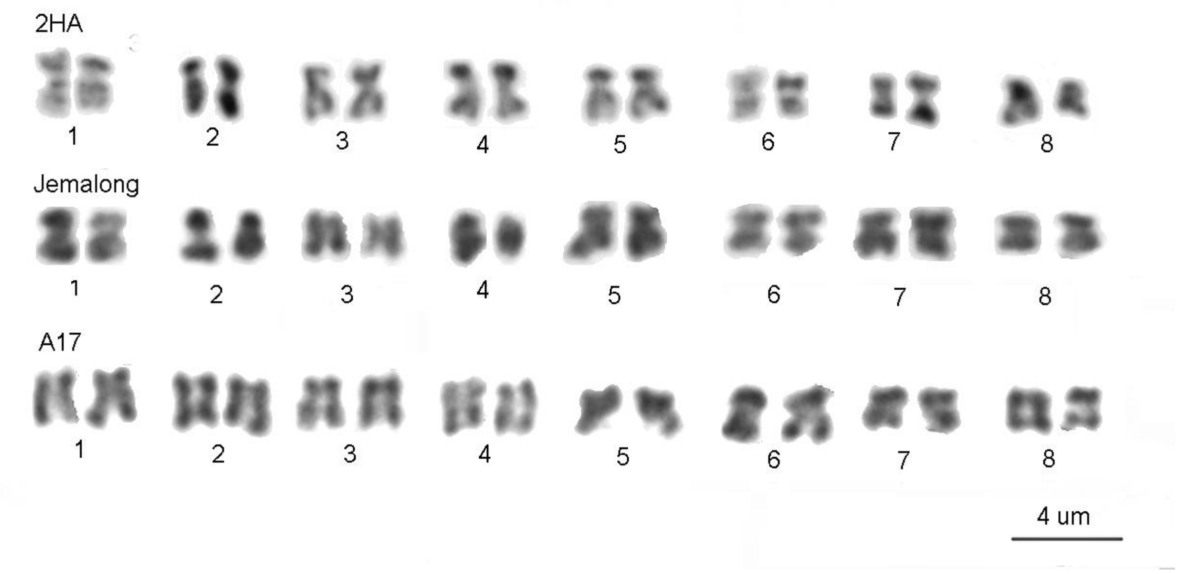

Supplementary Figure 5. Karyotype of 2HA and WT strains Jemalong and A17.

MtEIN2

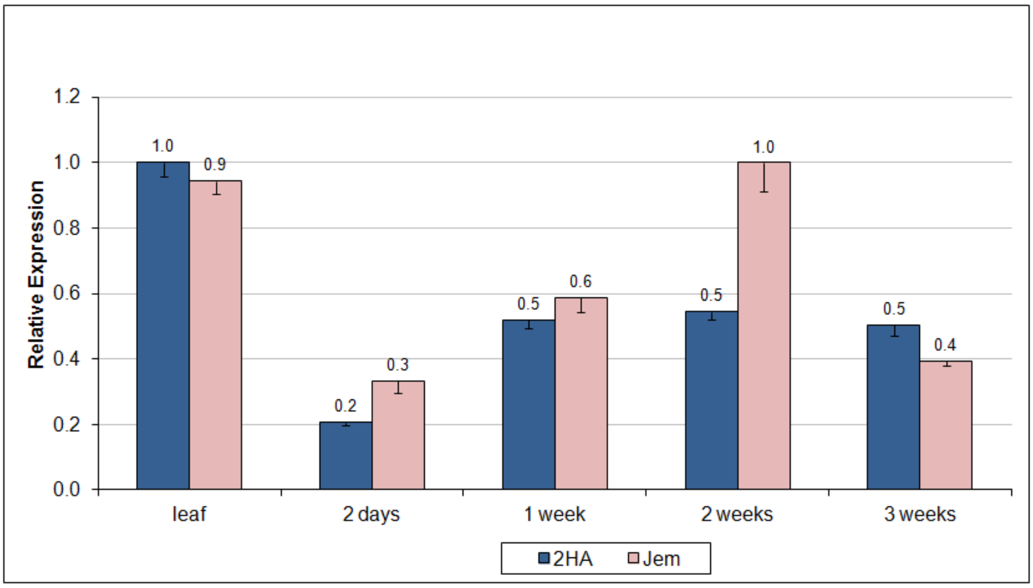

MtEIL2

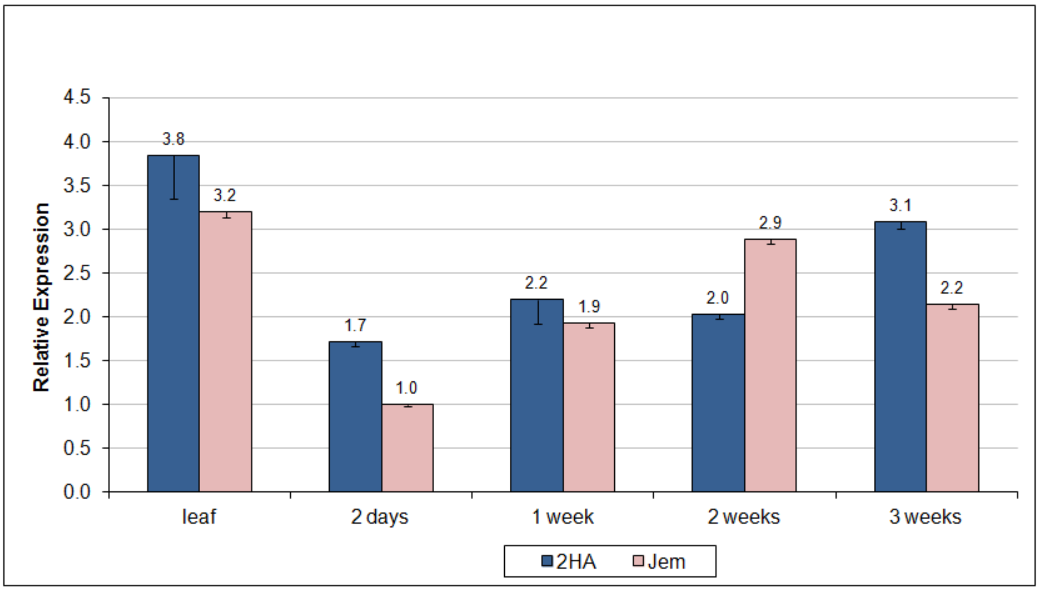

MtEIL-like

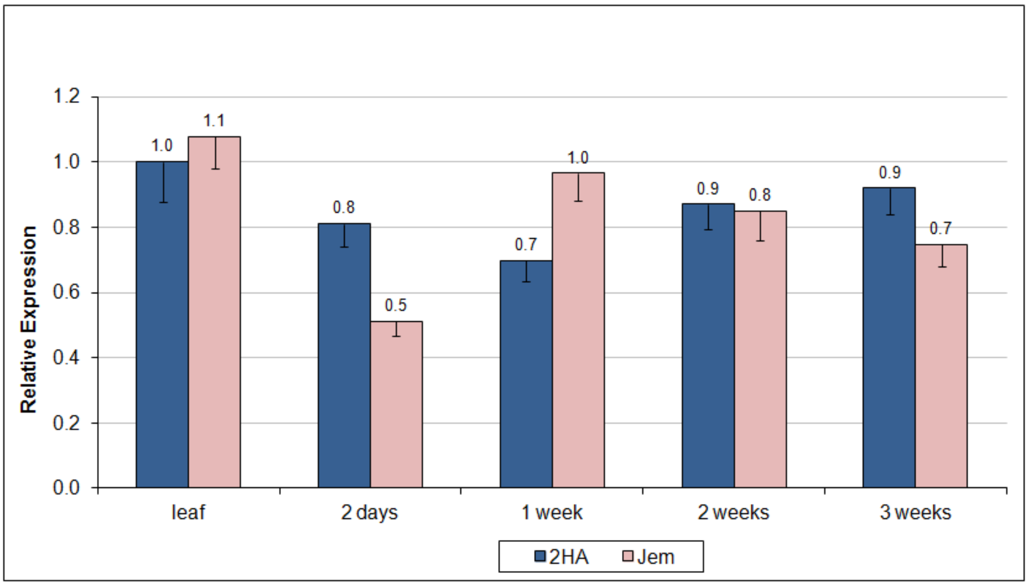

MtEIL1

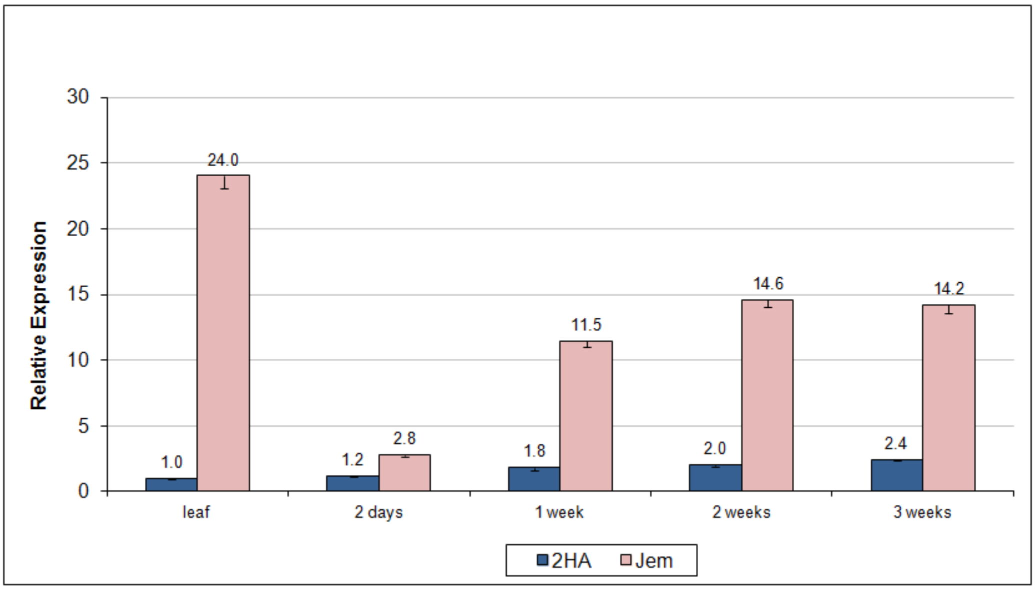

Supplementary Figure 6. Expression in leaves and cultured tissue of *MtEIN2*, *MtEIL2, MtEIL-like* (NCBI protein XM_003597428) in 2HA and WT Jemalong contrasting with *MtEIL1*, using RT-qPCR. SE indicated.

Jemalong

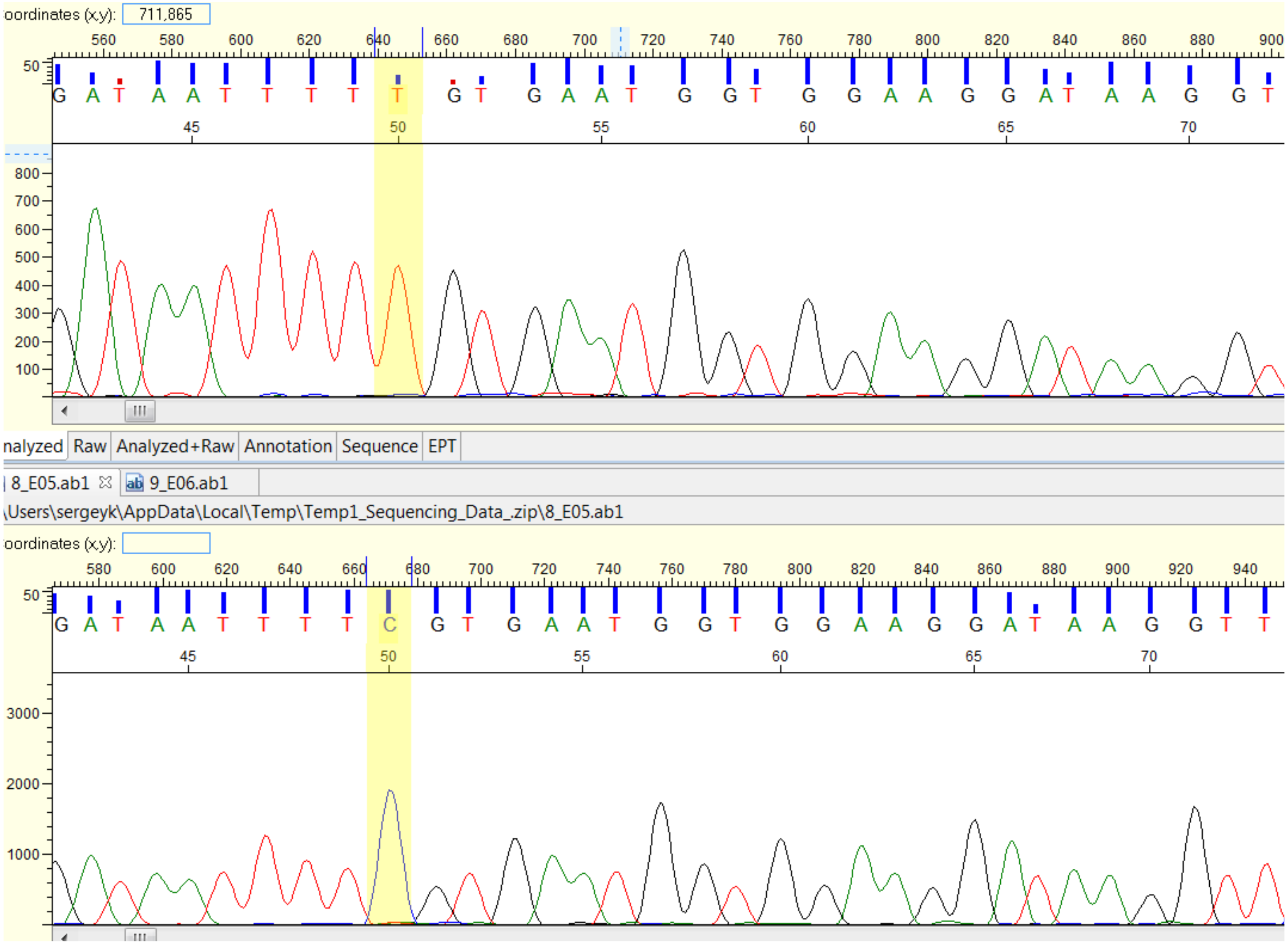

2HA

Jemalong

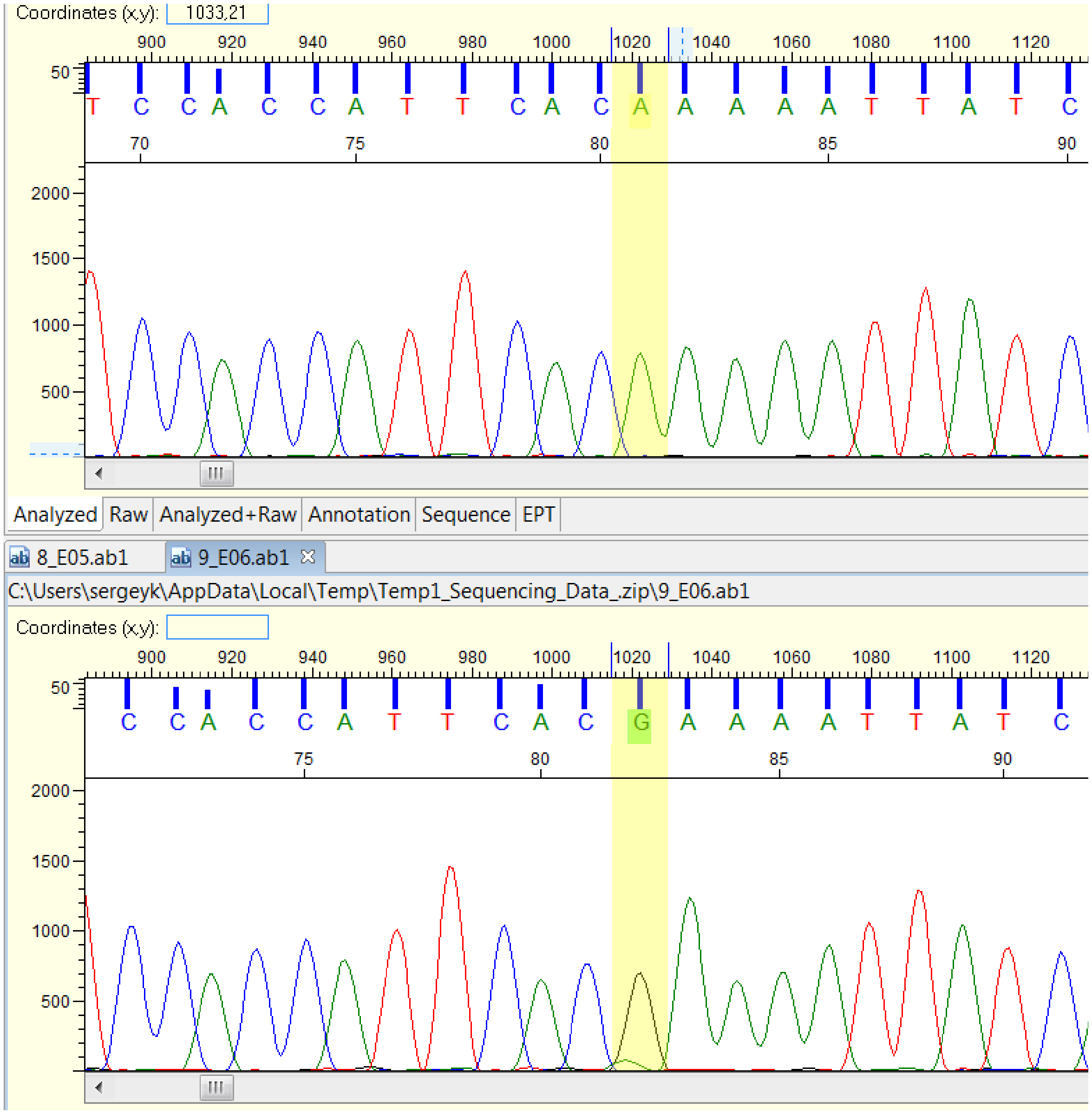

2HA

Jemalong

2HA

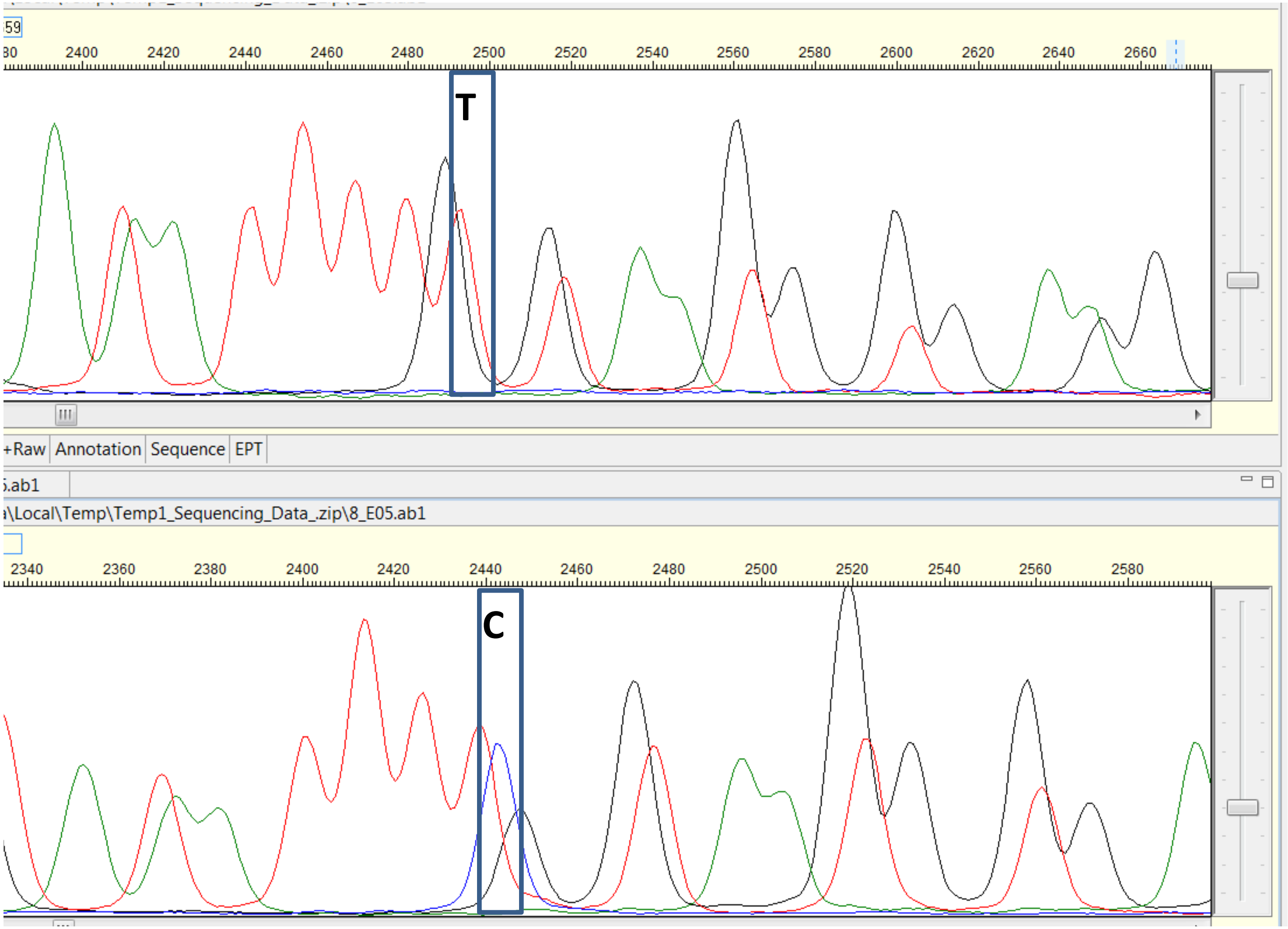

Supplementary Figure 8

ATGGTACCAG**TGA**ATTTAACAACTGGAATCTTCATACTCTTTTATTTATCTTGAACTCTTTTTATTTTCTTTCAAAGTTTGGCCACCCATATCATATC ATGTGAAGT

TTCCCTTTGGGCCAAAAAGGTGTATTCAATTTTCTTCGACATGTAATCCTGTAGTAGTCCTAGAAAAACTAGATTTTATTTTTATGCTTGTCCTTAG GTATGATAT

GGGGTTATAATAATAATAAAAGCTTGTTTTGTTTAGTCTATGT

Predicted small RNA sequence (ein3_w40_3461_as) after microarray hybridisation.

5’-GTCGAAGAAAATTGAATACACCTTTTTGGCCCAAAGGGAA-3’

Putative small RNA sequence (predicted after qPCR experiments).

5’-TGAATACACCTTTTTGGCCCA-3’

The 3’ end sequence for MtEIL1 gene. **TGA** – stop codon, AATAAAA – polyadenylation signal. Predicted binding site (from miRNA hybridisation array) is underlined. Precise location of predicted miRNA obtained after qPCR experiments (using primers M1-M7 in Supplementary Table 4)is highlighted in blue.
