# Supplementary material for: The 2HA line of Medicago truncatula has characteristics of an epigenetic mutant that is weakly ethylene insensitive
Source: BMC Plant Biol. 2014 Jun 21;14:174. doi: 10.1186/1471-2229-14-174 (PMC4082419; doi:10.1186/1471-2229-14-174)
Supplement: Additional file 1: Table S1 — Microarray analysis of 4 week 2HA tissue culture vs. Jemalong. [file 1471-2229-14-174-S1.pdf]

Supplementary Table 1. Microarray analysis of 4 week 2HA tissue culture vs. Jemalong. Microarray expression ratios for the *M. truncatula* embryogenic (2HA) and non-embryogenic WT Jemalong (J) tissue culture. All quantitative data are expressed as log2 (embryogenic:non-embryogenic) expression ratio

IDENTIFIER	af1c703-21-J1-	af1c703-22-J	af1c703-23-J	af1c703-24-H	af1c703-25-H	af1c703-26-H	avg(J)	stdev(J)	avg(2HA)	stdev(2HA)	2HA/J	log2(2HA/H)	ttest(2HA/J)
Mtr.39963.1.S1_at	11.06	3.63	9.26	10.23	10.09	10.27	7.98	3.88	10.20	0.09	1.28	0.353	3.8E-01
Mtr.20438.1.S1_at	12.20	4.53	8.15	10.20	7.94	10.15	8.29	3.84	9.43	1.29	1.14	0.186	6.5E-01
Mtr.24735.1.S1_at	2.92	9.52	3.27	3.27	4.09	3.26	5.24	3.71	3.54	0.47	0.68	-0.566	4.8E-01
Mtr.13960.1.S1_at	9.85	3.71	10.28	10.87	9.75	10.83	7.94	3.67	10.48	0.63	1.32	0.400	3.0E-01
Mtr.833.1.S1_at	2.63	8.91	2.61	2.67	2.58	2.66	4.71	3.63	2.64	0.05	0.56	-0.838	3.8E-01
Mtr.35915.1.S1_at	8.50	2.64	8.96	9.08	9.34	8.63	6.70	3.52	9.02	0.36	1.35	0.429	3.2E-01
Mtr.27396.1.S1_at	7.91	3.09	9.88	8.70	7.13	8.67	6.96	3.49	8.16	0.90	1.17	0.231	5.9E-01
Mtr.51360.1.S1_at	6.34	2.62	9.57	9.54	9.47	9.86	6.17	3.48	9.62	0.21	1.56	0.640	1.6E-01
Mtr.19319.1.S1_at	2.66	8.66	2.69	2.70	2.69	2.66	4.67	3.46	2.68	0.02	0.57	-0.800	3.8E-01
Mtr.40531.1.S1_at	8.62	3.39	9.89	9.40	8.17	9.45	7.30	3.44	9.01	0.72	1.23	0.303	4.5E-01
Mtr.44649.1.S1_at	6.96	1.82	8.24	8.45	7.34	8.28	5.68	3.40	8.02	0.60	1.41	0.500	3.0E-01
Mtr.3235.1.S1_at	8.84	2.98	8.86	9.06	9.18	8.92	6.89	3.39	9.05	0.13	1.31	0.393	3.3E-01
Mtr.47320.1.S1_s_at	5.11	10.82	4.87	4.93	4.75	5.18	6.93	3.37	4.95	0.22	0.71	-0.485	3.7E-01
Mtr.43877.1.S1_at	7.75	2.26	8.35	8.38	7.92	7.98	6.12	3.35	8.09	0.25	1.32	0.404	3.7E-01
Mtr.24893.1.S1_at	7.63	1.78	7.41	7.76	7.52	7.52	5.61	3.32	7.60	0.14	1.36	0.439	3.6E-01
Mtr.1368.1.S1_at	2.94	4.24	9.19	9.19	10.49	9.16	5.46	3.30	9.61	0.76	1.76	0.817	1.0E-01
Mtr.41147.1.S1_at	2.93	8.69	3.07	11.31	11.31	11.24	4.90	3.28	11.29	0.04	2.30	1.204	2.8E-02
Mtr.43627.1.S1_at	11.65	5.80	11.24	11.28	9.77	11.08	9.56	3.27	10.71	0.82	1.12	0.163	5.9E-01
Mtr.6626.1.S1_at	2.13	7.77	2.12	2.14	2.07	2.11	4.01	3.26	2.11	0.04	0.53	-0.927	3.7E-01
Mtr.41187.1.S1_at	8.96	2.96	8.09	9.00	9.53	9.50	6.67	3.25	9.34	0.30	1.40	0.486	2.3E-01
Mtr.8828.1.S1_at	8.18	2.91	8.81	9.40	8.45	9.64	6.63	3.24	9.17	0.63	1.38	0.467	2.5E-01
Mtr.29129.1.S1_at	9.43	3.71	9.19	8.58	7.80	8.64	7.44	3.23	8.34	0.47	1.12	0.165	6.6E-01
Mtr.21958.1.S1_at	2.68	8.21	2.67	2.67	2.22	2.81	4.52	3.20	2.57	0.31	0.57	-0.817	3.5E-01
Mtr.24020.1.S1_at	3.23	8.73	3.18	3.06	3.11	3.13	5.05	3.19	3.10	0.04	0.61	-0.705	3.5E-01
Mtr.45519.1.S1_at	3.24	9.60	6.18	8.15	8.60	7.67	6.34	3.18	8.14	0.47	1.28	0.360	3.9E-01
Mtr.16582.1.S1_at	2.53	7.98	2.49	2.51	2.51	2.50	4.33	3.16	2.51	0.00	0.58	-0.789	3.7E-01
Mtr.46226.1.S1_at	2.73	8.15	2.70	2.70	2.68	2.69	4.53	3.14	2.69	0.01	0.59	-0.751	3.7E-01
Mtr.38422.1.S1_at	6.29	2.80	9.07	9.15	7.94	9.02	6.05	3.14	8.70	0.66	1.44	0.524	2.3E-01
Mtr.40769.1.S1_at	5.38	2.78	9.03	8.96	7.97	8.90	5.73	3.14	8.61	0.55	1.50	0.588	1.9E-01
Mtr.43680.1.S1_at	7.00	2.92	9.07	8.89	9.97	9.67	6.33	3.13	9.51	0.56	1.50	0.588	1.6E-01
Mtr.31130.1.S1_at	4.06	9.45	4.08	4.45	4.05	4.05	5.86	3.11	4.18	0.23	0.71	-0.487	4.0E-01
Mtr.10114.1.S1_at	8.12	2.54	7.68	5.82	5.30	5.62	6.11	3.10	5.58	0.26	0.91	-0.131	7.8E-01
Mtr.11740.1.S1_at	9.24	3.41	8.18	8.62	8.31	8.68	6.94	3.10	8.54	0.20	1.23	0.298	4.2E-01
Mtr.345.1.S1_at	2.62	7.98	2.61	2.60	2.64	2.58	4.40	3.10	2.60	0.03	0.59	-0.758	3.7E-01
Mtr.9112.1.S1_at	8.09	2.48	7.55	7.68	7.33	7.27	6.04	3.09	7.43	0.22	1.23	0.299	4.8E-01
Mtr.7546.1.S1_at	10.24	5.46	11.23	11.95	12.17	12.29	8.98	3.08	12.14	0.17	1.35	0.435	1.5E-01
Mtr.27693.1.S1_at	10.13	5.51	4.30	6.85	7.19	7.11	6.64	3.07	7.05	0.18	1.06	0.086	8.3E-01
Mtr.34783.1.S1_at	8.49	3.28	8.70	8.91	8.73	8.82	6.82	3.07	8.82	0.09	1.29	0.371	3.2E-01
Mtr.41655.1.S1_at	9.11	3.00	6.16	8.06	8.91	8.72	6.09	3.05	8.56	0.44	1.41	0.492	2.4E-01
Mtr.17215.1.S1_x_at	2.26	7.52	2.24	2.22	2.47	2.22	4.01	3.04	2.31	0.14	0.58	-0.797	3.9E-01
Mtr.15123.1.S1_x_at	2.69	7.93	2.67	3.12	2.65	2.61	4.43	3.03	2.79	0.29	0.63	-0.665	4.0E-01
Mtr.44897.1.S1_at	8.74	3.25	8.22	8.84	8.67	8.67	6.74	3.03	8.73	0.10	1.30	0.374	3.2E-01
Mtr.49694.1.S1_x_at	3.86	9.01	3.80	3.84	3.71	3.52	5.55	2.99	3.69	0.16	0.66	-0.589	3.4E-01
Mtr.35082.1.S1_at	7.55	2.23	7.26	7.33	7.03	6.92	5.68	2.99	7.09	0.21	1.25	0.320	4.6E-01
Mtr.45470.1.S1_s_at	7.33	1.82	6.58	7.68	8.09	7.43	5.25	2.99	7.73	0.33	1.47	0.560	2.2E-01
Mtr.10740.1.S1_at	8.35	3.13	8.25	8.08	7.98	8.04	6.58	2.99	8.03	0.05	1.22	0.289	4.5E-01
Mtr.24231.1.S1_at	7.35	2.81	8.44	8.95	7.72	8.95	6.20	2.98	8.54	0.71	1.38	0.462	2.6E-01
Mtr.29662.1.S1_at	7.83	4.64	10.60	10.65	9.52	10.50	7.69	2.98	10.22	0.61	1.33	0.410	2.2E-01
Mtr.47266.1.S1_at	11.06	5.23	9.11	8.89	8.36	8.87	8.47	2.97	8.71	0.30	1.03	0.041	9.0E-01
Mtr.9594.1.S1_at	10.13	4.68	9.41	8.46	8.37	8.37	8.07	2.96	8.40	0.05	1.04	0.057	8.6E-01
Mtr.38410.1.S1_at	8.58	3.03	7.60	7.53	7.57	7.41	6.40	2.96	7.50	0.08	1.17	0.229	5.5E-01
Mtr.2058.1.S1_at	8.21	3.27	8.56	8.29	8.15	8.02	6.68	2.96	8.15	0.14	1.22	0.288	4.4E-01
Mtr.38855.1.S1_at	8.42	3.29	8.41	8.10	8.24	8.17	6.70	2.96	8.17	0.07	1.22	0.285	4.4E-01
Mtr.13662.1.S1_s_at	8.17	3.06	8.18	7.49	7.29	7.40	6.47	2.95	7.39	0.10	1.14	0.193	6.2E-01
Mtr.38810.1.S1_at	6.01	3.68	9.46	9.40	8.15	9.32	6.38	2.91	8.96	0.70	1.40	0.489	2.1E-01
Mtr.4496.1.S1_x_at	2.49	7.52	2.47	2.45	2.45	2.44	4.16	2.91	2.45	0.01	0.59	-0.765	3.7E-01
Mtr.34557.1.S1_at	3.35	8.34	3.27	3.35	3.30	3.33	4.99	2.91	3.33	0.03	0.67	-0.584	3.8E-01
Mtr.9270.1.S1_at	11.63	5.89	9.46	10.63	10.13	10.73	8.99	2.90	10.50	0.32	1.17	0.223	4.2E-01
Mtr.20896.1.S1_s_at	7.92	2.89	7.91	7.66	7.91	7.71	6.24	2.90	7.76	0.13	1.24	0.315	4.1E-01
Mtr.41515.1.S1_at	8.21	3.17	8.16	7.26	6.68	6.19	6.51	2.89	6.71	0.53	1.03	0.043	9.1E-01
Mtr.23540.1.S1_at	8.12	2.85	7.54	7.48	6.90	7.26	6.17	2.89	7.21	0.29	1.17	0.226	5.7E-01
Mtr.28297.1.S1_at	7.35	3.20	8.76	8.72	7.50	8.73	6.44	2.89	8.32	0.71	1.29	0.369	3.4E-01
Mtr.52039.1.S1_s_at	9.06	3.73	8.28	9.28	9.41	9.47	7.03	2.88	9.39	0.10	1.34	0.418	2.3E-01
Mtr.2913.1.S1_at	2.70	7.67	2.67	2.65	2.69	2.61	4.35	2.88	2.65	0.04	0.61	-0.714	3.6E-01
Mtr.39647.1.S1_at	5.66	11.07	10.03	8.57	8.99	9.35	8.92	2.87	8.97	0.39	1.01	0.008	9.8E-01
Mtr.306.1.S1_at	2.58	7.49	2.56	2.55	2.53	2.56	4.21	2.84	2.55	0.01	0.61	-0.725	3.7E-01
Mtr.9392.1.S1_at	8.02	3.24	8.25	9.11	7.91	9.23	6.51	2.83	8.75	0.73	1.34	0.427	2.5E-01
Mtr.4126.1.S1_at	7.64	2.97	8.04	7.90	7.81	7.61	6.22	2.81	7.77	0.15	1.25	0.322	3.9E-01
Mtr.33711.1.S1_at	2.25	7.11	2.25	2.24	2.23	2.24	3.87	2.81	2.24	0.01	0.58	-0.791	3.7E-01
Mtr.9891.1.S1_at	7.38	2.46	7.24	7.74	7.33	7.44	5.69	2.80	7.50	0.21	1.32	0.398	3.3E-01
Mtr.26895.1.S1_s_at	2.42	3.42	7.69	6.62	5.67	6.01	4.51	2.80	6.10	0.48	1.35	0.435	3.9E-01
Mtr.17663.1.S1_at	2.69	7.49	2.64	2.70	2.67	2.67	4.27	2.79	2.68	0.02	0.63	-0.674	3.8E-01
Mtr.20391.1.S1_at	9.87	4.94	9.65	10.00	10.67	10.40	8.15	2.78	10.36	0.34	1.27	0.345	2.4E-01
Mtr.41149.1.S1_at	9.54	4.39	8.75	9.54	10.14	9.65	7.56	2.77	9.78	0.32	1.29	0.371	2.4E-01
Mtr.37609.1.S1_at	7.85	3.47	8.58	6.87	6.51	6.43	6.63	2.77	6.60	0.23	1.00	-0.007	9.9E-01
Mtr.16671.1.S1_x_at	3.80	8.09	2.94	2.51	2.17	2.85	4.94	2.76	2.51	0.34	0.51	-0.978	2.0E-01
Mtr.44733.1.S1_at	9.20	4.30	8.94	9.09	9.36	8.89	7.48	2.76	9.11	0.24	1.22	0.285	3.6E-01
Mtr.38485.1.S1_at	7.20	2.39	7.13	7.17	7.25	7.08	5.57	2.76	7.16	0.08	1.29	0.362	3.7E-01
Mtr.8827.1.S1_at	9.60	5.22	10.29	10.79	9.98	10.84	8.37	2.75	10.54	0.48	1.26	0.332	2.5E-01
Mtr.14743.1.S1_at	7.83	3.13	7.96	8.06	8.20	7.92	6.31	2.75	8.06	0.14	1.28	0.354	3.3E-01
Mtr.21688.1.S1_at	2.66	7.41	2.63	2.63	2.61	2.60	4.24	2.75	2.61	0.02	0.62	-0.697	3.6E-01
Mtr.12566.1.S1_at	2.47	2.58	7.29	6.27	4.76	4.95	4.11	2.75	5.33	0.82	1.30	0.373	5.0E-01
Mtr.40562.1.S1_at	7.82	3.41	8.45	8.08	8.21	8.09	6.56	2.75	8.13	0.07	1.24	0.309	3.8E-01
Mtr.19575.1.S1_at	8.23	4.42	9.75	10.76	12.05	11.75	7.47	2.74	11.52	0.68	1.54	0.626	6.8E-02
Mtr.46448.1.S1_s_at	2.43	7.18	2.44	2.44	2.44	2.43	4.02	2.74	2.44	0.00	0.61	-0.720	3.7E-01
Mtr.36123.1.S1_at	9.25	13.98	9.24	9.19	8.93	9.41	10.82	2.74	9.18	0.24	0.85	-0.238	3.6E-01
Mtr.41905.1.S1_at	8.44	3.00	5.35	7.47	8.79	8.38	5.59	2.73	8.21	0.68	1.47	0.554	1.8E-01
Mtr.5242.1.S1_at	8.69	4.18	9.09	9.27	8.93	8.93	7.32	2.73	9.04	0.19	1.24	0.305	3.4E-01
Mtr.18639.1.S1_at	2.13	6.83	2.12	2.11	2.11	2.12	3.69	2.72	2.11	0.00	0.57	-0.805	3.7E-01
Mtr.4733.1.S1_s_at	8.09	3.62	8.52	8.23	8.27	7.92	6.74	2.71	8.14	0.19	1.21	0.272	4.2E-01
Mtr.13202.1.S1_at	8.03	3.48	8.30	8.75	8.24	8.35	6.60	2.71	8.45	0.27	1.28	0.355	3.1E-01
Mtr.13185.1.S1_at	4.66	2.57	7.93	7.92	6.84	7.76	5.05	2.70	7.51	0.58	1.49	0.571	2.0E-01
Mtr.3624.1.S1_at	8.19	13.28	12.21	10.75	11.32	11.40	11.23	2.69	11.15	0.36	0.99	-0.010	9.6E-01
Mtr.7040.1.S1_at	3.73	8.46	3.89	4.28	4.00	3.57	5.36	2.68	3.95	0.35	0.74	-0.440	4.2E-01
Mtr.44316.1.S1_at	7.60	2.83	7.34	8.98	8.31	8.74	5.92	2.68	8.67	0.34	1.46	0.550	1.5E-01
Mtr.2005.1.S1_at	8.17	3.96	3.20	4.21	4.55	4.78	5.11	2.68	4.51	0.29	0.88	-0.179	7.2E-01
Mtr.27637.1.S1_at	6.20	2.65	7.90	7.71	6.57	7.79	5.58	2.68	7.36	0.68	1.32	0.398	3.3E-01
Mtr.11126.1.S1_at	9.17	4.03	7.82	8.97	8.88	9.11	7.00	2.66	8.99	0.12	1.28	0.360	2.7E-01
Mtr.16277.1.S1_at	8.44	3.47	7.61	6.56	7.15	6.90	6.51	2.66	6.87	0.30	1.06	0.079	8.2E-01
Mtr.25314.1.S1_at	2.14	6.73	2.13	2.12	2.12	2.12	3.67	2.65	2.12	0.00	0.58	-0.790	3.7E-01
Mtr.1068.1.S1_s_at	8.01	3.37	7.88	8.30	7.99	8.26	6.42	2.64	8.18	0.16	1.27	0.350	3.1E-01
Mtr.721.1.S1_x_at	2.77	7.35	2.78	2.76	2.75	2.73	4.30	2.64	2.75	0.01	0.64	-0.646	3.7E-01
Mtr.44034.1.S1_at	2.75	6.58	7.81	9.75	10.14	9.96	5.71	2.64	9.95	0.19	1.74	0.801	5.0E-02
Mtr.4733.1.S1_at	7.92	3.55	8.29	8.32	8.18	7.86	6.59	2.64	8.12	0.24	1.23	0.302	3.7E-01
Mtr.10628.1.S1_at	9.12	4.76	9.50	9.86	8.87	9.56	7.79	2.63	9.43	0.51	1.21	0.275	3.5E-01
Mtr.36842.1.S1_s_at	11.04	5.78	8.64	9.81	7.32	9.67	8.49	2.63	8.93	1.40	1.05	0.074	8.1E-01
Mtr.52052.1.S1_at	3.12	7.65	3.09	3.04	3.00	3.07	4.62	2.63	3.04	0.04	0.66	-0.606	3.5E-01
Mtr.304.1.S1_at	2.66	7.17	2.65	2.64	2.64	2.64	4.16	2.61	2.64	0.00	0.63	-0.656	3.7E-01
Mtr.44643.1.S1_at	6.85	1.96	5.99	7.34	7.59	7.77	4.93	2.61	7.57	0.22	1.53	0.618	1.6E-01
Mtr.25314.1.S1_x_at	2.54	7.05	2.53	2.52	2.49	2.50	4.04	2.61	2.50	0.01	0.62	-0.691	3.6E-01
Mtr.13177.1.S1_at	8.93	4.25	8.58	9.17	9.51	9.30	7.25	2.61	9.33	0.17	1.29	0.363	2.4E-01
Mtr.50760.1.S1_at	2.89	2.50	7.19	7.33	5.97	7.01	4.19	2.60	6.77	0.71	1.61	0.691	1.7E-01
Mtr.48953.1.S1_at	2.79	7.27	2.74	2.62	2.76	2.76	4.27	2.60	2.71	0.08	0.64	-0.654	3.6E-01
Mtr.18385.1.S1_s_at	2.28	6.77	2.26	2.26	2.26	2.29	3.77	2.60	2.27	0.02	0.60	-0.732	3.7E-01
Mtr.10372.1.S1_at	6.35	2.96	8.06	8.83	9.02	8.84	5.79	2.60	8.90	0.11	1.54	0.620	1.1E-01
Mtr.27494.1.S1_at	8.35	3.55	7.66	8.08	8.64	8.04	6.52	2.60	8.25	0.34	1.27	0.340	3.2E-01
Mtr.40631.1.S1_at	9.17	4.06	7.39	8.52	8.18	8.51	6.87	2.59	8.41	0.19	1.22	0.290	3.7E-01
Mtr.29669.1.S1_at	2.63	7.11	2.62	2.62	2.60	2.62	4.12	2.59	2.61	0.01	0.64	-0.655	3.7E-01
Mtr.50276.1.S1_at	7.09	2.61	7.10	7.26	7.08	7.18	5.60	2.59	7.17	0.09	1.28	0.357	3.5E-01
Mtr.33572.1.S1_at	7.97	3.20	7.32	7.13	6.83	7.34	6.16	2.59	7.10	0.26	1.15	0.204	5.7E-01
Mtr.5517.1.S1_at	7.66	2.55	5.69	6.32	7.11	7.01	5.30	2.58	6.81	0.43	1.29	0.362	3.7E-01
Mtr.32088.1.S1_s_at	3.88	2.94	7.80	6.74	5.19	6.84	4.87	2.58	6.26	0.92	1.28	0.361	4.3E-01
Mtr.1484.1.S1_at	8.17	3.66	8.09	8.45	8.92	8.46	6.64	2.58	8.61	0.27	1.30	0.374	2.6E-01
Mtr.47085.1.S1_at	3.36	7.67	3.08	3.28	3.16	3.27	4.71	2.57	3.24	0.06	0.69	-0.539	3.8E-01
Mtr.9474.1.S1_at	9.11	4.09	7.51	7.96	8.00	8.30	6.90	2.56	8.09	0.19	1.17	0.228	4.7E-01
Mtr.41939.1.S1_at	9.97	5.62	10.12	9.27	8.73	8.82	8.57	2.56	8.94	0.29	1.04	0.061	8.2E-01
Mtr.34294.1.S1_at	2.11	6.53	2.10	2.09	2.09	2.09	3.58	2.55	2.09	0.00	0.58	-0.774	3.7E-01
Mtr.14295.1.S1_x_at	2.25	6.65	2.24	2.23	2.24	2.23	3.71	2.55	2.23	0.00	0.60	-0.734	3.7E-01
Mtr.37432.1.S1_at	8.21	3.66	7.89	7.78	7.79	8.06	6.58	2.54	7.88	0.16	1.20	0.258	4.3E-01
Mtr.5851.1.S1_at	4.54	3.52	8.33	8.58	7.04	8.34	5.46	2.54	7.99	0.83	1.46	0.548	1.8E-01

Mtr.556.1.S1_at	2.12	6.51	2.12	2.11	2.11	2.12	3.58	2.53	2.11	0.00	0.59	-0.761	3.7E-01
Mtr.35919.1.S1_at	7.88	3.37	7.59	8.89	9.44	8.94	6.28	2.52	9.09	0.30	1.45	0.534	1.3E-01
Mtr.6629.1.S1_at	2.55	6.94	2.60	2.59	2.59	2.58	4.03	2.52	2.59	0.01	0.64	-0.640	3.8E-01
Mtr.12981.1.S1_at	6.95	2.86	7.47	7.40	7.37	7.09	5.76	2.52	7.28	0.17	1.26	0.339	3.6E-01
Mtr.21107.1.S1_at	2.47	6.80	2.44	2.42	2.41	2.40	3.90	2.51	2.41	0.01	0.62	-0.694	3.6E-01
Mtr.15592.1.S1_at	3.15	7.44	3.04	3.14	3.14	2.93	4.54	2.51	3.07	0.12	0.68	-0.565	3.7E-01
Mtr.18597.1.S1_at	8.09	3.25	6.80	6.99	7.19	7.52	6.05	2.50	7.23	0.26	1.20	0.258	4.6E-01
Mtr.22120.1.S1_s_at	4.30	8.63	4.29	4.27	4.27	4.29	5.74	2.50	4.28	0.01	0.75	-0.424	3.7E-01
Mtr.2803.1.S1_at	2.24	6.56	2.23	2.23	2.23	2.23	3.68	2.50	2.23	0.00	0.61	-0.723	3.7E-01
Mtr.10058.1.S1_at	7.45	3.15	7.51	7.18	7.14	7.33	6.04	2.50	7.22	0.10	1.20	0.257	4.6E-01
Mtr.45162.1.S1_at	8.01	12.80	11.63	10.19	10.69	10.93	10.81	2.50	10.60	0.38	0.98	-0.028	8.9E-01
Mtr.47402.1.S1_at	2.61	6.93	2.60	2.60	2.60	2.60	4.05	2.50	2.60	0.00	0.64	-0.637	3.7E-01
Mtr.3086.1.S1_at	8.54	4.51	9.08	8.93	8.76	8.64	7.38	2.50	8.77	0.14	1.19	0.250	3.9E-01
Mtr.41570.1.S1_s_at	8.20	3.75	7.92	7.74	7.45	7.90	6.62	2.49	7.70	0.23	1.16	0.217	5.0E-01
Mtr.8606.1.S1_at	9.22	4.87	9.15	10.23	9.97	10.29	7.75	2.49	10.16	0.17	1.31	0.392	1.7E-01
Mtr.38330.1.S1_at	4.29	5.80	9.16	7.82	8.54	6.36	6.42	2.49	7.58	1.11	1.18	0.239	5.0E-01
Mtr.47320.1.S1_x_at	6.13	10.38	6.01	6.10	6.05	6.12	7.51	2.49	6.09	0.04	0.81	-0.302	3.8E-01
Mtr.31805.1.S1_at	7.05	2.73	7.04	7.13	7.71	6.94	5.61	2.49	7.26	0.40	1.29	0.373	3.2E-01
Mtr.23616.1.S1_at	6.36	2.06	2.05	2.03	2.00	2.03	3.49	2.48	2.02	0.02	0.58	-0.788	3.6E-01
Mtr.23596.1.S1_s_at	7.47	3.14	7.42	6.72	7.45	6.83	6.01	2.48	7.00	0.40	1.16	0.219	5.3E-01
Mtr.43295.1.S1_at	8.42	6.16	3.47	6.60	6.48	7.21	6.01	2.48	6.76	0.39	1.12	0.169	6.3E-01
Mtr.22023.1.S1_at	2.38	6.66	2.37	2.36	2.36	2.36	3.80	2.48	2.36	0.00	0.62	-0.688	3.7E-01
Mtr.3944.1.S1_at	8.00	4.15	8.77	8.48	8.42	8.18	6.98	2.48	8.36	0.16	1.20	0.261	3.9E-01
Mtr.51774.1.S1_at	7.58	2.94	6.72	7.52	7.81	7.95	5.75	2.47	7.76	0.22	1.35	0.434	2.3E-01
Mtr.15109.1.S1_at	7.61	3.19	7.31	7.60	8.05	7.81	6.04	2.46	7.82	0.22	1.30	0.374	2.8E-01
Mtr.43797.1.S1_at	4.11	3.22	7.85	7.31	5.79	7.18	5.06	2.46	6.76	0.84	1.34	0.417	3.2E-01
Mtr.43098.1.S1_at	10.17	5.29	8.15	8.39	7.65	8.49	7.87	2.45	8.18	0.46	1.04	0.055	8.4E-01
Mtr.51603.1.S1_at	2.86	7.09	2.83	3.10	2.79	2.79	4.26	2.45	2.90	0.18	0.68	-0.557	3.9E-01
Mtr.48232.1.S1_at	3.11	7.38	3.16	3.16	3.03	3.15	4.55	2.45	3.11	0.07	0.68	-0.546	3.7E-01
Mtr.50280.1.S1_s_at	7.06	2.83	7.07	6.60	6.64	6.50	5.65	2.45	6.58	0.07	1.16	0.219	5.5E-01
Mtr.36920.1.S1_at	2.53	6.75	2.50	2.47	2.45	2.71	3.93	2.45	2.54	0.14	0.65	-0.627	3.8E-01
Mtr.22710.1.S1_at	2.56	6.78	2.52	2.56	2.51	2.55	3.95	2.45	2.54	0.03	0.64	-0.638	3.7E-01
Mtr.7298.1.S1_at	8.11	4.53	9.20	8.92	8.13	8.91	7.28	2.45	8.66	0.45	1.19	0.249	3.9E-01
Mtr.35174.1.S1_at	7.78	3.31	7.25	8.26	7.91	8.15	6.11	2.44	8.11	0.18	1.33	0.407	2.3E-01
Mtr.14391.1.S1_at	2.39	6.61	2.38	2.37	2.37	2.36	3.79	2.44	2.36	0.01	0.62	-0.681	3.7E-01
Mtr.39593.1.S1_at	7.13	5.62	10.39	10.30	9.73	10.20	7.71	2.43	10.07	0.30	1.31	0.385	1.7E-01
Mtr.29666.1.S1_at	7.71	3.32	7.34	7.64	7.58	7.87	6.13	2.43	7.69	0.15	1.26	0.328	3.3E-01
Mtr.9645.1.S1_at	2.01	2.68	6.52	5.94	5.20	5.51	3.74	2.43	5.55	0.37	1.49	0.571	2.7E-01
Mtr.51557.1.S1_at	6.77	3.33	8.02	8.40	7.81	8.17	6.04	2.43	8.13	0.30	1.35	0.429	2.1E-01
Mtr.38264.1.S1_at	7.04	2.81	6.98	6.80	6.50	6.43	5.61	2.43	6.58	0.20	1.17	0.229	5.3E-01
Mtr.43089.1.S1_at	10.88	6.62	10.76	9.26	9.76	9.46	9.42	2.43	9.49	0.25	1.01	0.011	9.6E-01
Mtr.28963.1.S1_at	2.25	6.43	2.23	2.23	2.23	2.23	3.64	2.42	2.23	0.00	0.61	-0.706	3.7E-01
Mtr.48895.1.S1_s_at	7.97	12.07	7.81	7.93	7.92	8.18	9.28	2.42	8.01	0.15	0.86	-0.213	4.1E-01
Mtr.24309.1.S1_x_at	2.47	6.66	2.47	2.45	2.43	2.47	3.87	2.42	2.45	0.02	0.63	-0.658	3.7E-01
Mtr.13563.1.S1_s_at	7.14	2.85	6.91	7.24	7.35	7.62	5.63	2.42	7.40	0.19	1.31	0.394	2.7E-01
Mtr.47082.1.S1_at	2.38	6.55	2.36	2.36	2.36	2.34	3.76	2.41	2.35	0.01	0.62	-0.679	3.7E-01
Mtr.13008.1.S1_at	9.84	5.49	9.46	9.19	9.23	9.37	8.26	2.41	9.27	0.09	1.12	0.165	5.1E-01
Mtr.1007.1.S1_at	2.13	6.29	2.12	2.12	2.11	2.11	3.51	2.41	2.11	0.00	0.60	-0.732	3.7E-01
Mtr.5546.1.S1_at	7.45	2.80	4.05	6.55	6.60	6.53	4.77	2.40	6.56	0.03	1.38	0.461	2.7E-01
Mtr.34389.1.S1_at	8.45	4.29	8.44	7.64	7.63	7.61	7.06	2.40	7.62	0.02	1.08	0.112	7.0E-01
Mtr.33765.1.S1_s_at	8.51	4.20	8.18	8.98	8.93	8.75	6.96	2.40	8.89	0.12	1.28	0.352	2.4E-01
Mtr.7645.1.S1_at	2.55	6.69	2.52	2.50	2.55	2.52	3.92	2.40	2.52	0.02	0.64	-0.634	3.7E-01
Mtr.42042.1.S1_at	6.11	1.94	6.07	6.27	5.89	6.33	4.71	2.40	6.16	0.23	1.31	0.389	3.5E-01
Mtr.35183.1.S1_s_at	7.00	2.67	6.61	7.04	7.29	7.02	5.43	2.40	7.12	0.15	1.31	0.391	2.9E-01
Mtr.1214.1.S1_at	6.99	2.24	5.18	8.38	8.82	8.88	4.81	2.40	8.69	0.27	1.81	0.855	4.9E-02
Mtr.4937.1.S1_at	6.10	2.12	6.39	6.22	6.62	6.32	4.87	2.39	6.39	0.21	1.31	0.391	3.3E-01
Mtr.9505.1.S1_at	9.27	4.71	8.20	8.07	8.25	8.13	7.39	2.38	8.15	0.09	1.10	0.140	6.1E-01
Mtr.46009.1.S1_at	8.04	3.64	7.43	7.32	6.22	7.01	6.37	2.38	6.85	0.57	1.07	0.104	7.5E-01
Mtr.28282.1.S1_a_at	1.97	6.09	1.96	1.96	1.96	1.96	3.34	2.38	1.96	0.00	0.59	-0.771	3.7E-01
Mtr.10958.1.S1_at	8.99	4.84	8.90	9.42	9.30	9.80	7.58	2.37	9.51	0.26	1.25	0.328	2.3E-01
Mtr.14834.1.S1_at	6.19	2.97	7.60	6.89	6.67	6.77	5.59	2.37	6.78	0.11	1.21	0.279	4.3E-01
Mtr.41272.1.S1_at	7.44	2.88	6.28	5.00	4.76	4.13	5.53	2.37	4.63	0.45	0.84	-0.256	5.5E-01
Mtr.34580.1.S1_at	2.42	6.51	2.40	2.40	2.39	2.38	3.78	2.37	2.39	0.01	0.63	-0.659	3.7E-01
Mtr.35314.1.S1_at	8.32	4.13	8.12	8.65	8.64	8.23	6.86	2.36	8.51	0.24	1.24	0.311	3.0E-01
Mtr.41855.1.S1_at	8.70	4.02	6.93	7.23	7.44	7.26	6.55	2.36	7.31	0.12	1.11	0.157	6.1E-01
Mtr.6763.1.S1_at	2.48	6.53	2.41	2.39	2.39	2.43	3.81	2.36	2.40	0.02	0.63	-0.663	3.6E-01
Mtr.38421.1.S1_at	4.88	2.93	7.63	7.41	6.47	7.37	5.15	2.36	7.08	0.53	1.38	0.461	2.4E-01
Mtr.47683.1.S1_x_at	8.86	4.34	7.74	8.28	8.66	8.13	6.98	2.36	8.36	0.27	1.20	0.259	3.7E-01
Mtr.22852.1.S1_at	4.14	7.92	3.61	3.72	3.87	4.12	5.22	2.35	3.90	0.20	0.75	-0.420	3.9E-01
Mtr.35392.1.S1_x_at	7.93	11.86	7.67	7.80	7.73	7.81	9.15	2.35	7.78	0.04	0.85	-0.235	3.7E-01
Mtr.13855.1.S1_at	7.26	2.97	6.78	7.68	7.20	7.33	5.67	2.35	7.41	0.25	1.31	0.386	2.7E-01
Mtr.5939.1.S1_s_at	9.98	5.31	8.08	8.39	7.57	8.40	7.79	2.35	8.12	0.48	1.04	0.061	8.2E-01
Mtr.34376.1.S1_at	7.25	3.11	7.09	7.49	7.63	7.72	5.82	2.35	7.61	0.12	1.31	0.388	2.6E-01
Mtr.11016.1.S1_at	7.06	3.35	7.70	6.99	6.69	6.66	6.03	2.35	6.78	0.18	1.12	0.168	6.1E-01
Mtr.18799.1.S1_s_at	5.55	9.70	5.75	9.55	9.69	7.64	7.00	2.35	8.96	1.14	1.28	0.357	2.6E-01
Mtr.14527.1.S1_at	7.69	3.15	6.45	6.58	6.40	6.66	5.76	2.34	6.55	0.13	1.14	0.184	5.9E-01
Mtr.41570.1.S1_at	7.77	3.66	7.64	6.92	6.45	7.02	6.36	2.34	6.79	0.30	1.07	0.096	7.6E-01
Mtr.18757.1.S1_at	7.98	3.50	4.57	4.37	3.89	5.24	5.35	2.34	4.50	0.68	0.84	-0.250	5.8E-01
Mtr.16668.1.S1_x_at	2.12	6.15	2.10	2.10	2.07	2.10	3.46	2.33	2.09	0.02	0.60	-0.727	3.7E-01
Mtr.20428.1.S1_x_at	2.32	6.36	2.31	2.30	2.30	2.30	3.66	2.33	2.30	0.00	0.63	-0.672	3.7E-01
Mtr.27916.1.S1_at	5.47	3.19	7.84	8.17	6.72	8.02	5.50	2.33	7.64	0.80	1.39	0.474	2.1E-01
Mtr.9079.1.S1_at	10.54	6.08	9.47	9.03	7.87	8.75	8.70	2.33	8.55	0.61	0.98	-0.025	9.2E-01
Mtr.5560.1.S1_s_at	7.00	2.83	6.69	6.91	7.09	7.13	5.51	2.33	7.04	0.12	1.28	0.355	3.2E-01
Mtr.44766.1.S1_at	6.68	2.32	5.89	5.96	5.62	4.90	4.96	2.33	5.49	0.55	1.11	0.146	7.2E-01
Mtr.33789.1.S1_at	8.22	4.13	8.08	8.53	8.76	8.57	6.81	2.32	8.62	0.12	1.27	0.340	2.5E-01
Mtr.7159.1.S1_at	7.91	3.49	6.92	5.54	4.73	4.85	6.11	2.32	5.04	0.44	0.82	-0.278	4.8E-01
Mtr.49012.1.S1_at	7.30	3.19	7.11	6.93	7.23	7.10	5.87	2.32	7.09	0.15	1.21	0.272	4.1E-01
Mtr.19808.1.S1_at	8.98	5.13	9.29	9.26	9.07	9.10	7.80	2.32	9.14	0.10	1.17	0.230	3.7E-01
Mtr.28862.1.S1_at	2.38	6.38	2.36	2.36	2.36	2.38	3.71	2.31	2.37	0.01	0.64	-0.647	3.7E-01
Mtr.3002.1.S1_at	7.81	4.30	8.66	8.44	8.21	8.00	6.92	2.31	8.22	0.22	1.19	0.247	3.9E-01
Mtr.39376.1.S1_at	8.36	4.72	9.00	8.87	8.99	8.59	7.36	2.31	8.82	0.20	1.20	0.261	3.4E-01
Mtr.31658.1.S1_at	2.27	6.26	2.26	2.26	2.26	2.25	3.60	2.31	2.26	0.01	0.63	-0.672	3.7E-01
Mtr.24395.1.S1_at	3.39	2.02	6.52	6.57	4.57	5.68	3.98	2.31	5.61	1.00	1.41	0.496	3.2E-01
Mtr.11430.1.S1_s_at	6.49	2.51	6.51	5.89	6.76	6.44	5.17	2.30	6.36	0.44	1.23	0.299	4.3E-01
Mtr.35481.1.S1_at	6.28	2.32	6.34	5.89	6.25	6.01	4.98	2.30	6.05	0.19	1.21	0.280	4.7E-01
Mtr.27812.1.S1_at	6.35	2.50	6.62	6.02	7.26	6.53	5.16	2.30	6.61	0.63	1.28	0.357	3.5E-01
Mtr.44600.1.S1_at	4.40	2.55	7.11	6.99	5.75	6.89	4.69	2.30	6.54	0.69	1.40	0.481	2.5E-01
Mtr.15443.1.S1_at	7.31	3.12	6.84	6.92	7.33	7.20	5.76	2.29	7.15	0.21	1.24	0.312	3.5E-01
Mtr.33677.1.S1_s_at	6.98	2.92	6.78	7.18	6.40	6.76	5.56	2.29	6.78	0.39	1.22	0.286	4.1E-01
Mtr.22658.1.S1_x_at	2.79	6.75	2.77	2.75	2.62	2.72	4.10	2.29	2.70	0.07	0.66	-0.606	3.5E-01
Mtr.13595.1.S1_at	7.16	2.59	5.13	5.76	5.25	5.46	4.96	2.29	5.49	0.25	1.11	0.147	7.1E-01
Mtr.35224.1.S1_at	4.21	8.29	4.45	6.41	7.21	6.89	5.65	2.29	6.84	0.40	1.21	0.275	4.3E-01
Mtr.46619.1.S1_s_at	7.68	3.30	6.64	6.53	6.67	5.86	5.88	2.29	6.35	0.43	1.08	0.112	7.4E-01
Mtr.10264.1.S1_at	7.91	3.88	7.76	7.64	7.22	7.54	6.52	2.29	7.47	0.22	1.15	0.196	5.1E-01
Mtr.21701.1.S1_s_at	9.67	5.43	9.00	8.75	8.14	8.42	8.03	2.28	8.44	0.31	1.05	0.070	7.8E-01
Mtr.32329.1.S1_at	6.62	3.01	7.24	6.59	7.06	5.81	5.62	2.28	6.49	0.63	1.15	0.206	5.6E-01
Mtr.42856.1.S1_at	11.09	6.83	10.36	10.39	10.06	10.47	9.43	2.28	10.31	0.22	1.09	0.129	5.4E-01
Mtr.33522.1.S1_at	6.22	3.80	8.36	6.61	6.68	4.94	6.13	2.28	6.08	0.98	0.99	-0.011	9.7E-01
Mtr.16742.1.S1_x_at	2.85	6.83	2.91	2.89	2.71	2.89	4.20	2.28	2.83	0.11	0.67	-0.569	3.6E-01
Mtr.42341.1.S1_at	8.30	3.90	7.14	7.33	6.86	7.71	6.45	2.28	7.30	0.43	1.13	0.179	5.6E-01
Mtr.21071.1.S1_at	7.38	3.59	7.67	7.65	7.69	7.56	6.21	2.28	7.63	0.07	1.23	0.297	3.4E-01
Mtr.19020.1.S1_at	2.65	6.57	2.61	2.98	2.64	2.64	3.94	2.27	2.75	0.19	0.70	-0.517	4.2E-01
Mtr.38688.1.S1_at	8.27	3.81	6.79	6.98	6.29	7.06	6.29	2.27	6.77	0.42	1.08	0.107	7.4E-01
Mtr.13375.1.S1_at	8.50	4.05	7.09	7.53	6.94	7.08	6.55	2.27	7.18	0.31	1.10	0.134	6.6E-01
Mtr.38286.1.S1_at	7.77	3.35	6.43	5.44	7.11	6.81	5.85	2.27	6.45	0.89	1.10	0.141	6.9E-01
Mtr.48840.1.S1_x_at	2.60	6.54	2.63	2.61	2.38	2.58	3.92	2.27	2.52	0.12	0.64	-0.638	3.5E-01
Mtr.21783.1.S1_at	2.68	6.60	2.66	2.65	2.65	2.57	3.98	2.27	2.62	0.04	0.66	-0.602	3.6E-01
Mtr.34379.1.S1_s_at	5.49	2.63	7.10	7.22	6.49	7.22	5.07	2.26	6.98	0.42	1.38	0.460	2.3E-01
Mtr.40143.1.S1_at	8.27	4.15	7.83	7.57	7.51	7.52	6.75	2.26	7.53	0.03	1.12	0.159	5.8E-01
Mtr.44708.1.S1_at	7.26	3.19	6.94	7.14	6.90	6.77	5.80	2.26	6.94	0.18	1.20	0.259	4.3E-01
Mtr.33679.1.S1_at	2.07	5.98	2.06	2.06	2.06	2.07	3.37	2.26	2.06	0.01	0.61	-0.708	3.7E-01
Mtr.43508.1.S1_at	7.65	3.22	6.17	6.70	7.33	7.59	5.68	2.25	7.21	0.46	1.27	0.344	3.1E-01
Mtr.12856.1.S1_at	9.21	5.27	9.14	9.84	9.59	9.42	7.88	2.25	9.62	0.21	1.22	0.288	2.5E-01
Mtr.4626.1.S1_at	7.33	3.55	7.54	7.20	7.56	6.87	6.14	2.25	7.21	0.35	1.17	0.232	4.6E-01
Mtr.28439.1.S1_at	7.31	3.21	6.84	6.75	7.04	7.01	5.78	2.25	6.93	0.16	1.20	0.261	4.3E-01
Mtr.38635.1.S1_at	7.07	3.20	7.12	6.31	7.20	7.17	5.80	2.25	6.89	0.51	1.19	0.250	4.6E-01
Mtr.18951.1.S1_at	8.12	3.80	6.99	6.68	6.27	6.38	6.30	2.24	6.44	0.22	1.02	0.031	9.2E-01
Mtr.36954.1.S1_at	6.44	2.52	6.36	6.18	6.19	6.38	5.11	2.24	6.25	0.11	1.22	0.291	4.3E-01
Mtr.2337.1.S1_at	7.12	2.71	4.19	5.87	5.50	6.32	4.68	2.24	5.89	0.41	1.26	0.334	4.1E-01
Mtr.9427.1.S1_at	7.36	3.08	6.39	6.13	5.98	5.62	5.61	2.24	5.91	0.26	1.05	0.075	8.3E-01
Mtr.12807.1.S1_at	6.68	2.40	3.41	2.28	3.72	2.48	4.16	2.24	2.82	0.78	0.68	-0.560	3.8E-01

Mtr.13982.1.S1_at	6.67	2.42	3.32	2.91	3.04	3.24	4.14	2.24	3.06	0.17	0.74	-0.433	4.5E-01
Mtr.41113.1.S1_at	7.84	3.69	7.21	7.16	6.61	7.26	6.25	2.24	7.01	0.35	1.12	0.165	5.9E-01
Mtr.17323.1.S1_at	2.43	6.29	2.41	2.39	2.39	2.37	3.71	2.24	2.38	0.01	0.64	-0.639	3.6E-01
Mtr.34644.1.S1_at	2.55	6.53	2.78	2.54	2.53	2.49	3.95	2.23	2.52	0.03	0.64	-0.651	3.3E-01
Mtr.22013.1.S1_at	2.01	6.04	2.35	2.35	2.35	2.34	3.46	2.23	2.35	0.00	0.68	-0.562	4.4E-01
Mtr.49327.1.S1_at	6.84	2.46	5.40	6.90	5.90	6.74	4.90	2.23	6.52	0.54	1.33	0.412	2.9E-01
Mtr.748.1.S1_s_at	8.07	4.28	8.22	7.71	7.81	7.83	6.85	2.23	7.79	0.06	1.14	0.184	5.1E-01
Mtr.38928.1.S1_at	6.83	2.80	6.49	6.74	6.90	7.10	5.37	2.23	6.91	0.18	1.29	0.364	3.0E-01
Mtr.18491.1.S1_at	8.05	3.74	6.90	6.74	4.31	6.24	6.23	2.23	5.77	1.28	0.93	-0.112	7.7E-01
Mtr.40106.1.S1_s_at	6.94	4.70	9.16	6.92	7.39	5.64	6.93	2.23	6.65	0.91	0.96	-0.059	8.5E-01
Mtr.20682.1.S1_at	9.64	13.47	9.59	9.69	9.35	9.59	10.90	2.23	9.54	0.18	0.88	-0.192	3.5E-01
Mtr.41311.1.S1_at	9.60	5.91	9.93	9.93	9.64	9.66	8.48	2.23	9.74	0.16	1.15	0.201	3.8E-01
Mtr.12358.1.S1_at	7.57	3.12	5.46	5.15	4.83	5.38	5.38	2.23	5.12	0.28	0.95	-0.071	8.5E-01
Mtr.22515.1.S1_at	2.08	5.93	2.07	2.06	2.06	2.06	3.36	2.23	2.06	0.00	0.61	-0.704	3.7E-01
Mtr.26947.1.S1_at	7.18	2.96	6.28	4.56	6.11	5.65	5.47	2.22	5.44	0.79	0.99	-0.008	9.8E-01
Mtr.4496.1.S1_at	3.25	6.88	2.84	2.93	2.83	2.76	4.32	2.22	2.84	0.09	0.66	-0.608	3.1E-01
Mtr.12806.1.S1_at	10.19	5.94	6.92	9.58	9.13	9.90	7.68	2.22	9.54	0.39	1.24	0.312	2.3E-01
Mtr.12610.1.S1_at	10.73	6.37	9.20	8.59	8.85	8.65	8.77	2.21	8.70	0.13	0.99	-0.012	9.6E-01
Mtr.1538.1.S1_at	7.10	4.23	8.57	7.37	8.14	6.87	6.64	2.21	7.46	0.64	1.12	0.169	5.7E-01
Mtr.33566.1.S1_at	6.95	2.54	4.91	5.18	4.92	5.04	4.80	2.21	5.05	0.13	1.05	0.071	8.6E-01
Mtr.5598.1.S1_at	6.64	3.31	7.49	6.36	7.04	6.63	5.81	2.21	6.68	0.34	1.15	0.200	5.4E-01
Mtr.5043.1.S1_at	6.15	2.38	6.25	6.22	6.05	5.79	4.93	2.21	6.02	0.22	1.22	0.290	4.4E-01
Mtr.9203.1.S1_at	7.54	3.53	7.13	7.34	7.19	7.57	6.06	2.21	7.36	0.19	1.21	0.280	3.7E-01
Mtr.15109.1.S1_s_at	7.93	4.14	8.00	8.33	8.09	8.26	6.69	2.21	8.23	0.13	1.23	0.298	3.0E-01
Mtr.49444.1.S1_at	2.35	6.18	2.37	2.36	2.36	2.36	3.63	2.21	2.36	0.00	0.65	-0.621	3.7E-01
Mtr.44279.1.S1_at	9.20	5.29	9.01	9.77	9.31	10.00	7.83	2.21	9.69	0.35	1.24	0.308	2.2E-01
Mtr.9638.1.S1_at	6.62	2.65	6.30	6.83	6.40	6.35	5.19	2.20	6.52	0.26	1.26	0.330	3.6E-01
Mtr.45641.1.S1_x_at	2.30	6.11	2.29	2.29	2.29	2.29	3.56	2.20	2.29	0.00	0.64	-0.640	3.7E-01
Mtr.32551.1.S1_at	6.02	2.34	6.27	5.99	5.98	6.06	4.87	2.20	6.01	0.04	1.23	0.303	4.2E-01
Mtr.45183.1.S1_at	9.27	5.57	9.47	9.68	9.61	9.29	8.10	2.20	9.53	0.21	1.18	0.233	3.3E-01
Mtr.35239.1.S1_at	8.42	4.39	7.93	7.87	7.74	7.89	6.91	2.20	7.83	0.09	1.13	0.180	5.1E-01
Mtr.30420.1.S1_at	7.64	3.93	7.80	7.62	7.87	7.66	6.46	2.19	7.72	0.14	1.20	0.257	3.8E-01
Mtr.27864.1.S1_at	2.61	6.33	2.46	2.46	2.45	2.45	3.80	2.19	2.45	0.00	0.65	-0.631	3.5E-01
Mtr.33189.1.S1_s_at	8.23	4.10	7.44	7.96	8.04	8.33	6.59	2.19	8.11	0.19	1.23	0.299	3.0E-01
Mtr.13443.1.S1_at	7.08	3.34	7.17	7.26	7.19	6.77	5.87	2.19	7.07	0.27	1.21	0.270	4.0E-01
Mtr.39821.1.S1_at	5.74	1.78	5.37	5.34	5.33	5.63	4.30	2.19	5.43	0.17	1.26	0.339	4.2E-01
Mtr.27505.1.S1_at	6.59	3.26	7.38	7.29	6.97	6.98	5.74	2.19	7.08	0.18	1.23	0.302	3.5E-01
Mtr.18902.1.S1_x_at	2.29	6.07	2.28	2.27	2.27	2.27	3.55	2.18	2.27	0.00	0.64	-0.643	3.7E-01
Mtr.51457.1.S1_at	2.11	5.88	2.10	2.10	2.09	2.10	3.36	2.18	2.09	0.01	0.62	-0.683	3.7E-01
Mtr.23325.1.S1_at	8.55	4.58	8.12	8.68	8.37	8.63	7.08	2.18	8.56	0.17	1.21	0.273	3.1E-01
Mtr.2167.1.S1_s_at	7.79	3.61	6.73	6.97	6.92	7.43	6.04	2.18	7.11	0.28	1.18	0.234	4.5E-01
Mtr.26359.1.S1_at	2.59	6.36	2.59	2.58	2.58	2.57	3.85	2.18	2.58	0.01	0.67	-0.578	3.7E-01
Mtr.24929.1.S1_at	10.82	6.55	9.41	11.44	10.14	11.40	8.93	2.17	10.99	0.74	1.23	0.301	1.9E-01
Mtr.15115.1.S1_at	7.33	4.15	8.31	7.88	7.30	6.72	6.59	2.17	7.30	0.58	1.11	0.147	6.2E-01
Mtr.35413.1.S1_at	6.60	3.69	7.94	8.20	7.39	8.21	6.08	2.17	7.93	0.47	1.31	0.384	2.2E-01
Mtr.31533.1.S1_at	2.49	6.26	2.49	2.48	2.48	2.52	3.75	2.17	2.50	0.02	0.67	-0.586	3.8E-01
Mtr.17977.1.S1_x_at	2.79	6.57	2.84	2.79	2.89	2.76	4.06	2.17	2.82	0.07	0.69	-0.530	3.8E-01
Mtr.2633.1.S1_s_at	7.52	3.19	5.59	11.94	11.74	11.87	5.43	2.17	11.85	0.10	2.18	1.125	6.9E-03
Mtr.27095.1.S1_at	6.06	2.18	5.82	5.71	5.80	5.64	4.68	2.17	5.72	0.08	1.22	0.288	4.6E-01
Mtr.51621.1.S1_at	2.78	6.52	2.75	2.77	2.69	2.84	4.02	2.17	2.77	0.07	0.69	-0.536	3.8E-01
Mtr.1402.1.S1_at	7.51	3.42	6.71	7.65	7.81	7.94	5.88	2.17	7.80	0.14	1.33	0.408	2.0E-01
Mtr.10215.1.S1_at	6.84	3.18	7.02	7.21	7.22	7.39	5.68	2.16	7.27	0.10	1.28	0.357	2.7E-01
Mtr.4017.1.S1_at	2.09	5.83	2.08	2.07	2.07	2.07	3.33	2.16	2.07	0.00	0.62	-0.686	3.7E-01
Mtr.43024.1.S1_at	7.14	3.27	6.89	7.09	6.88	7.03	5.77	2.16	7.00	0.11	1.21	0.279	3.8E-01
Mtr.11271.1.S1_at	10.59	6.33	7.84	8.69	8.10	8.48	8.25	2.16	8.42	0.30	1.02	0.030	9.0E-01
Mtr.42205.1.S1_at	6.61	2.41	5.41	6.42	6.37	6.58	4.81	2.16	6.46	0.11	1.34	0.425	2.6E-01
Mtr.38183.1.S1_at	7.60	3.79	7.45	8.37	8.94	8.60	6.28	2.16	8.64	0.29	1.38	0.460	1.3E-01
Mtr.25358.1.S1_at	3.23	6.93	3.15	3.13	3.54	3.13	4.44	2.16	3.26	0.24	0.74	-0.443	4.0E-01
Mtr.48776.1.S1_at	2.33	6.07	2.32	2.32	2.28	2.35	3.57	2.16	2.32	0.04	0.65	-0.626	3.7E-01
Mtr.18659.1.S1_s_at	9.45	5.42	8.76	9.34	8.78	9.28	7.88	2.15	9.13	0.31	1.16	0.213	3.8E-01
Mtr.9310.1.S1_s_at	4.16	3.41	7.46	6.93	5.70	6.88	5.01	2.15	6.50	0.70	1.30	0.377	3.2E-01
Mtr.14203.1.S1_at	2.36	6.09	2.36	2.35	2.34	2.68	3.60	2.15	2.46	0.19	0.68	-0.551	4.1E-01
Mtr.4593.1.S1_at	7.86	3.61	6.35	6.82	6.99	7.03	5.94	2.15	6.95	0.11	1.17	0.226	4.6E-01
Mtr.10220.1.S1_s_at	10.17	5.87	8.13	8.40	7.79	8.45	8.05	2.15	8.21	0.37	1.02	0.028	9.1E-01
Mtr.9288.1.S1_at	5.69	2.64	6.79	6.27	6.99	5.98	5.04	2.15	6.42	0.52	1.27	0.348	3.4E-01
Mtr.29876.1.S1_at	7.43	3.86	7.72	8.06	8.42	8.09	6.34	2.15	8.19	0.20	1.29	0.370	2.1E-01
Mtr.7091.1.S1_at	6.80	3.53	2.75	4.02	3.34	3.92	4.36	2.15	3.76	0.37	0.86	-0.215	6.6E-01
Mtr.18605.1.S1_x_at	2.94	2.37	6.34	6.58	4.83	6.17	3.88	2.15	5.86	0.91	1.51	0.593	2.2E-01
Mtr.19068.1.S1_x_at	2.21	5.92	2.19	2.20	2.20	2.23	3.44	2.15	2.21	0.02	0.64	-0.637	3.8E-01
Mtr.15184.1.S1_s_at	2.71	6.34	2.54	2.66	2.41	2.62	3.86	2.15	2.56	0.13	0.66	-0.592	3.5E-01
Mtr.35754.1.S1_at	6.60	3.29	7.31	7.10	6.93	6.79	5.74	2.15	6.94	0.15	1.21	0.275	3.9E-01
Mtr.42241.1.S1_at	10.96	6.67	8.92	9.73	7.33	9.67	8.85	2.15	8.91	1.37	1.01	0.010	9.7E-01
Mtr.1702.1.S1_at	7.69	4.19	8.08	8.03	7.67	8.11	6.65	2.14	7.94	0.23	1.19	0.255	3.6E-01
Mtr.32106.1.S1_at	7.63	3.63	6.95	7.15	7.31	7.24	6.07	2.14	7.23	0.08	1.19	0.254	4.0E-01
Mtr.12143.1.S1_at	7.04	3.39	7.15	8.14	8.06	8.21	5.86	2.14	8.14	0.08	1.39	0.473	1.4E-01
Mtr.51408.1.S1_at	7.13	3.31	6.89	5.89	6.85	6.25	5.77	2.14	6.33	0.49	1.10	0.133	6.8E-01
Mtr.32387.1.S1_s_at	5.79	2.28	6.16	6.09	6.26	6.34	4.74	2.14	6.23	0.13	1.31	0.394	3.0E-01
Mtr.38697.1.S1_at	7.39	3.59	7.19	7.24	7.26	7.45	6.05	2.14	7.31	0.12	1.21	0.272	3.7E-01
Mtr.21905.1.S1_at	6.50	9.81	10.49	7.41	7.04	7.90	8.93	2.14	7.45	0.43	0.83	-0.263	3.0E-01
Mtr.304.1.S1_s_at	2.57	6.26	2.55	2.54	2.54	2.54	3.79	2.14	2.54	0.00	0.67	-0.577	3.7E-01
Mtr.676.1.S1_at	3.03	6.71	2.99	2.98	2.98	2.94	4.25	2.14	2.97	0.02	0.70	-0.519	3.6E-01
Mtr.8869.1.S1_at	8.25	4.09	7.04	8.15	7.54	8.30	6.46	2.14	8.00	0.41	1.24	0.308	2.9E-01
Mtr.16074.1.S1_at	7.41	3.46	6.86	7.22	7.43	7.50	5.91	2.14	7.38	0.14	1.25	0.321	3.0E-01
Mtr.16056.1.S1_at	3.78	7.46	3.74	3.72	3.71	3.70	4.99	2.14	3.71	0.01	0.74	-0.428	3.6E-01
Mtr.19846.1.S1_at	6.72	2.78	6.17	6.72	6.20	6.40	5.22	2.13	6.44	0.26	1.23	0.303	3.8E-01
Mtr.15769.1.S1_at	3.65	3.59	7.31	6.80	5.63	6.62	4.85	2.13	6.35	0.63	1.31	0.387	3.1E-01
Mtr.28337.1.S1_at	2.35	6.05	2.38	2.31	2.31	2.34	3.59	2.13	2.32	0.01	0.65	-0.629	3.6E-01
Mtr.33389.1.S1_at	6.79	3.08	6.75	6.71	6.90	6.53	5.54	2.13	6.71	0.19	1.21	0.278	4.0E-01
Mtr.33092.1.S1_at	5.91	2.29	6.04	6.17	5.70	5.88	4.75	2.13	5.92	0.24	1.25	0.318	4.0E-01
Mtr.42669.1.S1_s_at	7.02	3.51	7.36	6.65	6.91	6.26	5.97	2.13	6.61	0.33	1.11	0.147	6.3E-01
Mtr.40008.1.S1_at	7.90	4.76	8.82	7.59	7.45	7.68	7.16	2.13	7.58	0.11	1.06	0.081	7.5E-01
Mtr.27130.1.S1_s_at	8.49	4.29	6.96	7.48	7.25	7.70	6.58	2.13	7.48	0.22	1.14	0.185	5.1E-01
Mtr.44341.1.S1_at	8.10	4.05	7.20	7.10	7.37	7.54	6.45	2.13	7.34	0.22	1.14	0.185	5.1E-01
Mtr.12707.1.S1_at	7.24	3.14	6.18	7.34	7.35	7.11	5.52	2.13	7.26	0.14	1.32	0.396	2.3E-01
Mtr.51718.1.S1_at	8.11	4.57	8.37	8.76	8.67	8.58	7.02	2.13	8.67	0.09	1.24	0.305	2.5E-01
Mtr.49343.1.S1_at	9.65	6.11	9.92	9.58	9.82	9.71	8.56	2.12	9.70	0.12	1.13	0.181	4.0E-01
Mtr.18127.1.S1_at	9.00	4.76	7.02	7.07	6.70	7.25	6.93	2.12	7.01	0.28	1.01	0.017	9.5E-01
Mtr.45935.1.S1_at	9.88	6.90	11.02	9.16	8.97	9.17	9.27	2.12	9.10	0.11	0.98	-0.026	9.0E-01
Mtr.20751.1.S1_at	6.82	2.73	5.75	5.42	6.18	6.34	5.10	2.12	5.98	0.49	1.17	0.230	5.2E-01
Mtr.9913.1.S1_s_at	9.59	5.36	7.72	8.73	8.61	8.60	7.55	2.12	8.65	0.07	1.14	0.195	4.2E-01
Mtr.43572.1.S1_at	11.10	7.88	11.87	12.35	11.90	12.63	10.28	2.12	12.29	0.37	1.20	0.258	1.8E-01
Mtr.18815.1.S1_at	9.52	5.62	9.03	9.67	9.69	9.68	8.06	2.12	9.68	0.01	1.20	0.265	2.6E-01
Mtr.33770.1.S1_at	5.59	2.43	6.46	6.14	5.82	6.31	4.82	2.12	6.09	0.25	1.26	0.336	3.6E-01
Mtr.48353.1.S1_s_at	6.88	10.07	10.89	7.90	6.98	8.06	9.28	2.12	7.65	0.58	0.82	-0.279	2.7E-01
Mtr.29493.1.S1_at	3.73	6.87	2.85	3.71	3.64	3.71	4.49	2.11	3.68	0.04	0.82	-0.284	5.5E-01
Mtr.7850.1.S1_s_at	5.83	2.74	6.78	6.22	6.57	5.58	5.11	2.11	6.13	0.50	1.20	0.260	4.7E-01
Mtr.48824.1.S1_at	7.30	3.49	6.97	6.64	6.36	5.91	5.92	2.11	6.30	0.37	1.06	0.090	7.7E-01
Mtr.44023.1.S1_at	8.29	4.39	7.74	8.63	8.68	8.82	6.81	2.11	8.71	0.10	1.28	0.356	1.9E-01
Mtr.4407.1.S1_at	8.13	4.15	7.36	8.14	7.61	7.54	6.55	2.11	7.76	0.33	1.19	0.246	3.8E-01
Mtr.9416.1.S1_at	8.48	4.87	8.57	7.79	7.83	7.52	7.31	2.11	7.71	0.17	1.06	0.078	7.6E-01
Mtr.562.1.S1_at	5.97	2.75	6.71	5.96	6.18	5.65	5.14	2.11	5.93	0.27	1.15	0.205	5.6E-01
Mtr.50377.1.S1_s_at	7.69	3.81	4.33	6.83	6.60	6.62	5.28	2.11	6.68	0.13	1.27	0.340	3.1E-01
Mtr.27594.1.S1_at	1.87	1.92	5.54	5.53	3.90	5.07	3.11	2.11	4.83	0.84	1.55	0.635	2.6E-01
Mtr.13535.1.S1_at	7.56	3.44	6.26	6.86	7.30	6.83	5.76	2.11	7.00	0.26	1.22	0.281	3.7E-01
Mtr.18398.1.S1_at	7.02	3.26	6.80	6.67	6.21	5.42	5.69	2.11	6.10	0.63	1.07	0.099	7.7E-01
Mtr.8630.1.S1_at	10.65	7.02	10.68	11.04	11.07	11.47	9.45	2.10	11.20	0.24	1.18	0.244	2.3E-01
Mtr.39928.1.S1_at	6.37	2.95	6.78	6.58	6.69	6.41	5.37	2.10	6.56	0.14	1.22	0.289	3.8E-01
Mtr.7226.1.S1_at	6.82	2.73	5.63	7.37	7.90	7.96	5.06	2.10	7.74	0.33	1.53	0.614	9.4E-02
Mtr.36477.1.S1_at	3.14	6.66	2.91	2.89	2.82	2.93	4.24	2.10	2.88	0.05	0.68	-0.556	3.3E-01
Mtr.40190.1.S1_at	9.95	6.20	9.71	11.19	11.59	11.62	8.62	2.10	11.47	0.24	1.33	0.412	8.0E-02
Mtr.38735.1.S1_at	8.24	4.22	7.27	7.68	8.10	7.78	6.58	2.10	7.86	0.22	1.19	0.256	3.5E-01
Mtr.13683.1.S1_at	7.44	3.30	5.91	7.46	6.75	7.19	5.55	2.10	7.13	0.36	1.29	0.362	2.7E-01
Mtr.31707.1.S1_at	7.55	4.08	7.86	7.81	7.77	7.50	6.50	2.10	7.69	0.17	1.18	0.243	3.8E-01
Mtr.35988.1.S1_at	8.39	4.68	8.23	8.91	8.96	8.60	7.10	2.10	8.82	0.19	1.24	0.313	2.3E-01
Mtr.583.1.S1_at	7.73	3.81	7.05	7.08	6.74	6.70	6.20	2.10	6.84	0.21	1.10	0.142	6.3E-01
Mtr.21401.1.S1_at	6.91	3.31	6.98	7.00	7.08	6.78	5.73	2.10	6.96	0.15	1.21	0.279	3.7E-01
Mtr.15321.1.S1_at	7.35	3.43	6.68	7.65	7.84	7.65	5.82	2.09	7.71	0.11	1.32	0.406	1.9E-01
Mtr.17844.1.S1_at	8.24	4.33	7.59	8.39	8.44	8.73	6.72	2.09	8.52	0.18	1.27	0.342	2.1E-01
Mtr.9263.1.S1_at	7.03	3.50	7.21	6.62	7.08	6.81	5.91	2.09	6.84	0.23	1.16	0.209	4.9E-01
Mtr.47545.1.S1_x_at	3.32	6.91	3.25	3.25	1.60	3.25	4.49	2.09	2.70	0.95	0.60	-0.736	2.5E-01
Mtr.51317.1.S1_at	2.62	6.23	2.60	2.58	2.56	2.58	3.82	2.09	2.58	0.01	0.67	-0.567	3.6E-01

Mtr.42985.1.S1_at	12.87	10.10	14.20	14.59	14.54	14.75	12.39	2.09	14.63	0.11	1.18	0.239	1.4E-01
Mtr.20650.1.S1_at	7.21	3.22	6.27	6.29	5.94	6.04	5.57	2.09	6.09	0.18	1.09	0.130	6.9E-01
Mtr.27569.1.S1_s_at	6.62	3.59	7.58	7.29	7.49	6.69	5.93	2.08	7.16	0.41	1.21	0.272	3.7E-01
Mtr.19314.1.S1_s_at	3.08	6.54	2.80	2.79	2.66	2.83	4.14	2.08	2.76	0.09	0.67	-0.586	3.1E-01
Mtr.9988.1.S1_at	10.18	6.35	9.68	10.49	10.32	10.19	8.73	2.08	10.33	0.15	1.18	0.243	2.6E-01
Mtr.50417.1.S1_at	5.70	2.31	6.10	5.61	5.73	6.03	4.70	2.08	5.79	0.22	1.23	0.299	4.2E-01
Mtr.19497.1.S1_at	3.58	7.19	3.59	3.86	4.02	3.85	4.79	2.08	3.91	0.09	0.82	-0.293	5.0E-01
Mtr.980.1.S1_at	2.40	5.99	2.38	2.37	2.36	2.35	3.59	2.08	2.36	0.01	0.66	-0.605	3.6E-01
Mtr.42259.1.S1_at	6.14	2.59	6.23	6.15	5.86	6.33	4.99	2.08	6.12	0.24	1.23	0.294	4.0E-01
Mtr.12648.1.S1_at	2.86	6.48	2.90	2.88	2.90	2.80	4.08	2.08	2.86	0.05	0.70	-0.512	3.7E-01
Mtr.2806.1.S1_at	2.24	5.83	2.22	2.21	2.22	2.21	3.43	2.08	2.21	0.01	0.64	-0.633	3.7E-01
Mtr.9188.1.S1_at	8.11	3.99	6.45	6.04	7.28	6.28	6.18	2.08	6.53	0.66	1.06	0.079	7.9E-01
Mtr.2162.1.S1_at	6.82	3.07	6.48	6.82	6.69	7.05	5.46	2.08	6.85	0.18	1.26	0.328	3.1E-01
Mtr.46962.1.S1_at	8.14	4.26	7.49	8.28	8.33	8.23	6.63	2.08	8.28	0.05	1.25	0.320	2.4E-01
Mtr.27103.1.S1_at	6.11	3.44	7.53	7.28	6.91	7.56	5.69	2.07	7.25	0.32	1.27	0.349	2.7E-01
Mtr.44569.1.S1_at	5.63	4.04	8.16	7.69	8.46	7.51	5.94	2.07	7.89	0.50	1.33	0.408	1.9E-01
Mtr.30415.1.S1_at	6.71	3.11	6.69	8.11	8.49	8.44	5.50	2.07	8.35	0.21	1.52	0.601	7.7E-02
Mtr.5081.1.S1_at	9.25	5.38	8.59	9.59	9.76	9.61	7.74	2.07	9.65	0.09	1.25	0.319	1.8E-01
Mtr.41967.1.S1_at	7.47	3.36	5.88	6.42	6.16	6.43	5.57	2.07	6.34	0.15	1.14	0.186	5.6E-01
Mtr.39729.1.S1_at	7.28	3.82	7.53	7.64	7.53	7.55	6.21	2.07	7.57	0.06	1.22	0.286	3.2E-01
Mtr.42113.1.S1_at	4.70	8.25	8.32	11.14	11.33	11.12	7.09	2.07	11.19	0.11	1.58	0.659	2.7E-02
Mtr.41215.1.S1_at	10.24	6.78	10.47	10.34	10.10	10.16	9.16	2.07	10.20	0.12	1.11	0.154	4.4E-01
Mtr.5391.1.S1_s_at	6.40	3.13	6.95	6.98	6.62	6.82	5.49	2.07	6.80	0.18	1.24	0.309	3.4E-01
Mtr.32084.1.S1_at	9.52	5.70	8.98	9.19	9.81	9.43	8.07	2.07	9.48	0.31	1.18	0.233	3.1E-01
Mtr.8501.1.S1_s_at	7.81	4.11	7.57	7.73	7.51	7.57	6.50	2.07	7.60	0.11	1.17	0.227	4.1E-01
Mtr.12742.1.S1_at	2.99	6.59	3.03	2.93	2.93	2.92	4.21	2.07	2.93	0.01	0.70	-0.523	3.4E-01
Mtr.27537.1.S1_at	2.00	5.57	1.99	1.98	1.96	1.98	3.19	2.06	1.97	0.01	0.62	-0.690	3.7E-01
Mtr.48998.1.S1_at	7.54	3.93	7.46	8.10	7.79	7.84	6.31	2.06	7.91	0.17	1.25	0.326	2.5E-01
Mtr.6762.1.S1_at	6.80	3.32	6.97	6.91	6.91	6.82	5.70	2.06	6.88	0.05	1.21	0.272	3.8E-01
Mtr.39454.1.S1_at	8.22	4.55	8.02	8.18	7.00	8.17	6.93	2.06	7.79	0.68	1.12	0.168	5.3E-01
Mtr.46385.1.S1_x_at	2.41	5.96	2.38	2.38	2.37	2.37	3.58	2.06	2.37	0.00	0.66	-0.594	3.7E-01
Mtr.29221.1.S1_at	1.89	5.45	1.88	1.88	1.88	1.88	3.07	2.06	1.88	0.00	0.61	-0.710	3.7E-01
Mtr.37153.1.S1_at	7.32	3.56	6.89	6.97	7.39	6.80	5.92	2.06	7.05	0.30	1.19	0.252	4.0E-01
Mtr.52074.1.S1_at	7.74	3.63	5.51	5.88	5.37	5.98	5.62	2.06	5.74	0.33	1.02	0.030	9.3E-01
Mtr.4052.1.S1_at	7.79	4.29	7.90	7.81	7.58	7.48	6.66	2.06	7.62	0.17	1.14	0.195	4.6E-01
Mtr.42038.1.S1_s_at	8.03	4.37	7.83	8.93	8.32	8.94	6.74	2.05	8.73	0.36	1.29	0.372	1.7E-01
Mtr.38010.1.S1_at	3.21	2.74	6.51	7.24	7.48	7.57	4.16	2.05	7.43	0.17	1.79	0.839	5.1E-02
Mtr.51597.1.S1_at	6.32	2.85	6.49	6.43	6.33	5.58	5.22	2.05	6.11	0.47	1.17	0.228	5.0E-01
Mtr.32965.1.S1_at	7.21	3.45	6.75	6.44	5.77	6.17	5.80	2.05	6.13	0.33	1.06	0.078	8.0E-01
Mtr.51690.1.S1_at	2.87	6.39	2.81	2.79	2.80	2.74	4.02	2.05	2.78	0.03	0.69	-0.535	3.5E-01
Mtr.21790.1.S1_at	8.06	4.35	7.70	7.24	7.12	6.21	6.70	2.05	6.85	0.56	1.02	0.032	9.1E-01
Mtr.27695.1.S1_at	6.55	3.24	6.97	7.64	7.45	7.20	5.59	2.05	7.43	0.22	1.33	0.411	2.0E-01
Mtr.45364.1.S1_at	6.41	3.01	6.68	6.50	6.59	6.94	5.37	2.04	6.68	0.23	1.24	0.315	3.3E-01
Mtr.20710.1.S1_at	10.13	6.30	6.98	7.25	8.11	7.91	7.81	2.04	7.76	0.45	0.99	-0.009	9.7E-01
Mtr.37931.1.S1_at	7.96	4.06	7.06	7.59	8.45	8.27	6.36	2.04	8.11	0.45	1.27	0.350	2.2E-01
Mtr.36214.1.S1_at	2.43	5.96	2.41	2.41	2.39	2.65	3.60	2.04	2.48	0.14	0.69	-0.536	4.0E-01
Mtr.50087.1.S1_at	7.63	3.58	6.09	6.96	6.45	6.79	5.77	2.04	6.73	0.26	1.17	0.223	4.6E-01
Mtr.36854.1.S1_a_at	6.98	2.90	5.15	7.24	7.42	7.53	5.01	2.04	7.40	0.15	1.48	0.563	1.1E-01
Mtr.24422.1.S1_at	5.94	4.41	8.45	7.35	7.74	5.82	6.27	2.04	6.97	1.02	1.11	0.153	6.2E-01
Mtr.13929.1.S1_at	6.50	3.26	7.04	7.02	6.73	6.47	5.60	2.04	6.74	0.27	1.20	0.267	3.9E-01
Mtr.7068.1.S1_at	5.67	2.85	6.81	6.22	6.42	5.97	5.11	2.04	6.20	0.22	1.21	0.280	4.1E-01
Mtr.39830.1.S1_at	8.82	4.96	8.05	8.85	8.85	9.07	7.28	2.04	8.92	0.13	1.23	0.294	2.4E-01
Mtr.40222.1.S1_at	6.25	2.86	6.53	6.92	7.55	7.22	5.21	2.04	7.23	0.32	1.39	0.471	1.7E-01
Mtr.47322.1.S1_s_at	2.21	5.73	2.19	2.19	2.32	2.06	3.38	2.04	2.19	0.13	0.65	-0.626	3.7E-01
Mtr.14223.1.S1_at	5.40	2.34	6.20	6.37	6.56	6.24	4.65	2.04	6.39	0.16	1.38	0.460	2.1E-01
Mtr.5253.1.S1_s_at	9.46	5.92	9.44	9.41	9.49	9.52	8.27	2.04	9.47	0.06	1.14	0.195	3.7E-01
Mtr.23539.1.S1_at	2.12	5.63	2.11	2.10	2.10	2.10	3.29	2.03	2.10	0.00	0.64	-0.647	3.7E-01
Mtr.6855.1.S1_at	5.86	2.73	6.55	5.61	5.79	5.52	5.05	2.03	5.64	0.14	1.12	0.160	6.4E-01
Mtr.29075.1.S1_at	3.96	3.40	7.17	7.04	6.45	7.22	4.84	2.03	6.91	0.40	1.43	0.512	1.6E-01
Mtr.7120.1.S1_s_at	6.86	2.85	5.40	5.38	5.66	5.60	5.03	2.03	5.55	0.15	1.10	0.140	6.9E-01
Mtr.14827.1.S1_at	2.49	5.99	2.45	2.45	2.45	2.44	3.64	2.03	2.44	0.01	0.67	-0.576	3.6E-01
Mtr.10842.1.S1_at	5.60	4.14	8.15	8.00	8.86	8.61	5.96	2.03	8.49	0.44	1.42	0.509	1.0E-01
Mtr.1398.1.S1_s_at	8.73	4.86	7.84	9.06	9.01	8.23	7.14	2.03	8.77	0.46	1.23	0.296	2.5E-01
Mtr.7176.1.S1_at	3.62	7.07	3.50	3.52	3.51	3.51	4.73	2.03	3.51	0.00	0.74	-0.428	3.6E-01
Mtr.37276.1.S1_at	7.91	3.95	6.71	7.78	7.78	7.65	6.19	2.03	7.73	0.08	1.25	0.321	2.6E-01
Mtr.10927.1.S1_at	9.78	7.87	5.72	9.03	8.45	9.45	7.79	2.03	8.98	0.50	1.15	0.205	3.8E-01
Mtr.13280.1.S1_at	6.07	2.96	6.76	6.47	7.05	6.56	5.26	2.03	6.69	0.31	1.27	0.346	2.9E-01
Mtr.51237.1.S1_a_at	6.85	3.21	6.56	5.50	6.36	5.12	5.54	2.03	5.66	0.64	1.02	0.032	9.3E-01
Mtr.34096.1.S1_at	2.08	5.58	2.07	2.09	2.06	2.06	3.24	2.02	2.07	0.02	0.64	-0.647	3.7E-01
Mtr.33423.1.S1_at	7.85	4.28	7.72	8.11	8.50	7.93	6.61	2.02	8.18	0.29	1.24	0.306	2.6E-01
Mtr.32341.1.S1_at	8.14	4.60	8.07	7.97	7.80	8.10	6.94	2.02	7.96	0.15	1.15	0.198	4.3E-01
Mtr.31745.1.S1_at	10.83	6.94	9.85	10.51	10.97	10.49	9.21	2.02	10.66	0.27	1.16	0.211	2.9E-01
Mtr.47387.1.S1_at	6.61	3.29	6.95	6.90	7.32	6.77	5.62	2.02	7.00	0.29	1.25	0.317	3.1E-01
Mtr.44703.1.S1_at	6.50	2.73	3.37	3.65	2.78	3.27	4.20	2.02	3.23	0.43	0.77	-0.378	4.6E-01
Mtr.39749.1.S1_s_at	6.65	3.11	6.58	6.50	6.29	6.26	5.45	2.02	6.35	0.13	1.17	0.222	4.8E-01
Mtr.1067.1.S1_x_at	3.85	7.30	3.74	3.80	3.60	3.78	4.96	2.02	3.73	0.11	0.75	-0.413	3.5E-01
Mtr.10175.1.S1_at	6.80	3.74	7.55	7.64	6.77	7.14	6.03	2.02	7.18	0.44	1.19	0.252	3.9E-01
Mtr.32430.1.S1_s_at	7.57	4.03	7.48	7.26	7.81	7.33	6.36	2.02	7.47	0.30	1.17	0.231	4.0E-01
Mtr.27441.1.S1_at	2.11	5.61	2.10	2.18	2.09	2.09	3.27	2.02	2.12	0.05	0.65	-0.626	3.8E-01
Mtr.41216.1.S1_s_at	8.50	4.63	7.57	8.30	8.17	8.43	6.90	2.02	8.30	0.13	1.20	0.266	3.0E-01
Mtr.5031.1.S1_at	2.82	6.28	2.76	2.78	2.79	2.69	3.95	2.02	2.75	0.06	0.70	-0.522	3.6E-01
Mtr.15888.1.S1_at	7.08	3.83	7.53	7.68	7.36	6.98	6.15	2.02	7.34	0.35	1.19	0.256	3.7E-01
Mtr.2661.1.S1_at	7.36	3.67	6.94	7.29	5.85	6.91	5.99	2.02	6.68	0.75	1.12	0.157	6.1E-01
Mtr.19273.1.S1_at	7.70	4.42	8.10	8.02	7.85	7.89	6.74	2.02	7.92	0.09	1.17	0.232	3.7E-01
Mtr.7861.1.S1_at	2.84	6.32	2.83	3.51	2.74	2.81	4.00	2.01	3.02	0.43	0.76	-0.404	4.6E-01
Mtr.23150.1.S1_at	7.13	4.33	8.23	8.08	7.96	7.68	6.56	2.01	7.91	0.20	1.20	0.268	3.2E-01
Mtr.34323.1.S1_at	1.96	5.45	1.95	1.95	1.95	1.95	3.12	2.01	1.95	0.00	0.62	-0.679	3.7E-01
Mtr.20308.1.S1_at	6.26	2.39	5.31	6.26	6.64	6.17	4.65	2.01	6.36	0.25	1.37	0.451	2.2E-01
Mtr.8496.1.S1_at	8.70	4.68	6.47	7.72	7.96	7.43	6.62	2.01	7.70	0.27	1.16	0.219	4.1E-01
Mtr.4063.1.S1_at	7.44	3.49	6.12	6.89	7.23	6.84	5.68	2.01	6.99	0.21	1.23	0.298	3.3E-01
Mtr.17034.1.S1_s_at	3.45	6.88	3.37	3.39	3.17	3.37	4.57	2.01	3.31	0.12	0.72	-0.465	3.4E-01
Mtr.9311.1.S1_at	5.16	5.96	8.97	8.50	6.95	8.16	6.70	2.01	7.87	0.81	1.18	0.233	4.0E-01
Mtr.48573.1.S1_at	6.78	3.60	7.31	6.56	5.78	5.86	5.89	2.01	6.07	0.43	1.03	0.042	8.9E-01
Mtr.30436.1.S1_at	2.81	6.26	2.76	2.73	2.70	2.74	3.94	2.01	2.72	0.02	0.69	-0.534	3.5E-01
Mtr.9401.1.S1_at	6.58	3.84	7.75	8.86	9.65	9.20	6.06	2.01	9.24	0.40	1.53	0.609	5.4E-02
Mtr.40476.1.S1_at	7.02	3.24	6.28	7.03	8.28	8.07	5.51	2.00	7.79	0.67	1.41	0.500	1.3E-01
Mtr.44737.1.S1_at	8.90	5.09	8.07	8.31	7.85	8.18	7.36	2.00	8.11	0.23	1.10	0.141	5.5E-01
Mtr.1577.1.S1_s_at	10.02	6.13	8.89	10.16	10.07	10.38	8.35	2.00	10.20	0.16	1.22	0.290	1.8E-01
Mtr.35966.1.S1_s_at	6.88	3.48	7.00	6.64	6.65	6.88	5.79	2.00	6.72	0.14	1.16	0.215	4.7E-01
Mtr.13579.1.S1_at	6.41	2.78	6.04	6.54	6.35	6.01	5.08	2.00	6.30	0.27	1.24	0.311	3.5E-01
Mtr.2727.1.S1_at	6.97	3.42	6.79	7.14	7.28	7.20	5.72	2.00	7.21	0.07	1.26	0.333	2.7E-01
Mtr.2742.1.S1_at	7.43	4.35	8.09	8.19	8.38	8.47	6.62	2.00	8.35	0.14	1.26	0.334	2.1E-01
Mtr.49862.1.S1_at	9.46	5.75	8.90	9.19	8.91	9.33	8.04	2.00	9.14	0.21	1.14	0.186	3.9E-01
Mtr.10188.1.S1_at	6.07	2.29	5.29	5.34	5.74	5.80	4.55	2.00	5.63	0.25	1.24	0.306	4.1E-01
Mtr.12540.1.S1_s_at	8.01	4.38	7.65	8.06	8.18	7.95	6.68	2.00	8.07	0.12	1.21	0.272	3.0E-01
Mtr.15498.1.S1_x_at	2.81	6.26	2.80	2.80	2.67	2.79	3.96	2.00	2.75	0.07	0.70	-0.525	3.5E-01
Mtr.14559.1.S1_at	3.24	6.58	3.03	3.27	3.17	3.17	4.28	1.99	3.20	0.06	0.75	-0.421	4.0E-01
Mtr.35377.1.S1_at	3.65	7.10	3.64	3.64	3.64	3.64	4.79	1.99	3.64	0.00	0.76	-0.399	3.7E-01
Mtr.25.1.S1_at	3.26	6.67	3.18	3.16	3.03	3.11	4.37	1.99	3.10	0.07	0.71	-0.494	3.3E-01
Mtr.20782.1.S1_at	6.77	3.10	6.28	6.14	6.06	6.04	5.38	1.99	6.08	0.05	1.13	0.175	5.8E-01
Mtr.12499.1.S1_s_at	11.14	9.66	7.20	9.90	9.79	10.41	9.33	1.99	10.03	0.33	1.08	0.104	5.8E-01
Mtr.24936.1.S1_at	2.43	5.88	2.42	2.41	2.32	2.42	3.58	1.99	2.38	0.05	0.67	-0.585	3.6E-01
Mtr.41473.1.S1_at	6.88	3.67	7.30	7.04	6.91	7.15	5.95	1.99	7.03	0.12	1.18	0.241	4.0E-01
Mtr.44419.1.S1_at	6.94	3.52	6.98	6.49	6.54	6.89	5.82	1.99	6.64	0.22	1.14	0.191	5.1E-01
Mtr.41370.1.S1_at	7.90	4.87	8.60	7.86	7.86	7.78	7.13	1.98	7.83	0.04	1.10	0.137	5.7E-01
Mtr.42231.1.S1_at	5.51	3.76	7.71	8.09	9.23	8.06	5.66	1.98	8.46	0.67	1.49	0.580	8.1E-02
Mtr.6537.1.S1_s_at	6.83	3.57	7.14	7.42	7.90	7.65	5.85	1.98	7.66	0.24	1.31	0.390	1.9E-01
Mtr.46278.1.S1_at	2.41	5.83	2.39	2.38	2.37	2.36	3.54	1.98	2.37	0.01	0.67	-0.578	3.6E-01
Mtr.7567.1.S1_at	7.05	3.37	6.46	6.59	6.46	6.47	5.63	1.98	6.51	0.07	1.16	0.210	4.8E-01
Mtr.27256.1.S1_at	6.37	2.86	6.19	6.16	6.35	6.19	5.14	1.98	6.23	0.10	1.21	0.278	3.9E-01
Mtr.44523.1.S1_at	5.53	2.07	5.46	5.95	6.16	6.11	4.35	1.98	6.07	0.11	1.39	0.480	2.1E-01
Mtr.34172.1.S1_at	2.00	5.42	1.99	2.35	1.98	1.98	3.14	1.98	2.10	0.21	0.67	-0.575	4.2E-01
Mtr.29910.1.S1_at	6.61	3.03	6.28	5.79	6.43	6.16	5.31	1.98	6.13	0.32	1.15	0.207	5.2E-01
Mtr.32723.1.S1_at	7.39	3.81	7.04	6.43	5.66	6.34	6.08	1.98	6.14	0.42	1.01	0.014	9.6E-01
Mtr.36358.1.S1_at	2.28	5.69	2.27	2.26	2.26	2.26	3.41	1.97	2.26	0.01	0.66	-0.594	3.7E-01
Mtr.11294.1.S1_at	7.21	3.46	6.39	6.98	6.72	6.88	5.68	1.97	6.86	0.13	1.21	0.271	3.6E-01
Mtr.44560.1.S1_at	6.88	3.40	6.75	6.99	7.27	6.82	5.68	1.97	7.03	0.23	1.24	0.307	3.0E-01
Mtr.32011.1.S1_at	6.70	3.55	7.19	8.64	8.45	9.02	5.81	1.97	8.70	0.29	1.50	0.582	6.6E-02
Mtr.23572.1.S1_at	7.74	3.94	6.74	7.18	7.26	7.12	6.14	1.97	7.18	0.07	1.17	0.227	4.1E-01
Mtr.31140.1.S1_at	5.95	3.14	6.93	6.43	6.46	6.03	5.34	1.97	6.31	0.24	1.18	0.239	4.5E-01
Mtr.27090.1.S1_at	5.12	2.74	6.64	5.76	5.63	5.75	4.83	1.97	5.71	0.07	1.18	0.241	4.8E-01

Mtr.25409.1.S1_s_at	6.75	3.42	6.89	7.29	7.06	6.93	5.69	1.96	7.09	0.18	1.25	0.319	2.8E-01
Mtr.10553.1.S1_at	9.29	5.53	8.41	8.20	8.05	8.35	7.74	1.96	8.20	0.15	1.06	0.082	7.1E-01
Mtr.9383.1.S1_at	5.23	1.96	5.48	5.79	5.94	5.55	4.22	1.96	5.76	0.20	1.36	0.449	2.5E-01
Mtr.40553.1.S1_at	11.86	8.81	12.47	12.57	11.64	12.52	11.05	1.96	12.24	0.52	1.11	0.148	3.7E-01
Mtr.44935.1.S1_at	2.50	2.49	5.89	5.89	5.01	5.91	3.63	1.96	5.60	0.52	1.54	0.627	1.7E-01
Mtr.38820.1.S1_at	5.01	6.52	8.90	8.97	8.40	9.02	6.81	1.96	8.80	0.34	1.29	0.369	1.6E-01
Mtr.10054.1.S1_at	7.82	3.99	6.62	7.34	7.43	7.29	6.14	1.96	7.35	0.07	1.20	0.260	3.4E-01
Mtr.46192.1.S1_x_at	2.88	6.27	2.88	2.86	2.79	2.87	4.01	1.96	2.84	0.04	0.71	-0.495	3.6E-01
Mtr.10159.1.S1_at	6.67	3.15	6.40	6.58	6.45	6.62	5.41	1.96	6.55	0.09	1.21	0.276	3.7E-01
Mtr.11975.1.S1_at	10.06	6.37	9.35	9.98	9.91	9.95	8.60	1.96	9.95	0.04	1.16	0.211	3.0E-01
Mtr.10710.1.S1_at	2.84	6.21	2.80	2.78	2.76	2.76	3.95	1.96	2.76	0.01	0.70	-0.514	3.5E-01
Mtr.31600.1.S1_at	6.31	2.56	5.39	5.71	6.08	5.65	4.75	1.96	5.81	0.23	1.22	0.291	4.0E-01
Mtr.14580.1.S1_at	6.43	3.64	7.40	6.42	6.54	6.54	5.83	1.95	6.50	0.07	1.12	0.158	5.8E-01
Mtr.28665.1.S1_at	5.33	2.21	5.79	5.34	5.34	5.34	4.44	1.95	5.34	0.00	1.20	0.264	4.7E-01
Mtr.25786.1.S1_at	6.57	2.68	4.74	6.26	6.14	6.77	4.66	1.95	6.39	0.34	1.37	0.455	2.0E-01
Mtr.40975.1.S1_at	8.97	6.91	10.81	10.55	11.07	10.55	8.90	1.95	10.72	0.30	1.20	0.269	1.8E-01
Mtr.11183.1.S1_at	6.44	2.76	5.70	6.08	5.69	5.57	4.97	1.95	5.78	0.26	1.16	0.219	5.1E-01
Mtr.47073.1.S1_at	3.14	6.49	3.11	3.09	3.07	3.08	4.25	1.95	3.08	0.01	0.72	-0.465	3.6E-01
Mtr.32162.1.S1_at	6.81	3.44	3.44	3.41	3.40	3.72	4.56	1.95	3.51	0.18	0.77	-0.378	4.0E-01
Mtr.20542.1.S1_at	2.61	5.96	2.58	2.57	2.55	2.57	3.72	1.95	2.56	0.01	0.69	-0.536	3.6E-01
Mtr.1248.1.S1_at	3.00	6.11	2.53	2.47	2.47	2.63	3.88	1.95	2.52	0.10	0.65	-0.622	2.9E-01
Mtr.26943.1.S1_at	2.05	5.42	2.04	2.04	2.04	2.08	3.17	1.95	2.05	0.02	0.65	-0.628	3.8E-01
Mtr.27752.1.S1_at	4.32	7.83	4.62	4.48	4.48	4.46	5.59	1.94	4.47	0.01	0.80	-0.322	3.8E-01
Mtr.13067.1.S1_at	4.52	2.19	6.04	6.00	4.95	5.24	4.25	1.94	5.40	0.54	1.27	0.344	3.8E-01
Mtr.37594.1.S1_at	6.24	3.47	7.22	7.89	8.57	8.39	5.64	1.94	8.29	0.35	1.47	0.554	8.1E-02
Mtr.33154.1.S1_at	3.06	6.41	3.03	3.32	3.01	2.99	4.17	1.94	3.11	0.19	0.74	-0.425	4.0E-01
Mtr.41520.1.S1_at	7.26	3.84	7.14	7.23	7.28	7.29	6.08	1.94	7.27	0.03	1.19	0.257	3.5E-01
Mtr.5125.1.S1_s_at	4.68	2.28	6.11	5.00	4.71	4.66	4.36	1.94	4.79	0.19	1.10	0.137	7.2E-01
Mtr.9283.1.S1_at	6.01	2.88	6.42	5.99	5.99	5.98	5.10	1.94	5.99	0.01	1.17	0.232	4.7E-01
Mtr.49688.1.S1_at	3.47	3.25	6.71	7.78	8.39	7.72	4.48	1.94	7.96	0.37	1.78	0.831	3.8E-02
Mtr.40657.1.S1_at	8.43	4.87	7.96	8.73	8.81	8.68	7.09	1.93	8.74	0.06	1.23	0.302	2.1E-01
Mtr.9980.1.S1_at	7.72	3.86	5.69	5.13	5.62	5.85	5.76	1.93	5.53	0.37	0.96	-0.058	8.5E-01
Mtr.9069.1.S1_at	6.63	3.33	6.72	6.62	7.07	6.46	5.56	1.93	6.72	0.32	1.21	0.273	3.6E-01
Mtr.33509.1.S1_at	2.04	5.38	2.03	2.02	2.02	2.03	3.15	1.93	2.02	0.01	0.64	-0.638	3.7E-01
Mtr.916.1.S1_s_at	7.73	4.66	8.22	7.57	7.76	7.19	6.87	1.93	7.51	0.29	1.09	0.128	6.0E-01
Mtr.22589.1.S1_at	7.10	3.58	6.71	6.53	6.13	5.82	5.79	1.93	6.16	0.36	1.06	0.088	7.6E-01
Mtr.5035.1.S1_at	5.63	2.10	5.22	5.66	5.45	5.97	4.31	1.93	5.69	0.26	1.32	0.399	2.9E-01
Mtr.51724.1.S1_at	9.88	8.47	12.29	9.77	10.73	10.70	10.21	1.93	10.40	0.54	1.02	0.026	8.8E-01
Mtr.22665.1.S1_at	6.35	3.19	6.69	6.28	6.72	6.61	5.41	1.93	6.54	0.23	1.21	0.274	3.7E-01
Mtr.17227.1.S1_at	7.54	4.48	8.04	7.43	7.22	7.35	6.68	1.93	7.34	0.11	1.10	0.134	5.9E-01
Mtr.5702.1.S1_at	6.25	3.28	6.90	7.01	6.59	6.79	5.48	1.93	6.80	0.21	1.24	0.311	3.0E-01
Mtr.42711.1.S1_at	7.02	3.39	6.33	6.84	6.87	6.50	5.58	1.93	6.74	0.20	1.21	0.272	3.6E-01
Mtr.46278.1.S1_s_at	2.61	5.94	2.61	2.58	2.53	2.56	3.72	1.93	2.56	0.02	0.69	-0.540	3.5E-01
Mtr.50904.1.S1_at	7.78	4.64	8.15	8.18	7.95	7.97	6.86	1.93	8.03	0.13	1.17	0.228	3.5E-01
Mtr.52006.1.S1_s_at	8.26	5.29	8.89	8.38	9.01	8.93	7.48	1.92	8.77	0.35	1.17	0.230	3.2E-01
Mtr.11511.1.S1_at	6.35	3.13	2.91	2.90	2.93	2.92	4.13	1.92	2.92	0.01	0.71	-0.502	3.4E-01
Mtr.51371.1.S1_at	2.43	5.75	2.41	2.40	2.40	2.40	3.53	1.92	2.40	0.00	0.68	-0.554	3.7E-01
Mtr.44741.1.S1_at	6.54	2.71	4.43	6.23	5.62	6.26	4.56	1.92	6.04	0.36	1.32	0.405	2.6E-01
Mtr.45559.1.S1_at	6.50	3.37	6.86	5.58	5.07	5.70	5.58	1.92	5.45	0.33	0.98	-0.034	9.1E-01
Mtr.4708.1.S1_at	7.06	3.27	4.65	6.19	5.77	6.27	4.99	1.92	6.08	0.27	1.22	0.284	3.9E-01
Mtr.10013.1.S1_at	8.10	4.76	8.06	8.14	7.77	8.11	6.97	1.91	8.01	0.21	1.15	0.200	4.0E-01
Mtr.7130.1.S1_at	6.69	9.22	5.47	5.69	7.15	6.32	7.13	1.91	6.39	0.73	0.90	-0.158	5.7E-01
Mtr.1211.1.S1_x_at	2.97	6.22	2.84	3.17	2.68	2.91	4.01	1.91	2.92	0.24	0.73	-0.458	3.8E-01
Mtr.27969.1.S1_at	6.15	2.41	3.59	4.18	3.71	4.71	4.05	1.91	4.20	0.50	1.04	0.051	9.0E-01
Mtr.11814.1.S1_at	8.10	4.74	7.99	8.00	8.01	7.94	6.94	1.91	7.98	0.04	1.15	0.202	4.0E-01
Mtr.37393.1.S1_at	13.65	10.02	12.89	12.89	12.39	12.93	12.19	1.91	12.74	0.30	1.05	0.064	6.5E-01
Mtr.21107.1.S1_x_at	2.48	5.78	2.46	2.44	2.43	2.43	3.57	1.91	2.43	0.01	0.68	-0.554	3.6E-01
Mtr.21053.1.S1_at	2.91	6.21	2.90	2.92	2.86	2.85	4.01	1.91	2.88	0.04	0.72	-0.478	3.6E-01
Mtr.46936.1.S1_at	2.75	6.07	2.78	2.72	2.73	2.72	3.87	1.91	2.72	0.01	0.70	-0.506	3.6E-01
Mtr.11996.1.S1_at	6.58	3.02	5.98	6.59	6.78	6.96	5.19	1.91	6.78	0.18	1.31	0.384	2.3E-01
Mtr.1.1.S1_at	7.86	10.69	7.06	7.43	7.13	7.47	8.53	1.91	7.34	0.19	0.86	-0.217	3.4E-01
Mtr.27306.1.S1_at	2.21	5.51	2.21	2.21	2.20	2.19	3.31	1.91	2.20	0.01	0.67	-0.587	3.7E-01
Mtr.36121.1.S1_at	2.63	5.91	2.59	2.59	2.57	2.59	3.71	1.91	2.58	0.01	0.70	-0.525	3.6E-01
Mtr.37175.1.S1_at	6.52	3.26	6.59	6.67	6.34	6.29	5.45	1.91	6.44	0.21	1.18	0.238	4.3E-01
Mtr.45773.1.S1_at	3.20	6.48	3.17	3.16	2.94	2.98	4.29	1.90	3.03	0.12	0.71	-0.502	3.2E-01
Mtr.46819.1.S1_at	6.30	2.80	5.85	6.02	6.68	6.51	4.98	1.90	6.41	0.34	1.29	0.362	2.7E-01
Mtr.20572.1.S1_x_at	6.92	3.37	6.35	5.63	3.82	4.64	5.55	1.90	4.70	0.91	0.85	-0.240	5.2E-01
Mtr.39535.1.S1_at	3.85	2.68	6.40	4.02	5.08	4.30	4.31	1.90	4.47	0.55	1.04	0.052	9.0E-01
Mtr.49075.1.S1_at	7.92	4.58	7.82	7.85	7.75	7.17	6.78	1.90	7.59	0.37	1.12	0.164	5.1E-01
Mtr.40163.1.S1_at	10.98	7.23	9.61	9.96	9.55	8.92	9.27	1.90	9.48	0.52	1.02	0.032	8.7E-01
Mtr.12145.1.S1_at	8.38	5.13	8.46	8.67	8.79	8.49	7.32	1.90	8.65	0.15	1.18	0.240	2.9E-01
Mtr.24856.1.S1_x_at	2.45	5.73	2.44	2.44	2.39	2.43	3.54	1.90	2.42	0.02	0.68	-0.550	3.6E-01
Mtr.10386.1.S1_at	6.58	9.18	5.49	7.04	6.28	6.74	7.08	1.90	6.69	0.39	0.94	-0.083	7.4E-01
Mtr.44121.1.S1_at	7.10	4.33	7.96	7.41	7.58	7.30	6.46	1.90	7.43	0.14	1.15	0.202	4.3E-01
Mtr.19182.1.S1_at	6.16	2.94	6.29	6.63	7.49	6.74	5.13	1.90	6.95	0.47	1.36	0.438	1.8E-01
Mtr.13167.1.S1_at	5.82	4.79	8.46	8.49	8.11	8.38	6.36	1.89	8.33	0.19	1.31	0.390	1.5E-01
Mtr.34229.1.S1_at	2.08	5.36	2.07	2.07	2.07	2.07	3.17	1.89	2.07	0.00	0.65	-0.616	3.7E-01
Mtr.17375.1.S1_at	2.54	5.79	2.49	2.49	2.49	2.96	3.61	1.89	2.65	0.27	0.73	-0.446	4.3E-01
Mtr.3072.1.S1_at	7.14	3.62	6.57	6.05	6.38	6.68	5.78	1.89	6.37	0.32	1.10	0.141	6.2E-01
Mtr.1670.1.S1_at	4.61	2.28	6.02	2.24	2.02	2.23	4.30	1.89	2.16	0.13	0.50	-0.991	1.2E-01
Mtr.27583.1.S1_at	6.28	3.01	6.27	6.22	6.36	6.29	5.19	1.89	6.29	0.07	1.21	0.278	3.7E-01
Mtr.36859.1.S1_at	7.13	3.83	7.06	6.47	7.06	6.54	6.01	1.89	6.69	0.32	1.11	0.155	5.7E-01
Mtr.41275.1.S1_at	7.45	4.39	7.84	7.64	7.68	7.49	6.56	1.89	7.61	0.10	1.16	0.213	3.9E-01
Mtr.1526.1.S1_at	7.81	4.05	6.28	6.95	7.75	7.27	6.05	1.89	7.32	0.41	1.21	0.276	3.2E-01
Mtr.45201.1.S1_at	7.27	4.01	7.29	7.23	7.21	7.34	6.19	1.89	7.26	0.07	1.17	0.230	3.8E-01
Mtr.18689.1.S1_at	2.69	5.94	2.66	2.63	2.61	2.61	3.76	1.89	2.62	0.01	0.70	-0.523	3.5E-01
Mtr.21154.1.S1_at	1.89	5.15	1.88	1.88	1.88	1.88	2.98	1.89	1.88	0.00	0.63	-0.663	3.7E-01
Mtr.4734.1.S1_at	8.57	5.50	8.93	9.15	9.86	9.24	7.67	1.88	9.42	0.39	1.23	0.297	1.9E-01
Mtr.38746.1.S1_at	6.73	3.05	4.20	6.16	6.58	6.54	4.66	1.88	6.43	0.23	1.38	0.463	1.8E-01
Mtr.5513.1.S1_s_at	6.19	2.70	5.69	3.28	3.64	2.68	4.86	1.88	3.20	0.48	0.66	-0.604	2.1E-01
Mtr.42371.1.S1_at	6.84	3.09	4.62	4.86	5.53	5.33	4.85	1.88	5.24	0.35	1.08	0.112	7.4E-01
Mtr.27568.1.S1_at	6.77	3.66	7.05	6.85	6.79	6.80	5.83	1.88	6.81	0.03	1.17	0.226	4.2E-01
Mtr.785.1.S1_s_at	8.60	5.57	9.02	8.50	8.63	8.46	7.73	1.88	8.53	0.09	1.10	0.143	5.0E-01
Mtr.2079.1.S1_at	8.25	5.18	8.61	8.13	8.53	8.30	7.34	1.88	8.32	0.20	1.13	0.180	4.2E-01
Mtr.41063.1.S1_at	7.77	4.17	6.91	6.07	7.29	6.07	6.28	1.88	6.48	0.70	1.03	0.044	8.8E-01
Mtr.39677.1.S1_at	7.45	3.77	4.98	3.65	3.83	3.62	5.40	1.88	3.70	0.11	0.69	-0.544	1.9E-01
Mtr.39986.1.S1_at	2.38	5.62	2.37	2.36	2.36	2.35	3.46	1.88	2.36	0.01	0.68	-0.552	3.7E-01
Mtr.19612.1.S1_at	2.79	6.02	2.76	2.67	2.62	2.75	3.86	1.88	2.68	0.07	0.69	-0.525	3.4E-01
Mtr.38443.1.S1_at	8.17	4.61	7.40	7.61	7.11	7.50	6.73	1.88	7.41	0.26	1.10	0.140	5.7E-01
Mtr.6753.1.S1_at	5.57	2.32	5.55	6.27	6.21	5.60	4.48	1.87	6.03	0.37	1.35	0.428	2.3E-01
Mtr.2293.1.S1_at	7.03	4.00	7.42	7.94	7.96	7.77	6.15	1.87	7.89	0.11	1.28	0.359	1.8E-01
Mtr.39127.1.S1_at	7.17	3.76	6.79	6.80	6.64	6.97	5.91	1.87	6.80	0.17	1.15	0.204	4.6E-01
Mtr.50075.1.S1_s_at	7.24	5.30	9.04	9.35	9.36	9.81	7.19	1.87	9.51	0.26	1.32	0.402	1.0E-01
Mtr.20407.1.S1_at	6.67	3.13	5.94	6.75	6.93	6.91	5.25	1.87	6.86	0.10	1.31	0.387	2.1E-01
Mtr.39661.1.S1_at	5.49	2.29	5.56	5.58	5.43	5.47	4.45	1.87	5.49	0.08	1.23	0.303	3.9E-01
Mtr.6240.1.S1_at	2.57	5.80	2.56	2.56	2.55	2.54	3.64	1.87	2.55	0.01	0.70	-0.515	3.7E-01
Mtr.16172.1.S1_at	2.67	5.90	2.65	2.64	2.61	2.59	3.74	1.87	2.61	0.02	0.70	-0.516	3.6E-01
Mtr.25528.1.S1_at	2.46	5.69	2.44	2.44	2.44	2.43	3.53	1.87	2.43	0.01	0.69	-0.537	3.7E-01
Mtr.38776.1.S1_at	8.90	5.17	7.25	7.40	7.75	7.72	7.11	1.87	7.62	0.20	1.07	0.102	6.6E-01
Mtr.44187.1.S1_at	8.69	5.42	8.62	8.15	8.58	8.24	7.58	1.87	8.32	0.22	1.10	0.136	5.3E-01
Mtr.9028.1.S1_at	9.59	6.15	9.13	9.86	10.50	9.92	8.29	1.86	10.09	0.35	1.22	0.284	1.7E-01
Mtr.22225.1.S1_at	2.12	5.35	2.13	2.11	2.11	2.10	3.20	1.86	2.10	0.00	0.66	-0.605	3.7E-01
Mtr.15308.1.S1_s_at	4.89	8.10	4.85	4.98	4.79	4.69	5.95	1.86	4.82	0.15	0.81	-0.303	3.6E-01
Mtr.44047.1.S1_at	2.48	5.68	2.44	2.43	2.42	2.43	3.53	1.86	2.43	0.01	0.69	-0.541	3.6E-01
Mtr.50284.1.S1_at	3.26	6.47	3.24	3.23	3.19	3.46	4.32	1.86	3.30	0.15	0.76	-0.391	3.9E-01
Mtr.31573.1.S1_s_at	6.20	2.66	5.42	6.44	6.84	6.83	4.76	1.86	6.70	0.23	1.41	0.493	1.5E-01
Mtr.6369.1.S1_at	5.69	2.59	5.92	5.43	5.71	4.51	4.73	1.86	5.22	0.63	1.10	0.141	6.9E-01
Mtr.11031.1.S1_at	5.99	2.57	5.53	5.89	5.76	5.47	4.70	1.85	5.71	0.22	1.21	0.281	4.0E-01
Mtr.32924.1.S1_at	7.08	3.54	6.25	7.22	7.56	7.48	5.62	1.85	7.42	0.18	1.32	0.400	1.7E-01
Mtr.885.1.S1_at	6.77	3.37	6.35	6.36	6.74	6.45	5.49	1.85	6.52	0.20	1.19	0.246	4.0E-01
Mtr.6707.1.S1_s_at	5.93	3.10	6.58	6.10	5.75	5.59	5.21	1.85	5.81	0.26	1.12	0.160	6.0E-01
Mtr.1398.1.S1_at	6.86	3.37	6.18	7.13	7.06	6.44	5.47	1.85	6.88	0.38	1.26	0.330	2.7E-01
Mtr.32077.1.S1_at	7.96	4.42	7.11	7.58	7.29	7.65	6.49	1.85	7.51	0.19	1.16	0.209	4.0E-01
Mtr.50532.1.S1_at	6.15	2.84	5.91	5.59	5.59	5.36	4.96	1.85	5.51	0.13	1.11	0.152	6.3E-01
Mtr.2714.1.S1_at	6.51	3.89	7.45	6.97	7.12	6.99	5.95	1.85	7.03	0.08	1.18	0.240	3.7E-01
Mtr.37859.1.S1_at	5.58	2.45	2.32	2.31	2.31	2.31	3.45	1.85	2.31	0.00	0.67	-0.580	3.4E-01
Mtr.3029.1.S1_at	5.90	2.82	6.12	6.07	6.16	5.84	4.95	1.85	6.02	0.17	1.22	0.284	3.7E-01
Mtr.32494.1.S1_at	7.24	3.85	6.81	7.31	7.27	7.20	5.96	1.85	7.26	0.06	1.22	0.284	2.9E-01
Mtr.8269.1.S1_at	6.68	3.02	4.42	4.72	4.87	4.72	4.71	1.85	4.77	0.08	1.01	0.019	9.6E-01
Mtr.20242.1.S1_at	2.71	5.90	2.70	2.68	2.65	2.64	3.77	1.84	2.66	0.02	0.70	-0.504	3.5E-01
Mtr.9676.1.S1_at	8.14	4.58	5.53	6.70	6.27	6.37	6.08	1.84	6.45	0.22	1.06	0.084	7.5E-01

Mtr.6102.1.S1_at	5.23	3.38	7.07	6.82	5.73	6.89	5.23	1.84	6.48	0.65	1.24	0.309	3.3E-01
Mtr.4083.1.S1_at	7.14	4.02	7.28	7.36	7.37	7.56	6.15	1.84	7.43	0.11	1.21	0.273	3.0E-01
Mtr.33678.1.S1_at	9.34	6.15	9.33	9.16	8.76	8.97	8.27	1.84	8.96	0.20	1.08	0.115	5.5E-01
Mtr.6868.1.S1_at	8.64	5.61	8.93	9.00	8.99	8.78	7.73	1.84	8.92	0.12	1.15	0.208	3.2E-01
Mtr.7114.1.S1_at	8.18	4.82	7.79	7.46	7.50	7.69	6.93	1.84	7.55	0.12	1.09	0.123	5.9E-01
Mtr.21348.1.S1_at	4.57	2.65	6.33	6.01	6.10	5.93	4.52	1.84	6.01	0.08	1.33	0.413	2.3E-01
Mtr.44276.1.S1_at	7.70	4.04	6.20	7.19	7.24	7.34	5.98	1.84	7.26	0.08	1.21	0.279	3.0E-01
Mtr.49282.1.S1_at	8.17	4.51	6.09	6.99	6.94	7.13	6.26	1.84	7.02	0.09	1.12	0.166	5.1E-01
Mtr.45494.1.S1_s_at	6.60	3.27	6.29	6.45	6.24	6.63	5.39	1.84	6.44	0.19	1.20	0.257	3.8E-01
Mtr.18726.1.S1_x_at	3.69	6.77	3.50	3.62	3.49	3.86	4.65	1.84	3.66	0.19	0.79	-0.348	4.0E-01
Mtr.46164.1.S1_s_at	6.28	2.61	4.37	4.72	5.54	4.01	4.42	1.84	4.76	0.77	1.08	0.107	7.8E-01
Mtr.44868.1.S1_at	7.15	4.00	7.21	6.93	6.95	6.87	6.12	1.84	6.92	0.04	1.13	0.177	4.9E-01
Mtr.31473.1.S1_at	6.57	3.17	6.07	6.75	6.08	6.76	5.27	1.84	6.53	0.39	1.24	0.309	3.1E-01
Mtr.20846.1.S1_at	9.46	6.29	6.27	5.10	4.83	5.33	7.34	1.83	5.09	0.25	0.69	-0.529	1.0E-01
Mtr.27705.1.S1_at	6.16	3.21	6.58	6.69	6.63	6.36	5.32	1.83	6.56	0.18	1.23	0.303	3.1E-01
Mtr.4998.1.S1_s_at	5.75	3.10	6.62	6.45	5.89	6.25	5.15	1.83	6.20	0.28	1.20	0.266	3.8E-01
Mtr.50277.1.S1_at	10.54	7.03	9.67	10.34	10.60	10.71	9.08	1.83	10.55	0.19	1.16	0.217	2.4E-01
Mtr.6901.1.S1_at	7.12	4.05	7.31	7.05	7.20	6.96	6.16	1.83	7.07	0.12	1.15	0.200	4.4E-01
Mtr.30016.1.S1_at	6.61	3.61	6.93	6.89	6.91	6.61	5.72	1.83	6.80	0.16	1.19	0.251	3.6E-01
Mtr.14015.1.S1_at	5.72	2.37	5.32	4.79	4.61	5.06	4.47	1.83	4.82	0.23	1.08	0.109	7.6E-01
Mtr.2665.1.S1_at	6.72	3.44	6.49	6.32	6.43	6.57	5.55	1.83	6.44	0.12	1.16	0.215	4.5E-01
Mtr.9813.1.S1_at	5.20	1.78	4.63	4.85	4.85	4.85	3.87	1.83	4.85	0.00	1.25	0.326	4.1E-01
Mtr.32009.1.S1_at	7.36	3.81	6.37	6.35	6.39	6.30	5.85	1.83	6.34	0.04	1.09	0.118	6.6E-01
Mtr.9630.1.S1_at	8.37	4.86	7.50	7.60	7.73	7.88	6.91	1.83	7.73	0.14	1.12	0.162	4.8E-01
Mtr.41964.1.S1_at	3.33	6.60	3.55	3.23	3.10	3.30	4.49	1.83	3.21	0.10	0.72	-0.483	2.9E-01
Mtr.19906.1.S1_at	2.76	5.82	2.56	2.55	2.55	2.46	3.71	1.83	2.52	0.06	0.68	-0.558	3.2E-01
Mtr.42337.1.S1_at	9.06	5.79	8.83	8.75	8.33	8.48	7.89	1.83	8.52	0.21	1.08	0.111	5.8E-01
Mtr.27728.1.S1_s_at	6.88	3.34	4.33	4.67	4.73	4.48	4.85	1.83	4.63	0.13	0.95	-0.069	8.4E-01
Mtr.43813.1.S1_at	7.45	3.81	5.38	6.78	6.32	6.84	5.55	1.82	6.65	0.28	1.20	0.261	3.6E-01
Mtr.5650.1.S1_at	6.01	2.69	5.67	6.22	5.73	6.07	4.79	1.82	6.01	0.25	1.25	0.326	3.2E-01
Mtr.47896.1.S1_s_at	7.75	4.61	7.78	7.72	7.74	7.56	6.71	1.82	7.67	0.10	1.14	0.193	4.1E-01
Mtr.5364.1.S1_at	6.46	3.47	6.76	6.36	6.68	6.24	5.56	1.82	6.43	0.23	1.15	0.208	4.6E-01
Mtr.28302.1.S1_at	8.06	4.98	8.22	8.41	8.62	8.23	7.09	1.82	8.42	0.19	1.19	0.249	2.8E-01
Mtr.18605.1.S1_at	2.22	2.26	5.40	6.31	3.34	5.70	3.29	1.82	5.12	1.57	1.55	0.635	2.6E-01
Mtr.9099.1.S1_s_at	6.37	2.76	4.99	4.29	5.74	4.70	4.71	1.82	4.91	0.75	1.04	0.060	8.7E-01
Mtr.42047.1.S1_s_at	8.21	4.84	7.71	7.80	7.64	7.97	6.92	1.82	7.80	0.17	1.13	0.174	4.5E-01
Mtr.39208.1.S1_at	5.85	2.80	6.05	5.56	6.05	5.74	4.90	1.82	5.78	0.25	1.18	0.238	4.5E-01
Mtr.32316.1.S1_at	7.25	3.63	5.75	6.62	6.43	6.66	5.54	1.82	6.57	0.12	1.19	0.245	3.8E-01
Mtr.28255.1.S1_at	6.21	3.27	6.59	7.57	6.53	7.31	5.36	1.82	7.13	0.54	1.33	0.413	1.8E-01
Mtr.49874.1.S1_at	6.39	3.50	6.86	7.20	7.02	7.01	5.59	1.82	7.08	0.11	1.27	0.342	2.3E-01
Mtr.2646.1.S1_at	7.26	4.13	7.30	6.78	7.03	6.52	6.23	1.82	6.78	0.26	1.09	0.121	6.3E-01
Mtr.41614.1.S1_at	7.85	5.08	4.43	7.80	8.17	8.27	5.79	1.82	8.08	0.25	1.40	0.482	9.6E-02
Mtr.11192.1.S1_at	5.90	2.88	6.14	7.44	7.66	7.54	4.97	1.82	7.55	0.11	1.52	0.603	7.0E-02
Mtr.31475.1.S1_at	3.22	6.36	3.21	3.21	3.54	3.13	4.27	1.82	3.29	0.22	0.77	-0.373	4.1E-01
Mtr.8008.1.S1_at	2.38	5.51	2.35	2.34	2.34	2.33	3.41	1.82	2.34	0.00	0.69	-0.545	3.6E-01
Mtr.38798.1.S1_at	6.52	3.25	6.25	6.35	6.61	6.29	5.34	1.82	6.42	0.17	1.20	0.265	3.6E-01
Mtr.23311.1.S1_at	6.30	3.88	7.44	7.21	7.31	6.96	5.88	1.82	7.16	0.18	1.22	0.286	2.9E-01
Mtr.1869.1.S1_at	8.52	5.21	8.17	8.35	8.34	8.20	7.30	1.82	8.29	0.08	1.14	0.185	4.0E-01
Mtr.3273.1.S1_at	7.10	4.17	7.50	7.92	8.05	7.98	6.26	1.82	7.98	0.07	1.28	0.351	1.8E-01
Mtr.27993.1.S1_at	3.41	2.24	5.80	4.64	5.80	3.75	3.82	1.82	4.73	1.03	1.24	0.308	4.9E-01
Mtr.24407.1.S1_at	7.02	4.06	7.35	7.29	6.87	7.23	6.14	1.81	7.13	0.22	1.16	0.216	4.0E-01
Mtr.32833.1.S1_at	5.90	2.53	3.05	3.09	3.07	3.07	3.83	1.81	3.08	0.01	0.80	-0.315	5.1E-01
Mtr.39287.1.S1_at	7.96	4.49	7.15	6.95	6.87	6.96	6.53	1.81	6.93	0.05	1.06	0.085	7.3E-01
Mtr.26.1.S1_s_at	2.78	5.91	2.76	2.74	2.74	2.81	3.82	1.81	2.77	0.04	0.72	-0.465	3.7E-01
Mtr.25588.1.S1_at	6.43	3.27	6.39	6.45	6.60	6.36	5.36	1.81	6.47	0.12	1.21	0.271	3.5E-01
Mtr.24881.1.S1_at	5.29	2.49	5.89	5.30	5.32	4.64	4.56	1.81	5.09	0.39	1.12	0.160	6.4E-01
Mtr.38292.1.S1_at	4.32	3.17	6.72	2.99	3.92	3.36	4.74	1.81	3.42	0.47	0.72	-0.469	2.9E-01
Mtr.3362.1.S1_x_at	2.42	5.53	2.36	2.34	2.34	2.33	3.44	1.81	2.33	0.00	0.68	-0.560	3.5E-01
Mtr.38453.1.S1_at	10.10	6.82	9.79	9.99	9.74	9.79	8.90	1.81	9.84	0.13	1.11	0.144	4.2E-01
Mtr.18045.1.S1_at	3.05	6.18	3.04	3.03	2.82	3.03	4.09	1.81	2.96	0.12	0.72	-0.465	3.4E-01
Mtr.39976.1.S1_at	5.16	2.60	6.10	5.42	5.83	5.42	4.62	1.81	5.55	0.24	1.20	0.266	4.3E-01
Mtr.12397.1.S1_at	11.35	7.83	10.36	10.19	10.30	10.60	9.85	1.81	10.36	0.21	1.05	0.074	6.5E-01
Mtr.20682.1.S1_x_at	8.81	11.91	8.74	8.78	8.87	8.74	9.82	1.81	8.80	0.07	0.90	-0.158	3.8E-01
Mtr.13100.1.S1_at	6.39	2.78	4.86	6.85	7.56	7.28	4.68	1.81	7.23	0.36	1.55	0.629	7.5E-02
Mtr.5617.1.S1_at	5.32	2.24	5.42	4.26	5.34	5.32	4.33	1.81	4.97	0.62	1.15	0.202	5.9E-01
Mtr.18420.1.S1_at	3.12	3.67	6.49	6.92	6.82	6.89	4.43	1.81	6.88	0.05	1.55	0.635	7.9E-02
Mtr.11778.1.S1_at	6.05	2.93	6.05	6.23	6.63	6.46	5.01	1.81	6.44	0.20	1.28	0.362	2.4E-01
Mtr.17993.1.S1_at	2.74	5.85	2.70	2.70	2.64	2.69	3.76	1.81	2.68	0.03	0.71	-0.492	3.6E-01
Mtr.11949.1.S1_at	9.64	6.44	9.48	9.80	9.70	9.74	8.52	1.81	9.75	0.05	1.14	0.194	3.0E-01
Mtr.46334.1.S1_at	7.45	4.58	7.91	7.64	7.58	7.03	6.65	1.81	7.42	0.33	1.12	0.158	5.1E-01
Mtr.17818.1.S1_at	2.99	6.10	2.96	2.93	2.90	2.90	4.01	1.80	2.91	0.02	0.73	-0.463	3.5E-01
Mtr.13616.1.S1_s_at	5.71	2.96	6.36	6.13	6.26	5.98	5.01	1.80	6.12	0.14	1.22	0.289	3.5E-01
Mtr.41839.1.S1_at	6.16	3.93	7.50	6.56	5.78	5.89	5.87	1.80	6.08	0.42	1.04	0.051	8.5E-01
Mtr.9680.1.S1_at	6.05	2.63	5.32	5.45	5.53	5.35	4.66	1.80	5.44	0.09	1.17	0.223	5.0E-01
Mtr.14593.1.S1_at	8.87	5.67	8.70	8.96	9.15	9.07	7.75	1.80	9.06	0.10	1.17	0.226	2.8E-01
Mtr.17181.1.S1_at	6.80	4.25	7.72	5.36	6.02	5.06	6.26	1.80	5.48	0.49	0.88	-0.192	5.1E-01
Mtr.43593.1.S1_at	8.50	5.59	8.89	8.76	8.58	8.24	7.66	1.80	8.52	0.26	1.11	0.153	4.6E-01
Mtr.26735.1.S1_at	2.40	5.51	2.38	2.37	2.39	2.42	3.43	1.80	2.39	0.03	0.70	-0.521	3.7E-01
Mtr.5047.1.S1_at	7.56	4.43	7.54	7.36	7.11	6.99	6.51	1.80	7.15	0.19	1.10	0.136	5.7E-01
Mtr.6756.1.S1_at	5.16	1.80	4.61	4.90	4.74	4.74	3.86	1.80	4.79	0.10	1.24	0.314	4.2E-01
Mtr.39757.1.S1_at	5.81	2.90	6.18	5.70	6.11	5.16	4.96	1.80	5.66	0.47	1.14	0.189	5.5E-01
Mtr.20384.1.S1_s_at	2.39	5.50	2.37	2.36	2.37	2.35	3.42	1.80	2.36	0.01	0.69	-0.537	3.6E-01
Mtr.19480.1.S1_at	7.52	4.67	8.00	7.87	7.64	7.53	6.73	1.80	7.68	0.17	1.14	0.190	4.2E-01
Mtr.5719.1.S1_at	7.58	4.61	7.86	7.58	7.69	7.55	6.69	1.80	7.61	0.08	1.14	0.186	4.3E-01
Mtr.19567.1.S1_at	2.61	5.72	2.60	2.58	2.55	2.58	3.64	1.80	2.57	0.02	0.71	-0.501	3.6E-01
Mtr.1721.1.S1_at	6.25	3.10	6.17	5.78	5.62	5.61	5.17	1.80	5.67	0.10	1.10	0.133	6.6E-01
Mtr.27067.1.S1_at	8.17	4.90	7.82	7.88	7.52	8.07	6.96	1.80	7.82	0.28	1.12	0.168	4.6E-01
Mtr.18642.1.S1_at	3.64	3.11	6.45	5.66	6.83	5.67	4.40	1.80	6.05	0.67	1.38	0.460	2.1E-01
Mtr.44955.1.S1_s_at	6.25	3.03	6.03	6.45	6.99	6.42	5.10	1.80	6.62	0.32	1.30	0.376	2.2E-01
Mtr.14419.1.S1_s_at	6.82	4.14	7.56	7.58	7.45	7.19	6.17	1.80	7.40	0.20	1.20	0.262	3.0E-01
Mtr.37841.1.S1_at	7.92	5.89	9.47	8.64	8.66	8.73	7.76	1.80	8.68	0.05	1.12	0.161	4.3E-01
Mtr.48263.1.S1_s_at	5.79	2.82	6.05	5.75	6.05	5.81	4.89	1.79	5.87	0.16	1.20	0.265	4.0E-01
Mtr.15345.1.S1_at	8.36	5.16	8.17	8.39	7.82	8.46	7.23	1.79	8.23	0.35	1.14	0.186	4.0E-01
Mtr.26826.1.S1_at	7.01	3.54	4.50	3.46	3.37	3.31	5.02	1.79	3.38	0.07	0.67	-0.570	1.9E-01
Mtr.37944.1.S1_at	10.37	6.92	9.50	9.44	9.08	9.77	8.93	1.79	9.43	0.35	1.06	0.078	6.6E-01
Mtr.39060.1.S1_s_at	6.48	3.06	5.69	5.63	5.17	5.24	5.08	1.79	5.35	0.25	1.05	0.075	8.1E-01
Mtr.4486.1.S1_at	1.99	5.08	1.98	1.98	1.98	1.98	3.02	1.79	1.98	0.00	0.66	-0.610	3.7E-01
Mtr.17497.1.S1_at	8.15	5.19	8.42	8.50	9.16	8.58	7.25	1.79	8.75	0.36	1.21	0.270	2.3E-01
Mtr.44864.1.S1_at	6.93	3.69	6.64	6.61	6.67	6.87	5.75	1.79	6.72	0.14	1.17	0.224	4.0E-01
Mtr.43756.1.S1_at	8.22	5.00	7.97	7.81	7.72	8.00	7.06	1.79	7.84	0.14	1.11	0.152	4.9E-01
Mtr.44141.1.S1_at	8.11	4.53	6.33	6.29	6.62	6.63	6.32	1.79	6.51	0.19	1.03	0.043	8.6E-01
Mtr.16117.1.S1_s_at	7.77	4.26	5.43	7.00	6.91	7.20	5.82	1.79	7.04	0.15	1.21	0.273	3.1E-01
Mtr.48064.1.S1_at	5.57	3.36	6.90	5.77	6.15	5.25	5.27	1.79	5.72	0.45	1.09	0.118	6.9E-01
Mtr.31791.1.S1_at	7.39	4.15	7.07	7.27	7.13	7.28	6.20	1.79	7.23	0.08	1.17	0.221	3.8E-01
Mtr.44590.1.S1_s_at	5.73	2.89	6.18	5.77	5.86	5.56	4.93	1.78	5.73	0.15	1.16	0.217	4.8E-01
Mtr.13830.1.S1_s_at	10.22	6.68	8.81	8.80	8.86	9.04	8.57	1.78	8.90	0.13	1.04	0.054	7.7E-01
Mtr.40667.1.S1_at	10.19	6.71	7.79	8.90	8.59	8.98	8.23	1.78	8.82	0.20	1.07	0.100	6.0E-01
Mtr.1854.1.S1_at	6.45	3.01	5.56	6.50	7.03	7.13	5.01	1.78	6.89	0.34	1.38	0.460	1.5E-01
Mtr.24892.1.S1_at	8.20	5.41	8.73	8.75	8.72	8.66	7.45	1.78	8.71	0.05	1.17	0.226	2.9E-01
Mtr.35116.1.S1_at	6.22	2.91	5.73	5.84	6.01	5.72	4.95	1.78	5.86	0.14	1.18	0.242	4.3E-01
Mtr.30476.1.S1_at	6.76	3.66	6.72	5.93	5.94	5.01	5.71	1.78	5.63	0.53	0.98	-0.022	9.4E-01
Mtr.25578.1.S1_at	2.52	5.60	2.51	2.56	2.49	2.51	3.55	1.78	2.52	0.03	0.71	-0.493	3.7E-01
Mtr.13071.1.S1_at	7.39	3.87	5.20	5.20	5.68	5.65	5.49	1.78	5.51	0.27	1.00	0.006	9.8E-01
Mtr.23313.1.S1_at	4.67	2.37	5.87	5.46	5.45	5.81	4.30	1.78	5.57	0.21	1.30	0.373	2.9E-01
Mtr.12327.1.S1_s_at	10.23	7.77	6.78	8.17	7.70	8.25	8.26	1.78	8.04	0.30	0.97	-0.039	8.4E-01
Mtr.42785.1.S1_at	6.91	3.43	5.82	5.84	5.51	6.23	5.39	1.78	5.86	0.36	1.09	0.121	6.7E-01
Mtr.41777.1.S1_at	9.19	12.70	10.44	11.75	11.56	11.14	10.77	1.78	11.48	0.32	1.07	0.092	5.3E-01
Mtr.24002.1.S1_at	2.92	5.97	2.88	2.84	2.79	2.84	3.93	1.77	2.82	0.03	0.72	-0.475	3.4E-01
Mtr.37096.1.S1_a_at	6.16	3.13	6.25	6.18	7.33	6.36	5.18	1.77	6.62	0.62	1.28	0.355	2.5E-01
Mtr.26709.1.S1_at	2.18	5.28	2.24	2.17	2.17	2.15	3.24	1.77	2.16	0.01	0.67	-0.583	3.5E-01
Mtr.45439.1.S1_at	7.29	4.56	7.88	8.07	7.90	7.64	6.57	1.77	7.87	0.22	1.20	0.259	2.8E-01
Mtr.6304.1.S1_at	5.54	2.44	5.47	5.41	5.73	5.22	4.49	1.77	5.45	0.26	1.22	0.282	4.0E-01
Mtr.23198.1.S1_at	2.90	5.99	2.95	2.94	2.89	2.92	3.95	1.77	2.92	0.02	0.74	-0.435	3.7E-01
Mtr.39796.1.S1_at	6.86	4.05	7.33	6.94	6.88	6.52	6.08	1.77	6.78	0.23	1.11	0.157	5.4E-01
Mtr.52292.1.S1_at	7.34	4.35	4.21	5.18	4.67	5.13	5.30	1.77	4.99	0.28	0.94	-0.087	7.8E-01
Mtr.5285.1.S1_at	6.29	3.12	6.06	5.61	5.86	5.68	5.16	1.77	5.71	0.13	1.11	0.148	6.2E-01
Mtr.22779.1.S1_s_at	7.88	4.63	7.47	7.59	7.80	7.38	6.66	1.77	7.59	0.21	1.14	0.190	4.1E-01
Mtr.24496.1.S1_at	2.17	5.23	2.16	2.16	2.16	2.15	3.19	1.77	2.15	0.00	0.68	-0.566	3.7E-01
Mtr.52009.1.S1_s_at	4.37	1.50	4.74	4.40	4.81	3.97	3.54	1.77	4.39	0.42	1.24	0.313	4.6E-01
Mtr.34754.1.S1_at	2.80	3.41	6.12	5.05	5.00	4.04	4.11	1.77	4.70	0.57	1.14	0.193	6.1E-01
Mtr.20405.1.S1_x_at	2.48	5.52	2.44	2.43	2.42	2.43	3.48	1.77	2.43	0.01	0.70	-0.517	3.6E-01

Mtr.1017.1.S1_at	2.58	5.63	2.56	2.55	2.54	2.53	3.59	1.77	2.54	0.01	0.71	-0.498	3.6E-01
Mtr.51616.1.S1_at	8.84	5.74	8.77	8.89	8.89	8.80	7.78	1.77	8.86	0.05	1.14	0.187	3.5E-01
Mtr.1356.1.S1_s_at	5.97	3.58	7.02	7.51	5.94	7.34	5.52	1.77	6.93	0.86	1.25	0.328	2.8E-01
Mtr.21225.1.S1_at	3.06	6.09	3.01	3.01	3.02	3.02	4.05	1.77	3.01	0.01	0.74	-0.428	3.7E-01
Mtr.26990.1.S1_at	8.02	4.97	8.04	8.06	8.07	7.73	7.01	1.77	7.96	0.20	1.13	0.182	4.1E-01
Mtr.28757.1.S1_at	6.46	3.27	6.16	6.67	6.62	7.07	5.30	1.76	6.78	0.25	1.28	0.357	2.2E-01
Mtr.4456.1.S1_at	2.66	5.73	2.69	2.64	2.65	2.63	3.69	1.76	2.64	0.01	0.72	-0.484	3.6E-01
Mtr.23637.1.S1_x_at	9.62	6.11	7.60	6.72	8.49	7.74	7.78	1.76	7.65	0.89	0.98	-0.024	9.1E-01
Mtr.33875.1.S1_at	5.85	2.93	6.10	5.70	5.71	5.47	4.96	1.76	5.62	0.14	1.13	0.182	5.5E-01
Mtr.13649.1.S1_at	5.79	2.75	5.81	5.97	6.62	5.50	4.78	1.76	6.03	0.56	1.26	0.334	3.1E-01
Mtr.46259.1.S1_at	10.83	7.84	10.96	11.18	11.02	11.04	9.88	1.76	11.08	0.09	1.12	0.166	3.0E-01
Mtr.31644.1.S1_at	6.26	3.54	6.83	6.25	6.25	6.18	5.55	1.76	6.23	0.04	1.12	0.167	5.4E-01
Mtr.5722.1.S1_at	8.08	4.58	6.04	8.17	7.30	7.84	6.23	1.76	7.77	0.44	1.25	0.319	2.2E-01
Mtr.8440.1.S1_at	10.12	8.00	6.63	8.86	8.69	9.35	8.25	1.76	8.96	0.34	1.09	0.120	5.3E-01
Mtr.22379.1.S1_s_at	2.25	5.29	2.24	2.23	2.23	2.23	3.26	1.76	2.23	0.00	0.68	-0.547	3.7E-01
Mtr.18163.1.S1_x_at	4.62	7.66	4.61	4.67	4.58	4.57	5.63	1.76	4.61	0.05	0.82	-0.289	3.7E-01
Mtr.41622.1.S1_at	9.39	7.27	5.90	6.79	6.43	6.75	7.52	1.76	6.66	0.20	0.88	-0.176	4.4E-01
Mtr.33798.1.S1_at	5.74	3.17	6.54	5.77	5.83	5.91	5.15	1.76	5.84	0.07	1.13	0.181	5.4E-01
Mtr.26879.1.S1_s_at	5.80	2.45	5.05	5.03	4.62	4.47	4.43	1.76	4.71	0.29	1.06	0.087	8.0E-01
Mtr.21299.1.S1_s_at	2.33	5.37	2.32	2.32	2.31	2.31	3.34	1.76	2.31	0.01	0.69	-0.531	3.7E-01
Mtr.34245.1.S1_at	2.69	5.72	2.66	2.65	2.65	2.64	3.69	1.76	2.64	0.01	0.72	-0.481	3.6E-01
Mtr.18312.1.S1_at	7.63	4.58	4.59	4.78	4.40	4.40	5.60	1.76	4.53	0.22	0.81	-0.307	3.5E-01
Mtr.33682.1.S1_at	6.61	3.99	7.33	6.27	6.48	6.37	5.98	1.76	6.38	0.11	1.07	0.093	7.1E-01
Mtr.22453.1.S1_at	6.46	9.21	5.95	6.68	5.81	6.31	7.21	1.76	6.27	0.44	0.87	-0.202	4.2E-01
Mtr.35578.1.S1_at	8.49	5.59	8.75	8.86	8.78	8.45	7.61	1.75	8.70	0.22	1.14	0.192	3.5E-01
Mtr.42801.1.S1_at	9.18	6.19	9.27	9.61	9.74	9.54	8.21	1.75	9.63	0.10	1.17	0.230	2.3E-01
Mtr.30690.1.S1_at	5.65	2.28	4.77	4.55	5.32	4.61	4.23	1.75	4.83	0.43	1.14	0.189	6.0E-01
Mtr.20847.1.S1_at	9.12	6.45	5.82	4.24	4.88	5.52	7.13	1.75	4.88	0.64	0.68	-0.547	1.0E-01
Mtr.41078.1.S1_at	6.54	3.28	6.01	6.71	7.55	6.83	5.28	1.75	7.03	0.46	1.33	0.413	1.7E-01
Mtr.15871.1.S1_at	6.03	3.37	6.67	5.16	5.21	4.32	5.36	1.75	4.90	0.50	0.91	-0.130	6.8E-01
Mtr.19208.1.S1_at	9.24	5.78	7.04	7.55	6.67	7.36	7.36	1.75	7.20	0.46	0.98	-0.032	8.8E-01
Mtr.40236.1.S1_s_at	9.48	6.21	8.92	8.22	6.29	7.85	8.20	1.75	7.45	1.03	0.91	-0.138	5.6E-01
Mtr.46938.1.S1_at	3.02	6.01	2.94	2.93	2.92	2.85	3.99	1.75	2.90	0.04	0.73	-0.460	3.4E-01
Mtr.35513.1.S1_at	5.59	2.48	2.66	2.61	2.61	2.59	3.58	1.75	2.60	0.01	0.73	-0.459	3.9E-01
Mtr.30668.1.S1_at	7.27	4.02	6.76	7.66	7.91	7.83	6.02	1.75	7.80	0.13	1.30	0.374	1.5E-01
Mtr.38107.1.S1_at	7.62	4.78	4.43	4.07	5.34	4.42	5.61	1.75	4.61	0.65	0.82	-0.283	4.1E-01
Mtr.6415.1.S1_at	2.66	5.67	2.64	2.62	2.60	2.62	3.66	1.75	2.61	0.01	0.71	-0.484	3.6E-01
Mtr.38682.1.S1_s_at	6.82	3.80	6.83	6.32	6.86	6.56	5.82	1.75	6.58	0.27	1.13	0.178	4.9E-01
Mtr.18881.1.S1_s_at	2.56	5.55	2.50	2.49	2.47	2.51	3.54	1.75	2.49	0.02	0.70	-0.507	3.6E-01
Mtr.44343.1.S1_at	7.61	4.38	7.14	7.05	6.91	6.77	6.38	1.75	6.91	0.14	1.08	0.116	6.3E-01
Mtr.6697.1.S1_at	6.10	2.65	4.80	5.46	5.10	5.87	4.52	1.74	5.48	0.38	1.21	0.278	4.0E-01
Mtr.25377.1.S1_at	2.48	5.50	2.48	2.47	2.47	2.46	3.49	1.74	2.47	0.01	0.71	-0.500	3.7E-01
Mtr.34850.1.S1_s_at	9.04	5.80	8.54	8.19	7.69	8.18	7.79	1.74	8.02	0.29	1.03	0.042	8.3E-01
Mtr.38017.1.S1_at	5.49	2.72	5.95	5.28	6.20	5.28	4.72	1.74	5.58	0.53	1.18	0.243	4.6E-01
Mtr.22894.1.S1_at	2.06	5.07	2.05	2.05	2.04	2.04	3.06	1.74	2.04	0.00	0.67	-0.581	3.7E-01
Mtr.6672.1.S1_at	6.34	3.48	6.63	6.14	6.14	5.80	5.48	1.74	6.03	0.20	1.10	0.137	6.2E-01
Mtr.44215.1.S1_at	7.85	4.50	7.02	7.69	7.30	7.54	6.46	1.74	7.51	0.20	1.16	0.217	3.6E-01
Mtr.49755.1.S1_at	2.35	5.34	2.32	2.31	2.30	2.30	3.34	1.74	2.31	0.01	0.69	-0.533	3.6E-01
Mtr.14647.1.S1_s_at	4.43	7.43	4.41	4.41	4.04	4.41	5.42	1.74	4.29	0.22	0.79	-0.339	3.2E-01
Mtr.40052.1.S1_at	8.16	5.28	8.41	8.33	8.40	8.30	7.28	1.74	8.34	0.05	1.15	0.196	3.5E-01
Mtr.50224.1.S1_s_at	10.13	7.52	10.82	10.67	10.44	10.19	9.49	1.74	10.43	0.24	1.10	0.137	4.0E-01
Mtr.29580.1.S1_at	7.17	4.06	6.95	7.18	7.04	7.23	6.06	1.74	7.15	0.10	1.18	0.238	3.4E-01
Mtr.37178.1.S1_at	6.97	4.06	7.15	6.75	7.18	6.76	6.06	1.73	6.90	0.24	1.14	0.187	4.5E-01
Mtr.24223.1.S1_at	5.58	3.06	6.39	6.36	6.42	6.03	5.01	1.73	6.27	0.21	1.25	0.325	2.8E-01
Mtr.24489.1.S1_x_at	2.35	5.34	2.33	2.33	2.39	2.32	3.34	1.73	2.35	0.04	0.70	-0.511	3.8E-01
Mtr.42773.1.S1_at	5.69	2.54	5.37	5.35	5.35	5.04	4.53	1.73	5.25	0.18	1.16	0.211	5.2E-01
Mtr.51697.1.S1_at	5.91	3.23	6.47	5.68	6.30	5.46	5.20	1.73	5.81	0.44	1.12	0.160	5.9E-01
Mtr.28794.1.S1_at	6.73	3.72	6.71	6.81	7.14	6.93	5.72	1.73	6.96	0.17	1.22	0.283	2.8E-01
Mtr.28466.1.S1_at	5.46	8.27	5.12	5.20	5.12	5.01	6.29	1.73	5.11	0.10	0.81	-0.299	3.0E-01
Mtr.32075.1.S1_at	10.25	13.23	10.23	10.33	10.01	10.23	11.23	1.73	10.19	0.17	0.91	-0.141	3.6E-01
Mtr.13888.1.S1_at	5.67	3.37	6.75	5.88	6.32	6.21	5.26	1.73	6.14	0.23	1.17	0.221	4.3E-01
Mtr.9790.1.S1_at	6.70	3.84	6.95	6.76	6.74	6.65	5.83	1.73	6.72	0.06	1.15	0.204	4.3E-01
Mtr.20207.1.S1_at	8.25	5.17	8.06	8.40	8.11	8.31	7.16	1.73	8.27	0.15	1.16	0.208	3.3E-01
Mtr.2641.1.S1_at	7.09	4.51	7.78	7.23	7.49	7.12	6.46	1.73	7.28	0.19	1.13	0.172	4.6E-01
Mtr.41305.1.S1_at	7.34	3.89	5.63	6.70	7.02	6.41	5.62	1.73	6.71	0.30	1.19	0.255	3.4E-01
Mtr.15142.1.S1_at	2.45	5.43	2.43	2.43	2.41	2.40	3.44	1.72	2.41	0.02	0.70	-0.511	3.6E-01
Mtr.50440.1.S1_s_at	5.53	2.55	5.54	5.38	5.70	5.80	4.54	1.72	5.63	0.22	1.24	0.309	3.4E-01
Mtr.12032.1.S1_at	6.73	3.78	6.80	7.21	6.50	7.04	5.77	1.72	6.92	0.37	1.20	0.261	3.2E-01
Mtr.28830.1.S1_at	7.26	4.13	6.95	7.19	7.60	7.61	6.11	1.72	7.47	0.24	1.22	0.289	2.5E-01
Mtr.1561.1.S1_at	8.05	4.96	7.82	7.97	8.46	8.05	6.94	1.72	8.16	0.26	1.18	0.233	2.9E-01
Mtr.3007.1.S1_s_at	8.17	5.06	7.90	8.03	8.13	8.03	7.05	1.72	8.06	0.06	1.14	0.195	3.6E-01
Mtr.37504.1.S1_at	6.48	3.70	6.85	6.67	6.80	6.12	5.68	1.72	6.53	0.36	1.15	0.202	4.5E-01
Mtr.44862.1.S1_at	7.76	4.70	7.61	7.66	7.56	7.25	6.69	1.72	7.49	0.21	1.12	0.163	4.7E-01
Mtr.49616.1.S1_at	7.05	4.51	7.80	7.56	7.42	7.40	6.45	1.72	7.46	0.09	1.16	0.208	3.7E-01
Mtr.8406.1.S1_at	6.38	3.63	6.79	6.66	6.66	6.28	5.60	1.72	6.53	0.22	1.17	0.222	4.1E-01
Mtr.43468.1.S1_at	5.47	2.28	4.98	4.57	4.53	5.27	4.24	1.72	4.79	0.41	1.13	0.175	6.2E-01
Mtr.40711.1.S1_at	9.08	6.09	9.06	9.96	11.03	10.80	8.08	1.72	10.60	0.56	1.31	0.392	7.3E-02
Mtr.38541.1.S1_at	6.84	3.53	5.97	6.66	5.99	6.26	5.45	1.72	6.30	0.34	1.16	0.211	4.4E-01
Mtr.1922.1.S1_at	8.22	5.14	7.99	8.00	8.28	8.11	7.12	1.72	8.13	0.14	1.14	0.192	3.7E-01
Mtr.12393.1.S1_at	12.38	10.29	8.97	6.73	4.15	6.43	10.55	1.72	5.77	1.41	0.55	-0.870	2.0E-02
Mtr.38455.1.S1_at	8.80	6.09	9.28	9.12	9.01	8.98	8.06	1.72	9.04	0.08	1.12	0.165	3.8E-01
Mtr.5824.1.S1_at	8.32	5.22	8.06	7.98	8.50	8.10	7.20	1.72	8.20	0.27	1.14	0.187	3.8E-01
Mtr.43296.1.S1_at	10.93	9.58	7.52	9.75	9.89	10.30	9.35	1.72	9.98	0.28	1.07	0.095	5.6E-01
Mtr.5051.1.S1_at	2.61	5.65	2.75	2.57	2.89	3.09	3.67	1.72	2.85	0.26	0.78	-0.366	4.6E-01
Mtr.5382.1.S1_s_at	4.88	2.17	5.34	5.67	5.97	5.50	4.13	1.71	5.71	0.24	1.38	0.469	1.9E-01
Mtr.50591.1.S1_at	2.84	5.80	2.83	2.83	2.83	2.83	3.82	1.71	2.83	0.00	0.74	-0.433	3.7E-01
Mtr.4439.1.S1_s_at	7.02	3.97	6.83	6.41	7.26	6.84	5.94	1.71	6.83	0.42	1.15	0.203	4.3E-01
Mtr.21196.1.S1_at	2.39	5.35	2.38	2.38	2.34	2.37	3.37	1.71	2.36	0.02	0.70	-0.514	3.6E-01
Mtr.33683.1.S1_at	7.26	3.91	6.22	6.19	6.29	6.29	5.80	1.71	6.26	0.06	1.08	0.110	6.7E-01
Mtr.19487.1.S1_at	8.38	5.53	8.60	8.53	8.69	8.51	7.50	1.71	8.58	0.10	1.14	0.193	3.4E-01
Mtr.40995.1.S1_at	8.71	6.07	9.28	8.62	8.69	8.54	8.02	1.71	8.61	0.08	1.07	0.103	5.8E-01
Mtr.27572.1.S1_at	7.41	4.06	6.37	6.16	5.83	6.13	5.95	1.71	6.04	0.18	1.02	0.022	9.3E-01
Mtr.18067.1.S1_at	10.05	6.72	7.70	8.62	7.77	8.59	8.16	1.71	8.33	0.48	1.02	0.030	8.8E-01
Mtr.9454.1.S1_at	6.98	4.25	7.40	7.74	8.20	7.87	6.21	1.71	7.94	0.23	1.28	0.354	1.6E-01
Mtr.44343.1.S1_s_at	7.58	4.47	7.26	7.55	6.96	7.16	6.43	1.71	7.22	0.30	1.12	0.167	4.7E-01
Mtr.34470.1.S1_s_at	8.38	5.30	8.14	8.18	7.88	8.24	7.27	1.71	8.10	0.19	1.11	0.156	4.5E-01
Mtr.33655.1.S1_at	6.00	3.23	6.36	5.43	5.27	5.71	5.20	1.71	5.47	0.22	1.05	0.073	8.0E-01
Mtr.10023.1.S1_at	5.89	3.60	6.94	7.08	7.32	6.87	5.47	1.71	7.09	0.23	1.30	0.373	1.8E-01
Mtr.9887.1.S1_at	6.96	4.13	7.20	7.06	7.12	6.88	6.10	1.71	7.02	0.12	1.15	0.204	4.0E-01
Mtr.40082.1.S1_at	7.74	4.91	4.66	4.47	5.02	4.38	5.77	1.71	4.63	0.35	0.80	-0.319	3.2E-01
Mtr.44357.1.S1_at	9.69	6.98	10.14	9.43	9.25	9.49	8.94	1.71	9.39	0.13	1.05	0.072	6.7E-01
Mtr.38846.1.S1_at	7.19	4.34	7.39	7.57	7.81	7.43	6.31	1.71	7.60	0.19	1.21	0.269	2.6E-01
Mtr.39733.1.S1_at	5.32	2.18	4.92	5.18	4.19	4.93	4.14	1.71	4.76	0.52	1.15	0.202	5.8E-01
Mtr.5289.1.S1_at	5.92	3.06	6.11	6.10	6.15	6.33	5.03	1.71	6.19	0.12	1.23	0.300	3.0E-01
Mtr.46362.1.S1_at	7.75	4.60	5.05	5.13	5.72	6.13	5.80	1.71	5.66	0.51	0.98	-0.035	9.0E-01
Mtr.33044.1.S1_s_at	5.59	2.54	2.75	3.27	2.65	3.76	3.63	1.71	3.22	0.56	0.89	-0.170	7.2E-01
Mtr.32356.1.S1_at	8.03	5.08	8.04	7.95	7.88	7.75	7.05	1.71	7.86	0.10	1.12	0.158	4.6E-01
Mtr.41133.1.S1_at	9.03	5.68	7.90	8.70	8.38	8.62	7.54	1.71	8.57	0.17	1.14	0.185	3.6E-01
Mtr.19270.1.S1_at	6.81	3.43	4.79	5.20	4.25	4.60	5.01	1.70	4.68	0.48	0.93	-0.097	7.7E-01
Mtr.2220.1.S1_at	4.79	2.11	5.27	5.04	5.43	4.77	4.06	1.70	5.08	0.33	1.25	0.324	3.7E-01
Mtr.40399.1.S1_at	10.22	7.27	10.22	10.26	9.21	10.16	9.24	1.70	9.87	0.58	1.07	0.096	5.7E-01
Mtr.18009.1.S1_at	7.43	4.47	7.40	7.59	6.85	7.59	6.43	1.70	7.35	0.43	1.14	0.192	4.2E-01
Mtr.10890.1.S1_at	6.95	4.64	7.97	7.86	8.84	7.65	6.52	1.70	8.11	0.64	1.25	0.316	2.0E-01
Mtr.23059.1.S1_at	7.79	5.06	8.19	8.02	8.03	8.06	7.01	1.70	8.04	0.02	1.15	0.196	3.6E-01
Mtr.929.1.S1_s_at	8.64	5.51	8.22	8.22	8.17	8.20	7.46	1.70	8.20	0.03	1.10	0.137	4.9E-01
Mtr.32208.1.S1_at	8.22	4.96	7.44	7.80	7.80	8.03	6.87	1.70	7.88	0.14	1.15	0.197	3.7E-01
Mtr.35655.1.S1_at	6.17	3.23	6.19	6.18	6.60	5.02	5.20	1.70	5.93	0.82	1.14	0.191	5.4E-01
Mtr.1510.1.S1_s_at	8.04	5.16	8.16	7.86	8.14	8.17	7.12	1.70	8.06	0.17	1.13	0.178	4.0E-01
Mtr.18163.1.S1_at	3.43	6.36	3.40	3.39	3.36	3.35	4.40	1.70	3.36	0.02	0.76	-0.387	3.5E-01
Mtr.24226.1.S1_s_at	5.05	2.35	5.48	5.73	5.53	5.28	4.29	1.70	5.51	0.22	1.29	0.362	2.8E-01
Mtr.24048.1.S1_at	10.45	7.17	9.57	9.25	9.49	9.35	9.06	1.70	9.36	0.12	1.03	0.047	7.7E-01
Mtr.51876.1.S1_at	8.10	5.30	8.37	8.31	8.00	8.09	7.25	1.70	8.13	0.16	1.12	0.165	4.2E-01
Mtr.32354.1.S1_at	6.49	3.68	6.74	6.59	6.48	6.32	5.64	1.70	6.46	0.13	1.15	0.198	4.5E-01
Mtr.5402.1.S1_s_at	6.44	3.16	5.56	5.99	6.00	6.40	5.05	1.70	6.13	0.24	1.21	0.279	3.4E-01
Mtr.42343.1.S1_at	8.73	5.41	7.68	8.62	8.46	8.72	7.27	1.70	8.60	0.13	1.18	0.242	2.5E-01
Mtr.33482.1.S1_s_at	6.53	3.16	5.16	5.10	6.99	5.78	4.95	1.70	5.95	0.96	1.20	0.267	4.2E-01
Mtr.28200.1.S1_at	5.97	3.10	6.10	6.09	5.87	5.78	5.06	1.69	5.91	0.16	1.17	0.225	4.3E-01
Mtr.24911.1.S1_x_at	2.37	5.33	2.41	2.38	2.39	2.43	3.37	1.69	2.40	0.03	0.71	-0.490	3.8E-01
Mtr.43711.1.S1_at	7.86	5.42	4.61	4.52	4.32	4.46	5.96	1.69	4.44	0.10	0.74	-0.427	1.9E-01
Mtr.27999.1.S1_at	6.43	3.07	4.35	4.35	4.35	4.34	4.62	1.69	4.34	0.01	0.94	-0.089	7.9E-01
Mtr.11770.1.S1_s_at	6.59	3.28	5.57	5.33	5.27	5.52	5.15	1.69	5.38	0.13	1.04	0.063	8.3E-01

Mtr.1784.1.S1_at	7.87	4.89	7.76	7.11	7.18	6.99	6.84	1.69	7.09	0.10	1.04	0.052	8.1E-01
Mtr.1067.1.S1_at	4.84	7.65	4.61	4.60	3.79	4.55	5.70	1.69	4.31	0.46	0.76	-0.402	2.4E-01
Mtr.48134.1.S1_at	4.96	2.45	5.67	4.70	5.08	4.62	4.36	1.69	4.80	0.25	1.10	0.139	6.8E-01
Mtr.47490.1.S1_x_at	6.95	3.57	5.05	6.88	6.77	7.04	5.19	1.69	6.90	0.14	1.33	0.411	1.6E-01
Mtr.47490.1.S1_s_at	8.03	4.86	7.47	8.12	8.22	8.33	6.78	1.69	8.22	0.11	1.21	0.277	2.2E-01
Mtr.26005.1.S1_at	8.37	5.33	8.15	8.34	8.23	8.30	7.28	1.69	8.29	0.06	1.14	0.187	3.6E-01
Mtr.5306.1.S1_at	2.42	5.33	2.39	2.38	2.38	2.37	3.38	1.69	2.38	0.00	0.70	-0.509	3.6E-01
Mtr.11104.1.S1_s_at	6.74	3.50	5.96	7.31	7.77	7.71	5.40	1.69	7.60	0.25	1.41	0.492	9.0E-02
Mtr.12199.1.S1_at	10.54	7.88	7.41	9.97	9.51	10.23	8.61	1.69	9.90	0.37	1.15	0.202	2.6E-01
Mtr.39727.1.S1_s_at	9.76	6.96	9.99	10.18	9.90	9.85	8.91	1.69	9.98	0.18	1.12	0.164	3.4E-01
Mtr.11200.1.S1_at	7.33	5.53	8.91	8.23	8.38	7.69	7.26	1.69	8.10	0.36	1.12	0.158	4.5E-01
Mtr.40195.1.S1_at	13.02	9.68	10.90	11.27	11.02	11.52	11.20	1.69	11.27	0.25	1.01	0.009	9.5E-01
Mtr.40397.1.S1_at	8.59	5.24	6.55	8.09	8.56	8.13	6.79	1.69	8.26	0.26	1.22	0.282	2.1E-01
Mtr.32922.1.S1_at	8.07	4.78	7.10	6.74	6.74	7.01	6.65	1.69	6.83	0.15	1.03	0.039	8.6E-01
Mtr.41728.1.S1_at	5.46	2.22	4.65	4.30	4.13	3.66	4.11	1.69	4.03	0.33	0.98	-0.028	9.4E-01
Mtr.49269.1.S1_at	6.62	3.65	6.53	5.93	6.03	6.23	5.60	1.69	6.07	0.15	1.08	0.115	6.6E-01
Mtr.13551.1.S1_at	6.16	2.99	5.60	6.24	6.03	5.85	4.92	1.69	6.04	0.19	1.23	0.297	3.2E-01
Mtr.19279.1.S1_at	6.69	5.09	3.32	5.38	5.29	5.82	5.03	1.69	5.50	0.28	1.09	0.127	6.6E-01
Mtr.12736.1.S1_at	6.67	4.81	8.18	7.05	7.44	6.83	6.56	1.69	7.11	0.31	1.08	0.116	6.1E-01
Mtr.34295.1.S1_at	2.44	5.35	2.42	2.42	2.41	2.42	3.40	1.69	2.42	0.01	0.71	-0.495	3.7E-01
Mtr.11963.1.S1_s_at	5.19	2.13	2.43	2.43	2.46	2.38	3.25	1.69	2.42	0.04	0.75	-0.425	4.4E-01
Mtr.13898.1.S1_at	8.26	5.84	9.08	8.97	8.68	8.60	7.73	1.69	8.75	0.20	1.13	0.179	3.6E-01
Mtr.42734.1.S1_at	4.34	2.95	6.31	5.83	4.87	5.77	4.53	1.69	5.49	0.54	1.21	0.276	4.0E-01
Mtr.13437.1.S1_at	6.56	3.23	5.35	5.69	5.81	6.05	5.04	1.69	5.85	0.18	1.16	0.214	4.6E-01
Mtr.46129.1.S1_at	2.86	5.72	2.74	2.80	2.79	2.77	3.77	1.69	2.79	0.01	0.74	-0.436	3.7E-01
Mtr.16417.1.S1_at	4.53	2.43	5.76	4.47	5.89	4.34	4.24	1.68	4.90	0.86	1.16	0.209	5.8E-01
Mtr.37033.1.S1_s_at	7.03	4.34	7.45	7.54	6.71	7.43	6.27	1.68	7.23	0.45	1.15	0.204	4.0E-01
Mtr.20405.1.S1_at	3.40	6.32	3.40	3.58	3.35	3.35	4.37	1.68	3.43	0.13	0.78	-0.352	3.9E-01
Mtr.37329.1.S1_at	2.54	5.45	2.52	2.51	2.52	2.50	3.50	1.68	2.51	0.01	0.72	-0.481	3.6E-01
Mtr.42182.1.S1_at	5.03	2.96	6.29	5.07	6.26	5.29	4.76	1.68	5.54	0.64	1.16	0.218	5.0E-01
Mtr.22051.1.S1_at	8.60	5.51	8.19	8.43	8.00	8.37	7.43	1.68	8.27	0.24	1.11	0.153	4.4E-01
Mtr.32628.1.S1_at	2.35	5.25	2.34	2.34	2.34	2.34	3.31	1.68	2.34	0.00	0.71	-0.502	3.7E-01
Mtr.18524.1.S1_at	5.56	3.06	6.26	6.23	5.55	5.57	4.96	1.68	5.78	0.39	1.17	0.221	4.6E-01
Mtr.46849.1.S1_at	2.69	5.57	2.64	2.64	2.64	2.62	3.63	1.68	2.63	0.01	0.72	-0.465	3.6E-01
Mtr.3041.1.S1_at	2.40	5.32	2.43	2.41	2.39	2.39	3.39	1.68	2.40	0.01	0.71	-0.499	3.6E-01
Mtr.24557.1.S1_at	3.47	6.06	2.92	2.88	2.92	3.18	4.15	1.68	3.00	0.16	0.72	-0.471	3.0E-01
Mtr.32315.1.S1_at	6.04	2.69	4.54	5.22	5.33	5.41	4.42	1.68	5.32	0.09	1.20	0.267	4.1E-01
Mtr.4401.1.S1_at	5.65	2.74	5.65	5.82	5.85	5.61	4.68	1.68	5.76	0.13	1.23	0.300	3.3E-01
Mtr.15595.1.S1_at	2.79	5.72	2.85	2.77	2.77	3.48	3.79	1.68	3.01	0.41	0.79	-0.332	4.8E-01
Mtr.50995.1.S1_at	4.61	1.65	4.50	4.61	4.94	4.74	3.59	1.68	4.77	0.16	1.33	0.410	2.9E-01
Mtr.31515.1.S1_at	7.09	4.13	6.98	7.20	7.30	7.14	6.06	1.68	7.21	0.08	1.19	0.250	3.0E-01
Mtr.31859.1.S1_at	4.52	2.52	5.85	3.61	5.00	4.13	4.30	1.68	4.24	0.70	0.99	-0.018	9.6E-01
Mtr.52007.1.S1_at	7.47	5.78	9.13	8.73	8.56	7.94	7.46	1.68	8.41	0.42	1.13	0.174	3.9E-01
Mtr.11344.1.S1_at	7.01	4.14	7.08	7.00	7.18	7.52	6.08	1.68	7.23	0.26	1.19	0.251	3.0E-01
Mtr.42048.1.S1_at	7.61	4.66	7.50	7.62	7.75	7.88	6.59	1.68	7.75	0.13	1.18	0.234	3.0E-01
Mtr.38274.1.S1_at	8.84	5.72	8.34	8.11	7.97	7.82	7.63	1.67	7.97	0.15	1.04	0.061	7.5E-01
Mtr.18270.1.S1_x_at	1.96	4.85	1.95	1.95	1.94	1.95	2.92	1.67	1.95	0.00	0.67	-0.585	3.7E-01
Mtr.12774.1.S1_at	8.85	5.52	7.51	7.70	7.31	7.84	7.29	1.67	7.62	0.28	1.04	0.063	7.6E-01
Mtr.2929.1.S1_at	6.27	3.55	6.59	5.93	6.17	5.78	5.47	1.67	5.96	0.19	1.09	0.124	6.4E-01
Mtr.38430.1.S1_at	10.19	6.96	9.34	9.97	10.03	9.29	8.83	1.67	9.76	0.41	1.11	0.145	4.0E-01
Mtr.33545.1.S1_s_at	8.91	5.62	6.74	7.83	7.79	7.78	7.09	1.67	7.80	0.02	1.10	0.138	5.0E-01
Mtr.32894.1.S1_at	6.75	3.51	5.83	6.28	5.49	6.78	5.36	1.67	6.18	0.65	1.15	0.205	4.7E-01
Mtr.36177.1.S1_at	10.71	7.60	10.25	10.29	10.23	10.17	9.52	1.67	10.23	0.06	1.07	0.104	5.0E-01
Mtr.39522.1.S1_at	6.19	3.50	6.56	6.22	6.66	5.49	5.42	1.67	6.12	0.59	1.13	0.177	5.3E-01
Mtr.35103.1.S1_at	6.66	3.53	6.11	6.10	6.11	6.11	5.43	1.67	6.11	0.00	1.12	0.168	5.2E-01
Mtr.17239.1.S1_at	5.83	2.63	5.07	5.24	5.87	5.60	4.51	1.67	5.57	0.32	1.23	0.304	3.4E-01
Mtr.9259.1.S1_at	6.73	3.59	6.15	6.01	5.98	6.01	5.49	1.67	6.00	0.01	1.09	0.128	6.3E-01
Mtr.26307.1.S1_at	7.21	4.48	7.52	7.33	7.50	7.39	6.40	1.67	7.41	0.08	1.16	0.211	3.6E-01
Mtr.7363.1.S1_at	10.14	6.82	8.85	9.36	8.88	9.34	8.60	1.67	9.19	0.27	1.07	0.096	5.8E-01
Mtr.43233.1.S1_at	8.52	6.07	9.26	7.84	9.02	8.73	7.95	1.67	8.53	0.61	1.07	0.100	6.1E-01
Mtr.5360.1.S1_s_at	3.60	6.30	3.26	3.41	3.65	3.58	4.39	1.67	3.54	0.12	0.81	-0.309	4.3E-01
Mtr.43715.1.S1_at	8.32	5.56	8.55	8.37	8.10	8.47	7.48	1.67	8.31	0.19	1.11	0.153	4.4E-01
Mtr.12203.1.S1_at	8.87	7.03	10.36	9.01	9.29	8.55	8.75	1.67	8.95	0.38	1.02	0.032	8.5E-01
Mtr.16291.1.S1_x_at	4.36	6.36	3.06	3.90	4.25	3.81	4.59	1.67	3.99	0.24	0.87	-0.204	5.7E-01
Mtr.13775.1.S1_at	6.91	4.06	6.98	6.98	7.04	7.00	5.99	1.67	7.01	0.03	1.17	0.228	3.5E-01
Mtr.8083.1.S1_at	2.37	5.26	2.38	2.33	2.38	2.36	3.34	1.67	2.36	0.02	0.71	-0.502	3.7E-01
Mtr.14760.1.S1_at	8.32	5.04	7.19	6.91	6.67	7.28	6.85	1.67	6.95	0.30	1.01	0.021	9.2E-01
Mtr.35168.1.S1_s_at	8.70	11.92	9.58	10.91	10.74	10.39	10.07	1.67	10.68	0.27	1.06	0.085	5.6E-01
Mtr.28646.1.S1_s_at	6.72	3.39	5.08	5.35	6.56	6.30	5.07	1.67	6.07	0.63	1.20	0.260	3.9E-01
Mtr.25237.1.S1_x_at	3.26	6.06	3.10	3.13	3.05	3.32	4.14	1.66	3.17	0.14	0.77	-0.386	3.7E-01
Mtr.29302.1.S1_at	6.66	4.34	7.57	7.08	7.05	6.99	6.19	1.66	7.04	0.05	1.14	0.186	4.3E-01
Mtr.2500.1.S1_at	4.62	2.64	5.95	4.91	5.03	5.22	4.41	1.66	5.05	0.16	1.15	0.198	5.4E-01
Mtr.13648.1.S1_at	5.26	3.09	6.37	5.91	6.18	5.92	4.91	1.66	6.00	0.16	1.22	0.291	3.2E-01
Mtr.888.1.S1_at	3.61	6.52	3.67	3.65	3.65	3.64	4.60	1.66	3.65	0.00	0.79	-0.335	3.8E-01
Mtr.35089.1.S1_at	7.35	4.39	7.18	7.40	7.12	7.17	6.31	1.66	7.23	0.15	1.15	0.197	3.9E-01
Mtr.48361.1.S1_at	3.14	6.02	3.14	3.15	3.13	3.14	4.10	1.66	3.14	0.01	0.77	-0.384	3.7E-01
Mtr.15362.1.S1_at	2.68	5.55	2.66	2.65	2.38	2.64	3.63	1.66	2.56	0.15	0.70	-0.505	3.3E-01
Mtr.8386.1.S1_at	2.67	5.50	2.58	2.65	2.64	3.01	3.59	1.66	2.76	0.21	0.77	-0.376	4.4E-01
Mtr.10085.1.S1_at	8.39	5.38	8.09	8.29	8.65	8.59	7.29	1.66	8.51	0.20	1.17	0.224	2.7E-01
Mtr.2020.1.S1_at	8.22	5.05	5.78	7.86	7.33	8.07	6.35	1.66	7.75	0.38	1.22	0.288	2.3E-01
Mtr.46581.1.S1_at	2.79	5.57	2.61	2.60	2.57	2.61	3.66	1.66	2.59	0.02	0.71	-0.497	3.3E-01
Mtr.44821.1.S1_at	5.92	2.68	4.93	5.32	5.19	5.64	4.51	1.66	5.39	0.23	1.19	0.256	4.2E-01
Mtr.10167.1.S1_at	7.74	4.69	7.35	7.41	7.20	7.47	6.59	1.66	7.36	0.14	1.12	0.159	4.7E-01
Mtr.33424.1.S1_at	8.22	4.96	7.12	7.24	7.24	6.84	6.77	1.66	7.11	0.23	1.05	0.071	7.4E-01
Mtr.43288.1.S1_at	9.17	6.25	9.07	8.82	8.73	8.32	8.16	1.66	8.62	0.27	1.06	0.079	6.6E-01
Mtr.1285.1.S1_at	10.13	12.99	10.09	10.26	9.80	10.02	11.07	1.66	10.03	0.23	0.91	-0.143	3.4E-01
Mtr.28087.1.S1_s_at	7.51	4.23	5.42	6.18	5.80	6.32	5.72	1.66	6.10	0.27	1.07	0.093	7.1E-01
Mtr.8363.1.S1_at	8.51	5.65	8.54	7.97	7.88	8.11	7.57	1.66	7.99	0.12	1.06	0.078	6.9E-01
Mtr.35328.1.S1_at	5.45	3.27	6.53	8.30	9.02	8.70	5.08	1.66	8.67	0.36	1.71	0.771	2.2E-02
Mtr.6205.1.S1_at	2.47	5.45	2.70	2.44	2.43	2.42	3.54	1.66	2.43	0.01	0.69	-0.542	3.1E-01
Mtr.42967.1.S1_at	7.31	4.42	7.27	8.11	10.07	9.67	6.33	1.66	9.28	1.04	1.47	0.551	5.9E-02
Mtr.22658.1.S1_at	2.57	5.44	2.57	2.53	2.52	2.51	3.53	1.66	2.52	0.01	0.71	-0.486	3.5E-01
Mtr.563.1.S1_at	3.08	5.94	3.07	3.22	2.76	3.10	4.03	1.65	3.03	0.24	0.75	-0.413	3.6E-01
Mtr.34911.1.S1_at	6.31	3.48	6.39	6.27	6.26	6.27	5.39	1.65	6.26	0.00	1.16	0.216	4.1E-01
Mtr.38932.1.S1_at	2.27	3.53	5.55	4.65	6.60	4.98	3.78	1.65	5.41	1.05	1.43	0.516	2.2E-01
Mtr.226.1.S1_at	2.55	5.40	2.53	2.52	2.51	2.52	3.49	1.65	2.51	0.01	0.72	-0.475	3.6E-01
Mtr.49277.1.S1_at	5.50	3.02	6.15	5.84	5.51	5.35	4.89	1.65	5.57	0.25	1.14	0.188	5.2E-01
Mtr.41273.1.S1_at	10.73	7.44	8.92	8.16	7.42	8.12	9.03	1.65	7.90	0.42	0.87	-0.193	3.1E-01
Mtr.48892.1.S1_at	5.11	3.16	6.44	7.84	8.11	6.83	4.90	1.65	7.59	0.68	1.55	0.632	5.9E-02
Mtr.37357.1.S1_at	6.90	3.89	6.57	8.16	8.08	8.61	5.79	1.65	8.29	0.29	1.43	0.517	6.1E-02
Mtr.12519.1.S1_at	6.92	5.44	8.73	8.34	9.23	8.44	7.03	1.65	8.67	0.49	1.23	0.303	1.7E-01
Mtr.33547.1.S1_at	9.07	6.40	9.41	9.42	9.52	9.19	8.29	1.65	9.38	0.17	1.13	0.177	3.2E-01
Mtr.37624.1.S1_s_at	6.03	2.86	5.21	5.68	5.02	4.93	4.70	1.65	5.21	0.41	1.11	0.149	6.3E-01
Mtr.29991.1.S1_at	2.42	5.26	2.40	2.39	2.39	2.38	3.36	1.65	2.39	0.01	0.71	-0.491	3.7E-01
Mtr.31305.1.S1_at	5.04	2.67	5.84	5.62	5.36	5.56	4.52	1.65	5.51	0.14	1.22	0.288	3.5E-01
Mtr.1940.1.S1_at	10.86	8.09	11.02	10.52	10.01	10.30	9.99	1.64	10.28	0.26	1.03	0.042	7.8E-01
Mtr.8530.1.S1_at	8.41	5.35	7.91	7.42	7.71	7.29	7.23	1.64	7.47	0.21	1.03	0.048	8.1E-01
Mtr.9773.1.S1_at	6.91	3.63	5.43	6.31	6.24	6.38	5.32	1.64	6.31	0.07	1.19	0.247	3.6E-01
Mtr.20946.1.S1_at	7.66	4.98	7.97	8.58	8.16	8.60	6.87	1.64	8.45	0.25	1.23	0.298	1.8E-01
Mtr.43641.1.S1_at	8.08	5.50	8.55	8.43	8.21	8.21	7.37	1.64	8.28	0.13	1.12	0.168	3.9E-01
Mtr.4713.1.S1_at	7.40	4.77	7.78	7.90	7.75	7.93	6.65	1.64	7.86	0.10	1.18	0.241	2.7E-01
Mtr.16678.1.S1_at	6.83	9.77	7.05	6.90	6.57	6.89	7.88	1.64	6.79	0.18	0.86	-0.216	3.1E-01
Mtr.11654.1.S1_at	9.06	5.82	7.90	8.59	8.37	8.61	7.59	1.64	8.52	0.13	1.12	0.167	3.8E-01
Mtr.15307.1.S1_at	6.53	4.28	7.47	6.55	7.10	6.83	6.09	1.64	6.83	0.28	1.12	0.164	4.9E-01
Mtr.45072.1.S1_at	6.21	3.45	6.35	6.24	6.08	6.07	5.34	1.64	6.13	0.09	1.15	0.200	4.5E-01
Mtr.39922.1.S1_at	8.95	5.94	8.57	8.91	8.47	8.71	7.82	1.64	8.70	0.22	1.11	0.154	4.1E-01
Mtr.50464.1.S1_s_at	6.82	3.92	6.70	6.82	6.71	6.92	5.81	1.64	6.82	0.10	1.17	0.230	3.5E-01
Mtr.45178.1.S1_at	6.98	4.01	6.70	6.41	6.61	6.59	5.89	1.64	6.54	0.11	1.11	0.150	5.3E-01
Mtr.20062.1.S1_at	5.17	2.53	5.54	5.67	5.68	4.78	4.41	1.64	5.37	0.52	1.22	0.285	3.9E-01
Mtr.24954.1.S1_s_at	9.01	6.17	9.01	8.54	7.96	8.50	8.06	1.64	8.33	0.32	1.03	0.047	7.9E-01
Mtr.31425.1.S1_at	6.71	3.76	6.47	7.47	7.69	7.23	5.65	1.64	7.46	0.23	1.32	0.403	1.3E-01
Mtr.1474.1.S1_at	9.66	7.17	10.25	9.86	9.72	9.56	9.03	1.64	9.71	0.15	1.08	0.106	5.1E-01
Mtr.41993.1.S1_at	6.95	4.16	7.04	7.08	7.44	7.26	6.05	1.64	7.26	0.18	1.20	0.264	2.7E-01
Mtr.15399.1.S1_s_at	2.42	5.25	2.41	2.41	2.41	2.41	3.36	1.64	2.41	0.00	0.72	-0.481	3.7E-01
Mtr.47777.1.S1_at	6.39	3.69	6.64	6.28	6.37	6.14	5.58	1.64	6.26	0.12	1.12	0.168	5.1E-01
Mtr.45057.1.S1_at	6.54	3.59	6.30	7.30	5.53	7.21	5.48	1.64	6.68	1.00	1.22	0.286	3.4E-01
Mtr.10435.1.S1_s_at	11.12	7.86	9.75	10.31	10.05	10.52	9.58	1.64	10.29	0.24	1.07	0.104	5.0E-01
Mtr.33644.1.S1_at	2.38	5.22	2.38	2.37	2.37	2.37	3.33	1.64	2.37	0.00	0.71	-0.490	3.7E-01
Mtr.29480.1.S1_at	2.03	4.86	2.02	2.02	2.02	2.02	2.97	1.64	2.02	0.00	0.68	-0.559	3.7E-01
Mtr.10269.1.S1_at	5.81	3.31	6.38	6.22	6.07	5.81	5.17	1.64	6.03	0.21	1.17	0.223	4.1E-01
Mtr.23026.1.S1_at	6.91	3.91	6.53	6.61	6.33	6.88	5.78	1.64	6.61	0.27	1.14	0.193	4.4E-01

Mtr.31510.1.S1_at	6.58	4.12	7.21	7.91	7.34	7.28	5.97	1.64	7.51	0.35	1.26	0.332	1.9E-01
Mtr.40059.1.S1_at	8.02	4.93	7.42	8.06	8.35	7.85	6.79	1.63	8.09	0.25	1.19	0.252	2.5E-01
Mtr.27354.1.S1_at	2.70	5.52	2.67	2.67	2.63	2.66	3.63	1.63	2.65	0.02	0.73	-0.455	3.6E-01
Mtr.33792.1.S1_at	6.67	5.44	8.67	7.64	7.43	7.96	6.93	1.63	7.68	0.27	1.11	0.148	4.8E-01
Mtr.9670.1.S1_at	9.15	6.03	6.76	7.88	7.85	8.13	7.31	1.63	7.95	0.15	1.09	0.121	5.4E-01
Mtr.35957.1.S1_at	7.48	4.82	7.80	7.53	7.46	7.28	6.70	1.63	7.42	0.13	1.11	0.147	4.9E-01
Mtr.32470.1.S1_at	7.91	5.23	8.18	8.59	8.19	7.99	7.11	1.63	8.25	0.31	1.16	0.216	3.0E-01
Mtr.23254.1.S1_at	6.49	3.42	5.92	6.03	5.77	5.74	5.28	1.63	5.85	0.16	1.11	0.147	5.8E-01
Mtr.3634.1.S1_at	5.76	2.84	5.55	5.73	5.49	5.70	4.72	1.63	5.64	0.13	1.20	0.257	3.8E-01
Mtr.45367.1.S1_at	9.11	6.74	9.87	9.66	9.37	9.26	8.58	1.63	9.43	0.21	1.10	0.137	4.2E-01
Mtr.41048.1.S1_at	5.43	3.63	6.89	7.08	7.62	7.34	5.32	1.63	7.35	0.27	1.38	0.467	1.0E-01
Mtr.22443.1.S1_at	2.64	5.46	2.62	2.63	2.63	2.63	3.57	1.63	2.63	0.00	0.73	-0.444	3.7E-01
Mtr.1895.1.S1_at	7.14	4.22	6.94	7.26	7.19	6.97	6.10	1.63	7.14	0.15	1.17	0.227	3.3E-01
Mtr.13754.1.S1_at	6.37	3.75	6.75	6.38	6.49	6.36	5.62	1.63	6.41	0.07	1.14	0.189	4.5E-01
Mtr.6543.1.S1_at	8.91	6.23	9.17	9.47	9.75	9.47	8.10	1.63	9.56	0.16	1.18	0.239	2.0E-01
Mtr.31615.1.S1_s_at	6.96	4.36	7.36	7.32	7.85	7.91	6.23	1.63	7.69	0.33	1.24	0.305	2.0E-01
Mtr.40695.1.S1_at	5.68	2.43	4.27	3.92	3.69	3.68	4.13	1.63	3.76	0.14	0.91	-0.133	7.2E-01
Mtr.33206.1.S1_s_at	6.98	4.67	7.81	7.64	7.73	7.84	6.49	1.63	7.74	0.10	1.19	0.254	2.6E-01
Mtr.27492.1.S1_at	7.23	4.34	7.09	7.46	7.12	6.83	6.22	1.63	7.13	0.32	1.15	0.197	3.9E-01
Mtr.10433.1.S1_at	9.05	5.93	8.27	8.34	8.63	8.67	7.75	1.63	8.55	0.18	1.10	0.141	4.5E-01
Mtr.2253.1.S1_at	6.93	4.90	8.12	7.42	6.95	6.78	6.65	1.63	7.05	0.33	1.06	0.085	7.0E-01
Mtr.10615.1.S1_at	10.36	7.87	10.92	10.67	10.70	10.32	9.72	1.62	10.56	0.21	1.09	0.121	4.2E-01
Mtr.23876.1.S1_at	7.35	4.97	8.08	8.12	7.62	8.34	6.80	1.62	8.03	0.37	1.18	0.239	2.7E-01
Mtr.25105.1.S1_at	8.46	5.30	7.53	7.96	8.12	8.08	7.09	1.62	8.05	0.08	1.14	0.183	3.6E-01
Mtr.14994.1.S1_at	11.18	7.96	9.16	9.10	8.88	8.99	9.43	1.62	8.99	0.11	0.95	-0.069	6.6E-01
Mtr.52778.1.S1_s_at	2.81	5.62	2.80	2.82	2.80	2.78	3.74	1.62	2.80	0.02	0.75	-0.419	3.7E-01
Mtr.26738.1.S1_at	7.14	4.17	6.79	7.26	7.13	7.25	6.03	1.62	7.21	0.07	1.20	0.258	2.8E-01
Mtr.33362.1.S1_at	8.29	6.00	9.14	8.32	8.41	8.17	7.81	1.62	8.30	0.12	1.06	0.088	6.3E-01
Mtr.36642.1.S1_at	4.35	6.95	3.97	4.42	4.41	4.61	5.09	1.62	4.48	0.11	0.88	-0.185	5.5E-01
Mtr.1833.1.S1_at	6.70	4.00	6.91	6.34	6.99	7.02	5.87	1.62	6.78	0.38	1.16	0.209	4.0E-01
Mtr.2200.1.S1_at	7.39	4.51	7.25	6.81	6.11	6.01	6.38	1.62	6.31	0.44	0.99	-0.017	9.4E-01
Mtr.50872.1.S1_x_at	2.39	5.19	2.37	2.37	2.36	2.35	3.31	1.62	2.36	0.01	0.71	-0.490	3.7E-01
Mtr.8413.1.S1_at	8.22	5.41	8.22	8.05	7.97	7.69	7.28	1.62	7.90	0.19	1.09	0.118	5.5E-01
Mtr.7455.1.S1_at	2.67	2.57	5.43	4.67	4.67	4.28	3.56	1.62	4.54	0.23	1.28	0.352	3.6E-01
Mtr.37807.1.S1_at	6.38	5.56	8.69	7.23	7.12	6.38	6.88	1.62	6.91	0.46	1.00	0.007	9.8E-01
Mtr.42449.1.S1_at	6.04	2.87	5.02	6.42	6.31	5.98	4.64	1.62	6.24	0.23	1.34	0.426	1.7E-01
Mtr.27923.1.S1_at	5.40	8.22	5.42	5.59	5.92	5.21	6.35	1.62	5.57	0.36	0.88	-0.188	4.6E-01
Mtr.14755.1.S1_at	6.82	3.85	6.45	6.49	6.34	6.35	5.71	1.62	6.39	0.08	1.12	0.163	5.1E-01
Mtr.25742.1.S1_at	3.05	5.85	3.04	3.01	3.01	3.02	3.98	1.62	3.01	0.00	0.76	-0.402	3.6E-01
Mtr.27528.1.S1_at	6.78	3.66	5.96	5.92	5.92	6.31	5.46	1.62	6.05	0.23	1.11	0.148	5.7E-01
Mtr.3357.1.S1_at	8.12	5.10	7.62	7.43	8.11	7.90	6.95	1.62	7.81	0.34	1.12	0.169	4.2E-01
Mtr.23946.1.S1_at	4.50	2.87	6.11	6.03	4.47	4.88	4.49	1.62	5.13	0.81	1.14	0.190	5.8E-01
Mtr.18377.1.S1_at	3.15	5.93	3.11	3.20	3.26	3.00	4.06	1.62	3.16	0.14	0.78	-0.365	3.9E-01
Mtr.10084.1.S1_at	6.90	4.05	6.80	6.65	6.64	6.55	5.91	1.62	6.62	0.06	1.12	0.162	4.9E-01
Mtr.5362.1.S1_s_at	6.30	3.20	5.54	6.53	6.58	6.70	5.01	1.62	6.60	0.09	1.32	0.397	1.6E-01
Mtr.41481.1.S1_at	9.22	6.73	9.77	9.63	9.37	9.38	8.57	1.62	9.46	0.15	1.10	0.142	4.0E-01
Mtr.5792.1.S1_at	10.17	7.00	9.12	7.40	6.99	7.29	8.76	1.62	7.23	0.21	0.82	-0.278	1.8E-01
Mtr.36889.1.S1_s_at	12.67	9.44	11.12	13.30	12.07	13.36	11.08	1.62	12.91	0.73	1.17	0.221	1.5E-01
Mtr.26712.1.S1_at	2.47	5.26	2.46	2.46	2.45	2.45	3.40	1.62	2.45	0.01	0.72	-0.470	3.7E-01
Mtr.449.1.S1_at	10.32	7.35	9.92	10.04	9.93	9.93	9.20	1.62	9.96	0.06	1.08	0.116	4.6E-01
Mtr.13150.1.S1_at	6.42	3.49	6.12	5.81	5.24	5.69	5.34	1.62	5.58	0.30	1.04	0.062	8.2E-01
Mtr.6455.1.S1_at	2.29	5.08	2.28	2.27	2.27	2.27	3.22	1.61	2.27	0.00	0.71	-0.502	3.7E-01
Mtr.14679.1.S1_at	5.68	3.08	6.03	5.05	6.04	5.75	4.93	1.61	5.61	0.51	1.14	0.186	5.3E-01
Mtr.47442.1.S1_s_at	7.90	6.03	9.25	8.88	9.11	8.78	7.73	1.61	8.92	0.17	1.15	0.208	2.7E-01
Mtr.48369.1.S1_at	6.31	3.14	5.27	4.87	4.62	5.49	4.91	1.61	4.99	0.45	1.02	0.025	9.3E-01
Mtr.36003.1.S1_at	3.08	5.85	3.04	2.90	2.82	2.83	3.99	1.61	2.85	0.04	0.71	-0.485	2.9E-01
Mtr.25581.1.S1_at	2.56	5.32	2.49	2.95	2.50	2.51	3.46	1.61	2.65	0.26	0.77	-0.382	4.4E-01
Mtr.5680.1.S1_at	7.56	4.79	7.61	7.34	7.29	7.36	6.65	1.61	7.33	0.04	1.10	0.140	5.1E-01
Mtr.13374.1.S1_at	8.33	6.47	9.68	9.02	9.14	8.67	8.16	1.61	8.94	0.24	1.10	0.132	4.5E-01
Mtr.17591.1.S1_at	2.64	5.43	2.62	2.62	2.62	2.61	3.56	1.61	2.62	0.00	0.73	-0.445	3.7E-01
Mtr.14856.1.S1_at	8.58	6.28	9.39	9.35	9.17	9.13	8.08	1.61	9.22	0.11	1.14	0.190	2.9E-01
Mtr.51653.1.S1_at	8.08	5.28	8.07	8.17	7.92	7.80	7.14	1.61	7.96	0.19	1.12	0.157	4.3E-01
Mtr.18103.1.S1_at	6.37	3.94	6.99	6.31	6.80	6.72	5.76	1.61	6.61	0.26	1.15	0.197	4.2E-01
Mtr.39600.1.S1_at	2.48	5.27	2.48	2.47	2.47	2.47	3.41	1.61	2.47	0.00	0.72	-0.465	3.7E-01
Mtr.10410.1.S1_s_at	7.77	4.72	7.17	7.33	6.85	7.24	6.55	1.61	7.14	0.26	1.09	0.124	5.7E-01
Mtr.18743.1.S1_at	10.33	7.45	10.14	10.16	10.36	10.23	9.31	1.61	10.25	0.11	1.10	0.140	3.7E-01
Mtr.12321.1.S1_at	12.58	10.73	9.37	9.58	10.50	10.22	10.89	1.61	10.10	0.47	0.93	-0.109	4.6E-01
Mtr.5338.1.S1_at	6.68	3.88	6.66	7.02	7.06	7.07	5.74	1.61	7.05	0.03	1.23	0.297	2.3E-01
Mtr.42517.1.S1_at	6.27	3.86	6.91	6.54	6.31	6.31	5.68	1.61	6.39	0.13	1.13	0.170	4.9E-01
Mtr.35625.1.S1_s_at	6.14	4.01	2.98	3.93	4.10	4.64	4.38	1.61	4.22	0.37	0.96	-0.052	8.8E-01
Mtr.29654.1.S1_at	2.53	5.31	2.52	2.52	2.52	2.49	3.45	1.61	2.51	0.02	0.73	-0.461	3.7E-01
Mtr.27182.1.S1_s_at	6.32	3.51	6.26	6.05	6.50	6.13	5.37	1.61	6.23	0.24	1.16	0.214	4.1E-01
Mtr.5547.1.S1_s_at	9.23	6.65	9.59	9.62	9.62	9.09	8.49	1.61	9.45	0.30	1.11	0.154	3.7E-01
Mtr.32909.1.S1_at	4.93	2.45	5.47	4.91	4.55	5.22	4.28	1.61	4.89	0.33	1.14	0.193	5.5E-01
Mtr.50671.1.S1_at	2.77	5.52	2.70	2.72	2.45	2.93	3.66	1.61	2.70	0.24	0.74	-0.439	3.6E-01
Mtr.9513.1.S1_at	7.96	5.26	8.11	7.38	6.87	7.21	7.11	1.61	7.15	0.26	1.01	0.008	9.7E-01
Mtr.18939.1.S1_at	9.17	7.12	10.29	9.89	9.77	9.94	8.86	1.61	9.87	0.09	1.11	0.156	3.4E-01
Mtr.21140.1.S1_at	3.36	6.19	3.46	3.75	3.49	3.91	4.33	1.61	3.71	0.21	0.86	-0.223	5.4E-01
Mtr.12092.1.S1_at	5.20	3.62	6.83	8.15	8.11	8.51	5.21	1.61	8.26	0.22	1.58	0.663	3.1E-02
Mtr.26464.1.S1_at	5.73	2.85	5.52	3.88	6.00	5.41	4.70	1.60	5.09	1.09	1.08	0.117	7.4E-01
Mtr.2577.1.S1_at	6.54	3.76	6.53	6.78	6.60	5.97	5.61	1.60	6.45	0.42	1.15	0.202	4.3E-01
Mtr.6614.1.S1_at	2.46	5.24	2.45	2.45	2.31	2.45	3.38	1.60	2.40	0.08	0.71	-0.495	3.5E-01
Mtr.8967.1.S1_s_at	2.34	5.12	2.34	2.32	2.30	2.31	3.26	1.60	2.31	0.01	0.71	-0.498	3.6E-01
Mtr.31160.1.S1_at	6.39	3.43	3.85	3.18	3.51	3.52	4.56	1.60	3.40	0.20	0.75	-0.422	2.8E-01
Mtr.13612.1.S1_at	6.00	3.28	6.12	5.85	6.07	5.63	5.13	1.60	5.85	0.22	1.14	0.187	4.9E-01
Mtr.47879.1.S1_at	8.15	5.30	8.00	8.67	8.56	8.49	7.15	1.60	8.57	0.09	1.20	0.262	2.0E-01
Mtr.30695.1.S1_at	10.34	7.79	10.75	10.78	10.55	10.43	9.63	1.60	10.59	0.18	1.10	0.137	3.6E-01
Mtr.8319.1.S1_at	7.28	4.47	7.21	6.61	7.57	7.52	6.32	1.60	7.23	0.54	1.14	0.194	4.0E-01
Mtr.44251.1.S1_at	7.57	4.72	7.41	7.73	7.70	7.61	6.56	1.60	7.68	0.07	1.17	0.227	2.9E-01
Mtr.46819.1.S1_s_at	7.91	4.91	7.37	8.28	8.45	8.49	6.73	1.60	8.40	0.11	1.25	0.321	1.4E-01
Mtr.5103.1.S1_at	8.16	5.83	8.90	8.27	8.39	8.21	7.63	1.60	8.29	0.09	1.09	0.120	5.2E-01
Mtr.32585.1.S1_at	5.08	2.63	5.63	5.51	5.55	5.51	4.45	1.60	5.52	0.02	1.24	0.313	3.1E-01
Mtr.34342.1.S1_at	2.40	5.17	2.40	2.39	2.38	2.38	3.32	1.60	2.38	0.01	0.72	-0.480	3.7E-01
Mtr.6227.1.S1_at	2.00	4.76	1.99	1.99	1.98	1.99	2.91	1.60	1.98	0.00	0.68	-0.554	3.7E-01
Mtr.2311.1.S1_at	6.62	3.72	6.33	6.88	6.97	6.79	5.55	1.60	6.88	0.09	1.24	0.309	2.2E-01
Mtr.35241.1.S1_at	6.30	4.08	7.17	6.83	6.86	6.52	5.85	1.60	6.74	0.19	1.15	0.204	3.9E-01
Mtr.34913.1.S1_at	5.82	3.41	6.43	5.47	5.40	5.32	5.22	1.60	5.39	0.07	1.03	0.048	8.6E-01
Mtr.38093.1.S1_at	11.18	9.11	12.25	12.18	11.61	11.84	10.85	1.60	11.87	0.29	1.09	0.130	3.3E-01
Mtr.28476.1.S1_at	5.58	2.86	5.65	5.59	5.84	4.84	4.70	1.60	5.42	0.52	1.15	0.207	5.0E-01
Mtr.16621.1.S1_at	2.38	5.15	2.40	2.38	2.36	2.36	3.31	1.59	2.37	0.01	0.72	-0.482	3.6E-01
Mtr.47987.1.S1_x_at	7.34	4.29	6.63	5.60	4.14	5.70	6.09	1.59	5.15	0.87	0.85	-0.242	4.2E-01
Mtr.34220.1.S1_s_at	2.62	1.73	4.83	2.72	2.61	2.50	3.06	1.59	2.61	0.11	0.85	-0.231	6.5E-01
Mtr.28663.1.S1_at	7.29	4.78	7.74	7.63	7.67	7.34	6.61	1.59	7.55	0.18	1.14	0.192	3.7E-01
Mtr.9169.1.S1_at	10.05	7.58	10.56	10.44	10.31	10.23	9.40	1.59	10.33	0.11	1.10	0.136	3.7E-01
Mtr.22708.1.S1_at	8.08	5.42	8.27	8.16	8.43	8.20	7.26	1.59	8.27	0.15	1.14	0.188	3.4E-01
Mtr.39708.1.S1_at	6.56	3.56	4.15	4.87	4.02	4.00	4.76	1.59	4.30	0.50	0.90	-0.146	6.6E-01
Mtr.32376.1.S1_at	6.12	3.58	6.51	6.82	7.04	6.59	5.40	1.59	6.82	0.22	1.26	0.335	2.0E-01
Mtr.18895.1.S1_at	2.79	5.42	2.56	2.55	2.55	2.54	3.59	1.59	2.55	0.01	0.71	-0.495	3.2E-01
Mtr.6377.1.S1_at	5.10	2.24	4.89	4.89	4.89	4.96	4.07	1.59	4.91	0.04	1.21	0.269	4.1E-01
Mtr.32290.1.S1_at	7.27	4.94	7.98	7.79	7.56	7.54	6.73	1.59	7.63	0.14	1.13	0.181	3.8E-01
Mtr.32184.1.S1_at	9.46	6.39	8.66	8.88	8.69	9.01	8.17	1.59	8.86	0.16	1.08	0.117	5.0E-01
Mtr.6947.1.S1_at	5.16	2.42	2.39	2.21	2.35	2.35	3.32	1.59	2.30	0.08	0.69	-0.529	3.3E-01
Mtr.35800.1.S1_at	6.62	4.14	7.10	6.71	6.77	6.41	5.96	1.59	6.63	0.19	1.11	0.154	5.1E-01
Mtr.35868.1.S1_at	8.94	6.59	9.63	8.93	8.79	8.79	8.39	1.59	8.84	0.08	1.05	0.076	6.5E-01
Mtr.29531.1.S1_at	8.99	6.01	6.55	7.92	8.32	8.21	7.18	1.59	8.15	0.21	1.13	0.182	3.6E-01
Mtr.39017.1.S1_at	5.20	3.06	6.16	5.58	5.26	5.56	4.80	1.59	5.47	0.18	1.14	0.187	5.1E-01
Mtr.11389.1.S1_at	6.85	3.78	6.03	6.18	6.39	6.17	5.55	1.59	6.24	0.13	1.12	0.170	4.9E-01
Mtr.11701.1.S1_at	7.94	5.09	7.72	7.91	7.79	7.68	6.92	1.59	7.79	0.11	1.13	0.172	3.9E-01
Mtr.2009.1.S1_at	2.19	4.93	2.17	2.17	2.17	2.29	3.10	1.59	2.21	0.07	0.71	-0.487	3.9E-01
Mtr.24347.1.S1_at	5.32	2.80	5.74	5.77	5.69	5.69	4.62	1.59	5.72	0.05	1.24	0.308	3.0E-01
Mtr.43762.1.S1_at	10.51	7.34	9.05	9.85	9.61	10.15	8.97	1.59	9.87	0.27	1.10	0.138	3.9E-01
Mtr.1563.1.S1_at	4.35	4.18	7.01	5.66	5.77	5.74	5.18	1.59	5.72	0.06	1.11	0.144	5.8E-01
Mtr.24649.1.S1_at	2.28	4.99	2.22	2.26	2.24	2.26	3.16	1.59	2.25	0.01	0.71	-0.490	3.8E-01
Mtr.44510.1.S1_at	5.95	3.73	6.79	6.56	6.61	6.72	5.49	1.58	6.63	0.08	1.21	0.272	2.8E-01
Mtr.48878.1.S1_at	2.74	5.46	2.69	3.04	2.72	2.68	3.63	1.58	2.81	0.20	0.77	-0.368	4.3E-01
Mtr.10805.1.S1_at	5.89	4.51	7.67	8.57	8.98	8.81	6.03	1.58	8.78	0.21	1.46	0.544	4.0E-02
Mtr.13419.1.S1_at	7.22	4.27	6.73	6.22	6.72	6.50	6.07	1.58	6.48	0.25	1.07	0.093	6.9E-01
Mtr.48114.1.S1_at	2.46	5.20	2.45	2.45	2.43	2.42	3.37	1.58	2.43	0.02	0.72	-0.469	3.6E-01
Mtr.12087.1.S1_at	8.18	5.70	8.64	8.51	8.39	8.39	7.50	1.58	8.43	0.07	1.12	0.168	3.7E-01
Mtr.52189.1.S1_at	2.55	5.27	2.52	2.52	2.51	2.51	3.45	1.58	2.51	0.01	0.73	-0.457	3.6E-01
Mtr.45399.1.S1_at	5.78	3.65	6.74	5.89	6.26	5.88	5.39	1.58	6.01	0.22	1.11	0.156	5.4E-01

Mtr.13269.1.S1_at	6.84	4.23	7.08	6.76	7.03	6.82	6.05	1.58	6.87	0.14	1.14	0.183	4.2E-01
Mtr.48670.1.S1_at	5.74	3.31	6.26	5.68	6.06	5.73	5.10	1.58	5.82	0.21	1.14	0.190	4.8E-01
Mtr.27933.1.S1_at	7.71	5.48	8.53	8.13	7.58	7.84	7.24	1.58	7.85	0.28	1.08	0.117	5.4E-01
Mtr.17718.1.S1_at	5.74	2.92	5.56	5.63	6.01	5.91	4.74	1.58	5.85	0.20	1.24	0.305	2.9E-01
Mtr.7285.1.S1_s_at	5.03	3.04	6.15	5.86	5.49	5.30	4.74	1.58	5.55	0.29	1.17	0.228	4.3E-01
Mtr.39096.1.S1_at	6.08	3.74	6.74	6.75	6.86	6.44	5.52	1.58	6.68	0.22	1.21	0.276	2.7E-01
Mtr.46195.1.S1_s_at	5.20	2.69	5.59	5.00	4.78	4.19	4.49	1.58	4.66	0.42	1.04	0.052	8.7E-01
Mtr.38784.1.S1_at	7.06	4.53	7.42	7.90	7.62	7.58	6.34	1.58	7.70	0.17	1.21	0.281	2.1E-01
Mtr.12528.1.S1_at	12.83	10.24	13.07	13.75	13.55	13.70	12.05	1.57	13.67	0.11	1.13	0.182	1.5E-01
Mtr.17711.1.S1_at	4.82	2.30	5.19	5.54	4.95	4.62	4.10	1.57	5.04	0.47	1.23	0.296	3.8E-01
Mtr.33622.1.S1_at	5.35	2.65	5.39	5.23	5.86	5.03	4.46	1.57	5.37	0.43	1.20	0.268	3.9E-01
Mtr.37719.1.S1_at	6.09	3.10	5.43	5.74	6.09	5.43	4.87	1.57	5.75	0.33	1.18	0.240	4.0E-01
Mtr.47884.1.S1_at	3.26	5.74	2.84	3.75	2.81	3.25	3.95	1.57	3.27	0.47	0.83	-0.270	5.2E-01
Mtr.13641.1.S1_at	7.30	5.06	8.09	8.13	8.92	8.57	6.82	1.57	8.54	0.39	1.25	0.325	1.4E-01
Mtr.33726.1.S1_at	7.02	4.41	7.24	6.93	7.20	6.96	6.22	1.57	7.03	0.15	1.13	0.175	4.3E-01
Mtr.45579.1.S1_s_at	7.05	4.75	7.75	7.52	7.41	7.29	6.52	1.57	7.41	0.11	1.14	0.185	3.8E-01
Mtr.19126.1.S1_at	2.36	5.08	2.35	2.34	2.34	2.34	3.26	1.57	2.34	0.00	0.72	-0.479	3.7E-01
Mtr.13713.1.S1_at	7.45	4.58	7.11	7.17	7.23	7.45	6.38	1.57	7.28	0.15	1.14	0.191	3.8E-01
Mtr.48652.1.S1_at	6.67	3.79	6.32	6.55	6.34	6.53	5.59	1.57	6.47	0.12	1.16	0.211	3.9E-01
Mtr.8422.1.S1_at	7.80	4.93	7.47	7.46	7.46	7.49	6.74	1.57	7.47	0.02	1.11	0.149	4.6E-01
Mtr.11739.1.S1_s_at	5.32	2.94	5.91	5.38	5.38	5.37	4.72	1.57	5.38	0.00	1.14	0.187	5.1E-01
Mtr.35645.1.S1_at	2.77	5.48	2.75	2.75	2.72	2.67	3.67	1.57	2.71	0.04	0.74	-0.435	3.5E-01
Mtr.12062.1.S1_at	7.69	4.83	7.38	7.05	7.06	7.33	6.63	1.57	7.15	0.16	1.08	0.109	6.0E-01
Mtr.15442.1.S1_at	6.32	3.39	3.89	5.75	4.67	5.41	4.53	1.57	5.28	0.55	1.16	0.219	4.8E-01
Mtr.44598.1.S1_at	8.64	6.05	8.88	9.04	9.08	8.78	7.86	1.57	8.97	0.16	1.14	0.191	2.9E-01
Mtr.49091.1.S1_at	4.91	2.78	5.83	5.52	6.05	5.32	4.51	1.57	5.63	0.38	1.25	0.321	2.9E-01
Mtr.49341.1.S1_at	4.37	2.44	5.55	5.54	4.25	4.63	4.12	1.57	4.81	0.66	1.17	0.223	5.2E-01
Mtr.38471.1.S1_at	7.88	5.11	5.22	5.23	4.86	5.22	6.07	1.57	5.10	0.21	0.84	-0.250	3.5E-01
Mtr.22226.1.S1_at	2.36	5.06	2.33	2.33	2.26	2.32	3.25	1.57	2.30	0.04	0.71	-0.497	3.5E-01
Mtr.9149.1.S1_at	8.03	5.37	8.12	8.31	8.57	8.18	7.17	1.57	8.35	0.20	1.16	0.220	2.7E-01
Mtr.15448.1.S1_at	4.75	7.81	6.84	6.02	7.36	6.69	6.47	1.57	6.69	0.67	1.04	0.050	8.3E-01
Mtr.36530.1.S1_at	2.27	4.97	2.25	2.21	2.25	2.24	3.17	1.56	2.23	0.02	0.71	-0.504	3.6E-01
Mtr.47770.1.S1_at	9.01	5.93	7.93	8.47	8.56	8.57	7.63	1.56	8.53	0.05	1.12	0.162	3.7E-01
Mtr.1999.1.S1_at	2.67	5.33	2.57	2.57	2.56	2.57	3.52	1.56	2.57	0.00	0.73	-0.457	3.5E-01
Mtr.2008.1.S1_s_at	6.83	3.97	6.49	8.08	8.40	8.42	5.76	1.56	8.30	0.19	1.44	0.527	4.9E-02
Mtr.28226.1.S1_at	2.90	5.61	2.92	2.91	3.01	2.87	3.81	1.56	2.93	0.07	0.77	-0.378	3.9E-01
Mtr.21812.1.S1_s_at	10.04	7.12	9.56	9.73	9.26	9.68	8.91	1.56	9.56	0.26	1.07	0.102	5.1E-01
Mtr.5961.1.S1_at	6.51	3.61	6.07	6.50	6.59	6.13	5.39	1.56	6.40	0.24	1.19	0.248	3.3E-01
Mtr.14467.1.S1_s_at	2.84	5.53	2.80	2.88	2.76	2.77	3.72	1.56	2.80	0.06	0.75	-0.410	3.7E-01
Mtr.388.1.S1_at	8.64	5.83	8.41	8.46	8.54	8.44	7.63	1.56	8.48	0.05	1.11	0.153	4.0E-01
Mtr.5397.1.S1_at	10.67	7.68	9.95	10.43	10.78	10.45	9.43	1.56	10.55	0.19	1.12	0.162	2.9E-01
Mtr.42137.1.S1_at	6.22	3.10	4.60	4.59	5.28	4.91	4.64	1.56	4.93	0.35	1.06	0.086	7.7E-01
Mtr.21965.1.S1_at	3.45	6.12	3.40	3.40	3.39	3.26	4.32	1.56	3.35	0.08	0.77	-0.369	3.4E-01
Mtr.52019.1.S1_at	3.57	5.97	3.05	3.75	3.06	2.95	4.20	1.56	3.25	0.43	0.78	-0.368	3.7E-01
Mtr.37873.1.S1_at	7.56	7.06	9.97	8.65	9.01	8.63	8.20	1.56	8.76	0.21	1.07	0.096	5.7E-01
Mtr.43392.1.S1_s_at	9.87	7.87	10.94	11.04	10.01	10.73	9.56	1.56	10.60	0.53	1.11	0.148	3.4E-01
Mtr.42830.1.S1_at	2.79	3.01	5.59	6.12	5.05	5.37	3.80	1.56	5.51	0.55	1.45	0.538	1.5E-01
Mtr.40379.1.S1_at	7.69	5.27	8.18	6.64	6.28	6.47	7.05	1.56	6.46	0.18	0.92	-0.124	5.5E-01
Mtr.1900.1.S1_at	3.52	6.01	6.38	6.03	6.73	6.06	5.30	1.56	6.27	0.39	1.18	0.243	3.5E-01
Mtr.4904.1.S1_at	6.48	4.42	7.47	9.05	8.54	8.75	6.12	1.55	8.78	0.25	1.43	0.520	4.3E-02
Mtr.25104.1.S1_at	8.20	10.99	8.41	8.78	8.14	8.67	9.20	1.55	8.53	0.34	0.93	-0.109	5.1E-01
Mtr.35680.1.S1_s_at	6.66	3.66	5.86	6.29	6.14	6.31	5.39	1.55	6.25	0.09	1.16	0.213	3.9E-01
Mtr.47092.1.S1_s_at	6.99	4.45	7.27	6.74	6.65	6.46	6.23	1.55	6.62	0.15	1.06	0.086	6.9E-01
Mtr.39530.1.S1_at	7.36	5.01	7.94	7.83	8.36	8.07	6.77	1.55	8.09	0.26	1.19	0.256	2.2E-01
Mtr.44364.1.S1_at	7.97	5.06	7.45	8.32	8.83	8.70	6.83	1.55	8.62	0.27	1.26	0.336	1.2E-01
Mtr.3009.1.S1_at	5.55	2.54	3.41	4.01	4.41	4.01	3.83	1.55	4.14	0.23	1.08	0.111	7.5E-01
Mtr.1323.1.S1_at	8.61	6.42	9.42	9.11	9.17	8.79	8.15	1.55	9.02	0.20	1.11	0.147	3.9E-01
Mtr.45493.1.S1_at	6.49	4.01	6.87	6.86	6.66	6.50	5.79	1.55	6.67	0.18	1.15	0.205	3.8E-01
Mtr.39140.1.S1_at	8.73	5.77	8.07	8.40	8.22	8.31	7.52	1.55	8.31	0.09	1.10	0.144	4.3E-01
Mtr.9750.1.S1_at	8.13	5.26	5.67	6.27	6.55	5.98	6.35	1.55	6.27	0.29	0.99	-0.019	9.3E-01
Mtr.26068.1.S1_at	8.06	5.01	7.03	6.36	7.25	6.36	6.70	1.55	6.66	0.52	0.99	-0.009	9.7E-01
Mtr.11689.1.S1_at	9.21	9.93	6.96	5.95	6.24	6.07	8.70	1.55	6.09	0.15	0.70	-0.516	4.4E-02
Mtr.6835.1.S1_at	6.11	3.13	3.88	2.38	2.17	3.12	4.37	1.55	2.56	0.50	0.58	-0.774	1.3E-01
Mtr.13885.1.S1_at	8.62	5.97	8.68	8.53	8.49	8.34	7.76	1.55	8.45	0.10	1.09	0.124	4.8E-01
Mtr.3165.1.S1_at	7.08	4.19	6.59	6.57	6.67	6.46	5.95	1.55	6.57	0.11	1.10	0.142	5.3E-01
Mtr.39028.1.S1_at	9.58	6.63	8.94	9.31	9.74	9.38	8.38	1.55	9.48	0.23	1.13	0.177	2.9E-01
Mtr.46050.1.S1_at	8.58	5.78	8.33	7.92	7.56	7.81	7.57	1.55	7.76	0.19	1.03	0.037	8.4E-01
Mtr.13241.1.S1_at	2.12	2.17	4.82	4.86	4.81	5.37	3.03	1.55	5.01	0.31	1.65	0.724	9.6E-02
Mtr.9920.1.S1_at	8.02	5.19	7.69	7.95	7.84	7.99	6.97	1.55	7.93	0.07	1.14	0.186	3.4E-01
Mtr.13610.1.S1_at	6.50	4.01	6.85	7.16	7.13	6.98	5.79	1.55	7.09	0.10	1.22	0.293	2.2E-01
Mtr.7659.1.S1_at	4.28	2.28	5.32	4.27	4.27	4.59	3.96	1.55	4.37	0.19	1.11	0.144	6.7E-01
Mtr.36027.1.S1_s_at	8.02	5.33	8.00	7.41	7.32	7.31	7.12	1.55	7.35	0.05	1.03	0.046	8.1E-01
Mtr.33563.1.S1_at	7.19	4.20	6.37	6.67	6.45	6.80	5.92	1.54	6.64	0.18	1.12	0.166	4.7E-01
Mtr.17244.1.S1_at	8.30	5.24	6.37	7.37	8.11	8.32	6.64	1.54	7.94	0.50	1.20	0.257	2.4E-01
Mtr.2871.1.S1_at	5.74	2.66	4.02	2.56	2.63	2.60	4.14	1.54	2.60	0.04	0.63	-0.672	1.6E-01
Mtr.7469.1.S1_at	5.64	2.84	5.37	5.97	6.84	6.25	4.61	1.54	6.35	0.45	1.38	0.462	1.3E-01
Mtr.39677.1.S1_x_at	6.69	3.64	4.76	3.76	3.64	3.92	5.03	1.54	3.78	0.14	0.75	-0.413	2.3E-01
Mtr.28624.1.S1_at	6.34	3.62	6.25	6.69	6.24	6.27	5.40	1.54	6.40	0.25	1.18	0.244	3.3E-01
Mtr.12064.1.S1_at	5.70	3.50	6.48	5.72	5.81	5.61	5.23	1.54	5.71	0.10	1.09	0.129	6.1E-01
Mtr.40297.1.S1_at	7.68	5.69	8.72	7.54	7.77	7.19	7.36	1.54	7.50	0.29	1.02	0.026	8.9E-01
Mtr.22900.1.S1_x_at	3.07	5.63	2.85	2.78	3.30	3.09	3.85	1.54	3.06	0.26	0.79	-0.332	4.3E-01
Mtr.27518.1.S1_at	6.48	3.90	6.66	7.10	7.15	6.85	5.68	1.54	7.03	0.16	1.24	0.308	2.1E-01
Mtr.4046.1.S1_at	5.34	2.71	5.42	5.47	5.49	5.24	4.49	1.54	5.40	0.14	1.20	0.265	3.7E-01
Mtr.50080.1.S1_s_at	5.20	2.31	4.70	5.61	6.67	5.83	4.07	1.54	6.04	0.56	1.48	0.569	1.1E-01
Mtr.51203.1.S1_at	7.87	5.14	7.74	7.37	7.40	7.29	6.91	1.54	7.35	0.06	1.06	0.089	6.5E-01
Mtr.18690.1.S1_at	6.09	3.31	5.86	6.31	6.15	5.79	5.09	1.54	6.08	0.27	1.20	0.258	3.3E-01
Mtr.10183.1.S1_s_at	6.79	4.11	6.76	6.77	6.94	7.07	5.89	1.54	6.93	0.15	1.18	0.234	3.1E-01
Mtr.17162.1.S1_at	2.14	4.80	2.13	2.13	2.13	2.13	3.03	1.54	2.13	0.00	0.70	-0.508	3.7E-01
Mtr.51465.1.S1_at	6.29	4.54	7.61	6.96	6.77	6.74	6.14	1.54	6.82	0.12	1.11	0.151	4.9E-01
Mtr.34340.1.S1_at	2.50	5.07	2.32	2.32	2.29	2.31	3.30	1.54	2.31	0.01	0.70	-0.516	3.3E-01
Mtr.2985.1.S1_at	1.98	4.64	1.97	1.97	1.97	1.97	2.86	1.54	1.97	0.00	0.69	-0.539	3.7E-01
Mtr.42630.1.S1_at	4.99	3.34	6.42	5.64	5.90	5.72	4.92	1.54	5.75	0.13	1.17	0.226	4.0E-01
Mtr.24911.1.S1_at	2.28	4.94	2.27	2.26	2.26	2.26	3.16	1.54	2.26	0.00	0.71	-0.485	3.7E-01
Mtr.39568.1.S1_at	6.64	4.91	7.98	7.57	7.59	7.31	6.51	1.54	7.49	0.16	1.15	0.202	3.3E-01
Mtr.29021.1.S1_at	2.15	4.86	2.25	2.13	2.13	2.13	3.09	1.54	2.13	0.00	0.69	-0.534	3.4E-01
Mtr.9957.1.S1_s_at	7.06	4.37	7.01	7.05	7.03	6.85	6.15	1.54	6.98	0.11	1.14	0.183	4.0E-01
Mtr.11813.1.S1_at	9.16	6.19	8.38	8.59	9.01	8.85	7.91	1.54	8.82	0.21	1.11	0.156	3.7E-01
Mtr.21502.1.S1_at	2.47	5.13	2.45	2.44	2.45	2.42	3.35	1.54	2.43	0.02	0.73	-0.461	3.6E-01
Mtr.64.1.S1_s_at	2.66	5.31	2.63	2.62	2.54	2.60	3.53	1.54	2.59	0.04	0.73	-0.448	3.5E-01
Mtr.27725.1.S1_s_at	2.93	5.63	3.01	3.02	3.01	3.00	3.86	1.54	3.01	0.01	0.78	-0.357	3.9E-01
Mtr.42977.1.S1_x_at	8.18	5.63	8.39	8.15	7.84	8.22	7.40	1.54	8.07	0.21	1.09	0.125	4.9E-01
Mtr.10153.1.S1_s_at	6.42	4.09	7.00	6.53	6.78	6.02	5.84	1.54	6.44	0.38	1.10	0.142	5.4E-01
Mtr.39571.1.S1_at	7.11	4.06	5.88	6.33	6.28	6.47	5.68	1.54	6.36	0.10	1.12	0.162	4.9E-01
Mtr.30743.1.S1_at	7.60	5.14	7.97	8.29	8.46	7.93	6.90	1.54	8.23	0.27	1.19	0.253	2.1E-01
Mtr.15989.1.S1_at	7.01	4.58	7.43	6.85	7.03	7.20	6.34	1.54	7.03	0.17	1.11	0.148	4.9E-01
Mtr.39524.1.S1_at	10.17	7.97	7.21	9.81	8.78	10.11	8.45	1.54	9.57	0.70	1.13	0.179	3.2E-01
Mtr.38869.1.S1_at	5.91	3.60	6.51	6.14	5.38	5.60	5.34	1.54	5.71	0.39	1.07	0.096	7.1E-01
Mtr.35000.1.S1_at	6.30	3.57	6.16	6.58	6.41	6.47	5.34	1.54	6.49	0.08	1.21	0.280	2.7E-01
Mtr.36841.1.S1_at	6.20	3.14	4.80	5.70	5.94	5.20	4.71	1.54	5.61	0.38	1.19	0.252	3.8E-01
Mtr.48996.1.S1_at	6.84	4.11	4.25	4.21	5.15	4.62	5.07	1.54	4.66	0.47	0.92	-0.121	6.8E-01
Mtr.49054.1.S1_at	8.53	11.13	8.41	8.49	7.86	8.46	9.36	1.53	8.27	0.36	0.88	-0.179	3.0E-01
Mtr.29307.1.S1_at	5.61	2.66	4.87	5.55	5.27	5.66	4.38	1.53	5.49	0.20	1.25	0.327	2.8E-01
Mtr.50053.1.S1_at	10.16	7.52	10.20	10.17	10.30	10.09	9.30	1.53	10.19	0.11	1.10	0.132	3.7E-01
Mtr.2566.1.S1_at	3.29	5.99	3.37	3.25	3.06	3.36	4.21	1.53	3.22	0.15	0.76	-0.387	3.3E-01
Mtr.35822.1.S1_at	4.88	2.30	5.02	5.03	5.18	5.38	4.07	1.53	5.19	0.18	1.28	0.352	2.8E-01
Mtr.12641.1.S1_at	7.90	5.51	8.36	7.75	7.50	7.64	7.26	1.53	7.63	0.13	1.05	0.072	7.0E-01
Mtr.4956.1.S1_at	2.17	4.82	2.16	2.15	2.15	2.52	3.05	1.53	2.27	0.21	0.75	-0.422	4.4E-01
Mtr.51194.1.S1_s_at	7.27	4.26	5.28	5.97	6.59	6.21	5.60	1.53	6.26	0.31	1.12	0.159	5.1E-01
Mtr.50551.1.S1_at	2.65	5.29	2.62	2.60	2.58	2.58	3.52	1.53	2.59	0.01	0.73	-0.445	3.5E-01
Mtr.11479.1.S1_at	8.41	5.48	7.72	7.97	7.84	8.04	7.21	1.53	7.95	0.10	1.10	0.142	4.5E-01
Mtr.38948.1.S1_at	7.83	4.94	7.26	8.14	8.38	8.21	6.68	1.53	8.24	0.12	1.23	0.304	1.5E-01
Mtr.45430.1.S1_at	6.28	3.44	5.84	6.26	6.28	6.27	5.19	1.53	6.27	0.01	1.21	0.273	2.9E-01
Mtr.23326.1.S1_at	4.66	6.95	4.04	4.05	4.23	4.24	5.22	1.53	4.17	0.11	0.80	-0.322	3.0E-01
Mtr.43496.1.S1_at	6.59	3.77	6.21	6.71	6.55	6.82	5.53	1.53	6.69	0.14	1.21	0.276	2.6E-01
Mtr.40804.1.S1_at	8.29	5.86	8.69	8.49	9.20	8.32	7.62	1.53	8.67	0.46	1.14	0.187	3.2E-01
Mtr.45497.1.S1_s_at	7.79	4.80	6.88	7.45	7.50	8.03	6.49	1.53	7.66	0.32	1.18	0.239	2.6E-01
Mtr.51726.1.S1_x_at	3.73	6.36	3.71	4.20	3.70	3.72	4.60	1.53	3.87	0.28	0.84	-0.248	4.6E-01
Mtr.10649.1.S1_at	8.01	5.68	8.56	9.21	8.10	9.09	7.42	1.53	8.80	0.61	1.19	0.246	2.2E-01
Mtr.6128.1.S1_at	6.61	3.90	6.49	6.27	6.55	6.30	5.67	1.53	6.37	0.15	1.12	0.169	4.7E-01
Mtr.6648.1.S1_s_at	8.42	6.09	8.98	9.11	9.29	9.35	7.83	1.53	9.25	0.12	1.18	0.241	1.8E-01
Mtr.33542.1.S1_at	6.74	3.87	6.22	6.25	6.65	6.48	5.61	1.53	6.46	0.20	1.15	0.203	4.0E-01

Mtr.28734.1.S1_at	5.71	3.37	6.24	5.57	5.76	5.82	5.10	1.53	5.72	0.13	1.12	0.164	5.3E-01
Mtr.25104.1.S1_x_at	5.94	8.66	6.09	6.32	5.94	6.21	6.90	1.53	6.16	0.19	0.89	-0.164	4.5E-01
Mtr.25764.1.S1_x_at	2.58	5.22	2.57	2.55	2.54	2.53	3.45	1.53	2.54	0.01	0.74	-0.442	3.6E-01
Mtr.42728.1.S1_at	6.75	4.27	7.05	6.82	7.02	6.94	6.02	1.53	6.93	0.10	1.15	0.202	3.6E-01
Mtr.26358.1.S1_at	6.16	4.85	7.89	6.34	5.69	6.06	6.30	1.53	6.03	0.33	0.96	-0.064	7.8E-01
Mtr.44503.1.S1_at	8.25	5.45	7.89	7.78	8.00	7.89	7.20	1.53	7.89	0.11	1.10	0.133	4.8E-01
Mtr.47257.1.S1_s_at	12.16	9.16	10.16	9.87	10.96	10.53	10.49	1.53	10.45	0.55	1.00	-0.006	9.7E-01
Mtr.12486.1.S1_at	9.76	7.52	10.44	8.26	6.44	7.89	9.24	1.53	7.53	0.96	0.82	-0.295	1.8E-01
Mtr.35442.1.S1_at	7.84	5.16	5.23	6.33	6.14	6.13	6.08	1.53	6.20	0.11	1.02	0.029	9.0E-01
Mtr.5439.1.S1_at	4.91	3.00	6.01	4.66	4.72	4.64	4.64	1.52	4.67	0.04	1.01	0.011	9.7E-01
Mtr.19111.1.S1_s_at	5.09	3.31	6.33	5.23	5.54	4.55	4.91	1.52	5.11	0.50	1.04	0.057	8.4E-01
Mtr.38587.1.S1_at	6.53	3.87	6.48	6.48	6.69	6.47	5.63	1.52	6.55	0.12	1.16	0.219	3.6E-01
Mtr.50692.1.S1_at	6.28	3.82	6.61	7.09	6.47	6.44	5.57	1.52	6.67	0.37	1.20	0.259	2.9E-01
Mtr.4118.1.S1_at	2.40	5.06	2.45	2.66	2.32	2.45	3.31	1.52	2.48	0.17	0.75	-0.416	4.0E-01
Mtr.24049.1.S1_at	2.67	5.30	2.66	2.66	2.64	3.25	3.54	1.52	2.85	0.35	0.80	-0.314	4.8E-01
Mtr.27801.1.S1_at	6.42	4.00	6.81	6.82	6.67	6.63	5.74	1.52	6.71	0.10	1.17	0.224	3.3E-01
Mtr.5811.1.S1_at	6.13	3.68	6.47	6.40	6.54	6.58	5.43	1.52	6.51	0.09	1.20	0.262	2.9E-01
Mtr.48718.1.S1_at	7.55	4.77	7.22	7.90	7.93	7.68	6.51	1.52	7.84	0.14	1.20	0.267	2.1E-01
Mtr.18646.1.S1_at	9.25	6.57	9.14	9.69	10.17	9.83	8.32	1.52	9.90	0.25	1.19	0.250	1.5E-01
Mtr.49771.1.S1_at	6.96	4.31	6.92	7.38	7.25	7.28	6.06	1.52	7.30	0.07	1.20	0.268	2.3E-01
Mtr.34483.1.S1_at	2.97	5.58	2.91	2.89	2.82	2.82	3.82	1.52	2.84	0.04	0.74	-0.426	3.3E-01
Mtr.36248.1.S1_at	2.14	4.76	2.12	2.12	2.12	2.11	3.01	1.52	2.12	0.00	0.70	-0.507	3.7E-01
Mtr.2925.1.S1_at	5.63	2.99	5.62	5.43	5.50	4.96	4.75	1.52	5.30	0.29	1.12	0.158	5.7E-01
Mtr.36806.1.S1_at	6.88	5.25	8.28	7.09	7.66	7.22	6.80	1.52	7.32	0.30	1.08	0.106	5.9E-01
Mtr.2336.1.S1_at	2.33	4.94	2.29	2.29	2.29	2.29	3.19	1.52	2.29	0.00	0.72	-0.476	3.6E-01
Mtr.5757.1.S1_at	6.21	3.23	4.25	4.73	4.69	5.20	4.56	1.52	4.87	0.28	1.07	0.094	7.5E-01
Mtr.42076.1.S1_at	5.09	2.46	5.09	5.29	5.75	5.09	4.21	1.52	5.37	0.34	1.28	0.352	2.6E-01
Mtr.41885.1.S1_at	8.12	5.49	8.12	8.37	8.46	8.24	7.24	1.52	8.36	0.11	1.15	0.207	2.7E-01
Mtr.4996.1.S1_at	6.38	3.35	5.09	5.72	5.34	5.38	4.94	1.52	5.48	0.21	1.11	0.149	5.8E-01
Mtr.33379.1.S1_s_at	7.03	4.55	7.31	6.73	7.01	6.31	6.30	1.52	6.68	0.35	1.06	0.086	6.9E-01
Mtr.47315.1.S1_at	2.67	5.30	2.68	2.89	2.48	2.68	3.55	1.52	2.68	0.20	0.75	-0.407	3.8E-01
Mtr.51256.1.S1_at	5.53	2.50	3.97	4.89	6.24	5.78	4.00	1.52	5.64	0.68	1.41	0.496	1.6E-01
Mtr.13031.1.S1_at	7.70	4.67	6.33	6.67	6.40	6.97	6.23	1.52	6.68	0.28	1.07	0.100	6.4E-01
Mtr.41403.1.S1_at	2.50	2.25	4.99	5.57	6.11	5.67	3.25	1.52	5.79	0.29	1.78	0.834	4.6E-02
Mtr.30038.1.S1_at	5.85	3.75	6.70	6.04	6.25	5.49	5.43	1.51	5.93	0.39	1.09	0.126	6.1E-01
Mtr.48505.1.S1_at	1.72	4.35	1.73	1.71	1.71	1.72	2.60	1.51	1.71	0.00	0.66	-0.603	3.7E-01
Mtr.27111.1.S1_at	6.53	3.55	5.50	5.49	5.52	6.21	5.19	1.51	5.74	0.41	1.11	0.145	5.8E-01
Mtr.17827.1.S1_at	2.70	5.32	2.69	2.69	2.58	2.67	3.57	1.51	2.65	0.06	0.74	-0.431	3.5E-01
Mtr.45032.1.S1_at	5.88	3.31	5.98	5.84	5.63	5.62	5.06	1.51	5.70	0.12	1.13	0.172	5.1E-01
Mtr.35335.1.S1_s_at	11.49	8.57	9.34	10.05	9.94	10.34	9.80	1.51	10.11	0.20	1.03	0.045	7.4E-01
Mtr.31672.1.S1_s_at	8.44	5.82	8.44	8.44	8.26	8.06	7.56	1.51	8.25	0.19	1.09	0.125	4.8E-01
Mtr.18492.1.S1_s_at	6.14	9.16	7.81	8.51	9.08	8.49	7.70	1.51	8.69	0.34	1.13	0.174	3.3E-01
Mtr.9497.1.S1_at	6.00	3.38	5.99	6.30	6.40	6.08	5.12	1.51	6.26	0.16	1.22	0.289	2.6E-01
Mtr.28034.1.S1_at	7.77	5.24	7.94	8.17	8.13	7.73	6.98	1.51	8.01	0.24	1.15	0.198	3.1E-01
Mtr.33632.1.S1_s_at	5.59	3.50	6.43	5.71	4.60	4.76	5.17	1.51	5.02	0.60	0.97	-0.043	8.8E-01
Mtr.42098.1.S1_at	7.75	5.41	8.23	7.77	7.57	7.64	7.13	1.51	7.66	0.10	1.07	0.103	5.8E-01
Mtr.12900.1.S1_at	6.48	4.31	7.21	7.26	7.46	7.31	6.00	1.51	7.34	0.10	1.22	0.292	2.0E-01
Mtr.25995.1.S1_at	7.42	4.78	7.36	7.33	8.06	7.41	6.52	1.51	7.60	0.40	1.17	0.221	3.0E-01
Mtr.4752.1.S1_at	7.78	5.01	7.42	7.64	7.86	7.50	6.73	1.51	7.67	0.18	1.14	0.187	3.5E-01
Mtr.25797.1.S1_at	3.12	5.72	3.11	3.10	3.04	3.10	3.98	1.51	3.08	0.04	0.77	-0.371	3.6E-01
Mtr.14586.1.S1_s_at	6.37	4.49	7.47	6.97	8.05	6.59	6.11	1.51	7.20	0.76	1.18	0.238	3.2E-01
Mtr.17973.1.S1_at	3.99	6.60	3.98	4.21	3.98	4.02	4.86	1.51	4.07	0.12	0.84	-0.255	4.2E-01
Mtr.37632.1.S1_at	7.41	5.34	8.28	7.11	6.86	6.41	7.01	1.51	6.80	0.36	0.97	-0.045	8.2E-01
Mtr.39011.1.S1_at	7.05	4.95	7.87	6.18	5.64	6.11	6.63	1.51	5.98	0.29	0.90	-0.148	5.1E-01
Mtr.26949.1.S1_at	7.75	4.94	7.29	7.40	7.96	7.53	6.66	1.51	7.63	0.30	1.15	0.196	3.4E-01
Mtr.4831.1.S1_at	2.95	5.53	2.90	2.90	2.82	2.89	3.79	1.51	2.87	0.05	0.76	-0.403	3.5E-01
Mtr.24451.1.S1_at	8.04	5.52	8.21	8.78	8.65	8.71	7.26	1.51	8.72	0.06	1.20	0.264	1.7E-01
Mtr.43917.1.S1_at	9.00	6.06	8.11	8.10	7.91	8.14	7.72	1.51	8.05	0.12	1.04	0.059	7.3E-01
Mtr.5948.1.S1_at	6.42	3.87	6.53	6.51	7.11	6.84	5.61	1.51	6.82	0.30	1.22	0.282	2.4E-01
Mtr.2921.1.S1_at	8.16	5.51	8.08	8.09	8.23	8.45	7.25	1.51	8.26	0.18	1.14	0.187	3.2E-01
Mtr.51759.1.S1_at	6.91	4.50	7.27	7.14	6.65	7.03	6.22	1.50	6.94	0.26	1.11	0.157	4.6E-01
Mtr.49545.1.S1_at	6.02	8.53	5.84	6.04	5.68	6.08	6.80	1.50	5.93	0.22	0.87	-0.196	3.8E-01
Mtr.26197.1.S1_at	4.79	3.35	6.36	5.55	6.17	5.60	4.83	1.50	5.77	0.35	1.19	0.257	3.5E-01
Mtr.23635.1.S1_at	3.32	5.91	3.31	3.31	3.26	3.31	4.18	1.50	3.29	0.03	0.79	-0.344	3.6E-01
Mtr.34795.1.S1_at	6.76	4.15	6.74	7.05	6.57	6.90	5.89	1.50	6.84	0.24	1.16	0.216	3.4E-01
Mtr.43392.1.S1_at	10.15	8.23	11.18	11.13	10.24	10.94	9.85	1.50	10.77	0.47	1.09	0.129	3.7E-01
Mtr.9888.1.S1_s_at	9.65	6.65	8.03	8.71	8.67	8.99	8.11	1.50	8.79	0.18	1.08	0.116	4.8E-01
Mtr.49998.1.S1_at	8.34	5.33	6.83	5.99	5.12	5.83	6.83	1.50	5.65	0.46	0.83	-0.275	2.6E-01
Mtr.13999.1.S1_at	6.53	4.25	7.09	6.81	6.89	6.59	5.95	1.50	6.76	0.16	1.14	0.183	4.1E-01
Mtr.14152.1.S1_at	7.96	5.37	7.98	7.91	7.74	7.75	7.10	1.50	7.80	0.10	1.10	0.135	4.7E-01
Mtr.31028.1.S1_at	2.91	2.43	5.23	3.49	3.37	3.40	3.52	1.50	3.42	0.06	0.97	-0.043	9.1E-01
Mtr.42049.1.S1_at	9.03	6.76	9.58	8.77	8.58	8.93	8.46	1.50	8.76	0.18	1.04	0.050	7.5E-01
Mtr.41442.1.S1_at	7.73	5.05	7.55	8.17	8.21	8.34	6.77	1.50	8.24	0.09	1.22	0.283	1.7E-01
Mtr.24417.1.S1_at	2.75	5.33	2.71	2.70	2.66	2.69	3.60	1.50	2.68	0.02	0.75	-0.422	3.5E-01
Mtr.42553.1.S1_s_at	7.79	5.79	4.86	5.74	6.33	5.91	6.14	1.50	5.99	0.30	0.98	-0.036	8.7E-01
Mtr.5872.1.S1_at	4.98	2.82	5.70	5.02	5.52	4.94	4.50	1.50	5.16	0.31	1.15	0.198	5.0E-01
Mtr.33555.1.S1_s_at	6.73	4.30	7.02	6.74	6.36	6.81	6.02	1.50	6.64	0.24	1.10	0.141	5.2E-01
Mtr.10882.1.S1_at	7.65	4.68	6.46	6.57	6.70	6.97	6.26	1.50	6.75	0.20	1.08	0.107	6.1E-01
Mtr.11888.1.S1_at	8.51	6.02	8.70	8.35	8.52	8.38	7.74	1.50	8.42	0.09	1.09	0.121	4.8E-01
Mtr.32166.1.S1_at	7.47	4.89	7.48	7.64	7.95	7.46	6.61	1.50	7.68	0.24	1.16	0.217	2.9E-01
Mtr.10545.1.S1_at	6.12	3.54	6.14	5.85	5.84	5.57	5.27	1.49	5.75	0.16	1.09	0.127	6.1E-01
Mtr.44714.1.S1_at	6.99	5.57	8.56	7.39	7.54	7.56	7.04	1.49	7.50	0.09	1.07	0.091	6.2E-01
Mtr.27431.1.S1_at	6.49	3.59	5.67	5.49	5.41	5.02	5.25	1.49	5.30	0.25	1.01	0.014	9.6E-01
Mtr.40371.1.S1_at	7.63	5.08	7.70	8.07	8.02	8.41	6.80	1.49	8.17	0.21	1.20	0.263	1.9E-01
Mtr.29186.1.S1_at	1.94	4.53	1.93	1.93	1.93	1.93	2.80	1.49	1.93	0.00	0.69	-0.538	3.7E-01
Mtr.34274.1.S1_at	2.51	5.00	2.33	2.50	2.47	2.71	3.28	1.49	2.56	0.13	0.78	-0.358	4.5E-01
Mtr.26295.1.S1_at	5.52	3.73	6.69	5.72	5.94	5.77	5.31	1.49	5.81	0.12	1.09	0.129	5.9E-01
Mtr.11583.1.S1_at	9.26	8.56	6.40	8.45	7.78	8.69	8.07	1.49	8.31	0.47	1.03	0.041	8.1E-01
Mtr.11798.1.S1_at	9.99	7.72	10.53	10.38	10.20	10.16	9.41	1.49	10.24	0.12	1.09	0.122	3.9E-01
Mtr.33424.1.S1_s_at	7.93	5.02	7.03	6.99	7.51	7.21	6.66	1.49	7.23	0.26	1.09	0.120	5.5E-01
Mtr.45508.1.S1_s_at	6.60	3.98	6.52	6.59	6.71	6.63	5.70	1.49	6.64	0.06	1.17	0.222	3.3E-01
Mtr.24786.1.S1_s_at	5.74	3.61	6.49	3.83	5.35	5.36	5.28	1.49	4.85	0.88	0.92	-0.123	6.9E-01
Mtr.32237.1.S1_at	8.27	6.15	9.02	8.62	8.58	8.70	7.81	1.49	8.63	0.06	1.11	0.144	3.9E-01
Mtr.8717.1.S1_at	5.90	3.43	6.11	5.61	5.46	5.58	5.15	1.49	5.55	0.08	1.08	0.109	6.6E-01
Mtr.4664.1.S1_at	6.67	4.23	6.94	6.52	6.52	6.21	5.95	1.49	6.42	0.18	1.08	0.110	6.2E-01
Mtr.16278.1.S1_at	10.76	7.93	10.14	10.34	11.34	11.14	9.61	1.49	10.94	0.53	1.14	0.187	2.2E-01
Mtr.50217.1.S1_s_at	6.99	4.23	6.58	6.61	6.58	6.56	5.93	1.49	6.58	0.02	1.11	0.151	4.9E-01
Mtr.24870.1.S1_at	6.60	4.00	6.56	7.04	6.98	6.74	5.72	1.49	6.92	0.16	1.21	0.276	2.4E-01
Mtr.48096.1.S1_at	2.54	5.11	2.53	2.52	2.52	2.51	3.39	1.49	2.52	0.00	0.74	-0.429	3.7E-01
Mtr.26827.1.S1_at	6.77	3.80	5.52	5.97	6.12	5.96	5.36	1.49	6.02	0.09	1.12	0.166	4.9E-01
Mtr.10724.1.S1_s_at	7.39	4.71	7.19	6.98	7.31	7.03	6.43	1.49	7.11	0.17	1.11	0.145	4.8E-01
Mtr.19050.1.S1_at	5.79	8.35	5.76	6.24	6.26	5.75	6.64	1.49	6.08	0.29	0.92	-0.125	5.6E-01
Mtr.1956.1.S1_at	5.02	2.27	4.63	4.83	4.82	5.72	3.97	1.49	5.12	0.52	1.29	0.368	2.7E-01
Mtr.42960.1.S1_at	2.29	4.87	2.29	2.28	2.28	2.28	3.15	1.49	2.28	0.00	0.73	-0.463	3.7E-01
Mtr.1811.1.S1_at	7.64	5.17	7.83	7.75	8.01	7.69	6.88	1.49	7.81	0.17	1.14	0.183	3.4E-01
Mtr.27937.1.S1_at	6.07	4.32	7.28	6.54	8.11	7.31	5.89	1.49	7.32	0.79	1.24	0.312	2.2E-01
Mtr.5561.1.S1_at	5.14	2.57	5.16	5.40	5.39	5.02	4.29	1.49	5.27	0.22	1.23	0.298	3.2E-01
Mtr.36949.1.S1_a_at	7.49	10.06	7.48	7.48	7.35	7.53	8.34	1.49	7.45	0.09	0.89	-0.163	3.6E-01
Mtr.31050.1.S1_s_at	8.76	6.23	8.83	8.87	8.45	8.73	7.94	1.49	8.68	0.21	1.09	0.129	4.4E-01
Mtr.48270.1.S1_at	2.68	5.26	2.69	2.67	2.67	2.67	3.54	1.49	2.67	0.00	0.75	-0.408	3.7E-01
Mtr.38735.1.S1_s_at	6.95	4.09	6.21	6.83	6.86	6.88	5.75	1.49	6.86	0.03	1.19	0.254	2.7E-01
Mtr.26044.1.S1_s_at	9.58	7.22	6.83	9.09	8.13	9.44	7.88	1.49	8.89	0.68	1.13	0.174	3.4E-01
Mtr.25237.1.S1_at	3.68	5.73	2.85	2.43	2.47	2.82	4.09	1.48	2.57	0.21	0.63	-0.667	1.6E-01
Mtr.44480.1.S1_at	6.55	4.01	6.62	6.37	6.51	6.40	5.73	1.48	6.43	0.07	1.12	0.167	4.6E-01
Mtr.34957.1.S1_at	9.30	6.76	9.34	9.35	9.06	9.21	8.47	1.48	9.20	0.14	1.09	0.121	4.4E-01
Mtr.13509.1.S1_at	8.36	5.58	7.85	8.45	8.32	8.59	7.26	1.48	8.46	0.13	1.16	0.220	2.4E-01
Mtr.7039.1.S1_at	7.62	5.18	7.86	7.23	7.49	7.38	6.89	1.48	7.37	0.13	1.07	0.097	6.1E-01
Mtr.50116.1.S1_s_at	3.30	5.76	3.09	3.25	3.25	3.85	4.05	1.48	3.45	0.35	0.85	-0.233	5.3E-01
Mtr.12164.1.S1_at	7.53	4.57	5.90	6.65	6.08	6.77	6.00	1.48	6.50	0.37	1.08	0.115	6.0E-01
Mtr.43311.1.S1_at	8.96	6.03	7.88	8.96	9.30	9.12	7.63	1.48	9.13	0.17	1.20	0.259	1.6E-01
Mtr.26605.1.S1_at	4.67	2.17	4.80	4.69	4.97	4.63	3.88	1.48	4.76	0.18	1.23	0.297	3.6E-01
Mtr.5764.1.S1_at	4.99	2.41	4.97	4.95	5.36	4.98	4.12	1.48	5.10	0.23	1.24	0.306	3.2E-01
Mtr.39138.1.S1_at	6.61	4.66	7.56	6.76	6.91	6.99	6.28	1.48	6.89	0.11	1.10	0.134	5.2E-01
Mtr.37265.1.S1_at	7.10	4.20	6.16	6.44	7.07	6.75	5.82	1.48	6.75	0.32	1.16	0.215	3.4E-01
Mtr.33467.1.S1_at	6.13	4.34	7.28	7.55	7.65	7.49	5.92	1.48	7.56	0.08	1.28	0.354	1.3E-01
Mtr.44470.1.S1_at	4.13	2.10	4.98	5.42	5.41	5.15	3.74	1.48	5.32	0.15	1.42	0.511	1.4E-01
Mtr.4582.1.S1_at	9.00	8.01	10.92	9.01	9.83	9.71	9.31	1.48	9.52	0.44	1.02	0.032	8.3E-01
Mtr.35815.1.S1_at	2.22	2.16	4.75	4.90	5.35	6.41	3.04	1.48	5.55	0.78	1.82	0.866	6.0E-02
Mtr.28691.1.S1_at	6.95	9.88	8.78	4.85	6.67	5.44	8.54	1.48	5.65	0.93	0.66	-0.595	4.6E-02
Mtr.15510.1.S1_at	7.61	5.02	7.55	7.51	7.45	6.55	6.73	1.48	7.17	0.54	1.07	0.092	6.5E-01
Mtr.31015.1.S1_at	6.97	9.65	9.40	6.63	7.72	5.96	8.67	1.48	6.77	0.89	0.78	-0.357	1.3E-01

Mtr.44072.1.S1_at	5.26	2.36	4.31	4.96	5.65	5.32	3.98	1.48	5.31	0.34	1.34	0.417	2.0E-01
Mtr.13696.1.S1_at	7.27	4.67	7.19	7.18	7.36	7.23	6.38	1.48	7.26	0.09	1.14	0.187	3.6E-01
Mtr.35905.1.S1_at	2.63	5.18	2.61	2.58	2.61	2.55	3.47	1.48	2.58	0.03	0.74	-0.427	3.6E-01
Mtr.9139.1.S1_at	8.12	5.46	7.90	8.03	8.05	7.79	7.16	1.48	7.96	0.14	1.11	0.152	4.1E-01
Mtr.1662.1.S1_at	9.80	6.96	9.10	9.43	9.52	9.54	8.62	1.48	9.49	0.06	1.10	0.140	3.6E-01
Mtr.18769.1.S1_at	13.05	10.85	10.24	10.97	11.05	11.16	11.38	1.48	11.06	0.09	0.97	-0.041	7.3E-01
Mtr.5007.1.S1_at	2.82	5.38	2.81	2.79	2.81	3.03	3.67	1.48	2.88	0.13	0.78	-0.352	4.1E-01
Mtr.27377.1.S1_at	6.73	4.41	7.15	6.87	6.93	6.89	6.10	1.48	6.90	0.03	1.13	0.178	4.0E-01
Mtr.44122.1.S1_at	6.16	3.76	6.45	6.03	6.43	6.32	5.46	1.48	6.26	0.21	1.15	0.198	4.0E-01
Mtr.19209.1.S1_at	7.12	4.31	6.50	7.23	8.01	7.79	5.98	1.48	7.68	0.40	1.28	0.361	1.3E-01
Mtr.15276.1.S1_at	4.39	2.05	4.78	4.59	4.65	4.58	3.74	1.48	4.61	0.04	1.23	0.300	3.7E-01
Mtr.29365.1.S1_at	6.40	3.45	4.80	4.62	3.54	4.72	4.88	1.48	4.29	0.66	0.88	-0.186	5.6E-01
Mtr.33353.1.S1_at	7.82	5.04	7.30	7.52	6.83	7.19	6.72	1.48	7.18	0.34	1.07	0.095	6.3E-01
Mtr.13776.1.S1_s_at	5.51	3.24	6.00	5.30	5.44	5.41	4.92	1.48	5.38	0.08	1.09	0.131	6.1E-01
Mtr.28475.1.S1_at	4.72	2.71	5.59	6.74	7.29	7.08	4.34	1.47	7.04	0.28	1.62	0.697	3.6E-02
Mtr.20013.1.S1_at	2.23	4.78	2.22	2.21	2.21	2.21	3.07	1.47	2.21	0.00	0.72	-0.475	3.7E-01
Mtr.50592.1.S1_at	3.12	5.72	3.22	3.19	3.08	3.12	4.02	1.47	3.13	0.05	0.78	-0.360	3.6E-01
Mtr.41293.1.S1_at	6.62	3.88	6.18	6.19	6.24	6.17	5.56	1.47	6.20	0.03	1.12	0.157	4.9E-01
Mtr.6625.1.S1_at	2.26	4.81	2.26	2.24	2.24	2.25	3.11	1.47	2.24	0.00	0.72	-0.470	3.7E-01
Mtr.29298.1.S1_at	10.03	7.09	8.72	9.15	9.17	8.97	8.61	1.47	9.10	0.11	1.06	0.079	6.0E-01
Mtr.19257.1.S1_at	11.29	8.86	11.51	11.69	11.15	11.25	10.55	1.47	11.37	0.29	1.08	0.107	4.0E-01
Mtr.37761.1.S1_s_at	13.52	10.71	11.35	12.09	11.04	12.06	11.86	1.47	11.73	0.60	0.99	-0.016	9.0E-01
Mtr.44890.1.S1_at	6.55	4.31	7.09	7.07	7.96	7.09	5.98	1.47	7.37	0.51	1.23	0.302	2.0E-01
Mtr.51126.1.S1_at	2.52	5.06	2.50	2.49	2.48	2.47	3.36	1.47	2.48	0.01	0.74	-0.439	3.6E-01
Mtr.5845.1.S1_at	5.68	3.57	6.39	5.80	5.45	5.49	5.21	1.47	5.58	0.19	1.07	0.099	6.9E-01
Mtr.43763.1.S1_at	9.13	6.60	9.16	9.60	9.20	9.67	8.30	1.47	9.49	0.25	1.14	0.194	2.4E-01
Mtr.23045.1.S1_at	2.57	5.10	2.55	2.58	2.54	2.51	3.41	1.47	2.55	0.04	0.75	-0.419	3.7E-01
Mtr.6007.1.S1_at	5.31	3.01	5.75	5.76	5.29	5.65	4.69	1.47	5.57	0.24	1.19	0.248	3.6E-01
Mtr.49159.1.S1_x_at	3.14	5.72	3.22	3.18	3.18	3.66	4.03	1.47	3.34	0.28	0.83	-0.271	4.7E-01
Mtr.37058.1.S1_at	5.91	3.09	3.80	3.11	3.30	3.29	4.27	1.47	3.23	0.11	0.76	-0.400	2.9E-01
Mtr.41938.1.S1_at	9.66	7.17	9.76	10.62	10.88	10.77	8.86	1.47	10.76	0.13	1.21	0.279	9.0E-02
Mtr.20905.1.S1_x_at	2.56	5.10	2.55	2.55	2.55	2.54	3.40	1.47	2.55	0.00	0.75	-0.419	3.7E-01
Mtr.41157.1.S1_at	9.92	7.48	10.12	10.29	9.98	9.98	9.17	1.47	10.08	0.18	1.10	0.136	3.5E-01
Mtr.13733.1.S1_at	9.85	7.16	9.50	9.37	9.02	9.42	8.84	1.47	9.27	0.22	1.05	0.069	6.4E-01
Mtr.13944.1.S1_at	5.91	3.14	5.38	4.97	4.95	5.14	4.81	1.47	5.02	0.11	1.04	0.061	8.2E-01
Mtr.50444.1.S1_at	3.03	5.53	2.96	2.93	2.87	2.92	3.84	1.47	2.91	0.03	0.76	-0.402	3.3E-01
Mtr.13693.1.S1_at	6.40	3.68	5.99	5.82	6.29	5.83	5.36	1.47	5.98	0.27	1.12	0.158	5.1E-01
Mtr.44715.1.S1_at	6.14	3.77	6.45	5.95	5.98	5.79	5.45	1.47	5.91	0.10	1.08	0.116	6.2E-01
Mtr.51652.1.S1_at	5.85	2.96	4.87	4.82	3.85	4.41	4.56	1.47	4.36	0.49	0.96	-0.065	8.3E-01
Mtr.22936.1.S1_at	2.33	4.86	2.31	2.33	2.30	2.30	3.17	1.47	2.31	0.01	0.73	-0.456	3.7E-01
Mtr.1916.1.S1_at	5.22	2.57	4.97	4.80	4.70	4.92	4.25	1.46	4.81	0.11	1.13	0.176	5.5E-01
Mtr.39997.1.S1_at	7.91	5.25	7.64	7.73	7.77	7.80	6.93	1.46	7.77	0.03	1.12	0.163	3.8E-01
Mtr.50562.1.S1_s_at	8.70	5.98	6.41	8.29	7.51	8.46	7.03	1.46	8.09	0.50	1.15	0.202	3.0E-01
Mtr.25980.1.S1_s_at	5.73	2.90	3.67	3.44	3.21	3.52	4.10	1.46	3.39	0.16	0.83	-0.274	4.5E-01
Mtr.32117.1.S1_at	8.11	5.57	8.09	8.40	8.28	8.24	7.26	1.46	8.31	0.08	1.15	0.196	2.8E-01
Mtr.5908.1.S1_at	6.79	4.07	6.35	6.64	6.73	5.73	5.74	1.46	6.37	0.55	1.11	0.150	5.2E-01
Mtr.39612.1.S1_at	6.58	4.03	6.55	6.50	6.97	6.80	5.72	1.46	6.76	0.24	1.18	0.240	2.9E-01
Mtr.2571.1.S1_at	6.45	4.24	7.01	6.24	6.67	6.49	5.90	1.46	6.47	0.22	1.10	0.133	5.4E-01
Mtr.2778.1.S1_at	2.16	4.68	2.14	2.14	2.13	2.13	3.00	1.46	2.13	0.01	0.71	-0.490	3.6E-01
Mtr.49062.1.S1_at	7.78	5.29	7.86	7.52	7.30	7.62	6.97	1.46	7.48	0.16	1.07	0.101	5.8E-01
Mtr.29849.1.S1_at	6.72	3.88	5.89	6.61	7.14	6.64	5.50	1.46	6.80	0.30	1.24	0.306	2.1E-01
Mtr.12875.1.S1_at	2.74	5.25	2.71	2.68	2.69	2.68	3.57	1.46	2.68	0.01	0.75	-0.410	3.5E-01
Mtr.15982.1.S1_at	7.34	4.91	7.52	7.23	7.03	7.15	6.59	1.46	7.14	0.10	1.08	0.115	5.5E-01
Mtr.4367.1.S1_at	6.97	4.56	7.19	6.95	7.16	7.08	6.24	1.46	7.06	0.11	1.13	0.179	3.8E-01
Mtr.17179.1.S1_at	4.75	7.20	4.60	4.72	4.43	4.56	5.51	1.46	4.57	0.14	0.83	-0.271	3.3E-01
Mtr.4576.1.S1_at	1.88	4.40	1.87	1.87	1.87	1.87	2.72	1.46	1.87	0.00	0.69	-0.539	3.7E-01
Mtr.3225.1.S1_at	7.55	4.82	7.06	7.29	7.13	7.49	6.48	1.46	7.30	0.18	1.13	0.173	3.9E-01
Mtr.42053.1.S1_at	9.05	7.30	10.20	9.52	9.86	9.54	8.85	1.46	9.64	0.20	1.09	0.123	4.1E-01
Mtr.50502.1.S1_at	6.25	3.35	5.02	5.84	6.41	6.81	4.87	1.46	6.35	0.49	1.30	0.383	1.7E-01
Mtr.20179.1.S1_at	5.73	3.06	5.40	4.70	5.34	5.06	4.73	1.46	5.03	0.32	1.06	0.090	7.4E-01
Mtr.22822.1.S1_at	9.97	7.98	10.81	10.69	10.82	10.43	9.59	1.46	10.64	0.20	1.11	0.151	2.8E-01
Mtr.5204.1.S1_at	7.28	4.42	6.35	6.15	6.45	6.58	6.02	1.46	6.40	0.22	1.06	0.088	6.8E-01
Mtr.31498.1.S1_at	4.68	2.11	4.59	4.66	5.44	4.95	3.79	1.46	5.01	0.40	1.32	0.403	2.3E-01
Mtr.23204.1.S1_x_at	2.93	5.40	2.84	3.46	2.81	2.81	3.73	1.45	3.03	0.38	0.81	-0.300	4.7E-01
Mtr.47662.1.S1_at	2.10	4.61	2.08	2.08	2.08	2.08	2.93	1.45	2.08	0.00	0.71	-0.494	3.7E-01
Mtr.15938.1.S1_at	8.86	6.07	8.16	8.74	8.67	8.83	7.70	1.45	8.75	0.08	1.14	0.185	2.8E-01
Mtr.12001.1.S1_at	7.68	5.13	7.62	7.58	7.31	7.49	6.81	1.45	7.46	0.14	1.09	0.131	4.9E-01
Mtr.9230.1.S1_at	7.44	4.86	7.30	7.56	7.17	7.50	6.53	1.45	7.41	0.21	1.13	0.182	3.6E-01
Mtr.5039.1.S1_at	11.14	8.27	10.08	10.49	9.88	10.59	9.83	1.45	10.32	0.38	1.05	0.070	6.0E-01
Mtr.26920.1.S1_at	2.99	3.66	5.78	5.85	6.41	5.83	4.14	1.45	6.03	0.33	1.46	0.541	9.3E-02
Mtr.16223.1.S1_at	9.69	8.50	6.80	8.66	8.79	8.91	8.33	1.45	8.79	0.12	1.05	0.077	6.2E-01
Mtr.38088.1.S1_at	7.60	5.09	7.60	7.79	7.71	7.87	6.76	1.45	7.79	0.08	1.15	0.204	2.9E-01
Mtr.41110.1.S1_at	10.36	7.63	9.85	9.91	9.93	9.79	9.28	1.45	9.88	0.07	1.06	0.090	5.1E-01
Mtr.41106.1.S1_at	6.40	4.27	7.04	6.41	6.35	6.11	5.91	1.45	6.29	0.16	1.07	0.091	6.7E-01
Mtr.7824.1.S1_at	8.93	6.25	8.55	7.73	7.93	7.91	7.91	1.45	7.86	0.11	0.99	-0.010	9.5E-01
Mtr.25925.1.S1_at	2.49	5.00	2.48	2.47	2.47	2.46	3.32	1.45	2.47	0.01	0.74	-0.430	3.6E-01
Mtr.16048.1.S1_at	6.22	4.66	7.56	6.40	6.66	6.62	6.15	1.45	6.56	0.14	1.07	0.094	6.5E-01
Mtr.11230.1.S1_at	8.41	6.08	8.74	8.82	8.42	8.67	7.74	1.45	8.64	0.20	1.12	0.157	3.5E-01
Mtr.35661.1.S1_at	2.89	5.39	2.88	2.90	2.65	2.84	3.72	1.45	2.80	0.13	0.75	-0.411	3.3E-01
Mtr.13013.1.S1_at	6.93	4.32	6.72	6.78	6.77	6.40	5.99	1.45	6.65	0.21	1.11	0.152	4.8E-01
Mtr.5751.1.S1_s_at	5.71	2.90	4.92	5.08	5.55	6.00	4.51	1.45	5.54	0.46	1.23	0.297	3.1E-01
Mtr.1849.1.S1_a_at	5.29	2.78	5.28	6.27	6.18	5.42	4.45	1.45	5.95	0.47	1.34	0.419	1.6E-01
Mtr.12840.1.S1_at	7.19	7.50	4.84	7.45	9.13	7.75	6.51	1.45	8.11	0.89	1.25	0.317	1.8E-01
Mtr.31470.1.S1_s_at	4.97	2.16	4.20	2.64	4.03	2.83	3.78	1.45	3.17	0.75	0.84	-0.254	5.5E-01
Mtr.9991.1.S1_at	6.83	4.06	6.17	6.48	7.21	7.14	5.69	1.45	6.95	0.40	1.22	0.288	2.2E-01
Mtr.46949.1.S1_at	9.18	6.36	8.35	9.02	8.87	9.28	7.96	1.45	9.06	0.21	1.14	0.186	2.6E-01
Mtr.24598.1.S1_at	7.58	5.05	7.55	7.65	7.57	7.61	6.72	1.45	7.61	0.04	1.13	0.178	3.5E-01
Mtr.51042.1.S1_at	8.72	5.82	7.19	7.39	7.56	7.58	7.24	1.45	7.51	0.10	1.04	0.052	7.7E-01
Mtr.20124.1.S1_at	7.46	4.90	7.36	6.98	7.38	7.15	6.57	1.45	7.17	0.20	1.09	0.125	5.2E-01
Mtr.40209.1.S1_at	5.70	3.08	5.45	7.43	9.56	8.79	4.74	1.45	8.59	1.08	1.81	0.857	2.1E-02
Mtr.26561.1.S1_at	7.87	5.53	8.19	7.69	7.76	7.71	7.20	1.45	7.72	0.04	1.07	0.102	5.6E-01
Mtr.39369.1.S1_at	6.56	4.30	6.99	6.69	6.55	6.66	5.95	1.45	6.63	0.08	1.12	0.157	4.6E-01
Mtr.34346.1.S1_at	1.99	4.49	1.98	1.98	1.98	1.98	2.82	1.45	1.98	0.00	0.70	-0.512	3.7E-01
Mtr.44840.1.S1_at	6.00	3.11	4.34	6.05	6.33	6.16	4.48	1.45	6.18	0.15	1.38	0.463	1.1E-01
Mtr.35365.1.S1_at	6.55	3.72	5.66	6.27	6.30	6.19	5.31	1.45	6.25	0.06	1.18	0.235	3.2E-01
Mtr.47533.1.S1_at	2.52	5.02	2.52	2.51	2.51	2.51	3.36	1.45	2.51	0.00	0.75	-0.417	3.7E-01
Mtr.35592.1.S1_at	7.70	5.11	7.52	7.39	6.98	6.98	6.77	1.45	7.12	0.24	1.05	0.072	7.0E-01
Mtr.8108.1.S1_at	2.71	5.21	2.71	2.70	2.70	2.69	3.55	1.45	2.70	0.00	0.76	-0.395	3.7E-01
Mtr.29383.1.S1_at	9.34	6.71	9.06	8.87	8.21	8.56	8.37	1.45	8.55	0.33	1.02	0.030	8.4E-01
Mtr.46628.1.S1_s_at	9.79	7.13	9.43	9.22	9.38	9.23	8.78	1.44	9.28	0.09	1.06	0.079	5.9E-01
Mtr.17040.1.S1_s_at	7.26	4.71	7.17	7.47	7.24	6.96	6.38	1.44	7.22	0.25	1.13	0.179	3.8E-01
Mtr.31487.1.S1_s_at	7.95	5.06	6.57	6.67	6.29	6.42	6.52	1.44	6.46	0.19	0.99	-0.014	9.4E-01
Mtr.39169.1.S1_at	7.56	5.34	8.05	7.43	7.64	7.61	6.98	1.44	7.56	0.11	1.08	0.115	5.3E-01
Mtr.41952.1.S1_at	6.25	3.74	6.23	6.60	6.26	6.39	5.41	1.44	6.42	0.18	1.19	0.247	3.0E-01
Mtr.46935.1.S1_at	3.14	5.12	2.31	2.39	2.08	2.36	3.52	1.44	2.28	0.17	0.65	-0.631	2.1E-01
Mtr.11040.1.S1_s_at	8.56	5.71	7.51	8.42	8.46	8.75	7.26	1.44	8.54	0.18	1.18	0.235	2.0E-01
Mtr.4299.1.S1_s_at	4.27	2.02	4.70	2.14	4.96	4.60	3.66	1.44	3.90	1.53	1.06	0.090	8.6E-01
Mtr.20193.1.S1_x_at	2.54	5.03	2.52	2.50	2.49	2.48	3.36	1.44	2.49	0.01	0.74	-0.432	3.6E-01
Mtr.13838.1.S1_at	5.89	3.00	4.36	4.92	3.92	4.08	4.41	1.44	4.31	0.54	0.98	-0.036	9.1E-01
Mtr.23373.1.S1_at	6.91	4.88	7.67	7.13	6.96	7.19	6.49	1.44	7.10	0.12	1.09	0.129	5.1E-01
Mtr.2603.1.S1_at	7.06	4.36	6.60	7.31	7.65	7.30	6.01	1.44	7.42	0.20	1.23	0.304	1.7E-01
Mtr.40377.1.S1_at	11.62	8.79	9.72	10.62	11.23	10.87	10.04	1.44	10.91	0.30	1.09	0.119	3.7E-01
Mtr.18992.1.S1_at	3.19	5.68	3.19	3.23	3.18	3.18	4.02	1.44	3.20	0.03	0.80	-0.330	3.8E-01
Mtr.38585.1.S1_at	6.64	4.00	6.30	7.19	7.53	7.20	5.65	1.44	7.31	0.19	1.29	0.372	1.2E-01
Mtr.43409.1.S1_at	9.54	11.57	8.78	9.63	10.01	9.42	9.96	1.44	9.69	0.30	0.97	-0.041	7.6E-01
Mtr.28136.1.S1_at	5.40	3.07	5.70	4.23	5.54	5.29	4.72	1.44	5.02	0.70	1.06	0.089	7.6E-01
Mtr.41822.1.S1_at	7.40	5.09	7.74	7.41	7.39	7.58	6.74	1.44	7.46	0.11	1.11	0.146	4.4E-01
Mtr.22924.1.S1_at	5.99	3.57	6.13	6.03	5.74	5.53	5.23	1.44	5.77	0.25	1.10	0.140	5.6E-01
Mtr.27209.1.S1_at	9.85	7.15	7.65	8.69	8.40	9.04	8.22	1.44	8.71	0.32	1.06	0.084	5.9E-01
Mtr.44492.1.S1_at	5.23	2.52	3.03	2.72	2.45	2.50	3.59	1.44	2.55	0.14	0.71	-0.492	2.8E-01
Mtr.34804.1.S1_at	7.02	4.46	6.86	6.77	6.84	6.86	6.11	1.44	6.82	0.05	1.12	0.158	4.4E-01
Mtr.24755.1.S1_at	6.96	4.48	6.98	7.23	7.17	7.61	6.14	1.44	7.34	0.24	1.20	0.257	2.3E-01
Mtr.44968.1.S1_at	10.12	7.68	10.22	10.23	9.98	10.76	9.34	1.44	10.33	0.40	1.11	0.144	3.2E-01
Mtr.28573.1.S1_at	7.17	5.17	7.95	7.34	7.48	7.40	6.76	1.44	7.41	0.07	1.10	0.131	4.8E-01
Mtr.40345.1.S1_at	6.75	3.95	5.92	6.09	6.47	6.35	5.54	1.44	6.30	0.19	1.14	0.186	4.1E-01
Mtr.18975.1.S1_at	7.39	4.95	7.48	6.37	7.41	6.92	6.60	1.44	6.90	0.52	1.04	0.063	7.6E-01
Mtr.34745.1.S1_at	7.38	5.18	7.87	7.27	7.17	7.06	6.81	1.43	7.17	0.11	1.05	0.073	6.9E-01
Mtr.10231.1.S1_at	6.65	4.76	7.58	6.72	6.24	6.17	6.33	1.43	6.37	0.30	1.01	0.010	9.6E-01
Mtr.2560.1.S1_at	2.59	5.07	2.58	2.58	2.57	2.55	3.41	1.43	2.56	0.01	0.75	-0.412	3.6E-01
Mtr.45287.1.S1_at	6.90	4.13	6.13	6.49	6.38	6.21	5.72	1.43	6.36	0.14	1.11	0.153	4.9E-01
Mtr.1802.1.S1_at	6.80	4.03	6.05	6.25	6.13	6.03	5.62	1.43	6.14	0.11	1.09	0.126	5.7E-01

Mtr.28661.1.S1_at	4.98	2.88	5.62	6.44	5.57	5.33	4.50	1.43	5.78	0.59	1.29	0.362	2.2E-01
Mtr.9337.1.S1_at	8.35	5.93	8.47	8.36	8.21	7.92	7.58	1.43	8.16	0.22	1.08	0.106	5.3E-01
Mtr.41828.1.S1_at	9.51	6.66	8.35	7.52	6.16	7.27	8.17	1.43	6.98	0.72	0.85	-0.227	2.7E-01
Mtr.14329.1.S1_at	2.51	4.98	2.48	2.47	2.48	2.47	3.33	1.43	2.47	0.01	0.74	-0.427	3.6E-01
Mtr.22656.1.S1_s_at	10.18	10.91	8.15	8.94	10.33	9.94	9.75	1.43	9.74	0.72	1.00	-0.001	9.9E-01
Mtr.5725.1.S1_s_at	8.79	5.92	7.44	7.47	7.77	7.44	7.38	1.43	7.56	0.18	1.02	0.034	8.4E-01
Mtr.50819.1.S1_s_at	10.36	7.63	9.73	9.87	10.11	9.95	9.24	1.43	9.98	0.12	1.08	0.111	4.3E-01
Mtr.154.1.S1_at	4.80	2.25	4.66	5.41	4.30	5.03	3.90	1.43	4.92	0.56	1.26	0.332	3.2E-01
Mtr.6869.1.S1_s_at	6.61	3.80	5.64	6.08	5.99	6.44	5.35	1.43	6.17	0.24	1.15	0.206	3.8E-01
Mtr.9032.1.S1_at	7.64	5.03	7.34	7.32	7.41	7.53	6.67	1.43	7.42	0.10	1.11	0.154	4.1E-01
Mtr.474.1.S1_s_at	7.23	4.91	7.52	8.17	8.39	8.32	6.56	1.43	8.29	0.11	1.26	0.339	1.0E-01
Mtr.32790.1.S1_at	4.90	2.79	5.51	5.13	7.17	6.80	4.40	1.43	6.37	1.09	1.45	0.534	1.3E-01
Mtr.5844.1.S1_at	6.37	3.90	6.37	6.49	6.71	6.34	5.55	1.43	6.51	0.19	1.17	0.231	3.1E-01
Mtr.48210.1.S1_at	2.95	5.38	2.86	2.87	2.82	2.84	3.73	1.43	2.84	0.02	0.76	-0.393	3.4E-01
Mtr.43346.1.S1_at	11.90	9.22	11.42	11.40	11.82	11.32	10.85	1.43	11.51	0.27	1.06	0.086	4.7E-01
Mtr.6442.1.S1_at	2.63	5.08	2.60	2.58	2.61	2.57	3.44	1.43	2.59	0.02	0.75	-0.409	3.6E-01
Mtr.34785.1.S1_at	6.92	4.58	7.17	6.99	6.88	6.87	6.22	1.43	6.91	0.07	1.11	0.153	4.5E-01
Mtr.41148.1.S1_at	10.01	7.47	9.87	9.86	9.65	9.99	9.12	1.43	9.83	0.17	1.08	0.109	4.4E-01
Mtr.11851.1.S1_at	9.30	7.28	10.03	9.47	9.52	9.52	8.87	1.43	9.50	0.03	1.07	0.099	4.8E-01
Mtr.49141.1.S1_at	2.82	5.27	2.78	2.79	2.73	2.72	3.62	1.43	2.75	0.03	0.76	-0.399	3.5E-01
Mtr.34803.1.S1_at	5.39	2.62	4.60	5.34	5.56	5.43	4.21	1.43	5.44	0.11	1.29	0.372	2.1E-01
Mtr.40970.1.S1_at	10.65	8.21	10.71	11.06	11.02	11.22	9.86	1.43	11.10	0.10	1.13	0.171	2.1E-01
Mtr.29875.1.S1_at	2.88	2.61	5.20	7.48	7.90	7.29	3.56	1.43	7.56	0.31	2.12	1.085	9.0E-03
Mtr.13663.1.S1_at	2.61	5.08	2.61	2.60	2.60	2.63	3.43	1.42	2.61	0.01	0.76	-0.395	3.7E-01
Mtr.6099.1.S1_s_at	6.45	4.00	6.48	6.56	6.12	6.75	5.64	1.42	6.48	0.32	1.15	0.199	3.8E-01
Mtr.6784.1.S1_at	7.11	4.65	7.13	7.06	6.85	6.88	6.30	1.42	6.93	0.11	1.10	0.139	4.8E-01
Mtr.27644.1.S1_at	2.32	4.68	2.11	2.00	2.07	2.00	3.04	1.42	2.02	0.04	0.67	-0.585	2.9E-01
Mtr.10817.1.S1_at	5.51	7.77	8.14	9.51	10.66	10.06	7.14	1.42	10.08	0.58	1.41	0.497	3.0E-02
Mtr.14566.1.S1_at	7.98	6.32	9.15	9.10	8.24	8.94	7.81	1.42	8.76	0.46	1.12	0.165	3.3E-01
Mtr.18492.1.S1_at	4.92	7.61	7.06	7.39	8.02	7.32	6.53	1.42	7.58	0.39	1.16	0.214	2.9E-01
Mtr.18582.1.S1_at	6.61	4.27	6.84	6.61	6.55	6.14	5.91	1.42	6.43	0.25	1.09	0.123	5.6E-01
Mtr.32354.1.S1_s_at	8.13	6.11	8.85	8.35	8.33	8.15	7.69	1.42	8.28	0.11	1.08	0.105	5.2E-01
Mtr.44750.1.S1_at	6.10	3.47	5.73	6.06	5.68	5.99	5.10	1.42	5.91	0.20	1.16	0.214	3.8E-01
Mtr.27950.1.S1_at	6.91	4.74	7.42	7.05	7.11	6.82	6.36	1.42	7.00	0.15	1.10	0.138	4.8E-01
Mtr.40824.1.S1_at	8.05	5.30	6.09	6.80	6.36	6.55	6.48	1.42	6.57	0.22	1.01	0.020	9.2E-01
Mtr.20962.1.S1_at	5.62	4.63	7.43	7.65	7.27	7.28	5.90	1.42	7.40	0.22	1.25	0.327	1.4E-01
Mtr.43621.1.S1_at	12.03	9.30	9.97	10.59	10.58	10.75	10.43	1.42	10.64	0.10	1.02	0.028	8.1E-01
Mtr.33281.1.S1_s_at	12.89	10.97	10.12	11.39	12.37	11.88	11.33	1.42	11.88	0.49	1.05	0.069	5.6E-01
Mtr.3686.1.S1_at	7.74	5.76	8.51	7.90	8.48	7.88	7.34	1.42	8.09	0.34	1.10	0.141	4.2E-01
Mtr.1299.1.S1_s_at	11.32	8.58	10.59	10.86	10.00	10.85	10.16	1.42	10.57	0.50	1.04	0.057	6.6E-01
Mtr.19348.1.S1_s_at	8.39	5.63	7.59	7.59	7.87	7.54	7.21	1.42	7.67	0.18	1.06	0.090	6.1E-01
Mtr.16970.1.S1_at	2.21	4.66	2.20	2.19	2.19	2.18	3.02	1.42	2.19	0.00	0.72	-0.465	3.7E-01
Mtr.6018.1.S1_at	7.87	5.27	7.55	7.38	7.37	7.21	6.90	1.42	7.32	0.09	1.06	0.085	6.3E-01
Mtr.15324.1.S1_at	9.76	7.66	10.36	9.91	9.65	9.82	9.26	1.42	9.79	0.13	1.06	0.081	5.5E-01
Mtr.5583.1.S1_at	7.90	5.18	7.22	7.66	8.02	7.53	6.77	1.42	7.73	0.26	1.14	0.193	3.1E-01
Mtr.51650.1.S1_at	2.51	5.02	2.62	2.58	2.54	2.58	3.39	1.42	2.57	0.02	0.76	-0.398	3.8E-01
Mtr.1759.1.S1_at	7.09	4.90	7.55	7.46	7.25	7.22	6.51	1.42	7.31	0.13	1.12	0.167	3.9E-01
Mtr.1967.1.S1_at	5.70	3.60	6.31	6.34	7.08	6.09	5.20	1.42	6.50	0.51	1.25	0.322	2.1E-01
Mtr.46312.1.S1_a_at	4.45	6.82	4.29	4.70	4.17	4.25	5.19	1.42	4.37	0.28	0.84	-0.247	3.8E-01
Mtr.14003.1.S1_at	5.89	3.41	5.85	5.81	5.72	5.54	5.05	1.42	5.69	0.14	1.13	0.173	4.8E-01
Mtr.10498.1.S1_at	10.61	8.40	7.96	9.08	10.12	9.58	8.99	1.42	9.59	0.52	1.07	0.094	5.3E-01
Mtr.16027.1.S1_at	4.44	2.48	5.24	5.72	5.28	4.54	4.06	1.42	5.18	0.60	1.28	0.352	2.7E-01
Mtr.41824.1.S1_at	8.59	5.76	7.09	8.07	8.16	8.20	7.15	1.42	8.14	0.07	1.14	0.188	2.9E-01
Mtr.49670.1.S1_at	9.19	7.00	9.65	9.48	9.23	9.29	8.61	1.41	9.33	0.13	1.08	0.116	4.3E-01
Mtr.48863.1.S1_at	2.09	4.54	2.08	2.08	2.08	2.08	2.91	1.41	2.08	0.00	0.72	-0.482	3.7E-01
Mtr.31615.1.S1_at	5.16	2.90	5.50	5.17	5.63	5.22	4.52	1.41	5.34	0.25	1.18	0.242	3.8E-01
Mtr.3241.1.S1_at	2.51	4.95	2.50	2.49	2.49	2.72	3.32	1.41	2.57	0.13	0.77	-0.372	4.1E-01
Mtr.44524.1.S1_at	5.91	3.96	6.71	5.93	5.68	5.31	5.53	1.41	5.64	0.31	1.02	0.029	9.0E-01
Mtr.39876.1.S1_at	7.05	4.64	7.12	6.93	7.08	6.30	6.27	1.41	6.77	0.41	1.08	0.110	5.9E-01
Mtr.5885.1.S1_s_at	8.42	5.73	7.83	8.35	8.11	8.34	7.33	1.41	8.27	0.14	1.13	0.175	3.1E-01
Mtr.33214.1.S1_at	6.47	4.08	6.57	6.48	6.07	6.39	5.71	1.41	6.31	0.22	1.11	0.146	5.0E-01
Mtr.45220.1.S1_at	4.94	2.68	5.27	4.79	4.73	4.76	4.30	1.41	4.76	0.03	1.11	0.148	6.0E-01
Mtr.6074.1.S1_at	7.05	4.61	7.07	7.07	7.03	7.18	6.24	1.41	7.09	0.08	1.14	0.184	3.6E-01
Mtr.21367.1.S1_at	3.04	1.60	4.43	3.77	3.47	3.48	3.02	1.41	3.57	0.17	1.18	0.240	5.4E-01
Mtr.20386.1.S1_s_at	5.32	3.06	2.73	2.64	2.60	2.59	3.71	1.41	2.61	0.02	0.70	-0.506	2.5E-01
Mtr.15380.1.S1_at	7.69	5.54	8.20	7.80	8.13	7.12	7.15	1.41	7.68	0.52	1.08	0.105	5.7E-01
Mtr.33723.1.S1_at	3.55	6.05	3.67	3.44	3.18	3.37	4.42	1.41	3.33	0.13	0.75	-0.410	2.5E-01
Mtr.49172.1.S1_at	10.16	8.16	10.89	10.74	10.53	10.39	9.74	1.41	10.55	0.18	1.08	0.116	3.8E-01
Mtr.37413.1.S1_s_at	6.02	3.29	5.26	5.07	5.06	5.05	4.86	1.41	5.06	0.01	1.04	0.059	8.2E-01
Mtr.10312.1.S1_at	8.43	6.18	8.78	8.91	8.79	8.48	7.80	1.41	8.73	0.22	1.12	0.163	3.2E-01
Mtr.6919.1.S1_s_at	12.17	9.42	10.26	10.98	9.93	10.96	10.61	1.41	10.62	0.60	1.00	0.001	9.9E-01
Mtr.1679.1.S1_at	6.88	4.44	6.88	6.84	6.72	6.64	6.06	1.41	6.73	0.10	1.11	0.151	4.6E-01
Mtr.49526.1.S1_at	7.28	4.86	7.32	7.02	6.94	7.10	6.49	1.41	7.02	0.08	1.08	0.114	5.5E-01
Mtr.47840.1.S1_x_at	2.03	4.46	2.02	2.02	2.02	2.01	2.84	1.41	2.02	0.00	0.71	-0.491	3.7E-01
Mtr.33909.1.S1_at	7.12	4.73	7.22	7.13	7.32	6.93	6.36	1.41	7.13	0.19	1.12	0.164	4.0E-01
Mtr.43148.1.S1_at	11.68	8.92	9.80	10.92	10.48	11.08	10.13	1.41	10.83	0.31	1.07	0.096	4.5E-01
Mtr.1417.1.S1_at	2.97	5.35	2.85	2.76	2.76	2.72	3.72	1.41	2.75	0.03	0.74	-0.438	3.0E-01
Mtr.52272.1.S1_at	5.20	2.46	4.42	4.82	4.42	4.49	4.03	1.41	4.57	0.21	1.14	0.183	5.4E-01
Mtr.26753.1.S1_at	2.53	4.96	2.51	2.50	2.50	2.48	3.33	1.41	2.49	0.01	0.75	-0.418	3.6E-01
Mtr.28066.1.S1_s_at	6.48	3.89	4.23	3.77	3.85	3.69	4.87	1.41	3.77	0.08	0.77	-0.369	2.5E-01
Mtr.4092.1.S1_at	4.74	7.10	4.60	4.74	5.26	4.69	5.48	1.41	4.90	0.31	0.89	-0.162	5.2E-01
Mtr.45471.1.S1_at	4.72	2.52	5.15	5.27	5.83	5.89	4.13	1.41	5.66	0.34	1.37	0.456	1.4E-01
Mtr.23128.1.S1_at	3.49	6.02	3.68	3.76	3.89	3.62	4.40	1.41	3.76	0.13	0.85	-0.227	4.8E-01
Mtr.40223.1.S1_at	10.76	10.67	13.16	12.77	14.01	12.71	11.53	1.41	13.17	0.74	1.14	0.191	1.5E-01
Mtr.43742.1.S1_at	10.56	7.94	8.35	9.39	8.73	9.09	8.95	1.41	9.07	0.33	1.01	0.019	8.9E-01
Mtr.35737.1.S1_at	5.56	2.99	5.29	5.46	5.23	5.23	4.61	1.41	5.31	0.14	1.15	0.202	4.4E-01
Mtr.40695.1.S1_s_at	5.51	3.11	3.03	3.03	3.03	2.97	3.88	1.41	3.01	0.03	0.77	-0.368	3.4E-01
Mtr.7906.1.S1_at	7.04	4.42	6.62	6.40	6.45	6.59	6.02	1.41	6.48	0.10	1.08	0.105	6.1E-01
Mtr.4975.1.S1_s_at	9.38	6.57	7.83	8.71	8.64	8.72	7.92	1.41	8.69	0.04	1.10	0.133	4.0E-01
Mtr.11929.1.S1_at	8.53	5.91	8.11	8.09	7.94	8.10	7.52	1.41	8.04	0.09	1.07	0.098	5.5E-01
Mtr.29768.1.S1_at	3.18	5.59	3.12	3.06	3.13	3.33	3.96	1.41	3.17	0.14	0.80	-0.320	3.9E-01
Mtr.47216.1.S1_s_at	5.93	3.39	5.70	5.95	5.69	6.30	5.01	1.41	5.98	0.31	1.19	0.256	3.1E-01
Mtr.19667.1.S1_at	8.36	5.55	6.82	7.23	6.71	7.11	6.91	1.41	7.02	0.27	1.02	0.022	9.0E-01
Mtr.28366.1.S1_at	2.27	4.70	2.26	2.25	2.25	2.25	3.08	1.40	2.25	0.00	0.73	-0.451	3.7E-01
Mtr.5126.1.S1_s_at	7.57	5.32	7.91	8.47	8.21	8.06	6.93	1.40	8.24	0.21	1.19	0.250	1.8E-01
Mtr.2138.1.S1_at	4.27	3.15	5.94	4.71	4.57	4.33	4.45	1.40	4.54	0.19	1.02	0.027	9.2E-01
Mtr.39384.1.S1_at	5.31	3.49	6.25	5.55	5.66	5.32	5.01	1.40	5.51	0.17	1.10	0.136	5.8E-01
Mtr.33520.1.S1_at	8.26	5.77	8.15	7.92	7.80	7.69	7.39	1.40	7.80	0.11	1.06	0.078	6.4E-01
Mtr.27377.1.S1_s_at	6.27	3.80	6.18	6.00	6.16	5.78	5.42	1.40	5.98	0.19	1.10	0.143	5.3E-01
Mtr.26619.1.S1_at	3.31	6.10	4.42	5.90	6.43	5.51	4.61	1.40	5.95	0.46	1.29	0.367	1.9E-01
Mtr.45053.1.S1_at	10.12	7.43	9.46	9.81	9.51	9.78	9.00	1.40	9.70	0.16	1.08	0.107	4.4E-01
Mtr.7638.1.S1_s_at	11.34	9.55	8.57	9.22	9.12	9.59	9.82	1.40	9.31	0.24	0.95	-0.077	5.7E-01
Mtr.39539.1.S1_at	7.11	5.18	7.91	7.74	8.51	7.87	6.73	1.40	8.04	0.41	1.19	0.256	2.0E-01
Mtr.36953.1.S1_at	7.58	5.21	7.70	7.45	7.42	7.54	6.83	1.40	7.47	0.06	1.09	0.129	4.7E-01
Mtr.1915.1.S1_at	2.21	4.63	2.20	2.19	2.19	2.19	3.01	1.40	2.19	0.00	0.73	-0.459	3.7E-01
Mtr.11333.1.S1_at	8.04	5.60	8.01	8.32	8.17	8.62	7.21	1.40	8.37	0.23	1.16	0.214	2.3E-01
Mtr.2734.1.S1_at	5.86	3.19	5.26	5.52	5.62	4.83	4.77	1.40	5.32	0.43	1.12	0.158	5.5E-01
Mtr.16356.1.S1_s_at	5.04	3.78	6.58	6.27	5.87	6.26	5.13	1.40	6.13	0.22	1.19	0.257	2.9E-01
Mtr.32879.1.S1_at	6.93	4.47	6.86	6.86	6.73	7.10	6.09	1.40	6.90	0.19	1.13	0.179	3.8E-01
Mtr.31314.1.S1_at	6.68	5.21	8.01	8.41	8.53	8.32	6.63	1.40	8.42	0.10	1.27	0.344	9.2E-02
Mtr.44423.1.S1_at	9.17	6.95	9.54	9.34	9.09	9.11	8.55	1.40	9.18	0.14	1.07	0.102	4.8E-01
Mtr.12749.1.S1_at	7.86	5.83	8.51	9.24	9.98	9.82	7.40	1.40	9.68	0.39	1.31	0.388	5.3E-02
Mtr.10857.1.S1_at	8.16	5.98	8.59	7.87	7.88	7.74	7.58	1.40	7.83	0.08	1.03	0.047	7.7E-01
Mtr.5021.1.S1_at	2.25	4.66	2.24	2.28	2.20	2.20	3.05	1.40	2.23	0.04	0.73	-0.454	3.7E-01
Mtr.52065.1.S1_at	7.90	5.22	7.27	7.52	7.12	7.41	6.80	1.40	7.35	0.21	1.08	0.113	5.3E-01
Mtr.2437.1.S1_at	6.49	4.09	6.54	6.56	6.26	6.49	5.71	1.40	6.44	0.16	1.13	0.174	4.2E-01
Mtr.5255.1.S1_at	6.37	4.14	6.71	7.00	6.66	6.16	5.74	1.40	6.61	0.42	1.15	0.203	3.6E-01
Mtr.6132.1.S1_s_at	8.40	5.71	7.70	7.91	7.97	8.16	7.27	1.40	8.01	0.13	1.10	0.140	4.1E-01
Mtr.6035.1.S1_at	4.62	2.35	4.90	4.30	4.13	4.40	3.96	1.40	4.27	0.14	1.08	0.112	7.1E-01
Mtr.47815.1.S1_at	5.32	3.54	6.29	5.32	5.74	5.14	5.05	1.39	5.40	0.31	1.07	0.095	7.0E-01
Mtr.43628.1.S1_at	6.34	4.90	3.56	3.34	3.34	3.83	4.93	1.39	3.50	0.28	0.71	-0.494	1.6E-01
Mtr.50074.1.S1_at	5.12	5.89	7.82	7.83	7.72	8.25	6.28	1.39	7.93	0.28	1.26	0.337	1.1E-01
Mtr.7536.1.S1_at	6.05	3.54	5.83	5.82	6.23	5.82	5.14	1.39	5.95	0.24	1.16	0.212	3.8E-01
Mtr.14588.1.S1_s_at	6.67	5.25	8.04	7.08	8.04	7.17	6.65	1.39	7.43	0.53	1.12	0.160	4.2E-01
Mtr.17372.1.S1_at	6.65	4.08	6.30	5.71	5.53	6.11	5.67	1.39	5.78	0.30	1.02	0.027	9.0E-01
Mtr.12401.1.S1_s_at	12.45	9.67	11.00	12.05	11.59	12.08	11.04	1.39	11.90	0.27	1.08	0.109	3.5E-01
Mtr.12809.1.S1_at	6.71	4.91	7.65	6.60	6.79	6.42	6.42	1.39	6.60	0.19	1.03	0.040	8.3E-01
Mtr.11791.1.S1_at	5.19	3.34	6.06	4.91	4.86	4.69	4.86	1.39	4.82	0.11	0.99	-0.012	9.6E-01
Mtr.27718.1.S1_at	6.64	5.07	7.84	6.69	6.40	6.80	6.52	1.39	6.63	0.21	1.02	0.024	9.0E-01
Mtr.50641.1.S1_at	3.22	5.52	3.02	3.38	2.91	3.20	3.92	1.39	3.16	0.24	0.81	-0.310	4.0E-01
Mtr.32680.1.S1_at	4.04	2.62	5.40	4.23	5.46	5.15	4.02	1.39	4.95	0.64	1.23	0.298	3.6E-01

Mtr.24296.1.S1_at	7.05	4.55	6.86	7.09	6.09	6.76	6.16	1.39	6.65	0.51	1.08	0.110	6.0E-01
Mtr.27794.1.S1_at	7.02	4.87	7.48	7.37	7.37	7.24	6.46	1.39	7.33	0.07	1.13	0.182	3.4E-01
Mtr.784.1.S1_at	3.01	5.41	2.98	2.96	2.88	2.95	3.80	1.39	2.93	0.04	0.77	-0.377	3.4E-01
Mtr.25223.1.S1_s_at	6.26	3.86	6.27	6.07	6.06	6.12	5.46	1.39	6.08	0.04	1.11	0.155	4.8E-01
Mtr.43847.1.S1_at	7.26	4.94	7.43	7.17	7.42	7.21	6.54	1.39	7.26	0.13	1.11	0.150	4.2E-01
Mtr.28602.1.S1_at	9.64	6.98	9.01	8.61	8.53	8.80	8.54	1.39	8.65	0.14	1.01	0.018	9.0E-01
Mtr.42350.1.S1_at	6.73	4.16	6.36	6.73	6.93	6.45	5.75	1.39	6.70	0.24	1.17	0.222	3.1E-01
Mtr.5617.1.S1_s_at	6.47	4.03	6.39	6.45	6.37	6.28	5.63	1.39	6.37	0.08	1.13	0.178	4.1E-01
Mtr.19465.1.S1_at	11.10	8.91	11.48	11.12	10.94	10.94	10.50	1.39	11.00	0.10	1.05	0.067	5.7E-01
Mtr.4697.1.S1_at	4.84	3.91	6.64	5.82	5.11	5.99	5.13	1.39	5.64	0.47	1.10	0.137	5.8E-01
Mtr.17161.1.S1_at	3.22	5.61	3.19	3.15	3.19	3.14	4.01	1.39	3.16	0.03	0.79	-0.345	3.5E-01
Mtr.49845.1.S1_at	7.93	5.57	8.02	7.74	7.74	7.77	7.17	1.39	7.75	0.02	1.08	0.112	5.1E-01
Mtr.24203.1.S1_at	6.90	4.56	7.01	7.54	7.75	7.49	6.15	1.39	7.60	0.14	1.23	0.304	1.5E-01
Mtr.47257.1.S1_at	12.40	9.69	10.55	9.90	11.33	10.81	10.88	1.39	10.68	0.73	0.98	-0.027	8.3E-01
Mtr.25806.1.S1_at	3.07	5.54	3.22	3.40	3.21	3.26	3.94	1.39	3.29	0.10	0.83	-0.261	4.6E-01
Mtr.1539.1.S1_at	1.87	4.27	1.87	1.86	1.86	1.91	2.67	1.39	1.87	0.03	0.70	-0.508	3.8E-01
Mtr.8707.1.S1_at	7.17	4.44	6.18	6.43	5.81	6.51	5.93	1.39	6.25	0.38	1.05	0.075	7.2E-01
Mtr.2561.1.S1_at	7.25	4.69	6.88	5.32	5.79	5.73	6.27	1.38	5.61	0.26	0.89	-0.160	4.6E-01
Mtr.11489.1.S1_at	8.49	5.73	7.16	7.52	7.56	7.66	7.13	1.38	7.58	0.07	1.06	0.089	6.0E-01
Mtr.27907.1.S1_at	7.00	4.43	6.62	7.20	6.85	6.85	6.02	1.38	6.97	0.20	1.16	0.212	3.0E-01
Mtr.39435.1.S1_at	6.09	3.86	6.39	6.07	5.99	5.94	5.45	1.38	6.00	0.06	1.10	0.140	5.3E-01
Mtr.49210.1.S1_at	8.64	6.16	8.47	8.76	8.87	8.82	7.76	1.38	8.82	0.06	1.14	0.185	2.6E-01
Mtr.11624.1.S1_at	6.21	3.74	6.05	6.49	6.85	6.78	5.33	1.38	6.70	0.19	1.26	0.331	1.6E-01
Mtr.38920.1.S1_s_at	4.62	2.60	5.25	4.26	4.11	4.13	4.16	1.38	4.17	0.08	1.00	0.005	9.9E-01
Mtr.10285.1.S1_at	7.19	5.26	7.94	6.92	6.86	6.29	6.80	1.38	6.69	0.35	0.98	-0.024	9.0E-01
Mtr.44246.1.S1_at	7.31	4.61	6.48	6.68	6.50	6.72	6.14	1.38	6.63	0.12	1.08	0.113	5.7E-01
Mtr.33148.1.S1_at	4.39	3.81	6.44	5.90	5.26	5.23	4.88	1.38	5.46	0.38	1.12	0.163	5.2E-01
Mtr.25528.1.S1_x_at	2.53	4.91	2.51	2.50	2.49	2.49	3.32	1.38	2.49	0.01	0.75	-0.413	3.6E-01
Mtr.28050.1.S1_at	7.27	6.03	8.78	7.36	8.02	7.51	7.36	1.38	7.63	0.35	1.04	0.053	7.6E-01
Mtr.3501.1.S1_s_at	7.43	4.86	5.27	6.38	7.73	8.09	5.85	1.38	7.40	0.91	1.26	0.339	1.8E-01
Mtr.39076.1.S1_at	5.01	2.56	4.89	4.89	4.89	4.89	4.15	1.38	4.89	0.00	1.18	0.235	4.1E-01
Mtr.5786.1.S1_at	6.82	4.48	6.91	7.12	6.76	6.80	6.07	1.38	6.89	0.20	1.14	0.183	3.6E-01
Mtr.10835.1.S1_s_at	8.16	6.28	8.96	8.85	8.97	8.57	7.80	1.38	8.80	0.21	1.13	0.173	2.8E-01
Mtr.42030.1.S1_at	6.07	3.72	6.14	5.78	5.40	5.77	5.31	1.38	5.65	0.22	1.07	0.091	6.9E-01
Mtr.31352.1.S1_at	2.17	4.56	2.17	2.16	2.15	2.17	2.97	1.38	2.16	0.01	0.73	-0.458	3.7E-01
Mtr.51059.1.S1_x_at	8.08	7.43	5.43	7.97	7.51	7.14	6.98	1.38	7.54	0.42	1.08	0.111	5.4E-01
Mtr.11589.1.S1_at	8.32	5.95	8.36	8.76	8.06	9.25	7.54	1.38	8.69	0.60	1.15	0.204	2.6E-01
Mtr.1745.1.S1_at	6.26	4.07	6.61	7.02	6.87	7.03	5.64	1.38	6.97	0.09	1.24	0.305	1.7E-01
Mtr.949.1.S1_at	6.95	4.35	6.43	6.71	6.12	5.92	5.91	1.38	6.25	0.41	1.06	0.081	7.0E-01
Mtr.21665.1.S1_at	6.36	4.58	7.29	7.62	7.19	7.55	6.08	1.38	7.45	0.23	1.23	0.294	1.6E-01
Mtr.39788.1.S1_at	8.82	6.18	8.17	8.00	8.15	8.38	7.72	1.38	8.18	0.19	1.06	0.082	6.0E-01
Mtr.6096.1.S1_at	5.03	2.56	4.86	4.89	4.87	5.01	4.15	1.38	4.92	0.07	1.19	0.247	3.9E-01
Mtr.47198.1.S1_s_at	6.01	3.27	4.90	5.73	4.89	5.18	4.73	1.38	5.27	0.43	1.11	0.156	5.5E-01
Mtr.41949.1.S1_at	6.40	4.23	6.79	6.51	6.46	6.25	5.80	1.38	6.41	0.13	1.10	0.142	4.9E-01
Mtr.49651.1.S1_at	2.90	5.27	2.88	2.87	2.86	2.88	3.69	1.38	2.87	0.01	0.78	-0.362	3.6E-01
Mtr.42258.1.S1_at	9.40	6.85	9.01	9.56	9.80	9.69	8.42	1.37	9.68	0.12	1.15	0.202	1.9E-01
Mtr.43503.1.S1_at	8.50	6.46	9.07	8.17	7.98	8.06	8.01	1.37	8.07	0.09	1.01	0.011	9.4E-01
Mtr.30584.1.S1_at	2.13	4.50	2.12	2.12	2.11	2.12	2.92	1.37	2.12	0.00	0.73	-0.464	3.7E-01
Mtr.48925.1.S1_at	7.59	5.22	7.61	7.57	7.77	7.39	6.81	1.37	7.58	0.19	1.11	0.155	3.9E-01
Mtr.44959.1.S1_at	4.50	2.44	5.05	4.63	4.64	4.71	4.00	1.37	4.66	0.04	1.17	0.221	4.5E-01
Mtr.2508.1.S1_at	5.78	3.82	6.46	5.86	5.88	5.57	5.35	1.37	5.77	0.17	1.08	0.107	6.3E-01
Mtr.28463.1.S1_at	5.84	3.67	6.22	6.29	6.68	6.03	5.24	1.37	6.34	0.33	1.21	0.273	2.5E-01
Mtr.2148.1.S1_at	6.55	4.18	6.57	6.76	6.76	6.93	5.77	1.37	6.82	0.10	1.18	0.241	2.6E-01
Mtr.41054.1.S1_at	6.44	3.93	6.14	6.94	6.77	7.04	5.51	1.37	6.92	0.14	1.26	0.329	1.5E-01
Mtr.17926.1.S1_at	3.94	3.26	5.90	4.05	5.12	5.06	4.36	1.37	4.74	0.60	1.09	0.120	6.8E-01
Mtr.17611.1.S1_at	6.35	4.15	3.83	6.56	5.61	5.81	4.77	1.37	5.99	0.50	1.26	0.328	2.2E-01
Mtr.52315.1.S1_at	9.48	6.95	7.30	8.32	7.99	8.41	7.91	1.37	8.24	0.22	1.04	0.059	7.0E-01
Mtr.4034.1.S1_at	5.94	3.24	4.22	6.58	7.39	6.58	4.47	1.37	6.85	0.47	1.53	0.617	4.6E-02
Mtr.39063.1.S1_at	7.88	5.14	6.40	7.51	7.06	7.17	6.47	1.37	7.25	0.23	1.12	0.163	3.9E-01
Mtr.19973.1.S1_at	2.87	5.23	2.86	2.85	2.85	2.84	3.65	1.37	2.85	0.01	0.78	-0.360	3.7E-01
Mtr.46680.1.S1_at	4.67	2.27	4.62	4.62	4.82	4.62	3.85	1.37	4.69	0.11	1.22	0.283	3.5E-01
Mtr.12531.1.S1_s_at	11.97	9.38	11.43	11.73	11.81	11.64	10.93	1.37	11.73	0.09	1.07	0.102	3.7E-01
Mtr.52214.1.S1_at	7.93	5.20	6.52	6.13	5.92	5.21	6.55	1.37	5.75	0.48	0.88	-0.188	3.9E-01
Mtr.10361.1.S1_at	11.72	10.33	13.06	12.79	13.81	13.42	11.70	1.37	13.34	0.52	1.14	0.189	1.2E-01
Mtr.38552.1.S1_s_at	8.41	5.68	7.07	7.92	7.25	8.04	7.06	1.37	7.74	0.42	1.10	0.132	4.6E-01
Mtr.8205.1.S1_at	5.80	3.96	6.63	5.91	5.93	5.46	5.46	1.37	5.77	0.27	1.06	0.077	7.3E-01
Mtr.7192.1.S1_at	5.71	3.85	6.51	6.26	6.71	6.20	5.36	1.37	6.39	0.28	1.19	0.254	2.7E-01
Mtr.6399.1.S1_at	2.35	4.71	2.33	2.33	2.33	2.33	3.13	1.37	2.33	0.00	0.74	-0.427	3.7E-01
Mtr.31574.1.S1_s_at	9.26	6.58	8.41	9.30	9.47	9.44	8.08	1.37	9.40	0.09	1.16	0.218	1.7E-01
Mtr.15570.1.S1_at	7.28	5.18	7.75	7.40	7.29	7.30	6.74	1.36	7.33	0.06	1.09	0.122	4.9E-01
Mtr.50857.1.S1_at	10.25	8.04	10.52	10.28	10.33	10.35	9.60	1.36	10.32	0.04	1.07	0.104	4.1E-01
Mtr.32629.1.S1_s_at	12.48	10.31	9.97	10.48	10.78	10.92	10.92	1.36	10.73	0.22	0.98	-0.025	8.2E-01
Mtr.5593.1.S1_at	3.90	1.71	4.21	3.88	3.42	4.02	3.27	1.36	3.78	0.31	1.15	0.208	5.6E-01
Mtr.36441.1.S1_at	5.39	2.82	4.91	4.99	5.02	4.99	4.37	1.36	5.00	0.02	1.14	0.194	4.7E-01
Mtr.35002.1.S1_at	7.96	5.53	7.82	7.85	7.33	7.75	7.11	1.36	7.64	0.28	1.08	0.105	5.4E-01
Mtr.21024.1.S1_at	7.13	4.73	7.06	6.87	7.28	6.85	6.31	1.36	7.00	0.24	1.11	0.151	4.3E-01
Mtr.13054.1.S1_at	7.34	4.69	6.56	5.78	5.07	5.75	6.20	1.36	5.53	0.40	0.89	-0.163	4.6E-01
Mtr.12909.1.S1_at	9.30	6.58	7.97	8.21	7.71	8.08	7.95	1.36	8.00	0.26	1.01	0.009	9.5E-01
Mtr.7214.1.S1_at	7.88	5.16	6.37	7.73	8.20	7.98	6.47	1.36	7.97	0.24	1.23	0.301	1.3E-01
Mtr.15715.1.S1_at	7.56	5.20	7.55	7.72	7.56	7.38	6.77	1.36	7.55	0.17	1.12	0.157	3.8E-01
Mtr.29556.1.S1_at	5.58	4.04	6.75	5.53	5.96	5.79	5.46	1.36	5.76	0.22	1.06	0.078	7.2E-01
Mtr.39393.1.S1_at	6.62	4.36	6.80	5.80	5.86	5.86	5.93	1.36	5.84	0.04	0.99	-0.022	9.2E-01
Mtr.12772.1.S1_at	10.67	7.97	9.03	9.35	9.67	9.68	9.22	1.36	9.57	0.19	1.04	0.053	6.9E-01
Mtr.45059.1.S1_at	1.95	4.30	1.94	1.96	1.94	1.94	2.73	1.36	1.95	0.01	0.71	-0.487	3.8E-01
Mtr.27846.1.S1_at	6.87	4.30	6.35	6.33	6.60	6.04	5.84	1.36	6.33	0.28	1.08	0.116	5.8E-01
Mtr.9479.1.S1_at	5.94	3.42	5.58	4.98	5.54	5.47	4.98	1.36	5.33	0.31	1.07	0.098	6.9E-01
Mtr.9387.1.S1_at	6.65	5.00	7.70	6.78	7.14	6.76	6.45	1.36	6.90	0.22	1.07	0.096	6.1E-01
Mtr.35685.1.S1_at	3.21	5.34	2.81	2.81	2.95	2.95	3.79	1.36	2.90	0.08	0.77	-0.384	3.2E-01
Mtr.27945.1.S1_s_at	9.09	6.57	8.72	8.72	8.42	8.62	8.13	1.36	8.58	0.15	1.06	0.079	5.9E-01
Mtr.23539.1.S1_x_at	2.42	4.77	2.40	2.42	2.42	2.64	3.20	1.36	2.50	0.13	0.78	-0.358	4.2E-01
Mtr.35040.1.S1_at	7.72	5.69	8.26	7.85	8.48	8.02	7.22	1.36	8.12	0.33	1.12	0.168	3.3E-01
Mtr.36679.1.S1_s_at	11.08	8.62	8.86	9.36	8.53	9.31	9.52	1.36	9.07	0.47	0.95	-0.071	6.1E-01
Mtr.14320.1.S1_s_at	6.44	3.81	5.74	5.97	7.08	6.48	5.33	1.36	6.51	0.55	1.22	0.289	2.4E-01
Mtr.32809.1.S1_s_at	7.15	5.06	7.62	7.16	7.13	7.41	6.61	1.36	7.23	0.15	1.09	0.130	4.7E-01
Mtr.6965.1.S1_at	5.53	3.21	5.59	5.59	6.00	5.81	4.78	1.36	5.80	0.21	1.21	0.279	2.7E-01
Mtr.43642.1.S1_at	7.43	6.15	8.86	7.49	7.43	6.94	7.48	1.36	7.29	0.30	0.97	-0.037	8.2E-01
Mtr.40427.1.S1_at	4.37	2.39	4.99	4.81	5.65	5.18	3.92	1.36	5.21	0.42	1.33	0.413	1.9E-01
Mtr.1930.1.S1_at	5.18	2.95	5.41	3.11	3.67	3.88	4.51	1.36	3.55	0.40	0.79	-0.344	3.1E-01
Mtr.27904.1.S1_at	7.04	5.09	7.70	6.86	7.61	7.30	6.61	1.36	7.26	0.38	1.10	0.135	4.7E-01
Mtr.7305.1.S1_at	5.06	2.68	4.99	3.58	3.66	3.58	4.24	1.36	3.60	0.05	0.85	-0.236	4.6E-01
Mtr.26755.1.S1_at	3.04	5.35	2.97	2.97	2.96	2.90	3.79	1.36	2.95	0.04	0.78	-0.362	3.4E-01
Mtr.25921.1.S1_at	7.56	5.03	7.14	6.30	6.95	6.02	6.57	1.36	6.42	0.48	0.98	-0.034	8.6E-01
Mtr.50596.1.S1_at	5.61	3.29	5.67	4.46	5.49	4.83	4.86	1.36	4.93	0.52	1.01	0.020	9.4E-01
Mtr.33491.1.S1_at	6.86	5.07	7.72	7.15	7.42	6.91	6.55	1.36	7.16	0.25	1.09	0.128	4.9E-01
Mtr.28052.1.S1_at	8.73	6.36	8.69	8.80	8.86	8.68	7.93	1.36	8.78	0.09	1.11	0.148	3.4E-01
Mtr.2382.1.S1_at	3.22	5.53	3.15	3.29	3.11	3.01	3.97	1.36	3.14	0.14	0.79	-0.338	3.5E-01
Mtr.37017.1.S1_at	4.24	2.73	5.43	4.23	4.40	4.15	4.13	1.36	4.26	0.12	1.03	0.043	8.8E-01
Mtr.21310.1.S1_at	10.98	13.33	10.99	11.12	12.33	11.80	11.76	1.35	11.75	0.61	1.00	-0.002	9.9E-01
Mtr.33651.1.S1_at	6.98	4.87	7.39	6.24	6.30	6.24	6.41	1.35	6.26	0.03	0.98	-0.034	8.6E-01
Mtr.11656.1.S1_at	5.64	3.94	2.96	3.83	4.61	3.89	4.18	1.35	4.11	0.43	0.98	-0.023	9.4E-01
Mtr.12910.1.S1_s_at	8.38	5.87	8.00	7.83	8.38	7.98	7.42	1.35	8.06	0.28	1.09	0.120	4.6E-01
Mtr.33549.1.S1_at	6.58	8.15	5.45	6.15	8.02	6.93	6.73	1.35	7.03	0.94	1.05	0.064	7.6E-01
Mtr.39473.1.S1_at	5.51	4.57	7.24	6.04	6.34	5.39	5.78	1.35	5.92	0.49	1.03	0.036	8.7E-01
Mtr.12327.1.S1_at	5.73	3.76	3.13	3.58	3.58	3.57	4.21	1.35	3.58	0.01	0.85	-0.234	4.6E-01
Mtr.39566.1.S1_at	8.64	6.54	9.07	8.92	8.90	8.77	8.08	1.35	8.86	0.08	1.10	0.133	3.7E-01
Mtr.37420.1.S1_at	8.28	6.96	9.67	8.17	8.01	7.68	8.30	1.35	7.95	0.25	0.96	-0.062	6.8E-01
Mtr.18915.1.S1_x_at	2.33	4.67	2.33	2.30	2.32	2.32	3.11	1.35	2.31	0.02	0.74	-0.426	3.7E-01
Mtr.346.1.S1_at	2.59	4.92	2.56	2.56	2.56	2.56	3.35	1.35	2.56	0.00	0.76	-0.392	3.6E-01
Mtr.11526.1.S1_at	6.74	4.52	6.97	6.65	6.91	6.69	6.07	1.35	6.75	0.14	1.11	0.152	4.4E-01
Mtr.23230.1.S1_at	2.34	4.67	2.32	2.31	2.32	2.32	3.11	1.35	2.31	0.00	0.74	-0.428	3.6E-01
Mtr.27595.1.S1_at	6.93	4.56	6.89	6.81	6.75	6.55	6.13	1.35	6.70	0.13	1.09	0.130	5.0E-01
Mtr.33642.1.S1_at	2.03	4.37	2.02	2.02	2.01	2.02	2.80	1.35	2.02	0.00	0.72	-0.476	3.7E-01
Mtr.43755.1.S1_at	7.61	5.13	7.31	7.13	7.12	7.12	6.68	1.35	7.12	0.00	1.07	0.091	6.1E-01
Mtr.32262.1.S1_at	7.23	5.00	7.43	7.58	7.49	7.63	6.55	1.35	7.57	0.07	1.15	0.208	2.6E-01
Mtr.30173.1.S1_at	5.38	2.86	4.97	4.70	5.75	5.16	4.41	1.35	5.21	0.53	1.18	0.241	3.9E-01
Mtr.1505.1.S1_at	7.82	5.42	7.69	6.99	7.00	7.57	6.98	1.35	7.18	0.33	1.03	0.042	8.1E-01
Mtr.39058.1.S1_at	8.37	6.08	8.46	8.42	8.70	8.39	7.63	1.35	8.51	0.17	1.11	0.156	3.3E-01
Mtr.11783.1.S1_at	5.35	3.74	6.42	5.89	5.20	5.43	5.17	1.35	5.51	0.35	1.07	0.092	6.9E-01
Mtr.32044.1.S1_at	10.28	8.37	10.97	10.81	10.64	10.48	9.87	1.35	10.64	0.16	1.08	0.108	3.8E-01
Mtr.39852.1.S1_at	7.63	4.96	6.63	7.35	7.15	7.37	6.40	1.35	7.29	0.12	1.14	0.187	3.2E-01
Mtr.28020.1.S1_at	7.18	4.58	6.50	6.77	6.78	7.12	6.09	1.35	6.89	0.20	1.13	0.179	3.7E-01

Mtr.38772.1.S1_at	5.32	3.10	5.53	5.03	5.07	4.22	4.65	1.35	4.77	0.48	1.03	0.038	8.9E-01
Mtr.26919.1.S1_at	2.91	1.92	4.59	2.78	3.65	2.74	3.14	1.35	3.05	0.51	0.97	-0.040	9.2E-01
Mtr.46254.1.S1_at	6.87	4.31	6.30	6.68	6.27	7.20	5.83	1.35	6.71	0.47	1.15	0.204	3.4E-01
Mtr.5052.1.S1_at	7.35	4.97	7.25	7.42	7.41	7.36	6.52	1.35	7.40	0.03	1.13	0.181	3.3E-01
Mtr.28043.1.S1_at	5.78	3.54	5.97	5.90	5.86	5.83	5.10	1.35	5.86	0.03	1.15	0.202	3.8E-01
Mtr.47901.1.S1_at	4.95	3.01	5.60	5.16	4.69	4.84	4.52	1.35	4.90	0.24	1.08	0.115	6.6E-01
Mtr.14349.1.S1_s_at	5.84	4.13	6.79	6.49	6.22	5.77	5.59	1.35	6.16	0.36	1.10	0.141	5.2E-01
Mtr.29231.1.S1_at	6.29	4.01	6.39	6.44	6.82	6.53	5.56	1.35	6.60	0.20	1.19	0.246	2.6E-01
Mtr.49223.1.S1_at	6.62	4.40	6.83	6.66	6.52	6.42	5.95	1.35	6.53	0.12	1.10	0.136	4.9E-01
Mtr.13338.1.S1_at	7.66	5.38	7.78	7.74	7.95	8.12	6.94	1.35	7.94	0.19	1.14	0.194	2.7E-01
Mtr.31575.1.S1_at	9.16	6.62	8.68	9.19	9.54	9.37	8.15	1.35	9.37	0.18	1.15	0.200	2.0E-01
Mtr.42840.1.S1_at	11.13	8.94	11.40	11.19	11.08	10.84	10.49	1.35	11.04	0.18	1.05	0.073	5.2E-01
Mtr.35964.1.S1_x_at	3.05	5.40	3.10	3.09	2.97	2.96	3.85	1.35	3.01	0.07	0.78	-0.356	3.4E-01
Mtr.8224.1.S1_at	6.57	4.77	7.40	6.81	6.78	7.23	6.25	1.35	6.94	0.25	1.11	0.152	4.3E-01
Mtr.47807.1.S1_at	2.23	4.55	2.21	2.20	2.20	2.20	2.99	1.35	2.20	0.00	0.73	-0.445	3.6E-01
Mtr.10274.1.S1_at	6.36	4.34	6.90	6.73	6.33	6.37	5.87	1.35	6.48	0.22	1.10	0.142	4.8E-01
Mtr.8193.1.S1_at	2.76	5.11	2.78	2.74	2.72	2.73	3.55	1.35	2.73	0.01	0.77	-0.379	3.5E-01
Mtr.50503.1.S1_at	2.79	5.06	2.68	2.66	2.64	2.67	3.51	1.35	2.66	0.02	0.76	-0.402	3.3E-01
Mtr.14722.1.S1_at	4.95	2.67	5.05	5.03	5.30	5.11	4.23	1.35	5.15	0.14	1.22	0.284	3.0E-01
Mtr.50240.1.S1_at	7.08	4.81	7.20	7.00	6.91	7.13	6.36	1.35	7.01	0.11	1.10	0.141	4.5E-01
Mtr.11311.1.S1_at	7.09	4.68	6.91	7.13	7.40	7.27	6.23	1.35	7.27	0.13	1.17	0.223	2.5E-01
Mtr.46360.1.S1_x_at	1.75	4.08	1.74	1.74	1.74	1.74	2.53	1.34	1.74	0.00	0.69	-0.536	3.7E-01
Mtr.29117.1.S1_at	2.71	5.04	2.71	2.74	2.70	2.68	3.49	1.34	2.71	0.03	0.78	-0.367	3.7E-01
Mtr.21277.1.S1_at	4.00	3.75	6.19	4.28	5.28	5.41	4.65	1.34	4.99	0.62	1.07	0.103	7.1E-01
Mtr.28046.1.S1_at	8.74	6.40	8.72	8.64	8.78	8.45	7.95	1.34	8.63	0.17	1.08	0.117	4.4E-01
Mtr.49055.1.S1_at	6.69	4.25	6.45	6.30	6.44	6.41	5.80	1.34	6.38	0.07	1.10	0.140	4.9E-01
Mtr.40014.1.S1_at	7.53	5.15	7.42	7.42	7.42	7.47	6.70	1.34	7.43	0.03	1.11	0.150	4.0E-01
Mtr.32444.1.S1_at	8.02	5.93	8.44	8.14	8.16	8.26	7.46	1.34	8.19	0.07	1.10	0.134	4.0E-01
Mtr.39188.1.S1_at	4.95	4.60	7.08	5.40	5.99	5.17	5.54	1.34	5.52	0.43	1.00	-0.007	9.8E-01
Mtr.34162.1.S1_at	4.17	2.36	4.98	6.06	7.05	6.30	3.84	1.34	6.47	0.52	1.69	0.754	3.4E-02
Mtr.17151.1.S1_at	2.49	4.87	2.61	2.61	2.56	2.78	3.32	1.34	2.65	0.11	0.80	-0.326	4.4E-01
Mtr.31774.1.S1_at	8.18	5.56	7.35	7.73	7.60	7.86	7.03	1.34	7.73	0.13	1.10	0.137	4.2E-01
Mtr.16139.1.S1_at	7.13	5.37	8.00	7.64	7.63	7.56	6.83	1.34	7.61	0.05	1.11	0.156	3.7E-01
Mtr.42296.1.S1_at	4.71	2.73	5.28	4.71	4.89	4.43	4.24	1.34	4.68	0.23	1.10	0.141	6.1E-01
Mtr.40851.1.S1_s_at	7.69	5.16	7.21	7.51	7.54	7.68	6.69	1.34	7.58	0.09	1.13	0.181	3.1E-01
Mtr.50907.1.S1_at	7.39	4.82	6.77	7.35	7.62	7.00	6.33	1.34	7.32	0.31	1.16	0.210	2.8E-01
Mtr.45025.1.S1_at	7.59	5.08	7.15	7.17	7.10	6.83	6.61	1.34	7.03	0.17	1.06	0.090	6.2E-01
Mtr.15316.1.S1_s_at	6.83	4.50	6.80	6.74	6.87	6.54	6.04	1.34	6.72	0.17	1.11	0.153	4.3E-01
Mtr.32414.1.S1_s_at	6.13	3.61	5.66	6.12	6.33	6.47	5.13	1.34	6.31	0.18	1.23	0.297	2.1E-01
Mtr.43498.1.S1_at	8.80	6.79	9.32	9.18	9.22	8.92	8.30	1.34	9.11	0.16	1.10	0.134	3.6E-01
Mtr.31940.1.S1_at	2.40	4.71	2.39	2.35	2.38	2.37	3.16	1.34	2.37	0.01	0.75	-0.420	3.6E-01
Mtr.19692.1.S1_at	8.38	5.98	8.21	7.90	8.32	8.03	7.53	1.34	8.08	0.21	1.07	0.103	5.2E-01
Mtr.37252.1.S1_at	12.49	10.81	9.85	11.60	11.29	11.93	11.05	1.34	11.61	0.32	1.05	0.071	5.2E-01
Mtr.7652.1.S1_at	3.19	5.40	2.98	3.19	3.18	3.19	3.86	1.34	3.19	0.00	0.83	-0.275	4.3E-01
Mtr.40482.1.S1_at	11.23	8.69	10.67	10.70	10.47	10.99	10.20	1.34	10.72	0.26	1.05	0.072	5.4E-01
Mtr.9838.1.S1_at	9.06	6.77	9.12	9.06	8.90	8.73	8.32	1.34	8.90	0.17	1.07	0.098	5.0E-01
Mtr.2024.1.S1_at	6.84	4.32	6.36	6.43	6.26	6.16	5.84	1.34	6.28	0.14	1.08	0.106	6.0E-01
Mtr.42306.1.S1_at	5.84	3.59	5.97	6.62	6.39	6.41	5.13	1.34	6.47	0.13	1.26	0.335	1.6E-01
Mtr.46862.1.S1_at	2.39	4.70	2.38	2.39	2.36	2.39	3.16	1.34	2.38	0.01	0.75	-0.408	3.7E-01
Mtr.18579.1.S1_at	12.60	9.94	11.46	12.34	12.04	12.40	11.33	1.34	12.26	0.19	1.08	0.114	3.0E-01
Mtr.7396.1.S1_at	7.29	4.95	7.24	7.58	7.30	7.62	6.49	1.34	7.50	0.18	1.15	0.208	2.7E-01
Mtr.15858.1.S1_at	2.26	4.57	2.25	2.25	2.24	2.25	3.02	1.34	2.25	0.00	0.74	-0.430	3.7E-01
Mtr.14762.1.S1_at	4.99	2.64	4.90	4.83	4.83	4.70	4.18	1.34	4.79	0.07	1.15	0.196	4.7E-01
Mtr.31761.1.S1_at	6.21	3.72	5.80	5.84	5.88	6.31	5.25	1.34	6.01	0.26	1.15	0.196	3.9E-01
Mtr.32209.1.S1_at	3.79	2.30	4.96	7.64	7.48	7.71	3.68	1.33	7.61	0.11	2.07	1.047	7.1E-03
Mtr.18373.1.S1_at	2.91	5.21	2.89	2.93	2.86	2.85	3.67	1.33	2.88	0.04	0.78	-0.350	3.6E-01
Mtr.12682.1.S1_at	4.21	3.17	5.82	4.57	5.67	4.89	4.40	1.33	5.04	0.57	1.15	0.197	4.8E-01
Mtr.33387.1.S1_at	6.03	3.72	6.03	6.01	6.36	5.45	5.26	1.33	5.94	0.46	1.13	0.176	4.5E-01
Mtr.35315.1.S1_s_at	7.38	5.18	7.59	7.42	7.50	7.42	6.72	1.33	7.44	0.05	1.11	0.148	4.0E-01
Mtr.5654.1.S1_at	7.11	4.56	6.51	6.48	6.46	6.76	6.06	1.33	6.57	0.17	1.08	0.116	5.5E-01
Mtr.12542.1.S1_at	3.57	4.27	6.14	6.17	6.67	5.93	4.66	1.33	6.26	0.38	1.34	0.425	1.2E-01
Mtr.4295.1.S1_at	6.89	4.60	6.93	6.18	6.53	6.85	6.14	1.33	6.52	0.34	1.06	0.087	6.5E-01
Mtr.18494.1.S1_at	5.88	3.62	3.53	3.20	3.52	3.52	4.34	1.33	3.41	0.18	0.79	-0.347	3.0E-01
Mtr.11257.1.S1_at	2.43	4.73	2.41	2.40	2.39	2.37	3.19	1.33	2.39	0.02	0.75	-0.418	3.6E-01
Mtr.14120.1.S1_at	2.33	4.63	2.31	2.31	2.31	2.30	3.09	1.33	2.30	0.01	0.74	-0.425	3.6E-01
Mtr.5499.1.S1_at	6.13	5.04	7.69	6.84	6.97	6.76	6.28	1.33	6.86	0.11	1.09	0.126	5.0E-01
Mtr.5659.1.S1_at	6.43	3.89	5.84	5.94	6.06	5.71	5.39	1.33	5.90	0.18	1.10	0.131	5.4E-01
Mtr.15550.1.S1_at	8.48	5.95	7.93	8.22	8.15	8.28	7.46	1.33	8.22	0.07	1.10	0.140	3.8E-01
Mtr.7197.1.S1_at	7.99	6.03	8.57	8.42	8.03	8.13	7.53	1.33	8.19	0.20	1.09	0.122	4.4E-01
Mtr.40509.1.S1_at	11.71	9.10	10.83	11.72	11.85	12.04	10.55	1.33	11.87	0.16	1.13	0.171	1.6E-01
Mtr.15008.1.S1_at	6.59	4.57	7.08	6.72	6.67	6.78	6.08	1.33	6.72	0.05	1.11	0.145	4.5E-01
Mtr.31070.1.S1_at	4.19	6.40	4.01	3.69	3.89	3.99	4.87	1.33	3.85	0.15	0.79	-0.337	2.6E-01
Mtr.32677.1.S1_s_at	7.48	5.29	7.69	7.57	7.72	7.69	6.82	1.33	7.66	0.08	1.12	0.168	3.4E-01
Mtr.31146.1.S1_at	6.22	4.27	6.81	6.37	6.50	6.37	5.77	1.33	6.41	0.08	1.11	0.153	4.5E-01
Mtr.11672.1.S1_at	2.82	3.00	5.20	4.51	3.94	5.21	3.67	1.33	4.56	0.63	1.24	0.310	3.6E-01
Mtr.5678.1.S1_s_at	4.86	2.33	4.29	5.27	4.90	4.66	3.83	1.33	4.94	0.31	1.29	0.369	2.3E-01
Mtr.45681.1.S1_s_at	6.39	4.39	6.90	6.59	6.89	5.80	5.89	1.33	6.43	0.56	1.09	0.125	5.6E-01
Mtr.40966.1.S1_at	7.73	5.54	7.93	8.08	8.45	7.98	7.06	1.33	8.17	0.25	1.16	0.210	2.3E-01
Mtr.5107.1.S1_s_at	12.73	10.97	10.13	11.29	12.51	11.77	11.28	1.33	11.85	0.61	1.05	0.072	5.3E-01
Mtr.50058.1.S1_s_at	8.64	11.27	9.64	9.91	9.81	9.40	9.85	1.33	9.71	0.27	0.99	-0.021	8.7E-01
Mtr.29679.1.S1_at	2.08	4.37	2.07	2.07	2.07	2.07	2.84	1.33	2.07	0.00	0.73	-0.459	3.7E-01
Mtr.42223.1.S1_at	8.49	5.99	7.99	7.97	8.06	7.93	7.49	1.33	7.99	0.07	1.07	0.093	5.5E-01
Mtr.10164.1.S1_at	6.58	4.57	7.07	6.39	6.68	6.42	6.08	1.33	6.50	0.16	1.07	0.096	6.1E-01
Mtr.39943.1.S1_at	4.56	2.99	5.62	5.29	5.09	4.74	4.39	1.32	5.04	0.28	1.15	0.200	4.5E-01
Mtr.19227.1.S1_at	5.00	2.98	5.48	4.91	5.13	5.24	4.48	1.32	5.09	0.17	1.14	0.183	4.7E-01
Mtr.34762.1.S1_s_at	8.67	6.11	7.95	8.36	7.89	8.43	7.58	1.32	8.23	0.30	1.09	0.119	4.5E-01
Mtr.34893.1.S1_at	6.71	4.30	6.44	6.59	6.53	6.54	5.82	1.32	6.55	0.03	1.13	0.171	3.9E-01
Mtr.41482.1.S1_s_at	5.29	2.93	5.13	5.23	5.45	5.36	4.45	1.32	5.34	0.11	1.20	0.264	3.1E-01
Mtr.30261.1.S1_at	6.16	3.98	6.37	5.68	6.68	5.76	5.50	1.32	6.04	0.56	1.10	0.135	5.5E-01
Mtr.7144.1.S1_at	6.77	4.65	7.08	6.83	8.11	7.36	6.17	1.32	7.43	0.64	1.20	0.269	2.1E-01
Mtr.14269.1.S1_at	6.39	3.78	5.41	6.78	6.76	6.74	5.19	1.32	6.76	0.02	1.30	0.381	1.1E-01
Mtr.18461.1.S1_s_at	7.85	5.23	6.84	7.18	6.96	7.31	6.64	1.32	7.15	0.18	1.08	0.106	5.5E-01
Mtr.21404.1.S1_at	2.42	4.70	2.41	2.39	2.38	2.37	3.17	1.32	2.38	0.01	0.75	-0.416	3.6E-01
Mtr.28432.1.S1_at	8.80	6.37	8.49	8.22	7.98	8.00	7.89	1.32	8.07	0.13	1.02	0.032	8.3E-01
Mtr.2284.1.S1_at	3.76	6.32	4.47	6.74	6.80	6.59	4.85	1.32	6.71	0.11	1.38	0.468	7.2E-02
Mtr.28150.1.S1_at	6.22	4.23	6.73	5.96	5.76	6.00	5.73	1.32	5.91	0.13	1.03	0.044	8.3E-01
Mtr.34909.1.S1_at	4.67	2.32	4.53	4.69	4.75	4.66	3.84	1.32	4.70	0.04	1.22	0.292	3.2E-01
Mtr.6858.1.S1_at	6.54	4.31	4.19	3.58	4.19	4.17	5.01	1.32	3.98	0.35	0.79	-0.334	2.6E-01
Mtr.39979.1.S1_x_at	5.91	3.78	6.20	6.12	5.94	6.00	5.30	1.32	6.02	0.09	1.14	0.184	4.0E-01
Mtr.27813.1.S1_at	4.05	4.05	6.34	3.50	3.90	3.10	4.81	1.32	3.50	0.40	0.73	-0.460	1.7E-01
Mtr.28732.1.S1_at	4.98	2.36	3.36	4.13	5.77	4.46	3.57	1.32	4.79	0.87	1.34	0.425	2.5E-01
Mtr.2678.1.S1_at	6.11	4.11	6.60	6.33	6.22	5.67	5.61	1.32	6.07	0.35	1.08	0.115	5.9E-01
Mtr.844.1.S1_at	8.14	5.53	7.15	7.45	7.07	7.50	6.94	1.32	7.34	0.24	1.06	0.081	6.3E-01
Mtr.26260.1.S1_at	3.56	5.84	3.55	3.55	3.55	3.55	4.32	1.32	3.55	0.00	0.82	-0.284	3.7E-01
Mtr.46540.1.S1_at	6.39	3.96	6.06	4.48	4.47	3.85	5.47	1.32	4.27	0.37	0.78	-0.357	2.0E-01
Mtr.1243.1.S1_at	2.77	5.03	2.73	2.85	2.63	2.87	3.51	1.32	2.78	0.14	0.79	-0.336	3.9E-01
Mtr.31975.1.S1_at	2.22	4.49	2.20	2.20	2.20	2.20	2.97	1.32	2.20	0.00	0.74	-0.433	3.7E-01
Mtr.27737.1.S1_at	7.35	5.02	7.24	7.25	6.99	7.15	6.54	1.32	7.13	0.13	1.09	0.125	4.8E-01
Mtr.31130.1.S1_x_at	2.71	4.91	2.54	2.62	2.54	2.54	3.39	1.32	2.56	0.05	0.76	-0.401	3.4E-01
Mtr.31011.1.S1_at	7.70	5.55	7.94	7.55	7.98	7.40	7.06	1.32	7.64	0.30	1.08	0.114	5.0E-01
Mtr.43900.1.S1_at	10.55	7.92	9.40	9.70	9.99	10.02	9.29	1.32	9.90	0.17	1.07	0.092	4.7E-01
Mtr.52064.1.S1_at	7.29	5.00	7.26	7.29	7.30	7.52	6.52	1.32	7.37	0.13	1.13	0.178	3.3E-01
Mtr.12867.1.S1_s_at	6.67	4.54	6.95	7.73	7.51	7.89	6.05	1.32	7.71	0.19	1.27	0.350	9.7E-02
Mtr.31137.1.S1_at	6.87	5.17	7.76	6.96	7.28	7.14	6.60	1.32	7.13	0.16	1.08	0.111	5.3E-01
Mtr.14949.1.S1_s_at	8.07	7.18	9.77	7.98	8.78	8.01	8.34	1.32	8.25	0.45	0.99	-0.015	9.2E-01
Mtr.38534.1.S1_at	7.36	5.01	7.21	6.81	7.14	7.10	6.53	1.32	7.02	0.18	1.08	0.105	5.6E-01
Mtr.10896.1.S1_s_at	8.20	6.09	8.50	8.77	9.09	8.98	7.60	1.32	8.94	0.16	1.18	0.236	1.5E-01
Mtr.43143.1.S1_at	7.38	4.87	6.82	8.25	8.90	8.76	6.36	1.32	8.64	0.34	1.36	0.442	4.4E-02
Mtr.49461.1.S1_at	5.98	3.63	5.84	5.64	5.16	5.47	5.15	1.32	5.42	0.24	1.05	0.074	7.4E-01
Mtr.16733.1.S1_s_at	5.45	4.18	6.81	6.38	6.74	6.67	5.48	1.32	6.60	0.19	1.20	0.268	2.2E-01
Mtr.1042.1.S1_at	6.84	4.86	7.36	7.64	7.67	7.55	6.35	1.32	7.62	0.06	1.20	0.262	1.7E-01
Mtr.8117.1.S1_at	2.48	4.74	2.45	2.67	2.50	2.50	3.22	1.32	2.56	0.09	0.79	-0.333	4.3E-01
Mtr.9242.1.S1_at	7.50	5.34	7.72	7.61	7.53	7.69	6.85	1.31	7.61	0.08	1.11	0.151	3.8E-01
Mtr.48743.1.S1_at	7.87	6.55	5.24	3.74	5.16	5.00	6.55	1.31	4.63	0.78	0.71	-0.500	9.6E-02
Mtr.12120.1.S1_at	8.94	7.09	9.63	9.39	9.39	9.26	8.55	1.31	9.34	0.08	1.09	0.128	3.6E-01
Mtr.9220.1.S1_at	7.13	5.59	8.20	7.00	6.95	6.52	6.97	1.31	6.82	0.26	0.98	-0.031	8.6E-01
Mtr.46609.1.S1_at	2.52	4.79	2.50	2.49	2.50	2.49	3.27	1.31	2.49	0.01	0.76	-0.391	3.6E-01
Mtr.22047.1.S1_at	4.48	6.52	4.07	5.22	4.45	4.55	5.02	1.31	4.74	0.42	0.94	-0.083	7.4E-01
Mtr.42626.1.S1_at	7.07	4.49	6.20	6.69	7.46	7.21	5.92	1.31	7.12	0.39	1.20	0.267	2.0E-01
Mtr.38110.1.S1_at	4.68	4.22	6.69	8.83	10.98	10.21	5.20	1.31	10.01	1.09	1.93	0.946	8.1E-03
Mtr.34910.1.S1_at	7.20	4.93	7.21	7.03	6.97	7.14	6.45	1.31	7.05	0.09	1.09	0.129	4.7E-01

Mtr.44465.1.S1_at	8.62	6.05	6.85	7.57	7.29	7.47	7.17	1.31	7.44	0.14	1.04	0.054	7.4E-01
Mtr.41164.1.S1_at	8.28	5.67	6.75	5.34	5.34	5.05	6.90	1.31	5.24	0.17	0.76	-0.397	9.5E-02
Mtr.38723.1.S1_at	11.97	10.27	12.85	12.62	12.76	12.70	11.70	1.31	12.69	0.07	1.09	0.118	2.6E-01
Mtr.44760.1.S1_at	6.57	4.44	6.83	6.45	6.55	6.30	5.94	1.31	6.43	0.12	1.08	0.114	5.5E-01
Mtr.28494.1.S1_at	7.06	4.56	6.50	6.79	6.62	6.52	6.04	1.31	6.64	0.14	1.10	0.136	4.8E-01
Mtr.5287.1.S1_at	7.26	5.23	7.69	7.54	7.62	7.52	6.73	1.31	7.56	0.05	1.12	0.168	3.3E-01
Mtr.1717.1.S1_at	5.80	3.38	5.48	5.79	6.35	5.89	4.89	1.31	6.01	0.30	1.23	0.298	2.2E-01
Mtr.5345.1.S1_at	7.51	5.24	7.51	7.58	7.59	7.75	6.75	1.31	7.64	0.10	1.13	0.179	3.1E-01
Mtr.27879.1.S1_at	9.45	6.86	8.49	9.24	9.15	9.13	8.27	1.31	9.17	0.06	1.11	0.150	3.0E-01
Mtr.15098.1.S1_at	2.69	4.93	2.64	2.62	2.61	2.58	3.42	1.31	2.60	0.02	0.76	-0.393	3.4E-01
Mtr.26951.1.S1_at	8.76	6.30	8.31	8.46	8.49	8.61	7.79	1.31	8.52	0.08	1.09	0.129	3.9E-01
Mtr.19989.1.S1_at	2.47	4.71	2.41	2.40	2.37	2.41	3.19	1.31	2.39	0.02	0.75	-0.417	3.5E-01
Mtr.5590.1.S1_at	5.72	3.49	5.79	5.74	6.12	5.64	5.00	1.31	5.83	0.25	1.17	0.222	3.4E-01
Mtr.41029.1.S1_at	7.27	4.93	7.11	7.30	7.09	6.93	6.44	1.31	7.11	0.19	1.10	0.143	4.3E-01
Mtr.5633.1.S1_at	10.56	8.14	8.49	8.96	8.24	8.78	9.07	1.31	8.66	0.37	0.96	-0.066	6.3E-01
Mtr.30958.1.S1_at	8.31	6.05	8.31	8.20	7.69	7.90	7.56	1.31	7.93	0.26	1.05	0.070	6.5E-01
Mtr.10869.1.S1_at	3.32	5.32	2.85	2.91	2.82	2.81	3.83	1.31	2.85	0.06	0.74	-0.427	2.6E-01
Mtr.37995.1.S1_at	7.67	5.49	7.84	7.39	7.28	7.36	7.00	1.31	7.34	0.06	1.05	0.069	6.7E-01
Mtr.24547.1.S1_s_at	7.88	5.60	7.86	7.76	7.79	7.71	7.11	1.31	7.75	0.04	1.09	0.124	4.5E-01
Mtr.28272.1.S1_at	4.46	2.97	5.58	5.50	5.36	4.25	4.34	1.31	5.04	0.69	1.16	0.216	4.6E-01
Mtr.48379.1.S1_at	9.14	9.31	6.96	6.95	7.04	7.22	8.47	1.31	7.07	0.14	0.83	-0.261	1.4E-01
Mtr.44978.1.S1_at	5.18	3.24	5.73	5.24	5.19	4.61	4.72	1.31	5.01	0.35	1.06	0.089	7.2E-01
Mtr.46120.1.S1_at	2.39	4.63	2.35	2.40	2.40	2.39	3.12	1.31	2.40	0.01	0.77	-0.381	3.9E-01
Mtr.33355.1.S1_s_at	7.94	5.55	7.67	7.70	7.88	8.00	7.06	1.31	7.86	0.15	1.11	0.156	3.5E-01
Mtr.19664.1.S1_at	2.01	4.26	1.99	1.99	1.99	1.99	2.75	1.31	1.99	0.00	0.72	-0.470	3.7E-01
Mtr.39116.1.S1_at	7.99	5.57	7.65	7.55	7.51	7.46	7.07	1.31	7.51	0.05	1.06	0.087	5.9E-01
Mtr.13007.1.S1_at	7.75	5.31	7.34	7.20	7.46	7.47	6.80	1.31	7.38	0.15	1.08	0.118	4.9E-01
Mtr.31754.1.S1_at	4.90	2.80	5.20	4.97	5.54	4.98	4.30	1.31	5.16	0.33	1.20	0.264	3.3E-01
Mtr.20457.1.S1_s_at	2.63	4.89	2.62	2.59	2.59	2.58	3.38	1.31	2.59	0.01	0.77	-0.384	3.5E-01
Mtr.7366.1.S1_at	6.71	4.26	6.26	6.31	6.32	5.74	5.74	1.31	6.12	0.33	1.07	0.092	6.5E-01
Mtr.27647.1.S1_at	5.91	4.41	7.01	6.14	6.06	6.16	5.78	1.31	6.12	0.05	1.06	0.084	6.7E-01
Mtr.41980.1.S1_at	9.07	7.02	9.45	9.72	9.62	9.73	8.52	1.31	9.69	0.06	1.14	0.186	2.0E-01
Mtr.26958.1.S1_at	7.55	5.34	7.65	6.89	7.45	7.30	6.85	1.31	7.21	0.29	1.05	0.075	6.6E-01
Mtr.40706.1.S1_s_at	6.57	3.98	5.01	5.10	4.90	4.90	5.19	1.31	4.97	0.12	0.96	-0.063	7.8E-01
Mtr.33173.1.S1_at	7.41	5.31	7.70	7.56	7.44	7.78	6.81	1.31	7.59	0.17	1.12	0.158	3.6E-01
Mtr.24959.1.S1_s_at	2.27	4.52	2.25	2.25	2.25	2.24	3.01	1.31	2.24	0.00	0.74	-0.426	3.6E-01
Mtr.39832.1.S1_at	6.45	3.85	4.99	5.02	5.44	5.30	5.10	1.31	5.25	0.21	1.03	0.043	8.5E-01
Mtr.6904.1.S1_at	7.14	4.75	6.86	6.93	6.97	7.09	6.25	1.31	7.00	0.09	1.12	0.162	3.8E-01
Mtr.42722.1.S1_at	7.34	5.31	7.75	7.19	7.23	7.00	6.80	1.30	7.14	0.12	1.05	0.071	6.7E-01
Mtr.46071.1.S1_at	2.40	4.65	2.38	2.36	2.46	2.35	3.14	1.30	2.39	0.06	0.76	-0.393	3.8E-01
Mtr.13941.1.S1_at	7.32	5.04	7.27	7.55	7.39	7.31	6.54	1.30	7.42	0.13	1.13	0.181	3.1E-01
Mtr.45689.1.S1_at	3.03	5.26	2.98	2.97	2.92	2.88	3.76	1.30	2.92	0.05	0.78	-0.363	3.3E-01
Mtr.51092.1.S1_x_at	3.30	5.53	3.25	3.20	3.25	3.25	4.03	1.30	3.23	0.03	0.80	-0.317	3.5E-01
Mtr.19116.1.S1_at	2.89	5.14	2.87	2.82	2.78	2.76	3.63	1.30	2.78	0.03	0.77	-0.383	3.2E-01
Mtr.33224.1.S1_at	5.89	7.81	5.32	6.03	5.90	5.95	6.34	1.30	5.96	0.06	0.94	-0.089	6.4E-01
Mtr.32158.1.S1_at	7.91	5.36	7.14	7.38	7.64	7.77	6.80	1.30	7.60	0.20	1.12	0.159	3.6E-01
Mtr.16024.1.S1_at	8.83	6.55	8.79	8.75	8.47	8.72	8.06	1.30	8.65	0.15	1.07	0.102	4.8E-01
Mtr.20247.1.S1_s_at	6.51	4.39	6.76	6.78	7.12	6.86	5.88	1.30	6.92	0.18	1.18	0.233	2.5E-01
Mtr.35300.1.S1_at	6.61	4.58	7.01	6.03	5.53	5.04	6.07	1.30	5.54	0.49	0.91	-0.132	5.4E-01
Mtr.7595.1.S1_at	10.68	8.38	10.59	10.70	10.60	10.55	9.89	1.30	10.62	0.08	1.07	0.103	3.9E-01
Mtr.15396.1.S1_at	6.96	4.49	6.44	6.62	6.71	6.60	5.96	1.30	6.64	0.06	1.11	0.156	4.2E-01
Mtr.12950.1.S1_at	5.02	7.18	4.84	5.89	6.37	6.65	5.68	1.30	6.30	0.39	1.11	0.151	4.7E-01
Mtr.50550.1.S1_at	3.69	5.97	3.74	3.87	3.80	3.97	4.47	1.30	3.88	0.09	0.87	-0.203	4.8E-01
Mtr.11621.1.S1_at	6.47	3.88	4.95	5.74	5.19	6.03	5.10	1.30	5.65	0.42	1.11	0.148	5.2E-01
Mtr.42702.1.S1_at	6.86	4.55	6.75	7.35	7.03	7.18	6.05	1.30	7.19	0.16	1.19	0.248	2.1E-01
Mtr.46619.1.S1_x_at	8.08	5.57	7.42	7.21	7.27	7.16	7.02	1.30	7.21	0.06	1.03	0.038	8.1E-01
Mtr.11222.1.S1_at	8.17	5.72	7.70	7.68	7.44	7.60	7.20	1.30	7.57	0.12	1.05	0.073	6.5E-01
Mtr.14463.1.S1_s_at	7.45	4.99	6.94	6.92	7.17	6.86	6.46	1.30	6.98	0.17	1.08	0.112	5.3E-01
Mtr.6127.1.S1_s_at	8.10	5.64	7.59	8.10	8.38	8.24	7.11	1.30	8.24	0.14	1.16	0.212	2.1E-01
Mtr.3071.1.S1_at	6.22	4.01	6.31	5.66	6.24	6.29	5.51	1.30	6.06	0.35	1.10	0.138	5.2E-01
Mtr.21761.1.S1_at	7.27	4.92	7.07	7.43	7.30	7.34	6.42	1.30	7.36	0.07	1.15	0.196	2.8E-01
Mtr.46720.1.S1_at	5.90	3.43	3.96	5.53	4.89	5.59	4.43	1.30	5.34	0.39	1.20	0.269	3.1E-01
Mtr.2448.1.S1_at	2.42	4.66	2.39	2.38	2.38	2.38	3.15	1.30	2.38	0.00	0.76	-0.405	3.6E-01
Mtr.31018.1.S1_at	6.88	4.53	6.66	6.61	7.22	6.83	6.02	1.30	6.88	0.31	1.14	0.192	3.3E-01
Mtr.32972.1.S1_at	5.33	3.41	5.88	5.79	5.96	4.82	4.88	1.30	5.52	0.62	1.13	0.180	4.8E-01
Mtr.32197.1.S1_at	6.43	3.94	5.82	5.59	5.65	5.81	5.40	1.30	5.68	0.12	1.05	0.075	7.2E-01
Mtr.5442.1.S1_at	5.97	3.57	5.64	6.02	5.83	6.04	5.06	1.30	5.96	0.12	1.18	0.237	3.0E-01
Mtr.36332.1.S1_at	3.31	5.65	3.50	3.20	3.20	3.14	4.15	1.30	3.18	0.04	0.77	-0.385	2.6E-01
Mtr.3125.1.S1_at	5.43	3.35	5.74	5.70	6.24	6.25	4.84	1.30	6.06	0.31	1.25	0.325	1.9E-01
Mtr.13664.1.S1_s_at	6.16	3.86	6.07	6.85	6.57	6.23	5.36	1.30	6.55	0.31	1.22	0.288	2.0E-01
Mtr.45110.1.S1_at	5.43	3.02	5.07	5.06	5.21	5.30	4.51	1.30	5.19	0.12	1.15	0.203	4.2E-01
Mtr.396.1.S1_at	2.88	5.12	2.86	2.78	2.76	2.79	3.62	1.30	2.78	0.01	0.77	-0.382	3.2E-01
Mtr.42081.1.S1_at	4.36	2.08	4.29	5.78	5.00	6.15	3.58	1.30	5.64	0.59	1.58	0.658	6.6E-02
Mtr.4983.1.S1_at	9.80	7.73	7.41	8.46	7.70	8.47	8.32	1.30	8.21	0.44	0.99	-0.018	9.0E-01
Mtr.1554.1.S1_at	7.33	4.93	6.99	7.21	7.31	7.15	6.42	1.30	7.22	0.08	1.13	0.170	3.4E-01
Mtr.38344.1.S1_at	7.24	4.65	5.88	5.65	5.52	5.88	5.92	1.30	5.68	0.18	0.96	-0.059	7.7E-01
Mtr.14899.1.S1_s_at	5.07	3.01	5.40	4.79	4.92	4.81	4.49	1.30	4.84	0.07	1.08	0.108	6.7E-01
Mtr.32546.1.S1_at	5.43	3.50	5.97	5.08	5.76	5.06	4.97	1.30	5.30	0.40	1.07	0.094	6.9E-01
Mtr.27740.1.S1_at	7.26	5.72	8.29	7.88	7.87	7.47	7.09	1.30	7.74	0.23	1.09	0.126	4.4E-01
Mtr.29649.1.S1_at	2.20	4.44	2.18	2.20	2.18	2.18	2.94	1.30	2.19	0.01	0.74	-0.428	3.7E-01
Mtr.1471.1.S1_at	2.65	4.88	2.62	2.59	2.60	2.62	3.38	1.30	2.60	0.02	0.77	-0.378	3.6E-01
Mtr.50885.1.S1_at	5.24	3.00	5.26	5.23	5.23	5.23	4.50	1.30	5.23	0.00	1.16	0.216	3.9E-01
Mtr.26602.1.S1_at	2.03	4.25	1.98	1.98	1.95	1.97	2.75	1.30	1.97	0.01	0.71	-0.485	3.5E-01
Mtr.32802.1.S1_at	4.46	3.61	6.16	5.23	4.70	4.82	4.74	1.30	4.92	0.28	1.04	0.052	8.3E-01
Mtr.37403.1.S1_at	7.15	5.57	8.14	8.77	9.32	9.27	6.95	1.29	9.12	0.30	1.31	0.392	4.8E-02
Mtr.44552.1.S1_at	7.51	5.26	7.49	7.75	7.66	7.54	6.75	1.29	7.65	0.10	1.13	0.180	3.0E-01
Mtr.7495.1.S1_at	7.21	5.17	7.56	7.14	7.06	7.19	6.64	1.29	7.13	0.06	1.07	0.101	5.5E-01
Mtr.2953.1.S1_at	4.89	3.38	5.95	5.06	5.33	4.76	4.74	1.29	5.05	0.29	1.07	0.091	7.1E-01
Mtr.25317.1.S1_at	3.79	6.01	3.77	3.75	3.47	3.73	4.52	1.29	3.65	0.16	0.81	-0.309	3.1E-01
Mtr.11807.1.S1_at	10.23	8.10	10.44	10.58	10.43	10.44	9.59	1.29	10.48	0.08	1.09	0.129	3.0E-01
Mtr.6851.1.S1_at	8.03	5.79	8.02	7.87	7.94	7.85	7.28	1.29	7.89	0.04	1.08	0.116	4.6E-01
Mtr.25216.1.S1_at	2.92	5.14	2.89	2.88	2.89	2.88	3.65	1.29	2.88	0.00	0.79	-0.342	3.6E-01
Mtr.38333.1.S1_at	8.96	6.75	9.01	8.96	9.16	8.91	8.24	1.29	9.01	0.13	1.09	0.129	3.6E-01
Mtr.51431.1.S1_at	3.32	5.67	3.58	3.55	3.58	3.89	4.19	1.29	3.67	0.19	0.88	-0.189	5.3E-01
Mtr.14567.1.S1_s_at	5.94	3.74	3.68	4.99	4.94	5.67	4.45	1.29	5.20	0.41	1.17	0.224	3.9E-01
Mtr.27364.1.S1_at	8.04	5.79	8.02	7.73	7.68	7.81	7.28	1.29	7.74	0.07	1.06	0.088	5.7E-01
Mtr.2472.1.S1_s_at	7.28	5.38	7.85	7.24	6.88	6.96	6.84	1.29	7.03	0.19	1.03	0.039	8.1E-01
Mtr.4962.1.S1_at	6.47	4.59	7.07	6.58	6.49	6.14	6.04	1.29	6.40	0.23	1.06	0.082	6.6E-01
Mtr.49177.1.S1_at	3.18	5.40	3.15	3.14	3.14	3.36	3.91	1.29	3.21	0.13	0.82	-0.284	4.0E-01
Mtr.21639.1.S1_x_at	3.24	5.47	3.23	3.16	3.23	3.22	3.98	1.29	3.20	0.04	0.80	-0.314	3.5E-01
Mtr.10869.1.S1_x_at	2.60	4.78	2.50	2.50	2.49	2.48	3.30	1.29	2.49	0.01	0.76	-0.403	3.4E-01
Mtr.28801.1.S1_at	4.27	5.66	6.85	7.91	7.59	7.82	5.59	1.29	7.77	0.17	1.39	0.475	4.4E-02
Mtr.13198.1.S1_at	6.28	3.77	5.56	6.15	6.67	6.52	5.20	1.29	6.45	0.27	1.24	0.309	1.8E-01
Mtr.47577.1.S1_at	4.57	6.88	4.75	4.74	4.64	4.89	5.40	1.29	4.76	0.13	0.88	-0.182	4.4E-01
Mtr.19643.1.S1_at	6.77	4.74	7.14	6.83	6.79	6.65	6.22	1.29	6.76	0.10	1.09	0.120	5.1E-01
Mtr.28685.1.S1_at	7.35	4.92	6.89	6.88	7.03	6.83	6.39	1.29	6.91	0.10	1.08	0.113	5.2E-01
Mtr.32807.1.S1_at	6.70	4.69	7.10	6.98	7.15	6.79	6.17	1.29	6.97	0.18	1.13	0.178	3.4E-01
Mtr.7638.1.S1_at	12.21	10.31	9.75	10.10	9.98	10.40	10.76	1.29	10.16	0.21	0.94	-0.083	4.7E-01
Mtr.1406.1.S1_at	8.06	5.65	7.66	7.61	7.74	7.73	7.12	1.29	7.69	0.07	1.08	0.111	4.9E-01
Mtr.6925.1.S1_at	6.84	4.49	6.58	6.82	6.55	6.84	5.97	1.29	6.74	0.16	1.13	0.174	3.6E-01
Mtr.40498.1.S1_at	11.19	8.66	9.49	10.22	10.51	10.30	9.78	1.29	10.34	0.15	1.06	0.081	4.9E-01
Mtr.31872.1.S1_at	9.57	7.01	8.52	8.66	8.82	8.88	8.37	1.29	8.79	0.11	1.05	0.070	6.1E-01
Mtr.11664.1.S1_at	8.60	6.24	6.54	8.01	7.36	8.15	7.13	1.29	7.84	0.42	1.10	0.138	4.1E-01
Mtr.38036.1.S1_at	6.14	3.65	4.33	4.24	4.77	3.81	4.70	1.29	4.28	0.48	0.91	-0.138	6.2E-01
Mtr.13498.1.S1_at	7.45	5.07	7.12	7.87	8.40	8.27	6.54	1.29	8.18	0.27	1.25	0.322	9.7E-02
Mtr.47320.1.S1_at	2.93	5.08	2.78	2.89	2.72	2.89	3.60	1.29	2.84	0.10	0.79	-0.343	3.6E-01
Mtr.7618.1.S1_at	4.48	6.38	3.93	4.22	4.25	4.55	4.93	1.29	4.34	0.18	0.88	-0.185	4.7E-01
Mtr.29843.1.S1_s_at	9.53	7.36	9.63	9.81	9.54	10.07	8.84	1.29	9.80	0.26	1.11	0.149	2.7E-01
Mtr.22068.1.S1_at	6.89	6.62	8.97	7.86	8.41	8.25	7.49	1.29	8.17	0.28	1.09	0.126	4.2E-01
Mtr.33486.1.S1_at	7.11	4.86	7.05	7.11	7.20	6.76	6.34	1.29	7.02	0.24	1.11	0.148	4.2E-01
Mtr.12672.1.S1_at	7.28	5.01	7.18	6.89	6.83	7.12	6.49	1.28	6.95	0.15	1.07	0.099	5.7E-01
Mtr.38987.1.S1_at	7.83	5.37	7.26	7.63	7.59	7.62	6.82	1.28	7.61	0.02	1.12	0.159	3.4E-01
Mtr.37366.1.S1_at	8.95	6.63	8.74	8.65	8.59	8.62	8.11	1.28	8.62	0.03	1.06	0.089	5.3E-01
Mtr.2954.1.S1_s_at	8.98	6.64	8.74	8.06	8.45	8.30	8.12	1.28	8.27	0.20	1.02	0.027	8.5E-01
Mtr.103.1.S1_at	2.92	5.13	2.89	2.89	2.88	2.89	3.65	1.28	2.89	0.01	0.79	-0.337	3.6E-01
Mtr.41295.1.S1_at	8.53	6.12	8.09	8.38	8.41	8.54	7.58	1.28	8.44	0.08	1.11	0.155	3.1E-01
Mtr.31042.1.S1_at	6.20	3.75	5.64	5.63	6.50	6.16	5.20	1.28	6.10	0.44	1.17	0.231	3.1E-01
Mtr.46627.1.S1_at	12.46	14.63	12.36	12.82	12.13	12.28	13.15	1.28	12.41	0.36	0.94	-0.083	3.9E-01
Mtr.18598.1.S1_at	2.98	5.18	2.93	2.92	2.85	2.84	3.70	1.28	2.87	0.04	0.78	-0.366	3.3E-01
Mtr.11203.1.S1_at	8.89	6.89	6.50	7.17	6.52	7.31	7.43	1.28	7.00	0.42	0.94	-0.085	6.2E-01
Mtr.44687.1.S1_at	5.41	3.17	5.37	5.50	5.54	5.28	4.65	1.28	5.44	0.14	1.17	0.227	3.5E-01

Mtr.41634.1.S1_at	6.41	4.51	6.95	7.36	6.99	7.13	5.96	1.28	7.16	0.19	1.20	0.266	1.8E-01
Mtr.6666.1.S1_at	5.68	4.37	6.94	6.37	7.52	6.54	5.66	1.28	6.81	0.62	1.20	0.266	2.4E-01
Mtr.40252.1.S1_at	10.43	8.09	8.34	8.89	9.31	9.26	8.96	1.28	9.15	0.22	1.02	0.031	8.1E-01
Mtr.1358.1.S1_at	2.29	4.51	2.28	2.28	2.27	2.35	3.03	1.28	2.30	0.04	0.76	-0.396	3.8E-01
Mtr.11833.1.S1_at	8.19	5.63	7.05	7.35	7.09	7.83	6.96	1.28	7.42	0.38	1.07	0.093	5.8E-01
Mtr.43994.1.S1_at	7.56	5.62	8.04	7.59	7.39	7.70	7.07	1.28	7.56	0.16	1.07	0.096	5.5E-01
Mtr.22598.1.S1_at	7.84	5.71	8.00	7.68	8.17	7.78	7.18	1.28	7.88	0.26	1.10	0.133	4.1E-01
Mtr.13843.1.S1_at	6.76	4.59	6.86	6.53	6.67	6.46	6.07	1.28	6.55	0.11	1.08	0.110	5.5E-01
Mtr.337.1.S1_x_at	2.52	4.73	2.51	2.49	2.48	2.47	3.25	1.28	2.48	0.01	0.76	-0.392	3.5E-01
Mtr.29209.1.S1_at	4.67	2.20	4.00	4.11	4.14	4.06	3.62	1.28	4.11	0.04	1.13	0.180	5.5E-01
Mtr.20205.1.S1_at	3.37	5.54	3.28	3.25	3.18	3.27	4.06	1.28	3.23	0.05	0.80	-0.330	3.2E-01
Mtr.27051.1.S1_at	2.26	4.49	2.28	2.29	2.28	2.30	3.01	1.28	2.29	0.01	0.76	-0.397	3.8E-01
Mtr.3224.1.S1_at	7.38	5.89	8.43	7.84	8.14	7.46	7.23	1.28	7.81	0.34	1.08	0.112	4.9E-01
Mtr.7604.1.S1_at	4.85	2.68	2.59	2.51	2.58	2.57	3.37	1.28	2.55	0.04	0.76	-0.400	3.3E-01
Mtr.36769.1.S1_at	2.65	4.86	2.64	2.65	2.63	2.60	3.39	1.28	2.63	0.02	0.78	-0.364	3.6E-01
Mtr.24210.1.S1_at	5.98	4.93	7.47	6.34	5.63	5.85	6.13	1.28	5.94	0.36	0.97	-0.045	8.2E-01
Mtr.28803.1.S1_s_at	4.61	6.42	7.08	6.47	7.37	7.22	6.04	1.28	7.02	0.48	1.16	0.218	2.8E-01
Mtr.9728.1.S1_at	10.46	8.79	7.95	9.70	9.28	10.09	9.07	1.28	9.69	0.40	1.07	0.096	4.7E-01
Mtr.44400.1.S1_at	6.15	3.81	5.88	5.89	5.89	6.24	5.28	1.28	6.01	0.20	1.14	0.186	3.9E-01
Mtr.42224.1.S1_s_at	8.02	5.68	7.74	8.43	9.08	8.65	7.14	1.28	8.72	0.33	1.22	0.287	1.1E-01
Mtr.27954.1.S1_at	6.37	4.49	6.93	6.40	6.62	5.63	5.93	1.28	6.22	0.52	1.05	0.068	7.4E-01
Mtr.6874.1.S1_at	4.48	2.23	4.42	4.46	4.37	4.37	3.71	1.28	4.40	0.05	1.19	0.247	4.0E-01
Mtr.8651.1.S1_a_at	2.82	5.01	2.78	3.33	4.57	2.77	3.53	1.28	3.56	0.92	1.01	0.009	9.8E-01
Mtr.46748.1.S1_at	5.60	3.20	5.16	5.26	5.26	5.55	4.65	1.28	5.36	0.17	1.15	0.203	4.0E-01
Mtr.42464.1.S1_at	6.93	4.79	7.07	6.92	7.68	7.12	6.26	1.28	7.24	0.40	1.16	0.209	2.8E-01
Mtr.24634.1.S1_at	3.03	5.10	2.77	2.75	2.72	2.70	3.63	1.28	2.72	0.03	0.75	-0.414	2.9E-01
Mtr.21485.1.S1_at	2.49	4.79	2.68	2.48	2.48	2.48	3.32	1.28	2.48	0.00	0.75	-0.422	3.2E-01
Mtr.14231.1.S1_at	8.28	5.90	7.89	8.44	8.27	8.28	7.36	1.28	8.33	0.09	1.13	0.179	2.6E-01
Mtr.45224.1.S1_at	4.92	2.65	4.80	4.13	5.03	5.57	4.12	1.28	4.91	0.73	1.19	0.253	4.0E-01
Mtr.28239.1.S1_at	7.31	5.26	7.59	7.89	8.17	7.64	6.72	1.28	7.90	0.27	1.18	0.234	1.9E-01
Mtr.29525.1.S1_at	8.54	6.13	8.07	7.82	7.87	7.68	7.58	1.28	7.79	0.10	1.03	0.039	7.9E-01
Mtr.24222.1.S1_at	4.69	2.83	5.28	3.23	3.27	3.51	4.27	1.28	3.34	0.15	0.78	-0.355	2.8E-01
Mtr.41653.1.S1_at	7.43	5.01	5.53	5.26	4.84	5.62	5.99	1.28	5.24	0.39	0.88	-0.192	3.9E-01
Mtr.25914.1.S1_at	3.58	5.75	5.82	7.65	7.81	7.65	5.05	1.27	7.70	0.09	1.52	0.608	2.3E-02
Mtr.1828.1.S1_at	9.13	6.90	9.09	9.37	9.28	9.01	8.37	1.27	9.22	0.18	1.10	0.139	3.2E-01
Mtr.7520.1.S1_at	10.21	7.81	9.76	9.83	9.65	9.81	9.26	1.27	9.76	0.10	1.05	0.076	5.3E-01
Mtr.13764.1.S1_at	5.09	2.68	4.60	5.64	6.04	5.08	4.13	1.27	5.59	0.49	1.35	0.437	1.4E-01
Mtr.11916.1.S1_s_at	7.83	5.67	7.92	6.65	6.77	7.17	7.14	1.27	6.86	0.27	0.96	-0.057	7.3E-01
Mtr.1468.1.S1_at	8.01	5.56	7.38	7.94	8.37	8.07	6.98	1.27	8.12	0.22	1.16	0.218	2.0E-01
Mtr.29901.1.S1_at	3.13	5.33	3.11	3.12	3.10	3.12	3.86	1.27	3.11	0.01	0.81	-0.310	3.7E-01
Mtr.15029.1.S1_s_at	3.08	2.32	4.80	3.20	3.05	2.85	3.40	1.27	3.03	0.17	0.89	-0.165	6.5E-01
Mtr.30257.1.S1_at	7.58	5.04	6.39	6.29	7.32	6.97	6.33	1.27	6.86	0.52	1.08	0.115	5.5E-01
Mtr.28552.1.S1_at	8.30	5.98	8.04	8.26	8.09	7.91	7.44	1.27	8.09	0.17	1.09	0.121	4.3E-01
Mtr.27629.1.S1_s_at	7.07	4.88	7.09	7.24	7.03	6.96	6.35	1.27	7.08	0.15	1.12	0.157	3.8E-01
Mtr.28474.1.S1_at	5.96	4.05	6.46	5.76	5.24	5.68	5.49	1.27	5.56	0.28	1.01	0.018	9.3E-01
Mtr.31772.1.S1_s_at	2.87	5.00	2.73	3.23	2.72	2.78	3.54	1.27	2.91	0.28	0.82	-0.281	4.5E-01
Mtr.32274.1.S1_s_at	7.36	4.99	6.99	6.97	6.95	6.91	6.44	1.27	6.94	0.03	1.08	0.108	5.3E-01
Mtr.51483.1.S1_x_at	8.25	6.01	8.16	9.11	8.64	8.54	7.47	1.27	8.77	0.30	1.17	0.230	1.6E-01
Mtr.7123.1.S1_at	4.25	6.52	4.40	4.34	4.16	4.47	5.06	1.27	4.33	0.15	0.86	-0.225	3.8E-01
Mtr.40733.1.S1_at	9.71	7.56	9.81	10.40	9.66	10.41	9.03	1.27	10.15	0.43	1.13	0.170	2.2E-01
Mtr.44800.1.S1_at	9.65	7.10	8.41	8.80	8.50	8.72	8.39	1.27	8.67	0.15	1.03	0.048	7.2E-01
Mtr.50035.1.S1_at	10.53	8.01	9.54	10.16	9.61	10.16	9.36	1.27	9.98	0.31	1.07	0.092	4.6E-01
Mtr.9820.1.S1_at	5.90	3.36	4.67	4.66	5.04	3.86	4.65	1.27	4.52	0.60	0.97	-0.040	8.8E-01
Mtr.31010.1.S1_at	5.57	3.08	3.89	2.90	2.98	2.99	4.18	1.27	2.96	0.05	0.71	-0.499	1.7E-01
Mtr.5153.1.S1_at	4.45	2.45	4.80	3.80	5.02	3.83	3.90	1.27	4.22	0.70	1.08	0.113	7.2E-01
Mtr.10881.1.S1_at	6.95	5.39	7.91	7.14	7.19	6.92	6.75	1.27	7.09	0.14	1.05	0.071	6.7E-01
Mtr.46552.1.S1_at	7.23	5.26	7.64	7.26	7.36	6.99	6.71	1.27	7.20	0.19	1.07	0.102	5.4E-01
Mtr.6317.1.S1_at	9.00	6.46	7.56	7.71	7.41	7.62	7.67	1.27	7.58	0.15	0.99	-0.017	9.1E-01
Mtr.41571.1.S1_at	8.83	6.48	8.50	8.27	8.06	8.35	7.94	1.27	8.23	0.15	1.04	0.052	7.1E-01
Mtr.42674.1.S1_at	5.68	3.50	3.47	4.82	5.41	5.94	4.22	1.27	5.39	0.56	1.28	0.353	2.2E-01
Mtr.48555.1.S1_at	2.62	4.81	2.60	2.55	2.55	2.59	3.34	1.27	2.56	0.03	0.77	-0.382	3.5E-01
Mtr.6846.1.S1_s_at	6.44	4.05	5.99	6.41	6.29	6.56	5.49	1.27	6.42	0.14	1.17	0.225	2.8E-01
Mtr.16555.1.S1_s_at	4.97	2.72	2.82	2.80	2.64	2.84	3.50	1.27	2.76	0.10	0.79	-0.344	3.7E-01
Mtr.51555.1.S1_at	6.45	5.22	7.76	6.84	7.30	6.32	6.48	1.27	6.82	0.49	1.05	0.075	6.8E-01
Mtr.4651.1.S1_at	4.97	2.76	4.95	4.95	5.07	4.85	4.22	1.27	4.96	0.11	1.17	0.231	3.8E-01
Mtr.41537.1.S1_at	6.13	4.40	6.87	6.76	6.46	6.49	5.80	1.27	6.57	0.17	1.13	0.179	3.6E-01
Mtr.39300.1.S1_at	2.09	4.25	2.02	2.08	2.08	2.11	2.79	1.27	2.09	0.02	0.75	-0.415	4.0E-01
Mtr.34269.1.S1_at	1.82	4.17	2.18	2.18	2.18	2.18	2.72	1.27	2.18	0.00	0.80	-0.323	5.0E-01
Mtr.8564.1.S1_at	10.77	8.49	10.59	10.67	10.89	10.73	9.95	1.27	10.77	0.11	1.08	0.114	3.3E-01
Mtr.16275.1.S1_at	5.78	3.27	4.17	3.23	3.04	3.09	4.41	1.27	3.12	0.10	0.71	-0.498	1.5E-01
Mtr.41563.1.S1_at	6.33	4.49	6.92	6.90	6.42	6.53	5.91	1.27	6.62	0.25	1.12	0.163	4.0E-01
Mtr.2282.1.S1_at	10.37	8.16	10.34	10.46	10.49	10.56	9.62	1.27	10.51	0.05	1.09	0.127	2.9E-01
Mtr.42854.1.S1_at	6.06	5.13	7.64	6.22	6.01	6.29	6.28	1.27	6.17	0.14	0.98	-0.023	9.0E-01
Mtr.29523.1.S1_at	2.44	4.62	2.42	2.42	2.41	2.41	3.16	1.27	2.41	0.00	0.76	-0.388	3.7E-01
Mtr.12871.1.S1_at	8.29	6.20	8.49	8.23	7.89	7.99	7.66	1.27	8.04	0.17	1.05	0.069	6.4E-01
Mtr.40697.1.S1_at	4.67	2.87	5.32	4.22	4.26	4.21	4.29	1.27	4.23	0.03	0.99	-0.019	9.4E-01
Mtr.38349.1.S1_at	3.88	3.06	5.54	5.34	4.11	4.18	4.16	1.27	4.54	0.69	1.09	0.126	6.7E-01
Mtr.13188.1.S1_at	4.51	2.28	4.44	4.43	4.43	4.43	3.74	1.27	4.43	0.00	1.18	0.244	4.0E-01
Mtr.26952.1.S1_at	2.65	4.80	2.58	2.61	2.54	2.56	3.34	1.27	2.57	0.03	0.77	-0.379	3.5E-01
Mtr.29041.1.S1_at	6.93	4.75	6.96	6.35	7.08	6.95	6.21	1.27	6.80	0.39	1.09	0.129	4.9E-01
Mtr.22733.1.S1_at	3.61	5.29	2.81	3.56	3.56	3.61	3.90	1.27	3.58	0.03	0.92	-0.125	6.8E-01
Mtr.39343.1.S1_s_at	5.24	3.42	5.85	5.67	5.62	5.90	4.84	1.26	5.73	0.15	1.18	0.244	2.9E-01
Mtr.3169.1.S1_at	7.00	4.67	6.69	6.48	6.43	6.40	6.12	1.26	6.44	0.04	1.05	0.073	6.9E-01
Mtr.10989.1.S1_at	5.30	2.80	3.73	5.18	5.28	5.18	3.94	1.26	5.22	0.06	1.32	0.404	1.6E-01
Mtr.12363.1.S1_at	10.84	8.99	8.41	10.10	10.07	10.45	9.42	1.26	10.21	0.21	1.08	0.117	3.4E-01
Mtr.9870.1.S1_at	7.80	5.45	7.43	7.48	7.24	7.54	6.89	1.26	7.42	0.15	1.08	0.106	5.1E-01
Mtr.38819.1.S1_s_at	11.06	9.03	11.36	11.10	10.81	11.09	10.48	1.26	11.00	0.17	1.05	0.069	5.2E-01
Mtr.16031.1.S1_at	3.55	5.70	3.46	3.32	3.42	3.29	4.24	1.26	3.34	0.07	0.79	-0.342	2.9E-01
Mtr.28560.1.S1_at	5.13	2.63	4.22	3.15	2.94	2.62	3.99	1.26	2.90	0.27	0.73	-0.459	2.2E-01
Mtr.50208.1.S1_at	7.83	5.32	6.86	6.82	6.89	6.88	6.67	1.26	6.86	0.04	1.03	0.042	8.0E-01
Mtr.13466.1.S1_at	7.27	5.15	7.41	6.97	7.16	6.93	6.61	1.26	7.02	0.12	1.06	0.087	6.1E-01
Mtr.8820.1.S1_at	7.26	4.86	6.73	6.47	7.17	7.26	6.28	1.26	6.97	0.43	1.11	0.149	4.2E-01
Mtr.47251.1.S1_at	7.70	5.42	7.51	7.33	7.59	7.38	6.88	1.26	7.43	0.14	1.08	0.111	4.9E-01
Mtr.42476.1.S1_at	7.59	5.86	8.31	7.58	7.41	7.00	7.25	1.26	7.33	0.30	1.01	0.015	9.2E-01
Mtr.49004.1.S1_at	3.40	5.87	4.15	3.44	2.81	3.32	4.47	1.26	3.19	0.33	0.71	-0.488	1.6E-01
Mtr.16038.1.S1_at	3.09	2.67	5.04	4.10	3.35	4.33	3.60	1.26	3.93	0.51	1.09	0.127	7.0E-01
Mtr.25981.1.S1_s_at	5.89	3.43	4.16	3.90	4.09	4.03	4.49	1.26	4.01	0.10	0.89	-0.165	5.4E-01
Mtr.34598.1.S1_at	8.39	5.98	7.84	7.77	7.75	7.63	7.40	1.26	7.72	0.07	1.04	0.061	6.9E-01
Mtr.21741.1.S1_at	7.32	5.47	7.88	7.95	7.80	7.71	6.89	1.26	7.82	0.12	1.14	0.183	2.7E-01
Mtr.28405.1.S1_at	5.80	4.13	6.60	6.06	5.97	5.56	5.51	1.26	5.86	0.27	1.06	0.090	6.6E-01
Mtr.21952.1.S1_s_at	5.41	3.47	5.83	6.26	6.28	5.63	4.90	1.26	6.06	0.37	1.23	0.304	2.0E-01
Mtr.20716.1.S1_at	5.68	3.49	5.68	5.60	5.93	5.65	4.95	1.26	5.73	0.18	1.16	0.210	3.5E-01
Mtr.4652.1.S1_at	9.70	7.25	8.98	9.61	10.00	9.86	8.64	1.26	9.82	0.20	1.14	0.185	1.9E-01
Mtr.5241.1.S1_at	4.49	2.41	4.69	4.69	5.00	5.04	3.86	1.26	4.91	0.19	1.27	0.346	2.3E-01
Mtr.31205.1.S1_at	6.44	3.96	5.59	5.88	5.06	5.59	5.33	1.26	5.51	0.41	1.03	0.048	8.3E-01
Mtr.27864.1.S1_x_at	2.87	5.09	2.93	3.00	2.93	2.94	3.63	1.26	2.96	0.04	0.81	-0.296	4.1E-01
Mtr.47286.1.S1_at	7.04	4.85	7.03	6.86	7.16	7.07	6.31	1.26	7.03	0.16	1.11	0.157	3.8E-01
Mtr.10277.1.S1_at	6.18	4.36	6.78	6.17	6.17	6.47	5.78	1.26	6.27	0.17	1.09	0.118	5.4E-01
Mtr.7066.1.S1_at	10.39	8.32	10.60	11.09	10.79	10.96	9.77	1.26	10.95	0.15	1.12	0.164	1.8E-01
Mtr.43889.1.S1_at	7.63	5.52	7.76	7.53	6.59	7.24	6.97	1.26	7.12	0.48	1.02	0.031	8.5E-01
Mtr.12040.1.S1_at	5.31	3.04	5.13	5.25	5.35	5.44	4.49	1.26	5.34	0.10	1.19	0.251	3.1E-01
Mtr.33295.1.S1_at	6.54	4.31	6.44	6.43	6.44	6.40	5.76	1.26	6.42	0.02	1.11	0.156	4.2E-01
Mtr.29096.1.S1_at	6.88	5.16	7.61	7.26	6.93	6.75	6.55	1.26	6.98	0.26	1.07	0.091	6.0E-01
Mtr.39444.1.S1_at	7.08	5.23	7.64	7.27	7.16	7.24	6.65	1.26	7.23	0.06	1.09	0.120	4.7E-01
Mtr.5291.1.S1_at	7.36	4.96	6.81	7.11	7.04	6.90	6.38	1.26	7.02	0.11	1.10	0.138	4.3E-01
Mtr.9961.1.S1_at	5.86	3.78	6.06	5.96	6.18	6.45	5.23	1.26	6.20	0.25	1.18	0.244	2.6E-01
Mtr.38862.1.S1_at	5.43	3.37	5.66	5.94	5.34	5.47	4.82	1.26	5.58	0.32	1.16	0.211	3.7E-01
Mtr.50386.1.S1_at	5.18	7.41	5.27	5.20	5.14	5.20	5.95	1.26	5.18	0.04	0.87	-0.201	3.5E-01
Mtr.22088.1.S1_at	2.74	4.89	2.69	2.67	2.66	2.66	3.44	1.26	2.67	0.01	0.77	-0.368	3.5E-01
Mtr.33630.1.S1_at	5.70	3.90	6.33	5.68	5.66	5.69	5.31	1.26	5.68	0.02	1.07	0.096	6.4E-01
Mtr.27796.1.S1_at	7.76	5.41	7.36	7.69	7.49	7.66	6.84	1.26	7.62	0.11	1.11	0.155	3.5E-01
Mtr.26698.1.S1_at	7.36	5.61	8.06	7.59	7.42	7.50	7.01	1.26	7.50	0.09	1.07	0.098	5.3E-01
Mtr.6504.1.S1_at	7.36	5.32	7.61	7.73	7.65	7.58	6.76	1.26	7.65	0.08	1.13	0.179	2.9E-01
Mtr.38963.1.S1_at	6.41	4.43	6.76	6.55	6.39	6.52	5.87	1.26	6.48	0.09	1.11	0.144	4.4E-01
Mtr.16354.1.S1_at	7.41	4.96	5.69	5.88	5.51	5.94	6.02	1.26	5.78	0.23	0.96	-0.059	7.6E-01
Mtr.40091.1.S1_at	10.52	9.50	12.00	11.96	11.81	11.60	10.67	1.26	11.79	0.18	1.10	0.144	2.0E-01
Mtr.29052.1.S1_at	4.55	2.35	4.49	5.18	4.74	4.73	3.80	1.26	4.88	0.26	1.29	0.363	2.2E-01
Mtr.47111.1.S1_x_at	5.79	3.74	6.02	5.21	5.67	4.42	5.18	1.26	5.10	0.64	0.98	-0.024	9.2E-01
Mtr.21776.1.S1_at	7.40	5.16	7.25	7.91	7.75	7.53	6.60	1.26	7.73	0.19	1.17	0.227	2.0E-01
Mtr.40765.1.S1_at	6.26	4.52	6.96	8.34	9.22	8.72	5.91	1.26	8.76	0.44	1.48	0.568	2.1E-02
Mtr.39706.1.S1_at	11.27	9.34	11.69	11.81	11.69	11.56	10.76	1.26	11.69	0.12	1.09	0.119	2.7E-01

Mtr.52316.1.S1_at	6.82	4.38	6.11	7.00	7.26	7.04	5.77	1.26	7.10	0.14	1.23	0.300	1.4E-01
Mtr.36882.1.S1_at	9.05	7.97	10.47	9.94	9.91	9.32	9.16	1.25	9.72	0.35	1.06	0.086	5.0E-01
Mtr.31327.1.S1_at	7.32	5.26	7.52	7.16	7.40	7.19	6.70	1.25	7.25	0.13	1.08	0.113	4.9E-01
Mtr.15079.1.S1_at	2.86	5.00	2.79	2.79	2.78	2.78	3.55	1.25	2.78	0.01	0.78	-0.352	3.5E-01
Mtr.30489.1.S1_at	8.21	6.30	8.66	7.84	8.78	8.53	7.72	1.25	8.38	0.49	1.09	0.119	4.4E-01
Mtr.46083.1.S1_at	3.15	5.29	3.09	3.02	3.04	3.02	3.84	1.25	3.03	0.01	0.79	-0.344	3.2E-01
Mtr.26299.1.S1_at	10.13	7.63	9.09	8.97	9.09	8.87	8.95	1.25	8.98	0.11	1.00	0.005	9.7E-01
Mtr.10566.1.S1_at	5.63	3.14	4.64	2.94	3.08	3.07	4.47	1.25	3.03	0.08	0.68	-0.561	1.2E-01
Mtr.42742.1.S1_at	7.09	5.04	7.31	7.19	7.32	7.25	6.48	1.25	7.26	0.06	1.12	0.163	3.4E-01
Mtr.7754.1.S1_s_at	5.14	2.85	4.89	5.84	5.13	5.17	4.29	1.25	5.38	0.40	1.25	0.326	2.3E-01
Mtr.11908.1.S1_at	7.99	5.77	7.89	7.89	7.90	7.89	7.22	1.25	7.89	0.01	1.09	0.129	4.0E-01
Mtr.7104.1.S1_at	3.94	2.45	4.94	4.32	4.54	4.59	3.78	1.25	4.48	0.14	1.19	0.247	3.9E-01
Mtr.27871.1.S1_s_at	3.08	5.17	2.92	3.05	3.10	3.03	3.72	1.25	3.06	0.03	0.82	-0.284	4.1E-01
Mtr.21748.1.S1_at	3.84	6.11	4.05	3.85	3.56	3.56	4.67	1.25	3.65	0.17	0.78	-0.354	2.4E-01
Mtr.31269.1.S1_at	9.38	7.11	9.16	9.74	9.39	9.78	8.55	1.25	9.63	0.22	1.13	0.173	2.1E-01
Mtr.15322.1.S1_at	3.11	5.22	3.00	3.55	2.94	2.92	3.78	1.25	3.13	0.36	0.83	-0.269	4.4E-01
Mtr.9963.1.S1_at	5.48	3.20	5.24	5.47	4.79	5.24	4.64	1.25	5.17	0.35	1.11	0.155	5.2E-01
Mtr.29017.1.S1_at	2.62	4.77	2.59	2.58	2.57	2.57	3.33	1.25	2.58	0.01	0.77	-0.369	3.6E-01
Mtr.38482.1.S1_at	9.33	7.77	10.24	10.01	10.62	9.95	9.11	1.25	10.19	0.37	1.12	0.162	2.3E-01
Mtr.1664.1.S1_s_at	6.04	3.79	5.86	5.63	5.79	5.73	5.23	1.25	5.72	0.08	1.09	0.128	5.4E-01
Mtr.20690.1.S1_at	6.63	5.23	7.72	7.52	6.97	7.69	6.53	1.25	7.39	0.37	1.13	0.180	3.1E-01
Mtr.41768.1.S1_at	6.37	4.24	4.16	4.51	4.30	5.09	4.92	1.25	4.63	0.41	0.94	-0.088	7.2E-01
Mtr.32238.1.S1_at	9.16	6.79	8.66	8.67	8.66	8.62	8.21	1.25	8.65	0.03	1.05	0.076	5.7E-01
Mtr.12558.1.S1_at	11.73	9.50	11.59	11.94	12.09	11.73	10.94	1.25	11.92	0.18	1.09	0.124	2.5E-01
Mtr.32953.1.S1_at	4.85	2.63	4.74	4.12	4.12	4.05	4.08	1.25	4.10	0.05	1.01	0.007	9.8E-01
Mtr.10298.1.S1_at	5.41	3.14	5.19	3.35	3.35	3.04	4.58	1.25	3.25	0.18	0.71	-0.495	1.4E-01
Mtr.16265.1.S1_at	2.56	4.71	2.53	2.52	2.52	2.51	3.27	1.25	2.52	0.01	0.77	-0.377	3.6E-01
Mtr.18822.1.S1_at	7.92	5.48	6.24	6.27	5.44	6.27	6.54	1.25	5.99	0.48	0.92	-0.127	5.2E-01
Mtr.4273.1.S1_s_at	7.48	5.34	7.54	7.48	6.91	7.34	6.79	1.25	7.25	0.30	1.07	0.095	5.7E-01
Mtr.15521.1.S1_at	2.85	4.99	2.81	2.83	2.83	2.82	3.55	1.25	2.83	0.01	0.80	-0.328	3.7E-01
Mtr.37395.1.S1_at	11.88	12.42	10.04	10.76	12.15	11.53	11.44	1.25	11.48	0.70	1.00	0.005	9.7E-01
Mtr.28421.1.S1_at	6.07	3.57	4.78	4.98	4.58	4.64	4.81	1.25	4.73	0.22	0.98	-0.022	9.2E-01
Mtr.42139.1.S1_at	8.25	6.08	8.23	8.06	8.34	8.16	7.52	1.25	8.19	0.14	1.09	0.122	4.1E-01
Mtr.8565.1.S1_s_at	4.66	6.94	4.92	4.33	4.55	4.65	5.50	1.25	4.51	0.17	0.82	-0.286	2.4E-01
Mtr.34215.1.S1_at	6.58	4.18	5.96	6.87	6.59	6.83	5.57	1.25	6.77	0.15	1.21	0.280	1.8E-01
Mtr.37315.1.S1_at	9.58	7.22	9.12	9.61	9.25	9.26	8.64	1.25	9.38	0.21	1.09	0.118	3.7E-01
Mtr.17189.1.S1_at	2.18	4.34	2.17	2.17	2.17	2.17	2.89	1.25	2.17	0.00	0.75	-0.416	3.7E-01
Mtr.14723.1.S1_at	6.33	4.35	6.65	6.48	6.49	6.19	5.78	1.25	6.39	0.17	1.11	0.144	4.5E-01
Mtr.21518.1.S1_at	7.03	4.91	7.11	6.57	6.70	6.29	6.35	1.25	6.52	0.21	1.03	0.038	8.3E-01
Mtr.25016.1.S1_at	5.41	3.04	4.88	4.89	5.28	4.92	4.44	1.25	5.03	0.22	1.13	0.179	4.7E-01
Mtr.14790.1.S1_at	4.74	2.57	4.72	4.72	4.81	4.77	4.01	1.25	4.77	0.05	1.19	0.249	3.5E-01
Mtr.6192.1.S1_at	10.53	8.84	8.09	9.63	9.60	9.81	9.15	1.25	9.68	0.11	1.06	0.081	5.1E-01
Mtr.35.1.S1_at	7.56	6.89	9.31	7.83	7.81	8.13	7.92	1.25	7.92	0.18	1.00	0.000	1.0E+00
Mtr.21873.1.S1_s_at	3.30	5.50	3.38	3.38	3.38	3.35	4.06	1.25	3.37	0.02	0.83	-0.269	3.9E-01
Mtr.1946.1.S1_at	8.38	6.19	8.31	8.57	8.72	8.42	7.62	1.25	8.57	0.15	1.12	0.169	2.6E-01
Mtr.34806.1.S1_s_at	9.60	7.13	8.11	8.71	8.58	8.81	8.28	1.25	8.70	0.11	1.05	0.071	5.9E-01
Mtr.35219.1.S1_at	6.52	5.47	7.95	7.49	7.54	7.49	6.65	1.25	7.51	0.03	1.13	0.175	3.0E-01
Mtr.5237.1.S1_at	6.47	4.27	6.39	6.53	6.36	6.40	5.71	1.25	6.43	0.09	1.13	0.171	3.8E-01
Mtr.28730.1.S1_at	5.13	3.32	5.71	5.01	5.07	4.97	4.72	1.25	5.01	0.05	1.06	0.086	7.1E-01
Mtr.31860.1.S1_at	6.60	4.66	6.98	6.55	6.64	6.52	6.08	1.25	6.57	0.06	1.08	0.113	5.3E-01
Mtr.22591.1.S1_at	2.64	4.69	2.44	2.63	2.61	2.62	3.25	1.25	2.62	0.01	0.81	-0.313	4.3E-01
Mtr.15130.1.S1_s_at	2.45	4.59	2.42	2.40	2.41	2.63	3.15	1.25	2.48	0.13	0.79	-0.348	4.0E-01
Mtr.40778.1.S1_at	9.25	6.92	8.83	9.21	8.78	9.18	8.33	1.25	9.06	0.24	1.09	0.120	3.8E-01
Mtr.17053.1.S1_at	2.81	4.96	2.79	2.77	3.36	2.91	3.52	1.25	3.01	0.31	0.86	-0.224	5.3E-01
Mtr.9530.1.S1_at	8.17	6.01	8.16	8.01	8.18	8.42	7.45	1.25	8.20	0.21	1.10	0.139	3.6E-01
Mtr.773.1.S1_s_at	12.87	11.28	10.41	12.38	11.48	12.58	11.52	1.24	12.15	0.59	1.05	0.076	4.7E-01
Mtr.4768.1.S1_at	6.03	3.91	6.10	6.17	6.28	5.95	5.35	1.24	6.13	0.17	1.15	0.198	3.4E-01
Mtr.28169.1.S1_at	7.22	5.04	7.18	7.18	7.38	7.24	6.48	1.24	7.27	0.10	1.12	0.165	3.4E-01
Mtr.1281.1.S1_at	9.37	6.93	8.56	8.43	8.53	8.38	8.29	1.24	8.45	0.08	1.02	0.027	8.4E-01
Mtr.39909.1.S1_at	2.58	4.71	2.54	2.54	2.52	2.52	3.28	1.24	2.53	0.01	0.77	-0.377	3.5E-01
Mtr.16475.1.S1_at	2.41	4.56	2.40	2.38	2.37	2.40	3.12	1.24	2.38	0.01	0.76	-0.392	3.6E-01
Mtr.51462.1.S1_s_at	9.77	7.79	10.08	10.15	10.00	9.88	9.21	1.24	10.01	0.14	1.09	0.119	3.3E-01
Mtr.35444.1.S1_at	5.37	5.28	7.48	5.83	5.93	5.35	6.04	1.24	5.71	0.31	0.94	-0.083	6.7E-01
Mtr.1351.1.S1_at	6.96	4.92	7.17	7.16	7.37	7.04	6.35	1.24	7.19	0.17	1.13	0.178	3.1E-01
Mtr.40305.1.S1_at	11.64	9.16	10.55	11.05	10.67	11.28	10.45	1.24	11.00	0.30	1.05	0.073	5.0E-01
Mtr.5022.1.S1_at	4.67	2.20	3.65	4.12	3.60	3.85	3.51	1.24	3.86	0.26	1.10	0.137	6.6E-01
Mtr.30636.1.S1_at	3.50	5.64	3.48	3.47	3.46	3.69	4.21	1.24	3.54	0.13	0.84	-0.250	4.1E-01
Mtr.9913.1.S1_at	10.65	8.20	9.06	9.87	9.96	9.79	9.31	1.24	9.87	0.09	1.06	0.085	4.8E-01
Mtr.12006.1.S1_at	8.02	10.13	7.94	8.30	9.93	9.48	8.70	1.24	9.24	0.84	1.06	0.086	5.7E-01
Mtr.8402.1.S1_at	9.37	7.23	9.38	9.62	9.05	9.53	8.66	1.24	9.40	0.31	1.09	0.119	3.7E-01
Mtr.34409.1.S1_at	8.04	5.93	8.11	7.60	7.62	7.80	7.36	1.24	7.67	0.11	1.04	0.060	6.8E-01
Mtr.34718.1.S1_at	4.57	3.37	5.85	5.42	5.24	5.18	4.60	1.24	5.28	0.12	1.15	0.199	4.0E-01
Mtr.17829.1.S1_at	5.72	3.39	5.29	6.74	6.74	6.62	4.80	1.24	6.70	0.07	1.40	0.482	5.7E-02
Mtr.27166.1.S1_at	6.24	4.08	6.22	6.28	6.33	6.21	5.51	1.24	6.28	0.06	1.14	0.187	3.5E-01
Mtr.173.1.S1_at	3.38	5.51	3.35	3.34	3.33	3.32	4.08	1.24	3.33	0.01	0.82	-0.293	3.5E-01
Mtr.43282.1.S1_at	10.80	12.22	9.75	9.29	10.40	9.75	10.93	1.24	9.81	0.56	0.90	-0.155	2.3E-01
Mtr.18857.1.S1_at	5.56	3.42	5.56	5.56	5.63	5.99	4.84	1.24	5.73	0.23	1.18	0.242	2.9E-01
Mtr.45565.1.S1_s_at	5.33	3.02	4.94	4.96	4.91	5.46	4.43	1.24	5.11	0.30	1.15	0.205	4.1E-01
Mtr.5636.1.S1_at	5.74	4.04	6.45	6.06	6.25	5.68	5.41	1.24	6.00	0.29	1.11	0.148	4.7E-01
Mtr.31924.1.S1_at	7.10	4.64	6.09	6.76	7.05	6.45	5.95	1.24	6.75	0.30	1.14	0.184	3.3E-01
Mtr.22769.1.S1_at	7.60	5.90	5.19	5.47	5.71	5.99	6.23	1.24	5.72	0.26	0.92	-0.122	5.3E-01
Mtr.50275.1.S1_s_at	2.42	4.57	2.43	2.43	2.42	2.42	3.14	1.24	2.42	0.01	0.77	-0.373	3.7E-01
Mtr.45374.1.S1_at	6.99	4.75	6.78	7.09	7.09	7.00	6.17	1.24	7.06	0.05	1.14	0.193	2.8E-01
Mtr.9649.1.S1_at	3.87	2.64	5.11	5.55	5.64	5.12	3.87	1.24	5.44	0.28	1.40	0.489	1.0E-01
Mtr.9584.1.S1_at	5.91	3.49	4.26	4.70	4.96	4.74	4.55	1.24	4.80	0.14	1.06	0.078	7.4E-01
Mtr.6937.1.S1_at	8.43	7.06	9.52	8.22	8.27	7.84	8.34	1.24	8.11	0.23	0.97	-0.039	7.7E-01
Mtr.19232.1.S1_at	7.77	6.12	8.54	7.84	7.77	7.58	7.48	1.24	7.73	0.14	1.03	0.047	7.4E-01
Mtr.14090.1.S1_at	9.59	7.14	8.63	9.27	9.13	9.03	8.45	1.24	9.14	0.12	1.08	0.113	3.9E-01
Mtr.34734.1.S1_at	5.15	2.76	3.39	3.39	3.37	3.25	3.77	1.23	3.34	0.07	0.89	-0.174	5.8E-01
Mtr.36482.1.S1_at	2.70	4.79	2.60	2.77	2.85	2.63	3.36	1.23	2.75	0.11	0.82	-0.289	4.4E-01
Mtr.1344.1.S1_at	6.64	4.69	6.98	6.59	6.54	6.67	6.10	1.23	6.60	0.06	1.08	0.112	5.3E-01
Mtr.22444.1.S1_at	9.42	6.98	8.54	8.24	8.20	8.53	8.31	1.23	8.33	0.18	1.00	0.002	9.9E-01
Mtr.39062.1.S1_at	8.05	6.04	8.29	8.99	8.99	9.15	7.46	1.23	9.05	0.09	1.21	0.278	9.1E-02
Mtr.425.1.S1_at	6.06	7.90	5.55	6.30	5.83	5.98	6.50	1.23	6.04	0.24	0.93	-0.107	5.6E-01
Mtr.21552.1.S1_at	2.46	4.60	2.47	2.45	2.44	2.46	3.18	1.23	2.45	0.01	0.77	-0.377	3.6E-01
Mtr.11051.1.S1_at	7.54	5.43	7.60	7.52	7.86	7.43	6.86	1.23	7.60	0.23	1.11	0.148	3.6E-01
Mtr.44414.1.S1_at	2.93	4.87	2.59	3.14	2.95	2.95	3.46	1.23	3.01	0.11	0.87	-0.201	5.6E-01
Mtr.15573.1.S1_at	2.68	4.80	2.64	2.65	2.64	2.63	3.37	1.23	2.64	0.01	0.78	-0.354	3.6E-01
Mtr.3767.1.S1_at	2.40	4.53	2.39	2.38	2.36	2.38	3.10	1.23	2.37	0.01	0.76	-0.387	3.6E-01
Mtr.18406.1.S1_at	3.40	2.60	5.02	5.21	6.05	5.35	3.67	1.23	5.54	0.45	1.51	0.592	7.0E-02
Mtr.50072.1.S1_s_at	2.06	4.18	2.03	2.02	2.02	2.02	2.75	1.23	2.02	0.00	0.73	-0.448	3.6E-01
Mtr.20420.1.S1_at	3.24	5.40	3.29	3.29	3.16	3.40	3.97	1.23	3.28	0.12	0.83	-0.276	3.9E-01
Mtr.45589.1.S1_at	5.71	3.40	5.28	7.12	7.56	7.37	4.79	1.23	7.35	0.22	1.53	0.616	2.4E-02
Mtr.2204.1.S1_at	8.06	5.91	8.03	7.82	8.05	7.69	7.33	1.23	7.85	0.18	1.07	0.099	5.1E-01
Mtr.9411.1.S1_at	3.84	2.04	4.40	4.72	3.52	3.83	3.43	1.23	4.03	0.62	1.17	0.232	4.9E-01
Mtr.28859.1.S1_at	6.27	4.41	6.73	6.47	6.22	6.09	5.80	1.23	6.26	0.19	1.08	0.109	5.6E-01
Mtr.11816.1.S1_at	3.63	3.63	5.76	5.24	4.03	5.54	4.34	1.23	4.94	0.80	1.14	0.187	5.2E-01
Mtr.11286.1.S1_at	8.64	6.21	7.12	7.61	8.02	7.82	7.33	1.23	7.82	0.21	1.07	0.094	5.3E-01
Mtr.749.1.S1_at	2.54	4.65	2.51	2.50	2.49	2.49	3.24	1.23	2.49	0.01	0.77	-0.375	3.6E-01
Mtr.41137.1.S1_at	8.87	7.31	9.73	9.79	9.29	9.86	8.64	1.23	9.64	0.31	1.12	0.159	2.4E-01
Mtr.45729.1.S1_s_at	6.20	4.80	7.24	6.86	6.69	6.76	6.08	1.23	6.77	0.09	1.11	0.156	3.8E-01
Mtr.22006.1.S1_x_at	2.96	5.08	2.95	2.93	2.93	2.92	3.67	1.23	2.93	0.01	0.80	-0.326	3.5E-01
Mtr.44266.1.S1_at	5.65	3.94	6.33	5.60	5.72	5.60	5.31	1.23	5.64	0.07	1.06	0.088	6.6E-01
Mtr.9700.1.S1_at	9.02	7.19	9.52	8.76	8.61	8.61	8.58	1.23	8.66	0.08	1.01	0.014	9.1E-01
Mtr.29423.1.S1_at	5.13	3.32	5.67	5.74	5.66	5.85	4.70	1.23	5.75	0.09	1.22	0.290	2.1E-01
Mtr.21153.1.S1_at	6.60	4.20	4.96	7.47	7.20	7.62	5.25	1.23	7.43	0.21	1.42	0.501	3.9E-02
Mtr.8189.1.S1_at	3.10	5.23	3.11	3.12	3.12	3.12	3.81	1.23	3.12	0.00	0.82	-0.289	3.8E-01
Mtr.28125.1.S1_s_at	12.57	10.97	13.38	13.32	12.89	13.17	12.31	1.23	13.13	0.22	1.07	0.093	3.2E-01
Mtr.43659.1.S1_at	7.73	5.42	5.86	7.14	7.11	7.27	6.34	1.23	7.17	0.08	1.13	0.179	3.0E-01
Mtr.11071.1.S1_s_at	8.28	6.10	8.17	8.17	8.11	8.25	7.51	1.23	8.18	0.07	1.09	0.122	4.0E-01
Mtr.33919.1.S1_at	6.51	4.51	6.74	6.61	7.10	6.67	5.92	1.23	6.79	0.27	1.15	0.199	2.9E-01
Mtr.35047.1.S1_s_at	5.87	3.76	5.89	5.47	5.64	5.66	5.17	1.23	5.59	0.10	1.08	0.112	5.9E-01
Mtr.20409.1.S1_at	10.68	8.31	10.03	10.13	10.65	9.96	9.67	1.23	10.25	0.36	1.06	0.083	4.8E-01
Mtr.49256.1.S1_at	5.95	4.56	7.00	6.24	5.95	5.08	5.84	1.23	5.76	0.60	0.99	-0.021	9.2E-01
Mtr.33150.1.S1_s_at	7.41	5.59	7.92	7.92	8.24	8.47	6.98	1.23	8.21	0.27	1.18	0.235	1.6E-01
Mtr.12774.1.S1_s_at	9.69	7.24	8.46	9.02	8.77	9.18	8.46	1.22	8.99	0.20	1.06	0.087	5.1E-01
Mtr.44758.1.S1_at	5.22	2.83	3.55	2.96	2.75	3.35	3.87	1.22	3.02	0.31	0.78	-0.355	3.1E-01
Mtr.39315.1.S1_s_at	7.73	5.77	8.02	7.70	7.57	7.37	7.17	1.22	7.55	0.17	1.05	0.074	6.2E-01
Mtr.51587.1.S1_at	7.83	9.50	7.12	7.25	7.08	7.08	8.15	1.22	7.14	0.10	0.88	-0.191	2.3E-01
Mtr.18041.1.S1_at	2.23	2.18	4.32	4.08	3.39	3.85	2.91	1.22	3.77	0.35	1.30	0.375	3.1E-01
Mtr.13116.1.S1_at	3.81	5.64	6.12	4.88	6.37	5.75	5.19	1.22	5.67	0.75	1.09	0.127	5.9E-01

Mtr.32700.1.S1_s_at	3.02	1.64	4.08	3.00	3.17	2.53	2.91	1.22	2.90	0.33	1.00	-0.006	9.9E-01
Mtr.1167.1.S1_s_at	9.84	12.02	9.96	10.08	9.67	9.72	10.61	1.22	9.82	0.22	0.93	-0.111	3.4E-01
Mtr.5732.1.S1_at	5.09	6.83	7.45	4.57	4.22	4.36	6.46	1.22	4.38	0.18	0.68	-0.559	4.4E-02
Mtr.33174.1.S1_at	6.81	5.06	7.42	6.87	7.17	6.90	6.43	1.22	6.98	0.16	1.09	0.118	4.8E-01
Mtr.4639.1.S1_at	10.88	8.53	10.29	10.02	9.90	9.91	9.90	1.22	9.94	0.06	1.00	0.006	9.6E-01
Mtr.33203.1.S1_at	7.85	5.81	7.99	7.88	7.58	7.74	7.22	1.22	7.73	0.15	1.07	0.100	5.1E-01
Mtr.4988.1.S1_at	6.82	4.69	6.79	6.61	7.33	6.74	6.10	1.22	6.89	0.38	1.13	0.177	3.4E-01
Mtr.14434.1.S1_at	3.49	2.97	5.30	4.57	4.57	5.10	3.92	1.22	4.75	0.31	1.21	0.275	3.2E-01
Mtr.27170.1.S1_at	6.76	4.59	6.64	6.83	6.29	6.64	6.00	1.22	6.59	0.28	1.10	0.135	4.6E-01
Mtr.28536.1.S1_s_at	7.59	5.43	7.49	7.53	7.33	7.53	6.83	1.22	7.47	0.12	1.09	0.127	4.2E-01
Mtr.42775.1.S1_at	11.90	10.16	9.55	10.89	10.74	10.93	10.54	1.22	10.85	0.10	1.03	0.042	6.8E-01
Mtr.36688.1.S1_x_at	4.53	2.34	4.37	4.52	4.89	4.57	3.75	1.22	4.66	0.20	1.24	0.316	2.7E-01
Mtr.4610.1.S1_at	7.41	5.51	7.79	7.56	7.37	7.14	6.91	1.22	7.36	0.21	1.07	0.091	5.6E-01
Mtr.21545.1.S1_at	4.14	6.24	4.12	4.11	4.11	4.10	4.83	1.22	4.11	0.01	0.85	-0.236	3.6E-01
Mtr.43856.1.S1_at	4.78	3.71	6.14	3.43	4.28	4.04	4.88	1.22	3.92	0.44	0.80	-0.317	2.7E-01
Mtr.14341.1.S1_at	7.52	5.15	6.86	6.94	6.92	7.01	6.51	1.22	6.96	0.05	1.07	0.095	5.6E-01
Mtr.13139.1.S1_at	8.32	5.88	7.11	7.79	8.80	7.96	7.10	1.22	8.18	0.54	1.15	0.204	2.3E-01
Mtr.28249.1.S1_at	8.40	6.01	7.65	7.94	8.40	8.40	7.35	1.22	8.24	0.27	1.12	0.165	2.8E-01
Mtr.30534.1.S1_at	2.68	4.79	2.67	2.63	2.62	2.67	3.38	1.22	2.64	0.03	0.78	-0.358	3.5E-01
Mtr.4377.1.S1_at	8.55	6.41	8.49	9.55	9.92	9.76	7.82	1.22	9.75	0.19	1.25	0.318	5.4E-02
Mtr.11171.1.S1_at	10.39	8.33	10.49	10.21	10.42	10.14	9.74	1.22	10.26	0.15	1.05	0.075	5.0E-01
Mtr.1057.1.S1_at	2.55	4.63	2.48	2.46	2.45	2.42	3.22	1.22	2.44	0.02	0.76	-0.399	3.3E-01
Mtr.18871.1.S1_at	6.34	4.54	6.87	7.00	6.85	6.68	5.92	1.22	6.85	0.16	1.16	0.210	2.6E-01
Mtr.1392.1.S1_at	2.67	4.77	2.66	2.65	2.64	2.64	3.37	1.22	2.64	0.01	0.79	-0.348	3.6E-01
Mtr.39147.1.S1_at	6.06	4.02	6.19	6.19	6.19	6.26	5.42	1.22	6.21	0.04	1.15	0.196	3.3E-01
Mtr.2246.1.S1_at	8.25	6.24	8.45	7.78	7.99	7.73	7.65	1.22	7.83	0.13	1.02	0.035	8.0E-01
Mtr.3604.1.S1_at	2.02	4.12	2.01	2.01	2.01	2.00	2.72	1.22	2.01	0.00	0.74	-0.439	3.7E-01
Mtr.12071.1.S1_at	7.08	6.38	8.75	7.89	8.28	6.75	7.40	1.22	7.64	0.80	1.03	0.046	7.9E-01
Mtr.24972.1.S1_at	6.57	4.33	6.28	7.71	7.38	7.42	5.73	1.22	7.50	0.18	1.31	0.390	6.7E-02
Mtr.23479.1.S1_at	3.13	5.18	3.03	3.02	2.95	2.96	3.78	1.22	2.98	0.04	0.79	-0.345	3.2E-01
Mtr.11058.1.S1_at	10.13	8.02	10.12	10.08	9.83	10.01	9.42	1.22	9.97	0.13	1.06	0.082	4.8E-01
Mtr.14973.1.S1_s_at	7.84	5.43	6.95	7.80	7.86	7.70	6.74	1.22	7.79	0.08	1.16	0.208	2.1E-01
Mtr.42208.1.S1_at	4.83	2.41	3.75	3.86	3.80	3.82	3.66	1.22	3.82	0.03	1.04	0.062	8.3E-01
Mtr.29617.1.S1_at	5.55	3.57	5.78	5.84	5.77	6.19	4.97	1.22	5.94	0.22	1.19	0.257	2.5E-01
Mtr.13814.1.S1_at	7.59	5.53	7.68	7.67	7.52	7.51	6.94	1.22	7.57	0.09	1.09	0.125	4.2E-01
Mtr.5656.1.S1_at	8.45	6.04	7.00	7.22	7.25	7.40	7.16	1.22	7.29	0.10	1.02	0.025	8.7E-01
Mtr.17546.1.S1_at	6.02	3.89	5.97	6.22	6.56	5.90	5.30	1.22	6.22	0.33	1.18	0.233	2.7E-01
Mtr.37429.1.S1_at	9.35	7.46	7.09	7.76	8.00	8.07	7.97	1.21	7.95	0.16	1.00	-0.004	9.8E-01
Mtr.45450.1.S1_at	6.27	4.07	6.06	5.76	5.73	5.76	5.47	1.21	5.75	0.02	1.05	0.074	7.0E-01
Mtr.33463.1.S1_at	3.72	6.02	4.20	3.50	2.71	3.48	4.65	1.21	3.23	0.45	0.70	-0.524	1.3E-01
Mtr.17382.1.S1_x_at	5.13	2.91	4.88	4.76	4.96	4.69	4.31	1.21	4.80	0.14	1.12	0.157	5.2E-01
Mtr.35688.1.S1_at	2.68	2.82	4.85	4.72	3.79	5.97	3.45	1.21	4.83	1.09	1.40	0.484	2.2E-01
Mtr.10669.1.S1_at	12.32	10.44	12.71	12.65	12.58	12.44	11.82	1.21	12.56	0.11	1.06	0.087	3.5E-01
Mtr.20203.1.S1_at	10.93	8.65	10.51	10.29	10.17	10.64	10.03	1.21	10.37	0.24	1.03	0.047	6.6E-01
Mtr.22713.1.S1_s_at	5.64	4.41	6.84	5.85	5.91	5.91	5.63	1.21	5.89	0.03	1.05	0.066	7.3E-01
Mtr.40773.1.S1_at	12.56	10.28	10.71	11.91	11.72	12.01	11.18	1.21	11.88	0.15	1.06	0.087	3.8E-01
Mtr.50423.1.S1_at	2.90	4.95	2.80	2.77	2.93	2.72	3.55	1.21	2.81	0.11	0.79	-0.338	3.5E-01
Mtr.46408.1.S1_at	5.44	3.27	3.42	3.17	3.07	3.09	4.04	1.21	3.11	0.06	0.77	-0.377	2.6E-01
Mtr.10123.1.S1_at	7.06	4.90	6.94	7.14	7.19	7.07	6.30	1.21	7.13	0.06	1.13	0.180	3.0E-01
Mtr.6113.1.S1_at	7.25	5.27	7.48	8.24	8.65	8.05	6.67	1.21	8.31	0.30	1.25	0.318	8.5E-02
Mtr.49302.1.S1_x_at	2.23	4.32	2.22	2.21	2.21	2.21	2.92	1.21	2.21	0.00	0.76	-0.404	3.7E-01
Mtr.4918.1.S1_at	7.79	5.38	6.35	6.56	7.61	7.18	6.51	1.21	7.12	0.53	1.09	0.129	4.7E-01
Mtr.41288.1.S1_at	7.52	6.40	8.82	7.78	8.03	7.49	7.58	1.21	7.77	0.27	1.02	0.035	8.1E-01
Mtr.30415.1.S1_s_at	5.32	3.59	5.93	7.28	7.37	7.53	4.94	1.21	7.39	0.12	1.50	0.581	2.5E-02
Mtr.124.1.S1_s_at	5.76	4.02	6.35	5.75	5.74	5.74	5.37	1.21	5.74	0.01	1.07	0.096	6.2E-01
Mtr.42591.1.S1_at	5.31	3.37	5.60	5.88	5.75	5.43	4.76	1.21	5.69	0.23	1.19	0.256	2.6E-01
Mtr.39082.1.S1_at	7.80	5.72	7.83	7.29	7.11	7.09	7.12	1.21	7.16	0.11	1.01	0.009	9.5E-01
Mtr.44488.1.S1_at	10.16	8.67	11.07	10.68	10.75	10.37	9.97	1.21	10.60	0.20	1.06	0.088	4.3E-01
Mtr.35253.1.S1_at	7.32	5.77	8.16	7.98	7.86	7.62	7.08	1.21	7.82	0.18	1.10	0.143	3.6E-01
Mtr.24846.1.S1_at	4.12	6.21	4.11	4.11	3.92	4.12	4.82	1.21	4.05	0.11	0.84	-0.249	3.4E-01
Mtr.37769.1.S1_at	5.29	3.10	5.07	4.83	5.11	4.50	4.49	1.21	4.81	0.30	1.07	0.100	6.8E-01
Mtr.1964.1.S1_at	6.18	4.77	7.18	6.19	6.47	5.94	6.05	1.21	6.20	0.27	1.03	0.037	8.4E-01
Mtr.13930.1.S1_at	6.96	4.84	6.92	7.16	7.10	7.13	6.24	1.21	7.13	0.03	1.14	0.192	2.7E-01
Mtr.31882.1.S1_at	5.76	3.75	5.93	5.75	6.30	5.66	5.15	1.21	5.90	0.34	1.15	0.198	3.6E-01
Mtr.23810.1.S1_at	2.44	4.52	2.41	2.40	2.40	2.39	3.13	1.21	2.40	0.01	0.77	-0.383	3.5E-01
Mtr.37273.1.S1_at	4.71	2.90	5.19	4.62	4.90	4.64	4.27	1.21	4.72	0.16	1.11	0.145	5.6E-01
Mtr.6193.1.S1_at	6.23	4.43	6.72	6.38	6.73	6.16	5.79	1.21	6.42	0.29	1.11	0.149	4.3E-01
Mtr.20790.1.S1_at	5.49	3.73	6.05	5.88	5.51	5.84	5.09	1.21	5.74	0.20	1.13	0.173	4.1E-01
Mtr.428.1.S1_at	7.76	5.53	7.45	7.59	7.49	7.61	6.92	1.21	7.56	0.06	1.09	0.128	4.1E-01
Mtr.34997.1.S1_at	6.66	4.52	6.57	6.75	6.83	6.96	5.92	1.21	6.85	0.11	1.16	0.211	2.5E-01
Mtr.27856.1.S1_at	6.95	4.69	6.55	7.05	7.06	6.98	6.06	1.21	7.03	0.05	1.16	0.214	2.4E-01
Mtr.48891.1.S1_s_at	7.96	5.88	7.98	7.77	8.08	8.00	7.27	1.21	7.95	0.16	1.09	0.129	3.9E-01
Mtr.11782.1.S1_at	4.83	2.50	4.19	4.32	4.76	4.20	3.84	1.21	4.43	0.29	1.15	0.206	4.6E-01
Mtr.21141.1.S1_at	7.48	6.10	8.50	7.93	8.15	7.64	7.36	1.21	7.91	0.26	1.07	0.103	4.9E-01
Mtr.23981.1.S1_at	11.35	9.01	10.67	11.14	10.96	11.22	10.34	1.21	11.11	0.13	1.07	0.103	3.4E-01
Mtr.12487.1.S1_at	8.83	6.46	7.24	7.34	8.33	7.90	7.51	1.21	7.86	0.50	1.05	0.065	6.7E-01
Mtr.41659.1.S1_at	5.90	4.18	6.51	5.67	5.71	5.87	5.53	1.21	5.75	0.10	1.04	0.056	7.7E-01
Mtr.27991.1.S1_at	8.27	6.17	8.24	8.44	8.66	8.72	7.56	1.21	8.61	0.15	1.14	0.187	2.1E-01
Mtr.24228.1.S1_at	10.50	8.09	9.24	9.92	9.78	10.12	9.28	1.21	9.94	0.17	1.07	0.100	4.0E-01
Mtr.32874.1.S1_at	2.45	4.56	2.48	2.43	2.42	2.43	3.16	1.21	2.43	0.00	0.77	-0.384	3.5E-01
Mtr.16807.1.S1_at	2.30	4.38	2.29	2.28	2.28	2.28	2.99	1.21	2.28	0.00	0.76	-0.391	3.7E-01
Mtr.23587.1.S1_at	5.13	3.28	5.54	5.52	4.99	4.65	4.65	1.21	5.05	0.43	1.09	0.120	6.1E-01
Mtr.21659.1.S1_at	3.39	5.45	3.33	3.36	2.95	3.25	4.06	1.21	3.19	0.21	0.79	-0.347	2.9E-01
Mtr.51385.1.S1_a_at	3.22	5.26	3.13	3.16	3.12	3.31	3.87	1.20	3.20	0.10	0.83	-0.275	3.9E-01
Mtr.19552.1.S1_s_at	5.72	4.52	6.93	6.85	6.43	6.05	5.72	1.20	6.44	0.40	1.13	0.171	3.8E-01
Mtr.44450.1.S1_at	9.04	7.38	9.72	10.27	10.18	10.08	8.72	1.20	10.18	0.09	1.17	0.224	1.0E-01
Mtr.38370.1.S1_s_at	6.31	4.08	5.97	5.91	5.64	5.70	5.45	1.20	5.75	0.14	1.05	0.077	6.9E-01
Mtr.24935.1.S1_s_at	7.73	5.82	8.03	7.73	7.49	7.45	7.19	1.20	7.56	0.15	1.05	0.072	6.3E-01
Mtr.5944.1.S1_at	5.91	3.65	5.48	5.62	5.62	5.49	5.01	1.20	5.58	0.07	1.11	0.154	4.6E-01
Mtr.14430.1.S1_at	8.04	6.77	9.17	8.76	8.48	8.20	7.99	1.20	8.48	0.28	1.06	0.085	5.3E-01
Mtr.18771.1.S1_at	6.25	4.85	7.24	6.99	7.03	6.60	6.11	1.20	6.87	0.24	1.12	0.168	3.5E-01
Mtr.39079.1.S1_at	8.97	6.58	7.63	7.85	8.36	7.99	7.72	1.20	8.07	0.27	1.04	0.063	6.5E-01
Mtr.24540.1.S1_s_at	10.34	7.99	9.61	9.61	9.54	9.74	9.31	1.20	9.63	0.10	1.03	0.049	6.7E-01
Mtr.52133.1.S1_at	12.71	10.39	12.06	12.75	12.63	12.78	11.72	1.20	12.72	0.08	1.09	0.118	2.2E-01
Mtr.22226.1.S1_x_at	2.63	4.63	2.49	2.48	2.45	2.48	3.25	1.20	2.47	0.02	0.76	-0.396	3.2E-01
Mtr.2077.1.S1_at	2.57	4.67	2.61	2.60	2.60	2.59	3.28	1.20	2.60	0.00	0.79	-0.338	3.8E-01
Mtr.27699.1.S1_at	7.59	5.90	8.22	7.68	8.07	7.75	7.24	1.20	7.84	0.21	1.08	0.115	4.4E-01
Mtr.49165.1.S1_at	2.85	4.93	2.85	2.89	2.80	2.80	3.54	1.20	2.83	0.05	0.80	-0.324	3.6E-01
Mtr.31020.1.S1_at	5.14	3.06	5.13	5.13	5.13	5.14	4.45	1.20	5.13	0.01	1.15	0.207	3.8E-01
Mtr.35684.1.S1_at	4.79	4.23	6.52	5.41	5.00	4.69	5.18	1.20	5.03	0.36	0.97	-0.042	8.5E-01
Mtr.33664.1.S1_at	5.33	3.11	5.01	4.88	5.68	5.22	4.48	1.20	5.26	0.40	1.17	0.232	3.4E-01
Mtr.14208.1.S1_at	7.74	6.06	8.38	8.05	8.05	8.10	7.40	1.20	8.07	0.03	1.09	0.125	3.9E-01
Mtr.7712.1.S1_at	2.08	4.15	2.07	2.07	2.07	2.07	2.77	1.20	2.07	0.00	0.75	-0.421	3.7E-01
Mtr.6034.1.S1_at	6.58	4.32	6.15	6.32	6.49	6.68	5.68	1.20	6.49	0.18	1.14	0.193	3.1E-01
Mtr.47901.1.S1_x_at	5.88	4.10	6.37	6.14	5.37	5.64	5.45	1.20	5.71	0.39	1.05	0.067	7.4E-01
Mtr.3058.1.S1_at	5.01	6.71	7.31	6.50	6.73	6.92	6.34	1.20	6.72	0.21	1.06	0.083	6.2E-01
Mtr.31303.1.S1_at	3.85	1.65	3.57	4.25	4.05	3.80	3.02	1.19	4.03	0.23	1.33	0.416	2.2E-01
Mtr.25878.1.S1_at	2.62	4.68	2.61	2.59	2.60	2.61	3.30	1.19	2.60	0.01	0.79	-0.346	3.7E-01
Mtr.5492.1.S1_at	7.46	5.42	7.52	7.17	7.28	7.23	6.80	1.19	7.22	0.05	1.06	0.087	5.7E-01
Mtr.39358.1.S1_at	11.05	8.76	10.49	10.30	9.67	9.97	10.10	1.19	9.98	0.31	0.99	-0.017	8.8E-01
Mtr.41998.1.S1_at	5.58	3.30	5.04	5.26	5.47	5.85	4.64	1.19	5.53	0.30	1.19	0.252	2.8E-01
Mtr.27482.1.S1_at	4.80	3.13	5.45	5.54	6.27	5.69	4.46	1.19	5.84	0.39	1.31	0.387	1.3E-01
Mtr.42506.1.S1_at	4.42	2.72	5.02	4.69	4.92	4.10	4.06	1.19	4.57	0.42	1.13	0.172	5.2E-01
Mtr.11949.1.S1_s_at	10.49	8.30	10.22	10.58	10.48	10.49	9.67	1.19	10.51	0.05	1.09	0.121	2.9E-01
Mtr.9521.1.S1_at	11.65	9.71	9.48	10.45	10.67	10.59	10.28	1.19	10.57	0.12	1.03	0.041	6.9E-01
Mtr.34917.1.S1_at	7.17	4.92	6.73	6.67	6.54	6.67	6.28	1.19	6.63	0.08	1.06	0.079	6.4E-01
Mtr.30258.1.S1_at	4.44	2.15	3.87	4.20	4.42	3.80	3.49	1.19	4.14	0.32	1.19	0.247	4.1E-01
Mtr.24042.1.S1_at	2.61	4.64	2.54	2.53	2.53	2.53	3.26	1.19	2.53	0.00	0.78	-0.366	3.5E-01
Mtr.15412.1.S1_at	5.19	2.98	4.85	4.93	4.91	4.60	4.34	1.19	4.81	0.19	1.11	0.150	5.3E-01
Mtr.52247.1.S1_at	6.84	4.54	6.25	6.32	6.20	6.14	5.88	1.19	6.22	0.09	1.06	0.082	6.4E-01
Mtr.48668.1.S1_at	8.16	6.11	8.18	8.53	8.58	8.49	7.48	1.19	8.53	0.05	1.14	0.190	2.0E-01
Mtr.46599.1.S1_x_at	2.69	4.74	2.66	2.64	2.61	2.61	3.36	1.19	2.62	0.02	0.78	-0.361	3.4E-01
Mtr.12258.1.S1_at	3.65	5.64	5.79	6.92	6.77	5.95	5.03	1.19	6.54	0.52	1.30	0.381	1.1E-01
Mtr.30772.1.S1_at	7.93	5.82	7.83	7.94	9.09	8.01	7.19	1.19	8.34	0.64	1.16	0.214	2.2E-01
Mtr.50128.1.S1_at	2.73	4.78	2.70	2.68	2.69	2.68	3.40	1.19	2.68	0.01	0.79	-0.343	3.5E-01
Mtr.45978.1.S1_at	3.00	5.04	2.96	3.08	2.98	3.00	3.67	1.19	3.02	0.05	0.82	-0.280	4.0E-01
Mtr.51539.1.S1_at	2.49	4.50	2.38	2.46	2.47	2.66	3.12	1.19	2.53	0.11	0.81	-0.304	4.4E-01
Mtr.43470.1.S1_at	6.55	4.17	5.53	5.20	5.56	5.26	5.42	1.19	5.34	0.19	0.99	-0.021	9.2E-01
Mtr.39256.1.S1_at	5.76	3.61	5.59	6.29	5.66	5.72	4.99	1.19	5.89	0.35	1.18	0.241	2.7E-01
Mtr.18286.1.S1_at	6.25	4.06	5.97	5.77	6.46	6.24	5.43	1.19	6.16	0.35	1.13	0.181	3.7E-01
Mtr.5521.1.S1_at	6.37	4.31	6.37	7.85	7.07	7.80	5.68	1.19	7.57	0.43	1.33	0.414	6.1E-02

Mtr.7012.1.S1_x_at	6.42	4.32	6.34	6.51	6.52	6.34	5.70	1.19	6.45	0.10	1.13	0.180	3.3E-01
Mtr.41145.1.S1_at	10.69	8.43	10.21	10.64	10.56	10.68	9.78	1.19	10.63	0.06	1.09	0.120	2.8E-01
Mtr.29195.1.S1_at	1.96	4.02	1.95	1.95	1.95	1.95	2.64	1.19	1.95	0.00	0.74	-0.441	3.7E-01
Mtr.35607.1.S1_at	5.39	3.04	4.52	5.96	6.37	6.04	4.32	1.19	6.12	0.22	1.42	0.504	6.1E-02
Mtr.31951.1.S1_s_at	10.84	8.57	9.09	9.52	9.62	9.60	9.50	1.19	9.58	0.05	1.01	0.012	9.1E-01
Mtr.2169.1.S1_at	6.01	3.79	5.62	6.03	5.76	6.41	5.14	1.19	6.07	0.32	1.18	0.239	2.6E-01
Mtr.45131.1.S1_at	2.92	4.98	2.92	3.04	2.91	2.90	3.60	1.19	2.95	0.08	0.82	-0.289	4.0E-01
Mtr.39005.1.S1_at	4.47	2.23	4.05	4.41	3.99	4.08	3.58	1.19	4.16	0.22	1.16	0.215	4.5E-01
Mtr.41599.1.S1_s_at	7.83	5.71	7.71	7.64	7.93	7.77	7.08	1.19	7.78	0.15	1.10	0.135	3.7E-01
Mtr.13657.1.S1_at	9.51	7.70	9.94	9.83	9.99	9.71	9.05	1.19	9.84	0.14	1.09	0.122	3.1E-01
Mtr.40925.1.S1_at	10.27	7.98	9.68	9.95	9.71	9.89	9.31	1.19	9.85	0.13	1.06	0.081	4.8E-01
Mtr.3622.1.S1_at	4.13	1.98	3.94	4.57	5.80	4.50	3.35	1.19	4.96	0.74	1.48	0.566	1.2E-01
Mtr.8034.1.S1_at	2.20	4.25	2.19	2.18	2.12	2.18	2.88	1.19	2.16	0.04	0.75	-0.412	3.6E-01
Mtr.20378.1.S1_at	6.07	4.01	6.07	6.27	6.14	6.31	5.38	1.19	6.24	0.09	1.16	0.214	2.8E-01
Mtr.3798.1.S1_at	11.13	8.95	9.21	10.08	10.52	10.34	9.76	1.19	10.31	0.22	1.06	0.079	4.8E-01
Mtr.45254.1.S1_at	6.78	5.27	7.62	6.91	7.44	7.25	6.56	1.19	7.20	0.27	1.10	0.135	4.1E-01
Mtr.47211.1.S1_at	3.45	5.52	3.48	3.47	3.43	3.42	4.15	1.19	3.44	0.02	0.83	-0.270	3.6E-01
Mtr.50125.1.S1_at	4.91	3.88	6.25	4.98	5.60	4.93	5.01	1.19	5.17	0.37	1.03	0.045	8.4E-01
Mtr.42620.1.S1_at	11.20	8.84	9.77	10.52	10.79	10.68	9.94	1.19	10.66	0.13	1.07	0.102	3.5E-01
Mtr.43280.1.S1_x_at	7.57	5.20	6.19	6.23	6.50	6.65	6.32	1.19	6.46	0.21	1.02	0.031	8.5E-01
Mtr.660.1.S1_at	6.27	4.20	6.25	6.24	6.57	6.25	5.58	1.19	6.36	0.19	1.14	0.189	3.2E-01
Mtr.38403.1.S1_at	6.25	5.49	7.82	6.83	6.77	6.28	6.52	1.19	6.63	0.30	1.02	0.023	8.9E-01
Mtr.51895.1.S1_at	2.53	4.57	2.51	2.48	2.48	2.48	3.20	1.19	2.48	0.00	0.77	-0.370	3.5E-01
Mtr.23440.1.S1_at	3.21	5.25	3.18	3.00	3.16	3.32	3.88	1.19	3.16	0.16	0.81	-0.296	3.6E-01
Mtr.44620.1.S1_at	12.60	10.96	13.26	13.07	13.11	12.76	12.27	1.19	12.98	0.19	1.06	0.081	3.7E-01
Mtr.11959.1.S1_at	8.22	6.12	8.12	8.32	8.94	8.63	7.48	1.19	8.63	0.31	1.15	0.205	1.8E-01
Mtr.4236.1.S1_at	4.67	3.15	5.49	4.72	5.58	4.59	4.44	1.19	4.96	0.54	1.12	0.162	5.2E-01
Mtr.2554.1.S1_at	8.22	5.92	7.59	8.19	8.19	8.64	7.24	1.19	8.34	0.26	1.15	0.204	1.9E-01
Mtr.499.1.S1_s_at	8.76	6.72	8.80	8.89	8.84	8.84	8.09	1.19	8.86	0.03	1.09	0.130	3.3E-01
Mtr.34480.1.S1_at	4.93	2.91	5.00	4.65	4.64	4.63	4.28	1.19	4.64	0.01	1.09	0.118	6.2E-01
Mtr.43575.1.S1_at	6.21	4.83	7.19	7.22	6.75	7.01	6.08	1.19	6.99	0.23	1.15	0.202	2.6E-01
Mtr.14002.1.S1_at	9.26	7.17	9.19	9.12	9.12	9.13	8.54	1.18	9.13	0.01	1.07	0.096	4.4E-01
Mtr.36346.1.S1_at	3.15	5.18	3.11	3.11	2.81	3.11	3.81	1.18	3.01	0.17	0.79	-0.343	3.1E-01
Mtr.12427.1.S1_at	6.52	4.61	6.78	6.76	7.20	6.41	5.97	1.18	6.79	0.40	1.14	0.185	3.2E-01
Mtr.34958.1.S1_at	10.17	7.90	8.44	8.72	8.01	8.81	8.83	1.18	8.51	0.44	0.96	-0.053	6.8E-01
Mtr.37893.1.S1_at	6.65	4.91	7.17	7.36	8.42	7.88	6.25	1.18	7.89	0.53	1.26	0.336	9.4E-02
Mtr.24012.1.S1_at	3.19	5.19	3.09	3.14	3.08	3.04	3.82	1.18	3.09	0.05	0.81	-0.308	3.4E-01
Mtr.33004.1.S1_at	8.42	6.07	7.49	7.84	7.63	7.90	7.33	1.18	7.79	0.14	1.06	0.088	5.4E-01
Mtr.51968.1.S1_at	4.27	5.32	2.96	3.26	3.72	3.24	4.18	1.18	3.40	0.27	0.81	-0.297	3.3E-01
Mtr.13644.1.S1_s_at	7.21	4.99	6.81	7.24	7.01	6.88	6.33	1.18	7.05	0.18	1.11	0.154	3.6E-01
Mtr.48723.1.S1_at	9.15	6.78	7.97	8.76	8.56	9.02	7.97	1.18	8.78	0.23	1.10	0.140	3.1E-01
Mtr.39496.1.S1_at	5.41	3.65	5.89	5.58	5.55	5.54	4.98	1.18	5.56	0.02	1.11	0.157	4.5E-01
Mtr.20193.1.S1_at	2.51	4.54	2.47	2.47	2.46	2.45	3.18	1.18	2.46	0.01	0.77	-0.368	3.5E-01
Mtr.16841.1.S1_at	9.11	7.02	9.02	9.16	9.19	9.00	8.38	1.18	9.12	0.10	1.09	0.121	3.4E-01
Mtr.2495.1.S1_at	8.16	6.33	8.54	7.90	8.02	7.97	7.68	1.18	7.96	0.06	1.04	0.053	7.0E-01
Mtr.1320.1.S1_at	6.96	5.05	7.22	7.31	6.98	7.18	6.41	1.18	7.16	0.16	1.12	0.159	3.4E-01
Mtr.8580.1.S1_at	7.74	5.92	8.14	7.85	7.36	7.65	7.27	1.18	7.62	0.24	1.05	0.069	6.4E-01
Mtr.38278.1.S1_at	6.75	4.48	6.21	6.57	6.38	6.47	5.81	1.18	6.47	0.10	1.11	0.155	3.9E-01
Mtr.30206.1.S1_at	6.93	5.46	7.79	8.25	9.15	8.56	6.73	1.18	8.65	0.46	1.29	0.364	5.8E-02
Mtr.12031.1.S1_at	8.67	6.62	8.66	8.61	8.25	8.36	7.98	1.18	8.41	0.18	1.05	0.074	5.7E-01
Mtr.47794.1.S1_at	7.96	6.06	8.23	8.42	8.27	8.12	7.41	1.18	8.27	0.15	1.12	0.158	2.8E-01
Mtr.20711.1.S1_at	2.83	4.87	2.82	2.81	2.82	3.10	3.51	1.18	2.91	0.17	0.83	-0.268	4.4E-01
Mtr.4900.1.S1_at	9.69	7.34	8.75	8.77	8.92	8.61	8.59	1.18	8.77	0.15	1.02	0.029	8.1E-01
Mtr.37501.1.S1_at	6.32	5.02	7.38	7.98	7.08	8.12	6.24	1.18	7.72	0.56	1.24	0.308	1.2E-01
Mtr.5466.1.S1_at	7.86	5.57	7.23	7.14	7.30	7.54	6.89	1.18	7.33	0.20	1.06	0.089	5.6E-01
Mtr.35816.1.S1_at	8.21	5.85	7.03	8.70	8.26	8.59	7.03	1.18	8.52	0.23	1.21	0.278	9.8E-02
Mtr.8866.1.S1_at	12.55	10.23	11.76	12.32	11.78	12.47	11.52	1.18	12.19	0.36	1.06	0.082	4.0E-01
Mtr.35147.1.S1_s_at	11.11	9.03	11.04	11.18	11.07	11.08	10.39	1.18	11.11	0.06	1.07	0.096	3.5E-01
Mtr.6863.1.S1_at	7.12	4.77	6.18	6.19	6.36	6.58	6.02	1.18	6.38	0.20	1.06	0.083	6.3E-01
Mtr.35181.1.S1_at	8.41	6.06	7.43	8.30	7.62	8.15	7.30	1.18	8.02	0.36	1.10	0.137	3.7E-01
Mtr.41781.1.S1_at	7.66	5.88	8.12	7.75	7.76	7.69	7.22	1.18	7.73	0.04	1.07	0.099	4.9E-01
Mtr.11801.1.S1_at	8.87	6.99	9.17	8.88	8.83	8.84	8.34	1.18	8.85	0.03	1.06	0.085	5.0E-01
Mtr.44884.1.S1_at	8.90	6.77	8.71	8.75	8.61	8.66	8.13	1.18	8.67	0.07	1.07	0.094	4.7E-01
Mtr.38559.1.S1_at	9.27	6.92	7.96	8.13	7.85	8.05	8.05	1.18	8.01	0.14	1.00	-0.007	9.6E-01
Mtr.49912.1.S1_at	3.30	5.33	3.28	3.27	3.22	3.27	3.97	1.18	3.25	0.02	0.82	-0.287	3.5E-01
Mtr.41126.1.S1_at	8.13	6.02	7.97	8.25	8.12	7.88	7.37	1.18	8.08	0.19	1.10	0.132	3.6E-01
Mtr.3813.1.S1_at	5.90	3.55	4.82	5.87	7.67	6.45	4.75	1.18	6.66	0.92	1.40	0.487	9.1E-02
Mtr.1590.1.S1_at	7.26	5.31	7.42	7.47	7.51	7.38	6.67	1.18	7.45	0.07	1.12	0.161	3.1E-01
Mtr.7670.1.S1_at	6.75	9.05	7.45	8.70	10.48	10.47	7.75	1.18	9.88	1.02	1.28	0.351	7.7E-02
Mtr.6324.1.S1_at	6.19	3.93	4.49	4.97	4.22	4.11	4.87	1.18	4.43	0.47	0.91	-0.136	5.8E-01
Mtr.4614.1.S1_at	4.40	4.71	6.57	5.79	6.11	5.56	5.22	1.18	5.82	0.28	1.11	0.156	4.4E-01
Mtr.14164.1.S1_at	2.46	4.48	2.43	2.43	2.42	2.43	3.13	1.18	2.43	0.00	0.78	-0.365	3.6E-01
Mtr.9818.1.S1_at	11.64	10.65	9.30	10.64	10.41	11.17	10.53	1.17	10.74	0.39	1.02	0.029	7.8E-01
Mtr.32768.1.S1_at	6.12	3.97	5.87	6.33	6.03	6.43	5.32	1.17	6.26	0.21	1.18	0.235	2.4E-01
Mtr.13836.1.S1_at	6.79	4.74	6.75	6.58	6.25	6.36	6.09	1.17	6.40	0.17	1.05	0.070	6.8E-01
Mtr.44432.1.S1_at	4.37	6.47	4.50	4.93	4.77	4.22	5.11	1.17	4.64	0.37	0.91	-0.139	5.4E-01
Mtr.31456.1.S1_at	7.49	5.23	6.93	6.71	6.74	6.41	6.55	1.17	6.62	0.18	1.01	0.015	9.2E-01
Mtr.31088.1.S1_at	3.34	5.37	3.33	3.23	3.33	3.32	4.01	1.17	3.29	0.05	0.82	-0.286	3.5E-01
Mtr.11858.1.S1_at	2.26	4.29	2.25	2.25	2.24	2.25	2.93	1.17	2.25	0.01	0.77	-0.385	3.7E-01
Mtr.30784.1.S1_at	4.48	2.97	5.27	4.90	5.31	5.02	4.24	1.17	5.08	0.21	1.20	0.261	2.9E-01
Mtr.13623.1.S1_at	4.33	3.75	6.01	4.81	4.27	4.26	4.69	1.17	4.44	0.31	0.95	-0.079	7.4E-01
Mtr.42357.1.S1_at	8.29	6.00	7.58	7.71	7.62	7.78	7.29	1.17	7.70	0.08	1.06	0.080	5.7E-01
Mtr.13625.1.S1_at	8.52	6.33	8.15	8.34	8.31	7.99	7.67	1.17	8.21	0.20	1.07	0.099	4.7E-01
Mtr.22746.1.S1_at	6.08	4.11	6.19	6.29	6.27	6.18	5.46	1.17	6.25	0.06	1.14	0.194	3.1E-01
Mtr.13401.1.S1_at	6.95	4.77	5.12	5.62	6.39	6.27	5.61	1.17	6.09	0.42	1.09	0.118	5.4E-01
Mtr.40716.1.S1_at	12.32	10.00	11.45	11.83	12.24	12.08	11.26	1.17	12.05	0.21	1.07	0.098	3.1E-01
Mtr.45487.1.S1_at	7.46	5.28	7.10	7.34	7.36	7.20	6.61	1.17	7.30	0.09	1.10	0.142	3.7E-01
Mtr.36341.1.S1_s_at	8.15	6.34	8.54	8.69	8.37	8.09	7.68	1.17	8.38	0.30	1.09	0.127	3.7E-01
Mtr.28893.1.S1_at	2.56	4.58	2.55	2.54	2.52	2.53	3.23	1.17	2.53	0.01	0.78	-0.352	3.6E-01
Mtr.25523.1.S1_at	2.81	4.84	2.81	2.80	2.61	2.95	3.49	1.17	2.79	0.17	0.80	-0.323	3.6E-01
Mtr.34881.1.S1_at	2.85	4.76	2.63	2.62	2.84	2.62	3.41	1.17	2.69	0.13	0.79	-0.342	3.5E-01
Mtr.5928.1.S1_at	6.45	5.23	7.57	7.35	7.02	7.35	6.42	1.17	7.24	0.19	1.13	0.174	3.0E-01
Mtr.28728.1.S1_at	8.04	6.02	8.06	8.25	8.21	8.25	7.37	1.17	8.23	0.02	1.12	0.159	2.7E-01
Mtr.9568.1.S1_at	10.09	7.84	9.52	9.29	8.68	9.17	9.15	1.17	9.05	0.33	0.99	-0.016	8.9E-01
Mtr.12800.1.S1_at	12.00	9.85	10.11	10.65	10.71	10.86	10.65	1.17	10.74	0.11	1.01	0.012	9.0E-01
Mtr.20151.1.S1_at	5.37	3.54	5.71	5.36	5.17	5.28	4.87	1.17	5.27	0.09	1.08	0.112	5.9E-01
Mtr.41669.1.S1_at	8.39	6.23	8.09	8.43	8.58	8.49	7.57	1.17	8.50	0.07	1.12	0.168	2.4E-01
Mtr.38871.1.S1_at	5.15	3.83	2.82	3.87	3.84	4.42	3.94	1.17	4.04	0.33	1.03	0.039	8.9E-01
Mtr.14297.1.S1_at	4.08	6.16	4.19	4.07	3.91	4.18	4.81	1.17	4.05	0.14	0.84	-0.246	3.3E-01
Mtr.46947.1.S1_at	2.33	4.35	2.33	2.32	2.31	2.30	3.00	1.17	2.31	0.01	0.77	-0.379	3.6E-01
Mtr.27829.1.S1_at	6.88	4.87	6.91	6.51	6.84	6.64	6.22	1.17	6.67	0.17	1.07	0.100	5.5E-01
Mtr.40866.1.S1_at	7.22	5.26	7.35	7.31	7.14	7.02	6.61	1.17	7.16	0.15	1.08	0.115	4.6E-01
Mtr.11118.1.S1_at	10.02	8.12	7.89	8.28	8.56	8.24	8.68	1.17	8.36	0.17	0.96	-0.053	6.7E-01
Mtr.46430.1.S1_at	2.53	4.56	2.54	2.51	2.52	2.48	3.21	1.17	2.50	0.02	0.78	-0.359	3.5E-01
Mtr.43681.1.S1_at	11.18	9.10	11.07	11.80	11.88	11.85	10.45	1.17	11.84	0.04	1.13	0.180	1.1E-01
Mtr.14985.1.S1_at	2.03	4.25	3.76	4.39	4.19	4.45	3.35	1.17	4.34	0.14	1.30	0.375	2.2E-01
Mtr.11604.1.S1_s_at	7.26	5.46	7.65	7.65	7.61	7.45	6.79	1.17	7.57	0.11	1.11	0.156	3.2E-01
Mtr.21339.1.S1_at	2.75	4.76	2.74	2.93	2.72	2.73	3.42	1.17	2.80	0.12	0.82	-0.289	4.1E-01
Mtr.34190.1.S1_at	6.03	4.17	6.31	7.21	7.94	7.46	5.50	1.17	7.54	0.37	1.37	0.454	4.5E-02
Mtr.3428.1.S1_at	6.18	5.49	7.77	7.15	7.65	6.56	6.48	1.17	7.12	0.54	1.10	0.136	4.4E-01
Mtr.824.1.S1_s_at	6.41	4.65	6.85	6.60	6.21	6.34	5.97	1.17	6.38	0.20	1.07	0.097	5.8E-01
Mtr.19318.1.S1_at	6.96	5.45	7.75	7.33	7.62	7.32	6.72	1.17	7.42	0.17	1.10	0.143	3.6E-01
Mtr.28807.1.S1_at	6.70	5.13	7.41	6.99	6.94	7.24	6.41	1.17	7.05	0.16	1.10	0.137	4.0E-01
Mtr.24335.1.S1_at	8.25	10.17	8.06	8.09	7.96	8.29	8.82	1.17	8.11	0.17	0.92	-0.121	3.5E-01
Mtr.6939.1.S1_at	5.42	3.38	5.39	5.44	5.59	5.38	4.73	1.17	5.47	0.11	1.16	0.209	3.4E-01
Mtr.31139.1.S1_at	5.89	4.02	6.16	6.32	6.18	5.47	5.36	1.17	5.99	0.45	1.12	0.162	4.3E-01
Mtr.6048.1.S1_s_at	7.26	5.40	7.55	6.60	6.58	6.57	6.74	1.17	6.59	0.02	0.98	-0.033	8.3E-01
Mtr.18929.1.S1_at	7.61	5.74	7.88	7.77	7.71	7.69	7.08	1.17	7.72	0.04	1.09	0.126	3.9E-01
Mtr.14709.1.S1_at	7.12	5.42	7.65	7.28	7.10	7.41	6.73	1.16	7.26	0.16	1.08	0.109	4.8E-01
Mtr.37858.1.S1_s_at	7.79	5.74	7.72	8.08	7.91	7.93	7.08	1.16	7.97	0.09	1.13	0.171	2.6E-01
Mtr.25344.1.S1_at	10.20	8.17	10.17	9.36	9.64	9.30	9.51	1.16	9.43	0.18	0.99	-0.013	9.1E-01
Mtr.39193.1.S1_at	3.45	2.01	4.32	3.07	3.44	4.12	3.26	1.16	3.54	0.53	1.09	0.120	7.2E-01
Mtr.21857.1.S1_at	8.48	6.40	8.34	8.56	8.49	8.24	7.74	1.16	8.43	0.17	1.09	0.123	3.7E-01
Mtr.26262.1.S1_at	6.74	5.46	7.79	7.40	7.23	7.06	6.66	1.16	7.23	0.17	1.09	0.118	4.5E-01
Mtr.16200.1.S1_at	8.21	6.14	8.11	7.86	7.64	7.03	7.49	1.16	7.51	0.43	1.00	0.005	9.8E-01
Mtr.43091.1.S1_s_at	6.00	4.02	3.96	3.95	3.55	3.94	4.66	1.16	3.82	0.23	0.82	-0.288	2.8E-01
Mtr.1823.1.S1_at	2.67	4.68	2.66	2.68	2.66	2.65	3.34	1.16	2.66	0.01	0.80	-0.325	3.7E-01
Mtr.41197.1.S1_at	5.83	3.67	4.01	5.38	5.19	5.51	4.50	1.16	5.36	0.16	1.19	0.250	2.8E-01
Mtr.13329.1.S1_at	5.95	3.69	4.34	4.89	5.25	5.45	4.66	1.16	5.20	0.29	1.12	0.157	4.8E-01
Mtr.24085.1.S1_at	5.25	3.28	3.20	3.07	3.06	3.25	3.91	1.16	3.13	0.11	0.80	-0.321	3.1E-01
Mtr.12471.1.S1_at	5.60	3.41	5.17	5.71	5.51	5.85	4.72	1.16	5.69	0.17	1.20	0.268	2.3E-01

Mtr.44848.1.S1_at	8.30	6.08	7.79	8.20	8.13	8.36	7.39	1.16	8.23	0.12	1.11	0.156	2.8E-01
Mtr.19391.1.S1_at	2.92	4.92	2.90	2.90	2.90	2.88	3.58	1.16	2.89	0.01	0.81	-0.308	3.6E-01
Mtr.5815.1.S1_s_at	3.94	2.26	4.49	5.15	4.72	4.76	3.56	1.16	4.88	0.24	1.37	0.453	1.3E-01
Mtr.30766.1.S1_at	6.95	5.42	7.70	7.47	7.49	7.20	6.69	1.16	7.39	0.16	1.10	0.143	3.6E-01
Mtr.43879.1.S1_at	7.44	5.35	7.27	7.31	7.14	7.70	6.69	1.16	7.38	0.29	1.10	0.143	3.7E-01
Mtr.10434.1.S1_at	11.91	9.59	10.62	11.13	11.00	11.43	10.71	1.16	11.19	0.22	1.05	0.064	5.2E-01
Mtr.5731.1.S1_at	6.48	4.34	4.65	5.33	5.27	5.56	5.16	1.16	5.39	0.15	1.04	0.062	7.5E-01
Mtr.21742.1.S1_at	2.28	4.29	2.27	2.21	2.27	2.22	2.95	1.16	2.23	0.03	0.76	-0.400	3.5E-01
Mtr.20616.1.S1_at	2.31	4.32	2.31	2.29	2.31	2.30	2.98	1.16	2.30	0.01	0.77	-0.375	3.7E-01
Mtr.9495.1.S1_at	8.40	6.83	9.09	8.76	8.79	8.43	8.11	1.16	8.66	0.20	1.07	0.095	4.6E-01
Mtr.598.1.S1_at	6.87	4.56	5.81	6.77	7.52	7.13	5.75	1.16	7.14	0.38	1.24	0.313	1.2E-01
Mtr.42645.1.S1_at	5.08	4.94	7.01	5.71	6.01	5.69	5.68	1.16	5.80	0.18	1.02	0.032	8.6E-01
Mtr.46001.1.S1_s_at	10.93	8.66	10.23	9.83	9.46	9.92	9.94	1.16	9.74	0.25	0.98	-0.030	7.8E-01
Mtr.1921.1.S1_at	7.28	5.22	7.18	6.93	6.73	6.94	6.56	1.16	6.87	0.12	1.05	0.067	6.7E-01
Mtr.47380.1.S1_at	7.52	5.30	6.98	7.04	7.33	7.14	6.60	1.16	7.17	0.15	1.09	0.120	4.4E-01
Mtr.37283.1.S1_s_at	6.42	4.16	5.73	7.00	7.21	7.35	5.43	1.16	7.19	0.18	1.32	0.404	6.0E-02
Mtr.15046.1.S1_at	7.84	5.53	6.90	7.04	6.98	6.74	6.76	1.16	6.92	0.16	1.02	0.034	8.2E-01
Mtr.3735.1.S1_at	2.11	4.10	2.08	2.10	2.10	2.08	2.77	1.16	2.09	0.01	0.76	-0.403	3.7E-01
Mtr.15253.1.S1_at	2.47	4.46	2.45	2.45	2.45	2.45	3.13	1.16	2.45	0.00	0.78	-0.353	3.7E-01
Mtr.26482.1.S1_at	4.44	4.35	6.40	4.31	4.38	4.69	5.06	1.16	4.46	0.20	0.88	-0.182	4.3E-01
Mtr.52056.1.S1_s_at	7.27	5.88	8.18	7.97	7.84	7.71	7.11	1.16	7.84	0.13	1.10	0.142	3.4E-01
Mtr.22685.1.S1_at	4.20	2.20	2.20	3.60	3.49	3.50	2.87	1.16	3.53	0.06	1.23	0.302	3.7E-01
Mtr.12014.1.S1_at	4.39	6.54	4.74	5.58	6.17	5.25	5.22	1.16	5.67	0.46	1.09	0.118	5.7E-01
Mtr.5959.1.S1_at	5.70	3.67	5.65	5.99	5.84	5.44	5.01	1.16	5.76	0.29	1.15	0.201	3.4E-01
Mtr.29046.1.S1_at	5.43	3.49	5.55	5.00	6.15	6.42	4.82	1.16	5.86	0.76	1.21	0.280	2.7E-01
Mtr.10353.1.S1_at	9.34	11.31	9.27	9.91	9.81	9.73	9.97	1.16	9.81	0.09	0.98	-0.023	8.2E-01
Mtr.23292.1.S1_x_at	2.48	4.47	2.47	2.47	2.47	2.47	3.14	1.15	2.47	0.00	0.79	-0.349	3.7E-01
Mtr.28846.1.S1_at	5.40	3.26	5.07	4.28	4.27	3.47	4.58	1.15	4.01	0.47	0.88	-0.192	4.7E-01
Mtr.21631.1.S1_at	4.83	3.39	5.68	4.77	5.07	4.62	4.64	1.15	4.82	0.23	1.04	0.057	8.0E-01
Mtr.4441.1.S1_at	8.16	5.86	7.17	8.19	8.71	8.49	7.06	1.15	8.46	0.26	1.20	0.261	1.1E-01
Mtr.35532.1.S1_at	11.04	8.76	10.20	10.52	10.18	10.57	10.00	1.15	10.42	0.21	1.04	0.059	5.7E-01
Mtr.28695.1.S1_at	2.67	4.67	2.67	2.65	2.78	2.65	3.34	1.15	2.70	0.08	0.81	-0.309	3.9E-01
Mtr.15077.1.S1_at	2.64	4.63	2.63	2.61	2.63	2.62	3.30	1.15	2.62	0.01	0.79	-0.332	3.7E-01
Mtr.35796.1.S1_at	11.56	11.54	9.55	10.93	10.92	10.40	10.89	1.15	10.75	0.30	0.99	-0.018	8.5E-01
Mtr.9857.1.S1_at	9.99	7.68	8.93	9.57	9.68	9.84	8.87	1.15	9.69	0.14	1.09	0.128	2.9E-01
Mtr.29984.1.S1_at	6.60	4.88	7.07	6.77	6.89	6.63	6.18	1.15	6.76	0.13	1.09	0.129	4.4E-01
Mtr.13596.1.S1_at	4.54	2.93	5.17	4.81	5.04	4.36	4.22	1.15	4.73	0.35	1.12	0.167	5.0E-01
Mtr.4664.1.S1_s_at	7.98	6.08	8.16	8.09	8.22	8.11	7.41	1.15	8.14	0.07	1.10	0.136	3.3E-01
Mtr.18411.1.S1_s_at	2.63	4.62	2.60	2.58	2.57	2.55	3.28	1.15	2.57	0.02	0.78	-0.355	3.4E-01
Mtr.42288.1.S1_at	7.98	6.96	9.26	8.78	8.86	8.54	8.07	1.15	8.73	0.17	1.08	0.114	3.8E-01
Mtr.45126.1.S1_at	4.67	2.98	5.18	3.94	4.04	3.83	4.28	1.15	3.94	0.11	0.92	-0.119	6.4E-01
Mtr.48119.1.S1_at	2.51	4.49	2.49	2.48	2.48	2.48	3.16	1.15	2.48	0.00	0.78	-0.350	3.6E-01
Mtr.50100.1.S1_at	2.84	4.80	2.77	2.76	2.79	2.83	3.47	1.15	2.79	0.03	0.80	-0.313	3.7E-01
Mtr.23940.1.S1_at	9.28	7.16	9.01	8.75	8.32	8.73	8.48	1.15	8.60	0.24	1.01	0.020	8.7E-01
Mtr.34842.1.S1_at	4.87	2.78	4.66	4.20	4.50	4.09	4.11	1.15	4.27	0.21	1.04	0.055	8.3E-01
Mtr.2843.1.S1_at	7.29	4.99	6.29	6.67	6.64	6.53	6.19	1.15	6.62	0.07	1.07	0.096	5.6E-01
Mtr.39510.1.S1_s_at	7.92	6.04	8.13	7.85	7.92	7.92	7.36	1.15	7.90	0.04	1.07	0.101	4.7E-01
Mtr.32738.1.S1_s_at	4.65	6.43	4.27	4.27	3.78	3.41	5.12	1.15	3.82	0.43	0.75	-0.421	1.4E-01
Mtr.23614.1.S1_at	10.46	8.22	9.82	9.73	9.73	9.87	9.50	1.15	9.77	0.08	1.03	0.041	7.0E-01
Mtr.10964.1.S1_at	9.46	7.33	9.14	8.57	9.22	8.78	8.64	1.15	8.85	0.33	1.02	0.035	7.8E-01
Mtr.33135.1.S1_at	6.48	4.25	5.87	6.74	6.34	6.36	5.53	1.15	6.48	0.23	1.17	0.227	2.4E-01
Mtr.1581.1.S1_at	2.51	4.49	2.48	2.48	2.47	2.45	3.16	1.15	2.47	0.01	0.78	-0.359	3.5E-01
Mtr.32424.1.S1_at	7.56	5.34	6.98	6.99	7.32	7.36	6.62	1.15	7.22	0.20	1.09	0.125	4.3E-01
Mtr.47111.1.S1_at	5.91	4.07	6.19	5.51	5.61	4.85	5.39	1.15	5.32	0.42	0.99	-0.019	9.3E-01
Mtr.26511.1.S1_at	3.09	5.00	2.93	2.82	2.89	2.87	3.67	1.15	2.86	0.03	0.78	-0.361	2.9E-01
Mtr.34815.1.S1_at	5.12	3.38	5.56	5.13	5.24	4.86	4.69	1.15	5.08	0.20	1.08	0.116	5.9E-01
Mtr.50730.1.S1_at	8.61	6.86	9.03	9.72	10.26	9.89	8.16	1.15	9.96	0.28	1.22	0.286	5.8E-02
Mtr.29094.1.S1_s_at	5.06	4.62	6.79	5.40	5.04	5.03	5.49	1.15	5.16	0.21	0.94	-0.091	6.5E-01
Mtr.48230.1.S1_at	4.05	5.77	3.59	4.05	3.54	3.53	4.47	1.15	3.70	0.30	0.83	-0.270	3.3E-01
Mtr.36639.1.S1_at	2.36	4.35	2.36	2.36	2.35	2.33	3.02	1.15	2.34	0.02	0.78	-0.367	3.6E-01
Mtr.15800.1.S1_s_at	6.15	4.68	6.94	6.68	6.58	6.59	5.92	1.15	6.62	0.05	1.12	0.160	3.5E-01
Mtr.11345.1.S1_at	4.97	3.75	6.05	2.79	3.67	3.62	4.92	1.15	3.36	0.49	0.68	-0.552	9.6E-02
Mtr.44844.1.S1_at	10.42	8.28	8.64	8.66	8.01	8.33	9.11	1.15	8.33	0.33	0.91	-0.129	3.2E-01
Mtr.38398.1.S1_at	5.90	3.62	4.95	6.10	5.92	6.09	4.82	1.15	6.03	0.10	1.25	0.323	1.4E-01
Mtr.19598.1.S1_at	7.96	5.86	6.11	5.59	5.16	5.69	6.64	1.15	5.48	0.28	0.82	-0.278	1.6E-01
Mtr.40252.1.S1_x_at	10.92	8.87	9.00	9.75	9.95	9.88	9.59	1.15	9.86	0.10	1.03	0.040	7.1E-01
Mtr.45251.1.S1_at	2.40	4.37	2.37	2.37	2.36	2.36	3.05	1.15	2.36	0.00	0.78	-0.366	3.6E-01
Mtr.31209.1.S1_s_at	3.62	2.55	4.84	3.47	3.47	3.46	3.67	1.15	3.47	0.01	0.94	-0.082	7.7E-01
Mtr.34610.1.S1_at	7.30	5.20	7.05	7.19	6.91	7.20	6.52	1.15	7.10	0.16	1.09	0.124	4.3E-01
Mtr.11776.1.S1_at	7.63	5.33	6.46	6.70	6.77	7.04	6.47	1.15	6.83	0.18	1.06	0.078	6.2E-01
Mtr.48694.1.S1_at	5.79	3.63	5.36	3.82	3.79	3.75	4.93	1.15	3.79	0.03	0.77	-0.380	1.6E-01
Mtr.1560.1.S1_s_at	7.64	5.48	7.22	7.14	7.23	7.07	6.78	1.15	7.15	0.08	1.05	0.076	6.1E-01
Mtr.5566.1.S1_x_at	5.92	3.68	5.24	5.22	5.70	5.43	4.95	1.14	5.45	0.24	1.10	0.140	5.0E-01
Mtr.47184.1.S1_at	2.79	4.75	2.75	3.14	3.05	2.72	3.43	1.14	2.97	0.22	0.87	-0.209	5.3E-01
Mtr.2780.1.S1_at	4.43	2.37	4.26	4.46	4.44	4.28	3.68	1.14	4.39	0.10	1.19	0.254	3.4E-01
Mtr.44410.1.S1_at	5.07	3.11	3.07	3.06	3.06	3.07	3.75	1.14	3.07	0.00	0.82	-0.290	3.6E-01
Mtr.34402.1.S1_at	6.14	4.15	6.13	6.13	6.51	6.13	5.48	1.14	6.26	0.22	1.14	0.192	3.1E-01
Mtr.35945.1.S1_at	12.79	10.79	10.82	12.03	11.34	12.23	11.47	1.14	11.87	0.47	1.03	0.049	6.1E-01
Mtr.45146.1.S1_at	8.00	6.69	8.97	8.00	8.25	7.29	7.89	1.14	7.84	0.50	0.99	-0.007	9.6E-01
Mtr.42942.1.S1_at	10.10	7.81	9.01	8.99	8.64	9.12	8.97	1.14	8.92	0.25	0.99	-0.008	9.4E-01
Mtr.34883.1.S1_s_at	9.21	7.13	8.99	8.82	8.68	8.82	8.44	1.14	8.77	0.08	1.04	0.056	6.4E-01
Mtr.4069.1.S1_at	7.11	5.19	7.23	7.16	7.13	6.92	6.51	1.14	7.07	0.13	1.09	0.119	4.5E-01
Mtr.1787.1.S1_at	5.80	3.56	5.09	5.78	6.20	5.41	4.82	1.14	5.80	0.39	1.20	0.266	2.3E-01
Mtr.12337.1.S1_at	6.55	4.31	5.86	5.81	6.54	6.40	5.57	1.14	6.25	0.39	1.12	0.164	3.9E-01
Mtr.44280.1.S1_at	10.02	8.09	10.11	10.78	10.19	10.72	9.40	1.14	10.57	0.32	1.12	0.168	1.7E-01
Mtr.6372.1.S1_at	6.39	4.37	6.30	6.28	6.87	6.21	5.68	1.14	6.45	0.36	1.13	0.182	3.3E-01
Mtr.50457.1.S1_s_at	6.17	4.79	7.05	7.39	7.45	7.57	6.00	1.14	7.47	0.09	1.24	0.315	9.1E-02
Mtr.44883.1.S1_at	7.18	5.14	7.05	7.64	7.21	7.59	6.46	1.14	7.48	0.23	1.16	0.211	2.0E-01
Mtr.50858.1.S1_s_at	10.26	8.06	9.69	9.74	9.48	9.98	9.33	1.14	9.73	0.25	1.04	0.061	5.9E-01
Mtr.25373.1.S1_at	7.46	5.39	7.26	7.78	7.31	7.37	6.70	1.14	7.49	0.25	1.12	0.160	3.1E-01
Mtr.19709.1.S1_at	7.30	5.14	6.87	6.24	6.71	6.53	6.44	1.14	6.49	0.24	1.01	0.011	9.4E-01
Mtr.24400.1.S1_x_at	2.92	4.89	2.90	2.93	2.89	2.89	3.57	1.14	2.90	0.02	0.81	-0.299	3.7E-01
Mtr.13399.1.S1_s_at	7.83	5.73	7.56	7.44	7.62	7.58	7.04	1.14	7.54	0.10	1.07	0.100	4.9E-01
Mtr.31100.1.S1_s_at	7.44	9.22	7.10	6.93	7.07	7.51	7.92	1.14	7.17	0.30	0.90	-0.144	3.3E-01
Mtr.21307.1.S1_at	5.54	3.73	5.84	8.04	8.19	8.08	5.04	1.14	8.10	0.08	1.61	0.686	9.7E-03
Mtr.38853.1.S1_at	4.76	2.78	4.75	4.75	5.60	5.25	4.10	1.14	5.20	0.43	1.27	0.343	1.9E-01
Mtr.43717.1.S1_at	9.40	8.01	10.27	9.58	9.64	9.15	9.23	1.14	9.46	0.27	1.02	0.035	7.5E-01
Mtr.16311.1.S1_at	6.93	5.02	7.04	6.47	6.46	6.19	6.33	1.14	6.37	0.16	1.01	0.010	9.5E-01
Mtr.45389.1.S1_at	4.41	2.69	4.85	4.40	4.40	4.36	3.98	1.14	4.39	0.02	1.10	0.139	5.7E-01
Mtr.28632.1.S1_at	3.94	3.85	5.87	8.12	8.90	8.37	4.55	1.14	8.46	0.40	1.86	0.895	4.9E-03
Mtr.39159.1.S1_at	8.78	6.83	8.82	8.91	8.93	8.95	8.14	1.14	8.93	0.02	1.10	0.133	3.0E-01
Mtr.23012.1.S1_at	7.14	5.37	7.50	6.95	6.97	6.93	6.67	1.14	6.95	0.02	1.04	0.059	6.9E-01
Mtr.9255.1.S1_at	4.71	2.71	4.65	6.19	6.87	6.88	4.03	1.14	6.65	0.40	1.65	0.724	2.0E-02
Mtr.350.1.S1_at	6.87	4.69	6.34	5.58	6.10	5.78	5.96	1.14	5.82	0.26	0.98	-0.035	8.4E-01
Mtr.45013.1.S1_at	11.46	9.31	11.04	11.15	11.03	10.90	10.60	1.14	11.03	0.13	1.04	0.056	5.6E-01
Mtr.8637.1.S1_at	3.20	3.20	5.17	3.10	3.09	2.83	3.85	1.14	3.01	0.16	0.78	-0.359	2.7E-01
Mtr.43948.1.S1_at	5.59	4.33	6.61	8.74	8.89	8.03	5.51	1.14	8.55	0.46	1.55	0.634	1.3E-02
Mtr.15402.1.S1_at	6.07	4.03	5.92	6.53	5.74	5.92	5.34	1.14	6.06	0.41	1.14	0.184	3.6E-01
Mtr.17964.1.S1_at	8.78	6.61	8.27	8.47	8.53	8.42	7.89	1.14	8.47	0.05	1.07	0.103	4.2E-01
Mtr.31881.1.S1_at	6.50	4.82	7.00	5.98	5.98	5.97	6.11	1.14	5.98	0.01	0.98	-0.031	8.5E-01
Mtr.27998.1.S1_at	6.51	4.56	6.55	7.14	7.64	7.56	5.87	1.14	7.45	0.27	1.27	0.342	8.0E-02
Mtr.31368.1.S1_at	7.41	5.46	7.45	6.72	7.53	7.25	6.77	1.14	7.17	0.41	1.06	0.081	6.0E-01
Mtr.4591.1.S1_at	6.03	3.79	5.23	5.98	5.77	5.76	5.01	1.14	5.84	0.13	1.16	0.219	2.8E-01
Mtr.16622.1.S1_at	8.35	6.69	8.86	8.43	8.44	8.27	7.97	1.14	8.38	0.10	1.05	0.073	5.6E-01
Mtr.8409.1.S1_at	8.48	7.12	9.37	8.97	9.27	8.93	8.32	1.14	9.06	0.19	1.09	0.122	3.3E-01
Mtr.43988.1.S1_at	9.82	8.09	10.23	9.95	10.04	10.31	9.38	1.14	10.10	0.19	1.08	0.107	3.4E-01
Mtr.6541.1.S1_at	2.21	4.18	2.20	2.19	2.13	2.20	2.87	1.14	2.17	0.04	0.76	-0.399	3.5E-01
Mtr.45570.1.S1_at	8.40	6.48	8.50	8.62	8.44	8.45	7.80	1.14	8.50	0.10	1.09	0.125	3.5E-01
Mtr.19259.1.S1_x_at	2.53	4.49	2.51	2.49	2.48	3.38	3.18	1.14	2.78	0.51	0.88	-0.191	6.1E-01
Mtr.20079.1.S1_at	5.87	3.72	5.43	5.81	4.43	5.27	5.01	1.14	5.17	0.69	1.03	0.046	8.4E-01
Mtr.42034.1.S1_at	4.50	2.25	3.66	4.58	5.73	5.16	3.47	1.14	5.16	0.58	1.49	0.571	8.4E-02
Mtr.27180.1.S1_s_at	7.88	5.83	7.71	7.62	8.43	7.61	7.14	1.14	7.88	0.47	1.10	0.143	3.5E-01
Mtr.25969.1.S1_at	8.70	6.52	7.06	7.75	8.11	8.06	7.43	1.14	7.97	0.19	1.07	0.103	4.6E-01
Mtr.9602.1.S1_at	8.46	6.80	8.97	8.72	8.83	8.69	8.07	1.14	8.75	0.07	1.08	0.116	3.6E-01
Mtr.5615.1.S1_at	7.05	5.15	7.18	6.95	7.09	6.89	6.46	1.14	6.97	0.11	1.08	0.110	4.8E-01
Mtr.39257.1.S1_at	7.51	5.45	7.31	7.05	7.21	6.96	6.76	1.13	7.07	0.13	1.05	0.065	6.6E-01
Mtr.13189.1.S1_at	7.83	7.24	9.43	7.37	7.33	6.80	8.17	1.13	7.17	0.32	0.88	-0.188	2.2E-01
Mtr.39027.1.S1_s_at	6.14	4.16	6.11	5.70	6.41	6.19	5.47	1.13	6.10	0.36	1.12	0.158	4.1E-01
Mtr.34709.1.S1_at	8.19	6.37	8.45	8.27	8.26	7.90	7.67	1.13	8.14	0.21	1.06	0.086	5.2E-01
Mtr.3781.1.S1_s_at	8.47	6.38	8.18	8.04	8.38	8.24	7.68	1.13	8.22	0.17	1.07	0.099	4.6E-01
Mtr.31248.1.S1_s_at	9.70	7.82	9.86	9.84	10.04	9.87	9.13	1.13	9.92	0.11	1.09	0.119	3.0E-01

Mtr.7362.1.S1_at	5.04	3.03	4.94	5.31	4.51	4.93	4.34	1.13	4.92	0.40	1.13	0.181	4.5E-01
Mtr.21436.1.S1_at	2.85	4.77	2.77	2.81	2.76	2.66	3.47	1.13	2.74	0.08	0.79	-0.338	3.3E-01
Mtr.25828.1.S1_at	7.27	5.53	7.67	7.36	7.30	7.28	6.82	1.13	7.31	0.04	1.07	0.100	5.0E-01
Mtr.48949.1.S1_at	6.25	4.29	6.25	6.23	6.33	6.43	5.59	1.13	6.33	0.10	1.13	0.178	3.3E-01
Mtr.492.1.S1_at	7.78	5.53	6.45	6.75	6.74	6.83	6.59	1.13	6.77	0.05	1.03	0.040	7.9E-01
Mtr.14679.1.S1_x_at	5.52	3.44	5.26	5.57	5.63	5.59	4.74	1.13	5.60	0.03	1.18	0.238	2.6E-01
Mtr.4829.1.S1_at	5.87	4.19	6.35	5.53	6.06	6.12	5.47	1.13	5.90	0.33	1.08	0.110	5.6E-01
Mtr.45149.1.S1_at	6.10	4.53	6.73	6.54	6.37	6.64	5.79	1.13	6.52	0.14	1.13	0.172	3.3E-01
Mtr.29187.1.S1_at	2.39	4.35	2.40	2.38	2.37	2.36	3.05	1.13	2.37	0.01	0.78	-0.362	3.6E-01
Mtr.33022.1.S1_at	6.40	8.62	7.90	7.54	7.45	7.85	7.64	1.13	7.61	0.21	1.00	-0.004	9.8E-01
Mtr.34891.1.S1_s_at	6.80	4.62	6.23	6.82	6.72	7.08	5.88	1.13	6.87	0.18	1.17	0.224	2.1E-01
Mtr.31840.1.S1_at	10.32	8.15	9.79	8.28	7.36	8.51	9.42	1.13	8.05	0.61	0.85	-0.226	1.4E-01
Mtr.32855.1.S1_at	7.90	5.82	7.65	8.07	8.11	8.13	7.12	1.13	8.10	0.03	1.14	0.186	2.1E-01
Mtr.615.1.S1_at	4.53	2.75	4.86	5.22	5.68	4.70	4.05	1.13	5.20	0.49	1.29	0.362	1.8E-01
Mtr.4632.1.S1_at	5.84	4.17	6.32	6.22	6.81	6.06	5.44	1.13	6.37	0.40	1.17	0.226	2.5E-01
Mtr.33116.1.S1_at	7.79	5.55	6.96	7.56	7.13	7.59	6.76	1.13	7.43	0.25	1.10	0.135	3.8E-01
Mtr.39482.1.S1_at	6.88	4.86	6.76	6.08	6.30	6.64	6.17	1.13	6.34	0.28	1.03	0.040	8.1E-01
Mtr.19407.1.S1_at	5.88	7.31	5.07	6.15	5.52	5.85	6.09	1.13	5.84	0.32	0.96	-0.060	7.3E-01
Mtr.33691.1.S1_at	9.24	7.92	6.99	6.42	8.55	7.92	8.05	1.13	7.63	1.10	0.95	-0.077	6.7E-01
Mtr.51594.1.S1_at	2.83	4.78	2.82	2.74	2.73	2.73	3.48	1.13	2.73	0.01	0.79	-0.349	3.2E-01
Mtr.835.1.S1_at	2.58	4.52	2.55	2.53	2.51	2.52	3.22	1.13	2.52	0.01	0.78	-0.350	3.5E-01
Mtr.34711.1.S1_at	5.60	4.80	7.03	5.96	6.43	5.22	5.81	1.13	5.87	0.61	1.01	0.015	9.4E-01
Mtr.46646.1.S1_at	2.35	4.29	2.32	2.31	2.32	2.30	2.99	1.13	2.31	0.01	0.77	-0.370	3.6E-01
Mtr.46433.1.S1_at	8.84	7.35	9.57	8.96	9.03	8.96	8.58	1.13	8.99	0.04	1.05	0.066	5.7E-01
Mtr.44185.1.S1_at	8.78	6.66	8.39	8.62	8.58	8.63	7.94	1.13	8.61	0.03	1.08	0.116	3.6E-01
Mtr.36811.1.S1_at	7.16	5.81	8.06	7.25	7.45	6.97	7.01	1.13	7.22	0.24	1.03	0.044	7.6E-01
Mtr.8197.1.S1_at	3.58	5.40	3.33	3.57	3.78	3.58	4.10	1.13	3.65	0.12	0.89	-0.171	5.2E-01
Mtr.33154.1.S1_s_at	5.72	3.78	5.76	6.23	5.93	5.74	5.09	1.13	5.97	0.25	1.17	0.230	2.6E-01
Mtr.31452.1.S1_at	6.82	4.69	6.40	7.01	6.73	7.06	5.97	1.13	6.93	0.17	1.16	0.215	2.2E-01
Mtr.10854.1.S1_at	5.23	3.05	3.62	3.32	2.99	2.89	3.97	1.13	3.06	0.22	0.77	-0.372	2.5E-01
Mtr.14242.1.S1_s_at	5.36	3.12	3.98	5.30	5.51	5.34	4.15	1.13	5.38	0.11	1.30	0.374	1.3E-01
Mtr.11334.1.S1_at	7.15	4.94	6.47	7.00	6.96	7.14	6.19	1.13	7.03	0.09	1.14	0.185	2.6E-01
Mtr.16129.1.S1_at	11.69	9.74	11.69	12.15	11.79	11.87	11.04	1.13	11.93	0.19	1.08	0.112	2.5E-01
Mtr.3565.1.S1_at	4.74	2.87	4.90	4.89	5.70	5.09	4.17	1.13	5.23	0.42	1.25	0.327	2.0E-01
Mtr.25803.1.S1_at	5.92	4.40	6.60	6.09	6.09	6.08	5.64	1.13	6.09	0.00	1.08	0.111	5.3E-01
Mtr.27942.1.S1_at	4.19	2.61	4.79	4.18	4.18	4.17	3.86	1.13	4.17	0.01	1.08	0.112	6.6E-01
Mtr.1621.1.S1_s_at	4.07	2.50	4.69	3.98	3.98	3.93	3.76	1.13	3.96	0.03	1.06	0.078	7.7E-01
Mtr.13298.1.S1_at	7.91	6.02	8.04	8.16	7.94	8.05	7.32	1.13	8.05	0.11	1.10	0.136	3.3E-01
Mtr.3828.1.S1_at	2.67	4.59	2.61	2.60	2.60	2.59	3.29	1.13	2.60	0.00	0.79	-0.339	3.5E-01
Mtr.42895.1.S1_at	8.67	7.48	9.73	9.23	9.23	9.00	8.63	1.13	9.15	0.13	1.06	0.085	4.7E-01
Mtr.37301.1.S1_at	6.83	4.66	6.27	6.00	6.49	5.82	5.92	1.13	6.10	0.35	1.03	0.044	8.0E-01
Mtr.30754.1.S1_at	8.66	6.76	8.76	8.59	8.30	8.37	8.06	1.13	8.42	0.15	1.04	0.063	6.1E-01
Mtr.34387.1.S1_at	11.00	8.86	9.33	8.60	7.81	8.51	9.73	1.13	8.31	0.44	0.85	-0.228	1.1E-01
Mtr.45319.1.S1_at	4.17	2.49	4.63	5.25	4.51	5.32	3.76	1.13	5.03	0.45	1.34	0.417	1.5E-01
Mtr.34499.1.S1_at	5.16	3.84	6.08	6.01	6.31	5.80	5.03	1.13	6.04	0.26	1.20	0.265	2.0E-01
Mtr.9478.1.S1_at	8.01	6.29	8.41	8.20	6.93	8.19	7.57	1.13	7.77	0.73	1.03	0.038	8.1E-01
Mtr.2885.1.S1_at	5.15	3.84	6.08	5.53	5.58	5.42	5.02	1.13	5.51	0.08	1.10	0.133	5.0E-01
Mtr.7842.1.S1_at	8.20	7.18	9.43	8.74	8.83	8.24	8.27	1.13	8.60	0.32	1.04	0.057	6.5E-01
Mtr.34989.1.S1_at	3.12	5.05	5.09	5.59	5.75	5.99	4.42	1.13	5.78	0.20	1.31	0.386	1.1E-01
Mtr.4977.1.S1_at	7.87	5.91	7.85	7.70	7.55	7.74	7.21	1.13	7.67	0.10	1.06	0.088	5.2E-01
Mtr.49894.1.S1_s_at	7.34	5.43	7.41	7.10	7.35	7.13	6.73	1.13	7.19	0.13	1.07	0.097	5.2E-01
Mtr.29417.1.S1_at	5.79	3.73	5.53	5.83	5.74	5.56	5.02	1.12	5.71	0.14	1.14	0.187	3.5E-01
Mtr.10090.1.S1_at	7.57	6.04	8.24	7.95	8.07	7.31	7.28	1.12	7.78	0.41	1.07	0.095	5.1E-01
Mtr.19783.1.S1_at	4.48	2.68	4.75	6.23	5.87	6.26	3.97	1.12	6.12	0.22	1.54	0.624	3.1E-02
Mtr.44680.1.S1_at	4.29	2.96	5.19	4.96	5.01	3.33	4.15	1.12	4.43	0.95	1.07	0.097	7.5E-01
Mtr.30495.1.S1_at	7.75	5.53	6.95	6.39	6.23	6.36	6.75	1.12	6.33	0.09	0.94	-0.093	5.5E-01
Mtr.19966.1.S1_at	4.94	3.64	5.88	6.49	6.32	6.07	4.82	1.12	6.29	0.21	1.31	0.384	9.0E-02
Mtr.16539.1.S1_s_at	4.71	2.70	2.83	2.66	2.55	2.58	3.41	1.12	2.60	0.05	0.76	-0.394	2.8E-01
Mtr.20543.1.S1_at	2.57	4.51	2.56	2.60	2.54	2.54	3.21	1.12	2.56	0.03	0.80	-0.329	3.7E-01
Mtr.1272.1.S1_s_at	5.76	4.07	3.64	3.83	3.53	3.52	4.49	1.12	3.63	0.17	0.81	-0.308	2.6E-01
Mtr.7384.1.S1_at	8.91	6.96	8.89	8.55	8.82	9.00	8.25	1.12	8.79	0.23	1.06	0.091	4.6E-01
Mtr.9261.1.S1_s_at	10.61	8.43	9.97	10.55	10.21	10.54	9.67	1.12	10.43	0.20	1.08	0.109	3.1E-01
Mtr.41730.1.S1_at	4.29	2.73	4.90	4.31	4.54	5.37	3.97	1.12	4.74	0.56	1.19	0.255	3.5E-01
Mtr.11528.1.S1_at	8.09	6.01	7.78	7.52	7.90	7.74	7.30	1.12	7.72	0.19	1.06	0.081	5.5E-01
Mtr.5331.1.S1_at	2.10	4.04	2.09	2.08	2.08	2.08	2.74	1.12	2.08	0.00	0.76	-0.395	3.7E-01
Mtr.2090.1.S1_at	12.53	10.29	11.32	11.28	10.74	11.07	11.38	1.12	11.03	0.27	0.97	-0.045	6.3E-01
Mtr.21468.1.S1_x_at	7.90	9.87	7.96	8.11	7.89	7.94	8.58	1.12	7.98	0.11	0.93	-0.104	4.1E-01
Mtr.35825.1.S1_at	12.91	10.79	12.49	12.88	12.65	12.90	12.06	1.12	12.81	0.14	1.06	0.087	3.1E-01
Mtr.39551.1.S1_at	3.40	3.53	5.40	6.84	6.55	6.89	4.11	1.12	6.76	0.18	1.64	0.717	1.6E-02
Mtr.33366.1.S1_at	3.32	5.48	3.90	4.43	5.85	4.96	4.23	1.12	5.08	0.72	1.20	0.263	3.3E-01
Mtr.37623.1.S1_at	6.77	4.61	6.21	6.20	6.06	5.34	5.86	1.12	5.87	0.46	1.00	0.001	1.0E+00
Mtr.6020.1.S1_at	3.34	5.24	3.27	3.45	3.21	3.32	3.95	1.12	3.33	0.12	0.84	-0.249	3.9E-01
Mtr.31394.1.S1_at	7.92	6.22	8.33	8.31	8.34	8.46	7.49	1.12	8.37	0.08	1.12	0.160	2.5E-01
Mtr.45418.1.S1_at	8.16	6.41	8.49	7.96	8.02	7.53	7.69	1.12	7.84	0.27	1.02	0.028	8.3E-01
Mtr.27061.1.S1_at	6.25	4.33	6.29	6.23	6.76	6.19	5.62	1.12	6.39	0.32	1.14	0.185	3.2E-01
Mtr.34923.1.S1_at	6.35	4.21	5.83	6.40	6.29	6.50	5.46	1.12	6.40	0.11	1.17	0.228	2.2E-01
Mtr.36078.1.S1_at	8.05	6.16	8.13	8.54	8.86	8.57	7.45	1.12	8.66	0.18	1.16	0.218	1.4E-01
Mtr.38903.1.S1_at	7.57	5.63	7.57	7.84	7.80	7.41	6.92	1.12	7.68	0.24	1.11	0.151	3.1E-01
Mtr.48853.1.S1_at	3.06	4.80	2.71	2.88	2.73	2.87	3.52	1.12	2.83	0.09	0.80	-0.319	3.4E-01
Mtr.44550.1.S1_at	3.31	5.08	3.00	2.99	2.92	2.95	3.80	1.12	2.95	0.04	0.78	-0.363	2.6E-01
Mtr.6658.1.S1_at	9.47	7.57	9.54	9.06	8.75	9.08	8.86	1.12	8.97	0.18	1.01	0.017	8.8E-01
Mtr.5496.1.S1_at	8.49	7.19	9.42	9.03	8.90	8.58	8.37	1.12	8.83	0.23	1.06	0.078	5.2E-01
Mtr.33285.1.S1_at	4.68	2.79	4.77	4.63	3.94	5.09	4.08	1.12	4.55	0.58	1.12	0.158	5.5E-01
Mtr.36886.1.S1_at	9.82	8.15	10.27	9.92	10.08	9.80	9.41	1.12	9.93	0.14	1.06	0.078	4.7E-01
Mtr.38361.1.S1_s_at	7.47	5.49	7.37	7.39	7.33	7.42	6.78	1.12	7.38	0.04	1.09	0.123	4.0E-01
Mtr.49578.1.S1_at	5.87	4.66	6.89	7.54	7.93	7.81	5.81	1.12	7.76	0.20	1.34	0.419	4.0E-02
Mtr.13949.1.S1_at	4.23	2.18	3.97	4.77	3.96	3.81	3.46	1.12	4.18	0.51	1.21	0.274	3.7E-01
Mtr.5495.1.S1_at	5.56	4.06	6.23	5.14	5.63	5.19	5.28	1.12	5.32	0.27	1.01	0.008	9.7E-01
Mtr.30296.1.S1_at	4.97	3.04	4.97	5.18	5.28	4.91	4.33	1.12	5.12	0.19	1.18	0.243	2.9E-01
Mtr.32908.1.S1_at	8.04	6.03	7.88	7.91	7.98	7.76	7.32	1.12	7.89	0.12	1.08	0.107	4.3E-01
Mtr.44655.1.S1_at	8.80	6.88	8.82	8.88	8.94	8.95	8.17	1.12	8.92	0.04	1.09	0.128	3.1E-01
Mtr.3244.1.S1_s_at	3.37	5.29	3.36	3.34	3.26	3.33	4.01	1.12	3.31	0.04	0.83	-0.276	3.4E-01
Mtr.29936.1.S1_at	2.22	4.14	2.19	2.23	2.22	2.21	2.85	1.12	2.22	0.01	0.78	-0.361	3.8E-01
Mtr.12186.1.S1_at	4.81	2.97	4.98	4.74	4.44	4.35	4.25	1.12	4.51	0.21	1.06	0.085	7.1E-01
Mtr.42825.1.S1_at	6.29	4.51	6.57	6.42	6.30	5.75	5.79	1.12	6.16	0.36	1.06	0.089	6.2E-01
Mtr.27070.1.S1_at	6.51	4.59	6.53	6.59	6.73	6.24	5.88	1.12	6.52	0.25	1.11	0.150	3.9E-01
Mtr.50729.1.S1_at	10.34	8.62	10.71	11.66	12.30	12.06	9.89	1.11	12.01	0.33	1.21	0.280	3.4E-02
Mtr.44654.1.S1_at	4.68	4.33	6.41	4.74	4.63	3.02	5.14	1.11	4.13	0.96	0.80	-0.315	3.0E-01
Mtr.10385.1.S1_at	5.80	7.71	5.78	6.02	5.77	5.75	6.43	1.11	5.85	0.15	0.91	-0.137	4.2E-01
Mtr.12808.1.S1_at	6.13	4.05	5.76	6.61	7.44	7.12	5.31	1.11	7.06	0.42	1.33	0.409	6.4E-02
Mtr.44302.1.S1_at	7.27	5.05	6.27	7.01	6.24	6.46	6.20	1.11	6.57	0.40	1.06	0.085	6.1E-01
Mtr.5875.1.S1_at	7.76	5.66	7.36	7.46	7.22	7.36	6.93	1.11	7.34	0.12	1.06	0.085	5.5E-01
Mtr.38554.1.S1_at	7.87	5.65	6.68	6.59	6.41	6.50	6.73	1.11	6.50	0.09	0.97	-0.051	7.4E-01
Mtr.27191.1.S1_at	6.95	5.01	6.92	7.23	7.17	7.18	6.30	1.11	7.19	0.03	1.14	0.192	2.4E-01
Mtr.20873.1.S1_at	2.79	4.68	2.71	2.71	2.71	3.36	3.39	1.11	2.93	0.38	0.86	-0.214	5.3E-01
Mtr.10073.1.S1_at	7.18	5.15	6.95	6.94	7.12	7.03	6.43	1.11	7.03	0.09	1.09	0.129	4.0E-01
Mtr.39786.1.S1_at	8.68	6.46	7.49	7.49	7.49	7.73	7.55	1.11	7.57	0.14	1.00	0.005	9.7E-01
Mtr.42691.1.S1_at	6.28	4.65	6.78	6.33	6.25	6.24	5.90	1.11	6.27	0.05	1.06	0.088	6.0E-01
Mtr.51862.1.S1_at	8.18	6.24	8.15	7.68	8.14	7.35	7.52	1.11	7.73	0.40	1.03	0.038	7.8E-01
Mtr.26609.1.S1_at	2.50	4.41	2.48	2.47	2.47	2.47	3.13	1.11	2.47	0.00	0.79	-0.340	3.6E-01
Mtr.12725.1.S1_at	10.84	9.00	8.84	9.58	9.71	9.78	9.56	1.11	9.69	0.10	1.01	0.020	8.5E-01
Mtr.31331.1.S1_s_at	8.76	6.86	8.81	8.55	8.27	8.52	8.14	1.11	8.45	0.16	1.04	0.053	6.6E-01
Mtr.44652.1.S1_at	6.97	5.16	7.17	7.33	7.39	7.12	6.43	1.11	7.28	0.14	1.13	0.178	2.6E-01
Mtr.45366.1.S1_at	7.52	5.46	7.19	7.16	7.08	6.90	6.72	1.11	7.05	0.13	1.05	0.068	6.4E-01
Mtr.32744.1.S1_at	5.18	3.97	6.18	6.03	7.61	5.97	5.11	1.11	6.54	0.93	1.28	0.356	1.6E-01
Mtr.26629.1.S1_at	8.22	7.20	9.41	11.24	11.83	11.50	8.28	1.11	11.52	0.29	1.39	0.477	8.1E-03
Mtr.40048.1.S1_at	10.59	8.82	8.54	9.27	9.03	9.55	9.32	1.11	9.28	0.26	1.00	-0.005	9.7E-01
Mtr.10185.1.S1_at	7.10	4.92	5.64	7.36	7.53	7.42	5.89	1.11	7.44	0.08	1.26	0.337	7.3E-02
Mtr.7804.1.S1_at	2.52	4.43	2.50	2.49	2.50	2.50	3.15	1.11	2.49	0.00	0.79	-0.335	3.7E-01
Mtr.33365.1.S1_at	5.63	3.61	5.41	4.06	5.16	5.13	4.88	1.11	4.78	0.63	0.98	-0.030	9.0E-01
Mtr.15839.1.S1_s_at	6.46	6.78	8.52	9.83	10.37	9.98	7.25	1.11	10.06	0.28	1.39	0.472	1.3E-02
Mtr.12678.1.S1_at	10.98	8.78	9.69	10.70	10.74	10.89	9.82	1.11	10.77	0.10	1.10	0.134	2.1E-01
Mtr.26044.1.S1_at	6.12	5.77	4.05	6.19	6.07	6.52	5.31	1.11	6.26	0.24	1.18	0.237	2.2E-01
Mtr.27598.1.S1_at	6.47	6.21	8.25	7.16	7.56	6.82	6.98	1.11	7.18	0.37	1.03	0.041	7.8E-01
Mtr.22371.1.S1_at	2.38	4.28	2.35	2.35	2.31	2.36	3.01	1.11	2.34	0.02	0.78	-0.361	3.6E-01
Mtr.5614.1.S1_at	4.59	2.82	4.87	4.22	4.53	4.58	4.09	1.11	4.44	0.19	1.09	0.118	6.2E-01
Mtr.23078.1.S1_at	3.08	4.97	3.03	3.03	3.00	3.04	3.70	1.11	3.03	0.02	0.82	-0.289	3.5E-01
Mtr.11651.1.S1_at	6.77	4.81	6.67	7.03	6.60	6.81	6.08	1.11	6.81	0.21	1.12	0.164	3.2E-01
Mtr.40792.1.S1_at	8.53	6.34	7.67	7.65	7.85	7.72	7.51	1.11	7.74	0.10	1.03	0.043	7.4E-01
Mtr.20673.1.S1_at	7.77	5.56	6.73	6.65	7.30	6.67	6.69	1.11	6.87	0.37	1.03	0.040	8.0E-01
Mtr.40246.1.S1_at	4.08	3.41	5.57	6.00	6.14	5.57	4.35	1.11	5.90	0.30	1.36	0.439	7.9E-02

Mtr.50549.1.S1_at	6.15	4.58	6.71	6.58	6.51	6.67	5.81	1.11	6.58	0.08	1.13	0.179	3.0E-01
Mtr.26927.1.S1_at	6.98	4.77	5.84	6.86	6.83	6.96	5.86	1.11	6.88	0.07	1.17	0.231	1.9E-01
Mtr.6718.1.S1_at	5.10	2.89	4.06	3.15	3.29	2.73	4.02	1.11	3.06	0.29	0.76	-0.393	2.2E-01
Mtr.24258.1.S1_s_at	6.89	4.74	6.29	5.94	6.19	6.54	5.97	1.11	6.22	0.30	1.04	0.059	7.2E-01
Mtr.5098.1.S1_at	8.20	6.16	6.44	7.36	7.11	7.33	6.93	1.10	7.27	0.13	1.05	0.068	6.3E-01
Mtr.38931.1.S1_at	5.07	2.91	3.58	5.35	6.12	5.71	3.85	1.10	5.72	0.39	1.49	0.571	5.1E-02
Mtr.28550.1.S1_at	5.20	4.93	6.96	7.88	8.77	8.00	5.70	1.10	8.22	0.48	1.44	0.528	2.2E-02
Mtr.23674.1.S1_at	4.82	4.54	6.58	6.23	5.52	6.22	5.31	1.10	5.99	0.40	1.13	0.172	3.8E-01
Mtr.40137.1.S1_s_at	11.54	9.35	10.70	10.78	10.89	10.72	10.53	1.10	10.80	0.08	1.02	0.036	7.0E-01
Mtr.19691.1.S1_at	5.02	3.26	5.29	4.73	4.66	4.76	4.52	1.10	4.72	0.05	1.04	0.061	7.8E-01
Mtr.38297.1.S1_at	7.14	5.43	7.50	7.45	7.63	7.60	6.69	1.10	7.56	0.10	1.13	0.176	2.5E-01
Mtr.35894.1.S1_at	11.30	9.10	10.00	10.05	9.40	9.78	10.13	1.10	9.75	0.33	0.96	-0.056	5.9E-01
Mtr.35179.1.S1_at	7.21	5.75	7.91	7.46	7.47	7.10	6.96	1.10	7.34	0.21	1.06	0.078	5.8E-01
Mtr.50861.1.S1_x_at	3.54	5.21	3.13	3.12	2.90	3.00	3.96	1.10	3.01	0.11	0.76	-0.395	2.1E-01
Mtr.32851.1.S1_at	7.31	5.54	7.57	8.00	7.97	8.01	6.81	1.10	7.99	0.02	1.17	0.231	1.4E-01
Mtr.44234.1.S1_at	8.18	6.42	8.45	8.36	8.17	8.21	7.69	1.10	8.25	0.10	1.07	0.102	4.3E-01
Mtr.50162.1.S1_at	5.86	3.86	5.66	5.82	6.16	5.81	5.12	1.10	5.93	0.20	1.16	0.210	2.8E-01
Mtr.26154.1.S1_at	2.67	4.56	2.64	2.62	2.59	2.64	3.29	1.10	2.62	0.02	0.80	-0.330	3.5E-01
Mtr.42510.1.S1_at	8.50	6.50	6.69	7.71	7.61	7.87	7.23	1.10	7.73	0.13	1.07	0.097	4.8E-01
Mtr.13997.1.S1_at	3.00	4.89	2.96	2.95	2.95	2.92	3.62	1.10	2.94	0.02	0.81	-0.299	3.5E-01
Mtr.15822.1.S1_x_at	4.15	5.88	3.83	4.14	4.12	4.20	4.62	1.10	4.15	0.04	0.90	-0.153	5.1E-01
Mtr.38938.1.S1_at	9.79	7.59	8.75	8.85	8.64	8.99	8.71	1.10	8.82	0.18	1.01	0.019	8.7E-01
Mtr.16167.1.S1_at	7.95	6.24	8.30	8.24	8.33	8.22	7.50	1.10	8.26	0.06	1.10	0.140	2.9E-01
Mtr.5277.1.S1_at	4.50	6.63	5.05	5.11	5.01	5.72	5.39	1.10	5.28	0.38	0.98	-0.030	8.8E-01
Mtr.11707.1.S1_at	7.88	5.89	7.71	7.86	8.11	7.77	7.16	1.10	7.92	0.18	1.11	0.145	3.0E-01
Mtr.43419.1.S1_at	9.46	7.59	7.52	7.94	8.06	8.37	8.19	1.10	8.12	0.22	0.99	-0.012	9.2E-01
Mtr.35887.1.S1_at	5.29	3.34	5.19	5.58	4.87	4.92	4.61	1.10	5.12	0.40	1.11	0.152	4.9E-01
Mtr.43078.1.S1_s_at	8.89	6.94	8.78	8.44	8.13	8.71	8.20	1.10	8.42	0.29	1.03	0.038	7.5E-01
Mtr.11987.1.S1_at	7.61	5.48	7.02	7.18	7.25	7.15	6.70	1.10	7.19	0.05	1.07	0.102	4.8E-01
Mtr.1834.1.S1_at	7.35	5.43	7.31	6.95	7.67	7.43	6.70	1.10	7.35	0.36	1.10	0.135	3.8E-01
Mtr.17915.1.S1_s_at	7.59	5.54	7.25	7.09	7.24	7.00	6.79	1.10	7.11	0.13	1.05	0.066	6.4E-01
Mtr.51369.1.S1_at	2.59	4.05	4.74	3.57	4.17	3.47	3.79	1.10	3.74	0.38	0.99	-0.020	9.4E-01
Mtr.30702.1.S1_at	7.90	6.29	8.40	8.03	8.12	8.10	7.53	1.10	8.08	0.04	1.07	0.102	4.3E-01
Mtr.49160.1.S1_at	6.61	4.44	5.82	5.36	5.99	5.70	5.62	1.10	5.68	0.32	1.01	0.014	9.4E-01
Mtr.32461.1.S1_at	4.85	3.38	5.53	5.40	4.75	4.88	4.58	1.10	5.01	0.35	1.09	0.128	5.6E-01
Mtr.10895.1.S1_at	7.57	5.60	7.43	7.19	7.41	7.04	6.87	1.10	7.21	0.19	1.05	0.070	6.2E-01
Mtr.10627.1.S1_at	10.78	8.81	10.64	10.49	10.50	10.39	10.08	1.10	10.46	0.06	1.04	0.053	5.8E-01
Mtr.35515.1.S1_at	7.16	5.76	7.92	7.81	7.60	7.87	6.95	1.10	7.76	0.14	1.12	0.159	2.7E-01
Mtr.31076.1.S1_at	6.55	4.51	6.25	6.46	6.11	5.71	5.77	1.10	6.10	0.37	1.06	0.079	6.5E-01
Mtr.4406.1.S1_at	4.41	2.51	4.42	3.96	3.95	3.96	3.78	1.10	3.96	0.01	1.05	0.067	7.9E-01
Mtr.24396.1.S1_at	5.76	5.91	7.73	7.71	6.71	7.78	6.47	1.10	7.40	0.60	1.14	0.194	2.7E-01
Mtr.15484.1.S1_at	10.65	8.89	10.90	11.12	10.39	10.88	10.15	1.10	10.80	0.37	1.06	0.090	3.9E-01
Mtr.32733.1.S1_at	7.48	5.34	6.82	7.22	7.49	7.73	6.55	1.10	7.48	0.26	1.14	0.193	2.2E-01
Mtr.925.1.S1_x_at	2.39	4.28	2.35	2.34	2.33	2.51	3.01	1.10	2.39	0.10	0.80	-0.329	3.9E-01
Mtr.12700.1.S1_at	8.93	6.99	8.85	8.65	8.69	8.67	8.26	1.10	8.67	0.02	1.05	0.071	5.5E-01
Mtr.7796.1.S1_at	5.90	4.47	6.63	6.51	6.52	5.88	5.67	1.10	6.30	0.37	1.11	0.153	4.0E-01
Mtr.33314.1.S1_at	5.57	3.44	4.05	5.24	6.25	6.36	4.36	1.10	5.95	0.62	1.37	0.451	9.3E-02
Mtr.15947.1.S1_at	3.18	5.08	3.18	3.16	3.13	3.14	3.81	1.10	3.14	0.02	0.82	-0.279	3.5E-01
Mtr.52297.1.S1_at	3.85	5.40	3.28	3.86	3.38	3.42	4.18	1.10	3.55	0.27	0.85	-0.235	3.9E-01
Mtr.8780.1.S1_at	8.09	6.12	7.93	7.69	8.03	7.94	7.38	1.10	7.89	0.18	1.07	0.096	4.7E-01
Mtr.7295.1.S1_at	6.53	4.88	6.95	6.48	7.38	6.50	6.12	1.10	6.79	0.51	1.11	0.150	3.9E-01
Mtr.29280.1.S1_at	7.75	5.91	7.86	7.61	8.02	7.72	7.17	1.10	7.78	0.22	1.08	0.117	4.0E-01
Mtr.30902.1.S1_at	7.18	4.99	6.11	6.11	5.18	5.78	6.09	1.10	5.69	0.47	0.93	-0.099	5.9E-01
Mtr.39990.1.S1_at	4.61	2.77	2.66	2.61	2.63	2.91	3.35	1.10	2.72	0.17	0.81	-0.298	3.8E-01
Mtr.2154.1.S1_at	7.62	5.81	7.78	7.35	7.74	7.33	7.07	1.10	7.47	0.23	1.06	0.081	5.6E-01
Mtr.43034.1.S1_at	6.92	5.08	4.98	5.97	6.00	5.90	5.66	1.09	5.96	0.05	1.05	0.074	6.6E-01
Mtr.8987.1.S1_at	6.75	5.19	7.30	7.91	7.68	7.81	6.41	1.09	7.80	0.11	1.22	0.282	9.5E-02
Mtr.8763.1.S1_at	14.75	13.15	12.65	13.05	13.31	13.35	13.52	1.09	13.23	0.16	0.98	-0.030	6.8E-01
Mtr.25210.1.S1_at	6.32	4.37	6.21	6.06	6.13	5.58	5.64	1.09	5.93	0.30	1.05	0.072	6.8E-01
Mtr.45338.1.S1_at	8.45	6.58	8.49	8.44	8.25	8.51	7.84	1.09	8.40	0.14	1.07	0.099	4.3E-01
Mtr.28815.1.S1_at	8.63	7.39	9.58	8.97	9.11	9.13	8.54	1.09	9.07	0.09	1.06	0.088	4.5E-01
Mtr.41903.1.S1_at	5.17	4.11	6.30	6.32	6.12	5.71	5.19	1.09	6.05	0.31	1.16	0.220	2.6E-01
Mtr.45887.1.S1_at	6.16	4.93	7.11	6.85	7.27	6.54	6.07	1.09	6.89	0.37	1.14	0.183	2.9E-01
Mtr.41456.1.S1_at	6.08	7.61	8.20	9.45	9.73	9.59	7.30	1.09	9.59	0.14	1.31	0.394	2.3E-02
Mtr.9964.1.S1_at	5.64	3.88	5.88	5.68	5.57	5.46	5.14	1.09	5.57	0.11	1.08	0.116	5.3E-01
Mtr.48642.1.S1_at	2.39	4.27	2.37	2.37	2.37	2.36	3.01	1.09	2.37	0.00	0.79	-0.348	3.6E-01
Mtr.50768.1.S1_at	10.69	8.78	10.65	10.70	10.63	10.51	10.04	1.09	10.61	0.10	1.06	0.081	4.1E-01
Mtr.209.1.S1_at	7.15	5.40	7.41	6.76	6.53	6.54	6.66	1.09	6.61	0.13	0.99	-0.010	9.5E-01
Mtr.41715.1.S1_at	12.25	10.10	11.50	11.58	11.34	11.73	11.28	1.09	11.55	0.20	1.02	0.034	7.0E-01
Mtr.47706.1.S1_at	13.51	11.34	12.19	13.15	12.68	13.14	12.35	1.09	12.99	0.27	1.05	0.073	3.8E-01
Mtr.31477.1.S1_at	8.31	6.16	7.57	7.70	7.62	7.29	7.35	1.09	7.53	0.21	1.03	0.037	7.8E-01
Mtr.11879.1.S1_at	7.05	4.88	6.16	6.01	6.22	5.97	6.03	1.09	6.06	0.13	1.01	0.008	9.6E-01
Mtr.5274.1.S1_s_at	8.34	6.46	8.37	8.49	8.52	8.49	7.72	1.09	8.50	0.02	1.10	0.138	2.8E-01
Mtr.7053.1.S1_at	7.98	6.18	8.15	7.27	8.04	7.50	7.43	1.09	7.60	0.40	1.02	0.032	8.1E-01
Mtr.14656.1.S1_at	5.83	6.70	8.00	6.90	8.45	8.02	6.84	1.09	7.79	0.80	1.14	0.187	2.9E-01
Mtr.16033.1.S1_x_at	6.26	4.38	6.27	6.15	6.25	6.27	5.63	1.09	6.22	0.06	1.10	0.143	4.0E-01
Mtr.34408.1.S1_s_at	10.92	8.94	10.73	11.45	11.19	11.24	10.20	1.09	11.30	0.14	1.11	0.147	1.6E-01
Mtr.6056.1.S1_at	8.39	6.83	6.30	7.21	7.31	6.77	7.17	1.09	7.10	0.29	0.99	-0.015	9.2E-01
Mtr.27549.1.S1_at	5.27	3.09	4.05	4.78	4.75	4.99	4.14	1.09	4.84	0.13	1.17	0.227	3.3E-01
Mtr.44289.1.S1_at	5.25	3.41	5.34	5.10	5.09	5.03	4.66	1.09	5.07	0.04	1.09	0.121	5.5E-01
Mtr.5195.1.S1_at	6.49	4.51	6.29	6.29	6.33	6.31	5.76	1.09	6.31	0.02	1.09	0.130	4.4E-01
Mtr.6909.1.S1_at	4.48	2.35	3.81	4.00	4.47	4.00	3.55	1.09	4.15	0.27	1.17	0.228	4.0E-01
Mtr.28948.1.S1_at	9.24	7.07	7.96	8.18	8.31	8.26	8.09	1.09	8.25	0.07	1.02	0.029	8.1E-01
Mtr.17204.1.S1_at	2.56	4.53	2.75	2.42	2.55	2.53	3.28	1.09	2.50	0.07	0.76	-0.392	2.8E-01
Mtr.31654.1.S1_s_at	5.04	3.16	5.05	4.63	6.09	5.98	4.42	1.09	5.57	0.81	1.26	0.333	2.2E-01
Mtr.2745.1.S1_at	2.37	4.24	2.34	2.34	2.35	2.35	2.98	1.09	2.35	0.01	0.79	-0.346	3.7E-01
Mtr.6004.1.S1_at	5.33	3.36	5.14	5.24	5.54	5.64	4.61	1.09	5.47	0.21	1.19	0.248	2.5E-01
Mtr.49564.1.S1_at	5.16	3.35	3.21	3.20	3.20	3.20	3.91	1.09	3.20	0.00	0.82	-0.288	3.2E-01
Mtr.49002.1.S1_at	5.35	3.47	5.37	5.39	5.59	5.23	4.73	1.09	5.40	0.18	1.14	0.192	3.5E-01
Mtr.18374.1.S1_s_at	3.18	3.18	5.06	4.11	4.24	4.35	3.81	1.09	4.24	0.12	1.11	0.154	5.3E-01
Mtr.3202.1.S1_at	3.37	2.79	4.90	5.12	4.64	4.19	3.69	1.09	4.65	0.46	1.26	0.336	2.3E-01
Mtr.16967.1.S1_x_at	2.70	4.58	2.69	2.61	2.68	3.06	3.32	1.09	2.78	0.24	0.84	-0.255	4.5E-01
Mtr.17199.1.S1_at	4.92	3.01	4.87	5.39	4.85	5.60	4.27	1.09	5.28	0.39	1.24	0.306	2.0E-01
Mtr.5314.1.S1_at	5.62	3.80	5.74	5.63	5.94	5.77	5.06	1.09	5.78	0.16	1.14	0.192	3.2E-01
Mtr.11845.1.S1_at	7.45	5.70	7.70	7.37	7.37	7.42	6.95	1.09	7.39	0.03	1.06	0.089	5.2E-01
Mtr.9763.1.S1_at	8.08	5.98	6.54	6.30	7.01	6.89	6.87	1.09	6.73	0.38	0.98	-0.029	8.5E-01
Mtr.7480.1.S1_s_at	11.43	9.44	11.18	11.82	11.75	11.78	10.68	1.09	11.78	0.04	1.10	0.141	1.5E-01
Mtr.2701.1.S1_at	8.48	6.52	8.30	8.20	8.42	8.37	7.77	1.09	8.33	0.11	1.07	0.101	4.2E-01
Mtr.4637.1.S1_at	9.56	7.57	9.32	9.80	9.67	9.59	8.81	1.09	9.68	0.11	1.10	0.136	2.4E-01
Mtr.33352.1.S1_at	8.26	6.23	7.92	8.00	8.38	8.10	7.47	1.09	8.16	0.19	1.09	0.128	3.4E-01
Mtr.564.1.S1_at	9.14	7.23	9.10	8.88	8.77	8.72	8.49	1.09	8.79	0.08	1.04	0.051	6.6E-01
Mtr.38860.1.S1_at	6.88	4.84	6.51	6.87	6.84	6.88	6.08	1.09	6.86	0.02	1.13	0.176	2.8E-01
Mtr.4304.1.S1_s_at	6.96	4.97	6.72	6.34	6.53	6.55	6.21	1.09	6.47	0.12	1.04	0.058	7.0E-01
Mtr.29219.1.S1_at	7.72	5.70	7.39	7.69	7.19	7.44	6.94	1.09	7.44	0.25	1.07	0.100	4.8E-01
Mtr.26603.1.S1_at	2.64	4.48	2.56	2.54	2.54	2.53	3.22	1.09	2.54	0.01	0.79	-0.345	3.4E-01
Mtr.37724.1.S1_at	9.38	11.18	9.22	8.36	9.53	9.25	9.93	1.09	9.05	0.61	0.91	-0.134	2.9E-01
Mtr.34581.1.S1_at	2.22	4.10	2.22	2.22	2.22	2.22	2.85	1.09	2.22	0.00	0.78	-0.362	3.7E-01
Mtr.51909.1.S1_at	10.32	8.20	9.63	9.27	8.96	9.24	9.38	1.09	9.15	0.17	0.98	-0.036	7.4E-01
Mtr.47870.1.S1_at	3.86	5.69	3.76	3.92	3.02	3.85	4.44	1.09	3.59	0.50	0.81	-0.304	2.9E-01
Mtr.27985.1.S1_at	3.24	3.21	5.11	3.26	3.19	3.24	3.85	1.08	3.23	0.04	0.84	-0.256	3.7E-01
Mtr.37195.1.S1_at	8.01	6.27	8.27	8.18	8.20	7.88	7.52	1.08	8.09	0.18	1.08	0.105	4.2E-01
Mtr.18916.1.S1_at	7.30	5.18	5.84	6.81	6.59	6.91	6.11	1.08	6.77	0.17	1.11	0.148	3.6E-01
Mtr.5456.1.S1_at	6.40	4.53	6.41	6.55	6.51	6.19	5.78	1.08	6.41	0.20	1.11	0.150	3.8E-01
Mtr.39931.1.S1_at	6.60	4.72	6.59	6.36	6.23	6.08	5.97	1.08	6.22	0.14	1.04	0.059	7.1E-01
Mtr.1870.1.S1_at	9.13	7.19	8.99	9.05	8.82	8.75	8.43	1.08	8.88	0.16	1.05	0.074	5.2E-01
Mtr.40558.1.S1_at	4.45	2.64	2.52	2.51	2.48	2.51	3.20	1.08	2.50	0.02	0.78	-0.356	3.3E-01
Mtr.42822.1.S1_at	7.32	9.25	7.44	7.50	7.97	7.50	8.01	1.08	7.66	0.27	0.96	-0.064	6.2E-01
Mtr.23525.1.S1_at	2.43	4.30	2.42	2.42	2.42	2.41	3.05	1.08	2.42	0.00	0.79	-0.338	3.7E-01
Mtr.32302.1.S1_at	5.59	3.56	5.22	3.75	4.12	4.28	4.79	1.08	4.05	0.27	0.85	-0.242	3.2E-01
Mtr.30270.1.S1_at	8.79	6.77	8.48	8.48	8.54	8.48	8.01	1.08	8.50	0.04	1.06	0.085	4.8E-01
Mtr.3159.1.S1_at	7.21	5.35	7.25	7.07	7.21	7.27	6.60	1.08	7.18	0.10	1.09	0.122	4.1E-01
Mtr.14771.1.S1_at	8.08	6.38	8.40	7.90	8.41	7.68	7.62	1.08	8.00	0.37	1.05	0.070	6.0E-01
Mtr.28510.1.S1_at	6.15	4.33	4.21	4.72	5.04	5.14	4.90	1.08	4.97	0.22	1.01	0.020	9.2E-01
Mtr.44130.1.S1_s_at	7.95	5.82	7.24	7.65	7.55	7.99	7.00	1.08	7.73	0.23	1.10	0.142	3.2E-01
Mtr.4083.1.S1_s_at	8.26	6.56	8.57	8.61	8.85	8.42	7.79	1.08	8.63	0.22	1.11	0.146	2.6E-01
Mtr.107.1.S1_at	3.05	4.88	2.96	2.96	2.88	2.96	3.63	1.08	2.93	0.05	0.81	-0.307	3.3E-01
Mtr.36965.1.S1_at	7.69	5.89	5.75	4.87	5.75	5.60	6.44	1.08	5.41	0.47	0.84	-0.253	2.0E-01
Mtr.9440.1.S1_at	6.94	4.80	6.18	6.89	7.16	7.29	5.97	1.08	7.12	0.20	1.19	0.252	1.5E-01
Mtr.29409.1.S1_at	5.19	3.04	4.26	4.33	5.36	5.94	4.16	1.08	5.21	0.82	1.25	0.325	2.5E-01
Mtr.9414.1.S1_at	6.68	4.55	5.94	6.09	6.23	6.66	5.73	1.08	6.33	0.30	1.11	0.144	4.1E-01
Mtr.3786.1.S1_at	2.01	3.86	1.96	1.96	1.96	1.96	2.61	1.08	1.96	0.00	0.75	-0.416	3.5E-01

Mtr.10145.1.S1_at	6.84	5.09	7.08	7.39	6.67	6.95	6.34	1.08	7.00	0.36	1.10	0.144	3.7E-01
Mtr.44744.1.S1_at	7.89	6.43	8.55	8.48	8.52	8.28	7.62	1.08	8.43	0.13	1.11	0.145	2.7E-01
Mtr.26509.1.S1_at	7.39	5.66	7.65	7.07	6.82	7.06	6.90	1.08	6.99	0.14	1.01	0.018	9.0E-01
Mtr.27953.1.S1_at	6.92	5.65	7.81	7.11	7.44	6.72	6.79	1.08	7.09	0.36	1.04	0.062	6.7E-01
Mtr.13609.1.S1_at	4.80	3.77	5.93	5.03	5.28	5.27	4.84	1.08	5.19	0.14	1.07	0.103	6.0E-01
Mtr.38752.1.S1_at	6.74	4.58	5.87	5.84	5.62	5.68	5.73	1.08	5.71	0.12	1.00	-0.004	9.8E-01
Mtr.41495.1.S1_at	11.35	9.20	10.49	11.37	11.23	11.59	10.35	1.08	11.39	0.18	1.10	0.139	1.7E-01
Mtr.768.1.S1_at	9.08	8.01	6.92	7.74	7.96	8.32	8.00	1.08	8.01	0.29	1.00	0.001	1.0E+00
Mtr.20232.1.S1_at	2.79	3.79	4.95	7.71	8.36	7.84	3.84	1.08	7.97	0.35	2.07	1.052	3.3E-03
Mtr.13395.1.S1_at	5.70	3.54	4.64	5.11	5.17	4.99	4.63	1.08	5.09	0.09	1.10	0.137	5.0E-01
Mtr.13553.1.S1_at	8.41	6.42	8.16	7.76	8.10	7.78	7.66	1.08	7.88	0.19	1.03	0.040	7.5E-01
Mtr.15596.1.S1_at	7.59	5.68	7.51	7.06	7.32	7.29	6.92	1.08	7.22	0.14	1.04	0.061	6.6E-01
Mtr.45515.1.S1_at	3.37	5.24	3.36	3.40	4.01	3.24	3.99	1.08	3.55	0.41	0.89	-0.168	5.5E-01
Mtr.5400.1.S1_at	2.76	4.62	2.73	2.71	2.73	2.72	3.37	1.08	2.72	0.01	0.81	-0.309	3.6E-01
Mtr.42955.1.S1_at	11.89	9.78	10.44	10.83	10.89	11.05	10.70	1.08	10.93	0.11	1.02	0.030	7.4E-01
Mtr.34078.1.S1_at	2.51	4.38	2.51	2.50	2.49	2.48	3.13	1.08	2.49	0.01	0.79	-0.332	3.6E-01
Mtr.7558.1.S1_at	6.33	4.49	6.39	6.10	6.82	5.76	5.73	1.08	6.23	0.54	1.09	0.119	5.2E-01
Mtr.30403.1.S1_at	6.97	5.03	6.81	6.55	6.87	6.22	6.27	1.08	6.55	0.33	1.04	0.062	6.9E-01
Mtr.49088.1.S1_at	6.74	7.34	8.84	7.89	7.80	7.54	7.64	1.08	7.74	0.18	1.01	0.019	8.8E-01
Mtr.15014.1.S1_at	6.83	4.68	5.95	5.89	5.87	6.39	5.82	1.08	6.05	0.30	1.04	0.057	7.3E-01
Mtr.6009.1.S1_at	9.04	8.57	10.63	10.66	10.93	10.32	9.41	1.08	10.64	0.31	1.13	0.176	1.3E-01
Mtr.44983.1.S1_at	7.01	5.25	7.21	7.20	7.40	7.08	6.49	1.08	7.23	0.16	1.11	0.156	3.1E-01
Mtr.35490.1.S1_at	7.96	6.27	8.29	7.78	8.02	7.65	7.50	1.08	7.82	0.19	1.04	0.059	6.5E-01
Mtr.41968.1.S1_at	4.32	2.34	2.58	2.45	3.34	2.56	3.08	1.08	2.78	0.49	0.90	-0.144	6.9E-01
Mtr.10792.1.S1_at	9.98	8.10	8.13	8.88	9.71	9.48	8.74	1.08	9.36	0.43	1.07	0.099	4.1E-01
Mtr.24044.1.S1_s_at	8.98	7.02	8.78	9.16	9.26	9.39	8.26	1.08	9.27	0.12	1.12	0.166	1.8E-01
Mtr.28421.1.S1_x_at	7.10	5.27	5.18	5.25	5.34	5.50	5.85	1.08	5.36	0.13	0.92	-0.125	4.8E-01
Mtr.46342.1.S1_at	3.63	5.27	3.24	3.94	3.62	3.17	4.05	1.08	3.57	0.39	0.88	-0.180	5.1E-01
Mtr.12073.1.S1_at	9.53	8.73	10.87	10.37	9.90	10.22	9.71	1.08	10.16	0.24	1.05	0.066	5.1E-01
Mtr.24520.1.S1_at	6.63	8.71	7.17	5.38	6.20	6.32	7.50	1.08	5.97	0.52	0.80	-0.330	9.0E-02
Mtr.32597.1.S1_s_at	8.46	6.51	8.29	8.28	8.31	8.25	7.75	1.08	8.28	0.03	1.07	0.094	4.5E-01
Mtr.32865.1.S1_at	6.18	4.54	6.57	4.17	4.42	3.81	5.76	1.08	4.13	0.31	0.72	-0.480	6.5E-02
Mtr.24829.1.S1_at	3.06	2.00	4.16	5.06	5.51	4.20	3.07	1.08	4.92	0.66	1.60	0.680	6.5E-02
Mtr.41566.1.S1_at	5.82	5.71	7.63	4.95	5.64	5.54	6.39	1.08	5.38	0.37	0.84	-0.248	2.0E-01
Mtr.31078.1.S1_at	2.31	2.38	4.21	2.22	2.34	2.28	2.97	1.08	2.28	0.06	0.77	-0.381	3.3E-01
Mtr.42444.1.S1_at	5.40	3.90	5.99	6.07	5.83	6.49	5.09	1.08	6.13	0.33	1.20	0.267	1.9E-01
Mtr.28519.1.S1_at	6.61	4.56	6.15	6.99	6.84	6.70	5.77	1.08	6.84	0.14	1.19	0.246	1.6E-01
Mtr.5846.1.S1_at	5.07	3.96	6.12	5.13	5.12	5.03	5.05	1.08	5.09	0.06	1.01	0.013	9.5E-01
Mtr.50299.1.S1_s_at	9.21	7.12	8.62	9.00	9.03	9.04	8.32	1.08	9.03	0.02	1.09	0.118	3.2E-01
Mtr.21662.1.S1_at	11.32	9.35	9.57	10.02	10.76	10.58	10.08	1.08	10.45	0.38	1.04	0.052	6.0E-01
Mtr.25159.1.S1_at	2.09	3.95	2.07	2.07	2.07	2.07	2.70	1.08	2.07	0.00	0.77	-0.384	3.7E-01
Mtr.12291.1.S1_s_at	7.19	6.19	8.34	7.77	8.47	8.07	7.24	1.08	8.10	0.35	1.12	0.161	2.6E-01
Mtr.37806.1.S1_at	10.86	9.15	11.13	10.89	11.02	10.62	10.38	1.08	10.85	0.20	1.04	0.063	5.0E-01
Mtr.15775.1.S1_at	11.52	10.03	12.12	11.97	11.86	11.69	11.22	1.08	11.84	0.14	1.05	0.077	3.8E-01
Mtr.12805.1.S1_at	10.73	8.99	10.96	10.60	10.67	10.45	10.22	1.08	10.57	0.11	1.03	0.049	6.0E-01
Mtr.18694.1.S1_x_at	3.06	5.17	3.77	3.66	3.57	3.62	4.00	1.08	3.62	0.04	0.90	-0.146	5.7E-01
Mtr.9145.1.S1_at	6.93	5.04	6.88	6.78	7.05	6.51	6.29	1.08	6.78	0.27	1.08	0.109	4.8E-01
Mtr.31276.1.S1_at	5.28	3.16	4.56	3.11	3.52	3.11	4.33	1.08	3.25	0.24	0.75	-0.416	1.6E-01
Mtr.20071.1.S1_at	5.05	3.44	5.49	4.66	4.83	4.83	4.66	1.08	4.77	0.10	1.02	0.034	8.7E-01
Mtr.19666.1.S1_at	6.69	4.54	5.72	5.67	5.69	5.93	5.65	1.08	5.76	0.14	1.02	0.028	8.7E-01
Mtr.27646.1.S1_s_at	2.04	3.83	1.91	1.90	1.58	1.94	2.60	1.08	1.81	0.20	0.70	-0.520	2.8E-01
Mtr.42038.1.S1_at	8.16	6.29	8.15	9.09	8.91	8.83	7.53	1.08	8.94	0.13	1.19	0.247	8.7E-02
Mtr.22800.1.S1_at	8.50	6.48	8.12	8.05	8.14	8.13	7.70	1.08	8.11	0.05	1.05	0.074	5.5E-01
Mtr.47610.1.S1_at	2.44	4.28	2.40	2.40	2.40	2.39	3.04	1.07	2.40	0.00	0.79	-0.344	3.6E-01
Mtr.32892.1.S1_at	4.82	4.43	6.45	6.08	6.00	6.23	5.23	1.07	6.10	0.12	1.17	0.222	2.4E-01
Mtr.31175.1.S1_at	6.90	5.23	7.24	6.58	6.80	6.41	6.46	1.07	6.60	0.19	1.02	0.032	8.3E-01
Mtr.9236.1.S1_at	8.98	7.42	9.48	8.92	8.78	8.67	8.63	1.07	8.79	0.13	1.02	0.027	8.1E-01
Mtr.37345.1.S1_at	11.72	9.66	11.21	11.38	11.51	11.18	10.86	1.07	11.36	0.16	1.05	0.064	4.8E-01
Mtr.26082.1.S1_at	5.63	7.64	7.29	6.80	7.03	6.33	6.85	1.07	6.72	0.36	0.98	-0.028	8.5E-01
Mtr.17121.1.S1_at	4.21	5.62	3.52	3.48	3.46	3.38	4.45	1.07	3.44	0.05	0.77	-0.371	1.8E-01
Mtr.25354.1.S1_s_at	4.97	3.05	4.85	4.72	4.72	4.50	4.29	1.07	4.65	0.13	1.08	0.116	6.0E-01
Mtr.5635.1.S1_at	8.31	7.55	9.67	9.34	8.74	9.12	8.51	1.07	9.07	0.30	1.07	0.092	4.3E-01
Mtr.11609.1.S1_at	8.48	6.60	8.44	8.19	8.27	8.31	7.84	1.07	8.26	0.07	1.05	0.075	5.4E-01
Mtr.16868.1.S1_at	2.63	4.47	2.60	2.60	2.52	2.59	3.23	1.07	2.57	0.04	0.79	-0.332	3.4E-01
Mtr.48082.1.S1_at	6.52	4.56	6.31	7.67	7.33	7.77	5.79	1.07	7.59	0.23	1.31	0.389	4.7E-02
Mtr.34115.1.S1_at	2.48	4.33	2.47	2.46	2.44	2.45	3.09	1.07	2.45	0.01	0.79	-0.337	3.6E-01
Mtr.40154.1.S1_at	7.99	5.85	6.90	6.09	6.29	6.49	6.91	1.07	6.29	0.20	0.91	-0.136	3.8E-01
Mtr.49843.1.S1_s_at	2.95	4.78	2.90	2.89	2.84	2.99	3.54	1.07	2.91	0.08	0.82	-0.285	3.6E-01
Mtr.11904.1.S1_at	5.26	3.39	5.24	5.16	5.42	5.20	4.63	1.07	5.26	0.14	1.14	0.185	3.7E-01
Mtr.9135.1.S1_at	7.71	5.66	7.23	7.53	7.44	7.59	6.87	1.07	7.52	0.07	1.10	0.131	3.5E-01
Mtr.6178.1.S1_at	6.23	5.23	7.37	6.27	6.96	6.19	6.27	1.07	6.47	0.42	1.03	0.045	7.8E-01
Mtr.6850.1.S1_at	7.08	5.23	7.08	6.96	7.00	6.67	6.46	1.07	6.88	0.18	1.06	0.089	5.5E-01
Mtr.4452.1.S1_at	5.36	3.54	5.42	5.52	6.59	5.41	4.77	1.07	5.84	0.65	1.22	0.291	2.1E-01
Mtr.6357.1.S1_at	4.72	2.77	4.51	4.42	4.51	5.15	4.00	1.07	4.69	0.40	1.17	0.230	3.5E-01
Mtr.10063.1.S1_at	5.12	3.33	3.20	3.17	3.21	3.17	3.88	1.07	3.18	0.02	0.82	-0.286	3.2E-01
Mtr.12866.1.S1_at	9.60	7.46	8.54	8.55	8.77	8.63	8.54	1.07	8.65	0.11	1.01	0.020	8.6E-01
Mtr.12309.1.S1_at	8.92	7.00	8.79	8.98	9.11	8.71	8.24	1.07	8.93	0.20	1.08	0.117	3.3E-01
Mtr.36297.1.S1_at	2.59	4.43	2.55	2.59	2.59	2.58	3.19	1.07	2.59	0.00	0.81	-0.303	3.8E-01
Mtr.42718.1.S1_at	4.03	2.46	4.51	4.33	4.00	4.99	3.67	1.07	4.44	0.50	1.21	0.275	3.2E-01
Mtr.44119.1.S1_at	8.76	7.09	9.09	9.31	9.37	9.04	8.32	1.07	9.24	0.17	1.11	0.152	2.1E-01
Mtr.12159.1.S1_at	6.28	4.48	6.38	6.39	6.44	6.17	5.71	1.07	6.33	0.14	1.11	0.149	3.8E-01
Mtr.28675.1.S1_s_at	6.73	5.16	7.20	6.82	6.90	6.16	6.36	1.07	6.63	0.41	1.04	0.058	7.1E-01
Mtr.33080.1.S1_at	6.06	4.75	6.87	6.67	6.81	6.70	5.89	1.07	6.72	0.07	1.14	0.190	2.5E-01
Mtr.10829.1.S1_at	10.32	8.64	10.63	10.05	10.00	9.87	9.86	1.07	9.97	0.09	1.01	0.016	8.7E-01
Mtr.37637.1.S1_at	12.55	10.53	12.17	12.34	12.49	12.11	11.75	1.07	12.31	0.19	1.05	0.067	4.2E-01
Mtr.46626.1.S1_at	2.38	4.23	2.37	2.37	2.37	2.36	2.99	1.07	2.37	0.01	0.79	-0.339	3.7E-01
Mtr.2003.1.S1_at	6.34	4.26	5.75	6.86	6.39	6.34	5.45	1.07	6.53	0.29	1.20	0.261	1.7E-01
Mtr.3561.1.S1_at	10.17	8.74	10.84	10.61	10.79	10.37	9.92	1.07	10.59	0.21	1.07	0.094	3.5E-01
Mtr.5646.1.S1_at	10.91	8.77	9.75	9.49	8.99	9.38	9.81	1.07	9.29	0.26	0.95	-0.078	4.6E-01
Mtr.5387.1.S1_s_at	8.06	6.26	8.16	8.16	8.10	8.11	7.49	1.07	8.12	0.03	1.08	0.117	3.6E-01
Mtr.27891.1.S1_at	6.17	4.83	6.95	6.34	6.01	6.51	5.98	1.07	6.29	0.25	1.05	0.072	6.6E-01
Mtr.36868.1.S1_at	2.64	4.48	2.62	2.61	2.61	2.60	3.25	1.07	2.60	0.01	0.80	-0.318	3.6E-01
Mtr.13274.1.S1_at	2.68	2.82	4.60	6.06	6.31	5.60	3.37	1.07	5.99	0.36	1.78	0.830	1.6E-02
Mtr.49164.1.S1_at	6.46	5.07	7.17	6.73	7.19	6.78	6.23	1.07	6.90	0.25	1.11	0.147	3.5E-01
Mtr.37202.1.S1_at	8.48	6.39	7.05	7.79	7.65	7.68	7.30	1.07	7.71	0.08	1.05	0.077	5.5E-01
Mtr.40615.1.S1_at	10.03	7.92	9.27	10.24	10.53	10.40	9.07	1.07	10.39	0.14	1.15	0.196	1.0E-01
Mtr.35232.1.S1_at	6.00	4.18	6.06	6.04	6.05	6.07	5.41	1.07	6.05	0.02	1.12	0.161	3.6E-01
Mtr.30708.1.S1_at	5.40	4.34	6.48	5.64	5.86	5.56	5.41	1.07	5.69	0.16	1.05	0.072	6.8E-01
Mtr.7720.1.S1_at	2.31	4.16	2.30	2.29	2.29	2.28	2.92	1.07	2.29	0.01	0.78	-0.353	3.6E-01
Mtr.44061.1.S1_at	6.74	4.71	6.31	6.89	6.66	6.68	5.92	1.07	6.74	0.13	1.14	0.187	2.6E-01
Mtr.370.1.S1_at	6.48	5.16	7.28	6.52	6.92	6.37	6.31	1.07	6.60	0.29	1.05	0.067	6.7E-01
Mtr.3827.1.S1_at	5.80	4.02	5.94	5.58	5.78	6.15	5.25	1.07	5.84	0.29	1.11	0.152	4.1E-01
Mtr.38949.1.S1_x_at	3.08	4.91	3.05	3.05	3.00	2.99	3.68	1.07	3.01	0.03	0.82	-0.290	3.4E-01
Mtr.16273.1.S1_at	5.92	4.70	6.82	6.61	6.66	6.68	5.81	1.07	6.65	0.04	1.14	0.193	2.5E-01
Mtr.13533.1.S1_at	6.19	4.63	6.66	5.88	6.33	6.03	5.83	1.06	6.08	0.23	1.04	0.061	7.1E-01
Mtr.42815.1.S1_at	5.05	3.41	3.06	3.13	3.04	2.98	3.84	1.06	3.05	0.08	0.80	-0.330	2.7E-01
Mtr.7063.1.S1_at	8.73	6.71	7.13	7.23	7.17	7.40	7.52	1.06	7.26	0.12	0.97	-0.050	7.0E-01
Mtr.32212.1.S1_at	7.39	5.47	7.23	7.23	7.21	7.54	6.70	1.06	7.33	0.19	1.09	0.130	3.7E-01
Mtr.50278.1.S1_at	11.13	9.63	11.68	11.54	11.50	11.21	10.81	1.06	11.42	0.18	1.06	0.078	3.9E-01
Mtr.43116.1.S1_at	7.37	9.40	8.94	8.68	9.23	8.85	8.57	1.06	8.92	0.28	1.04	0.057	6.1E-01
Mtr.2510.1.S1_at	5.11	3.18	4.93	4.63	4.51	5.15	4.41	1.06	4.76	0.34	1.08	0.112	6.1E-01
Mtr.23070.1.S1_at	6.78	5.31	7.38	6.85	6.93	6.59	6.49	1.06	6.79	0.18	1.05	0.066	6.5E-01
Mtr.44534.1.S1_at	10.73	8.91	8.86	9.16	8.48	8.84	9.50	1.06	8.83	0.34	0.93	-0.106	3.6E-01
Mtr.44634.1.S1_at	3.74	5.79	4.29	5.53	6.75	6.21	4.61	1.06	6.17	0.61	1.34	0.421	9.2E-02
Mtr.10952.1.S1_at	5.84	4.57	6.69	6.00	6.08	6.19	5.70	1.06	6.09	0.09	1.07	0.096	5.6E-01
Mtr.25634.1.S1_at	7.29	5.41	7.21	7.46	7.34	7.33	6.64	1.06	7.38	0.07	1.11	0.153	3.0E-01
Mtr.5694.1.S1_at	7.42	5.55	7.37	7.58	7.80	7.51	6.78	1.06	7.63	0.15	1.13	0.170	2.4E-01
Mtr.14023.1.S1_at	8.14	6.23	7.98	7.83	7.81	7.95	7.45	1.06	7.86	0.08	1.06	0.078	5.4E-01
Mtr.32058.1.S1_at	6.16	4.35	6.22	6.07	5.78	6.30	5.58	1.06	6.05	0.26	1.08	0.117	5.0E-01
Mtr.50516.1.S1_at	4.38	6.49	5.65	3.59	3.39	2.73	5.50	1.06	3.24	0.45	0.59	-0.766	2.7E-02
Mtr.37181.1.S1_at	13.49	11.89	13.90	13.64	13.53	13.50	13.09	1.06	13.56	0.08	1.04	0.050	4.9E-01
Mtr.17733.1.S1_at	5.20	3.19	3.59	3.84	3.63	3.58	3.99	1.06	3.68	0.14	0.92	-0.117	6.4E-01
Mtr.48606.1.S1_at	4.52	4.83	6.50	4.60	7.14	5.84	5.28	1.06	5.86	1.27	1.11	0.149	5.8E-01
Mtr.14220.1.S1_at	2.87	4.70	2.85	2.84	2.79	2.82	3.48	1.06	2.82	0.03	0.81	-0.304	3.4E-01
Mtr.7891.1.S1_at	5.87	4.39	6.45	5.60	6.12	5.66	5.57	1.06	5.79	0.29	1.04	0.057	7.4E-01
Mtr.27404.1.S1_at	6.74	4.84	6.61	6.50	6.40	6.19	6.06	1.06	6.36	0.16	1.05	0.070	6.5E-01
Mtr.2821.1.S1_s_at	8.05	6.29	8.20	7.76	8.40	7.96	7.51	1.06	8.04	0.33	1.07	0.098	4.6E-01
Mtr.23981.1.S1_s_at	11.48	9.49	11.14	11.58	11.27	11.32	10.71	1.06	11.39	0.17	1.06	0.090	3.3E-01
Mtr.29086.1.S1_at	9.28	7.52	7.37	8.02	7.39	8.32	8.06	1.06	7.91	0.48	0.98	-0.026	8.4E-01
Mtr.4394.1.S1_at	5.08	3.03	4.53	4.84	4.70	4.89	4.21	1.06	4.81	0.10	1.14	0.193	3.8E-01
Mtr.2228.1.S1_at	5.47	3.78	5.74	5.38	5.43	5.38	5.00	1.06	5.40	0.03	1.08	0.110	5.5E-01

Mtr.44035.1.S1_s_at	8.06	6.20	8.01	7.80	7.92	7.86	7.42	1.06	7.86	0.06	1.06	0.082	5.2E-01
Mtr.9508.1.S1_at	7.60	6.63	8.75	8.78	8.92	8.78	7.66	1.06	8.83	0.08	1.15	0.204	1.3E-01
Mtr.2856.1.S1_at	2.08	3.92	2.08	2.07	2.07	2.12	2.69	1.06	2.08	0.03	0.77	-0.370	3.8E-01
Mtr.35546.1.S1_at	7.27	5.26	6.86	7.35	7.35	7.44	6.46	1.06	7.38	0.05	1.14	0.192	2.1E-01
Mtr.33588.1.S1_at	8.07	6.44	8.42	8.11	8.27	8.01	7.64	1.06	8.13	0.13	1.06	0.089	4.7E-01
Mtr.31558.1.S1_at	6.62	5.75	7.86	7.13	7.44	6.87	6.74	1.06	7.15	0.29	1.06	0.085	5.6E-01
Mtr.39152.1.S1_at	5.50	3.51	5.13	5.00	5.04	5.16	4.72	1.06	5.07	0.08	1.07	0.103	6.0E-01
Mtr.39516.1.S1_at	8.13	6.26	8.07	8.10	7.94	8.27	7.49	1.06	8.10	0.16	1.08	0.114	3.8E-01
Mtr.20948.1.S1_s_at	4.25	2.47	2.37	2.88	3.09	3.82	3.03	1.06	3.26	0.49	1.08	0.107	7.5E-01
Mtr.40060.1.S1_at	8.51	6.68	8.52	8.73	8.52	8.60	7.90	1.06	8.62	0.11	1.09	0.125	3.1E-01
Mtr.3377.1.S1_at	3.43	3.66	5.37	3.76	3.59	3.56	4.15	1.06	3.64	0.10	0.88	-0.192	4.5E-01
Mtr.39308.1.S1_s_at	9.55	11.61	10.19	10.70	10.93	10.74	10.45	1.06	10.79	0.12	1.03	0.046	6.1E-01
Mtr.50095.1.S1_at	7.00	5.08	6.81	6.82	6.70	6.87	6.30	1.06	6.80	0.09	1.08	0.110	4.6E-01
Mtr.2159.1.S1_at	6.83	5.52	7.62	7.15	7.12	6.96	6.66	1.06	7.08	0.10	1.06	0.088	5.3E-01
Mtr.37814.1.S1_at	9.74	7.74	8.13	9.21	8.96	9.32	8.53	1.06	9.16	0.18	1.07	0.103	3.7E-01
Mtr.38078.1.S1_at	8.40	6.65	8.55	8.32	8.49	8.28	7.87	1.06	8.37	0.11	1.06	0.089	4.6E-01
Mtr.28085.1.S1_at	9.79	8.03	7.89	8.54	8.41	8.76	8.57	1.06	8.57	0.18	1.00	0.000	1.0E+00
Mtr.9864.1.S1_at	8.43	7.18	9.28	8.65	8.68	8.70	8.30	1.06	8.68	0.03	1.05	0.065	5.7E-01
Mtr.9718.1.S1_at	7.61	5.65	7.34	7.53	7.44	7.81	6.87	1.06	7.60	0.19	1.11	0.146	3.0E-01
Mtr.47674.1.S1_at	5.52	4.04	6.09	5.71	5.35	5.54	5.22	1.06	5.53	0.18	1.06	0.084	6.4E-01
Mtr.31518.1.S1_at	8.36	6.48	8.26	7.75	8.00	7.72	7.70	1.06	7.82	0.16	1.02	0.022	8.6E-01
Mtr.35429.1.S1_at	8.97	7.21	9.12	8.50	8.95	8.42	8.43	1.06	8.62	0.29	1.02	0.032	7.8E-01
Mtr.43393.1.S1_at	11.42	9.63	9.54	10.09	10.39	10.48	10.20	1.06	10.32	0.21	1.01	0.018	8.5E-01
Mtr.32626.1.S1_at	8.96	6.89	8.32	9.14	8.92	9.29	8.06	1.06	9.12	0.19	1.13	0.178	1.6E-01
Mtr.51154.1.S1_at	11.20	13.08	11.31	12.41	12.52	12.33	11.86	1.06	12.42	0.09	1.05	0.066	4.2E-01
Mtr.738.1.S1_at	7.28	5.75	7.79	7.69	7.83	7.48	6.94	1.06	7.67	0.17	1.10	0.144	3.0E-01
Mtr.50411.1.S1_at	3.34	2.04	4.14	4.05	4.11	3.69	3.17	1.06	3.95	0.23	1.24	0.316	2.8E-01
Mtr.44502.1.S1_at	7.67	5.72	7.42	7.37	7.61	7.57	6.94	1.06	7.52	0.13	1.08	0.116	4.0E-01
Mtr.44254.1.S1_at	11.40	9.73	11.69	11.75	11.67	11.57	10.94	1.06	11.66	0.09	1.07	0.093	3.0E-01
Mtr.46133.1.S1_at	2.79	4.61	2.77	2.72	2.68	2.64	3.39	1.06	2.68	0.04	0.79	-0.340	3.1E-01
Mtr.13724.1.S1_at	6.95	5.12	6.95	7.00	7.02	7.33	6.34	1.06	7.12	0.18	1.12	0.167	2.8E-01
Mtr.49305.1.S1_at	2.63	2.74	4.51	2.97	3.77	2.80	3.30	1.06	3.18	0.52	0.96	-0.053	8.7E-01
Mtr.38648.1.S1_at	6.71	4.65	5.29	4.90	5.35	5.17	5.55	1.06	5.14	0.23	0.93	-0.110	5.5E-01
Mtr.43969.1.S1_s_at	8.98	7.20	9.09	8.56	8.58	8.78	8.42	1.06	8.64	0.12	1.03	0.037	7.4E-01
Mtr.40684.1.S1_at	6.87	4.83	6.32	6.14	6.08	6.87	6.01	1.06	6.37	0.44	1.06	0.083	6.2E-01
Mtr.47990.1.S1_at	9.86	7.80	8.41	8.47	8.66	8.54	8.69	1.06	8.56	0.10	0.98	-0.022	8.4E-01
Mtr.27562.1.S1_at	4.04	5.86	4.02	4.03	4.02	4.03	4.64	1.06	4.03	0.00	0.87	-0.206	3.7E-01
Mtr.19371.1.S1_at	9.40	7.51	9.27	9.08	8.93	8.83	8.72	1.06	8.95	0.13	1.03	0.037	7.3E-01
Mtr.41387.1.S1_at	8.20	6.45	6.30	6.25	5.41	6.37	6.98	1.06	6.01	0.52	0.86	-0.216	2.3E-01
Mtr.43807.1.S1_at	10.97	8.93	9.49	10.07	9.98	9.93	9.80	1.06	9.99	0.07	1.02	0.029	7.6E-01
Mtr.10898.1.S1_at	9.72	7.61	8.65	8.60	8.17	8.50	8.66	1.06	8.42	0.22	0.97	-0.040	7.2E-01
Mtr.6152.1.S1_at	4.84	3.10	5.01	4.75	5.13	4.97	4.32	1.06	4.95	0.19	1.15	0.197	3.7E-01
Mtr.33176.1.S1_at	8.11	6.26	8.07	7.84	7.80	7.82	7.48	1.06	7.82	0.02	1.05	0.064	6.1E-01
Mtr.11292.1.S1_s_at	7.73	5.90	7.72	7.56	7.57	7.61	7.11	1.06	7.58	0.03	1.07	0.091	4.9E-01
Mtr.7315.1.S1_at	7.19	5.39	7.24	6.95	7.39	7.09	6.61	1.06	7.14	0.23	1.08	0.112	4.4E-01
Mtr.3655.1.S1_at	5.43	3.60	5.43	5.60	5.66	5.43	4.82	1.06	5.56	0.12	1.15	0.207	2.9E-01
Mtr.4342.1.S1_at	6.59	4.65	6.33	6.22	6.52	6.33	5.85	1.05	6.36	0.15	1.09	0.119	4.6E-01
Mtr.28711.1.S1_at	8.36	6.54	8.37	8.06	8.26	7.86	7.75	1.05	8.06	0.20	1.04	0.056	6.5E-01
Mtr.46937.1.S1_at	3.96	5.72	3.83	4.00	3.53	3.88	4.50	1.05	3.81	0.24	0.85	-0.242	3.3E-01
Mtr.42189.1.S1_at	5.01	2.93	3.66	5.47	7.45	5.23	3.86	1.05	6.05	1.22	1.57	0.647	7.9E-02
Mtr.42321.1.S1_at	6.74	4.69	6.13	6.80	7.19	7.14	5.86	1.05	7.04	0.21	1.20	0.266	1.3E-01
Mtr.13594.1.S1_s_at	8.15	6.31	8.13	7.77	7.59	7.25	7.53	1.05	7.53	0.26	1.00	0.001	9.9E-01
Mtr.34640.1.S1_at	9.23	7.42	9.27	8.90	8.78	8.79	8.64	1.05	8.82	0.06	1.02	0.030	7.8E-01
Mtr.42672.1.S1_at	5.11	3.27	5.08	5.74	5.17	5.47	4.49	1.05	5.46	0.29	1.22	0.283	2.0E-01
Mtr.37836.1.S1_at	7.43	5.96	8.00	8.16	8.15	8.03	7.13	1.05	8.11	0.07	1.14	0.186	1.8E-01
Mtr.8498.1.S1_s_at	13.23	11.71	11.20	12.32	12.77	12.46	12.05	1.05	12.52	0.23	1.04	0.055	4.9E-01
Mtr.36668.1.S1_at	2.48	4.31	2.49	2.48	2.37	2.51	3.09	1.05	2.45	0.08	0.79	-0.335	3.5E-01
Mtr.23442.1.S1_at	2.57	4.34	2.47	2.52	2.51	2.50	3.13	1.05	2.51	0.01	0.80	-0.318	3.7E-01
Mtr.40084.1.S1_at	12.57	10.68	10.81	11.65	11.47	11.71	11.35	1.05	11.61	0.13	1.02	0.032	7.0E-01
Mtr.11085.1.S1_at	13.75	12.22	11.73	13.27	12.67	13.37	12.57	1.05	13.11	0.38	1.04	0.061	4.5E-01
Mtr.2444.1.S1_at	7.35	5.51	7.32	7.43	7.36	7.34	6.73	1.05	7.37	0.05	1.10	0.132	3.5E-01
Mtr.7722.1.S1_at	6.13	4.03	5.19	5.58	5.44	5.45	5.12	1.05	5.49	0.08	1.07	0.101	5.7E-01
Mtr.50623.1.S1_at	7.20	5.31	7.06	7.19	7.43	7.10	6.52	1.05	7.24	0.17	1.11	0.151	3.1E-01
Mtr.46410.1.S1_at	6.73	4.79	6.47	6.49	7.03	6.81	6.00	1.05	6.77	0.27	1.13	0.176	2.8E-01
Mtr.51473.1.S1_at	3.25	4.94	3.01	3.09	3.14	3.23	3.73	1.05	3.15	0.07	0.84	-0.245	3.9E-01
Mtr.19471.1.S1_at	4.99	6.76	4.88	5.20	5.28	5.21	5.55	1.05	5.23	0.04	0.94	-0.084	6.3E-01
Mtr.47837.1.S1_at	7.07	5.46	7.44	5.85	6.54	6.46	6.66	1.05	6.29	0.38	0.94	-0.083	6.0E-01
Mtr.36918.1.S1_at	7.31	5.36	7.00	7.12	7.02	6.91	6.56	1.05	7.02	0.10	1.07	0.098	4.9E-01
Mtr.38588.1.S1_at	8.55	6.70	8.48	8.29	8.13	8.31	7.91	1.05	8.24	0.10	1.04	0.059	6.1E-01
Mtr.38479.1.S1_at	8.64	6.62	8.14	7.89	7.93	7.68	7.80	1.05	7.84	0.13	1.00	0.007	9.6E-01
Mtr.33795.1.S1_at	7.69	5.75	7.41	7.95	8.23	8.19	6.95	1.05	8.13	0.15	1.17	0.226	1.3E-01
Mtr.36172.1.S1_at	4.46	2.87	4.85	3.68	4.27	3.77	4.06	1.05	3.91	0.32	0.96	-0.055	8.2E-01
Mtr.25882.1.S1_at	5.10	3.16	4.82	4.72	4.30	4.54	4.36	1.05	4.52	0.21	1.04	0.053	8.1E-01
Mtr.5236.1.S1_s_at	10.82	10.35	8.81	10.48	10.50	11.08	10.00	1.05	10.69	0.34	1.07	0.096	3.4E-01
Mtr.42019.1.S1_at	8.02	6.02	7.57	7.98	7.65	7.71	7.20	1.05	7.78	0.17	1.08	0.111	4.0E-01
Mtr.33282.1.S1_s_at	6.56	4.69	6.44	6.52	6.28	6.43	5.90	1.05	6.41	0.12	1.09	0.120	4.5E-01
Mtr.37242.1.S1_at	5.95	4.15	6.00	6.11	5.99	5.60	5.37	1.05	5.90	0.27	1.10	0.137	4.4E-01
Mtr.29529.1.S1_at	8.84	7.02	7.03	7.64	7.67	7.44	7.63	1.05	7.58	0.13	0.99	-0.009	9.4E-01
Mtr.33063.1.S1_at	2.61	4.42	2.60	2.56	2.56	2.55	3.21	1.05	2.56	0.00	0.80	-0.330	3.4E-01
Mtr.3275.1.S1_at	4.59	3.20	2.53	2.67	2.47	2.44	3.44	1.05	2.53	0.12	0.74	-0.443	2.1E-01
Mtr.43047.1.S1_at	13.40	14.37	12.27	12.77	12.42	12.28	13.35	1.05	12.49	0.25	0.94	-0.095	2.4E-01
Mtr.28182.1.S1_at	7.50	5.86	7.82	7.70	7.63	7.39	7.06	1.05	7.57	0.16	1.07	0.101	4.5E-01
Mtr.37363.1.S1_at	6.44	5.85	4.40	4.01	4.07	4.96	5.56	1.05	4.35	0.53	0.78	-0.356	1.5E-01
Mtr.17573.1.S1_at	7.51	5.68	7.49	7.11	7.22	7.50	6.89	1.05	7.28	0.20	1.06	0.078	5.7E-01
Mtr.38046.1.S1_s_at	8.95	6.86	7.79	7.92	7.22	7.73	7.86	1.05	7.62	0.36	0.97	-0.045	7.2E-01
Mtr.15516.1.S1_at	9.41	11.45	10.00	10.65	10.84	10.53	10.29	1.05	10.68	0.16	1.04	0.053	5.6E-01
Mtr.32322.1.S1_at	5.76	3.69	5.02	5.38	5.32	5.04	4.82	1.05	5.25	0.18	1.09	0.121	5.3E-01
Mtr.1101.1.S1_s_at	6.93	5.47	7.50	6.91	7.44	6.93	6.63	1.05	7.09	0.30	1.07	0.097	5.0E-01
Mtr.35696.1.S1_s_at	9.45	7.36	8.29	8.88	8.64	9.05	8.36	1.05	8.86	0.21	1.06	0.083	4.7E-01
Mtr.45992.1.S1_at	3.73	5.12	3.06	6.08	6.66	6.56	3.97	1.05	6.43	0.31	1.62	0.695	1.8E-02
Mtr.20075.1.S1_at	4.60	2.89	4.80	4.58	4.45	4.62	4.09	1.05	4.55	0.09	1.11	0.153	4.9E-01
Mtr.27440.1.S1_at	9.12	7.42	9.33	8.92	8.96	8.68	8.62	1.05	8.86	0.15	1.03	0.038	7.2E-01
Mtr.32665.1.S1_s_at	11.96	10.00	10.32	10.72	9.87	10.86	10.76	1.05	10.48	0.54	0.97	-0.037	7.1E-01
Mtr.3114.1.S1_at	6.93	5.33	7.31	6.80	7.30	6.82	6.52	1.05	6.98	0.28	1.07	0.097	5.1E-01
Mtr.30501.1.S1_at	2.39	4.19	2.36	2.37	2.35	2.34	2.98	1.05	2.35	0.02	0.79	-0.340	3.6E-01
Mtr.18213.1.S1_at	2.47	4.27	2.45	2.43	2.42	2.41	3.06	1.05	2.42	0.01	0.79	-0.339	3.5E-01
Mtr.33916.1.S1_at	8.91	7.28	9.23	9.53	9.95	9.77	8.47	1.05	9.75	0.21	1.15	0.203	1.1E-01
Mtr.32726.1.S1_at	4.95	4.01	6.10	6.29	6.88	6.77	5.02	1.05	6.65	0.31	1.32	0.406	6.1E-02
Mtr.51694.1.S1_at	2.40	4.22	2.41	2.38	2.39	2.38	3.01	1.05	2.38	0.00	0.79	-0.335	3.6E-01
Mtr.16669.1.S1_at	3.75	2.29	4.32	4.75	5.12	4.78	3.46	1.05	4.88	0.21	1.41	0.499	8.1E-02
Mtr.5418.1.S1_at	6.10	4.39	4.20	5.84	5.85	6.10	4.90	1.05	5.93	0.15	1.21	0.275	1.7E-01
Mtr.769.1.S1_at	7.96	6.14	7.95	7.93	7.88	8.02	7.35	1.05	7.94	0.07	1.08	0.112	3.8E-01
Mtr.43424.1.S1_at	6.14	4.29	6.05	6.32	6.18	6.14	5.49	1.05	6.21	0.10	1.13	0.177	3.0E-01
Mtr.8672.1.S1_at	10.63	8.56	9.29	9.21	9.72	9.39	9.49	1.05	9.44	0.26	0.99	-0.008	9.3E-01
Mtr.15770.1.S1_at	7.92	6.49	8.53	8.17	8.10	7.90	7.65	1.05	8.06	0.14	1.05	0.075	5.4E-01
Mtr.33794.1.S1_at	10.49	8.40	9.44	9.76	9.69	9.95	9.44	1.05	9.80	0.14	1.04	0.054	5.9E-01
Mtr.42740.1.S1_at	7.48	5.54	7.19	7.54	7.53	7.68	6.74	1.05	7.58	0.08	1.13	0.171	2.4E-01
Mtr.44319.1.S1_at	4.73	3.09	5.04	5.08	5.16	4.12	4.29	1.05	4.79	0.58	1.12	0.160	5.1E-01
Mtr.27987.1.S1_at	6.75	4.75	6.29	5.60	5.74	5.40	5.93	1.05	5.58	0.17	0.94	-0.087	6.0E-01
Mtr.5185.1.S1_at	7.68	6.19	8.21	7.78	7.76	7.81	7.36	1.05	7.78	0.02	1.06	0.081	5.2E-01
Mtr.43644.1.S1_at	7.39	5.73	7.66	7.84	6.31	7.76	6.93	1.05	7.31	0.86	1.05	0.076	6.6E-01
Mtr.10332.1.S1_at	11.54	9.59	11.21	11.99	11.99	12.15	10.78	1.05	12.04	0.09	1.12	0.160	1.1E-01
Mtr.51379.1.S1_at	6.35	4.33	5.81	6.33	5.93	6.34	5.50	1.05	6.20	0.23	1.13	0.174	3.2E-01
Mtr.41153.1.S1_at	5.28	3.52	5.38	5.26	5.73	5.58	4.73	1.04	5.52	0.24	1.17	0.224	2.7E-01
Mtr.45065.1.S1_at	7.19	5.56	7.51	7.08	7.34	7.02	6.75	1.04	7.15	0.17	1.06	0.082	5.5E-01
Mtr.20408.1.S1_s_at	8.87	6.98	8.70	8.87	9.66	8.92	8.19	1.04	9.15	0.45	1.12	0.161	2.1E-01
Mtr.45451.1.S1_at	7.00	5.41	7.39	7.25	6.89	7.00	6.60	1.04	7.05	0.18	1.07	0.095	5.0E-01
Mtr.37633.1.S1_at	8.00	6.06	7.68	7.63	8.06	7.60	7.25	1.04	7.76	0.26	1.07	0.099	4.5E-01
Mtr.29387.1.S1_at	4.55	2.66	4.38	4.31	4.51	4.27	3.86	1.04	4.36	0.13	1.13	0.176	4.6E-01
Mtr.43526.1.S1_at	10.06	7.98	9.15	9.78	9.80	9.80	9.06	1.04	9.79	0.01	1.08	0.112	2.9E-01
Mtr.23683.1.S1_at	2.59	4.25	2.32	2.58	2.58	2.64	3.05	1.04	2.60	0.04	0.85	-0.232	4.9E-01
Mtr.44604.1.S1_at	9.07	7.01	8.30	8.89	9.18	9.08	8.13	1.04	9.05	0.15	1.11	0.155	2.0E-01
Mtr.12140.1.S1_at	5.50	3.93	5.91	6.13	5.67	5.70	5.11	1.04	5.83	0.26	1.14	0.190	3.1E-01
Mtr.7692.1.S1_at	3.38	4.97	3.00	3.16	2.66	3.13	3.78	1.04	2.99	0.28	0.79	-0.341	2.7E-01
Mtr.47069.1.S1_at	2.22	4.02	2.21	2.20	2.20	2.20	2.82	1.04	2.20	0.00	0.78	-0.357	3.6E-01
Mtr.27766.1.S1_at	2.86	4.57	2.68	2.79	2.80	2.77	3.37	1.04	2.78	0.01	0.83	-0.275	3.9E-01
Mtr.23682.1.S1_at	2.36	4.17	2.36	2.35	2.35	2.34	2.96	1.04	2.34	0.01	0.79	-0.338	3.6E-01
Mtr.11427.1.S1_at	6.93	5.01	6.67	7.08	7.49	6.87	6.20	1.04	7.15	0.32	1.15	0.205	2.1E-01
Mtr.25205.1.S1_at	6.94	5.42	7.41	6.93	6.97	6.92	6.59	1.04	6.94	0.02	1.05	0.075	5.9E-01
Mtr.25997.1.S1_at	3.44	5.19	3.33	3.32	3.43	3.31	3.99	1.04	3.35	0.07	0.84	-0.250	3.5E-01
Mtr.5705.1.S1_at	7.25	5.18	6.07	7.33	7.41	7.53	6.17	1.04	7.42	0.10	1.20	0.267	1.1E-01

Mtr.39514.1.S1_at	9.12	7.43	9.33	8.91	8.95	8.90	8.63	1.04	8.92	0.02	1.03	0.048	6.5E-01
Mtr.18417.1.S1_s_at	5.77	4.10	6.01	6.63	6.19	6.24	5.29	1.04	6.35	0.24	1.20	0.264	1.6E-01
Mtr.37609.1.S1_s_at	13.24	11.28	12.87	11.92	11.38	11.90	12.46	1.04	11.73	0.30	0.94	-0.087	3.1E-01
Mtr.14881.1.S1_at	5.92	3.84	4.85	4.86	4.85	4.83	4.87	1.04	4.85	0.02	0.99	-0.007	9.7E-01
Mtr.17194.1.S1_at	7.78	6.16	8.10	7.95	8.15	7.92	7.34	1.04	8.01	0.12	1.09	0.125	3.3E-01
Mtr.44814.1.S1_at	8.19	6.82	8.86	8.06	7.84	8.00	7.96	1.04	7.96	0.11	1.00	0.001	9.9E-01
Mtr.31311.1.S1_at	3.00	4.45	2.44	2.43	2.43	2.41	3.30	1.04	2.42	0.01	0.74	-0.444	2.2E-01
Mtr.20701.1.S1_at	6.43	4.35	5.47	5.76	5.79	5.89	5.42	1.04	5.81	0.07	1.07	0.102	5.4E-01
Mtr.11601.1.S1_at	6.03	4.07	5.65	6.41	6.42	6.00	5.25	1.04	6.28	0.24	1.20	0.258	1.7E-01
Mtr.10028.1.S1_at	7.82	6.03	7.83	7.71	8.12	7.62	7.23	1.04	7.82	0.27	1.08	0.113	3.9E-01
Mtr.34625.1.S1_at	2.61	4.39	2.57	2.57	2.54	2.56	3.19	1.04	2.55	0.01	0.80	-0.319	3.5E-01
Mtr.33697.1.S1_at	7.29	6.33	8.41	7.90	7.95	8.08	7.34	1.04	7.98	0.09	1.09	0.120	3.5E-01
Mtr.29286.1.S1_at	6.91	5.83	7.91	7.33	7.41	7.07	6.88	1.04	7.27	0.18	1.06	0.079	5.6E-01
Mtr.9425.1.S1_at	6.38	4.32	5.18	5.97	6.28	5.99	5.29	1.04	6.08	0.18	1.15	0.200	2.7E-01
Mtr.11417.1.S1_at	8.59	6.71	8.41	8.61	8.72	8.59	7.90	1.04	8.64	0.07	1.09	0.128	2.9E-01
Mtr.45322.1.S1_at	7.79	5.92	7.63	7.29	6.97	7.10	7.11	1.04	7.12	0.16	1.00	0.002	9.9E-01
Mtr.42551.1.S1_at	7.03	4.96	6.13	5.79	6.45	5.85	6.04	1.04	6.03	0.37	1.00	-0.002	9.9E-01
Mtr.1068.1.S1_at	2.61	4.40	2.59	2.59	2.59	2.75	3.20	1.04	2.65	0.09	0.83	-0.274	4.1E-01
Mtr.15905.1.S1_at	4.38	4.30	6.14	6.13	6.54	6.08	4.94	1.04	6.25	0.25	1.27	0.340	1.0E-01
Mtr.40320.1.S1_at	10.87	8.85	10.28	10.31	10.56	10.34	10.00	1.04	10.40	0.14	1.04	0.057	5.4E-01
Mtr.38612.1.S1_at	10.19	8.16	9.55	9.89	9.00	9.98	9.30	1.04	9.62	0.54	1.04	0.050	6.6E-01
Mtr.44479.1.S1_at	5.11	3.09	4.55	4.45	4.53	4.52	4.25	1.04	4.50	0.04	1.06	0.083	7.0E-01
Mtr.15245.1.S1_at	3.23	5.01	3.20	3.19	3.14	3.20	3.81	1.04	3.18	0.04	0.83	-0.263	3.5E-01
Mtr.12939.1.S1_at	6.86	5.99	8.06	7.26	7.20	7.41	6.97	1.04	7.29	0.11	1.05	0.065	6.2E-01
Mtr.1941.1.S1_s_at	4.09	5.82	5.94	5.71	5.88	5.01	5.28	1.04	5.53	0.46	1.05	0.066	7.2E-01
Mtr.45994.1.S1_at	5.95	7.69	5.85	6.73	6.25	6.91	6.50	1.04	6.63	0.34	1.02	0.029	8.5E-01
Mtr.16895.1.S1_at	7.37	6.98	5.41	6.95	6.66	7.10	6.59	1.04	6.90	0.22	1.05	0.068	6.3E-01
Mtr.40085.1.S1_s_at	11.58	9.82	9.76	10.58	10.23	10.74	10.39	1.04	10.52	0.26	1.01	0.018	8.4E-01
Mtr.34882.1.S1_s_at	7.64	5.89	7.72	7.46	7.50	7.59	7.08	1.04	7.52	0.07	1.06	0.086	5.1E-01
Mtr.5135.1.S1_at	3.13	3.05	4.88	3.09	3.39	3.09	3.69	1.04	3.19	0.17	0.87	-0.209	4.6E-01
Mtr.13537.1.S1_at	11.02	9.09	10.71	10.83	10.91	10.70	10.27	1.04	10.81	0.10	1.05	0.073	4.2E-01
Mtr.22422.1.S1_at	6.36	4.88	4.37	4.83	5.40	5.30	5.20	1.04	5.18	0.31	0.99	-0.008	9.7E-01
Mtr.41411.1.S1_at	10.47	8.46	9.90	10.00	10.29	9.83	9.61	1.04	10.04	0.23	1.05	0.064	5.2E-01
Mtr.32904.1.S1_s_at	8.66	7.30	9.33	8.47	8.23	7.83	8.43	1.04	8.18	0.32	0.97	-0.044	7.1E-01
Mtr.14550.1.S1_s_at	3.60	5.35	3.52	3.52	3.52	3.43	4.16	1.04	3.49	0.05	0.84	-0.251	3.3E-01
Mtr.2590.1.S1_x_at	4.55	2.71	4.45	4.43	4.41	4.43	3.90	1.04	4.42	0.01	1.13	0.181	4.3E-01
Mtr.22669.1.S1_at	6.65	4.83	6.60	6.68	6.59	6.59	6.03	1.04	6.62	0.05	1.10	0.135	3.8E-01
Mtr.31895.1.S1_at	7.09	5.39	7.27	7.26	7.15	7.12	6.58	1.03	7.18	0.07	1.09	0.125	3.8E-01
Mtr.13407.1.S1_at	4.31	2.34	3.88	3.36	4.35	3.94	3.51	1.03	3.88	0.50	1.11	0.146	6.0E-01
Mtr.41683.1.S1_at	13.25	11.48	13.30	13.50	13.56	13.30	12.68	1.03	13.45	0.13	1.06	0.086	2.7E-01
Mtr.10091.1.S1_at	6.03	4.28	6.11	6.22	6.27	5.54	5.47	1.03	6.01	0.41	1.10	0.134	4.5E-01
Mtr.24446.1.S1_at	6.82	4.91	6.56	7.10	6.91	6.71	6.10	1.03	6.90	0.20	1.13	0.179	2.6E-01
Mtr.6386.1.S1_at	2.25	4.04	2.24	2.29	2.23	2.22	2.84	1.03	2.25	0.04	0.79	-0.339	3.8E-01
Mtr.17599.1.S1_at	5.28	3.45	5.21	5.15	6.12	5.21	4.65	1.03	5.49	0.54	1.18	0.242	2.8E-01
Mtr.35121.1.S1_at	8.56	6.84	8.69	8.38	8.32	8.36	8.03	1.03	8.36	0.03	1.04	0.058	6.1E-01
Mtr.10630.1.S1_at	5.11	3.26	4.98	5.01	6.05	4.97	4.45	1.03	5.35	0.61	1.20	0.265	2.7E-01
Mtr.33049.1.S1_at	10.15	8.33	8.38	9.69	9.45	9.87	8.96	1.03	9.67	0.21	1.08	0.110	3.1E-01
Mtr.37646.1.S1_at	4.55	2.51	3.82	4.05	3.74	4.37	3.62	1.03	4.05	0.31	1.12	0.162	5.3E-01
Mtr.18430.1.S1_at	6.30	5.17	7.23	7.22	7.33	6.94	6.23	1.03	7.16	0.20	1.15	0.201	2.0E-01
Mtr.35838.1.S1_at	3.81	2.02	3.80	4.08	5.29	6.22	3.21	1.03	5.20	1.07	1.62	0.696	8.2E-02
Mtr.30467.1.S1_at	9.01	7.15	8.86	8.78	8.83	8.82	8.34	1.03	8.81	0.03	1.06	0.079	4.7E-01
Mtr.42473.1.S1_at	5.97	4.01	5.54	6.10	5.97	6.00	5.17	1.03	6.03	0.07	1.16	0.220	2.3E-01
Mtr.51168.1.S1_x_at	2.94	4.70	2.88	2.86	2.81	2.80	3.51	1.03	2.82	0.03	0.81	-0.313	3.2E-01
Mtr.22161.1.S1_at	3.09	4.31	2.26	6.18	6.16	4.93	3.22	1.03	5.76	0.71	1.79	0.837	2.5E-02
Mtr.14224.1.S1_at	10.13	8.30	10.04	10.63	9.82	10.56	9.49	1.03	10.34	0.45	1.09	0.123	2.6E-01
Mtr.42210.1.S1_s_at	8.93	7.02	8.66	8.70	8.85	8.73	8.20	1.03	8.76	0.08	1.07	0.095	4.0E-01
Mtr.29533.1.S1_at	9.63	8.25	10.27	10.10	10.04	9.92	9.38	1.03	10.02	0.09	1.07	0.095	3.5E-01
Mtr.24080.1.S1_at	2.41	4.19	2.40	2.39	2.39	2.39	3.00	1.03	2.39	0.00	0.80	-0.326	3.7E-01
Mtr.35584.1.S1_at	6.61	5.47	7.53	7.03	6.60	6.75	6.53	1.03	6.79	0.22	1.04	0.056	6.9E-01
Mtr.9275.1.S1_s_at	8.00	6.02	7.51	7.72	7.97	7.84	7.17	1.03	7.84	0.13	1.09	0.128	3.3E-01
Mtr.2960.1.S1_at	8.44	6.38	7.38	8.12	7.71	8.49	7.40	1.03	8.11	0.39	1.10	0.132	3.3E-01
Mtr.42032.1.S1_at	6.48	4.96	6.93	6.75	6.58	5.81	6.12	1.03	6.38	0.50	1.04	0.059	7.2E-01
Mtr.30682.1.S1_at	11.91	10.42	12.40	11.84	11.49	11.71	11.58	1.03	11.68	0.18	1.01	0.013	8.7E-01
Mtr.5438.1.S1_at	7.95	9.71	7.91	7.87	7.85	8.12	8.52	1.03	7.94	0.15	0.93	-0.102	3.9E-01
Mtr.30369.1.S1_at	5.98	4.14	5.87	5.32	5.52	5.34	5.33	1.03	5.39	0.11	1.01	0.017	9.2E-01
Mtr.32750.1.S1_at	9.99	8.48	8.03	8.57	8.89	8.59	8.83	1.03	8.68	0.18	0.98	-0.025	8.1E-01
Mtr.50743.1.S1_at	8.32	6.57	8.38	7.73	7.87	7.57	7.76	1.03	7.72	0.15	1.00	-0.006	9.6E-01
Mtr.6711.1.S1_s_at	9.70	7.78	8.10	9.28	9.96	9.63	8.53	1.03	9.62	0.34	1.13	0.175	1.5E-01
Mtr.29367.1.S1_at	8.69	6.66	8.02	8.43	8.12	8.53	7.79	1.03	8.36	0.22	1.07	0.102	4.0E-01
Mtr.16247.1.S1_at	2.82	4.48	2.60	2.98	2.80	2.80	3.30	1.03	2.86	0.10	0.87	-0.208	5.0E-01
Mtr.26247.1.S1_at	2.67	4.45	2.65	2.66	2.66	2.64	3.26	1.03	2.65	0.01	0.81	-0.296	3.7E-01
Mtr.9660.1.S1_at	6.23	5.01	7.06	6.91	6.44	6.83	6.10	1.03	6.73	0.25	1.10	0.141	3.7E-01
Mtr.44736.1.S1_at	6.23	4.44	6.21	5.87	6.52	6.20	5.63	1.03	6.20	0.32	1.10	0.139	4.1E-01
Mtr.3851.1.S1_at	3.59	1.54	2.71	2.61	3.04	2.66	2.61	1.03	2.77	0.24	1.06	0.086	8.0E-01
Mtr.32155.1.S1_at	7.29	5.25	6.51	5.03	5.27	5.33	6.35	1.03	5.21	0.16	0.82	-0.285	1.3E-01
Mtr.35065.1.S1_at	5.79	3.93	5.64	5.33	5.46	5.43	5.12	1.03	5.40	0.07	1.06	0.078	6.6E-01
Mtr.32183.1.S1_at	7.46	5.71	7.53	7.41	7.32	7.57	6.90	1.03	7.44	0.13	1.08	0.108	4.2E-01
Mtr.10820.1.S1_at	7.76	5.75	7.14	7.03	6.60	6.77	6.88	1.03	6.80	0.22	0.99	-0.018	9.0E-01
Mtr.8715.1.S1_at	9.50	7.61	9.25	9.07	8.87	8.92	8.79	1.03	8.96	0.10	1.02	0.028	7.9E-01
Mtr.41814.1.S1_at	8.73	6.84	8.49	8.15	8.20	8.22	8.02	1.03	8.19	0.04	1.02	0.030	7.9E-01
Mtr.39209.1.S1_at	8.64	6.69	8.23	8.08	7.96	8.04	7.85	1.03	8.02	0.06	1.02	0.031	7.9E-01
Mtr.41114.1.S1_at	10.53	8.78	10.59	11.02	11.31	10.91	9.97	1.03	11.08	0.21	1.11	0.153	1.4E-01
Mtr.2933.1.S1_at	4.98	2.99	4.42	4.85	4.98	4.40	4.13	1.03	4.74	0.30	1.15	0.200	3.8E-01
Mtr.44087.1.S1_at	3.78	3.79	5.57	5.29	5.15	4.90	4.38	1.03	5.11	0.20	1.17	0.222	2.9E-01
Mtr.1545.1.S1_at	5.07	4.20	6.25	6.68	7.09	6.54	5.17	1.03	6.77	0.28	1.31	0.388	6.1E-02
Mtr.13207.1.S1_at	8.53	6.99	8.94	8.14	8.44	8.15	8.15	1.03	8.25	0.17	1.01	0.016	8.9E-01
Mtr.15731.1.S1_at	2.87	4.64	2.86	2.84	2.75	2.88	3.46	1.03	2.82	0.07	0.82	-0.293	3.5E-01
Mtr.30944.1.S1_at	2.46	4.23	2.44	2.63	2.42	2.41	3.04	1.03	2.49	0.13	0.82	-0.292	4.0E-01
Mtr.32520.1.S1_at	6.66	4.73	6.29	6.35	6.62	6.04	5.89	1.03	6.34	0.29	1.08	0.105	5.1E-01
Mtr.31633.1.S1_at	8.38	6.78	8.69	8.21	8.15	8.04	7.95	1.03	8.14	0.09	1.02	0.034	7.7E-01
Mtr.33750.1.S1_at	8.48	6.47	7.84	8.01	7.80	8.02	7.60	1.03	7.94	0.13	1.05	0.064	5.9E-01
Mtr.42611.1.S1_at	5.65	4.04	5.95	5.09	5.10	5.28	5.21	1.03	5.15	0.11	0.99	-0.016	9.3E-01
Mtr.41169.1.S1_at	8.42	7.08	9.10	8.35	8.74	8.35	8.20	1.03	8.48	0.23	1.03	0.049	6.6E-01
Mtr.42351.1.S1_at	7.87	5.97	7.60	7.82	7.78	7.69	7.14	1.03	7.76	0.07	1.09	0.120	3.6E-01
Mtr.22609.1.S1_x_at	2.28	4.04	2.26	2.25	2.25	2.25	2.86	1.03	2.25	0.00	0.79	-0.344	3.6E-01
Mtr.11034.1.S1_at	2.39	4.16	2.38	2.38	2.38	2.38	2.97	1.03	2.38	0.00	0.80	-0.323	3.7E-01
Mtr.19239.1.S1_at	2.71	4.48	2.70	2.70	2.68	2.69	3.30	1.03	2.69	0.01	0.82	-0.294	3.6E-01
Mtr.24815.1.S1_at	8.02	6.14	6.36	7.15	6.93	6.99	6.84	1.02	7.02	0.11	1.03	0.038	7.7E-01
Mtr.50278.1.S1_s_at	10.37	8.93	10.91	10.74	10.59	10.46	10.07	1.02	10.60	0.14	1.05	0.074	4.3E-01
Mtr.42652.1.S1_at	7.61	5.79	7.53	7.09	7.22	7.27	6.98	1.02	7.19	0.09	1.03	0.044	7.3E-01
Mtr.22611.1.S1_at	2.90	4.65	2.85	2.83	2.81	2.78	3.47	1.02	2.81	0.02	0.81	-0.304	3.3E-01
Mtr.32521.1.S1_at	5.14	3.55	5.47	5.05	4.76	4.90	4.72	1.02	4.90	0.15	1.04	0.054	7.8E-01
Mtr.41991.1.S1_s_at	10.11	8.78	10.79	10.70	10.79	10.73	9.89	1.02	10.74	0.05	1.09	0.118	2.3E-01
Mtr.33590.1.S1_at	2.95	4.72	2.94	2.94	2.94	2.89	3.53	1.02	2.92	0.03	0.83	-0.274	3.6E-01
Mtr.1927.1.S1_at	9.44	7.52	9.09	9.00	8.85	8.95	8.68	1.02	8.93	0.08	1.03	0.041	7.0E-01
Mtr.90.1.S1_at	7.28	6.00	8.03	8.00	7.75	7.36	7.10	1.02	7.70	0.32	1.08	0.117	3.9E-01
Mtr.2872.1.S1_at	8.27	6.48	8.24	8.25	8.07	8.30	7.66	1.02	8.20	0.12	1.07	0.098	4.1E-01
Mtr.11993.1.S1_at	6.47	5.91	7.90	6.14	5.91	6.27	6.76	1.02	6.10	0.18	0.90	-0.147	3.4E-01
Mtr.27919.1.S1_at	8.06	6.05	7.40	7.18	7.68	7.50	7.17	1.02	7.45	0.25	1.04	0.055	6.7E-01
Mtr.24411.1.S1_at	8.91	6.94	8.40	8.47	8.52	8.48	8.08	1.02	8.49	0.03	1.05	0.070	5.3E-01
Mtr.27955.1.S1_s_at	7.45	6.16	8.18	7.64	7.58	7.73	7.26	1.02	7.65	0.08	1.05	0.075	5.5E-01
Mtr.2811.1.S1_at	11.11	9.10	9.76	10.57	11.64	10.67	9.99	1.02	10.96	0.59	1.10	0.134	2.3E-01
Mtr.10993.1.S1_at	6.05	4.65	6.64	7.60	7.55	7.73	5.78	1.02	7.63	0.09	1.32	0.399	3.6E-02
Mtr.28496.1.S1_at	5.21	7.22	6.58	8.69	8.93	8.69	6.34	1.02	8.77	0.14	1.38	0.469	1.5E-02
Mtr.30724.1.S1_at	9.58	7.66	9.24	9.10	9.19	9.13	8.83	1.02	9.14	0.05	1.04	0.051	6.2E-01
Mtr.17472.1.S1_at	6.72	5.16	7.08	6.90	7.05	7.13	6.32	1.02	7.03	0.12	1.11	0.153	3.0E-01
Mtr.28448.1.S1_s_at	9.05	7.14	8.75	8.90	9.00	9.15	8.31	1.02	9.02	0.13	1.08	0.117	3.0E-01
Mtr.45018.1.S1_at	8.81	7.17	9.05	8.88	8.80	8.87	8.34	1.02	8.85	0.05	1.06	0.085	4.4E-01
Mtr.48288.1.S1_s_at	9.26	7.92	9.92	8.99	9.03	8.95	9.03	1.02	8.99	0.04	1.00	-0.007	9.4E-01
Mtr.45331.1.S1_at	8.89	7.85	9.89	9.51	10.07	9.88	8.88	1.02	9.82	0.29	1.11	0.146	2.0E-01
Mtr.8229.1.S1_at	8.50	9.43	7.39	8.24	8.24	8.19	8.44	1.02	8.22	0.03	0.97	-0.038	7.3E-01
Mtr.31507.1.S1_at	2.10	3.86	2.09	2.08	2.08	2.09	2.69	1.02	2.08	0.00	0.77	-0.368	3.6E-01
Mtr.33405.1.S1_at	5.36	3.34	4.13	4.08	3.91	3.84	4.28	1.02	3.95	0.12	0.92	-0.116	6.1E-01
Mtr.42729.1.S1_at	9.48	7.45	8.66	9.11	8.91	8.94	8.53	1.02	8.99	0.11	1.05	0.075	4.9E-01
Mtr.11109.1.S1_at	7.74	6.05	7.88	9.56	9.96	9.94	7.22	1.02	9.82	0.23	1.36	0.443	1.3E-02
Mtr.36786.1.S1_at	4.54	6.36	4.65	4.17	5.07	4.89	5.18	1.02	4.71	0.48	0.91	-0.138	5.1E-01
Mtr.41045.1.S1_at	7.14	5.40	7.19	7.07	7.51	7.21	6.58	1.02	7.26	0.23	1.10	0.143	3.2E-01
Mtr.34186.1.S1_at	5.84	5.75	4.03	5.41	5.05	4.88	5.21	1.02	5.11	0.27	0.98	-0.028	8.8E-01
Mtr.3132.1.S1_at	6.18	4.43	6.20	6.42	6.53	6.15	5.60	1.02	6.37	0.20	1.14	0.184	2.7E-01
Mtr.6883.1.S1_at	4.57	2.80	4.56	4.55	4.64	4.24	3.98	1.02	4.48	0.21	1.13	0.172	4.5E-01
Mtr.25883.1.S1_at	5.34	7.29	5.80	5.75	5.54	5.52	6.14	1.02	5.60	0.13	0.91	-0.133	4.1E-01

Mtr.28274.1.S1_at	9.08	7.17	7.52	8.16	7.60	8.37	7.92	1.02	8.04	0.40	1.02	0.022	8.6E-01
Mtr.27591.1.S1_at	6.68	4.68	6.03	7.60	7.64	7.88	5.80	1.02	7.71	0.15	1.33	0.411	3.2E-02
Mtr.35922.1.S1_at	8.26	6.23	7.39	7.49	8.02	7.69	7.30	1.02	7.74	0.27	1.06	0.085	5.1E-01
Mtr.36855.1.S1_at	4.99	2.97	3.76	3.95	4.92	3.75	3.91	1.02	4.21	0.62	1.08	0.106	6.9E-01
Mtr.26281.1.S1_at	5.49	7.11	5.23	5.49	5.61	5.52	5.94	1.02	5.54	0.06	0.93	-0.102	5.3E-01
Mtr.42944.1.S1_a_at	4.44	5.55	3.52	5.64	5.54	5.83	4.50	1.02	5.67	0.15	1.26	0.332	1.2E-01
Mtr.14940.1.S1_at	2.75	4.49	2.71	2.72	2.72	2.72	3.32	1.02	2.72	0.00	0.82	-0.286	3.7E-01
Mtr.33284.1.S1_at	5.61	3.80	5.52	5.38	5.52	5.71	4.98	1.02	5.54	0.16	1.11	0.154	4.0E-01
Mtr.8500.1.S1_at	9.79	8.77	7.75	8.46	8.29	8.65	8.77	1.02	8.47	0.18	0.97	-0.051	6.4E-01
Mtr.45282.1.S1_at	7.33	5.68	7.54	7.25	7.29	7.39	6.85	1.02	7.31	0.07	1.07	0.094	4.8E-01
Mtr.11953.1.S1_s_at	2.30	4.05	2.29	2.28	2.28	2.28	2.88	1.02	2.28	0.00	0.79	-0.336	3.7E-01
Mtr.31728.1.S1_at	7.31	5.62	7.45	6.99	7.21	7.11	6.80	1.02	7.10	0.11	1.05	0.064	6.3E-01
Mtr.3006.1.S1_at	7.19	5.30	6.91	7.70	7.72	7.78	6.47	1.02	7.73	0.04	1.20	0.258	9.8E-02
Mtr.32717.1.S1_at	8.88	6.91	7.47	8.12	8.38	8.50	7.75	1.02	8.33	0.19	1.07	0.104	3.9E-01
Mtr.11355.1.S1_at	6.74	5.72	7.75	6.96	7.15	6.41	6.74	1.02	6.84	0.38	1.01	0.021	8.8E-01
Mtr.8468.1.S1_at	6.20	4.40	6.11	5.47	6.22	5.92	5.57	1.02	5.87	0.38	1.05	0.075	6.6E-01
Mtr.34416.1.S1_at	4.20	2.38	2.51	2.83	4.14	3.19	3.03	1.02	3.39	0.68	1.12	0.160	6.4E-01
Mtr.51560.1.S1_at	8.54	6.75	8.49	7.70	7.95	7.82	7.93	1.02	7.83	0.13	0.99	-0.018	8.7E-01
Mtr.11717.1.S1_at	5.33	3.33	4.61	5.48	5.62	5.81	4.42	1.02	5.64	0.17	1.27	0.350	1.1E-01
Mtr.51302.1.S1_at	8.07	6.04	7.08	6.26	6.63	6.51	7.06	1.02	6.47	0.19	0.92	-0.128	3.7E-01
Mtr.7096.1.S1_at	6.05	4.18	5.80	5.88	6.54	5.76	5.34	1.02	6.06	0.42	1.13	0.181	3.2E-01
Mtr.36249.1.S1_at	9.21	10.98	9.24	9.05	9.40	9.15	9.81	1.02	9.20	0.18	0.94	-0.093	3.6E-01
Mtr.42753.1.S1_at	5.96	4.41	6.31	6.05	6.16	6.17	5.56	1.02	6.13	0.06	1.10	0.140	3.9E-01
Mtr.37097.1.S1_at	8.06	6.08	7.49	7.13	7.25	7.54	7.21	1.02	7.31	0.21	1.01	0.019	8.8E-01
Mtr.47283.1.S1_at	3.62	2.40	4.41	3.58	3.83	3.48	3.48	1.02	3.63	0.18	1.04	0.063	8.1E-01
Mtr.48257.1.S1_at	7.04	5.14	6.70	6.88	6.61	6.68	6.29	1.02	6.72	0.14	1.07	0.096	5.1E-01
Mtr.5668.1.S1_at	7.73	6.33	8.31	7.28	7.14	6.80	7.46	1.02	7.07	0.25	0.95	-0.076	5.6E-01
Mtr.32839.1.S1_at	6.73	4.88	6.53	6.95	7.03	7.18	6.05	1.01	7.05	0.12	1.17	0.222	1.6E-01
Mtr.11161.1.S1_at	10.53	9.04	10.97	10.76	10.49	10.76	10.18	1.01	10.67	0.16	1.05	0.068	4.6E-01
Mtr.27491.1.S1_at	4.10	2.38	4.16	4.06	4.09	4.17	3.55	1.01	4.10	0.06	1.16	0.211	3.9E-01
Mtr.5795.1.S1_at	6.83	4.80	5.82	6.36	6.49	6.73	5.82	1.01	6.53	0.19	1.12	0.166	3.0E-01
Mtr.41974.1.S1_at	8.05	6.07	7.46	7.57	7.44	7.69	7.19	1.01	7.57	0.12	1.05	0.073	5.6E-01
Mtr.41867.1.S1_at	6.38	5.21	7.23	6.54	6.82	6.35	6.28	1.01	6.57	0.24	1.05	0.066	6.5E-01
Mtr.18060.1.S1_at	8.04	6.07	7.46	7.89	7.96	7.60	7.19	1.01	7.82	0.19	1.09	0.120	3.5E-01
Mtr.31504.1.S1_s_at	6.64	5.97	7.96	6.81	6.77	6.81	6.85	1.01	6.80	0.02	0.99	-0.012	9.3E-01
Mtr.33286.1.S1_at	7.38	5.38	6.65	6.41	6.41	7.09	6.47	1.01	6.64	0.39	1.03	0.037	8.0E-01
Mtr.22523.1.S1_x_at	3.30	5.05	3.28	3.13	3.09	3.45	3.88	1.01	3.22	0.20	0.83	-0.266	3.4E-01
Mtr.42385.1.S1_s_at	6.22	4.39	6.05	6.48	6.24	6.66	5.55	1.01	6.46	0.21	1.16	0.218	2.0E-01
Mtr.45422.1.S1_at	6.84	4.91	6.40	6.49	6.65	6.46	6.05	1.01	6.54	0.10	1.08	0.111	4.6E-01
Mtr.42181.1.S1_at	7.00	5.15	6.78	6.08	7.47	6.70	6.31	1.01	6.75	0.70	1.07	0.098	5.7E-01
Mtr.13973.1.S1_at	4.18	3.17	5.20	3.87	4.45	3.93	4.19	1.01	4.08	0.32	0.98	-0.036	8.8E-01
Mtr.18908.1.S1_at	4.25	2.50	4.26	4.33	4.21	3.68	3.67	1.01	4.07	0.35	1.11	0.151	5.5E-01
Mtr.12296.1.S1_at	5.83	4.07	5.82	5.85	5.79	6.14	5.24	1.01	5.93	0.19	1.13	0.179	3.1E-01
Mtr.24351.1.S1_s_at	7.58	5.57	6.78	7.45	7.97	7.58	6.64	1.01	7.67	0.27	1.15	0.207	1.7E-01
Mtr.28002.1.S1_at	6.69	4.67	5.79	6.28	5.41	6.27	5.71	1.01	5.99	0.50	1.05	0.067	7.0E-01
Mtr.6088.1.S1_at	7.23	5.41	7.08	7.08	7.20	7.16	6.57	1.01	7.15	0.06	1.09	0.121	3.8E-01
Mtr.6912.1.S1_at	6.38	4.41	5.80	5.95	5.95	5.94	5.53	1.01	5.95	0.00	1.07	0.104	5.2E-01
Mtr.3153.1.S1_at	8.04	6.04	7.33	6.85	7.27	7.28	7.14	1.01	7.13	0.24	1.00	-0.001	9.9E-01
Mtr.29791.1.S1_at	2.90	4.64	2.87	2.79	2.85	2.88	3.47	1.01	2.84	0.05	0.82	-0.288	3.4E-01
Mtr.9175.1.S1_at	7.70	5.71	7.04	7.44	6.84	7.49	6.81	1.01	7.26	0.36	1.06	0.091	5.2E-01
Mtr.35617.1.S1_at	4.87	3.47	5.43	4.77	4.85	4.73	4.59	1.01	4.78	0.06	1.04	0.058	7.6E-01
Mtr.5121.1.S1_at	7.93	5.90	6.96	7.97	8.26	8.15	6.93	1.01	8.13	0.14	1.17	0.231	1.1E-01
Mtr.16942.1.S1_at	9.33	7.40	8.90	8.96	8.86	9.02	8.54	1.01	8.95	0.08	1.05	0.067	5.2E-01
Mtr.31299.1.S1_at	4.42	2.73	2.62	2.74	2.63	2.68	3.26	1.01	2.68	0.05	0.82	-0.279	3.8E-01
Mtr.13957.1.S1_s_at	8.38	6.73	8.57	8.00	8.10	8.12	7.89	1.01	8.07	0.07	1.02	0.032	7.8E-01
Mtr.34124.1.S1_at	7.35	5.57	7.30	7.28	7.50	7.40	6.74	1.01	7.39	0.11	1.10	0.133	3.3E-01
Mtr.28450.1.S1_at	6.89	4.97	6.47	6.86	6.88	7.21	6.11	1.01	6.98	0.20	1.14	0.192	2.2E-01
Mtr.51977.1.S1_at	2.79	4.53	2.77	2.77	2.75	2.75	3.36	1.01	2.76	0.01	0.82	-0.287	3.6E-01
Mtr.10190.1.S1_at	6.44	5.08	7.05	6.62	6.45	6.57	6.19	1.01	6.55	0.09	1.06	0.081	5.8E-01
Mtr.8878.1.S1_at	9.87	8.29	10.17	9.48	9.35	9.38	9.44	1.01	9.40	0.07	1.00	-0.006	9.5E-01
Mtr.4.1.S1_at	3.27	5.08	3.38	3.46	3.30	3.33	3.91	1.01	3.36	0.09	0.86	-0.217	4.0E-01
Mtr.15935.1.S1_at	5.77	3.91	5.54	5.77	5.71	5.58	5.07	1.01	5.69	0.09	1.12	0.165	3.5E-01
Mtr.27178.1.S1_s_at	7.97	6.73	8.73	7.36	8.59	8.12	7.81	1.01	8.02	0.62	1.03	0.039	7.7E-01
Mtr.43090.1.S1_at	7.87	5.88	7.20	7.21	6.97	7.40	6.98	1.01	7.19	0.22	1.03	0.042	7.5E-01
Mtr.41708.1.S1_at	10.00	7.99	8.82	8.97	8.98	9.38	8.93	1.01	9.11	0.23	1.02	0.028	7.8E-01
Mtr.21918.1.S1_at	2.19	3.94	2.18	2.18	2.18	2.17	2.77	1.01	2.18	0.00	0.79	-0.348	3.7E-01
Mtr.9837.1.S1_at	9.70	7.73	9.11	8.99	9.13	9.27	8.84	1.01	9.13	0.14	1.03	0.046	6.5E-01
Mtr.46384.1.S1_at	2.78	4.50	2.74	2.72	2.66	2.94	3.34	1.01	2.77	0.15	0.83	-0.268	3.9E-01
Mtr.9195.1.S1_at	11.37	9.48	9.83	10.46	10.40	10.59	10.23	1.01	10.49	0.10	1.03	0.036	6.8E-01
Mtr.28372.1.S1_at	6.04	4.52	6.43	6.15	6.02	6.03	5.66	1.01	6.07	0.07	1.07	0.099	5.3E-01
Mtr.18307.1.S1_at	6.48	4.77	6.56	6.18	6.37	6.40	5.94	1.01	6.32	0.12	1.06	0.089	5.6E-01
Mtr.5796.1.S1_s_at	5.56	3.67	5.24	5.54	5.36	5.57	4.82	1.01	5.49	0.12	1.14	0.187	3.2E-01
Mtr.1712.1.S1_at	5.19	4.14	6.16	5.52	6.11	5.15	5.16	1.01	5.59	0.48	1.08	0.115	5.4E-01
Mtr.40987.1.S1_at	8.01	6.89	8.90	7.94	8.00	7.25	7.93	1.01	7.73	0.42	0.97	-0.038	7.6E-01
Mtr.24469.1.S1_at	2.37	4.11	2.36	2.34	2.35	2.35	2.95	1.01	2.35	0.01	0.80	-0.327	3.6E-01
Mtr.5485.1.S1_s_at	7.61	6.18	8.13	7.86	8.04	7.72	7.31	1.01	7.87	0.16	1.08	0.107	3.9E-01
Mtr.22332.1.S1_at	9.82	8.04	8.11	9.21	8.78	9.11	8.66	1.01	9.04	0.22	1.04	0.062	5.6E-01
Mtr.16703.1.S1_at	7.07	5.14	6.63	7.05	7.38	6.86	6.28	1.01	7.10	0.26	1.13	0.176	2.5E-01
Mtr.28278.1.S1_at	5.89	4.25	6.08	5.15	5.90	5.45	5.41	1.01	5.50	0.38	1.02	0.025	8.9E-01
Mtr.31521.1.S1_at	7.20	5.21	6.47	6.59	6.57	6.88	6.29	1.01	6.68	0.17	1.06	0.086	5.5E-01
Mtr.4718.1.S1_s_at	7.78	6.27	8.17	8.04	7.94	8.06	7.41	1.01	8.01	0.07	1.08	0.113	3.6E-01
Mtr.34218.1.S1_s_at	8.36	6.35	7.45	7.86	7.72	7.96	7.38	1.01	7.85	0.12	1.06	0.088	4.7E-01
Mtr.43914.1.S1_at	11.97	9.96	11.14	11.53	10.91	11.67	11.02	1.01	11.37	0.41	1.03	0.045	6.1E-01
Mtr.28608.1.S1_at	4.69	6.63	5.19	4.79	4.98	4.72	5.50	1.01	4.83	0.14	0.88	-0.189	3.1E-01
Mtr.28500.1.S1_at	5.55	3.59	4.17	4.10	4.04	4.22	4.44	1.01	4.12	0.09	0.93	-0.108	6.1E-01
Mtr.10393.1.S1_at	13.91	12.10	13.77	13.59	13.15	13.47	13.26	1.01	13.40	0.23	1.01	0.016	8.2E-01
Mtr.44161.1.S1_at	6.73	5.06	6.87	6.56	6.40	6.61	6.22	1.01	6.52	0.11	1.05	0.068	6.3E-01
Mtr.30085.1.S1_at	4.68	2.91	4.62	4.94	5.11	4.67	4.07	1.01	4.91	0.22	1.21	0.270	2.3E-01
Mtr.29236.1.S1_at	6.67	5.32	7.29	6.99	6.94	6.81	6.42	1.01	6.91	0.09	1.08	0.106	4.5E-01
Mtr.12197.1.S1_at	7.58	5.83	7.57	7.74	7.85	8.05	6.99	1.01	7.88	0.15	1.13	0.172	2.1E-01
Mtr.8127.1.S1_at	2.47	4.19	2.44	2.43	2.43	2.42	3.03	1.01	2.43	0.00	0.80	-0.320	3.6E-01
Mtr.32507.1.S1_at	4.77	4.50	6.36	6.04	6.39	6.45	5.21	1.00	6.29	0.22	1.21	0.273	1.4E-01
Mtr.20601.1.S1_at	2.09	3.83	2.09	2.08	2.08	2.08	2.67	1.00	2.08	0.00	0.78	-0.359	3.7E-01
Mtr.32815.1.S1_at	5.87	4.59	6.57	5.63	6.03	5.88	5.67	1.00	5.85	0.20	1.03	0.043	7.9E-01
Mtr.18764.1.S1_s_at	5.14	3.38	5.11	4.84	4.77	4.51	4.54	1.00	4.71	0.17	1.04	0.052	7.9E-01
Mtr.32827.1.S1_at	7.31	5.54	7.25	7.23	7.42	7.11	6.70	1.00	7.25	0.16	1.08	0.114	4.0E-01
Mtr.37125.1.S1_at	6.95	5.28	7.07	6.87	7.03	6.88	6.44	1.00	6.93	0.09	1.08	0.106	4.5E-01
Mtr.8212.1.S1_at	7.44	5.43	6.57	6.76	6.79	6.53	6.48	1.00	6.69	0.14	1.03	0.046	7.4E-01
Mtr.45766.1.S1_at	5.50	3.55	4.94	4.35	4.93	4.26	4.67	1.00	4.52	0.36	0.97	-0.047	8.2E-01
Mtr.16097.1.S1_at	2.28	4.01	2.27	2.26	2.26	2.27	2.85	1.00	2.26	0.00	0.79	-0.334	3.7E-01
Mtr.45719.1.S1_at	5.67	4.78	6.79	5.95	6.46	5.80	5.74	1.00	6.07	0.34	1.06	0.079	6.2E-01
Mtr.37716.1.S1_at	11.77	9.87	11.40	11.87	11.82	11.84	11.01	1.00	11.84	0.03	1.08	0.105	2.3E-01
Mtr.40734.1.S1_at	10.13	8.94	10.94	9.55	9.75	9.54	10.00	1.00	9.61	0.12	0.96	-0.057	5.4E-01
Mtr.37312.1.S1_at	13.82	12.75	11.81	13.24	13.00	13.31	12.79	1.00	13.18	0.16	1.03	0.043	5.4E-01
Mtr.39289.1.S1_at	6.76	4.77	5.97	5.04	5.02	4.82	5.83	1.00	4.96	0.12	0.85	-0.234	2.1E-01
Mtr.32790.1.S1_s_at	6.30	5.04	7.02	6.70	8.48	7.93	6.12	1.00	7.70	0.91	1.26	0.333	1.1E-01
Mtr.32808.1.S1_at	3.69	3.17	5.11	4.46	5.72	4.63	3.99	1.00	4.93	0.69	1.24	0.306	2.5E-01
Mtr.9534.1.S1_at	8.07	6.21	7.79	7.93	7.81	7.84	7.35	1.00	7.86	0.06	1.07	0.095	4.3E-01
Mtr.24419.1.S1_at	3.08	4.75	2.96	3.09	3.06	3.22	3.60	1.00	3.12	0.08	0.87	-0.203	4.6E-01
Mtr.7039.1.S1_s_at	6.87	5.33	7.21	6.98	7.08	6.91	6.47	1.00	6.99	0.09	1.08	0.111	4.2E-01
Mtr.848.1.S1_s_at	10.91	8.98	10.39	10.14	9.88	9.89	10.09	1.00	9.97	0.15	0.99	-0.018	8.4E-01
Mtr.34978.1.S1_at	6.39	4.73	6.53	6.31	6.49	6.19	5.88	1.00	6.33	0.15	1.08	0.105	4.9E-01
Mtr.35338.1.S1_s_at	4.22	2.48	4.21	4.35	4.19	4.39	3.64	1.00	4.31	0.11	1.18	0.244	3.1E-01
Mtr.7172.1.S1_at	8.00	6.24	7.95	7.70	7.65	7.84	7.40	1.00	7.73	0.10	1.04	0.063	6.0E-01
Mtr.46876.1.S1_at	3.49	3.19	5.06	3.78	5.82	3.27	3.91	1.00	4.29	1.35	1.10	0.133	7.2E-01
Mtr.36929.1.S1_s_at	8.88	6.99	8.52	8.35	8.29	8.31	8.13	1.00	8.32	0.03	1.02	0.033	7.6E-01
Mtr.44805.1.S1_at	7.82	7.02	9.01	7.63	8.00	7.26	7.95	1.00	7.63	0.37	0.96	-0.059	6.3E-01
Mtr.11021.1.S1_at	11.43	9.43	10.27	10.34	10.79	10.50	10.38	1.00	10.54	0.23	1.02	0.023	7.9E-01
Mtr.30046.1.S1_at	5.98	3.97	4.99	5.16	5.24	5.21	4.98	1.00	5.20	0.04	1.05	0.064	7.2E-01
Mtr.18813.1.S1_at	10.84	12.63	10.96	11.85	12.58	12.25	11.48	1.00	12.23	0.37	1.07	0.092	2.9E-01
Mtr.4965.1.S1_at	6.21	4.48	6.21	6.45	6.92	6.61	5.63	1.00	6.66	0.24	1.18	0.242	1.6E-01
Mtr.2042.1.S1_at	2.33	4.06	2.34	2.33	2.33	2.33	2.91	1.00	2.33	0.00	0.80	-0.320	3.7E-01
Mtr.14528.1.S1_at	6.72	5.70	7.70	7.17	7.30	7.07	6.71	1.00	7.18	0.12	1.07	0.098	4.6E-01
Mtr.35210.1.S1_at	2.68	4.41	2.67	2.67	2.67	2.61	3.26	1.00	2.65	0.03	0.81	-0.298	3.5E-01
Mtr.51747.1.S1_at	7.70	6.46	8.44	7.55	7.75	7.63	7.53	1.00	7.65	0.10	1.02	0.022	8.5E-01
Mtr.39741.1.S1_s_at	5.87	4.25	6.07	5.23	5.66	5.28	5.40	1.00	5.39	0.23	1.00	-0.002	9.9E-01
Mtr.35314.1.S1_s_at	7.98	6.02	7.34	8.12	8.19	8.04	7.11	1.00	8.12	0.08	1.14	0.190	1.6E-01
Mtr.15779.1.S1_at	7.68	5.99	7.75	7.61	7.44	7.05	7.14	1.00	7.37	0.29	1.03	0.045	7.2E-01
Mtr.18240.1.S1_at	10.74	8.76	9.99	9.94	9.64	9.85	9.83	1.00	9.81	0.15	1.00	-0.003	9.7E-01
Mtr.15812.1.S1_at	11.28	9.45	11.05	11.78	12.03	11.87	10.59	1.00	11.89	0.13	1.12	0.167	8.9E-02
Mtr.14403.1.S1_at	3.38	5.08	3.32	3.33	3.24	3.29	3.93	1.00	3.29	0.04	0.84	-0.257	3.3E-01

Mtr.2565.1.S1_at	10.76	12.46	10.70	11.02	10.41	10.61	11.31	1.00	10.68	0.31	0.94	-0.082	3.6E-01
Mtr.9699.1.S1_at	9.52	7.53	8.65	8.64	8.72	8.90	8.57	1.00	8.75	0.13	1.02	0.031	7.7E-01
Mtr.47086.1.S1_s_at	8.51	6.51	7.59	7.95	7.70	7.94	7.54	1.00	7.86	0.14	1.04	0.061	6.1E-01
Mtr.9845.1.S1_at	7.44	5.69	7.40	7.35	7.57	7.37	6.85	1.00	7.43	0.12	1.09	0.119	3.7E-01
Mtr.13205.1.S1_s_at	8.19	6.44	8.15	8.15	8.09	8.27	7.59	1.00	8.17	0.09	1.08	0.106	3.7E-01
Mtr.5664.1.S1_at	5.63	4.11	5.99	6.29	6.07	5.72	5.24	1.00	6.03	0.29	1.15	0.202	2.6E-01
Mtr.51700.1.S1_s_at	7.69	6.17	8.05	7.89	7.87	7.66	7.30	1.00	7.81	0.13	1.07	0.096	4.3E-01
Mtr.23272.1.S1_at	5.46	3.48	4.25	5.20	5.10	5.20	4.40	1.00	5.17	0.06	1.18	0.233	2.5E-01
Mtr.41660.1.S1_at	5.15	5.18	6.89	5.47	6.06	5.15	5.74	1.00	5.56	0.46	0.97	-0.046	7.9E-01
Mtr.1389.1.S1_at	6.61	4.90	6.64	6.41	6.73	6.74	6.05	1.00	6.63	0.19	1.09	0.130	3.8E-01
Mtr.20147.1.S1_s_at	3.57	2.37	4.35	4.11	4.52	3.48	3.43	1.00	4.04	0.52	1.18	0.236	4.0E-01
Mtr.39911.1.S1_at	6.25	5.00	6.97	6.30	6.23	6.30	6.07	1.00	6.28	0.04	1.03	0.048	7.4E-01
Mtr.33276.1.S1_s_at	7.42	5.56	7.11	7.27	7.57	7.26	6.69	1.00	7.36	0.18	1.10	0.138	3.1E-01
Mtr.9491.1.S1_at	8.15	6.41	8.12	7.97	7.87	8.28	7.56	1.00	8.04	0.22	1.06	0.089	4.6E-01
Mtr.44245.1.S1_at	9.99	8.00	8.81	9.46	9.23	9.33	8.93	1.00	9.34	0.12	1.05	0.065	5.2E-01
Mtr.27915.1.S1_at	4.76	3.21	5.07	4.90	4.50	4.76	4.35	1.00	4.72	0.20	1.09	0.119	5.6E-01
Mtr.21051.1.S1_at	2.76	2.79	4.50	3.70	2.68	2.74	3.35	1.00	3.04	0.57	0.91	-0.139	6.7E-01
Mtr.7234.1.S1_at	6.47	4.64	4.88	5.90	6.24	6.11	5.33	1.00	6.08	0.17	1.14	0.190	2.7E-01
Mtr.1170.1.S1_s_at	6.35	4.59	6.28	6.36	6.14	6.26	5.74	1.00	6.25	0.11	1.09	0.123	4.3E-01
Mtr.2133.1.S1_at	7.82	6.02	7.66	7.52	7.47	7.63	7.17	1.00	7.54	0.09	1.05	0.072	5.6E-01
Mtr.32351.1.S1_s_at	6.36	5.34	7.33	6.24	6.64	6.36	6.34	1.00	6.41	0.21	1.01	0.016	9.1E-01
Mtr.11390.1.S1_at	8.45	7.01	8.92	8.77	8.67	8.74	8.13	1.00	8.73	0.05	1.07	0.103	3.6E-01
Mtr.6165.1.S1_at	6.84	5.44	7.37	7.04	7.31	7.16	6.55	1.00	7.17	0.14	1.09	0.131	3.5E-01
Mtr.32372.1.S1_at	4.78	2.81	4.03	2.99	2.99	2.96	3.88	1.00	2.98	0.02	0.77	-0.379	1.9E-01
Mtr.16219.1.S1_at	11.05	10.61	9.15	10.83	10.96	11.18	10.27	1.00	10.99	0.18	1.07	0.098	2.9E-01
Mtr.44149.1.S1_at	11.40	9.85	11.70	11.52	11.44	11.36	10.98	1.00	11.44	0.08	1.04	0.059	4.7E-01
Mtr.42952.1.S1_at	6.27	4.69	6.52	5.66	5.56	5.54	5.83	1.00	5.59	0.07	0.96	-0.061	7.0E-01
Mtr.16550.1.S1_at	7.11	5.28	6.87	7.48	8.01	7.26	6.42	1.00	7.59	0.39	1.18	0.241	1.3E-01
Mtr.47896.1.S1_at	7.43	5.67	7.36	7.42	7.64	7.47	6.82	0.99	7.51	0.12	1.10	0.139	3.0E-01
Mtr.29328.1.S1_at	4.30	2.43	3.94	3.93	4.06	3.89	3.56	0.99	3.96	0.09	1.11	0.155	5.2E-01
Mtr.6371.1.S1_s_at	7.20	5.55	7.34	7.04	7.32	7.10	6.70	0.99	7.15	0.15	1.07	0.095	4.8E-01
Mtr.34335.1.S1_at	2.27	3.98	2.25	2.25	2.25	2.25	2.84	0.99	2.25	0.00	0.79	-0.335	3.6E-01
Mtr.7424.1.S1_at	7.36	5.81	7.67	7.19	7.27	7.31	6.95	0.99	7.26	0.06	1.04	0.062	6.2E-01
Mtr.39748.1.S1_at	4.02	2.09	3.47	3.45	3.12	4.15	3.19	0.99	3.57	0.53	1.12	0.162	5.9E-01
Mtr.47433.1.S1_at	7.76	5.82	7.16	7.03	7.16	6.93	6.91	0.99	7.04	0.12	1.02	0.026	8.4E-01
Mtr.4862.1.S1_at	7.84	6.28	8.12	7.71	7.92	7.82	7.42	0.99	7.82	0.11	1.05	0.076	5.2E-01
Mtr.11537.1.S1_at	7.35	5.46	6.95	6.83	6.94	7.13	6.59	0.99	6.97	0.15	1.06	0.080	5.5E-01
Mtr.20066.1.S1_at	4.42	2.93	4.81	4.35	4.60	5.07	4.05	0.99	4.68	0.37	1.15	0.206	3.7E-01
Mtr.14511.1.S1_at	2.40	4.12	2.39	2.38	2.39	2.39	2.97	0.99	2.39	0.00	0.80	-0.317	3.7E-01
Mtr.32542.1.S1_at	5.42	4.23	6.20	6.24	6.23	5.79	5.29	0.99	6.09	0.26	1.15	0.203	2.5E-01
Mtr.49618.1.S1_s_at	4.52	4.64	6.30	7.68	8.09	7.82	5.15	0.99	7.86	0.21	1.53	0.610	9.8E-03
Mtr.42294.1.S1_at	7.25	5.57	7.32	7.08	7.20	7.17	6.71	0.99	7.15	0.06	1.07	0.091	4.9E-01
Mtr.38933.1.S1_at	7.98	6.26	7.98	8.14	7.92	8.11	7.41	0.99	8.06	0.12	1.09	0.121	3.2E-01
Mtr.31897.1.S1_at	7.05	5.47	7.29	7.41	7.11	7.66	6.60	0.99	7.39	0.28	1.12	0.163	2.6E-01
Mtr.39000.1.S1_s_at	5.45	3.87	5.70	5.55	5.70	5.53	5.01	0.99	5.60	0.09	1.12	0.160	3.6E-01
Mtr.18113.1.S1_at	7.22	5.28	6.64	7.66	8.32	8.08	6.38	0.99	8.02	0.34	1.26	0.329	5.4E-02
Mtr.35865.1.S1_at	8.47	6.66	8.26	8.38	8.53	8.15	7.80	0.99	8.35	0.19	1.07	0.099	4.0E-01
Mtr.39022.1.S1_at	8.03	6.17	7.68	7.48	7.59	7.59	7.29	0.99	7.55	0.06	1.04	0.050	6.8E-01
Mtr.40010.1.S1_at	4.62	2.64	3.74	3.81	3.72	3.68	3.67	0.99	3.74	0.06	1.02	0.027	9.1E-01
Mtr.39455.1.S1_at	4.96	3.22	4.91	5.08	4.95	5.23	4.36	0.99	5.09	0.14	1.17	0.222	2.8E-01
Mtr.4233.1.S1_at	4.29	6.27	5.31	5.04	5.63	5.28	5.29	0.99	5.32	0.29	1.00	0.007	9.7E-01
Mtr.6503.1.S1_s_at	2.11	2.29	3.91	2.34	2.34	2.56	2.77	0.99	2.41	0.12	0.87	-0.198	5.7E-01
Mtr.26368.1.S1_at	3.39	4.84	2.94	3.04	2.93	2.97	3.73	0.99	2.98	0.05	0.80	-0.322	2.6E-01
Mtr.13993.1.S1_at	7.37	5.79	7.62	7.43	7.60	7.36	6.93	0.99	7.46	0.13	1.08	0.107	4.1E-01
Mtr.9635.1.S1_at	6.45	4.57	6.05	7.15	7.39	7.71	5.69	0.99	7.41	0.28	1.30	0.382	4.4E-02
Mtr.41940.1.S1_at	8.32	6.39	7.75	7.55	7.22	6.92	7.48	0.99	7.23	0.32	0.97	-0.050	6.9E-01
Mtr.41954.1.S1_s_at	6.54	4.58	5.81	6.15	6.31	6.15	5.64	0.99	6.20	0.10	1.10	0.137	3.8E-01
Mtr.40506.1.S1_at	10.41	9.23	11.20	10.78	10.81	10.61	10.28	0.99	10.73	0.11	1.04	0.062	4.8E-01
Mtr.33731.1.S1_at	6.21	4.51	6.24	6.18	6.07	5.98	5.65	0.99	6.08	0.10	1.08	0.105	5.0E-01
Mtr.39043.1.S1_at	7.56	5.66	7.11	7.24	7.31	7.29	6.77	0.99	7.28	0.04	1.07	0.103	4.3E-01
Mtr.20217.1.S1_at	3.56	3.24	5.09	3.18	4.20	3.21	3.96	0.99	3.53	0.58	0.89	-0.166	5.5E-01
Mtr.32925.1.S1_at	4.98	3.03	3.67	3.98	3.82	3.58	3.89	0.99	3.79	0.20	0.97	-0.038	8.7E-01
Mtr.20653.1.S1_at	7.28	5.99	7.94	7.48	7.45	7.36	7.07	0.99	7.43	0.06	1.05	0.072	5.6E-01
Mtr.3658.1.S1_at	8.90	6.94	7.63	7.67	8.25	7.80	7.83	0.99	7.91	0.30	1.01	0.015	9.0E-01
Mtr.36808.1.S1_at	7.25	5.48	7.14	7.40	7.00	6.66	6.62	0.99	7.02	0.37	1.06	0.084	5.5E-01
Mtr.5506.1.S1_at	8.68	7.12	8.96	8.55	8.94	8.81	8.25	0.99	8.77	0.20	1.06	0.088	4.2E-01
Mtr.6936.1.S1_s_at	8.04	6.10	7.41	7.57	7.70	7.67	7.18	0.99	7.65	0.07	1.06	0.091	4.6E-01
Mtr.14961.1.S1_x_at	3.21	4.90	3.15	3.46	2.60	3.15	3.75	0.99	3.07	0.43	0.82	-0.291	3.3E-01
Mtr.10258.1.S1_at	6.98	5.55	7.45	6.48	6.89	6.55	6.66	0.99	6.64	0.22	1.00	-0.004	9.7E-01
Mtr.31951.1.S1_at	10.56	8.90	8.79	9.38	9.52	9.23	9.42	0.99	9.38	0.15	1.00	-0.006	9.5E-01
Mtr.18198.1.S1_s_at	3.22	4.91	3.18	3.44	3.16	3.17	3.77	0.99	3.26	0.16	0.86	-0.212	4.2E-01
Mtr.52185.1.S1_at	9.68	7.78	8.27	8.48	8.61	8.63	8.58	0.99	8.57	0.08	1.00	-0.001	9.9E-01
Mtr.1502.1.S1_at	7.82	5.90	7.26	7.27	7.56	7.68	6.99	0.99	7.50	0.21	1.07	0.102	4.3E-01
Mtr.24836.1.S1_s_at	8.35	6.70	8.48	8.31	8.40	8.24	7.84	0.99	8.32	0.08	1.06	0.085	4.5E-01
Mtr.8088.1.S1_at	4.22	5.91	4.18	4.31	4.25	4.25	4.77	0.99	4.27	0.03	0.90	-0.159	4.3E-01
Mtr.9832.1.S1_at	7.05	5.25	6.87	7.05	7.10	6.84	6.39	0.99	7.00	0.14	1.10	0.131	3.5E-01
Mtr.35345.1.S1_at	4.67	4.15	6.06	7.02	7.89	6.84	4.96	0.99	7.25	0.56	1.46	0.547	2.5E-02
Mtr.1813.1.S1_at	7.68	6.02	7.77	8.24	8.07	7.90	7.16	0.99	8.07	0.17	1.13	0.174	1.9E-01
Mtr.31572.1.S1_at	3.92	5.61	3.88	3.85	3.63	4.08	4.47	0.99	3.85	0.23	0.86	-0.216	3.5E-01
Mtr.18882.1.S1_at	8.32	6.49	8.05	7.96	8.14	7.94	7.62	0.99	8.01	0.11	1.05	0.072	5.3E-01
Mtr.47136.1.S1_at	5.39	3.87	5.73	6.15	6.05	6.22	5.00	0.99	6.14	0.08	1.23	0.297	1.2E-01
Mtr.21678.1.S1_at	6.92	5.82	7.79	8.02	7.72	7.79	6.84	0.99	7.84	0.16	1.15	0.197	1.6E-01
Mtr.5504.1.S1_at	12.76	11.57	13.53	13.48	13.40	13.19	12.62	0.99	13.36	0.15	1.06	0.081	2.7E-01
Mtr.11999.1.S1_at	4.15	2.21	2.85	2.68	3.21	2.68	3.07	0.99	2.86	0.31	0.93	-0.104	7.4E-01
Mtr.7160.1.S1_at	6.51	4.71	6.31	6.51	6.43	6.94	5.84	0.99	6.62	0.27	1.13	0.182	2.6E-01
Mtr.34188.1.S1_at	2.20	3.84	2.06	2.20	2.20	2.20	2.70	0.99	2.20	0.00	0.81	-0.297	4.3E-01
Mtr.6715.1.S1_at	5.41	3.44	4.53	4.64	5.60	3.99	4.46	0.99	4.74	0.81	1.06	0.087	7.2E-01
Mtr.46661.1.S1_s_at	4.09	5.75	4.00	4.00	3.53	4.13	4.61	0.99	3.89	0.31	0.84	-0.247	2.9E-01
Mtr.29955.1.S1_at	5.29	3.81	5.68	6.10	7.27	6.97	4.93	0.99	6.78	0.61	1.38	0.461	5.0E-02
Mtr.5445.1.S1_at	8.14	6.65	8.51	8.59	8.79	8.31	7.76	0.99	8.56	0.24	1.10	0.141	2.4E-01
Mtr.28781.1.S1_at	11.17	9.32	10.83	10.96	11.00	10.90	10.44	0.99	10.95	0.05	1.05	0.069	4.2E-01
Mtr.45027.1.S1_at	4.10	2.55	4.37	4.78	4.35	4.39	3.67	0.99	4.51	0.24	1.23	0.295	2.3E-01
Mtr.3808.1.S1_at	3.23	4.92	3.20	3.18	3.21	3.21	3.78	0.99	3.20	0.01	0.85	-0.240	3.7E-01
Mtr.52005.1.S1_at	2.30	4.00	2.28	2.27	2.27	2.27	2.86	0.99	2.27	0.00	0.79	-0.332	3.6E-01
Mtr.4425.1.S1_at	8.58	6.76	8.32	8.31	8.40	8.40	7.89	0.99	8.37	0.05	1.06	0.086	4.4E-01
Mtr.30773.1.S1_at	7.91	5.98	6.61	6.61	6.54	6.60	6.84	0.99	6.58	0.04	0.96	-0.055	6.8E-01
Mtr.40585.1.S1_at	6.07	7.57	5.72	6.01	6.07	5.94	6.46	0.98	6.01	0.07	0.93	-0.103	4.8E-01
Mtr.25957.1.S1_s_at	8.09	6.22	7.70	7.73	7.86	7.79	7.34	0.98	7.79	0.06	1.06	0.086	4.7E-01
Mtr.375.1.S1_s_at	3.89	2.32	4.13	3.73	3.67	3.69	3.45	0.98	3.70	0.03	1.07	0.101	6.8E-01
Mtr.7173.1.S1_at	4.33	4.61	6.16	6.25	7.03	6.37	5.03	0.98	6.55	0.42	1.30	0.381	7.0E-02
Mtr.39939.1.S1_at	6.49	5.30	7.25	6.32	7.21	6.36	6.35	0.98	6.63	0.50	1.04	0.062	6.9E-01
Mtr.46928.1.S1_at	2.67	4.34	2.61	3.13	2.65	2.65	3.21	0.98	2.81	0.28	0.88	-0.192	5.4E-01
Mtr.30014.1.S1_at	5.57	4.37	6.32	5.55	6.31	6.01	5.42	0.98	5.96	0.38	1.10	0.138	4.2E-01
Mtr.35342.1.S1_at	5.69	4.03	3.94	3.79	3.68	3.93	4.56	0.98	3.80	0.13	0.83	-0.261	2.6E-01
Mtr.41918.1.S1_at	8.47	6.73	8.39	8.21	8.28	8.35	7.86	0.98	8.28	0.07	1.05	0.074	5.1E-01
Mtr.38096.1.S1_at	9.80	7.85	9.03	9.07	9.09	9.07	8.89	0.98	9.07	0.02	1.02	0.029	7.6E-01
Mtr.27923.1.S1_s_at	8.49	6.53	7.62	7.81	7.48	8.08	7.55	0.98	7.79	0.30	1.03	0.045	7.1E-01
Mtr.40497.1.S1_at	9.20	7.29	8.68	8.55	9.03	8.57	8.39	0.98	8.71	0.27	1.04	0.055	6.1E-01
Mtr.44543.1.S1_at	5.39	3.81	5.61	5.32	5.31	5.50	4.93	0.98	5.37	0.11	1.09	0.123	4.8E-01
Mtr.35176.1.S1_at	7.98	6.27	7.96	8.00	7.94	8.11	7.41	0.98	8.02	0.08	1.08	0.114	3.4E-01
Mtr.17456.1.S1_at	6.37	4.65	6.34	4.47	4.62	3.73	5.79	0.98	4.27	0.48	0.74	-0.437	7.5E-02
Mtr.40306.1.S1_at	12.86	11.39	11.00	11.30	10.96	11.59	11.75	0.98	11.28	0.32	0.96	-0.059	4.8E-01
Mtr.6627.1.S1_at	2.50	4.19	2.47	2.49	2.48	2.47	3.05	0.98	2.48	0.01	0.81	-0.300	3.7E-01
Mtr.32878.1.S1_at	8.63	6.68	7.88	8.32	7.92	8.44	7.73	0.98	8.23	0.27	1.06	0.090	4.5E-01
Mtr.45958.1.S1_s_at	8.37	7.06	8.98	8.46	8.60	8.51	8.14	0.98	8.53	0.07	1.05	0.067	5.3E-01
Mtr.21989.1.S1_x_at	7.39	6.15	8.09	7.33	7.22	7.25	7.21	0.98	7.27	0.05	1.01	0.012	9.2E-01
Mtr.7386.1.S1_at	8.99	7.35	9.11	9.45	9.35	9.24	8.49	0.98	9.35	0.11	1.10	0.139	2.1E-01
Mtr.1564.1.S1_s_at	8.14	6.30	7.81	8.22	8.57	8.18	7.41	0.98	8.32	0.21	1.12	0.167	1.9E-01
Mtr.31071.1.S1_at	4.42	3.08	4.99	4.25	4.90	4.42	4.16	0.98	4.53	0.34	1.09	0.120	5.8E-01
Mtr.46103.1.S1_at	2.96	4.65	2.93	2.91	2.85	2.92	3.51	0.98	2.89	0.04	0.82	-0.280	3.4E-01
Mtr.27457.1.S1_at	9.23	7.40	8.92	8.97	8.80	9.06	8.52	0.98	8.94	0.13	1.05	0.070	5.0E-01
Mtr.43425.1.S1_at	10.33	8.47	8.87	9.67	9.80	9.69	9.22	0.98	9.72	0.07	1.05	0.076	4.3E-01
Mtr.33305.1.S1_at	6.22	4.55	6.27	5.74	6.38	5.83	5.68	0.98	5.98	0.35	1.05	0.075	6.4E-01
Mtr.49054.1.S1_x_at	5.81	7.47	5.74	5.73	5.68	5.90	6.34	0.98	5.77	0.11	0.91	-0.137	3.7E-01
Mtr.33020.1.S1_at	7.15	5.24	6.61	6.90	6.89	6.91	6.33	0.98	6.90	0.01	1.09	0.123	3.7E-01
Mtr.10224.1.S1_at	5.30	3.77	5.60	5.60	6.48	5.58	4.89	0.98	5.89	0.52	1.20	0.268	1.9E-01
Mtr.32752.1.S1_s_at	6.11	4.46	6.20	5.76	5.75	5.74	5.59	0.98	5.75	0.01	1.03	0.041	7.9E-01
Mtr.5802.1.S1_at	4.93	3.28	5.02	5.72	5.15	5.31	4.41	0.98	5.39	0.29	1.22	0.291	1.7E-01
Mtr.37585.1.S1_a_at	9.72	8.15	9.95	10.58	9.33	10.17	9.27	0.98	10.03	0.64	1.08	0.112	3.3E-01
Mtr.6925.1.S1_x_at	8.06	6.12	7.35	7.68	7.64	7.71	7.17	0.98	7.68	0.04	1.07	0.098	4.2E-01
Mtr.3043.1.S1_at	2.55	4.25	2.55	2.53	2.54	2.54	3.12	0.98	2.54	0.01	0.81	-0.296	3.6E-01

Mtr.38328.1.S1_at	8.30	6.59	8.28	8.29	8.33	8.39	7.72	0.98	8.34	0.05	1.08	0.111	3.4E-01
Mtr.34459.1.S1_at	4.97	3.11	4.58	4.90	4.91	4.75	4.22	0.98	4.85	0.09	1.15	0.203	3.3E-01
Mtr.2694.1.S1_at	9.80	9.05	7.86	9.37	8.86	9.65	8.90	0.98	9.29	0.40	1.04	0.062	5.6E-01
Mtr.39412.1.S1_at	6.22	4.86	6.76	6.55	6.08	6.08	5.95	0.98	6.24	0.27	1.05	0.068	6.5E-01
Mtr.21596.1.S1_x_at	5.58	6.86	4.93	5.71	5.29	5.55	5.79	0.98	5.51	0.21	0.95	-0.070	6.6E-01
Mtr.35044.1.S1_at	7.86	6.55	5.95	8.05	7.48	8.21	6.79	0.98	7.91	0.38	1.17	0.221	1.4E-01
Mtr.43433.1.S1_at	8.87	7.04	8.57	8.98	10.12	9.20	8.16	0.98	9.43	0.60	1.16	0.209	1.3E-01
Mtr.6724.1.S1_at	8.74	7.01	8.66	8.26	8.46	8.21	8.14	0.98	8.31	0.13	1.02	0.031	7.7E-01
Mtr.43880.1.S1_at	5.97	4.17	5.73	5.89	5.67	5.73	5.29	0.98	5.76	0.12	1.09	0.124	4.5E-01
Mtr.48485.1.S1_at	2.36	4.06	2.36	2.37	2.36	2.34	2.92	0.98	2.36	0.02	0.81	-0.312	3.7E-01
Mtr.28537.1.S1_at	5.90	4.06	5.56	5.31	5.72	5.48	5.17	0.98	5.50	0.20	1.06	0.090	6.0E-01
Mtr.16612.1.S1_s_at	5.54	4.20	6.10	5.65	5.35	5.38	5.28	0.98	5.46	0.16	1.03	0.048	7.7E-01
Mtr.14078.1.S1_x_at	2.45	4.14	2.44	2.45	2.38	2.44	3.01	0.98	2.42	0.04	0.80	-0.316	3.5E-01
Mtr.44976.1.S1_at	3.80	2.49	4.40	4.89	4.11	4.54	3.56	0.98	4.52	0.39	1.27	0.343	1.9E-01
Mtr.27578.1.S1_at	6.92	5.01	6.30	6.39	6.77	6.74	6.08	0.98	6.63	0.21	1.09	0.126	3.9E-01
Mtr.18918.1.S1_at	2.47	4.22	2.60	2.46	2.59	3.10	3.10	0.98	2.72	0.34	0.88	-0.186	5.6E-01
Mtr.32801.1.S1_at	5.15	7.06	5.78	5.68	5.94	5.61	6.00	0.98	5.74	0.18	0.96	-0.063	6.8E-01
Mtr.49421.1.S1_at	9.65	8.11	9.93	10.41	9.90	10.43	9.23	0.98	10.25	0.30	1.11	0.151	1.6E-01
Mtr.29737.1.S1_at	4.87	3.26	5.03	4.40	4.75	4.51	4.39	0.98	4.55	0.18	1.04	0.053	7.9E-01
Mtr.40991.1.S1_at	5.29	3.57	3.64	3.39	4.37	3.43	4.17	0.98	3.73	0.55	0.90	-0.160	5.4E-01
Mtr.12880.1.S1_at	7.94	6.08	7.52	7.03	7.01	6.84	7.18	0.98	6.96	0.11	0.97	-0.045	7.2E-01
Mtr.43478.1.S1_at	13.57	11.92	13.65	13.82	13.65	13.60	13.04	0.98	13.69	0.12	1.05	0.070	3.2E-01
Mtr.4750.1.S1_at	7.11	5.63	7.48	6.98	7.04	6.58	6.74	0.98	6.86	0.25	1.02	0.026	8.4E-01
Mtr.9254.1.S1_at	12.41	10.51	11.87	12.03	11.33	11.94	11.60	0.98	11.76	0.38	1.01	0.021	7.9E-01
Mtr.32487.1.S1_s_at	6.54	5.01	6.83	7.08	6.94	6.59	6.13	0.98	6.87	0.25	1.12	0.166	2.7E-01
Mtr.3396.1.S1_at	7.01	5.24	6.83	6.66	7.11	6.90	6.36	0.98	6.89	0.22	1.08	0.116	4.1E-01
Mtr.38678.1.S1_at	8.93	7.33	9.10	8.95	9.08	9.22	8.45	0.98	9.08	0.14	1.07	0.104	3.3E-01
Mtr.9760.1.S1_at	5.35	3.42	4.63	4.08	4.96	4.09	4.47	0.98	4.38	0.51	0.98	-0.029	9.0E-01
Mtr.48622.1.S1_x_at	3.02	4.63	2.88	3.00	3.01	3.00	3.51	0.98	3.00	0.01	0.85	-0.226	4.2E-01
Mtr.44963.1.S1_at	5.08	3.44	5.17	5.03	5.79	5.15	4.57	0.98	5.32	0.41	1.17	0.221	2.8E-01
Mtr.20832.1.S1_at	2.65	4.34	2.65	2.63	2.63	2.52	3.21	0.97	2.59	0.06	0.81	-0.309	3.3E-01
Mtr.1824.1.S1_at	6.41	4.71	6.39	6.37	6.09	6.35	5.84	0.97	6.27	0.16	1.07	0.103	4.9E-01
Mtr.14112.1.S1_s_at	6.43	4.58	6.03	6.13	5.75	5.16	5.68	0.97	5.68	0.49	1.00	0.001	1.0E+00
Mtr.44860.1.S1_at	7.07	5.17	5.74	5.84	5.26	4.70	5.99	0.97	5.27	0.57	0.88	-0.187	3.3E-01
Mtr.38830.1.S1_at	6.64	4.82	6.34	6.53	6.62	6.93	5.93	0.97	6.69	0.21	1.13	0.174	2.6E-01
Mtr.1867.1.S1_at	5.36	3.70	5.41	5.42	5.34	5.31	4.82	0.97	5.36	0.06	1.11	0.151	4.0E-01
Mtr.6447.1.S1_at	2.24	3.93	2.24	2.23	2.23	2.23	2.80	0.97	2.23	0.00	0.79	-0.331	3.6E-01
Mtr.4780.1.S1_at	6.94	5.27	6.98	7.04	6.91	6.84	6.40	0.97	6.93	0.10	1.08	0.115	4.0E-01
Mtr.19013.1.S1_at	7.98	6.04	6.88	7.25	6.80	7.29	6.97	0.97	7.11	0.27	1.02	0.030	8.2E-01
Mtr.38123.1.S1_at	10.19	8.32	9.75	9.68	9.65	9.69	9.42	0.97	9.67	0.02	1.03	0.038	6.8E-01
Mtr.10188.1.S1_x_at	5.70	3.80	5.11	5.56	5.20	5.57	4.87	0.97	5.44	0.21	1.12	0.160	3.8E-01
Mtr.43104.1.S1_at	10.06	8.48	8.29	9.14	9.72	9.46	8.94	0.97	9.44	0.29	1.06	0.078	4.5E-01
Mtr.17478.1.S1_at	7.55	5.73	7.25	7.31	7.93	7.47	6.84	0.97	7.57	0.32	1.11	0.146	2.9E-01
Mtr.34747.1.S1_at	5.77	4.92	6.86	5.22	5.18	5.07	5.85	0.97	5.16	0.08	0.88	-0.182	2.9E-01
Mtr.34193.1.S1_at	1.82	3.50	1.81	1.81	1.81	1.81	2.38	0.97	1.81	0.00	0.76	-0.394	3.7E-01
Mtr.12277.1.S1_at	12.86	11.03	11.38	12.05	11.83	12.10	11.76	0.97	11.99	0.14	1.02	0.028	7.0E-01
Mtr.2146.1.S1_at	2.53	4.21	2.52	2.50	2.52	2.51	3.09	0.97	2.51	0.01	0.81	-0.301	3.6E-01
Mtr.27330.1.S1_at	8.02	6.10	7.33	7.67	7.46	7.79	7.15	0.97	7.64	0.17	1.07	0.096	4.4E-01
Mtr.30443.1.S1_at	6.14	4.90	6.82	6.34	6.02	6.70	5.95	0.97	6.35	0.34	1.07	0.094	5.4E-01
Mtr.1393.1.S1_at	7.90	6.04	7.47	8.03	7.74	7.75	7.14	0.97	7.84	0.16	1.10	0.135	2.8E-01
Mtr.35814.1.S1_at	7.49	5.91	7.68	7.51	7.68	7.40	7.02	0.97	7.53	0.14	1.07	0.101	4.2E-01
Mtr.38717.1.S1_at	6.50	4.80	6.47	6.15	6.46	6.19	5.93	0.97	6.27	0.17	1.06	0.081	5.8E-01
Mtr.21271.1.S1_at	4.42	2.84	4.61	5.17	5.85	4.95	3.96	0.97	5.32	0.47	1.34	0.427	9.4E-02
Mtr.689.1.S1_at	5.53	3.94	5.70	5.94	3.95	5.03	5.06	0.97	4.97	1.00	0.98	-0.023	9.2E-01
Mtr.50937.1.S1_at	2.61	4.29	2.60	2.60	2.60	2.60	3.17	0.97	2.60	0.00	0.82	-0.286	3.7E-01
Mtr.9739.1.S1_at	9.29	11.18	10.63	10.01	11.05	10.61	10.37	0.97	10.55	0.52	1.02	0.026	7.8E-01
Mtr.22878.1.S1_x_at	2.87	4.54	2.84	2.83	2.75	2.82	3.42	0.97	2.80	0.04	0.82	-0.287	3.3E-01
Mtr.20122.1.S1_at	5.69	4.01	5.70	5.35	5.50	5.17	5.13	0.97	5.34	0.17	1.04	0.057	7.3E-01
Mtr.19706.1.S1_s_at	10.29	8.48	8.77	8.77	9.49	9.34	9.18	0.97	9.20	0.38	1.00	0.003	9.7E-01
Mtr.15285.1.S1_at	12.44	10.67	10.87	11.38	10.25	11.40	11.33	0.97	11.01	0.66	0.97	-0.041	6.6E-01
Mtr.33674.1.S1_s_at	7.17	6.21	8.15	7.50	7.53	6.89	7.18	0.97	7.31	0.36	1.02	0.026	8.4E-01
Mtr.11025.1.S1_at	7.51	5.84	7.52	7.31	7.38	7.54	6.96	0.97	7.41	0.12	1.07	0.091	4.7E-01
Mtr.32131.1.S1_at	7.92	6.31	8.05	7.90	7.93	7.98	7.42	0.97	7.93	0.04	1.07	0.096	4.1E-01
Mtr.34530.1.S1_at	2.87	4.53	2.82	2.85	2.82	2.83	3.41	0.97	2.84	0.02	0.83	-0.266	3.6E-01
Mtr.11503.1.S1_at	7.95	6.19	6.35	6.33	6.55	6.98	6.83	0.97	6.62	0.33	0.97	-0.045	7.4E-01
Mtr.8511.1.S1_at	8.37	6.68	8.35	8.63	8.36	8.68	7.80	0.97	8.56	0.17	1.10	0.134	2.5E-01
Mtr.30562.1.S1_at	2.23	3.91	2.22	2.23	2.21	2.21	2.79	0.97	2.22	0.01	0.79	-0.331	3.6E-01
Mtr.34318.1.S1_at	7.61	6.42	8.34	7.85	8.02	7.73	7.45	0.97	7.87	0.15	1.06	0.078	5.1E-01
Mtr.18035.1.S1_at	9.65	7.83	9.32	9.17	9.03	9.12	8.94	0.97	9.11	0.07	1.02	0.027	7.8E-01
Mtr.22911.1.S1_at	2.24	3.90	2.20	2.26	2.19	2.24	2.78	0.97	2.23	0.04	0.80	-0.318	3.8E-01
Mtr.4438.1.S1_at	3.38	2.54	4.47	5.35	3.21	4.83	3.47	0.97	4.47	1.12	1.29	0.366	3.1E-01
Mtr.21198.1.S1_at	1.97	3.65	1.96	1.96	1.96	1.96	2.53	0.97	1.96	0.00	0.77	-0.369	3.7E-01
Mtr.42826.1.S1_at	9.38	7.46	8.67	9.27	9.06	9.40	8.50	0.97	9.24	0.17	1.09	0.120	2.6E-01
Mtr.29045.1.S1_at	6.48	4.79	6.45	6.62	6.73	6.54	5.90	0.97	6.63	0.09	1.12	0.167	2.7E-01
Mtr.15347.1.S1_at	7.81	6.28	8.08	8.03	7.87	7.79	7.39	0.97	7.90	0.12	1.07	0.096	4.2E-01
Mtr.28806.1.S1_at	7.89	6.17	7.80	7.83	7.82	7.67	7.29	0.97	7.77	0.09	1.07	0.093	4.4E-01
Mtr.23668.1.S1_s_at	5.91	4.39	6.19	5.93	6.42	5.02	5.49	0.97	5.79	0.71	1.05	0.076	6.9E-01
Mtr.43320.1.S1_at	5.60	5.26	7.08	6.81	7.14	6.30	5.98	0.97	6.75	0.42	1.13	0.174	2.8E-01
Mtr.50822.1.S1_x_at	4.39	6.15	4.57	4.38	4.39	4.37	5.04	0.97	4.38	0.01	0.87	-0.202	3.0E-01
Mtr.36821.1.S1_at	2.94	4.61	2.93	2.93	2.93	2.93	3.49	0.97	2.93	0.00	0.84	-0.254	3.7E-01
Mtr.38070.1.S1_at	8.65	6.72	7.64	8.08	8.19	8.14	7.67	0.97	8.13	0.05	1.06	0.085	4.5E-01
Mtr.8142.1.S1_at	1.99	3.66	1.97	1.97	1.97	1.97	2.54	0.97	1.97	0.00	0.78	-0.367	3.6E-01
Mtr.41960.1.S1_at	10.82	9.70	8.90	10.40	9.89	10.62	9.81	0.97	10.30	0.37	1.05	0.071	4.5E-01
Mtr.39214.1.S1_at	13.39	11.81	11.63	12.37	12.35	12.53	12.28	0.97	12.42	0.10	1.01	0.017	8.1E-01
Mtr.29433.1.S1_at	4.59	5.59	6.52	5.11	6.70	5.30	5.57	0.97	5.70	0.87	1.02	0.035	8.7E-01
Mtr.37188.1.S1_at	8.12	6.43	8.09	7.74	7.82	7.53	7.54	0.97	7.70	0.15	1.02	0.029	8.0E-01
Mtr.40668.1.S1_at	9.50	8.09	7.65	8.54	8.11	8.60	8.41	0.97	8.42	0.26	1.00	0.001	9.9E-01
Mtr.35704.1.S1_at	9.04	7.19	8.61	9.03	9.73	9.22	8.28	0.97	9.33	0.36	1.13	0.171	1.5E-01
Mtr.35144.1.S1_s_at	8.46	6.56	7.81	8.15	8.15	8.08	7.61	0.97	8.13	0.04	1.07	0.096	4.0E-01
Mtr.972.1.S1_at	8.80	8.08	6.88	8.09	7.90	8.32	7.92	0.97	8.10	0.21	1.02	0.033	7.6E-01
Mtr.48583.1.S1_at	6.76	5.61	7.53	6.76	6.83	6.66	6.63	0.97	6.75	0.08	1.02	0.026	8.4E-01
Mtr.51090.1.S1_at	2.59	4.34	2.76	2.56	2.55	2.56	3.23	0.97	2.55	0.01	0.79	-0.340	2.9E-01
Mtr.28436.1.S1_s_at	7.46	5.60	6.99	6.97	7.07	7.06	6.69	0.97	7.03	0.05	1.05	0.073	5.7E-01
Mtr.35667.1.S1_at	7.71	5.89	7.37	7.67	7.73	7.42	6.99	0.97	7.61	0.17	1.09	0.122	3.4E-01
Mtr.8965.1.S1_at	4.33	3.07	4.97	4.65	4.30	4.03	4.12	0.97	4.32	0.31	1.05	0.070	7.4E-01
Mtr.23.1.S1_at	3.98	2.57	4.41	4.08	4.09	4.27	3.65	0.97	4.15	0.11	1.14	0.184	4.3E-01
Mtr.35609.1.S1_at	5.97	5.11	7.04	6.48	6.41	6.46	6.04	0.97	6.45	0.03	1.07	0.095	5.0E-01
Mtr.5628.1.S1_s_at	5.07	3.55	3.28	3.40	3.28	3.40	3.97	0.97	3.36	0.07	0.85	-0.239	3.4E-01
Mtr.39787.1.S1_at	8.13	6.53	8.26	8.10	8.32	8.05	7.64	0.97	8.16	0.14	1.07	0.095	4.1E-01
Mtr.33062.1.S1_at	4.99	3.60	5.46	4.79	4.76	4.74	4.68	0.97	4.76	0.02	1.02	0.024	9.0E-01
Mtr.11391.1.S1_at	10.10	8.38	10.00	10.54	10.58	10.47	9.49	0.97	10.53	0.05	1.11	0.150	1.4E-01
Mtr.42097.1.S1_at	6.93	5.05	6.39	5.87	5.82	5.79	6.12	0.97	5.83	0.04	0.95	-0.071	6.2E-01
Mtr.1276.1.S1_at	2.29	3.96	2.27	2.27	2.26	2.27	2.84	0.97	2.27	0.01	0.80	-0.325	3.6E-01
Mtr.35409.1.S1_at	10.66	8.93	10.54	10.04	10.07	9.97	10.04	0.97	10.03	0.05	1.00	-0.002	9.8E-01
Mtr.35112.1.S1_at	6.75	5.26	7.07	6.98	7.02	6.81	6.36	0.97	6.94	0.11	1.09	0.126	3.6E-01
Mtr.9486.1.S1_at	9.21	8.65	10.53	9.59	9.90	9.06	9.46	0.96	9.52	0.43	1.01	0.008	9.3E-01
Mtr.40636.1.S1_at	12.97	11.11	12.51	12.63	12.36	12.84	12.19	0.96	12.61	0.24	1.03	0.048	5.1E-01
Mtr.5298.1.S1_s_at	3.54	5.21	3.53	3.54	3.52	3.52	4.09	0.96	3.53	0.01	0.86	-0.215	3.7E-01
Mtr.19346.1.S1_s_at	4.40	4.29	6.01	6.62	6.54	5.60	4.90	0.96	6.25	0.57	1.28	0.351	1.0E-01
Mtr.39422.1.S1_s_at	7.64	5.92	7.54	7.50	7.46	7.59	7.04	0.96	7.52	0.07	1.07	0.096	4.3E-01
Mtr.32852.1.S1_at	6.13	4.46	6.12	5.56	6.02	6.23	5.57	0.96	5.94	0.34	1.07	0.092	5.7E-01
Mtr.24546.1.S1_at	3.06	4.77	3.16	3.16	3.06	3.15	3.66	0.96	3.12	0.05	0.85	-0.230	3.9E-01
Mtr.28526.1.S1_at	7.53	5.61	6.73	7.21	7.00	7.44	6.62	0.96	7.22	0.22	1.09	0.124	3.6E-01
Mtr.47134.1.S1_at	2.34	3.99	2.30	2.35	2.30	2.33	2.88	0.96	2.32	0.03	0.81	-0.308	3.8E-01
Mtr.39065.1.S1_at	3.52	4.11	5.40	4.53	4.50	4.23	4.34	0.96	4.42	0.16	1.02	0.026	9.0E-01
Mtr.8830.1.S1_at	11.11	9.88	11.78	11.24	11.25	11.03	10.93	0.96	11.17	0.12	1.02	0.032	6.8E-01
Mtr.47024.1.S1_x_at	2.68	4.34	2.66	2.65	2.63	2.62	3.23	0.96	2.63	0.01	0.81	-0.295	3.4E-01
Mtr.10198.1.S1_at	8.06	6.33	6.45	6.88	6.19	6.60	6.95	0.96	6.56	0.35	0.94	-0.082	5.5E-01
Mtr.34165.1.S1_at	6.03	4.40	6.11	7.07	7.03	7.01	5.51	0.96	7.03	0.03	1.28	0.351	5.2E-02
Mtr.37908.1.S1_at	13.59	12.05	13.83	13.88	13.68	13.65	13.15	0.96	13.74	0.13	1.04	0.063	3.6E-01
Mtr.45264.1.S1_at	7.57	6.57	8.50	7.60	7.49	7.39	7.55	0.96	7.49	0.10	0.99	-0.010	9.3E-01
Mtr.32243.1.S1_at	6.95	5.09	6.46	6.59	6.97	7.14	6.17	0.96	6.90	0.28	1.12	0.161	2.8E-01
Mtr.43931.1.S1_at	6.46	4.66	6.14	5.64	6.16	5.98	5.75	0.96	5.93	0.26	1.03	0.042	7.8E-01
Mtr.26676.1.S1_at	4.88	3.84	5.76	5.07	5.32	5.03	4.82	0.96	5.14	0.16	1.07	0.091	6.1E-01
Mtr.48662.1.S1_s_at	5.25	3.60	5.29	4.61	5.08	4.18	4.71	0.96	4.62	0.45	0.98	-0.027	8.9E-01
Mtr.24188.1.S1_at	4.51	3.54	5.47	4.62	4.64	4.38	4.51	0.96	4.55	0.15	1.01	0.011	9.5E-01
Mtr.52320.1.S1_at	5.23	3.45	4.97	4.63	4.51	4.59	4.55	0.96	4.58	0.06	1.01	0.009	9.6E-01
Mtr.17315.1.S1_at	2.62	4.27	2.59	2.60	2.57	2.60	3.16	0.96	2.59	0.02	0.82	-0.287	3.6E-01
Mtr.50590.1.S1_at	2.56	4.23	2.55	2.54	2.54	2.54	3.11	0.96	2.54	0.00	0.82	-0.294	3.6E-01
Mtr.22569.1.S1_s_at	1.91	1.87	3.56	5.75	4.25	4.60	2.45	0.96	4.87	0.78	1.99	0.993	2.8E-02
Mtr.37015.1.S1_at	8.75	7.09	8.75	8.64	8.53	8.51	8.20	0.96	8.56	0.07	1.04	0.063	5.5E-01

Mtr.27955.1.S1_at	6.38	5.54	7.46	6.97	6.86	7.27	6.46	0.96	7.03	0.21	1.09	0.123	3.7E-01
Mtr.261.1.S1_at	4.47	2.94	4.72	4.41	4.59	4.48	4.05	0.96	4.50	0.09	1.11	0.152	4.7E-01
Mtr.32259.1.S1_at	8.38	7.04	8.90	8.45	8.97	8.50	8.11	0.96	8.64	0.28	1.07	0.092	4.1E-01
Mtr.38441.1.S1_at	6.99	5.19	6.69	6.57	6.81	6.47	6.29	0.96	6.62	0.17	1.05	0.074	5.9E-01
Mtr.3697.1.S1_at	2.73	4.62	3.38	2.92	2.85	2.66	3.57	0.96	2.81	0.14	0.79	-0.349	2.4E-01
Mtr.37688.1.S1_at	11.44	9.74	11.37	11.80	11.65	11.99	10.85	0.96	11.82	0.17	1.09	0.123	1.6E-01
Mtr.27069.1.S1_at	6.89	5.59	7.46	7.10	7.29	7.23	6.65	0.96	7.21	0.10	1.08	0.117	3.7E-01
Mtr.4723.1.S1_at	7.56	5.64	6.54	6.41	6.78	6.52	6.58	0.96	6.57	0.19	1.00	-0.003	9.8E-01
Mtr.42129.1.S1_at	7.02	8.56	6.79	7.48	7.70	7.65	7.46	0.96	7.61	0.12	1.02	0.029	8.0E-01
Mtr.13491.1.S1_at	9.27	8.74	10.60	10.09	10.60	10.10	9.54	0.96	10.26	0.29	1.08	0.106	2.8E-01
Mtr.39483.1.S1_at	9.04	7.47	9.22	9.08	8.98	8.78	8.57	0.96	8.94	0.15	1.04	0.061	5.5E-01
Mtr.39616.1.S1_at	3.48	1.98	3.77	3.65	3.46	4.04	3.07	0.96	3.72	0.29	1.21	0.274	3.3E-01
Mtr.6036.1.S1_s_at	9.76	8.04	9.64	9.95	9.70	9.91	9.15	0.96	9.85	0.13	1.08	0.107	2.8E-01
Mtr.28542.1.S1_at	7.04	5.75	7.63	7.52	7.36	7.59	6.81	0.96	7.49	0.12	1.10	0.138	2.9E-01
Mtr.35821.1.S1_at	7.78	6.03	7.60	7.75	7.80	7.65	7.14	0.96	7.73	0.08	1.08	0.116	3.4E-01
Mtr.27821.1.S1_at	6.46	4.68	6.18	6.63	6.91	6.73	5.77	0.96	6.76	0.14	1.17	0.228	1.5E-01
Mtr.16274.1.S1_at	10.29	8.44	9.81	9.45	9.05	9.15	9.51	0.96	9.22	0.20	0.97	-0.046	6.3E-01
Mtr.5384.1.S1_at	7.56	5.91	7.58	8.03	8.17	8.10	7.02	0.96	8.10	0.07	1.15	0.207	1.2E-01
Mtr.34710.1.S1_at	7.12	5.43	7.06	7.20	7.31	7.05	6.54	0.96	7.19	0.13	1.10	0.136	3.1E-01
Mtr.10365.1.S1_s_at	13.06	11.14	12.15	12.17	12.48	12.56	12.12	0.96	12.40	0.21	1.02	0.034	6.4E-01
Mtr.29577.1.S1_at	8.04	6.21	7.64	6.98	6.97	6.95	7.29	0.96	6.97	0.01	0.96	-0.066	5.9E-01
Mtr.47158.1.S1_at	5.02	3.11	4.20	4.24	4.24	4.46	4.11	0.96	4.31	0.13	1.05	0.069	7.4E-01
Mtr.50563.1.S1_at	4.21	2.45	4.00	4.03	3.73	3.74	3.55	0.96	3.83	0.17	1.08	0.110	6.4E-01
Mtr.45294.1.S1_at	5.11	3.28	3.71	3.65	3.62	3.64	4.03	0.96	3.64	0.02	0.90	-0.150	5.1E-01
Mtr.1951.1.S1_s_at	9.18	8.62	10.49	9.60	9.95	8.79	9.43	0.96	9.45	0.59	1.00	0.002	9.9E-01
Mtr.8859.1.S1_at	11.02	9.33	10.95	11.27	11.16	11.00	10.43	0.96	11.14	0.14	1.07	0.095	2.7E-01
Mtr.32510.1.S1_at	6.98	5.39	7.11	6.75	6.74	6.97	6.50	0.96	6.82	0.13	1.05	0.070	5.9E-01
Mtr.48643.1.S1_at	7.14	6.00	7.91	7.53	7.22	7.14	7.02	0.96	7.30	0.21	1.04	0.056	6.5E-01
Mtr.16186.1.S1_at	5.35	4.11	5.99	6.48	5.87	5.73	5.15	0.96	6.03	0.40	1.17	0.228	2.2E-01
Mtr.5789.1.S1_s_at	9.59	8.25	10.10	10.13	9.79	10.09	9.31	0.96	10.00	0.19	1.07	0.103	2.9E-01
Mtr.6047.1.S1_at	5.97	4.39	6.11	5.92	6.47	5.73	5.49	0.96	6.04	0.38	1.10	0.139	4.1E-01
Mtr.42746.1.S1_at	6.93	5.61	7.47	7.30	7.30	7.41	6.67	0.96	7.34	0.06	1.10	0.137	3.0E-01
Mtr.32054.1.S1_at	4.83	3.17	4.81	5.03	4.82	4.81	4.27	0.96	4.89	0.12	1.14	0.195	3.3E-01
Mtr.50321.1.S1_at	3.39	5.03	3.37	3.35	3.32	3.36	3.93	0.96	3.34	0.02	0.85	-0.233	3.5E-01
Mtr.15135.1.S1_s_at	7.30	6.06	7.94	7.66	6.99	7.16	7.10	0.96	7.27	0.35	1.02	0.035	7.8E-01
Mtr.14949.1.S1_at	7.23	6.70	8.56	6.30	7.70	6.89	7.50	0.96	6.97	0.70	0.93	-0.106	4.8E-01
Mtr.22607.1.S1_at	3.52	5.14	3.46	4.21	3.40	3.45	4.04	0.96	3.69	0.45	0.91	-0.132	5.9E-01
Mtr.30327.1.S1_at	6.45	4.78	6.43	6.43	7.02	6.43	5.89	0.96	6.63	0.34	1.13	0.170	2.8E-01
Mtr.39157.1.S1_s_at	7.74	5.83	6.91	7.32	7.39	7.50	6.83	0.96	7.40	0.09	1.08	0.117	3.6E-01
Mtr.4684.1.S1_s_at	9.02	7.87	9.76	9.42	9.63	9.40	8.88	0.96	9.48	0.13	1.07	0.095	3.4E-01
Mtr.48950.1.S1_at	5.28	4.15	6.05	5.54	5.26	5.33	5.16	0.96	5.38	0.15	1.04	0.059	7.2E-01
Mtr.3107.1.S1_at	5.57	4.07	5.85	6.54	6.70	6.39	5.16	0.96	6.54	0.16	1.27	0.342	6.9E-02
Mtr.43514.1.S1_at	6.76	7.76	8.67	9.87	10.50	10.01	7.73	0.96	10.13	0.33	1.31	0.389	1.5E-02
Mtr.41912.1.S1_s_at	7.75	6.07	7.69	7.91	7.59	7.94	7.17	0.96	7.81	0.19	1.09	0.124	3.2E-01
Mtr.46108.1.S1_s_at	2.45	4.09	2.43	2.41	2.41	2.41	2.99	0.96	2.41	0.00	0.81	-0.311	3.5E-01
Mtr.4561.1.S1_at	5.10	3.48	5.17	4.84	6.63	5.09	4.58	0.96	5.52	0.97	1.21	0.270	3.0E-01
Mtr.42188.1.S1_at	5.22	4.83	6.64	6.13	5.86	5.86	5.56	0.95	5.95	0.16	1.07	0.098	5.2E-01
Mtr.41776.1.S1_at	7.25	6.22	8.13	8.10	7.34	8.47	7.20	0.95	7.97	0.58	1.11	0.146	3.0E-01
Mtr.40816.1.S1_at	3.90	3.23	5.11	4.19	3.92	3.72	4.08	0.95	3.94	0.23	0.97	-0.049	8.2E-01
Mtr.14828.1.S1_at	9.28	8.87	10.69	10.30	10.47	10.12	9.61	0.95	10.30	0.17	1.07	0.099	2.9E-01
Mtr.1636.1.S1_a_at	7.47	5.97	7.74	8.04	7.87	8.10	7.06	0.95	8.00	0.12	1.13	0.181	1.6E-01
Mtr.33395.1.S1_at	5.28	3.44	4.81	5.78	5.75	5.75	4.51	0.95	5.76	0.02	1.28	0.353	8.6E-02
Mtr.12086.1.S1_at	5.96	4.26	5.86	5.49	5.48	5.80	5.36	0.95	5.59	0.18	1.04	0.061	7.0E-01
Mtr.23663.1.S1_at	10.33	8.79	10.53	11.10	10.19	10.63	9.88	0.95	10.64	0.46	1.08	0.106	2.8E-01
Mtr.1861.1.S1_at	6.57	5.02	6.76	5.55	6.70	6.08	6.12	0.95	6.11	0.57	1.00	-0.002	9.9E-01
Mtr.13559.1.S1_s_at	7.73	6.37	8.21	8.38	9.50	9.37	7.44	0.95	9.08	0.61	1.22	0.289	6.5E-02
Mtr.25847.1.S1_at	6.51	7.93	8.32	7.98	7.90	7.50	7.59	0.95	7.79	0.25	1.03	0.038	7.4E-01
Mtr.27551.1.S1_at	2.62	2.53	4.22	2.68	3.85	3.10	3.12	0.95	3.21	0.59	1.03	0.042	8.9E-01
Mtr.2708.1.S1_s_at	8.36	6.52	7.86	7.61	7.38	7.75	7.58	0.95	7.58	0.19	1.00	0.000	1.0E+00
Mtr.9481.1.S1_at	7.33	5.80	7.56	7.41	7.40	7.38	6.90	0.95	7.39	0.01	1.07	0.101	4.2E-01
Mtr.41524.1.S1_at	10.59	8.86	10.42	10.11	9.81	10.05	9.96	0.95	9.99	0.16	1.00	0.005	9.6E-01
Mtr.43897.1.S1_at	4.23	3.25	2.33	3.68	4.94	3.89	3.27	0.95	4.17	0.68	1.27	0.350	2.5E-01
Mtr.48631.1.S1_at	11.39	9.67	9.82	10.29	9.62	10.30	10.29	0.95	10.07	0.39	0.98	-0.031	7.3E-01
Mtr.36309.1.S1_at	4.75	4.31	6.14	5.60	6.24	5.84	5.07	0.95	5.89	0.32	1.16	0.218	2.3E-01
Mtr.14012.1.S1_at	7.56	5.76	7.19	7.75	7.53	7.75	6.84	0.95	7.68	0.13	1.12	0.167	2.1E-01
Mtr.9698.1.S1_at	4.04	2.47	4.19	3.61	3.56	3.55	3.57	0.95	3.57	0.03	1.00	0.002	9.9E-01
Mtr.44312.1.S1_at	9.10	7.24	7.82	8.26	8.22	8.13	8.05	0.95	8.20	0.06	1.02	0.027	8.0E-01
Mtr.43151.1.S1_at	3.93	2.33	2.23	4.57	5.51	4.05	2.83	0.95	4.71	0.74	1.66	0.735	5.4E-02
Mtr.31074.1.S1_s_at	9.07	7.17	8.22	8.74	8.41	8.83	8.15	0.95	8.66	0.22	1.06	0.087	4.2E-01
Mtr.16229.1.S1_at	8.73	7.07	8.70	9.18	9.09	9.10	8.17	0.95	9.12	0.05	1.12	0.160	1.6E-01
Mtr.29515.1.S1_at	5.84	4.12	5.68	5.81	6.21	6.00	5.22	0.95	6.00	0.20	1.15	0.203	2.3E-01
Mtr.38596.1.S1_at	12.18	10.31	10.94	11.52	10.96	11.74	11.14	0.95	11.41	0.40	1.02	0.034	6.8E-01
Mtr.23447.1.S1_at	4.81	3.04	4.51	4.80	4.86	5.04	4.12	0.95	4.90	0.12	1.19	0.250	2.3E-01
Mtr.6352.1.S1_s_at	9.71	7.92	8.27	8.48	8.48	8.56	8.63	0.95	8.51	0.05	0.99	-0.021	8.3E-01
Mtr.25952.1.S1_at	2.51	4.13	2.46	2.45	2.42	2.44	3.04	0.95	2.44	0.02	0.80	-0.316	3.4E-01
Mtr.37685.1.S1_at	6.98	5.30	6.90	7.10	7.40	7.26	6.39	0.95	7.25	0.15	1.13	0.182	2.0E-01
Mtr.34174.1.S1_at	8.88	7.02	8.28	8.52	8.31	8.41	8.06	0.95	8.42	0.11	1.04	0.063	5.5E-01
Mtr.49306.1.S1_at	5.59	7.49	6.52	8.46	8.47	8.57	6.54	0.95	8.50	0.06	1.30	0.379	2.3E-02
Mtr.45535.1.S1_at	3.52	2.50	4.40	2.95	3.09	2.75	3.47	0.95	2.93	0.17	0.84	-0.246	3.8E-01
Mtr.996.1.S1_s_at	3.60	5.20	3.51	3.47	3.31	3.33	4.10	0.95	3.37	0.09	0.82	-0.282	2.6E-01
Mtr.44852.1.S1_at	7.18	5.74	7.54	7.04	7.10	7.30	6.82	0.95	7.15	0.14	1.05	0.068	5.9E-01
Mtr.52180.1.S1_at	7.91	9.13	9.78	11.55	12.33	11.86	8.94	0.95	11.91	0.39	1.33	0.414	7.4E-03
Mtr.38878.1.S1_at	5.67	7.48	7.05	8.42	9.02	8.44	6.73	0.95	8.63	0.34	1.28	0.358	3.1E-02
Mtr.35907.1.S1_at	4.51	3.18	5.02	3.93	4.95	3.88	4.23	0.95	4.25	0.60	1.00	0.007	9.8E-01
Mtr.42014.1.S1_at	7.10	5.58	7.32	7.35	7.22	7.13	6.67	0.95	7.23	0.11	1.08	0.118	3.6E-01
Mtr.36454.1.S1_at	6.99	5.24	5.50	5.41	5.55	6.02	5.91	0.95	5.66	0.32	0.96	-0.062	6.9E-01
Mtr.39993.1.S1_at	5.92	4.25	5.85	6.26	5.60	5.95	5.34	0.95	5.94	0.33	1.11	0.153	3.6E-01
Mtr.49931.1.S1_at	9.53	7.65	8.78	8.87	8.98	9.15	8.65	0.95	9.00	0.14	1.04	0.057	5.6E-01
Mtr.50720.1.S1_s_at	10.11	8.22	9.20	9.61	9.39	9.48	9.17	0.95	9.49	0.11	1.03	0.049	5.9E-01
Mtr.1886.1.S1_at	7.93	6.39	8.11	8.37	8.20	8.40	7.48	0.95	8.32	0.11	1.11	0.155	2.0E-01
Mtr.9983.1.S1_at	7.70	5.85	6.44	6.81	7.22	7.16	6.67	0.95	7.07	0.22	1.06	0.084	5.2E-01
Mtr.39859.1.S1_at	5.50	3.98	5.72	5.42	5.16	5.54	5.07	0.95	5.37	0.19	1.06	0.085	6.1E-01
Mtr.14524.1.S1_at	8.82	7.31	9.06	8.59	8.38	8.47	8.40	0.95	8.48	0.11	1.01	0.014	8.9E-01
Mtr.31489.1.S1_at	8.24	6.59	8.22	8.57	8.08	8.42	7.68	0.95	8.36	0.25	1.09	0.122	3.0E-01
Mtr.14236.1.S1_at	5.17	3.63	5.36	5.14	5.18	4.98	4.72	0.95	5.10	0.10	1.08	0.113	5.2E-01
Mtr.14880.1.S1_at	2.49	4.12	2.48	2.46	2.51	2.45	3.03	0.95	2.47	0.03	0.82	-0.295	3.6E-01
Mtr.38783.1.S1_at	7.75	6.20	7.91	7.78	7.76	7.66	7.28	0.95	7.73	0.06	1.06	0.086	4.6E-01
Mtr.9804.1.S1_at	4.82	3.27	4.98	5.06	4.85	5.24	4.35	0.95	5.05	0.20	1.16	0.214	2.8E-01
Mtr.39276.1.S1_at	7.70	6.16	7.89	7.94	7.73	7.62	7.25	0.95	7.76	0.16	1.07	0.099	4.1E-01
Mtr.10086.1.S1_at	7.45	6.04	7.84	7.34	7.73	7.38	7.11	0.95	7.48	0.22	1.05	0.074	5.4E-01
Mtr.7834.1.S1_at	4.32	2.86	4.64	4.42	4.45	4.79	3.94	0.95	4.55	0.21	1.16	0.209	3.3E-01
Mtr.43009.1.S1_at	11.76	9.94	11.32	11.57	11.71	11.87	11.01	0.95	11.72	0.15	1.06	0.090	2.7E-01
Mtr.46674.1.S1_at	8.11	6.22	7.27	7.87	7.89	8.03	7.20	0.95	7.93	0.09	1.10	0.139	2.6E-01
Mtr.18110.1.S1_s_at	7.30	5.55	7.04	6.83	6.63	6.80	6.63	0.95	6.75	0.10	1.02	0.027	8.3E-01
Mtr.39537.1.S1_at	7.56	6.34	8.20	7.52	7.58	7.35	7.37	0.95	7.48	0.12	1.02	0.023	8.4E-01
Mtr.43554.1.S1_s_at	10.87	9.17	10.75	11.22	11.13	11.34	10.26	0.95	11.23	0.11	1.09	0.130	1.5E-01
Mtr.21625.1.S1_at	7.57	6.00	7.69	7.59	7.62	7.76	7.09	0.95	7.66	0.09	1.08	0.112	3.6E-01
Mtr.25546.1.S1_at	9.35	8.23	10.11	9.81	9.51	9.78	9.23	0.94	9.70	0.17	1.05	0.072	4.4E-01
Mtr.10716.1.S1_at	4.27	4.17	5.85	7.03	7.35	7.09	4.76	0.94	7.16	0.17	1.50	0.588	1.2E-02
Mtr.11698.1.S1_at	5.60	3.76	4.31	4.31	4.70	4.32	4.56	0.94	4.44	0.23	0.97	-0.038	8.4E-01
Mtr.23611.1.S1_at	7.02	5.54	7.29	6.91	7.36	6.93	6.61	0.94	7.07	0.25	1.07	0.096	4.7E-01
Mtr.35520.1.S1_at	9.66	7.79	8.93	9.09	9.03	9.42	8.80	0.94	9.18	0.21	1.04	0.062	5.3E-01
Mtr.47336.1.S1_at	2.75	4.39	2.76	2.74	2.74	2.72	3.30	0.94	2.73	0.01	0.83	-0.273	3.6E-01
Mtr.27499.1.S1_at	3.28	3.40	4.97	3.25	3.24	3.23	3.88	0.94	3.24	0.01	0.83	-0.260	3.0E-01
Mtr.39591.1.S1_at	6.54	5.75	7.63	7.20	7.55	7.65	6.64	0.94	7.47	0.23	1.12	0.168	2.2E-01
Mtr.1810.1.S1_at	6.74	4.86	5.99	6.38	6.26	6.45	5.86	0.94	6.36	0.09	1.08	0.118	4.1E-01
Mtr.46174.1.S1_at	8.73	6.88	8.16	9.21	9.30	9.31	7.92	0.94	9.27	0.05	1.17	0.227	6.9E-02
Mtr.41407.1.S1_at	9.57	7.74	9.05	9.43	9.75	9.56	8.79	0.94	9.58	0.16	1.09	0.125	2.2E-01
Mtr.27707.1.S1_at	6.76	5.05	6.59	7.05	7.72	7.01	6.13	0.94	7.26	0.40	1.18	0.243	1.3E-01
Mtr.11110.1.S1_at	7.13	5.27	6.48	6.35	5.99	6.08	6.29	0.94	6.14	0.19	0.98	-0.035	8.0E-01
Mtr.11406.1.S1_at	8.26	6.55	8.10	8.21	7.79	8.14	7.64	0.94	8.04	0.22	1.05	0.075	5.1E-01
Mtr.25486.1.S1_at	3.05	4.66	3.00	3.00	2.96	2.95	3.57	0.94	2.97	0.03	0.83	-0.265	3.3E-01
Mtr.34921.1.S1_at	7.37	5.80	7.49	7.45	7.31	7.15	6.89	0.94	7.30	0.15	1.06	0.085	4.9E-01
Mtr.7309.1.S1_at	9.94	8.06	9.13	9.31	9.00	9.13	9.04	0.94	9.14	0.16	1.01	0.016	8.6E-01
Mtr.24655.1.S1_at	2.86	4.46	2.81	2.80	2.78	2.79	3.38	0.94	2.79	0.01	0.83	-0.274	3.4E-01
Mtr.6861.1.S1_s_at	5.28	5.06	6.79	6.24	5.72	5.26	5.71	0.94	5.74	0.49	1.00	0.007	9.7E-01
Mtr.38111.1.S1_at	12.49	10.64	11.26	11.46	11.32	11.72	11.46	0.94	11.50	0.20	1.00	0.004	9.5E-01
Mtr.1857.1.S1_at	7.26	5.38	6.19	6.80	6.75	6.75	6.28	0.94	6.77	0.03	1.08	0.110	4.1E-01
Mtr.32609.1.S1_at	7.22	5.42	6.81	6.71	6.78	7.01	6.48	0.94	6.83	0.16	1.05	0.076	5.6E-01
Mtr.29707.1.S1_at	6.10	4.47	6.10	5.74	5.73	5.35	5.56	0.94	5.60	0.22	1.01	0.013	9.3E-01
Mtr.49336.1.S1_x_at	2.35	3.97	2.32	2.50	2.32	2.31	2.88	0.94	2.38	0.11	0.83	-0.275	4.1E-01

Mtr.35622.1.S1_at	9.24	7.47	8.91	8.54	8.27	8.26	8.54	0.94	8.36	0.16	0.98	-0.031	7.6E-01
Mtr.50387.1.S1_at	3.14	4.79	3.18	4.16	5.66	4.54	3.70	0.94	4.79	0.78	1.29	0.371	2.0E-01
Mtr.5625.1.S1_at	6.07	4.57	6.30	6.48	6.22	6.40	5.64	0.94	6.37	0.13	1.13	0.174	2.6E-01
Mtr.50585.1.S1_at	12.68	13.08	11.28	12.89	13.01	13.11	12.35	0.94	13.00	0.11	1.05	0.075	3.0E-01
Mtr.37062.1.S1_at	4.31	2.43	3.46	3.39	3.43	4.30	3.40	0.94	3.71	0.51	1.09	0.124	6.5E-01
Mtr.3265.1.S1_at	7.52	6.61	5.64	4.92	5.05	5.21	6.59	0.94	5.06	0.15	0.77	-0.380	5.0E-02
Mtr.48516.1.S1_at	7.52	6.30	8.15	7.57	7.50	7.51	7.32	0.94	7.53	0.04	1.03	0.040	7.3E-01
Mtr.44723.1.S1_at	9.37	7.69	9.26	9.11	9.03	9.08	8.77	0.94	9.08	0.04	1.03	0.049	6.1E-01
Mtr.5478.1.S1_at	9.33	8.28	7.45	8.74	8.43	8.60	8.35	0.94	8.59	0.15	1.03	0.040	6.9E-01
Mtr.41927.1.S1_at	7.52	5.94	7.60	7.47	7.54	7.54	7.02	0.94	7.52	0.04	1.07	0.098	4.1E-01
Mtr.10141.1.S1_at	8.16	9.86	9.72	9.68	9.55	9.62	9.25	0.94	9.62	0.06	1.04	0.057	5.3E-01
Mtr.2630.1.S1_at	2.55	1.76	3.64	2.93	2.54	3.00	2.65	0.94	2.82	0.25	1.06	0.090	7.8E-01
Mtr.46287.1.S1_at	3.91	4.01	5.59	3.86	3.86	3.64	4.50	0.94	3.79	0.13	0.84	-0.249	2.6E-01
Mtr.10679.1.S1_at	7.91	6.49	8.26	7.85	7.87	7.68	7.55	0.94	7.80	0.10	1.03	0.047	6.7E-01
Mtr.17226.1.S1_at	5.38	7.19	5.83	5.35	6.07	5.69	6.13	0.94	5.70	0.36	0.93	-0.105	5.0E-01
Mtr.28293.1.S1_s_at	8.38	7.21	9.08	9.57	9.73	9.56	8.22	0.94	9.62	0.09	1.17	0.227	6.2E-02
Mtr.18291.1.S1_at	6.33	5.08	6.92	6.44	6.40	6.54	6.11	0.94	6.46	0.07	1.06	0.080	5.5E-01
Mtr.45788.1.S1_at	8.42	7.01	8.80	8.53	8.66	8.22	8.07	0.94	8.47	0.22	1.05	0.069	5.2E-01
Mtr.25209.1.S1_x_at	5.34	3.74	3.68	4.20	4.27	5.20	4.25	0.94	4.56	0.56	1.07	0.099	6.6E-01
Mtr.48151.1.S1_at	2.59	4.21	2.58	2.57	2.57	2.56	3.13	0.94	2.57	0.01	0.82	-0.285	3.6E-01
Mtr.39518.1.S1_at	6.48	5.44	7.31	6.70	6.62	6.70	6.41	0.94	6.67	0.05	1.04	0.058	6.5E-01
Mtr.44719.1.S1_at	7.49	5.64	6.86	7.72	7.45	7.33	6.66	0.94	7.50	0.20	1.13	0.171	2.0E-01
Mtr.18567.1.S1_at	5.26	3.50	4.95	4.95	4.74	4.61	4.57	0.94	4.77	0.17	1.04	0.062	7.3E-01
Mtr.29156.1.S1_s_at	5.46	4.48	6.36	6.21	6.72	6.21	5.44	0.94	6.38	0.29	1.17	0.231	1.7E-01
Mtr.21118.1.S1_at	4.75	3.03	3.24	2.85	2.84	2.83	3.67	0.94	2.84	0.01	0.77	-0.372	2.0E-01
Mtr.44827.1.S1_at	6.46	6.34	8.02	7.11	7.27	7.04	6.94	0.94	7.14	0.12	1.03	0.041	7.3E-01
Mtr.10187.1.S1_at	10.46	11.86	12.24	10.75	11.39	10.87	11.52	0.94	11.00	0.34	0.96	-0.066	4.2E-01
Mtr.27305.1.S1_at	3.94	2.12	3.44	3.68	3.27	3.25	3.16	0.94	3.40	0.25	1.07	0.104	6.9E-01
Mtr.45093.1.S1_at	7.49	5.68	6.14	6.39	6.42	6.44	6.44	0.94	6.42	0.03	1.00	-0.004	9.8E-01
Mtr.8757.1.S1_at	11.53	10.98	9.70	10.71	11.03	11.23	10.74	0.94	10.99	0.26	1.02	0.033	6.8E-01
Mtr.41829.1.S1_at	8.15	6.35	7.71	7.89	7.70	7.67	7.40	0.94	7.75	0.12	1.05	0.067	5.5E-01
Mtr.34867.1.S1_s_at	9.31	8.42	10.29	9.83	10.50	9.74	9.34	0.94	10.02	0.42	1.07	0.102	3.1E-01
Mtr.14426.1.S1_at	6.84	5.18	5.26	5.12	4.42	5.01	5.76	0.94	4.85	0.37	0.84	-0.248	1.9E-01
Mtr.27291.1.S1_at	3.13	4.74	3.11	3.24	3.11	2.41	3.66	0.94	2.92	0.44	0.80	-0.326	2.8E-01
Mtr.51499.1.S1_at	6.45	4.95	6.68	6.95	6.54	6.53	6.03	0.94	6.68	0.24	1.11	0.148	3.1E-01
Mtr.36316.1.S1_at	8.14	6.69	8.44	7.86	8.26	7.87	7.76	0.94	8.00	0.23	1.03	0.044	6.9E-01
Mtr.22853.1.S1_at	2.91	4.51	2.86	2.85	2.81	2.80	3.43	0.94	2.82	0.03	0.82	-0.281	3.3E-01
Mtr.29874.1.S1_at	5.90	4.26	5.86	4.97	6.00	5.53	5.34	0.94	5.50	0.52	1.03	0.044	8.0E-01
Mtr.37453.1.S1_at	10.46	9.10	10.90	11.03	10.47	10.81	10.16	0.94	10.77	0.28	1.06	0.085	3.4E-01
Mtr.15074.1.S1_at	2.78	4.39	2.75	2.73	2.72	2.73	3.31	0.94	2.73	0.01	0.82	-0.280	3.4E-01
Mtr.2329.1.S1_at	4.71	3.58	5.44	5.07	5.77	5.10	4.58	0.94	5.31	0.39	1.16	0.215	2.8E-01
Mtr.3650.1.S1_at	2.16	3.77	2.15	2.14	2.14	2.14	2.69	0.94	2.14	0.00	0.79	-0.331	3.6E-01
Mtr.30703.1.S1_at	6.66	4.89	6.31	7.97	7.90	7.75	5.95	0.94	7.87	0.11	1.32	0.403	2.4E-02
Mtr.40393.1.S1_s_at	11.47	10.54	9.60	11.21	10.93	11.59	10.54	0.94	11.24	0.33	1.07	0.094	2.9E-01
Mtr.38805.1.S1_at	5.69	5.10	6.93	6.81	7.71	6.81	5.90	0.94	7.11	0.52	1.20	0.268	1.2E-01
Mtr.15801.1.S1_at	3.04	4.63	2.98	2.96	2.98	2.88	3.55	0.94	2.94	0.05	0.83	-0.274	3.2E-01
Mtr.17345.1.S1_at	3.91	2.47	4.23	3.90	4.18	3.99	3.54	0.94	4.02	0.15	1.14	0.186	4.2E-01
Mtr.2814.1.S1_at	9.41	7.60	8.89	9.03	9.39	9.17	8.63	0.93	9.20	0.18	1.07	0.091	3.6E-01
Mtr.22618.1.S1_at	6.89	5.94	7.81	7.54	7.39	7.34	6.88	0.93	7.42	0.10	1.08	0.109	3.8E-01
Mtr.10845.1.S1_at	9.70	11.32	9.69	9.07	9.28	9.47	10.24	0.93	9.27	0.20	0.91	-0.143	1.6E-01
Mtr.45385.1.S1_at	8.19	6.63	8.30	8.13	8.16	7.89	7.71	0.93	8.06	0.15	1.05	0.064	5.5E-01
Mtr.36593.1.S1_at	4.85	6.30	4.56	5.09	4.84	4.79	5.24	0.93	4.91	0.16	0.94	-0.094	5.8E-01
Mtr.20976.1.S1_at	3.64	5.16	5.34	5.29	5.29	4.11	4.71	0.93	4.90	0.69	1.04	0.056	8.0E-01
Mtr.10873.1.S1_at	12.69	11.11	12.77	12.92	12.96	12.82	12.19	0.93	12.90	0.07	1.06	0.082	2.6E-01
Mtr.19269.1.S1_at	6.57	4.73	5.40	4.75	4.57	4.76	5.57	0.93	4.69	0.10	0.84	-0.246	1.8E-01
Mtr.44454.1.S1_at	7.39	5.80	7.44	7.40	7.43	7.37	6.87	0.93	7.40	0.03	1.08	0.107	3.8E-01
Mtr.12945.1.S1_at	7.41	5.68	7.16	7.21	7.41	7.39	6.75	0.93	7.34	0.11	1.09	0.120	3.4E-01
Mtr.5152.1.S1_at	7.88	6.23	7.81	8.00	7.86	7.89	7.30	0.93	7.91	0.07	1.08	0.116	3.2E-01
Mtr.40522.1.S1_at	10.13	9.03	10.88	9.81	9.61	9.45	10.01	0.93	9.62	0.18	0.96	-0.057	5.2E-01
Mtr.11293.1.S1_at	9.33	10.81	9.08	9.68	10.56	10.17	9.74	0.93	10.13	0.44	1.04	0.057	5.4E-01
Mtr.31471.1.S1_at	8.26	6.47	7.84	7.43	7.74	7.24	7.52	0.93	7.47	0.25	0.99	-0.009	9.4E-01
Mtr.26909.1.S1_at	5.60	4.54	6.40	5.21	6.17	5.48	5.52	0.93	5.62	0.49	1.02	0.027	8.7E-01
Mtr.26369.1.S1_at	8.52	6.97	8.65	8.75	8.52	8.64	8.05	0.93	8.63	0.11	1.07	0.101	3.4E-01
Mtr.48426.1.S1_at	2.58	4.19	2.57	2.56	2.56	2.56	3.11	0.93	2.56	0.00	0.82	-0.281	3.6E-01
Mtr.29076.1.S1_at	4.40	2.83	4.48	4.36	4.41	4.20	3.90	0.93	4.32	0.11	1.11	0.147	4.8E-01
Mtr.10111.1.S1_at	6.45	5.00	6.74	6.44	7.00	6.39	6.06	0.93	6.61	0.34	1.09	0.124	3.9E-01
Mtr.38320.1.S1_at	10.32	8.46	9.32	9.77	9.71	9.67	9.37	0.93	9.72	0.05	1.04	0.053	5.5E-01
Mtr.8367.1.S1_s_at	3.77	2.28	2.05	3.45	4.52	4.34	2.70	0.93	4.10	0.58	1.52	0.604	9.1E-02
Mtr.27212.1.S1_at	7.38	5.68	7.20	7.17	7.10	7.17	6.75	0.93	7.15	0.04	1.06	0.082	5.0E-01
Mtr.6497.1.S1_at	6.13	4.64	6.35	6.02	6.10	5.94	5.70	0.93	6.02	0.08	1.06	0.077	5.9E-01
Mtr.43189.1.S1_at	8.47	8.38	6.81	7.35	8.26	7.99	7.89	0.93	7.86	0.47	1.00	-0.005	9.7E-01
Mtr.5570.1.S1_at	6.36	4.51	5.60	5.35	5.71	5.38	5.49	0.93	5.48	0.20	1.00	-0.003	9.9E-01
Mtr.5585.1.S1_at	8.63	6.84	8.20	8.33	8.59	8.40	7.89	0.93	8.44	0.13	1.07	0.097	3.7E-01
Mtr.28525.1.S1_at	6.82	5.32	7.02	6.80	6.81	6.90	6.39	0.93	6.83	0.05	1.07	0.098	4.5E-01
Mtr.21630.1.S1_at	4.61	3.41	5.24	4.77	4.61	4.76	4.42	0.93	4.71	0.09	1.07	0.093	6.2E-01
Mtr.37377.1.S1_at	7.01	8.87	7.94	8.04	8.11	7.67	7.94	0.93	7.94	0.24	1.00	0.001	1.0E+00
Mtr.19576.1.S1_at	6.99	8.44	6.71	6.88	6.83	6.80	7.38	0.93	6.84	0.04	0.93	-0.111	3.7E-01
Mtr.35113.1.S1_s_at	4.40	2.90	4.61	4.63	5.15	3.64	3.97	0.93	4.47	0.77	1.13	0.172	5.1E-01
Mtr.16753.1.S1_at	2.79	4.38	2.75	2.76	2.72	2.70	3.31	0.93	2.72	0.03	0.82	-0.280	3.4E-01
Mtr.35832.1.S1_at	5.54	3.84	5.35	4.72	5.57	4.77	4.91	0.93	5.02	0.48	1.02	0.032	8.7E-01
Mtr.34743.1.S1_at	7.68	6.19	7.90	7.77	7.64	7.55	7.26	0.93	7.65	0.12	1.05	0.076	5.1E-01
Mtr.6363.1.S1_at	5.53	3.76	5.16	5.77	6.54	6.47	4.82	0.93	6.26	0.42	1.30	0.378	7.1E-02
Mtr.5273.1.S1_at	8.42	6.79	8.38	8.37	8.29	8.39	7.86	0.93	8.35	0.05	1.06	0.086	4.2E-01
Mtr.8362.1.S1_at	8.10	7.09	8.95	8.35	8.58	8.13	8.05	0.93	8.35	0.23	1.04	0.054	6.1E-01
Mtr.38595.1.S1_at	6.46	5.92	7.73	7.24	7.10	6.55	6.71	0.93	6.97	0.37	1.04	0.055	6.7E-01
Mtr.27278.1.S1_at	8.02	6.16	7.11	7.88	8.25	8.12	7.10	0.93	8.08	0.19	1.14	0.188	1.5E-01
Mtr.40245.1.S1_s_at	6.57	8.37	7.86	5.15	6.62	6.09	7.60	0.93	5.95	0.75	0.78	-0.352	7.5E-02
Mtr.6049.1.S1_s_at	8.36	6.61	6.93	6.81	7.00	7.22	7.30	0.93	7.01	0.20	0.96	-0.059	6.2E-01
Mtr.34334.1.S1_at	2.52	4.22	2.72	3.38	2.51	2.71	3.15	0.93	2.87	0.45	0.91	-0.136	6.6E-01
Mtr.28044.1.S1_at	6.01	4.38	5.97	6.25	6.56	6.21	5.45	0.93	6.34	0.19	1.16	0.217	1.8E-01
Mtr.11731.1.S1_at	3.38	2.79	4.61	4.63	4.92	4.61	3.59	0.93	4.72	0.17	1.31	0.393	1.1E-01
Mtr.12613.1.S1_at	3.41	5.24	4.61	5.13	5.41	4.93	4.42	0.93	5.15	0.24	1.17	0.223	2.5E-01
Mtr.7025.1.S1_at	7.48	6.22	8.04	8.37	8.59	8.28	7.25	0.93	8.41	0.16	1.16	0.215	9.9E-02
Mtr.13282.1.S1_at	6.50	4.70	5.99	6.43	6.35	6.36	5.73	0.93	6.38	0.04	1.11	0.155	2.9E-01
Mtr.48412.1.S1_at	2.67	4.30	2.71	2.69	2.65	2.60	3.23	0.93	2.65	0.05	0.82	-0.287	3.4E-01
Mtr.52056.1.S1_at	4.19	3.98	5.68	4.44	4.89	4.48	4.62	0.93	4.60	0.25	1.00	-0.004	9.8E-01
Mtr.34794.1.S1_at	7.07	5.42	6.98	7.01	6.56	6.94	6.49	0.93	6.84	0.24	1.05	0.076	5.6E-01
Mtr.38982.1.S1_at	8.49	6.81	8.35	8.49	8.57	8.72	7.88	0.93	8.59	0.12	1.09	0.124	2.6E-01
Mtr.27807.1.S1_s_at	8.03	6.45	8.10	7.99	7.89	8.20	7.53	0.93	8.03	0.16	1.07	0.093	4.1E-01
Mtr.8609.1.S1_at	7.28	6.21	8.06	7.47	7.25	7.26	7.18	0.93	7.33	0.12	1.02	0.029	8.0E-01
Mtr.33531.1.S1_at	3.71	5.34	3.75	3.74	3.55	3.74	4.26	0.93	3.68	0.11	0.86	-0.214	3.4E-01
Mtr.3395.1.S1_at	2.47	4.00	2.32	2.32	2.32	2.31	2.93	0.93	2.32	0.00	0.79	-0.340	3.1E-01
Mtr.42428.1.S1_at	6.28	4.97	6.76	6.83	6.89	6.72	6.01	0.93	6.82	0.09	1.13	0.183	2.1E-01
Mtr.31542.1.S1_s_at	3.01	4.56	2.89	3.11	3.16	3.43	3.49	0.93	3.23	0.17	0.93	-0.110	6.6E-01
Mtr.1584.1.S1_at	5.31	3.48	4.13	4.67	5.18	5.15	4.31	0.93	5.00	0.28	1.16	0.214	2.9E-01
Mtr.35496.1.S1_at	9.77	8.47	10.27	9.88	10.04	9.75	9.50	0.93	9.89	0.14	1.04	0.057	5.2E-01
Mtr.43522.1.S1_at	5.89	4.07	5.30	4.79	5.31	4.67	5.09	0.93	4.92	0.34	0.97	-0.048	7.9E-01
Mtr.17914.1.S1_at	7.00	5.41	5.38	5.81	5.76	5.82	5.93	0.93	5.79	0.03	0.98	-0.033	8.1E-01
Mtr.24598.1.S1_x_at	7.14	5.32	5.91	6.43	6.58	6.55	6.12	0.93	6.52	0.08	1.06	0.091	5.0E-01
Mtr.3515.1.S1_at	4.90	3.38	5.06	4.90	4.89	4.89	4.45	0.93	4.89	0.01	1.10	0.138	4.5E-01
Mtr.9572.1.S1_at	10.75	8.90	9.96	10.42	10.24	10.59	9.87	0.93	10.42	0.18	1.06	0.078	3.7E-01
Mtr.40884.1.S1_at	5.10	3.78	5.57	6.30	6.58	6.76	4.82	0.93	6.54	0.23	1.36	0.442	3.5E-02
Mtr.35650.1.S1_at	8.61	7.63	9.49	9.25	9.61	9.51	8.57	0.93	9.46	0.19	1.10	0.142	1.8E-01
Mtr.9696.1.S1_at	9.11	7.29	7.90	8.67	8.86	9.02	8.10	0.93	8.85	0.17	1.09	0.128	2.4E-01
Mtr.40450.1.S1_at	9.88	8.10	9.46	10.00	10.13	10.17	9.15	0.93	10.10	0.09	1.10	0.143	1.5E-01
Mtr.6560.1.S1_at	2.60	4.18	2.55	2.54	2.52	2.52	3.11	0.93	2.52	0.01	0.81	-0.300	3.4E-01
Mtr.28837.1.S1_at	5.44	3.96	5.68	5.65	5.18	5.74	5.03	0.93	5.52	0.30	1.10	0.136	4.3E-01
Mtr.40047.1.S1_s_at	8.53	7.87	6.70	8.33	7.06	8.00	7.70	0.93	7.80	0.66	1.01	0.019	8.9E-01
Mtr.43588.1.S1_at	7.24	5.39	6.19	7.24	6.82	6.85	6.27	0.93	6.97	0.23	1.11	0.152	2.8E-01
Mtr.19032.1.S1_x_at	5.72	4.36	6.12	5.86	6.53	5.85	5.40	0.93	6.08	0.39	1.13	0.171	3.1E-01
Mtr.43962.1.S1_at	8.85	7.01	7.76	7.91	7.70	7.84	7.87	0.93	7.82	0.10	0.99	-0.010	9.2E-01
Mtr.45526.1.S1_at	7.88	6.22	7.75	7.79	7.67	7.66	7.28	0.93	7.70	0.07	1.06	0.081	4.8E-01
Mtr.4879.1.S1_at	9.04	7.39	7.49	8.19	7.76	8.15	7.98	0.93	8.03	0.24	1.01	0.010	9.2E-01
Mtr.13291.1.S1_at	7.84	6.21	7.79	7.97	8.08	8.27	7.28	0.93	8.11	0.15	1.11	0.154	2.0E-01
Mtr.2657.1.S1_at	5.65	4.18	5.90	5.21	5.91	5.66	5.24	0.93	5.59	0.35	1.07	0.094	5.7E-01
Mtr.38636.1.S1_s_at	7.83	6.42	8.17	7.84	7.72	7.78	7.47	0.93	7.78	0.06	1.04	0.058	6.0E-01
Mtr.46475.1.S1_s_at	4.72	4.25	6.03	6.59	7.01	5.69	5.00	0.93	6.43	0.67	1.29	0.364	9.6E-02
Mtr.39088.1.S1_s_at	7.70	5.87	7.05	7.26	7.28	7.39	6.88	0.93	7.31	0.07	1.06	0.088	4.6E-01
Mtr.41996.1.S1_at	8.90	7.11	8.42	8.74	8.85	8.74	8.15	0.93	8.78	0.07	1.08	0.108	3.0E-01
Mtr.24820.1.S1_s_at	5.38	3.53	4.39	3.75	4.86	4.92	4.43	0.93	4.51	0.66	1.02	0.023	9.2E-01
Mtr.13921.1.S1_at	7.45	5.92	7.59	7.37	7.54	7.50	6.99	0.93	7.47	0.09	1.07	0.096	4.2E-01

Mtr.11197.1.S1_at	13.45	12.41	14.26	13.98	14.23	13.98	13.37	0.92	14.06	0.15	1.05	0.073	2.7E-01
Mtr.16499.1.S1_at	3.51	5.10	3.48	3.47	3.34	3.42	4.03	0.92	3.41	0.07	0.84	-0.243	3.1E-01
Mtr.11843.1.S1 at	4.70	3.09	4.67	4.95	4.77	4.50	4.15	0.92	4.74	0.23	1.14	0.190	3.5E-01
Mtr.43550.1.S1 at	11.97	11.12	10.12	10.64	11.02	10.98	11.07	0.92	10.88	0.21	0.98	-0.025	7.4E-01
Mtr.49304.1.S1_at	7.17	5.85	7.63	7.41	7.16	7.26	6.88	0.92	7.28	0.12	1.06	0.080	5.1E-01
Mtr.34792.1.S1_s_at	7.14	5.37	6.72	7.09	7.00	6.99	6.41	0.92	7.02	0.06	1.10	0.131	3.2E-01
Mtr.37010.1.S1_at	8.17	6.60	8.23	8.17	8.27	7.90	7.67	0.92	8.11	0.19	1.06	0.082	4.6E-01
Mtr.25144.1.S1 at	2.67	4.27	2.66	2.66	2.61	2.65	3.20	0.92	2.64	0.03	0.83	-0.275	3.6E-01
Mtr.27912.1.S1 at	3.75	2.59	4.42	3.91	4.07	3.73	3.59	0.92	3.91	0.17	1.09	0.124	5.9E-01
Mtr.41245.1.S1 at	9.79	8.42	10.19	10.31	10.07	10.08	9.47	0.92	10.15	0.13	1.07	0.101	2.7E-01
Mtr.4754.1.S1 at	5.67	3.82	4.70	3.34	4.17	4.09	4.73	0.92	3.87	0.45	0.82	-0.291	2.2E-01
Mtr.13933.1.S1_at	4.14	3.59	5.39	4.90	5.20	4.46	4.37	0.92	4.85	0.37	1.11	0.150	4.5E-01
Mtr.9840.1.S1_at	8.39	6.73	8.26	8.05	8.12	8.19	7.79	0.92	8.12	0.07	1.04	0.059	5.8E-01
Mtr.16525.1.S1_at	2.73	4.32	2.72	2.71	2.70	2.69	3.26	0.92	2.70	0.01	0.83	-0.269	3.6E-01
Mtr.25447.1.S1 at	7.84	6.69	8.52	7.66	7.45	7.31	7.68	0.92	7.47	0.17	0.97	-0.040	7.2E-01
Mtr.18269.1.S1 at	9.15	7.37	8.70	8.44	9.10	8.98	8.41	0.92	8.84	0.35	1.05	0.073	4.9E-01
Mtr.7838.1.S1 at	5.73	5.55	4.05	5.49	5.56	5.62	5.11	0.92	5.56	0.07	1.09	0.120	4.5E-01
Mtr.5137.1.S1_at	2.59	2.55	4.17	2.60	2.57	2.38	3.10	0.92	2.52	0.12	0.81	-0.300	3.4E-01
Mtr.24081.1.S1_at	4.59	4.58	6.18	4.82	4.80	4.90	5.12	0.92	4.84	0.06	0.95	-0.080	6.3E-01
Mtr.9437.1.S1_at	9.42	7.98	9.70	8.80	8.77	8.76	9.04	0.92	8.78	0.02	0.97	-0.042	6.5E-01
Mtr.33357.1.S1 at	6.21	4.53	6.04	5.64	5.66	5.62	5.59	0.92	5.64	0.02	1.01	0.012	9.4E-01
Mtr.7448.1.S1 s at	8.09	6.49	8.08	7.52	8.05	8.04	7.56	0.92	7.87	0.31	1.04	0.058	6.1E-01
Mtr.49948.1.S1 at	2.64	4.22	2.61	2.59	2.58	2.58	3.16	0.92	2.58	0.01	0.82	-0.290	3.4E-01
Mtr.24488.1.S1_s_at	4.77	3.54	5.34	5.46	5.98	5.39	4.55	0.92	5.61	0.32	1.23	0.302	1.3E-01
Mtr.34920.1.S1_at	2.30	3.89	2.29	2.28	2.28	2.28	2.82	0.92	2.28	0.00	0.81	-0.308	3.7E-01
Mtr.45381.1.S1_at	4.72	2.94	4.24	4.48	4.18	4.18	3.97	0.92	4.28	0.17	1.08	0.109	6.0E-01
Mtr.7939.1.S1 at	4.58	4.06	5.85	4.42	5.74	5.54	4.83	0.92	5.23	0.71	1.08	0.115	5.8E-01
Mtr.11004.1.S1 s at	9.70	7.86	8.70	8.98	8.85	9.03	8.75	0.92	8.96	0.10	1.02	0.033	7.2E-01
Mtr.28076.1.S1 at	6.35	5.08	6.87	6.81	6.51	6.26	6.10	0.92	6.53	0.28	1.07	0.098	4.8E-01
Mtr.9876.1.S1_at	10.38	9.59	8.54	10.10	9.50	10.42	9.50	0.92	10.00	0.46	1.05	0.074	4.5E-01
Mtr.28959.1.S1_at	3.75	5.31	3.68	3.68	3.63	3.73	4.25	0.92	3.68	0.05	0.87	-0.207	3.5E-01
Mtr.16543.1.S1_at	3.00	4.48	2.79	2.78	2.77	2.76	3.42	0.92	2.77	0.01	0.81	-0.305	2.9E-01
Mtr.30211.1.S1 at	3.81	5.41	3.82	3.79	3.51	3.78	4.35	0.92	3.69	0.16	0.85	-0.235	2.9E-01
Mtr.38526.1.S1 at	9.04	7.43	9.01	8.99	9.18	8.94	8.49	0.92	9.04	0.13	1.06	0.090	3.7E-01
Mtr.12715.1.S1 at	7.36	8.94	7.34	7.21	8.67	7.96	7.88	0.92	7.95	0.73	1.01	0.012	9.3E-01
Mtr.44573.1.S1 at	5.46	3.77	5.25	5.58	6.01	6.28	4.83	0.92	5.96	0.35	1.23	0.302	1.2E-01
Mtr.6966.1.S1_at	6.10	4.42	5.92	5.88	5.80	6.11	5.48	0.92	5.93	0.16	1.08	0.114	4.5E-01
Mtr.12044.1.S1 at	8.45	6.67	7.97	8.41	8.16	8.53	7.70	0.92	8.37	0.19	1.09	0.120	2.8E-01
Mtr.22579.1.S1 s at	9.27	7.81	9.51	9.53	9.64	9.57	8.86	0.92	9.58	0.06	1.08	0.112	2.5E-01
Mtr.44099.1.S1 at	10.03	8.20	8.91	8.89	9.00	8.90	9.05	0.92	8.93	0.06	0.99	-0.018	8.4E-01
Mtr.13901.1.S1 at	8.35	8.04	9.77	9.39	9.53	9.16	8.72	0.92	9.36	0.19	1.07	0.103	3.0E-01
Mtr.32312.1.S1_at	5.93	4.25	4.44	4.11	4.22	4.57	4.88	0.92	4.30	0.24	0.88	-0.181	3.6E-01
Mtr.2826.1.S1_at	2.76	4.35	2.75	2.75	2.69	2.69	3.28	0.92	2.71	0.03	0.82	-0.278	3.4E-01
Mtr.47954.1.S1 at	8.17	6.60	8.22	8.36	8.15	8.15	7.66	0.92	8.22	0.12	1.07	0.101	3.6E-01
Mtr.4036.1.S1 at	6.32	5.58	7.41	5.51	5.50	5.24	6.44	0.92	5.42	0.16	0.84	-0.249	1.3E-01
Mtr.32900.1.S1 at	5.71	4.74	6.58	6.44	6.81	6.40	5.68	0.92	6.55	0.23	1.15	0.207	1.9E-01
Mtr.41681.1.S1 at	3.58	2.56	4.39	3.42	3.46	3.35	3.51	0.92	3.41	0.06	0.97	-0.041	8.6E-01
Mtr.33745.1.S1_s_at	6.82	5.72	4.99	5.64	5.66	6.08	5.85	0.92	5.79	0.25	0.99	-0.013	9.3E-01
Mtr.13258.1.S1_at	8.41	6.92	8.60	8.84	8.82	8.99	7.98	0.92	8.88	0.09	1.11	0.155	1.6E-01
Mtr.15568.1.S1 s at	6.28	4.52	5.86	6.55	6.25	6.39	5.55	0.92	6.40	0.15	1.15	0.204	1.9E-01
Mtr.41796.1.S1 at	7.51	5.86	7.39	7.16	7.45	7.19	6.92	0.92	7.27	0.16	1.05	0.070	5.6E-01
Mtr.29312.1.S1 at	9.28	7.66	9.23	9.17	9.33	9.20	8.72	0.92	9.23	0.08	1.06	0.082	3.9E-01
Mtr.35247.1.S1 at	7.71	5.87	6.72	7.70	7.01	7.84	6.77	0.92	7.52	0.45	1.11	0.152	2.7E-01
Mtr.11998.1.S1_at	4.59	2.78	3.95	4.59	3.83	4.32	3.77	0.92	4.25	0.39	1.13	0.171	4.6E-01
Mtr.29567.1.S1_at	4.29	2.78	2.62	3.11	3.83	3.48	3.23	0.92	3.47	0.36	1.07	0.103	6.9E-01
Mtr.39006.1.S1_at	9.14	7.45	8.92	8.98	9.03	8.88	8.50	0.92	8.96	0.08	1.05	0.076	4.4E-01
Mtr.46485.1.S1 at	12.30	10.49	11.13	11.66	11.37	11.79	11.31	0.92	11.61	0.21	1.03	0.038	6.1E-01
Mtr.28309.1.S1 at	9.26	7.65	9.22	9.07	9.05	9.00	8.71	0.92	9.04	0.04	1.04	0.053	5.7E-01
Mtr.12237.1.S1 at	8.94	7.14	7.74	8.10	8.67	8.44	7.94	0.92	8.40	0.29	1.06	0.082	4.5E-01
Mtr.34540.1.S1_s_at	12.65	10.86	12.11	11.84	11.52	11.53	11.88	0.92	11.63	0.18	0.98	-0.030	6.7E-01
Mtr.10522.1.S1_s_at	7.54	9.28	8.91	9.23	8.95	9.18	8.58	0.92	9.12	0.15	1.06	0.089	3.7E-01
Mtr.11502.1.S1_at	6.93	5.17	6.51	6.34	6.50	6.49	6.20	0.92	6.45	0.09	1.04	0.055	6.7E-01
Mtr.16219.1.S1 s at	11.25	10.98	9.54	11.05	10.97	11.48	10.59	0.92	11.17	0.28	1.05	0.076	3.6E-01
Mtr.5831.1.S1 s at	8.84	7.11	8.51	8.75	8.87	8.86	8.15	0.92	8.82	0.07	1.08	0.115	2.7E-01
Mtr.34991.1.S1 at	5.89	4.44	6.14	6.10	6.35	5.79	5.49	0.92	6.08	0.28	1.11	0.148	3.4E-01
Mtr.6103.1.S1_at	5.32	3.63	3.87	4.73	3.89	4.03	4.27	0.92	4.22	0.45	0.99	-0.019	9.3E-01
Mtr.7438.1.S1_at	5.10	4.18	6.01	5.16	5.49	5.31	5.10	0.92	5.32	0.17	1.04	0.062	7.0E-01
Mtr.11518.1.S1_at	5.98	4.25	5.66	5.38	5.52	4.69	5.30	0.92	5.20	0.44	0.98	-0.027	8.8E-01
Mtr.44069.1.S1 at	8.13	9.85	9.54	9.26	9.32	9.63	9.17	0.92	9.40	0.20	1.02	0.035	7.0E-01
Mtr.29495.1.S1 at	5.14	5.68	6.93	5.82	6.22	5.28	5.92	0.92	5.77	0.47	0.98	-0.036	8.2E-01
Mtr.6318.1.S1 at	5.75	4.15	5.72	5.70	5.73	5.69	5.20	0.92	5.70	0.02	1.10	0.132	4.0E-01
Mtr.44199.1.S1_at	4.41	4.74	6.13	4.70	4.70	4.70	5.09	0.92	4.70	0.00	0.92	-0.115	5.0E-01
Mtr.19608.1.S1_at	3.03	4.74	3.32	2.93	2.93	2.99	3.70	0.92	2.95	0.03	0.80	-0.327	2.3E-01
Mtr.48428.1.S1 at	2.34	3.92	2.33	2.31	2.43	2.34	2.87	0.92	2.36	0.06	0.82	-0.280	3.9E-01
Mtr.24765.1.S1 at	8.06	6.32	7.70	7.77	7.20	7.43	7.36	0.92	7.47	0.29	1.01	0.021	8.5E-01
Mtr.1112.1.S1 at	2.23	3.89	2.40	2.61	2.42	2.39	2.84	0.92	2.47	0.12	0.87	-0.200	5.3E-01
Mtr.43130.1.S1 at	5.72	6.10	7.46	6.07	5.93	6.12	6.43	0.92	6.04	0.10	0.94	-0.090	5.1E-01
Mtr.39978.1.S1_at	5.85	4.47	6.21	6.85	7.04	7.10	5.51	0.92	7.00	0.13	1.27	0.344	5.0E-02
Mtr.49986.1.S1_x_at	2.93	4.49	2.88	2.88	2.86	2.88	3.43	0.92	2.87	0.01	0.84	-0.256	3.5E-01
Mtr.33634.1.S1_at	6.64	5.10	6.73	6.45	6.53	6.42	6.16	0.92	6.47	0.06	1.05	0.071	5.9E-01
Mtr.47390.1.S1 at	3.83	2.29	2.20	2.18	2.18	2.53	2.77	0.92	2.29	0.20	0.83	-0.274	4.3E-01
Mtr.46141.1.S1 s at	10.33	8.78	10.41	10.53	10.42	10.26	9.84	0.92	10.41	0.14	1.06	0.081	3.5E-01
Mtr.2029.1.S1 a at	8.81	7.18	8.72	8.78	9.11	8.81	8.24	0.92	8.90	0.18	1.08	0.112	2.9E-01
Mtr.13772.1.S1_at	3.16	4.74	3.15	2.78	3.14	3.00	3.68	0.92	2.97	0.19	0.81	-0.309	2.6E-01
Mtr.44554.1.S1_at	6.87	5.34	6.97	6.76	7.16	6.83	6.39	0.92	6.92	0.21	1.08	0.114	3.9E-01
Mtr.9877.1.S1_at	8.12	6.65	8.34	8.49	8.33	8.37	7.70	0.92	8.40	0.09	1.09	0.124	2.6E-01
Mtr.42537.1.S1 s at	7.64	6.01	7.54	7.67	7.65	7.87	7.06	0.92	7.73	0.12	1.09	0.130	2.8E-01
Mtr.11076.1.S1 at	4.78	3.41	5.15	5.25	5.88	5.38	4.45	0.92	5.50	0.33	1.24	0.308	1.3E-01
Mtr.35964.1.S1 at	3.28	4.75	3.06	3.27	3.04	3.19	3.70	0.92	3.17	0.12	0.86	-0.222	3.8E-01
Mtr.36445.1.S1_at	7.27	6.71	8.50	7.74	8.12	7.67	7.49	0.92	7.84	0.24	1.05	0.065	5.6E-01
Mtr.52161.1.S1_at	7.36	5.97	7.69	7.40	7.44	7.32	7.01	0.92	7.39	0.06	1.05	0.076	5.1E-01
Mtr.143.1.S1_at	6.24	5.09	6.89	7.43	7.55	7.33	6.07	0.92	7.43	0.11	1.22	0.292	6.3E-02
Mtr.40890.1.S1 at	9.69	8.33	10.07	10.24	10.81	10.53	9.36	0.92	10.52	0.28	1.12	0.168	1.0E-01
Mtr.15301.1.S1 at	4.67	3.41	5.20	5.05	4.44	4.99	4.43	0.92	4.83	0.33	1.09	0.124	5.2E-01
Mtr.44644.1.S1 s at	4.92	3.60	5.36	5.00	4.80	4.80	4.63	0.91	4.87	0.11	1.05	0.073	6.8E-01
Mtr.49754.1.S1_at	7.16	5.97	7.77	7.73	7.49	7.24	6.97	0.91	7.49	0.25	1.07	0.104	4.0E-01
Mtr.51089.1.S1_at	4.22	4.12	5.75	6.77	6.72	6.89	4.70	0.91	6.79	0.09	1.45	0.532	1.7E-02
Mtr.6825.1.S1_at	4.27	2.46	3.17	3.11	2.97	3.10	3.30	0.91	3.06	0.08	0.93	-0.110	6.7E-01
Mtr.12757.1.S1 at	9.52	7.76	9.05	8.80	9.12	8.65	8.78	0.91	8.86	0.24	1.01	0.013	8.9E-01
Mtr.36406.1.S1 at	4.80	3.72	5.54	4.35	5.57	5.33	4.69	0.91	5.08	0.65	1.08	0.117	5.7E-01
Mtr.13186.1.S1 at	9.97	11.79	10.73	11.11	11.15	10.63	10.83	0.91	10.96	0.29	1.01	0.017	8.3E-01
Mtr.20947.1.S1 x at	5.55	3.80	4.21	6.74	5.20	5.79	4.52	0.91	5.91	0.78	1.31	0.388	1.1E-01
Mtr.34160.1.S1_at	5.53	3.76	5.02	5.35	5.34	6.21	4.77	0.91	5.63	0.50	1.18	0.240	2.2E-01
Mtr.44674.1.S1 at	2.79	2.83	4.39	2.77	2.83	2.35	3.34	0.91	2.65	0.26	0.79	-0.333	2.8E-01
Mtr.7245.1.S1 at	2.52	2.60	4.14	2.50	2.51	2.49	3.08	0.91	2.50	0.01	0.81	-0.302	3.3E-01
Mtr.33824.1.S1 at	3.81	3.26	5.04	3.99	4.47	4.34	4.04	0.91	4.27	0.25	1.06	0.079	7.0E-01
Mtr.42361.1.S1 at	7.88	6.27	7.81	8.10	7.83	7.89	7.32	0.91	7.94	0.14	1.08	0.117	3.1E-01
Mtr.39871.1.S1_at	7.85	6.04	7.12	7.55	7.30	7.50	7.01	0.91	7.45	0.13	1.06	0.088	4.5E-01
Mtr.32041.1.S1_at	10.88	9.52	11.25	11.44	11.24	11.25	10.55	0.91	11.31	0.11	1.07	0.100	2.3E-01
Mtr.38005.1.S1 at	9.28	7.47	8.59	8.71	8.91	8.75	8.45	0.91	8.79	0.10	1.04	0.057	5.5E-01
Mtr.45416.1.S1 at	7.97	6.38	7.94	7.74	7.63	7.77	7.43	0.91	7.71	0.07	1.04	0.053	6.3E-01
Mtr.10045.1.S1 at	9.64	8.14	7.98	8.56	8.10	8.60	8.59	0.91	8.42	0.28	0.98	-0.028	7.8E-01
Mtr.28246.1.S1 at	6.99	5.27	6.67	7.08	7.03	7.01	6.31	0.91	7.04	0.04	1.12	0.158	2.4E-01
Mtr.51723.1.S1_at	6.17	7.83	6.35	6.15	6.51	6.16	6.78	0.91	6.27	0.20	0.93	-0.112	4.0E-01
Mtr.42283.1.S1_at	9.96	11.30	11.70	10.39	10.79	10.48	10.99	0.91	10.55	0.21	0.96	-0.058	4.6E-01
Mtr.10402.1.S1_at	11.45	9.66	10.84	10.58	10.10	10.28	10.65	0.91	10.32	0.25	0.97	-0.045	5.8E-01
Mtr.8397.1.S1 at	3.78	5.35	3.77	4.27	3.64	3.76	4.30	0.91	3.89	0.33	0.90	-0.146	5.0E-01
Mtr.40066.1.S1 at	13.99	12.78	12.21	12.43	12.00	12.39	12.99	0.91	12.27	0.24	0.94	-0.082	2.6E-01
Mtr.31555.1.S1 at	8.98	7.26	8.66	9.07	9.66	9.13	8.30	0.91	9.29	0.33	1.12	0.162	1.5E-01
Mtr.18700.1.S1_at	8.29	6.53	7.82	8.10	8.18	8.13	7.55	0.91	8.14	0.04	1.08	0.109	3.3E-01
Mtr.11398.1.S1_at	6.19	4.42	5.69	6.44	6.30	6.05	5.43	0.91	6.26	0.20	1.15	0.205	2.0E-01
Mtr.43094.1.S1_at	7.17	5.46	6.85	5.75	6.43	5.56	6.49	0.91	5.91	0.46	0.91	-0.135	3.8E-01
Mtr.14413.1.S1 at	10.37	12.15	10.93	10.03	10.15	9.96	11.15	0.91	10.05	0.10	0.90	-0.150	1.1E-01
Mtr.37253.1.S1 at	7.57	5.86	7.25	7.42	7.37	7.68	6.90	0.91	7.49	0.17	1.09	0.119	3.3E-01
Mtr.27129.1.S1 at	10.14	9.89	11.58	10.54	10.88	9.87	10.54	0.91	10.43	0.51	0.99	-0.015	8.6E-01
Mtr.39559.1.S1_at	8.08	6.43	7.92	8.37	8.32	8.47	7.48	0.91	8.39	0.07	1.12	0.166	1.6E-01
Mtr.33731.1.S1_s_at	7.38	5.94	7.63	7.26	7.28	7.20	6.98	0.91	7.25	0.04	1.04	0.053	6.4E-01
Mtr.25451.1.S1_s_at	7.85	9.41	9.45	9.47	9.49	9.42	8.91	0.91	9.46	0.04	1.06	0.087	3.5E-01
Mtr.38980.1.S1 at	7.53	6.35	8.14	7.91	7.57	7.94	7.34	0.91	7.81	0.21	1.06	0.089	4.4E-01
Mtr.38253.1.S1 at	2.60	2.88	4.30	4.38	3.15	3.88	3.26	0.91	3.80	0.62	1.17	0.223	4.4E-01
Mtr.35215.1.S1 at	7.83	6.15	7.59	7.64	7.63	7.46	7.19	0.91	7.58	0.10	1.05	0.076	5.0E-01

Mtr.11652.1.S1_at	8.00	6.24	7.51	7.73	7.92	8.34	7.25	0.91	7.99	0.31	1.10	0.141	2.5E-01
Mtr.49248.1.S1_at	2.68	4.21	2.59	2.60	2.55	2.54	3.16	0.91	2.56	0.03	0.81	-0.303	3.2E-01
Mtr.13593.1.S1_at	5.56	3.80	5.08	5.06	5.09	5.13	4.81	0.91	5.10	0.03	1.06	0.082	6.2E-01
Mtr.48994.1.S1_at	10.55	9.35	11.13	11.11	11.03	10.93	10.34	0.91	11.02	0.09	1.07	0.092	2.7E-01
Mtr.12280.1.S1_at	6.09	5.24	4.27	5.61	5.31	5.88	5.20	0.91	5.60	0.28	1.08	0.107	5.1E-01
Mtr.42770.1.S1_at	9.49	7.92	7.91	8.96	9.00	9.09	8.44	0.91	9.02	0.07	1.07	0.095	3.4E-01
Mtr.23693.1.S1_at	2.69	4.25	2.67	2.59	2.62	2.65	3.20	0.91	2.62	0.03	0.82	-0.288	3.3E-01
Mtr.51976.1.S1_at	11.12	9.76	11.49	11.83	12.25	11.93	10.79	0.91	12.00	0.22	1.11	0.154	8.8E-02
Mtr.47946.1.S1_at	5.08	3.28	4.42	4.20	3.91	3.36	4.26	0.91	3.83	0.43	0.90	-0.156	4.9E-01
Mtr.50940.1.S1_s_at	2.47	4.04	2.45	2.44	2.44	2.43	2.99	0.91	2.44	0.01	0.82	-0.292	3.6E-01
Mtr.13046.1.S1_at	10.63	9.33	8.88	9.82	9.67	9.90	9.61	0.91	9.80	0.12	1.02	0.028	7.4E-01
Mtr.21060.1.S1_at	3.26	3.12	4.76	3.62	4.20	3.08	3.71	0.91	3.63	0.56	0.98	-0.031	9.0E-01
Mtr.30611.1.S1_at	6.25	4.88	6.60	6.23	6.59	6.03	5.91	0.91	6.29	0.28	1.06	0.090	5.3E-01
Mtr.24082.1.S1_at	8.71	7.08	8.59	8.25	8.26	8.24	8.13	0.91	8.25	0.01	1.02	0.022	8.2E-01
Mtr.44653.1.S1_at	10.79	9.27	9.17	10.16	9.74	10.39	9.74	0.91	10.10	0.33	1.04	0.051	5.6E-01
Mtr.19748.1.S1_at	4.28	2.57	3.96	4.11	4.87	4.10	3.60	0.91	4.36	0.44	1.21	0.274	2.7E-01
AFFX-Mtr-gapc-3_s_at	13.05	11.23	12.10	12.15	12.40	12.55	12.13	0.91	12.37	0.20	1.02	0.028	6.8E-01
Mtr.50700.1.S1_s_at	5.07	3.58	5.22	5.46	4.96	5.97	4.62	0.91	5.47	0.51	1.18	0.242	2.3E-01
Mtr.26506.1.S1_at	4.86	3.19	4.65	3.30	4.52	3.61	4.24	0.91	3.81	0.64	0.90	-0.154	5.4E-01
Mtr.20517.1.S1_at	3.61	2.03	3.60	3.55	3.60	3.63	3.08	0.91	3.59	0.04	1.17	0.222	3.8E-01
Mtr.28901.1.S1_at	1.99	3.56	1.98	1.98	1.98	1.98	2.51	0.91	1.98	0.00	0.79	-0.342	3.7E-01
Mtr.33078.1.S1_x_at	7.19	8.79	7.24	7.43	6.98	7.31	7.74	0.91	7.24	0.23	0.94	-0.096	4.1E-01
Mtr.32629.1.S1_x_at	5.94	4.43	4.32	4.35	4.28	4.66	4.90	0.91	4.43	0.20	0.91	-0.144	4.4E-01
Mtr.40771.1.S1_at	11.48	9.91	9.90	10.20	9.75	10.37	10.43	0.91	10.11	0.32	0.97	-0.046	5.9E-01
Mtr.23266.1.S1_at	3.29	4.62	5.03	3.15	2.26	3.08	4.32	0.91	2.83	0.49	0.66	-0.607	6.8E-02
Mtr.37852.1.S1_at	3.78	2.68	4.48	7.27	7.90	8.09	3.65	0.91	7.75	0.43	2.12	1.087	2.1E-03
Mtr.49910.1.S1_at	9.19	7.78	9.48	9.62	9.77	9.42	8.82	0.91	9.60	0.18	1.09	0.123	2.2E-01
Mtr.1806.1.S1_s_at	3.42	2.52	4.33	3.40	3.92	3.41	3.42	0.91	3.58	0.30	1.04	0.062	8.0E-01
Mtr.20107.1.S1_at	11.03	9.56	11.22	11.41	11.86	11.63	10.61	0.91	11.63	0.23	1.10	0.134	1.3E-01
Mtr.7603.1.S1_at	7.14	5.66	7.31	7.46	7.50	7.36	6.70	0.91	7.44	0.07	1.11	0.150	2.4E-01
Mtr.9203.1.S1_s_at	8.93	7.14	8.26	8.47	8.46	8.70	8.11	0.91	8.54	0.14	1.05	0.075	4.6E-01
Mtr.47455.1.S1_at	2.39	3.96	2.38	2.38	2.38	2.37	2.91	0.91	2.38	0.00	0.82	-0.292	3.7E-01
Mtr.6737.1.S1_s_at	7.10	5.43	5.66	5.54	6.09	6.08	6.07	0.91	5.90	0.31	0.97	-0.039	7.8E-01
Mtr.12402.1.S1_s_at	9.56	7.84	8.21	9.16	10.21	9.80	8.54	0.91	9.72	0.53	1.14	0.187	1.2E-01
Mtr.15310.1.S1_at	6.68	5.53	7.32	6.98	7.04	7.13	6.51	0.91	7.05	0.08	1.08	0.114	3.7E-01
Mtr.18371.1.S1_at	4.08	5.63	5.66	4.32	5.03	3.86	5.12	0.91	4.40	0.59	0.86	-0.218	3.1E-01
Mtr.16786.1.S1_at	14.15	12.80	14.53	14.48	14.43	14.34	13.83	0.91	14.42	0.07	1.04	0.060	3.2E-01
Mtr.15413.1.S1_at	12.54	10.99	10.94	11.78	11.32	11.94	11.49	0.91	11.68	0.32	1.02	0.023	7.5E-01
Mtr.13821.1.S1_at	4.35	2.92	4.60	4.51	4.56	4.37	3.96	0.90	4.48	0.10	1.13	0.179	3.8E-01
Mtr.10458.1.S1_at	14.15	12.50	13.98	14.45	14.76	14.50	13.54	0.90	14.57	0.16	1.08	0.105	1.3E-01
Mtr.28700.1.S1_at	9.26	7.60	9.07	8.32	8.52	8.18	8.64	0.90	8.34	0.17	0.96	-0.052	5.9E-01
Mtr.28114.1.S1_at	5.64	4.04	5.56	5.27	4.52	4.89	5.08	0.90	4.89	0.38	0.96	-0.054	7.6E-01
Mtr.11440.1.S1_at	7.74	6.82	8.63	7.95	8.20	7.85	7.73	0.90	8.00	0.18	1.03	0.049	6.4E-01
Mtr.39383.1.S1_at	10.81	9.42	9.11	9.83	10.09	10.29	9.78	0.90	10.07	0.23	1.03	0.042	6.2E-01
Mtr.31247.1.S1_s_at	8.97	7.41	8.99	8.64	8.86	9.17	8.46	0.90	8.89	0.26	1.05	0.072	4.7E-01
Mtr.48657.1.S1_at	2.46	4.02	2.44	2.44	2.43	2.44	2.97	0.90	2.44	0.00	0.82	-0.287	3.6E-01
Mtr.13483.1.S1_at	7.25	6.59	8.38	8.27	7.94	8.18	7.40	0.90	8.13	0.17	1.10	0.135	2.4E-01
Mtr.51711.1.S1_at	2.23	3.77	2.18	2.21	2.19	2.21	2.73	0.90	2.20	0.01	0.81	-0.308	3.7E-01
Mtr.29451.1.S1_at	5.01	3.20	4.17	4.34	3.83	4.22	4.12	0.90	4.13	0.27	1.00	0.002	9.9E-01
Mtr.32974.1.S1_s_at	5.85	4.16	5.55	5.66	5.66	5.77	5.19	0.90	5.70	0.06	1.10	0.135	3.8E-01
Mtr.42714.1.S1_at	8.60	7.15	8.81	8.78	8.80	8.62	8.19	0.90	8.73	0.10	1.07	0.093	3.6E-01
Mtr.29411.1.S1_at	7.67	5.94	7.24	8.16	8.63	8.37	6.95	0.90	8.39	0.24	1.21	0.271	5.6E-02
Mtr.13853.1.S1_s_at	2.99	2.75	4.43	3.75	4.70	4.13	3.39	0.90	4.19	0.48	1.24	0.306	2.5E-01
Mtr.44705.1.S1_at	7.42	5.70	7.04	7.17	7.06	7.17	6.72	0.90	7.13	0.06	1.06	0.086	4.7E-01
Mtr.12928.1.S1_at	7.58	6.18	7.87	7.48	7.78	7.58	7.21	0.90	7.61	0.16	1.06	0.079	4.9E-01
Mtr.33060.1.S1_at	7.16	5.63	7.23	7.23	7.25	7.10	6.67	0.90	7.20	0.08	1.08	0.108	3.8E-01
Mtr.12474.1.S1_at	9.42	7.71	9.09	8.58	8.81	8.77	8.74	0.90	8.72	0.12	1.00	-0.003	9.7E-01
Mtr.3175.1.S1_at	4.19	3.40	5.20	4.26	5.15	4.23	4.26	0.90	4.55	0.52	1.07	0.093	6.6E-01
Mtr.44563.1.S1_at	10.14	8.42	9.77	10.00	9.94	9.87	9.44	0.90	9.94	0.06	1.05	0.073	4.0E-01
Mtr.29913.1.S1_at	2.20	3.75	2.17	2.17	2.17	2.16	2.71	0.90	2.16	0.00	0.80	-0.322	3.6E-01
Mtr.9936.1.S1_at	6.84	5.19	6.66	6.85	7.29	7.01	6.23	0.90	7.05	0.22	1.13	0.179	2.0E-01
Mtr.21028.1.S1_at	10.57	8.77	9.66	9.87	10.04	9.68	9.67	0.90	9.86	0.18	1.02	0.029	7.3E-01
Mtr.32529.1.S1_at	7.82	6.10	7.43	7.56	7.53	7.57	7.11	0.90	7.55	0.02	1.06	0.087	4.5E-01
Mtr.27872.1.S1_at	8.50	6.71	7.43	7.01	7.35	7.24	7.55	0.90	7.20	0.17	0.95	-0.068	5.5E-01
Mtr.34973.1.S1_at	4.25	2.58	4.01	4.24	5.04	4.79	3.61	0.90	4.69	0.41	1.30	0.376	1.3E-01
Mtr.32669.1.S1_at	5.52	4.19	5.91	6.14	5.64	5.99	5.21	0.90	5.93	0.26	1.14	0.187	2.5E-01
Mtr.11525.1.S1_at	5.25	3.68	5.22	5.41	5.23	5.30	4.72	0.90	5.31	0.09	1.13	0.172	3.2E-01
Mtr.13083.1.S1_at	8.14	6.41	7.72	7.68	7.76	7.78	7.42	0.90	7.74	0.05	1.04	0.061	5.7E-01
Mtr.11509.1.S1_at	11.07	9.44	9.59	10.25	9.93	10.32	10.03	0.90	10.16	0.21	1.01	0.019	8.2E-01
Mtr.1193.1.S1_at	6.95	5.29	6.73	6.95	6.95	7.07	6.33	0.90	6.99	0.07	1.10	0.144	2.7E-01
Mtr.51199.1.S1_at	8.31	7.20	8.98	8.77	8.91	8.60	8.17	0.90	8.76	0.15	1.07	0.101	3.2E-01
Mtr.26124.1.S1_at	7.42	6.15	7.89	7.74	7.96	7.74	7.16	0.90	7.81	0.13	1.09	0.127	2.8E-01
Mtr.4139.1.S1_at	8.57	7.15	8.82	8.95	9.32	9.01	8.18	0.90	9.09	0.20	1.11	0.152	1.6E-01
Mtr.4887.1.S1_at	2.50	4.05	2.48	2.48	2.47	2.41	3.01	0.90	2.45	0.04	0.81	-0.296	3.4E-01
Mtr.11472.1.S1_s_at	7.89	6.56	8.27	7.91	7.93	7.68	7.57	0.90	7.84	0.14	1.04	0.050	6.4E-01
Mtr.41222.1.S1_at	10.84	9.08	10.29	10.54	10.47	10.60	10.07	0.90	10.54	0.06	1.05	0.065	4.2E-01
Mtr.24481.1.S1_at	13.00	11.20	12.04	12.38	12.38	12.52	12.08	0.90	12.43	0.08	1.03	0.041	5.4E-01
Mtr.5850.1.S1_at	5.07	4.59	6.33	5.58	5.34	5.15	5.33	0.90	5.36	0.21	1.01	0.008	9.6E-01
Mtr.27932.1.S1_at	2.03	3.58	2.02	2.01	2.01	2.01	2.55	0.90	2.01	0.00	0.79	-0.339	3.6E-01
Mtr.756.1.S1_at	7.82	6.31	7.90	7.78	7.65	7.64	7.34	0.90	7.69	0.08	1.05	0.067	5.4E-01
Mtr.11908.1.S1_s_at	8.60	7.06	8.64	8.27	8.32	8.32	8.10	0.90	8.30	0.03	1.03	0.036	7.1E-01
Mtr.33037.1.S1_s_at	7.84	6.17	7.57	7.41	7.42	7.58	7.19	0.90	7.47	0.10	1.04	0.055	6.2E-01
Mtr.37608.1.S1_at	13.77	12.01	13.22	12.46	12.10	12.41	13.00	0.90	12.32	0.19	0.95	-0.077	2.7E-01
Mtr.40271.1.S1_at	8.87	7.88	9.68	9.21	9.10	9.02	8.81	0.90	9.11	0.10	1.03	0.048	6.0E-01
Mtr.12525.1.S1_at	12.90	11.10	11.94	12.77	12.54	12.89	11.98	0.90	12.73	0.18	1.06	0.088	2.3E-01
Mtr.10799.1.S1_at	8.52	6.74	7.88	8.40	8.32	8.60	7.71	0.90	8.44	0.14	1.09	0.130	2.4E-01
Mtr.18249.1.S1_at	3.32	4.87	3.30	3.26	3.04	3.28	3.83	0.90	3.20	0.14	0.83	-0.261	2.9E-01
Mtr.1866.1.S1_at	7.77	6.23	7.80	8.27	8.08	8.29	7.27	0.90	8.21	0.12	1.13	0.176	1.5E-01
Mtr.41049.1.S1_at	4.14	4.21	5.73	4.13	4.36	4.51	4.70	0.90	4.33	0.19	0.92	-0.116	5.3E-01
Mtr.42013.1.S1_at	6.17	4.52	5.96	5.00	5.01	4.56	5.55	0.90	4.85	0.26	0.87	-0.193	2.7E-01
Mtr.17952.1.S1_s_at	3.85	3.52	5.22	4.00	4.00	4.00	4.20	0.90	4.00	0.00	0.95	-0.068	7.3E-01
Mtr.33073.1.S1_at	7.95	6.35	7.86	7.85	8.14	8.16	7.39	0.90	8.05	0.17	1.09	0.124	2.8E-01
Mtr.35159.1.S1_at	7.18	5.44	6.71	6.92	6.83	6.63	6.44	0.90	6.79	0.15	1.05	0.076	5.4E-01
Mtr.18676.1.S1_at	9.81	8.29	9.88	10.19	10.09	9.92	9.33	0.90	10.07	0.14	1.08	0.110	2.3E-01
Mtr.11812.1.S1_at	7.89	6.24	7.68	7.84	7.81	7.85	7.27	0.90	7.84	0.02	1.08	0.108	3.4E-01
Mtr.15732.1.S1_at	2.15	3.71	2.15	2.21	2.14	2.13	2.67	0.90	2.16	0.04	0.81	-0.306	3.8E-01
Mtr.35083.1.S1_at	6.85	5.27	6.80	6.57	6.71	6.68	6.31	0.90	6.65	0.08	1.05	0.077	5.4E-01
Mtr.11958.1.S1_at	8.55	6.77	7.88	7.92	8.03	8.11	7.74	0.90	8.02	0.10	1.04	0.052	6.2E-01
Mtr.6869.1.S1_at	4.64	2.85	3.64	3.82	4.03	3.83	3.71	0.90	3.89	0.12	1.05	0.070	7.4E-01
Mtr.37751.1.S1_at	12.19	10.83	12.52	12.85	12.28	12.83	11.85	0.90	12.65	0.32	1.07	0.095	2.2E-01
Mtr.1031.1.S1_at	2.93	4.48	2.92	2.91	3.30	2.60	3.44	0.90	2.94	0.35	0.85	-0.227	4.2E-01
Mtr.49892.1.S1_at	4.02	2.46	4.01	4.01	4.14	4.01	3.50	0.90	4.05	0.07	1.16	0.212	3.5E-01
Mtr.43561.1.S1_s_at	8.73	7.01	8.30	8.47	8.30	8.25	8.01	0.90	8.34	0.11	1.04	0.058	5.7E-01
Mtr.6731.1.S1_at	7.71	6.45	8.19	7.80	7.69	7.68	7.45	0.90	7.72	0.07	1.04	0.052	6.3E-01
Mtr.47834.1.S1_at	5.56	4.30	6.04	5.25	5.04	5.20	5.30	0.90	5.17	0.11	0.97	-0.037	8.1E-01
Mtr.38500.1.S1_at	8.64	6.88	8.05	9.07	9.35	9.33	7.85	0.90	9.25	0.15	1.18	0.236	5.6E-02
Mtr.32127.1.S1_at	4.86	3.70	5.46	4.77	4.93	4.83	4.67	0.90	4.84	0.08	1.04	0.051	7.6E-01
Mtr.24148.1.S1_at	4.16	2.68	4.30	2.98	2.40	2.97	3.72	0.90	2.78	0.33	0.75	-0.418	1.7E-01
Mtr.26547.1.S1_at	6.30	4.68	6.16	5.63	6.54	5.46	5.71	0.90	5.88	0.58	1.03	0.041	8.0E-01
Mtr.32536.1.S1_at	6.71	4.95	6.11	6.74	6.40	6.78	5.92	0.90	6.64	0.21	1.12	0.164	2.5E-01
Mtr.16054.1.S1_at	6.10	4.40	5.72	6.75	6.96	6.26	5.41	0.90	6.66	0.36	1.23	0.300	8.8E-02
Mtr.9352.1.S1_at	11.46	9.67	10.49	11.18	11.36	11.52	10.54	0.90	11.35	0.17	1.08	0.107	2.0E-01
Mtr.43745.1.S1_at	4.66	2.90	4.07	3.76	5.13	3.75	3.87	0.90	4.21	0.79	1.09	0.120	6.5E-01
Mtr.29590.1.S1_at	6.49	5.87	7.63	6.93	6.83	6.55	6.66	0.90	6.77	0.19	1.02	0.023	8.5E-01
Mtr.45669.1.S1_at	6.33	5.43	7.22	6.32	6.38	6.32	6.33	0.90	6.34	0.04	1.00	0.003	9.8E-01
Mtr.11966.1.S1_at	3.91	2.54	4.23	4.23	3.91	3.89	3.56	0.90	4.01	0.19	1.13	0.170	4.5E-01
Mtr.50993.1.S1_s_at	3.51	5.07	3.53	3.47	3.59	3.52	4.04	0.89	3.52	0.06	0.87	-0.197	3.8E-01
Mtr.3073.1.S1_s_at	8.45	7.05	8.72	8.63	8.58	8.45	8.07	0.89	8.55	0.10	1.06	0.083	4.1E-01
Mtr.28042.1.S1_at	7.11	5.90	7.64	7.19	7.57	7.22	6.88	0.89	7.32	0.21	1.06	0.090	4.5E-01
Mtr.23608.1.S1_at	7.32	5.79	7.36	7.00	7.27	7.14	6.83	0.89	7.14	0.13	1.05	0.064	5.8E-01
Mtr.19822.1.S1_at	4.05	4.62	5.81	4.47	4.68	4.24	4.83	0.89	4.46	0.22	0.92	-0.114	5.3E-01
Mtr.17292.1.S1_at	7.21	5.73	7.34	7.34	7.40	7.14	6.76	0.89	7.30	0.14	1.08	0.110	3.6E-01
Mtr.50583.1.S1_at	7.96	6.55	8.21	7.95	8.40	8.36	7.58	0.89	8.24	0.25	1.09	0.121	2.8E-01
Mtr.34641.1.S1_at	2.44	3.98	2.43	2.42	2.42	2.41	2.95	0.89	2.42	0.01	0.82	-0.288	3.6E-01
Mtr.37762.1.S1_at	4.86	3.29	4.81	4.41	4.15	4.28	4.32	0.89	4.28	0.13	0.99	-0.014	9.4E-01
Mtr.2621.1.S1_at	8.81	7.13	8.49	8.44	8.56	8.64	8.14	0.89	8.55	0.10	1.05	0.070	4.8E-01
Mtr.5469.1.S1_at	2.01	3.54	1.98	1.97	1.97	1.97	2.51	0.89	1.97	0.00	0.79	-0.349	3.6E-01
Mtr.6022.1.S1_at	5.56	4.53	6.31	5.78	5.59	5.83	5.47	0.89	5.74	0.13	1.05	0.069	6.4E-01
Mtr.28034.1.S1_s_at	8.34	6.92	8.56	8.98	8.81	8.74	7.94	0.89	8.84	0.12	1.11	0.155	1.6E-01
Mtr.33377.1.S1_s_at	7.81	8.65	9.59	9.64	9.81	9.37	8.68	0.89	9.61	0.22	1.11	0.147	1.6E-01
Mtr.40392.1.S1_s_at	10.44	9.22	8.70	10.00	9.90	10.41	9.45	0.89	10.10	0.27	1.07	0.096	2.9E-01

Mtr.9577.1.S1_at	8.18	7.34	6.39	7.67	7.77	7.86	7.30	0.89	7.76	0.09	1.06	0.088	4.3E-01
Mtr.15278.1.S1_s_at	2.31	3.87	2.33	2.34	2.35	2.30	2.84	0.89	2.33	0.03	0.82	-0.283	3.8E-01
Mtr.25613.1.S1_s_at	2.40	3.94	2.39	2.38	2.38	2.37	2.91	0.89	2.38	0.00	0.82	-0.290	3.6E-01
Mtr.44086.1.S1_at	12.27	11.91	10.58	12.02	12.16	12.34	11.59	0.89	12.17	0.16	1.05	0.071	3.3E-01
Mtr.5228.1.S1_at	8.42	6.90	8.47	8.71	8.63	8.50	7.93	0.89	8.61	0.10	1.09	0.119	2.6E-01
Mtr.6872.1.S1_at	3.01	3.10	4.60	2.93	2.95	2.94	3.57	0.89	2.94	0.01	0.82	-0.281	2.9E-01
Mtr.28440.1.S1_at	5.03	3.45	4.95	5.19	5.19	5.25	4.48	0.89	5.21	0.03	1.16	0.218	2.3E-01
Mtr.7973.1.S1_at	2.76	3.88	2.12	2.75	2.74	2.75	2.92	0.89	2.75	0.00	0.94	-0.089	7.5E-01
Mtr.12953.1.S1_at	11.19	9.41	10.26	10.68	11.07	11.01	10.29	0.89	10.92	0.21	1.06	0.086	3.0E-01
Mtr.13536.1.S1_at	11.42	9.68	10.23	10.50	10.44	10.60	10.45	0.89	10.51	0.08	1.01	0.009	9.1E-01
Mtr.45354.1.S1_at	9.54	7.94	8.05	8.83	8.74	8.98	8.51	0.89	8.85	0.12	1.04	0.056	5.5E-01
Mtr.46655.1.S1_at	7.01	5.23	6.24	6.95	6.46	6.89	6.16	0.89	6.77	0.27	1.10	0.136	3.2E-01
Mtr.1923.1.S1_at	7.20	5.61	7.10	7.08	7.45	6.97	6.64	0.89	7.17	0.25	1.08	0.111	3.8E-01
Mtr.31296.1.S1_at	8.51	6.78	8.01	8.10	7.89	8.14	7.77	0.89	8.04	0.13	1.04	0.050	6.3E-01
Mtr.8858.1.S1_at	7.73	6.04	7.38	7.37	7.50	7.62	7.05	0.89	7.50	0.13	1.06	0.089	4.4E-01
Mtr.27992.1.S1_at	9.00	7.29	8.59	8.68	8.74	8.85	8.29	0.89	8.76	0.09	1.06	0.078	4.2E-01
Mtr.41665.1.S1_at	11.95	10.50	12.12	12.17	12.65	12.21	11.52	0.89	12.34	0.27	1.07	0.099	2.0E-01
Mtr.14302.1.S1_at	6.89	8.38	6.80	7.06	7.14	7.02	7.36	0.89	7.07	0.06	0.96	-0.057	6.1E-01
Mtr.22555.1.S1_at	3.04	4.57	3.02	3.66	2.89	2.91	3.54	0.89	3.15	0.44	0.89	-0.169	5.3E-01
Mtr.41731.1.S1_at	4.77	3.25	4.81	4.20	4.61	4.62	4.28	0.89	4.48	0.24	1.05	0.066	7.3E-01
Mtr.36619.1.S1_at	2.74	4.22	2.62	2.61	2.50	2.56	3.19	0.89	2.56	0.05	0.80	-0.319	2.9E-01
Mtr.13082.1.S1_at	7.96	6.48	8.08	7.60	7.98	7.89	7.51	0.89	7.82	0.20	1.04	0.059	5.8E-01
Mtr.50028.1.S1_x_at	2.81	4.33	2.78	2.71	2.78	2.75	3.31	0.89	2.75	0.04	0.83	-0.268	3.4E-01
Mtr.4186.1.S1_at	8.05	6.44	7.91	8.08	8.09	8.00	7.47	0.89	8.06	0.05	1.08	0.110	3.1E-01
Mtr.27086.1.S1_at	3.48	2.25	3.98	3.49	3.81	3.57	3.24	0.89	3.62	0.16	1.12	0.162	5.0E-01
Mtr.23580.1.S1_at	11.06	12.50	10.88	11.51	12.02	11.87	11.48	0.89	11.80	0.26	1.03	0.040	5.8E-01
Mtr.48408.1.S1_x_at	2.39	3.93	2.38	2.38	2.38	2.38	2.90	0.89	2.38	0.00	0.82	-0.286	3.7E-01
Mtr.23786.1.S1_at	4.79	3.05	4.22	4.21	3.59	4.18	4.02	0.89	3.99	0.35	0.99	-0.010	9.6E-01
Mtr.26941.1.S1_at	7.79	6.35	7.97	7.57	7.82	7.31	7.37	0.89	7.57	0.25	1.03	0.038	7.3E-01
Mtr.22059.1.S1_at	10.98	9.27	10.56	10.87	10.85	10.99	10.27	0.89	10.91	0.07	1.06	0.086	2.9E-01
Mtr.41550.1.S1_at	8.10	6.49	7.96	7.83	7.95	7.57	7.52	0.89	7.78	0.20	1.03	0.050	6.4E-01
Mtr.33684.1.S1_at	7.11	5.83	7.54	7.52	7.47	7.59	6.83	0.89	7.53	0.06	1.10	0.140	2.5E-01
Mtr.3251.1.S1_at	8.39	6.95	8.58	8.58	8.57	8.44	7.97	0.89	8.53	0.08	1.07	0.097	3.4E-01
Mtr.18243.1.S1_s_at	3.25	2.06	3.80	3.78	3.54	3.36	3.04	0.89	3.56	0.21	1.17	0.229	3.8E-01
Mtr.41422.1.S1_at	5.51	4.21	5.91	5.87	6.57	6.33	5.21	0.89	6.26	0.36	1.20	0.264	1.3E-01
Mtr.7569.1.S1_at	4.93	3.58	3.26	3.46	3.83	3.25	3.93	0.89	3.52	0.29	0.90	-0.159	4.9E-01
Mtr.8975.1.S1_at	11.06	9.36	9.75	9.97	10.05	10.28	10.06	0.89	10.10	0.16	1.00	0.006	9.4E-01
Mtr.8341.1.S1_at	7.20	5.50	6.78	6.47	7.13	7.03	6.49	0.89	6.87	0.35	1.06	0.082	5.3E-01
Mtr.42839.1.S1_at	9.31	8.71	10.46	10.24	10.35	10.05	9.50	0.89	10.21	0.15	1.08	0.105	2.4E-01
Mtr.49859.1.S1_s_at	7.23	5.49	6.06	6.08	6.17	5.55	6.26	0.89	5.93	0.34	0.95	-0.077	5.8E-01
Mtr.10101.1.S1_at	6.56	5.95	7.70	7.24	7.27	7.38	6.74	0.89	7.30	0.07	1.08	0.115	3.4E-01
Mtr.39725.1.S1_at	7.62	6.16	7.76	7.53	7.49	7.51	7.18	0.89	7.51	0.02	1.05	0.065	5.5E-01
Mtr.1727.1.S1_at	4.63	2.94	4.27	4.74	4.27	4.27	3.95	0.89	4.43	0.27	1.12	0.165	4.2E-01
Mtr.13080.1.S1_at	4.10	4.15	5.66	4.27	4.23	4.08	4.64	0.89	4.19	0.10	0.90	-0.144	4.4E-01
Mtr.32861.1.S1_s_at	5.46	3.69	4.50	4.20	4.60	4.16	4.55	0.89	4.32	0.24	0.95	-0.075	6.9E-01
Mtr.42174.1.S1_at	6.44	4.73	6.01	5.96	6.21	6.06	5.73	0.89	6.08	0.13	1.06	0.086	5.4E-01
Mtr.6163.1.S1_at	5.61	4.96	6.71	5.16	6.09	5.64	5.76	0.89	5.63	0.46	0.98	-0.032	8.4E-01
Mtr.45697.1.S1_at	8.10	6.55	8.09	7.93	7.94	7.95	7.58	0.89	7.94	0.01	1.05	0.067	5.2E-01
Mtr.36799.1.S1_s_at	8.95	7.60	9.28	9.03	9.16	9.02	8.61	0.89	9.07	0.08	1.05	0.075	4.2E-01
Mtr.33797.1.S1_at	9.65	8.05	9.51	9.35	9.21	9.32	9.07	0.89	9.29	0.07	1.02	0.035	6.9E-01
Mtr.5801.1.S1_at	7.79	6.06	7.27	7.61	7.61	7.78	7.04	0.89	7.67	0.10	1.09	0.122	2.9E-01
Mtr.26651.1.S1_at	3.31	4.82	3.26	3.24	3.19	3.26	3.80	0.89	3.23	0.03	0.85	-0.234	3.3E-01
Mtr.10707.1.S1_at	9.80	8.10	9.39	10.07	9.42	10.31	9.09	0.89	9.93	0.46	1.09	0.127	2.2E-01
Mtr.38390.1.S1_at	10.06	8.87	8.33	8.90	8.61	9.02	9.09	0.89	8.85	0.21	0.97	-0.039	6.7E-01
Mtr.9942.1.S1_at	7.73	6.13	7.59	7.70	7.85	7.51	7.15	0.89	7.69	0.17	1.08	0.104	3.6E-01
Mtr.2135.1.S1_s_at	7.16	5.62	7.15	7.14	7.01	6.73	6.64	0.89	6.96	0.21	1.05	0.067	5.8E-01
Mtr.29223.1.S1_at	5.81	4.35	5.94	5.17	5.97	4.88	5.37	0.89	5.34	0.56	0.99	-0.008	9.6E-01
Mtr.4013.1.S1_at	8.15	6.50	7.88	7.54	8.20	7.92	7.51	0.89	7.89	0.33	1.05	0.071	5.3E-01
Mtr.1819.1.S1_at	6.68	5.12	6.63	6.78	6.45	6.66	6.14	0.89	6.63	0.17	1.08	0.110	4.0E-01
Mtr.44743.1.S1_at	7.55	5.86	7.16	7.38	7.67	7.70	6.86	0.89	7.58	0.18	1.11	0.145	2.4E-01
Mtr.5564.1.S1_at	5.01	3.30	4.58	4.42	4.69	4.84	4.29	0.89	4.65	0.21	1.08	0.114	5.4E-01
Mtr.50813.1.S1_at	5.41	3.87	5.40	5.40	5.04	4.68	4.90	0.89	5.04	0.36	1.03	0.042	8.0E-01
Mtr.40194.1.S1_at	8.99	10.75	9.75	9.95	10.41	9.85	9.83	0.89	10.07	0.30	1.02	0.035	6.8E-01
Mtr.33321.1.S1_at	6.35	5.14	6.86	7.10	7.15	7.15	6.12	0.89	7.13	0.03	1.17	0.222	1.2E-01
Mtr.23812.1.S1_at	6.74	5.63	7.38	7.85	7.98	7.94	6.59	0.89	7.92	0.06	1.20	0.267	5.9E-02
Mtr.11831.1.S1_at	9.40	7.79	9.23	9.29	9.41	9.37	8.81	0.88	9.36	0.06	1.06	0.087	3.4E-01
Mtr.2920.1.S1_at	6.47	4.95	6.49	6.40	6.54	6.36	5.97	0.88	6.43	0.09	1.08	0.109	4.2E-01
Mtr.18475.1.S1_at	2.44	3.96	2.41	2.41	2.40	2.39	2.94	0.88	2.40	0.01	0.82	-0.292	3.5E-01
Mtr.29087.1.S1_s_at	7.53	6.40	8.15	8.86	8.45	8.59	7.36	0.88	8.63	0.21	1.17	0.230	7.2E-02
Mtr.7054.1.S1_s_at	13.31	12.21	13.96	13.72	13.76	13.51	13.16	0.88	13.66	0.14	1.04	0.054	3.9E-01
Mtr.40728.1.S1_at	8.79	7.17	8.58	8.61	8.85	8.59	8.18	0.88	8.69	0.15	1.06	0.087	3.8E-01
Mtr.41386.1.S1_at	8.48	6.84	8.23	8.18	8.23	8.37	7.85	0.88	8.26	0.10	1.05	0.073	4.7E-01
Mtr.8779.1.S1_at	13.11	11.36	12.45	12.41	13.04	12.90	12.31	0.88	12.78	0.33	1.04	0.055	4.3E-01
Mtr.44846.1.S1_at	7.88	6.33	7.85	7.85	8.07	7.61	7.35	0.88	7.84	0.23	1.07	0.093	4.0E-01
Mtr.33081.1.S1_at	7.76	6.49	8.20	7.43	8.29	7.79	7.48	0.88	7.84	0.43	1.05	0.067	5.7E-01
Mtr.42654.1.S1_at	7.93	6.31	7.72	7.84	7.96	8.22	7.32	0.88	8.01	0.19	1.09	0.129	2.6E-01
Mtr.1130.1.S1_s_at	5.98	4.78	6.50	6.43	6.27	6.25	5.75	0.88	6.32	0.10	1.10	0.135	3.3E-01
Mtr.43759.1.S1_at	7.86	6.43	8.04	8.06	8.02	7.98	7.44	0.88	8.02	0.04	1.08	0.107	3.2E-01
Mtr.37759.1.S1_at	8.84	7.70	9.43	9.27	9.50	8.91	8.66	0.88	9.23	0.30	1.07	0.092	3.5E-01
Mtr.42808.1.S1_at	5.43	4.94	3.71	5.29	5.42	6.03	4.69	0.88	5.58	0.39	1.19	0.250	1.9E-01
Mtr.23370.1.S1_at	8.67	7.09	8.55	8.50	8.47	8.54	8.10	0.88	8.50	0.03	1.05	0.070	4.8E-01
Mtr.48826.1.S1_s_at	7.08	6.01	7.76	6.91	7.27	7.11	6.95	0.88	7.09	0.18	1.02	0.030	8.0E-01
Mtr.40687.1.S1_at	10.11	8.44	9.77	9.88	9.82	9.87	9.44	0.88	9.86	0.03	1.04	0.062	4.6E-01
Mtr.44150.1.S1_at	8.17	6.47	7.73	7.18	7.42	7.66	7.46	0.88	7.42	0.24	1.00	-0.007	9.5E-01
Mtr.34713.1.S1_s_at	5.78	4.03	5.13	5.10	5.31	4.97	4.98	0.88	5.13	0.17	1.03	0.042	7.9E-01
Mtr.45021.1.S1_at	5.93	5.67	7.31	7.01	7.52	7.16	6.30	0.88	7.23	0.26	1.15	0.198	1.6E-01
Mtr.6789.1.S1_at	7.28	5.74	7.26	7.21	7.47	7.15	6.76	0.88	7.28	0.17	1.08	0.106	3.7E-01
Mtr.13203.1.S1_at	8.30	6.56	7.66	7.99	7.41	7.62	7.51	0.88	7.67	0.29	1.02	0.032	7.7E-01
Mtr.4844.1.S1_at	2.82	4.40	2.93	2.91	3.03	2.92	3.38	0.88	2.95	0.07	0.87	-0.196	4.5E-01
Mtr.7824.1.S1_s_at	8.85	7.11	8.23	8.07	7.75	8.30	8.06	0.88	8.04	0.27	1.00	-0.004	9.7E-01
Mtr.33755.1.S1_at	5.74	4.88	6.64	5.73	6.17	5.62	5.75	0.88	5.84	0.29	1.02	0.022	8.8E-01
Mtr.32891.1.S1_at	5.17	3.61	5.09	4.43	5.40	4.56	4.62	0.88	4.80	0.53	1.04	0.053	7.9E-01
Mtr.35177.1.S1_s_at	7.92	6.22	7.47	7.67	7.52	7.51	7.20	0.88	7.57	0.09	1.05	0.071	5.2E-01
Mtr.15460.1.S1_at	2.92	4.44	2.91	2.90	2.90	2.91	3.42	0.88	2.90	0.00	0.85	-0.236	3.7E-01
Mtr.34938.1.S1_s_at	8.36	7.01	8.67	8.40	8.62	8.45	8.01	0.88	8.49	0.12	1.06	0.083	4.1E-01
Mtr.45188.1.S1_at	7.56	7.21	8.88	9.38	8.21	8.65	7.88	0.88	8.75	0.59	1.11	0.150	2.3E-01
Mtr.38100.1.S1_at	8.76	7.09	8.43	8.92	9.50	9.36	8.09	0.88	9.26	0.30	1.14	0.194	9.6E-02
Mtr.20947.1.S1_at	5.42	3.74	4.10	5.29	4.78	5.38	4.42	0.88	5.15	0.32	1.17	0.221	2.5E-01
Mtr.27792.1.S1_at	7.01	5.96	7.71	6.66	6.98	6.73	6.90	0.88	6.79	0.17	0.99	-0.022	8.5E-01
Mtr.17412.1.S1_x_at	7.04	5.35	6.63	8.27	8.76	8.23	6.34	0.88	8.42	0.29	1.33	0.410	1.8E-02
Mtr.4716.1.S1_at	9.65	8.18	9.76	10.15	10.52	10.29	9.19	0.88	10.32	0.19	1.12	0.167	9.6E-02
Mtr.27172.1.S1_at	7.91	6.94	8.69	8.29	8.26	8.28	7.85	0.88	8.28	0.01	1.05	0.077	4.5E-01
Mtr.11558.1.S1_s_at	9.69	7.95	9.00	9.38	9.19	9.44	8.88	0.88	9.33	0.13	1.05	0.072	4.3E-01
Mtr.2574.1.S1_at	4.89	3.95	5.71	4.87	5.06	4.80	4.85	0.88	4.91	0.14	1.01	0.019	9.1E-01
Mtr.45098.1.S1_at	7.49	5.75	6.83	7.13	7.11	7.39	6.69	0.88	7.21	0.16	1.08	0.108	3.7E-01
Mtr.46214.1.S1_at	2.66	4.20	2.69	2.63	2.61	2.59	3.18	0.88	2.61	0.02	0.82	-0.286	3.2E-01
Mtr.12881.1.S1_at	7.18	5.59	7.03	7.38	7.66	7.03	6.60	0.88	7.35	0.32	1.11	0.157	2.3E-01
Mtr.50469.1.S1_at	7.46	6.04	7.65	7.43	7.38	7.19	7.05	0.88	7.33	0.13	1.04	0.057	6.1E-01
Mtr.42933.1.S1_s_at	10.06	11.42	11.71	12.12	12.40	11.85	11.06	0.88	12.12	0.27	1.10	0.132	1.2E-01
Mtr.38132.1.S1_a_at	9.98	8.56	10.16	9.90	10.07	9.79	9.57	0.88	9.92	0.14	1.04	0.052	5.3E-01
Mtr.5282.1.S1_at	4.42	3.11	4.79	5.17	4.78	5.17	4.11	0.88	5.04	0.22	1.23	0.296	1.5E-01
Mtr.44578.1.S1_at	9.02	7.32	8.59	8.55	8.52	8.47	8.31	0.88	8.51	0.04	1.02	0.034	7.1E-01
Mtr.12434.1.S1_at	12.70	10.94	11.83	12.36	12.56	12.47	11.82	0.88	12.47	0.10	1.05	0.077	2.8E-01
Mtr.19981.1.S1_at	6.03	4.84	4.31	4.72	4.53	5.27	5.06	0.88	4.84	0.38	0.96	-0.064	7.1E-01
Mtr.49483.1.S1_s_at	6.67	5.28	6.90	6.70	6.75	6.52	6.28	0.88	6.65	0.12	1.06	0.082	5.1E-01
Mtr.15058.1.S1_at	5.76	4.10	5.44	6.19	7.27	6.04	5.10	0.88	6.50	0.67	1.27	0.350	9.3E-02
Mtr.17702.1.S1_at	7.82	6.43	8.06	7.55	7.81	7.91	7.44	0.88	7.76	0.18	1.04	0.061	5.7E-01
Mtr.51942.1.S1_at	8.13	6.42	7.62	7.75	8.06	7.84	7.39	0.88	7.88	0.16	1.07	0.093	4.0E-01
Mtr.40054.1.S1_at	5.32	4.46	6.22	5.11	5.34	5.29	5.33	0.88	5.25	0.12	0.98	-0.024	8.7E-01
Mtr.44386.1.S1_at	5.88	4.37	4.34	6.49	6.25	6.43	4.86	0.88	6.39	0.13	1.31	0.394	4.1E-02
Mtr.24685.1.S1_at	5.13	4.06	5.81	5.33	6.08	4.64	5.00	0.88	5.35	0.72	1.07	0.098	6.2E-01
Mtr.27780.1.S1_at	4.36	4.17	2.76	7.55	8.18	7.85	3.76	0.88	7.86	0.31	2.09	1.062	1.6E-03
Mtr.1885.1.S1_at	9.28	7.54	8.58	8.43	8.15	8.50	8.47	0.88	8.36	0.19	0.99	-0.019	8.4E-01
Mtr.14987.1.S1_at	2.49	4.01	2.49	2.48	2.47	2.47	3.00	0.88	2.47	0.01	0.82	-0.278	3.6E-01
Mtr.5030.1.S1_at	7.03	5.33	6.57	6.76	6.77	7.03	6.31	0.88	6.85	0.15	1.09	0.119	3.5E-01
Mtr.28738.1.S1_at	6.30	5.15	6.88	6.88	6.58	6.43	6.11	0.88	6.63	0.23	1.08	0.118	3.8E-01
Mtr.12161.1.S1_at	6.58	5.49	7.23	8.63	7.97	8.25	6.44	0.88	8.28	0.33	1.29	0.364	2.7E-02
Mtr.33297.1.S1_s_at	8.33	6.80	8.32	8.25	8.15	8.40	7.82	0.88	8.26	0.13	1.06	0.080	4.3E-01
Mtr.22822.1.S1_s_at	11.41	10.35	12.10	12.03	12.09	11.85	11.28	0.88	11.99	0.13	1.06	0.087	2.4E-01
Mtr.31760.1.S1_at	6.87	5.20	6.50	6.68	6.58	6.57	6.19	0.88	6.61	0.06	1.07	0.094	4.6E-01
Mtr.21157.1.S1_at	2.34	3.85	2.32	2.54	2.31	2.31	2.84	0.88	2.38	0.13	0.84	-0.251	4.3E-01

Mtr.33919.1.S1_s_at	7.55	5.92	7.31	7.71	7.59	7.52	6.93	0.88	7.61	0.09	1.10	0.135	2.5E-01
Mtr.30940.1.S1_at	2.86	4.37	2.85	2.84	2.84	2.83	3.36	0.88	2.84	0.01	0.84	-0.245	3.6E-01
Mtr.1664.1.S1_at	4.28	2.84	4.43	4.18	4.20	3.84	3.85	0.88	4.07	0.20	1.06	0.083	6.9E-01
Mtr.33421.1.S1_at	7.24	5.49	6.20	6.22	6.05	6.30	6.31	0.88	6.19	0.13	0.98	-0.028	8.3E-01
Mtr.30915.1.S1_at	7.78	6.57	8.27	8.01	8.14	7.67	7.54	0.88	7.94	0.24	1.05	0.075	4.9E-01
Mtr.10243.1.S1_at	7.80	6.48	8.15	8.19	7.94	7.99	7.48	0.88	8.04	0.13	1.08	0.105	3.3E-01
Mtr.11876.1.S1_at	7.04	5.37	5.73	6.10	6.17	6.35	6.05	0.88	6.21	0.13	1.03	0.038	7.7E-01
Mtr.35488.1.S1_at	11.31	9.77	9.82	10.57	10.48	10.68	10.30	0.88	10.58	0.10	1.03	0.038	6.2E-01
Mtr.34591.1.S1_s_at	6.45	4.82	6.19	6.66	5.54	6.84	5.82	0.88	6.35	0.70	1.09	0.125	4.6E-01
Mtr.32820.1.S1_s_at	3.23	3.49	4.86	3.23	3.20	3.13	3.86	0.88	3.19	0.05	0.83	-0.277	2.5E-01
Mtr.440.1.S1_at	2.75	4.27	2.74	2.74	2.74	2.74	3.25	0.88	2.74	0.00	0.84	-0.248	3.7E-01
Mtr.4531.1.S1_s_at	11.38	9.62	10.45	10.81	10.60	10.79	10.48	0.88	10.73	0.11	1.02	0.034	6.5E-01
Mtr.11988.1.S1_at	5.95	5.07	6.83	6.53	6.53	6.64	5.95	0.88	6.57	0.06	1.10	0.142	2.9E-01
Mtr.39513.1.S1_at	8.55	6.93	8.31	8.14	8.54	8.38	7.93	0.88	8.35	0.20	1.05	0.075	4.6E-01
Mtr.6804.1.S1_at	7.37	6.42	8.17	7.73	7.42	7.33	7.32	0.88	7.49	0.21	1.02	0.033	7.6E-01
Mtr.36169.1.S1_at	6.28	4.79	6.33	6.90	6.73	6.58	5.80	0.88	6.74	0.16	1.16	0.217	1.4E-01
Mtr.38873.1.S1_at	5.26	5.28	6.79	5.27	5.02	5.26	5.78	0.88	5.18	0.14	0.90	-0.157	3.1E-01
Mtr.2026.1.S1_at	6.77	5.41	7.05	7.23	6.96	6.75	6.41	0.88	6.98	0.24	1.09	0.122	3.4E-01
Mtr.14160.1.S1_at	7.76	6.67	8.41	8.00	7.94	7.65	7.61	0.88	7.86	0.19	1.03	0.047	6.5E-01
Mtr.40999.1.S1_at	10.94	9.33	9.54	9.72	9.84	9.84	9.93	0.88	9.80	0.07	0.99	-0.020	8.0E-01
Mtr.13774.1.S1_at	8.64	7.48	9.20	8.57	8.48	8.09	8.44	0.88	8.38	0.26	0.99	-0.010	9.1E-01
Mtr.31372.1.S1_at	3.78	5.19	3.58	3.83	3.45	3.62	4.19	0.88	3.63	0.19	0.87	-0.204	3.5E-01
Mtr.42236.1.S1_at	2.68	4.17	2.63	2.64	2.64	2.61	3.16	0.88	2.63	0.02	0.83	-0.263	3.6E-01
Mtr.42210.1.S1_at	8.02	6.30	7.45	7.60	7.93	7.59	7.26	0.88	7.71	0.19	1.06	0.086	4.4E-01
Mtr.43284.1.S1_at	12.04	10.52	10.54	11.45	11.23	11.82	11.03	0.88	11.50	0.30	1.04	0.059	4.3E-01
Mtr.27655.1.S1_at	4.77	3.10	4.38	4.77	4.79	5.15	4.08	0.88	4.91	0.22	1.20	0.265	1.9E-01
Mtr.27635.1.S1_at	6.73	5.02	6.18	6.50	6.06	6.37	5.98	0.87	6.31	0.23	1.06	0.078	5.6E-01
Mtr.39716.1.S1_at	7.04	5.51	5.54	5.99	6.04	6.22	6.03	0.87	6.08	0.12	1.01	0.013	9.2E-01
Mtr.33923.1.S1_at	6.17	4.89	6.56	7.42	6.95	6.55	5.87	0.87	6.97	0.43	1.19	0.248	1.2E-01
Mtr.13654.1.S1_at	7.98	7.87	9.44	8.54	8.41	8.52	8.43	0.87	8.49	0.07	1.01	0.010	9.1E-01
Mtr.35449.1.S1_s_at	5.65	4.04	4.27	4.24	4.24	4.03	4.65	0.87	4.17	0.12	0.90	-0.159	4.0E-01
Mtr.11696.1.S1_at	11.19	9.45	10.48	10.66	10.44	10.44	10.38	0.87	10.51	0.13	1.01	0.018	8.1E-01
Mtr.29354.1.S1_at	2.22	3.73	2.21	2.20	2.27	2.20	2.72	0.87	2.22	0.04	0.82	-0.289	3.8E-01
Mtr.22277.1.S1_at	2.75	4.24	2.71	2.70	2.67	2.74	3.23	0.87	2.71	0.03	0.84	-0.256	3.6E-01
Mtr.39743.1.S1_at	5.16	3.41	4.34	4.92	4.85	4.86	4.30	0.87	4.88	0.03	1.13	0.180	3.2E-01
Mtr.41298.1.S1_at	4.93	3.45	5.00	5.16	4.92	4.91	4.46	0.87	5.00	0.14	1.12	0.164	3.5E-01
Mtr.8371.1.S1_at	3.85	2.13	3.28	3.26	2.78	2.78	3.09	0.87	2.94	0.28	0.95	-0.070	8.0E-01
Mtr.42933.1.S1_x_at	9.30	10.74	10.87	11.63	12.24	11.41	10.30	0.87	11.76	0.43	1.14	0.191	6.0E-02
Mtr.14151.1.S1_at	7.49	6.01	7.56	7.25	7.50	7.11	7.02	0.87	7.29	0.20	1.04	0.054	6.3E-01
Mtr.41638.1.S1_at	4.79	3.44	5.07	5.05	5.05	5.59	4.43	0.87	5.23	0.31	1.18	0.239	2.1E-01
Mtr.17102.1.S1_at	3.79	2.31	3.85	5.09	3.63	4.08	3.32	0.87	4.27	0.75	1.29	0.364	2.3E-01
Mtr.45107.1.S1_at	5.80	4.09	5.26	5.19	4.93	5.07	5.05	0.87	5.06	0.13	1.00	0.005	9.8E-01
Mtr.27743.1.S1_s_at	5.72	4.18	5.68	6.00	5.42	5.95	5.19	0.87	5.79	0.32	1.11	0.157	3.3E-01
Mtr.14658.1.S1_at	3.64	5.35	4.79	5.36	6.43	5.63	4.59	0.87	5.81	0.56	1.26	0.339	1.1E-01
Mtr.34013.1.S1_at	2.67	4.15	2.61	2.61	2.59	2.58	3.14	0.87	2.59	0.02	0.83	-0.277	3.4E-01
Mtr.41541.1.S1_at	6.85	5.52	7.17	7.73	7.89	7.57	6.51	0.87	7.73	0.16	1.19	0.247	7.6E-02
Mtr.3801.1.S1_at	8.55	7.00	8.48	8.23	8.60	8.39	8.01	0.87	8.41	0.18	1.05	0.070	4.8E-01
Mtr.34980.1.S1_s_at	7.23	5.80	7.38	7.39	7.08	7.39	6.80	0.87	7.29	0.18	1.07	0.099	4.0E-01
Mtr.45238.1.S1_at	7.34	5.98	7.61	7.29	7.46	7.37	6.97	0.87	7.37	0.08	1.06	0.080	4.8E-01
Mtr.16014.1.S1_at	8.77	7.24	8.72	8.97	8.67	8.58	8.24	0.87	8.74	0.20	1.06	0.084	3.9E-01
Mtr.33275.1.S1_s_at	5.78	4.79	6.52	5.77	5.88	6.00	5.70	0.87	5.88	0.11	1.03	0.046	7.3E-01
Mtr.35254.1.S1_at	5.14	4.90	6.51	5.02	5.00	5.00	5.52	0.87	5.01	0.01	0.91	-0.140	3.7E-01
Mtr.28333.1.S1_at	8.41	7.20	8.89	8.56	8.71	8.51	8.17	0.87	8.59	0.11	1.05	0.073	4.5E-01
Mtr.25831.1.S1_at	3.00	4.39	2.77	2.79	2.77	2.79	3.39	0.87	2.78	0.01	0.82	-0.283	3.0E-01
Mtr.36137.1.S1_at	2.73	4.23	2.71	2.68	2.67	2.67	3.22	0.87	2.67	0.01	0.83	-0.270	3.4E-01
Mtr.19974.1.S1_at	2.64	4.14	2.63	2.62	2.60	2.61	3.13	0.87	2.61	0.01	0.83	-0.263	3.6E-01
Mtr.20095.1.S1_s_at	6.59	5.20	6.80	6.86	6.81	6.52	6.20	0.87	6.73	0.18	1.09	0.118	3.6E-01
Mtr.4913.1.S1_at	7.37	5.95	7.54	7.69	7.72	7.39	6.95	0.87	7.60	0.18	1.09	0.129	2.8E-01
Mtr.34929.1.S1_at	8.20	7.81	9.48	8.82	9.16	8.35	8.50	0.87	8.78	0.41	1.03	0.047	6.4E-01
Mtr.33597.1.S1_at	7.53	5.96	6.09	6.93	6.91	6.25	6.53	0.87	6.70	0.39	1.03	0.037	7.7E-01
Mtr.48232.1.S1_x_at	2.38	3.89	2.37	2.37	2.37	2.36	2.88	0.87	2.36	0.00	0.82	-0.285	3.6E-01
Mtr.10222.1.S1_at	7.47	6.33	8.04	7.84	7.65	7.77	7.28	0.87	7.75	0.09	1.07	0.091	4.0E-01
Mtr.37268.1.S1_s_at	8.89	7.75	7.18	6.78	5.97	6.90	7.94	0.87	6.55	0.50	0.82	-0.278	7.4E-02
Mtr.33128.1.S1_at	8.27	6.64	7.97	8.18	8.12	8.24	7.63	0.87	8.18	0.06	1.07	0.101	3.3E-01
Mtr.42194.1.S1_at	10.18	8.51	9.78	9.47	9.20	9.54	9.49	0.87	9.40	0.18	0.99	-0.013	8.7E-01
Mtr.8552.1.S1_at	13.47	12.25	11.79	12.25	12.26	12.62	12.50	0.87	12.38	0.21	0.99	-0.014	8.2E-01
Mtr.13480.1.S1_at	8.01	6.63	8.24	8.17	7.80	7.84	7.63	0.87	7.94	0.20	1.04	0.057	5.8E-01
Mtr.21185.1.S1_at	2.89	4.38	2.85	2.85	2.83	2.79	3.37	0.87	2.82	0.03	0.84	-0.256	3.4E-01
Mtr.12857.1.S1_at	6.66	5.85	7.59	6.66	7.15	6.55	6.70	0.87	6.78	0.32	1.01	0.017	8.9E-01
Mtr.19042.1.S1_at	7.54	6.42	8.12	7.57	7.98	7.35	7.36	0.87	7.63	0.32	1.04	0.052	6.4E-01
Mtr.13420.1.S1_at	7.54	6.66	8.40	7.78	7.71	7.45	7.53	0.87	7.65	0.17	1.01	0.021	8.4E-01
Mtr.37387.1.S1_s_at	7.71	6.43	8.09	7.86	7.87	7.82	7.41	0.87	7.85	0.03	1.06	0.083	4.3E-01
Mtr.33788.1.S1_at	7.19	6.07	7.78	7.35	7.16	7.15	7.01	0.87	7.22	0.11	1.03	0.042	7.1E-01
Mtr.42392.1.S1_at	9.00	10.74	9.90	9.99	10.68	10.14	9.88	0.87	10.27	0.36	1.04	0.056	5.1E-01
Mtr.5783.1.S1_at	5.96	4.63	6.27	6.20	5.83	6.40	5.62	0.87	6.14	0.29	1.09	0.129	3.8E-01
Mtr.47691.1.S1_at	4.96	3.67	5.32	10.57	10.60	10.68	4.65	0.87	10.62	0.06	2.28	1.191	2.9E-04
Mtr.7001.1.S1_at	8.83	7.42	9.01	8.48	8.58	8.36	8.42	0.87	8.47	0.11	1.01	0.009	9.2E-01
Mtr.5848.1.S1_at	6.93	5.28	6.58	6.74	6.57	7.03	6.26	0.87	6.78	0.23	1.08	0.115	3.7E-01
Mtr.5419.1.S1_at	6.71	5.04	6.28	6.59	6.56	6.41	6.01	0.87	6.52	0.09	1.08	0.116	3.7E-01
Mtr.35290.1.S1_at	9.14	8.09	9.81	9.50	9.55	9.24	9.01	0.87	9.43	0.16	1.05	0.065	4.6E-01
Mtr.5755.1.S1_s_at	10.65	10.18	11.86	11.66	12.09	11.73	10.90	0.87	11.83	0.23	1.08	0.118	1.5E-01
Mtr.43587.1.S1_at	10.50	8.79	9.38	9.86	9.91	9.75	9.56	0.87	9.84	0.08	1.03	0.042	6.0E-01
Mtr.13947.1.S1_x_at	8.56	6.83	7.85	8.14	7.83	8.18	7.75	0.87	8.05	0.19	1.04	0.055	5.9E-01
Mtr.1881.1.S1_at	8.68	7.77	9.51	9.16	9.12	8.75	8.65	0.87	9.01	0.22	1.04	0.058	5.3E-01
Mtr.20422.1.S1_s_at	4.90	3.38	4.86	5.22	6.07	5.60	4.38	0.87	5.63	0.43	1.29	0.362	8.9E-02
Mtr.6878.1.S1_s_at	8.40	6.72	7.17	8.08	7.96	8.30	7.43	0.87	8.11	0.17	1.09	0.127	2.5E-01
Mtr.3027.1.S1_at	5.25	3.95	5.60	5.90	5.95	5.37	4.94	0.87	5.74	0.32	1.16	0.217	2.1E-01
Mtr.44712.1.S1_at	8.54	6.96	8.38	8.20	8.34	8.22	7.96	0.87	8.26	0.08	1.04	0.052	5.9E-01
Mtr.13779.1.S1_at	7.04	5.35	6.55	6.56	6.87	6.56	6.31	0.87	6.66	0.18	1.05	0.077	5.4E-01
Mtr.39192.1.S1_at	6.52	5.67	7.40	7.57	7.81	7.37	6.53	0.87	7.58	0.22	1.16	0.216	1.1E-01
Mtr.39394.1.S1_at	8.09	6.36	7.24	7.91	7.71	7.79	7.23	0.87	7.80	0.10	1.08	0.110	3.2E-01
Mtr.7565.1.S1_at	5.68	4.56	6.27	7.05	6.83	6.47	5.50	0.87	6.78	0.29	1.23	0.302	7.2E-02
Mtr.51667.1.S1_at	6.87	5.50	7.10	6.64	6.71	6.81	6.49	0.87	6.72	0.08	1.04	0.051	6.7E-01
Mtr.39651.1.S1_at	8.56	6.89	8.10	7.82	8.01	7.95	7.85	0.87	7.93	0.10	1.01	0.015	8.8E-01
Mtr.31049.1.S1_at	4.60	6.10	4.60	4.62	4.15	4.83	5.10	0.87	4.53	0.35	0.89	-0.170	3.5E-01
Mtr.8339.1.S1_at	8.43	6.94	8.45	8.36	8.73	8.46	7.94	0.87	8.51	0.19	1.07	0.100	3.3E-01
Mtr.32311.1.S1_at	9.23	9.23	10.73	9.52	9.67	9.14	9.73	0.87	9.44	0.27	0.97	-0.044	6.1E-01
Mtr.5661.1.S1_at	8.88	7.17	8.27	8.83	8.54	8.65	8.11	0.87	8.67	0.15	1.07	0.097	3.3E-01
Mtr.6181.1.S1_at	8.47	6.89	8.29	8.13	7.78	7.83	7.88	0.87	7.92	0.19	1.00	0.006	9.6E-01
Mtr.39528.1.S1_at	8.37	6.74	8.07	8.67	8.58	8.54	7.73	0.87	8.60	0.07	1.11	0.154	1.6E-01
Mtr.24264.1.S1_at	10.43	9.41	8.71	7.91	7.62	7.95	9.51	0.87	7.83	0.18	0.82	-0.282	3.0E-02
Mtr.27432.1.S1_at	6.72	6.22	5.03	5.81	4.69	5.54	5.99	0.87	5.35	0.58	0.89	-0.164	3.4E-01
Mtr.11452.1.S1_at	6.91	5.51	7.10	7.16	7.57	6.77	6.51	0.86	7.17	0.40	1.10	0.140	3.0E-01
Mtr.37191.1.S1_at	7.15	5.58	7.00	7.50	7.78	7.44	6.57	0.86	7.57	0.18	1.15	0.204	1.2E-01
Mtr.13541.1.S1_at	7.28	5.83	7.37	7.20	7.08	7.41	6.83	0.86	7.23	0.17	1.06	0.082	4.8E-01
Mtr.50614.1.S1_at	5.96	4.59	6.19	5.60	5.78	5.17	5.58	0.86	5.52	0.32	0.99	-0.017	9.1E-01
Mtr.9518.1.S1_at	7.34	5.61	6.45	6.73	6.96	6.84	6.47	0.86	6.85	0.11	1.06	0.082	4.9E-01
Mtr.49570.1.S1_at	3.14	4.59	3.05	3.08	3.00	3.03	3.59	0.86	3.03	0.04	0.84	-0.244	3.2E-01
Mtr.7593.1.S1_at	3.08	4.51	2.96	2.94	2.92	2.91	3.52	0.86	2.92	0.01	0.83	-0.267	3.0E-01
Mtr.21567.1.S1_at	7.20	5.68	7.16	7.10	7.24	7.14	6.68	0.86	7.16	0.08	1.07	0.100	3.9E-01
Mtr.498.1.S1_at	2.85	2.20	3.91	3.11	3.12	3.26	2.98	0.86	3.16	0.08	1.06	0.085	7.4E-01
Mtr.21925.1.S1_s_at	6.25	4.74	6.22	5.92	6.62	6.58	5.74	0.86	6.37	0.39	1.11	0.152	3.1E-01
Mtr.18336.1.S1_s_at	4.95	5.93	4.21	4.12	4.30	4.29	5.03	0.86	4.24	0.10	0.84	-0.247	1.9E-01
Mtr.1855.1.S1_at	7.18	5.56	6.89	7.08	6.88	6.80	6.54	0.86	6.92	0.15	1.06	0.081	4.9E-01
Mtr.6637.1.S1_s_at	11.87	10.21	10.63	10.79	10.92	10.86	10.90	0.86	10.86	0.07	1.00	-0.006	9.3E-01
Mtr.13865.1.S1_at	3.90	2.46	2.36	2.32	2.36	2.36	2.91	0.86	2.35	0.02	0.81	-0.309	3.2E-01
Mtr.42579.1.S1_s_at	7.05	5.32	6.26	5.81	5.94	5.62	6.21	0.86	5.79	0.16	0.93	-0.101	4.5E-01
Mtr.51385.1.S1_x_at	2.94	4.40	2.88	2.88	2.87	2.86	3.41	0.86	2.87	0.01	0.84	-0.248	3.4E-01
Mtr.31304.1.S1_at	2.98	4.47	2.96	2.82	2.93	3.00	3.47	0.86	2.92	0.09	0.84	-0.252	3.3E-01
Mtr.34453.1.S1_at	4.37	2.87	2.88	3.23	3.35	3.27	3.37	0.86	3.29	0.06	0.97	-0.038	8.7E-01
Mtr.41388.1.S1_at	8.82	7.34	8.86	8.73	9.08	8.79	8.34	0.86	8.87	0.18	1.06	0.089	3.6E-01
Mtr.42964.1.S1_at	2.46	3.94	2.43	2.41	2.42	2.40	2.94	0.86	2.41	0.01	0.82	-0.288	3.5E-01
Mtr.5262.1.S1_at	7.91	6.69	8.35	8.17	8.30	8.14	7.65	0.86	8.20	0.09	1.07	0.101	3.3E-01
Mtr.51561.1.S1_at	10.66	9.01	9.38	9.60	9.48	9.68	9.68	0.86	9.59	0.10	0.99	-0.014	8.6E-01
Mtr.44189.1.S1_at	10.51	8.81	9.42	10.17	9.85	9.92	9.58	0.86	9.98	0.17	1.04	0.059	4.8E-01
Mtr.2922.1.S1_at	3.48	1.98	3.47	3.45	4.13	3.95	2.98	0.86	3.85	0.35	1.29	0.370	1.8E-01
Mtr.10824.1.S1_at	11.25	9.94	11.56	11.29	11.44	11.14	10.92	0.86	11.29	0.15	1.03	0.049	5.0E-01
Mtr.11707.1.S1_x_at	8.35	7.21	8.90	8.77	8.66	8.80	8.16	0.86	8.74	0.07	1.07	0.100	3.1E-01
Mtr.49151.1.S1_at	7.63	6.48	8.17	7.86	7.95	7.73	7.43	0.86	7.85	0.11	1.06	0.079	4.5E-01
Mtr.41816.1.S1_at	6.44	5.63	7.36	6.46	6.28	6.47	6.48	0.86	6.40	0.10	0.99	-0.016	8.9E-01
Mtr.19213.1.S1_at	3.12	2.67	4.34	2.94	3.71	3.10	3.38	0.86	3.25	0.41	0.96	-0.054	8.3E-01

Mtr.21070.1.S1_at	4.99	3.27	4.07	3.75	4.04	3.65	4.11	0.86	3.82	0.20	0.93	-0.107	6.0E-01
Mtr.33478.1.S1_at	5.00	6.47	4.95	4.96	4.95	4.95	5.47	0.86	4.95	0.01	0.90	-0.144	3.5E-01
Mtr.1605.1.S1_at	3.52	2.14	3.72	3.47	3.47	3.46	3.13	0.86	3.47	0.00	1.11	0.150	5.3E-01
Mtr.38680.1.S1_at	8.52	7.10	8.65	8.71	9.22	8.79	8.09	0.86	8.91	0.27	1.10	0.139	1.9E-01
Mtr.39335.1.S1_at	6.59	5.71	7.43	7.14	7.14	7.27	6.58	0.86	7.18	0.07	1.09	0.127	2.9E-01
Mtr.43517.1.S1_at	7.33	6.04	7.67	7.42	7.40	7.40	7.01	0.86	7.41	0.01	1.06	0.078	4.8E-01
Mtr.11748.1.S1_at	8.22	6.74	8.25	8.12	8.37	8.09	7.74	0.86	8.19	0.15	1.06	0.082	4.2E-01
Mtr.28774.1.S1_at	4.78	3.83	5.54	4.14	3.55	3.52	4.72	0.86	3.74	0.35	0.79	-0.337	1.4E-01
Mtr.11291.1.S1_at	5.03	5.04	6.52	5.91	5.75	5.79	5.53	0.86	5.82	0.08	1.05	0.073	6.0E-01
Mtr.17359.1.S1_at	6.68	4.99	6.13	5.36	5.33	6.14	5.94	0.86	5.61	0.46	0.95	-0.081	5.9E-01
Mtr.1960.1.S1_at	7.66	6.69	8.41	7.76	7.99	7.71	7.59	0.86	7.82	0.15	1.03	0.044	6.6E-01
Mtr.33657.1.S1_at	6.11	4.39	5.20	5.55	5.78	5.49	5.23	0.86	5.61	0.15	1.07	0.099	5.0E-01
Mtr.24402.1.S1_at	7.78	6.47	8.10	7.96	7.72	7.84	7.45	0.86	7.84	0.12	1.05	0.074	4.8E-01
Mtr.15921.1.S1_at	10.75	9.54	11.20	10.74	10.82	10.73	10.50	0.86	10.76	0.05	1.03	0.036	6.2E-01
Mtr.32275.1.S1_s_at	8.39	6.70	7.30	8.32	8.10	8.22	7.46	0.86	8.21	0.11	1.10	0.138	2.1E-01
Mtr.17096.1.S1_at	4.13	4.19	5.65	4.16	4.58	3.77	4.66	0.86	4.17	0.40	0.90	-0.159	4.3E-01
Mtr.13864.1.S1_at	8.63	7.03	8.39	8.46	8.35	8.56	8.02	0.86	8.46	0.10	1.05	0.077	4.3E-01
Mtr.41447.1.S1_at	7.08	6.32	8.04	6.96	6.96	6.33	7.15	0.86	6.75	0.36	0.94	-0.082	5.0E-01
Mtr.788.1.S1_at	2.51	3.98	2.48	2.48	2.47	2.47	2.99	0.86	2.47	0.00	0.83	-0.274	3.6E-01
Mtr.31780.1.S1_at	2.87	2.18	3.89	3.63	2.44	2.61	2.98	0.86	2.89	0.65	0.97	-0.040	9.0E-01
Mtr.29213.1.S1_s_at	13.25	11.55	12.18	12.45	12.58	12.46	12.33	0.86	12.50	0.07	1.01	0.020	7.5E-01
Mtr.35071.1.S1_at	5.58	4.34	5.98	5.55	5.71	5.59	5.30	0.86	5.62	0.08	1.06	0.084	5.6E-01
Mtr.131.1.S1_at	2.50	3.98	2.48	2.47	2.46	2.44	2.98	0.86	2.46	0.01	0.82	-0.280	3.5E-01
Mtr.46967.1.S1_at	5.34	3.89	5.41	5.30	5.29	5.29	4.88	0.86	5.29	0.01	1.08	0.118	4.5E-01
Mtr.45133.1.S1_at	12.71	11.02	11.60	11.97	11.61	12.24	11.78	0.86	11.94	0.32	1.01	0.020	7.7E-01
Mtr.25331.1.S1_at	9.49	7.83	9.04	9.22	9.12	9.28	8.79	0.86	9.21	0.08	1.05	0.067	4.5E-01
Mtr.10510.1.S1_s_at	9.41	8.71	10.42	9.49	9.35	9.15	9.51	0.86	9.33	0.17	0.98	-0.027	7.4E-01
Mtr.46604.1.S1_x_at	3.42	1.81	3.15	4.22	3.15	4.28	2.79	0.86	3.88	0.64	1.39	0.475	1.5E-01
Mtr.41240.1.S1_at	11.50	10.19	11.80	11.96	11.85	11.81	11.17	0.86	11.87	0.08	1.06	0.089	2.3E-01
Mtr.6906.1.S1_at	5.43	3.81	5.12	5.46	5.41	5.89	4.79	0.86	5.59	0.26	1.17	0.224	2.0E-01
Mtr.2417.1.S1_at	2.23	3.72	2.22	2.21	2.22	2.24	2.72	0.86	2.22	0.01	0.82	-0.292	3.7E-01
Mtr.8766.1.S1_at	9.74	8.05	9.17	9.62	8.90	9.34	8.98	0.86	9.29	0.36	1.03	0.048	6.1E-01
Mtr.33034.1.S1_at	3.53	2.06	2.02	2.02	2.02	2.02	2.54	0.86	2.02	0.00	0.80	-0.331	3.5E-01
Mtr.39544.1.S1_s_at	9.40	7.98	9.53	9.14	9.02	9.08	8.97	0.86	9.08	0.06	1.01	0.018	8.4E-01
Mtr.36144.1.S1_at	5.24	3.92	5.53	5.48	5.36	5.37	4.90	0.86	5.40	0.07	1.10	0.142	3.7E-01
Mtr.45288.1.S1_at	5.39	5.02	6.66	5.37	5.86	5.37	5.69	0.86	5.53	0.28	0.97	-0.040	7.8E-01
Mtr.22780.1.S1_s_at	8.74	7.15	8.52	8.39	8.40	8.33	8.14	0.86	8.38	0.04	1.03	0.042	6.5E-01
Mtr.32509.1.S1_at	8.16	6.59	7.96	7.93	8.07	7.65	7.57	0.86	7.88	0.22	1.04	0.059	5.7E-01
Mtr.19288.1.S1_s_at	7.49	6.00	7.49	7.40	7.42	7.31	6.99	0.86	7.37	0.06	1.05	0.077	4.8E-01
Mtr.13918.1.S1_at	8.85	7.15	7.81	7.45	8.11	8.06	7.94	0.86	7.87	0.37	0.99	-0.011	9.2E-01
Mtr.36300.1.S1_at	3.41	4.88	3.37	3.37	3.15	3.33	3.89	0.86	3.28	0.12	0.84	-0.245	2.9E-01
Mtr.39050.1.S1_at	6.05	4.44	4.72	5.51	5.65	5.65	5.07	0.86	5.60	0.08	1.10	0.144	3.4E-01
Mtr.23011.1.S1_s_at	6.76	5.05	5.90	6.06	6.19	5.98	5.90	0.86	6.08	0.10	1.03	0.041	7.5E-01
Mtr.7343.1.S1_at	10.54	9.24	10.85	10.12	10.11	9.79	10.21	0.86	10.01	0.19	0.98	-0.029	7.1E-01
Mtr.35967.1.S1_s_at	10.05	8.70	10.28	10.21	10.10	10.10	9.68	0.86	10.13	0.06	1.05	0.067	4.1E-01
Mtr.8752.1.S1_at	6.57	6.27	4.96	4.19	3.39	3.78	5.93	0.86	3.79	0.40	0.64	-0.646	1.7E-02
Mtr.43407.1.S1_at	8.68	7.04	8.29	8.39	8.64	8.24	8.01	0.86	8.42	0.20	1.05	0.073	4.6E-01
Mtr.28268.1.S1_at	2.23	3.71	2.22	2.22	2.22	2.22	2.72	0.86	2.22	0.00	0.82	-0.293	3.7E-01
Mtr.13124.1.S1_at	7.62	6.28	7.87	7.61	7.59	7.62	7.26	0.86	7.61	0.02	1.05	0.069	5.1E-01
Mtr.11673.1.S1_at	9.59	8.04	8.18	8.49	8.49	8.83	8.60	0.86	8.60	0.20	1.00	0.001	9.9E-01
Mtr.33455.1.S1_at	8.49	6.99	8.47	9.14	8.95	8.83	7.98	0.86	8.97	0.16	1.12	0.169	1.2E-01
Mtr.33269.1.S1_at	7.87	6.41	7.91	8.38	8.34	8.35	7.40	0.86	8.36	0.02	1.13	0.176	1.2E-01
Mtr.30976.1.S1_at	2.33	3.81	2.33	2.31	2.31	2.30	2.82	0.86	2.31	0.01	0.82	-0.290	3.6E-01
Mtr.49066.1.S1_at	4.72	6.29	4.91	4.86	4.88	4.71	5.31	0.86	4.82	0.09	0.91	-0.140	3.8E-01
Mtr.39485.1.S1_s_at	7.59	8.02	9.24	9.39	9.34	9.18	8.28	0.86	9.30	0.11	1.12	0.168	1.1E-01
Mtr.2051.1.S1_s_at	7.21	5.72	7.19	6.74	7.03	6.70	6.70	0.86	6.82	0.18	1.02	0.025	8.3E-01
Mtr.43939.1.S1_s_at	9.23	8.46	10.17	9.52	9.43	8.90	9.29	0.86	9.28	0.34	1.00	-0.001	9.9E-01
Mtr.27437.1.S1_at	8.55	6.94	8.26	8.32	8.44	8.27	7.92	0.86	8.34	0.09	1.05	0.076	4.4E-01
Mtr.29274.1.S1_at	6.44	4.99	6.49	6.77	6.87	6.88	5.98	0.86	6.84	0.06	1.15	0.196	1.5E-01
Mtr.36817.1.S1_at	5.92	5.12	6.83	6.12	6.92	6.34	5.95	0.86	6.46	0.41	1.09	0.118	4.1E-01
Mtr.30866.1.S1_at	7.09	5.46	6.75	6.41	6.42	6.42	6.43	0.86	6.42	0.00	1.00	-0.004	9.7E-01
Mtr.31309.1.S1_at	2.50	2.39	3.92	2.35	2.81	2.77	2.94	0.85	2.64	0.25	0.90	-0.155	5.9E-01
Mtr.44384.1.S1_at	11.36	9.67	10.71	11.04	10.96	10.83	10.58	0.85	10.94	0.10	1.03	0.048	5.1E-01
Mtr.13293.1.S1_at	10.12	8.54	8.76	9.05	8.06	8.91	9.14	0.85	8.67	0.54	0.95	-0.075	4.7E-01
Mtr.31469.1.S1_at	7.57	6.29	7.91	7.70	7.87	7.89	7.26	0.85	7.82	0.10	1.08	0.108	3.2E-01
Mtr.52236.1.S1_at	5.21	4.11	5.80	5.58	5.15	4.94	5.04	0.85	5.22	0.33	1.04	0.051	7.5E-01
Mtr.27931.1.S1_at	2.09	3.57	2.08	2.08	2.08	2.07	2.58	0.85	2.08	0.00	0.80	-0.313	3.7E-01
Mtr.40909.1.S1_at	7.26	5.99	7.61	7.05	7.42	7.00	6.95	0.85	7.16	0.23	1.03	0.042	7.1E-01
Mtr.41585.1.S1_at	6.66	5.13	6.56	6.42	6.35	6.40	6.12	0.85	6.39	0.03	1.05	0.064	6.0E-01
Mtr.29721.1.S1_at	4.44	2.93	4.37	4.70	5.51	3.44	3.91	0.85	4.55	1.05	1.16	0.217	4.6E-01
Mtr.10397.1.S1_at	11.63	9.92	10.75	10.69	10.65	10.74	10.76	0.85	10.70	0.05	0.99	-0.009	9.0E-01
Mtr.39131.1.S1_at	7.90	6.31	7.62	7.97	8.02	7.96	7.28	0.85	7.98	0.03	1.10	0.133	2.3E-01
Mtr.43884.1.S1_at	8.19	6.49	7.49	7.75	7.76	7.77	7.39	0.85	7.76	0.01	1.05	0.071	4.9E-01
Mtr.46619.1.S1_at	8.63	6.99	8.24	7.95	7.98	7.88	7.95	0.85	7.94	0.05	1.00	-0.002	9.8E-01
Mtr.25206.1.S1_at	7.03	6.20	7.90	7.38	7.36	7.24	7.04	0.85	7.33	0.08	1.04	0.057	6.0E-01
Mtr.11885.1.S1_at	6.77	5.23	6.65	6.30	6.80	6.41	6.22	0.85	6.50	0.26	1.05	0.065	6.1E-01
Mtr.9873.1.S1_at	8.95	7.54	9.08	8.64	9.37	8.85	8.52	0.85	8.95	0.38	1.05	0.071	4.7E-01
Mtr.1054.1.S1_at	2.88	4.36	2.88	2.87	2.87	2.87	3.37	0.85	2.87	0.00	0.85	-0.232	3.7E-01
Mtr.7166.1.S1_at	5.85	4.25	5.56	5.72	5.90	5.93	5.22	0.85	5.85	0.11	1.12	0.165	2.7E-01
Mtr.23314.1.S1_at	7.63	7.17	8.82	10.02	10.31	10.08	7.88	0.85	10.14	0.15	1.29	0.364	1.1E-02
Mtr.41859.1.S1_at	9.18	7.48	8.42	8.29	8.15	8.34	8.36	0.85	8.26	0.10	0.99	-0.018	8.5E-01
Mtr.39267.1.S1_at	10.22	11.82	10.51	10.64	10.90	10.90	10.85	0.85	10.81	0.15	1.00	-0.005	9.5E-01
Mtr.19594.1.S1_at	8.31	6.62	7.66	7.76	7.98	7.73	7.53	0.85	7.82	0.14	1.04	0.056	5.8E-01
Mtr.39948.1.S1_at	3.91	3.66	5.25	3.91	3.90	3.89	4.27	0.85	3.90	0.01	0.91	-0.132	4.9E-01
Mtr.15958.1.S1_s_at	12.66	10.98	11.58	11.83	10.97	11.52	11.74	0.85	11.44	0.43	0.97	-0.037	6.2E-01
Mtr.16740.1.S1_at	5.32	3.97	5.55	5.21	5.59	5.21	4.95	0.85	5.34	0.22	1.08	0.109	4.9E-01
Mtr.49686.1.S1_at	1.99	3.46	1.98	1.97	1.97	1.97	2.47	0.85	1.97	0.00	0.80	-0.327	3.7E-01
Mtr.14514.1.S1_at	2.87	4.32	2.82	2.85	2.82	2.82	3.33	0.85	2.83	0.02	0.85	-0.236	3.6E-01
Mtr.39792.1.S1_at	8.10	6.53	7.88	7.86	8.10	7.71	7.50	0.85	7.89	0.20	1.05	0.073	4.8E-01
Mtr.17330.1.S1_x_at	6.61	5.18	6.70	6.48	7.01	6.31	6.16	0.85	6.60	0.36	1.07	0.099	4.6E-01
Mtr.31735.1.S1_s_at	8.81	7.69	9.36	9.05	9.13	9.18	8.62	0.85	9.12	0.07	1.06	0.082	3.7E-01
Mtr.30531.1.S1_at	2.16	3.63	2.15	2.14	2.14	2.14	2.64	0.85	2.14	0.00	0.81	-0.303	3.7E-01
Mtr.4285.1.S1_at	7.50	5.97	7.38	7.61	7.56	7.61	6.95	0.85	7.59	0.03	1.09	0.127	2.6E-01
Mtr.28127.1.S1_at	7.34	5.86	7.34	6.93	6.76	6.47	6.85	0.85	6.72	0.24	0.98	-0.027	8.2E-01
Mtr.24750.1.S1_at	7.77	6.35	7.87	8.04	7.95	8.49	7.33	0.85	8.16	0.29	1.11	0.155	1.9E-01
Mtr.3967.1.S1_at	6.89	5.69	7.33	6.43	7.09	6.53	6.64	0.85	6.68	0.36	1.01	0.009	9.4E-01
Mtr.50015.1.S1_at	7.09	6.45	8.13	7.55	8.01	7.41	7.22	0.85	7.66	0.32	1.06	0.084	4.6E-01
Mtr.44828.1.S1_at	8.02	6.53	7.99	7.83	7.80	7.68	7.51	0.85	7.77	0.08	1.03	0.049	6.3E-01
Mtr.31758.1.S1_at	8.12	6.77	8.33	7.90	7.88	7.57	7.74	0.85	7.78	0.19	1.01	0.009	9.3E-01
Mtr.48338.1.S1_a_at	2.58	4.04	2.55	2.55	2.54	2.48	3.05	0.85	2.52	0.04	0.83	-0.275	3.4E-01
Mtr.20567.1.S1_at	8.07	6.62	8.11	7.98	7.96	8.19	7.60	0.85	8.05	0.13	1.06	0.083	4.2E-01
Mtr.33827.1.S1_at	7.48	6.40	8.08	7.04	7.63	6.85	7.32	0.85	7.17	0.41	0.98	-0.029	8.0E-01
Mtr.27335.1.S1_at	7.38	6.03	7.60	7.47	7.54	7.35	7.00	0.85	7.45	0.09	1.06	0.090	4.1E-01
Mtr.37671.1.S1_x_at	7.28	5.71	7.05	6.95	7.13	7.00	6.68	0.85	7.03	0.09	1.05	0.073	5.2E-01
Mtr.31880.1.S1_at	9.04	7.61	7.53	7.86	7.55	7.83	8.06	0.85	7.75	0.17	0.96	-0.057	5.7E-01
Mtr.46208.1.S1_at	11.03	9.33	10.31	9.91	10.00	10.26	10.22	0.85	10.06	0.18	0.98	-0.023	7.6E-01
Mtr.17978.1.S1_at	2.73	4.19	2.71	2.70	2.69	2.71	3.21	0.85	2.70	0.01	0.84	-0.251	3.5E-01
Mtr.5325.1.S1_at	9.30	8.07	7.67	8.21	8.34	8.47	8.34	0.85	8.34	0.13	1.00	0.000	1.0E+00
Mtr.24288.1.S1_at	5.02	6.52	5.07	3.90	4.33	4.86	5.54	0.85	4.36	0.48	0.79	-0.343	1.1E-01
Mtr.49311.1.S1_at	8.71	7.50	9.14	8.84	8.68	8.47	8.45	0.85	8.67	0.19	1.03	0.037	6.8E-01
Mtr.16372.1.S1_x_at	7.52	5.91	7.20	8.02	8.90	8.76	6.88	0.85	8.56	0.47	1.25	0.316	4.0E-02
Mtr.6079.1.S1_at	7.64	6.00	7.22	7.37	7.01	7.06	6.95	0.85	7.14	0.20	1.03	0.039	7.2E-01
Mtr.34371.1.S1_at	9.25	10.22	10.94	11.50	11.38	11.16	10.14	0.85	11.35	0.17	1.12	0.162	7.3E-02
Mtr.13634.1.S1_at	4.23	3.04	2.59	3.03	2.58	2.55	3.29	0.85	2.72	0.27	0.83	-0.274	3.3E-01
Mtr.12877.1.S1_at	7.55	6.16	7.71	7.40	7.74	7.70	7.14	0.85	7.61	0.18	1.07	0.093	4.0E-01
Mtr.31952.1.S1_at	5.84	7.11	7.45	9.61	9.86	9.34	6.80	0.85	9.60	0.26	1.41	0.498	5.4E-03
Mtr.22927.1.S1_at	12.84	11.18	12.33	12.40	12.06	12.45	12.12	0.85	12.30	0.21	1.02	0.022	7.3E-01
Mtr.38700.1.S1_at	7.42	6.36	8.03	7.48	8.08	7.83	7.27	0.85	7.80	0.30	1.07	0.101	3.7E-01
Mtr.49260.1.S1_at	6.26	4.63	5.83	6.30	5.91	5.56	5.57	0.85	5.92	0.37	1.06	0.087	5.5E-01
Mtr.15140.1.S1_at	5.08	3.50	4.82	4.86	4.77	5.00	4.46	0.85	4.88	0.12	1.09	0.128	4.5E-01
Mtr.46523.1.S1_at	5.14	4.13	5.81	5.44	5.51	4.55	5.03	0.85	5.17	0.54	1.03	0.039	8.2E-01
Mtr.9616.1.S1_at	6.05	4.88	6.53	6.14	6.28	5.95	5.82	0.85	6.12	0.16	1.05	0.073	5.8E-01
Mtr.45056.1.S1_s_at	7.84	6.45	7.98	7.86	7.88	7.85	7.42	0.85	7.87	0.01	1.06	0.083	4.2E-01
Mtr.16358.1.S1_at	8.57	6.92	8.06	8.20	7.73	7.91	7.85	0.85	7.95	0.24	1.01	0.018	8.6E-01
Mtr.3017.1.S1_at	5.59	3.90	4.91	5.49	5.50	4.96	4.80	0.85	5.32	0.30	1.11	0.148	3.8E-01
Mtr.42124.1.S1_at	4.24	3.11	4.77	5.34	5.16	4.78	4.04	0.85	5.10	0.29	1.26	0.336	1.1E-01
Mtr.28720.1.S1_s_at	6.33	6.89	8.00	7.64	7.55	7.41	7.07	0.85	7.54	0.12	1.07	0.091	4.0E-01
Mtr.19596.1.S1_at	7.52	6.61	8.30	7.80	7.68	7.34	7.48	0.85	7.61	0.24	1.02	0.025	8.1E-01
Mtr.29121.1.S1_at	3.89	2.40	3.85	3.50	3.50	3.49	3.38	0.85	3.50	0.00	1.03	0.048	8.3E-01
Mtr.27755.1.S1_at	2.20	3.65	2.16	2.12	2.13	2.06	2.67	0.85	2.10	0.04	0.79	-0.345	3.1E-01
Mtr.22619.1.S1_at	3.69	5.15	3.67	3.62	3.60	3.61	4.17	0.85	3.61	0.01	0.87	-0.208	3.2E-01

Mtr.10112.1.S1_at	9.75	8.42	8.17	8.59	8.39	8.73	8.78	0.85	8.57	0.17	0.98	-0.034	7.0E-01
Mtr.7307.1.S1_at	5.84	4.23	4.60	10.03	9.91	9.91	4.89	0.85	9.95	0.07	2.03	1.025	5.0E-04
Mtr.49999.1.S1_at	10.31	8.63	9.30	9.35	9.82	9.55	9.42	0.85	9.57	0.23	1.02	0.024	7.7E-01
Mtr.6040.1.S1_at	4.63	4.27	5.88	4.93	5.10	4.62	4.93	0.85	4.88	0.25	0.99	-0.014	9.3E-01
Mtr.22762.1.S1_at	6.03	6.43	7.66	8.51	9.22	8.65	6.71	0.85	8.79	0.37	1.31	0.391	1.7E-02
Mtr.925.1.S1_at	2.36	3.82	2.35	2.34	2.25	2.62	2.84	0.85	2.41	0.19	0.85	-0.241	4.3E-01
Mtr.31118.1.S1_at	7.00	5.39	6.64	6.37	6.30	6.33	6.34	0.85	6.33	0.04	1.00	-0.002	9.9E-01
Mtr.40683.1.S1_at	7.25	7.14	8.66	7.62	7.44	6.74	7.68	0.85	7.27	0.47	0.95	-0.081	5.0E-01
Mtr.45336.1.S1_at	5.91	4.34	5.66	5.38	6.50	5.39	5.31	0.85	5.75	0.64	1.08	0.117	5.0E-01
Mtr.2580.1.S1_at	4.89	3.20	4.06	4.67	4.55	4.29	4.05	0.85	4.50	0.19	1.11	0.152	4.2E-01
Mtr.9995.1.S1_at	3.85	2.19	2.75	3.11	2.77	2.75	2.93	0.85	2.88	0.20	0.98	-0.028	9.2E-01
Mtr.26688.1.S1_at	3.36	3.91	5.02	3.81	4.04	4.21	4.10	0.85	4.02	0.20	0.98	-0.028	8.8E-01
Mtr.50169.1.S1_at	2.73	4.18	2.70	2.70	2.66	2.69	3.20	0.85	2.69	0.02	0.84	-0.253	3.5E-01
Mtr.11990.1.S1_at	7.83	6.26	7.60	7.93	7.87	8.04	7.23	0.85	7.95	0.08	1.10	0.136	2.2E-01
Mtr.3993.1.S1_at	2.26	3.72	2.26	2.25	2.24	2.25	2.75	0.85	2.25	0.00	0.82	-0.290	3.6E-01
Mtr.50244.1.S1_at	3.12	4.56	3.08	3.11	3.03	3.01	3.59	0.85	3.05	0.05	0.85	-0.234	3.3E-01
Mtr.10065.1.S1_at	7.06	5.76	7.34	7.17	7.05	7.34	6.72	0.85	7.19	0.15	1.07	0.097	4.0E-01
Mtr.7704.1.S1_at	2.95	4.38	2.88	2.86	2.83	2.87	3.41	0.85	2.85	0.02	0.84	-0.257	3.2E-01
Mtr.28668.1.S1_at	7.75	6.11	7.28	7.38	7.34	7.68	7.04	0.84	7.47	0.19	1.06	0.084	4.5E-01
Mtr.24465.1.S1_at	4.47	2.86	3.22	3.80	4.35	3.05	3.51	0.84	3.73	0.65	1.06	0.087	7.4E-01
Mtr.30707.1.S1_at	3.04	4.49	3.01	3.00	2.87	2.97	3.51	0.84	2.95	0.07	0.84	-0.253	3.1E-01
Mtr.44525.1.S1_at	8.16	6.81	8.36	8.59	8.79	8.47	7.78	0.84	8.62	0.16	1.11	0.148	1.7E-01
Mtr.32226.1.S1_at	5.88	4.33	5.70	6.23	6.10	6.42	5.30	0.84	6.25	0.16	1.18	0.238	1.3E-01
Mtr.1706.1.S1_s_at	10.00	8.31	9.19	9.56	8.95	9.50	9.17	0.84	9.34	0.34	1.02	0.026	7.7E-01
Mtr.29292.1.S1_at	6.83	5.35	6.79	6.78	6.95	6.75	6.32	0.84	6.83	0.11	1.08	0.111	3.6E-01
Mtr.40774.1.S1_s_at	6.86	5.99	7.68	6.88	7.28	6.80	6.85	0.84	6.99	0.26	1.02	0.029	8.0E-01
Mtr.34626.1.S1_at	4.79	3.48	5.06	4.11	4.40	4.69	4.44	0.84	4.40	0.29	0.99	-0.014	9.4E-01
Mtr.45067.1.S1_at	7.90	6.39	7.80	8.17	7.86	7.92	7.36	0.84	7.99	0.16	1.08	0.117	2.8E-01
Mtr.39991.1.S1_at	6.19	4.63	5.96	5.95	5.51	5.77	5.59	0.84	5.74	0.22	1.03	0.039	7.8E-01
Mtr.4745.1.S1_at	7.62	6.39	8.00	7.64	7.70	7.71	7.33	0.84	7.68	0.04	1.05	0.067	5.1E-01
Mtr.39403.1.S1_at	7.29	5.86	7.35	7.25	7.37	7.27	6.83	0.84	7.30	0.06	1.07	0.095	3.9E-01
Mtr.23576.1.S1_at	2.85	4.30	2.82	2.82	2.76	2.81	3.32	0.84	2.79	0.03	0.84	-0.251	3.4E-01
Mtr.36072.1.S1_at	3.82	5.25	3.77	3.75	3.23	3.65	4.28	0.84	3.54	0.28	0.83	-0.273	2.2E-01
Mtr.45529.1.S1_at	5.34	3.66	4.46	5.17	5.04	5.19	4.49	0.84	5.13	0.08	1.14	0.194	2.6E-01
Mtr.44505.1.S1_at	4.95	3.58	3.41	3.93	3.34	3.39	3.98	0.84	3.55	0.33	0.89	-0.163	4.6E-01
Mtr.5339.1.S1_at	4.34	3.78	5.44	3.89	4.43	4.17	4.52	0.84	4.16	0.27	0.92	-0.119	5.2E-01
Mtr.24337.1.S1_s_at	2.99	4.18	2.55	2.54	2.60	2.63	3.24	0.84	2.59	0.05	0.80	-0.320	2.6E-01
Mtr.16822.1.S1_at	10.70	9.06	10.21	10.28	10.06	10.37	9.99	0.84	10.23	0.16	1.02	0.035	6.5E-01
Mtr.6956.1.S1_at	6.72	5.39	6.95	6.62	6.90	7.16	6.35	0.84	6.89	0.27	1.08	0.118	3.5E-01
Mtr.12904.1.S1_at	9.04	7.36	8.28	8.88	8.92	8.93	8.22	0.84	8.91	0.02	1.08	0.116	2.3E-01
Mtr.314.1.S1_at	7.06	6.36	8.03	7.73	7.94	7.40	7.15	0.84	7.69	0.27	1.08	0.106	3.5E-01
Mtr.38865.1.S1_at	8.50	7.08	8.58	8.11	8.29	8.12	8.05	0.84	8.17	0.11	1.01	0.021	8.2E-01
Mtr.27411.1.S1_s_at	8.01	6.58	8.05	7.89	7.57	7.62	7.55	0.84	7.69	0.18	1.02	0.028	7.8E-01
Mtr.43371.1.S1_at	14.52	13.25	12.92	13.71	13.50	13.92	13.56	0.84	13.71	0.21	1.01	0.016	7.8E-01
Mtr.7058.1.S1_at	8.50	6.93	8.25	8.14	8.43	8.36	7.89	0.84	8.31	0.15	1.05	0.074	4.5E-01
Mtr.22192.1.S1_at	2.42	3.86	2.38	2.38	2.36	2.35	2.89	0.84	2.37	0.01	0.82	-0.287	3.4E-01
Mtr.31647.1.S1_at	3.00	3.57	4.65	3.87	3.50	3.78	3.74	0.84	3.72	0.20	0.99	-0.010	9.6E-01
Mtr.33444.1.S1_at	7.67	9.21	7.87	7.79	7.80	7.94	8.25	0.84	7.84	0.08	0.95	-0.073	4.5E-01
Mtr.36923.1.S1_at	2.63	4.09	2.63	2.62	2.61	2.60	3.12	0.84	2.61	0.01	0.84	-0.255	3.6E-01
Mtr.48327.1.S1_at	3.63	2.81	4.49	2.89	3.44	3.08	3.64	0.84	3.14	0.28	0.86	-0.214	3.8E-01
Mtr.42755.1.S1_at	6.56	5.25	6.82	6.82	7.01	6.66	6.21	0.84	6.83	0.17	1.10	0.138	2.8E-01
Mtr.24508.1.S1_at	5.35	5.21	6.73	5.78	5.82	5.79	5.76	0.84	5.79	0.02	1.01	0.007	9.5E-01
Mtr.9810.1.S1_at	4.86	3.46	4.98	4.55	5.01	4.44	4.43	0.84	4.67	0.30	1.05	0.074	6.7E-01
Mtr.22299.1.S1_at	7.04	5.55	6.96	6.96	7.42	6.85	6.52	0.84	7.08	0.30	1.09	0.119	3.4E-01
Mtr.42054.1.S1_at	7.86	6.61	8.20	7.79	8.15	7.83	7.56	0.84	7.93	0.19	1.05	0.069	5.0E-01
Mtr.21816.1.S1_at	4.25	3.67	2.60	3.74	3.75	3.62	3.51	0.84	3.70	0.07	1.06	0.079	7.1E-01
Mtr.29610.1.S1_at	6.11	5.34	7.02	6.34	6.31	5.78	6.16	0.84	6.14	0.31	1.00	-0.004	9.8E-01
Mtr.10169.1.S1_at	5.67	4.38	5.96	5.78	5.67	5.67	5.34	0.84	5.71	0.06	1.07	0.096	4.9E-01
Mtr.36518.1.S1_at	2.61	2.07	3.72	6.58	6.59	7.09	2.80	0.84	6.75	0.29	2.41	1.271	1.5E-03
Mtr.40035.1.S1_at	9.21	7.54	8.54	8.21	8.53	7.85	8.43	0.84	8.20	0.34	0.97	-0.040	6.8E-01
Mtr.2053.1.S1_at	7.01	5.39	6.57	6.42	6.62	6.52	6.33	0.84	6.52	0.10	1.03	0.044	7.1E-01
Mtr.17870.1.S1_at	2.02	3.47	2.01	2.01	2.01	2.00	2.50	0.84	2.00	0.00	0.80	-0.318	3.7E-01
Mtr.24805.1.S1_x_at	2.92	4.37	2.90	2.88	2.86	2.85	3.40	0.84	2.87	0.02	0.84	-0.245	3.4E-01
Mtr.34001.1.S1_at	4.10	2.88	4.49	4.15	3.25	3.43	3.82	0.84	3.61	0.47	0.95	-0.082	7.2E-01
Mtr.37715.1.S1_at	6.92	5.25	6.02	6.45	5.97	6.59	6.06	0.84	6.34	0.32	1.05	0.064	6.2E-01
Mtr.30204.1.S1_at	7.41	5.74	6.60	6.69	6.15	6.59	6.59	0.84	6.48	0.29	0.98	-0.024	8.4E-01
Mtr.3130.1.S1_at	4.70	4.05	5.72	5.35	5.58	4.94	4.83	0.84	5.29	0.32	1.10	0.134	4.2E-01
Mtr.47994.1.S1_s_at	7.04	5.79	7.39	7.06	7.13	6.94	6.74	0.84	7.05	0.09	1.05	0.065	5.6E-01
Mtr.16422.1.S1_x_at	10.22	8.55	9.54	9.80	9.88	10.13	9.44	0.84	9.94	0.17	1.05	0.074	3.7E-01
Mtr.35749.1.S1_at	4.32	2.86	2.88	3.26	2.82	2.72	3.36	0.84	2.93	0.29	0.87	-0.194	4.6E-01
Mtr.38413.1.S1_at	8.25	6.61	7.11	7.40	7.16	7.42	7.32	0.84	7.33	0.15	1.00	0.001	9.9E-01
Mtr.1504.1.S1_at	6.26	4.65	5.84	5.52	6.43	5.80	5.58	0.84	5.92	0.47	1.06	0.084	5.8E-01
Mtr.46211.1.S1_s_at	7.46	6.10	7.63	6.63	7.10	6.80	7.07	0.84	6.84	0.24	0.97	-0.046	6.8E-01
Mtr.44101.1.S1_at	7.91	6.60	8.16	7.96	7.82	7.86	7.56	0.84	7.88	0.07	1.04	0.061	5.4E-01
Mtr.24920.1.S1_at	12.69	11.68	13.34	13.23	13.14	13.01	12.57	0.84	13.13	0.11	1.04	0.062	3.2E-01
Mtr.38254.1.S1_at	8.88	7.25	7.74	7.98	7.32	7.79	7.96	0.84	7.70	0.34	0.97	-0.048	6.4E-01
Mtr.190.1.S1_at	8.00	6.38	7.58	7.77	7.36	7.21	7.32	0.84	7.45	0.29	1.02	0.025	8.2E-01
Mtr.42963.1.S1_at	5.71	4.73	6.40	5.70	5.88	5.28	5.62	0.84	5.62	0.31	1.00	0.001	9.9E-01
Mtr.4341.1.S1_at	5.83	4.19	5.31	5.25	5.20	5.24	5.11	0.84	5.23	0.03	1.02	0.033	8.2E-01
Mtr.20203.1.S1_s_at	11.91	10.32	11.57	11.51	11.44	11.67	11.27	0.84	11.54	0.12	1.02	0.035	6.1E-01
Mtr.11226.1.S1_at	9.04	10.60	9.29	9.52	9.68	9.68	9.65	0.84	9.63	0.09	1.00	-0.003	9.7E-01
Mtr.44992.1.S1_s_at	7.03	5.66	7.17	6.15	6.35	5.83	6.62	0.84	6.11	0.26	0.92	-0.116	3.7E-01
Mtr.42936.1.S1_at	11.23	10.01	11.61	11.96	12.18	11.89	10.95	0.84	12.01	0.15	1.10	0.133	9.8E-02
Mtr.27873.1.S1_at	9.13	7.52	8.74	8.79	8.58	8.84	8.46	0.84	8.74	0.13	1.03	0.046	6.1E-01
Mtr.41123.1.S1_at	7.79	6.44	7.97	8.16	8.10	7.89	7.40	0.84	8.05	0.14	1.09	0.121	2.6E-01
Mtr.9284.1.S1_at	7.76	6.48	8.05	8.33	8.10	8.18	7.43	0.84	8.20	0.12	1.10	0.143	1.9E-01
Mtr.31885.1.S1_at	4.40	2.91	2.99	3.36	2.82	3.06	3.44	0.84	3.08	0.27	0.90	-0.158	5.2E-01
Mtr.37599.1.S1_at	3.85	2.29	3.59	3.59	3.59	4.54	3.24	0.84	3.91	0.55	1.20	0.269	3.1E-01
Mtr.7375.1.S1_at	7.60	6.01	7.28	7.62	7.42	7.56	6.96	0.84	7.53	0.10	1.08	0.113	3.1E-01
Mtr.42115.1.S1_at	2.39	3.83	2.37	2.28	2.44	2.36	2.86	0.84	2.36	0.08	0.82	-0.281	3.6E-01
Mtr.45205.1.S1_at	3.67	2.46	4.06	2.40	2.96	2.45	3.40	0.84	2.60	0.31	0.77	-0.385	2.0E-01
Mtr.32213.1.S1_at	9.99	8.53	9.97	10.84	10.80	10.91	9.49	0.84	10.85	0.06	1.14	0.193	4.9E-02
Mtr.2000.1.S1_at	8.08	6.87	8.47	8.33	8.61	8.17	7.81	0.84	8.37	0.22	1.07	0.100	3.2E-01
Mtr.5968.1.S1_at	10.23	8.57	9.19	9.56	9.58	9.27	9.33	0.84	9.47	0.17	1.02	0.022	7.9E-01
Mtr.18040.1.S1_at	7.27	8.68	8.75	10.36	11.00	10.29	8.23	0.84	10.55	0.39	1.28	0.358	1.2E-02
Mtr.42419.1.S1_at	7.73	6.31	7.79	7.94	8.05	8.05	7.28	0.84	8.01	0.06	1.10	0.139	2.0E-01
Mtr.46318.1.S1_at	8.78	7.18	8.39	8.28	8.49	8.53	8.12	0.84	8.43	0.13	1.04	0.056	5.5E-01
Mtr.39746.1.S1_at	7.18	6.09	7.73	7.18	7.32	7.28	7.00	0.84	7.26	0.07	1.04	0.052	6.2E-01
Mtr.51355.1.S1_at	3.98	3.32	4.98	4.57	4.12	4.41	4.09	0.84	4.37	0.23	1.07	0.092	6.2E-01
Mtr.44468.1.S1_at	6.60	4.94	5.60	5.74	5.77	5.74	5.71	0.84	5.75	0.02	1.01	0.010	9.4E-01
Mtr.9807.1.S1_at	3.29	4.27	4.96	4.43	4.49	3.18	4.17	0.84	4.03	0.74	0.97	-0.049	8.4E-01
Mtr.17356.1.S1_at	6.26	5.17	6.81	6.15	6.57	6.16	6.08	0.84	6.30	0.24	1.04	0.051	6.9E-01
Mtr.38375.1.S1_at	4.77	6.01	6.37	7.70	8.29	7.61	5.71	0.84	7.87	0.37	1.38	0.461	1.5E-02
Mtr.4412.1.S1_at	7.57	6.10	7.52	7.82	7.47	7.40	7.07	0.84	7.56	0.23	1.07	0.098	3.8E-01
Mtr.28697.1.S1_at	8.00	6.81	8.43	7.66	7.93	7.57	7.75	0.84	7.72	0.19	1.00	-0.005	9.6E-01
Mtr.42641.1.S1_s_at	9.33	7.99	9.52	9.57	9.43	9.55	8.95	0.84	9.52	0.07	1.06	0.089	3.1E-01
Mtr.40694.1.S1_at	7.28	5.62	6.65	6.94	6.78	6.75	6.52	0.84	6.82	0.10	1.05	0.066	5.7E-01
Mtr.13958.1.S1_at	9.79	9.68	8.29	9.17	9.19	9.59	9.25	0.84	9.32	0.24	1.01	0.010	9.0E-01
Mtr.35588.1.S1_at	2.37	3.90	2.56	2.37	2.37	2.39	2.95	0.84	2.38	0.01	0.81	-0.310	3.0E-01
Mtr.31402.1.S1_at	3.93	2.34	3.59	4.00	4.01	4.00	3.29	0.84	4.00	0.01	1.22	0.285	2.1E-01
Mtr.32841.1.S1_at	6.09	5.07	6.73	6.53	6.20	6.59	5.96	0.83	6.44	0.21	1.08	0.111	3.9E-01
Mtr.40848.1.S1_at	6.13	5.46	7.12	5.96	6.00	5.44	6.24	0.83	5.80	0.31	0.93	-0.104	4.5E-01
Mtr.9528.1.S1_at	6.92	5.27	6.36	6.72	6.85	6.42	6.18	0.83	6.66	0.22	1.08	0.108	3.9E-01
Mtr.4451.1.S1_s_at	4.40	2.85	3.08	4.72	5.48	5.15	3.45	0.83	5.12	0.38	1.48	0.570	3.5E-02
Mtr.5488.1.S1_at	8.12	6.62	8.01	8.17	8.58	8.05	7.58	0.83	8.27	0.28	1.09	0.125	2.5E-01
Mtr.11921.1.S1_at	7.02	5.57	7.01	7.03	6.98	6.91	6.53	0.83	6.98	0.06	1.07	0.094	4.1E-01
Mtr.12502.1.S1_at	11.39	9.81	11.06	11.45	10.77	11.32	10.75	0.83	11.18	0.36	1.04	0.057	4.6E-01
Mtr.44724.1.S1_at	8.36	6.79	8.08	8.11	7.82	8.43	7.74	0.83	8.12	0.30	1.05	0.068	5.0E-01
Mtr.43418.1.S1_s_at	11.93	10.65	10.37	10.62	10.47	10.75	10.98	0.83	10.62	0.14	0.97	-0.049	4.9E-01
Mtr.36326.1.S1_at	2.42	3.86	2.41	2.41	2.41	2.41	2.90	0.83	2.41	0.00	0.83	-0.266	3.7E-01
Mtr.17483.1.S1_at	8.29	7.30	8.95	8.51	8.72	8.57	8.18	0.83	8.60	0.11	1.05	0.071	4.4E-01
Mtr.13886.1.S1_at	2.49	3.88	2.38	2.49	3.04	2.48	2.92	0.83	2.67	0.32	0.92	-0.127	6.6E-01
Mtr.27885.1.S1_s_at	5.81	4.59	6.19	5.78	5.96	5.62	5.53	0.83	5.79	0.17	1.05	0.067	6.2E-01
Mtr.31561.1.S1_at	6.01	4.55	5.97	6.03	5.97	5.98	5.51	0.83	5.99	0.03	1.09	0.121	3.7E-01
Mtr.28238.1.S1_at	10.20	8.95	8.62	9.07	9.41	9.61	9.25	0.83	9.36	0.27	1.01	0.016	8.4E-01
Mtr.33254.1.S1_at	9.03	8.89	7.52	7.94	8.06	8.20	8.48	0.83	8.07	0.13	0.95	-0.072	4.5E-01
Mtr.22603.1.S1_at	9.22	8.04	9.64	9.25	8.68	8.86	8.97	0.83	8.93	0.29	1.00	-0.006	9.5E-01
Mtr.12839.1.S1_at	8.91	7.37	8.68	8.55	8.65	8.61	8.32	0.83	8.60	0.05	1.03	0.048	5.9E-01
Mtr.39310.1.S1_at	7.76	6.37	7.86	7.87	7.75	7.80	7.33	0.83	7.81	0.06	1.06	0.090	3.8E-01
Mtr.16620.1.S1_at	4.33	3.01	4.55	3.65	3.54	4.12	3.96	0.83	3.77	0.31	0.95	-0.071	7.3E-01
Mtr.37463.1.S1_at	7.93	6.36	7.64	7.80	7.44	8.03	7.31	0.83	7.75	0.30	1.06	0.085	4.3E-01

Mtr.37043.1.S1_at	5.54	5.01	6.64	6.94	6.90	7.20	5.73	0.83	7.01	0.17	1.22	0.292	5.9E-02
Mtr.39849.1.S1_at	7.94	6.33	7.51	7.96	7.83	7.17	7.26	0.83	7.65	0.42	1.05	0.075	5.1E-01
Mtr.8895.1.S1_at	6.87	5.20	5.97	6.19	6.18	6.27	6.01	0.83	6.21	0.05	1.03	0.047	7.0E-01
Mtr.6646.1.S1_s_at	8.80	7.15	7.80	8.13	8.35	8.29	7.92	0.83	8.26	0.12	1.04	0.060	5.2E-01
Mtr.2995.1.S1_at	2.22	3.65	2.20	2.20	2.20	2.19	2.69	0.83	2.19	0.00	0.82	-0.294	3.6E-01
Mtr.41162.1.S1_at	5.58	4.24	4.06	4.04	4.02	3.65	4.63	0.83	3.90	0.22	0.84	-0.245	2.2E-01
Mtr.27851.1.S1_at	5.14	3.49	4.51	4.49	4.70	4.42	4.38	0.83	4.54	0.14	1.04	0.051	7.6E-01
Mtr.6645.1.S1_at	11.08	9.47	10.63	10.81	10.64	10.82	10.39	0.83	10.76	0.10	1.03	0.050	4.9E-01
Mtr.33107.1.S1_at	7.79	6.67	8.29	7.84	7.98	7.08	7.58	0.83	7.64	0.48	1.01	0.010	9.3E-01
Mtr.48835.1.S1_at	2.72	4.15	2.70	2.67	2.67	2.66	3.19	0.83	2.67	0.00	0.84	-0.256	3.4E-01
Mtr.969.1.S1_s_at	8.79	7.16	8.27	8.54	8.56	8.22	8.07	0.83	8.44	0.19	1.05	0.064	5.0E-01
Mtr.28490.1.S1_at	8.86	7.21	8.20	8.59	8.66	8.71	8.09	0.83	8.65	0.06	1.07	0.097	3.1E-01
Mtr.38743.1.S1_at	3.49	2.88	4.52	3.48	3.20	3.22	3.63	0.83	3.30	0.16	0.91	-0.140	5.3E-01
Mtr.40412.1.S1_at	6.47	5.72	4.81	6.85	6.77	6.45	5.67	0.83	6.69	0.21	1.18	0.239	1.1E-01
Mtr.10573.1.S1_at	12.32	11.74	10.68	11.63	11.62	11.58	11.58	0.83	11.61	0.03	1.00	0.004	9.5E-01
Mtr.51572.1.S1_at	3.46	4.88	3.42	3.45	3.45	3.45	3.92	0.83	3.45	0.00	0.88	-0.184	3.8E-01
Mtr.47584.1.S1_at	7.04	5.80	5.46	6.30	6.67	6.41	6.10	0.83	6.46	0.19	1.06	0.082	5.1E-01
Mtr.39573.1.S1_at	4.41	4.04	5.63	4.98	4.85	4.71	4.69	0.83	4.85	0.14	1.03	0.046	7.7E-01
Mtr.5028.1.S1_at	8.04	6.42	7.53	7.64	7.84	8.01	7.33	0.83	7.83	0.19	1.07	0.095	3.7E-01
Mtr.16403.1.S1_at	2.63	4.05	2.59	2.77	2.59	2.59	3.09	0.83	2.65	0.11	0.86	-0.222	4.1E-01
Mtr.40919.1.S1_at	7.74	6.14	7.32	7.58	7.45	7.35	7.06	0.83	7.46	0.11	1.06	0.079	4.6E-01
Mtr.29340.1.S1_at	4.51	3.07	3.07	3.11	3.05	2.91	3.55	0.83	3.02	0.10	0.85	-0.232	3.4E-01
Mtr.44533.1.S1_at	3.76	3.41	4.99	3.51	3.86	3.65	4.06	0.83	3.67	0.18	0.91	-0.143	4.8E-01
Mtr.25333.1.S1_at	4.47	4.12	5.70	4.69	5.86	5.02	4.76	0.83	5.19	0.60	1.09	0.123	5.1E-01
Mtr.47445.1.S1_at	4.18	2.68	4.05	4.04	4.04	4.04	3.64	0.83	4.04	0.00	1.11	0.152	4.5E-01
Mtr.33459.1.S1_s_at	2.71	2.77	4.17	2.70	2.69	2.69	3.22	0.83	2.69	0.00	0.84	-0.255	3.4E-01
Mtr.35665.1.S1_at	13.65	12.92	11.99	11.79	11.47	11.87	12.85	0.83	11.71	0.21	0.91	-0.134	8.2E-02
Mtr.43615.1.S1_at	10.42	8.80	9.90	10.45	10.06	10.46	9.71	0.83	10.32	0.23	1.06	0.089	2.8E-01
Mtr.27522.1.S1_at	8.67	7.04	8.08	8.54	8.30	8.54	7.93	0.83	8.46	0.14	1.07	0.093	3.4E-01
Mtr.33109.1.S1_s_at	4.99	3.57	5.02	5.19	5.47	4.90	4.53	0.83	5.19	0.28	1.15	0.196	2.6E-01
Mtr.41069.1.S1_at	6.80	5.17	6.24	6.00	6.03	6.21	6.07	0.83	6.08	0.12	1.00	0.003	9.8E-01
Mtr.13010.1.S1_at	9.85	11.50	10.57	11.77	12.12	11.83	10.64	0.83	11.90	0.19	1.12	0.162	6.1E-02
Mtr.4031.1.S1_at	6.24	5.27	6.92	6.12	6.53	6.25	6.14	0.83	6.30	0.21	1.03	0.037	7.7E-01
Mtr.32988.1.S1_at	7.85	6.21	6.82	7.10	6.98	7.19	6.96	0.83	7.09	0.11	1.02	0.026	8.1E-01
Mtr.21093.1.S1_at	2.87	4.29	2.85	2.83	2.79	2.82	3.34	0.83	2.81	0.02	0.84	-0.246	3.4E-01
Mtr.25568.1.S1_at	3.02	4.66	3.67	3.02	2.99	2.99	3.78	0.83	3.00	0.02	0.79	-0.334	1.8E-01
Mtr.45730.1.S1_at	12.18	10.93	10.62	10.51	11.16	10.85	11.24	0.83	10.84	0.32	0.96	-0.053	4.8E-01
Mtr.13805.1.S1_s_at	6.92	6.08	7.73	7.21	7.41	7.29	6.91	0.83	7.30	0.10	1.06	0.080	4.6E-01
Mtr.49557.1.S1_at	10.84	9.52	9.31	9.35	9.53	9.78	9.89	0.83	9.56	0.21	0.97	-0.050	5.3E-01
Mtr.9819.1.S1_at	3.94	2.52	3.95	4.68	4.33	4.51	3.47	0.83	4.51	0.18	1.30	0.378	1.0E-01
Mtr.23103.1.S1_at	6.81	5.73	7.35	6.91	6.76	6.84	6.63	0.83	6.84	0.08	1.03	0.044	6.9E-01
Mtr.46001.1.S1_at	12.09	10.45	11.45	11.17	10.70	11.08	11.33	0.83	10.98	0.25	0.97	-0.045	5.2E-01
Mtr.20473.1.S1_at	9.42	7.78	8.75	8.72	8.52	8.91	8.65	0.83	8.72	0.20	1.01	0.011	9.0E-01
Mtr.23203.1.S1_at	5.66	4.38	5.93	5.65	5.83	5.65	5.33	0.83	5.71	0.10	1.07	0.101	4.6E-01
Mtr.40391.1.S1_at	12.48	11.70	10.83	12.07	12.01	12.39	11.67	0.83	12.16	0.21	1.04	0.059	3.8E-01
Mtr.37199.1.S1_at	9.55	8.26	9.80	9.23	9.56	9.21	9.21	0.83	9.33	0.20	1.01	0.020	8.1E-01
Mtr.52235.1.S1_at	2.97	4.62	3.77	3.73	4.82	3.62	3.79	0.83	4.06	0.66	1.07	0.099	6.8E-01
Mtr.29166.1.S1_at	4.21	3.53	5.17	4.05	4.35	3.85	4.30	0.83	4.08	0.25	0.95	-0.077	6.8E-01
Mtr.9896.1.S1_s_at	9.47	8.02	9.43	9.27	9.38	9.38	8.97	0.83	9.34	0.06	1.04	0.059	4.8E-01
Mtr.39447.1.S1_at	8.73	7.34	8.80	9.26	9.44	9.18	8.29	0.83	9.29	0.13	1.12	0.165	1.1E-01
Mtr.13362.1.S1_at	7.08	5.59	6.95	7.38	7.46	7.66	6.54	0.83	7.50	0.14	1.15	0.197	1.2E-01
Mtr.5900.1.S1_at	2.42	3.83	2.38	2.38	2.38	2.37	2.87	0.83	2.38	0.00	0.83	-0.275	3.5E-01
Mtr.5363.1.S1_at	7.33	6.41	8.06	7.22	7.36	7.05	7.27	0.83	7.21	0.16	0.99	-0.011	9.1E-01
Mtr.32662.1.S1_at	9.72	9.05	8.08	8.29	8.34	8.68	8.95	0.83	8.44	0.21	0.94	-0.085	3.6E-01
Mtr.11541.1.S1_at	7.30	6.28	7.91	8.04	7.73	7.45	7.16	0.83	7.74	0.29	1.08	0.111	3.2E-01
Mtr.24792.1.S1_at	2.15	3.57	2.14	2.14	2.14	2.13	2.62	0.82	2.14	0.00	0.81	-0.296	3.6E-01
Mtr.12424.1.S1_at	8.39	9.75	8.27	8.72	9.61	9.47	8.80	0.82	9.27	0.48	1.05	0.074	4.5E-01
Mtr.51237.1.S1_at	7.11	5.94	7.53	7.02	7.24	6.89	6.86	0.82	7.05	0.18	1.03	0.040	7.1E-01
Mtr.42823.1.S1_at	6.40	4.94	6.32	5.99	5.72	5.93	5.89	0.82	5.88	0.14	1.00	-0.002	9.9E-01
Mtr.40893.1.S1_s_at	7.36	5.81	7.07	7.38	7.00	7.48	6.75	0.82	7.29	0.25	1.08	0.112	3.4E-01
Mtr.12910.1.S1_at	8.85	7.28	8.49	8.23	8.86	8.28	8.21	0.82	8.46	0.35	1.03	0.043	6.5E-01
Mtr.2032.1.S1_at	8.29	6.67	7.76	8.05	8.09	8.13	7.57	0.82	8.09	0.04	1.07	0.095	3.4E-01
Mtr.48266.1.S1_at	5.81	4.19	5.25	5.46	4.81	5.27	5.09	0.82	5.18	0.34	1.02	0.026	8.6E-01
Mtr.1343.1.S1_at	5.39	4.23	5.83	5.43	6.15	6.29	5.15	0.82	5.96	0.46	1.16	0.210	2.1E-01
Mtr.18970.1.S1_at	2.75	4.17	2.73	2.73	2.71	2.71	3.22	0.82	2.72	0.01	0.84	-0.245	3.5E-01
Mtr.28089.1.S1_at	9.67	8.04	9.08	9.50	9.25	9.35	8.93	0.82	9.37	0.13	1.05	0.069	4.1E-01
Mtr.39285.1.S1_at	5.80	4.64	6.23	6.23	6.50	6.12	5.56	0.82	6.28	0.19	1.13	0.176	2.1E-01
Mtr.41747.1.S1_at	5.81	4.34	5.72	5.68	5.70	6.10	5.29	0.82	5.83	0.24	1.10	0.140	3.4E-01
Mtr.48910.1.S1_s_at	2.60	4.00	2.56	2.54	2.54	2.53	3.05	0.82	2.54	0.01	0.83	-0.266	3.4E-01
Mtr.39723.1.S1_at	3.80	5.42	4.84	3.99	4.45	4.67	4.69	0.82	4.37	0.35	0.93	-0.101	5.7E-01
Mtr.40578.1.S1_at	7.34	5.90	7.32	7.20	7.16	6.85	6.85	0.82	7.07	0.19	1.03	0.045	6.8E-01
Mtr.24662.1.S1_at	7.26	5.63	6.28	6.88	7.17	6.69	6.39	0.82	6.91	0.24	1.08	0.114	3.5E-01
Mtr.40255.1.S1_at	11.39	9.78	10.88	10.86	11.19	11.06	10.68	0.82	11.03	0.16	1.03	0.047	5.1E-01
Mtr.4549.1.S1_at	7.92	6.39	7.68	7.91	7.85	7.81	7.33	0.82	7.86	0.05	1.07	0.100	3.3E-01
Mtr.42858.1.S1_at	10.94	10.45	9.34	9.45	10.41	10.26	10.24	0.82	10.04	0.52	0.98	-0.029	7.3E-01
Mtr.27849.1.S1_at	8.42	6.78	7.61	8.93	8.65	8.93	7.60	0.82	8.83	0.16	1.16	0.217	6.4E-02
Mtr.44282.1.S1_at	9.54	8.03	9.35	9.17	9.10	9.16	8.97	0.82	9.14	0.04	1.02	0.027	7.4E-01
Mtr.6108.1.S1_at	5.58	3.99	5.16	5.51	5.73	5.40	4.91	0.82	5.55	0.17	1.13	0.176	2.6E-01
Mtr.18322.1.S1_s_at	6.75	5.17	5.55	5.78	5.88	5.86	5.82	0.82	5.84	0.05	1.00	0.004	9.8E-01
Mtr.49988.1.S1_at	2.37	2.41	3.81	2.36	2.30	2.35	2.86	0.82	2.34	0.03	0.82	-0.292	3.3E-01
Mtr.41555.1.S1_at	4.22	3.56	5.19	4.07	4.19	4.32	4.32	0.82	4.19	0.13	0.97	-0.045	8.0E-01
Mtr.4222.1.S1_at	7.70	6.16	7.42	7.44	7.42	7.23	7.10	0.82	7.36	0.12	1.04	0.054	6.0E-01
Mtr.5980.1.S1_at	11.07	9.47	10.61	11.00	10.98	11.09	10.38	0.82	11.02	0.06	1.06	0.086	2.5E-01
Mtr.42106.1.S1_at	6.35	4.85	6.16	5.53	5.80	5.68	5.79	0.82	5.67	0.14	0.98	-0.030	8.2E-01
Mtr.16157.1.S1_at	3.42	1.78	2.75	3.56	3.18	3.18	2.65	0.82	3.31	0.22	1.25	0.319	2.5E-01
Mtr.12100.1.S1_at	7.77	6.44	7.94	8.02	7.98	8.26	7.39	0.82	8.08	0.15	1.09	0.130	2.2E-01
Mtr.20281.1.S1_at	6.69	5.42	6.96	7.13	6.79	6.78	6.36	0.82	6.90	0.20	1.08	0.117	3.3E-01
Mtr.18643.1.S1_at	10.77	9.30	10.66	11.18	11.55	11.29	10.24	0.82	11.34	0.19	1.11	0.147	8.7E-02
Mtr.26041.1.S1_at	8.36	6.82	8.07	7.97	8.00	8.20	7.75	0.82	8.06	0.13	1.04	0.057	5.5E-01
Mtr.4595.1.S1_at	4.13	3.57	5.19	3.58	3.63	3.63	4.30	0.82	3.61	0.03	0.84	-0.250	2.2E-01
Mtr.33500.1.S1_at	6.12	4.66	6.03	5.59	5.37	6.09	5.60	0.82	5.69	0.37	1.02	0.022	8.8E-01
Mtr.41043.1.S1_at	8.55	7.04	8.35	8.38	8.51	8.48	7.98	0.82	8.45	0.07	1.06	0.083	3.8E-01
Mtr.48721.1.S1_at	4.20	2.64	3.87	3.86	4.07	3.54	3.57	0.82	3.82	0.27	1.07	0.101	6.3E-01
Mtr.30896.1.S1_at	2.48	3.90	2.47	2.30	2.47	2.47	2.95	0.82	2.41	0.10	0.82	-0.291	3.2E-01
Mtr.28009.1.S1_at	3.72	3.77	5.16	3.66	4.32	4.21	4.22	0.82	4.06	0.36	0.96	-0.054	7.8E-01
Mtr.39838.1.S1_at	5.91	4.73	6.30	6.50	6.15	6.24	5.65	0.82	6.30	0.18	1.12	0.157	2.5E-01
Mtr.25013.1.S1_at	6.08	7.61	7.34	6.27	6.94	6.89	7.01	0.82	6.70	0.37	0.96	-0.065	5.8E-01
Mtr.44900.1.S1_at	3.62	4.90	3.38	4.06	4.34	3.58	3.97	0.82	3.99	0.39	1.01	0.010	9.6E-01
Mtr.5308.1.S1_at	3.72	2.15	3.33	3.33	4.26	3.33	3.07	0.82	3.64	0.53	1.19	0.246	3.7E-01
Mtr.5017.1.S1_at	1.93	3.35	1.92	1.92	1.92	1.92	2.40	0.82	1.92	0.00	0.80	-0.323	3.7E-01
Mtr.17384.1.S1_at	4.68	3.59	5.20	4.75	4.63	4.46	4.49	0.82	4.61	0.15	1.03	0.038	8.1E-01
Mtr.45601.1.S1_s_at	9.55	10.66	11.14	10.40	10.86	9.70	10.45	0.82	10.32	0.58	0.99	-0.018	8.3E-01
Mtr.39769.1.S1_at	4.25	2.84	4.26	4.34	4.72	4.44	3.78	0.82	4.50	0.20	1.19	0.251	2.1E-01
Mtr.41788.1.S1_s_at	8.71	7.55	9.13	8.81	9.11	8.81	8.46	0.82	8.91	0.18	1.05	0.075	4.1E-01
Mtr.36194.1.S1_at	5.02	3.46	4.68	4.11	5.04	5.02	4.39	0.82	4.72	0.53	1.08	0.107	5.8E-01
Mtr.1706.1.S1_at	2.56	4.16	3.06	2.86	2.87	2.82	3.26	0.82	2.85	0.03	0.87	-0.196	4.3E-01
Mtr.21006.1.S1_at	11.85	10.21	11.01	11.10	10.49	10.93	11.03	0.82	10.84	0.31	0.98	-0.024	7.3E-01
Mtr.41408.1.S1_at	4.17	3.20	4.83	4.71	4.89	3.78	4.07	0.82	4.46	0.60	1.10	0.134	5.4E-01
Mtr.16894.1.S1_at	9.29	8.86	7.70	8.75	8.21	8.92	8.62	0.82	8.63	0.37	1.00	0.002	9.8E-01
Mtr.44545.1.S1_at	9.38	7.92	9.28	9.16	8.20	8.63	8.86	0.82	8.66	0.48	0.98	-0.033	7.4E-01
Mtr.46651.1.S1_x_at	7.63	9.26	8.56	8.47	8.60	8.21	8.48	0.82	8.43	0.20	0.99	-0.010	9.1E-01
Mtr.9011.1.S1_at	7.71	8.10	9.28	8.40	8.68	8.00	8.37	0.82	8.36	0.34	1.00	-0.001	9.9E-01
Mtr.2606.1.S1_at	4.20	2.70	2.87	2.85	2.84	2.77	3.26	0.82	2.82	0.04	0.87	-0.208	4.1E-01
Mtr.45167.1.S1_at	7.31	5.83	7.16	7.53	7.51	7.72	6.77	0.82	7.59	0.12	1.12	0.165	1.6E-01
Mtr.43280.1.S1_at	7.28	5.74	6.02	6.42	6.11	6.59	6.35	0.82	6.37	0.25	1.00	0.007	9.6E-01
Mtr.46020.1.S1_at	8.42	7.32	8.92	8.64	8.96	8.60	8.22	0.82	8.73	0.20	1.06	0.088	3.5E-01
Mtr.33420.1.S1_at	9.02	7.39	8.15	8.57	8.58	8.80	8.19	0.82	8.65	0.13	1.06	0.078	3.9E-01
Mtr.8410.1.S1_at	10.03	8.43	9.54	9.52	9.03	9.65	9.34	0.82	9.40	0.33	1.01	0.010	9.1E-01
Mtr.33912.1.S1_at	6.16	5.44	7.07	6.46	6.60	6.46	6.22	0.82	6.51	0.08	1.05	0.064	5.8E-01
Mtr.7534.1.S1_at	7.12	5.48	6.30	6.93	6.99	7.09	6.30	0.82	7.00	0.08	1.11	0.153	2.1E-01
Mtr.5181.1.S1_s_at	6.03	4.67	6.14	5.85	6.33	5.08	5.61	0.82	5.75	0.63	1.03	0.036	8.2E-01
Mtr.12858.1.S1_at	8.08	6.79	8.31	8.08	8.09	7.87	7.73	0.82	8.01	0.13	1.04	0.052	5.8E-01
Mtr.19411.1.S1_at	7.77	6.29	7.64	7.87	7.55	7.75	7.23	0.82	7.72	0.16	1.07	0.094	3.7E-01
Mtr.38815.1.S1_at	12.36	10.94	10.96	11.25	11.58	11.60	11.42	0.82	11.47	0.20	1.00	0.007	9.2E-01
Mtr.49361.1.S1_at	9.84	8.28	9.46	9.65	9.51	9.61	9.19	0.82	9.59	0.07	1.04	0.061	4.5E-01
Mtr.29022.1.S1_at	6.66	5.41	6.94	6.58	6.75	6.30	6.34	0.82	6.54	0.23	1.03	0.046	7.0E-01
Mtr.31638.1.S1_at	7.78	6.39	7.82	7.97	7.82	8.06	7.33	0.82	7.95	0.12	1.08	0.117	2.7E-01
Mtr.11681.1.S1_at	8.02	6.39	7.17	7.55	7.56	7.55	7.19	0.82	7.55	0.01	1.05	0.070	4.9E-01
Mtr.37045.1.S1_at	8.55	7.03	8.31	8.37	8.70	8.32	7.96	0.82	8.46	0.21	1.06	0.087	3.6E-01
Mtr.45505.1.S1_at	4.78	3.37	4.79	4.58	5.18	4.83	4.31	0.82	4.86	0.30	1.13	0.174	3.3E-01
Mtr.28024.1.S1_at	2.87	2.92	4.31	3.17	3.04	2.76	3.37	0.82	2.99	0.20	0.89	-0.171	4.8E-01

Mtr.16200.1.S1_x_at	9.21	7.79	9.20	8.94	8.82	8.78	8.73	0.82	8.85	0.08	1.01	0.019	8.2E-01
Mtr.5271.1.S1_at	6.57	5.60	7.22	7.22	7.79	7.38	6.47	0.82	7.46	0.30	1.15	0.207	1.2E-01
Mtr.18805.1.S1_at	10.53	11.89	10.43	11.05	11.05	10.93	10.95	0.82	11.01	0.07	1.01	0.008	9.0E-01
Mtr.15414.1.S1_at	6.90	5.83	7.43	7.14	6.82	6.82	6.72	0.82	6.93	0.18	1.03	0.044	6.9E-01
Mtr.37895.1.S1_at	8.27	6.73	7.95	8.16	8.17	8.15	7.65	0.82	8.16	0.01	1.07	0.092	3.4E-01
Mtr.38714.1.S1_at	9.22	7.59	8.43	9.04	8.69	8.89	8.41	0.81	8.87	0.17	1.05	0.077	3.9E-01
Mtr.33428.1.S1_at	7.05	5.50	6.72	6.75	6.82	6.71	6.42	0.81	6.76	0.05	1.05	0.074	5.1E-01
Mtr.14667.1.S1_at	8.77	7.26	8.53	7.95	7.95	8.31	8.19	0.81	8.07	0.21	0.99	-0.021	8.2E-01
Mtr.31310.1.S1_at	7.20	5.97	7.51	7.41	7.49	7.19	6.90	0.81	7.36	0.15	1.07	0.094	3.9E-01
Mtr.43767.1.S1_at	12.21	11.86	10.65	11.64	11.65	12.12	11.57	0.81	11.80	0.27	1.02	0.029	6.7E-01
Mtr.3669.1.S1_at	7.65	6.30	7.76	7.31	7.55	7.14	7.24	0.81	7.33	0.21	1.01	0.019	8.5E-01
Mtr.48759.1.S1_at	3.09	4.45	2.99	2.99	3.02	3.24	3.51	0.81	3.08	0.13	0.88	-0.187	4.2E-01
Mtr.42451.1.S1_at	4.43	5.83	4.41	4.12	4.25	4.65	4.89	0.81	4.34	0.28	0.89	-0.174	3.3E-01
Mtr.49846.1.S1_at	7.10	5.66	7.05	6.85	7.24	6.75	6.60	0.81	6.95	0.26	1.05	0.073	5.3E-01
Mtr.9067.1.S1_s_at	8.07	6.95	8.53	8.37	8.58	8.31	7.85	0.81	8.42	0.14	1.07	0.100	3.0E-01
Mtr.42782.1.S1_s_at	5.26	3.66	4.24	3.97	3.53	3.92	4.39	0.81	3.80	0.24	0.87	-0.205	3.0E-01
Mtr.41251.1.S1_at	9.05	8.18	9.81	9.14	8.82	8.60	9.01	0.81	8.85	0.27	0.98	-0.026	7.6E-01
Mtr.45851.1.S1_x_at	2.65	4.21	3.05	2.61	2.60	2.51	3.30	0.81	2.57	0.06	0.78	-0.360	2.0E-01
Mtr.19606.1.S1_at	8.89	7.34	8.54	8.36	8.52	8.37	8.25	0.81	8.42	0.09	1.02	0.028	7.5E-01
Mtr.48874.1.S1_at	7.65	6.06	7.12	7.28	7.32	7.12	6.94	0.81	7.24	0.10	1.04	0.061	5.6E-01
Mtr.28582.1.S1_at	3.55	2.09	3.44	3.97	3.67	3.53	3.03	0.81	3.72	0.22	1.23	0.299	2.3E-01
Mtr.11381.1.S1_at	4.21	5.63	4.24	4.01	4.60	4.77	4.69	0.81	4.46	0.40	0.95	-0.074	6.8E-01
Mtr.25885.1.S1_s_at	8.55	10.13	8.99	7.36	8.04	7.40	9.22	0.81	7.60	0.38	0.82	-0.279	3.5E-02
Mtr.35086.1.S1_at	8.08	6.74	8.21	8.03	7.95	7.96	7.68	0.81	7.98	0.04	1.04	0.056	5.5E-01
Mtr.8671.1.S1_at	5.26	4.03	5.57	5.22	5.55	5.38	4.96	0.81	5.38	0.16	1.09	0.119	4.2E-01
Mtr.12026.1.S1_at	3.36	4.95	3.85	3.80	4.63	4.00	4.05	0.81	4.14	0.43	1.02	0.031	8.8E-01
Mtr.45088.1.S1_at	9.23	7.95	9.45	9.31	9.11	9.18	8.88	0.81	9.20	0.11	1.04	0.052	5.3E-01
Mtr.20798.1.S1_at	2.70	4.10	2.69	2.69	2.69	2.67	3.16	0.81	2.68	0.01	0.85	-0.239	3.6E-01
Mtr.46837.1.S1_at	7.01	5.50	6.78	6.95	6.79	6.65	6.43	0.81	6.80	0.15	1.06	0.079	4.9E-01
Mtr.13174.1.S1_at	7.62	6.55	8.14	7.47	7.49	7.61	7.44	0.81	7.52	0.07	1.01	0.017	8.6E-01
Mtr.42245.1.S1_at	7.27	5.67	6.70	7.26	7.31	7.49	6.55	0.81	7.36	0.12	1.12	0.167	1.6E-01
Mtr.40408.1.S1_at	8.62	8.96	10.17	9.36	9.38	9.05	9.25	0.81	9.26	0.19	1.00	0.002	9.8E-01
Mtr.22017.1.S1_at	6.07	4.65	6.05	5.98	6.14	6.12	5.59	0.81	6.08	0.08	1.09	0.121	3.6E-01
Mtr.44304.1.S1_at	7.55	6.11	7.49	7.68	7.81	7.99	7.05	0.81	7.82	0.16	1.11	0.150	1.8E-01
Mtr.16077.1.S1_at	8.93	7.39	8.58	8.66	8.80	8.77	8.30	0.81	8.74	0.08	1.05	0.075	4.0E-01
Mtr.28190.1.S1_at	6.92	5.44	6.75	6.75	6.94	6.66	6.37	0.81	6.79	0.14	1.07	0.092	4.3E-01
Mtr.5296.1.S1_at	5.20	3.71	5.02	5.18	5.41	5.42	4.64	0.81	5.34	0.14	1.15	0.201	2.2E-01
Mtr.8144.1.S1_at	2.79	4.19	2.78	2.77	2.76	2.75	3.26	0.81	2.76	0.01	0.85	-0.238	3.5E-01
Mtr.20250.1.S1_s_at	6.67	5.08	6.16	6.13	6.36	6.00	5.97	0.81	6.16	0.18	1.03	0.046	7.1E-01
Mtr.32451.1.S1_s_at	6.96	5.67	7.17	7.25	6.90	6.85	6.60	0.81	7.00	0.22	1.06	0.084	4.6E-01
Mtr.35235.1.S1_at	4.03	3.22	4.85	3.56	4.93	4.49	4.03	0.81	4.33	0.70	1.07	0.101	6.6E-01
Mtr.37166.1.S1_at	6.30	4.72	5.18	5.65	6.15	6.35	5.40	0.81	6.05	0.36	1.12	0.164	2.7E-01
Mtr.8416.1.S1_s_at	8.72	8.49	10.00	9.20	9.19	9.22	9.07	0.81	9.20	0.02	1.01	0.021	7.9E-01
Mtr.34337.1.S1_at	2.20	3.58	2.15	2.21	2.15	2.34	2.65	0.81	2.23	0.10	0.84	-0.245	4.3E-01
Mtr.29583.1.S1_at	6.50	5.19	6.67	6.84	6.75	6.67	6.12	0.81	6.75	0.09	1.10	0.142	2.5E-01
Mtr.34936.1.S1_at	3.05	4.53	3.21	2.97	2.94	2.89	3.59	0.81	2.93	0.04	0.82	-0.294	2.3E-01
Mtr.44056.1.S1_at	8.40	6.86	8.06	8.91	8.86	8.73	7.77	0.81	8.83	0.09	1.14	0.184	8.8E-02
Mtr.9494.1.S1_at	7.15	5.68	7.02	7.04	7.20	6.99	6.62	0.81	7.08	0.11	1.07	0.097	3.8E-01
Mtr.35316.1.S1_at	8.24	9.55	9.73	8.42	8.84	8.71	9.17	0.81	8.66	0.21	0.94	-0.083	3.5E-01
Mtr.14692.1.S1_at	7.22	5.62	6.62	6.41	5.39	6.19	6.49	0.81	6.00	0.54	0.92	-0.114	4.3E-01
Mtr.31871.1.S1_at	6.54	6.55	7.95	7.00	7.26	7.15	7.01	0.81	7.14	0.13	1.02	0.026	8.0E-01
Mtr.19877.1.S1_at	3.80	3.01	2.18	2.98	2.94	3.04	2.99	0.81	2.98	0.05	1.00	-0.005	9.8E-01
Mtr.14578.1.S1_at	6.87	5.53	7.00	6.63	6.59	6.26	6.47	0.81	6.50	0.20	1.00	0.006	9.6E-01
Mtr.42303.1.S1_at	9.30	7.79	9.07	9.12	9.27	9.05	8.72	0.81	9.15	0.11	1.05	0.069	4.2E-01
Mtr.44763.1.S1_at	3.90	2.54	2.46	2.45	2.67	2.45	2.97	0.81	2.53	0.13	0.85	-0.233	4.0E-01
Mtr.10018.1.S1_at	5.16	4.77	6.33	6.92	6.90	6.81	5.42	0.81	6.88	0.06	1.27	0.344	3.6E-02
Mtr.49724.1.S1_at	2.69	4.16	2.84	2.67	2.67	2.66	3.23	0.81	2.67	0.00	0.83	-0.277	2.9E-01
Mtr.22096.1.S1_at	6.15	4.82	6.28	5.89	5.40	6.36	5.75	0.81	5.88	0.48	1.02	0.033	8.2E-01
Mtr.13860.1.S1_at	7.84	6.23	7.23	7.69	7.56	7.87	7.10	0.81	7.71	0.16	1.09	0.118	2.7E-01
Mtr.41335.1.S1_at	6.48	5.27	6.80	6.72	6.79	6.34	6.18	0.81	6.61	0.24	1.07	0.098	4.2E-01
Mtr.52215.1.S1_at	12.45	11.21	10.92	11.42	10.81	11.45	11.53	0.81	11.23	0.36	0.97	-0.038	5.9E-01
Mtr.34636.1.S1_at	10.31	8.97	8.85	8.64	8.34	8.63	9.38	0.81	8.54	0.17	0.91	-0.135	1.5E-01
Mtr.34417.1.S1_at	7.50	5.96	7.17	7.13	7.26	7.02	6.88	0.81	7.14	0.12	1.04	0.054	6.1E-01
Mtr.9205.1.S1_at	4.46	3.24	2.93	3.15	3.35	3.26	3.54	0.81	3.25	0.10	0.92	-0.122	5.8E-01
Mtr.34494.1.S1_at	3.43	2.35	3.93	2.63	2.46	3.47	3.24	0.81	2.85	0.55	0.88	-0.182	5.3E-01
Mtr.35203.1.S1_x_at	7.42	6.43	8.03	7.43	7.69	7.33	7.29	0.81	7.48	0.19	1.03	0.037	7.1E-01
Mtr.14609.1.S1_at	7.68	7.65	9.07	8.36	9.10	8.22	8.13	0.81	8.56	0.47	1.05	0.074	4.7E-01
Mtr.33628.1.S1_at	8.23	6.80	8.19	8.10	8.18	7.68	7.74	0.81	7.99	0.27	1.03	0.046	6.4E-01
Mtr.6807.1.S1_at	7.53	6.38	7.93	7.51	7.80	7.67	7.28	0.81	7.66	0.15	1.05	0.074	4.7E-01
Mtr.13847.1.S1_at	7.72	8.94	7.41	7.65	7.65	7.71	8.02	0.81	7.67	0.03	0.96	-0.065	4.9E-01
Mtr.44933.1.S1_at	4.60	3.09	4.36	4.27	4.28	4.30	4.02	0.81	4.28	0.01	1.07	0.093	6.0E-01
Mtr.12578.1.S1_at	10.04	8.59	8.70	9.42	9.47	9.64	9.11	0.81	9.51	0.12	1.04	0.062	4.4E-01
Mtr.47103.1.S1_at	2.67	4.05	2.63	2.63	2.62	2.61	3.11	0.81	2.62	0.01	0.84	-0.252	3.5E-01
Mtr.7227.1.S1_at	4.07	2.91	4.46	5.08	4.60	4.02	3.81	0.81	4.57	0.53	1.20	0.260	2.5E-01
Mtr.20961.1.S1_at	5.36	6.56	6.90	6.56	6.77	6.46	6.27	0.81	6.60	0.16	1.05	0.073	5.3E-01
Mtr.45753.1.S1_at	3.23	4.63	3.24	3.22	3.34	3.10	3.70	0.81	3.22	0.12	0.87	-0.200	3.7E-01
Mtr.43724.1.S1_at	8.14	6.58	7.01	7.46	7.19	7.45	7.24	0.81	7.37	0.15	1.02	0.025	8.1E-01
Mtr.29604.1.S1_at	2.24	3.63	2.22	2.22	2.22	2.28	2.70	0.81	2.24	0.04	0.83	-0.268	3.8E-01
Mtr.19730.1.S1_at	9.34	7.84	9.10	9.29	9.04	9.21	8.76	0.81	9.18	0.13	1.05	0.068	4.2E-01
Mtr.19422.1.S1_at	11.11	9.83	11.31	10.90	10.74	10.71	10.75	0.81	10.78	0.10	1.00	0.005	9.5E-01
Mtr.40076.1.S1_at	9.78	10.50	8.88	9.56	9.73	9.43	9.72	0.81	9.58	0.15	0.98	-0.022	7.7E-01
Mtr.2175.1.S1_at	7.02	5.58	6.93	6.89	7.17	6.55	6.51	0.81	6.87	0.31	1.06	0.078	5.1E-01
Mtr.52148.1.S1_at	9.40	7.84	8.96	8.30	8.55	8.48	8.73	0.81	8.44	0.13	0.97	-0.049	5.7E-01
Mtr.8289.1.S1_at	4.72	3.50	3.19	4.14	4.60	4.81	3.80	0.81	4.51	0.35	1.19	0.248	2.3E-01
Mtr.19921.1.S1_at	2.34	3.72	2.31	2.31	2.31	2.30	2.79	0.81	2.31	0.00	0.83	-0.276	3.6E-01
Mtr.47916.1.S1_at	8.77	7.56	9.09	8.69	8.74	8.42	8.48	0.81	8.62	0.17	1.02	0.024	7.8E-01
Mtr.24891.1.S1_at	7.98	6.82	8.37	7.97	8.12	7.67	7.72	0.81	7.92	0.23	1.03	0.036	7.1E-01
Mtr.29167.1.S1_at	7.35	6.11	7.63	7.47	7.19	7.35	7.03	0.81	7.33	0.14	1.04	0.061	5.6E-01
Mtr.13487.1.S1_at	6.95	5.55	6.93	7.18	7.22	6.92	6.48	0.81	7.11	0.17	1.10	0.134	2.6E-01
Mtr.28033.1.S1_at	6.86	5.70	5.31	6.74	7.37	7.18	5.96	0.81	7.10	0.33	1.19	0.252	8.6E-02
Mtr.25841.1.S1_at	9.81	11.41	10.43	10.04	10.29	10.11	10.55	0.81	10.14	0.13	0.96	-0.057	4.4E-01
Mtr.36020.1.S1_a_at	2.82	4.21	2.82	2.79	2.75	2.74	3.28	0.81	2.76	0.03	0.84	-0.249	3.3E-01
Mtr.31109.1.S1_at	5.68	4.33	5.77	5.41	5.60	5.55	5.26	0.80	5.52	0.10	1.05	0.070	6.1E-01
Mtr.16517.1.S1_at	2.51	3.90	2.51	2.50	2.50	2.59	2.97	0.80	2.53	0.05	0.85	-0.232	4.0E-01
Mtr.29366.1.S1_at	10.63	9.02	9.76	10.15	10.06	10.19	9.80	0.80	10.13	0.07	1.03	0.048	5.2E-01
Mtr.1889.1.S1_at	9.43	7.83	8.74	8.88	8.75	8.90	8.67	0.80	8.85	0.08	1.02	0.029	7.2E-01
Mtr.36292.1.S1_at	2.86	2.84	4.24	2.93	2.93	2.99	3.31	0.80	2.95	0.04	0.89	-0.166	4.8E-01
Mtr.26988.1.S1_s_at	8.82	7.21	7.94	8.64	8.90	8.64	7.99	0.80	8.73	0.15	1.09	0.128	1.9E-01
Mtr.9709.1.S1_at	9.10	8.30	7.49	8.26	8.19	8.67	8.30	0.80	8.37	0.26	1.01	0.013	8.8E-01
Mtr.27888.1.S1_at	6.62	6.88	8.12	9.50	10.03	9.54	7.21	0.80	9.69	0.29	1.34	0.426	7.4E-03
Mtr.9874.1.S1_at	7.69	6.32	7.73	7.74	7.78	7.82	7.25	0.80	7.78	0.04	1.07	0.102	3.2E-01
Mtr.30914.1.S1_at	3.27	4.58	3.12	2.82	2.80	3.31	3.65	0.80	2.98	0.29	0.82	-0.294	2.4E-01
Mtr.11829.1.S1_at	7.03	5.84	7.37	6.76	7.01	6.89	6.74	0.80	6.89	0.12	1.02	0.030	7.8E-01
Mtr.27203.1.S1_at	7.04	5.71	7.16	6.83	6.86	7.29	6.64	0.80	6.99	0.26	1.05	0.075	5.1E-01
Mtr.13370.1.S1_at	6.99	6.59	5.44	5.02	5.75	6.12	6.34	0.80	5.63	0.56	0.89	-0.171	2.8E-01
Mtr.42757.1.S1_at	7.49	6.08	7.45	7.26	7.23	7.26	7.01	0.80	7.25	0.02	1.03	0.050	6.3E-01
Mtr.32695.1.S1_at	3.99	2.79	4.31	3.52	3.72	4.75	3.70	0.80	4.00	0.66	1.08	0.113	6.4E-01
Mtr.42373.1.S1_at	4.77	6.36	5.78	5.71	7.39	6.32	5.64	0.80	6.47	0.85	1.15	0.199	2.8E-01
Mtr.19035.1.S1_at	6.65	5.92	7.52	7.05	6.88	6.89	6.70	0.80	6.94	0.10	1.04	0.051	6.3E-01
Mtr.13600.1.S1_at	7.25	5.83	7.19	7.82	7.26	7.61	6.76	0.80	7.56	0.28	1.12	0.162	1.8E-01
Mtr.33155.1.S1_at	8.01	6.49	7.70	8.02	8.03	8.19	7.40	0.80	8.08	0.10	1.09	0.127	2.2E-01
Mtr.43735.1.S1_at	6.01	4.40	5.21	6.14	6.00	6.03	5.21	0.80	6.06	0.07	1.16	0.219	1.4E-01
Mtr.12072.1.S1_at	4.64	3.37	4.85	4.64	4.82	4.48	4.29	0.80	4.65	0.17	1.08	0.117	4.9E-01
Mtr.25826.1.S1_at	2.03	3.42	2.02	2.02	2.02	2.02	2.49	0.80	2.02	0.00	0.81	-0.303	3.7E-01
Mtr.22811.1.S1_s_at	3.92	2.58	2.48	2.45	2.34	2.41	2.99	0.80	2.40	0.05	0.80	-0.317	2.7E-01
Mtr.35624.1.S1_at	7.18	5.84	7.28	7.36	7.29	7.28	6.77	0.80	7.31	0.04	1.08	0.111	3.1E-01
Mtr.18858.1.S1_at	9.74	8.45	9.92	9.82	9.92	9.82	9.37	0.80	9.85	0.06	1.05	0.072	3.6E-01
Mtr.12144.1.S1_s_at	8.54	7.10	8.42	8.67	8.40	8.46	8.02	0.80	8.51	0.14	1.06	0.085	3.6E-01
Mtr.30704.1.S1_at	8.52	8.34	9.81	8.58	8.89	8.49	8.89	0.80	8.65	0.21	0.97	-0.039	6.5E-01
Mtr.43196.1.S1_at	12.89	13.65	12.05	13.04	13.09	13.14	12.86	0.80	13.09	0.05	1.02	0.025	6.5E-01
Mtr.4302.1.S1_at	6.12	7.72	6.83	6.39	6.09	6.07	6.89	0.80	6.18	0.18	0.90	-0.155	2.1E-01
Mtr.9686.1.S1_at	8.47	7.11	8.53	8.07	7.68	7.92	8.04	0.80	7.89	0.20	0.98	-0.027	7.7E-01
Mtr.5883.1.S1_at	5.36	3.77	4.39	4.89	4.48	4.53	4.51	0.80	4.63	0.22	1.03	0.041	8.0E-01
Mtr.29390.1.S1_at	3.48	1.97	2.26	2.60	2.75	2.80	2.57	0.80	2.72	0.10	1.06	0.081	7.7E-01
Mtr.30691.1.S1_at	8.36	6.88	8.16	8.13	7.86	7.85	7.80	0.80	7.95	0.16	1.02	0.027	7.7E-01
Mtr.39090.1.S1_at	4.90	3.69	5.21	5.50	5.38	5.19	4.60	0.80	5.35	0.15	1.16	0.219	1.9E-01
Mtr.49847.1.S1_at	5.37	5.27	6.70	6.49	6.74	6.14	5.78	0.80	6.46	0.30	1.12	0.160	2.4E-01
Mtr.5709.1.S1_at	8.00	6.78	8.29	8.45	8.44	8.72	7.69	0.80	8.53	0.16	1.11	0.151	1.5E-01
Mtr.5436.1.S1_at	4.77	6.15	4.75	4.75	4.75	3.92	5.23	0.80	4.47	0.48	0.86	-0.224	2.4E-01
Mtr.32337.1.S1_at	7.54	5.94	6.78	7.25	7.22	7.56	6.75	0.80	7.34	0.19	1.09	0.121	2.8E-01
Mtr.5565.1.S1_at	5.96	5.04	6.64	6.50	7.09	6.37	5.88	0.80	6.65	0.38	1.13	0.178	2.1E-01

Mtr.5543.1.S1_at	9.37	7.84	8.99	9.27	8.83	9.26	8.73	0.80	9.12	0.25	1.04	0.062	4.7E-01
Mtr.6669.1.S1_at	5.73	5.71	7.11	5.72	5.97	5.44	6.18	0.80	5.71	0.27	0.92	-0.114	3.9E-01
Mtr.41436.1.S1_at	7.62	6.05	7.11	7.57	7.55	7.49	6.93	0.80	7.53	0.04	1.09	0.121	2.6E-01
Mtr.39254.1.S1_at	6.63	5.72	7.32	6.84	6.80	7.01	6.56	0.80	6.88	0.11	1.05	0.070	5.2E-01
Mtr.13926.1.S1_at	7.27	5.87	7.24	7.55	7.57	7.38	6.79	0.80	7.50	0.11	1.10	0.143	2.0E-01
Mtr.11198.1.S1_at	5.33	5.31	6.71	6.28	6.40	6.31	5.78	0.80	6.33	0.06	1.09	0.130	3.0E-01
Mtr.29157.1.S1_at	2.83	4.17	4.26	4.46	4.69	4.13	3.76	0.80	4.42	0.28	1.18	0.236	2.4E-01
Mtr.9289.1.S1_at	9.41	7.91	9.15	9.15	9.43	8.97	8.83	0.80	9.18	0.23	1.04	0.057	5.0E-01
Mtr.27975.1.S1_at	5.96	4.60	6.01	6.17	5.97	5.86	5.52	0.80	6.00	0.16	1.09	0.120	3.7E-01
Mtr.42446.1.S1_at	9.17	10.55	9.16	10.15	10.96	10.68	9.62	0.80	10.60	0.41	1.10	0.139	1.3E-01
Mtr.11602.1.S1_at	9.23	7.64	8.53	8.79	8.99	8.83	8.46	0.80	8.87	0.10	1.05	0.068	4.3E-01
Mtr.37963.1.S1_at	9.46	8.06	9.43	9.24	9.42	9.09	8.98	0.80	9.25	0.16	1.03	0.043	6.0E-01
Mtr.37724.1.S1_s_at	11.75	12.09	10.57	10.91	11.42	11.40	11.47	0.80	11.24	0.29	0.98	-0.029	6.7E-01
Mtr.42886.1.S1_at	7.59	6.36	7.86	7.46	7.66	7.06	7.27	0.80	7.39	0.31	1.02	0.024	8.2E-01
Mtr.41947.1.S1_at	8.84	7.70	9.23	9.39	9.57	9.21	8.59	0.80	9.39	0.18	1.09	0.129	1.6E-01
Mtr.17440.1.S1_at	7.44	5.89	6.99	7.03	7.08	6.99	6.77	0.80	7.03	0.05	1.04	0.054	6.0E-01
Mtr.1495.1.S1_at	7.47	5.96	7.17	7.39	7.50	7.71	6.87	0.80	7.54	0.16	1.10	0.134	2.3E-01
Mtr.30293.1.S1_at	5.75	4.55	6.06	5.81	6.03	5.74	5.45	0.80	5.86	0.15	1.07	0.104	4.3E-01
Mtr.3512.1.S1_at	6.89	5.34	6.43	6.59	6.49	6.60	6.22	0.80	6.56	0.06	1.05	0.077	5.0E-01
Mtr.16372.1.S1_at	5.38	4.13	5.61	6.02	7.13	6.87	5.04	0.80	6.67	0.58	1.32	0.404	4.6E-02
Mtr.39823.1.S1_at	6.77	5.40	6.80	6.80	6.93	6.85	6.32	0.80	6.86	0.07	1.08	0.118	3.1E-01
Mtr.4724.1.S1_at	12.37	10.79	11.39	11.70	12.18	11.87	11.52	0.80	11.92	0.24	1.03	0.049	4.5E-01
Mtr.19772.1.S1_at	13.89	12.45	12.57	13.32	13.38	13.52	12.97	0.80	13.41	0.11	1.03	0.048	4.0E-01
Mtr.15574.1.S1_at	2.94	4.53	3.91	3.36	3.04	3.05	3.79	0.80	3.15	0.18	0.83	-0.269	2.4E-01
Mtr.45470.1.S1_at	5.47	3.95	5.14	5.43	6.48	5.51	4.85	0.80	5.81	0.58	1.20	0.259	1.7E-01
Mtr.4727.1.S1_a_at	6.71	6.12	7.70	7.49	7.67	7.67	6.85	0.80	7.61	0.11	1.11	0.152	1.8E-01
Mtr.8934.1.S1_at	10.36	8.89	9.09	9.12	9.11	9.40	9.45	0.80	9.21	0.17	0.98	-0.036	6.4E-01
Mtr.40349.1.S1_at	2.51	2.39	3.83	2.50	2.50	2.45	2.91	0.80	2.48	0.03	0.85	-0.227	4.1E-01
Mtr.18369.1.S1_at	14.21	12.93	14.39	14.70	14.28	14.76	13.84	0.80	14.58	0.26	1.05	0.075	2.0E-01
Mtr.10293.1.S1_at	6.97	5.44	6.59	6.95	6.61	6.91	6.34	0.80	6.82	0.19	1.08	0.107	3.6E-01
Mtr.12141.1.S1_at	8.58	9.73	10.11	9.78	10.15	9.51	9.48	0.80	9.81	0.32	1.04	0.051	5.3E-01
Mtr.32930.1.S1_at	10.97	9.40	9.96	10.37	9.85	10.42	10.11	0.80	10.22	0.31	1.01	0.015	8.4E-01
Mtr.16560.1.S1_at	4.17	2.70	3.97	4.08	4.37	4.00	3.62	0.80	4.15	0.19	1.15	0.197	3.3E-01
Mtr.24104.1.S1_s_at	5.74	6.42	4.83	5.31	5.55	5.53	5.66	0.80	5.46	0.13	0.96	-0.052	6.9E-01
Mtr.19235.1.S1_at	10.44	10.45	11.82	10.87	10.81	10.42	10.91	0.80	10.70	0.24	0.98	-0.027	6.9E-01
Mtr.90.1.S1_s_at	7.80	6.82	8.40	8.10	8.18	7.93	7.67	0.80	8.07	0.13	1.05	0.072	4.5E-01
Mtr.39878.1.S1_at	4.72	4.32	5.86	6.13	5.81	5.64	4.97	0.80	5.86	0.25	1.18	0.239	1.4E-01
Mtr.41919.1.S1_s_at	5.73	4.26	5.51	5.24	5.05	5.14	5.17	0.80	5.14	0.10	1.00	-0.006	9.6E-01
Mtr.17498.1.S1_at	2.86	4.22	2.81	2.77	2.76	2.75	3.30	0.80	2.76	0.01	0.84	-0.256	3.1E-01
Mtr.49167.1.S1_at	6.69	5.25	6.57	6.66	7.03	6.60	6.17	0.80	6.76	0.24	1.10	0.133	2.8E-01
Mtr.31435.1.S1_at	7.51	8.99	8.77	8.49	9.14	8.19	8.42	0.80	8.61	0.49	1.02	0.031	7.5E-01
Mtr.24230.1.S1_at	6.41	6.05	7.57	6.63	6.45	6.40	6.68	0.80	6.49	0.12	0.97	-0.040	7.1E-01
Mtr.13468.1.S1_at	9.79	8.20	8.99	9.71	9.30	9.73	8.99	0.80	9.58	0.24	1.07	0.091	2.9E-01
Mtr.10893.1.S1_at	8.64	7.35	7.19	7.66	6.72	7.32	7.73	0.80	7.24	0.48	0.94	-0.095	4.1E-01
Mtr.16974.1.S1_at	3.96	5.26	3.82	4.21	4.10	4.10	4.35	0.80	4.13	0.06	0.95	-0.072	6.7E-01
Mtr.4509.1.S1_s_at	8.03	6.55	7.81	8.35	8.20	8.21	7.46	0.80	8.26	0.08	1.11	0.145	1.6E-01
Mtr.10555.1.S1_at	5.88	4.51	5.90	6.16	6.86	5.91	5.43	0.80	6.31	0.49	1.16	0.218	1.8E-01
Mtr.43071.1.S1_at	6.40	5.05	6.45	6.74	6.58	6.31	5.97	0.80	6.54	0.21	1.10	0.132	2.9E-01
Mtr.24427.1.S1_at	7.53	6.10	7.42	7.25	7.31	7.15	7.02	0.80	7.24	0.08	1.03	0.045	6.6E-01
Mtr.35936.1.S1_at	8.34	6.96	8.33	8.35	8.58	8.09	7.88	0.79	8.34	0.24	1.06	0.083	3.9E-01
Mtr.32686.1.S1_at	6.74	5.70	7.26	6.98	7.24	6.96	6.57	0.79	7.06	0.16	1.07	0.104	3.5E-01
Mtr.33139.1.S1_at	7.17	5.85	7.27	7.32	7.25	7.39	6.76	0.79	7.32	0.07	1.08	0.113	3.0E-01
Mtr.17800.1.S1_at	9.13	7.70	9.00	8.87	8.76	8.97	8.61	0.79	8.87	0.10	1.03	0.043	6.1E-01
Mtr.21787.1.S1_at	7.41	5.85	6.87	6.91	7.20	6.81	6.71	0.79	6.97	0.20	1.04	0.056	6.1E-01
Mtr.44338.1.S1_at	6.55	5.33	6.82	7.73	7.80	7.53	6.23	0.79	7.69	0.14	1.23	0.302	3.5E-02
Mtr.33345.1.S1_s_at	8.28	6.72	7.79	8.11	8.08	7.85	7.60	0.79	8.01	0.14	1.05	0.077	4.2E-01
Mtr.39532.1.S1_at	7.48	6.20	7.65	7.51	7.66	7.57	7.11	0.79	7.58	0.07	1.07	0.092	3.7E-01
Mtr.40705.1.S1_s_at	9.07	7.74	9.16	10.63	11.13	10.66	8.66	0.79	10.81	0.28	1.25	0.321	1.1E-02
Mtr.12632.1.S1_at	14.19	12.71	12.97	13.46	12.97	13.49	13.29	0.79	13.31	0.30	1.00	0.002	9.8E-01
Mtr.42559.1.S1_at	8.42	6.84	7.71	6.58	6.70	6.85	7.66	0.79	6.71	0.14	0.88	-0.190	1.1E-01
Mtr.4296.1.S1_at	7.71	6.31	7.65	7.84	7.63	7.66	7.22	0.79	7.71	0.11	1.07	0.094	3.5E-01
Mtr.2332.1.S1_at	8.95	7.56	8.92	8.59	8.34	8.41	8.48	0.79	8.45	0.13	1.00	-0.006	9.5E-01
Mtr.44115.1.S1_s_at	7.73	6.29	7.59	7.58	7.50	7.58	7.20	0.79	7.55	0.05	1.05	0.068	4.9E-01
Mtr.4059.1.S1_at	4.51	2.97	3.42	2.93	2.86	2.84	3.63	0.79	2.88	0.05	0.79	-0.336	1.8E-01
Mtr.10700.1.S1_at	10.47	8.95	10.10	9.86	9.98	10.06	9.84	0.79	9.96	0.10	1.01	0.019	8.0E-01
Mtr.42059.1.S1_at	10.72	9.16	10.19	10.51	10.98	10.88	10.02	0.79	10.79	0.25	1.08	0.106	1.9E-01
Mtr.38525.1.S1_at	6.63	5.15	6.40	6.71	6.71	6.99	6.06	0.79	6.80	0.16	1.12	0.167	1.9E-01
Mtr.17968.1.S1_at	5.79	4.41	5.78	5.99	5.81	6.00	5.32	0.79	5.93	0.11	1.11	0.156	2.6E-01
Mtr.30516.1.S1_at	4.67	3.30	4.67	4.67	5.28	4.97	4.21	0.79	4.97	0.30	1.18	0.239	2.0E-01
Mtr.48113.1.S1_at	2.98	4.50	3.33	3.29	3.29	3.14	3.60	0.79	3.24	0.09	0.90	-0.153	4.7E-01
Mtr.26042.1.S1_at	4.40	3.74	5.32	4.32	3.94	3.78	4.49	0.79	4.01	0.28	0.89	-0.161	3.8E-01
Mtr.19482.1.S1_at	4.15	5.70	4.63	4.11	3.90	3.96	4.83	0.79	3.99	0.11	0.83	-0.275	1.4E-01
Mtr.26318.1.S1_at	7.20	6.27	7.84	7.52	7.31	7.08	7.10	0.79	7.30	0.22	1.03	0.041	6.9E-01
Mtr.32477.1.S1_at	7.40	6.03	7.39	7.51	7.41	7.37	6.94	0.79	7.43	0.07	1.07	0.099	3.4E-01
Mtr.10382.1.S1_at	11.69	10.12	10.72	11.17	10.88	11.29	10.85	0.79	11.11	0.21	1.02	0.035	6.1E-01
Mtr.16221.1.S1_at	7.16	6.17	7.74	7.36	7.28	7.17	7.02	0.79	7.27	0.09	1.03	0.050	6.2E-01
Mtr.21597.1.S1_s_at	7.88	6.61	8.06	7.88	8.09	7.75	7.52	0.79	7.90	0.17	1.05	0.073	4.5E-01
Mtr.37214.1.S1_at	12.90	11.52	11.53	11.84	12.09	11.95	11.98	0.79	11.96	0.13	1.00	-0.002	9.7E-01
Mtr.37636.1.S1_at	13.00	12.12	11.42	12.40	12.24	12.75	12.18	0.79	12.46	0.26	1.02	0.033	5.9E-01
Mtr.44404.1.S1_s_at	8.99	7.61	8.97	8.56	8.54	8.60	8.52	0.79	8.57	0.03	1.00	0.007	9.3E-01
Mtr.33570.1.S1_at	3.99	2.74	4.21	3.84	3.92	4.12	3.65	0.79	3.96	0.14	1.09	0.118	5.4E-01
Mtr.44566.1.S1_at	8.73	7.41	8.82	8.66	8.64	8.69	8.32	0.79	8.66	0.02	1.04	0.059	4.9E-01
Mtr.12723.1.S1_at	10.03	10.82	9.23	10.03	10.02	10.23	10.03	0.79	10.09	0.12	1.01	0.010	8.9E-01
Mtr.44781.1.S1_at	2.32	3.68	2.30	2.29	2.29	2.28	2.76	0.79	2.29	0.01	0.83	-0.273	3.6E-01
Mtr.4228.1.S1_at	5.37	4.30	5.84	5.55	5.82	5.54	5.17	0.79	5.64	0.16	1.09	0.125	3.7E-01
Mtr.6327.1.S1_at	6.58	5.03	6.11	6.54	6.52	6.65	5.91	0.79	6.57	0.07	1.11	0.153	2.2E-01
Mtr.41432.1.S1_at	7.31	5.83	7.06	7.16	7.36	7.26	6.73	0.79	7.26	0.10	1.08	0.109	3.2E-01
Mtr.41657.1.S1_at	9.00	7.87	9.39	8.84	9.02	8.92	8.76	0.79	8.93	0.09	1.02	0.028	7.3E-01
Mtr.9904.1.S1_at	9.95	8.72	10.20	10.34	10.11	10.22	9.63	0.79	10.23	0.12	1.06	0.087	2.6E-01
Mtr.36856.1.S1_at	4.22	2.96	4.41	4.17	3.97	4.30	3.86	0.79	4.15	0.17	1.07	0.102	5.8E-01
Mtr.15890.1.S1_at	6.26	5.23	6.78	6.76	6.79	6.77	6.09	0.79	6.77	0.01	1.11	0.154	2.1E-01
Mtr.45293.1.S1_s_at	6.40	5.43	6.99	6.49	6.66	6.53	6.27	0.79	6.56	0.09	1.05	0.065	5.6E-01
Mtr.29369.1.S1_at	7.95	6.43	7.57	7.82	8.01	7.74	7.32	0.79	7.86	0.14	1.07	0.103	3.1E-01
Mtr.7199.1.S1_at	8.18	6.61	7.21	8.02	8.59	8.05	7.34	0.79	8.22	0.32	1.12	0.164	1.5E-01
Mtr.41392.1.S1_at	6.18	5.31	6.89	6.42	6.27	6.17	6.13	0.79	6.29	0.13	1.03	0.037	7.5E-01
Mtr.43181.1.S1_at	11.76	10.49	11.94	11.91	12.03	11.79	11.39	0.79	11.91	0.12	1.05	0.064	3.3E-01
Mtr.21.1.S1_s_at	8.24	6.68	7.64	8.12	8.25	8.42	7.52	0.79	8.26	0.15	1.10	0.136	1.9E-01
Mtr.37835.1.S1_at	9.89	8.40	8.68	9.46	9.61	9.30	8.99	0.79	9.45	0.16	1.05	0.073	3.7E-01
Mtr.24811.1.S1_at	8.08	7.01	8.55	8.04	8.11	8.10	7.88	0.79	8.08	0.04	1.03	0.036	6.8E-01
Mtr.5171.1.S1_at	8.39	6.87	8.00	8.12	7.85	7.66	7.75	0.79	7.88	0.23	1.02	0.022	8.1E-01
Mtr.32793.1.S1_at	6.73	5.25	6.47	6.62	6.61	6.57	6.15	0.79	6.60	0.03	1.07	0.103	3.8E-01
Mtr.33304.1.S1_at	7.32	6.72	8.29	7.85	7.47	7.89	7.44	0.79	7.74	0.23	1.04	0.056	5.7E-01
Mtr.39595.1.S1_at	8.85	8.05	9.63	9.25	9.37	8.94	8.84	0.79	9.18	0.22	1.04	0.055	5.1E-01
Mtr.13794.1.S1_at	9.03	7.45	8.31	8.41	8.58	8.29	8.26	0.79	8.42	0.15	1.02	0.028	7.4E-01
Mtr.47039.1.S1_at	5.12	3.88	5.35	4.73	4.26	4.62	4.78	0.79	4.54	0.25	0.95	-0.076	6.3E-01
Mtr.6271.1.S1_at	2.66	3.98	2.57	2.87	2.59	2.61	3.07	0.79	2.69	0.16	0.88	-0.189	4.6E-01
Mtr.41463.1.S1_at	9.27	8.12	9.62	8.87	8.81	8.58	9.00	0.79	8.75	0.15	0.97	-0.041	6.2E-01
Mtr.20215.1.S1_s_at	10.67	9.52	9.16	9.11	9.55	9.57	9.78	0.79	9.41	0.26	0.96	-0.056	4.8E-01
Mtr.51734.1.S1_at	7.77	6.48	7.92	7.98	7.46	7.78	7.39	0.79	7.74	0.26	1.05	0.066	5.1E-01
Mtr.8172.1.S1_at	9.46	8.07	8.11	8.55	9.21	9.10	8.55	0.79	8.95	0.35	1.05	0.067	4.6E-01
Mtr.7709.1.S1_at	5.01	3.57	4.85	4.42	5.01	5.68	4.48	0.79	5.03	0.63	1.12	0.170	3.9E-01
Mtr.27738.1.S1_at	10.26	8.76	9.95	9.75	9.86	9.72	9.65	0.79	9.78	0.08	1.01	0.018	8.0E-01
Mtr.23405.1.S1_at	9.80	8.26	8.74	9.17	8.95	9.50	8.93	0.79	9.21	0.28	1.03	0.044	6.0E-01
Mtr.19721.1.S1_at	9.38	7.87	9.02	9.19	9.10	9.10	8.76	0.79	9.13	0.05	1.04	0.061	4.5E-01
Mtr.16464.1.S1_at	9.58	8.20	9.56	9.39	9.44	9.37	9.11	0.79	9.40	0.03	1.03	0.044	5.7E-01
Mtr.12941.1.S1_at	5.32	4.05	3.87	10.32	10.05	10.30	4.41	0.79	10.22	0.15	2.32	1.211	2.3E-04
Mtr.4986.1.S1_at	7.08	5.63	6.90	6.86	6.88	6.94	6.54	0.79	6.89	0.04	1.05	0.076	4.8E-01
Mtr.21211.1.S1_at	8.72	7.46	8.91	8.69	8.74	8.76	8.36	0.79	8.73	0.03	1.04	0.063	4.6E-01
Mtr.22480.1.S1_s_at	5.11	3.96	5.47	5.47	5.08	4.77	4.85	0.79	5.10	0.35	1.05	0.075	6.3E-01
Mtr.39091.1.S1_at	8.01	6.65	8.02	8.14	8.08	8.34	7.56	0.79	8.18	0.14	1.08	0.115	2.5E-01
Mtr.7580.1.S1_x_at	6.76	5.57	7.06	6.68	6.96	6.75	6.46	0.79	6.79	0.14	1.05	0.072	5.2E-01
Mtr.753.1.S1_at	11.11	9.98	9.59	10.13	10.16	10.27	10.22	0.79	10.18	0.07	1.00	-0.006	9.4E-01
Mtr.16691.1.S1_at	4.32	5.85	5.41	5.74	5.83	5.05	5.20	0.79	5.54	0.42	1.07	0.093	5.4E-01
Mtr.52255.1.S1_at	8.01	6.51	7.70	7.95	7.96	7.59	7.40	0.79	7.83	0.21	1.06	0.081	4.1E-01
Mtr.16137.1.S1_at	5.58	4.25	5.66	5.58	5.57	5.55	5.16	0.79	5.56	0.02	1.08	0.108	4.3E-01
Mtr.41893.1.S1_at	4.67	3.65	5.21	5.18	4.94	5.04	4.51	0.79	5.05	0.12	1.12	0.164	3.0E-01
Mtr.8417.1.S1_at	10.09	11.62	11.17	10.20	10.15	10.07	10.96	0.79	10.14	0.07	0.93	-0.112	1.5E-01
Mtr.10177.1.S1_at	4.03	3.37	4.94	4.45	4.69	4.45	4.12	0.79	4.53	0.14	1.10	0.138	4.2E-01
Mtr.2441.1.S1_s_at	9.18	8.36	9.93	9.55	9.53	9.51	9.16	0.79	9.53	0.02	1.04	0.058	4.6E-01
Mtr.39357.1.S1_s_at	8.26	6.71	7.70	7.68	7.74	7.79	7.56	0.79	7.74	0.05	1.02	0.034	7.1E-01
Mtr.51264.1.S1_at	3.46	2.13	2.07	2.07	2.06	2.06	2.55	0.79	2.06	0.00	0.81	-0.308	3.4E-01

Mtr.31756.1.S1_at	8.65	7.24	8.56	8.74	8.44	8.56	8.15	0.79	8.58	0.15	1.05	0.073	4.1E-01
Mtr.7043.1.S1_x_at	7.81	6.44	7.79	7.95	7.82	7.80	7.34	0.79	7.86	0.08	1.07	0.097	3.2E-01
Mtr.40881.1.S1_s_at	9.65	8.11	9.15	9.40	9.85	9.71	8.97	0.79	9.66	0.23	1.08	0.107	2.2E-01
Mtr.6540.1.S1_at	7.92	7.80	9.22	8.96	8.39	8.74	8.32	0.79	8.70	0.29	1.05	0.065	4.8E-01
Mtr.5312.1.S1_at	8.18	6.69	7.86	8.18	7.85	8.10	7.58	0.79	8.04	0.18	1.06	0.087	3.7E-01
Mtr.11456.1.S1_at	7.74	6.41	7.79	7.98	7.61	7.93	7.31	0.79	7.84	0.20	1.07	0.100	3.3E-01
Mtr.2926.1.S1_at	7.58	6.17	7.49	7.16	7.45	7.05	7.08	0.79	7.22	0.21	1.02	0.029	7.8E-01
Mtr.21996.1.S1_x_at	5.61	4.04	4.82	5.52	5.12	5.02	4.82	0.79	5.22	0.27	1.08	0.114	4.5E-01
Mtr.16297.1.S1_at	6.34	5.00	6.38	6.12	6.02	6.48	5.91	0.79	6.21	0.24	1.05	0.072	5.6E-01
Mtr.31536.1.S1_at	8.40	6.93	8.15	8.41	8.65	8.45	7.83	0.79	8.50	0.13	1.09	0.120	2.2E-01
Mtr.4459.1.S1_at	3.16	4.47	3.05	3.03	3.02	3.03	3.56	0.79	3.02	0.00	0.85	-0.235	3.0E-01
Mtr.27576.1.S1_s_at	6.20	4.83	6.19	6.03	6.03	6.24	5.74	0.79	6.10	0.12	1.06	0.087	4.8E-01
Mtr.29652.1.S1_at	6.29	5.19	6.71	6.69	7.35	6.73	6.06	0.79	6.92	0.37	1.14	0.192	1.6E-01
Mtr.17691.1.S1_at	7.43	6.05	7.39	7.18	6.99	6.85	6.96	0.79	7.01	0.16	1.01	0.011	9.2E-01
Mtr.6097.1.S1_at	3.82	2.65	4.15	3.80	3.84	3.41	3.54	0.79	3.69	0.24	1.04	0.059	7.7E-01
Mtr.28811.1.S1_at	6.40	4.97	6.25	8.37	8.07	8.07	5.87	0.79	8.17	0.17	1.39	0.477	7.8E-03
Mtr.24912.1.S1_at	9.79	8.77	10.31	10.05	10.04	9.87	9.62	0.79	9.99	0.10	1.04	0.054	4.7E-01
Mtr.28659.1.S1_at	8.64	7.19	8.43	8.69	8.68	8.66	8.09	0.79	8.68	0.02	1.07	0.102	2.6E-01
Mtr.1392.1.S1_s_at	8.27	6.70	7.46	8.35	8.27	8.24	7.48	0.79	8.28	0.06	1.11	0.148	1.5E-01
Mtr.39298.1.S1_at	8.92	7.35	8.25	9.14	9.45	9.62	8.17	0.79	9.40	0.25	1.15	0.202	6.1E-02
Mtr.33447.1.S1_s_at	8.86	7.46	8.77	8.54	8.77	8.63	8.37	0.79	8.65	0.11	1.03	0.047	5.8E-01
Mtr.18447.1.S1_at	5.87	4.66	6.14	6.23	6.34	6.09	5.56	0.79	6.22	0.13	1.12	0.162	2.2E-01
Mtr.24781.1.S1_at	4.34	3.12	4.59	4.80	4.80	4.56	4.02	0.79	4.72	0.14	1.17	0.232	2.0E-01
Mtr.23371.1.S1_at	7.60	6.23	7.59	8.01	7.87	7.70	7.14	0.79	7.86	0.15	1.10	0.138	2.0E-01
Mtr.6997.1.S1_s_at	6.91	5.39	6.50	6.48	6.47	6.48	6.27	0.79	6.48	0.00	1.03	0.047	6.7E-01
Mtr.45320.1.S1_at	9.21	7.66	8.68	8.69	8.60	8.86	8.52	0.79	8.72	0.13	1.02	0.033	6.9E-01
Mtr.29062.1.S1_at	8.96	7.74	9.21	8.44	8.32	8.02	8.64	0.79	8.26	0.21	0.96	-0.064	4.7E-01
Mtr.41714.1.S1_at	7.68	6.66	8.21	8.05	7.92	7.80	7.52	0.79	7.92	0.13	1.05	0.076	4.3E-01
Mtr.42445.1.S1_at	8.27	7.04	8.50	7.88	7.47	7.49	7.94	0.79	7.61	0.23	0.96	-0.060	5.3E-01
Mtr.25422.1.S1_at	2.36	2.17	3.61	2.36	2.35	2.42	2.71	0.78	2.38	0.04	0.88	-0.191	5.0E-01
Mtr.32305.1.S1_at	5.26	3.70	4.63	4.59	4.53	4.59	4.53	0.78	4.57	0.04	1.01	0.014	9.3E-01
Mtr.44938.1.S1_at	8.11	6.93	8.41	8.43	8.79	8.06	7.81	0.78	8.42	0.36	1.08	0.108	2.9E-01
Mtr.21943.1.S1_s_at	9.14	7.58	8.55	8.87	8.67	8.68	8.42	0.78	8.74	0.11	1.04	0.053	5.3E-01
Mtr.36849.1.S1_at	7.22	5.99	7.45	7.17	7.81	7.20	6.89	0.78	7.39	0.36	1.07	0.102	3.7E-01
Mtr.5136.1.S1_s_at	3.82	2.77	4.30	4.46	3.89	3.89	3.63	0.78	4.08	0.33	1.12	0.169	4.1E-01
Mtr.45871.1.S1_at	3.92	5.06	3.56	3.84	3.84	3.84	4.18	0.78	3.84	0.00	0.92	-0.122	5.0E-01
Mtr.50859.1.S1_s_at	10.44	8.89	9.87	10.04	9.75	10.11	9.73	0.78	9.97	0.19	1.02	0.034	6.4E-01
Mtr.37024.1.S1_at	3.72	2.27	3.52	3.24	3.48	3.23	3.17	0.78	3.32	0.14	1.05	0.066	7.6E-01
Mtr.11548.1.S1_at	5.85	4.39	5.61	5.61	5.82	6.31	5.28	0.78	5.92	0.36	1.12	0.163	2.7E-01
Mtr.9262.1.S1_at	5.42	3.93	5.08	5.33	5.39	5.18	4.81	0.78	5.30	0.11	1.10	0.140	3.4E-01
Mtr.2723.1.S1_s_at	6.43	5.38	6.91	6.25	6.20	6.10	6.24	0.78	6.18	0.08	0.99	-0.014	9.0E-01
Mtr.18900.1.S1_at	9.58	8.36	9.82	10.00	10.09	9.83	9.25	0.78	9.97	0.13	1.08	0.108	1.9E-01
Mtr.6797.1.S1_at	7.98	6.46	7.54	7.66	7.62	7.82	7.32	0.78	7.70	0.11	1.05	0.072	4.6E-01
Mtr.40945.1.S1_at	9.27	7.98	9.39	9.30	9.42	9.29	8.88	0.78	9.34	0.07	1.05	0.073	3.7E-01
Mtr.36881.1.S1_at	5.37	4.57	6.13	5.28	5.90	5.31	5.36	0.78	5.49	0.35	1.03	0.036	8.0E-01
Mtr.22745.1.S1_at	8.60	7.06	8.07	8.09	7.58	7.81	7.91	0.78	7.83	0.25	0.99	-0.015	8.7E-01
Mtr.29080.1.S1_at	4.01	2.69	2.62	2.59	2.59	2.70	3.10	0.78	2.63	0.06	0.85	-0.239	3.5E-01
Mtr.27543.1.S1_s_at	7.34	5.78	6.70	6.86	6.94	6.99	6.61	0.78	6.93	0.07	1.05	0.068	5.2E-01
Mtr.51083.1.S1_at	7.51	6.68	8.24	8.08	8.14	7.96	7.48	0.78	8.06	0.09	1.08	0.109	2.7E-01
Mtr.39698.1.S1_at	7.36	5.79	6.50	7.07	7.13	7.37	6.55	0.78	7.19	0.16	1.10	0.134	2.4E-01
Mtr.44198.1.S1_at	9.85	8.30	8.92	8.66	8.68	8.61	9.02	0.78	8.65	0.04	0.96	-0.060	4.6E-01
Mtr.49176.1.S1_at	5.25	3.74	4.85	4.89	5.53	4.95	4.61	0.78	5.12	0.36	1.11	0.151	3.6E-01
Mtr.38732.1.S1_at	7.45	6.72	8.28	7.43	7.50	7.22	7.48	0.78	7.39	0.14	0.99	-0.019	8.4E-01
Mtr.10847.1.S1_at	3.13	4.58	4.36	10.25	10.35	10.12	4.02	0.78	10.24	0.12	2.55	1.348	1.7E-04
Mtr.7233.1.S1_at	7.42	6.30	7.81	7.58	7.70	7.69	7.18	0.78	7.65	0.07	1.07	0.093	3.5E-01
Mtr.26285.1.S1_s_at	5.76	5.85	7.16	6.20	7.34	5.81	6.26	0.78	6.45	0.79	1.03	0.043	7.8E-01
Mtr.2389.1.S1_at	11.08	9.63	10.86	11.11	11.04	10.99	10.52	0.78	11.05	0.06	1.05	0.070	3.1E-01
Mtr.5121.1.S1_x_at	7.95	6.40	7.41	7.99	8.32	8.53	7.25	0.78	8.28	0.27	1.14	0.191	9.9E-02
Mtr.32798.1.S1_at	4.91	5.20	6.39	5.58	5.63	5.41	5.50	0.78	5.54	0.11	1.01	0.012	9.3E-01
Mtr.33611.1.S1_at	6.04	4.78	6.21	6.05	6.21	5.98	5.68	0.78	6.08	0.12	1.07	0.098	4.3E-01
Mtr.49661.1.S1_at	6.14	4.58	5.22	5.83	6.46	6.26	5.31	0.78	6.18	0.32	1.16	0.219	1.5E-01
Mtr.11650.1.S1_at	7.84	6.86	8.40	7.70	7.68	7.64	7.70	0.78	7.67	0.03	1.00	-0.005	9.5E-01
Mtr.23391.1.S1_at	2.74	4.07	2.69	2.67	2.66	2.68	3.16	0.78	2.67	0.01	0.84	-0.245	3.3E-01
Mtr.9402.1.S1_at	6.69	5.53	7.01	6.45	6.44	6.37	6.41	0.78	6.42	0.05	1.00	0.002	9.9E-01
Mtr.27353.1.S1_at	3.79	2.32	3.51	3.74	3.51	4.27	3.21	0.78	3.84	0.39	1.20	0.261	2.8E-01
Mtr.27764.1.S1_at	2.93	4.28	2.93	3.33	2.88	2.88	3.38	0.78	3.03	0.26	0.90	-0.158	5.0E-01
Mtr.26830.1.S1_at	4.17	2.86	4.25	5.00	5.16	4.67	3.76	0.78	4.94	0.25	1.31	0.394	6.7E-02
Mtr.32409.1.S1_at	9.04	7.49	8.08	8.57	8.92	8.93	8.20	0.78	8.81	0.21	1.07	0.103	2.7E-01
Mtr.6879.1.S1_s_at	7.52	6.60	8.15	8.25	7.97	8.02	7.42	0.78	8.08	0.15	1.09	0.123	2.2E-01
Mtr.9586.1.S1_at	7.91	6.36	7.02	7.03	6.84	7.09	7.10	0.78	6.99	0.13	0.98	-0.022	8.3E-01
Mtr.5724.1.S1_s_at	7.18	6.60	8.14	7.63	7.95	7.48	7.31	0.78	7.69	0.24	1.05	0.073	4.7E-01
Mtr.7334.1.S1_at	5.65	4.14	4.55	5.30	5.10	5.10	4.78	0.78	5.17	0.12	1.08	0.114	4.4E-01
Mtr.11088.1.S1_at	2.34	3.70	2.35	2.36	2.36	2.36	2.80	0.78	2.36	0.00	0.84	-0.247	3.8E-01
Mtr.22565.1.S1_at	2.31	3.66	2.30	2.30	2.29	2.40	2.75	0.78	2.33	0.06	0.85	-0.241	4.0E-01
Mtr.41525.1.S1_at	3.59	3.65	4.97	8.79	9.05	8.87	4.07	0.78	8.90	0.13	2.19	1.129	4.5E-04
Mtr.33448.1.S1_at	10.82	10.27	11.81	11.16	11.32	11.06	10.97	0.78	11.18	0.13	1.02	0.028	6.6E-01
Mtr.244.1.S1_at	10.76	10.81	9.44	11.46	11.10	11.54	10.34	0.78	11.37	0.23	1.10	0.137	9.4E-02
Mtr.38957.1.S1_at	6.93	5.79	7.29	7.52	9.29	8.38	6.67	0.78	8.40	0.88	1.26	0.332	6.4E-02
Mtr.21159.1.S1_x_at	2.55	4.04	2.90	2.88	2.90	3.14	3.16	0.78	2.97	0.15	0.94	-0.090	7.0E-01
Mtr.31506.1.S1_at	7.99	6.55	7.78	7.97	8.24	7.99	7.44	0.78	8.07	0.15	1.08	0.116	2.4E-01
Mtr.17459.1.S1_at	2.56	3.90	2.53	2.51	2.50	2.51	3.00	0.78	2.51	0.01	0.84	-0.259	3.4E-01
Mtr.27837.1.S1_at	5.62	4.15	4.45	5.93	5.94	6.26	4.74	0.78	6.05	0.18	1.28	0.351	4.8E-02
Mtr.46512.1.S1_at	2.88	4.25	2.92	2.81	2.81	2.83	3.35	0.78	2.81	0.01	0.84	-0.252	3.0E-01
Mtr.24318.1.S1_at	3.10	4.45	3.11	3.09	3.07	3.06	3.55	0.78	3.07	0.01	0.87	-0.209	3.5E-01
Mtr.28847.1.S1_at	2.51	3.83	2.46	2.52	2.55	2.52	2.94	0.78	2.53	0.01	0.86	-0.213	4.2E-01
Mtr.4979.1.S1_at	7.70	6.97	8.53	8.30	8.16	8.23	7.73	0.78	8.23	0.07	1.06	0.090	3.3E-01
Mtr.45003.1.S1_at	4.21	2.84	4.17	4.61	4.85	4.93	3.74	0.78	4.79	0.17	1.28	0.358	8.4E-02
Mtr.23726.1.S1_s_at	10.33	8.80	9.84	10.04	9.87	10.07	9.65	0.78	9.99	0.11	1.04	0.050	5.0E-01
Mtr.1801.1.S1_s_at	10.40	9.09	10.47	9.67	7.04	9.79	9.99	0.78	8.83	1.55	0.88	-0.177	3.1E-01
Mtr.23883.1.S1_at	6.11	4.60	5.68	5.47	5.08	5.66	5.46	0.78	5.41	0.30	0.99	-0.014	9.2E-01
Mtr.13392.1.S1_at	6.79	5.48	6.87	6.86	7.14	7.05	6.38	0.78	7.02	0.14	1.10	0.137	2.4E-01
Mtr.41649.1.S1_at	6.65	5.59	7.11	6.98	6.84	6.75	6.45	0.78	6.86	0.12	1.06	0.088	4.2E-01
Mtr.48594.1.S1_at	2.68	4.01	2.65	2.70	2.64	2.63	3.11	0.78	2.65	0.04	0.85	-0.230	3.7E-01
Mtr.23807.1.S1_at	4.45	3.11	3.09	3.46	3.46	3.61	3.55	0.78	3.51	0.09	0.99	-0.017	9.3E-01
Mtr.29329.1.S1_s_at	9.23	7.71	8.78	8.94	9.06	9.12	8.57	0.78	9.04	0.09	1.05	0.076	3.6E-01
Mtr.40518.1.S1_at	3.16	4.54	3.23	3.19	3.23	3.11	3.64	0.78	3.18	0.06	0.87	-0.198	3.6E-01
Mtr.12889.1.S1_at	7.50	6.15	6.16	5.60	6.41	6.16	6.61	0.78	6.05	0.41	0.92	-0.126	3.4E-01
Mtr.24478.1.S1_at	3.08	4.42	3.07	2.62	3.16	3.06	3.52	0.78	2.95	0.29	0.84	-0.258	2.9E-01
Mtr.49741.1.S1_x_at	2.68	4.02	2.67	2.66	2.63	2.62	3.12	0.78	2.64	0.02	0.84	-0.243	3.4E-01
Mtr.45085.1.S1_s_at	8.33	6.99	8.35	8.64	8.56	8.49	7.89	0.78	8.56	0.08	1.09	0.118	2.1E-01
Mtr.20268.1.S1_at	8.55	7.21	8.56	7.92	8.04	7.96	8.11	0.78	7.97	0.06	0.98	-0.024	7.8E-01
Mtr.5881.1.S1_at	6.37	4.88	6.00	6.34	6.18	6.13	5.75	0.78	6.22	0.11	1.08	0.112	3.6E-01
Mtr.30179.1.S1_at	7.28	5.93	7.28	7.27	7.43	7.17	6.83	0.78	7.29	0.13	1.07	0.094	3.7E-01
Mtr.45700.1.S1_at	9.74	8.23	9.28	9.47	9.46	9.34	9.08	0.78	9.42	0.07	1.04	0.053	4.9E-01
Mtr.38548.1.S1_at	6.83	5.52	6.90	6.74	6.84	6.46	6.42	0.78	6.68	0.20	1.04	0.058	6.0E-01
Mtr.20479.1.S1_at	8.45	7.05	8.32	8.20	8.11	7.93	7.94	0.78	8.08	0.14	1.02	0.025	7.8E-01
Mtr.25812.1.S1_at	4.52	2.97	3.82	3.65	3.06	4.03	3.77	0.78	3.58	0.49	0.95	-0.073	7.4E-01
Mtr.7101.1.S1_at	9.50	8.39	9.88	9.63	9.55	9.54	9.26	0.78	9.57	0.05	1.03	0.048	5.2E-01
Mtr.2700.1.S1_s_at	9.17	7.80	9.11	8.96	9.01	8.98	8.69	0.78	8.99	0.03	1.03	0.048	5.5E-01
Mtr.40847.1.S1_at	6.62	5.37	6.79	6.90	7.29	6.87	6.26	0.78	7.02	0.23	1.12	0.165	1.8E-01
Mtr.40517.1.S1_s_at	9.21	10.55	9.21	9.69	10.82	10.36	9.66	0.78	10.29	0.57	1.07	0.091	3.2E-01
Mtr.13014.1.S1_at	14.06	12.51	13.24	13.45	13.17	13.51	13.27	0.78	13.37	0.18	1.01	0.011	8.3E-01
Mtr.2717.1.S1_at	5.08	4.74	6.22	5.81	5.95	4.91	5.34	0.78	5.56	0.57	1.04	0.056	7.2E-01
Mtr.24603.1.S1_at	2.28	3.62	2.27	2.27	2.27	2.26	2.73	0.78	2.26	0.01	0.83	-0.268	3.6E-01
Mtr.22122.1.S1_s_at	2.95	4.23	2.83	2.92	2.81	2.90	3.34	0.78	2.88	0.06	0.86	-0.214	3.6E-01
Mtr.48271.1.S1_at	7.22	5.88	7.23	7.03	6.93	6.66	6.78	0.78	6.87	0.20	1.01	0.021	8.4E-01
Mtr.919.1.S1_at	4.71	3.21	4.30	4.49	4.18	3.67	4.07	0.78	4.11	0.41	1.01	0.015	9.4E-01
Mtr.44998.1.S1_s_at	8.95	7.48	8.65	8.63	8.57	8.59	8.36	0.78	8.60	0.03	1.03	0.041	6.2E-01
Mtr.31857.1.S1_at	4.80	6.17	6.11	6.30	6.82	6.62	5.69	0.78	6.58	0.26	1.16	0.209	1.3E-01
Mtr.51234.1.S1_s_at	4.41	2.86	3.57	3.78	4.36	3.05	3.62	0.78	3.73	0.66	1.03	0.046	8.5E-01
Mtr.13555.1.S1_at	5.85	5.90	7.22	5.59	5.84	5.55	6.32	0.78	5.66	0.16	0.89	-0.160	2.2E-01
Mtr.20401.1.S1_at	2.13	3.47	2.12	2.18	2.12	2.11	2.57	0.78	2.14	0.04	0.83	-0.268	3.8E-01
Mtr.44919.1.S1_at	7.15	6.04	7.54	7.33	7.15	7.08	6.91	0.77	7.19	0.13	1.04	0.057	5.8E-01
Mtr.5448.1.S1_s_at	7.57	6.16	7.43	7.47	7.80	7.65	7.05	0.77	7.64	0.16	1.08	0.116	2.7E-01
Mtr.34963.1.S1_at	8.41	7.00	7.15	7.83	8.04	7.79	7.52	0.77	7.89	0.13	1.05	0.069	4.6E-01
Mtr.27874.1.S1_at	5.75	4.36	5.64	5.94	5.56	5.83	5.25	0.77	5.78	0.19	1.10	0.138	3.2E-01
Mtr.39816.1.S1_at	8.76	7.43	8.78	8.73	8.66	8.89	8.33	0.77	8.76	0.12	1.05	0.073	3.9E-01
Mtr.11951.1.S1_at	8.79	7.46	7.43	8.10	7.53	8.16	7.89	0.77	7.93	0.35	1.01	0.007	9.4E-01
Mtr.32898.1.S1_at	5.18	3.63	4.32	3.87	3.68	3.44	4.38	0.77	3.66	0.22	0.84	-0.256	2.0E-01
Mtr.2150.1.S1_s_at	7.45	6.25	7.69	7.49	7.75	7.47	7.13	0.77	7.57	0.16	1.06	0.086	3.9E-01
Mtr.43776.1.S1_at	6.08	4.62	5.80	6.34	6.65	6.67	5.50	0.77	6.55	0.19	1.19	0.253	8.4E-02

Mtr.23937.1.S1_at	7.35	6.08	7.48	7.78	7.78	7.68	6.97	0.77	7.75	0.05	1.11	0.152	1.6E-01
Mtr.38267.1.S1_at	5.39	6.63	5.21	4.96	4.94	5.16	5.75	0.77	5.02	0.12	0.87	-0.195	1.8E-01
Mtr.16823.1.S1_s_at	8.43	7.38	8.90	8.37	8.61	8.47	8.24	0.77	8.48	0.12	1.03	0.042	6.2E-01
Mtr.9701.1.S1_at	8.48	8.51	9.84	9.34	9.24	9.22	8.94	0.77	9.27	0.06	1.04	0.051	5.1E-01
Mtr.1456.1.S1_at	9.37	7.89	9.01	8.99	9.08	8.96	8.76	0.77	9.01	0.06	1.03	0.041	6.1E-01
Mtr.28946.1.S1_at	2.16	3.50	2.16	2.16	2.15	2.15	2.61	0.77	2.15	0.00	0.82	-0.278	3.6E-01
Mtr.10597.1.S1_at	7.80	6.71	8.21	7.97	8.79	8.27	7.57	0.77	8.34	0.42	1.10	0.140	2.0E-01
Mtr.18886.1.S1_at	2.00	3.34	1.99	1.99	1.99	1.99	2.44	0.77	1.99	0.00	0.81	-0.295	3.7E-01
Mtr.30214.1.S1_at	7.49	8.79	7.42	7.71	7.19	7.59	7.90	0.77	7.50	0.28	0.95	-0.075	4.4E-01
Mtr.2522.1.S1_s_at	9.99	8.92	10.42	10.26	10.10	10.11	9.78	0.77	10.15	0.09	1.04	0.055	4.5E-01
Mtr.20414.1.S1_at	2.59	3.93	2.59	2.58	2.58	2.58	3.04	0.77	2.58	0.00	0.85	-0.235	3.6E-01
Mtr.12259.1.S1_at	9.26	7.74	8.75	8.71	8.29	8.25	8.59	0.77	8.42	0.26	0.98	-0.029	7.4E-01
Mtr.14778.1.S1_at	2.45	3.79	2.44	2.43	2.42	2.42	2.89	0.77	2.42	0.01	0.84	-0.256	3.5E-01
Mtr.38531.1.S1_at	5.71	4.18	5.18	5.28	5.62	5.16	5.02	0.77	5.35	0.24	1.07	0.092	5.2E-01
Mtr.16661.1.S1_x_at	2.09	3.49	2.22	2.25	2.37	2.44	2.60	0.77	2.35	0.09	0.90	-0.147	6.0E-01
Mtr.48785.1.S1_s_at	7.66	6.22	7.44	6.61	7.01	6.84	7.11	0.77	6.82	0.20	0.96	-0.059	5.7E-01
Mtr.33377.1.S1_at	10.13	10.54	11.63	11.63	11.79	11.44	10.77	0.77	11.62	0.17	1.08	0.110	1.4E-01
Mtr.41680.1.S1_at	7.92	6.92	8.44	8.35	8.80	8.52	7.76	0.77	8.56	0.23	1.10	0.141	1.6E-01
Mtr.41252.1.S1_at	8.73	7.19	7.95	8.52	8.36	8.63	7.96	0.77	8.50	0.13	1.07	0.096	2.9E-01
Mtr.33892.1.S1_at	3.82	2.88	4.41	4.68	4.76	3.89	3.71	0.77	4.44	0.48	1.20	0.261	2.3E-01
Mtr.19037.1.S1_at	7.47	6.57	8.11	7.33	7.66	7.35	7.39	0.77	7.45	0.19	1.01	0.012	9.0E-01
Mtr.6992.1.S1_at	8.67	7.37	8.74	9.09	9.21	9.26	8.26	0.77	9.19	0.09	1.11	0.153	1.1E-01
Mtr.137.1.S1_at	11.92	10.66	10.53	11.41	11.14	11.52	11.04	0.77	11.36	0.20	1.03	0.042	5.2E-01
Mtr.27242.1.S1_at	5.51	4.27	5.68	6.32	6.43	6.39	5.15	0.77	6.38	0.06	1.24	0.308	5.2E-02
Mtr.41565.1.S1_s_at	8.59	7.05	7.79	7.94	8.30	8.14	7.81	0.77	8.13	0.18	1.04	0.057	5.3E-01
Mtr.2591.1.S1_at	8.17	6.68	7.77	8.02	8.02	8.25	7.54	0.77	8.10	0.14	1.07	0.103	2.8E-01
Mtr.38449.1.S1_at	8.26	7.27	8.79	8.39	8.34	8.33	8.11	0.77	8.35	0.03	1.03	0.044	6.1E-01
Mtr.34555.1.S1_at	2.49	3.81	2.46	2.46	2.44	2.45	2.92	0.77	2.45	0.01	0.84	-0.253	3.5E-01
Mtr.37163.1.S1_at	5.38	3.89	4.28	4.62	4.62	4.65	4.52	0.77	4.63	0.02	1.02	0.035	8.2E-01
Mtr.4748.1.S1_at	9.36	8.21	9.67	9.64	9.54	9.75	9.08	0.77	9.64	0.11	1.06	0.086	2.8E-01
Mtr.6524.1.S1_at	7.68	6.34	7.68	7.40	7.28	7.19	7.23	0.77	7.29	0.11	1.01	0.011	9.1E-01
Mtr.32657.1.S1_s_at	10.98	10.47	9.47	9.56	9.59	9.77	10.30	0.77	9.64	0.12	0.94	-0.096	2.1E-01
Mtr.39700.1.S1_s_at	4.04	2.72	4.08	4.08	4.19	4.07	3.61	0.77	4.12	0.06	1.14	0.188	3.2E-01
Mtr.32399.1.S1_at	4.14	2.75	4.03	4.03	4.03	4.00	3.64	0.77	4.02	0.02	1.10	0.143	4.4E-01
Mtr.12817.1.S1_at	5.04	3.73	5.09	5.08	5.17	5.30	4.62	0.77	5.18	0.11	1.12	0.167	2.8E-01
Mtr.33637.1.S1_at	5.23	3.95	5.33	5.21	5.14	5.01	4.84	0.77	5.12	0.10	1.06	0.081	5.7E-01
Mtr.28166.1.S1_at	7.83	6.35	7.48	7.13	7.80	7.55	7.22	0.77	7.49	0.34	1.04	0.054	6.0E-01
Mtr.13154.1.S1_s_at	8.36	6.90	8.06	8.25	8.27	8.49	7.77	0.77	8.34	0.13	1.07	0.102	2.8E-01
Mtr.9620.1.S1_at	2.09	3.42	2.08	2.08	2.08	2.08	2.53	0.77	2.08	0.00	0.82	-0.284	3.7E-01
Mtr.32573.1.S1_x_at	6.89	5.57	6.91	6.95	6.80	6.70	6.45	0.77	6.82	0.13	1.06	0.079	4.7E-01
Mtr.28980.1.S1_at	4.26	2.87	2.99	3.75	4.81	4.01	3.37	0.77	4.19	0.55	1.24	0.313	2.1E-01
Mtr.46330.1.S1_at	7.87	6.66	8.08	7.50	7.25	7.82	7.54	0.77	7.53	0.28	1.00	-0.002	9.8E-01
Mtr.34320.1.S1_at	2.35	3.68	2.34	2.33	2.33	2.33	2.79	0.77	2.33	0.00	0.84	-0.260	3.6E-01
Mtr.29988.1.S1_at	5.68	4.15	5.08	5.65	5.62	5.56	4.97	0.77	5.61	0.04	1.13	0.174	2.3E-01
Mtr.12485.1.S1_at	13.54	12.37	12.08	12.24	12.46	12.38	12.66	0.77	12.36	0.11	0.98	-0.035	5.3E-01
Mtr.32475.1.S1_s_at	5.11	3.92	5.37	5.32	5.30	5.34	4.80	0.77	5.32	0.02	1.11	0.149	3.1E-01
Mtr.11080.1.S1_s_at	8.43	6.95	8.04	8.80	9.26	9.10	7.81	0.77	9.06	0.23	1.16	0.214	5.5E-02
Mtr.39779.1.S1_at	7.69	6.96	8.50	7.65	7.80	7.54	7.72	0.77	7.66	0.13	0.99	-0.011	9.1E-01
Mtr.7116.1.S1_at	3.85	3.88	5.20	3.64	3.93	3.51	4.31	0.77	3.69	0.22	0.86	-0.222	2.5E-01
Mtr.6170.1.S1_at	8.42	7.20	8.63	8.19	8.39	7.97	8.08	0.77	8.18	0.21	1.01	0.018	8.4E-01
Mtr.24361.1.S1_at	9.05	7.75	9.12	8.82	8.89	8.85	8.64	0.77	8.85	0.04	1.02	0.035	6.6E-01
Mtr.41297.1.S1_at	5.64	4.65	6.17	7.06	7.80	6.98	5.49	0.77	7.28	0.45	1.33	0.408	2.5E-02
Mtr.39831.1.S1_at	8.11	6.78	8.12	7.95	8.18	8.29	7.67	0.77	8.14	0.17	1.06	0.086	3.6E-01
Mtr.33541.1.S1_x_at	6.23	4.78	5.96	6.33	6.78	6.47	5.65	0.77	6.53	0.23	1.15	0.207	1.3E-01
Mtr.31516.1.S1_s_at	4.22	2.69	3.26	3.34	3.44	3.27	3.39	0.77	3.35	0.08	0.99	-0.018	9.3E-01
Mtr.15379.1.S1_at	7.48	7.59	8.86	8.79	9.59	9.05	7.98	0.77	9.14	0.41	1.15	0.196	8.2E-02
Mtr.23791.1.S1_at	5.83	7.30	6.94	7.36	7.39	7.47	6.69	0.77	7.41	0.06	1.11	0.147	1.8E-01
Mtr.36990.1.S1_at	3.88	2.43	3.59	3.55	3.59	3.64	3.30	0.77	3.59	0.05	1.09	0.122	5.5E-01
Mtr.48952.1.S1_s_at	8.70	10.08	8.79	9.80	10.00	9.73	9.19	0.77	9.84	0.14	1.07	0.099	2.2E-01
Mtr.50473.1.S1_s_at	2.68	4.00	2.66	3.20	3.30	3.28	3.11	0.77	3.26	0.05	1.05	0.066	7.6E-01
Mtr.13254.1.S1_at	5.34	4.58	6.11	5.62	6.45	5.50	5.34	0.77	5.85	0.52	1.10	0.132	3.9E-01
Mtr.21789.1.S1_at	5.86	4.53	5.86	6.61	6.36	5.82	5.42	0.77	6.26	0.41	1.16	0.209	1.7E-01
Mtr.38683.1.S1_at	7.71	6.42	7.79	7.59	7.72	7.51	7.31	0.77	7.60	0.11	1.04	0.058	5.4E-01
Mtr.17333.1.S1_at	7.33	5.99	7.32	7.36	7.22	7.30	6.88	0.77	7.29	0.07	1.06	0.084	4.1E-01
Mtr.43954.1.S1_at	11.47	9.98	11.04	9.93	9.74	9.90	10.83	0.77	9.86	0.10	0.91	-0.136	9.5E-02
Mtr.9321.1.S1_at	10.06	8.52	9.29	9.79	9.67	9.94	9.29	0.77	9.80	0.14	1.05	0.077	3.2E-01
Mtr.1840.1.S1_at	6.78	8.00	6.59	6.76	6.76	6.75	7.12	0.77	6.75	0.00	0.95	-0.077	4.5E-01
Mtr.51529.1.S1_at	7.95	7.01	8.53	8.16	8.22	8.14	7.83	0.77	8.17	0.04	1.04	0.062	4.8E-01
Mtr.17830.1.S1_at	11.81	10.50	10.45	10.95	10.73	10.98	10.92	0.77	10.89	0.14	1.00	-0.004	9.5E-01
Mtr.41505.1.S1_at	6.31	5.07	6.47	6.31	6.57	6.58	5.95	0.77	6.48	0.15	1.09	0.124	3.0E-01
Mtr.8401.1.S1_at	10.02	8.66	9.96	10.00	10.18	9.93	9.55	0.77	10.04	0.13	1.05	0.072	3.4E-01
Mtr.3100.1.S1_at	8.86	10.33	9.23	9.37	9.71	9.37	9.47	0.77	9.48	0.19	1.00	0.001	9.9E-01
Mtr.40730.1.S1_at	8.56	7.60	9.11	8.69	8.57	8.49	8.42	0.77	8.58	0.10	1.02	0.027	7.4E-01
Mtr.35178.1.S1_at	8.34	8.78	7.29	7.30	8.11	7.80	8.14	0.77	7.74	0.41	0.95	-0.073	4.7E-01
Mtr.43966.1.S1_at	9.67	8.20	9.30	9.97	10.10	10.03	9.06	0.77	10.03	0.07	1.11	0.148	9.3E-02
Mtr.31565.1.S1_s_at	9.34	8.08	9.46	9.16	8.63	8.86	8.96	0.77	8.88	0.26	0.99	-0.012	8.8E-01
Mtr.48448.1.S1_at	7.98	6.87	8.34	8.14	7.98	8.06	7.73	0.77	8.06	0.08	1.04	0.060	5.0E-01
Mtr.5197.1.S1_at	3.78	2.59	4.03	3.85	4.15	3.77	3.47	0.77	3.93	0.20	1.13	0.179	3.7E-01
Mtr.19409.1.S1_at	4.11	3.62	5.12	4.48	5.41	4.16	4.28	0.77	4.68	0.65	1.09	0.128	5.3E-01
Mtr.11270.1.S1_at	6.36	4.99	6.27	6.86	6.45	6.32	5.87	0.77	6.54	0.28	1.11	0.156	2.3E-01
Mtr.32486.1.S1_at	7.07	5.68	6.93	7.21	7.05	7.09	6.56	0.77	7.12	0.08	1.08	0.117	2.8E-01
Mtr.50002.1.S1_at	10.16	8.68	9.77	10.77	10.56	10.40	9.53	0.77	10.57	0.19	1.11	0.149	8.4E-02
Mtr.1459.1.S1_at	2.55	3.87	2.53	2.51	2.50	2.48	2.98	0.77	2.50	0.02	0.84	-0.256	3.3E-01
Mtr.41195.1.S1_at	6.08	4.85	6.25	6.15	6.63	6.06	5.73	0.77	6.28	0.31	1.10	0.133	3.1E-01
Mtr.37508.1.S1_at	13.28	12.60	11.75	12.38	12.60	12.54	12.54	0.77	12.51	0.11	1.00	-0.004	9.4E-01
Mtr.51082.1.S1_at	10.85	10.94	9.57	10.43	10.69	10.87	10.45	0.77	10.66	0.22	1.02	0.029	6.7E-01
Mtr.24091.1.S1_at	5.60	4.48	5.95	5.74	5.86	5.01	5.34	0.77	5.54	0.46	1.04	0.051	7.3E-01
Mtr.10450.1.S1_at	10.63	9.10	9.81	9.73	10.41	9.99	9.85	0.77	10.04	0.34	1.02	0.028	7.1E-01
Mtr.27924.1.S1_at	8.06	6.93	8.38	8.02	7.93	7.61	7.79	0.77	7.85	0.22	1.01	0.011	9.0E-01
Mtr.42388.1.S1_at	11.97	10.44	11.05	11.11	11.14	11.19	11.15	0.77	11.15	0.04	1.00	-0.001	9.9E-01
Mtr.41673.1.S1_at	9.40	8.36	9.85	9.14	9.77	9.33	9.20	0.77	9.41	0.32	1.02	0.033	6.8E-01
Mtr.7416.1.S1_at	10.03	9.55	8.53	8.49	8.43	8.57	9.37	0.77	8.50	0.07	0.91	-0.141	1.2E-01
Mtr.4028.1.S1_at	9.54	8.02	8.96	9.03	8.91	8.95	8.84	0.77	8.96	0.06	1.01	0.020	7.9E-01
Mtr.19810.1.S1_at	10.04	8.69	10.00	10.53	10.08	10.47	9.58	0.77	10.36	0.24	1.08	0.113	1.7E-01
Mtr.607.1.S1_at	4.56	3.13	4.32	4.28	4.27	4.27	4.00	0.77	4.27	0.00	1.07	0.095	5.7E-01
Mtr.12961.1.S1_at	12.45	10.93	11.55	11.98	11.91	11.98	11.64	0.77	11.96	0.04	1.03	0.038	5.2E-01
Mtr.31819.1.S1_at	9.56	8.03	8.88	9.18	8.92	9.33	8.82	0.77	9.14	0.21	1.04	0.051	5.3E-01
Mtr.37476.1.S1_at	12.19	12.09	13.46	13.12	13.67	13.49	12.58	0.77	13.43	0.28	1.07	0.094	1.5E-01
Mtr.20077.1.S1_at	6.35	4.87	5.92	5.86	5.93	5.91	5.71	0.77	5.90	0.03	1.03	0.046	6.9E-01
Mtr.50857.1.S1_s_at	10.82	9.61	11.04	10.92	10.85	11.00	10.49	0.77	10.92	0.07	1.04	0.059	3.8E-01
Mtr.45600.1.S1_s_at	8.21	9.16	9.73	9.06	9.40	8.39	9.03	0.77	8.95	0.51	0.99	-0.013	8.9E-01
Mtr.14128.1.S1_at	12.82	11.54	12.90	13.08	12.75	12.92	12.42	0.77	12.92	0.16	1.04	0.057	3.3E-01
Mtr.24705.1.S1_s_at	5.43	4.00	5.20	5.25	5.25	5.30	4.88	0.77	5.27	0.03	1.08	0.111	4.3E-01
Mtr.21806.1.S1_at	2.56	3.86	2.50	2.51	2.52	2.50	2.97	0.77	2.51	0.01	0.84	-0.245	3.5E-01
Mtr.34715.1.S1_at	8.56	7.15	8.37	8.30	8.50	8.50	8.03	0.76	8.43	0.11	1.05	0.071	4.2E-01
Mtr.12687.1.S1_at	8.96	7.47	8.52	9.32	8.17	8.58	8.32	0.76	8.69	0.58	1.04	0.063	5.4E-01
Mtr.35962.1.S1_at	8.57	7.51	9.00	8.91	8.94	8.59	8.36	0.76	8.81	0.20	1.05	0.076	3.8E-01
Mtr.40595.1.S1_at	10.06	8.82	10.22	8.80	9.98	9.55	9.70	0.76	9.44	0.60	0.97	-0.038	6.7E-01
Mtr.17471.1.S1_at	7.54	6.40	7.84	7.56	7.76	7.67	7.26	0.76	7.66	0.10	1.06	0.078	4.2E-01
Mtr.27449.1.S1_at	6.40	5.26	6.71	6.61	6.66	6.60	6.12	0.76	6.62	0.03	1.08	0.114	3.2E-01
Mtr.37113.1.S1_at	2.66	3.98	2.65	2.65	2.61	2.60	3.10	0.76	2.62	0.02	0.85	-0.241	3.4E-01
Mtr.33744.1.S1_at	6.47	5.20	6.57	5.98	6.30	6.12	6.08	0.76	6.13	0.16	1.01	0.012	9.1E-01
Mtr.39509.1.S1_at	6.66	5.13	5.86	6.91	6.40	6.23	5.89	0.76	6.51	0.36	1.11	0.146	2.7E-01
Mtr.19215.1.S1_at	5.72	4.48	5.87	5.46	5.60	5.23	5.36	0.76	5.43	0.18	1.01	0.020	8.7E-01
Mtr.50986.1.S1_at	5.15	4.13	5.62	5.15	5.47	5.13	4.97	0.76	5.25	0.19	1.06	0.080	5.7E-01
Mtr.38765.1.S1_at	10.13	10.00	11.38	10.98	10.83	11.08	10.50	0.76	10.96	0.12	1.04	0.062	3.6E-01
Mtr.17960.1.S1_at	2.77	4.08	2.75	2.77	2.75	2.73	3.20	0.76	2.75	0.02	0.86	-0.218	3.7E-01
Mtr.44422.1.S1_at	8.63	7.36	8.73	8.47	8.64	8.54	8.24	0.76	8.55	0.09	1.04	0.053	5.2E-01
Mtr.4462.1.S1_at	8.31	7.20	8.66	8.57	8.95	8.43	8.05	0.76	8.65	0.27	1.07	0.103	2.7E-01
Mtr.5686.1.S1_at	7.36	5.95	7.15	7.54	7.37	7.31	6.82	0.76	7.41	0.12	1.09	0.120	2.6E-01
Mtr.24898.1.S1_at	3.38	2.88	4.38	3.33	3.33	3.29	3.54	0.76	3.32	0.02	0.94	-0.096	6.3E-01
Mtr.38208.1.S1_at	9.19	7.72	8.83	8.86	8.91	9.11	8.58	0.76	8.96	0.13	1.04	0.062	4.5E-01
Mtr.33257.1.S1_s_at	8.05	6.79	8.17	8.12	8.28	8.22	7.67	0.76	8.21	0.08	1.07	0.098	2.9E-01
Mtr.43646.1.S1_at	8.65	7.19	8.31	8.86	8.92	9.07	8.05	0.76	8.95	0.11	1.11	0.152	1.1E-01
Mtr.41661.1.S1_s_at	3.49	3.12	4.59	3.23	3.95	3.48	3.73	0.76	3.55	0.37	0.95	-0.072	7.3E-01
Mtr.2588.1.S1_at	4.31	3.05	4.43	4.50	4.99	4.41	3.93	0.76	4.63	0.31	1.18	0.237	2.1E-01
Mtr.16739.1.S1_s_at	5.24	6.76	5.94	5.95	6.00	5.95	5.98	0.76	5.97	0.03	1.00	-0.003	9.8E-01
Mtr.49760.1.S1_x_at	4.89	3.57	4.89	6.47	7.21	6.42	4.45	0.76	6.70	0.44	1.51	0.590	1.2E-02
Mtr.45524.1.S1_at	7.64	6.49	7.94	7.11	7.37	7.36	7.36	0.76	7.28	0.15	0.99	-0.015	8.8E-01
Mtr.49448.1.S1_at	12.86	11.40	12.50	12.21	11.47	12.25	12.25	0.76	11.98	0.44	0.98	-0.033	6.2E-01
Mtr.43938.1.S1_at	11.49	9.99	10.99	11.08	11.20	11.12	10.82	0.76	11.14	0.06	1.03	0.041	5.2E-01

Mtr.6747.1.S1_at	4.83	3.85	5.35	4.69	5.09	4.75	4.68	0.76	4.85	0.21	1.04	0.051	7.3E-01
Mtr.20215.1.S1_at	3.76	2.48	2.40	2.34	2.33	2.32	2.88	0.76	2.33	0.01	0.81	-0.305	2.8E-01
Mtr.15856.1.S1_x_at	10.69	10.31	11.78	11.25	11.06	10.51	10.93	0.76	10.94	0.38	1.00	0.002	9.8E-01
Mtr.3845.1.S1_at	7.20	5.81	7.04	8.07	9.32	8.33	6.68	0.76	8.57	0.66	1.28	0.360	3.1E-02
Mtr.603.1.S1_at	5.23	4.19	5.68	5.87	5.78	5.50	5.04	0.76	5.72	0.19	1.14	0.184	2.1E-01
Mtr.39119.1.S1_at	7.99	8.42	9.47	8.64	9.01	8.04	8.63	0.76	8.56	0.49	0.99	-0.011	9.1E-01
Mtr.37617.1.S1_at	9.41	8.19	9.60	9.49	9.54	9.56	9.07	0.76	9.53	0.04	1.05	0.072	3.5E-01
Mtr.10689.1.S1_at	9.35	10.65	9.31	9.83	10.13	9.98	9.77	0.76	9.98	0.15	1.02	0.030	6.7E-01
Mtr.41185.1.S1_at	9.62	8.22	9.42	9.47	9.48	9.42	9.09	0.76	9.46	0.03	1.04	0.058	4.5E-01
Mtr.49479.1.S1_at	7.96	6.56	7.79	7.83	7.87	7.71	7.43	0.76	7.80	0.08	1.05	0.070	4.5E-01
Mtr.26554.1.S1_at	2.73	4.03	2.68	2.67	2.65	2.63	3.15	0.76	2.65	0.02	0.84	-0.247	3.2E-01
Mtr.43157.1.S1_at	9.88	9.37	10.87	10.38	10.07	10.22	10.04	0.76	10.22	0.16	1.02	0.026	7.1E-01
Mtr.23567.1.S1_x_at	9.28	7.81	8.18	7.64	8.26	8.18	8.43	0.76	8.03	0.33	0.95	-0.070	4.5E-01
Mtr.38184.1.S1_at	9.23	7.80	8.96	8.91	8.83	8.64	8.66	0.76	8.79	0.14	1.02	0.022	7.8E-01
Mtr.33733.1.S1_at	5.95	4.50	5.63	5.92	5.98	5.97	5.36	0.76	5.96	0.03	1.11	0.153	2.5E-01
Mtr.15019.1.S1_at	4.66	3.73	5.24	4.56	4.67	4.59	4.54	0.76	4.61	0.06	1.01	0.020	8.9E-01
Mtr.48712.1.S1_s_at	5.23	4.66	6.17	5.42	5.21	5.17	5.35	0.76	5.27	0.13	0.98	-0.023	8.6E-01
Mtr.33511.1.S1_at	5.39	4.07	5.39	4.55	4.60	4.42	4.95	0.76	4.52	0.09	0.91	-0.131	3.9E-01
Mtr.6955.1.S1_at	4.61	5.91	4.57	6.02	6.42	5.85	5.03	0.76	6.10	0.29	1.21	0.278	8.6E-02
Mtr.45091.1.S1_x_at	4.71	3.42	4.77	5.01	5.83	5.08	4.30	0.76	5.31	0.46	1.23	0.304	1.2E-01
Mtr.2672.1.S1_at	4.91	3.45	4.55	4.53	4.99	4.53	4.31	0.76	4.68	0.27	1.09	0.121	4.6E-01
Mtr.51569.1.S1_at	8.62	7.11	8.04	8.06	8.31	8.11	7.92	0.76	8.16	0.13	1.03	0.043	6.2E-01
Mtr.18558.1.S1_at	7.30	5.98	7.29	7.21	7.17	7.04	6.86	0.76	7.14	0.09	1.04	0.059	5.6E-01
Mtr.39607.1.S1_at	7.30	5.84	6.96	7.00	7.26	7.01	6.70	0.76	7.09	0.15	1.06	0.081	4.4E-01
Mtr.16922.1.S1_at	8.10	6.78	8.09	8.03	7.93	8.09	7.66	0.76	8.02	0.08	1.05	0.066	4.6E-01
Mtr.5404.1.S1_at	8.38	6.97	8.17	8.19	8.50	8.10	7.84	0.76	8.27	0.21	1.05	0.077	4.0E-01
Mtr.47718.1.S1_at	2.36	3.67	2.34	2.32	2.33	2.34	2.79	0.76	2.33	0.01	0.84	-0.260	3.5E-01
Mtr.42375.1.S1_at	7.75	9.12	9.01	11.06	11.75	11.08	8.63	0.76	11.30	0.40	1.31	0.389	5.7E-03
Mtr.3373.1.S1_at	9.05	8.43	9.94	8.48	8.48	8.55	9.14	0.76	8.50	0.04	0.93	-0.104	2.2E-01
Mtr.1427.1.S1_at	9.13	9.45	10.58	8.96	9.38	9.26	9.72	0.76	9.20	0.22	0.95	-0.079	3.2E-01
Mtr.5431.1.S1_at	8.65	7.17	8.21	8.44	8.36	8.63	8.01	0.76	8.48	0.14	1.06	0.081	3.6E-01
Mtr.22036.1.S1_at	5.58	5.01	6.52	5.78	6.47	4.88	5.70	0.76	5.71	0.79	1.00	0.002	9.9E-01
Mtr.13242.1.S1_at	7.66	6.23	6.50	7.10	6.54	7.09	6.80	0.76	6.91	0.32	1.02	0.023	8.3E-01
Mtr.21664.1.S1_at	6.70	5.31	6.54	6.80	7.27	6.97	6.18	0.76	7.01	0.24	1.13	0.182	1.5E-01
Mtr.7855.1.S1_at	3.28	3.30	4.61	3.24	3.23	2.94	3.73	0.76	3.14	0.17	0.84	-0.250	2.6E-01
Mtr.27917.1.S1_at	8.64	7.46	8.88	8.52	9.03	8.55	8.33	0.76	8.70	0.29	1.04	0.063	4.7E-01
Mtr.9808.1.S1_at	6.82	5.84	7.33	6.67	6.23	5.71	6.66	0.76	6.20	0.48	0.93	-0.103	4.3E-01
Mtr.18393.1.S1_x_at	2.96	4.26	2.94	2.94	2.87	2.94	3.38	0.76	2.92	0.04	0.86	-0.215	3.5E-01
Mtr.45872.1.S1_at	7.04	5.75	7.09	7.00	6.80	6.86	6.63	0.76	6.89	0.10	1.04	0.055	5.9E-01
Mtr.10894.1.S1_at	5.75	4.29	5.41	5.75	6.10	6.46	5.15	0.76	6.10	0.35	1.19	0.245	1.2E-01
Mtr.33773.1.S1_at	7.28	6.01	7.38	7.20	7.88	7.50	6.89	0.76	7.53	0.34	1.09	0.128	2.6E-01
Mtr.5349.1.S1_s_at	8.14	6.85	8.19	8.15	8.32	7.87	7.73	0.76	8.11	0.23	1.05	0.070	4.5E-01
Mtr.6123.1.S1_s_at	7.15	6.54	8.05	7.21	7.42	7.43	7.25	0.76	7.35	0.12	1.01	0.021	8.2E-01
Mtr.29895.1.S1_at	3.45	2.25	3.66	3.26	3.41	3.30	3.12	0.76	3.32	0.07	1.06	0.090	6.7E-01
Mtr.4049.1.S1_at	8.51	7.30	8.71	8.58	8.76	8.34	8.17	0.76	8.56	0.21	1.05	0.066	4.5E-01
Mtr.26833.1.S1_at	4.88	5.44	6.39	5.72	5.15	5.93	5.57	0.76	5.60	0.40	1.00	0.007	9.6E-01
Mtr.7506.1.S1_at	8.78	7.30	8.31	8.24	8.36	8.14	8.13	0.76	8.25	0.11	1.01	0.021	8.0E-01
Mtr.7410.1.S1_at	7.51	6.58	8.08	7.86	8.00	7.16	7.39	0.76	7.67	0.45	1.04	0.055	6.1E-01
Mtr.35867.1.S1_at	10.18	8.67	9.35	9.57	9.41	9.51	9.40	0.76	9.50	0.08	1.01	0.014	8.4E-01
Mtr.49774.1.S1_at	5.99	4.56	4.84	5.96	5.96	7.04	5.13	0.76	6.32	0.62	1.23	0.301	1.0E-01
Mtr.25405.1.S1_x_at	2.70	4.00	2.66	2.64	2.63	2.60	3.12	0.76	2.62	0.02	0.84	-0.250	3.2E-01
Mtr.6551.1.S1_at	8.80	7.51	8.84	8.76	8.44	8.50	8.38	0.76	8.57	0.17	1.02	0.031	7.0E-01
Mtr.33018.1.S1_at	5.76	4.65	4.31	4.50	4.24	4.54	4.90	0.76	4.43	0.16	0.90	-0.148	3.5E-01
Mtr.40708.1.S1_at	7.85	6.82	8.29	7.85	8.09	7.85	7.65	0.76	7.93	0.14	1.04	0.051	5.7E-01
Mtr.51559.1.S1_at	7.45	6.34	7.79	7.40	7.36	7.06	7.19	0.76	7.27	0.19	1.01	0.016	8.7E-01
Mtr.16192.1.S1_at	10.46	8.98	10.01	10.09	10.27	9.95	9.82	0.76	10.10	0.16	1.03	0.041	5.6E-01
Mtr.26514.1.S1_s_at	7.89	6.53	7.78	7.84	7.95	7.76	7.40	0.76	7.85	0.09	1.06	0.086	3.6E-01
Mtr.41376.1.S1_at	5.47	4.15	5.46	4.91	5.06	5.06	5.02	0.76	5.01	0.08	1.00	-0.004	9.8E-01
Mtr.52358.1.S1_at	3.32	4.62	3.29	3.32	3.27	3.85	3.74	0.76	3.48	0.32	0.93	-0.105	6.1E-01
Mtr.43554.1.S1_at	11.11	9.76	11.03	11.58	11.41	11.75	10.64	0.76	11.58	0.17	1.09	0.123	1.0E-01
Mtr.51518.1.S1_x_at	1.86	3.16	1.83	1.97	1.87	1.87	2.28	0.76	1.90	0.06	0.83	-0.261	4.4E-01
Mtr.41435.1.S1_s_at	8.70	7.90	7.18	8.01	7.77	8.01	7.93	0.76	7.93	0.14	1.00	0.000	1.0E+00
Mtr.5373.1.S1_at	5.25	4.74	6.23	5.49	5.31	5.31	5.41	0.76	5.37	0.11	0.99	-0.009	9.4E-01
Mtr.43202.1.S1_at	11.93	10.42	11.18	11.97	12.12	12.18	11.18	0.76	12.09	0.11	1.08	0.113	1.1E-01
Mtr.32773.1.S1_at	4.55	3.06	4.04	5.11	4.19	4.18	3.88	0.76	4.49	0.53	1.16	0.210	3.2E-01
Mtr.10504.1.S1_at	7.62	6.25	7.48	7.61	8.07	7.42	7.12	0.76	7.70	0.33	1.08	0.114	2.9E-01
Mtr.48248.1.S1_at	4.16	2.81	4.08	4.07	3.99	4.22	3.68	0.76	4.10	0.12	1.11	0.153	4.0E-01
Mtr.10743.1.S1_at	10.02	8.59	9.75	9.92	9.89	10.00	9.45	0.76	9.93	0.06	1.05	0.072	3.3E-01
Mtr.35697.1.S1_s_at	2.49	2.68	3.89	6.36	6.97	5.89	3.02	0.76	6.40	0.54	2.12	1.083	3.3E-03
Mtr.42591.1.S1_s_at	4.41	3.46	4.96	4.72	4.75	4.29	4.28	0.76	4.59	0.26	1.07	0.100	5.4E-01
Mtr.1758.1.S1_at	8.65	7.14	7.75	8.52	8.43	8.73	7.85	0.76	8.56	0.16	1.09	0.125	1.9E-01
Mtr.41664.1.S1_at	9.92	8.41	9.26	9.16	9.54	9.33	9.20	0.76	9.35	0.19	1.02	0.023	7.6E-01
Mtr.20350.1.S1_s_at	2.43	3.73	2.40	2.39	2.40	2.39	2.85	0.76	2.39	0.01	0.84	-0.252	3.5E-01
Mtr.33099.1.S1_at	7.56	6.08	7.09	7.52	7.30	7.35	6.91	0.76	7.39	0.12	1.07	0.097	3.4E-01
Mtr.19660.1.S1_at	5.68	4.50	5.91	5.34	5.76	5.63	5.36	0.76	5.58	0.21	1.04	0.056	6.6E-01
Mtr.32760.1.S1_at	5.20	4.03	5.45	5.97	5.55	5.56	4.89	0.76	5.69	0.24	1.16	0.218	1.6E-01
Mtr.21947.1.S1_at	2.99	4.26	2.91	2.79	2.87	2.86	3.39	0.76	2.84	0.04	0.84	-0.254	2.8E-01
Mtr.5221.1.S1_at	4.80	3.37	4.50	4.21	4.61	4.44	4.22	0.76	4.42	0.20	1.05	0.066	6.9E-01
Mtr.8207.1.S1_at	2.26	2.35	3.62	2.24	2.24	2.23	2.74	0.76	2.24	0.01	0.82	-0.293	3.1E-01
Mtr.6717.1.S1_at	7.27	6.17	7.62	7.26	7.39	6.88	7.02	0.76	7.18	0.27	1.02	0.031	7.6E-01
Mtr.39098.1.S1_at	6.24	5.53	7.04	6.92	6.80	6.66	6.27	0.76	6.79	0.13	1.08	0.116	3.0E-01
Mtr.44969.1.S1_at	8.53	7.28	8.63	8.30	8.49	8.42	8.15	0.76	8.40	0.10	1.03	0.045	5.9E-01
Mtr.33734.1.S1_at	7.57	6.85	8.36	8.11	7.99	8.12	7.59	0.76	8.08	0.07	1.06	0.089	3.3E-01
Mtr.410.1.S1_s_at	14.44	12.93	13.69	13.94	13.68	14.15	13.69	0.76	13.92	0.23	1.02	0.025	6.3E-01
Mtr.2569.1.S1_at	7.63	6.15	7.14	6.77	6.92	6.81	6.97	0.76	6.83	0.08	0.98	-0.030	7.6E-01
Mtr.2548.1.S1_s_at	8.21	6.87	8.14	8.34	8.48	8.69	7.74	0.76	8.50	0.18	1.10	0.135	1.6E-01
Mtr.28019.1.S1_at	6.79	5.91	7.42	7.27	7.19	7.23	6.71	0.76	7.23	0.04	1.08	0.109	2.9E-01
Mtr.28286.1.S1_at	5.95	4.64	5.95	5.94	5.94	5.95	5.51	0.76	5.94	0.01	1.08	0.109	3.8E-01
Mtr.35931.1.S1_at	7.96	6.53	7.69	7.64	7.75	7.73	7.39	0.76	7.71	0.05	1.04	0.060	5.1E-01
Mtr.20933.1.S1_at	7.09	5.97	7.41	7.07	7.20	7.11	6.82	0.76	7.13	0.06	1.04	0.062	5.3E-01
Mtr.42193.1.S1_at	8.63	7.37	8.71	8.60	8.61	8.63	8.24	0.75	8.61	0.01	1.05	0.065	4.4E-01
Mtr.9731.1.S1_at	6.19	5.46	6.97	6.88	6.92	6.71	6.21	0.75	6.84	0.11	1.10	0.139	2.3E-01
Mtr.10849.1.S1_at	8.66	7.31	8.57	8.63	8.47	8.62	8.18	0.75	8.57	0.09	1.05	0.068	4.2E-01
Mtr.12814.1.S1_at	8.05	6.80	8.17	7.98	8.24	8.12	7.67	0.75	8.11	0.13	1.06	0.080	3.8E-01
Mtr.22643.1.S1_at	3.26	4.08	2.57	5.45	5.65	3.66	3.30	0.75	4.92	1.10	1.49	0.576	1.0E-01
Mtr.10216.1.S1_at	6.31	5.06	6.41	6.43	6.90	6.69	5.93	0.75	6.68	0.24	1.13	0.172	1.8E-01
Mtr.18997.1.S1_at	9.69	9.35	8.25	9.20	9.03	9.44	9.09	0.75	9.23	0.21	1.01	0.021	7.8E-01
Mtr.34372.1.S1_at	6.56	7.65	8.00	7.96	8.45	7.57	7.40	0.75	7.99	0.44	1.08	0.110	3.1E-01
Mtr.40483.1.S1_at	10.36	9.18	10.58	10.26	10.34	10.00	10.04	0.75	10.20	0.18	1.02	0.023	7.4E-01
Mtr.4484.1.S1_s_at	5.85	5.86	7.16	6.24	5.91	5.73	6.29	0.75	5.96	0.26	0.95	-0.077	5.1E-01
Mtr.1821.1.S1_s_at	4.92	3.43	4.39	5.00	5.07	4.91	4.25	0.75	4.99	0.08	1.18	0.233	1.6E-01
Mtr.14830.1.S1_at	7.80	6.76	8.23	8.31	8.52	8.07	7.60	0.75	8.30	0.22	1.09	0.128	2.0E-01
Mtr.40788.1.S1_at	7.26	5.76	6.42	6.19	6.17	6.24	6.48	0.75	6.20	0.04	0.96	-0.064	5.5E-01
Mtr.33562.1.S1_at	4.50	3.01	3.55	5.07	4.93	4.75	3.68	0.75	4.91	0.16	1.33	0.416	5.1E-02
Mtr.23448.1.S1_at	7.34	6.31	7.78	7.52	7.74	7.69	7.14	0.75	7.65	0.12	1.07	0.100	3.1E-01
Mtr.32236.1.S1_at	7.63	6.21	7.37	7.62	7.27	7.53	7.07	0.75	7.48	0.18	1.06	0.081	4.2E-01
Mtr.22455.1.S1_at	7.03	7.23	8.43	7.63	7.46	7.28	7.57	0.75	7.46	0.18	0.99	-0.021	8.2E-01
Mtr.34002.1.S1_s_at	6.76	5.50	6.84	6.84	6.80	6.89	6.37	0.75	6.84	0.05	1.07	0.104	3.4E-01
Mtr.43328.1.S1_at	8.12	6.84	8.17	8.03	8.40	7.99	7.71	0.75	8.14	0.23	1.06	0.078	4.0E-01
Mtr.27160.1.S1_s_at	3.03	4.07	4.50	5.38	6.53	6.51	3.87	0.75	6.14	0.66	1.59	0.666	1.7E-02
Mtr.15478.1.S1_s_at	7.42	6.36	7.82	6.97	7.29	6.59	7.20	0.75	6.95	0.35	0.97	-0.051	6.3E-01
Mtr.16850.1.S1_at	6.36	4.86	5.71	6.08	6.42	6.58	5.64	0.75	6.36	0.26	1.13	0.172	1.9E-01
Mtr.5639.1.S1_at	5.11	3.70	4.87	4.97	4.77	5.10	4.56	0.75	4.95	0.17	1.08	0.117	4.4E-01
Mtr.36947.1.S1_at	2.14	3.43	2.12	2.12	2.11	2.11	2.56	0.75	2.11	0.01	0.83	-0.277	3.6E-01
Mtr.5736.1.S1_at	7.66	6.45	7.83	7.81	7.62	7.74	7.31	0.75	7.72	0.10	1.06	0.079	4.0E-01
Mtr.20285.1.S1_at	6.59	5.23	6.47	6.20	6.42	6.56	6.10	0.75	6.39	0.18	1.05	0.068	5.4E-01
Mtr.780.1.S1_at	9.49	8.14	9.41	9.01	8.80	9.34	9.01	0.75	9.05	0.27	1.00	0.006	9.4E-01
Mtr.46207.1.S1_at	5.19	3.99	5.38	6.08	5.91	5.44	4.86	0.75	5.81	0.33	1.20	0.259	1.1E-01
Mtr.42264.1.S1_at	6.03	5.46	6.95	6.46	6.44	6.51	6.14	0.75	6.47	0.04	1.05	0.075	4.9E-01
Mtr.22750.1.S1_at	5.22	4.50	6.00	5.20	5.26	4.99	5.24	0.75	5.15	0.14	0.98	-0.024	8.6E-01
Mtr.2846.1.S1_at	11.77	10.28	10.83	11.21	10.79	11.09	10.96	0.75	11.03	0.22	1.01	0.009	8.9E-01
Mtr.8498.1.S1_at	13.66	12.47	12.27	13.00	13.44	13.21	12.80	0.75	13.21	0.22	1.03	0.046	4.1E-01
Mtr.2690.1.S1_at	6.42	5.34	6.78	6.79	6.94	6.75	6.18	0.75	6.83	0.10	1.10	0.144	2.1E-01
Mtr.17316.1.S1_at	8.24	9.02	9.74	8.96	9.10	8.84	9.00	0.75	8.97	0.13	1.00	-0.005	9.4E-01
Mtr.43279.1.S1_at	5.10	6.42	5.13	5.14	5.05	4.89	5.55	0.75	5.03	0.12	0.91	-0.142	3.0E-01
Mtr.20635.1.S1_at	2.41	3.71	2.39	2.38	2.38	2.37	2.84	0.75	2.38	0.01	0.84	-0.254	3.5E-01
Mtr.39474.1.S1_at	8.06	6.97	8.42	8.39	7.84	8.15	7.82	0.75	8.13	0.28	1.04	0.056	5.4E-01
Mtr.41303.1.S1_at	10.98	12.49	11.72	12.38	12.54	12.11	11.73	0.75	12.34	0.22	1.05	0.073	2.5E-01
Mtr.34513.1.S1_at	4.37	5.66	4.36	4.49	4.43	4.35	4.80	0.75	4.43	0.07	0.92	-0.116	4.4E-01
Mtr.2155.1.S1_at	5.41	3.97	5.09	5.04	4.82	4.53	4.82	0.75	4.80	0.26	0.99	-0.008	9.6E-01
Mtr.5868.1.S1_s_at	6.35	5.19	6.59	5.95	6.54	6.29	6.04	0.75	6.26	0.30	1.04	0.051	6.7E-01

Mtr.40700.1.S1_at	11.86	10.81	12.26	12.86	13.30	13.07	11.64	0.75	13.07	0.22	1.12	0.167	3.4E-02
Mtr.42578.1.S1_at	7.51	6.32	7.71	7.61	7.72	7.60	7.18	0.75	7.65	0.06	1.06	0.091	3.4E-01
Mtr.12559.1.S1_s_at	2.82	3.10	4.24	4.38	4.93	4.14	3.39	0.75	4.49	0.41	1.32	0.405	9.0E-02
Mtr.11553.1.S1_at	7.27	8.59	8.56	7.38	6.88	7.27	8.14	0.75	7.18	0.26	0.88	-0.182	1.0E-01
Mtr.44859.1.S1_at	7.95	6.45	7.22	7.10	7.54	7.39	7.21	0.75	7.34	0.22	1.02	0.027	7.8E-01
Mtr.12930.1.S1_at	9.41	7.99	8.28	8.53	8.13	8.61	8.56	0.75	8.42	0.26	0.98	-0.023	7.8E-01
Mtr.1133.1.S1_at	3.25	4.57	3.30	3.34	3.47	3.21	3.71	0.75	3.34	0.13	0.90	-0.150	4.5E-01
Mtr.8572.1.S1_at	14.74	13.24	13.99	14.10	13.77	14.26	13.99	0.75	14.04	0.25	1.00	0.005	9.2E-01
Mtr.51819.1.S1_at	6.00	4.52	5.46	5.98	5.78	5.86	5.33	0.75	5.87	0.10	1.10	0.140	2.8E-01
Mtr.9281.1.S1_at	11.19	9.72	10.17	10.12	10.05	10.12	10.36	0.75	10.09	0.04	0.97	-0.038	5.7E-01
Mtr.39434.1.S1_at	6.87	5.37	6.15	6.96	7.33	7.31	6.13	0.75	7.20	0.20	1.17	0.232	7.6E-02
Mtr.37749.1.S1_at	11.21	9.80	10.07	10.02	9.83	9.93	10.36	0.75	9.93	0.10	0.96	-0.061	3.8E-01
Mtr.37174.1.S1_at	4.97	5.01	6.29	4.99	5.17	5.26	5.42	0.75	5.14	0.14	0.95	-0.077	5.5E-01
Mtr.36338.1.S1_at	6.93	5.91	7.38	6.85	7.31	6.64	6.74	0.75	6.94	0.34	1.03	0.041	7.0E-01
Mtr.19446.1.S1_at	5.25	3.78	4.78	5.29	5.18	4.50	4.60	0.75	4.99	0.43	1.08	0.116	4.8E-01
Mtr.30694.1.S1_at	7.79	6.48	7.77	7.72	7.76	7.50	7.35	0.75	7.66	0.14	1.04	0.059	5.2E-01
Mtr.5822.1.S1_at	6.64	5.44	6.82	5.98	5.90	5.78	6.30	0.75	5.89	0.10	0.93	-0.098	4.0E-01
Mtr.35273.1.S1_at	7.51	6.14	7.36	7.42	7.80	7.31	7.01	0.75	7.51	0.26	1.07	0.100	3.3E-01
Mtr.42570.1.S1_at	7.33	5.96	7.18	7.39	7.30	7.36	6.82	0.75	7.35	0.05	1.08	0.108	2.9E-01
Mtr.42902.1.S1_at	10.00	8.70	8.70	8.82	8.29	8.71	9.13	0.75	8.60	0.28	0.94	-0.086	3.2E-01
Mtr.10924.1.S1_at	9.16	8.05	9.47	9.31	9.25	9.26	8.89	0.75	9.27	0.03	1.04	0.060	4.3E-01
Mtr.11463.1.S1_at	4.28	3.09	4.48	4.34	4.75	4.34	3.95	0.75	4.48	0.24	1.13	0.182	3.1E-01
Mtr.11243.1.S1_at	8.35	6.99	8.22	8.20	7.89	8.17	7.86	0.75	8.09	0.17	1.03	0.042	6.3E-01
Mtr.29449.1.S1_at	9.13	7.80	9.06	9.23	9.72	9.24	8.66	0.75	9.39	0.28	1.08	0.117	1.9E-01
Mtr.37989.1.S1_at	9.13	8.66	10.12	8.89	8.70	8.64	9.30	0.75	8.74	0.13	0.94	-0.090	2.7E-01
Mtr.44988.1.S1_at	5.74	5.09	6.58	6.35	6.51	6.14	5.80	0.75	6.33	0.19	1.09	0.127	3.0E-01
Mtr.34769.1.S1_at	7.49	6.63	8.12	7.54	7.84	7.50	7.41	0.75	7.63	0.19	1.03	0.041	6.6E-01
Mtr.36014.1.S1_at	7.01	5.80	7.16	6.91	6.90	6.74	6.66	0.75	6.85	0.10	1.03	0.041	6.8E-01
Mtr.10731.1.S1_at	11.13	9.65	10.17	10.00	10.02	10.21	10.32	0.75	10.08	0.12	0.98	-0.034	6.1E-01
Mtr.5715.1.S1_at	6.56	5.73	7.23	6.82	7.04	6.87	6.51	0.75	6.91	0.12	1.06	0.087	4.1E-01
Mtr.38134.1.S1_at	7.44	6.52	8.01	7.72	7.97	7.54	7.32	0.75	7.74	0.22	1.06	0.081	4.0E-01
Mtr.7532.1.S1_at	6.76	5.67	7.10	7.12	7.36	6.91	6.51	0.75	7.13	0.23	1.10	0.132	2.4E-01
Mtr.21846.1.S1_at	8.05	6.62	7.72	7.82	7.88	7.81	7.47	0.75	7.84	0.04	1.05	0.070	4.4E-01
Mtr.3.1.S1_at	4.50	4.43	3.17	4.69	3.99	4.10	4.03	0.75	4.26	0.38	1.06	0.079	6.6E-01
Mtr.27560.1.S1_at	4.07	3.22	4.71	4.72	4.20	4.15	4.00	0.75	4.36	0.31	1.09	0.123	4.9E-01
Mtr.25019.1.S1_at	4.35	3.63	5.13	6.43	5.40	6.16	4.37	0.75	5.99	0.54	1.37	0.455	3.8E-02
Mtr.23615.1.S1_at	5.65	4.31	5.55	6.86	6.91	6.87	5.17	0.75	6.88	0.03	1.33	0.413	1.7E-02
Mtr.19414.1.S1_at	9.19	8.27	9.75	9.42	9.61	9.21	9.07	0.75	9.41	0.20	1.04	0.054	4.8E-01
Mtr.12005.1.S1_at	8.13	6.63	7.48	8.13	8.00	8.22	7.41	0.75	8.12	0.11	1.10	0.131	1.8E-01
Mtr.4820.1.S1_at	6.01	5.52	6.99	6.34	6.27	6.48	6.17	0.75	6.36	0.11	1.03	0.044	6.9E-01
Mtr.10481.1.S1_at	9.72	8.74	10.21	9.94	10.14	9.88	9.56	0.75	9.99	0.14	1.04	0.063	3.8E-01
Mtr.9924.1.S1_at	7.98	8.61	7.12	7.70	7.52	7.62	7.90	0.75	7.61	0.09	0.96	-0.053	5.5E-01
Mtr.12684.1.S1_at	8.94	7.45	8.13	8.45	8.31	8.35	8.17	0.75	8.37	0.07	1.02	0.034	6.7E-01
Mtr.12275.1.S1_at	4.25	2.75	3.56	3.60	2.94	3.44	3.52	0.75	3.33	0.34	0.95	-0.081	7.1E-01
Mtr.21036.1.S1_at	8.49	7.05	8.14	7.83	8.14	7.67	7.89	0.75	7.88	0.24	1.00	-0.003	9.8E-01
Mtr.2880.1.S1_at	2.89	4.18	2.87	2.88	2.87	2.87	3.31	0.75	2.88	0.00	0.87	-0.205	3.7E-01
Mtr.12667.1.S1_at	9.93	8.86	10.31	10.05	9.90	10.05	9.70	0.75	10.00	0.08	1.03	0.044	5.3E-01
Mtr.6083.1.S1_s_at	7.75	6.54	7.91	7.99	7.68	8.08	7.40	0.75	7.92	0.21	1.07	0.097	3.1E-01
Mtr.1401.1.S1_at	8.03	6.71	7.98	7.92	8.04	7.66	7.58	0.75	7.87	0.19	1.04	0.055	5.4E-01
Mtr.16209.1.S1_at	14.25	12.93	12.98	13.36	13.37	13.68	13.38	0.75	13.47	0.18	1.01	0.009	8.5E-01
Mtr.35662.1.S1_at	9.14	7.81	9.07	8.97	9.02	8.91	8.68	0.75	8.97	0.05	1.03	0.048	5.4E-01
Mtr.41544.1.S1_at	10.29	8.98	9.01	9.50	9.30	9.70	9.42	0.75	9.50	0.20	1.01	0.012	8.7E-01
Mtr.31781.1.S1_at	8.31	7.11	8.49	8.32	8.49	8.11	7.97	0.75	8.31	0.19	1.04	0.060	4.9E-01
Mtr.19134.1.S1_at	5.74	7.02	5.72	5.79	5.56	5.49	6.16	0.75	5.61	0.15	0.91	-0.135	2.8E-01
Mtr.27746.1.S1_at	3.16	3.00	4.37	3.18	5.25	4.30	3.51	0.75	4.24	1.03	1.21	0.273	3.8E-01
Mtr.40845.1.S1_at	8.34	7.12	8.49	8.29	8.20	8.41	7.98	0.75	8.30	0.11	1.04	0.057	5.0E-01
Mtr.10229.1.S1_at	12.12	10.70	11.80	12.05	11.39	12.01	11.54	0.75	11.82	0.37	1.02	0.034	6.0E-01
Mtr.7373.1.S1_at	9.74	8.58	9.97	9.95	9.99	9.94	9.43	0.75	9.96	0.03	1.06	0.078	2.9E-01
Mtr.12009.1.S1_at	8.27	6.91	8.12	8.08	8.13	8.13	7.77	0.75	8.11	0.03	1.04	0.062	4.7E-01
Mtr.26298.1.S1_at	2.65	3.92	2.61	2.58	2.58	2.56	3.06	0.75	2.57	0.01	0.84	-0.250	3.2E-01
Mtr.1526.1.S1_a_at	10.03	8.57	9.04	9.56	9.92	9.77	9.21	0.75	9.75	0.18	1.06	0.082	2.9E-01
Mtr.45276.1.S1_at	4.83	3.54	4.83	5.96	5.54	5.93	4.40	0.75	5.81	0.23	1.32	0.401	3.5E-02
Mtr.30558.1.S1_at	2.12	3.41	2.11	2.10	2.10	2.10	2.54	0.75	2.10	0.00	0.83	-0.275	3.6E-01
Mtr.10309.1.S1_at	13.76	12.28	12.87	12.83	13.18	12.96	12.97	0.75	12.99	0.18	1.00	0.002	9.7E-01
Mtr.12624.1.S1_at	7.76	6.40	6.55	6.95	6.84	6.82	6.90	0.75	6.87	0.07	1.00	-0.006	9.5E-01
Mtr.32702.1.S1_at	11.53	10.05	10.63	10.87	11.07	11.11	10.74	0.75	11.02	0.13	1.03	0.037	5.5E-01
Mtr.29137.1.S1_at	8.57	7.23	8.46	8.73	8.59	8.53	8.08	0.75	8.62	0.10	1.07	0.092	2.9E-01
Mtr.12740.1.S1_at	11.48	10.26	10.13	11.16	11.00	11.26	10.62	0.75	11.14	0.13	1.05	0.069	3.0E-01
Mtr.4145.1.S1_at	6.88	5.46	6.56	6.49	6.91	6.58	6.30	0.75	6.66	0.22	1.06	0.079	4.7E-01
Mtr.18310.1.S1_at	7.07	5.93	7.33	6.89	6.86	6.93	6.78	0.75	6.89	0.04	1.02	0.025	8.0E-01
Mtr.39957.1.S1_s_at	5.99	4.84	6.23	6.30	6.23	6.42	5.69	0.75	6.32	0.09	1.11	0.151	2.2E-01
Mtr.36351.1.S1_at	9.36	8.11	9.43	9.22	9.21	9.04	8.97	0.74	9.16	0.10	1.02	0.031	6.8E-01
Mtr.28304.1.S1_at	6.44	5.52	7.00	6.34	6.86	6.12	6.32	0.74	6.44	0.38	1.02	0.028	8.1E-01
Mtr.45960.1.S1_at	7.24	6.16	7.59	7.61	7.69	7.48	6.99	0.74	7.59	0.11	1.09	0.119	2.4E-01
Mtr.15081.1.S1_at	10.92	9.55	9.73	10.20	10.05	10.47	10.06	0.74	10.24	0.21	1.02	0.025	7.1E-01
Mtr.21711.1.S1_at	9.69	10.92	9.59	10.04	10.13	10.38	10.07	0.74	10.18	0.18	1.01	0.017	8.0E-01
Mtr.4737.1.S1_at	3.39	3.04	4.47	3.30	3.29	3.26	3.63	0.74	3.28	0.02	0.90	-0.146	4.6E-01
Mtr.43956.1.S1_at	9.46	8.52	9.99	9.67	10.37	9.86	9.32	0.74	9.96	0.36	1.07	0.096	2.5E-01
Mtr.28293.1.S1_at	10.12	9.18	10.65	11.09	11.49	11.08	9.98	0.74	11.22	0.23	1.12	0.168	5.2E-02
Mtr.13506.1.S1_at	8.48	7.44	8.88	8.92	9.03	8.89	8.27	0.74	8.95	0.08	1.08	0.114	1.9E-01
Mtr.40782.1.S1_at	10.92	9.51	9.81	9.86	10.00	9.90	10.08	0.74	9.92	0.07	0.98	-0.024	7.2E-01
Mtr.50675.1.S1_at	6.83	5.46	6.65	6.80	6.42	6.50	6.31	0.74	6.57	0.20	1.04	0.058	5.9E-01
Mtr.9796.1.S1_at	10.54	9.96	9.06	9.66	9.68	9.91	9.85	0.74	9.75	0.14	0.99	-0.015	8.3E-01
Mtr.49101.1.S1_at	9.02	7.54	8.20	8.41	8.70	8.64	8.25	0.74	8.58	0.15	1.04	0.057	4.9E-01
Mtr.41284.1.S1_at	4.19	2.79	3.93	3.97	5.04	3.97	3.64	0.74	4.33	0.61	1.19	0.251	2.8E-01
Mtr.43009.1.S1_s_at	14.54	13.11	14.19	14.42	14.35	14.54	13.95	0.74	14.44	0.09	1.04	0.050	3.2E-01
Mtr.29081.1.S1_at	6.96	5.59	6.78	8.14	8.68	8.39	6.45	0.74	8.40	0.27	1.30	0.382	1.3E-02
Mtr.14010.1.S1_at	7.50	6.47	7.92	7.36	7.28	7.38	7.29	0.74	7.34	0.05	1.01	0.009	9.2E-01
Mtr.36890.1.S1_at	3.14	2.49	3.98	3.15	3.76	3.21	3.20	0.74	3.37	0.33	1.05	0.074	7.4E-01
Mtr.30462.1.S1_at	2.82	2.76	4.08	2.67	3.66	2.66	3.22	0.74	2.99	0.57	0.93	-0.105	7.0E-01
Mtr.39026.1.S1_at	6.32	4.91	6.02	6.40	6.44	7.00	5.75	0.74	6.62	0.34	1.15	0.202	1.4E-01
Mtr.12056.1.S1_s_at	4.27	2.99	2.97	2.95	2.95	2.83	3.41	0.74	2.91	0.07	0.85	-0.226	3.2E-01
Mtr.2673.1.S1_s_at	9.05	8.26	9.74	9.86	10.40	10.07	9.02	0.74	10.11	0.27	1.12	0.165	7.5E-02
Mtr.48202.1.S1_at	5.75	4.41	5.65	5.54	5.73	5.64	5.27	0.74	5.64	0.09	1.07	0.097	4.4E-01
Mtr.2873.1.S1_s_at	8.13	7.11	8.55	8.04	8.22	8.09	7.93	0.74	8.12	0.09	1.02	0.034	6.8E-01
Mtr.51843.1.S1_x_at	5.42	3.94	4.66	5.10	5.95	5.61	4.67	0.74	5.55	0.43	1.19	0.249	1.5E-01
Mtr.5623.1.S1_s_at	5.80	4.51	5.79	5.85	5.82	5.81	5.37	0.74	5.83	0.02	1.09	0.118	3.5E-01
Mtr.2468.1.S1_at	5.51	4.11	5.23	5.58	5.45	5.58	4.95	0.74	5.54	0.08	1.12	0.162	2.5E-01
Mtr.27239.1.S1_at	6.97	5.56	6.67	6.66	6.86	6.55	6.40	0.74	6.69	0.16	1.05	0.064	5.4E-01
Mtr.11952.1.S1_s_at	6.73	5.39	6.62	6.37	6.63	6.38	6.25	0.74	6.46	0.15	1.03	0.049	6.5E-01
Mtr.38177.1.S1_at	7.20	6.98	8.36	7.98	7.85	7.78	7.51	0.74	7.87	0.10	1.05	0.067	4.6E-01
Mtr.297.1.S1_s_at	8.97	7.58	8.75	8.83	8.83	8.83	8.43	0.74	8.83	0.00	1.05	0.066	4.1E-01
Mtr.12974.1.S1_at	6.36	6.29	7.61	6.73	6.88	6.41	6.75	0.74	6.68	0.24	0.99	-0.017	8.7E-01
Mtr.33674.1.S1_at	3.94	3.04	4.52	3.74	4.30	3.65	3.83	0.74	3.89	0.35	1.02	0.023	9.0E-01
Mtr.44585.1.S1_at	7.69	6.30	7.45	7.68	8.15	7.91	7.15	0.74	7.92	0.24	1.11	0.147	1.6E-01
Mtr.30946.1.S1_at	5.89	4.79	6.21	5.75	6.36	5.59	5.63	0.74	5.90	0.41	1.05	0.068	6.1E-01
Mtr.11982.1.S1_at	7.83	7.26	8.73	7.86	8.26	7.66	7.94	0.74	7.93	0.31	1.00	-0.003	9.7E-01
Mtr.13869.1.S1_at	6.47	5.42	6.85	7.23	7.63	7.55	6.25	0.74	7.47	0.21	1.20	0.258	5.2E-02
Mtr.23311.1.S1_s_at	7.21	6.54	8.03	8.00	8.35	8.12	7.26	0.74	8.16	0.18	1.12	0.168	1.1E-01
Mtr.2173.1.S1_at	4.70	4.01	5.49	4.42	6.02	4.93	4.73	0.74	5.12	0.82	1.08	0.114	5.8E-01
Mtr.37518.1.S1_at	14.93	13.54	14.70	14.91	14.68	14.84	14.39	0.74	14.81	0.12	1.03	0.041	3.9E-01
Mtr.4420.1.S1_at	4.76	3.40	4.59	4.59	4.59	4.59	4.25	0.74	4.59	0.00	1.08	0.110	4.8E-01
Mtr.5330.1.S1_at	8.08	6.78	6.81	7.01	6.57	7.08	7.22	0.74	6.89	0.28	0.95	-0.068	5.1E-01
Mtr.5399.1.S1_at	2.99	4.27	2.98	2.96	2.96	2.98	3.41	0.74	2.97	0.01	0.87	-0.202	3.6E-01
Mtr.9319.1.S1_at	11.45	12.68	11.35	10.56	11.02	10.82	11.83	0.74	10.80	0.23	0.91	-0.131	8.3E-02
Mtr.46177.1.S1_at	4.31	4.27	5.57	4.71	4.63	4.62	4.72	0.74	4.65	0.05	0.99	-0.019	8.9E-01
Mtr.47429.1.S1_at	7.35	5.98	7.16	7.15	7.21	7.16	6.83	0.74	7.17	0.03	1.05	0.071	4.7E-01
Mtr.13676.1.S1_at	7.97	6.67	7.94	8.05	7.73	7.95	7.53	0.74	7.91	0.16	1.05	0.071	4.3E-01
Mtr.30112.1.S1_at	7.77	6.41	7.60	7.58	7.58	7.84	7.26	0.74	7.67	0.15	1.06	0.079	4.0E-01
Mtr.12506.1.S1_x_at	5.05	3.58	4.11	5.22	5.78	5.75	4.24	0.74	5.58	0.31	1.32	0.396	4.5E-02
Mtr.52250.1.S1_at	5.63	4.23	4.49	5.25	7.14	5.57	4.78	0.74	5.99	1.01	1.25	0.324	1.7E-01
Mtr.45042.1.S1_at	8.39	7.08	8.34	8.48	8.23	8.35	7.94	0.74	8.36	0.13	1.05	0.074	3.9E-01
Mtr.25451.1.S1_at	9.40	10.65	10.71	10.82	10.66	10.62	10.25	0.74	10.70	0.11	1.04	0.062	3.6E-01
Mtr.9487.1.S1_at	2.64	2.52	3.86	2.57	4.65	2.71	3.00	0.74	3.31	1.16	1.10	0.140	7.2E-01
Mtr.9679.1.S1_at	13.82	12.34	13.23	13.21	12.84	12.84	13.13	0.74	12.96	0.21	0.99	-0.019	7.2E-01
Mtr.31710.1.S1_at	8.76	9.37	10.24	8.89	8.98	9.02	9.46	0.74	8.96	0.07	0.95	-0.077	3.1E-01
Mtr.20477.1.S1_at	5.19	3.90	5.17	5.46	5.76	5.30	4.76	0.74	5.51	0.23	1.16	0.211	1.7E-01
Mtr.37186.1.S1_at	9.63	8.22	9.31	9.91	10.40	9.97	9.05	0.74	10.09	0.27	1.11	0.157	8.4E-02
Mtr.45380.1.S1_at	4.16	4.25	5.48	4.73	5.02	4.14	4.63	0.74	4.63	0.45	1.00	0.000	1.0E+00
Mtr.50655.1.S1_at	5.50	4.20	5.46	5.27	5.29	4.41	5.05	0.74	4.99	0.50	0.99	-0.018	9.1E-01
Mtr.37693.1.S1_at	9.10	7.86	9.18	9.06	9.09	9.36	8.71	0.74	9.17	0.16	1.05	0.074	3.5E-01

Mtr.21923.1.S1_at	8.68	7.46	8.79	8.88	9.06	9.16	8.31	0.74	9.03	0.14	1.09	0.120	1.7E-01
Mtr.14389.1.S1_at	2.92	4.18	2.88	2.87	2.76	2.86	3.32	0.74	2.83	0.06	0.85	-0.232	3.1E-01
Mtr.35045.1.S1_at	3.62	2.37	3.68	3.54	4.28	4.25	3.23	0.74	4.02	0.42	1.25	0.319	1.8E-01
Mtr.38022.1.S1_at	11.20	11.91	12.68	11.81	11.83	11.77	11.93	0.74	11.80	0.03	0.99	-0.016	7.8E-01
Mtr.45361.1.S1_at	8.28	6.99	8.26	8.35	8.15	8.53	7.84	0.74	8.34	0.19	1.06	0.089	3.2E-01
Mtr.33506.1.S1_at	5.65	4.62	6.06	5.64	5.64	5.77	5.44	0.74	5.68	0.08	1.04	0.062	6.1E-01
Mtr.11295.1.S1_at	3.80	4.19	5.23	3.81	4.87	3.75	4.40	0.74	4.14	0.63	0.94	-0.088	6.6E-01
Mtr.41172.1.S1_s_at	8.70	7.23	7.88	8.40	8.44	8.45	7.94	0.74	8.43	0.03	1.06	0.087	3.1E-01
Mtr.36140.1.S1_at	8.99	7.61	7.84	8.13	7.68	7.93	8.15	0.74	7.92	0.22	0.97	-0.042	6.3E-01
Mtr.15473.1.S1_s_at	5.67	5.20	6.65	5.29	5.71	6.20	5.84	0.74	5.74	0.45	0.98	-0.027	8.4E-01
Mtr.14917.1.S1_at	6.83	6.07	7.55	7.40	7.73	7.18	6.82	0.74	7.44	0.28	1.09	0.126	2.5E-01
Mtr.50414.1.S1_at	9.22	7.84	9.00	9.04	9.01	9.03	8.68	0.74	9.03	0.01	1.04	0.056	4.7E-01
Mtr.24241.1.S1_at	7.80	6.83	8.29	8.00	8.02	7.96	7.64	0.74	7.99	0.03	1.05	0.065	4.6E-01
Mtr.13646.1.S1_at	5.85	4.90	6.35	6.19	6.95	6.52	5.70	0.74	6.56	0.38	1.15	0.202	1.5E-01
Mtr.8977.1.S1_at	9.82	8.65	10.02	9.27	9.12	9.36	9.50	0.74	9.25	0.12	0.97	-0.038	6.0E-01
Mtr.30650.1.S1_at	6.91	5.45	6.36	5.93	7.01	6.59	6.24	0.74	6.51	0.55	1.04	0.061	6.4E-01
Mtr.5192.1.S1_s_at	5.46	4.51	4.00	4.55	4.56	4.97	4.66	0.74	4.69	0.24	1.01	0.011	9.4E-01
Mtr.29571.1.S1_at	8.29	6.88	7.98	7.92	7.88	8.01	7.71	0.74	7.94	0.07	1.03	0.041	6.3E-01
Mtr.13270.1.S1_at	9.59	8.11	8.80	9.18	8.87	9.41	8.83	0.74	9.15	0.27	1.04	0.052	5.2E-01
Mtr.1939.1.S1_at	4.99	3.77	5.10	5.68	5.01	5.52	4.62	0.74	5.40	0.35	1.17	0.225	1.7E-01
Mtr.22829.1.S1_at	2.93	4.20	2.91	2.90	2.87	2.87	3.35	0.74	2.88	0.02	0.86	-0.215	3.4E-01
Mtr.3094.1.S1_at	6.65	5.18	5.83	5.95	5.92	6.38	5.89	0.74	6.08	0.26	1.03	0.047	6.9E-01
Mtr.49301.1.S1_s_at	7.67	6.60	8.02	7.50	7.55	7.49	7.43	0.74	7.51	0.03	1.01	0.016	8.6E-01
Mtr.44237.1.S1_at	6.91	5.82	7.23	6.49	7.07	6.59	6.65	0.74	6.72	0.31	1.01	0.014	9.0E-01
Mtr.48951.1.S1_at	7.10	6.38	7.86	7.49	7.95	7.58	7.12	0.74	7.67	0.24	1.08	0.109	2.8E-01
Mtr.7056.1.S1_at	6.60	5.75	7.22	6.59	6.62	6.58	6.52	0.74	6.59	0.02	1.01	0.016	8.7E-01
Mtr.40124.1.S1_at	10.25	9.74	11.19	10.67	10.73	10.64	10.39	0.74	10.68	0.05	1.03	0.039	5.4E-01
Mtr.35214.1.S1_at	8.31	7.02	8.27	8.21	8.22	8.13	7.87	0.74	8.19	0.05	1.04	0.058	5.0E-01
Mtr.39460.1.S1_at	8.50	7.25	8.54	8.24	8.45	8.69	8.10	0.74	8.46	0.23	1.04	0.063	4.6E-01
Mtr.48934.1.S1_at	2.84	4.10	2.80	2.78	2.77	2.76	3.25	0.74	2.77	0.01	0.85	-0.229	3.3E-01
Mtr.35643.1.S1_at	8.35	7.04	8.28	8.28	8.26	8.14	7.89	0.74	8.23	0.07	1.04	0.060	4.8E-01
Mtr.11802.1.S1_at	10.91	9.44	10.18	10.06	10.07	9.94	10.17	0.74	10.02	0.07	0.99	-0.022	7.4E-01
Mtr.5941.1.S1_at	7.10	5.72	6.85	6.96	6.92	6.67	6.56	0.74	6.85	0.16	1.04	0.063	5.4E-01
Mtr.7342.1.S1_at	10.42	8.95	9.60	9.90	10.21	9.93	9.66	0.74	10.01	0.17	1.04	0.052	4.6E-01
Mtr.28412.1.S1_at	6.61	5.34	6.62	6.61	6.77	6.90	6.19	0.74	6.76	0.15	1.09	0.126	2.6E-01
Mtr.8475.1.S1_s_at	10.99	9.52	10.12	10.18	9.88	9.86	10.21	0.74	9.97	0.18	0.98	-0.034	6.2E-01
Mtr.16948.1.S1_s_at	7.94	6.88	8.31	8.45	8.42	8.16	7.71	0.74	8.35	0.16	1.08	0.115	2.2E-01
Mtr.43749.1.S1_at	8.52	7.29	8.62	7.93	8.48	7.97	8.14	0.74	8.13	0.30	1.00	-0.002	9.8E-01
Mtr.35041.1.S1_s_at	7.51	6.16	7.35	7.65	7.65	7.62	7.01	0.74	7.64	0.02	1.09	0.125	2.1E-01
Mtr.38262.1.S1_at	9.51	8.64	10.11	9.83	9.66	9.68	9.42	0.74	9.72	0.10	1.03	0.046	5.2E-01
Mtr.40301.1.S1_at	11.49	10.06	10.46	10.76	10.33	10.96	10.67	0.74	10.68	0.32	1.00	0.002	9.8E-01
Mtr.10822.1.S1_at	11.71	11.15	10.25	11.25	11.14	11.48	11.04	0.74	11.29	0.17	1.02	0.032	6.0E-01
Mtr.41787.1.S1_at	6.41	5.02	6.14	6.42	6.56	6.11	5.86	0.74	6.36	0.23	1.09	0.120	3.2E-01
Mtr.5158.1.S1_at	7.85	6.62	7.94	7.87	7.90	7.96	7.47	0.74	7.91	0.04	1.06	0.083	3.6E-01
Mtr.16918.1.S1_at	9.12	7.71	8.76	8.76	8.66	8.68	8.53	0.74	8.70	0.05	1.02	0.028	7.1E-01
Mtr.9082.1.S1_at	9.19	7.71	8.44	8.89	8.68	9.08	8.45	0.74	8.88	0.20	1.05	0.073	3.8E-01
Mtr.33917.1.S1_at	9.80	8.61	9.96	9.32	9.65	9.30	9.46	0.74	9.42	0.19	1.00	-0.005	9.4E-01
Mtr.35263.1.S1_at	5.29	5.64	6.71	5.90	6.37	5.43	5.88	0.74	5.90	0.47	1.00	0.005	9.7E-01
Mtr.13879.1.S1_at	8.49	7.06	8.06	8.15	8.23	8.24	7.87	0.74	8.21	0.05	1.04	0.060	4.7E-01
Mtr.30788.1.S1_at	2.28	3.56	2.28	2.28	2.28	2.27	2.71	0.74	2.28	0.00	0.84	-0.250	3.7E-01
Mtr.18554.1.S1_at	2.59	3.86	2.58	2.58	2.53	2.56	3.01	0.74	2.56	0.02	0.85	-0.236	3.5E-01
Mtr.44952.1.S1_at	7.21	6.61	8.08	7.08	7.33	7.03	7.30	0.74	7.15	0.16	0.98	-0.030	7.4E-01
Mtr.4374.1.S1_at	9.40	7.96	8.94	8.96	8.90	8.85	8.77	0.74	8.90	0.05	1.02	0.022	7.7E-01
Mtr.38771.1.S1_at	8.33	7.16	8.51	8.24	8.26	8.58	8.00	0.74	8.36	0.19	1.04	0.063	4.6E-01
Mtr.25964.1.S1_s_at	8.69	7.22	7.91	7.78	7.65	7.47	7.94	0.74	7.63	0.16	0.96	-0.057	5.2E-01
Mtr.20146.1.S1_at	11.14	9.66	10.40	10.77	10.72	10.64	10.40	0.74	10.71	0.06	1.03	0.042	5.1E-01
Mtr.29026.1.S1_at	11.60	12.99	12.71	12.73	13.10	12.77	12.43	0.74	12.87	0.20	1.03	0.049	3.8E-01
Mtr.40239.1.S1_at	11.01	12.43	11.36	11.91	12.29	12.14	11.60	0.74	12.12	0.19	1.04	0.063	3.1E-01
Mtr.12560.1.S1_at	8.24	9.60	9.40	9.45	9.64	9.28	9.08	0.74	9.45	0.18	1.04	0.058	4.4E-01
Mtr.44498.1.S1_at	3.50	2.27	3.58	3.21	3.21	3.21	3.12	0.74	3.21	0.00	1.03	0.042	8.4E-01
Mtr.33201.1.S1_at	7.82	6.45	7.59	7.38	7.45	7.51	7.29	0.74	7.45	0.07	1.02	0.031	7.3E-01
Mtr.46577.1.S1_s_at	6.85	5.39	6.03	6.24	6.55	6.88	6.09	0.74	6.56	0.32	1.08	0.106	3.7E-01
Mtr.18508.1.S1_at	6.34	5.08	5.05	5.53	5.00	5.08	5.49	0.74	5.20	0.29	0.95	-0.078	5.6E-01
Mtr.10301.1.S1_s_at	8.00	6.88	8.27	8.02	7.99	8.04	7.72	0.74	8.01	0.02	1.04	0.055	5.2E-01
Mtr.39105.1.S1_at	11.31	9.91	10.98	11.06	10.96	11.14	10.73	0.73	11.05	0.09	1.03	0.042	5.0E-01
Mtr.26329.1.S1_at	3.08	4.34	3.07	3.06	3.06	3.06	3.50	0.73	3.06	0.00	0.88	-0.191	3.6E-01
Mtr.37164.1.S1_at	5.44	4.21	4.13	4.57	5.20	5.13	4.59	0.73	4.97	0.35	1.08	0.113	4.7E-01
Mtr.28721.1.S1_at	5.50	4.34	5.69	4.74	4.77	4.72	5.18	0.73	4.74	0.02	0.92	-0.127	3.6E-01
Mtr.44417.1.S1_at	9.55	8.24	9.47	9.18	9.20	9.13	9.09	0.73	9.17	0.04	1.01	0.013	8.5E-01
Mtr.42201.1.S1_at	8.84	7.81	9.23	8.68	8.93	8.40	8.63	0.73	8.67	0.27	1.00	0.007	9.3E-01
Mtr.7597.1.S1_at	6.67	5.68	7.11	6.23	6.74	6.22	6.49	0.73	6.39	0.30	0.99	-0.021	8.5E-01
Mtr.10990.1.S1_at	8.74	7.29	8.20	8.56	8.53	8.39	8.07	0.73	8.49	0.09	1.05	0.073	3.8E-01
Mtr.45156.1.S1_at	6.73	5.74	7.17	7.10	7.75	7.32	6.55	0.73	7.39	0.33	1.13	0.175	1.4E-01
Mtr.28857.1.S1_s_at	7.69	6.85	8.31	7.75	7.88	7.68	7.61	0.73	7.77	0.10	1.02	0.029	7.4E-01
Mtr.7047.1.S1_at	6.74	7.96	6.64	6.64	6.86	6.63	7.11	0.73	6.71	0.13	0.94	-0.084	4.0E-01
Mtr.29568.1.S1_at	5.23	3.81	4.23	5.79	5.71	5.05	4.42	0.73	5.52	0.40	1.25	0.319	8.6E-02
Mtr.8064.1.S1_at	5.53	4.15	5.27	5.07	5.10	4.65	4.98	0.73	4.94	0.25	0.99	-0.013	9.3E-01
Mtr.35753.1.S1_at	7.06	5.70	6.86	6.94	7.06	6.80	6.54	0.73	6.93	0.13	1.06	0.084	4.2E-01
Mtr.40826.1.S1_at	5.19	3.76	4.20	4.35	3.88	4.18	4.38	0.73	4.14	0.24	0.94	-0.083	6.1E-01
Mtr.13804.1.S1_at	4.90	3.57	3.71	3.73	3.72	4.04	4.06	0.73	3.83	0.18	0.94	-0.085	6.2E-01
Mtr.23372.1.S1_s_at	8.11	6.70	7.75	7.89	7.78	7.65	7.52	0.73	7.77	0.12	1.03	0.047	5.9E-01
Mtr.3600.1.S1_at	8.72	7.34	8.46	8.66	8.68	8.61	8.17	0.73	8.65	0.04	1.06	0.082	3.2E-01
Mtr.22947.1.S1_x_at	4.78	3.73	3.36	4.41	4.97	3.73	3.96	0.73	4.37	0.62	1.10	0.143	5.0E-01
Mtr.12933.1.S1_at	9.85	8.73	10.11	9.78	9.85	9.68	9.56	0.73	9.77	0.09	1.02	0.031	6.5E-01
Mtr.49085.1.S1_at	5.91	5.20	6.67	6.68	7.05	7.00	5.93	0.73	6.91	0.20	1.17	0.221	8.9E-02
Mtr.42640.1.S1_s_at	8.75	7.29	8.17	8.45	8.39	8.38	8.07	0.73	8.41	0.04	1.04	0.059	4.7E-01
Mtr.20826.1.S1_at	7.49	6.28	7.60	7.25	7.25	7.35	7.12	0.73	7.28	0.06	1.02	0.032	7.3E-01
Mtr.40726.1.S1_at	7.50	6.16	7.34	7.33	7.52	6.83	7.00	0.73	7.23	0.36	1.03	0.046	6.6E-01
Mtr.30752.1.S1_at	4.34	3.23	4.62	4.66	5.13	4.81	4.07	0.73	4.87	0.24	1.20	0.259	1.5E-01
Mtr.37973.1.S1_at	9.70	8.24	8.95	8.94	9.04	9.12	8.96	0.73	9.03	0.09	1.01	0.011	8.8E-01
Mtr.2157.1.S1_s_at	6.99	5.56	6.57	6.92	6.63	7.03	6.37	0.73	6.86	0.21	1.08	0.106	3.3E-01
Mtr.35184.1.S1_at	8.12	6.66	7.37	7.81	7.90	7.67	7.38	0.73	7.79	0.11	1.06	0.078	3.9E-01
Mtr.47285.1.S1_at	5.88	4.67	5.99	6.10	6.05	6.18	5.51	0.73	6.11	0.06	1.11	0.148	2.3E-01
Mtr.37821.1.S1_at	9.14	7.86	9.12	8.91	9.06	8.85	8.71	0.73	8.94	0.11	1.03	0.038	6.1E-01
Mtr.49044.1.S1_at	4.42	3.00	4.03	5.01	4.82	4.70	3.82	0.73	4.84	0.16	1.27	0.344	7.6E-02
Mtr.21216.1.S1_at	7.49	6.17	7.37	8.15	8.22	8.40	7.01	0.73	8.26	0.13	1.18	0.236	4.4E-02
Mtr.47986.1.S1_at	10.88	10.59	11.98	11.93	12.27	11.88	11.15	0.73	12.03	0.21	1.08	0.109	1.2E-01
Mtr.36035.1.S1_at	5.30	4.31	5.74	4.43	4.54	3.77	5.12	0.73	4.25	0.42	0.83	-0.270	1.5E-01
Mtr.48557.1.S1_at	6.43	5.51	6.95	6.66	6.81	6.05	6.30	0.73	6.51	0.41	1.03	0.047	6.9E-01
Mtr.41362.1.S1_at	6.24	5.19	6.59	6.33	6.47	6.20	6.01	0.73	6.33	0.14	1.05	0.077	4.9E-01
Mtr.3001.1.S1_at	5.21	3.81	4.87	5.02	5.08	4.85	4.63	0.73	4.98	0.12	1.08	0.106	4.6E-01
Mtr.11468.1.S1_at	5.93	4.74	6.07	6.78	7.40	7.10	5.58	0.73	7.09	0.31	1.27	0.346	3.0E-02
Mtr.22570.1.S1_at	7.99	6.87	8.25	8.14	8.10	8.24	7.70	0.73	8.16	0.07	1.06	0.084	3.4E-01
Mtr.51636.1.S1_at	7.04	5.87	7.22	7.34	7.23	6.92	6.71	0.73	7.16	0.22	1.07	0.094	3.6E-01
Mtr.4684.1.S1_at	9.77	8.94	10.40	9.96	10.15	9.98	9.70	0.73	10.03	0.11	1.03	0.048	4.8E-01
Mtr.27613.1.S1_at	4.85	3.40	4.29	5.03	5.12	4.03	4.18	0.73	4.72	0.61	1.13	0.177	3.8E-01
Mtr.13964.1.S1_at	7.12	5.96	7.31	7.02	7.17	6.77	6.80	0.73	6.99	0.20	1.03	0.039	6.9E-01
Mtr.32078.1.S1_at	4.70	3.28	3.68	3.77	5.33	3.64	3.89	0.73	4.25	0.94	1.09	0.127	6.3E-01
Mtr.41982.1.S1_at	8.27	9.32	9.68	7.93	8.82	8.68	9.09	0.73	8.48	0.48	0.93	-0.100	2.9E-01
Mtr.32153.1.S1_s_at	8.22	7.01	8.33	8.89	8.46	8.48	7.85	0.73	8.61	0.24	1.10	0.133	1.6E-01
Mtr.16579.1.S1_x_at	4.14	3.01	2.78	2.92	2.92	2.91	3.31	0.73	2.91	0.00	0.88	-0.184	4.0E-01
Mtr.43659.1.S1_s_at	10.43	9.56	8.97	10.06	9.95	10.18	9.65	0.73	10.06	0.11	1.04	0.060	3.9E-01
Mtr.2618.1.S1_at	2.22	3.50	2.24	2.25	2.24	2.24	2.65	0.73	2.24	0.00	0.84	-0.244	3.8E-01
Mtr.42658.1.S1_at	6.12	5.07	6.47	7.17	7.06	7.78	5.89	0.73	7.34	0.39	1.25	0.318	3.9E-02
Mtr.21614.1.S1_at	7.82	7.32	8.76	7.98	7.90	7.58	7.97	0.73	7.82	0.21	0.98	-0.028	7.5E-01
Mtr.31405.1.S1_at	9.52	9.84	10.91	9.31	9.37	9.25	10.09	0.73	9.31	0.06	0.92	-0.116	1.4E-01
Mtr.44950.1.S1_at	6.18	5.07	6.44	5.96	5.61	5.95	5.90	0.73	5.84	0.20	0.99	-0.014	9.0E-01
Mtr.35336.1.S1_at	7.83	6.44	7.53	7.94	8.42	7.99	7.26	0.73	8.12	0.26	1.12	0.160	1.3E-01
Mtr.4006.1.S1_at	5.85	4.74	6.12	5.82	6.47	6.17	5.57	0.73	6.15	0.32	1.10	0.144	2.7E-01
Mtr.7338.1.S1_at	4.95	3.50	4.12	4.64	4.45	5.14	4.19	0.73	4.74	0.35	1.13	0.178	3.1E-01
Mtr.45533.1.S1_at	7.28	6.07	7.38	7.29	7.26	7.55	6.91	0.73	7.37	0.16	1.07	0.093	3.5E-01
Mtr.33918.1.S1_at	6.86	5.53	6.72	6.70	6.88	6.82	6.37	0.73	6.80	0.09	1.07	0.095	3.7E-01
Mtr.9948.1.S1_at	5.83	4.53	5.75	5.52	5.84	5.56	5.37	0.73	5.64	0.17	1.05	0.071	5.7E-01
Mtr.11007.1.S1_at	8.46	7.30	7.12	8.33	8.87	8.65	7.63	0.73	8.62	0.27	1.13	0.176	9.3E-02
Mtr.4912.1.S1_s_at	7.89	8.26	9.30	8.39	8.30	8.31	8.48	0.73	8.33	0.05	0.98	-0.026	7.4E-01
Mtr.32685.1.S1_at	6.03	4.75	5.99	6.59	6.25	6.35	5.59	0.73	6.40	0.17	1.14	0.195	1.4E-01
Mtr.33233.1.S1_at	8.89	7.77	9.14	9.04	8.77	9.14	8.60	0.73	8.98	0.19	1.04	0.062	4.3E-01
Mtr.43385.1.S1_at	10.02	8.84	10.17	10.03	9.92	9.88	9.67	0.73	9.95	0.08	1.03	0.040	5.6E-01
Mtr.33687.1.S1_at	8.95	7.83	9.20	9.08	9.24	8.79	8.66	0.73	9.04	0.23	1.04	0.061	4.5E-01
Mtr.27263.1.S1_at	6.49	5.28	6.59	6.48	6.79	6.31	6.12	0.73	6.53	0.24	1.07	0.093	4.1E-01
Mtr.38773.1.S1_at	8.21	6.75	7.57	7.68	7.85	7.98	7.51	0.73	7.84	0.15	1.04	0.061	4.9E-01

Mtr.44041.1.S1_s_at	7.95	6.72	8.01	7.84	7.90	8.10	7.56	0.73	7.94	0.13	1.05	0.072	4.2E-01
Mtr.25330.1.S1_at	11.46	10.19	10.22	10.59	10.84	10.77	10.62	0.73	10.73	0.13	1.01	0.015	8.1E-01
Mtr.51660.1.S1_at	7.23	5.89	7.05	8.14	8.12	8.27	6.72	0.73	8.18	0.08	1.22	0.282	2.7E-02
Mtr.46061.1.S1_s_at	4.60	3.42	3.27	4.05	4.27	4.02	3.76	0.73	4.11	0.14	1.09	0.128	4.6E-01
Mtr.5441.1.S1_at	5.68	4.22	5.00	5.38	5.39	5.47	4.97	0.73	5.41	0.05	1.09	0.124	3.5E-01
Mtr.51449.1.S1_at	5.05	3.61	4.48	4.04	4.33	3.45	4.38	0.73	3.94	0.45	0.90	-0.152	4.2E-01
Mtr.50504.1.S1_at	9.23	7.78	8.64	8.89	8.71	9.11	8.55	0.73	8.90	0.20	1.04	0.059	4.6E-01
Mtr.35149.1.S1_at	6.88	8.26	7.99	8.36	8.09	8.30	7.71	0.73	8.25	0.14	1.07	0.098	2.8E-01
Mtr.9258.1.S1_at	9.40	7.94	8.75	8.89	8.84	9.20	8.70	0.73	8.98	0.19	1.03	0.046	5.5E-01
Mtr.2045.1.S1_at	4.15	4.05	5.36	4.59	4.62	4.59	4.52	0.73	4.60	0.02	1.02	0.025	8.6E-01
Mtr.44793.1.S1_at	9.41	8.05	9.19	8.97	9.41	8.80	8.89	0.73	9.06	0.31	1.02	0.028	7.2E-01
Mtr.24070.1.S1_at	6.05	4.66	5.72	5.50	5.50	5.09	5.48	0.73	5.36	0.24	0.98	-0.030	8.1E-01
Mtr.26548.1.S1_at	8.82	7.41	8.41	8.57	8.41	8.40	8.21	0.73	8.46	0.10	1.03	0.042	5.9E-01
Mtr.21883.1.S1_at	2.59	3.83	2.54	2.53	2.53	2.53	2.98	0.73	2.53	0.00	0.85	-0.238	3.4E-01
Mtr.37189.1.S1_at	9.51	8.37	9.72	9.35	9.62	8.92	9.20	0.73	9.29	0.35	1.01	0.014	8.6E-01
Mtr.33595.1.S1_s_at	6.44	5.75	7.21	7.30	7.88	7.38	6.47	0.73	7.52	0.32	1.16	0.218	8.3E-02
Mtr.5766.1.S1_at	8.37	7.19	8.53	8.36	8.30	8.32	8.03	0.73	8.33	0.03	1.04	0.053	5.1E-01
Mtr.38758.1.S1_at	12.39	10.95	11.90	11.82	11.72	11.79	11.75	0.73	11.78	0.05	1.00	0.004	9.5E-01
Mtr.6030.1.S1_at	2.38	3.63	2.35	2.33	2.35	2.34	2.78	0.73	2.34	0.01	0.84	-0.253	3.5E-01
Mtr.10039.1.S1_at	9.11	8.04	9.43	8.64	8.66	8.49	8.86	0.73	8.60	0.09	0.97	-0.043	5.7E-01
Mtr.18211.1.S1_at	2.76	2.90	4.09	2.73	2.67	2.72	3.25	0.73	2.71	0.03	0.83	-0.264	2.7E-01
Mtr.1435.1.S1_at	6.22	6.03	7.38	7.05	7.35	6.40	6.54	0.73	6.93	0.48	1.06	0.083	4.8E-01
Mtr.42087.1.S1_at	6.27	5.46	6.92	6.57	6.94	6.00	6.22	0.73	6.50	0.47	1.05	0.065	6.0E-01
Mtr.7222.1.S1_at	8.67	7.23	8.14	8.29	8.41	7.91	8.02	0.73	8.20	0.27	1.02	0.033	7.0E-01
Mtr.13761.1.S1_at	6.14	4.80	4.98	5.01	5.25	5.45	5.31	0.73	5.23	0.22	0.99	-0.020	8.7E-01
Mtr.42634.1.S1_at	7.21	5.79	6.22	6.23	7.14	6.66	6.41	0.73	6.68	0.45	1.04	0.060	6.1E-01
Mtr.20619.1.S1_x_at	2.96	3.05	4.26	3.48	3.08	2.72	3.42	0.73	3.09	0.38	0.90	-0.149	5.2E-01
Mtr.6699.1.S1_at	7.25	6.12	7.47	7.34	7.34	7.10	6.95	0.73	7.26	0.14	1.05	0.064	5.0E-01
Mtr.40519.1.S1_s_at	6.56	5.18	6.28	6.05	6.82	6.24	6.01	0.73	6.37	0.40	1.06	0.085	4.9E-01
Mtr.50954.1.S1_s_at	11.42	9.97	10.71	11.00	11.06	11.25	10.70	0.73	11.10	0.13	1.04	0.053	4.0E-01
Mtr.10019.1.S1_at	8.29	6.94	8.07	7.99	7.98	8.11	7.77	0.73	8.02	0.07	1.03	0.047	5.7E-01
Mtr.13187.1.S1_at	6.28	5.13	6.48	6.26	6.65	6.34	5.96	0.73	6.42	0.21	1.08	0.105	3.6E-01
Mtr.32195.1.S1_at	5.11	3.80	3.91	3.91	3.91	3.98	4.27	0.73	3.93	0.04	0.92	-0.120	4.6E-01
Mtr.2096.1.S1_at	5.07	4.84	6.20	5.67	6.72	5.77	5.37	0.73	6.05	0.58	1.13	0.173	2.7E-01
Mtr.41642.1.S1_at	7.51	6.29	7.58	7.70	7.56	7.69	7.13	0.73	7.65	0.08	1.07	0.102	2.8E-01
Mtr.37171.1.S1_at	9.23	7.78	8.56	8.94	9.20	8.99	8.53	0.73	9.04	0.14	1.06	0.085	2.9E-01
Mtr.50430.1.S1_at	10.93	11.74	10.29	8.90	9.19	9.05	10.98	0.73	9.05	0.14	0.82	-0.280	1.1E-02
Mtr.35507.1.S1_at	9.40	8.33	9.72	9.40	9.35	9.16	9.15	0.73	9.30	0.13	1.02	0.024	7.4E-01
Mtr.32634.1.S1_s_at	8.14	6.91	8.18	8.03	7.72	8.03	7.74	0.73	7.93	0.18	1.02	0.033	7.0E-01
Mtr.44894.1.S1_at	10.25	8.84	9.86	10.05	10.04	10.15	9.65	0.73	10.08	0.06	1.04	0.063	3.7E-01
Mtr.27721.1.S1_at	3.43	2.99	4.41	2.78	2.73	2.74	3.61	0.73	2.75	0.03	0.76	-0.392	1.1E-01
Mtr.5707.1.S1_at	7.00	5.56	6.43	7.53	7.47	7.37	6.33	0.73	7.46	0.08	1.18	0.237	5.5E-02
Mtr.15500.1.S1_at	3.01	4.24	2.96	2.94	2.86	2.94	3.41	0.73	2.92	0.04	0.86	-0.225	3.1E-01
Mtr.30325.1.S1_at	8.25	6.86	7.94	7.28	7.38	7.51	7.68	0.73	7.39	0.12	0.96	-0.057	5.2E-01
Mtr.45821.1.S1_at	6.60	5.70	7.13	6.63	6.53	6.42	6.48	0.73	6.53	0.10	1.01	0.011	9.2E-01
Mtr.44816.1.S1_at	6.91	5.46	6.11	6.48	6.76	6.37	6.16	0.73	6.54	0.20	1.06	0.086	4.4E-01
Mtr.46021.1.S1_at	2.74	3.05	4.12	5.05	5.24	3.94	3.30	0.73	4.75	0.71	1.44	0.522	6.9E-02
Mtr.38084.1.S1_at	10.26	8.83	9.73	9.49	9.53	9.67	9.61	0.73	9.56	0.09	1.00	-0.007	9.2E-01
Mtr.43832.1.S1_at	9.90	9.06	8.45	9.43	9.38	9.67	9.14	0.73	9.49	0.16	1.04	0.055	4.5E-01
Mtr.27857.1.S1_at	10.62	9.22	10.24	10.89	10.86	10.83	10.03	0.73	10.86	0.03	1.08	0.115	1.2E-01
Mtr.34128.1.S1_at	7.44	6.01	6.93	7.15	7.37	7.17	6.79	0.73	7.23	0.12	1.06	0.090	3.6E-01
Mtr.9532.1.S1_at	7.68	6.93	6.23	7.15	6.96	6.84	6.95	0.73	6.98	0.15	1.01	0.008	9.3E-01
Mtr.49934.1.S1_x_at	4.93	3.61	4.80	4.51	5.09	4.97	4.45	0.73	4.86	0.31	1.09	0.127	4.2E-01
Mtr.22451.1.S1_at	4.02	5.27	4.01	4.01	3.86	4.20	4.43	0.73	4.02	0.17	0.91	-0.140	3.9E-01
Mtr.32868.1.S1_s_at	8.08	6.81	8.06	7.48	7.74	7.76	7.65	0.73	7.66	0.16	1.00	0.001	9.9E-01
Mtr.27388.1.S1_s_at	7.88	6.49	7.55	7.52	6.79	6.71	7.31	0.73	7.01	0.45	0.96	-0.061	5.7E-01
Mtr.32472.1.S1_at	5.97	4.52	5.32	5.47	6.38	6.36	5.27	0.73	6.07	0.52	1.15	0.204	2.0E-01
Mtr.30492.1.S1_at	7.11	5.84	7.09	5.93	7.16	6.69	6.68	0.72	6.59	0.62	0.99	-0.019	8.8E-01
Mtr.10452.1.S1_at	9.83	8.40	9.30	8.56	6.77	8.43	9.17	0.72	7.92	1.00	0.86	-0.212	1.5E-01
Mtr.15535.1.S1_at	8.94	7.82	9.18	8.80	8.82	8.78	8.65	0.72	8.80	0.02	1.02	0.026	7.3E-01
Mtr.36933.1.S1_at	8.11	6.89	8.18	7.86	8.39	8.06	7.73	0.72	8.10	0.26	1.05	0.068	4.5E-01
Mtr.42763.1.S1_at	8.37	7.22	8.56	8.42	8.43	8.50	8.05	0.72	8.45	0.04	1.05	0.070	4.0E-01
Mtr.27556.1.S1_at	6.69	5.90	7.35	6.67	6.76	6.47	6.65	0.72	6.64	0.15	1.00	-0.002	9.8E-01
Mtr.5472.1.S1_at	7.98	6.75	8.03	8.05	7.95	7.89	7.59	0.72	7.96	0.08	1.05	0.070	4.2E-01
Mtr.38543.1.S1_at	8.85	7.72	9.07	9.04	8.95	8.83	8.54	0.72	8.94	0.11	1.05	0.065	4.0E-01
Mtr.12359.1.S1_at	10.70	9.44	10.70	10.92	10.83	10.97	10.28	0.72	10.90	0.07	1.06	0.085	2.1E-01
Mtr.38080.1.S1_at	11.37	10.49	9.93	11.18	10.93	11.39	10.60	0.72	11.17	0.23	1.05	0.075	2.6E-01
Mtr.39810.1.S1_at	5.35	3.94	4.94	4.97	5.24	5.02	4.75	0.72	5.08	0.14	1.07	0.097	4.8E-01
Mtr.12175.1.S1_at	6.35	4.92	5.87	5.44	5.91	6.09	5.71	0.72	5.81	0.34	1.02	0.024	8.4E-01
Mtr.34141.1.S1_at	7.83	6.86	8.28	7.90	7.69	7.64	7.65	0.72	7.74	0.14	1.01	0.017	8.5E-01
Mtr.41916.1.S1_at	9.87	8.44	8.98	9.33	9.16	9.45	9.10	0.72	9.32	0.15	1.02	0.034	6.3E-01
Mtr.10242.1.S1_at	8.61	7.24	8.34	8.42	8.45	8.34	8.06	0.72	8.40	0.06	1.04	0.060	4.6E-01
Mtr.11786.1.S1_at	7.19	7.89	6.44	7.41	7.09	7.67	7.17	0.72	7.39	0.29	1.03	0.043	6.6E-01
Mtr.45173.1.S1_at	2.94	2.55	3.95	2.46	3.05	2.92	3.14	0.72	2.81	0.31	0.89	-0.162	5.0E-01
Mtr.40502.1.S1_at	5.46	5.45	6.71	8.98	9.60	9.05	5.87	0.72	9.21	0.34	1.57	0.649	1.9E-03
Mtr.37018.1.S1_at	6.60	5.15	5.82	6.41	6.69	6.88	5.86	0.72	6.66	0.24	1.14	0.186	1.4E-01
Mtr.47857.1.S1_at	9.46	9.07	8.06	7.86	8.30	8.05	8.86	0.72	8.07	0.22	0.91	-0.135	1.4E-01
Mtr.49780.1.S1_at	4.64	5.95	5.83	5.81	6.01	5.82	5.47	0.72	5.88	0.11	1.07	0.103	3.9E-01
Mtr.32940.1.S1_at	7.71	8.53	7.08	7.44	6.96	7.34	7.77	0.72	7.24	0.25	0.93	-0.102	3.0E-01
Mtr.44222.1.S1_at	9.21	8.01	9.32	9.04	9.38	9.09	8.85	0.72	9.17	0.19	1.04	0.051	5.0E-01
Mtr.44856.1.S1_at	11.01	10.43	9.57	9.61	9.45	9.66	10.34	0.72	9.58	0.11	0.93	-0.110	1.5E-01
Mtr.2620.1.S1_at	7.43	6.04	7.10	7.56	7.58	7.55	6.86	0.72	7.56	0.01	1.10	0.141	1.7E-01
Mtr.27292.1.S1_s_at	9.68	8.25	9.15	9.25	9.22	9.32	9.02	0.72	9.27	0.05	1.03	0.038	5.9E-01
Mtr.32016.1.S1_at	4.59	3.29	4.48	4.94	4.76	5.59	4.12	0.72	5.10	0.44	1.24	0.307	1.2E-01
Mtr.5423.1.S1_s_at	9.20	7.87	8.05	8.40	8.47	8.44	8.37	0.72	8.44	0.03	1.01	0.011	8.8E-01
Mtr.30180.1.S1_at	3.62	2.31	3.49	2.58	2.65	2.21	3.14	0.72	2.48	0.23	0.79	-0.339	2.1E-01
Mtr.32230.1.S1_at	7.69	6.27	6.72	7.13	7.09	7.16	6.89	0.72	7.13	0.04	1.03	0.048	6.0E-01
Mtr.42752.1.S1_at	7.47	6.33	7.67	7.20	7.58	6.92	7.15	0.72	7.23	0.34	1.01	0.016	8.7E-01
Mtr.48952.1.S1_at	9.93	11.29	10.19	11.06	11.22	11.08	10.47	0.72	11.12	0.08	1.06	0.087	2.0E-01
Mtr.2502.1.S1_at	3.27	1.89	2.22	2.21	3.29	2.21	2.46	0.72	2.57	0.62	1.05	0.066	8.4E-01
Mtr.23604.1.S1_at	3.17	4.39	3.11	3.19	3.08	3.11	3.56	0.72	3.13	0.06	0.88	-0.187	3.6E-01
Mtr.33320.1.S1_at	8.44	7.10	8.24	8.03	8.01	8.28	7.93	0.72	8.10	0.15	1.02	0.032	7.0E-01
Mtr.41888.1.S1_at	5.51	4.29	5.58	6.25	6.21	5.08	5.13	0.72	5.85	0.67	1.14	0.189	2.7E-01
Mtr.39074.1.S1_at	7.42	6.39	7.79	7.64	7.75	7.33	7.20	0.72	7.57	0.22	1.05	0.073	4.4E-01
Mtr.17468.1.S1_at	6.22	5.49	6.94	6.93	6.75	6.40	6.22	0.72	6.69	0.27	1.08	0.107	3.4E-01
Mtr.10451.1.S1_at	13.79	12.41	12.72	12.82	12.40	13.13	12.97	0.72	12.78	0.37	0.99	-0.021	7.1E-01
Mtr.26842.1.S1_at	8.11	7.10	8.50	8.42	8.21	8.25	7.91	0.72	8.29	0.11	1.05	0.069	4.1E-01
Mtr.5582.1.S1_s_at	7.08	5.81	7.05	7.61	7.15	7.50	6.64	0.72	7.42	0.24	1.12	0.159	1.5E-01
Mtr.46957.1.S1_at	7.85	6.80	8.18	8.40	8.05	7.90	7.61	0.72	8.11	0.26	1.07	0.093	3.2E-01
Mtr.32565.1.S1_at	10.88	10.54	11.93	11.44	11.50	11.46	11.12	0.72	11.46	0.03	1.03	0.044	4.5E-01
Mtr.45897.1.S1_at	9.91	8.48	9.34	9.61	9.68	9.78	9.24	0.72	9.69	0.09	1.05	0.068	3.5E-01
Mtr.45271.1.S1_at	9.62	10.68	10.99	10.40	11.32	10.13	10.43	0.72	10.61	0.63	1.02	0.025	7.6E-01
Mtr.33743.1.S1_at	5.53	4.39	5.73	5.55	5.66	6.03	5.21	0.72	5.75	0.25	1.10	0.141	2.9E-01
Mtr.41887.1.S1_at	8.00	6.75	8.00	8.23	7.84	8.16	7.58	0.72	8.07	0.21	1.06	0.091	3.2E-01
Mtr.25198.1.S1_at	2.74	3.84	2.49	2.67	2.66	2.94	3.02	0.72	2.76	0.16	0.91	-0.132	5.7E-01
Mtr.18357.1.S1_s_at	3.13	4.30	2.99	3.08	3.82	3.59	3.47	0.72	3.50	0.38	1.01	0.009	9.7E-01
Mtr.42546.1.S1_at	7.25	6.15	7.51	7.57	7.40	7.50	6.97	0.72	7.49	0.08	1.07	0.104	2.8E-01
Mtr.18245.1.S1_at	2.93	2.23	3.67	3.12	3.74	3.03	2.94	0.72	3.30	0.39	1.12	0.164	5.0E-01
Mtr.28323.1.S1_at	7.67	6.50	7.82	7.53	7.85	7.42	7.33	0.72	7.60	0.23	1.04	0.053	5.7E-01
Mtr.2598.1.S1_at	7.44	6.16	7.36	7.50	7.38	7.77	6.99	0.72	7.55	0.20	1.08	0.111	2.7E-01
Mtr.7124.1.S1_at	5.68	4.40	5.60	5.54	5.60	5.54	5.23	0.72	5.56	0.03	1.06	0.089	4.7E-01
Mtr.17554.1.S1_at	5.06	4.00	5.38	4.93	4.90	5.03	4.81	0.72	4.95	0.07	1.03	0.042	7.5E-01
Mtr.35042.1.S1_at	9.14	8.27	9.70	9.41	9.52	9.37	9.04	0.72	9.43	0.07	1.04	0.062	4.0E-01
Mtr.16120.1.S1_x_at	5.54	4.11	4.96	6.05	6.58	6.15	4.87	0.72	6.26	0.28	1.29	0.362	3.6E-02
Mtr.3830.1.S1_at	8.93	7.88	9.26	8.85	9.17	8.92	8.69	0.72	8.98	0.17	1.03	0.047	5.4E-01
Mtr.44167.1.S1_at	3.08	3.16	4.36	3.04	4.29	2.92	3.53	0.72	3.42	0.76	0.97	-0.047	8.6E-01
Mtr.1094.1.S1_at	9.28	9.47	10.61	9.70	10.05	9.24	9.79	0.72	9.67	0.41	0.99	-0.018	8.1E-01
Mtr.23592.1.S1_at	2.52	3.76	2.51	2.51	2.64	2.50	2.93	0.72	2.55	0.08	0.87	-0.201	4.1E-01
Mtr.14076.1.S1_x_at	3.92	3.46	4.87	4.20	3.95	3.93	4.08	0.72	4.02	0.15	0.99	-0.020	9.0E-01
Mtr.17920.1.S1_s_at	7.92	6.55	7.61	7.63	7.56	7.63	7.36	0.72	7.61	0.04	1.03	0.048	5.8E-01
Mtr.31335.1.S1_s_at	7.68	6.82	8.25	8.15	8.20	8.15	7.59	0.72	8.17	0.03	1.08	0.106	2.3E-01
Mtr.33832.1.S1_at	5.03	3.78	5.02	4.21	4.05	4.41	4.61	0.72	4.22	0.18	0.92	-0.128	4.1E-01
Mtr.28401.1.S1_at	4.25	2.92	4.05	4.10	4.55	4.05	3.74	0.72	4.23	0.27	1.13	0.179	3.3E-01
Mtr.7741.1.S1_at	7.83	8.82	9.24	8.74	8.80	8.44	8.63	0.72	8.66	0.19	1.00	0.005	9.5E-01
Mtr.1569.1.S1_at	5.65	4.35	5.54	5.56	5.53	5.51	5.18	0.72	5.53	0.03	1.07	0.096	4.4E-01
Mtr.19277.1.S1_at	7.48	6.20	7.42	7.82	7.57	7.64	7.03	0.72	7.68	0.13	1.09	0.126	2.0E-01
Mtr.50000.1.S1_at	7.83	6.74	8.10	8.30	8.43	8.08	7.56	0.72	8.27	0.18	1.09	0.129	1.7E-01
Mtr.42251.1.S1_at	6.77	5.92	7.35	7.04	7.16	6.95	6.68	0.72	7.05	0.11	1.06	0.078	4.3E-01
Mtr.29434.1.S1_at	7.28	6.18	7.53	7.28	7.39	7.29	7.00	0.72	7.32	0.06	1.05	0.065	4.8E-01
Mtr.42539.1.S1_at	6.89	5.99	7.41	7.19	7.12	6.98	6.76	0.72	7.10	0.11	1.05	0.071	4.6E-01
Mtr.41129.1.S1_s_at	8.91	7.50	8.45	8.61	7.94	8.55	8.29	0.72	8.37	0.37	1.01	0.014	8.7E-01

Mtr.7367.1.S1_s_at	8.71	7.80	9.22	9.24	10.10	9.30	8.58	0.72	9.55	0.48	1.11	0.154	1.2E-01
Mtr.35738.1.S1_at	10.27	11.37	10.02	10.35	11.27	10.79	10.55	0.72	10.80	0.46	1.02	0.034	6.4E-01
Mtr.40120.1.S1_at	13.90	12.65	12.67	13.37	13.87	13.76	13.07	0.72	13.66	0.26	1.05	0.064	2.5E-01
Mtr.24522.1.S1_s_at	4.94	6.37	5.79	5.87	5.90	5.88	5.70	0.72	5.89	0.01	1.03	0.046	6.8E-01
Mtr.9909.1.S1_at	10.44	9.14	10.32	10.37	10.35	10.27	9.96	0.72	10.33	0.05	1.04	0.052	4.3E-01
Mtr.28009.1.S1_s_at	5.50	5.46	6.73	5.90	5.95	5.87	5.90	0.72	5.91	0.04	1.00	0.002	9.9E-01
Mtr.39445.1.S1_at	10.23	9.06	10.37	10.53	10.51	10.46	9.89	0.72	10.50	0.04	1.06	0.087	2.1E-01
Mtr.33742.1.S1_at	6.64	5.27	6.34	6.22	6.44	6.37	6.08	0.72	6.34	0.11	1.04	0.060	5.7E-01
Mtr.7481.1.S1_at	13.60	12.48	12.26	13.17	13.66	13.36	12.78	0.72	13.40	0.25	1.05	0.068	2.3E-01
Mtr.20247.1.S1_at	3.87	3.02	4.45	4.25	4.48	4.25	3.78	0.72	4.32	0.13	1.14	0.194	2.7E-01
Mtr.27713.1.S1_at	5.90	5.63	6.99	7.48	6.75	7.87	6.17	0.72	7.36	0.57	1.19	0.254	8.8E-02
Mtr.45216.1.S1_at	8.42	7.19	8.45	8.11	8.50	8.26	8.02	0.72	8.29	0.20	1.03	0.048	5.6E-01
Mtr.33404.1.S1_s_at	4.46	3.12	4.24	4.56	4.39	4.50	3.94	0.72	4.48	0.09	1.14	0.186	2.6E-01
Mtr.41800.1.S1_at	6.92	5.84	7.19	7.23	7.26	7.28	6.65	0.72	7.26	0.03	1.09	0.126	2.2E-01
Mtr.46789.1.S1_at	3.74	2.35	3.34	3.07	2.81	3.06	3.14	0.72	2.98	0.15	0.95	-0.075	7.2E-01
Mtr.39837.1.S1_at	8.02	6.75	7.97	8.47	8.52	8.31	7.58	0.72	8.43	0.11	1.11	0.153	1.1E-01
Mtr.29133.1.S1_at	9.79	8.60	9.90	9.66	9.80	9.77	9.43	0.72	9.74	0.07	1.03	0.047	5.0E-01
Mtr.52006.1.S1_at	6.69	6.72	7.95	7.10	7.83	7.78	7.12	0.72	7.57	0.41	1.06	0.088	4.0E-01
Mtr.41161.1.S1_at	8.20	6.80	7.25	7.64	7.66	7.52	7.42	0.72	7.61	0.08	1.03	0.037	6.7E-01
Mtr.22174.1.S1_at	4.03	4.99	3.58	3.46	3.04	3.56	4.20	0.72	3.36	0.28	0.80	-0.323	1.3E-01
Mtr.36531.1.S1_at	2.92	4.16	2.91	2.91	2.91	2.90	3.33	0.72	2.91	0.00	0.87	-0.197	3.6E-01
Mtr.2364.1.S1_at	4.79	3.41	4.43	4.79	5.52	4.79	4.21	0.72	5.03	0.42	1.20	0.257	1.6E-01
Mtr.42526.1.S1_at	6.31	4.89	5.76	7.07	7.07	6.87	5.65	0.72	7.00	0.12	1.24	0.310	3.2E-02
Mtr.44918.1.S1_at	5.20	3.78	4.63	5.33	4.90	5.29	4.54	0.72	5.17	0.23	1.14	0.189	2.2E-01
Mtr.3281.1.S1_at	4.32	3.72	5.14	4.58	4.86	4.57	4.39	0.72	4.67	0.17	1.06	0.088	5.5E-01
Mtr.35003.1.S1_at	7.09	5.78	6.95	7.11	7.21	7.50	6.61	0.72	7.27	0.20	1.10	0.138	2.0E-01
Mtr.28577.1.S1_at	9.19	7.83	8.91	9.09	9.11	8.92	8.64	0.72	9.04	0.10	1.05	0.065	4.0E-01
Mtr.31838.1.S1_at	9.71	8.28	8.90	8.91	8.78	8.79	8.96	0.72	8.83	0.07	0.98	-0.022	7.6E-01
Mtr.5148.1.S1_at	4.80	3.44	4.50	3.71	4.51	3.98	4.25	0.72	4.07	0.41	0.96	-0.062	7.3E-01
Mtr.7464.1.S1_at	8.62	7.34	8.54	8.68	8.53	8.53	8.17	0.72	8.58	0.09	1.05	0.071	3.8E-01
Mtr.41492.1.S1_at	9.10	7.77	8.89	8.77	8.83	8.90	8.59	0.72	8.83	0.07	1.03	0.041	5.9E-01
Mtr.35498.1.S1_at	3.48	3.12	4.50	3.20	3.66	3.47	3.70	0.72	3.44	0.23	0.93	-0.105	5.8E-01
Mtr.30241.1.S1_at	6.29	7.02	7.72	5.73	6.23	6.38	7.01	0.72	6.11	0.34	0.87	-0.197	1.2E-01
Mtr.42453.1.S1_at	4.04	2.84	2.76	5.90	6.94	6.08	3.21	0.72	6.31	0.55	1.96	0.973	4.1E-03
Mtr.17441.1.S1_at	5.88	4.64	4.64	5.75	5.75	6.14	5.05	0.72	5.88	0.22	1.16	0.219	1.3E-01
Mtr.5493.1.S1_at	3.97	2.55	3.16	3.14	3.57	3.12	3.23	0.72	3.28	0.25	1.02	0.022	9.2E-01
Mtr.25672.1.S1_a_at	5.81	4.38	5.19	7.07	7.45	6.70	5.13	0.72	7.07	0.38	1.38	0.464	1.4E-02
Mtr.50015.1.S1_s_at	5.64	4.98	6.41	5.52	6.53	5.96	5.68	0.72	6.01	0.51	1.06	0.081	5.5E-01
Mtr.3160.1.S1_at	7.92	6.94	8.34	8.12	8.04	7.89	7.73	0.72	8.02	0.12	1.04	0.053	5.3E-01
Mtr.35156.1.S1_at	7.82	6.49	7.62	7.72	7.55	7.52	7.31	0.72	7.60	0.11	1.04	0.056	5.3E-01
Mtr.50412.1.S1_at	4.46	3.22	4.47	4.02	4.40	4.48	4.05	0.72	4.30	0.25	1.06	0.086	6.0E-01
Mtr.1040.1.S1_at	9.27	8.05	9.31	9.11	9.11	9.11	8.88	0.72	9.11	0.00	1.03	0.037	6.1E-01
Mtr.26065.1.S1_s_at	9.24	8.03	9.30	9.08	9.18	8.97	8.86	0.72	9.08	0.11	1.02	0.035	6.3E-01
Mtr.50674.1.S1_at	8.77	7.35	7.94	8.09	7.82	8.21	8.02	0.72	8.04	0.20	1.00	0.003	9.7E-01
Mtr.19417.1.S1_at	4.38	3.57	4.99	4.96	4.27	4.58	4.31	0.72	4.60	0.34	1.07	0.094	5.6E-01
Mtr.10556.1.S1_at	12.47	11.05	11.91	11.78	11.69	11.84	11.81	0.72	11.77	0.08	1.00	-0.005	9.3E-01
Mtr.21098.1.S1_at	7.56	8.54	8.96	7.46	7.89	7.49	8.35	0.72	7.61	0.24	0.91	-0.134	1.6E-01
Mtr.15051.1.S1_s_at	7.32	6.73	8.16	7.81	7.60	7.42	7.40	0.71	7.61	0.19	1.03	0.040	6.5E-01
Mtr.27941.1.S1_at	7.43	6.22	7.49	6.54	6.55	6.54	7.05	0.71	6.54	0.00	0.93	-0.107	2.9E-01
Mtr.26731.1.S1_s_at	3.11	4.22	2.88	3.08	2.84	2.83	3.40	0.71	2.92	0.14	0.86	-0.223	3.1E-01
Mtr.21360.1.S1_at	6.53	6.77	7.87	7.25	7.48	6.98	7.06	0.71	7.24	0.25	1.03	0.036	7.0E-01
Mtr.15655.1.S1_at	4.74	3.49	3.52	3.65	3.32	3.17	3.92	0.71	3.38	0.24	0.86	-0.213	2.9E-01
Mtr.2870.1.S1_at	8.68	7.69	9.08	8.81	9.01	8.71	8.48	0.71	8.84	0.16	1.04	0.060	4.4E-01
Mtr.12512.1.S1_at	6.68	5.43	5.45	5.44	6.38	5.98	5.85	0.71	5.94	0.47	1.01	0.020	8.8E-01
Mtr.22577.1.S1_at	6.26	4.89	5.22	5.22	4.89	5.55	5.46	0.71	5.22	0.33	0.96	-0.065	6.3E-01
Mtr.34147.1.S1_at	7.65	6.58	7.93	7.83	8.36	8.13	7.39	0.71	8.10	0.27	1.10	0.133	1.8E-01
Mtr.44274.1.S1_at	8.66	7.38	8.57	8.45	8.55	8.13	8.20	0.71	8.37	0.22	1.02	0.030	7.1E-01
Mtr.15612.1.S1_at	2.37	3.60	2.36	2.35	2.34	2.35	2.78	0.71	2.35	0.00	0.84	-0.243	3.6E-01
Mtr.12396.1.S1_at	9.43	8.08	9.15	10.15	11.04	10.72	8.88	0.71	10.63	0.45	1.20	0.259	2.3E-02
Mtr.40009.1.S1_at	4.83	5.93	6.16	5.56	5.82	5.93	5.64	0.71	5.77	0.19	1.02	0.033	7.8E-01
Mtr.27177.1.S1_at	8.26	6.85	7.39	7.53	7.08	7.50	7.50	0.71	7.37	0.25	0.98	-0.025	7.8E-01
Mtr.36944.1.S1_at	11.99	13.32	12.21	12.27	12.47	12.26	12.51	0.71	12.34	0.12	0.99	-0.020	7.0E-01
Mtr.25865.1.S1_at	3.20	4.41	3.15	3.05	3.14	3.13	3.59	0.71	3.11	0.05	0.87	-0.208	3.1E-01
Mtr.32914.1.S1_at	4.84	3.42	4.01	4.07	4.80	4.13	4.09	0.71	4.33	0.41	1.06	0.083	6.4E-01
Mtr.19521.1.S1_at	5.28	4.16	5.49	5.73	5.80	4.40	4.98	0.71	5.31	0.79	1.07	0.093	6.2E-01
Mtr.9376.1.S1_at	9.39	8.31	9.66	9.41	9.78	9.33	9.12	0.71	9.51	0.24	1.04	0.060	4.2E-01
Mtr.33656.1.S1_at	8.40	6.98	7.77	7.93	7.66	8.11	7.72	0.71	7.90	0.23	1.02	0.034	6.9E-01
Mtr.42277.1.S1_at	7.32	6.28	7.65	7.66	8.07	7.55	7.08	0.71	7.76	0.28	1.10	0.132	2.0E-01
Mtr.10327.1.S1_at	8.87	7.64	8.88	8.94	9.12	9.00	8.47	0.71	9.02	0.09	1.07	0.091	2.5E-01
Mtr.32404.1.S1_at	11.20	9.90	11.07	11.58	11.48	11.57	10.72	0.71	11.55	0.05	1.08	0.106	1.2E-01
Mtr.37642.1.S1_at	7.16	5.73	6.42	6.70	6.48	6.75	6.44	0.71	6.64	0.14	1.03	0.046	6.5E-01
Mtr.17798.1.S1_at	9.16	7.74	8.45	8.95	8.58	8.76	8.45	0.71	8.76	0.19	1.04	0.053	5.0E-01
Mtr.51319.1.S1_at	3.05	4.25	2.98	3.01	2.99	3.20	3.42	0.71	3.06	0.12	0.90	-0.160	4.4E-01
Mtr.3900.1.S1_at	8.80	7.90	9.31	8.63	9.25	8.56	8.67	0.71	8.81	0.38	1.02	0.023	7.8E-01
Mtr.14956.1.S1_at	8.07	7.09	8.47	8.12	8.61	8.26	7.88	0.71	8.33	0.25	1.06	0.081	3.6E-01
Mtr.38900.1.S1_at	9.77	8.35	8.99	9.45	9.34	9.57	9.04	0.71	9.45	0.12	1.05	0.065	3.8E-01
Mtr.23014.1.S1_at	2.65	3.88	2.64	2.63	2.64	2.62	3.06	0.71	2.63	0.01	0.86	-0.218	3.6E-01
Mtr.42363.1.S1_at	7.71	6.31	7.25	7.43	7.52	7.56	7.09	0.71	7.50	0.07	1.06	0.082	3.7E-01
Mtr.30452.1.S1_at	2.84	2.60	3.94	4.38	3.93	4.10	3.13	0.71	4.14	0.23	1.32	0.404	7.9E-02
Mtr.34604.1.S1_at	9.10	7.86	9.08	11.42	11.22	11.26	8.68	0.71	11.30	0.11	1.30	0.380	3.2E-03
Mtr.40274.1.S1_x_at	7.13	6.80	8.17	7.55	7.91	7.36	7.37	0.71	7.61	0.28	1.03	0.046	6.2E-01
Mtr.41850.1.S1_at	8.60	7.36	8.59	8.50	8.47	8.43	8.18	0.71	8.47	0.04	1.03	0.049	5.3E-01
Mtr.41538.1.S1_at	12.41	11.05	11.36	11.53	11.24	11.75	11.61	0.71	11.50	0.25	0.99	-0.013	8.3E-01
Mtr.32118.1.S1_at	9.28	10.64	9.60	9.24	9.30	9.34	9.84	0.71	9.29	0.05	0.94	-0.083	2.5E-01
Mtr.28017.1.S1_s_at	7.37	6.38	7.76	8.55	8.75	8.78	7.17	0.71	8.69	0.13	1.21	0.278	2.2E-02
Mtr.40777.1.S1_at	11.29	12.71	12.11	11.14	10.80	11.10	12.04	0.71	11.01	0.18	0.92	-0.128	7.4E-02
Mtr.47542.1.S1_at	7.30	5.93	6.94	7.39	7.88	7.89	6.72	0.71	7.72	0.29	1.15	0.200	8.7E-02
Mtr.31069.1.S1_at	3.74	2.40	3.49	3.72	4.26	3.72	3.21	0.71	3.90	0.31	1.21	0.280	2.0E-01
Mtr.44973.1.S1_at	7.27	6.14	7.46	7.07	7.15	6.82	6.96	0.71	7.01	0.17	1.01	0.012	9.0E-01
Mtr.14921.1.S1_at	2.58	3.80	2.54	2.53	2.55	2.93	2.97	0.71	2.67	0.22	0.90	-0.156	5.2E-01
Mtr.5905.1.S1_at	5.00	3.60	4.06	4.54	4.66	4.54	4.22	0.71	4.58	0.07	1.09	0.118	4.3E-01
Mtr.40385.1.S1_at	12.95	11.55	12.45	12.76	13.03	12.88	12.32	0.71	12.89	0.13	1.05	0.066	2.4E-01
Mtr.28177.1.S1_at	10.88	9.46	10.09	10.76	11.07	10.79	10.14	0.71	10.87	0.17	1.07	0.101	1.6E-01
Mtr.40775.1.S1_at	8.45	8.04	9.42	8.52	8.84	8.22	8.64	0.71	8.53	0.31	0.99	-0.018	8.2E-01
Mtr.38473.1.S1_at	5.39	4.91	6.31	5.76	5.93	5.70	5.53	0.71	5.80	0.12	1.05	0.066	5.6E-01
Mtr.49050.1.S1_at	7.04	6.06	7.44	7.36	7.12	7.14	6.85	0.71	7.21	0.13	1.05	0.074	4.4E-01
Mtr.41108.1.S1_at	8.97	8.02	9.41	9.24	9.25	9.09	8.80	0.71	9.20	0.09	1.04	0.063	3.9E-01
Mtr.30437.1.S1_at	2.44	3.66	2.42	2.41	2.41	2.42	2.84	0.71	2.41	0.01	0.85	-0.238	3.5E-01
Mtr.47734.1.S1_at	14.13	12.74	13.16	12.46	13.10	12.64	13.34	0.71	12.73	0.33	0.95	-0.068	2.5E-01
Mtr.13397.1.S1_at	9.17	8.72	7.78	8.46	8.55	8.36	8.56	0.71	8.46	0.09	0.99	-0.017	8.2E-01
Mtr.42023.1.S1_at	11.14	9.72	10.49	10.63	10.34	10.63	10.45	0.71	10.53	0.17	1.01	0.011	8.6E-01
Mtr.37066.1.S1_at	3.63	2.54	3.88	3.66	3.66	3.80	3.35	0.71	3.71	0.08	1.11	0.146	4.4E-01
Mtr.10648.1.S1_at	8.06	6.65	7.48	7.48	7.44	7.50	7.40	0.71	7.48	0.03	1.01	0.015	8.6E-01
Mtr.32449.1.S1_at	6.11	5.00	6.33	6.53	6.29	6.58	5.81	0.71	6.47	0.15	1.11	0.154	1.9E-01
Mtr.43913.1.S1_s_at	6.70	5.42	6.58	6.67	6.43	6.67	6.23	0.71	6.59	0.14	1.06	0.081	4.4E-01
Mtr.33880.1.S1_s_at	8.92	7.98	9.37	8.86	8.86	8.91	8.76	0.71	8.88	0.03	1.01	0.020	7.8E-01
Mtr.26570.1.S1_s_at	3.70	4.47	3.06	3.89	3.49	3.48	3.74	0.71	3.62	0.23	0.97	-0.049	7.9E-01
Mtr.1455.1.S1_at	7.75	6.91	8.32	7.45	7.82	7.72	7.66	0.71	7.66	0.19	1.00	0.000	1.0E+00
Mtr.45186.1.S1_at	7.99	6.62	7.61	7.50	7.55	7.70	7.41	0.71	7.58	0.11	1.02	0.034	6.9E-01
Mtr.28814.1.S1_at	7.87	7.01	8.41	7.73	7.87	7.80	7.76	0.71	7.80	0.07	1.00	0.007	9.4E-01
Mtr.33852.1.S1_at	8.08	6.68	7.19	7.65	7.64	8.04	7.32	0.71	7.78	0.23	1.06	0.088	3.4E-01
Mtr.20705.1.S1_at	8.27	7.38	8.78	8.40	8.26	8.11	8.15	0.71	8.26	0.14	1.01	0.020	8.0E-01
Mtr.34203.1.S1_at	7.47	6.27	7.51	7.46	7.35	7.56	7.08	0.71	7.46	0.10	1.05	0.074	4.2E-01
Mtr.28459.1.S1_at	6.79	5.41	6.37	6.83	6.78	6.91	6.19	0.71	6.84	0.07	1.10	0.143	1.9E-01
Mtr.31290.1.S1_at	4.45	3.53	4.93	4.75	5.00	4.30	4.30	0.71	4.68	0.36	1.09	0.122	4.5E-01
Mtr.37902.1.S1_s_at	10.31	8.90	9.60	9.92	9.55	10.02	9.60	0.71	9.83	0.25	1.02	0.033	6.3E-01
Mtr.34482.1.S1_at	6.49	5.55	6.94	6.25	6.42	6.75	6.33	0.71	6.47	0.25	1.02	0.033	7.5E-01
Mtr.19853.1.S1_at	10.16	8.85	9.97	9.89	9.68	9.86	9.66	0.71	9.81	0.11	1.02	0.022	7.4E-01
Mtr.45008.1.S1_at	8.24	6.91	7.99	8.20	8.05	8.09	7.72	0.71	8.11	0.08	1.05	0.072	3.9E-01
Mtr.17643.1.S1_at	3.98	5.16	3.90	3.89	3.80	4.24	4.35	0.71	3.98	0.23	0.91	-0.129	4.4E-01
Mtr.39630.1.S1_at	7.73	6.45	7.61	7.51	7.24	7.34	7.26	0.71	7.36	0.13	1.01	0.020	8.2E-01
Mtr.26645.1.S1_at	2.75	3.95	2.71	2.71	2.68	2.66	3.14	0.71	2.68	0.02	0.85	-0.227	3.3E-01
Mtr.34628.1.S1_at	8.96	7.79	9.07	8.92	9.21	8.86	8.61	0.71	9.00	0.19	1.05	0.064	4.1E-01
Mtr.39766.1.S1_s_at	6.28	4.96	5.17	5.46	5.07	5.62	5.47	0.71	5.38	0.28	0.98	-0.023	8.5E-01
Mtr.40715.1.S1_at	10.32	9.38	10.76	10.54	10.44	10.30	10.15	0.71	10.42	0.12	1.03	0.038	5.5E-01
Mtr.21112.1.S1_at	9.01	7.79	9.03	9.04	9.12	8.92	8.61	0.71	9.03	0.10	1.05	0.068	3.7E-01
Mtr.42581.1.S1_at	9.75	8.48	9.65	9.61	9.28	9.60	9.29	0.71	9.50	0.19	1.02	0.031	6.5E-01
Mtr.41928.1.S1_at	5.64	4.91	6.32	6.23	6.67	5.95	5.62	0.71	6.28	0.36	1.12	0.160	2.2E-01
Mtr.16949.1.S1_x_at	3.00	4.04	2.69	2.76	2.71	2.67	3.24	0.71	2.71	0.04	0.84	-0.256	2.7E-01
Mtr.9869.1.S1_at	8.08	6.89	8.14	8.35	8.78	8.30	7.70	0.71	8.48	0.26	1.10	0.138	1.5E-01
Mtr.39847.1.S1_at	7.21	6.09	7.40	7.24	7.13	7.27	6.90	0.71	7.22	0.07	1.05	0.064	4.9E-01

Mtr.8962.1.S1_at	6.00	7.37	6.98	8.16	8.78	8.56	6.78	0.71	8.50	0.32	1.25	0.325	1.9E-02
Mtr.31168.1.S1_at	8.31	7.03	8.17	8.37	8.51	8.24	7.84	0.71	8.37	0.13	1.07	0.095	2.7E-01
Mtr.10395.1.S1_at	8.01	6.61	7.12	6.40	6.50	6.84	7.25	0.71	6.58	0.23	0.91	-0.138	2.0E-01
Mtr.11229.1.S1_at	6.78	5.57	6.81	6.19	6.35	6.33	6.39	0.71	6.29	0.09	0.98	-0.022	8.2E-01
Mtr.42213.1.S1_at	5.93	5.13	6.54	5.89	6.82	5.73	5.87	0.71	6.15	0.59	1.05	0.067	6.3E-01
Mtr.13762.1.S1_at	8.55	7.48	8.81	8.49	8.39	8.34	8.28	0.71	8.41	0.08	1.02	0.022	7.7E-01
Mtr.40724.1.S1_at	6.29	4.99	6.12	7.69	8.51	8.26	5.80	0.71	8.15	0.42	1.41	0.491	7.6E-03
Mtr.44169.1.S1_at	10.85	11.51	12.26	10.97	11.14	11.17	11.54	0.71	11.09	0.11	0.96	-0.057	3.4E-01
Mtr.18479.1.S1_at	8.93	7.69	8.89	9.41	9.21	9.30	8.50	0.71	9.31	0.10	1.09	0.131	1.2E-01
Mtr.24984.1.S1_at	5.39	4.06	5.11	3.61	5.70	4.87	4.85	0.71	4.73	1.05	0.97	-0.039	8.7E-01
Mtr.10081.1.S1_s_at	5.48	4.40	5.73	6.12	5.87	6.17	5.20	0.71	6.05	0.16	1.16	0.218	1.1E-01
Mtr.13571.1.S1_at	6.84	5.98	7.38	7.43	7.67	7.48	6.73	0.71	7.53	0.12	1.12	0.162	1.3E-01
Mtr.33298.1.S1_s_at	12.01	10.96	12.30	13.13	13.56	13.40	11.76	0.71	13.36	0.21	1.14	0.184	2.0E-02
Mtr.11972.1.S1_at	8.05	6.66	7.57	7.87	7.69	8.08	7.43	0.71	7.88	0.19	1.06	0.085	3.4E-01
Mtr.7254.1.S1_at	7.92	6.69	7.90	7.70	7.89	7.69	7.50	0.71	7.76	0.11	1.03	0.049	5.7E-01
Mtr.17091.1.S1_at	9.12	9.49	10.49	9.49	9.55	9.19	9.70	0.71	9.41	0.19	0.97	-0.044	5.3E-01
Mtr.26263.1.S1_at	2.97	4.21	3.02	2.97	2.94	2.98	3.40	0.71	2.96	0.02	0.87	-0.199	3.4E-01
Mtr.13967.1.S1_at	9.84	8.91	8.46	8.99	8.29	8.66	9.07	0.71	8.65	0.35	0.95	-0.069	4.0E-01
Mtr.51127.1.S1_at	4.24	3.98	5.31	4.54	5.08	4.08	4.51	0.71	4.57	0.50	1.01	0.019	9.1E-01
Mtr.40432.1.S1_at	4.03	2.81	2.81	2.65	2.78	3.28	3.22	0.71	2.90	0.33	0.90	-0.150	5.2E-01
Mtr.5452.1.S1_at	4.38	3.26	4.56	4.83	5.57	4.87	4.06	0.70	5.09	0.42	1.25	0.326	9.5E-02
Mtr.51464.1.S1_at	10.22	8.87	9.89	9.99	9.62	10.08	9.66	0.70	9.89	0.24	1.02	0.035	6.1E-01
Mtr.50508.1.S1_at	11.63	10.30	10.54	10.99	10.97	11.02	10.83	0.70	11.00	0.03	1.02	0.023	7.0E-01
Mtr.31770.1.S1_at	9.63	8.27	9.29	9.15	8.85	8.90	9.06	0.70	8.96	0.16	0.99	-0.016	8.2E-01
Mtr.28434.1.S1_at	3.84	2.53	3.64	3.61	3.90	4.86	3.34	0.70	4.12	0.66	1.24	0.306	2.3E-01
Mtr.49419.1.S1_s_at	9.47	8.06	8.75	9.03	8.88	9.00	8.76	0.70	8.97	0.08	1.02	0.033	6.4E-01
Mtr.29833.1.S1_s_at	14.36	13.86	12.97	13.61	13.79	13.72	13.73	0.70	13.71	0.09	1.00	-0.002	9.6E-01
Mtr.33882.1.S1_at	8.76	7.52	8.72	8.51	8.79	8.46	8.33	0.70	8.59	0.18	1.03	0.044	5.7E-01
Mtr.33828.1.S1_at	6.59	7.15	7.99	6.51	6.64	5.98	7.24	0.70	6.38	0.35	0.88	-0.183	1.3E-01
Mtr.8267.1.S1_at	2.74	4.01	2.84	2.67	2.62	2.52	3.20	0.70	2.60	0.08	0.81	-0.296	2.2E-01
Mtr.9909.1.S1_s_at	10.95	9.73	10.95	11.02	11.15	10.76	10.54	0.70	10.98	0.20	1.04	0.058	3.7E-01
Mtr.14798.1.S1_at	6.57	5.40	6.66	6.54	6.61	6.41	6.21	0.70	6.52	0.10	1.05	0.070	4.9E-01
Mtr.36396.1.S1_at	7.58	6.51	7.84	7.39	7.31	7.11	7.31	0.70	7.27	0.14	0.99	-0.008	9.2E-01
Mtr.42432.1.S1_at	8.54	7.24	8.36	8.40	8.55	8.25	8.05	0.70	8.40	0.15	1.04	0.062	4.5E-01
Mtr.12376.1.S1_at	4.88	3.52	4.53	4.78	4.80	4.16	4.31	0.70	4.58	0.36	1.06	0.088	5.9E-01
Mtr.39386.1.S1_at	8.87	7.89	9.26	8.72	8.93	8.71	8.67	0.70	8.79	0.12	1.01	0.019	8.0E-01
Mtr.32572.1.S1_at	7.97	6.57	7.14	7.58	7.52	7.72	7.23	0.70	7.61	0.10	1.05	0.074	4.1E-01
Mtr.21375.1.S1_s_at	8.36	7.68	9.09	8.75	8.79	8.72	8.38	0.70	8.76	0.03	1.04	0.063	4.1E-01
Mtr.26413.1.S1_at	8.59	7.60	8.96	8.55	8.80	8.64	8.38	0.70	8.66	0.13	1.03	0.047	5.4E-01
Mtr.32364.1.S1_at	4.69	3.57	3.40	3.91	4.80	3.90	3.89	0.70	4.20	0.52	1.08	0.114	5.6E-01
Mtr.15072.1.S1_at	4.46	5.77	4.66	4.66	4.66	4.65	4.96	0.70	4.66	0.00	0.94	-0.090	5.0E-01
Mtr.36209.1.S1_at	4.89	4.25	5.66	4.99	6.07	4.89	4.93	0.70	5.32	0.65	1.08	0.107	5.3E-01
Mtr.7993.1.S1_s_at	4.31	3.29	2.96	2.94	2.88	2.85	3.52	0.70	2.89	0.05	0.82	-0.285	2.0E-01
Mtr.19451.1.S1_at	2.97	4.17	2.93	3.17	2.86	2.89	3.35	0.70	2.97	0.17	0.89	-0.174	4.1E-01
Mtr.7567.1.S1_s_at	6.04	4.67	5.62	5.44	5.36	5.42	5.45	0.70	5.41	0.04	0.99	-0.010	9.3E-01
Mtr.44611.1.S1_at	7.59	6.74	8.14	7.71	7.41	7.48	7.49	0.70	7.54	0.16	1.01	0.009	9.2E-01
Mtr.41327.1.S1_at	8.34	7.12	8.33	8.24	8.46	8.40	7.93	0.70	8.37	0.12	1.06	0.078	3.5E-01
Mtr.32564.1.S1_at	8.04	6.69	7.70	8.15	8.37	8.23	7.48	0.70	8.25	0.11	1.10	0.142	1.3E-01
Mtr.13726.1.S1_at	7.32	5.96	6.33	6.93	6.44	6.93	6.54	0.70	6.77	0.28	1.04	0.050	6.3E-01
Mtr.18478.1.S1_at	12.01	10.60	11.34	11.26	11.27	11.30	11.32	0.70	11.27	0.02	1.00	-0.005	9.2E-01
Mtr.42918.1.S1_at	13.11	12.14	11.74	11.57	10.70	11.39	12.33	0.70	11.22	0.46	0.91	-0.136	8.4E-02
Mtr.23586.1.S1_at	8.79	7.66	8.95	8.53	8.50	8.11	8.47	0.70	8.38	0.24	0.99	-0.015	8.5E-01
Mtr.43833.1.S1_at	12.47	11.64	11.07	11.32	10.96	11.34	11.73	0.70	11.21	0.21	0.96	-0.066	2.9E-01
Mtr.27443.1.S1_at	5.56	6.79	5.60	5.56	5.62	5.54	5.98	0.70	5.57	0.04	0.93	-0.102	3.7E-01
Mtr.51080.1.S1_at	8.04	6.72	7.79	7.66	7.76	7.51	7.52	0.70	7.64	0.13	1.02	0.024	7.8E-01
Mtr.34204.1.S1_at	3.18	4.41	3.20	3.28	3.17	3.16	3.60	0.70	3.20	0.07	0.89	-0.167	3.9E-01
Mtr.41085.1.S1_at	2.53	3.91	3.02	2.51	2.50	2.50	3.15	0.70	2.51	0.01	0.79	-0.332	1.9E-01
Mtr.27926.1.S1_s_at	8.36	7.12	8.32	8.00	7.91	8.01	7.93	0.70	7.98	0.05	1.01	0.007	9.2E-01
Mtr.32200.1.S1_at	7.76	6.57	7.81	7.67	7.18	7.82	7.38	0.70	7.56	0.34	1.02	0.035	7.1E-01
Mtr.24901.1.S1_at	3.82	2.61	3.84	3.83	5.46	3.83	3.43	0.70	4.38	0.94	1.28	0.353	2.3E-01
Mtr.40566.1.S1_s_at	8.77	7.60	8.85	8.73	8.82	8.70	8.40	0.70	8.75	0.07	1.04	0.058	4.5E-01
Mtr.21902.1.S1_at	4.13	4.52	5.49	6.87	6.97	7.29	4.71	0.70	7.04	0.22	1.50	0.580	5.3E-03
Mtr.4744.1.S1_at	6.66	6.20	7.58	6.89	6.76	6.83	6.81	0.70	6.83	0.06	1.00	0.003	9.7E-01
Mtr.45512.1.S1_at	8.65	7.57	8.89	8.65	8.69	8.83	8.37	0.70	8.72	0.09	1.04	0.059	4.4E-01
Mtr.36729.1.S1_at	2.29	3.50	2.28	2.28	2.28	2.28	2.69	0.70	2.28	0.00	0.85	-0.240	3.7E-01
Mtr.41238.1.S1_at	11.75	10.89	10.36	10.66	10.68	10.83	11.00	0.70	10.73	0.09	0.97	-0.037	5.4E-01
Mtr.44825.1.S1_at	8.58	7.23	8.25	8.59	8.24	8.46	8.02	0.70	8.43	0.18	1.05	0.072	3.8E-01
Mtr.22057.1.S1_at	3.89	3.03	4.42	4.00	3.82	3.77	3.78	0.70	3.86	0.12	1.02	0.030	8.6E-01
Mtr.29764.1.S1_at	4.62	3.35	4.50	4.39	5.59	4.23	4.16	0.70	4.73	0.74	1.14	0.187	3.8E-01
Mtr.33518.1.S1_at	3.34	2.50	3.89	3.55	3.33	3.31	3.24	0.70	3.40	0.13	1.05	0.067	7.3E-01
Mtr.31793.1.S1_at	9.85	8.65	9.87	9.92	9.87	9.73	9.45	0.70	9.84	0.10	1.04	0.058	4.0E-01
Mtr.17542.1.S1_at	2.24	3.44	2.22	2.22	2.22	2.22	2.63	0.70	2.22	0.00	0.84	-0.247	3.6E-01
Mtr.1230.1.S1_s_at	9.12	7.75	8.71	8.92	8.91	9.01	8.52	0.70	8.94	0.05	1.05	0.069	3.6E-01
Mtr.13337.1.S1_at	8.45	7.22	8.42	8.47	8.41	8.48	8.03	0.70	8.46	0.04	1.05	0.075	3.5E-01
Mtr.38645.1.S1_at	7.07	6.40	7.80	7.11	7.40	7.16	7.09	0.70	7.22	0.16	1.02	0.026	7.7E-01
Mtr.12550.1.S1_at	9.71	9.81	10.97	11.28	11.68	11.43	10.16	0.70	11.46	0.20	1.13	0.173	3.7E-02
Mtr.27118.1.S1_at	6.05	7.44	6.57	6.02	6.33	6.11	6.69	0.70	6.15	0.16	0.92	-0.120	2.7E-01
Mtr.32495.1.S1_at	4.31	2.99	4.07	3.82	4.08	4.21	3.79	0.70	4.04	0.19	1.07	0.091	5.9E-01
Mtr.1354.1.S1_at	3.75	2.43	3.48	3.44	3.43	3.44	3.22	0.70	3.44	0.00	1.07	0.094	6.2E-01
Mtr.10767.1.S1_at	8.52	7.25	7.37	7.35	6.95	7.22	7.71	0.70	7.17	0.20	0.93	-0.104	2.7E-01
Mtr.26023.1.S1_s_at	6.85	5.45	6.22	6.21	6.21	6.32	6.17	0.70	6.25	0.06	1.01	0.018	8.6E-01
Mtr.11757.1.S1_at	6.84	5.73	7.03	6.60	6.87	6.06	6.53	0.70	6.51	0.41	1.00	-0.005	9.6E-01
Mtr.43617.1.S1_at	9.22	8.38	9.77	9.08	9.17	9.18	9.12	0.70	9.14	0.06	1.00	0.003	9.7E-01
Mtr.21245.1.S1_at	8.31	6.93	7.86	8.34	8.33	8.06	7.70	0.70	8.24	0.16	1.07	0.098	2.6E-01
Mtr.21335.1.S1_at	3.07	4.47	3.71	3.62	2.97	3.40	3.75	0.70	3.33	0.33	0.89	-0.172	4.0E-01
Mtr.41266.1.S1_at	2.21	3.41	2.20	2.19	2.19	2.16	2.61	0.70	2.18	0.02	0.84	-0.256	3.5E-01
Mtr.44867.1.S1_at	9.49	8.25	9.43	9.61	9.41	9.64	9.05	0.70	9.55	0.13	1.06	0.077	2.9E-01
Mtr.989.1.S1_s_at	5.41	6.66	6.58	7.12	7.19	6.62	6.21	0.70	6.98	0.31	1.12	0.167	1.6E-01
Mtr.32893.1.S1_at	4.36	3.76	2.96	3.80	3.48	3.61	3.69	0.70	3.63	0.16	0.98	-0.023	8.9E-01
Mtr.15800.1.S1_at	8.17	9.28	7.99	8.02	8.02	7.91	8.48	0.70	7.99	0.06	0.94	-0.087	2.9E-01
Mtr.31339.1.S1_at	6.22	7.58	6.62	7.98	8.05	8.15	6.81	0.70	8.06	0.09	1.18	0.244	3.7E-02
Mtr.35725.1.S1_at	8.75	10.14	9.29	7.77	8.43	7.71	9.39	0.70	7.97	0.40	0.85	-0.237	3.7E-02
Mtr.9155.1.S1_at	3.91	2.79	4.08	4.22	3.90	3.90	3.59	0.70	4.00	0.18	1.11	0.156	3.8E-01
Mtr.24310.1.S1_at	2.78	3.95	2.71	2.65	2.65	2.63	3.15	0.70	2.64	0.01	0.84	-0.252	2.8E-01
Mtr.21922.1.S1_at	6.61	5.51	6.80	6.83	6.60	6.40	6.31	0.70	6.61	0.22	1.05	0.067	5.1E-01
Mtr.33296.1.S1_at	3.95	2.59	3.53	3.51	4.05	3.23	3.36	0.70	3.60	0.41	1.07	0.101	6.3E-01
Mtr.22634.1.S1_at	8.42	7.03	7.77	8.10	8.11	8.29	7.74	0.70	8.17	0.11	1.06	0.077	3.5E-01
Mtr.4526.1.S1_at	7.07	7.59	8.45	7.72	7.87	7.29	7.71	0.70	7.63	0.30	0.99	-0.015	8.6E-01
Mtr.3091.1.S1_s_at	7.82	6.57	7.73	7.78	7.66	7.71	7.37	0.70	7.72	0.06	1.05	0.066	4.4E-01
Mtr.1561.1.S1_s_at	8.25	6.97	8.10	7.98	8.38	8.23	7.77	0.70	8.20	0.21	1.05	0.076	3.7E-01
Mtr.47432.1.S1_at	4.28	4.75	3.37	4.12	3.93	4.09	4.13	0.70	4.04	0.10	0.98	-0.031	8.4E-01
Mtr.12188.1.S1_at	4.90	4.82	6.06	4.77	4.99	5.08	5.26	0.70	4.95	0.16	0.94	-0.088	4.9E-01
Mtr.16188.1.S1_at	8.23	7.87	9.22	8.50	8.68	8.32	8.44	0.70	8.50	0.18	1.01	0.010	8.9E-01
Mtr.27163.1.S1_at	4.55	3.46	4.77	4.54	4.58	4.74	4.26	0.70	4.62	0.11	1.08	0.118	4.3E-01
Mtr.17662.1.S1_x_at	7.75	6.52	7.71	7.69	7.84	7.70	7.32	0.70	7.74	0.08	1.06	0.080	3.6E-01
Mtr.27246.1.S1_at	6.12	5.11	6.45	7.28	8.42	7.69	5.89	0.70	7.80	0.58	1.32	0.405	2.2E-02
Mtr.29582.1.S1_at	4.21	5.41	4.19	4.17	4.19	4.18	4.60	0.70	4.18	0.01	0.91	-0.137	3.6E-01
Mtr.6322.1.S1_s_at	10.03	10.36	11.37	10.90	11.13	10.35	10.58	0.70	10.79	0.40	1.02	0.028	6.8E-01
Mtr.31751.1.S1_at	8.57	7.48	8.78	7.91	8.52	7.98	8.28	0.70	8.14	0.34	0.98	-0.025	7.7E-01
Mtr.16204.1.S1_at	10.12	9.17	10.53	10.02	10.02	9.89	9.94	0.70	9.98	0.07	1.00	0.005	9.3E-01
Mtr.10995.1.S1_at	9.43	8.12	9.20	9.30	9.03	9.18	8.92	0.70	9.17	0.14	1.03	0.041	5.7E-01
Mtr.6100.1.S1_at	10.62	9.51	9.33	9.65	9.41	9.80	9.82	0.70	9.62	0.19	0.98	-0.030	6.5E-01
Mtr.40122.1.S1_s_at	7.17	8.19	6.85	5.91	6.13	6.60	7.40	0.70	6.21	0.35	0.84	-0.253	5.8E-02
Mtr.10601.1.S1_at	11.38	10.01	10.46	10.69	10.80	10.70	10.62	0.70	10.73	0.06	1.01	0.015	8.0E-01
Mtr.16456.1.S1_at	2.42	3.62	2.40	2.39	2.37	2.37	2.81	0.70	2.38	0.01	0.85	-0.243	3.4E-01
Mtr.12034.1.S1_at	9.23	7.92	8.98	8.79	9.20	8.79	8.71	0.70	8.93	0.24	1.03	0.036	6.4E-01
Mtr.27120.1.S1_at	3.59	2.74	4.12	4.06	3.64	4.53	3.48	0.70	4.08	0.44	1.17	0.229	2.8E-01
Mtr.14131.1.S1_at	3.97	5.18	3.98	3.96	4.10	3.96	4.38	0.70	4.01	0.08	0.92	-0.128	4.1E-01
Mtr.51593.1.S1_at	5.31	6.32	4.98	5.53	5.54	5.80	5.53	0.70	5.62	0.16	1.02	0.023	8.4E-01
Mtr.5245.1.S1_at	11.11	11.56	12.48	11.82	11.76	11.73	11.72	0.70	11.77	0.05	1.00	0.007	9.0E-01
Mtr.41503.1.S1_at	3.55	2.36	2.33	2.81	2.28	2.28	2.75	0.70	2.46	0.31	0.90	-0.159	5.5E-01
Mtr.40455.1.S1_s_at	8.28	6.94	7.95	8.10	8.09	8.11	7.72	0.70	8.10	0.01	1.05	0.069	4.0E-01
Mtr.12778.1.S1_at	4.94	4.46	3.57	4.60	4.97	4.39	4.32	0.70	4.65	0.30	1.08	0.106	4.9E-01
Mtr.28779.1.S1_at	8.13	6.74	7.32	7.64	7.71	7.69	7.40	0.70	7.68	0.04	1.04	0.054	5.2E-01
Mtr.6812.1.S1_at	7.02	7.14	8.29	8.18	8.38	8.35	7.48	0.70	8.30	0.11	1.11	0.150	1.1E-01
Mtr.11554.1.S1_at	9.94	8.59	9.00	9.62	9.61	9.71	9.18	0.70	9.65	0.06	1.05	0.072	3.1E-01
Mtr.15904.1.S1_at	7.25	7.91	8.64	8.90	9.14	9.13	7.93	0.70	9.06	0.13	1.14	0.191	5.2E-02
Mtr.10968.1.S1_at	12.45	12.69	11.38	11.48	11.75	11.70	12.17	0.70	11.64	0.15	0.96	-0.065	2.6E-01
Mtr.21894.1.S1_at	6.40	5.23	6.47	6.50	6.40	6.09	6.04	0.70	6.33	0.22	1.05	0.069	5.2E-01
Mtr.32838.1.S1_at	6.30	4.91	5.72	6.63	6.85	6.72	5.64	0.70	6.73	0.11	1.19	0.255	5.6E-02
Mtr.40772.1.S1_at	4.43	3.38	4.69	4.15	4.12	4.02	4.17	0.70	4.10	0.07	0.98	-0.025	8.7E-01

Mtr.39696.1.S1_at	4.59	4.47	5.73	4.45	4.65	3.93	4.93	0.70	4.34	0.37	0.88	-0.182	2.7E-01
Mtr.41631.1.S1_at	7.91	6.60	7.65	7.85	7.66	7.75	7.39	0.70	7.75	0.09	1.05	0.070	4.2E-01
Mtr.27116.1.S1_at	5.75	4.60	5.84	5.69	5.60	5.86	5.40	0.70	5.71	0.13	1.06	0.082	4.8E-01
Mtr.35043.1.S1_at	6.59	5.47	6.75	6.73	7.00	6.76	6.27	0.70	6.83	0.15	1.09	0.123	2.5E-01
Mtr.6562.1.S1_at	4.67	3.42	4.58	4.99	4.79	4.44	4.23	0.70	4.74	0.28	1.12	0.165	3.0E-01
Mtr.4382.1.S1_at	8.46	7.34	8.60	8.59	8.64	8.39	8.13	0.70	8.54	0.13	1.05	0.070	3.8E-01
Mtr.51200.1.S1_at	7.58	6.43	7.68	7.72	7.84	7.60	7.23	0.70	7.72	0.12	1.07	0.094	3.0E-01
Mtr.43008.1.S1_at	10.20	11.53	11.24	10.61	11.08	10.64	10.99	0.70	10.78	0.26	0.98	-0.028	6.5E-01
Mtr.40149.1.S1_at	14.14	12.82	13.87	13.72	13.64	13.38	13.61	0.70	13.58	0.18	1.00	-0.003	9.5E-01
Mtr.45828.1.S1_at	8.61	7.33	8.45	8.40	8.46	8.36	8.13	0.70	8.41	0.05	1.03	0.049	5.3E-01
Mtr.14257.1.S1_at	8.26	8.37	9.52	8.71	8.65	8.59	8.71	0.70	8.65	0.06	0.99	-0.011	8.8E-01
Mtr.13357.1.S1_at	13.37	12.13	13.29	13.29	12.83	13.14	12.93	0.70	13.08	0.23	1.01	0.017	7.3E-01
Mtr.27995.1.S1_at	8.53	7.48	8.80	8.75	8.69	8.60	8.27	0.70	8.68	0.08	1.05	0.071	3.6E-01
Mtr.2050.1.S1_at	4.52	3.41	4.69	5.21	4.68	4.63	4.20	0.69	4.84	0.33	1.15	0.203	2.2E-01
Mtr.39219.1.S1_at	7.77	6.99	8.38	8.03	8.04	8.01	7.71	0.69	8.03	0.01	1.04	0.058	4.8E-01
Mtr.22643.1.S1_s_at	13.42	12.23	13.45	13.85	14.01	13.96	13.04	0.69	13.94	0.08	1.07	0.097	8.8E-02
Mtr.9816.1.S1_at	8.25	7.56	8.95	8.55	8.24	8.29	8.25	0.69	8.36	0.17	1.01	0.019	8.1E-01
Mtr.5190.1.S1_at	6.33	5.17	6.41	6.56	6.30	6.49	5.97	0.69	6.45	0.13	1.08	0.111	3.1E-01
Mtr.13524.1.S1_at	7.01	6.30	7.69	7.13	7.00	6.84	7.00	0.69	6.99	0.14	1.00	-0.003	9.8E-01
Mtr.44497.1.S1_at	9.66	9.30	10.64	9.75	9.85	9.63	9.87	0.69	9.74	0.11	0.99	-0.018	7.7E-01
Mtr.37669.1.S1_at	7.66	6.49	7.72	7.62	7.48	7.49	7.29	0.69	7.53	0.08	1.03	0.047	5.8E-01
Mtr.8245.1.S1_at	3.15	4.34	3.12	3.12	3.32	3.30	3.54	0.69	3.25	0.11	0.92	-0.122	5.2E-01
Mtr.11768.1.S1_at	9.43	8.14	8.33	8.49	8.40	8.72	8.63	0.69	8.54	0.16	0.99	-0.016	8.3E-01
Mtr.41988.1.S1_at	7.61	6.23	7.03	7.67	7.60	7.95	6.96	0.69	7.74	0.18	1.11	0.154	1.3E-01
Mtr.17411.1.S1_at	5.03	3.67	4.59	4.93	5.10	5.02	4.43	0.69	5.01	0.08	1.13	0.180	2.2E-01
Mtr.43486.1.S1_at	11.59	10.70	10.22	11.11	10.99	11.17	10.84	0.69	11.09	0.09	1.02	0.033	5.7E-01
Mtr.31490.1.S1_at	8.89	7.69	8.88	8.99	8.88	8.99	8.49	0.69	8.95	0.06	1.06	0.077	3.1E-01
Mtr.5403.1.S1_at	7.40	6.29	7.56	7.61	7.38	7.10	7.08	0.69	7.36	0.26	1.04	0.056	5.5E-01
Mtr.33340.1.S1_at	3.25	2.95	4.28	3.24	4.81	3.24	3.49	0.69	3.76	0.90	1.08	0.107	7.0E-01
Mtr.16363.1.S1_s_at	8.39	8.70	7.38	9.13	9.43	9.16	8.16	0.69	9.24	0.16	1.13	0.180	5.8E-02
Mtr.42663.1.S1_at	6.53	5.29	5.38	5.20	5.35	5.45	5.74	0.69	5.33	0.12	0.93	-0.105	3.8E-01
Mtr.4018.1.S1_at	2.65	3.83	2.60	2.60	2.60	2.58	3.03	0.69	2.59	0.01	0.86	-0.224	3.4E-01
Mtr.5634.1.S1_at	8.49	7.18	7.45	8.01	7.81	7.86	7.71	0.69	7.89	0.11	1.02	0.034	6.7E-01
Mtr.6926.1.S1_at	8.27	6.90	7.76	8.03	8.28	7.92	7.64	0.69	8.08	0.19	1.06	0.080	3.5E-01
Mtr.42501.1.S1_at	7.93	6.86	8.16	8.16	8.10	8.21	7.65	0.69	8.16	0.06	1.07	0.093	2.7E-01
Mtr.44899.1.S1_at	8.60	7.23	8.11	8.33	8.26	8.32	7.98	0.69	8.30	0.03	1.04	0.057	4.6E-01
Mtr.29967.1.S1_at	2.08	3.27	2.07	2.07	2.07	2.07	2.47	0.69	2.07	0.00	0.84	-0.259	3.7E-01
Mtr.39313.1.S1_at	9.05	9.91	8.54	10.52	10.68	10.36	9.16	0.69	10.52	0.16	1.15	0.199	3.0E-02
Mtr.51135.1.S1_x_at	5.20	3.97	5.13	5.03	5.89	5.03	4.77	0.69	5.32	0.50	1.12	0.158	3.2E-01
Mtr.34388.1.S1_at	5.54	4.84	6.22	6.01	5.85	5.95	5.53	0.69	5.94	0.08	1.07	0.102	3.7E-01
Mtr.1639.1.S1_at	10.15	9.17	8.81	9.46	8.86	9.78	9.38	0.69	9.37	0.47	1.00	-0.002	9.8E-01
Mtr.21466.1.S1_at	8.19	6.81	7.54	7.36	7.48	7.56	7.51	0.69	7.47	0.10	0.99	-0.008	9.2E-01
Mtr.24776.1.S1_at	2.45	3.63	2.42	2.41	2.41	2.43	2.83	0.69	2.42	0.01	0.85	-0.229	3.6E-01
Mtr.21646.1.S1_s_at	11.57	10.20	10.72	11.59	12.45	12.39	10.83	0.69	12.14	0.48	1.12	0.165	5.4E-02
Mtr.33693.1.S1_at	7.20	6.79	8.14	8.34	8.29	8.09	7.37	0.69	8.24	0.13	1.12	0.160	1.0E-01
Mtr.33602.1.S1_at	8.29	7.04	8.18	8.12	8.03	8.20	7.84	0.69	8.12	0.09	1.04	0.051	5.2E-01
Mtr.28413.1.S1_at	5.92	4.77	4.69	4.82	4.67	4.32	5.13	0.69	4.60	0.26	0.90	-0.156	2.8E-01
Mtr.2888.1.S1_at	5.87	4.58	5.66	5.50	5.81	5.95	5.37	0.69	5.75	0.23	1.07	0.100	4.1E-01
Mtr.40322.1.S1_at	9.92	8.54	9.27	10.08	10.09	9.82	9.24	0.69	10.00	0.16	1.08	0.113	1.4E-01
Mtr.47588.1.S1_x_at	9.78	9.64	10.90	10.33	10.46	10.28	10.11	0.69	10.36	0.09	1.02	0.035	5.7E-01
Mtr.40407.1.S1_at	8.27	7.85	9.20	8.53	8.50	8.30	8.44	0.69	8.44	0.12	1.00	0.001	9.9E-01
Mtr.20529.1.S1_at	2.32	3.51	2.31	2.30	2.30	2.30	2.71	0.69	2.30	0.00	0.85	-0.239	3.6E-01
Mtr.7689.1.S1_at	2.93	4.13	2.93	2.93	2.92	2.92	3.33	0.69	2.92	0.00	0.88	-0.188	3.7E-01
Mtr.2082.1.S1_at	8.10	7.07	8.39	8.26	8.10	8.23	7.85	0.69	8.20	0.09	1.04	0.062	4.4E-01
Mtr.27227.1.S1_at	7.03	5.75	6.83	7.08	7.09	7.08	6.54	0.69	7.08	0.01	1.08	0.116	2.4E-01
Mtr.11643.1.S1_at	8.31	6.98	7.98	8.16	8.05	8.33	7.76	0.69	8.18	0.14	1.05	0.076	3.6E-01
Mtr.40546.1.S1_at	6.56	5.66	7.01	5.96	6.45	6.10	6.41	0.69	6.17	0.26	0.96	-0.056	6.0E-01
Mtr.33869.1.S1_at	5.50	4.80	6.19	5.64	5.69	5.68	5.50	0.69	5.67	0.03	1.03	0.046	6.8E-01
Mtr.10286.1.S1_at	5.49	4.21	5.29	5.35	5.53	6.35	5.00	0.69	5.74	0.54	1.15	0.201	2.1E-01
Mtr.9426.1.S1_at	10.43	9.05	9.72	10.12	10.14	10.13	9.73	0.69	10.13	0.01	1.04	0.058	3.8E-01
Mtr.35105.1.S1_s_at	7.45	6.21	7.36	7.32	7.39	7.42	7.00	0.69	7.38	0.05	1.05	0.074	4.1E-01
Mtr.13153.1.S1_at	8.31	8.79	9.67	9.36	9.60	8.91	8.92	0.69	9.29	0.35	1.04	0.058	4.6E-01
Mtr.43078.1.S1_at	8.67	7.49	8.69	8.38	8.24	8.38	8.28	0.69	8.33	0.08	1.01	0.009	9.1E-01
Mtr.19469.1.S1_at	5.90	4.54	5.44	6.60	6.63	6.95	5.29	0.69	6.73	0.20	1.27	0.346	2.6E-02
Mtr.33221.1.S1_s_at	7.96	6.58	7.30	7.47	7.75	7.68	7.28	0.69	7.63	0.14	1.05	0.068	4.4E-01
Mtr.34624.1.S1_at	7.46	6.27	6.27	6.14	6.56	5.94	6.67	0.69	6.21	0.32	0.93	-0.102	3.6E-01
Mtr.39989.1.S1_at	5.18	4.34	5.70	5.60	5.70	5.45	5.07	0.69	5.58	0.13	1.10	0.138	2.8E-01
Mtr.9814.1.S1_at	5.53	6.51	6.87	5.76	5.57	5.65	6.31	0.69	5.66	0.09	0.90	-0.155	1.9E-01
Mtr.18981.1.S1_at	7.01	6.37	7.75	7.72	7.45	7.28	7.04	0.69	7.48	0.22	1.06	0.088	3.5E-01
Mtr.28581.1.S1_at	7.93	6.55	7.30	7.78	7.51	7.48	7.26	0.69	7.59	0.16	1.05	0.064	4.7E-01
Mtr.27720.1.S1_at	5.95	4.86	6.13	5.81	5.54	5.82	5.65	0.69	5.72	0.16	1.01	0.020	8.6E-01
Mtr.19967.1.S1_x_at	6.96	8.26	7.21	7.64	8.01	7.88	7.48	0.69	7.84	0.19	1.05	0.069	4.3E-01
Mtr.6817.1.S1_s_at	9.18	7.88	8.94	8.86	8.81	8.73	8.66	0.69	8.80	0.06	1.02	0.022	7.5E-01
Mtr.34199.1.S1_at	8.63	7.82	9.19	8.86	7.66	8.89	8.55	0.69	8.47	0.70	0.99	-0.013	9.0E-01
Mtr.42313.1.S1_s_at	6.86	5.59	5.76	6.19	6.51	6.39	6.07	0.69	6.36	0.16	1.05	0.067	5.2E-01
Mtr.20842.1.S1_at	9.33	8.21	9.46	9.88	10.07	9.78	9.00	0.69	9.91	0.15	1.10	0.139	8.9E-02
Mtr.19044.1.S1_at	6.04	4.88	6.11	5.90	6.19	5.94	5.68	0.69	6.01	0.15	1.06	0.082	4.6E-01
Mtr.41031.1.S1_at	11.16	10.02	9.92	10.41	10.05	10.48	10.37	0.69	10.32	0.23	1.00	-0.007	9.1E-01
Mtr.7429.1.S1_s_at	8.45	7.39	8.68	8.56	8.32	8.17	8.17	0.69	8.35	0.20	1.02	0.031	6.9E-01
Mtr.29432.1.S1_at	3.14	2.15	3.47	3.08	3.68	3.13	2.92	0.69	3.30	0.33	1.13	0.176	4.4E-01
Mtr.40827.1.S1_at	11.93	10.63	10.89	10.45	11.13	10.93	11.15	0.69	10.83	0.35	0.97	-0.042	5.1E-01
Mtr.51581.1.S1_s_at	6.45	5.78	7.16	6.58	7.46	6.56	6.46	0.69	6.87	0.51	1.06	0.087	4.6E-01
Mtr.12693.1.S1_at	8.28	6.96	7.97	8.28	8.39	8.42	7.74	0.69	8.36	0.07	1.08	0.112	1.9E-01
Mtr.170.1.S1_at	6.91	5.90	7.22	7.21	7.07	7.17	6.67	0.69	7.15	0.07	1.07	0.099	3.0E-01
Mtr.19649.1.S1_at	3.70	2.47	3.63	3.43	3.45	3.44	3.27	0.69	3.44	0.01	1.05	0.074	6.9E-01
Mtr.98.1.S1_at	3.61	3.56	4.78	4.42	4.62	4.35	3.98	0.69	4.46	0.14	1.12	0.164	3.0E-01
Mtr.6204.1.S1_at	2.34	3.52	2.32	2.31	2.30	2.30	2.73	0.69	2.30	0.01	0.84	-0.243	3.5E-01
Mtr.1691.1.S1_at	3.70	2.67	3.98	3.56	3.54	3.49	3.45	0.69	3.53	0.04	1.02	0.032	8.5E-01
Mtr.17235.1.S1_at	6.53	5.33	6.52	6.16	6.05	5.60	6.13	0.69	5.93	0.30	0.97	-0.046	6.8E-01
Mtr.33278.1.S1_at	7.00	6.21	7.59	7.17	7.36	7.55	6.93	0.69	7.36	0.19	1.06	0.086	3.6E-01
Mtr.35357.1.S1_at	4.76	5.29	3.93	5.19	4.86	4.96	4.66	0.69	5.00	0.17	1.07	0.103	4.5E-01
Mtr.13469.1.S1_at	6.71	5.55	6.78	6.74	6.68	6.40	6.35	0.69	6.61	0.18	1.04	0.058	5.6E-01
Mtr.13089.1.S1_at	10.16	8.78	9.52	9.81	9.85	9.87	9.49	0.69	9.84	0.03	1.04	0.053	4.2E-01
Mtr.11414.1.S1_at	8.78	7.41	8.00	8.59	8.19	8.61	8.06	0.69	8.46	0.23	1.05	0.070	4.0E-01
Mtr.34944.1.S1_at	8.28	7.04	7.14	7.86	8.00	8.19	7.49	0.69	8.02	0.16	1.07	0.099	2.6E-01
Mtr.16411.1.S1_at	6.80	5.87	7.21	6.84	7.24	6.98	6.63	0.69	7.02	0.20	1.06	0.083	4.0E-01
Mtr.42850.1.S1_at	9.93	9.22	8.56	8.12	8.55	8.62	9.24	0.69	8.43	0.27	0.91	-0.132	1.3E-01
Mtr.12025.1.S1_at	6.37	7.60	6.45	6.76	8.12	7.54	6.81	0.69	7.47	0.68	1.10	0.135	3.0E-01
Mtr.49099.1.S1_at	7.82	7.24	6.45	7.84	7.79	7.92	7.17	0.69	7.85	0.06	1.09	0.130	1.6E-01
Mtr.40027.1.S1_at	13.86	13.75	12.62	13.53	13.67	13.63	13.41	0.69	13.61	0.07	1.02	0.022	6.4E-01
Mtr.29593.1.S1_at	5.64	4.27	5.00	5.58	5.58	5.68	4.97	0.69	5.61	0.06	1.13	0.176	1.8E-01
Mtr.20327.1.S1_at	7.41	6.29	7.53	7.10	6.91	7.21	7.08	0.69	7.07	0.15	1.00	-0.001	9.9E-01
Mtr.36283.1.S1_at	9.07	8.09	9.42	9.06	9.19	8.77	8.86	0.69	9.00	0.22	1.02	0.024	7.4E-01
Mtr.10672.1.S1_s_at	8.56	7.27	8.32	8.08	7.99	8.24	8.05	0.69	8.10	0.12	1.01	0.009	9.0E-01
Mtr.6903.1.S1_at	8.30	7.44	8.80	8.94	8.69	8.81	8.18	0.69	8.81	0.13	1.08	0.107	1.9E-01
Mtr.38193.1.S1_at	10.39	9.06	9.41	9.09	9.52	9.22	9.62	0.69	9.28	0.22	0.96	-0.052	4.6E-01
Mtr.11520.1.S1_at	6.93	5.56	6.16	5.79	6.13	5.82	6.22	0.69	5.91	0.19	0.95	-0.073	5.0E-01
Mtr.26282.1.S1_x_at	7.80	6.81	8.13	7.74	7.72	7.76	7.58	0.69	7.74	0.02	1.02	0.030	7.1E-01
Mtr.34975.1.S1_at	7.61	7.50	8.74	8.20	8.39	7.94	7.95	0.69	8.18	0.23	1.03	0.040	6.2E-01
Mtr.10207.1.S1_at	7.40	6.04	6.89	6.89	6.89	6.55	6.78	0.69	6.78	0.20	1.00	0.000	1.0E+00
Mtr.10569.1.S1_at	11.04	10.20	11.56	11.16	10.96	10.95	10.93	0.69	11.02	0.12	1.01	0.012	8.3E-01
Mtr.29039.1.S1_at	6.15	4.77	5.46	5.65	5.28	5.27	5.46	0.69	5.40	0.22	0.99	-0.016	8.9E-01
Mtr.41970.1.S1_at	9.79	8.49	8.76	9.55	10.00	9.76	9.01	0.69	9.77	0.23	1.08	0.116	1.4E-01
Mtr.11742.1.S1_at	6.88	8.25	7.44	7.32	7.53	7.29	7.52	0.69	7.38	0.13	0.98	-0.027	7.4E-01
Mtr.27101.1.S1_at	4.17	3.32	4.67	4.82	4.94	4.71	4.05	0.69	4.83	0.11	1.19	0.251	1.3E-01
Mtr.33922.1.S1_s_at	6.88	5.54	6.48	6.21	6.73	6.70	6.30	0.69	6.55	0.29	1.04	0.056	5.9E-01
Mtr.32235.1.S1_at	8.48	7.13	8.00	8.35	8.08	8.48	7.87	0.69	8.30	0.20	1.05	0.077	3.6E-01
Mtr.33591.1.S1_at	8.60	7.42	8.63	8.56	8.60	8.55	8.22	0.69	8.57	0.03	1.04	0.061	4.2E-01
Mtr.34994.1.S1_at	7.68	6.57	7.83	7.40	7.76	7.63	7.36	0.69	7.59	0.18	1.03	0.045	6.0E-01
Mtr.5933.1.S1_at	7.83	6.61	7.76	7.97	8.02	7.80	7.40	0.69	7.93	0.12	1.07	0.101	2.5E-01
Mtr.21397.1.S1_at	8.13	7.03	8.29	8.22	7.97	8.06	7.81	0.69	8.08	0.13	1.03	0.049	5.4E-01
Mtr.33251.1.S1_at	7.67	6.32	7.22	7.52	7.34	7.42	7.07	0.69	7.43	0.09	1.05	0.071	4.2E-01
Mtr.9827.1.S1_at	11.24	10.32	9.90	10.57	10.53	10.79	10.48	0.69	10.63	0.14	1.01	0.020	7.4E-01
Mtr.2874.1.S1_at	6.37	5.50	6.85	6.59	6.90	6.59	6.24	0.69	6.69	0.18	1.07	0.100	3.4E-01
Mtr.21646.1.S1_at	12.65	11.35	11.62	12.45	13.47	13.15	11.87	0.69	13.02	0.52	1.10	0.133	8.2E-02
Mtr.11558.1.S1_at	12.36	10.99	11.74	11.95	12.03	12.04	11.69	0.69	12.01	0.05	1.03	0.038	4.7E-01
Mtr.10562.1.S1_at	6.98	6.05	7.38	5.68	5.83	5.78	6.80	0.69	5.76	0.08	0.85	-0.239	5.9E-02
Mtr.17308.1.S1_at	10.60	9.25	9.74	10.18	9.79	10.26	9.86	0.69	10.08	0.25	1.02	0.031	6.4E-01
Mtr.45041.1.S1_at	7.81	6.45	7.32	7.46	7.43	7.33	7.20	0.69	7.41	0.07	1.03	0.042	6.2E-01
Mtr.50948.1.S1_at	11.67	10.66	10.36	10.41	10.32	10.38	10.90	0.69	10.37	0.05	0.95	-0.072	2.5E-01
Mtr.35303.1.S1_at	6.89	5.70	6.89	6.87	7.35	6.73	6.49	0.69	6.98	0.33	1.08	0.105	3.3E-01

Mtr.39815.1.S1_at	6.13	4.92	6.08	5.91	6.51	6.08	5.71	0.69	6.17	0.31	1.08	0.110	3.6E-01
Mtr.11471.1.S1_at	7.74	6.37	7.14	7.68	7.80	7.59	7.09	0.69	7.69	0.11	1.08	0.117	2.1E-01
Mtr.15805.1.S1_at	6.13	5.10	6.40	6.05	6.22	6.11	5.88	0.68	6.12	0.09	1.04	0.060	5.7E-01
Mtr.48392.1.S1_at	2.07	3.25	2.06	2.06	2.05	2.06	2.46	0.68	2.05	0.00	0.83	-0.261	3.6E-01
Mtr.45551.1.S1_at	8.98	8.49	9.84	9.10	8.94	8.61	9.10	0.68	8.89	0.25	0.98	-0.034	6.4E-01
Mtr.3611.1.S1_at	3.92	2.99	4.33	3.93	4.48	3.68	3.75	0.68	4.03	0.41	1.08	0.106	5.7E-01
Mtr.39873.1.S1_s_at	8.47	7.13	7.54	8.60	8.93	8.71	7.71	0.68	8.75	0.17	1.13	0.181	6.4E-02
Mtr.1493.1.S1_at	12.25	13.09	11.73	12.61	12.74	12.61	12.36	0.68	12.65	0.07	1.02	0.034	5.0E-01
Mtr.11408.1.S1_at	7.39	6.71	8.08	7.44	7.05	7.01	7.39	0.68	7.17	0.24	0.97	-0.045	6.2E-01
Mtr.38447.1.S1_at	11.66	10.53	11.77	11.81	11.44	11.65	11.32	0.68	11.63	0.18	1.03	0.039	4.9E-01
Mtr.44209.1.S1_at	2.85	3.02	4.11	5.53	3.87	5.29	3.33	0.68	4.90	0.89	1.47	0.558	7.3E-02
Mtr.13369.1.S1_s_at	8.02	6.77	7.89	7.89	7.90	7.89	7.56	0.68	7.89	0.01	1.04	0.063	4.4E-01
Mtr.51830.1.S1_at	3.49	4.63	3.40	3.59	3.59	3.58	3.84	0.68	3.58	0.01	0.93	-0.099	5.5E-01
Mtr.32534.1.S1_s_at	8.22	7.36	8.71	8.59	8.83	8.57	8.10	0.68	8.66	0.15	1.07	0.098	2.3E-01
Mtr.12447.1.S1_at	13.63	12.27	13.07	13.06	13.16	13.13	12.99	0.68	13.11	0.05	1.01	0.013	7.8E-01
Mtr.5406.1.S1_at	4.43	3.07	3.67	3.80	3.68	3.64	3.72	0.68	3.71	0.08	1.00	-0.006	9.7E-01
Mtr.32412.1.S1_at	6.96	5.85	7.10	6.98	6.84	6.82	6.64	0.68	6.88	0.09	1.04	0.052	5.7E-01
Mtr.17734.1.S1_at	4.03	2.93	2.77	2.77	2.82	2.83	3.24	0.68	2.81	0.03	0.87	-0.208	3.3E-01
Mtr.8618.1.S1_at	14.29	12.93	13.70	13.79	13.33	13.78	13.64	0.68	13.63	0.26	1.00	-0.001	9.9E-01
Mtr.45685.1.S1_at	3.91	4.86	3.54	3.56	3.51	3.50	4.10	0.68	3.52	0.03	0.86	-0.220	2.2E-01
Mtr.46880.1.S1_at	2.61	3.79	2.60	2.59	2.59	2.59	3.00	0.68	2.59	0.00	0.86	-0.210	3.6E-01
Mtr.35920.1.S1_s_at	8.98	7.71	8.79	8.84	8.65	8.65	8.49	0.68	8.71	0.11	1.03	0.037	6.1E-01
Mtr.47479.1.S1_at	6.31	5.16	6.38	6.71	6.59	6.40	5.95	0.68	6.57	0.16	1.10	0.143	2.0E-01
Mtr.5884.1.S1_s_at	5.15	4.02	5.25	4.49	5.24	5.31	4.81	0.68	5.01	0.45	1.04	0.061	6.8E-01
Mtr.32783.1.S1_at	8.99	7.69	8.70	9.06	8.95	9.27	8.46	0.68	9.09	0.17	1.07	0.104	1.9E-01
Mtr.6293.1.S1_at	8.38	7.39	8.70	8.62	8.66	8.34	8.15	0.68	8.54	0.18	1.05	0.066	4.0E-01
Mtr.45274.1.S1_at	7.89	6.58	7.54	7.73	7.58	7.53	7.34	0.68	7.61	0.10	1.04	0.053	5.3E-01
Mtr.5386.1.S1_at	8.04	6.76	7.81	7.96	7.70	7.91	7.53	0.68	7.86	0.14	1.04	0.060	4.7E-01
Mtr.12264.1.S1_s_at	5.16	4.29	5.63	4.65	6.56	5.76	5.03	0.68	5.66	0.96	1.13	0.170	4.1E-01
Mtr.37867.1.S1_at	12.02	10.68	11.09	11.49	11.53	11.59	11.26	0.68	11.54	0.05	1.02	0.035	5.3E-01
Mtr.9849.1.S1_at	7.28	6.23	7.51	7.48	7.78	7.59	7.01	0.68	7.61	0.15	1.09	0.120	2.1E-01
Mtr.47389.1.S1_at	3.78	3.85	4.99	3.76	3.65	3.35	4.21	0.68	3.59	0.21	0.85	-0.230	2.1E-01
Mtr.2340.1.S1_s_at	6.38	5.07	6.05	5.51	6.05	6.31	5.83	0.68	5.95	0.41	1.02	0.030	8.1E-01
Mtr.13781.1.S1_at	8.89	7.66	7.76	8.21	8.25	8.30	8.11	0.68	8.25	0.04	1.02	0.026	7.3E-01
Mtr.44174.1.S1_at	10.20	9.18	10.47	10.50	10.63	10.53	9.95	0.68	10.55	0.06	1.06	0.085	2.0E-01
Mtr.47478.1.S1_at	7.54	6.18	6.98	8.40	8.16	8.24	6.90	0.68	8.27	0.12	1.20	0.261	2.7E-02
Mtr.44740.1.S1_at	9.44	8.08	8.88	9.20	8.81	8.96	8.80	0.68	8.99	0.20	1.02	0.031	6.6E-01
Mtr.30126.1.S1_at	4.01	2.66	3.14	3.04	2.95	3.03	3.27	0.68	3.01	0.05	0.92	-0.119	5.5E-01
Mtr.36873.1.S1_at	8.66	7.70	9.02	8.95	9.08	9.01	8.46	0.68	9.01	0.07	1.07	0.092	2.3E-01
Mtr.26166.1.S1_at	9.19	8.12	7.93	9.10	8.93	9.26	8.42	0.68	9.10	0.17	1.08	0.112	1.7E-01
Mtr.35706.1.S1_at	8.61	7.92	9.28	8.70	9.09	8.82	8.61	0.68	8.87	0.20	1.03	0.044	5.5E-01
Mtr.7426.1.S1_s_at	8.23	7.21	8.51	8.22	8.47	8.46	7.99	0.68	8.38	0.14	1.05	0.070	3.8E-01
Mtr.40302.1.S1_at	11.65	10.29	11.05	11.22	10.69	11.15	10.99	0.68	11.02	0.28	1.00	0.003	9.5E-01
Mtr.27356.1.S1_at	2.95	2.87	4.09	2.83	3.73	2.90	3.30	0.68	3.15	0.50	0.95	-0.068	7.7E-01
Mtr.35038.1.S1_at	8.03	6.85	8.03	8.06	8.31	8.30	7.64	0.68	8.22	0.14	1.08	0.106	2.2E-01
Mtr.16367.1.S1_at	5.30	4.20	5.45	5.52	5.79	5.17	4.99	0.68	5.49	0.31	1.10	0.140	3.0E-01
Mtr.38289.1.S1_at	9.38	8.10	9.13	9.31	9.30	9.28	8.87	0.68	9.30	0.01	1.05	0.068	3.4E-01
Mtr.7247.1.S1_at	11.58	10.22	10.93	11.19	11.16	11.19	10.91	0.68	11.18	0.02	1.02	0.035	5.3E-01
Mtr.52115.1.S1_s_at	5.89	4.53	5.27	5.33	4.96	5.25	5.23	0.68	5.18	0.20	0.99	-0.014	9.1E-01
Mtr.32220.1.S1_at	7.63	6.44	7.62	8.09	8.24	8.01	7.23	0.68	8.11	0.12	1.12	0.166	9.2E-02
Mtr.18505.1.S1_at	2.38	3.55	2.36	2.34	2.34	2.35	2.76	0.68	2.35	0.00	0.85	-0.235	3.5E-01
Mtr.33854.1.S1_at	4.08	2.98	2.84	2.89	2.70	3.37	3.30	0.68	2.99	0.35	0.90	-0.144	5.2E-01
Mtr.32490.1.S1_at	4.71	4.83	5.95	4.75	5.28	5.29	5.16	0.68	5.11	0.31	0.99	-0.016	9.0E-01
Mtr.2890.1.S1_at	6.61	5.43	6.61	6.42	6.30	6.43	6.22	0.68	6.38	0.07	1.03	0.038	6.9E-01
Mtr.12248.1.S1_at	12.22	11.68	13.04	12.67	12.25	12.23	12.31	0.68	12.38	0.25	1.01	0.008	8.7E-01
Mtr.44128.1.S1_at	8.69	7.82	9.17	8.90	9.15	9.04	8.56	0.68	9.03	0.13	1.06	0.078	3.0E-01
Mtr.50380.1.S1_at	8.04	6.68	7.41	8.61	8.55	8.57	7.38	0.68	8.58	0.03	1.16	0.217	3.8E-02
Mtr.6151.1.S1_at	9.57	10.56	10.87	9.91	10.51	10.06	10.33	0.68	10.16	0.31	0.98	-0.025	7.1E-01
Mtr.35720.1.S1_at	3.85	2.49	3.03	3.83	4.39	4.30	3.12	0.68	4.17	0.30	1.34	0.419	7.1E-02
Mtr.33086.1.S1_at	8.39	7.09	8.07	8.05	8.02	8.15	7.85	0.68	8.07	0.07	1.03	0.040	6.0E-01
Mtr.12917.1.S1_at	7.26	7.37	6.14	6.05	5.98	6.20	6.92	0.68	6.08	0.11	0.88	-0.189	1.0E-01
Mtr.44192.1.S1_at	9.52	8.28	9.40	9.27	9.68	9.36	9.07	0.68	9.44	0.21	1.04	0.058	4.2E-01
Mtr.38226.1.S1_at	9.04	8.45	9.81	9.21	9.35	9.25	9.10	0.68	9.27	0.07	1.02	0.027	6.9E-01
Mtr.19019.1.S1_at	12.87	11.94	11.55	11.86	12.07	12.05	12.12	0.68	11.99	0.11	0.99	-0.015	7.7E-01
Mtr.49013.1.S1_at	7.17	5.98	7.15	7.15	7.21	7.15	6.77	0.68	7.17	0.04	1.06	0.083	3.7E-01
Mtr.25570.1.S1_at	8.39	9.71	8.75	8.48	7.90	8.15	8.95	0.68	8.18	0.29	0.91	-0.130	1.4E-01
Mtr.39258.1.S1_at	8.03	6.70	7.58	7.06	6.56	6.84	7.44	0.68	6.82	0.25	0.92	-0.125	2.1E-01
Mtr.23410.1.S1_at	9.09	7.85	8.97	8.89	8.91	8.77	8.64	0.68	8.85	0.08	1.03	0.036	6.1E-01
Mtr.44462.1.S1_at	10.36	9.21	9.15	10.06	9.77	9.86	9.57	0.68	9.90	0.15	1.03	0.048	4.7E-01
Mtr.7074.1.S1_at	6.36	5.68	7.04	6.44	7.03	6.23	6.36	0.68	6.57	0.41	1.03	0.047	6.7E-01
Mtr.36170.1.S1_at	6.17	5.09	6.34	5.88	6.25	6.06	5.86	0.68	6.06	0.18	1.03	0.047	6.5E-01
Mtr.27909.1.S1_at	7.28	6.03	7.13	7.18	7.01	7.13	6.81	0.68	7.11	0.09	1.04	0.061	5.0E-01
Mtr.18576.1.S1_s_at	9.75	8.40	9.24	9.33	9.15	9.24	9.13	0.68	9.24	0.09	1.01	0.017	8.0E-01
Mtr.9592.1.S1_at	4.02	2.86	2.83	2.82	2.82	2.78	3.24	0.68	2.81	0.03	0.87	-0.205	3.4E-01
Mtr.50943.1.S1_at	7.27	5.93	6.82	6.88	6.68	6.95	6.67	0.68	6.84	0.14	1.02	0.035	7.1E-01
Mtr.12010.1.S1_at	4.69	3.65	4.94	3.89	3.86	3.72	4.42	0.68	3.82	0.09	0.86	-0.211	2.0E-01
Mtr.27041.1.S1_at	2.34	3.51	2.33	2.33	2.32	2.32	2.73	0.68	2.32	0.00	0.85	-0.231	3.6E-01
Mtr.32917.1.S1_s_at	7.80	6.48	7.44	7.35	7.60	7.26	7.24	0.68	7.40	0.18	1.02	0.032	7.1E-01
Mtr.27398.1.S1_at	7.74	6.88	8.23	8.03	8.12	7.82	7.62	0.68	7.99	0.16	1.05	0.069	4.0E-01
Mtr.16049.1.S1_at	10.92	9.57	10.11	10.66	10.90	11.11	10.20	0.68	10.89	0.23	1.07	0.095	1.7E-01
Mtr.27845.1.S1_at	2.79	1.85	3.17	2.79	3.52	2.86	2.60	0.68	3.06	0.40	1.17	0.232	3.7E-01
Mtr.39910.1.S1_at	8.12	7.64	6.78	7.78	7.89	7.83	7.51	0.68	7.83	0.06	1.04	0.060	4.6E-01
Mtr.33147.1.S1_at	9.20	10.54	9.68	8.86	9.52	8.96	9.81	0.68	9.11	0.35	0.93	-0.106	1.9E-01
Mtr.19370.1.S1_at	9.45	8.15	9.14	8.95	8.95	8.78	8.91	0.68	8.89	0.10	1.00	-0.003	9.7E-01
Mtr.25007.1.S1_at	7.33	5.97	6.63	6.81	6.12	6.89	6.64	0.68	6.61	0.42	0.99	-0.008	9.4E-01
Mtr.14074.1.S1_at	8.78	7.65	8.87	9.04	9.14	8.97	8.43	0.68	9.05	0.08	1.07	0.103	1.9E-01
Mtr.3044.1.S1_at	9.95	8.59	9.21	9.28	8.75	9.14	9.25	0.68	9.06	0.27	0.98	-0.030	6.7E-01
Mtr.32505.1.S1_at	7.87	6.75	7.98	7.10	6.95	7.08	7.54	0.68	7.04	0.08	0.93	-0.098	2.8E-01
Mtr.28796.1.S1_at	2.99	4.20	3.06	3.31	3.04	2.97	3.42	0.68	3.11	0.18	0.91	-0.137	4.9E-01
Mtr.11304.1.S1_at	9.75	8.51	9.61	9.68	9.43	9.68	9.29	0.68	9.59	0.15	1.03	0.047	4.9E-01
Mtr.3698.1.S1_at	6.03	4.92	6.15	6.39	6.13	5.50	5.70	0.68	6.00	0.46	1.05	0.075	5.5E-01
Mtr.44568.1.S1_at	9.31	7.96	8.78	9.02	8.89	8.90	8.68	0.68	8.94	0.07	1.03	0.042	5.5E-01
Mtr.39303.1.S1_at	7.37	6.36	7.65	7.30	7.45	7.44	7.13	0.68	7.40	0.08	1.04	0.053	5.3E-01
Mtr.8460.1.S1_s_at	6.90	5.64	6.70	7.79	8.05	7.97	6.41	0.68	7.94	0.13	1.24	0.308	1.9E-02
Mtr.36378.1.S1_at	12.50	11.31	11.34	11.70	12.29	12.10	11.72	0.68	12.03	0.30	1.03	0.038	5.0E-01
Mtr.47270.1.S1_at	10.36	9.59	10.94	10.75	10.63	10.51	10.29	0.68	10.63	0.12	1.03	0.046	4.5E-01
Mtr.42772.1.S1_at	8.99	7.86	9.08	8.76	8.94	8.78	8.64	0.68	8.83	0.10	1.02	0.031	6.6E-01
Mtr.33818.1.S1_at	8.87	7.75	8.97	8.86	8.81	8.74	8.53	0.68	8.80	0.06	1.03	0.046	5.2E-01
Mtr.22960.1.S1_at	3.97	3.48	2.63	2.51	3.18	3.44	3.36	0.68	3.04	0.48	0.91	-0.143	5.5E-01
Mtr.18197.1.S1_at	2.96	4.15	3.00	2.94	2.89	2.92	3.37	0.68	2.92	0.03	0.87	-0.207	3.1E-01
Mtr.46772.1.S1_at	2.63	3.80	2.62	2.57	2.61	2.60	3.01	0.68	2.60	0.02	0.86	-0.215	3.5E-01
Mtr.44548.1.S1_at	10.68	9.32	10.03	10.10	10.06	10.22	10.01	0.68	10.13	0.09	1.01	0.017	7.8E-01
Mtr.27707.1.S1_s_at	5.47	4.37	5.60	5.39	6.10	5.45	5.15	0.68	5.65	0.40	1.10	0.133	3.4E-01
Mtr.1892.1.S1_at	9.47	8.36	9.59	9.27	9.61	9.30	9.14	0.68	9.39	0.19	1.03	0.040	5.6E-01
Mtr.39824.1.S1_at	9.52	8.53	9.82	8.96	9.37	9.08	9.29	0.68	9.14	0.21	0.98	-0.024	7.3E-01
Mtr.32418.1.S1_s_at	4.43	3.24	4.39	4.07	4.52	4.51	4.02	0.68	4.37	0.26	1.09	0.120	4.5E-01
Mtr.15457.1.S1_at	6.80	6.08	7.44	7.20	7.21	7.37	6.77	0.68	7.26	0.10	1.07	0.100	2.9E-01
Mtr.20528.1.S1_at	2.79	3.94	2.74	2.74	2.75	2.74	3.16	0.68	2.74	0.01	0.87	-0.202	3.5E-01
Mtr.11154.1.S1_at	8.35	7.15	8.29	8.21	8.45	8.53	7.93	0.68	8.40	0.17	1.06	0.082	3.2E-01
Mtr.44100.1.S1_at	13.53	12.21	12.60	12.92	12.57	13.10	12.78	0.68	12.86	0.27	1.01	0.009	8.6E-01
Mtr.45077.1.S1_at	3.94	2.75	3.90	3.90	4.58	3.91	3.53	0.68	4.13	0.39	1.17	0.226	2.6E-01
Mtr.35771.1.S1_s_at	9.16	7.82	8.65	8.97	8.66	8.87	8.55	0.68	8.83	0.16	1.03	0.048	5.1E-01
Mtr.22007.1.S1_at	12.17	10.86	11.20	10.98	10.38	10.95	11.41	0.68	10.77	0.33	0.94	-0.083	2.2E-01
Mtr.40006.1.S1_at	3.43	3.26	4.51	4.10	4.17	3.81	3.73	0.68	4.02	0.19	1.08	0.109	5.1E-01
Mtr.24772.1.S1_s_at	5.43	4.19	5.28	5.28	5.27	5.27	4.97	0.68	5.27	0.00	1.06	0.086	4.8E-01
Mtr.4757.1.S1_at	2.90	4.03	2.82	2.90	2.81	2.90	3.25	0.68	2.87	0.05	0.88	-0.182	3.8E-01
Mtr.29268.1.S1_at	5.56	4.24	5.16	4.66	5.59	5.45	4.98	0.68	5.23	0.50	1.05	0.071	6.4E-01
Mtr.38833.1.S1_at	7.79	6.57	7.69	7.80	7.73	7.82	7.35	0.68	7.78	0.05	1.06	0.083	3.3E-01
Mtr.45532.1.S1_at	6.50	5.42	6.66	6.43	6.49	6.58	6.19	0.68	6.50	0.08	1.05	0.070	4.8E-01
Mtr.47295.1.S1_x_at	13.25	13.30	12.10	13.02	13.11	13.24	12.88	0.68	13.13	0.11	1.02	0.027	5.7E-01
Mtr.32824.1.S1_at	7.36	6.37	7.66	7.67	7.86	7.81	7.13	0.68	7.78	0.10	1.09	0.126	1.8E-01
Mtr.9503.1.S1_at	4.88	3.53	4.17	4.76	3.47	3.46	4.20	0.68	3.90	0.75	0.93	-0.107	6.3E-01
Mtr.51381.1.S1_at	9.18	7.85	8.33	8.65	8.66	8.80	8.45	0.68	8.71	0.08	1.03	0.043	5.5E-01
Mtr.9931.1.S1_at	10.67	9.67	10.96	10.92	10.86	10.71	10.43	0.68	10.83	0.11	1.04	0.054	3.7E-01
Mtr.2061.1.S1_at	7.98	6.84	8.04	7.97	7.72	7.83	7.62	0.68	7.84	0.12	1.03	0.042	6.0E-01
Mtr.27339.1.S1_at	8.12	7.08	8.34	8.31	8.33	8.32	7.85	0.68	8.32	0.01	1.06	0.085	2.9E-01
Mtr.10864.1.S1_at	8.35	7.14	8.26	8.30	8.17	8.26	7.92	0.68	8.24	0.07	1.04	0.058	4.5E-01
Mtr.33124.1.S1_at	10.24	8.89	9.57	9.98	9.69	10.08	9.57	0.68	9.92	0.20	1.04	0.052	4.4E-01
Mtr.21886.1.S1_at	5.19	4.03	5.22	4.83	5.40	5.40	4.81	0.68	5.21	0.33	1.08	0.114	4.1E-01
Mtr.49572.1.S1_x_at	11.93	10.59	11.39	11.44	11.10	11.40	11.30	0.67	11.32	0.18	1.00	0.002	9.8E-01
Mtr.8005.1.S1_at	2.19	3.35	2.18	2.18	2.18	2.13	2.57	0.67	2.16	0.03	0.84	-0.253	3.5E-01

Mtr.40021.1.S1_at	5.02	5.23	3.97	5.65	6.93	6.76	4.74	0.67	6.44	0.70	1.36	0.443	3.8E-02
Mtr.38555.1.S1_at	7.27	6.16	7.37	7.43	7.64	7.33	6.93	0.67	7.46	0.16	1.08	0.106	2.6E-01
Mtr.43770.1.S1_at	12.75	11.67	11.51	12.04	12.20	12.21	11.98	0.67	12.15	0.09	1.01	0.021	6.8E-01
Mtr.28976.1.S1_at	7.28	6.67	8.01	7.33	7.32	7.28	7.32	0.67	7.31	0.03	1.00	-0.002	9.8E-01
Mtr.35835.1.S1_at	7.21	6.06	7.24	6.83	7.22	7.02	6.84	0.67	7.02	0.20	1.03	0.039	6.7E-01
Mtr.31102.1.S1_at	7.66	8.06	8.98	8.35	8.72	8.30	8.23	0.67	8.46	0.23	1.03	0.038	6.2E-01
Mtr.35470.1.S1_at	3.45	2.28	3.45	4.04	3.21	3.64	3.06	0.67	3.63	0.42	1.19	0.247	2.8E-01
Mtr.47062.1.S1_at	2.77	3.93	2.75	2.74	2.86	2.74	3.15	0.67	2.78	0.07	0.88	-0.182	3.9E-01
Mtr.35603.1.S1_at	3.73	2.60	3.80	4.33	5.29	4.49	3.38	0.67	4.70	0.51	1.39	0.478	5.4E-02
Mtr.21417.1.S1_at	6.66	5.75	7.07	7.20	7.00	6.89	6.49	0.67	7.03	0.16	1.08	0.115	2.5E-01
Mtr.36995.1.S1_at	10.08	8.84	9.92	9.52	9.61	9.55	9.62	0.67	9.56	0.05	0.99	-0.008	9.0E-01
Mtr.12320.1.S1_at	7.73	6.47	7.52	7.75	7.96	8.07	7.24	0.67	7.93	0.16	1.09	0.130	1.6E-01
Mtr.6859.1.S1_at	6.54	5.22	5.64	6.05	6.30	6.55	5.80	0.67	6.30	0.25	1.09	0.120	2.9E-01
Mtr.32705.1.S1_at	8.20	7.32	8.64	8.32	8.30	8.19	8.05	0.67	8.27	0.07	1.03	0.038	6.2E-01
Mtr.3278.1.S1_at	2.50	3.65	2.47	2.46	2.46	2.45	2.87	0.67	2.45	0.01	0.85	-0.227	3.4E-01
Mtr.38366.1.S1_at	11.62	10.51	10.40	10.72	10.28	10.71	10.84	0.67	10.57	0.25	0.97	-0.037	5.4E-01
Mtr.51905.1.S1_at	2.41	3.57	2.39	2.40	2.38	2.39	2.79	0.67	2.39	0.01	0.86	-0.225	3.6E-01
Mtr.2704.1.S1_at	8.13	7.01	8.22	8.31	8.54	8.23	7.79	0.67	8.36	0.16	1.07	0.102	2.3E-01
Mtr.41000.1.S1_at	9.45	8.12	8.97	8.67	9.00	8.99	8.85	0.67	8.88	0.19	1.00	0.006	9.3E-01
Mtr.36908.1.S1_at	5.49	4.29	5.42	5.63	5.38	5.36	5.07	0.67	5.46	0.15	1.08	0.107	3.8E-01
Mtr.41963.1.S1_at	5.36	4.28	5.52	5.28	5.30	5.38	5.05	0.67	5.32	0.05	1.05	0.074	5.3E-01
Mtr.30850.1.S1_at	6.69	5.73	7.03	6.40	6.98	6.93	6.48	0.67	6.77	0.32	1.04	0.062	5.5E-01
Mtr.10632.1.S1_at	9.37	8.19	9.33	9.17	9.55	9.21	8.96	0.67	9.31	0.21	1.04	0.055	4.4E-01
Mtr.50164.1.S1_at	5.27	4.47	3.93	5.90	5.61	5.77	4.56	0.67	5.76	0.14	1.26	0.338	3.9E-02
Mtr.27254.1.S1_at	3.30	2.39	3.71	2.64	4.00	3.25	3.13	0.67	3.30	0.68	1.05	0.073	7.8E-01
Mtr.33161.1.S1_at	8.28	6.97	7.89	7.68	7.83	7.88	7.72	0.67	7.80	0.10	1.01	0.015	8.5E-01
Mtr.12042.1.S1_at	7.39	6.18	7.30	7.27	8.10	7.76	6.96	0.67	7.71	0.42	1.11	0.147	1.8E-01
Mtr.42684.1.S1_at	7.20	5.95	6.99	6.97	6.82	6.96	6.71	0.67	6.92	0.08	1.03	0.043	6.3E-01
Mtr.10280.1.S1_at	4.13	2.82	3.73	3.72	4.05	3.82	3.56	0.67	3.86	0.17	1.09	0.119	4.9E-01
Mtr.21322.1.S1_s_at	6.08	4.77	5.70	7.31	7.21	5.73	5.52	0.67	6.75	0.88	1.22	0.291	1.3E-01
Mtr.3768.1.S1_at	9.71	8.37	9.20	9.17	9.37	9.23	9.09	0.67	9.26	0.10	1.02	0.026	7.0E-01
Mtr.11620.1.S1_at	9.13	7.93	8.01	8.18	7.68	8.07	8.36	0.67	7.97	0.26	0.95	-0.068	4.1E-01
Mtr.43567.1.S1_at	12.28	11.03	11.23	11.46	11.18	11.56	11.51	0.67	11.40	0.20	0.99	-0.014	7.9E-01
Mtr.25211.1.S1_s_at	9.64	10.74	9.51	8.86	10.04	9.88	9.96	0.67	9.60	0.64	0.96	-0.055	5.3E-01
Mtr.5463.1.S1_at	8.34	7.09	8.15	8.35	8.35	8.40	7.86	0.67	8.36	0.03	1.06	0.089	2.7E-01
Mtr.18338.1.S1_at	4.70	3.53	4.70	6.28	6.22	6.13	4.31	0.67	6.21	0.08	1.44	0.527	8.2E-03
Mtr.6557.1.S1_at	7.69	6.47	7.59	7.56	7.64	7.42	7.25	0.67	7.54	0.11	1.04	0.057	5.0E-01
Mtr.42778.1.S1_at	7.09	5.87	6.98	7.02	6.97	6.75	6.65	0.67	6.92	0.14	1.04	0.057	5.3E-01
Mtr.52307.1.S1_at	2.61	1.94	3.28	2.43	2.51	2.32	2.61	0.67	2.42	0.10	0.93	-0.109	6.5E-01
Mtr.41748.1.S1_at	8.18	6.91	7.92	7.79	8.06	7.88	7.67	0.67	7.91	0.14	1.03	0.044	5.8E-01
Mtr.29763.1.S1_at	3.73	3.16	4.50	4.16	4.54	3.52	3.80	0.67	4.07	0.52	1.07	0.102	6.0E-01
Mtr.13659.1.S1_at	10.59	9.24	9.87	10.07	9.92	10.12	9.90	0.67	10.04	0.10	1.01	0.020	7.5E-01
Mtr.10727.1.S1_at	7.28	7.56	8.55	7.84	7.49	7.64	7.80	0.67	7.66	0.17	0.98	-0.026	7.4E-01
Mtr.32556.1.S1_at	5.68	4.34	5.11	5.57	5.25	5.25	5.04	0.67	5.36	0.19	1.06	0.088	4.8E-01
Mtr.21355.1.S1_x_at	2.30	3.45	2.27	2.27	2.27	2.26	2.67	0.67	2.27	0.01	0.85	-0.238	3.5E-01
Mtr.33242.1.S1_at	6.82	6.15	7.49	7.06	6.90	7.16	6.82	0.67	7.04	0.13	1.03	0.046	6.1E-01
Mtr.26834.1.S1_at	9.03	7.84	8.97	8.91	8.93	8.95	8.61	0.67	8.93	0.02	1.04	0.053	4.5E-01
Mtr.32351.1.S1_at	4.35	3.42	4.72	4.56	4.80	4.33	4.16	0.67	4.57	0.23	1.10	0.134	3.8E-01
Mtr.7202.1.S1_at	7.54	6.23	7.13	6.99	7.43	7.17	6.97	0.67	7.20	0.22	1.03	0.046	6.1E-01
Mtr.9477.1.S1_at	7.82	6.57	7.63	7.53	8.17	7.72	7.34	0.67	7.81	0.33	1.06	0.089	3.4E-01
Mtr.5415.1.S1_s_at	7.87	6.74	7.94	7.86	7.67	7.82	7.52	0.67	7.78	0.10	1.04	0.050	5.4E-01
Mtr.34976.1.S1_at	4.95	4.09	5.41	4.94	4.85	5.25	4.81	0.67	5.01	0.21	1.04	0.057	6.6E-01
Mtr.5269.1.S1_at	4.75	5.48	4.14	4.64	4.64	4.64	4.79	0.67	4.64	0.00	0.97	-0.047	7.1E-01
Mtr.38972.1.S1_at	2.98	3.39	4.29	3.80	4.90	3.81	3.55	0.67	4.17	0.63	1.17	0.231	3.1E-01
Mtr.23329.1.S1_at	4.26	3.28	4.56	6.43	6.61	5.23	4.03	0.67	6.09	0.75	1.51	0.594	2.4E-02
Mtr.44402.1.S1_at	9.99	8.65	9.36	9.46	9.40	9.69	9.33	0.67	9.52	0.15	1.02	0.028	6.7E-01
Mtr.38291.1.S1_at	8.48	8.43	9.62	8.66	8.82	8.72	8.84	0.67	8.73	0.08	0.99	-0.018	7.9E-01
Mtr.26210.1.S1_at	8.26	6.93	7.75	8.53	8.63	8.60	7.65	0.67	8.59	0.06	1.12	0.166	7.4E-02
Mtr.11730.1.S1_at	8.63	7.45	8.60	8.53	8.75	8.70	8.23	0.67	8.66	0.11	1.05	0.075	3.3E-01
Mtr.6659.1.S1_at	7.56	6.43	7.62	7.38	7.56	7.09	7.20	0.67	7.35	0.24	1.02	0.028	7.4E-01
Mtr.9989.1.S1_at	2.60	3.75	2.57	2.55	2.51	2.56	2.97	0.67	2.54	0.03	0.85	-0.230	3.2E-01
Mtr.32182.1.S1_at	6.05	5.19	4.73	4.86	4.95	4.78	5.32	0.67	4.87	0.08	0.91	-0.130	3.1E-01
Mtr.2167.1.S1_at	4.00	2.66	3.37	4.07	4.23	4.30	3.34	0.67	4.20	0.12	1.26	0.329	9.5E-02
Mtr.14925.1.S1_at	7.20	6.06	7.23	7.49	7.02	6.95	6.83	0.67	7.15	0.29	1.05	0.066	4.9E-01
Mtr.34970.1.S1_at	4.31	3.72	5.05	4.79	5.40	3.87	4.36	0.67	4.68	0.77	1.07	0.104	6.1E-01
Mtr.43381.1.S1_at	5.61	4.36	5.42	5.30	5.37	5.37	5.13	0.67	5.35	0.04	1.04	0.061	6.0E-01
Mtr.33351.1.S1_at	6.26	5.18	6.41	5.25	6.47	6.37	5.95	0.67	6.03	0.67	1.01	0.019	8.9E-01
Mtr.50244.1.S1_x_at	2.86	4.20	3.53	2.73	2.92	2.92	3.53	0.67	2.86	0.11	0.81	-0.303	1.6E-01
Mtr.9776.1.S1_at	7.64	7.03	8.37	8.06	8.43	8.00	7.68	0.67	8.17	0.23	1.06	0.088	3.0E-01
Mtr.4395.1.S1_at	2.31	3.47	2.30	2.30	2.49	2.29	2.69	0.67	2.36	0.11	0.88	-0.190	4.4E-01
Mtr.32794.1.S1_at	8.47	7.17	8.08	8.27	8.26	8.26	7.91	0.67	8.26	0.00	1.05	0.064	4.1E-01
Mtr.7106.1.S1_at	5.84	4.50	5.25	5.38	5.76	5.12	5.20	0.67	5.42	0.32	1.04	0.061	6.3E-01
Mtr.9770.1.S1_at	4.06	2.74	3.17	3.18	3.17	3.18	3.32	0.67	3.18	0.00	0.96	-0.066	7.2E-01
Mtr.38004.1.S1_s_at	8.91	7.57	8.29	8.28	8.44	8.37	8.25	0.67	8.37	0.08	1.01	0.019	7.9E-01
Mtr.41455.1.S1_at	9.46	8.21	9.25	9.52	9.60	9.84	8.97	0.67	9.65	0.17	1.08	0.105	1.6E-01
Mtr.50823.1.S1_at	2.54	3.69	2.52	2.52	2.50	2.49	2.91	0.67	2.50	0.01	0.86	-0.219	3.5E-01
Mtr.21416.1.S1_at	11.07	10.65	9.76	9.49	9.56	9.37	10.49	0.67	9.47	0.09	0.90	-0.148	5.9E-02
Mtr.26654.1.S1_s_at	9.29	8.17	9.35	9.06	9.26	9.33	8.94	0.67	9.21	0.14	1.03	0.044	5.2E-01
Mtr.39352.1.S1_at	9.62	8.41	9.50	9.39	9.23	9.19	9.18	0.67	9.27	0.11	1.01	0.015	8.2E-01
Mtr.23365.1.S1_at	3.05	3.61	4.38	4.30	3.84	2.90	3.68	0.67	3.68	0.71	1.00	0.000	1.0E+00
Mtr.44861.1.S1_at	3.77	2.46	3.34	3.27	3.19	3.63	3.19	0.67	3.37	0.23	1.06	0.079	6.9E-01
Mtr.43848.1.S1_at	9.59	8.25	8.98	9.37	9.42	9.61	8.94	0.67	9.47	0.13	1.06	0.083	2.5E-01
Mtr.10950.1.S1_at	9.50	8.22	8.52	8.57	8.92	8.77	8.75	0.67	8.75	0.18	1.00	0.001	9.9E-01
Mtr.42829.1.S1_at	8.83	7.63	8.75	8.64	8.76	8.68	8.40	0.67	8.69	0.06	1.03	0.049	4.9E-01
Mtr.16292.1.S1_at	2.66	3.81	2.64	2.62	2.61	2.61	3.04	0.67	2.61	0.00	0.86	-0.217	3.3E-01
Mtr.42405.1.S1_at	8.79	7.68	8.88	9.17	8.98	8.94	8.45	0.67	9.03	0.12	1.07	0.095	2.2E-01
Mtr.15795.1.S1_at	4.76	4.60	5.83	6.80	5.37	7.01	5.06	0.67	6.39	0.89	1.26	0.337	1.1E-01
Mtr.6212.1.S1_at	2.22	3.37	2.21	2.20	2.20	2.20	2.60	0.67	2.20	0.00	0.85	-0.242	3.6E-01
Mtr.7145.1.S1_at	8.27	7.42	8.73	8.45	8.49	8.36	8.14	0.67	8.44	0.07	1.04	0.052	4.9E-01
Mtr.28251.1.S1_at	4.67	4.30	5.60	5.33	5.60	5.59	4.86	0.67	5.51	0.15	1.13	0.181	1.8E-01
Mtr.11920.1.S1_at	8.17	6.85	7.71	8.07	7.97	8.05	7.58	0.67	8.03	0.05	1.06	0.084	3.1E-01
Mtr.8815.1.S1_at	3.94	2.63	3.52	3.56	3.62	3.48	3.36	0.67	3.55	0.07	1.06	0.079	6.5E-01
Mtr.9621.1.S1_at	10.78	9.68	10.88	10.93	10.85	10.79	10.44	0.67	10.86	0.07	1.04	0.056	3.5E-01
Mtr.52010.1.S1_at	8.39	7.39	8.67	8.39	8.27	7.91	8.15	0.67	8.19	0.25	1.01	0.007	9.3E-01
Mtr.6783.1.S1_at	4.73	3.75	5.03	4.92	5.49	4.94	4.50	0.67	5.12	0.32	1.14	0.185	2.2E-01
Mtr.9221.1.S1_at	10.17	8.84	9.59	10.01	9.95	10.48	9.53	0.67	10.15	0.29	1.06	0.090	2.2E-01
Mtr.27390.1.S1_at	7.78	7.45	8.74	7.82	8.03	7.89	7.99	0.67	7.91	0.11	0.99	-0.014	8.5E-01
Mtr.42179.1.S1_at	4.38	3.21	4.37	4.38	4.42	4.37	3.99	0.67	4.39	0.03	1.10	0.140	3.5E-01
Mtr.43459.1.S1_at	11.00	9.70	10.64	10.93	10.90	10.75	10.45	0.67	10.86	0.09	1.04	0.056	3.5E-01
Mtr.1533.1.S1_at	4.79	3.46	4.18	3.48	3.61	3.42	4.14	0.67	3.50	0.10	0.85	-0.242	1.8E-01
Mtr.5718.1.S1_at	6.82	5.66	6.82	6.94	7.14	6.74	6.43	0.67	6.94	0.20	1.08	0.110	2.8E-01
Mtr.9725.1.S1_at	7.01	5.92	7.14	7.14	6.92	7.17	6.69	0.67	7.08	0.14	1.06	0.081	3.8E-01
Mtr.42682.1.S1_at	5.61	4.35	5.38	5.55	5.15	5.73	5.11	0.67	5.47	0.30	1.07	0.098	4.4E-01
Mtr.25726.1.S1_s_at	7.69	6.87	8.19	7.63	7.76	7.57	7.59	0.67	7.65	0.10	1.01	0.013	8.7E-01
Mtr.12008.1.S1_at	8.97	7.76	8.85	8.33	8.78	8.61	8.53	0.67	8.57	0.23	1.00	0.007	9.2E-01
Mtr.28605.1.S1_at	6.84	5.76	6.98	6.82	7.09	6.82	6.53	0.67	6.91	0.15	1.06	0.082	3.9E-01
Mtr.41042.1.S1_s_at	9.17	9.91	10.50	9.81	9.84	9.58	9.86	0.67	9.74	0.14	0.99	-0.017	7.8E-01
Mtr.44435.1.S1_at	8.61	7.28	7.84	8.07	7.92	7.99	7.91	0.67	7.99	0.07	1.01	0.016	8.3E-01
Mtr.35334.1.S1_s_at	9.25	10.13	8.82	8.69	8.48	8.66	9.40	0.67	8.61	0.11	0.92	-0.126	1.1E-01
Mtr.25112.1.S1_at	2.75	3.89	2.72	2.70	2.68	2.67	3.12	0.67	2.68	0.02	0.86	-0.218	3.2E-01
Mtr.23551.1.S1_at	11.76	10.62	10.58	10.82	10.41	10.85	10.99	0.67	10.69	0.25	0.97	-0.039	5.1E-01
Mtr.42148.1.S1_at	10.48	9.15	9.88	9.89	10.45	10.02	9.84	0.67	10.12	0.29	1.03	0.040	5.5E-01
Mtr.40534.1.S1_at	11.95	10.62	11.19	11.24	11.31	10.98	11.25	0.67	11.18	0.17	0.99	-0.010	8.6E-01
Mtr.41264.1.S1_at	12.22	11.08	12.25	11.98	12.38	12.09	11.85	0.67	12.15	0.21	1.03	0.036	5.0E-01
Mtr.44513.1.S1_at	4.61	4.48	5.69	5.06	5.98	5.53	4.93	0.67	5.52	0.46	1.12	0.165	2.7E-01
Mtr.29425.1.S1_at	7.96	6.65	7.51	7.49	7.37	7.68	7.37	0.67	7.51	0.16	1.02	0.028	7.4E-01
Mtr.11488.1.S1_at	8.87	7.54	8.25	8.32	8.66	8.41	8.22	0.67	8.46	0.18	1.03	0.042	5.8E-01
Mtr.41635.1.S1_at	10.71	9.56	10.72	10.55	10.67	10.50	10.33	0.67	10.58	0.09	1.02	0.034	5.6E-01
Mtr.4463.1.S1_at	2.77	3.91	2.74	2.72	2.72	2.71	3.14	0.67	2.72	0.01	0.87	-0.208	3.3E-01
Mtr.21518.1.S1_s_at	8.34	7.44	8.74	8.04	8.21	7.76	8.17	0.67	8.00	0.23	0.98	-0.030	7.0E-01
Mtr.34857.1.S1_s_at	5.71	4.45	4.69	5.56	5.80	5.45	4.95	0.67	5.60	0.18	1.13	0.179	1.8E-01
Mtr.44131.1.S1_at	5.87	4.78	5.99	5.80	5.05	5.50	5.55	0.67	5.45	0.38	0.98	-0.026	8.3E-01
Mtr.29518.1.S1_at	8.14	7.01	8.19	7.67	8.05	7.81	7.78	0.67	7.84	0.19	1.01	0.012	8.8E-01
Mtr.51614.1.S1_at	8.69	7.37	8.19	8.66	8.46	8.42	8.09	0.67	8.52	0.13	1.05	0.075	3.3E-01
Mtr.36419.1.S1_at	3.21	4.35	3.17	3.18	2.96	3.22	3.58	0.67	3.12	0.14	0.87	-0.198	3.1E-01
Mtr.20821.1.S1_at	7.69	8.73	7.49	7.57	7.39	7.45	7.97	0.67	7.47	0.09	0.94	-0.093	2.7E-01
Mtr.25125.1.S1_at	6.65	5.59	6.83	6.69	6.78	6.28	6.36	0.67	6.58	0.27	1.04	0.050	6.2E-01
Mtr.41329.1.S1_at	10.25	11.51	11.25	11.77	11.77	11.54	11.00	0.67	11.69	0.13	1.06	0.088	1.5E-01
Mtr.11506.1.S1_at	7.11	5.88	6.93	6.94	6.79	6.79	6.64	0.67	6.84	0.09	1.03	0.042	6.4E-01
Mtr.19623.1.S1_at	7.07	7.86	6.53	6.93	6.61	6.82	7.15	0.67	6.78	0.16	0.95	-0.077	4.0E-01
Mtr.42056.1.S1_at	7.07	5.76	6.59	6.40	6.50	6.47	6.47	0.67	6.46	0.05	1.00	-0.004	9.7E-01

Mtr.45525.1.S1_at	8.03	6.75	7.08	7.64	7.44	7.47	7.29	0.67	7.52	0.11	1.03	0.045	5.9E-01
Mtr.8971.1.S1_at	7.79	7.43	8.72	8.10	7.88	7.85	7.98	0.67	7.95	0.14	1.00	-0.006	9.4E-01
Mtr.14325.1.S1_at	9.29	9.86	10.61	9.01	9.32	9.32	9.92	0.67	9.22	0.18	0.93	-0.106	1.5E-01
Mtr.12753.1.S1_at	8.87	7.65	8.70	8.61	8.93	8.85	8.41	0.67	8.80	0.17	1.05	0.066	3.8E-01
Mtr.20634.1.S1_s_at	8.38	7.09	8.04	8.27	8.13	8.01	7.84	0.67	8.14	0.13	1.04	0.054	4.9E-01
Mtr.49905.1.S1_at	5.63	4.47	5.62	5.64	5.33	5.94	5.24	0.67	5.64	0.31	1.08	0.105	4.0E-01
Mtr.13587.1.S1_at	8.44	7.16	8.11	8.26	8.27	8.03	7.90	0.67	8.19	0.14	1.04	0.052	5.0E-01
Mtr.31170.1.S1_at	7.40	6.56	7.87	7.43	7.74	7.49	7.28	0.67	7.55	0.16	1.04	0.053	5.3E-01
Mtr.9546.1.S1_at	7.34	6.08	7.09	7.34	7.45	7.73	6.83	0.66	7.51	0.20	1.10	0.136	1.7E-01
Mtr.16283.1.S1_s_at	7.76	7.21	8.54	8.06	8.17	7.52	7.84	0.66	7.92	0.35	1.01	0.014	8.7E-01
Mtr.11212.1.S1_s_at	8.78	7.89	7.47	7.31	7.36	7.79	8.05	0.66	7.49	0.27	0.93	-0.104	2.5E-01
Mtr.6948.1.S1_at	7.00	5.92	7.14	7.20	6.80	6.61	6.69	0.66	6.87	0.30	1.03	0.039	6.9E-01
Mtr.1634.1.S1_at	8.38	7.17	8.25	8.15	8.05	8.27	7.93	0.66	8.16	0.11	1.03	0.040	5.9E-01
Mtr.13910.1.S1_s_at	7.36	8.45	7.25	7.49	7.53	7.65	7.69	0.66	7.56	0.09	0.98	-0.025	7.5E-01
Mtr.48644.1.S1_at	7.95	9.25	8.85	8.19	7.77	7.77	8.68	0.66	7.91	0.24	0.91	-0.135	1.3E-01
Mtr.38008.1.S1_at	10.00	8.89	8.82	9.15	9.26	9.51	9.24	0.66	9.30	0.18	1.01	0.010	8.8E-01
Mtr.29109.1.S1_at	4.27	5.35	4.14	4.64	4.88	4.52	4.58	0.66	4.68	0.18	1.02	0.030	8.2E-01
Mtr.34315.1.S1_at	7.66	6.43	7.47	8.22	8.61	8.62	7.19	0.66	8.48	0.23	1.18	0.240	3.3E-02
Mtr.24722.1.S1_at	5.29	3.99	4.88	5.21	5.09	5.04	4.72	0.66	5.11	0.09	1.08	0.115	3.7E-01
Mtr.10279.1.S1_s_at	8.91	8.00	9.30	8.94	8.77	8.73	8.74	0.66	8.81	0.12	1.01	0.012	8.5E-01
Mtr.32961.1.S1_at	8.22	7.14	8.35	8.36	8.32	8.42	7.90	0.66	8.37	0.05	1.06	0.083	2.9E-01
Mtr.47298.1.S1_at	7.92	7.04	6.61	7.91	9.01	8.58	7.19	0.66	8.50	0.55	1.18	0.241	5.9E-02
Mtr.20245.1.S1_s_at	8.58	7.49	8.70	8.55	8.80	8.64	8.26	0.66	8.66	0.12	1.05	0.069	3.6E-01
Mtr.23404.1.S1_at	3.91	3.01	4.30	3.81	3.79	3.77	3.74	0.66	3.79	0.02	1.01	0.019	9.0E-01
Mtr.50514.1.S1_x_at	4.16	2.85	3.67	3.67	3.63	4.43	3.56	0.66	3.91	0.45	1.10	0.135	4.9E-01
Mtr.3186.1.S1_at	3.17	2.21	3.48	3.02	3.59	3.29	2.95	0.66	3.30	0.28	1.12	0.160	4.5E-01
Mtr.23594.1.S1_at	8.32	7.06	8.06	8.18	8.19	8.09	7.81	0.66	8.15	0.06	1.04	0.061	4.3E-01
Mtr.31909.1.S1_at	2.58	3.72	2.56	2.54	2.56	2.55	2.96	0.66	2.55	0.01	0.86	-0.212	3.5E-01
Mtr.25263.1.S1_s_at	3.91	2.84	4.05	3.55	3.52	3.51	3.60	0.66	3.52	0.02	0.98	-0.032	8.5E-01
Mtr.41122.1.S1_at	8.69	7.40	8.29	8.66	8.56	8.55	8.13	0.66	8.59	0.06	1.06	0.080	2.9E-01
Mtr.41864.1.S1_at	3.65	3.07	2.32	3.29	3.29	3.80	3.01	0.66	3.46	0.30	1.15	0.198	3.5E-01
Mtr.43911.1.S1_at	8.66	7.34	7.93	8.19	7.87	8.26	7.98	0.66	8.11	0.20	1.02	0.024	7.6E-01
Mtr.43896.1.S1_at	8.85	7.82	9.05	8.62	8.70	8.48	8.57	0.66	8.60	0.11	1.00	0.005	9.5E-01
Mtr.14064.1.S1_s_at	6.62	6.53	7.72	7.09	8.16	7.08	6.96	0.66	7.44	0.62	1.07	0.097	4.1E-01
Mtr.10815.1.S1_at	5.71	6.82	6.89	7.47	8.26	7.78	6.47	0.66	7.84	0.40	1.21	0.277	3.8E-02
Mtr.9059.1.S1_at	10.04	8.81	9.87	10.02	9.78	9.85	9.57	0.66	9.88	0.12	1.03	0.046	4.7E-01
Mtr.17972.1.S1_at	8.03	6.95	8.16	7.60	8.07	7.85	7.72	0.66	7.84	0.24	1.02	0.023	7.7E-01
Mtr.50220.1.S1_at	7.02	5.77	6.78	6.96	6.76	6.76	6.52	0.66	6.83	0.12	1.05	0.066	4.7E-01
Mtr.16721.1.S1_s_at	5.23	6.37	5.22	5.22	5.22	5.22	5.61	0.66	5.22	0.00	0.93	-0.104	3.6E-01
Mtr.48948.1.S1_at	9.35	8.35	9.61	8.91	8.90	8.75	9.10	0.66	8.86	0.09	0.97	-0.040	5.5E-01
Mtr.28982.1.S1_at	2.67	3.81	2.66	2.65	2.65	2.56	3.05	0.66	2.62	0.05	0.86	-0.216	3.3E-01
Mtr.35948.1.S1_at	5.34	4.28	4.12	4.22	4.21	4.21	4.58	0.66	4.21	0.00	0.92	-0.119	4.0E-01
Mtr.9017.1.S1_at	7.31	7.66	8.59	7.81	7.89	7.64	7.86	0.66	7.78	0.13	0.99	-0.014	8.5E-01
Mtr.1016.1.S1_at	4.94	6.22	5.30	5.01	4.94	4.92	5.48	0.66	4.96	0.05	0.90	-0.146	2.4E-01
Mtr.39640.1.S1_at	7.98	6.89	8.09	7.94	7.74	7.84	7.66	0.66	7.84	0.10	1.02	0.034	6.6E-01
Mtr.17044.1.S1_at	5.11	3.84	4.14	3.73	3.75	3.72	4.36	0.66	3.73	0.02	0.86	-0.225	1.7E-01
Mtr.36254.1.S1_at	8.14	7.39	8.71	8.02	8.47	8.06	8.08	0.66	8.18	0.25	1.01	0.018	8.1E-01
Mtr.18526.1.S1_s_at	8.47	8.50	9.63	8.77	8.68	8.38	8.86	0.66	8.61	0.20	0.97	-0.042	5.5E-01
Mtr.42310.1.S1_at	9.46	8.26	9.35	9.03	9.20	9.03	9.02	0.66	9.09	0.09	1.01	0.010	8.8E-01
Mtr.17581.1.S1_at	8.30	7.21	8.40	8.39	8.39	8.19	7.97	0.66	8.32	0.12	1.04	0.063	4.1E-01
Mtr.44383.1.S1_at	7.44	6.20	7.21	7.75	7.94	7.51	6.95	0.66	7.73	0.21	1.11	0.154	1.2E-01
Mtr.5829.1.S1_at	9.06	7.81	8.80	8.95	9.09	9.07	8.55	0.66	9.04	0.08	1.06	0.079	2.8E-01
Mtr.44795.1.S1_at	8.55	7.29	8.27	8.59	8.48	8.66	8.04	0.66	8.57	0.09	1.07	0.093	2.4E-01
Mtr.23411.1.S1_s_at	8.45	7.16	8.08	8.47	8.38	8.66	7.90	0.66	8.50	0.14	1.08	0.107	2.0E-01
Mtr.40913.1.S1_at	7.88	6.76	6.71	7.32	7.26	6.99	7.12	0.66	7.19	0.17	1.01	0.015	8.6E-01
Mtr.12053.1.S1_at	10.55	9.40	10.54	10.60	10.49	10.74	10.16	0.66	10.61	0.13	1.04	0.062	3.2E-01
Mtr.50484.1.S1_s_at	8.74	7.49	8.49	8.61	8.66	8.56	8.24	0.66	8.61	0.05	1.04	0.063	3.9E-01
Mtr.50625.1.S1_at	6.38	5.94	7.24	6.66	6.70	6.59	6.52	0.66	6.65	0.06	1.02	0.028	7.6E-01
Mtr.45127.1.S1_at	8.58	7.74	9.04	8.60	8.67	8.47	8.45	0.66	8.58	0.10	1.01	0.021	7.6E-01
Mtr.17363.1.S1_x_at	3.02	4.15	3.00	2.99	2.99	2.99	3.39	0.66	2.99	0.00	0.88	-0.180	3.6E-01
Mtr.33166.1.S1_at	6.03	4.84	5.94	6.46	6.38	6.71	5.61	0.66	6.52	0.17	1.16	0.217	8.2E-02
Mtr.20489.1.S1_at	9.12	7.90	8.95	8.96	8.99	9.00	8.66	0.66	8.98	0.02	1.04	0.054	4.4E-01
Mtr.38665.1.S1_at	9.76	8.46	9.30	9.34	9.39	9.42	9.17	0.66	9.38	0.04	1.02	0.032	6.2E-01
Mtr.17276.1.S1_at	2.95	4.11	2.98	2.94	2.95	2.94	3.35	0.66	2.94	0.01	0.88	-0.186	3.5E-01
Mtr.27810.1.S1_at	3.15	2.44	3.76	2.64	2.59	2.64	3.12	0.66	2.62	0.03	0.84	-0.248	2.7E-01
Mtr.1015.1.S1_at	6.45	7.49	6.27	6.39	6.22	5.91	6.74	0.66	6.17	0.24	0.92	-0.126	2.4E-01
Mtr.24690.1.S1_at	6.43	5.77	7.09	8.19	8.14	8.04	6.43	0.66	8.12	0.08	1.26	0.337	1.2E-02
Mtr.23121.1.S1_s_at	8.46	7.14	7.76	8.17	8.33	8.24	7.79	0.66	8.25	0.08	1.06	0.083	3.0E-01
Mtr.13906.1.S1_at	8.51	7.66	8.96	9.03	8.80	8.73	8.38	0.66	8.85	0.16	1.06	0.080	2.9E-01
Mtr.21097.1.S1_at	2.34	3.48	2.33	2.32	2.32	2.31	2.71	0.66	2.32	0.01	0.85	-0.227	3.6E-01
Mtr.7790.1.S1_s_at	8.18	6.86	7.51	7.61	8.02	8.15	7.52	0.66	7.93	0.28	1.05	0.077	3.8E-01
Mtr.12472.1.S1_at	4.21	3.06	4.21	5.11	5.50	3.64	3.82	0.66	4.75	0.98	1.24	0.313	2.5E-01
Mtr.42242.1.S1_at	9.92	9.36	10.68	10.43	11.02	10.72	9.99	0.66	10.72	0.29	1.07	0.103	1.5E-01
Mtr.41518.1.S1_at	11.11	9.80	10.61	10.60	10.12	10.64	10.51	0.66	10.46	0.29	1.00	-0.007	9.1E-01
Mtr.29135.1.S1_at	9.07	8.18	9.47	8.64	8.94	8.83	8.91	0.66	8.80	0.15	0.99	-0.017	8.0E-01
Mtr.42537.1.S1_at	7.07	8.03	6.77	7.62	7.33	7.60	7.29	0.66	7.52	0.16	1.03	0.044	6.0E-01
Mtr.11561.1.S1_at	5.71	4.79	4.43	4.81	4.27	5.17	4.97	0.66	4.75	0.45	0.95	-0.067	6.5E-01
Mtr.32580.1.S1_at	7.09	7.19	8.28	7.25	7.52	7.26	7.52	0.66	7.34	0.15	0.98	-0.034	6.8E-01
Mtr.14782.1.S1_at	3.66	2.43	3.45	3.44	3.34	3.43	3.18	0.66	3.40	0.05	1.07	0.097	6.0E-01
Mtr.9879.1.S1_at	6.24	4.93	5.70	6.13	6.24	6.09	5.62	0.66	6.16	0.08	1.10	0.131	2.4E-01
Mtr.51674.1.S1_at	7.86	6.66	7.74	7.39	7.59	7.83	7.42	0.66	7.60	0.22	1.02	0.034	6.8E-01
Mtr.39503.1.S1_at	7.98	7.82	9.04	8.55	8.54	8.16	8.28	0.66	8.42	0.23	1.02	0.024	7.5E-01
Mtr.9442.1.S1_at	7.85	6.78	7.99	8.06	8.06	7.83	7.54	0.66	7.98	0.13	1.06	0.082	3.2E-01
Mtr.5804.1.S1_at	8.19	7.69	9.00	8.01	7.31	7.74	8.29	0.66	7.68	0.35	0.93	-0.110	2.3E-01
Mtr.46630.1.S1_at	4.56	3.96	5.27	4.16	4.66	4.13	4.60	0.66	4.32	0.30	0.94	-0.091	5.4E-01
Mtr.5817.1.S1_at	6.96	5.71	6.71	7.32	6.83	7.24	6.46	0.66	7.13	0.26	1.10	0.142	1.8E-01
Mtr.2153.1.S1_at	5.12	5.93	4.62	5.09	5.77	5.64	5.22	0.66	5.50	0.36	1.05	0.075	5.5E-01
Mtr.15724.1.S1_at	3.44	4.53	3.35	3.36	4.08	3.32	3.77	0.66	3.59	0.43	0.95	-0.073	7.0E-01
Mtr.50762.1.S1_at	5.67	6.99	6.37	6.20	6.13	6.53	6.34	0.66	6.28	0.21	0.99	-0.014	8.9E-01
Mtr.14053.1.S1_s_at	7.41	7.82	8.70	7.98	8.78	7.79	7.98	0.66	8.18	0.52	1.03	0.037	6.9E-01
Mtr.40959.1.S1_at	8.10	7.14	6.84	6.86	7.13	7.23	7.36	0.66	7.07	0.19	0.96	-0.057	5.1E-01
Mtr.39023.1.S1_at	7.11	6.01	7.19	6.86	7.06	6.95	6.77	0.66	6.96	0.10	1.03	0.040	6.5E-01
Mtr.31389.1.S1_s_at	6.96	6.16	7.47	6.44	7.17	6.63	6.86	0.66	6.75	0.38	0.98	-0.025	8.0E-01
Mtr.8824.1.S1_at	11.53	10.93	10.21	10.62	10.23	10.64	10.89	0.66	10.50	0.23	0.96	-0.053	3.8E-01
Mtr.17363.1.S1_at	2.84	3.94	2.76	2.73	2.68	2.75	3.18	0.66	2.72	0.03	0.85	-0.226	2.9E-01
Mtr.11941.1.S1_at	5.91	4.84	6.05	6.12	6.10	5.96	5.60	0.66	6.06	0.09	1.08	0.113	3.0E-01
Mtr.49274.1.S1_at	9.72	11.02	10.16	9.49	9.71	9.40	10.30	0.66	9.53	0.16	0.93	-0.111	1.2E-01
Mtr.8093.1.S1_at	2.63	3.77	2.64	2.76	2.48	2.64	3.01	0.66	2.63	0.14	0.87	-0.198	3.8E-01
Mtr.21739.1.S1_at	9.83	8.80	10.02	9.83	9.52	9.60	9.55	0.66	9.65	0.16	1.01	0.015	8.1E-01
Mtr.45511.1.S1_at	6.38	5.19	6.28	5.57	5.82	5.76	5.95	0.66	5.71	0.13	0.96	-0.059	5.7E-01
Mtr.11205.1.S1_at	7.86	7.25	8.56	8.04	7.98	8.09	7.89	0.66	8.04	0.05	1.02	0.027	7.2E-01
Mtr.32840.1.S1_at	8.12	7.00	8.16	8.30	8.22	8.21	7.76	0.66	8.24	0.05	1.06	0.087	2.8E-01
Mtr.40781.1.S1_s_at	2.95	4.20	3.23	2.85	2.80	2.76	3.46	0.66	2.80	0.04	0.81	-0.303	1.6E-01
Mtr.17280.1.S1_at	11.33	10.03	10.49	10.87	10.74	10.72	10.62	0.66	10.78	0.08	1.02	0.022	7.0E-01
Mtr.36037.1.S1_at	5.82	4.74	4.64	5.52	6.14	6.15	5.07	0.66	5.93	0.36	1.17	0.228	1.2E-01
Mtr.12168.1.S1_at	8.44	7.32	8.48	8.80	8.37	8.97	8.08	0.66	8.71	0.31	1.08	0.109	2.0E-01
Mtr.12565.1.S1_at	3.28	3.75	4.58	3.28	3.19	3.15	3.87	0.66	3.21	0.07	0.83	-0.271	1.6E-01
Mtr.19874.1.S1_at	9.28	8.13	9.27	9.79	9.91	9.69	8.89	0.66	9.80	0.11	1.10	0.140	7.8E-02
Mtr.14934.1.S1_s_at	4.10	3.89	5.12	6.10	7.29	6.21	4.37	0.66	6.53	0.65	1.50	0.580	1.6E-02
Mtr.1560.1.S1_x_at	3.81	2.61	3.68	3.90	3.48	3.62	3.37	0.66	3.67	0.21	1.09	0.124	4.9E-01
Mtr.38288.1.S1_at	7.71	6.56	7.68	7.61	7.74	7.69	7.32	0.66	7.68	0.07	1.05	0.069	4.0E-01
Mtr.1086.1.S1_at	8.21	7.14	8.34	8.45	8.39	8.61	7.90	0.66	8.48	0.11	1.07	0.103	2.0E-01
Mtr.37984.1.S1_at	5.48	4.19	4.63	4.09	4.07	3.88	4.77	0.66	4.01	0.11	0.84	-0.248	1.2E-01
Mtr.51797.1.S1_at	7.32	7.38	8.49	7.42	7.58	7.41	7.73	0.66	7.47	0.09	0.97	-0.050	5.3E-01
Mtr.32875.1.S1_at	7.80	6.52	7.39	7.71	7.69	7.48	7.24	0.66	7.62	0.13	1.05	0.075	3.7E-01
Mtr.1331.1.S1_at	6.72	5.79	7.06	7.13	6.93	6.96	6.52	0.66	7.01	0.11	1.07	0.104	2.8E-01
Mtr.39424.1.S1_at	10.00	8.74	9.69	9.64	10.09	9.97	9.47	0.66	9.90	0.24	1.04	0.063	3.5E-01
Mtr.10972.1.S1_at	6.88	6.21	7.53	6.51	6.75	6.32	6.87	0.66	6.53	0.21	0.95	-0.074	4.3E-01
Mtr.9672.1.S1_at	9.52	8.24	8.61	9.09	9.10	9.21	8.79	0.66	9.13	0.07	1.04	0.055	4.2E-01
Mtr.33391.1.S1_at	10.46	9.99	11.29	10.88	10.56	10.12	10.58	0.66	10.52	0.38	0.99	-0.008	9.0E-01
Mtr.51405.1.S1_at	8.16	6.87	7.72	7.81	7.36	7.80	7.58	0.66	7.66	0.26	1.01	0.014	8.7E-01
Mtr.36993.1.S1_at	7.48	6.18	7.00	7.01	7.48	7.32	6.89	0.66	7.27	0.24	1.06	0.078	4.0E-01
Mtr.21895.1.S1_at	8.89	7.66	8.69	8.57	8.43	8.34	8.41	0.66	8.45	0.12	1.00	0.006	9.3E-01
Mtr.4753.1.S1_at	7.58	6.93	8.24	7.79	7.99	8.11	7.58	0.66	7.96	0.16	1.05	0.070	3.9E-01
Mtr.37020.1.S1_at	6.88	6.07	7.37	7.49	8.38	7.92	6.78	0.66	7.93	0.44	1.17	0.227	6.5E-02
Mtr.34726.1.S1_at	3.45	3.60	4.66	3.92	3.74	2.93	3.90	0.66	3.53	0.53	0.90	-0.146	4.8E-01
Mtr.8893.1.S1_at	7.86	6.74	6.70	6.44	6.71	6.69	7.10	0.66	6.61	0.15	0.93	-0.102	2.8E-01
Mtr.45182.1.S1_at	7.22	7.12	8.30	7.16	7.51	7.25	7.55	0.66	7.30	0.18	0.97	-0.047	5.7E-01
Mtr.38631.1.S1_at	8.51	7.53	8.78	8.40	8.91	8.42	8.27	0.66	8.58	0.29	1.04	0.052	5.0E-01
Mtr.22058.1.S1_at	8.70	7.41	8.27	8.69	8.59	8.67	8.13	0.66	8.65	0.05	1.06	0.090	2.4E-01
Mtr.5591.1.S1_at	5.36	4.06	4.88	4.88	4.88	4.88	4.77	0.66	4.88	0.00	1.02	0.033	7.9E-01

Mtr.39629.1.S1_s_at	7.77	6.59	7.68	7.82	7.82	7.64	7.35	0.66	7.76	0.11	1.06	0.078	3.5E-01
Mtr.5165.1.S1_at	3.94	2.96	4.20	3.76	3.76	3.73	3.70	0.66	3.75	0.02	1.01	0.020	9.0E-01
Mtr.23121.1.S1_at	9.45	8.21	8.46	8.90	8.90	9.23	8.71	0.66	9.01	0.19	1.03	0.049	4.8E-01
Mtr.27001.1.S1_at	2.84	4.03	2.94	2.81	2.83	2.86	3.27	0.66	2.83	0.02	0.87	-0.208	3.1E-01
Mtr.37042.1.S1_at	3.56	3.09	2.26	3.04	3.06	4.25	2.97	0.66	3.45	0.70	1.16	0.218	4.3E-01
Mtr.4098.1.S1_at	4.13	3.07	4.28	4.22	4.12	4.07	3.83	0.66	4.14	0.08	1.08	0.113	4.6E-01
Mtr.51756.1.S1_at	2.70	3.84	2.70	2.70	2.70	2.70	3.08	0.66	2.70	0.00	0.88	-0.190	3.7E-01
Mtr.52081.1.S1_at	8.90	7.62	8.52	8.30	8.17	8.39	8.34	0.66	8.29	0.11	0.99	-0.010	8.8E-01
Mtr.4007.1.S1_at	6.39	5.57	6.87	6.32	7.30	6.89	6.28	0.66	6.84	0.49	1.09	0.123	3.0E-01
Mtr.12364.1.S1_at	9.01	7.99	9.21	9.26	8.98	8.95	8.74	0.66	9.06	0.17	1.04	0.053	4.5E-01
Mtr.30504.1.S1_x_at	5.13	3.88	4.84	4.86	5.05	5.23	4.62	0.66	5.05	0.18	1.09	0.129	3.3E-01
Mtr.35125.1.S1_at	4.12	5.29	4.21	4.31	4.51	4.01	4.54	0.66	4.27	0.25	0.94	-0.087	5.5E-01
Mtr.7249.1.S1_at	7.16	5.86	6.33	7.30	7.64	7.76	6.45	0.66	7.57	0.24	1.17	0.231	5.0E-02
Mtr.49583.1.S1_at	5.76	4.82	6.08	5.35	6.05	5.24	5.55	0.66	5.54	0.44	1.00	-0.001	9.9E-01
Mtr.9971.1.S1_at	7.58	6.27	7.02	6.88	7.34	7.18	6.96	0.66	7.13	0.23	1.03	0.036	6.9E-01
Mtr.34148.1.S1_at	2.32	3.45	2.31	2.30	2.19	2.28	2.69	0.66	2.26	0.06	0.84	-0.253	3.2E-01
Mtr.32340.1.S1_at	5.74	4.59	5.72	6.07	5.82	5.64	5.35	0.66	5.84	0.22	1.09	0.127	2.8E-01
Mtr.20248.1.S1_s_at	6.22	5.90	7.16	6.74	6.92	6.77	6.43	0.66	6.81	0.10	1.06	0.083	3.8E-01
Mtr.3353.1.S1_at	10.51	9.24	10.15	10.06	10.43	10.15	9.96	0.66	10.21	0.19	1.03	0.036	5.6E-01
Mtr.32928.1.S1_s_at	9.19	7.90	8.77	8.65	8.54	8.44	8.62	0.66	8.54	0.10	0.99	-0.013	8.5E-01
Mtr.25008.1.S1_x_at	7.72	6.45	7.39	7.56	6.56	7.77	7.19	0.66	7.30	0.65	1.02	0.022	8.4E-01
Mtr.39946.1.S1_s_at	8.82	7.51	8.30	8.66	8.62	8.54	8.21	0.66	8.60	0.06	1.05	0.068	3.6E-01
Mtr.39881.1.S1_at	9.26	8.09	9.18	9.04	9.37	8.83	8.84	0.66	9.08	0.27	1.03	0.038	5.9E-01
Mtr.7389.1.S1_s_at	9.46	8.48	9.72	9.45	9.17	9.31	9.22	0.66	9.31	0.14	1.01	0.014	8.2E-01
Mtr.10116.1.S1_s_at	9.42	8.15	9.07	8.85	8.99	9.05	8.88	0.66	8.97	0.10	1.01	0.014	8.3E-01
Mtr.11023.1.S1_at	7.09	6.32	7.62	7.07	7.27	7.10	7.01	0.66	7.15	0.11	1.02	0.028	7.4E-01
Mtr.4149.1.S1_at	10.03	8.93	8.87	9.48	9.22	9.70	9.28	0.66	9.47	0.24	1.02	0.029	6.6E-01
Mtr.46961.1.S1_at	8.65	7.34	8.04	8.91	8.90	9.02	8.01	0.65	8.94	0.07	1.12	0.158	7.1E-02
Mtr.10674.1.S1_at	8.82	8.28	9.58	9.08	8.93	9.03	8.89	0.65	9.01	0.07	1.01	0.019	7.7E-01
Mtr.36794.1.S1_at	6.99	6.81	5.78	4.99	5.57	5.57	6.53	0.65	5.38	0.33	0.82	-0.279	5.4E-02
Mtr.22070.1.S1_at	4.28	5.15	3.87	3.82	3.78	3.52	4.43	0.65	3.71	0.16	0.84	-0.258	1.4E-01
Mtr.15847.1.S1_at	7.50	6.40	7.56	7.41	6.86	7.22	7.16	0.65	7.17	0.28	1.00	0.002	9.8E-01
Mtr.15876.1.S1_at	3.04	4.15	3.00	3.04	3.03	3.26	3.40	0.65	3.11	0.13	0.92	-0.128	5.0E-01
Mtr.44847.1.S1_at	9.07	7.91	9.02	8.65	8.62	8.60	8.67	0.65	8.62	0.02	0.99	-0.007	9.1E-01
Mtr.43250.1.S1_at	12.11	10.95	12.06	12.31	12.06	12.35	11.71	0.65	12.24	0.15	1.05	0.064	2.4E-01
Mtr.11228.1.S1_at	2.95	3.45	4.25	3.58	4.87	3.70	3.55	0.65	4.05	0.71	1.14	0.191	4.2E-01
Mtr.41849.1.S1_at	9.36	8.10	9.02	9.19	9.18	9.31	8.83	0.65	9.23	0.07	1.05	0.064	3.5E-01
Mtr.13810.1.S1_at	7.02	5.74	6.16	6.17	6.98	6.52	6.31	0.65	6.56	0.41	1.04	0.057	6.0E-01
Mtr.4621.1.S1_at	2.66	3.76	2.60	2.59	2.59	2.58	3.01	0.65	2.59	0.00	0.86	-0.215	3.3E-01
Mtr.43903.1.S1_at	9.40	8.11	8.93	8.81	8.86	8.96	8.81	0.65	8.88	0.08	1.01	0.011	8.7E-01
Mtr.20259.1.S1_at	2.49	3.61	2.47	2.45	2.44	2.48	2.86	0.65	2.46	0.02	0.86	-0.217	3.5E-01
Mtr.20510.1.S1_at	2.71	3.82	2.66	2.66	2.65	2.64	3.06	0.65	2.65	0.01	0.86	-0.210	3.3E-01
Mtr.47143.1.S1_s_at	11.21	12.16	10.90	10.77	10.89	11.01	11.42	0.65	10.89	0.12	0.95	-0.069	2.4E-01
Mtr.13844.1.S1_at	8.10	6.89	7.91	7.79	7.78	7.92	7.63	0.65	7.83	0.08	1.03	0.037	6.3E-01
Mtr.34012.1.S1_at	2.76	3.87	2.71	2.75	2.75	2.75	3.11	0.65	2.75	0.00	0.88	-0.178	3.9E-01
Mtr.9073.1.S1_at	3.35	4.58	4.36	4.32	5.72	4.79	4.10	0.65	4.94	0.71	1.21	0.270	2.0E-01
Mtr.50176.1.S1_s_at	9.23	8.08	9.18	9.08	9.08	9.12	8.83	0.65	9.09	0.03	1.03	0.042	5.3E-01
Mtr.11318.1.S1_at	10.76	9.81	11.06	11.07	10.90	10.90	10.54	0.65	10.96	0.10	1.04	0.055	3.4E-01
Mtr.18909.1.S1_s_at	8.94	7.74	8.80	8.80	8.66	8.89	8.49	0.65	8.78	0.12	1.03	0.049	4.9E-01
Mtr.34158.1.S1_s_at	9.07	9.86	8.57	8.70	9.30	8.99	9.17	0.65	9.00	0.30	0.98	-0.027	7.0E-01
Mtr.13584.1.S1_at	10.51	9.22	9.70	9.87	9.86	9.99	9.81	0.65	9.90	0.07	1.01	0.013	8.2E-01
Mtr.16732.1.S1_at	3.45	4.53	3.36	3.41	3.16	3.84	3.78	0.65	3.47	0.34	0.92	-0.121	5.1E-01
Mtr.28784.1.S1_at	8.03	7.14	8.42	8.50	8.46	8.44	7.86	0.65	8.47	0.03	1.08	0.107	1.9E-01
Mtr.14785.1.S1_at	5.86	5.14	6.44	5.49	5.72	5.96	5.81	0.65	5.72	0.23	0.98	-0.023	8.3E-01
Mtr.35755.1.S1_at	10.11	10.82	9.51	9.70	10.02	9.86	10.15	0.65	9.86	0.16	0.97	-0.041	5.0E-01
Mtr.35343.1.S1_at	3.20	4.30	3.15	3.37	3.10	3.08	3.55	0.65	3.18	0.16	0.90	-0.158	4.0E-01
Mtr.40641.1.S1_at	7.89	6.68	7.71	7.72	7.60	7.81	7.43	0.65	7.71	0.11	1.04	0.054	5.0E-01
Mtr.33924.1.S1_at	8.73	7.61	8.76	8.93	8.79	8.82	8.37	0.65	8.85	0.07	1.06	0.080	2.8E-01
Mtr.29522.1.S1_at	10.25	9.05	10.10	10.66	10.24	10.59	9.80	0.65	10.49	0.23	1.07	0.099	1.6E-01
Mtr.582.1.S1_at	11.50	10.22	10.68	11.13	11.15	11.23	10.80	0.65	11.17	0.06	1.03	0.048	3.8E-01
Mtr.18810.1.S1_at	9.23	7.94	8.38	8.71	8.89	8.89	8.51	0.65	8.83	0.11	1.04	0.052	4.6E-01
Mtr.45248.1.S1_at	4.51	3.23	4.08	4.59	4.69	5.11	3.94	0.65	4.80	0.28	1.22	0.283	1.0E-01
Mtr.35703.1.S1_at	8.16	7.03	8.16	8.26	8.28	8.19	7.79	0.65	8.24	0.05	1.06	0.082	2.9E-01
Mtr.51133.1.S1_at	2.89	4.04	2.93	2.71	2.76	2.88	3.29	0.65	2.78	0.09	0.85	-0.241	2.5E-01
Mtr.44186.1.S1_at	7.81	6.57	7.55	7.67	7.57	7.63	7.31	0.65	7.62	0.05	1.04	0.061	4.5E-01
Mtr.49884.1.S1_at	2.81	3.93	2.79	2.79	2.78	2.76	3.18	0.65	2.78	0.01	0.87	-0.195	3.5E-01
Mtr.41166.1.S1_at	8.12	7.00	8.14	8.02	8.32	7.84	7.76	0.65	8.06	0.24	1.04	0.055	4.9E-01
Mtr.33127.1.S1_at	5.14	3.92	4.93	4.96	5.22	5.18	4.66	0.65	5.12	0.14	1.10	0.135	3.0E-01
Mtr.30997.1.S1_at	3.54	2.51	2.33	2.81	3.18	2.47	2.79	0.65	2.82	0.35	1.01	0.015	9.5E-01
Mtr.10135.1.S1_at	6.85	5.73	6.87	6.43	6.94	6.75	6.49	0.65	6.71	0.26	1.03	0.048	6.2E-01
Mtr.4571.1.S1_at	5.85	4.71	4.72	5.32	5.18	5.25	5.10	0.65	5.25	0.07	1.03	0.042	7.1E-01
Mtr.43537.1.S1_at	8.80	7.61	8.66	8.63	8.84	8.84	8.36	0.65	8.77	0.12	1.05	0.069	3.4E-01
Mtr.1566.1.S1_at	4.08	2.97	2.93	2.92	2.92	2.93	3.32	0.65	2.92	0.00	0.88	-0.185	3.5E-01
Mtr.36026.1.S1_at	5.88	4.59	5.43	5.35	5.52	5.07	5.30	0.65	5.31	0.22	1.00	0.003	9.8E-01
Mtr.13126.1.S1_at	6.84	5.63	6.64	6.99	7.09	6.82	6.37	0.65	6.97	0.14	1.09	0.129	2.0E-01
Mtr.40968.1.S1_at	7.56	6.59	7.83	7.57	7.76	7.39	7.33	0.65	7.57	0.19	1.03	0.048	5.6E-01
Mtr.7263.1.S1_at	7.77	6.57	7.60	7.95	7.85	7.77	7.31	0.65	7.86	0.09	1.07	0.104	2.2E-01
Mtr.1741.1.S1_at	6.96	5.82	6.95	7.17	7.27	7.59	6.58	0.65	7.34	0.22	1.12	0.159	1.3E-01
Mtr.39305.1.S1_at	5.71	4.61	5.76	5.32	5.42	5.34	5.36	0.65	5.36	0.06	1.00	0.000	1.0E+00
Mtr.9705.1.S1_s_at	9.49	8.34	9.45	9.47	9.47	9.51	9.09	0.65	9.49	0.02	1.04	0.061	3.5E-01
Mtr.22448.1.S1_s_at	6.25	5.12	6.25	6.13	6.29	5.57	5.88	0.65	6.00	0.37	1.02	0.029	7.9E-01
Mtr.6290.1.S1_x_at	5.68	6.55	6.95	6.26	5.60	6.21	6.39	0.65	6.02	0.36	0.94	-0.086	4.4E-01
Mtr.34789.1.S1_at	8.51	7.23	8.04	7.78	8.08	7.92	7.93	0.65	7.92	0.15	1.00	-0.001	9.9E-01
Mtr.36010.1.S1_at	9.29	8.16	9.29	9.21	9.29	9.31	8.91	0.65	9.27	0.05	1.04	0.056	4.0E-01
Mtr.36207.1.S1_at	3.08	2.13	3.38	3.02	3.02	2.94	2.86	0.65	2.99	0.04	1.04	0.062	7.5E-01
Mtr.15124.1.S1_x_at	1.66	2.78	1.65	1.65	1.65	1.65	2.03	0.65	1.65	0.00	0.81	-0.300	3.7E-01
Mtr.28376.1.S1_at	5.34	4.06	4.91	5.71	5.17	5.69	4.77	0.65	5.52	0.31	1.16	0.211	1.4E-01
Mtr.32244.1.S1_at	8.03	6.73	7.31	8.03	8.13	7.77	7.36	0.65	7.98	0.19	1.08	0.117	1.9E-01
Mtr.24566.1.S1_at	2.99	4.02	2.81	2.80	2.77	2.77	3.27	0.65	2.78	0.02	0.85	-0.235	2.6E-01
Mtr.42368.1.S1_at	7.18	6.75	8.03	7.13	7.39	6.93	7.32	0.65	7.15	0.23	0.98	-0.034	6.9E-01
Mtr.35206.1.S1_at	5.48	4.60	5.87	6.05	5.94	5.88	5.32	0.65	5.96	0.09	1.12	0.163	1.7E-01
Mtr.17177.1.S1_at	4.29	3.17	4.30	4.29	4.27	4.30	3.92	0.65	4.28	0.02	1.09	0.127	3.9E-01
Mtr.5217.1.S1_at	7.44	6.52	7.78	7.54	7.63	7.59	7.25	0.65	7.59	0.04	1.05	0.066	4.2E-01
Mtr.45747.1.S1_at	2.06	3.19	2.06	2.05	2.05	2.05	2.43	0.65	2.05	0.00	0.84	-0.246	3.7E-01
Mtr.15326.1.S1_at	6.58	7.20	7.88	8.23	9.03	8.20	7.22	0.65	8.49	0.47	1.18	0.233	5.2E-02
Mtr.44294.1.S1_at	11.84	10.55	11.03	11.33	11.17	11.38	11.14	0.65	11.29	0.11	1.01	0.020	7.1E-01
Mtr.6489.1.S1_at	11.05	10.09	9.81	10.10	9.80	10.47	10.32	0.65	10.12	0.34	0.98	-0.027	6.7E-01
Mtr.48589.1.S1_at	7.03	6.06	7.29	6.69	7.14	6.93	6.79	0.65	6.92	0.23	1.02	0.026	7.7E-01
Mtr.33589.1.S1_at	9.86	8.76	9.91	8.86	9.07	8.85	9.51	0.65	8.92	0.13	0.94	-0.092	2.0E-01
Mtr.44754.1.S1_at	5.67	4.54	5.66	5.73	5.66	5.84	5.29	0.65	5.74	0.09	1.09	0.119	3.0E-01
Mtr.2476.1.S1_s_at	6.82	5.72	5.68	6.17	5.83	6.38	6.07	0.65	6.13	0.28	1.01	0.013	9.0E-01
Mtr.3126.1.S1_at	3.74	5.03	4.45	3.55	3.93	4.14	4.41	0.65	3.87	0.30	0.88	-0.187	2.6E-01
Mtr.49432.1.S1_at	2.64	3.76	2.63	2.63	2.63	2.63	3.01	0.65	2.63	0.00	0.87	-0.196	3.6E-01
Mtr.31309.1.S1_s_at	6.66	5.59	6.76	7.03	6.84	6.77	6.34	0.65	6.88	0.14	1.09	0.119	2.3E-01
Mtr.15895.1.S1_s_at	11.03	9.76	10.65	10.24	11.11	10.64	10.48	0.65	10.66	0.43	1.02	0.025	7.1E-01
Mtr.33303.1.S1_at	6.33	5.16	6.22	6.28	6.32	6.29	5.90	0.65	6.30	0.02	1.07	0.093	3.5E-01
Mtr.17673.1.S1_at	6.62	5.73	7.00	6.36	7.15	6.87	6.45	0.65	6.79	0.40	1.05	0.075	4.8E-01
Mtr.37848.1.S1_at	8.48	7.75	9.04	8.51	8.44	8.39	8.43	0.65	8.44	0.06	1.00	0.003	9.6E-01
Mtr.50365.1.S1_at	10.40	9.13	9.52	9.59	9.63	9.62	9.68	0.65	9.61	0.03	0.99	-0.011	8.6E-01
Mtr.21493.1.S1_at	2.17	3.28	2.15	2.15	2.15	2.15	2.53	0.65	2.15	0.00	0.85	-0.237	3.6E-01
Mtr.2302.1.S1_s_at	8.28	7.11	8.19	8.19	8.30	8.29	7.86	0.65	8.26	0.06	1.05	0.072	3.5E-01
Mtr.12534.1.S1_at	6.47	5.42	6.61	6.63	6.22	6.49	6.17	0.65	6.45	0.21	1.05	0.064	5.2E-01
Mtr.24055.1.S1_at	2.80	3.90	2.76	2.75	2.75	2.74	3.15	0.65	2.75	0.01	0.87	-0.200	3.4E-01
Mtr.9331.1.S1_at	6.80	5.72	6.89	6.93	7.12	7.03	6.47	0.65	7.03	0.09	1.09	0.119	2.1E-01
Mtr.44182.1.S1_s_at	12.21	11.18	11.02	11.20	10.80	11.01	11.47	0.65	11.00	0.20	0.96	-0.060	3.0E-01
Mtr.1274.1.S1_at	7.98	7.36	8.65	8.11	8.83	7.78	8.00	0.65	8.24	0.54	1.03	0.043	6.4E-01
Mtr.39040.1.S1_s_at	8.47	7.46	8.67	8.57	8.55	8.34	8.20	0.65	8.48	0.12	1.03	0.050	4.9E-01
Mtr.6655.1.S1_at	9.96	8.78	9.84	9.70	9.69	9.56	9.53	0.65	9.65	0.08	1.01	0.019	7.6E-01
Mtr.43734.1.S1_at	9.62	8.47	9.57	9.76	9.70	9.88	9.22	0.65	9.78	0.09	1.06	0.085	2.1E-01
Mtr.20639.1.S1_s_at	2.71	3.83	2.70	2.68	2.65	2.67	3.08	0.65	2.67	0.01	0.87	-0.208	3.3E-01
Mtr.25464.1.S1_at	8.96	7.68	8.49	8.56	8.54	8.56	8.38	0.65	8.55	0.01	1.02	0.030	6.7E-01
Mtr.2558.1.S1_at	5.42	4.19	5.16	4.97	5.40	5.12	4.92	0.65	5.16	0.22	1.05	0.069	5.8E-01
Mtr.32566.1.S1_s_at	3.18	1.94	2.88	3.40	3.75	3.17	2.66	0.65	3.44	0.29	1.29	0.369	1.3E-01
Mtr.37200.1.S1_at	7.77	6.88	8.13	7.85	7.92	7.92	7.59	0.65	7.89	0.04	1.04	0.056	4.7E-01
Mtr.37078.1.S1_at	8.86	8.63	9.85	9.55	9.35	9.31	9.11	0.65	9.40	0.13	1.03	0.045	4.9E-01
Mtr.33195.1.S1_at	7.26	6.29	7.51	7.52	7.29	6.95	7.02	0.65	7.26	0.29	1.03	0.048	5.9E-01
Mtr.12146.1.S1_at	9.47	9.02	10.29	9.46	9.50	9.18	9.59	0.65	9.38	0.18	0.98	-0.033	6.1E-01
Mtr.45300.1.S1_at	9.79	9.15	10.44	9.98	10.34	10.01	9.79	0.65	10.11	0.20	1.03	0.046	4.6E-01
Mtr.9514.1.S1_at	10.64	10.87	9.65	9.79	10.19	10.25	10.39	0.65	10.07	0.25	0.97	-0.044	4.8E-01
Mtr.50780.1.S1_at	4.44	3.20	3.49	3.53	3.55	3.62	3.71	0.65	3.57	0.04	0.96	-0.057	7.2E-01
Mtr.32731.1.S1_at	7.01	6.00	7.21	7.18	7.12	7.22	6.74	0.65	7.17	0.05	1.06	0.090	3.1E-01

Mtr.4430.1.S1_at	7.06	5.78	6.57	6.57	6.57	6.55	6.47	0.65	6.56	0.01	1.01	0.020	8.2E-01
Mtr.40893.1.S1_at	3.86	2.88	4.10	3.94	4.07	5.24	3.61	0.65	4.42	0.72	1.22	0.290	2.2E-01
Mtr.10325.1.S1_at	9.53	8.88	10.17	10.83	10.38	10.66	9.53	0.65	10.63	0.23	1.12	0.158	5.0E-02
Mtr.28435.1.S1_at	4.46	3.23	3.50	4.76	4.36	4.68	3.73	0.65	4.60	0.21	1.23	0.300	9.3E-02
Mtr.6823.1.S1_at	5.96	6.98	5.79	6.08	5.55	6.00	6.24	0.65	5.87	0.29	0.94	-0.088	4.2E-01
Mtr.1617.1.S1_s_at	9.16	8.09	9.25	8.84	9.15	8.90	8.84	0.65	8.97	0.16	1.01	0.021	7.5E-01
Mtr.15742.1.S1_at	10.20	9.62	10.91	10.38	10.10	10.28	10.24	0.65	10.25	0.14	1.00	0.001	9.8E-01
Mtr.20515.1.S1_at	7.86	6.82	8.00	7.80	7.92	7.84	7.56	0.65	7.86	0.06	1.04	0.056	4.7E-01
Mtr.1982.1.S1_at	4.21	2.93	3.49	3.62	5.36	4.81	3.54	0.65	4.59	0.89	1.30	0.375	1.7E-01
Mtr.39819.1.S1_at	8.38	7.28	8.42	8.38	8.38	8.37	8.03	0.65	8.38	0.00	1.04	0.061	4.0E-01
Mtr.35087.1.S1_s_at	7.13	6.04	7.19	6.99	7.40	7.35	6.79	0.65	7.25	0.23	1.07	0.094	3.1E-01
Mtr.28938.1.S1_at	9.43	8.50	8.19	8.91	8.57	8.84	8.71	0.65	8.77	0.18	1.01	0.011	8.8E-01
Mtr.32521.1.S1_s_at	7.05	6.05	7.27	7.30	7.28	7.22	6.79	0.65	7.27	0.04	1.07	0.098	2.7E-01
Mtr.11220.1.S1_at	10.87	9.58	10.23	11.06	10.73	11.18	10.23	0.65	10.99	0.23	1.07	0.104	1.3E-01
Mtr.9382.1.S1_at	10.83	9.93	11.19	11.05	11.30	11.07	10.65	0.65	11.14	0.14	1.05	0.065	2.7E-01
Mtr.18236.1.S1_at	8.55	7.34	8.34	9.03	9.19	9.06	8.08	0.65	9.09	0.08	1.13	0.171	5.4E-02
Mtr.29960.1.S1_at	4.80	3.52	3.99	4.09	4.24	4.10	4.11	0.65	4.15	0.08	1.01	0.014	9.2E-01
Mtr.42256.1.S1_at	9.25	9.02	8.03	8.14	8.45	8.51	8.77	0.65	8.37	0.20	0.95	-0.068	3.6E-01
Mtr.15517.1.S1_at	3.06	4.09	2.90	2.98	2.93	2.93	3.35	0.65	2.94	0.03	0.88	-0.186	3.4E-01
Mtr.38329.1.S1_at	8.64	7.55	8.69	8.60	8.75	8.51	8.29	0.65	8.62	0.12	1.04	0.055	4.4E-01
Mtr.43442.1.S1_at	7.50	7.27	8.49	7.53	7.27	7.16	7.75	0.65	7.32	0.19	0.94	-0.084	3.2E-01
Mtr.16408.1.S1_at	8.08	7.15	8.40	8.35	8.63	8.27	7.88	0.65	8.42	0.19	1.07	0.096	2.3E-01
Mtr.33279.1.S1_at	8.50	9.78	9.32	9.44	9.67	9.46	9.20	0.64	9.52	0.13	1.04	0.050	4.4E-01
Mtr.23144.1.S1_at	3.44	4.54	3.40	3.34	3.28	3.28	3.79	0.64	3.30	0.04	0.87	-0.201	2.6E-01
Mtr.45436.1.S1_at	5.60	5.63	6.73	7.57	7.88	7.18	5.99	0.64	7.54	0.35	1.26	0.333	2.1E-02
Mtr.1038.1.S1_at	7.59	6.32	7.16	7.24	7.40	7.30	7.03	0.64	7.32	0.08	1.04	0.059	4.8E-01
Mtr.44481.1.S1_at	6.21	5.41	6.68	6.22	6.09	6.45	6.10	0.64	6.25	0.18	1.03	0.036	7.1E-01
Mtr.41718.1.S1_at	8.39	7.34	8.51	8.35	8.36	8.36	8.08	0.64	8.36	0.01	1.03	0.048	5.0E-01
Mtr.10265.1.S1_at	10.25	9.03	9.27	9.32	9.46	9.31	9.52	0.64	9.36	0.09	0.98	-0.023	7.0E-01
Mtr.48146.1.S1_s_at	8.48	7.26	8.21	8.08	7.73	8.17	7.98	0.64	8.00	0.23	1.00	0.002	9.8E-01
Mtr.22400.1.S1_at	3.06	4.18	3.06	3.05	2.99	2.98	3.43	0.64	3.01	0.04	0.88	-0.191	3.2E-01
Mtr.5001.1.S1_at	6.94	5.82	6.93	6.90	7.19	6.92	6.56	0.64	7.00	0.16	1.07	0.094	3.1E-01
Mtr.17339.1.S1_at	8.30	8.32	9.42	8.61	8.64	8.61	8.68	0.64	8.62	0.02	0.99	-0.010	8.8E-01
Mtr.10464.1.S1_at	10.38	9.51	10.77	10.24	10.01	9.55	10.22	0.64	9.93	0.35	0.97	-0.041	5.3E-01
Mtr.45321.1.S1_at	3.86	2.65	3.63	3.88	3.63	3.63	3.38	0.64	3.71	0.15	1.10	0.135	4.3E-01
Mtr.30751.1.S1_at	8.81	7.73	8.88	8.88	8.38	8.54	8.48	0.64	8.60	0.26	1.01	0.021	7.7E-01
Mtr.40843.1.S1_at	9.83	11.11	10.37	9.66	9.92	9.77	10.44	0.64	9.78	0.13	0.94	-0.094	1.6E-01
Mtr.15729.1.S1_at	8.86	7.63	8.57	8.73	8.64	8.73	8.35	0.64	8.70	0.05	1.04	0.059	4.0E-01
Mtr.13355.1.S1_at	6.28	5.19	6.32	6.24	6.35	6.27	5.93	0.64	6.29	0.06	1.06	0.085	3.9E-01
Mtr.39541.1.S1_at	8.26	7.13	8.22	8.28	8.17	8.46	7.87	0.64	8.30	0.15	1.05	0.077	3.2E-01
Mtr.14711.1.S1_at	9.14	7.98	9.04	8.90	8.93	9.00	8.72	0.64	8.94	0.05	1.03	0.037	5.8E-01
Mtr.7250.1.S1_at	2.39	3.50	2.38	2.37	2.36	2.38	2.76	0.64	2.37	0.01	0.86	-0.218	3.6E-01
Mtr.50038.1.S1_at	11.06	10.12	9.83	10.53	10.85	10.54	10.33	0.64	10.64	0.18	1.03	0.042	4.7E-01
Mtr.6191.1.S1_at	4.80	3.54	3.94	4.83	4.75	4.52	4.09	0.64	4.70	0.16	1.15	0.200	1.9E-01
Mtr.17046.1.S1_at	1.83	2.94	1.82	1.82	1.81	1.82	2.19	0.64	1.81	0.00	0.83	-0.273	3.7E-01
Mtr.48549.1.S1_at	4.62	3.88	5.16	4.78	5.46	5.83	4.56	0.64	5.36	0.53	1.18	0.234	1.7E-01
Mtr.15624.1.S1_at	6.51	5.46	6.62	7.01	7.35	6.64	6.20	0.64	7.00	0.36	1.13	0.176	1.3E-01
Mtr.42926.1.S1_s_at	13.88	12.65	13.61	13.87	14.36	14.30	13.38	0.64	14.17	0.27	1.06	0.083	1.2E-01
Mtr.31065.1.S1_at	6.17	5.28	6.52	6.43	6.57	6.14	5.99	0.64	6.38	0.22	1.06	0.091	3.8E-01
Mtr.14007.1.S1_s_at	7.43	6.42	7.62	7.56	7.24	7.51	7.16	0.64	7.44	0.17	1.04	0.055	5.1E-01
Mtr.43055.1.S1_s_at	11.85	10.96	10.59	11.07	10.77	11.29	11.13	0.64	11.04	0.26	0.99	-0.012	8.3E-01
Mtr.41966.1.S1_at	5.53	6.62	5.49	5.52	5.52	5.70	5.88	0.64	5.58	0.10	0.95	-0.077	4.6E-01
Mtr.5770.1.S1_at	3.85	2.91	2.63	2.63	2.62	2.60	3.13	0.64	2.62	0.02	0.84	-0.259	2.4E-01
Mtr.41088.1.S1_at	10.65	9.49	10.55	10.52	10.75	10.56	10.23	0.64	10.61	0.12	1.04	0.052	3.7E-01
Mtr.27938.1.S1_at	8.35	7.30	8.47	8.59	8.46	8.22	8.04	0.64	8.42	0.19	1.05	0.067	3.8E-01
Mtr.27259.1.S1_at	7.62	6.65	7.86	7.82	8.15	7.74	7.38	0.64	7.90	0.22	1.07	0.099	2.5E-01
Mtr.33763.1.S1_at	4.48	5.46	5.68	5.70	5.20	5.60	5.21	0.64	5.50	0.26	1.06	0.079	5.0E-01
Mtr.42701.1.S1_at	2.02	3.13	2.01	2.01	2.01	2.00	2.39	0.64	2.01	0.00	0.84	-0.251	3.6E-01
Mtr.50261.1.S1_at	6.09	5.97	7.14	6.30	6.16	5.99	6.40	0.64	6.15	0.15	0.96	-0.058	5.4E-01
Mtr.41812.1.S1_at	8.52	7.53	8.73	8.33	8.42	8.38	8.26	0.64	8.38	0.04	1.01	0.021	7.7E-01
Mtr.21562.1.S1_at	2.50	3.61	2.49	2.49	2.46	2.49	2.87	0.64	2.48	0.01	0.86	-0.210	3.5E-01
Mtr.29915.1.S1_at	3.76	2.77	2.55	2.60	2.58	2.53	3.03	0.64	2.57	0.04	0.85	-0.236	2.9E-01
Mtr.32819.1.S1_at	2.90	2.97	1.83	2.95	4.03	3.18	2.57	0.64	3.38	0.57	1.32	0.398	1.7E-01
Mtr.21663.1.S1_at	8.60	7.34	8.16	8.08	8.22	8.08	8.03	0.64	8.13	0.08	1.01	0.017	8.2E-01
Mtr.29156.1.S1_at	4.33	5.37	5.50	5.28	5.61	5.06	5.06	0.64	5.32	0.28	1.05	0.070	5.7E-01
Mtr.13468.1.S1_s_at	10.48	9.24	9.60	10.16	9.76	10.36	9.77	0.64	10.09	0.31	1.03	0.047	4.8E-01
Mtr.5671.1.S1_at	10.46	9.35	10.47	10.63	10.37	10.48	10.09	0.64	10.49	0.13	1.04	0.056	3.5E-01
Mtr.29090.1.S1_at	6.63	5.35	5.94	6.24	6.39	6.24	5.97	0.64	6.29	0.09	1.05	0.075	4.4E-01
Mtr.11274.1.S1_at	7.21	5.99	6.94	6.52	6.91	7.08	6.72	0.64	6.84	0.29	1.02	0.026	7.8E-01
Mtr.24867.1.S1_at	2.70	3.83	2.74	2.69	2.66	2.66	3.09	0.64	2.67	0.02	0.86	-0.212	3.2E-01
Mtr.40917.1.S1_at	8.93	7.67	8.07	8.35	8.81	8.78	8.22	0.64	8.65	0.26	1.05	0.072	3.5E-01
Mtr.38458.1.S1_at	8.50	7.67	8.93	8.52	8.48	8.40	8.37	0.64	8.46	0.06	1.01	0.017	8.0E-01
Mtr.12421.1.S1_at	9.19	8.37	9.63	9.19	8.89	8.96	9.06	0.64	9.01	0.16	0.99	-0.008	9.0E-01
Mtr.22815.1.S1_at	3.34	3.41	4.48	4.01	3.77	4.21	3.74	0.64	4.00	0.22	1.07	0.095	5.5E-01
Mtr.3088.1.S1_at	5.66	4.46	5.46	5.54	5.68	5.54	5.20	0.64	5.59	0.09	1.08	0.104	3.6E-01
Mtr.35101.1.S1_at	7.93	6.73	7.74	7.92	7.73	8.01	7.47	0.64	7.88	0.14	1.06	0.079	3.3E-01
Mtr.12685.1.S1_at	10.02	8.74	9.37	9.54	9.47	9.70	9.38	0.64	9.57	0.11	1.02	0.029	6.3E-01
Mtr.38694.1.S1_at	7.69	6.55	7.62	7.66	7.52	7.96	7.29	0.64	7.71	0.23	1.06	0.082	3.4E-01
Mtr.16833.1.S1_at	3.49	4.51	3.32	3.45	3.47	3.34	3.77	0.64	3.42	0.07	0.91	-0.142	4.0E-01
Mtr.41116.1.S1_at	8.02	8.04	9.14	7.85	7.93	7.70	8.40	0.64	7.83	0.12	0.93	-0.101	2.0E-01
Mtr.8771.1.S1_at	8.05	6.78	7.55	7.08	7.06	7.13	7.46	0.64	7.09	0.04	0.95	-0.073	3.8E-01
Mtr.31251.1.S1_at	8.91	7.85	9.01	8.83	8.84	9.03	8.59	0.64	8.90	0.11	1.04	0.051	4.5E-01
Mtr.38866.1.S1_at	9.09	7.99	9.11	9.44	9.20	9.37	8.73	0.64	9.34	0.12	1.07	0.097	1.8E-01
Mtr.7356.1.S1_at	11.22	10.87	12.11	11.90	11.84	11.71	11.40	0.64	11.82	0.10	1.04	0.052	3.3E-01
Mtr.49671.1.S1_at	5.42	4.81	6.09	5.28	4.82	5.26	5.44	0.64	5.12	0.26	0.94	-0.087	4.7E-01
Mtr.26907.1.S1_at	7.21	6.68	7.96	8.35	8.39	8.31	7.28	0.64	8.35	0.04	1.15	0.197	4.5E-02
Mtr.18480.1.S1_at	9.27	8.06	9.02	9.01	8.94	9.03	8.79	0.64	8.99	0.05	1.02	0.034	6.0E-01
Mtr.6197.1.S1_at	2.87	3.96	2.83	2.81	2.76	3.04	3.22	0.64	2.87	0.15	0.89	-0.165	4.1E-01
Mtr.19344.1.S1_at	6.75	5.86	7.10	6.78	6.83	6.80	6.57	0.64	6.80	0.03	1.04	0.051	5.6E-01
Mtr.7521.1.S1_at	5.28	4.18	5.30	5.53	4.24	5.63	4.92	0.64	5.13	0.78	1.04	0.061	7.3E-01
Mtr.6628.1.S1_at	2.33	3.44	2.32	2.32	2.32	2.32	2.70	0.64	2.32	0.00	0.86	-0.218	3.6E-01
Mtr.9836.1.S1_at	8.15	6.90	7.75	8.04	8.16	8.35	7.60	0.64	8.18	0.16	1.08	0.107	2.0E-01
Mtr.32670.1.S1_at	6.29	5.39	6.63	6.45	6.28	6.54	6.10	0.64	6.42	0.13	1.05	0.074	4.4E-01
Mtr.26486.1.S1_at	4.85	3.72	4.80	5.13	4.84	4.89	4.45	0.64	4.95	0.15	1.11	0.153	2.6E-01
Mtr.10161.1.S1_at	9.38	8.13	8.98	8.95	8.99	8.91	8.83	0.64	8.95	0.04	1.01	0.020	7.5E-01
Mtr.42959.1.S1_at	2.20	3.30	2.19	2.19	2.19	2.17	2.56	0.64	2.18	0.01	0.85	-0.231	3.6E-01
Mtr.23145.1.S1_at	2.80	3.08	4.02	3.58	3.21	2.91	3.30	0.64	3.23	0.34	0.98	-0.031	8.7E-01
Mtr.33661.1.S1_at	7.74	9.02	8.44	8.15	8.46	8.55	8.40	0.64	8.39	0.21	1.00	-0.002	9.8E-01
Mtr.43919.1.S1_at	10.35	9.11	10.01	10.04	10.08	9.79	9.82	0.64	9.97	0.16	1.02	0.022	7.2E-01
Mtr.50478.1.S1_at	13.06	11.80	12.64	12.87	12.14	12.82	12.50	0.64	12.61	0.41	1.01	0.013	8.1E-01
Mtr.3092.1.S1_at	2.16	3.30	2.24	2.14	2.14	2.14	2.57	0.64	2.14	0.00	0.83	-0.261	3.1E-01
Mtr.49783.1.S1_at	8.46	8.80	7.57	8.00	8.66	8.43	8.28	0.64	8.36	0.33	1.01	0.015	8.5E-01
Mtr.41977.1.S1_at	4.56	3.48	4.61	3.54	5.82	4.64	4.22	0.64	4.67	1.14	1.11	0.147	5.8E-01
Mtr.14183.1.S1_at	8.22	8.78	9.49	11.03	9.95	11.02	8.83	0.64	10.67	0.62	1.21	0.272	2.4E-02
Mtr.8901.1.S1_at	11.86	10.83	12.00	11.11	8.92	11.33	11.56	0.64	10.45	1.33	0.90	-0.145	2.6E-01
Mtr.29592.1.S1_at	6.71	5.50	6.45	6.52	6.51	6.41	6.22	0.64	6.48	0.06	1.04	0.059	5.2E-01
Mtr.13795.1.S1_at	5.55	5.54	6.65	5.46	5.52	5.48	5.92	0.64	5.49	0.03	0.93	-0.109	3.1E-01
Mtr.40983.1.S1_at	10.37	9.15	10.08	9.89	9.86	9.84	9.87	0.64	9.86	0.03	1.00	-0.001	9.9E-01
Mtr.18177.1.S1_at	2.95	4.04	2.93	2.90	2.95	2.87	3.31	0.64	2.91	0.04	0.88	-0.185	3.4E-01
Mtr.41871.1.S1_at	2.79	3.84	2.68	2.87	2.67	2.65	3.10	0.64	2.73	0.12	0.88	-0.184	3.8E-01
Mtr.11191.1.S1_at	5.23	4.21	5.38	5.16	5.53	5.10	4.94	0.64	5.26	0.23	1.07	0.091	4.6E-01
Mtr.30152.1.S1_at	7.82	7.00	8.26	7.87	7.94	7.89	7.69	0.64	7.90	0.04	1.03	0.039	6.0E-01
Mtr.31247.1.S1_at	4.12	2.98	4.05	3.83	3.99	4.42	3.72	0.64	4.08	0.30	1.10	0.134	4.3E-01
Mtr.12787.1.S1_at	9.00	7.91	9.04	8.84	9.00	8.95	8.65	0.64	8.93	0.08	1.03	0.046	4.9E-01
Mtr.42975.1.S1_at	7.94	6.77	6.92	7.34	7.13	7.56	7.21	0.64	7.34	0.22	1.02	0.027	7.4E-01
Mtr.27724.1.S1_at	7.25	6.10	7.15	7.25	7.27	7.15	6.83	0.64	7.22	0.06	1.06	0.080	3.5E-01
Mtr.16617.1.S1_at	2.97	3.17	4.16	3.14	3.66	3.12	3.43	0.64	3.31	0.31	0.96	-0.055	7.7E-01
Mtr.39203.1.S1_at	2.45	2.46	3.56	2.44	2.44	2.42	2.83	0.64	2.43	0.01	0.86	-0.215	3.5E-01
Mtr.19055.1.S1_at	2.26	3.36	2.25	2.51	2.24	2.45	2.62	0.64	2.40	0.14	0.92	-0.127	5.9E-01
Mtr.34308.1.S1_at	2.23	3.33	2.22	2.22	2.22	2.22	2.60	0.64	2.22	0.00	0.85	-0.228	3.6E-01
Mtr.39390.1.S1_at	10.46	9.29	9.44	9.51	9.04	9.42	9.73	0.64	9.33	0.25	0.96	-0.060	3.7E-01
Mtr.11042.1.S1_at	9.71	10.88	10.73	9.22	9.34	9.13	10.44	0.64	9.23	0.10	0.88	-0.177	3.2E-02
Mtr.10659.1.S1_s_at	7.90	7.00	8.23	7.09	7.28	6.91	7.71	0.64	7.09	0.19	0.92	-0.120	1.8E-01
Mtr.635.1.S1_at	7.81	6.90	6.58	6.68	6.98	7.24	7.10	0.64	6.97	0.28	0.98	-0.028	7.5E-01
Mtr.11532.1.S1_at	7.88	8.41	7.14	7.31	6.66	6.70	7.81	0.64	6.89	0.36	0.88	-0.181	9.5E-02
Mtr.2640.1.S1_at	8.66	7.84	9.09	8.90	8.84	8.56	8.53	0.64	8.76	0.18	1.03	0.039	5.7E-01
Mtr.8156.1.S1_at	2.67	3.82	2.76	2.85	2.57	2.69	3.08	0.64	2.70	0.14	0.88	-0.190	3.7E-01
Mtr.10780.1.S1_at	4.86	4.11	5.38	5.55	6.53	5.90	4.79	0.64	5.99	0.50	1.25	0.324	6.1E-02
Mtr.47728.1.S1_x_at	9.91	8.67	9.54	10.76	10.60	10.66	9.37	0.64	10.67	0.08	1.14	0.187	2.5E-02
Mtr.4665.1.S1_at	8.29	8.81	9.56	8.32	8.70	8.71	8.88	0.64	8.58	0.22	0.97	-0.051	4.7E-01
Mtr.36057.1.S1_at	2.67	3.74	2.61	2.61	2.61	2.61	3.01	0.64	2.61	0.00	0.87	-0.207	3.4E-01

Mtr.50464.1.S1_at	6.42	5.29	6.38	6.09	5.88	5.32	6.03	0.64	5.76	0.40	0.96	-0.065	5.7E-01
Mtr.43158.1.S1_s_at	9.00	9.02	10.12	9.72	9.32	9.48	9.38	0.64	9.51	0.20	1.01	0.019	7.6E-01
Mtr.994.1.S1_at	9.95	8.68	9.41	9.49	9.29	9.53	9.35	0.64	9.44	0.10	1.01	0.014	8.2E-01
Mtr.37791.1.S1_at	8.36	7.09	7.80	8.19	8.01	8.03	7.75	0.64	8.08	0.10	1.04	0.060	4.2E-01
Mtr.30076.1.S1_at	2.67	2.74	3.81	2.80	2.97	2.88	3.07	0.64	2.88	0.08	0.94	-0.094	6.3E-01
Mtr.39857.1.S1_at	5.29	4.45	5.70	5.70	5.58	5.45	5.15	0.64	5.58	0.13	1.08	0.116	3.2E-01
Mtr.16474.1.S1_at	2.50	3.59	2.48	2.49	2.48	2.47	2.86	0.64	2.48	0.01	0.87	-0.204	3.7E-01
Mtr.28134.1.S1_at	7.77	6.88	8.11	7.85	7.77	7.87	7.59	0.64	7.83	0.05	1.03	0.046	5.5E-01
Mtr.32437.1.S1_at	6.12	5.49	6.77	6.46	6.25	6.61	6.13	0.64	6.44	0.18	1.05	0.072	4.6E-01
Mtr.4332.1.S1_at	2.22	3.32	2.21	2.21	2.21	2.20	2.59	0.64	2.21	0.00	0.85	-0.228	3.6E-01
Mtr.27194.1.S1_at	7.05	5.85	6.83	7.51	7.31	7.03	6.57	0.64	7.28	0.24	1.11	0.148	1.5E-01
Mtr.28579.1.S1_at	8.94	7.85	8.96	9.00	8.99	8.92	8.58	0.64	8.97	0.05	1.05	0.064	3.5E-01
Mtr.47312.1.S1_at	10.76	9.66	9.66	9.85	9.92	9.94	10.03	0.64	9.91	0.05	0.99	-0.018	7.6E-01
Mtr.18923.1.S1_x_at	6.84	7.57	6.30	6.86	6.73	6.71	6.91	0.64	6.77	0.08	0.98	-0.029	7.3E-01
Mtr.5690.1.S1_at	5.86	5.32	6.58	6.13	5.85	5.99	5.92	0.64	5.99	0.14	1.01	0.017	8.6E-01
Mtr.52360.1.S1_at	7.99	7.27	8.54	7.86	8.20	8.03	7.93	0.64	8.03	0.17	1.01	0.018	8.1E-01
Mtr.16778.1.S1_at	7.50	6.57	7.79	7.98	7.71	7.83	7.29	0.64	7.84	0.14	1.08	0.105	2.2E-01
Mtr.27515.1.S1_at	2.80	2.32	3.58	4.11	3.60	3.64	2.90	0.64	3.78	0.28	1.30	0.384	9.3E-02
Mtr.2855.1.S1_s_at	8.11	7.17	8.38	7.74	7.68	7.73	7.88	0.64	7.72	0.03	0.98	-0.030	6.8E-01
Mtr.26376.1.S1_at	9.12	8.60	9.87	9.44	9.64	9.28	9.20	0.64	9.45	0.18	1.03	0.040	5.4E-01
Mtr.43364.1.S1_at	10.01	8.78	9.68	9.91	10.40	10.05	9.49	0.64	10.12	0.25	1.07	0.092	1.9E-01
Mtr.8505.1.S1_at	5.95	5.06	6.29	9.23	11.74	11.44	5.77	0.64	10.80	1.37	1.87	0.905	4.5E-03
Mtr.35350.1.S1_at	7.80	6.67	7.75	7.78	7.68	7.72	7.41	0.64	7.73	0.05	1.04	0.061	4.4E-01
Mtr.34596.1.S1_at	10.51	9.29	10.23	10.16	10.07	10.31	10.01	0.64	10.18	0.12	1.02	0.024	6.7E-01
Mtr.10594.1.S1_at	13.39	12.36	12.23	12.70	12.89	13.00	12.66	0.64	12.86	0.15	1.02	0.023	6.2E-01
Mtr.43960.1.S1_at	8.42	7.19	8.07	8.53	8.24	8.41	7.90	0.64	8.39	0.15	1.06	0.088	2.6E-01
Mtr.12416.1.S1_at	10.19	8.93	9.67	9.71	9.77	9.69	9.60	0.64	9.72	0.04	1.01	0.019	7.4E-01
Mtr.45666.1.S1_at	10.05	11.22	11.07	10.21	10.04	10.15	10.78	0.64	10.13	0.09	0.94	-0.089	1.6E-01
Mtr.32247.1.S1_at	6.45	5.37	6.49	6.59	6.51	6.60	6.10	0.64	6.57	0.05	1.08	0.106	2.8E-01
Mtr.25905.1.S1_s_at	8.40	7.91	9.17	8.77	8.64	8.77	8.49	0.64	8.72	0.07	1.03	0.039	5.6E-01
Mtr.5498.1.S1_at	8.33	7.29	8.44	7.76	7.92	7.87	8.02	0.64	7.85	0.09	0.98	-0.031	6.7E-01
Mtr.40168.1.S1_at	9.34	8.07	8.69	9.24	9.14	9.50	8.70	0.64	9.29	0.18	1.07	0.095	2.0E-01
Mtr.6119.1.S1_at	3.78	3.68	4.83	3.65	4.05	3.52	4.10	0.64	3.74	0.28	0.91	-0.131	4.2E-01
Mtr.26293.1.S1_s_at	6.98	5.76	6.68	6.10	6.14	5.65	6.47	0.64	5.97	0.27	0.92	-0.117	2.7E-01
Mtr.29314.1.S1_at	7.18	6.14	6.04	5.64	6.23	6.16	6.45	0.64	6.01	0.32	0.93	-0.103	3.4E-01
Mtr.34894.1.S1_at	3.78	3.77	4.88	3.94	4.06	3.73	4.14	0.64	3.91	0.17	0.94	-0.082	5.8E-01
Mtr.49836.1.S1_at	2.80	3.89	2.78	2.76	2.72	2.74	3.16	0.64	2.74	0.02	0.87	-0.203	3.2E-01
Mtr.11924.1.S1_at	8.60	8.08	9.35	8.91	8.81	8.93	8.68	0.64	8.89	0.07	1.02	0.034	6.0E-01
Mtr.42206.1.S1_at	9.61	8.47	9.54	9.42	9.49	9.34	9.20	0.64	9.41	0.08	1.02	0.033	6.0E-01
Mtr.7605.1.S1_x_at	9.95	8.78	9.79	9.32	9.31	9.34	9.51	0.64	9.32	0.01	0.98	-0.028	6.4E-01
Mtr.37714.1.S1_at	8.77	9.16	10.02	11.57	12.11	11.70	9.32	0.64	11.79	0.28	1.27	0.340	3.5E-03
Mtr.40763.1.S1_at	10.31	9.13	10.13	10.11	10.07	10.26	9.86	0.64	10.15	0.10	1.03	0.042	4.8E-01
Mtr.12078.1.S1_at	9.84	8.68	9.71	10.10	10.15	10.10	9.41	0.63	10.12	0.03	1.08	0.105	1.3E-01
Mtr.29505.1.S1_s_at	11.17	9.90	10.53	10.72	10.62	10.80	10.54	0.63	10.71	0.09	1.02	0.024	6.6E-01
Mtr.43130.1.S1_s_at	11.24	11.98	12.51	11.47	11.54	11.69	11.91	0.63	11.56	0.12	0.97	-0.042	4.1E-01
Mtr.21461.1.S1_at	11.32	10.10	11.02	11.12	11.12	11.23	10.81	0.63	11.16	0.06	1.03	0.045	4.0E-01
Mtr.25374.1.S1_at	6.89	6.10	7.35	7.18	7.35	7.27	6.78	0.63	7.27	0.08	1.07	0.100	2.6E-01
Mtr.24883.1.S1_at	10.42	9.16	9.90	10.05	9.62	9.77	9.83	0.63	9.82	0.22	1.00	-0.002	9.8E-01
Mtr.1714.1.S1_at	8.05	6.84	7.80	7.98	7.95	8.01	7.56	0.63	7.98	0.03	1.05	0.077	3.2E-01
Mtr.33076.1.S1_at	7.80	6.54	7.31	7.87	7.44	7.82	7.22	0.63	7.71	0.24	1.07	0.096	2.7E-01
Mtr.28159.1.S1_at	7.75	6.75	7.94	8.42	8.57	8.15	7.48	0.63	8.38	0.21	1.12	0.164	8.0E-02
Mtr.46863.1.S1_s_at	13.40	12.70	12.13	12.40	12.54	12.77	12.74	0.63	12.57	0.19	0.99	-0.020	6.7E-01
Mtr.2076.1.S1_at	7.52	6.35	7.34	7.23	7.42	7.21	7.07	0.63	7.28	0.12	1.03	0.043	6.0E-01
Mtr.18080.1.S1_x_at	3.01	4.08	2.96	2.93	2.93	2.92	3.35	0.63	2.93	0.01	0.87	-0.194	3.1E-01
Mtr.41247.1.S1_at	8.36	8.19	9.36	8.34	8.69	8.18	8.64	0.63	8.40	0.26	0.97	-0.040	5.8E-01
Mtr.9775.1.S1_at	2.83	1.99	3.23	2.90	2.75	3.55	2.68	0.63	3.07	0.42	1.14	0.194	4.3E-01
Mtr.8418.1.S1_s_at	4.58	3.62	4.82	4.56	4.56	4.56	4.34	0.63	4.56	0.00	1.05	0.072	5.7E-01
Mtr.6131.1.S1_at	6.56	6.61	7.68	7.42	7.32	7.02	6.95	0.63	7.26	0.21	1.04	0.062	4.7E-01
Mtr.2943.1.S1_at	7.79	7.25	8.52	8.09	7.96	8.00	7.86	0.63	8.02	0.07	1.02	0.030	6.8E-01
Mtr.41901.1.S1_at	8.26	7.35	8.57	8.05	8.39	8.32	8.06	0.63	8.25	0.18	1.02	0.034	6.4E-01
Mtr.10913.1.S1_at	10.53	9.73	10.99	10.98	10.99	10.60	10.42	0.63	10.86	0.22	1.04	0.059	3.2E-01
Mtr.33199.1.S1_at	7.16	6.03	7.09	6.75	7.16	6.87	6.76	0.63	6.93	0.21	1.02	0.035	6.9E-01
Mtr.42600.1.S1_at	9.67	8.42	9.22	9.43	9.61	9.59	9.11	0.63	9.54	0.10	1.05	0.068	3.0E-01
Mtr.44614.1.S1_at	12.57	13.17	13.83	12.74	12.93	12.79	13.19	0.63	12.82	0.10	0.97	-0.041	3.8E-01
Mtr.27324.1.S1_s_at	12.37	11.34	11.22	11.59	11.85	12.04	11.64	0.63	11.83	0.23	1.02	0.023	6.6E-01
Mtr.12349.1.S1_at	10.30	9.45	9.07	9.00	8.99	9.02	9.61	0.63	9.00	0.02	0.94	-0.093	1.8E-01
Mtr.33519.1.S1_at	6.65	6.32	7.55	8.33	8.55	8.44	6.84	0.63	8.44	0.11	1.23	0.303	1.3E-02
Mtr.33323.1.S1_at	4.83	3.60	4.48	4.46	4.80	5.06	4.31	0.63	4.78	0.30	1.11	0.150	3.1E-01
Mtr.18921.1.S1_at	11.15	10.00	11.02	11.43	11.62	11.69	10.72	0.63	11.58	0.13	1.08	0.111	8.4E-02
Mtr.44939.1.S1_at	12.32	11.57	11.06	11.45	11.59	11.73	11.65	0.63	11.59	0.14	0.99	-0.007	8.8E-01
Mtr.22963.1.S1_at	8.30	7.34	8.54	8.06	7.79	7.88	8.06	0.63	7.91	0.14	0.98	-0.027	7.1E-01
Mtr.28149.1.S1_at	7.96	9.07	9.03	11.12	11.65	11.46	8.69	0.63	11.41	0.27	1.31	0.393	2.4E-03
Mtr.24581.1.S1_s_at	9.64	8.40	9.21	8.83	8.60	8.69	9.08	0.63	8.70	0.12	0.96	-0.061	3.7E-01
Mtr.9932.1.S1_at	5.97	5.41	6.67	6.08	6.15	5.96	6.02	0.63	6.06	0.10	1.01	0.011	9.1E-01
Mtr.7090.1.S1_at	6.51	5.38	6.43	6.56	6.43	6.37	6.11	0.63	6.45	0.10	1.06	0.080	4.0E-01
Mtr.11359.1.S1_at	8.27	8.39	9.42	8.82	8.55	8.95	8.69	0.63	8.77	0.20	1.01	0.013	8.4E-01
Mtr.26711.1.S1_at	8.30	9.36	8.24	8.27	8.36	8.34	8.63	0.63	8.32	0.05	0.96	-0.052	4.5E-01
Mtr.42466.1.S1_at	8.52	7.31	7.60	8.88	8.99	9.23	7.81	0.63	9.03	0.18	1.16	0.210	3.2E-02
Mtr.31144.1.S1_at	2.42	3.51	2.41	2.39	2.40	2.40	2.78	0.63	2.40	0.01	0.86	-0.215	3.5E-01
Mtr.7253.1.S1_at	4.48	3.48	4.65	4.73	4.45	4.37	4.20	0.63	4.52	0.19	1.08	0.105	4.5E-01
Mtr.7341.1.S1_at	8.66	7.70	8.90	8.68	8.73	8.15	8.42	0.63	8.52	0.32	1.01	0.017	8.2E-01
Mtr.32713.1.S1_s_at	4.82	5.87	4.73	5.27	4.62	5.54	5.14	0.63	5.14	0.48	1.00	0.001	1.0E+00
Mtr.454.1.S1_at	6.87	5.66	6.58	6.77	6.69	6.48	6.37	0.63	6.64	0.15	1.04	0.060	5.1E-01
Mtr.29863.1.S1_at	8.18	7.87	9.09	8.64	8.15	8.48	8.38	0.63	8.42	0.25	1.01	0.007	9.2E-01
Mtr.43335.1.S1_at	12.86	11.60	12.25	12.36	12.06	12.48	12.23	0.63	12.30	0.22	1.01	0.008	8.7E-01
Mtr.39255.1.S1_s_at	7.55	6.31	7.13	7.21	7.24	7.14	7.00	0.63	7.20	0.05	1.03	0.040	6.2E-01
Mtr.43495.1.S1_at	9.33	8.12	8.42	8.86	8.74	9.06	8.62	0.63	8.89	0.17	1.03	0.044	5.2E-01
Mtr.44429.1.S1_at	11.12	9.88	10.31	10.73	10.94	10.89	10.44	0.63	10.85	0.11	1.04	0.056	3.3E-01
Mtr.27852.1.S1_at	4.22	4.04	5.21	4.68	4.21	4.15	4.49	0.63	4.34	0.29	0.97	-0.049	7.3E-01
Mtr.35933.1.S1_at	6.12	5.13	6.31	6.29	6.03	6.05	5.85	0.63	6.12	0.14	1.05	0.065	5.1E-01
Mtr.40073.1.S1_at	7.81	7.85	8.92	9.90	10.26	10.08	8.19	0.63	10.08	0.18	1.23	0.299	7.6E-03
Mtr.26021.1.S1_x_at	2.77	3.85	2.74	2.72	2.71	2.68	3.12	0.63	2.70	0.02	0.87	-0.208	3.2E-01
Mtr.33650.1.S1_at	9.16	8.05	9.13	9.31	9.30	9.17	8.78	0.63	9.26	0.07	1.05	0.077	2.6E-01
Mtr.5716.1.S1_at	6.27	5.47	6.72	6.68	6.67	6.69	6.15	0.63	6.68	0.01	1.09	0.119	2.2E-01
Mtr.41843.1.S1_at	7.58	6.90	8.16	7.64	7.60	7.72	7.54	0.63	7.66	0.06	1.01	0.021	7.8E-01
Mtr.11627.1.S1_s_at	3.31	4.38	3.27	3.26	3.07	3.15	3.65	0.63	3.16	0.09	0.86	-0.210	2.5E-01
Mtr.39710.1.S1_at	10.54	9.96	11.22	11.59	11.78	11.54	10.58	0.63	11.64	0.13	1.10	0.138	4.6E-02
Mtr.13341.1.S1_at	8.89	7.84	8.97	8.77	9.03	8.75	8.57	0.63	8.85	0.16	1.03	0.047	4.9E-01
Mtr.48412.1.S1_s_at	11.31	10.21	10.22	10.99	11.88	11.42	10.58	0.63	11.43	0.44	1.08	0.112	1.3E-01
Mtr.29082.1.S1_at	9.25	7.99	8.66	9.45	9.05	9.42	8.63	0.63	9.31	0.22	1.08	0.108	1.6E-01
Mtr.17317.1.S1_at	7.79	8.42	9.05	8.44	8.34	8.29	8.42	0.63	8.36	0.07	0.99	-0.011	8.7E-01
Mtr.14669.1.S1_at	7.82	7.11	8.36	8.21	8.19	8.15	7.76	0.63	8.18	0.03	1.05	0.076	3.1E-01
Mtr.19483.1.S1_at	10.34	9.23	10.30	10.63	10.32	10.43	9.96	0.63	10.46	0.16	1.05	0.071	2.5E-01
Mtr.38604.1.S1_at	10.10	8.98	10.04	9.80	9.82	10.03	9.71	0.63	9.89	0.12	1.02	0.026	6.6E-01
Mtr.39641.1.S1_at	8.76	7.83	9.04	8.90	8.93	8.95	8.54	0.63	8.93	0.02	1.04	0.063	3.5E-01
Mtr.49242.1.S1_at	3.46	4.54	3.44	3.40	3.35	3.40	3.81	0.63	3.38	0.03	0.89	-0.173	3.0E-01
Mtr.27699.1.S1_s_at	9.42	8.63	9.88	9.63	9.82	9.48	9.31	0.63	9.64	0.17	1.04	0.051	4.2E-01
Mtr.46706.1.S1_at	4.76	5.60	4.36	4.84	4.35	4.46	4.91	0.63	4.55	0.26	0.93	-0.109	4.1E-01
Mtr.39283.1.S1_at	7.97	7.57	8.81	8.21	8.40	8.35	8.11	0.63	8.32	0.10	1.03	0.036	6.0E-01
Mtr.9711.1.S1_at	10.09	8.84	9.59	9.81	9.87	9.85	9.51	0.63	9.85	0.03	1.04	0.050	4.1E-01
Mtr.31337.1.S1_at	8.59	7.37	8.24	8.13	8.15	8.30	8.07	0.63	8.19	0.09	1.02	0.022	7.6E-01
Mtr.41375.1.S1_at	9.15	8.33	9.56	9.16	9.06	9.15	9.01	0.63	9.12	0.06	1.01	0.018	7.8E-01
Mtr.5765.1.S1_at	10.23	8.97	9.56	9.60	9.42	9.65	9.58	0.63	9.55	0.12	1.00	-0.005	9.4E-01
Mtr.44000.1.S1_at	7.63	6.39	7.22	7.24	7.30	7.33	7.08	0.63	7.29	0.05	1.03	0.043	5.9E-01
Mtr.24523.1.S1_at	9.50	10.68	9.70	8.99	9.88	9.56	9.96	0.63	9.48	0.45	0.95	-0.072	3.4E-01
Mtr.40185.1.S1_at	11.11	10.32	9.86	10.16	10.10	10.21	10.43	0.63	10.16	0.06	0.97	-0.039	4.9E-01
Mtr.6323.1.S1_at	2.16	3.24	2.15	2.14	2.14	2.14	2.52	0.63	2.14	0.00	0.85	-0.233	3.6E-01
Mtr.39411.1.S1_at	8.54	7.30	8.13	8.14	7.85	8.21	7.99	0.63	8.06	0.19	1.01	0.013	8.6E-01
Mtr.6733.1.S1_at	9.86	8.61	9.36	9.24	9.08	9.06	9.28	0.63	9.13	0.10	0.98	-0.023	7.1E-01
Mtr.8745.1.S1_at	11.87	10.62	11.13	11.70	11.73	11.80	11.21	0.63	11.74	0.06	1.05	0.067	2.2E-01
Mtr.2752.1.S1_at	2.20	3.28	2.18	2.18	2.18	2.17	2.55	0.63	2.17	0.01	0.85	-0.232	3.6E-01
Mtr.21374.1.S1_at	5.79	6.29	7.04	5.65	5.54	5.51	6.38	0.63	5.57	0.07	0.87	-0.196	9.1E-02
Mtr.32001.1.S1_at	2.71	3.79	2.68	2.66	2.65	2.64	3.06	0.63	2.65	0.01	0.87	-0.208	3.2E-01
Mtr.13120.1.S1_at	4.25	3.07	4.02	4.29	4.42	3.95	3.78	0.63	4.22	0.24	1.12	0.159	3.2E-01
Mtr.28374.1.S1_at	2.49	3.57	2.48	2.55	2.46	2.44	2.85	0.63	2.48	0.06	0.87	-0.197	3.8E-01
Mtr.43258.1.S1_s_at	11.45	11.27	10.28	11.04	10.76	10.90	11.00	0.63	10.90	0.14	0.99	-0.013	8.1E-01
Mtr.24691.1.S1_at	2.80	3.84	2.71	2.68	2.71	2.70	3.12	0.63	2.70	0.02	0.87	-0.207	3.1E-01
Mtr.23932.1.S1_at	3.01	4.08	2.98	2.98	2.99	3.18	3.36	0.63	3.05	0.12	0.91	-0.140	4.5E-01
Mtr.43714.1.S1_at	9.11	7.87	8.66	9.07	8.94	9.08	8.54	0.63	9.03	0.08	1.06	0.080	2.5E-01
Mtr.5961.1.S1_x_at	7.49	6.53	6.31	7.50	7.69	7.48	6.77	0.63	7.56	0.12	1.12	0.158	1.0E-01
Mtr.33929.1.S1_s_at	10.82	9.63	9.89	10.38	10.29	10.63	10.11	0.63	10.43	0.18	1.03	0.045	4.4E-01

Mtr.28137.1.S1_at	5.75	4.65	5.73	5.62	5.78	6.06	5.38	0.63	5.82	0.22	1.08	0.113	3.2E-01
Mtr.6307.1.S1_at	4.46	3.22	4.05	4.29	5.66	4.66	3.91	0.63	4.87	0.71	1.25	0.317	1.5E-01
Mtr.39861.1.S1_at	8.52	7.29	7.69	8.25	8.32	8.59	7.83	0.63	8.39	0.18	1.07	0.099	2.2E-01
Mtr.32436.1.S1_at	5.57	4.46	5.52	5.57	5.25	5.56	5.18	0.63	5.46	0.18	1.05	0.076	5.0E-01
Mtr.27095.1.S1_s_at	8.22	7.23	8.40	8.14	8.48	8.43	7.95	0.63	8.35	0.18	1.05	0.071	3.5E-01
Mtr.34697.1.S1_at	3.93	2.69	3.14	2.81	2.89	2.88	3.25	0.63	2.86	0.04	0.88	-0.188	3.3E-01
Mtr.35670.1.S1_at	11.27	10.02	10.61	11.01	10.87	11.00	10.63	0.63	10.96	0.08	1.03	0.044	4.2E-01
Mtr.15708.1.S1_at	7.93	9.18	8.66	8.01	8.56	8.30	8.59	0.63	8.29	0.28	0.96	-0.052	4.9E-01
Mtr.18949.1.S1_at	8.88	7.80	8.88	8.80	8.87	8.63	8.52	0.63	8.77	0.12	1.03	0.041	5.4E-01
Mtr.10230.1.S1_at	8.16	6.92	7.72	7.93	8.06	8.03	7.60	0.63	8.01	0.07	1.05	0.075	3.3E-01
Mtr.33398.1.S1_s_at	10.60	9.47	9.57	10.13	10.07	10.21	9.88	0.63	10.14	0.07	1.03	0.037	5.2E-01
Mtr.2918.1.S1_at	4.37	4.22	5.37	5.07	5.50	5.03	4.65	0.63	5.20	0.26	1.12	0.160	2.4E-01
Mtr.45242.1.S1_at	9.60	8.36	8.80	8.68	8.80	8.91	8.92	0.63	8.79	0.11	0.99	-0.020	7.5E-01
Mtr.14186.1.S1_at	12.57	11.32	12.02	12.38	12.27	12.33	11.97	0.63	12.33	0.06	1.03	0.043	3.8E-01
Mtr.38509.1.S1_at	5.53	4.29	5.08	4.68	5.07	5.27	4.97	0.63	5.01	0.30	1.01	0.011	9.3E-01
Mtr.43532.1.S1_at	2.79	3.87	2.77	2.76	2.76	2.75	3.14	0.63	2.76	0.00	0.88	-0.189	3.5E-01
Mtr.33011.1.S1_at	9.74	8.62	9.67	9.66	9.15	9.46	9.34	0.63	9.42	0.26	1.01	0.013	8.4E-01
Mtr.25322.1.S1_at	8.46	7.50	8.67	8.49	8.38	8.46	8.21	0.63	8.44	0.06	1.03	0.041	5.5E-01
Mtr.17484.1.S1_at	7.36	6.42	7.61	7.41	7.27	7.28	7.13	0.63	7.32	0.08	1.03	0.037	6.3E-01
Mtr.26440.1.S1_at	3.98	2.95	2.85	2.84	2.88	4.25	3.26	0.63	3.32	0.81	1.02	0.030	9.1E-01
Mtr.21855.1.S1_at	8.34	7.28	8.39	8.38	8.17	8.23	8.00	0.63	8.26	0.11	1.03	0.046	5.2E-01
Mtr.31385.1.S1_at	10.44	9.25	10.20	10.20	10.21	10.23	9.96	0.63	10.21	0.01	1.02	0.035	5.3E-01
Mtr.28524.1.S1_at	8.83	7.61	8.46	8.53	8.75	8.56	8.30	0.63	8.61	0.12	1.04	0.053	4.4E-01
Mtr.14599.1.S1_at	8.30	9.16	9.52	9.12	9.27	8.23	8.99	0.63	8.87	0.56	0.99	-0.019	8.2E-01
Mtr.9002.1.S1_at	4.60	4.20	5.43	5.47	5.10	5.52	4.74	0.63	5.37	0.23	1.13	0.178	1.8E-01
Mtr.18667.1.S1_at	5.39	4.24	5.24	5.22	5.34	4.96	4.96	0.63	5.17	0.20	1.04	0.062	6.0E-01
Mtr.40439.1.S1_at	7.50	6.25	6.97	6.94	6.70	6.97	6.90	0.63	6.87	0.15	0.99	-0.008	9.2E-01
Mtr.38321.1.S1_at	10.58	9.46	10.51	10.49	10.47	10.26	10.18	0.63	10.41	0.13	1.02	0.032	5.7E-01
Mtr.12788.1.S1_at	9.96	8.77	9.70	9.82	9.74	9.93	9.48	0.63	9.83	0.10	1.04	0.053	3.9E-01
Mtr.35600.1.S1_at	8.16	6.91	7.58	7.45	7.40	7.30	7.55	0.63	7.38	0.07	0.98	-0.032	6.7E-01
Mtr.34686.1.S1_at	7.52	6.69	7.92	7.72	7.94	7.44	7.38	0.63	7.70	0.25	1.04	0.062	4.5E-01
Mtr.29254.1.S1_s_at	7.38	6.59	7.82	7.63	7.70	7.69	7.26	0.63	7.67	0.04	1.06	0.079	3.2E-01
Mtr.27200.1.S1_at	4.78	3.54	4.25	4.23	4.61	4.19	4.19	0.63	4.34	0.23	1.04	0.052	7.1E-01
Mtr.14048.1.S1_s_at	9.61	10.40	10.84	10.07	10.77	10.22	10.28	0.63	10.35	0.37	1.01	0.010	8.8E-01
Mtr.14421.1.S1_s_at	8.72	8.17	9.42	9.08	9.11	8.87	8.77	0.63	9.02	0.13	1.03	0.041	5.3E-01
Mtr.12180.1.S1_at	7.43	7.26	8.42	8.14	8.06	8.15	7.70	0.63	8.12	0.05	1.05	0.075	3.2E-01
Mtr.48267.1.S1_at	9.72	9.07	10.32	9.95	9.95	9.83	9.70	0.63	9.91	0.07	1.02	0.031	5.9E-01
Mtr.11416.1.S1_at	9.22	8.60	9.85	9.24	9.20	8.97	9.23	0.63	9.14	0.14	0.99	-0.014	8.2E-01
Mtr.27400.1.S1_at	6.07	4.87	5.79	6.06	5.90	6.76	5.58	0.63	6.24	0.46	1.12	0.162	2.1E-01
Mtr.25033.1.S1_at	13.64	12.49	12.63	13.15	13.05	13.22	12.92	0.63	13.14	0.08	1.02	0.024	5.8E-01
Mtr.37415.1.S1_s_at	14.82	13.92	15.12	15.12	14.90	15.06	14.62	0.63	15.03	0.12	1.03	0.040	3.3E-01
Mtr.33126.1.S1_at	4.55	3.38	4.36	4.43	4.32	4.28	4.10	0.63	4.34	0.08	1.06	0.084	5.4E-01
Mtr.5356.1.S1_s_at	10.60	11.66	10.55	10.31	10.52	10.54	10.94	0.63	10.46	0.12	0.96	-0.064	2.6E-01
Mtr.43347.1.S1_s_at	12.73	11.53	11.83	12.56	12.90	12.71	12.03	0.63	12.72	0.17	1.06	0.080	1.4E-01
Mtr.24232.1.S1_at	3.02	4.16	3.14	3.03	3.00	2.99	3.44	0.63	3.01	0.02	0.87	-0.195	2.9E-01
Mtr.32853.1.S1_at	7.78	6.54	7.03	7.64	7.49	7.64	7.12	0.63	7.59	0.09	1.07	0.093	2.6E-01
Mtr.14096.1.S1_at	8.90	7.77	8.80	8.53	8.73	8.58	8.49	0.63	8.61	0.11	1.01	0.021	7.5E-01
Mtr.10887.1.S1_at	7.14	6.29	7.51	7.05	7.13	7.01	6.98	0.63	7.06	0.06	1.01	0.018	8.3E-01
Mtr.40144.1.S1_at	14.97	15.00	13.91	14.25	14.43	14.30	14.63	0.62	14.33	0.09	0.98	-0.030	4.6E-01
Mtr.29283.1.S1_at	5.00	4.28	5.53	4.72	4.59	4.69	4.94	0.62	4.67	0.07	0.95	-0.081	5.0E-01
Mtr.16615.1.S1_at	11.75	10.50	11.04	10.97	10.88	11.06	11.10	0.62	10.97	0.09	0.99	-0.017	7.5E-01
Mtr.33380.1.S1_s_at	8.45	7.22	8.07	8.34	8.05	7.96	7.91	0.62	8.12	0.20	1.03	0.037	6.1E-01
Mtr.6521.1.S1_at	8.77	7.66	8.71	8.43	8.61	8.67	8.38	0.62	8.57	0.12	1.02	0.033	6.3E-01
Mtr.45480.1.S1_at	8.09	7.21	8.42	8.35	8.02	8.13	7.90	0.62	8.17	0.17	1.03	0.047	5.2E-01
Mtr.45153.1.S1_at	13.08	11.93	12.93	13.00	13.02	12.88	12.64	0.62	12.96	0.08	1.03	0.036	4.3E-01
Mtr.21815.1.S1_s_at	7.14	6.29	7.50	6.95	7.23	7.41	6.98	0.62	7.20	0.23	1.03	0.045	6.0E-01
Mtr.28315.1.S1_at	9.18	8.66	9.91	8.34	8.14	8.49	9.25	0.62	8.32	0.17	0.90	-0.152	6.9E-02
Mtr.23935.1.S1_at	6.28	5.08	5.98	8.53	8.46	8.66	5.78	0.62	8.55	0.10	1.48	0.564	1.6E-03
Mtr.37524.1.S1_at	11.63	10.44	10.69	11.03	11.13	11.24	10.92	0.62	11.13	0.11	1.02	0.028	5.9E-01
Mtr.24083.1.S1_at	7.23	6.37	7.58	6.97	7.17	7.02	7.06	0.62	7.06	0.10	1.00	-0.001	9.9E-01
Mtr.40394.1.S1_at	10.54	10.14	11.37	10.75	10.91	10.33	10.68	0.62	10.66	0.30	1.00	-0.003	9.6E-01
Mtr.45256.1.S1_at	10.11	9.03	10.12	9.90	10.02	9.92	9.75	0.62	9.95	0.06	1.02	0.029	6.2E-01
Mtr.51485.1.S1_at	7.14	6.14	7.28	6.36	6.21	6.29	6.85	0.62	6.29	0.08	0.92	-0.124	1.9E-01
Mtr.49767.1.S1_x_at	3.61	3.65	4.71	5.06	6.27	6.01	3.99	0.62	5.78	0.63	1.45	0.534	2.5E-02
Mtr.43803.1.S1_at	6.83	8.02	7.11	7.48	7.21	7.20	7.32	0.62	7.30	0.15	1.00	-0.004	9.6E-01
Mtr.41803.1.S1_at	9.76	10.70	10.94	9.57	9.38	9.61	10.47	0.62	9.52	0.13	0.91	-0.137	6.1E-02
Mtr.11863.1.S1_at	7.06	5.95	7.00	7.06	7.11	6.99	6.67	0.62	7.05	0.06	1.06	0.080	3.5E-01
Mtr.50835.1.S1_at	9.56	9.17	10.39	9.79	10.20	9.49	9.71	0.62	9.82	0.36	1.01	0.018	7.9E-01
Mtr.5657.1.S1_at	7.88	6.68	7.59	7.95	7.56	7.65	7.38	0.62	7.72	0.20	1.05	0.064	4.3E-01
Mtr.41037.1.S1_at	8.54	7.49	8.61	8.73	8.64	8.75	8.21	0.62	8.71	0.06	1.06	0.084	2.4E-01
Mtr.7724.1.S1_at	6.85	6.58	7.77	7.07	7.11	7.25	7.07	0.62	7.14	0.10	1.01	0.016	8.4E-01
Mtr.28780.1.S1_s_at	9.79	8.62	8.82	9.30	9.17	9.50	9.08	0.62	9.32	0.17	1.03	0.039	5.4E-01
Mtr.28419.1.S1_at	8.69	7.86	7.47	7.52	7.91	7.85	8.01	0.62	7.76	0.21	0.97	-0.045	5.5E-01
Mtr.42804.1.S1_at	7.14	6.27	7.48	7.11	7.07	7.10	6.96	0.62	7.09	0.02	1.02	0.027	7.3E-01
Mtr.16079.1.S1_at	6.11	5.02	6.08	6.07	6.22	6.54	5.74	0.62	6.27	0.24	1.09	0.130	2.3E-01
Mtr.18819.1.S1_at	4.26	3.26	3.12	3.08	3.86	3.27	3.55	0.62	3.40	0.40	0.96	-0.061	7.5E-01
Mtr.32251.1.S1_at	6.21	5.34	6.55	6.51	6.81	6.45	6.03	0.62	6.59	0.19	1.09	0.128	2.1E-01
Mtr.20521.1.S1_at	6.04	5.09	6.26	5.91	5.91	5.78	5.79	0.62	5.87	0.07	1.01	0.017	8.5E-01
Mtr.32983.1.S1_at	8.70	7.52	8.46	8.06	8.06	7.59	8.23	0.62	7.90	0.27	0.96	-0.058	4.5E-01
Mtr.16147.1.S1_x_at	3.41	3.99	4.65	3.71	5.61	3.52	4.01	0.62	4.28	1.15	1.07	0.092	7.4E-01
Mtr.19219.1.S1_at	8.23	7.14	8.19	8.13	8.33	8.19	7.85	0.62	8.22	0.10	1.05	0.065	3.8E-01
Mtr.32799.1.S1_at	9.65	8.48	9.43	9.64	9.37	9.56	9.19	0.62	9.52	0.14	1.04	0.051	4.2E-01
Mtr.20818.1.S1_at	4.93	3.85	4.93	4.89	5.45	4.41	4.57	0.62	4.92	0.52	1.07	0.104	5.0E-01
Mtr.26658.1.S1_at	2.54	3.62	2.54	2.53	2.53	2.52	2.90	0.62	2.53	0.01	0.87	-0.200	3.6E-01
Mtr.44349.1.S1_at	13.49	12.76	14.00	13.88	13.63	13.75	13.42	0.62	13.75	0.13	1.02	0.036	4.1E-01
Mtr.41602.1.S1_at	9.52	8.80	10.04	9.75	9.78	9.79	9.45	0.62	9.77	0.02	1.03	0.048	4.2E-01
Mtr.24447.1.S1_at	6.05	5.18	6.39	5.99	6.09	5.89	5.88	0.62	5.99	0.10	1.02	0.028	7.7E-01
Mtr.2441.1.S1_at	4.51	3.43	4.51	4.52	4.56	4.51	4.15	0.62	4.53	0.03	1.09	0.126	3.5E-01
Mtr.24821.1.S1_x_at	3.15	2.76	3.98	3.71	4.02	4.23	3.29	0.62	3.98	0.26	1.21	0.275	1.5E-01
Mtr.38978.1.S1_at	3.05	3.99	2.81	7.21	7.29	7.71	3.28	0.62	7.40	0.27	2.26	1.174	4.6E-04
Mtr.43906.1.S1_at	10.86	9.65	10.01	10.19	10.38	10.52	10.17	0.62	10.37	0.17	1.02	0.027	6.3E-01
Mtr.6347.1.S1_at	5.28	4.28	5.41	5.23	5.18	5.23	4.99	0.62	5.21	0.03	1.05	0.064	5.7E-01
Mtr.31790.1.S1_at	9.14	8.32	9.55	9.34	9.23	9.13	9.00	0.62	9.23	0.10	1.03	0.036	5.6E-01
Mtr.2583.1.S1_at	9.86	8.61	9.18	9.30	9.33	9.36	9.22	0.62	9.33	0.03	1.01	0.017	7.7E-01
Mtr.25329.1.S1_at	9.31	8.08	8.86	8.90	8.91	8.99	8.75	0.62	8.93	0.05	1.02	0.030	6.4E-01
Mtr.760.1.S1_s_at	4.61	3.47	4.45	4.45	4.87	4.25	4.18	0.62	4.52	0.31	1.08	0.115	4.4E-01
Mtr.42257.1.S1_at	13.25	12.14	12.20	12.22	11.70	12.21	12.53	0.62	12.04	0.30	0.96	-0.057	2.9E-01
Mtr.11252.1.S1_s_at	8.31	7.16	7.32	8.04	7.95	8.34	7.60	0.62	8.11	0.21	1.07	0.094	2.5E-01
Mtr.40666.1.S1_at	4.09	5.28	4.38	4.39	4.06	4.40	4.59	0.62	4.28	0.19	0.93	-0.098	4.7E-01
Mtr.6003.1.S1_at	9.06	7.95	8.99	8.94	8.85	8.97	8.67	0.62	8.92	0.06	1.03	0.041	5.2E-01
Mtr.28614.1.S1_s_at	10.80	9.90	9.61	10.17	10.16	10.44	10.11	0.62	10.26	0.16	1.01	0.021	7.1E-01
Mtr.1686.1.S1_at	3.71	2.70	3.83	3.69	3.69	3.72	3.41	0.62	3.70	0.02	1.09	0.118	4.6E-01
Mtr.37671.1.S1_at	7.38	6.21	7.16	7.44	7.31	6.95	6.92	0.62	7.23	0.26	1.05	0.065	4.6E-01
Mtr.11250.1.S1_at	11.75	10.84	12.03	11.72	11.77	11.34	11.54	0.62	11.61	0.24	1.01	0.009	8.6E-01
Mtr.11377.1.S1_at	9.25	8.36	9.55	10.06	9.69	10.04	9.05	0.62	9.93	0.21	1.10	0.134	8.0E-02
Mtr.5173.1.S1_at	6.47	5.82	7.06	6.51	6.84	6.38	6.45	0.62	6.58	0.24	1.02	0.028	7.6E-01
Mtr.5768.1.S1_at	8.67	7.51	8.47	8.14	8.10	7.98	8.22	0.62	8.07	0.08	0.98	-0.025	7.1E-01
Mtr.17421.1.S1_at	3.37	3.40	4.46	3.48	3.56	3.36	3.74	0.62	3.47	0.10	0.93	-0.110	4.9E-01
Mtr.9520.1.S1_at	9.73	8.56	9.52	9.61	9.34	9.44	9.27	0.62	9.46	0.14	1.02	0.029	6.3E-01
Mtr.11022.1.S1_at	9.63	8.40	9.20	9.33	9.32	9.36	9.08	0.62	9.34	0.02	1.03	0.041	5.1E-01
Mtr.18872.1.S1_at	8.04	7.00	8.10	8.00	7.97	7.84	7.71	0.62	7.94	0.08	1.03	0.041	5.7E-01
Mtr.32513.1.S1_at	5.97	5.07	6.26	6.15	6.06	6.31	5.76	0.62	6.17	0.13	1.07	0.099	3.3E-01
Mtr.35940.1.S1_at	7.95	7.65	8.84	8.14	8.43	7.88	8.15	0.62	8.15	0.27	1.00	0.000	1.0E+00
Mtr.4998.1.S1_at	5.69	5.68	6.76	6.50	6.26	6.20	6.04	0.62	6.32	0.16	1.05	0.065	5.0E-01
Mtr.8933.1.S1_at	11.80	10.70	10.76	11.23	11.30	11.13	11.09	0.62	11.22	0.09	1.01	0.017	7.4E-01
Mtr.30761.1.S1_at	9.12	7.88	8.50	8.88	8.71	8.52	8.50	0.62	8.70	0.18	1.02	0.034	6.1E-01
Mtr.33145.1.S1_at	2.98	2.48	3.71	2.90	3.47	3.09	3.06	0.62	3.15	0.29	1.03	0.044	8.2E-01
Mtr.38097.1.S1_at	11.37	10.13	10.69	10.66	11.01	10.42	10.73	0.62	10.70	0.29	1.00	-0.004	9.4E-01
Mtr.35437.1.S1_at	7.43	6.26	7.19	7.22	7.46	7.16	6.96	0.62	7.28	0.16	1.05	0.065	4.3E-01
Mtr.33768.1.S1_at	4.41	3.42	3.27	3.30	3.18	3.03	3.70	0.62	3.17	0.14	0.86	-0.225	2.2E-01
Mtr.18584.1.S1_at	6.06	5.18	6.38	5.58	5.42	5.38	5.87	0.62	5.46	0.11	0.93	-0.104	3.2E-01
Mtr.4337.1.S1_at	7.53	6.48	7.57	7.18	7.76	7.03	7.20	0.62	7.32	0.39	1.02	0.026	7.8E-01
Mtr.398.1.S1_s_at	4.61	5.70	4.65	4.57	4.59	4.60	4.98	0.62	4.58	0.02	0.92	-0.120	3.3E-01
Mtr.3702.1.S1_x_at	6.82	5.58	6.32	6.89	7.01	6.72	6.24	0.62	6.87	0.15	1.10	0.140	1.6E-01
Mtr.3676.1.S1_at	2.03	3.10	2.02	2.05	2.02	2.02	2.38	0.62	2.03	0.02	0.85	-0.229	3.8E-01
Mtr.44682.1.S1_at	6.66	5.78	6.98	6.43	6.38	6.12	6.48	0.62	6.31	0.16	0.97	-0.037	6.8E-01
Mtr.46946.1.S1_at	4.47	4.59	5.60	2.87	2.92	2.80	4.89	0.62	2.87	0.06	0.59	-0.770	4.9E-03
Mtr.13915.1.S1_at	8.09	6.94	7.92	7.90	7.83	8.09	7.65	0.62	7.94	0.13	1.04	0.052	4.8E-01
Mtr.51156.1.S1_x_at	6.46	5.67	6.89	9.54	9.83	9.83	6.34	0.62	9.73	0.17	1.53	0.618	7.9E-04
Mtr.2927.1.S1_at	8.60	7.47	8.48	8.45	8.56	8.35	8.18	0.62	8.45	0.10	1.03	0.048	4.9E-01
Mtr.38812.1.S1_at	13.42	12.26	13.20	13.55	13.40	13.57	12.96	0.62	13.51	0.09	1.04	0.060	2.1E-01

Mtr.45262.1.S1_at	9.06	9.16	10.18	9.52	9.93	9.40	9.47	0.62	9.62	0.28	1.02	0.023	7.2E-01
Mtr.7456.1.S1_at	7.61	7.06	8.29	7.84	8.12	7.84	7.65	0.62	7.94	0.16	1.04	0.052	4.9E-01
Mtr.5632.1.S1_at	7.19	6.16	7.26	7.50	7.21	7.21	6.87	0.62	7.31	0.17	1.06	0.089	3.0E-01
Mtr.27820.1.S1_at	5.89	4.86	4.77	4.78	4.97	4.79	5.17	0.62	4.85	0.11	0.94	-0.094	4.2E-01
Mtr.29088.1.S1_at	6.99	6.47	7.70	7.32	7.35	7.24	7.05	0.62	7.30	0.05	1.04	0.050	5.3E-01
Mtr.10814.1.S1_at	12.65	11.50	11.68	11.92	11.72	11.98	11.94	0.62	11.88	0.14	0.99	-0.008	8.6E-01
Mtr.38793.1.S1_at	9.26	10.43	10.20	9.30	10.02	9.24	9.97	0.62	9.52	0.43	0.96	-0.066	3.6E-01
Mtr.38300.1.S1_at	6.38	6.35	7.44	7.00	6.97	7.05	6.73	0.62	7.01	0.04	1.04	0.059	4.7E-01
Mtr.12256.1.S1_at	8.04	8.73	9.28	8.76	9.27	8.55	8.68	0.62	8.86	0.37	1.02	0.029	6.9E-01
Mtr.35096.1.S1_s_at	5.64	4.53	5.55	6.74	6.42	7.43	5.24	0.62	6.86	0.52	1.31	0.390	2.5E-02
Mtr.2878.1.S1_x_at	6.11	5.05	5.03	6.18	5.73	6.31	5.39	0.62	6.07	0.30	1.13	0.171	1.6E-01
Mtr.29380.1.S1_x_at	9.36	8.13	8.83	9.30	9.71	9.54	8.78	0.62	9.52	0.21	1.08	0.117	1.2E-01
Mtr.40280.1.S1_at	10.60	9.37	10.08	10.65	10.71	10.93	10.02	0.62	10.76	0.14	1.07	0.103	1.1E-01
Mtr.28491.1.S1_at	8.46	7.67	7.24	6.84	7.23	6.95	7.79	0.62	7.01	0.20	0.90	-0.153	1.0E-01
Mtr.27278.1.S1_x_at	8.55	7.32	7.80	8.43	8.54	8.69	7.89	0.62	8.55	0.13	1.08	0.116	1.4E-01
Mtr.22037.1.S1_at	6.28	7.20	7.46	6.79	7.19	7.22	6.98	0.62	7.07	0.24	1.01	0.017	8.4E-01
Mtr.27626.1.S1_s_at	2.89	3.95	2.88	2.87	2.78	2.87	3.24	0.62	2.84	0.06	0.88	-0.191	3.3E-01
Mtr.6051.1.S1_at	4.20	3.11	4.16	6.14	7.33	6.11	3.82	0.62	6.53	0.70	1.71	0.771	7.4E-03
Mtr.3884.1.S1_at	5.82	5.10	6.33	5.75	5.81	5.81	5.75	0.62	5.79	0.03	1.01	0.010	9.2E-01
Mtr.20983.1.S1_at	8.36	7.31	8.40	8.40	8.32	8.23	8.02	0.62	8.32	0.08	1.04	0.053	4.5E-01
Mtr.9445.1.S1_at	10.09	8.91	9.17	9.48	9.58	9.85	9.39	0.62	9.64	0.19	1.03	0.038	5.4E-01
Mtr.294.1.S1_at	4.27	3.25	3.16	3.48	4.03	3.67	3.56	0.62	3.73	0.28	1.05	0.065	7.0E-01
Mtr.33652.1.S1_at	7.06	6.20	7.40	7.18	7.41	6.71	6.89	0.62	7.10	0.35	1.03	0.044	6.3E-01
Mtr.32653.1.S1_at	11.72	10.67	10.64	10.95	10.54	10.89	11.01	0.62	10.79	0.22	0.98	-0.029	6.0E-01
Mtr.9664.1.S1_at	8.83	7.99	9.19	8.94	9.08	9.03	8.67	0.62	9.02	0.07	1.04	0.057	3.8E-01
Mtr.51125.1.S1_at	3.06	4.13	3.05	3.04	2.94	2.92	3.41	0.62	2.96	0.06	0.87	-0.204	2.8E-01
Mtr.47993.1.S1_at	5.92	5.65	6.83	6.89	7.25	6.63	6.13	0.62	6.92	0.31	1.13	0.175	1.2E-01
Mtr.39387.1.S1_at	7.44	6.43	7.55	7.48	7.46	7.63	7.14	0.62	7.52	0.09	1.05	0.075	3.5E-01
Mtr.35722.1.S1_at	8.49	7.72	8.94	8.20	8.37	7.88	8.38	0.62	8.15	0.25	0.97	-0.041	5.7E-01
Mtr.39664.1.S1_at	8.34	9.56	9.08	10.01	10.19	10.06	9.00	0.62	10.09	0.09	1.12	0.165	3.9E-02
Mtr.41109.1.S1_at	2.36	3.46	2.41	2.48	2.34	2.34	2.74	0.62	2.39	0.08	0.87	-0.201	3.8E-01
Mtr.43488.1.S1_at	7.79	6.90	8.09	7.99	7.48	7.77	7.59	0.62	7.74	0.26	1.02	0.028	7.2E-01
Mtr.6050.1.S1_at	9.27	8.15	9.17	9.28	9.17	9.32	8.86	0.62	9.26	0.08	1.04	0.063	3.3E-01
Mtr.12506.1.S1_at	4.67	3.59	4.65	5.19	5.33	4.77	4.30	0.62	5.10	0.29	1.18	0.244	1.2E-01
Mtr.10108.1.S1_at	3.39	3.33	4.43	3.46	4.67	3.63	3.72	0.62	3.92	0.66	1.06	0.078	7.1E-01
Mtr.43912.1.S1_at	9.86	8.78	9.84	9.52	9.58	9.54	9.49	0.62	9.54	0.03	1.01	0.008	8.9E-01
Mtr.32400.1.S1_at	3.73	3.31	4.53	3.79	4.12	3.76	3.86	0.62	3.89	0.20	1.01	0.011	9.4E-01
Mtr.5329.1.S1_at	6.36	5.29	6.35	6.27	6.42	6.39	6.00	0.62	6.36	0.08	1.06	0.084	3.7E-01
Mtr.27136.1.S1_at	4.38	3.56	4.76	4.37	4.48	4.35	4.23	0.62	4.40	0.07	1.04	0.057	6.6E-01
Mtr.20769.1.S1_at	8.95	7.83	8.85	8.87	8.81	8.79	8.54	0.62	8.82	0.04	1.03	0.046	4.8E-01
Mtr.19297.1.S1_at	2.73	3.82	2.76	2.71	2.71	2.71	3.10	0.62	2.71	0.00	0.87	-0.195	3.3E-01
Mtr.27543.1.S1_at	8.60	7.38	7.88	8.38	8.05	8.49	7.95	0.62	8.31	0.23	1.04	0.062	4.1E-01
Mtr.32371.1.S1_x_at	7.87	6.97	8.15	8.08	8.26	7.97	7.66	0.62	8.10	0.15	1.06	0.081	3.0E-01
Mtr.18323.1.S1_at	8.15	7.15	8.27	8.12	8.11	8.14	7.86	0.62	8.12	0.01	1.03	0.048	5.0E-01
Mtr.26209.1.S1_at	7.93	7.35	8.58	8.11	8.25	7.72	7.95	0.62	8.03	0.27	1.01	0.013	8.6E-01
Mtr.41444.1.S1_at	6.33	5.19	6.16	6.11	6.06	5.85	5.89	0.62	6.00	0.14	1.02	0.027	7.7E-01
Mtr.39776.1.S1_at	4.47	3.61	4.80	3.83	4.45	4.56	4.30	0.62	4.28	0.39	1.00	-0.005	9.7E-01
Mtr.10825.1.S1_at	4.92	3.71	4.08	3.48	3.04	2.75	4.24	0.62	3.09	0.37	0.73	-0.454	5.1E-02
Mtr.52130.1.S1_at	8.14	7.06	8.11	8.37	8.15	7.97	7.77	0.62	8.16	0.20	1.05	0.071	3.5E-01
Mtr.11859.1.S1_s_at	7.31	7.61	8.50	7.95	8.71	7.86	7.81	0.62	8.17	0.46	1.05	0.066	4.6E-01
Mtr.1954.1.S1_at	9.26	10.49	9.94	9.77	9.57	9.32	9.90	0.62	9.55	0.22	0.96	-0.051	4.1E-01
Mtr.32500.1.S1_at	6.94	5.70	6.34	6.66	6.82	6.92	6.33	0.62	6.80	0.13	1.08	0.104	2.6E-01
Mtr.26474.1.S1_at	9.05	7.84	8.65	8.95	8.76	9.08	8.52	0.62	8.93	0.16	1.05	0.068	3.2E-01
Mtr.2512.1.S1_at	5.73	5.12	6.35	6.19	6.35	5.75	5.74	0.62	6.10	0.31	1.06	0.088	4.2E-01
Mtr.45337.1.S1_at	8.70	7.89	9.10	8.72	9.00	8.72	8.56	0.62	8.81	0.16	1.03	0.041	5.4E-01
Mtr.10283.1.S1_at	11.97	10.76	11.55	11.84	11.61	11.87	11.43	0.62	11.77	0.14	1.03	0.043	4.0E-01
Mtr.5976.1.S1_at	8.67	7.51	7.73	8.08	7.86	7.95	7.97	0.62	7.96	0.11	1.00	-0.001	9.9E-01
Mtr.2156.1.S1_at	2.61	3.66	2.58	2.56	2.54	2.56	2.95	0.62	2.55	0.01	0.87	-0.208	3.3E-01
Mtr.28503.1.S1_at	7.30	6.64	7.87	7.36	7.73	7.51	7.27	0.62	7.54	0.19	1.04	0.052	5.1E-01
Mtr.35061.1.S1_at	7.93	6.76	7.68	7.78	7.68	7.74	7.46	0.62	7.73	0.05	1.04	0.052	4.8E-01
Mtr.43910.1.S1_at	2.27	3.33	2.26	2.25	2.25	2.32	2.62	0.62	2.27	0.04	0.87	-0.205	3.8E-01
Mtr.38340.1.S1_at	9.12	7.91	8.37	9.20	9.17	9.20	8.47	0.62	9.19	0.02	1.09	0.118	1.1E-01
Mtr.17209.1.S1_at	9.74	10.92	10.03	10.17	10.57	10.49	10.23	0.62	10.41	0.21	1.02	0.025	6.6E-01
Mtr.19042.1.S1_x_at	6.96	6.31	7.54	7.37	7.66	7.37	6.94	0.62	7.47	0.17	1.08	0.106	2.2E-01
Mtr.13436.1.S1_at	9.12	8.39	7.89	8.03	7.63	7.81	8.47	0.62	7.82	0.20	0.92	-0.114	1.6E-01
Mtr.44811.1.S1_at	8.51	9.74	9.03	9.05	9.87	9.49	9.09	0.62	9.47	0.41	1.04	0.059	4.3E-01
Mtr.45568.1.S1_at	13.16	12.04	12.16	12.55	12.05	12.57	12.45	0.62	12.39	0.29	1.00	-0.007	8.9E-01
Mtr.29665.1.S1_at	6.14	6.94	7.35	6.74	7.63	6.81	6.81	0.62	7.06	0.50	1.04	0.051	6.2E-01
Mtr.23578.1.S1_s_at	14.69	14.46	13.53	14.19	14.44	14.43	14.23	0.61	14.35	0.14	1.01	0.013	7.4E-01
Mtr.14664.1.S1_at	6.33	5.62	6.85	6.31	6.45	6.21	6.27	0.61	6.33	0.12	1.01	0.014	8.8E-01
Mtr.44050.1.S1_at	8.59	7.45	8.42	8.28	8.58	8.66	8.15	0.61	8.51	0.20	1.04	0.061	4.0E-01
Mtr.21755.1.S1_at	5.58	5.21	6.41	5.64	5.58	5.56	5.73	0.61	5.59	0.04	0.98	-0.036	7.1E-01
Mtr.9418.1.S1_s_at	6.58	5.98	7.21	7.03	7.54	6.60	6.59	0.61	7.05	0.47	1.07	0.097	3.6E-01
Mtr.31275.1.S1_at	7.98	7.02	8.16	7.86	7.87	8.07	7.72	0.61	7.94	0.12	1.03	0.040	5.8E-01
Mtr.22903.1.S1_at	3.59	2.62	3.76	2.75	2.77	2.74	3.32	0.61	2.76	0.01	0.83	-0.270	1.9E-01
Mtr.18636.1.S1_at	5.84	6.80	6.98	6.37	6.65	6.22	6.54	0.61	6.41	0.22	0.98	-0.028	7.5E-01
Mtr.4396.1.S1_at	8.37	7.19	8.08	8.25	8.15	8.32	7.88	0.61	8.24	0.08	1.05	0.064	3.7E-01
Mtr.20258.1.S1_x_at	3.29	4.34	3.27	3.28	3.13	3.33	3.63	0.61	3.25	0.10	0.89	-0.163	3.4E-01
Mtr.39610.1.S1_at	6.74	7.82	6.79	5.64	6.26	6.38	7.11	0.61	6.09	0.40	0.86	-0.224	7.3E-02
Mtr.38889.1.S1_at	6.04	4.82	5.36	6.16	5.95	5.56	5.41	0.61	5.89	0.30	1.09	0.123	2.9E-01
Mtr.32847.1.S1_at	7.63	6.55	7.61	7.71	7.93	7.91	7.26	0.61	7.85	0.12	1.08	0.113	1.8E-01
Mtr.13848.1.S1_at	9.98	8.76	9.37	9.54	9.35	9.28	9.37	0.61	9.39	0.14	1.00	0.003	9.5E-01
Mtr.27031.1.S1_at	7.04	6.53	7.75	8.09	8.53	7.76	7.10	0.61	8.12	0.39	1.14	0.194	7.2E-02
Mtr.41934.1.S1_at	9.42	10.58	9.65	10.06	10.33	9.81	9.88	0.61	10.07	0.26	1.02	0.027	6.5E-01
Mtr.10848.1.S1_s_at	8.12	6.99	7.97	7.84	8.11	8.05	7.69	0.61	8.00	0.15	1.04	0.057	4.4E-01
Mtr.18185.1.S1_at	2.90	3.97	2.93	2.88	2.84	2.94	3.27	0.61	2.89	0.05	0.88	-0.177	3.5E-01
Mtr.14668.1.S1_at	10.14	9.01	9.17	9.46	10.01	9.87	9.44	0.61	9.78	0.29	1.04	0.052	4.3E-01
Mtr.26913.1.S1_s_at	3.65	2.56	3.59	3.63	3.89	3.87	3.27	0.61	3.80	0.14	1.16	0.216	2.2E-01
Mtr.17466.1.S1_x_at	3.61	4.64	3.55	3.43	3.46	3.47	3.94	0.61	3.45	0.02	0.88	-0.189	2.4E-01
Mtr.33053.1.S1_s_at	3.67	2.81	4.00	3.65	3.65	3.65	3.50	0.61	3.65	0.00	1.04	0.063	6.8E-01
Mtr.34899.1.S1_at	4.44	3.32	3.45	3.39	3.39	3.40	3.73	0.61	3.39	0.00	0.91	-0.139	3.9E-01
Mtr.16731.1.S1_at	7.57	6.71	7.90	7.69	7.75	7.58	7.39	0.61	7.67	0.09	1.04	0.054	4.8E-01
Mtr.9084.1.S1_at	6.93	7.03	8.04	7.39	8.16	7.29	7.33	0.61	7.61	0.48	1.04	0.054	5.7E-01
Mtr.21673.1.S1_at	2.57	3.61	2.54	2.52	2.52	2.51	2.91	0.61	2.52	0.01	0.87	-0.206	3.4E-01
Mtr.3734.1.S1_at	8.90	7.74	8.67	8.46	8.41	8.33	8.43	0.61	8.40	0.07	1.00	-0.005	9.3E-01
Mtr.38691.1.S1_s_at	8.38	7.44	8.59	8.53	8.46	8.46	8.13	0.61	8.48	0.04	1.04	0.060	3.8E-01
Mtr.35012.1.S1_at	7.48	6.41	7.46	8.00	7.98	8.20	7.11	0.61	8.06	0.12	1.13	0.180	5.8E-02
Mtr.42841.1.S1_at	11.41	10.52	10.23	10.89	10.83	10.97	10.72	0.61	10.90	0.07	1.02	0.024	6.5E-01
Mtr.29252.1.S1_at	7.79	6.94	8.13	7.68	7.90	7.78	7.62	0.61	7.79	0.11	1.02	0.031	6.7E-01
Mtr.7152.1.S1_at	8.55	7.71	8.91	8.40	8.30	8.69	8.39	0.61	8.46	0.20	1.01	0.012	8.6E-01
Mtr.37096.1.S1_at	4.51	3.62	4.80	4.46	4.76	4.54	4.31	0.61	4.59	0.16	1.06	0.090	4.9E-01
Mtr.7548.1.S1_at	8.17	7.08	8.11	7.95	7.94	7.90	7.79	0.61	7.93	0.03	1.02	0.026	7.1E-01
Mtr.7878.1.S1_at	3.04	3.83	2.63	2.98	2.96	2.95	3.17	0.61	2.96	0.01	0.94	-0.096	5.9E-01
Mtr.14547.1.S1_at	5.12	5.66	4.44	3.76	4.85	3.40	5.07	0.61	4.00	0.76	0.79	-0.341	1.3E-01
Mtr.45136.1.S1_at	5.13	4.10	5.19	5.26	5.65	5.01	4.81	0.61	5.31	0.32	1.10	0.142	2.8E-01
Mtr.44455.1.S1_at	9.61	8.40	9.14	9.78	9.77	9.95	9.05	0.61	9.83	0.10	1.09	0.120	9.4E-02
Mtr.19820.1.S1_at	8.61	8.84	9.77	9.60	9.45	9.52	9.07	0.61	9.52	0.08	1.05	0.070	2.7E-01
Mtr.29378.1.S1_at	10.36	9.16	9.94	9.97	10.01	10.01	9.82	0.61	10.00	0.02	1.02	0.026	6.4E-01
Mtr.38513.1.S1_at	8.85	7.63	8.22	9.30	9.97	9.66	8.23	0.61	9.64	0.33	1.17	0.228	2.5E-02
Mtr.32046.1.S1_at	5.65	4.43	5.10	4.45	4.64	4.45	5.06	0.61	4.51	0.11	0.89	-0.165	2.0E-01
Mtr.43146.1.S1_at	12.48	11.74	11.27	11.54	12.23	11.84	11.83	0.61	11.87	0.35	1.00	0.005	9.3E-01
Mtr.40549.1.S1_at	9.88	8.80	9.84	10.01	10.32	10.33	9.51	0.61	10.22	0.18	1.07	0.104	1.3E-01
Mtr.39296.1.S1_at	4.55	4.57	3.50	4.72	4.43	4.42	4.20	0.61	4.52	0.17	1.08	0.105	4.3E-01
Mtr.44721.1.S1_at	7.96	7.01	8.15	7.64	7.36	7.56	7.71	0.61	7.52	0.14	0.98	-0.036	6.3E-01
Mtr.37409.1.S1_s_at	8.29	8.50	7.35	6.99	7.22	7.26	8.05	0.61	7.16	0.15	0.89	-0.170	7.0E-02
Mtr.12582.1.S1_at	10.34	9.98	11.17	10.34	10.16	10.15	10.50	0.61	10.22	0.10	0.97	-0.039	4.8E-01
Mtr.34703.1.S1_at	3.09	2.79	3.97	3.24	2.91	2.92	3.28	0.61	3.02	0.19	0.92	-0.118	5.2E-01
Mtr.30508.1.S1_at	14.05	12.83	13.51	13.68	13.52	13.64	13.46	0.61	13.61	0.08	1.01	0.016	6.9E-01
Mtr.10618.1.S1_at	9.14	7.98	8.89	8.95	8.85	8.98	8.67	0.61	8.93	0.07	1.03	0.042	5.1E-01
Mtr.11982.1.S1_s_at	7.79	7.56	8.72	8.06	8.18	7.96	8.02	0.61	8.07	0.11	1.01	0.008	9.0E-01
Mtr.15949.1.S1_at	9.45	9.72	10.62	9.81	9.92	9.68	9.93	0.61	9.80	0.12	0.99	-0.018	7.5E-01
Mtr.9297.1.S1_at	9.70	8.79	9.95	9.91	9.89	9.78	9.48	0.61	9.86	0.07	1.04	0.057	3.4E-01
Mtr.11586.1.S1_at	8.55	8.78	9.70	8.49	8.41	8.64	9.01	0.61	8.51	0.11	0.94	-0.082	2.4E-01
Mtr.11923.1.S1_at	6.68	6.22	7.43	6.92	7.07	6.45	6.78	0.61	6.82	0.32	1.01	0.008	9.3E-01
Mtr.14599.1.S1_x_at	8.38	9.33	9.52	9.13	9.31	8.56	9.08	0.61	9.00	0.39	0.99	-0.013	8.6E-01
Mtr.33796.1.S1_at	5.23	6.27	6.30	5.95	6.20	5.68	5.93	0.61	5.94	0.26	1.00	0.002	9.9E-01
Mtr.8751.1.S1_at	9.48	8.25	8.86	8.97	8.75	9.00	8.86	0.61	8.91	0.14	1.00	0.007	9.1E-01
Mtr.41489.1.S1_at	9.29	8.50	9.70	9.66	9.66	9.60	9.16	0.61	9.64	0.03	1.05	0.074	2.5E-01
Mtr.12123.1.S1_at	9.41	8.21	8.62	8.48	8.73	8.47	8.75	0.61	8.56	0.15	0.98	-0.032	6.3E-01
Mtr.33374.1.S1_at	8.04	6.82	7.50	7.44	7.26	7.64	7.45	0.61	7.45	0.19	1.00	-0.001	9.9E-01
Mtr.24415.1.S1_s_at	11.15	10.62	9.93	9.93	10.12	9.91	10.56	0.61	9.99	0.12	0.95	-0.081	1.8E-01

Mtr.44541.1.S1_at	8.26	7.82	9.03	8.38	8.27	8.26	8.37	0.61	8.31	0.07	0.99	-0.011	8.7E-01
Mtr.3533.1.S1_at	10.61	11.65	11.69	10.68	11.07	10.37	11.32	0.61	10.71	0.35	0.95	-0.080	2.1E-01
Mtr.43559.1.S1_at	8.85	7.73	8.71	8.74	8.56	8.68	8.43	0.61	8.66	0.09	1.03	0.038	5.6E-01
Mtr.16816.1.S1_at	7.79	7.34	8.54	8.00	8.24	8.07	7.89	0.61	8.10	0.12	1.03	0.039	5.8E-01
Mtr.46487.1.S1_at	6.77	7.81	7.84	6.92	7.30	7.16	7.47	0.61	7.13	0.19	0.95	-0.069	4.0E-01
Mtr.33237.1.S1_at	7.51	6.30	7.05	7.53	7.70	7.46	6.95	0.61	7.56	0.13	1.09	0.122	1.6E-01
Mtr.26118.1.S1_x_at	3.32	4.44	3.45	3.20	3.06	3.06	3.74	0.61	3.11	0.08	0.83	-0.267	1.5E-01
Mtr.26072.1.S1_at	2.72	3.76	2.69	2.62	2.66	2.63	3.05	0.61	2.64	0.02	0.86	-0.212	3.0E-01
Mtr.21943.1.S1_x_at	10.48	9.26	9.86	10.09	9.92	9.99	9.87	0.61	10.00	0.08	1.01	0.019	7.3E-01
Mtr.924.1.S1_at	2.42	3.53	2.53	2.40	2.23	2.41	2.83	0.61	2.35	0.10	0.83	-0.267	2.5E-01
Mtr.6808.1.S1_at	8.83	7.92	9.08	8.75	8.99	8.76	8.61	0.61	8.83	0.13	1.03	0.037	5.7E-01
Mtr.37966.1.S1_at	9.97	11.06	10.05	11.61	11.55	11.68	10.36	0.61	11.61	0.06	1.12	0.165	2.4E-02
Mtr.29471.1.S1_s_at	8.77	7.61	8.51	8.69	8.72	8.73	8.30	0.61	8.71	0.02	1.05	0.070	3.0E-01
Mtr.45675.1.S1_at	2.34	3.40	2.36	2.32	2.32	2.33	2.70	0.61	2.32	0.01	0.86	-0.218	3.4E-01
Mtr.28420.1.S1_at	9.76	8.58	9.43	9.71	9.70	9.69	9.26	0.61	9.70	0.01	1.05	0.068	2.8E-01
Mtr.31634.1.S1_at	2.53	2.68	3.65	2.51	2.51	2.50	2.95	0.61	2.51	0.00	0.85	-0.236	2.7E-01
Mtr.2134.1.S1_at	5.45	4.35	5.36	5.43	5.37	5.19	5.05	0.61	5.33	0.12	1.06	0.077	4.8E-01
Mtr.50231.1.S1_at	5.09	4.95	6.07	5.16	5.17	5.09	5.37	0.61	5.14	0.04	0.96	-0.063	5.5E-01
Mtr.51019.1.S1_at	8.51	7.64	8.81	8.39	8.31	8.42	8.32	0.61	8.37	0.06	1.01	0.009	8.9E-01
Mtr.27307.1.S1_at	4.68	3.57	4.55	4.05	3.90	3.92	4.26	0.61	3.96	0.08	0.93	-0.108	4.4E-01
Mtr.12235.1.S1_at	10.10	9.05	10.12	10.04	9.89	9.91	9.76	0.61	9.95	0.08	1.02	0.028	6.2E-01
Mtr.5662.1.S1_at	7.50	6.45	7.51	7.30	7.47	7.46	7.15	0.61	7.41	0.09	1.04	0.051	5.1E-01
Mtr.6117.1.S1_at	11.22	10.04	10.90	10.79	10.85	10.77	10.72	0.61	10.80	0.04	1.01	0.011	8.3E-01
Mtr.3365.1.S1_at	2.80	3.83	2.75	2.74	2.78	2.85	3.12	0.61	2.79	0.05	0.89	-0.163	4.0E-01
Mtr.12047.1.S1_at	8.06	6.85	7.36	7.58	7.45	7.70	7.42	0.61	7.58	0.13	1.02	0.029	6.9E-01
Mtr.22684.1.S1_at	8.63	7.71	8.87	9.27	9.18	8.75	8.40	0.61	9.07	0.28	1.08	0.110	1.6E-01
Mtr.39992.1.S1_s_at	5.84	4.62	5.27	5.85	5.03	5.22	5.24	0.61	5.37	0.43	1.02	0.034	7.9E-01
Mtr.47604.1.S1_at	2.56	3.58	2.50	2.49	2.49	2.49	2.88	0.61	2.49	0.00	0.87	-0.209	3.3E-01
Mtr.9168.1.S1_at	9.32	8.21	9.19	9.28	8.94	9.24	8.91	0.61	9.16	0.19	1.03	0.040	5.3E-01
Mtr.28727.1.S1_at	9.80	8.58	9.14	9.45	9.90	9.55	9.17	0.61	9.63	0.23	1.05	0.071	2.9E-01
Mtr.41226.1.S1_at	9.50	8.52	8.39	8.54	8.56	8.55	8.81	0.61	8.55	0.01	0.97	-0.042	5.1E-01
Mtr.25196.1.S1_x_at	12.31	11.89	13.09	13.30	13.46	12.76	12.43	0.61	13.17	0.37	1.06	0.084	1.4E-01
Mtr.44508.1.S1_s_at	7.16	6.19	7.31	7.08	7.52	7.44	6.89	0.61	7.35	0.23	1.07	0.094	2.9E-01
Mtr.37527.1.S1_at	11.45	12.22	11.02	10.75	11.25	10.98	11.57	0.61	10.99	0.25	0.95	-0.073	2.1E-01
Mtr.46116.1.S1_at	2.63	3.68	2.62	2.58	2.56	2.61	2.98	0.61	2.58	0.02	0.87	-0.205	3.2E-01
Mtr.4625.1.S1_at	7.85	7.07	8.27	8.18	8.07	7.97	7.73	0.61	8.07	0.11	1.04	0.063	3.9E-01
Mtr.39979.1.S1_at	5.31	4.29	5.37	5.64	5.52	5.27	4.99	0.61	5.48	0.19	1.10	0.135	2.6E-01
Mtr.18904.1.S1_at	2.61	3.64	2.56	2.55	2.55	2.54	2.94	0.61	2.55	0.01	0.87	-0.206	3.3E-01
Mtr.37863.1.S1_at	2.77	3.82	2.76	2.80	2.71	2.73	3.12	0.61	2.75	0.05	0.88	-0.181	3.6E-01
Mtr.45293.1.S1_at	3.97	3.44	4.66	3.89	3.95	3.98	4.02	0.61	3.94	0.04	0.98	-0.030	8.3E-01
Mtr.34708.1.S1_at	8.20	7.21	8.32	8.00	7.74	7.66	7.91	0.61	7.80	0.18	0.99	-0.021	7.7E-01
Mtr.1619.1.S1_s_at	5.48	4.29	5.11	5.69	5.62	5.56	4.96	0.61	5.62	0.06	1.13	0.181	1.3E-01
Mtr.26005.1.S1_s_at	9.44	8.28	9.17	9.13	9.23	9.09	8.96	0.61	9.15	0.08	1.02	0.030	6.3E-01
Mtr.12824.1.S1_at	7.63	6.42	7.01	7.41	7.39	7.60	7.02	0.61	7.47	0.12	1.06	0.089	2.8E-01
Mtr.36844.1.S1_at	2.00	3.05	1.99	1.99	1.98	1.98	2.34	0.61	1.98	0.00	0.85	-0.242	3.6E-01
Mtr.49400.1.S1_at	8.32	7.27	8.32	7.18	7.32	7.13	7.97	0.61	7.21	0.10	0.90	-0.145	9.9E-02
Mtr.13588.1.S1_s_at	6.78	5.83	6.96	7.07	7.02	7.33	6.52	0.61	7.14	0.16	1.09	0.131	1.6E-01
Mtr.18220.1.S1_s_at	6.04	6.02	7.08	6.25	6.02	5.81	6.38	0.61	6.03	0.22	0.94	-0.083	3.9E-01
Mtr.15882.1.S1_at	5.57	6.17	4.96	4.89	5.14	4.82	5.57	0.61	4.95	0.17	0.89	-0.170	1.6E-01
Mtr.12157.1.S1_at	7.60	6.40	7.18	7.09	7.34	7.55	7.06	0.61	7.33	0.23	1.04	0.054	5.1E-01
Mtr.45888.1.S1_s_at	4.61	3.66	3.48	3.81	3.59	3.50	3.92	0.61	3.63	0.16	0.93	-0.109	4.8E-01
Mtr.758.1.S1_at	3.08	4.13	3.08	3.07	3.06	3.04	3.43	0.61	3.05	0.02	0.89	-0.168	3.4E-01
Mtr.4467.1.S1_at	9.48	10.08	8.87	8.48	9.17	8.94	9.48	0.61	8.86	0.35	0.94	-0.097	2.0E-01
Mtr.30190.1.S1_at	3.48	3.54	4.56	3.48	4.15	3.47	3.86	0.61	3.70	0.39	0.96	-0.061	7.2E-01
Mtr.46721.1.S1_x_at	8.43	7.91	9.12	8.71	9.01	8.51	8.48	0.61	8.75	0.25	1.03	0.044	5.3E-01
Mtr.38442.1.S1_s_at	8.58	7.48	8.47	8.77	8.67	9.13	8.18	0.61	8.85	0.24	1.08	0.115	1.5E-01
Mtr.33249.1.S1_at	6.65	5.68	6.80	6.85	7.13	7.27	6.38	0.61	7.08	0.22	1.11	0.151	1.3E-01
Mtr.41125.1.S1_s_at	7.52	6.41	7.39	7.36	7.58	7.54	7.11	0.61	7.49	0.12	1.05	0.076	3.4E-01
Mtr.13356.1.S1_at	6.94	7.29	6.11	6.01	5.38	5.91	6.78	0.61	5.76	0.34	0.85	-0.234	6.4E-02
Mtr.17596.1.S1_at	7.42	6.86	8.07	7.59	7.49	7.68	7.45	0.61	7.59	0.10	1.02	0.026	7.2E-01
Mtr.41644.1.S1_at	11.60	10.51	10.59	10.92	11.04	11.07	10.90	0.61	11.01	0.08	1.01	0.014	7.7E-01
Mtr.32222.1.S1_at	8.77	7.63	8.55	8.56	8.52	8.49	8.32	0.61	8.52	0.03	1.02	0.035	5.9E-01
Mtr.24476.1.S1_s_at	10.70	9.59	10.55	10.96	10.89	11.07	10.28	0.61	10.97	0.09	1.07	0.094	1.2E-01
Mtr.19224.1.S1_at	3.42	3.50	4.51	3.29	3.30	3.47	3.81	0.61	3.35	0.10	0.88	-0.185	2.7E-01
Mtr.22560.1.S1_x_at	3.15	4.34	3.55	3.46	3.45	3.44	3.68	0.61	3.45	0.01	0.94	-0.094	5.4E-01
Mtr.20006.1.S1_at	3.29	4.27	3.16	3.84	3.25	3.28	3.58	0.61	3.46	0.33	0.97	-0.049	7.8E-01
Mtr.652.1.S1_at	5.71	5.49	6.63	5.98	5.90	5.55	5.94	0.61	5.81	0.23	0.98	-0.033	7.4E-01
Mtr.4237.1.S1_at	8.37	7.19	8.00	8.03	8.14	8.32	7.85	0.61	8.16	0.15	1.04	0.056	4.4E-01
Mtr.13404.1.S1_at	8.57	7.99	9.20	9.02	9.11	8.97	8.58	0.61	9.03	0.07	1.05	0.074	2.7E-01
Mtr.31467.1.S1_at	5.32	6.42	5.44	5.54	5.39	5.47	5.73	0.61	5.47	0.08	0.95	-0.067	5.0E-01
Mtr.25984.1.S1_at	6.35	6.25	7.34	6.66	6.99	6.65	6.65	0.61	6.77	0.19	1.02	0.025	7.6E-01
Mtr.1980.1.S1_at	9.84	8.85	8.74	8.64	8.41	8.53	9.14	0.61	8.52	0.11	0.93	-0.101	1.6E-01
Mtr.46902.1.S1_s_at	4.27	3.30	4.42	5.31	5.55	4.84	4.00	0.61	5.23	0.36	1.31	0.388	3.9E-02
Mtr.35478.1.S1_at	8.07	7.17	8.33	8.01	8.11	8.00	7.86	0.61	8.04	0.06	1.02	0.033	6.3E-01
Mtr.27883.1.S1_s_at	11.78	10.84	10.65	11.15	11.32	11.36	11.09	0.60	11.28	0.11	1.02	0.024	6.2E-01
Mtr.49212.1.S1_s_at	10.40	9.22	9.60	10.12	9.82	10.11	9.74	0.60	10.02	0.17	1.03	0.041	4.8E-01
Mtr.2713.1.S1_s_at	9.07	8.25	9.42	9.21	9.47	9.21	8.91	0.60	9.30	0.15	1.04	0.061	3.5E-01
Mtr.9279.1.S1_at	10.09	8.89	9.66	9.84	9.52	9.82	9.55	0.60	9.73	0.18	1.02	0.027	6.5E-01
Mtr.8966.1.S1_at	10.12	9.29	8.95	9.47	9.94	9.77	9.45	0.60	9.73	0.24	1.03	0.041	5.1E-01
Mtr.34844.1.S1_at	3.37	2.45	3.59	3.40	3.36	3.35	3.13	0.60	3.37	0.03	1.08	0.106	5.3E-01
Mtr.51799.1.S1_s_at	9.92	8.73	9.54	9.77	9.71	9.92	9.39	0.60	9.80	0.11	1.04	0.061	3.2E-01
Mtr.28905.1.S1_at	2.49	3.53	2.48	2.47	2.47	2.46	2.83	0.60	2.47	0.00	0.87	-0.198	3.6E-01
Mtr.14315.1.S1_at	4.20	3.90	5.06	4.32	4.34	4.13	4.39	0.60	4.26	0.12	0.97	-0.041	7.4E-01
Mtr.47157.1.S1_at	4.52	3.72	4.90	4.54	4.54	4.53	4.38	0.60	4.54	0.01	1.04	0.052	6.7E-01
Mtr.9559.1.S1_at	8.84	7.77	8.78	8.84	8.84	8.84	8.46	0.60	8.84	0.00	1.04	0.063	3.4E-01
Mtr.42134.1.S1_at	6.31	5.55	6.75	6.35	6.35	6.35	6.20	0.60	6.35	0.00	1.02	0.034	6.9E-01
Mtr.4628.1.S1_at	6.69	6.05	7.26	7.03	6.98	6.85	6.66	0.60	6.95	0.09	1.04	0.061	4.6E-01
Mtr.42914.1.S1_at	2.69	3.80	2.82	2.75	2.63	2.62	3.11	0.60	2.67	0.08	0.86	-0.220	2.8E-01
Mtr.38524.1.S1_at	8.33	7.61	8.81	8.58	8.22	8.07	8.25	0.60	8.29	0.26	1.00	0.007	9.2E-01
Mtr.23449.1.S1_at	4.62	3.57	4.62	5.08	4.44	4.84	4.27	0.60	4.78	0.32	1.12	0.163	2.6E-01
Mtr.27819.1.S1_at	8.26	7.12	8.04	7.83	7.93	7.95	7.81	0.60	7.90	0.06	1.01	0.018	7.9E-01
Mtr.20717.1.S1_at	2.76	3.78	2.72	2.71	2.67	2.67	3.09	0.60	2.68	0.02	0.87	-0.203	3.1E-01
Mtr.13842.1.S1_at	5.11	4.87	3.96	5.01	4.60	4.93	4.65	0.60	4.84	0.22	1.04	0.059	6.3E-01
Mtr.33235.1.S1_at	4.86	4.35	5.55	4.85	4.88	4.66	4.92	0.60	4.80	0.12	0.98	-0.036	7.5E-01
Mtr.32932.1.S1_at	7.97	7.05	8.19	7.93	7.93	7.87	7.74	0.60	7.91	0.03	1.02	0.032	6.4E-01
Mtr.5322.1.S1_s_at	10.18	9.03	9.92	10.00	10.05	10.04	9.71	0.60	10.03	0.03	1.03	0.047	4.1E-01
Mtr.38245.1.S1_at	12.77	11.60	11.94	12.27	12.19	12.32	12.10	0.60	12.26	0.06	1.01	0.019	6.7E-01
Mtr.5323.1.S1_at	10.75	9.72	9.69	10.00	9.70	10.08	10.05	0.60	9.93	0.20	0.99	-0.018	7.5E-01
Mtr.44718.1.S1_at	11.11	10.15	10.00	10.51	10.65	10.51	10.42	0.60	10.56	0.08	1.01	0.018	7.2E-01
Mtr.27189.1.S1_at	7.12	5.95	6.79	6.96	6.96	6.93	6.62	0.60	6.95	0.02	1.05	0.071	3.9E-01
Mtr.41401.1.S1_at	10.94	9.79	10.07	10.52	10.37	10.50	10.27	0.60	10.46	0.08	1.02	0.027	6.1E-01
Mtr.27260.1.S1_at	4.87	3.82	4.87	4.90	4.77	5.03	4.52	0.60	4.90	0.13	1.08	0.116	3.5E-01
Mtr.51824.1.S1_at	4.95	6.09	5.19	6.10	7.29	6.20	5.41	0.60	6.53	0.66	1.21	0.271	9.6E-02
Mtr.33751.1.S1_at	8.29	7.30	8.39	8.46	8.32	8.37	7.99	0.60	8.38	0.07	1.05	0.069	3.3E-01
Mtr.15734.1.S1_at	2.63	3.66	2.60	2.58	2.58	2.55	2.96	0.60	2.57	0.02	0.87	-0.206	3.2E-01
Mtr.27351.1.S1_at	8.94	7.74	8.37	8.52	8.63	8.70	8.35	0.60	8.62	0.09	1.03	0.045	4.9E-01
Mtr.5775.1.S1_at	8.05	6.97	7.98	7.86	7.70	7.55	7.67	0.60	7.70	0.16	1.00	0.006	9.3E-01
Mtr.1304.1.S1_at	11.34	10.54	10.15	10.43	10.56	10.77	10.67	0.60	10.59	0.17	0.99	-0.012	8.2E-01
Mtr.42479.1.S1_at	6.16	5.04	5.21	6.47	5.36	6.25	5.47	0.60	6.03	0.59	1.10	0.141	3.1E-01
Mtr.21899.1.S1_at	8.66	7.56	8.54	8.65	8.75	8.91	8.25	0.60	8.77	0.13	1.06	0.088	2.2E-01
Mtr.4535.1.S1_at	9.65	8.45	8.97	8.80	8.99	9.09	9.03	0.60	8.96	0.15	0.99	-0.011	8.6E-01
Mtr.32544.1.S1_at	8.25	7.05	7.53	7.66	7.82	7.51	7.61	0.60	7.66	0.16	1.01	0.010	8.9E-01
Mtr.33107.1.S1_x_at	7.23	6.21	7.27	6.53	7.38	6.09	6.90	0.60	6.67	0.66	0.97	-0.051	6.7E-01
Mtr.35271.1.S1_at	3.50	3.61	4.59	4.33	3.48	3.67	3.90	0.60	3.83	0.45	0.98	-0.028	8.7E-01
Mtr.41848.1.S1_at	8.53	7.36	8.18	8.37	8.08	8.55	8.02	0.60	8.33	0.24	1.04	0.055	4.5E-01
Mtr.31504.1.S1_at	5.69	4.76	5.89	5.28	5.27	5.05	5.45	0.60	5.20	0.13	0.95	-0.067	5.2E-01
Mtr.44747.1.S1_at	8.91	7.79	7.96	8.53	7.88	8.59	8.22	0.60	8.33	0.39	1.01	0.019	8.0E-01
Mtr.39341.1.S1_at	9.21	8.02	8.45	8.79	8.44	8.99	8.56	0.60	8.74	0.28	1.02	0.031	6.6E-01
Mtr.13604.1.S1_at	6.63	6.08	7.29	6.41	6.49	6.65	6.67	0.60	6.51	0.12	0.98	-0.034	6.9E-01
Mtr.27569.1.S1_at	3.07	3.09	4.13	3.70	3.36	3.12	3.43	0.60	3.40	0.29	0.99	-0.015	9.3E-01
Mtr.46110.1.S1_at	11.27	10.13	10.36	10.89	10.82	10.97	10.59	0.60	10.89	0.08	1.03	0.041	4.3E-01
Mtr.20784.1.S1_s_at	11.22	10.02	10.58	10.78	10.67	10.90	10.61	0.60	10.78	0.12	1.02	0.023	6.5E-01
Mtr.46189.1.S1_s_at	7.63	6.80	7.97	7.75	7.80	7.72	7.47	0.60	7.76	0.04	1.04	0.055	4.5E-01
Mtr.31294.1.S1_at	11.33	10.12	10.73	10.96	11.02	10.97	10.72	0.60	10.98	0.03	1.02	0.034	5.0E-01
Mtr.51073.1.S1_at	8.58	7.67	8.81	9.04	9.08	8.85	8.35	0.60	8.99	0.12	1.08	0.105	1.5E-01
Mtr.28714.1.S1_at	7.59	6.47	6.64	6.47	6.33	6.52	6.90	0.60	6.44	0.10	0.93	-0.100	2.6E-01
Mtr.37228.1.S1_at	11.91	10.80	11.74	11.81	11.58	11.87	11.48	0.60	11.75	0.15	1.02	0.034	4.9E-01
Mtr.24211.1.S1_at	12.13	10.93	11.45	11.73	11.83	11.95	11.50	0.60	11.84	0.11	1.03	0.041	4.0E-01
Mtr.11871.1.S1_at	6.61	5.56	6.60	6.73	6.79	6.59	6.26	0.60	6.70	0.10	1.07	0.100	2.7E-01
Mtr.52172.1.S1_s_at	5.14	4.08	5.11	5.33	5.26	4.99	4.78	0.60	5.19	0.18	1.09	0.120	3.1E-01
Mtr.38256.1.S1_s_at	8.78	7.61	7.93	8.31	8.26	8.55	8.11	0.60	8.37	0.16	1.03	0.046	5.0E-01

Mtr.11641.1.S1_at	6.76	5.65	6.60	7.13	6.90	7.06	6.34	0.60	7.03	0.12	1.11	0.150	1.2E-01
Mtr.50911.1.S1_s_at	8.28	7.09	7.81	7.53	8.01	7.60	7.73	0.60	7.72	0.26	1.00	-0.002	9.8E-01
Mtr.26036.1.S1_s_at	3.94	4.10	5.05	5.19	4.12	4.90	4.36	0.60	4.74	0.55	1.09	0.118	4.7E-01
Mtr.24800.1.S1_at	4.55	5.63	4.64	4.73	4.69	5.34	4.94	0.60	4.92	0.36	1.00	-0.007	9.6E-01
Mtr.18441.1.S1_s_at	8.03	7.47	8.67	7.95	8.10	7.78	8.06	0.60	7.94	0.16	0.99	-0.021	7.6E-01
Mtr.44594.1.S1_at	4.01	3.37	2.81	3.74	3.29	3.13	3.39	0.60	3.38	0.31	1.00	-0.004	9.8E-01
Mtr.5676.1.S1_at	4.16	3.09	4.10	4.08	4.07	4.32	3.78	0.60	4.16	0.14	1.10	0.136	3.5E-01
Mtr.42219.1.S1_at	8.06	6.90	7.73	7.97	8.01	8.10	7.56	0.60	8.03	0.07	1.06	0.086	2.5E-01
Mtr.38622.1.S1_at	7.80	7.17	8.37	8.03	8.11	7.98	7.78	0.60	8.04	0.06	1.03	0.048	4.9E-01
Mtr.17457.1.S1_at	5.79	4.80	5.89	6.15	6.12	6.67	5.49	0.60	6.32	0.31	1.15	0.201	1.0E-01
Mtr.31281.1.S1_s_at	7.56	6.77	7.95	7.65	7.95	7.27	7.43	0.60	7.62	0.34	1.03	0.038	6.5E-01
Mtr.33052.1.S1_at	5.37	4.42	5.53	5.49	5.76	5.50	5.10	0.60	5.58	0.15	1.09	0.129	2.5E-01
Mtr.7024.1.S1_at	3.44	2.55	3.69	3.82	3.83	3.67	3.22	0.60	3.77	0.09	1.17	0.227	1.9E-01
Mtr.1515.1.S1_at	5.78	4.73	5.77	5.84	5.74	5.83	5.43	0.60	5.80	0.05	1.07	0.097	3.4E-01
Mtr.35545.1.S1_at	11.73	10.56	10.92	11.06	11.45	11.21	11.07	0.60	11.24	0.20	1.02	0.022	6.6E-01
Mtr.37929.1.S1_at	9.08	9.43	10.25	9.21	9.39	9.00	9.59	0.60	9.20	0.20	0.96	-0.060	3.5E-01
Mtr.41341.1.S1_at	9.65	8.47	9.21	9.26	9.50	9.41	9.11	0.60	9.39	0.13	1.03	0.044	4.7E-01
Mtr.1715.1.S1_at	8.43	7.75	8.95	8.68	8.81	8.70	8.38	0.60	8.73	0.07	1.04	0.059	3.7E-01
Mtr.52241.1.S1_at	10.73	11.49	10.31	10.38	11.20	10.89	10.84	0.60	10.82	0.41	1.00	-0.003	9.6E-01
Mtr.43953.1.S1_s_at	11.17	9.98	10.70	9.77	9.25	9.89	10.62	0.60	9.63	0.34	0.91	-0.140	6.9E-02
Mtr.19082.1.S1_at	8.69	7.65	8.70	8.47	8.14	8.26	8.35	0.60	8.29	0.17	0.99	-0.010	8.9E-01
Mtr.39409.1.S1_at	8.85	7.71	8.59	8.67	9.01	8.81	8.38	0.60	8.83	0.17	1.05	0.075	2.8E-01
Mtr.19629.1.S1_at	8.84	7.92	9.04	8.68	8.51	8.21	8.60	0.60	8.47	0.24	0.98	-0.022	7.4E-01
Mtr.7142.1.S1_at	3.99	2.94	2.95	2.96	2.91	2.94	3.29	0.60	2.94	0.03	0.89	-0.166	3.6E-01
Mtr.12860.1.S1_at	9.68	8.58	8.72	8.88	9.00	8.84	8.99	0.60	8.91	0.08	0.99	-0.013	8.2E-01
Mtr.3287.1.S1_at	2.36	3.39	2.35	2.33	2.34	2.28	2.70	0.60	2.32	0.03	0.86	-0.222	3.3E-01
Mtr.28911.1.S1_at	5.39	4.53	5.69	5.55	5.63	5.56	5.20	0.60	5.58	0.05	1.07	0.100	3.4E-01
Mtr.33479.1.S1_at	5.61	4.68	5.80	5.68	5.71	5.89	5.36	0.60	5.76	0.11	1.07	0.103	3.2E-01
Mtr.45549.1.S1_at	8.14	7.09	8.12	8.06	8.22	8.00	7.78	0.60	8.09	0.11	1.04	0.056	4.3E-01
Mtr.49600.1.S1_s_at	9.50	8.30	8.88	8.92	8.99	9.04	8.89	0.60	8.98	0.06	1.01	0.015	8.0E-01
Mtr.1781.1.S1_s_at	7.82	7.03	8.21	7.87	7.74	7.67	7.68	0.60	7.76	0.11	1.01	0.014	8.4E-01
Mtr.22375.1.S1_at	8.05	6.93	7.85	8.00	7.86	7.92	7.61	0.60	7.92	0.07	1.04	0.058	4.2E-01
Mtr.45450.1.S1_x_at	6.42	5.37	6.40	6.17	6.36	6.22	6.06	0.60	6.25	0.10	1.03	0.043	6.2E-01
Mtr.37494.1.S1_at	10.86	11.92	10.91	11.87	12.21	12.06	11.23	0.60	12.05	0.17	1.07	0.101	8.6E-02
Mtr.41639.1.S1_at	8.55	7.37	8.14	8.28	8.08	8.49	8.02	0.60	8.28	0.20	1.03	0.046	5.1E-01
Mtr.44261.1.S1_at	11.32	11.12	12.24	11.63	11.98	11.62	11.56	0.60	11.74	0.20	1.02	0.023	6.3E-01
Mtr.9209.1.S1_at	6.44	5.48	6.59	6.46	6.20	6.13	6.17	0.60	6.26	0.17	1.02	0.022	8.1E-01
Mtr.31787.1.S1_at	7.28	6.54	7.73	7.51	7.77	7.33	7.18	0.60	7.54	0.22	1.05	0.070	3.9E-01
Mtr.17616.1.S1_x_at	10.57	9.38	10.05	9.99	9.91	10.20	10.00	0.60	10.03	0.15	1.00	0.005	9.3E-01
Mtr.9799.1.S1_at	8.22	8.03	9.15	8.44	8.19	8.12	8.47	0.60	8.25	0.17	0.97	-0.038	5.7E-01
Mtr.39625.1.S1_at	5.82	5.55	6.69	5.30	5.93	5.69	6.02	0.60	5.64	0.32	0.94	-0.095	3.8E-01
Mtr.25196.1.S1_at	11.90	11.51	12.69	12.96	13.10	12.45	12.03	0.60	12.84	0.34	1.07	0.094	1.1E-01
Mtr.15171.1.S1_at	2.64	3.72	2.74	2.72	2.57	2.71	3.03	0.60	2.67	0.08	0.88	-0.187	3.5E-01
Mtr.38681.1.S1_at	6.68	5.75	6.87	6.87	7.16	6.32	6.43	0.60	6.78	0.43	1.05	0.076	4.6E-01
Mtr.20264.1.S1_at	3.44	4.38	3.26	3.06	3.06	3.09	3.69	0.60	3.07	0.01	0.83	-0.267	1.5E-01
Mtr.32298.1.S1_at	7.53	6.48	7.49	7.55	7.74	7.61	7.17	0.60	7.63	0.09	1.06	0.091	2.5E-01
Mtr.9888.1.S1_at	3.58	2.56	2.52	2.51	3.30	2.69	2.88	0.60	2.83	0.42	0.98	-0.026	9.1E-01
Mtr.37858.1.S1_at	4.76	3.83	4.94	5.66	4.34	4.62	4.51	0.60	4.87	0.69	1.08	0.111	5.3E-01
Mtr.4804.1.S1_at	4.57	3.42	4.29	3.99	3.52	3.75	4.09	0.60	3.75	0.23	0.92	-0.125	4.1E-01
Mtr.42427.1.S1_at	9.04	8.09	7.94	8.60	9.41	8.77	8.36	0.60	8.92	0.43	1.07	0.094	2.5E-01
Mtr.51035.1.S1_at	6.69	6.05	7.25	6.36	6.68	6.56	6.66	0.60	6.53	0.16	0.98	-0.028	7.4E-01
Mtr.12744.1.S1_at	6.42	5.34	6.33	6.57	6.30	6.35	6.03	0.60	6.41	0.14	1.06	0.087	3.5E-01
Mtr.34436.1.S1_at	8.87	7.91	9.01	9.01	8.77	8.88	8.60	0.60	8.89	0.12	1.03	0.048	4.6E-01
Mtr.38606.1.S1_at	8.33	8.80	9.52	9.12	9.12	8.83	8.89	0.60	9.02	0.17	1.02	0.022	7.2E-01
Mtr.12654.1.S1_at	11.16	10.19	11.28	11.12	11.02	11.07	10.88	0.60	11.07	0.05	1.02	0.025	6.1E-01
Mtr.22420.1.S1_at	7.56	6.37	6.87	7.29	7.61	7.28	6.93	0.60	7.39	0.19	1.07	0.093	2.7E-01
Mtr.5263.1.S1_at	2.65	2.72	3.72	2.71	2.72	2.71	3.03	0.60	2.71	0.00	0.90	-0.159	4.1E-01
Mtr.11808.1.S1_at	9.72	9.07	10.26	10.02	10.01	9.85	9.68	0.60	9.96	0.09	1.03	0.041	4.7E-01
Mtr.8915.1.S1_at	2.63	3.74	3.57	3.67	4.14	3.70	3.31	0.60	3.83	0.26	1.16	0.211	2.4E-01
Mtr.37810.1.S1_at	8.74	7.77	8.86	8.69	8.64	8.70	8.46	0.60	8.68	0.03	1.03	0.037	5.6E-01
Mtr.12295.1.S1_at	9.10	8.61	9.80	8.87	8.79	8.41	9.17	0.60	8.69	0.25	0.95	-0.077	2.7E-01
Mtr.32186.1.S1_at	7.84	6.85	7.91	8.15	8.12	8.00	7.53	0.60	8.09	0.08	1.07	0.103	1.8E-01
Mtr.1704.1.S1_at	6.89	5.98	7.11	6.92	7.40	6.73	6.66	0.60	7.02	0.34	1.05	0.076	4.2E-01
Mtr.33064.1.S1_at	6.81	5.75	6.74	6.77	6.83	7.10	6.43	0.60	6.90	0.18	1.07	0.101	2.7E-01
Mtr.1011.1.S1_at	4.18	3.15	4.19	3.88	4.57	4.18	3.84	0.60	4.21	0.34	1.10	0.132	4.1E-01
Mtr.15885.1.S1_at	10.19	9.11	10.09	10.08	10.11	10.20	9.80	0.60	10.13	0.07	1.03	0.048	3.9E-01
Mtr.34872.1.S1_at	3.73	2.72	2.67	2.67	2.67	2.23	3.04	0.60	2.52	0.25	0.83	-0.270	2.4E-01
Mtr.10486.1.S1_at	12.10	11.29	12.46	12.34	12.27	12.14	11.95	0.60	12.25	0.10	1.03	0.036	4.4E-01
Mtr.32271.1.S1_at	2.58	3.59	3.64	2.56	2.12	2.56	3.27	0.60	2.41	0.25	0.74	-0.439	8.4E-02
Mtr.49920.1.S1_s_at	6.38	5.21	6.01	6.32	6.00	6.17	5.87	0.60	6.16	0.16	1.05	0.072	4.5E-01
Mtr.33645.1.S1_at	4.07	3.82	4.95	4.31	4.10	4.24	4.28	0.60	4.21	0.11	0.98	-0.022	8.6E-01
Mtr.41274.1.S1_at	8.96	8.23	9.42	9.08	9.12	8.83	8.87	0.60	9.01	0.16	1.02	0.023	7.1E-01
Mtr.29146.1.S1_at	8.57	9.75	9.08	8.16	8.64	7.72	9.13	0.60	8.17	0.46	0.89	-0.160	9.1E-02
Mtr.6811.1.S1_at	5.54	4.72	5.88	5.81	6.27	5.92	5.38	0.60	6.00	0.24	1.12	0.158	1.7E-01
Mtr.37469.1.S1_s_at	12.89	12.19	11.70	12.23	12.03	12.36	12.26	0.60	12.20	0.17	1.00	-0.006	8.9E-01
Mtr.36941.1.S1_at	9.66	8.59	9.57	10.40	10.17	10.12	9.27	0.60	10.23	0.15	1.10	0.142	5.4E-02
Mtr.45266.1.S1_at	9.53	8.34	9.00	9.41	9.06	9.33	8.96	0.60	9.27	0.19	1.03	0.049	4.4E-01
Mtr.38839.1.S1_s_at	6.03	5.27	6.44	6.37	6.40	6.50	5.91	0.60	6.42	0.07	1.09	0.119	2.2E-01
Mtr.26304.1.S1_at	8.74	7.68	8.68	8.62	8.20	8.59	8.37	0.60	8.47	0.23	1.01	0.018	7.9E-01
Mtr.5826.1.S1_x_at	6.24	5.27	6.35	5.96	6.64	6.93	5.95	0.60	6.51	0.50	1.09	0.129	2.8E-01
Mtr.28447.1.S1_at	4.10	3.05	3.09	2.98	3.66	3.88	3.41	0.60	3.51	0.47	1.03	0.039	8.4E-01
Mtr.28156.1.S1_at	8.35	7.20	8.05	8.16	8.39	7.90	7.87	0.60	8.15	0.25	1.04	0.051	4.9E-01
Mtr.12272.1.S1_at	14.20	13.02	13.48	13.40	12.76	13.24	13.57	0.60	13.14	0.33	0.97	-0.047	3.3E-01
Mtr.38047.1.S1_at	10.51	9.34	10.13	9.69	9.46	9.52	9.99	0.60	9.56	0.12	0.96	-0.064	2.8E-01
Mtr.35626.1.S1_at	11.06	10.20	11.34	10.75	10.89	10.58	10.87	0.60	10.74	0.16	0.99	-0.017	7.4E-01
Mtr.50934.1.S1_at	5.72	4.57	5.40	5.22	5.80	5.36	5.23	0.60	5.46	0.30	1.04	0.062	5.8E-01
Mtr.40748.1.S1_at	9.58	8.52	9.51	9.41	9.29	9.29	9.21	0.60	9.33	0.07	1.01	0.019	7.4E-01
Mtr.24865.1.S1_x_at	3.61	2.57	3.59	3.57	4.01	3.55	3.26	0.60	3.71	0.26	1.14	0.189	2.9E-01
Mtr.31207.1.S1_s_at	7.72	7.32	8.49	7.84	7.87	7.89	7.85	0.60	7.87	0.02	1.00	0.004	9.6E-01
Mtr.19006.1.S1_s_at	7.56	6.81	7.98	7.75	8.11	8.01	7.45	0.60	7.96	0.19	1.07	0.095	2.3E-01
Mtr.44591.1.S1_at	2.59	2.59	3.62	2.57	2.57	2.57	2.93	0.60	2.57	0.00	0.88	-0.189	3.5E-01
Mtr.19888.1.S1_at	3.33	3.41	4.39	3.36	5.53	4.23	3.71	0.60	4.37	1.09	1.18	0.238	4.1E-01
Mtr.12791.1.S1_at	10.16	9.13	9.13	9.51	9.75	9.79	9.48	0.60	9.68	0.15	1.02	0.031	6.0E-01
Mtr.50139.1.S1_at	5.84	6.85	5.80	5.79	5.75	5.88	6.16	0.59	5.81	0.07	0.94	-0.086	3.6E-01
Mtr.24334.1.S1_at	7.54	8.71	7.94	5.92	6.75	6.42	8.06	0.59	6.36	0.42	0.79	-0.342	1.5E-02
Mtr.33448.1.S1_s_at	11.77	11.51	12.65	12.27	12.29	12.13	11.98	0.59	12.23	0.09	1.02	0.030	5.1E-01
Mtr.38459.1.S1_at	10.50	9.31	9.84	10.05	10.11	10.08	9.88	0.59	10.08	0.03	1.02	0.029	6.0E-01
Mtr.1685.1.S1_at	5.08	4.03	5.04	5.06	4.96	5.17	4.72	0.59	5.06	0.10	1.07	0.102	3.8E-01
Mtr.34086.1.S1_at	7.80	7.18	8.37	7.94	8.09	7.72	7.78	0.59	7.92	0.19	1.02	0.025	7.3E-01
Mtr.35126.1.S1_at	2.48	3.51	2.47	2.46	2.47	2.45	2.82	0.59	2.46	0.01	0.87	-0.197	3.5E-01
Mtr.42646.1.S1_at	8.32	7.34	8.41	8.45	8.14	8.20	8.02	0.59	8.26	0.16	1.03	0.042	5.4E-01
Mtr.13796.1.S1_at	8.74	7.57	8.35	8.37	8.45	8.69	8.22	0.59	8.50	0.17	1.03	0.049	4.7E-01
Mtr.40465.1.S1_s_at	6.74	6.15	7.34	7.37	8.24	7.42	6.74	0.59	7.68	0.49	1.14	0.187	1.0E-01
Mtr.18590.1.S1_s_at	7.72	6.86	8.00	7.54	7.78	7.57	7.53	0.59	7.63	0.13	1.01	0.019	7.9E-01
Mtr.26382.1.S1_at	7.33	6.40	7.51	7.46	7.53	7.31	7.08	0.59	7.43	0.12	1.05	0.070	3.7E-01
Mtr.215.1.S1_at	6.37	6.16	7.28	6.54	7.05	6.66	6.60	0.59	6.75	0.27	1.02	0.031	7.2E-01
Mtr.17965.1.S1_at	4.97	4.00	5.07	4.46	4.29	4.46	4.68	0.59	4.41	0.10	0.94	-0.087	4.7E-01
Mtr.10790.1.S1_at	8.61	8.28	9.43	8.76	9.00	8.77	8.77	0.59	8.84	0.14	1.01	0.012	8.4E-01
Mtr.39699.1.S1_at	12.15	11.11	11.12	11.67	11.47	11.79	11.46	0.59	11.64	0.16	1.02	0.023	6.3E-01
Mtr.34816.1.S1_at	4.39	3.31	3.42	3.49	3.81	2.69	3.71	0.59	3.33	0.58	0.90	-0.154	4.8E-01
Mtr.30034.1.S1_at	10.54	9.53	10.58	10.16	10.41	10.14	10.22	0.59	10.24	0.15	1.00	0.003	9.6E-01
Mtr.39033.1.S1_at	6.83	5.87	6.95	7.02	6.59	7.00	6.55	0.59	6.87	0.24	1.05	0.068	4.4E-01
Mtr.23263.1.S1_at	6.97	8.03	7.03	7.32	6.93	7.40	7.34	0.59	7.22	0.25	0.98	-0.025	7.5E-01
Mtr.43953.1.S1_at	11.12	9.95	10.70	9.93	9.28	9.76	10.59	0.59	9.66	0.33	0.91	-0.133	7.7E-02
Mtr.24253.1.S1_at	9.47	8.29	8.96	8.98	9.13	8.86	8.91	0.59	8.99	0.13	1.01	0.014	8.2E-01
Mtr.39888.1.S1_at	6.36	5.32	6.34	6.69	7.44	7.01	6.01	0.59	7.05	0.38	1.17	0.231	6.2E-02
Mtr.5727.1.S1_at	7.82	7.10	8.28	7.99	7.78	7.83	7.73	0.59	7.87	0.11	1.02	0.025	7.2E-01
Mtr.35581.1.S1_at	8.00	8.85	9.14	8.57	8.54	8.30	8.66	0.59	8.47	0.15	0.98	-0.032	6.2E-01
Mtr.3075.1.S1_at	2.61	2.90	3.75	5.85	6.44	5.56	3.09	0.59	5.95	0.45	1.93	0.946	2.6E-03
Mtr.27045.1.S1_at	7.21	6.29	7.41	7.01	7.07	7.06	6.97	0.59	7.05	0.03	1.01	0.016	8.3E-01
Mtr.3187.1.S1_at	8.48	7.29	7.94	7.63	8.05	7.54	7.90	0.59	7.74	0.27	0.98	-0.030	6.8E-01
Mtr.7204.1.S1_at	10.08	9.07	10.12	10.06	9.97	10.02	9.76	0.59	10.02	0.05	1.03	0.038	4.9E-01
Mtr.8621.1.S1_s_at	7.58	6.56	7.60	9.08	9.42	8.87	7.25	0.59	9.12	0.28	1.26	0.331	7.8E-03
Mtr.44971.1.S1_at	6.72	6.42	7.56	6.59	6.97	6.74	6.90	0.59	6.77	0.19	0.98	-0.028	7.3E-01
Mtr.50754.1.S1_at	9.98	8.82	9.64	9.60	9.11	9.69	9.48	0.59	9.46	0.31	1.00	-0.002	9.7E-01
Mtr.50185.1.S1_at	8.87	7.94	7.76	7.94	8.20	8.03	8.19	0.59	8.06	0.13	0.98	-0.024	7.2E-01
Mtr.31126.1.S1_at	6.49	5.47	6.50	6.97	6.42	6.43	6.15	0.59	6.61	0.32	1.07	0.103	3.1E-01
Mtr.39155.1.S1_s_at	7.09	6.30	7.46	6.91	7.03	6.94	6.95	0.59	6.96	0.06	1.00	0.002	9.8E-01
Mtr.42286.1.S1_at	7.64	6.87	8.03	7.62	7.69	7.73	7.51	0.59	7.68	0.05	1.02	0.032	6.5E-01
Mtr.1550.1.S1_at	7.60	6.86	6.42	6.25	6.13	6.31	6.96	0.59	6.23	0.09	0.90	-0.159	1.0E-01
Mtr.27374.1.S1_at	8.51	8.14	7.35	7.82	7.58	7.85	8.00	0.59	7.75	0.15	0.97	-0.045	5.2E-01

Mtr.9086.1.S1_at	9.56	8.40	8.74	9.16	9.03	9.25	8.90	0.59	9.15	0.11	1.03	0.039	5.2E-01
Mtr.6696.1.S1_at	2.62	3.80	3.15	2.58	2.66	2.61	3.19	0.59	2.62	0.04	0.82	-0.285	1.7E-01
Mtr.39070.1.S1_at	7.50	6.34	7.13	6.84	7.12	6.74	6.99	0.59	6.90	0.20	0.99	-0.018	8.2E-01
Mtr.37074.1.S1_at	7.81	6.91	8.03	8.16	8.33	7.82	7.58	0.59	8.10	0.26	1.07	0.096	2.4E-01
Mtr.8963.1.S1_at	5.74	5.09	4.56	4.97	5.17	5.58	5.13	0.59	5.24	0.31	1.02	0.031	7.9E-01
Mtr.2487.1.S1_at	7.09	5.95	6.80	6.89	6.70	6.73	6.61	0.59	6.77	0.10	1.02	0.035	6.7E-01
Mtr.39095.1.S1_at	2.32	2.38	3.37	3.72	2.52	2.51	2.69	0.59	2.92	0.70	1.08	0.116	6.9E-01
Mtr.46779.1.S1_at	2.64	3.65	2.61	2.59	2.57	2.57	2.97	0.59	2.58	0.01	0.87	-0.202	3.2E-01
Mtr.2091.1.S1_at	9.31	8.34	9.42	9.35	9.42	9.24	9.02	0.59	9.34	0.09	1.03	0.049	4.2E-01
Mtr.37384.1.S1_at	6.40	6.72	7.55	6.40	5.88	5.68	6.89	0.59	5.99	0.37	0.87	-0.202	8.9E-02
Mtr.41237.1.S1_at	12.20	12.75	11.56	12.14	12.26	12.18	12.17	0.59	12.19	0.06	1.00	0.003	9.5E-01
Mtr.40354.1.S1_at	3.58	4.59	3.55	3.86	3.32	3.47	3.91	0.59	3.55	0.28	0.91	-0.140	3.9E-01
Mtr.4443.1.S1_at	5.13	4.50	5.68	4.22	4.16	4.53	5.10	0.59	4.30	0.20	0.84	-0.245	9.1E-02
Mtr.9181.1.S1_at	9.12	10.29	9.85	9.07	9.26	9.21	9.76	0.59	9.18	0.10	0.94	-0.088	1.7E-01
Mtr.39778.1.S1_at	7.72	6.58	7.43	7.73	7.70	7.76	7.24	0.59	7.73	0.03	1.07	0.094	2.3E-01
Mtr.4454.1.S1_at	8.63	9.62	9.69	8.54	8.87	8.34	9.31	0.59	8.58	0.27	0.92	-0.118	1.2E-01
Mtr.2859.1.S1_at	7.80	6.76	7.77	7.79	7.57	7.48	7.44	0.59	7.61	0.16	1.02	0.032	6.6E-01
Mtr.38198.1.S1_at	7.98	6.90	7.85	7.70	7.87	7.88	7.58	0.59	7.82	0.11	1.03	0.045	5.2E-01
Mtr.43920.1.S1_at	8.73	7.58	8.41	8.35	8.43	8.43	8.24	0.59	8.40	0.05	1.02	0.028	6.6E-01
Mtr.27165.1.S1_at	8.73	7.78	8.86	9.00	8.86	8.86	8.46	0.59	8.91	0.08	1.05	0.075	2.6E-01
Mtr.48959.1.S1_at	10.86	10.26	11.44	10.84	10.78	10.70	10.85	0.59	10.77	0.07	0.99	-0.010	8.3E-01
Mtr.9074.1.S1_at	11.72	11.05	10.54	11.21	11.39	11.22	11.10	0.59	11.27	0.10	1.02	0.022	6.5E-01
Mtr.50713.1.S1_at	9.90	9.05	8.77	9.47	9.36	9.57	9.24	0.59	9.47	0.10	1.02	0.035	5.5E-01
Mtr.50914.1.S1_at	2.72	3.74	2.71	2.71	2.67	2.66	3.06	0.59	2.68	0.03	0.88	-0.190	3.3E-01
Mtr.33130.1.S1_at	6.39	5.26	6.13	5.96	5.92	6.20	5.93	0.59	6.02	0.15	1.02	0.023	8.0E-01
Mtr.6850.1.S1_s_at	6.76	5.68	6.63	6.22	6.24	6.45	6.36	0.59	6.30	0.13	0.99	-0.012	8.9E-01
Mtr.45060.1.S1_at	11.11	9.99	10.21	10.68	9.94	10.71	10.44	0.59	10.44	0.44	1.00	0.000	1.0E+00
Mtr.21135.1.S1_at	11.64	10.51	11.39	10.87	10.45	10.87	11.18	0.59	10.73	0.24	0.96	-0.059	2.9E-01
Mtr.32583.1.S1_at	8.77	7.62	8.43	8.44	8.48	8.42	8.27	0.59	8.45	0.03	1.02	0.030	6.4E-01
Mtr.4250.1.S1_at	7.52	6.56	7.64	7.09	7.53	7.30	7.24	0.59	7.31	0.22	1.01	0.014	8.6E-01
Mtr.35431.1.S1_at	3.00	3.42	4.17	3.43	3.79	3.93	3.53	0.59	3.72	0.26	1.05	0.075	6.4E-01
Mtr.45785.1.S1_s_at	5.95	6.24	7.08	6.38	6.39	5.98	6.42	0.59	6.25	0.24	0.97	-0.039	6.6E-01
Mtr.19871.1.S1_at	9.36	8.24	9.12	9.52	9.98	9.88	8.90	0.59	9.79	0.24	1.10	0.137	7.3E-02
Mtr.36253.1.S1_at	7.53	6.55	7.61	7.20	7.61	7.28	7.23	0.59	7.36	0.22	1.02	0.026	7.3E-01
Mtr.14290.1.S1_at	8.18	7.13	8.12	7.85	7.53	7.40	7.81	0.59	7.59	0.23	0.97	-0.041	5.8E-01
Mtr.50207.1.S1_s_at	7.96	6.99	8.06	8.08	7.71	7.82	7.67	0.59	7.87	0.19	1.03	0.038	6.0E-01
Mtr.14235.1.S1_s_at	11.70	10.53	10.95	11.20	11.19	11.31	11.06	0.59	11.23	0.06	1.02	0.023	6.3E-01
Mtr.10244.1.S1_at	7.50	6.47	7.48	7.67	7.48	7.45	7.15	0.59	7.53	0.12	1.05	0.075	3.3E-01
Mtr.35748.1.S1_at	8.21	7.35	8.49	8.40	8.29	8.41	8.01	0.59	8.37	0.07	1.04	0.062	3.6E-01
Mtr.33816.1.S1_at	7.96	6.82	7.11	7.84	7.40	7.80	7.30	0.59	7.68	0.24	1.05	0.074	3.6E-01
Mtr.46489.1.S1_at	2.68	3.68	2.64	2.62	2.61	2.60	3.00	0.59	2.61	0.01	0.87	-0.200	3.2E-01
Mtr.32325.1.S1_at	10.51	11.69	11.19	10.68	11.30	10.94	11.13	0.59	10.97	0.31	0.99	-0.021	7.0E-01
Mtr.21261.1.S1_at	3.09	2.41	3.58	3.19	3.76	3.86	3.03	0.59	3.60	0.36	1.19	0.252	2.2E-01
Mtr.43651.1.S1_at	7.88	6.95	8.04	7.92	7.93	7.73	7.62	0.59	7.86	0.12	1.03	0.044	5.3E-01
Mtr.5779.1.S1_at	8.13	7.08	8.07	8.29	8.35	8.68	7.76	0.59	8.44	0.21	1.09	0.121	1.3E-01
Mtr.45001.1.S1_at	7.80	7.03	8.19	7.73	7.78	7.92	7.67	0.59	7.81	0.10	1.02	0.025	7.1E-01
Mtr.29814.1.S1_at	2.49	3.51	2.49	2.48	2.48	2.45	2.83	0.59	2.47	0.02	0.87	-0.198	3.5E-01
Mtr.43259.1.S1_at	12.25	12.93	11.76	12.23	12.54	12.49	12.31	0.59	12.42	0.17	1.01	0.012	7.8E-01
Mtr.40886.1.S1_at	10.73	9.98	11.15	10.90	10.57	10.45	10.62	0.59	10.64	0.23	1.00	0.003	9.5E-01
Mtr.32765.1.S1_at	8.18	7.00	7.60	7.99	7.98	8.28	7.60	0.59	8.08	0.17	1.06	0.089	2.4E-01
Mtr.24567.1.S1_at	2.29	3.30	2.27	2.26	2.25	2.24	2.62	0.59	2.25	0.01	0.86	-0.219	3.4E-01
Mtr.36138.1.S1_at	3.27	3.11	4.20	3.18	3.35	3.25	3.53	0.59	3.26	0.09	0.92	-0.114	4.8E-01
Mtr.3015.1.S1_at	8.13	6.99	7.28	8.39	8.92	8.44	7.47	0.59	8.58	0.29	1.15	0.201	4.3E-02
Mtr.29071.1.S1_at	5.85	6.20	7.00	6.36	6.13	6.28	6.35	0.59	6.26	0.12	0.99	-0.021	8.1E-01
Mtr.18042.1.S1_at	7.78	6.73	7.73	7.77	8.12	8.12	7.41	0.59	8.00	0.20	1.08	0.110	1.8E-01
Mtr.42596.1.S1_at	8.50	7.61	8.73	8.01	8.15	7.84	8.28	0.59	8.00	0.16	0.97	-0.050	4.7E-01
Mtr.8928.1.S1_at	9.06	8.62	9.79	9.29	9.71	9.12	9.16	0.59	9.37	0.31	1.02	0.034	6.0E-01
Mtr.48176.1.S1_at	2.93	3.93	2.89	2.88	2.82	2.80	3.25	0.59	2.83	0.04	0.87	-0.200	2.9E-01
Mtr.31302.1.S1_at	4.99	3.96	3.98	4.53	4.67	4.76	4.31	0.59	4.65	0.12	1.08	0.111	3.8E-01
Mtr.37510.1.S1_s_at	8.32	7.46	7.19	8.13	7.89	8.29	7.66	0.59	8.10	0.20	1.06	0.081	2.8E-01
Mtr.19442.1.S1_at	3.34	2.46	3.58	3.29	3.63	3.31	3.12	0.59	3.41	0.19	1.09	0.126	4.7E-01
Mtr.41509.1.S1_at	8.93	7.79	8.10	8.43	8.46	8.69	8.27	0.59	8.52	0.14	1.03	0.044	5.1E-01
Mtr.34869.1.S1_at	4.51	3.38	4.23	4.32	4.55	4.32	4.04	0.59	4.40	0.14	1.09	0.122	3.7E-01
Mtr.47345.1.S1_x_at	3.07	4.09	3.06	3.06	3.41	3.15	3.41	0.59	3.21	0.18	0.94	-0.087	6.0E-01
Mtr.45621.1.S1_s_at	9.48	10.29	10.62	10.04	10.50	10.16	10.13	0.59	10.23	0.24	1.01	0.014	8.0E-01
Mtr.6477.1.S1_at	2.17	3.18	2.15	2.15	2.15	2.14	2.50	0.59	2.15	0.00	0.86	-0.220	3.6E-01
Mtr.39195.1.S1_at	9.45	8.33	9.19	9.03	9.08	9.03	8.99	0.59	9.05	0.03	1.01	0.010	8.7E-01
Mtr.9919.1.S1_at	6.36	5.29	6.26	5.71	5.99	6.50	5.97	0.59	6.07	0.40	1.02	0.023	8.2E-01
Mtr.31713.1.S1_at	7.25	6.70	7.88	9.03	9.51	9.43	7.28	0.59	9.33	0.26	1.28	0.358	5.3E-03
Mtr.8201.1.S1_at	7.85	7.33	8.50	8.05	7.98	7.75	7.90	0.59	7.93	0.15	1.00	0.006	9.3E-01
Mtr.12607.1.S1_at	9.90	8.99	8.79	9.84	9.48	9.76	9.23	0.59	9.69	0.19	1.05	0.071	2.6E-01
Mtr.28048.1.S1_at	9.24	8.11	8.97	9.23	8.89	8.90	8.77	0.59	9.01	0.19	1.03	0.038	5.5E-01
Mtr.13852.1.S1_at	6.33	5.22	6.11	6.38	6.97	6.90	5.89	0.59	6.75	0.32	1.15	0.198	8.9E-02
Mtr.44638.1.S1_at	7.73	8.63	8.84	8.53	8.51	8.31	8.40	0.59	8.45	0.12	1.01	0.009	8.9E-01
Mtr.36838.1.S1_at	5.65	4.87	6.02	5.86	5.71	5.89	5.52	0.59	5.82	0.09	1.05	0.076	4.3E-01
Mtr.18566.1.S1_at	6.52	5.41	6.30	6.04	6.01	6.05	6.08	0.59	6.03	0.02	0.99	-0.011	9.0E-01
Mtr.27830.1.S1_at	6.93	5.90	6.90	7.11	6.92	6.91	6.58	0.59	6.98	0.11	1.06	0.086	3.1E-01
Mtr.27697.1.S1_at	3.10	3.14	4.14	4.89	4.30	4.44	3.46	0.59	4.54	0.31	1.31	0.392	4.8E-02
Mtr.7522.1.S1_at	8.62	7.62	8.65	8.70	8.63	8.78	8.30	0.59	8.71	0.07	1.05	0.069	3.0E-01
Mtr.9251.1.S1_at	9.98	8.87	9.08	9.71	9.47	9.72	9.31	0.59	9.63	0.14	1.03	0.049	4.1E-01
Mtr.18515.1.S1_at	8.06	7.07	8.11	8.09	8.05	8.02	7.75	0.59	8.06	0.03	1.04	0.057	4.1E-01
Mtr.2287.1.S1_at	6.45	5.45	6.48	6.46	6.36	6.58	6.13	0.59	6.47	0.11	1.06	0.078	3.8E-01
Mtr.8265.1.S1_at	5.19	4.10	4.26	4.04	5.18	4.27	4.52	0.59	4.50	0.61	1.00	-0.007	9.7E-01
Mtr.8585.1.S1_at	3.25	3.24	4.26	7.23	8.57	7.29	3.58	0.59	7.70	0.76	2.15	1.103	1.8E-03
Mtr.32055.1.S1_at	3.92	2.91	3.93	3.97	3.84	3.89	3.58	0.59	3.90	0.07	1.09	0.122	4.1E-01
Mtr.49212.1.S1_at	10.67	9.51	9.95	10.32	10.16	10.28	10.04	0.59	10.25	0.08	1.02	0.030	5.7E-01
Mtr.2857.1.S1_s_at	10.24	9.06	9.65	9.94	10.05	10.07	9.65	0.59	10.02	0.07	1.04	0.054	3.4E-01
Mtr.50547.1.S1_at	6.29	5.77	6.95	6.82	6.92	6.72	6.34	0.59	6.82	0.10	1.08	0.105	2.3E-01
Mtr.39210.1.S1_at	7.48	8.09	8.65	8.06	7.80	7.59	8.07	0.59	7.81	0.23	0.97	-0.047	5.2E-01
Mtr.23427.1.S1_at	5.98	7.15	6.65	6.38	6.45	6.28	6.59	0.59	6.37	0.09	0.97	-0.050	5.5E-01
Mtr.25428.1.S1_at	6.46	5.29	5.94	6.33	6.18	6.11	5.90	0.59	6.21	0.11	1.05	0.075	4.2E-01
Mtr.28989.1.S1_at	9.27	8.51	9.67	9.40	9.50	9.22	9.15	0.59	9.38	0.14	1.02	0.035	5.6E-01
Mtr.50952.1.S1_at	9.09	8.49	9.67	9.05	9.18	9.00	9.08	0.59	9.08	0.09	1.00	-0.001	9.9E-01
Mtr.40876.1.S1_at	10.64	9.47	10.08	10.24	10.02	10.37	10.07	0.59	10.21	0.18	1.01	0.021	7.0E-01
Mtr.17547.1.S1_at	3.34	2.36	3.40	4.46	5.52	3.94	3.04	0.59	4.64	0.81	1.53	0.612	5.0E-02
Mtr.44288.1.S1_at	11.22	10.12	10.31	10.74	10.90	10.75	10.55	0.59	10.80	0.09	1.02	0.033	5.1E-01
Mtr.10256.1.S1_at	7.76	6.77	7.82	7.82	7.73	7.80	7.45	0.59	7.78	0.05	1.04	0.063	3.8E-01
Mtr.15409.1.S1_at	6.13	5.01	5.88	5.97	5.71	5.91	5.68	0.59	5.86	0.14	1.03	0.047	6.2E-01
Mtr.42721.1.S1_at	6.55	5.94	7.12	6.39	6.71	6.80	6.54	0.59	6.63	0.21	1.01	0.021	8.0E-01
Mtr.23797.1.S1_s_at	11.27	11.98	10.81	10.70	10.92	10.98	11.36	0.59	10.87	0.14	0.96	-0.064	2.3E-01
Mtr.32161.1.S1_at	5.66	4.87	6.02	5.79	6.01	5.57	5.52	0.59	5.79	0.22	1.05	0.068	5.0E-01
Mtr.42007.1.S1_at	11.85	10.68	11.27	11.54	11.69	11.52	11.26	0.59	11.58	0.09	1.03	0.040	4.1E-01
Mtr.42385.1.S1_at	3.94	4.15	5.04	4.51	4.40	4.22	4.38	0.59	4.38	0.15	1.00	0.001	1.0E+00
Mtr.12763.1.S1_at	4.35	3.31	4.30	4.13	4.58	4.27	3.98	0.59	4.33	0.23	1.09	0.119	4.0E-01
Mtr.3722.1.S1_at	5.94	4.86	5.78	5.77	5.93	6.23	5.53	0.59	5.98	0.23	1.08	0.113	2.8E-01
Mtr.9023.1.S1_s_at	9.40	9.31	8.34	8.85	9.09	9.36	9.02	0.59	9.10	0.25	1.01	0.013	8.4E-01
Mtr.42638.1.S1_at	6.90	5.78	6.04	6.55	6.57	6.78	6.24	0.59	6.64	0.13	1.06	0.088	3.2E-01
Mtr.7262.1.S1_at	7.52	7.28	8.39	7.86	8.37	8.18	7.73	0.59	8.13	0.26	1.05	0.074	3.3E-01
Mtr.21508.1.S1_at	7.03	6.15	7.26	6.35	6.54	6.16	6.81	0.59	6.35	0.19	0.93	-0.102	2.6E-01
Mtr.5022.1.S1_s_at	6.51	5.36	6.13	6.26	6.22	6.25	6.00	0.59	6.24	0.02	1.04	0.059	5.0E-01
Mtr.20968.1.S1_s_at	5.81	5.53	6.66	6.22	6.08	6.10	6.00	0.59	6.13	0.07	1.02	0.031	7.2E-01
Mtr.15583.1.S1_at	8.16	7.25	8.34	7.64	8.06	7.72	7.92	0.59	7.80	0.22	0.99	-0.021	7.7E-01
Mtr.44327.1.S1_at	8.29	7.26	8.26	8.08	8.11	8.25	7.94	0.59	8.15	0.09	1.03	0.037	5.8E-01
Mtr.44642.1.S1_at	4.57	3.54	4.54	4.25	4.40	4.35	4.22	0.59	4.33	0.07	1.03	0.039	7.5E-01
Mtr.45029.1.S1_at	12.76	12.18	11.59	11.97	12.17	12.26	12.18	0.59	12.13	0.15	1.00	-0.006	9.0E-01
Mtr.42058.1.S1_s_at	8.55	8.19	9.33	8.68	8.72	8.84	8.69	0.59	8.75	0.08	1.01	0.009	8.8E-01
Mtr.5993.1.S1_at	2.36	3.37	2.35	2.35	2.35	2.35	2.69	0.59	2.35	0.00	0.87	-0.198	3.7E-01
Mtr.47791.1.S1_at	8.47	7.37	8.27	8.51	8.40	8.30	8.04	0.59	8.41	0.10	1.05	0.065	3.4E-01
Mtr.27561.1.S1_s_at	8.20	7.03	7.58	8.55	8.65	8.85	7.61	0.59	8.69	0.16	1.14	0.191	3.7E-02
Mtr.11995.1.S1_at	8.62	7.73	8.83	8.55	8.47	8.39	8.39	0.59	8.47	0.08	1.01	0.013	8.3E-01
Mtr.7580.1.S1_at	7.11	6.30	7.44	7.01	7.38	7.11	6.95	0.59	7.17	0.19	1.03	0.044	5.8E-01
Mtr.45284.1.S1_at	8.57	7.56	8.57	8.63	8.56	8.91	8.23	0.59	8.70	0.18	1.06	0.080	2.6E-01
Mtr.10290.1.S1_at	8.76	7.59	8.12	8.68	8.35	8.43	8.16	0.58	8.49	0.18	1.04	0.057	4.0E-01
Mtr.33344.1.S1_at	6.04	4.87	5.51	5.80	5.87	6.09	5.47	0.58	5.92	0.15	1.08	0.113	2.7E-01
Mtr.40419.1.S1_at	12.00	10.84	11.54	11.05	10.50	10.94	11.46	0.58	10.83	0.29	0.94	-0.082	1.7E-01
Mtr.27632.1.S1_at	6.21	5.05	5.75	6.03	5.99	5.81	5.67	0.58	5.94	0.12	1.05	0.068	4.7E-01
Mtr.7560.1.S1_at	8.85	7.88	8.93	8.75	8.78	8.50	8.55	0.58	8.68	0.15	1.01	0.021	7.4E-01
Mtr.40293.1.S1_at	10.44	10.76	9.63	10.61	10.49	10.61	10.28	0.58	10.57	0.07	1.03	0.041	4.4E-01
Mtr.16003.1.S1_at	8.23	7.10	7.94	8.44	8.67	8.88	7.76	0.58	8.66	0.22	1.12	0.159	6.6E-02
Mtr.22636.1.S1_at	11.74	10.58	11.29	11.99	11.98	11.69	11.20	0.58	11.89	0.17	1.06	0.086	1.2E-01
Mtr.13478.1.S1_at	6.27	5.39	6.49	6.52	6.62	6.57	6.05	0.58	6.57	0.05	1.09	0.120	2.0E-01

Mtr.35461.1.S1_at	10.08	10.75	11.25	10.75	10.74	10.80	10.69	0.58	10.76	0.03	1.01	0.010	8.4E-01
Mtr.6793.1.S1_at	4.30	3.46	4.59	4.50	4.29	3.72	4.12	0.58	4.17	0.40	1.01	0.019	9.0E-01
Mtr.27365.1.S1_at	10.43	9.30	10.12	10.12	10.15	10.18	9.95	0.58	10.15	0.03	1.02	0.029	5.8E-01
Mtr.1882.1.S1_at	7.72	6.92	8.06	7.85	8.00	7.84	7.57	0.58	7.90	0.09	1.04	0.062	3.8E-01
Mtr.38054.1.S1_at	11.10	10.13	11.18	10.88	10.71	10.83	10.80	0.58	10.81	0.09	1.00	0.001	9.9E-01
Mtr.17069.1.S1_x_at	4.26	4.39	3.32	4.70	5.52	4.61	3.99	0.58	4.94	0.50	1.24	0.310	9.7E-02
Mtr.49326.1.S1_at	5.46	6.27	5.14	5.14	4.38	4.82	5.62	0.58	4.78	0.38	0.85	-0.235	1.0E-01
Mtr.35517.1.S1_at	7.05	5.89	6.62	6.95	7.30	6.86	6.52	0.58	7.04	0.23	1.08	0.111	2.3E-01
Mtr.42372.1.S1_at	2.90	2.50	3.65	3.04	3.16	2.93	3.02	0.58	3.04	0.12	1.01	0.012	9.4E-01
Mtr.27149.1.S1_at	7.07	6.05	7.05	7.48	7.18	7.07	6.72	0.58	7.24	0.21	1.08	0.107	2.2E-01
Mtr.3008.1.S1_at	9.10	8.00	8.90	8.62	8.80	8.65	8.66	0.58	8.69	0.09	1.00	0.004	9.4E-01
Mtr.4727.1.S1_at	7.66	8.53	8.77	8.45	8.65	8.53	8.32	0.58	8.54	0.10	1.03	0.039	5.4E-01
Mtr.10567.1.S1_s_at	4.06	2.96	3.18	2.53	2.68	2.66	3.40	0.58	2.62	0.08	0.77	-0.374	8.5E-02
Mtr.6139.1.S1_at	7.95	7.19	8.34	8.34	8.09	7.97	7.83	0.58	8.13	0.19	1.04	0.055	4.4E-01
Mtr.45377.1.S1_at	5.33	4.25	5.17	4.93	5.58	5.53	4.92	0.58	5.35	0.36	1.09	0.120	3.4E-01
Mtr.27361.1.S1_at	7.58	6.48	7.38	7.59	7.53	7.48	7.15	0.58	7.53	0.06	1.05	0.077	3.1E-01
Mtr.10128.1.S1_at	7.53	6.60	7.67	7.49	7.55	7.69	7.27	0.58	7.58	0.10	1.04	0.061	4.1E-01
Mtr.20150.1.S1_at	8.95	8.30	9.46	8.98	9.03	9.28	8.90	0.58	9.09	0.16	1.02	0.031	6.1E-01
Mtr.34445.1.S1_at	8.63	7.51	8.34	8.38	8.75	8.46	8.16	0.58	8.53	0.19	1.05	0.064	3.6E-01
Mtr.41962.1.S1_at	8.09	6.99	7.87	8.29	8.19	8.32	7.65	0.58	8.27	0.07	1.08	0.112	1.4E-01
Mtr.41751.1.S1_at	9.70	8.99	10.15	9.29	9.35	8.89	9.61	0.58	9.17	0.25	0.95	-0.068	3.0E-01
Mtr.7484.1.S1_at	9.77	8.62	9.36	9.05	9.23	9.14	9.25	0.58	9.14	0.09	0.99	-0.017	7.6E-01
Mtr.13428.1.S1_at	10.97	11.61	12.14	11.44	11.66	11.38	11.57	0.58	11.49	0.15	0.99	-0.010	8.3E-01
Mtr.7562.1.S1_at	2.85	2.93	3.90	2.72	2.81	2.70	3.23	0.58	2.74	0.06	0.85	-0.235	2.3E-01
Mtr.33057.1.S1_at	6.18	5.38	6.51	6.41	5.94	5.88	6.02	0.58	6.08	0.29	1.01	0.013	8.9E-01
Mtr.26373.1.S1_at	2.98	3.98	2.96	2.96	2.93	2.96	3.31	0.58	2.95	0.02	0.89	-0.166	3.4E-01
Mtr.6054.1.S1_at	7.92	6.87	7.83	7.59	7.70	8.02	7.54	0.58	7.77	0.22	1.03	0.043	5.6E-01
Mtr.15511.1.S1_at	7.94	8.21	9.06	8.50	8.61	8.16	8.40	0.58	8.42	0.23	1.00	0.004	9.5E-01
Mtr.43555.1.S1_at	12.71	11.67	12.65	13.06	12.99	13.16	12.35	0.58	13.07	0.08	1.06	0.083	9.9E-02
Mtr.45342.1.S1_at	7.58	6.44	7.24	7.22	7.60	7.05	7.09	0.58	7.29	0.28	1.03	0.041	6.2E-01
Mtr.22065.1.S1_at	6.74	6.39	7.53	7.21	6.96	6.72	6.88	0.58	6.96	0.25	1.01	0.016	8.4E-01
Mtr.44428.1.S1_at	7.50	7.28	8.38	7.84	7.88	7.56	7.72	0.58	7.76	0.18	1.00	0.007	9.2E-01
Mtr.3232.1.S1_s_at	3.49	4.14	2.97	2.80	2.83	2.91	3.53	0.58	2.85	0.06	0.81	-0.310	1.1E-01
Mtr.27096.1.S1_at	10.70	9.67	10.64	10.77	10.30	10.86	10.34	0.58	10.64	0.30	1.03	0.042	4.6E-01
Mtr.11678.1.S1_at	9.96	8.82	9.59	9.64	9.68	9.56	9.45	0.58	9.63	0.06	1.02	0.026	6.3E-01
Mtr.36829.1.S1_at	9.46	8.31	8.99	9.09	9.24	9.00	8.92	0.58	9.11	0.12	1.02	0.031	6.1E-01
Mtr.20160.1.S1_s_at	8.22	7.48	8.62	8.03	8.11	7.79	8.11	0.58	7.98	0.16	0.98	-0.024	7.2E-01
Mtr.40112.1.S1_at	10.69	9.99	11.15	10.77	10.72	10.66	10.61	0.58	10.72	0.06	1.01	0.015	7.6E-01
Mtr.44799.1.S1_at	2.59	2.65	3.63	2.62	2.62	2.62	2.96	0.58	2.62	0.00	0.88	-0.177	3.7E-01
Mtr.10942.1.S1_at	9.37	8.20	8.81	8.48	8.78	8.48	8.79	0.58	8.58	0.17	0.98	-0.035	5.8E-01
Mtr.13668.1.S1_at	11.20	10.36	11.48	10.65	10.66	10.83	11.01	0.58	10.72	0.10	0.97	-0.039	4.4E-01
Mtr.13068.1.S1_at	8.48	7.89	9.06	8.98	8.92	8.91	8.48	0.58	8.93	0.04	1.05	0.076	2.4E-01
Mtr.34585.1.S1_at	9.85	8.71	9.48	9.16	9.57	9.23	9.35	0.58	9.32	0.22	1.00	-0.004	9.5E-01
Mtr.11573.1.S1_at	12.81	11.66	12.08	12.65	12.19	12.70	12.18	0.58	12.51	0.28	1.03	0.039	4.2E-01
Mtr.31257.1.S1_at	4.90	3.83	3.96	4.65	5.11	4.90	4.23	0.58	4.89	0.23	1.16	0.209	1.4E-01
Mtr.41352.1.S1_at	9.16	8.41	9.56	9.34	9.19	9.13	9.04	0.58	9.22	0.11	1.02	0.028	6.4E-01
Mtr.45109.1.S1_at	8.46	7.36	8.25	8.22	8.46	8.13	8.02	0.58	8.27	0.17	1.03	0.044	5.2E-01
Mtr.41950.1.S1_at	8.00	6.84	7.46	7.74	7.45	7.73	7.43	0.58	7.64	0.17	1.03	0.040	5.9E-01
Mtr.8553.1.S1_at	8.99	8.99	7.98	7.46	7.99	7.98	8.66	0.58	7.81	0.31	0.90	-0.148	9.0E-02
Mtr.14874.1.S1_x_at	3.17	4.16	3.13	3.10	3.29	3.15	3.48	0.58	3.18	0.10	0.91	-0.133	4.2E-01
Mtr.42315.1.S1_at	7.72	6.57	7.31	7.64	7.88	7.77	7.20	0.58	7.76	0.12	1.08	0.109	1.7E-01
Mtr.34496.1.S1_at	9.43	8.47	9.51	9.64	9.38	9.47	9.14	0.58	9.49	0.13	1.04	0.055	3.6E-01
Mtr.6905.1.S1_at	3.34	3.55	4.44	4.22	3.55	3.28	3.78	0.58	3.68	0.48	0.98	-0.036	8.4E-01
Mtr.21727.1.S1_at	2.88	3.74	2.63	3.08	3.46	4.08	3.08	0.58	3.54	0.51	1.15	0.201	3.6E-01
Mtr.20732.1.S1_at	8.74	7.91	9.03	9.18	9.13	9.04	8.56	0.58	9.11	0.07	1.06	0.091	1.8E-01
Mtr.21309.1.S1_s_at	9.82	8.71	9.55	9.51	9.67	9.37	9.36	0.58	9.51	0.15	1.02	0.024	6.8E-01
Mtr.21807.1.S1_at	3.21	4.11	3.03	3.27	3.20	3.18	3.45	0.58	3.22	0.05	0.93	-0.100	5.3E-01
Mtr.10642.1.S1_at	7.58	6.55	7.53	7.43	8.38	7.56	7.22	0.58	7.79	0.51	1.08	0.110	2.7E-01
Mtr.4658.1.S1_at	8.73	9.81	9.65	9.62	9.98	10.12	9.40	0.58	9.91	0.26	1.05	0.076	2.4E-01
Mtr.38116.1.S1_at	7.57	6.62	7.68	7.53	8.51	8.06	7.29	0.58	8.03	0.49	1.10	0.139	1.7E-01
Mtr.41861.1.S1_at	9.45	8.43	8.46	9.20	8.92	9.38	8.78	0.58	9.17	0.23	1.04	0.061	3.5E-01
Mtr.2343.1.S1_at	3.74	2.58	3.14	2.37	2.69	2.72	3.15	0.58	2.59	0.19	0.82	-0.283	1.9E-01
Mtr.1457.1.S1_at	5.86	4.92	5.99	5.96	5.76	5.55	5.59	0.58	5.76	0.21	1.03	0.042	6.7E-01
Mtr.43045.1.S1_at	4.41	5.42	4.42	4.40	4.33	4.34	4.75	0.58	4.36	0.04	0.92	-0.125	3.1E-01
Mtr.13255.1.S1_at	8.49	7.40	8.29	8.07	8.21	8.20	8.06	0.58	8.16	0.08	1.01	0.017	7.9E-01
Mtr.42305.1.S1_at	8.08	7.02	7.96	7.76	8.01	7.74	7.69	0.58	7.84	0.15	1.02	0.028	6.9E-01
Mtr.16538.1.S1_at	6.41	6.13	7.25	6.88	7.14	6.44	6.60	0.58	6.82	0.36	1.03	0.048	6.0E-01
Mtr.48411.1.S1_s_at	10.47	9.59	9.38	10.44	11.24	10.76	9.81	0.58	10.81	0.40	1.10	0.140	7.1E-02
Mtr.31667.1.S1_at	9.35	8.65	9.80	9.45	9.54	9.45	9.27	0.58	9.48	0.05	1.02	0.033	5.6E-01
Mtr.24388.1.S1_at	8.96	7.80	8.51	8.72	8.56	8.78	8.42	0.58	8.69	0.12	1.03	0.044	4.8E-01
Mtr.6833.1.S1_at	7.11	6.05	6.98	6.98	6.98	6.96	6.71	0.58	6.97	0.01	1.04	0.055	4.8E-01
Mtr.44730.1.S1_at	3.71	2.59	3.41	3.81	3.69	3.69	3.24	0.58	3.73	0.07	1.15	0.206	2.2E-01
Mtr.32433.1.S1_at	4.52	3.36	3.98	3.97	3.98	4.02	3.95	0.58	3.99	0.03	1.01	0.014	9.2E-01
Mtr.40994.1.S1_at	9.08	8.05	9.03	8.92	8.60	8.69	8.72	0.58	8.74	0.16	1.00	0.003	9.7E-01
Mtr.41568.1.S1_at	11.74	12.58	11.48	12.00	12.35	12.23	11.93	0.58	12.19	0.18	1.02	0.031	5.0E-01
Mtr.28513.1.S1_at	6.68	5.57	6.41	6.26	5.90	6.50	6.22	0.58	6.22	0.30	1.00	0.000	1.0E+00
Mtr.4576.1.S1_s_at	7.75	6.74	7.75	7.96	7.70	7.78	7.41	0.58	7.81	0.13	1.05	0.076	3.1E-01
Mtr.1151.1.S1_at	9.06	8.10	9.14	9.35	8.96	8.94	8.76	0.58	9.08	0.23	1.04	0.051	4.3E-01
Mtr.40933.1.S1_at	8.56	7.93	7.41	8.05	8.08	8.25	7.97	0.58	8.13	0.10	1.02	0.029	6.6E-01
Mtr.12513.1.S1_at	6.39	5.34	5.45	7.65	8.94	8.86	5.73	0.58	8.49	0.73	1.48	0.567	6.8E-03
Mtr.43241.1.S1_at	7.67	6.51	7.00	9.09	9.62	9.10	7.06	0.58	9.27	0.30	1.31	0.393	4.2E-03
Mtr.30103.1.S1_at	5.95	5.41	6.57	6.16	6.46	6.16	5.98	0.58	6.26	0.17	1.05	0.066	4.7E-01
Mtr.51081.1.S1_at	7.90	7.05	8.16	8.22	8.27	8.05	7.70	0.58	8.18	0.12	1.06	0.086	2.4E-01
Mtr.37992.1.S1_at	8.48	9.27	9.61	9.09	9.16	8.87	9.12	0.58	9.04	0.15	0.99	-0.012	8.3E-01
Mtr.26267.1.S1_at	3.05	4.00	2.96	2.93	2.93	2.92	3.34	0.58	2.93	0.00	0.88	-0.188	2.9E-01
Mtr.39261.1.S1_at	9.99	8.87	9.69	9.67	9.73	9.72	9.52	0.58	9.71	0.03	1.02	0.028	6.0E-01
Mtr.46025.1.S1_at	6.23	5.95	7.06	6.23	6.56	6.63	6.41	0.58	6.47	0.21	1.01	0.013	8.8E-01
Mtr.44093.1.S1_at	7.81	6.68	7.48	7.48	7.49	7.48	7.32	0.58	7.49	0.01	1.02	0.032	6.5E-01
Mtr.13650.1.S1_at	7.71	6.78	7.85	7.75	8.23	7.74	7.44	0.58	7.90	0.28	1.06	0.086	2.8E-01
Mtr.36371.1.S1_at	3.40	4.38	3.36	3.45	3.40	3.40	3.72	0.58	3.42	0.03	0.92	-0.120	4.2E-01
Mtr.1155.1.S1_at	3.91	3.16	4.29	4.28	4.07	3.79	3.79	0.58	4.05	0.25	1.07	0.096	5.1E-01
Mtr.34481.1.S1_at	6.37	5.73	6.88	6.48	6.46	6.03	6.33	0.58	6.32	0.25	1.00	-0.001	9.9E-01
Mtr.41079.1.S1_at	8.33	7.93	9.07	8.30	8.29	8.13	8.44	0.58	8.24	0.10	0.98	-0.035	5.9E-01
Mtr.39448.1.S1_at	10.73	9.58	10.09	10.45	10.41	10.41	10.13	0.58	10.42	0.03	1.03	0.041	4.3E-01
Mtr.32553.1.S1_at	7.94	6.91	7.88	8.05	8.17	7.64	7.58	0.58	7.95	0.28	1.05	0.069	3.7E-01
Mtr.44078.1.S1_at	8.53	7.39	7.82	7.88	7.97	7.88	7.92	0.58	7.91	0.05	1.00	-0.001	9.9E-01
Mtr.31379.1.S1_at	3.62	2.46	3.03	2.44	3.03	3.27	3.04	0.58	2.91	0.43	0.96	-0.060	7.8E-01
Mtr.49299.1.S1_at	2.95	3.93	2.90	2.93	2.84	2.90	3.26	0.58	2.89	0.04	0.89	-0.175	3.3E-01
Mtr.37670.1.S1_x_at	7.79	6.66	7.42	7.50	7.49	7.41	7.29	0.58	7.47	0.05	1.02	0.034	6.3E-01
Mtr.7512.1.S1_at	10.13	10.62	11.28	10.72	10.69	10.39	10.68	0.58	10.60	0.18	0.99	-0.011	8.3E-01
Mtr.45310.1.S1_at	6.77	5.67	5.92	5.89	5.97	5.89	6.12	0.58	5.92	0.04	0.97	-0.049	5.8E-01
Mtr.44416.1.S1_at	10.56	9.41	10.06	9.86	9.88	10.12	10.01	0.58	9.95	0.15	0.99	-0.009	8.7E-01
Mtr.6498.1.S1_at	7.69	7.59	8.64	8.26	8.02	8.10	7.97	0.58	8.13	0.12	1.02	0.027	6.8E-01
Mtr.45780.1.S1_at	6.86	6.00	7.09	6.85	6.85	6.81	6.65	0.58	6.83	0.02	1.03	0.039	6.1E-01
Mtr.21397.1.S1_s_at	7.20	6.81	7.95	7.45	7.53	7.49	7.32	0.58	7.49	0.04	1.02	0.032	6.4E-01
Mtr.15874.1.S1_s_at	7.83	6.86	7.88	7.82	7.84	7.64	7.52	0.58	7.77	0.11	1.03	0.046	5.1E-01
Mtr.42240.1.S1_at	6.37	5.22	5.69	7.11	6.57	7.10	5.76	0.58	6.93	0.31	1.20	0.266	3.7E-02
Mtr.21741.1.S1_s_at	7.87	7.69	8.77	8.40	8.35	8.44	8.11	0.58	8.40	0.04	1.04	0.050	4.4E-01
Mtr.34741.1.S1_at	3.98	3.56	4.70	4.65	5.37	4.68	4.08	0.58	4.90	0.41	1.20	0.262	1.2E-01
Mtr.24415.1.S1_at	10.87	10.51	9.74	9.56	9.79	9.91	10.37	0.58	9.75	0.17	0.94	-0.089	1.5E-01
Mtr.18823.1.S1_at	7.02	6.11	7.17	7.11	7.24	7.19	6.77	0.58	7.18	0.07	1.06	0.086	2.8E-01
Mtr.40628.1.S1_at	7.59	7.42	8.49	7.51	8.13	7.83	7.83	0.58	7.83	0.31	1.00	-0.001	9.9E-01
Mtr.37996.1.S1_at	9.78	8.63	9.16	9.55	9.48	9.76	9.19	0.58	9.60	0.14	1.04	0.063	3.0E-01
Mtr.17484.1.S1_x_at	8.32	7.48	8.59	8.12	8.19	7.80	8.13	0.58	8.04	0.21	0.99	-0.017	8.0E-01
Mtr.17996.1.S1_at	5.63	4.88	6.01	5.07	5.56	5.81	5.50	0.58	5.48	0.38	1.00	-0.006	9.6E-01
Mtr.11378.1.S1_at	6.22	5.10	5.93	6.07	6.17	5.88	5.75	0.58	6.04	0.15	1.05	0.072	4.4E-01
Mtr.41992.1.S1_at	7.73	6.75	7.76	7.61	7.54	7.66	7.41	0.58	7.60	0.06	1.03	0.036	6.0E-01
Mtr.16300.1.S1_at	4.01	3.37	2.86	3.26	3.13	3.12	3.41	0.58	3.17	0.08	0.93	-0.105	5.1E-01
Mtr.11170.1.S1_at	9.36	10.48	9.69	10.01	10.04	9.36	9.84	0.58	9.80	0.39	1.00	-0.006	9.2E-01
Mtr.8555.1.S1_at	5.99	4.84	5.30	5.17	5.38	5.36	5.38	0.58	5.31	0.11	0.99	-0.020	8.4E-01
Mtr.49797.1.S1_at	2.68	3.67	2.66	2.63	2.61	2.81	3.00	0.58	2.68	0.11	0.89	-0.162	4.0E-01
Mtr.1667.1.S1_at	6.81	5.91	6.98	6.96	6.93	6.79	6.57	0.58	6.89	0.09	1.05	0.070	3.9E-01
Mtr.1755.1.S1_at	7.11	6.29	7.40	7.41	7.57	7.36	6.93	0.58	7.45	0.11	1.07	0.104	2.0E-01
Mtr.34671.1.S1_at	5.63	4.51	5.31	5.51	5.81	5.25	5.15	0.58	5.52	0.28	1.07	0.101	3.7E-01
Mtr.50594.1.S1_x_at	5.38	4.51	5.59	5.39	5.50	4.80	5.16	0.58	5.23	0.38	1.01	0.020	8.7E-01
Mtr.14653.1.S1_at	2.48	3.42	2.37	2.47	2.46	2.46	2.76	0.58	2.46	0.01	0.89	-0.164	4.2E-01
Mtr.29313.1.S1_at	7.60	6.74	7.84	7.48	7.69	7.68	7.39	0.58	7.61	0.12	1.03	0.043	5.5E-01
Mtr.45901.1.S1_s_at	8.50	7.97	9.12	9.07	8.79	8.98	8.53	0.58	8.94	0.14	1.05	0.068	2.9E-01
Mtr.12065.1.S1_at	6.07	5.01	5.94	6.03	5.87	5.97	5.67	0.58	5.96	0.08	1.05	0.070	4.5E-01
Mtr.14736.1.S1_at	6.94	8.06	7.74	7.11	6.81	6.83	7.58	0.58	6.91	0.17	0.91	-0.133	1.3E-01
Mtr.34161.1.S1_at	9.41	8.63	8.29	8.58	8.55	8.77	8.78	0.58	8.63	0.12	0.98	-0.024	6.9E-01

Mtr.15133.1.S1_at	5.32	4.31	5.30	5.33	5.54	5.41	4.98	0.58	5.42	0.11	1.09	0.123	2.6E-01
Mtr.9380.1.S1_at	6.11	4.96	5.47	5.60	5.48	5.49	5.51	0.58	5.52	0.07	1.00	0.003	9.8E-01
Mtr.40656.1.S1_at	7.87	7.50	8.63	8.37	8.70	8.32	8.00	0.58	8.46	0.20	1.06	0.081	2.6E-01
Mtr.9308.1.S1_at	10.36	11.09	9.95	9.80	10.36	10.01	10.47	0.58	10.05	0.28	0.96	-0.058	3.3E-01
Mtr.45465.1.S1_at	5.76	4.63	5.01	4.67	5.36	5.36	5.13	0.58	5.13	0.40	1.00	0.000	1.0E+00
Mtr.1777.1.S1_at	8.47	7.78	8.92	8.82	8.78	8.78	8.39	0.58	8.79	0.02	1.05	0.067	2.9E-01
Mtr.22622.1.S1_at	2.01	3.01	2.00	2.00	2.00	2.00	2.34	0.58	2.00	0.00	0.85	-0.228	3.6E-01
Mtr.42878.1.S1_at	13.66	12.71	12.63	13.37	12.95	13.39	13.00	0.58	13.24	0.25	1.02	0.026	5.5E-01
Mtr.8408.1.S1_at	7.88	6.75	7.51	7.67	7.73	7.63	7.38	0.58	7.67	0.05	1.04	0.056	4.3E-01
Mtr.40609.1.S1_at	9.53	8.62	9.68	9.64	9.71	9.53	9.27	0.58	9.63	0.09	1.04	0.054	3.5E-01
Mtr.7739.1.S1_at	6.09	4.98	5.80	5.82	6.05	6.02	5.62	0.58	5.96	0.13	1.06	0.086	3.7E-01
Mtr.38689.1.S1_s_at	7.95	6.80	7.41	7.55	7.55	7.69	7.39	0.58	7.59	0.08	1.03	0.039	5.8E-01
Mtr.34175.1.S1_at	11.96	12.90	11.85	12.09	12.78	12.62	12.24	0.58	12.50	0.36	1.02	0.030	5.5E-01
Mtr.10583.1.S1_at	7.66	7.86	8.74	8.59	9.23	8.80	8.09	0.57	8.88	0.33	1.10	0.134	1.1E-01
Mtr.50787.1.S1_at	4.02	3.18	4.28	3.90	4.13	3.66	3.83	0.57	3.90	0.24	1.02	0.026	8.5E-01
Mtr.21944.1.S1_s_at	9.35	8.55	8.24	8.45	8.62	8.58	8.71	0.57	8.55	0.09	0.98	-0.027	6.5E-01
Mtr.2719.1.S1_at	10.69	9.59	10.43	10.43	10.16	10.52	10.23	0.57	10.37	0.19	1.01	0.019	7.2E-01
Mtr.7131.1.S1_at	7.54	8.16	8.69	8.13	8.28	8.19	8.13	0.57	8.20	0.07	1.01	0.012	8.5E-01
Mtr.11805.1.S1_at	8.88	7.91	8.94	8.82	8.71	8.91	8.58	0.57	8.81	0.10	1.03	0.039	5.2E-01
Mtr.9506.1.S1_at	8.97	7.95	8.91	8.82	9.16	9.20	8.61	0.57	9.06	0.21	1.05	0.073	2.7E-01
Mtr.16378.1.S1_at	2.49	3.56	2.65	2.48	2.49	2.48	2.90	0.57	2.48	0.01	0.86	-0.224	2.8E-01
Mtr.19104.1.S1_at	8.41	7.70	8.84	8.70	8.58	8.59	8.32	0.57	8.62	0.07	1.04	0.052	4.1E-01
Mtr.25360.1.S1_at	3.44	4.43	3.43	3.90	3.05	3.44	3.76	0.57	3.46	0.42	0.92	-0.120	5.1E-01
Mtr.39170.1.S1_at	6.07	5.51	6.65	5.86	5.76	6.09	6.08	0.57	5.90	0.17	0.97	-0.042	6.4E-01
Mtr.20539.1.S1_at	3.91	3.95	4.93	3.25	3.36	3.39	4.26	0.57	3.33	0.07	0.78	-0.355	5.0E-02
Mtr.37266.1.S1_s_at	5.92	6.02	6.96	6.34	6.65	6.51	6.30	0.57	6.50	0.16	1.03	0.045	5.9E-01
Mtr.26960.1.S1_at	7.43	6.28	6.77	6.82	7.33	7.08	6.82	0.57	7.08	0.25	1.04	0.052	5.3E-01
Mtr.44268.1.S1_at	9.58	8.53	9.45	9.38	9.85	9.51	9.18	0.57	9.58	0.24	1.04	0.061	3.3E-01
Mtr.34169.1.S1_at	8.26	7.28	8.29	8.41	8.30	8.38	7.95	0.57	8.36	0.06	1.05	0.073	2.8E-01
Mtr.17346.1.S1_s_at	4.40	3.30	4.12	4.11	4.56	4.10	3.94	0.57	4.26	0.26	1.08	0.111	4.4E-01
Mtr.29335.1.S1_at	8.36	7.38	7.34	7.58	7.37	7.50	7.69	0.57	7.48	0.10	0.97	-0.040	5.7E-01
Mtr.35681.1.S1_at	3.25	2.19	3.10	2.30	2.36	2.21	2.85	0.57	2.29	0.08	0.80	-0.316	1.7E-01
Mtr.15469.1.S1_at	8.48	7.47	8.46	8.53	8.43	8.48	8.14	0.57	8.48	0.05	1.04	0.060	3.6E-01
Mtr.7493.1.S1_at	4.80	3.98	5.09	5.00	5.13	4.75	4.62	0.57	4.96	0.19	1.07	0.101	3.9E-01
Mtr.42562.1.S1_at	9.34	8.48	9.56	9.33	9.50	9.22	9.12	0.57	9.35	0.14	1.02	0.035	5.4E-01
Mtr.9690.1.S1_at	9.05	8.12	9.17	8.94	8.91	8.85	8.78	0.57	8.90	0.05	1.01	0.020	7.3E-01
Mtr.29452.1.S1_at	7.61	7.52	8.55	8.21	8.46	7.86	7.89	0.57	8.18	0.31	1.04	0.051	4.9E-01
Mtr.31505.1.S1_at	8.75	8.24	9.38	8.91	9.05	8.62	8.79	0.57	8.86	0.22	1.01	0.012	8.5E-01
Mtr.14700.1.S1_s_at	8.23	7.25	8.26	8.13	8.22	8.20	7.92	0.57	8.18	0.05	1.03	0.048	4.7E-01
Mtr.17822.1.S1_at	8.21	7.21	8.20	8.12	8.02	7.93	7.87	0.57	8.02	0.10	1.02	0.027	6.8E-01
Mtr.5910.1.S1_at	7.10	6.49	7.63	7.55	7.42	7.62	7.07	0.57	7.53	0.10	1.06	0.091	2.4E-01
Mtr.47067.1.S1_at	8.98	8.36	9.51	9.14	9.13	8.84	8.95	0.57	9.04	0.17	1.01	0.014	8.2E-01
Mtr.38511.1.S1_at	9.13	8.25	9.32	9.26	9.19	9.02	8.90	0.57	9.16	0.12	1.03	0.041	4.9E-01
Mtr.9625.1.S1_at	8.98	8.40	7.83	8.98	8.65	9.13	8.40	0.57	8.92	0.24	1.06	0.086	2.2E-01
Mtr.42556.1.S1_at	8.00	8.59	9.15	8.63	8.75	8.68	8.58	0.57	8.69	0.06	1.01	0.017	7.7E-01
Mtr.18011.1.S1_at	7.75	8.24	8.89	8.33	8.19	8.26	8.29	0.57	8.26	0.07	1.00	-0.006	9.3E-01
Mtr.4105.1.S1_at	2.01	3.00	2.00	1.99	1.99	2.04	2.33	0.57	2.01	0.02	0.86	-0.218	3.8E-01
Mtr.589.1.S1_at	4.69	3.65	4.60	4.58	5.48	5.36	4.31	0.57	5.14	0.49	1.19	0.252	1.3E-01
Mtr.50721.1.S1_at	3.95	4.73	3.61	3.90	3.68	3.83	4.09	0.57	3.80	0.11	0.93	-0.106	4.4E-01
Mtr.31786.1.S1_at	5.17	4.20	5.22	4.74	4.92	4.91	4.86	0.57	4.86	0.10	1.00	0.000	1.0E+00
Mtr.7564.1.S1_at	12.02	11.12	10.96	11.41	11.36	11.61	11.37	0.57	11.46	0.13	1.01	0.012	8.0E-01
Mtr.45523.1.S1_s_at	7.93	6.88	7.81	8.09	7.85	7.94	7.54	0.57	7.96	0.12	1.06	0.078	2.8E-01
Mtr.13371.1.S1_at	8.60	7.62	8.62	8.60	8.47	8.61	8.28	0.57	8.56	0.08	1.03	0.048	4.5E-01
Mtr.44923.1.S1_at	9.20	8.47	9.60	9.63	9.69	9.56	9.09	0.57	9.63	0.06	1.06	0.083	1.8E-01
Mtr.31632.1.S1_at	7.10	7.00	8.04	6.45	7.39	5.90	7.38	0.57	6.58	0.75	0.89	-0.165	2.2E-01
Mtr.1564.1.S1_at	2.92	2.08	3.18	2.92	2.91	2.92	2.73	0.57	2.92	0.00	1.07	0.096	6.0E-01
Mtr.51024.1.S1_x_at	6.97	5.86	6.66	8.37	8.41	8.11	6.50	0.57	8.30	0.16	1.28	0.352	6.4E-03
Mtr.50767.1.S1_at	2.48	3.47	2.48	2.45	2.56	2.45	2.81	0.57	2.49	0.06	0.88	-0.179	3.8E-01
Mtr.16847.1.S1_at	6.68	7.10	7.81	7.21	7.08	6.81	7.19	0.57	7.03	0.21	0.98	-0.032	6.7E-01
Mtr.35074.1.S1_at	2.02	3.04	2.08	2.08	3.20	2.01	2.38	0.57	2.43	0.67	1.02	0.031	9.2E-01
Mtr.41857.1.S1_at	7.90	6.90	7.87	7.81	7.76	7.82	7.56	0.57	7.79	0.03	1.03	0.045	5.1E-01
Mtr.23090.1.S1_at	4.95	5.23	6.05	9.27	9.16	9.09	5.41	0.57	9.17	0.09	1.69	0.761	3.6E-04
Mtr.41449.1.S1_at	11.38	10.25	10.66	11.07	10.96	11.23	10.76	0.57	11.09	0.13	1.03	0.043	4.0E-01
Mtr.33312.1.S1_at	7.36	6.22	6.88	7.04	6.79	7.12	6.82	0.57	6.99	0.17	1.02	0.035	6.5E-01
Mtr.30223.1.S1_at	3.54	2.56	3.56	3.50	3.50	3.59	3.22	0.57	3.53	0.05	1.10	0.132	4.0E-01
Mtr.8714.1.S1_at	10.42	9.31	10.09	10.19	10.14	10.38	9.94	0.57	10.24	0.13	1.03	0.043	4.2E-01
Mtr.26384.1.S1_x_at	3.45	3.47	4.45	3.46	3.40	3.44	3.79	0.57	3.43	0.03	0.91	-0.143	3.4E-01
Mtr.50682.1.S1_at	12.04	11.10	11.01	11.29	11.13	11.40	11.38	0.57	11.28	0.14	0.99	-0.014	7.7E-01
Mtr.33009.1.S1_at	5.51	4.62	5.69	5.40	5.26	5.19	5.27	0.57	5.28	0.11	1.00	0.003	9.7E-01
Mtr.9565.1.S1_at	9.47	8.46	9.43	9.47	9.42	9.60	9.12	0.57	9.50	0.10	1.04	0.058	3.3E-01
Mtr.10130.1.S1_at	8.12	7.23	8.30	8.33	8.21	8.12	7.89	0.57	8.22	0.11	1.04	0.060	3.8E-01
Mtr.45252.1.S1_s_at	10.08	8.93	9.48	9.64	9.74	9.84	9.49	0.57	9.74	0.10	1.03	0.037	5.0E-01
Mtr.8171.1.S1_at	7.72	7.51	8.59	7.52	8.15	7.92	7.94	0.57	7.86	0.32	0.99	-0.014	8.5E-01
Mtr.38718.1.S1_s_at	10.10	9.00	9.27	9.68	9.60	9.80	9.46	0.57	9.69	0.10	1.02	0.036	5.2E-01
Mtr.33409.1.S1_at	4.51	3.36	3.97	4.50	4.49	5.10	3.95	0.57	4.70	0.35	1.19	0.250	1.2E-01
Mtr.36790.1.S1_at	7.14	6.47	7.61	7.53	7.69	7.57	7.07	0.57	7.60	0.08	1.07	0.103	1.9E-01
Mtr.32382.1.S1_at	8.16	7.07	7.93	8.19	8.28	8.38	7.72	0.57	8.28	0.09	1.07	0.101	1.7E-01
Mtr.6493.1.S1_at	7.71	6.85	7.93	7.97	7.74	7.87	7.49	0.57	7.86	0.11	1.05	0.069	3.4E-01
Mtr.38177.1.S1_s_at	6.96	6.74	7.82	7.66	7.45	7.62	7.18	0.57	7.58	0.11	1.06	0.078	3.0E-01
Mtr.41431.1.S1_at	9.10	7.96	8.59	8.52	8.50	8.87	8.55	0.57	8.63	0.21	1.01	0.013	8.3E-01
Mtr.48811.1.S1_at	11.86	11.08	12.19	12.13	11.99	11.87	11.71	0.57	11.99	0.13	1.02	0.035	4.5E-01
Mtr.26709.1.S1_s_at	8.62	7.79	8.88	8.85	8.91	8.73	8.43	0.57	8.83	0.09	1.05	0.067	3.0E-01
Mtr.23581.1.S1_at	3.60	3.04	4.18	3.66	3.52	3.38	3.61	0.57	3.52	0.14	0.98	-0.035	8.1E-01
Mtr.11072.1.S1_at	10.96	9.84	10.56	10.86	10.77	10.95	10.45	0.57	10.86	0.09	1.04	0.055	2.9E-01
Mtr.33769.1.S1_s_at	9.02	7.93	8.78	8.96	8.74	8.92	8.58	0.57	8.87	0.12	1.03	0.049	4.3E-01
Mtr.46013.1.S1_at	12.64	12.15	11.50	11.44	11.29	11.38	12.10	0.57	11.37	0.08	0.94	-0.089	9.5E-02
Mtr.8683.1.S1_at	6.03	5.63	6.75	6.83	7.52	7.06	6.14	0.57	7.14	0.35	1.16	0.218	6.1E-02
Mtr.38461.1.S1_at	10.30	9.17	9.88	10.12	10.15	10.06	9.78	0.57	10.11	0.05	1.03	0.047	3.8E-01
Mtr.4659.1.S1_at	8.88	9.71	9.97	9.71	10.21	10.02	9.52	0.57	9.98	0.25	1.05	0.068	2.7E-01
Mtr.48222.1.S1_s_at	7.77	6.64	7.34	7.28	7.60	7.76	7.25	0.57	7.55	0.25	1.04	0.058	4.6E-01
Mtr.6351.1.S1_at	3.16	2.28	3.34	3.18	3.70	3.42	2.93	0.57	3.43	0.26	1.17	0.229	2.4E-01
Mtr.39239.1.S1_at	7.53	6.54	7.54	8.15	7.85	7.67	7.20	0.57	7.89	0.24	1.10	0.132	1.3E-01
Mtr.30475.1.S1_at	7.22	6.22	7.21	6.94	7.17	7.24	6.88	0.57	7.12	0.16	1.03	0.049	5.3E-01
Mtr.27421.1.S1_s_at	4.22	3.15	4.01	4.09	3.32	4.01	3.79	0.57	3.81	0.42	1.00	0.004	9.8E-01
Mtr.40081.1.S1_at	7.28	7.04	8.13	7.67	7.48	7.68	7.48	0.57	7.61	0.12	1.02	0.025	7.2E-01
Mtr.34423.1.S1_at	8.77	9.23	9.91	9.29	9.29	9.09	9.30	0.57	9.22	0.12	0.99	-0.013	8.2E-01
Mtr.39328.1.S1_at	8.44	7.45	8.43	8.44	8.53	8.36	8.11	0.57	8.44	0.09	1.04	0.059	3.7E-01
Mtr.23604.1.S1_s_at	7.52	6.54	7.53	7.63	7.51	7.52	7.20	0.57	7.55	0.07	1.05	0.070	3.4E-01
Mtr.14624.1.S1_at	9.14	8.23	9.28	9.32	9.24	9.15	8.88	0.57	9.24	0.09	1.04	0.056	3.5E-01
Mtr.6065.1.S1_at	6.99	6.18	7.28	7.32	7.29	7.45	6.82	0.57	7.35	0.09	1.08	0.109	1.8E-01
Mtr.46435.1.S1_s_at	11.45	12.31	12.53	11.82	11.61	11.73	12.10	0.57	11.72	0.11	0.97	-0.046	3.2E-01
Mtr.13114.1.S1_at	7.23	6.24	7.22	7.22	7.19	7.26	6.89	0.57	7.22	0.04	1.05	0.067	3.7E-01
Mtr.28045.1.S1_at	6.91	5.77	6.37	6.39	6.39	6.65	6.35	0.57	6.48	0.15	1.02	0.028	7.3E-01
Mtr.175.1.S1_at	5.31	4.56	5.68	5.26	5.16	5.25	5.18	0.57	5.22	0.06	1.01	0.011	9.1E-01
Mtr.4140.1.S1_at	10.09	9.03	9.93	9.89	10.03	9.87	9.68	0.57	9.93	0.09	1.03	0.037	5.0E-01
Mtr.45069.1.S1_at	7.23	6.31	7.35	7.30	7.00	6.81	6.96	0.57	7.04	0.25	1.01	0.015	8.5E-01
Mtr.32215.1.S1_at	7.27	6.21	7.11	6.93	7.00	6.85	6.87	0.57	6.93	0.08	1.01	0.012	8.7E-01
Mtr.28556.1.S1_at	7.06	7.38	6.27	6.11	6.66	7.18	6.90	0.57	6.65	0.53	0.96	-0.054	6.0E-01
Mtr.44216.1.S1_at	8.26	7.48	8.59	7.86	8.14	8.19	8.11	0.57	8.06	0.18	0.99	-0.009	8.9E-01
Mtr.45376.1.S1_at	8.97	7.96	8.93	9.94	9.74	9.97	8.62	0.57	9.88	0.13	1.15	0.197	2.0E-02
Mtr.38153.1.S1_at	7.63	7.11	8.25	6.82	7.09	6.99	7.66	0.57	6.97	0.14	0.91	-0.136	1.1E-01
Mtr.10035.1.S1_at	6.89	6.50	7.62	7.23	6.86	6.53	7.00	0.57	6.87	0.35	0.98	-0.026	7.6E-01
Mtr.45667.1.S1_x_at	12.44	11.31	11.74	11.80	11.56	11.89	11.83	0.57	11.75	0.17	0.99	-0.010	8.2E-01
Mtr.30753.1.S1_at	3.24	2.29	2.22	2.19	2.20	2.23	2.58	0.57	2.21	0.02	0.86	-0.226	3.2E-01
Mtr.4868.1.S1_at	2.82	3.75	2.71	2.75	3.17	2.70	3.10	0.57	2.88	0.26	0.93	-0.105	5.8E-01
Mtr.34818.1.S1_at	4.65	3.85	4.96	4.09	4.22	4.11	4.48	0.57	4.14	0.07	0.92	-0.117	3.5E-01
Mtr.9603.1.S1_at	8.77	7.72	8.61	8.76	8.63	8.50	8.37	0.57	8.63	0.13	1.03	0.045	4.8E-01
Mtr.49390.1.S1_at	4.38	3.25	3.93	4.58	5.16	4.70	3.85	0.57	4.81	0.31	1.25	0.320	6.2E-02
Mtr.7073.1.S1_at	6.60	6.62	7.60	7.29	6.97	6.59	6.94	0.57	6.95	0.35	1.00	0.002	9.8E-01
Mtr.14004.1.S1_at	5.93	5.61	6.72	6.80	6.08	6.73	6.09	0.57	6.54	0.40	1.07	0.103	3.2E-01
Mtr.39942.1.S1_at	2.00	2.99	2.00	1.99	1.99	2.00	2.33	0.57	1.99	0.00	0.86	-0.223	3.7E-01
Mtr.11542.1.S1_s_at	8.57	7.61	8.62	8.46	8.48	8.48	8.26	0.57	8.47	0.01	1.03	0.036	5.6E-01
Mtr.8260.1.S1_at	3.75	2.64	3.40	3.08	3.11	3.08	3.26	0.57	3.09	0.02	0.95	-0.079	6.3E-01
Mtr.3872.1.S1_at	7.36	6.52	7.60	7.96	7.79	7.61	7.16	0.57	7.79	0.18	1.09	0.121	1.4E-01
Mtr.52309.1.S1_at	8.91	7.92	8.90	8.86	8.86	8.77	8.58	0.57	8.83	0.05	1.03	0.042	4.9E-01
Mtr.7775.1.S1_at	3.55	4.00	4.68	3.65	3.47	3.45	4.08	0.57	3.52	0.11	0.86	-0.212	1.7E-01
Mtr.18908.1.S1_s_at	7.75	6.62	7.32	7.08	7.27	7.14	7.23	0.57	7.16	0.10	0.99	-0.014	8.4E-01
Mtr.23468.1.S1_at	2.35	3.33	2.34	2.40	2.34	2.30	2.68	0.57	2.35	0.05	0.88	-0.190	3.7E-01
Mtr.26672.1.S1_s_at	4.14	3.78	4.89	4.27	4.70	4.32	4.27	0.57	4.43	0.24	1.04	0.052	6.8E-01
Mtr.25557.1.S1_at	10.69	9.55	10.07	10.42	10.35	10.71	10.10	0.57	10.49	0.20	1.04	0.055	3.2E-01
Mtr.34146.1.S1_at	7.42	6.76	7.90	7.57	7.51	7.52	7.36	0.57	7.53	0.03	1.02	0.033	6.3E-01

Mtr.7502.1.S1_at	7.85	6.87	7.87	7.92	7.78	7.38	7.53	0.57	7.69	0.28	1.02	0.031	6.8E-01
Mtr.23564.1.S1_at	2.51	3.49	2.50	2.50	2.49	2.49	2.84	0.57	2.49	0.01	0.88	-0.189	3.5E-01
Mtr.10456.1.S1_at	3.00	3.98	2.99	2.85	2.98	2.97	3.32	0.57	2.93	0.07	0.88	-0.180	3.1E-01
Mtr.23571.1.S1_at	2.70	3.58	2.52	2.67	2.67	2.92	2.94	0.57	2.75	0.14	0.94	-0.094	6.1E-01
Mtr.43217.1.S1_at	5.44	4.66	4.34	5.65	6.49	5.75	4.81	0.57	5.96	0.46	1.24	0.309	5.3E-02
Mtr.50407.1.S1_at	3.12	4.09	3.09	3.05	3.01	3.06	3.43	0.57	3.04	0.03	0.89	-0.175	3.0E-01
Mtr.41755.1.S1_at	7.12	8.08	8.12	7.60	7.39	7.67	7.77	0.57	7.55	0.14	0.97	-0.041	5.5E-01
Mtr.38930.1.S1_at	8.06	7.45	8.59	8.27	8.38	8.20	8.03	0.57	8.28	0.09	1.03	0.044	4.9E-01
Mtr.21519.1.S1_at	6.87	5.88	6.85	6.19	6.44	6.26	6.54	0.57	6.30	0.13	0.96	-0.054	5.2E-01
Mtr.7449.1.S1_at	11.84	10.71	11.29	11.41	11.17	11.50	11.28	0.57	11.36	0.17	1.01	0.010	8.3E-01
Mtr.37839.1.S1_at	7.77	7.16	8.30	7.69	7.64	7.24	7.74	0.57	7.52	0.25	0.97	-0.041	5.8E-01
Mtr.28628.1.S1_at	8.66	7.67	8.65	8.66	8.33	8.45	8.33	0.57	8.48	0.17	1.02	0.026	6.8E-01
Mtr.34912.1.S1_at	7.72	6.58	7.14	7.73	7.86	7.91	7.15	0.57	7.83	0.09	1.10	0.132	1.1E-01
Mtr.3503.1.S1_s_at	7.36	6.43	7.46	7.78	7.68	7.29	7.08	0.57	7.58	0.26	1.07	0.098	2.4E-01
Mtr.21319.1.S1_at	11.82	10.68	11.24	11.28	11.34	11.59	11.25	0.57	11.40	0.16	1.01	0.020	6.7E-01
Mtr.29962.1.S1_at	3.71	3.50	4.57	3.81	4.80	3.80	3.93	0.57	4.14	0.57	1.05	0.075	6.8E-01
Mtr.28332.1.S1_at	5.49	4.65	5.72	5.90	6.26	5.86	5.29	0.57	6.01	0.22	1.14	0.184	1.1E-01
Mtr.32034.1.S1_at	4.54	5.68	5.12	5.28	5.45	5.63	5.11	0.57	5.45	0.18	1.07	0.093	3.8E-01
Mtr.10044.1.S1_at	11.77	10.88	11.93	11.49	11.25	11.21	11.53	0.57	11.32	0.15	0.98	-0.026	5.7E-01
Mtr.4153.1.S1_at	3.42	2.33	2.60	2.26	3.12	2.70	2.79	0.57	2.69	0.43	0.97	-0.049	8.3E-01
Mtr.43149.1.S1_at	12.17	11.15	11.23	11.79	12.92	12.27	11.51	0.57	12.33	0.57	1.07	0.098	1.5E-01
Mtr.37579.1.S1_at	12.13	11.13	11.17	11.55	11.64	11.64	11.48	0.57	11.61	0.05	1.01	0.016	7.1E-01
Mtr.696.1.S1_at	3.44	3.42	4.41	4.43	4.87	4.49	3.76	0.57	4.60	0.24	1.22	0.291	7.8E-02
Mtr.41835.1.S1_at	9.43	8.43	9.40	9.33	9.29	9.37	9.09	0.57	9.33	0.04	1.03	0.038	5.0E-01
Mtr.24059.1.S1_at	2.47	3.44	2.44	2.45	2.43	2.44	2.78	0.57	2.44	0.01	0.88	-0.190	3.5E-01
Mtr.10861.1.S1_at	6.46	5.44	6.39	5.92	6.73	6.27	6.10	0.57	6.31	0.41	1.03	0.049	6.3E-01
Mtr.46955.1.S1_at	2.22	3.16	2.15	2.18	2.17	2.17	2.51	0.57	2.17	0.00	0.87	-0.208	3.6E-01
Mtr.1496.1.S1_at	8.40	9.48	9.23	8.65	9.46	9.16	9.03	0.57	9.09	0.41	1.01	0.009	9.0E-01
Mtr.11129.1.S1_at	6.29	6.36	7.31	6.51	6.53	5.83	6.65	0.57	6.29	0.40	0.95	-0.081	4.1E-01
Mtr.41872.1.S1_at	3.14	2.11	3.04	3.87	4.46	4.08	2.77	0.57	4.14	0.30	1.50	0.582	2.1E-02
Mtr.45017.1.S1_at	4.53	3.48	3.63	3.65	3.62	3.59	3.88	0.57	3.62	0.03	0.93	-0.100	4.7E-01
Mtr.4735.1.S1_at	7.92	7.21	8.33	7.97	8.30	7.90	7.82	0.57	8.06	0.22	1.03	0.043	5.4E-01
Mtr.5869.1.S1_at	7.57	6.50	7.35	7.27	6.44	7.05	7.14	0.57	6.92	0.43	0.97	-0.045	6.2E-01
Mtr.41675.1.S1_at	3.51	2.58	3.60	2.90	4.20	3.44	3.23	0.57	3.51	0.65	1.09	0.120	6.0E-01
Mtr.34934.1.S1_at	3.80	3.04	4.14	3.79	3.78	3.75	3.66	0.57	3.77	0.02	1.03	0.043	7.5E-01
Mtr.21924.1.S1_at	8.99	7.89	8.66	8.88	9.00	9.03	8.52	0.57	8.97	0.08	1.05	0.075	2.4E-01
Mtr.1705.1.S1_at	5.71	4.97	6.09	5.24	5.49	5.40	5.59	0.57	5.37	0.13	0.96	-0.057	5.5E-01
Mtr.35691.1.S1_at	8.43	7.56	8.63	8.27	8.63	8.25	8.20	0.57	8.38	0.21	1.02	0.031	6.4E-01
Mtr.728.1.S1_at	7.80	7.32	8.45	8.01	7.90	7.78	7.85	0.57	7.90	0.12	1.01	0.008	9.1E-01
Mtr.12228.1.S1_at	7.75	8.76	8.69	7.93	7.95	7.71	8.40	0.57	7.86	0.13	0.94	-0.095	1.9E-01
Mtr.5798.1.S1_at	8.06	7.04	7.99	7.65	7.69	7.56	7.70	0.57	7.63	0.07	0.99	-0.012	8.5E-01
Mtr.28800.1.S1_at	8.30	7.39	8.43	8.24	8.49	8.43	8.04	0.57	8.39	0.13	1.04	0.061	3.6E-01
Mtr.7917.1.S1_at	3.81	4.84	3.90	3.96	3.88	3.85	4.18	0.57	3.90	0.06	0.93	-0.102	4.3E-01
Mtr.17256.1.S1_s_at	7.99	7.08	8.12	7.52	8.00	7.71	7.73	0.57	7.74	0.24	1.00	0.003	9.7E-01
Mtr.39762.1.S1_at	4.30	3.31	4.29	4.07	4.88	4.48	3.97	0.57	4.48	0.41	1.13	0.174	2.7E-01
Mtr.32255.1.S1_at	2.90	3.88	2.90	2.94	3.04	2.93	3.23	0.57	2.97	0.06	0.92	-0.120	4.8E-01
Mtr.22956.1.S1_at	5.02	4.37	5.50	5.28	5.47	5.10	4.96	0.57	5.28	0.18	1.06	0.090	4.1E-01
Mtr.13965.1.S1_at	10.33	9.49	9.25	9.60	9.37	9.77	9.69	0.57	9.58	0.20	0.99	-0.016	7.7E-01
Mtr.35826.1.S1_at	10.54	9.49	10.38	10.05	9.97	9.84	10.14	0.57	9.95	0.11	0.98	-0.026	6.1E-01
Mtr.32021.1.S1_at	9.70	8.64	8.82	8.80	8.79	9.07	9.05	0.57	8.89	0.16	0.98	-0.027	6.5E-01
Mtr.36162.1.S1_at	9.14	8.08	8.96	8.77	8.80	8.70	8.73	0.57	8.75	0.05	1.00	0.005	9.3E-01
Mtr.191.1.S1_at	3.10	3.92	4.19	4.44	3.78	3.68	3.74	0.57	3.97	0.41	1.06	0.085	6.0E-01
Mtr.40707.1.S1_s_at	7.91	6.87	7.77	8.03	7.78	7.91	7.51	0.57	7.91	0.13	1.05	0.073	3.1E-01
Mtr.45123.1.S1_at	7.55	6.45	7.21	7.22	7.45	7.39	7.07	0.57	7.35	0.12	1.04	0.057	4.4E-01
Mtr.26339.1.S1_at	8.59	7.69	8.73	8.79	8.70	8.72	8.34	0.57	8.73	0.05	1.05	0.067	2.9E-01
Mtr.35382.1.S1_x_at	2.45	3.43	2.44	2.44	2.44	2.44	2.77	0.57	2.44	0.00	0.88	-0.184	3.7E-01
Mtr.13824.1.S1_at	8.49	7.40	7.67	7.70	7.69	7.69	7.85	0.57	7.69	0.01	0.98	-0.029	6.6E-01
Mtr.9668.1.S1_at	8.12	7.11	7.18	7.54	7.69	7.86	7.47	0.57	7.70	0.16	1.03	0.043	5.4E-01
Mtr.27546.1.S1_at	7.84	8.01	8.89	8.49	8.23	8.16	8.25	0.57	8.29	0.18	1.01	0.008	9.0E-01
Mtr.13513.1.S1_s_at	9.62	8.99	8.49	9.09	9.03	9.58	9.03	0.57	9.24	0.30	1.02	0.032	6.1E-01
Mtr.41624.1.S1_at	8.02	7.68	8.79	8.10	8.28	8.10	8.17	0.57	8.16	0.10	1.00	-0.002	9.8E-01
Mtr.24235.1.S1_at	9.54	8.45	9.26	9.34	9.19	9.27	9.08	0.57	9.27	0.08	1.02	0.030	6.0E-01
Mtr.8601.1.S1_at	12.20	11.08	11.80	12.52	12.27	12.58	11.69	0.57	12.46	0.17	1.07	0.092	8.8E-02
Mtr.18624.1.S1_at	7.25	6.47	7.57	7.08	7.07	6.98	7.10	0.57	7.04	0.06	0.99	-0.012	8.7E-01
Mtr.10844.1.S1_at	5.80	5.85	6.80	5.94	5.79	5.64	6.15	0.57	5.79	0.15	0.94	-0.087	3.5E-01
Mtr.17528.1.S1_at	5.05	4.88	5.93	5.47	5.52	5.50	5.29	0.57	5.50	0.03	1.04	0.056	5.6E-01
Mtr.27048.1.S1_at	7.88	7.15	8.27	8.10	8.27	8.23	7.77	0.57	8.20	0.09	1.06	0.078	2.6E-01
Mtr.27927.1.S1_at	8.01	7.10	8.14	8.13	7.99	7.98	7.75	0.57	8.03	0.09	1.04	0.052	4.4E-01
Mtr.36173.1.S1_at	4.88	3.94	4.96	5.07	4.96	5.05	4.59	0.57	5.02	0.06	1.09	0.129	2.6E-01
Mtr.2667.1.S1_at	8.86	8.17	7.74	8.96	8.21	8.78	8.26	0.57	8.65	0.39	1.05	0.066	3.8E-01
Mtr.50992.1.S1_at	9.97	8.88	9.69	9.80	9.61	9.63	9.51	0.57	9.68	0.10	1.02	0.025	6.4E-01
Mtr.21100.1.S1_at	4.32	5.21	4.16	4.43	4.24	4.41	4.56	0.57	4.36	0.10	0.96	-0.065	5.8E-01
Mtr.31606.1.S1_s_at	10.95	11.47	12.08	11.49	11.48	11.32	11.50	0.57	11.43	0.09	0.99	-0.009	8.5E-01
Mtr.38161.1.S1_at	8.71	8.11	9.24	8.85	9.07	8.95	8.69	0.57	8.96	0.11	1.03	0.044	4.7E-01
Mtr.41211.1.S1_at	10.44	9.54	10.58	10.20	10.37	10.42	10.19	0.57	10.33	0.12	1.01	0.020	6.9E-01
Mtr.31950.1.S1_at	10.09	9.07	9.15	9.84	9.91	10.11	9.44	0.56	9.95	0.14	1.06	0.077	2.0E-01
Mtr.28478.1.S1_at	8.73	7.67	8.55	8.67	8.78	8.75	8.32	0.56	8.73	0.06	1.05	0.071	2.7E-01
Mtr.29215.1.S1_at	6.35	6.04	7.14	6.96	6.77	7.09	6.51	0.56	6.94	0.16	1.07	0.092	2.8E-01
Mtr.5788.1.S1_s_at	8.34	7.39	8.40	8.30	8.11	8.26	8.05	0.56	8.22	0.10	1.02	0.031	6.2E-01
Mtr.19674.1.S1_at	6.17	5.95	7.02	6.82	7.14	6.70	6.38	0.56	6.89	0.23	1.08	0.111	2.2E-01
Mtr.39199.1.S1_at	4.64	3.51	4.03	3.99	5.35	3.68	4.06	0.56	4.34	0.89	1.07	0.097	6.7E-01
Mtr.39319.1.S1_at	9.17	8.18	9.16	9.07	9.02	8.91	8.84	0.56	9.00	0.08	1.02	0.027	6.4E-01
Mtr.3091.1.S1_at	5.85	5.32	6.45	5.84	5.84	5.84	5.87	0.56	5.84	0.00	0.99	-0.009	9.2E-01
Mtr.43636.1.S1_at	8.22	7.20	8.12	7.95	8.09	7.96	7.85	0.56	8.00	0.08	1.02	0.028	6.6E-01
Mtr.8882.1.S1_at	10.01	8.90	9.29	9.60	9.32	9.76	9.40	0.56	9.56	0.22	1.02	0.024	6.7E-01
Mtr.9227.1.S1_at	2.15	3.12	2.14	2.13	2.13	2.13	2.47	0.56	2.13	0.00	0.86	-0.212	3.6E-01
Mtr.30952.1.S1_at	7.01	7.26	8.09	7.44	7.46	6.67	7.45	0.56	7.19	0.45	0.96	-0.052	5.6E-01
Mtr.6873.1.S1_at	5.38	4.55	5.63	5.75	6.23	6.12	5.19	0.56	6.03	0.25	1.16	0.217	7.8E-02
Mtr.20242.1.S1_x_at	2.56	3.52	2.53	2.72	2.54	2.53	2.87	0.56	2.60	0.11	0.90	-0.145	4.5E-01
Mtr.29294.1.S1_at	7.55	7.50	8.50	8.41	8.12	8.17	7.85	0.56	8.23	0.16	1.05	0.068	3.3E-01
Mtr.5815.1.S1_at	5.33	4.69	5.81	5.35	5.52	5.44	5.28	0.56	5.44	0.09	1.03	0.043	6.5E-01
Mtr.5085.1.S1_at	5.81	4.72	5.54	5.50	5.66	5.47	5.36	0.56	5.54	0.10	1.03	0.049	6.0E-01
Mtr.50958.1.S1_s_at	3.28	2.31	3.29	3.14	3.77	3.12	2.96	0.56	3.34	0.37	1.13	0.177	3.8E-01
Mtr.35143.1.S1_s_at	8.49	7.52	8.50	8.53	8.60	8.39	8.17	0.56	8.51	0.10	1.04	0.058	3.7E-01
Mtr.45647.1.S1_at	7.61	6.51	7.28	7.71	7.43	7.57	7.13	0.56	7.57	0.14	1.06	0.086	2.6E-01
Mtr.13463.1.S1_at	7.14	6.86	7.95	7.48	7.67	7.51	7.32	0.56	7.55	0.10	1.03	0.046	5.2E-01
Mtr.44080.1.S1_at	11.20	10.41	11.50	12.14	12.03	12.17	11.03	0.56	12.11	0.07	1.10	0.135	3.0E-02
Mtr.5443.1.S1_at	6.39	5.28	5.99	6.72	6.74	6.65	5.89	0.56	6.70	0.05	1.14	0.187	6.7E-02
Mtr.11741.1.S1_at	5.07	6.07	6.01	5.87	6.06	5.95	5.72	0.56	5.96	0.10	1.04	0.061	5.0E-01
Mtr.29620.1.S1_at	7.21	6.51	6.10	6.99	7.29	7.15	6.60	0.56	7.14	0.15	1.08	0.113	1.9E-01
Mtr.44555.1.S1_at	9.22	8.10	8.52	8.39	8.42	8.38	8.62	0.56	8.40	0.02	0.97	-0.037	5.4E-01
Mtr.26801.1.S1_at	7.55	7.76	8.62	8.22	8.16	8.03	7.98	0.56	8.14	0.10	1.02	0.029	6.5E-01
Mtr.14364.1.S1_at	2.61	3.57	2.58	2.60	2.59	2.61	2.92	0.56	2.60	0.01	0.89	-0.167	3.8E-01
Mtr.39679.1.S1_at	7.98	8.65	7.53	9.30	9.99	9.92	8.06	0.56	9.74	0.38	1.21	0.273	1.3E-02
Mtr.26825.1.S1_at	7.77	7.00	8.09	7.99	8.20	7.86	7.62	0.56	8.02	0.17	1.05	0.073	3.1E-01
Mtr.37173.1.S1_s_at	5.32	4.21	4.59	4.97	4.94	5.13	4.71	0.56	5.01	0.10	1.06	0.090	4.1E-01
Mtr.16999.1.S1_at	3.01	3.16	4.05	3.21	3.67	2.90	3.41	0.56	3.26	0.39	0.96	-0.062	7.3E-01
Mtr.8653.1.S1_at	5.43	4.32	5.07	4.20	4.25	4.24	4.94	0.56	4.23	0.03	0.86	-0.223	9.6E-02
Mtr.50808.1.S1_at	9.23	8.20	9.11	8.89	9.16	8.73	8.85	0.56	8.93	0.21	1.01	0.013	8.3E-01
Mtr.33886.1.S1_at	9.43	8.33	9.10	9.42	9.29	9.18	8.95	0.56	9.30	0.12	1.04	0.054	3.6E-01
Mtr.15714.1.S1_s_at	7.63	8.06	8.75	7.97	7.95	7.74	8.14	0.56	7.89	0.13	0.97	-0.047	4.8E-01
Mtr.17931.1.S1_at	2.65	3.62	2.64	2.62	2.62	2.89	2.97	0.56	2.71	0.16	0.91	-0.129	4.9E-01
Mtr.39436.1.S1_at	9.09	7.98	8.68	8.87	8.99	8.35	8.58	0.56	8.74	0.34	1.02	0.026	7.0E-01
Mtr.44843.1.S1_at	7.93	8.91	7.94	8.27	8.20	8.40	8.26	0.56	8.29	0.10	1.00	0.006	9.3E-01
Mtr.2596.1.S1_at	3.36	2.24	2.70	3.68	3.34	3.43	2.76	0.56	3.49	0.18	1.26	0.335	1.0E-01
Mtr.33449.1.S1_at	7.91	6.81	7.56	7.72	7.86	7.78	7.42	0.56	7.79	0.07	1.05	0.069	3.3E-01
Mtr.22056.1.S1_at	3.81	4.77	3.79	3.77	3.77	3.78	4.12	0.56	3.77	0.01	0.91	-0.129	3.4E-01
Mtr.13372.1.S1_at	8.03	7.87	8.91	8.59	8.55	8.53	8.27	0.56	8.56	0.03	1.03	0.049	4.3E-01
Mtr.49068.1.S1_at	7.17	6.62	7.74	7.19	7.21	7.04	7.18	0.56	7.15	0.09	1.00	-0.006	9.3E-01
Mtr.40658.1.S1_x_at	6.46	5.44	6.37	7.10	6.94	6.96	6.09	0.56	7.00	0.09	1.15	0.200	5.1E-02
Mtr.40998.1.S1_at	7.91	6.99	8.01	7.83	7.83	7.95	7.64	0.56	7.87	0.07	1.03	0.043	5.2E-01
Mtr.46681.1.S1_at	6.26	5.31	6.30	6.37	6.23	6.21	5.96	0.56	6.27	0.09	1.05	0.074	4.0E-01
Mtr.31814.1.S1_at	9.35	8.44	9.48	9.59	9.34	9.40	9.09	0.56	9.44	0.13	1.04	0.055	3.5E-01
Mtr.5225.1.S1_at	7.10	6.02	6.82	7.07	7.04	7.00	6.65	0.56	7.04	0.04	1.06	0.083	3.0E-01
Mtr.28175.1.S1_at	5.48	6.41	6.50	6.28	5.85	5.41	6.13	0.56	5.84	0.44	0.95	-0.070	5.2E-01
Mtr.14686.1.S1_at	10.12	9.07	9.95	9.63	9.57	9.49	9.71	0.56	9.56	0.07	0.98	-0.023	6.7E-01
Mtr.49250.1.S1_at	8.25	7.50	8.60	8.51	8.55	8.76	8.12	0.56	8.61	0.13	1.06	0.085	2.2E-01
Mtr.14246.1.S1_at	7.01	5.89	6.38	6.76	6.64	6.64	6.43	0.56	6.68	0.07	1.04	0.056	4.8E-01
Mtr.281.1.S1_at	7.68	8.73	7.86	7.62	7.74	7.70	8.09	0.56	7.69	0.06	0.95	-0.074	2.8E-01
Mtr.25211.1.S1_at	9.39	10.27	9.23	8.62	9.68	9.62	9.63	0.56	9.30	0.60	0.97	-0.050	5.3E-01
Mtr.39945.1.S1_at	6.90	5.86	6.02	6.35	5.82	6.40	6.26	0.56	6.19	0.32	0.99	-0.016	8.6E-01

Mtr.1725.1.S1_at	7.41	6.37	7.26	7.34	7.17	7.39	7.02	0.56	7.30	0.12	1.04	0.057	4.4E-01
Mtr.20672.1.S1_at	7.59	7.20	8.30	7.77	8.07	7.69	7.70	0.56	7.85	0.20	1.02	0.028	6.9E-01
Mtr.11891.1.S1_at	4.71	5.69	4.72	5.79	5.77	5.86	5.04	0.56	5.81	0.05	1.15	0.204	7.8E-02
Mtr.2023.1.S1_at	5.34	5.09	6.16	5.73	6.51	5.71	5.53	0.56	5.99	0.46	1.08	0.115	3.3E-01
Mtr.8688.1.S1_at	10.20	9.92	11.00	10.58	11.25	10.30	10.37	0.56	10.71	0.49	1.03	0.046	4.8E-01
Mtr.22135.1.S1_s_at	4.32	3.73	3.20	4.33	4.30	4.29	3.75	0.56	4.31	0.02	1.15	0.199	1.6E-01
Mtr.39670.1.S1_at	6.07	4.97	5.71	5.55	6.33	5.94	5.59	0.56	5.94	0.39	1.06	0.088	4.2E-01
Mtr.20085.1.S1_at	6.01	4.90	5.59	6.29	5.92	6.05	5.50	0.56	6.09	0.19	1.11	0.147	1.6E-01
Mtr.41617.1.S1_at	8.92	8.04	9.08	8.96	8.98	8.99	8.68	0.56	8.98	0.02	1.03	0.049	4.0E-01
Mtr.36996.1.S1_at	8.54	7.51	8.42	7.61	8.03	7.80	8.16	0.56	7.81	0.21	0.96	-0.062	3.8E-01
Mtr.47862.1.S1_at	3.49	4.40	3.38	3.55	3.37	3.77	3.76	0.56	3.56	0.20	0.95	-0.077	6.0E-01
Mtr.35895.1.S1_at	8.26	7.61	8.73	8.15	8.20	8.19	8.20	0.56	8.18	0.03	1.00	-0.003	9.5E-01
Mtr.7004.1.S1_at	8.26	7.15	7.88	8.21	7.89	8.19	7.76	0.56	8.10	0.18	1.04	0.061	3.8E-01
Mtr.9905.1.S1_at	8.82	7.70	8.14	8.36	8.51	8.66	8.22	0.56	8.51	0.15	1.03	0.050	4.4E-01
Mtr.36348.1.S1_at	7.99	7.28	8.39	7.95	7.97	7.73	7.89	0.56	7.89	0.13	1.00	0.000	9.9E-01
Mtr.30416.1.S1_at	3.97	2.85	3.41	3.24	3.40	3.31	3.41	0.56	3.32	0.08	0.97	-0.039	7.9E-01
Mtr.8696.1.S1_at	12.70	12.48	11.64	12.85	12.87	13.01	12.28	0.56	12.91	0.09	1.05	0.073	1.3E-01
Mtr.27000.1.S1_at	9.29	8.45	9.51	9.42	9.37	9.26	9.08	0.56	9.35	0.08	1.03	0.042	4.6E-01
Mtr.30496.1.S1_at	7.37	6.52	7.58	7.42	7.54	7.56	7.16	0.56	7.50	0.08	1.05	0.068	3.5E-01
Mtr.26627.1.S1_at	8.81	7.75	8.61	8.83	8.90	8.87	8.39	0.56	8.87	0.04	1.06	0.079	2.2E-01
Mtr.13171.1.S1_at	11.62	10.90	12.00	11.67	12.05	11.81	11.51	0.56	11.85	0.19	1.03	0.042	3.8E-01
Mtr.20426.1.S1_at	2.77	2.12	3.23	3.28	3.90	3.65	2.71	0.56	3.61	0.31	1.33	0.415	7.1E-02
Mtr.49989.1.S1_at	6.88	6.38	7.50	6.99	7.05	6.83	6.92	0.56	6.96	0.11	1.01	0.008	9.1E-01
Mtr.35020.1.S1_s_at	8.18	7.24	8.25	8.09	8.20	8.26	7.89	0.56	8.18	0.09	1.04	0.053	4.2E-01
Mtr.32250.1.S1_s_at	9.46	8.77	9.88	9.69	9.51	9.76	9.37	0.56	9.65	0.13	1.03	0.042	4.5E-01
Mtr.20727.1.S1_at	6.68	7.80	7.32	7.12	7.87	6.92	7.27	0.56	7.30	0.50	1.00	0.007	9.4E-01
Mtr.5150.1.S1_at	3.97	2.86	3.24	3.84	4.05	3.85	3.36	0.56	3.91	0.12	1.17	0.221	1.7E-01
Mtr.32156.1.S1_at	6.65	5.83	6.91	6.83	6.20	6.58	6.46	0.56	6.54	0.32	1.01	0.016	8.5E-01
Mtr.46691.1.S1_at	7.28	6.32	7.30	7.53	7.49	7.35	6.97	0.56	7.46	0.10	1.07	0.098	2.1E-01
Mtr.1331.1.S1_s_at	7.58	6.79	7.88	7.74	7.50	7.65	7.42	0.56	7.63	0.12	1.03	0.041	5.6E-01
Mtr.12466.1.S1_at	8.56	8.46	9.47	9.88	10.18	9.98	8.83	0.56	10.01	0.15	1.13	0.182	2.4E-02
Mtr.14519.1.S1_at	8.14	7.62	8.74	8.18	8.27	8.20	8.17	0.56	8.22	0.05	1.01	0.009	8.8E-01
Mtr.37180.1.S1_at	6.72	6.50	7.56	7.33	7.26	7.35	6.93	0.56	7.31	0.05	1.06	0.078	3.0E-01
Mtr.33503.1.S1_x_at	4.16	3.46	4.57	4.10	4.16	4.65	4.06	0.56	4.30	0.30	1.06	0.084	5.5E-01
Mtr.41758.1.S1_at	11.25	10.14	10.79	10.45	10.39	10.35	10.72	0.56	10.40	0.05	0.97	-0.045	3.7E-01
Mtr.29320.1.S1_at	8.28	7.27	8.18	8.31	8.28	8.06	7.91	0.56	8.22	0.14	1.04	0.055	4.1E-01
Mtr.48826.1.S1_at	6.56	6.74	7.60	6.80	6.84	6.86	6.97	0.56	6.83	0.03	0.98	-0.028	7.0E-01
Mtr.38216.1.S1_at	12.69	11.58	12.29	12.19	12.17	12.15	12.19	0.56	12.17	0.02	1.00	-0.002	9.6E-01
Mtr.29718.1.S1_at	7.66	6.56	7.29	7.07	7.43	7.28	7.17	0.56	7.26	0.18	1.01	0.018	8.0E-01
Mtr.38247.1.S1_at	12.16	11.13	11.28	11.30	11.42	11.48	11.52	0.56	11.40	0.09	0.99	-0.015	7.3E-01
Mtr.49081.1.S1_at	2.25	3.20	3.24	2.88	3.11	3.18	2.90	0.56	3.06	0.16	1.06	0.080	6.5E-01
Mtr.33450.1.S1_at	2.13	3.10	2.13	2.13	2.13	2.12	2.45	0.56	2.13	0.00	0.87	-0.207	3.7E-01
Mtr.1186.1.S1_s_at	3.98	3.07	2.96	3.01	2.96	2.90	3.34	0.56	2.95	0.06	0.89	-0.175	3.1E-01
Mtr.44267.1.S1_at	9.13	8.43	9.54	9.16	9.08	8.96	9.03	0.56	9.07	0.10	1.00	0.005	9.2E-01
Mtr.37819.1.S1_at	9.10	8.26	9.31	8.79	9.09	8.98	8.89	0.56	8.95	0.16	1.01	0.011	8.6E-01
Mtr.2501.1.S1_at	9.30	8.22	8.53	9.18	9.03	9.23	8.68	0.56	9.15	0.11	1.05	0.075	2.3E-01
Mtr.22992.1.S1_at	3.39	3.04	4.13	3.13	3.38	3.41	3.52	0.56	3.31	0.15	0.94	-0.091	5.6E-01
Mtr.39336.1.S1_at	8.85	8.04	9.12	8.87	8.87	8.97	8.67	0.56	8.90	0.05	1.03	0.038	5.1E-01
Mtr.27185.1.S1_at	3.84	2.86	3.82	4.06	3.79	3.80	3.50	0.56	3.89	0.15	1.11	0.150	3.2E-01
Mtr.40339.1.S1_s_at	10.70	10.47	11.54	11.14	11.44	11.06	10.90	0.56	11.21	0.20	1.03	0.040	4.2E-01
Mtr.32881.1.S1_at	5.42	4.31	4.96	5.21	5.66	5.20	4.90	0.56	5.35	0.26	1.09	0.129	2.7E-01
Mtr.16730.1.S1_at	11.15	11.90	10.81	10.18	11.39	11.03	11.29	0.56	10.87	0.62	0.96	-0.055	4.3E-01
Mtr.7266.1.S1_at	8.58	7.52	7.74	8.67	8.54	8.62	7.95	0.56	8.61	0.07	1.08	0.116	1.1E-01
Mtr.39794.1.S1_at	10.52	9.50	9.63	9.16	8.73	8.79	9.88	0.56	8.89	0.24	0.90	-0.152	4.7E-02
Mtr.3146.1.S1_a_at	3.71	2.59	3.18	3.17	4.03	3.44	3.16	0.56	3.55	0.44	1.12	0.167	4.0E-01
Mtr.52248.1.S1_at	6.55	6.01	7.13	6.45	6.02	5.96	6.57	0.56	6.14	0.26	0.94	-0.096	3.0E-01
Mtr.9305.1.S1_at	8.32	7.94	9.05	8.63	8.72	8.43	8.44	0.56	8.59	0.15	1.02	0.026	6.7E-01
Mtr.29554.1.S1_s_at	10.06	8.95	9.61	9.65	9.65	9.75	9.54	0.56	9.68	0.06	1.02	0.022	6.8E-01
Mtr.5182.1.S1_at	8.65	8.06	9.18	8.68	8.29	8.53	8.63	0.56	8.50	0.20	0.98	-0.022	7.2E-01
Mtr.2521.1.S1_at	5.72	4.60	5.14	5.30	5.05	5.50	5.15	0.56	5.28	0.23	1.03	0.036	7.3E-01
Mtr.40352.1.S1_at	13.88	12.77	13.24	13.38	13.25	13.32	13.30	0.56	13.31	0.06	1.00	0.002	9.6E-01
Mtr.33887.1.S1_at	11.39	10.46	11.46	11.62	11.42	11.42	11.10	0.56	11.49	0.12	1.03	0.049	3.1E-01
Mtr.49355.1.S1_x_at	7.47	6.41	7.27	7.31	7.20	7.17	7.05	0.56	7.23	0.07	1.02	0.036	6.2E-01
Mtr.23092.1.S1_at	7.61	7.13	8.25	7.93	7.61	7.63	7.66	0.56	7.72	0.18	1.01	0.012	8.6E-01
Mtr.29453.1.S1_at	9.00	7.88	8.43	8.45	8.52	8.69	8.44	0.56	8.55	0.12	1.01	0.019	7.5E-01
Mtr.46558.1.S1_at	2.86	2.78	3.78	2.70	2.59	2.72	3.14	0.56	2.67	0.07	0.85	-0.232	2.2E-01
Mtr.33466.1.S1_at	5.34	4.25	4.99	4.83	5.04	5.26	4.86	0.56	5.04	0.22	1.04	0.053	6.3E-01
Mtr.21285.1.S1_at	9.40	9.61	10.45	11.78	12.27	11.99	9.82	0.56	12.01	0.25	1.22	0.291	3.4E-03
Mtr.9892.1.S1_s_at	9.26	8.15	8.61	8.95	8.93	9.14	8.68	0.56	9.01	0.12	1.04	0.054	3.7E-01
Mtr.32981.1.S1_at	6.10	5.00	5.70	6.23	6.11	6.34	5.60	0.56	6.23	0.11	1.11	0.154	1.3E-01
Mtr.34561.1.S1_x_at	2.87	3.82	2.84	2.82	2.77	2.87	3.18	0.56	2.82	0.05	0.89	-0.173	3.3E-01
Mtr.9637.1.S1_at	8.48	7.50	8.46	7.90	8.68	8.28	8.15	0.56	8.29	0.39	1.02	0.025	7.4E-01
Mtr.10904.1.S1_at	10.44	9.33	9.96	10.45	10.57	10.40	9.91	0.56	10.47	0.09	1.06	0.080	1.6E-01
Mtr.44693.1.S1_at	8.15	7.07	7.83	7.81	7.48	7.83	7.68	0.56	7.71	0.19	1.00	0.004	9.5E-01
Mtr.4968.1.S1_at	7.48	6.47	7.39	7.64	7.41	7.14	7.11	0.56	7.40	0.25	1.04	0.057	4.6E-01
Mtr.39688.1.S1_s_at	6.09	5.51	6.63	6.04	6.17	6.34	6.07	0.56	6.18	0.15	1.02	0.025	7.6E-01
Mtr.44432.1.S1_s_at	5.78	4.95	6.00	6.24	6.01	6.78	5.58	0.56	6.34	0.39	1.14	0.186	1.2E-01
Mtr.43656.1.S1_at	5.88	6.31	6.98	6.88	7.50	7.24	6.39	0.56	7.20	0.31	1.13	0.173	9.2E-02
Mtr.5083.1.S1_at	7.08	6.14	7.13	7.20	7.35	7.32	6.78	0.56	7.29	0.08	1.07	0.104	1.9E-01
Mtr.9749.1.S1_at	9.00	7.93	8.73	8.72	8.82	8.93	8.55	0.56	8.82	0.11	1.03	0.045	4.6E-01
Mtr.27566.1.S1_at	9.63	8.57	8.78	8.97	8.69	8.96	8.99	0.56	8.87	0.16	0.99	-0.019	7.4E-01
Mtr.46832.1.S1_at	3.24	2.29	2.25	2.21	2.25	2.25	2.60	0.56	2.24	0.02	0.86	-0.215	3.3E-01
Mtr.39034.1.S1_at	10.56	9.45	10.10	10.35	10.37	10.36	10.04	0.56	10.36	0.01	1.03	0.046	3.7E-01
Mtr.2239.1.S1_at	7.45	8.06	8.56	9.47	9.90	9.50	8.02	0.56	9.62	0.24	1.20	0.263	1.0E-02
Mtr.20687.1.S1_at	4.16	4.22	5.15	4.32	4.26	4.00	4.51	0.56	4.19	0.17	0.93	-0.106	4.0E-01
Mtr.42021.1.S1_s_at	10.47	9.36	10.01	10.19	10.25	10.61	9.94	0.56	10.35	0.23	1.04	0.057	3.1E-01
Mtr.24766.1.S1_at	9.16	8.09	8.88	9.11	9.01	8.90	8.71	0.56	9.01	0.10	1.03	0.049	4.1E-01
Mtr.8314.1.S1_at	10.96	10.22	11.31	10.75	10.82	10.73	10.83	0.56	10.77	0.04	0.99	-0.008	8.6E-01
Mtr.41306.1.S1_s_at	9.31	8.73	9.85	9.52	9.16	9.44	9.30	0.56	9.37	0.19	1.01	0.011	8.4E-01
Mtr.3833.1.S1_s_at	4.30	3.32	3.34	3.31	3.30	4.29	3.65	0.56	3.63	0.57	0.99	-0.009	9.6E-01
Mtr.42673.1.S1_at	7.26	6.22	7.09	7.11	7.21	7.33	6.85	0.56	7.22	0.11	1.05	0.075	3.3E-01
Mtr.20641.1.S1_s_at	4.35	3.23	3.78	3.98	4.00	4.15	3.79	0.56	4.05	0.09	1.07	0.095	4.7E-01
Mtr.18951.1.S1_s_at	7.57	6.45	6.95	6.50	6.31	6.40	6.99	0.56	6.40	0.10	0.92	-0.127	1.5E-01
Mtr.14132.1.S1_at	5.77	4.77	5.69	4.60	4.73	4.72	5.41	0.56	4.68	0.07	0.87	-0.209	8.8E-02
Mtr.31792.1.S1_at	9.84	8.82	9.71	9.61	9.50	9.60	9.45	0.56	9.57	0.06	1.01	0.018	7.4E-01
Mtr.1800.1.S1_at	4.26	3.35	3.25	3.18	3.16	3.31	3.62	0.56	3.22	0.08	0.89	-0.171	2.8E-01
Mtr.39894.1.S1_at	9.84	8.76	9.54	9.51	9.32	9.69	9.38	0.56	9.51	0.18	1.01	0.019	7.3E-01
Mtr.37901.1.S1_at	11.76	10.65	11.27	11.42	11.59	11.49	11.23	0.56	11.50	0.08	1.02	0.035	4.4E-01
Mtr.13367.1.S1_at	8.09	7.20	8.22	8.47	8.26	8.30	7.84	0.56	8.34	0.11	1.06	0.090	2.0E-01
Mtr.51170.1.S1_at	2.80	3.76	2.79	2.77	2.72	2.75	3.12	0.56	2.75	0.02	0.88	-0.182	3.1E-01
Mtr.16870.1.S1_at	2.88	3.84	2.87	2.86	2.86	2.87	3.20	0.56	2.86	0.00	0.90	-0.159	3.6E-01
Mtr.25095.1.S1_at	10.10	9.31	10.39	10.05	10.08	10.11	9.94	0.56	10.08	0.03	1.01	0.021	6.7E-01
Mtr.9356.1.S1_at	8.90	8.57	9.65	9.22	9.45	9.19	9.04	0.56	9.29	0.14	1.03	0.039	5.0E-01
Mtr.23346.1.S1_at	7.28	6.21	7.02	7.06	7.39	6.86	6.84	0.56	7.10	0.27	1.04	0.055	5.0E-01
Mtr.43929.1.S1_at	9.54	8.56	9.50	9.34	9.35	9.28	9.20	0.56	9.32	0.04	1.01	0.019	7.2E-01
Mtr.6834.1.S1_at	8.95	8.21	9.30	8.89	8.71	9.03	8.82	0.56	8.88	0.16	1.01	0.009	8.7E-01
Mtr.17620.1.S1_at	2.47	2.05	3.16	2.56	4.11	2.71	2.56	0.56	3.13	0.85	1.22	0.289	3.9E-01
Mtr.43691.1.S1_at	9.09	8.27	9.33	8.87	8.74	8.91	8.90	0.56	8.84	0.09	0.99	-0.009	8.7E-01
Mtr.8750.1.S1_at	12.72	11.67	12.51	12.72	12.74	12.65	12.30	0.56	12.70	0.05	1.03	0.047	2.8E-01
Mtr.12983.1.S1_at	12.82	11.87	11.85	11.95	11.68	12.00	12.18	0.56	11.88	0.17	0.97	-0.037	4.2E-01
Mtr.18268.1.S1_at	9.84	8.73	9.26	9.61	10.05	9.76	9.28	0.56	9.80	0.22	1.06	0.080	2.0E-01
Mtr.49487.1.S1_at	12.37	11.95	13.05	12.59	12.77	12.56	12.46	0.56	12.64	0.11	1.01	0.021	6.1E-01
Mtr.13396.1.S1_at	10.04	8.93	9.36	9.28	9.09	9.17	9.44	0.56	9.18	0.10	0.97	-0.041	4.6E-01
Mtr.33859.1.S1_at	7.13	6.52	7.63	6.84	7.26	7.21	7.09	0.56	7.10	0.23	1.00	0.003	9.7E-01
Mtr.43821.1.S1_at	10.50	9.49	10.40	10.52	10.41	10.50	10.13	0.56	10.47	0.06	1.03	0.048	3.5E-01
Mtr.45249.1.S1_at	8.18	7.18	8.10	8.17	8.17	8.01	7.82	0.56	8.12	0.09	1.04	0.053	4.2E-01
Mtr.44874.1.S1_at	8.07	7.02	7.86	7.55	7.26	7.73	7.65	0.56	7.51	0.24	0.98	-0.026	7.1E-01
Mtr.9488.1.S1_s_at	7.24	6.31	7.30	7.26	7.55	7.27	6.95	0.56	7.36	0.17	1.06	0.082	2.9E-01
Mtr.37876.1.S1_at	9.73	9.16	8.62	8.50	8.29	8.68	9.17	0.56	8.49	0.19	0.93	-0.111	1.2E-01
Mtr.5799.1.S1_s_at	3.40	2.29	2.82	2.82	3.36	2.83	2.84	0.56	3.00	0.31	1.06	0.080	6.8E-01
Mtr.29637.1.S1_at	9.66	8.63	9.50	9.22	9.57	9.40	9.26	0.56	9.40	0.17	1.01	0.021	7.1E-01
Mtr.42072.1.S1_at	9.73	10.21	9.11	9.88	9.70	9.69	9.68	0.56	9.75	0.11	1.01	0.011	8.4E-01
Mtr.11685.1.S1_s_at	5.74	4.70	5.56	5.60	5.84	5.08	5.33	0.56	5.51	0.39	1.03	0.048	6.7E-01
Mtr.43957.1.S1_s_at	7.60	6.65	7.62	7.91	7.73	8.05	7.29	0.56	7.89	0.16	1.08	0.115	1.5E-01
Mtr.19966.1.S1_x_at	6.51	5.74	6.82	6.91	6.76	7.02	6.36	0.56	6.90	0.13	1.08	0.118	1.8E-01
Mtr.35605.1.S1_at	11.34	10.42	11.42	11.74	12.01	11.75	11.06	0.56	11.83	0.15	1.07	0.098	8.0E-02
Mtr.10596.1.S1_at	12.06	11.06	11.14	11.86	11.82	12.07	11.42	0.56	11.92	0.13	1.04	0.061	2.1E-01
Mtr.2177.1.S1_at	8.83	7.72	8.34	8.85	8.70	8.92	8.29	0.56	8.82	0.11	1.06	0.089	1.8E-01
Mtr.22805.1.S1_at	7.40	6.87	7.98	7.41	7.30	7.32	7.42	0.56	7.34	0.06	0.99	-0.014	8.4E-01
Mtr.32064.1.S1_s_at	8.81	7.96	9.00	8.96	9.18	9.16	8.59	0.56	9.10	0.12	1.06	0.083	1.9E-01
Mtr.35280.1.S1_at	5.46	6.22	5.14	6.88	6.05	6.65	5.61	0.56	6.53	0.43	1.16	0.219	8.6E-02

Mtr.8463.1.S1_at	3.50	2.95	4.06	4.28	6.85	5.88	3.50	0.56	5.67	1.30	1.62	0.696	5.6E-02
Mtr.37046.1.S1_at	6.85	5.78	6.56	7.29	7.14	7.19	6.40	0.56	7.20	0.08	1.13	0.171	6.7E-02
Mtr.4372.1.S1_at	7.60	8.51	7.51	8.32	8.85	8.39	7.87	0.56	8.52	0.29	1.08	0.114	1.5E-01
Mtr.1972.1.S1_at	7.50	6.87	7.98	7.50	7.99	7.59	7.45	0.56	7.69	0.26	1.03	0.047	5.3E-01
Mtr.50494.1.S1_s_at	10.18	9.12	9.36	9.89	9.97	10.05	9.56	0.56	9.97	0.08	1.04	0.061	2.7E-01
Mtr.10971.1.S1_s_at	8.39	7.28	7.82	7.85	7.62	7.97	7.83	0.56	7.81	0.18	1.00	-0.004	9.6E-01
Mtr.35137.1.S1_at	8.73	7.64	8.35	8.72	8.67	8.77	8.24	0.56	8.72	0.05	1.06	0.082	2.1E-01
Mtr.12456.1.S1_at	11.92	10.97	11.93	12.16	12.23	12.24	11.61	0.56	12.21	0.05	1.05	0.073	1.4E-01
Mtr.15120.1.S1_at	7.44	7.05	8.15	7.88	7.50	7.27	7.55	0.56	7.55	0.31	1.00	0.001	9.9E-01
Mtr.51907.1.S1_at	9.09	8.02	8.80	8.12	8.60	8.54	8.63	0.56	8.42	0.26	0.98	-0.036	5.8E-01
Mtr.30198.1.S1_at	8.98	9.79	8.73	8.77	9.26	9.01	9.17	0.56	9.01	0.25	0.98	-0.024	6.8E-01
Mtr.4249.1.S1_at	8.20	7.20	8.13	7.87	8.23	7.85	7.84	0.56	7.99	0.21	1.02	0.026	7.0E-01
Mtr.46900.1.S1_at	11.30	10.39	11.41	10.80	10.50	10.92	11.03	0.56	10.74	0.21	0.97	-0.039	4.4E-01
Mtr.34111.1.S1_at	7.63	7.13	8.24	7.62	7.74	7.46	7.67	0.56	7.61	0.14	0.99	-0.012	8.6E-01
Mtr.12227.1.S1_at	8.47	7.82	7.37	8.00	7.76	7.61	7.89	0.55	7.79	0.20	0.99	-0.018	7.9E-01
Mtr.9982.1.S1_at	7.16	6.12	7.00	7.13	7.07	7.36	6.76	0.55	7.19	0.15	1.06	0.089	2.6E-01
Mtr.40799.1.S1_at	13.82	12.98	12.76	12.81	13.10	13.07	13.19	0.55	12.99	0.16	0.99	-0.021	5.9E-01
Mtr.42119.1.S1_at	8.42	7.56	8.59	8.18	8.40	8.25	8.19	0.55	8.28	0.11	1.01	0.016	8.0E-01
Mtr.18210.1.S1_at	6.53	5.62	6.62	6.75	6.52	6.52	6.26	0.55	6.60	0.13	1.05	0.077	3.6E-01
Mtr.17069.1.S1_at	2.98	4.09	3.59	4.41	5.52	4.05	3.55	0.55	4.66	0.77	1.31	0.391	1.1E-01
Mtr.9313.1.S1_at	9.20	8.10	8.50	9.46	10.19	9.74	8.60	0.55	9.80	0.37	1.14	0.188	3.6E-02
Mtr.2002.1.S1_at	2.78	2.30	3.41	2.92	2.92	2.92	2.83	0.55	2.92	0.00	1.03	0.046	7.9E-01
Mtr.41846.1.S1_s_at	8.39	7.75	8.85	8.63	8.62	8.51	8.33	0.55	8.59	0.06	1.03	0.043	4.8E-01
Mtr.20657.1.S1_at	11.08	11.44	12.17	11.45	11.37	11.54	11.56	0.55	11.45	0.09	0.99	-0.014	7.5E-01
Mtr.12022.1.S1_at	9.88	9.53	10.61	10.18	10.09	10.01	10.01	0.55	10.09	0.09	1.01	0.013	8.0E-01
Mtr.51134.1.S1_at	2.21	3.17	2.20	2.19	2.20	2.19	2.53	0.55	2.19	0.00	0.87	-0.204	3.6E-01
Mtr.34714.1.S1_at	9.65	8.54	9.03	9.21	9.01	8.94	9.07	0.55	9.05	0.14	1.00	-0.003	9.5E-01
Mtr.32937.1.S1_at	5.05	4.12	5.11	5.64	5.38	4.95	4.76	0.55	5.33	0.35	1.12	0.161	2.1E-01
Mtr.25525.1.S1_at	6.49	5.44	5.66	5.69	6.60	6.14	5.87	0.55	6.14	0.46	1.05	0.067	5.4E-01
Mtr.14229.1.S1_at	8.71	7.64	8.45	8.70	8.94	8.85	8.26	0.55	8.83	0.12	1.07	0.095	1.6E-01
Mtr.49300.1.S1_at	5.18	4.88	5.95	7.99	8.37	8.06	5.34	0.55	8.14	0.20	1.52	0.608	1.2E-03
Mtr.3140.1.S1_at	6.77	7.51	7.86	7.90	8.18	7.86	7.38	0.55	7.98	0.18	1.08	0.113	1.5E-01
Mtr.43562.1.S1_at	9.69	8.62	9.39	9.48	9.46	9.41	9.24	0.55	9.45	0.03	1.02	0.033	5.4E-01
Mtr.10182.1.S1_at	4.18	3.21	4.16	4.39	4.72	4.72	3.85	0.55	4.61	0.19	1.20	0.262	8.6E-02
Mtr.38169.1.S1_at	10.23	9.12	9.58	9.54	9.66	9.57	9.64	0.55	9.59	0.06	0.99	-0.008	8.8E-01
Mtr.45349.1.S1_at	8.58	8.50	9.50	8.82	8.84	8.99	8.86	0.55	8.89	0.09	1.00	0.004	9.4E-01
Mtr.42355.1.S1_at	7.46	6.51	7.49	7.85	7.57	7.76	7.15	0.55	7.72	0.14	1.08	0.111	1.6E-01
Mtr.18228.1.S1_at	7.21	6.17	6.36	6.66	6.10	6.44	6.58	0.55	6.40	0.28	0.97	-0.039	6.5E-01
Mtr.21341.1.S1_at	8.52	8.28	9.34	8.71	8.55	8.46	8.71	0.55	8.57	0.13	0.98	-0.023	6.9E-01
Mtr.44686.1.S1_at	9.62	8.99	10.09	9.68	9.86	9.77	9.57	0.55	9.77	0.09	1.02	0.030	5.6E-01
Mtr.6175.1.S1_at	9.50	8.40	8.88	9.54	9.38	9.64	8.93	0.55	9.52	0.13	1.07	0.093	1.5E-01
Mtr.49945.1.S1_at	3.46	4.08	4.56	3.89	3.88	3.84	4.03	0.55	3.87	0.03	0.96	-0.060	6.3E-01
Mtr.7153.1.S1_at	10.20	9.12	9.86	9.83	9.83	9.93	9.73	0.55	9.86	0.06	1.01	0.020	6.9E-01
Mtr.35543.1.S1_at	7.09	6.55	7.66	6.86	7.21	7.00	7.10	0.55	7.03	0.18	0.99	-0.015	8.4E-01
Mtr.38295.1.S1_at	8.29	7.25	8.11	8.12	8.15	8.42	7.88	0.55	8.23	0.16	1.04	0.062	3.6E-01
Mtr.1096.1.S1_at	4.16	4.19	5.13	4.75	4.69	4.26	4.49	0.55	4.57	0.27	1.02	0.024	8.4E-01
Mtr.15318.1.S1_at	8.10	7.13	8.07	8.04	8.14	7.98	7.76	0.55	8.05	0.08	1.04	0.053	4.2E-01
Mtr.13753.1.S1_at	7.19	8.21	7.34	7.86	7.71	7.50	7.58	0.55	7.69	0.18	1.01	0.021	7.6E-01
Mtr.13199.1.S1_at	8.53	7.47	8.26	8.47	8.54	8.45	8.08	0.55	8.49	0.04	1.05	0.070	2.8E-01
Mtr.9583.1.S1_at	7.86	6.84	7.71	7.26	7.32	7.86	7.47	0.55	7.48	0.33	1.00	0.002	9.8E-01
Mtr.34018.1.S1_at	10.73	9.74	9.81	10.31	9.91	10.35	10.09	0.55	10.19	0.24	1.01	0.014	7.9E-01
Mtr.7393.1.S1_at	8.84	7.74	8.41	8.67	8.58	8.60	8.33	0.55	8.62	0.05	1.03	0.048	4.2E-01
Mtr.40347.1.S1_at	6.60	6.13	7.23	6.61	6.61	6.86	6.66	0.55	6.69	0.14	1.01	0.008	9.1E-01
Mtr.23923.1.S1_at	2.72	3.65	2.67	2.68	2.64	2.62	3.01	0.55	2.64	0.03	0.88	-0.187	3.2E-01
Mtr.14564.1.S1_s_at	2.38	3.34	2.39	2.36	2.36	2.36	2.71	0.55	2.36	0.00	0.87	-0.197	3.4E-01
Mtr.10725.1.S1_at	9.19	10.25	9.44	9.34	10.18	9.67	9.63	0.55	9.73	0.42	1.01	0.015	8.1E-01
Mtr.26419.1.S1_at	7.89	6.79	7.40	7.47	7.47	7.75	7.36	0.55	7.56	0.16	1.03	0.039	5.8E-01
Mtr.50019.1.S1_at	5.46	4.60	4.43	5.22	5.84	5.73	4.83	0.55	5.60	0.33	1.16	0.214	1.1E-01
Mtr.36186.1.S1_at	10.98	9.92	10.70	10.58	10.65	10.61	10.53	0.55	10.61	0.04	1.01	0.011	8.1E-01
Mtr.4080.1.S1_s_at	7.24	6.42	7.48	7.21	6.96	7.15	7.05	0.55	7.11	0.13	1.01	0.013	8.6E-01
Mtr.12050.1.S1_at	8.30	7.32	8.25	8.37	8.24	8.26	7.96	0.55	8.29	0.07	1.04	0.059	3.6E-01
Mtr.4993.1.S1_at	8.67	7.99	9.08	8.75	8.94	8.66	8.58	0.55	8.78	0.14	1.02	0.034	5.7E-01
Mtr.41591.1.S1_at	8.38	7.54	8.58	8.15	8.19	8.19	8.17	0.55	8.18	0.03	1.00	0.002	9.7E-01
Mtr.4067.1.S1_at	3.75	2.87	3.88	4.02	3.68	4.60	3.50	0.55	4.10	0.46	1.17	0.228	2.2E-01
Mtr.21239.1.S1_at	6.79	6.27	5.69	7.44	8.22	7.48	6.25	0.55	7.71	0.44	1.23	0.303	2.3E-02
Mtr.16661.1.S1_at	2.36	3.39	2.55	2.07	2.13	2.12	2.77	0.55	2.11	0.03	0.76	-0.393	1.1E-01
Mtr.38990.1.S1_at	8.51	7.60	8.60	8.58	8.78	8.70	8.23	0.55	8.69	0.10	1.05	0.077	2.3E-01
Mtr.41712.1.S1_at	10.17	9.09	9.44	9.63	9.45	9.74	9.56	0.55	9.61	0.15	1.00	0.007	9.0E-01
Mtr.39937.1.S1_at	4.71	3.89	4.94	4.61	4.59	4.61	4.51	0.55	4.60	0.01	1.02	0.028	7.9E-01
Mtr.3514.1.S1_at	2.52	2.64	3.53	2.50	2.49	2.47	2.90	0.55	2.49	0.01	0.86	-0.222	2.6E-01
Mtr.3183.1.S1_at	8.01	7.27	8.35	8.40	8.24	8.11	7.87	0.55	8.25	0.15	1.05	0.067	3.2E-01
Mtr.14876.1.S1_s_at	10.18	9.11	9.86	10.09	9.84	9.85	9.71	0.55	9.93	0.14	1.02	0.031	5.5E-01
Mtr.20639.1.S1_at	2.69	3.62	2.64	2.64	2.64	2.65	2.99	0.55	2.64	0.01	0.88	-0.178	3.4E-01
Mtr.29608.1.S1_at	5.10	4.01	4.67	5.10	5.09	5.50	4.59	0.55	5.23	0.23	1.14	0.186	1.4E-01
Mtr.5401.1.S1_s_at	8.68	7.77	8.76	8.32	8.57	8.48	8.40	0.55	8.46	0.13	1.01	0.009	8.7E-01
Mtr.49603.1.S1_at	10.61	9.51	10.00	10.17	10.13	10.21	10.04	0.55	10.17	0.04	1.01	0.019	7.0E-01
Mtr.34766.1.S1_at	7.70	6.66	7.49	7.49	7.49	7.49	7.28	0.55	7.49	0.00	1.03	0.040	5.5E-01
Mtr.42037.1.S1_at	10.67	9.68	10.59	10.86	10.65	10.73	10.32	0.55	10.75	0.10	1.04	0.059	2.5E-01
Mtr.45978.1.S1_x_at	2.87	3.49	2.39	2.43	2.38	2.37	2.91	0.55	2.40	0.03	0.82	-0.283	1.8E-01
Mtr.5952.1.S1_at	6.67	5.84	6.89	6.77	6.73	6.73	6.47	0.55	6.74	0.02	1.04	0.060	4.3E-01
Mtr.26086.1.S1_at	3.19	4.13	3.15	3.14	3.14	3.12	3.49	0.55	3.14	0.01	0.90	-0.154	3.3E-01
Mtr.31778.1.S1_at	8.50	7.89	8.99	8.67	8.61	8.34	8.46	0.55	8.54	0.18	1.01	0.013	8.3E-01
Mtr.47027.1.S1_at	10.31	9.25	10.02	10.34	10.43	10.51	9.86	0.55	10.43	0.09	1.06	0.081	1.5E-01
Mtr.13994.1.S1_at	9.50	8.40	8.88	9.26	9.12	9.37	8.93	0.55	9.25	0.13	1.04	0.051	3.8E-01
Mtr.46349.1.S1_at	2.33	3.25	2.26	2.31	2.31	2.31	2.61	0.55	2.31	0.00	0.88	-0.178	3.9E-01
Mtr.1142.1.S1_at	5.59	4.61	5.53	5.66	6.56	5.58	5.24	0.55	5.94	0.55	1.13	0.179	2.0E-01
Mtr.44158.1.S1_at	8.44	7.38	8.19	8.09	8.18	8.05	8.00	0.55	8.11	0.07	1.01	0.018	7.7E-01
Mtr.9743.1.S1_at	8.92	7.94	8.87	8.50	8.63	8.39	8.58	0.55	8.50	0.12	0.99	-0.012	8.3E-01
Mtr.5486.1.S1_s_at	10.69	9.59	10.14	9.90	9.34	9.92	10.14	0.55	9.72	0.33	0.96	-0.060	3.2E-01
Mtr.15439.1.S1_at	5.32	6.05	4.98	5.01	4.82	5.00	5.45	0.55	4.94	0.11	0.91	-0.140	1.9E-01
Mtr.25907.1.S1_at	3.59	4.28	3.20	3.44	3.12	3.38	3.69	0.55	3.31	0.17	0.90	-0.155	3.2E-01
Mtr.5901.1.S1_at	6.56	6.45	7.45	6.83	7.55	6.67	6.82	0.55	7.02	0.47	1.03	0.041	6.6E-01
Mtr.48872.1.S1_at	8.07	8.16	9.06	8.05	8.35	8.07	8.43	0.55	8.16	0.17	0.97	-0.047	4.6E-01
Mtr.8804.1.S1_at	9.54	8.73	9.77	9.60	9.41	9.42	9.35	0.55	9.47	0.11	1.01	0.020	7.1E-01
Mtr.21620.1.S1_at	8.82	7.82	8.72	8.61	8.63	8.61	8.45	0.55	8.61	0.02	1.02	0.027	6.4E-01
Mtr.16099.1.S1_at	5.91	5.26	6.35	5.53	5.50	4.59	5.84	0.55	5.21	0.54	0.89	-0.166	2.3E-01
Mtr.15923.1.S1_at	7.15	6.56	7.66	7.27	6.72	6.77	7.13	0.55	6.92	0.30	0.97	-0.042	6.0E-01
Mtr.42289.1.S1_at	8.82	7.97	9.01	8.89	8.98	8.86	8.60	0.55	8.91	0.06	1.04	0.052	3.8E-01
Mtr.12371.1.S1_at	12.27	11.17	11.80	11.46	11.27	11.31	11.75	0.55	11.35	0.10	0.97	-0.050	2.9E-01
Mtr.10853.1.S1_at	12.42	11.75	11.33	11.52	11.42	11.61	11.83	0.55	11.52	0.10	0.97	-0.039	3.8E-01
Mtr.44871.1.S1_at	7.48	6.90	7.99	7.64	7.64	7.77	7.46	0.55	7.68	0.07	1.03	0.043	5.2E-01
Mtr.29826.1.S1_at	7.97	8.14	9.00	7.87	8.08	8.06	8.37	0.55	8.00	0.11	0.96	-0.064	3.2E-01
Mtr.29855.1.S1_at	2.70	2.74	3.67	2.68	3.10	2.79	3.04	0.55	2.86	0.21	0.94	-0.089	6.2E-01
Mtr.36453.1.S1_at	6.64	5.65	6.56	6.85	7.24	6.62	6.29	0.55	6.90	0.31	1.10	0.135	1.7E-01
Mtr.40569.1.S1_at	9.72	9.26	10.35	10.12	10.73	10.27	9.78	0.55	10.37	0.32	1.06	0.086	1.8E-01
Mtr.8246.1.S1_at	2.23	3.23	2.32	2.27	2.27	2.27	2.59	0.55	2.27	0.00	0.88	-0.191	3.7E-01
Mtr.30170.1.S1_at	9.08	8.20	9.21	8.94	9.12	8.82	8.83	0.55	8.96	0.15	1.01	0.021	7.1E-01
Mtr.44904.1.S1_at	6.49	5.65	6.68	6.48	6.61	6.36	6.27	0.55	6.49	0.13	1.03	0.049	5.4E-01
Mtr.9061.1.S1_at	9.30	8.64	9.73	9.53	9.58	9.40	9.22	0.55	9.50	0.09	1.03	0.043	4.3E-01
Mtr.43843.1.S1_at	9.08	8.14	9.09	9.03	9.14	8.91	8.77	0.55	9.03	0.11	1.03	0.042	4.7E-01
Mtr.18767.1.S1_at	8.50	7.40	7.88	8.07	8.16	7.89	7.93	0.55	8.04	0.14	1.01	0.020	7.5E-01
Mtr.5310.1.S1_s_at	11.84	10.80	11.02	11.31	10.80	11.22	11.22	0.55	11.11	0.27	0.99	-0.014	7.7E-01
Mtr.12218.1.S1_at	10.80	10.89	11.79	11.03	10.87	10.71	11.16	0.55	10.87	0.16	0.97	-0.038	4.2E-01
Mtr.18268.1.S1_s_at	10.80	9.70	10.27	10.66	10.99	10.86	10.26	0.55	10.84	0.17	1.06	0.080	1.5E-01
Mtr.38051.1.S1_at	5.89	6.49	6.98	6.69	7.18	6.69	6.45	0.55	6.86	0.28	1.06	0.087	3.2E-01
Mtr.33901.1.S1_at	5.63	4.56	4.91	5.43	5.56	4.94	5.03	0.55	5.31	0.33	1.05	0.077	5.0E-01
Mtr.27980.1.S1_at	4.76	4.26	5.35	4.89	6.26	5.28	4.79	0.55	5.48	0.71	1.14	0.193	2.6E-01
Mtr.9522.1.S1_at	10.40	9.30	9.83	9.87	9.97	10.11	9.84	0.55	9.99	0.12	1.01	0.021	6.8E-01
Mtr.36040.1.S1_at	9.62	8.90	9.97	9.52	9.70	9.61	9.49	0.55	9.61	0.09	1.01	0.017	7.4E-01
Mtr.39370.1.S1_at	5.52	5.44	6.43	5.61	5.31	5.50	5.79	0.55	5.48	0.15	0.94	-0.082	3.9E-01
Mtr.27379.1.S1_at	7.55	8.21	8.64	7.96	8.36	7.63	8.13	0.55	7.98	0.36	0.98	-0.026	7.2E-01
Mtr.32884.1.S1_s_at	4.47	3.39	4.08	3.81	3.23	3.84	3.98	0.55	3.63	0.34	0.91	-0.132	4.1E-01
Mtr.33417.1.S1_at	8.11	7.12	8.02	7.87	7.58	7.97	7.75	0.55	7.81	0.20	1.01	0.010	8.8E-01
Mtr.21087.1.S1_at	3.35	2.66	2.27	2.52	2.51	2.52	2.76	0.55	2.52	0.01	0.91	-0.133	4.9E-01
Mtr.35547.1.S1_at	7.87	8.04	8.90	8.57	8.93	8.47	8.27	0.55	8.66	0.24	1.05	0.066	3.3E-01
Mtr.35072.1.S1_s_at	7.59	6.50	7.12	7.29	7.18	7.41	7.07	0.55	7.29	0.11	1.03	0.045	5.3E-01
Mtr.25753.1.S1_at	6.53	7.01	5.91	7.09	7.38	6.92	6.48	0.55	7.13	0.23	1.10	0.137	1.3E-01
Mtr.32093.1.S1_at	7.10	6.08	6.92	7.02	6.92	6.68	6.70	0.55	6.87	0.18	1.03	0.037	6.3E-01
Mtr.42564.1.S1_at	5.22	4.62	5.71	5.32	5.43	5.20	5.18	0.55	5.32	0.11	1.03	0.036	7.1E-01
Mtr.16340.1.S1_at	7.45	6.90	7.99	7.51	7.63	7.14	7.45	0.55	7.43	0.26	1.00	-0.004	9.5E-01
Mtr.20339.1.S1_s_at	3.10	3.39	4.16	3.11	2.72	2.91	3.55	0.55	2.91	0.19	0.82	-0.286	1.3E-01

Mtr.6652.1.S1_at	4.04	3.06	3.11	3.16	3.13	3.24	3.40	0.55	3.18	0.06	0.93	-0.099	5.2E-01
Mtr.15739.1.S1_at	8.71	8.06	9.15	8.62	8.76	8.78	8.64	0.55	8.72	0.09	1.01	0.013	8.2E-01
Mtr.37198.1.S1_at	9.53	8.46	8.79	8.67	8.41	8.46	8.93	0.55	8.51	0.14	0.95	-0.069	2.7E-01
Mtr.41600.1.S1_at	9.75	8.86	9.86	9.75	9.84	9.66	9.49	0.55	9.75	0.09	1.03	0.039	4.7E-01
Mtr.43342.1.S1_at	6.84	5.76	6.43	7.01	6.94	6.88	6.34	0.55	6.94	0.06	1.09	0.130	1.3E-01
Mtr.43577.1.S1_s_at	9.81	8.73	9.13	9.24	9.30	9.35	9.23	0.55	9.30	0.05	1.01	0.011	8.3E-01
Mtr.45277.1.S1_at	8.36	7.37	8.25	8.32	8.28	8.35	7.99	0.55	8.32	0.04	1.04	0.057	3.7E-01
Mtr.38870.1.S1_at	10.48	10.74	11.53	11.28	11.11	11.16	10.92	0.55	11.18	0.09	1.02	0.035	4.5E-01
Mtr.41535.1.S1_at	8.42	7.53	8.52	8.52	8.49	8.63	8.16	0.55	8.54	0.07	1.05	0.067	2.9E-01
Mtr.38233.1.S1_at	7.72	8.24	8.81	8.48	8.79	8.14	8.26	0.55	8.47	0.32	1.03	0.037	6.0E-01
Mtr.7600.1.S1_at	6.37	5.66	6.73	6.26	6.00	6.27	6.25	0.55	6.18	0.16	0.99	-0.018	8.2E-01
Mtr.46760.1.S1_at	3.54	4.46	3.48	3.50	3.52	3.51	3.83	0.55	3.51	0.01	0.92	-0.126	3.7E-01
Mtr.42720.1.S1_at	9.17	8.08	8.54	8.80	8.87	8.84	8.60	0.55	8.84	0.03	1.03	0.040	4.9E-01
Mtr.17506.1.S1_at	11.05	9.96	10.42	10.56	10.50	10.69	10.48	0.55	10.58	0.10	1.01	0.014	7.6E-01
Mtr.45244.1.S1_at	8.51	9.46	8.52	9.56	9.04	9.05	8.83	0.55	9.22	0.30	1.04	0.061	3.4E-01
Mtr.9846.1.S1_at	8.75	7.68	8.40	8.31	8.50	8.45	8.28	0.55	8.42	0.10	1.02	0.025	6.8E-01
Mtr.6341.1.S1_at	2.45	3.39	2.44	2.47	2.40	2.39	2.76	0.55	2.42	0.04	0.88	-0.189	3.4E-01
Mtr.43666.1.S1_at	9.55	10.00	10.64	9.72	9.89	9.50	10.06	0.55	9.70	0.19	0.96	-0.053	3.4E-01
Mtr.9207.1.S1_at	8.97	7.96	8.84	8.77	8.63	8.63	8.59	0.55	8.68	0.08	1.01	0.015	8.0E-01
Mtr.10941.1.S1_at	10.78	9.84	9.82	10.41	10.51	10.75	10.15	0.55	10.56	0.18	1.04	0.057	2.8E-01
Mtr.10105.1.S1_s_at	9.55	8.47	8.91	8.93	8.87	9.07	8.98	0.55	8.96	0.10	1.00	-0.003	9.5E-01
Mtr.10705.1.S1_at	5.39	4.85	5.95	6.73	8.14	6.81	5.40	0.55	7.23	0.79	1.34	0.422	3.0E-02
Mtr.41047.1.S1_at	7.38	6.29	6.94	6.61	6.56	6.37	6.87	0.55	6.51	0.13	0.95	-0.076	3.4E-01
Mtr.28539.1.S1_at	3.91	2.84	3.57	3.68	3.56	3.55	3.44	0.55	3.59	0.07	1.05	0.065	6.4E-01
Mtr.34809.1.S1_s_at	10.19	9.15	9.38	9.25	9.27	9.61	9.57	0.55	9.38	0.21	0.98	-0.030	5.9E-01
Mtr.34614.1.S1_at	9.96	9.51	10.60	10.33	10.35	10.21	10.02	0.55	10.30	0.08	1.03	0.039	4.4E-01
Mtr.4721.1.S1_at	7.94	7.14	8.19	7.85	7.88	7.88	7.75	0.55	7.87	0.02	1.02	0.022	7.3E-01
Mtr.4995.1.S1_at	10.56	9.65	10.64	10.45	10.57	10.67	10.28	0.55	10.56	0.11	1.03	0.039	4.3E-01
Mtr.25816.1.S1_x_at	3.44	4.38	3.43	3.41	3.40	3.39	3.75	0.55	3.40	0.01	0.91	-0.140	3.3E-01
Mtr.51296.1.S1_at	9.77	8.69	9.06	9.31	9.40	9.41	9.17	0.55	9.37	0.05	1.02	0.031	5.7E-01
Mtr.1395.1.S1_at	7.36	6.94	8.02	7.74	7.75	7.35	7.44	0.55	7.61	0.23	1.02	0.033	6.4E-01
Mtr.14180.1.S1_at	2.73	3.67	2.72	2.70	2.86	2.71	3.04	0.55	2.76	0.09	0.91	-0.141	4.2E-01
Mtr.26973.1.S1_at	5.83	6.80	6.75	6.09	7.31	7.13	6.46	0.55	6.84	0.66	1.06	0.083	4.8E-01
Mtr.8435.1.S1_at	11.66	10.58	11.25	11.19	10.97	11.18	11.16	0.55	11.11	0.13	1.00	-0.006	8.9E-01
Mtr.18234.1.S1_at	3.39	4.31	3.33	3.34	3.27	3.26	3.68	0.55	3.29	0.05	0.89	-0.161	2.9E-01
Mtr.20466.1.S1_at	5.49	6.40	6.47	5.92	5.59	5.49	6.12	0.55	5.67	0.23	0.93	-0.111	2.5E-01
Mtr.41382.1.S1_at	9.24	8.15	8.77	8.93	9.19	9.04	8.72	0.55	9.05	0.13	1.04	0.054	3.6E-01
Mtr.43986.1.S1_at	9.04	8.25	9.30	9.12	9.19	9.15	8.86	0.55	9.15	0.04	1.03	0.046	4.1E-01
Mtr.43961.1.S1_at	12.06	11.04	11.87	11.93	12.01	12.06	11.66	0.55	12.00	0.06	1.03	0.042	3.4E-01
Mtr.12121.1.S1_at	7.30	6.39	7.37	6.71	7.52	6.84	7.02	0.55	7.02	0.44	1.00	0.000	1.0E+00
Mtr.35553.1.S1_at	5.00	5.38	6.07	5.13	5.51	5.15	5.48	0.55	5.26	0.21	0.96	-0.059	5.5E-01
Mtr.36635.1.S1_at	3.18	2.24	2.23	2.24	2.23	2.23	2.55	0.55	2.23	0.00	0.87	-0.194	3.7E-01
Mtr.5261.1.S1_at	9.11	8.53	9.62	9.46	9.56	9.42	9.09	0.55	9.48	0.07	1.04	0.060	2.9E-01
Mtr.19365.1.S1_at	6.14	6.00	7.01	6.23	6.17	6.24	6.38	0.55	6.22	0.04	0.97	-0.038	6.2E-01
Mtr.13470.1.S1_s_at	7.67	6.59	7.29	8.44	8.16	8.49	7.18	0.55	8.36	0.18	1.16	0.219	2.4E-02
Mtr.19517.1.S1_at	7.64	7.59	8.56	8.72	8.65	8.27	7.93	0.55	8.55	0.24	1.08	0.108	1.5E-01
Mtr.42944.1.S1_x_at	4.26	3.87	3.19	4.25	4.99	4.51	3.77	0.55	4.58	0.37	1.21	0.280	1.0E-01
Mtr.39450.1.S1_at	10.37	9.38	9.49	9.94	9.84	10.12	9.75	0.54	9.97	0.14	1.02	0.033	5.3E-01
Mtr.48358.1.S1_x_at	1.94	1.96	2.89	1.93	1.93	1.93	2.26	0.54	1.93	0.00	0.85	-0.231	3.5E-01
Mtr.8675.1.S1_at	12.76	11.67	12.24	12.36	12.28	12.42	12.22	0.54	12.35	0.07	1.01	0.016	7.0E-01
Mtr.18300.1.S1_at	9.40	10.09	10.48	10.13	10.09	10.00	9.99	0.54	10.07	0.07	1.01	0.012	8.0E-01
Mtr.9733.1.S1_at	6.66	5.73	6.68	6.68	6.68	6.95	6.36	0.54	6.77	0.16	1.06	0.090	2.8E-01
Mtr.11709.1.S1_at	6.89	5.99	6.97	7.21	7.08	7.16	6.62	0.54	7.15	0.06	1.08	0.112	1.7E-01
Mtr.22322.1.S1_at	6.25	5.35	6.33	6.45	6.53	6.60	5.97	0.54	6.53	0.07	1.09	0.128	1.6E-01
Mtr.7240.1.S1_at	6.56	5.63	6.59	6.22	6.56	6.75	6.26	0.54	6.51	0.27	1.04	0.056	5.2E-01
Mtr.3675.1.S1_at	3.84	2.76	3.39	2.78	4.53	3.73	3.33	0.54	3.68	0.88	1.11	0.145	5.9E-01
Mtr.42328.1.S1_at	7.08	6.42	7.50	7.40	7.68	7.43	7.00	0.54	7.50	0.15	1.07	0.101	2.0E-01
Mtr.16352.1.S1_x_at	3.79	3.51	2.74	2.90	2.68	2.94	3.34	0.54	2.84	0.14	0.85	-0.236	1.9E-01
Mtr.45048.1.S1_s_at	3.71	2.71	3.59	3.68	3.82	3.68	3.34	0.54	3.73	0.08	1.12	0.161	2.8E-01
Mtr.33638.1.S1_at	8.92	7.84	8.29	8.58	8.71	8.83	8.35	0.54	8.70	0.13	1.04	0.060	3.3E-01
Mtr.39395.1.S1_at	6.88	7.31	7.96	7.51	7.21	7.39	7.38	0.54	7.37	0.15	1.00	-0.001	9.8E-01
Mtr.32823.1.S1_at	3.63	2.99	4.07	4.55	4.63	3.63	3.56	0.54	4.27	0.56	1.20	0.262	1.9E-01
Mtr.40812.1.S1_at	3.24	2.56	3.63	3.06	3.06	3.01	3.14	0.54	3.04	0.03	0.97	-0.046	7.7E-01
Mtr.2723.1.S1_at	6.48	5.58	6.57	6.07	6.24	5.71	6.21	0.54	6.01	0.27	0.97	-0.047	6.0E-01
Mtr.30102.1.S1_at	5.93	5.79	6.80	6.40	6.88	6.08	6.17	0.54	6.45	0.40	1.04	0.063	5.2E-01
Mtr.20450.1.S1_at	6.63	5.56	6.27	6.81	6.44	6.42	6.15	0.54	6.56	0.22	1.07	0.092	3.0E-01
Mtr.27087.1.S1_at	4.29	3.43	4.44	4.03	4.03	3.63	4.05	0.54	3.90	0.23	0.96	-0.055	6.8E-01
Mtr.6518.1.S1_at	7.96	7.62	8.69	8.00	8.19	7.87	8.09	0.54	8.02	0.16	0.99	-0.012	8.4E-01
Mtr.11950.1.S1_at	6.69	5.81	6.81	7.04	7.22	6.93	6.44	0.54	7.07	0.15	1.10	0.135	1.2E-01
Mtr.26411.1.S1_at	4.34	3.67	4.74	4.24	4.37	4.24	4.25	0.54	4.28	0.07	1.01	0.011	9.2E-01
Mtr.9573.1.S1_at	7.04	5.96	6.63	6.85	6.60	6.77	6.54	0.54	6.74	0.13	1.03	0.043	5.7E-01
Mtr.2735.1.S1_at	7.88	6.79	7.35	7.76	8.03	7.97	7.34	0.54	7.92	0.14	1.08	0.110	1.5E-01
Mtr.41516.1.S1_at	8.69	7.67	8.51	8.50	8.54	8.24	8.29	0.54	8.43	0.17	1.02	0.024	7.0E-01
Mtr.39475.1.S1_at	9.77	8.70	9.10	9.40	9.20	9.48	9.19	0.54	9.36	0.14	1.02	0.027	6.2E-01
Mtr.33891.1.S1_at	2.72	3.68	2.75	2.75	2.83	2.70	3.05	0.54	2.76	0.07	0.90	-0.145	4.1E-01
Mtr.13959.1.S1_at	9.58	9.23	10.30	9.34	9.42	9.04	9.70	0.54	9.26	0.20	0.95	-0.067	2.6E-01
Mtr.41506.1.S1_at	10.34	9.40	10.35	10.41	10.62	10.65	10.03	0.54	10.56	0.13	1.05	0.074	1.8E-01
Mtr.32445.1.S1_at	8.52	7.52	8.39	8.78	8.48	8.78	8.14	0.54	8.68	0.17	1.07	0.091	1.8E-01
Mtr.1518.1.S1_at	3.54	4.46	3.50	3.55	3.74	3.49	3.83	0.54	3.59	0.13	0.94	-0.094	4.9E-01
Mtr.41889.1.S1_s_at	7.62	6.70	7.67	7.21	7.24	6.87	7.33	0.54	7.11	0.20	0.97	-0.045	5.4E-01
Mtr.18787.1.S1_at	7.62	6.76	7.76	7.78	7.63	7.47	7.38	0.54	7.63	0.16	1.03	0.047	5.0E-01
Mtr.32814.1.S1_at	10.36	9.28	9.68	9.97	9.86	9.79	9.77	0.54	9.87	0.09	1.01	0.015	7.7E-01
Mtr.27313.1.S1_at	6.61	5.69	6.65	6.75	6.88	7.48	6.32	0.54	7.04	0.39	1.11	0.156	1.3E-01
Mtr.13984.1.S1_at	10.03	8.95	9.55	9.67	9.56	9.68	9.51	0.54	9.64	0.07	1.01	0.019	7.1E-01
Mtr.17436.1.S1_at	6.69	5.68	5.83	6.43	6.39	6.52	6.07	0.54	6.45	0.07	1.06	0.087	3.0E-01
Mtr.9708.1.S1_at	9.20	8.34	8.20	8.19	8.02	8.01	8.58	0.54	8.07	0.10	0.94	-0.088	1.9E-01
Mtr.20228.1.S1_at	4.09	4.82	5.15	7.27	7.66	7.11	4.69	0.54	7.34	0.28	1.57	0.647	1.7E-03
Mtr.29244.1.S1_at	5.01	3.96	4.74	4.81	4.77	4.67	4.57	0.54	4.75	0.07	1.04	0.055	6.0E-01
Mtr.26689.1.S1_at	9.94	9.05	10.04	10.13	10.06	9.88	9.68	0.54	10.02	0.13	1.04	0.050	3.5E-01
Mtr.3596.1.S1_at	2.31	1.94	3.01	2.17	2.28	2.03	2.42	0.54	2.16	0.12	0.89	-0.164	4.6E-01
Mtr.38237.1.S1_at	9.89	8.84	9.60	9.73	9.67	9.87	9.44	0.54	9.76	0.10	1.03	0.048	3.7E-01
Mtr.26811.1.S1_at	4.00	3.40	4.48	3.61	4.29	4.03	3.96	0.54	3.98	0.34	1.00	0.006	9.7E-01
Mtr.47707.1.S1_at	4.21	3.22	4.10	4.25	4.09	4.11	3.84	0.54	4.15	0.09	1.08	0.112	3.8E-01
Mtr.42442.1.S1_at	9.13	8.71	9.78	9.35	9.41	9.36	9.21	0.54	9.37	0.03	1.02	0.026	6.2E-01
Mtr.15429.1.S1_at	3.22	4.16	3.21	3.20	3.18	3.19	3.53	0.54	3.19	0.01	0.90	-0.145	3.4E-01
Mtr.13588.1.S1_at	6.59	5.90	6.97	7.06	7.04	7.16	6.49	0.54	7.09	0.07	1.09	0.127	1.3E-01
Mtr.35446.1.S1_at	7.38	6.64	7.70	7.93	7.65	7.62	7.24	0.54	7.74	0.17	1.07	0.096	2.0E-01
Mtr.4177.1.S1_at	10.05	9.02	9.82	9.81	9.91	9.82	9.63	0.54	9.84	0.05	1.02	0.032	5.4E-01
Mtr.18499.1.S1_at	6.49	6.08	5.41	6.19	6.44	6.07	5.99	0.54	6.23	0.19	1.04	0.056	5.1E-01
Mtr.14190.1.S1_s_at	12.06	11.04	11.23	11.66	11.67	12.04	11.45	0.54	11.79	0.22	1.03	0.042	3.7E-01
Mtr.26543.1.S1_at	2.98	3.91	2.95	2.95	2.94	2.94	3.28	0.54	2.94	0.00	0.90	-0.157	3.4E-01
Mtr.31244.1.S1_at	8.11	7.49	8.57	8.17	8.19	7.99	8.06	0.54	8.12	0.11	1.01	0.011	8.6E-01
Mtr.42745.1.S1_at	6.51	5.77	6.83	6.66	6.46	6.46	6.37	0.54	6.53	0.12	1.03	0.036	6.4E-01
Mtr.20561.1.S1_at	8.64	7.78	8.79	8.64	8.59	8.55	8.40	0.54	8.59	0.05	1.02	0.032	5.8E-01
Mtr.13736.1.S1_at	9.08	8.37	9.43	9.05	9.25	9.11	8.96	0.54	9.14	0.10	1.02	0.029	6.0E-01
Mtr.31696.1.S1_at	6.99	6.58	7.65	7.10	6.98	7.02	7.07	0.54	7.03	0.06	0.99	-0.009	9.0E-01
Mtr.23095.1.S1_at	2.66	3.57	2.61	2.62	2.56	2.62	2.95	0.54	2.60	0.04	0.88	-0.181	3.3E-01
Mtr.355.1.S1_at	8.78	7.89	8.87	8.80	8.74	8.51	8.51	0.54	8.68	0.16	1.02	0.028	6.3E-01
Mtr.4265.1.S1_at	2.53	3.60	3.25	2.53	2.86	2.53	3.13	0.54	2.64	0.19	0.84	-0.247	2.1E-01
Mtr.4275.1.S1_at	6.43	5.61	6.63	6.41	6.22	6.51	6.23	0.54	6.38	0.15	1.02	0.035	6.6E-01
Mtr.49402.1.S1_at	3.38	2.66	3.72	3.29	4.01	4.28	3.25	0.54	3.86	0.51	1.19	0.248	2.3E-01
Mtr.31723.1.S1_at	8.99	8.05	8.99	8.86	8.83	8.89	8.68	0.54	8.86	0.03	1.02	0.030	6.0E-01
Mtr.38360.1.S1_at	8.42	7.63	8.66	8.21	8.19	8.06	8.24	0.54	8.15	0.08	0.99	-0.015	8.1E-01
Mtr.5254.1.S1_s_at	9.23	8.24	9.11	9.06	9.08	9.01	8.86	0.54	9.05	0.04	1.02	0.031	5.8E-01
Mtr.1820.1.S1_s_at	8.47	7.62	8.62	8.52	8.51	8.56	8.24	0.54	8.53	0.03	1.04	0.050	4.0E-01
Mtr.32890.1.S1_at	6.74	7.77	7.55	8.62	8.92	8.19	7.35	0.54	8.58	0.37	1.17	0.223	3.2E-02
Mtr.18075.1.S1_x_at	4.48	3.57	3.52	3.39	3.43	3.98	3.86	0.54	3.60	0.33	0.93	-0.099	5.3E-01
Mtr.6736.1.S1_at	4.07	3.44	4.52	3.74	3.97	3.97	4.01	0.54	3.90	0.13	0.97	-0.041	7.5E-01
Mtr.47194.1.S1_at	2.97	3.90	2.95	2.94	2.92	2.92	3.27	0.54	2.92	0.01	0.89	-0.163	3.3E-01
Mtr.36679.1.S1_at	5.80	5.71	4.82	5.02	5.09	4.90	5.44	0.54	5.00	0.10	0.92	-0.122	2.4E-01
Mtr.11029.1.S1_at	12.75	11.84	12.80	12.59	12.50	12.57	12.46	0.54	12.55	0.05	1.01	0.011	7.9E-01
Mtr.16676.1.S1_at	3.14	4.09	3.16	3.14	3.11	3.18	3.46	0.54	3.15	0.04	0.91	-0.138	3.7E-01
Mtr.37172.1.S1_at	12.06	13.08	12.87	13.56	13.34	13.04	12.67	0.54	13.31	0.26	1.05	0.072	1.4E-01
Mtr.15373.1.S1_s_at	9.39	8.60	9.64	9.35	9.28	9.43	9.21	0.54	9.35	0.07	1.02	0.022	6.7E-01
Mtr.42268.1.S1_at	9.58	10.09	9.01	9.49	9.94	9.25	9.56	0.54	9.56	0.35	1.00	0.000	1.0E+00
Mtr.24207.1.S1_at	6.03	4.95	5.39	5.49	5.16	5.10	5.45	0.54	5.25	0.21	0.96	-0.055	5.7E-01
Mtr.45558.1.S1_at	8.32	7.29	8.10	7.75	7.68	7.61	7.90	0.54	7.68	0.07	0.97	-0.042	5.2E-01
Mtr.33928.1.S1_s_at	10.56	9.56	10.42	10.40	10.24	10.27	10.18	0.54	10.31	0.08	1.01	0.018	7.1E-01
Mtr.38603.1.S1_at	8.87	7.87	8.74	8.79	8.92	8.57	8.49	0.54	8.76	0.18	1.03	0.044	4.7E-01
Mtr.9847.1.S1_at	7.14	6.51	7.59	7.97	7.58	7.06	7.08	0.54	7.54	0.46	1.06	0.091	3.3E-01
Mtr.16051.1.S1_at	8.79	7.89	8.87	8.72	8.78	8.67	8.52	0.54	8.72	0.05	1.02	0.034	5.5E-01

Mtr.42882.1.S1_at	11.64	10.66	11.56	11.58	11.67	11.44	11.29	0.54	11.56	0.12	1.02	0.035	4.4E-01
Mtr.23315.1.S1_at	5.74	4.66	5.24	5.42	4.75	5.79	5.21	0.54	5.32	0.52	1.02	0.029	8.2E-01
Mtr.51758.1.S1_at	8.75	7.93	8.95	8.79	8.84	8.62	8.54	0.54	8.75	0.11	1.02	0.034	5.6E-01
Mtr.48010.1.S1_at	2.66	3.58	2.62	2.59	2.61	2.58	2.96	0.54	2.60	0.02	0.88	-0.187	3.2E-01
Mtr.1527.1.S1_at	5.21	4.20	5.05	5.14	5.28	5.13	4.82	0.54	5.18	0.09	1.08	0.105	3.2E-01
Mtr.13461.1.S1_at	8.41	7.49	8.44	8.27	8.27	8.31	8.11	0.54	8.29	0.02	1.02	0.030	6.1E-01
Mtr.569.1.S1_s_at	8.49	7.48	8.31	8.32	8.13	8.47	8.10	0.54	8.31	0.17	1.03	0.037	5.5E-01
Mtr.34432.1.S1_at	4.33	3.39	4.32	4.96	5.93	5.72	4.01	0.54	5.54	0.51	1.38	0.464	2.4E-02
Mtr.45535.1.S1_s_at	5.82	5.88	6.78	5.80	5.59	5.87	6.16	0.54	5.76	0.15	0.93	-0.098	2.8E-01
Mtr.45148.1.S1_at	5.10	4.35	5.40	5.55	5.78	5.43	4.95	0.54	5.59	0.18	1.13	0.175	1.2E-01
Mtr.32150.1.S1_at	6.80	5.84	6.76	7.04	6.85	6.92	6.47	0.54	6.94	0.10	1.07	0.101	2.1E-01
Mtr.35990.1.S1_at	8.99	8.06	9.00	8.90	9.01	8.72	8.69	0.54	8.88	0.15	1.02	0.032	5.8E-01
Mtr.16314.1.S1_at	9.27	8.58	9.65	9.17	9.24	9.18	9.17	0.54	9.19	0.04	1.00	0.004	9.3E-01
Mtr.27346.1.S1_at	7.24	8.13	8.21	7.55	7.99	7.76	7.86	0.54	7.77	0.22	0.99	-0.017	8.0E-01
Mtr.38234.1.S1_at	10.03	9.04	9.89	10.09	9.97	10.29	9.65	0.54	10.11	0.16	1.05	0.067	2.3E-01
Mtr.2715.1.S1_at	4.47	3.88	4.96	4.83	5.39	4.72	4.44	0.54	4.98	0.36	1.12	0.167	2.2E-01
Mtr.51896.1.S1_s_at	12.41	11.54	11.42	11.71	11.52	11.71	11.79	0.54	11.64	0.11	0.99	-0.018	6.8E-01
Mtr.4234.1.S1_at	4.38	3.37	4.21	4.41	6.32	5.05	3.99	0.54	5.26	0.97	1.32	0.399	1.2E-01
Mtr.2369.1.S1_at	5.98	5.71	6.75	6.53	6.76	6.37	6.15	0.54	6.55	0.20	1.07	0.092	2.9E-01
Mtr.7577.1.S1_at	2.33	3.30	2.40	2.39	2.39	2.39	2.68	0.54	2.39	0.00	0.89	-0.164	4.1E-01
Mtr.17893.1.S1_at	3.03	3.17	4.03	3.00	2.94	2.93	3.41	0.54	2.95	0.04	0.87	-0.208	2.2E-01
Mtr.37468.1.S1_at	13.66	12.92	12.61	13.08	12.91	13.09	13.07	0.54	13.03	0.10	1.00	-0.004	9.1E-01
Mtr.4168.1.S1_at	2.46	2.49	3.41	2.46	2.73	2.45	2.78	0.54	2.55	0.16	0.92	-0.128	5.1E-01
Mtr.39030.1.S1_at	8.53	7.54	8.40	8.42	8.24	8.32	8.16	0.54	8.33	0.09	1.02	0.030	6.2E-01
Mtr.38699.1.S1_at	9.36	8.33	9.13	9.64	9.67	9.72	8.94	0.54	9.67	0.04	1.08	0.114	7.9E-02
Mtr.51340.1.S1_at	9.42	8.47	9.40	9.26	9.28	9.16	9.10	0.54	9.23	0.06	1.01	0.021	6.9E-01
Mtr.31200.1.S1_at	7.43	6.56	7.55	7.29	7.29	7.28	7.18	0.54	7.29	0.01	1.01	0.021	7.5E-01
Mtr.13096.1.S1_at	8.46	7.82	8.89	8.39	8.41	8.40	8.39	0.54	8.40	0.01	1.00	0.001	9.8E-01
Mtr.11591.1.S1_at	9.23	8.15	8.73	8.76	8.71	9.09	8.71	0.54	8.85	0.21	1.02	0.024	6.9E-01
Mtr.33803.1.S1_at	8.20	7.35	8.35	7.80	7.94	7.86	7.97	0.54	7.87	0.07	0.99	-0.018	7.6E-01
Mtr.49411.1.S1_at	4.78	5.73	5.71	4.62	4.93	4.98	5.40	0.54	4.84	0.20	0.90	-0.158	1.7E-01
Mtr.13788.1.S1_at	8.73	7.77	8.67	8.54	8.54	8.69	8.39	0.54	8.59	0.08	1.02	0.034	5.6E-01
Mtr.21588.1.S1_at	2.73	3.73	3.58	4.90	3.35	4.97	3.34	0.54	4.40	0.92	1.32	0.398	1.6E-01
Mtr.11997.1.S1_at	7.24	6.71	7.78	7.25	7.43	6.98	7.24	0.54	7.22	0.23	1.00	-0.005	9.5E-01
Mtr.930.1.S1_at	9.55	8.54	9.38	8.85	8.81	8.91	9.16	0.54	8.86	0.05	0.97	-0.049	3.9E-01
Mtr.38059.1.S1_at	3.72	2.81	3.77	3.22	3.55	3.49	3.44	0.54	3.42	0.18	1.00	-0.006	9.6E-01
Mtr.8225.1.S1_at	1.95	2.88	1.94	1.94	1.94	1.94	2.26	0.54	1.94	0.00	0.86	-0.220	3.6E-01
Mtr.37389.1.S1_at	9.95	9.33	10.40	9.97	9.61	9.54	9.89	0.54	9.71	0.23	0.98	-0.027	6.1E-01
Mtr.11805.1.S1_s_at	8.86	7.96	8.92	8.81	8.67	8.77	8.58	0.54	8.75	0.07	1.02	0.029	6.1E-01
Mtr.47865.1.S1_at	12.65	11.90	12.94	13.01	12.56	12.75	12.50	0.54	12.77	0.22	1.02	0.031	4.6E-01
Mtr.8908.1.S1_at	8.10	7.20	8.16	7.72	8.30	8.07	7.82	0.54	8.03	0.30	1.03	0.039	5.8E-01
Mtr.16500.1.S1_at	3.71	2.68	2.93	3.61	3.69	4.19	3.11	0.54	3.83	0.31	1.23	0.301	1.2E-01
Mtr.7432.1.S1_at	7.92	7.69	8.72	8.70	8.96	8.53	8.11	0.54	8.73	0.21	1.08	0.106	1.4E-01
Mtr.32770.1.S1_at	7.91	7.08	8.08	8.09	8.08	7.88	7.69	0.54	8.02	0.12	1.04	0.059	3.7E-01
Mtr.27344.1.S1_at	4.06	3.14	4.08	4.24	4.05	3.98	3.76	0.54	4.09	0.14	1.09	0.123	3.6E-01
Mtr.13656.1.S1_at	10.28	11.36	10.82	10.37	10.59	10.46	10.82	0.54	10.47	0.11	0.97	-0.047	3.3E-01
Mtr.24473.1.S1_at	6.14	5.36	6.39	6.54	5.96	5.73	5.96	0.54	6.07	0.42	1.02	0.026	7.9E-01
Mtr.48881.1.S1_at	6.10	5.32	6.35	6.04	6.57	6.00	5.92	0.54	6.20	0.32	1.05	0.067	4.8E-01
Mtr.42531.1.S1_s_at	4.11	3.41	4.47	3.80	4.12	3.79	4.00	0.54	3.90	0.18	0.98	-0.035	7.9E-01
Mtr.39825.1.S1_at	12.21	12.15	11.25	11.86	11.99	12.06	11.87	0.54	11.97	0.10	1.01	0.012	7.6E-01
Mtr.43887.1.S1_at	9.95	9.78	10.79	10.78	10.68	10.48	10.18	0.54	10.65	0.15	1.05	0.066	2.2E-01
Mtr.15502.1.S1_s_at	9.58	8.65	9.59	9.47	9.42	9.52	9.27	0.54	9.47	0.05	1.02	0.030	5.6E-01
Mtr.4564.1.S1_at	6.23	5.97	7.00	6.63	7.30	6.64	6.40	0.54	6.85	0.39	1.07	0.099	3.0E-01
Mtr.19516.1.S1_at	10.63	10.07	11.15	11.04	10.42	10.83	10.62	0.54	10.76	0.31	1.01	0.020	7.0E-01
Mtr.42450.1.S1_at	8.84	8.86	9.78	9.69	9.62	9.38	9.16	0.54	9.56	0.16	1.04	0.062	2.8E-01
Mtr.6129.1.S1_at	9.15	8.56	9.63	9.07	9.22	8.98	9.11	0.54	9.09	0.12	1.00	-0.003	9.5E-01
Mtr.33404.1.S1_at	3.76	2.94	3.95	4.21	3.67	3.49	3.55	0.54	3.79	0.37	1.07	0.097	5.5E-01
Mtr.43822.1.S1_at	9.42	8.43	9.29	9.21	9.29	9.09	9.04	0.54	9.19	0.10	1.02	0.024	6.6E-01
Mtr.2675.1.S1_s_at	3.75	2.84	2.80	2.80	2.61	2.80	3.13	0.54	2.73	0.11	0.87	-0.196	2.8E-01
Mtr.5455.1.S1_s_at	10.01	9.03	9.91	9.87	9.87	10.02	9.65	0.54	9.92	0.09	1.03	0.040	4.4E-01
Mtr.43553.1.S1_at	8.96	8.15	9.17	9.03	8.57	8.98	8.76	0.54	8.86	0.25	1.01	0.016	7.9E-01
Mtr.7157.1.S1_at	7.97	6.95	7.16	7.71	7.53	7.77	7.36	0.54	7.67	0.13	1.04	0.060	3.9E-01
Mtr.32947.1.S1_at	8.09	8.76	9.16	8.20	8.42	8.12	8.67	0.54	8.25	0.16	0.95	-0.071	2.6E-01
Mtr.10897.1.S1_at	8.59	7.53	8.22	8.52	8.31	8.51	8.11	0.54	8.45	0.12	1.04	0.058	3.5E-01
Mtr.43987.1.S1_at	11.18	12.24	11.89	11.33	11.37	11.50	11.77	0.54	11.40	0.09	0.97	-0.046	3.0E-01
Mtr.42057.1.S1_at	6.69	5.84	6.83	6.65	6.78	6.93	6.45	0.54	6.79	0.14	1.05	0.073	3.6E-01
Mtr.11256.1.S1_at	11.52	11.75	12.55	12.24	12.59	12.35	11.94	0.54	12.40	0.18	1.04	0.054	2.4E-01
Mtr.35204.1.S1_at	10.31	10.08	9.28	9.97	9.86	9.66	9.89	0.54	9.83	0.16	0.99	-0.009	8.6E-01
Mtr.5357.1.S1_at	3.34	3.27	4.23	3.32	3.41	3.22	3.61	0.54	3.32	0.10	0.92	-0.122	4.0E-01
Mtr.30212.1.S1_a_at	3.78	2.91	2.80	2.70	2.70	3.54	3.16	0.54	2.98	0.48	0.94	-0.087	6.8E-01
Mtr.4738.1.S1_s_at	2.38	2.60	3.40	2.69	2.36	2.64	2.79	0.54	2.57	0.18	0.92	-0.122	5.3E-01
Mtr.44831.1.S1_at	4.35	3.46	4.43	3.97	4.12	4.08	4.08	0.54	4.06	0.08	0.99	-0.009	9.4E-01
Mtr.12289.1.S1_at	6.01	7.08	6.59	7.11	7.33	7.38	6.56	0.54	7.27	0.14	1.11	0.148	9.1E-02
Mtr.48993.1.S1_at	2.46	3.39	2.45	2.45	2.45	2.45	2.77	0.54	2.45	0.00	0.89	-0.174	3.7E-01
Mtr.2656.1.S1_at	7.29	6.85	7.92	7.85	8.00	7.84	7.35	0.54	7.90	0.09	1.07	0.103	1.6E-01
Mtr.43688.1.S1_at	9.69	8.62	9.12	9.06	9.20	8.83	9.14	0.54	9.03	0.19	0.99	-0.018	7.5E-01
Mtr.13700.1.S1_at	5.78	4.72	5.11	5.00	5.01	5.04	5.20	0.54	5.02	0.02	0.96	-0.052	5.9E-01
Mtr.39092.1.S1_at	9.57	8.50	9.15	8.93	9.34	9.04	9.07	0.54	9.11	0.21	1.00	0.006	9.2E-01
Mtr.14895.1.S1_at	2.31	3.24	2.30	2.30	2.29	2.30	2.62	0.54	2.29	0.01	0.88	-0.189	3.6E-01
Mtr.4284.1.S1_at	7.81	6.81	7.64	7.69	7.86	7.87	7.42	0.54	7.81	0.10	1.05	0.073	2.9E-01
Mtr.32270.1.S1_at	9.99	8.93	9.33	9.60	9.39	9.60	9.42	0.54	9.53	0.12	1.01	0.017	7.4E-01
Mtr.23529.1.S1_x_at	2.83	3.74	2.80	2.78	2.75	2.66	3.12	0.54	2.73	0.06	0.87	-0.194	2.8E-01
Mtr.25640.1.S1_at	2.90	3.81	2.87	2.85	2.86	2.85	3.19	0.54	2.85	0.01	0.89	-0.162	3.4E-01
Mtr.1523.1.S1_at	9.67	8.88	9.90	9.94	9.75	9.91	9.48	0.54	9.87	0.10	1.04	0.057	2.9E-01
Mtr.39259.1.S1_at	5.67	4.61	5.30	5.26	5.57	5.38	5.20	0.54	5.41	0.15	1.04	0.057	5.5E-01
Mtr.10823.1.S1_s_at	8.96	7.99	8.86	9.24	9.21	9.49	8.60	0.54	9.32	0.16	1.08	0.115	9.2E-02
Mtr.34761.1.S1_at	7.91	6.99	6.96	7.56	7.98	7.80	7.29	0.54	7.78	0.21	1.07	0.094	2.1E-01
Mtr.17754.1.S1_at	8.40	7.70	8.75	8.43	8.47	8.36	8.28	0.54	8.42	0.06	1.02	0.024	6.8E-01
Mtr.39802.1.S1_at	8.16	7.21	8.12	8.38	8.25	8.62	7.83	0.54	8.42	0.19	1.07	0.104	1.5E-01
Mtr.4655.1.S1_at	6.28	5.62	6.68	6.13	6.25	6.27	6.19	0.54	6.22	0.08	1.00	0.005	9.5E-01
Mtr.4513.1.S1_x_at	7.01	7.12	6.14	7.44	7.32	7.55	6.76	0.54	7.44	0.12	1.10	0.138	9.9E-02
Mtr.31618.1.S1_at	8.10	7.23	8.21	8.25	7.92	8.14	7.84	0.54	8.11	0.17	1.03	0.047	4.7E-01
Mtr.41620.1.S1_at	8.46	7.73	8.78	8.89	8.68	8.84	8.32	0.54	8.80	0.11	1.06	0.081	2.0E-01
Mtr.32982.1.S1_at	8.51	8.63	9.50	8.85	8.52	8.77	8.88	0.54	8.71	0.17	0.98	-0.028	6.3E-01
Mtr.12015.1.S1_s_at	2.53	3.46	2.52	2.50	2.50	2.50	2.84	0.54	2.50	0.00	0.88	-0.182	3.4E-01
Mtr.40670.1.S1_at	12.47	13.26	13.50	12.75	12.92	12.93	13.08	0.54	12.87	0.10	0.98	-0.023	5.4E-01
Mtr.35261.1.S1_at	9.93	8.88	9.58	10.11	10.39	10.20	9.46	0.54	10.23	0.14	1.08	0.113	7.4E-02
Mtr.50393.1.S1_s_at	3.01	3.92	2.97	2.96	2.92	2.92	3.30	0.54	2.93	0.02	0.89	-0.170	3.0E-01
Mtr.18881.1.S1_x_at	2.66	3.57	2.63	2.61	2.62	2.61	2.95	0.54	2.62	0.00	0.89	-0.175	3.4E-01
Mtr.39714.1.S1_at	5.29	5.30	6.22	5.24	5.26	4.96	5.60	0.54	5.15	0.16	0.92	-0.121	2.4E-01
Mtr.499.1.S1_at	3.44	4.35	3.41	3.64	3.40	3.33	3.73	0.54	3.46	0.16	0.93	-0.110	4.4E-01
Mtr.21521.1.S1_at	6.72	6.45	7.48	6.91	7.46	6.55	6.88	0.54	6.97	0.46	1.01	0.019	8.4E-01
Mtr.37869.1.S1_at	11.85	10.78	11.30	11.41	11.42	11.56	11.31	0.54	11.46	0.09	1.01	0.019	6.6E-01
Mtr.20364.1.S1_at	2.46	3.42	3.34	4.02	3.32	3.44	3.07	0.54	3.59	0.37	1.17	0.225	2.4E-01
Mtr.40067.1.S1_x_at	11.45	11.02	10.39	11.50	10.67	11.15	10.95	0.54	11.11	0.42	1.01	0.020	7.2E-01
Mtr.42750.1.S1_at	8.24	7.22	8.01	8.24	8.25	8.31	7.82	0.54	8.27	0.04	1.06	0.080	2.2E-01
Mtr.18416.1.S1_at	2.37	3.29	2.36	2.36	2.36	2.36	2.67	0.54	2.36	0.00	0.88	-0.181	3.6E-01
Mtr.30374.1.S1_at	3.82	2.98	3.97	3.20	3.28	2.97	3.59	0.54	3.15	0.16	0.88	-0.189	2.4E-01
Mtr.10020.1.S1_at	4.19	3.37	4.38	4.13	4.86	3.74	3.98	0.54	4.24	0.57	1.07	0.092	5.9E-01
Mtr.41920.1.S1_at	7.88	7.69	8.70	8.64	8.73	8.71	8.09	0.54	8.70	0.05	1.07	0.104	1.2E-01
Mtr.39603.1.S1_at	6.97	5.90	6.50	6.45	6.48	6.44	6.46	0.54	6.46	0.02	1.00	0.000	1.0E+00
Mtr.49039.1.S1_s_at	9.85	10.51	10.91	10.28	11.26	10.13	10.42	0.54	10.56	0.61	1.01	0.018	7.9E-01
Mtr.39061.1.S1_at	7.19	6.21	7.07	6.89	6.99	7.01	6.82	0.54	6.96	0.06	1.02	0.030	6.7E-01
Mtr.5626.1.S1_at	8.24	7.29	8.19	8.27	7.92	8.21	7.91	0.53	8.13	0.19	1.03	0.040	5.3E-01
Mtr.13546.1.S1_at	7.79	6.83	7.72	7.78	7.59	7.96	7.44	0.53	7.78	0.19	1.04	0.063	3.7E-01
Mtr.10709.1.S1_at	8.46	7.50	8.39	8.34	8.25	8.06	8.12	0.53	8.22	0.15	1.01	0.017	7.7E-01
Mtr.17483.1.S1_x_at	9.00	8.34	9.40	9.14	9.19	9.16	8.91	0.53	9.16	0.03	1.03	0.040	4.6E-01
Mtr.40527.1.S1_at	12.33	11.28	11.62	11.98	12.05	11.94	11.74	0.53	11.99	0.05	1.02	0.030	4.7E-01
Mtr.6403.1.S1_at	3.07	2.19	2.11	2.10	2.10	2.10	2.45	0.53	2.10	0.00	0.86	-0.223	3.2E-01
Mtr.33831.1.S1_at	5.27	4.43	5.42	5.66	5.58	5.59	5.04	0.53	5.61	0.05	1.11	0.154	1.4E-01
Mtr.38847.1.S1_at	6.86	7.28	7.92	7.54	7.75	7.40	7.35	0.53	7.56	0.18	1.03	0.041	5.5E-01
Mtr.37149.1.S1_at	6.43	5.96	7.03	5.96	6.92	6.27	6.47	0.53	6.38	0.49	0.99	-0.020	8.5E-01
Mtr.7519.1.S1_at	8.19	7.13	7.61	8.17	8.22	8.29	7.64	0.53	8.23	0.06	1.08	0.106	1.3E-01
Mtr.6671.1.S1_at	8.50	7.45	8.13	8.10	8.33	8.33	8.03	0.53	8.25	0.13	1.03	0.040	5.2E-01
Mtr.41662.1.S1_at	9.29	8.34	9.23	9.22	9.59	9.14	8.95	0.53	9.32	0.24	1.04	0.057	3.5E-01
Mtr.51698.1.S1_at	9.09	8.39	8.04	8.85	8.65	9.14	8.50	0.53	8.88	0.25	1.04	0.062	3.3E-01
Mtr.2196.1.S1_at	6.02	5.01	5.23	5.76	5.98	6.04	5.42	0.53	5.92	0.15	1.09	0.128	1.9E-01
Mtr.34712.1.S1_at	8.40	9.41	8.59	8.74	8.60	8.49	8.80	0.53	8.61	0.12	0.98	-0.032	5.8E-01
Mtr.29735.1.S1_at	5.23	6.14	5.21	5.20	5.20	5.16	5.53	0.53	5.19	0.03	0.94	-0.091	3.3E-01
Mtr.21870.1.S1_at	5.94	5.10	6.10	6.33	5.81	5.94	5.71	0.53	6.02	0.27	1.05	0.076	4.2E-01
Mtr.37542.1.S1_at	10.04	10.19	11.03	11.29	11.51	11.44	10.42	0.53	11.41	0.12	1.10	0.131	3.5E-02

Mtr.33331.1.S1_s_at	7.98	6.97	7.77	7.74	8.16	7.90	7.57	0.53	7.94	0.21	1.05	0.067	3.4E-01
Mtr.42078.1.S1_at	11.44	10.42	10.66	10.51	10.93	11.03	10.84	0.53	10.82	0.28	1.00	-0.003	9.5E-01
Mtr.39463.1.S1_at	9.47	9.59	10.45	9.92	9.94	9.81	9.84	0.53	9.89	0.07	1.01	0.007	8.8E-01
Mtr.8657.1.S1_at	10.22	9.16	9.56	9.93	10.09	10.16	9.65	0.53	10.06	0.12	1.04	0.061	2.6E-01
Mtr.48086.1.S1_at	9.19	10.07	10.15	9.50	9.67	9.31	9.80	0.53	9.49	0.18	0.97	-0.047	3.9E-01
Mtr.24249.1.S1_at	10.29	9.37	10.31	10.35	10.30	10.38	9.99	0.53	10.34	0.04	1.04	0.050	3.2E-01
Mtr.3345.1.S1_at	4.71	4.75	5.65	4.01	5.58	4.56	5.04	0.53	4.72	0.80	0.94	-0.095	6.0E-01
Mtr.39142.1.S1_at	9.84	8.79	9.19	9.34	9.30	9.45	9.27	0.53	9.36	0.08	1.01	0.014	7.9E-01
Mtr.12993.1.S1_x_at	2.83	3.73	2.78	2.76	2.72	2.73	3.11	0.53	2.74	0.02	0.88	-0.185	2.9E-01
Mtr.8794.1.S1_at	9.49	8.85	9.91	9.47	9.48	9.42	9.42	0.53	9.45	0.03	1.00	0.006	9.1E-01
Mtr.12847.1.S1_at	9.11	8.37	9.40	9.16	9.08	8.88	8.96	0.53	9.04	0.14	1.01	0.013	8.2E-01
Mtr.46014.1.S1_at	3.81	2.92	2.85	2.89	2.88	2.88	3.19	0.53	2.88	0.01	0.90	-0.147	3.7E-01
Mtr.33524.1.S1_at	4.99	4.72	5.75	4.65	4.66	4.61	5.15	0.53	4.64	0.03	0.90	-0.152	1.7E-01
Mtr.45998.1.S1_at	9.14	8.12	8.91	8.88	8.62	8.79	8.72	0.53	8.76	0.13	1.00	0.006	9.1E-01
Mtr.4814.1.S1_at	9.94	11.00	10.58	9.89	10.57	10.19	10.51	0.53	10.22	0.34	0.97	-0.040	4.8E-01
Mtr.27394.1.S1_at	6.84	5.91	6.83	7.00	6.66	6.94	6.53	0.53	6.87	0.18	1.05	0.072	3.6E-01
Mtr.2306.1.S1_at	2.75	2.77	3.68	2.74	2.74	2.71	3.07	0.53	2.73	0.02	0.89	-0.169	3.3E-01
Mtr.12160.1.S1_at	3.75	3.77	4.69	3.80	4.01	3.74	4.07	0.53	3.85	0.14	0.95	-0.081	5.2E-01
Mtr.11499.1.S1_at	10.72	11.67	10.77	11.02	10.83	11.09	11.05	0.53	10.98	0.13	0.99	-0.010	8.3E-01
Mtr.48940.1.S1_at	10.53	9.82	10.86	10.56	10.48	10.52	10.40	0.53	10.52	0.04	1.01	0.016	7.2E-01
Mtr.12833.1.S1_at	9.87	10.62	10.91	10.42	10.29	10.38	10.46	0.53	10.36	0.07	0.99	-0.014	7.6E-01
Mtr.39751.1.S1_at	10.67	9.77	10.71	10.59	10.45	10.61	10.38	0.53	10.55	0.09	1.02	0.023	6.2E-01
Mtr.38486.1.S1_at	12.28	11.36	11.37	11.83	11.81	11.83	11.67	0.53	11.82	0.01	1.01	0.019	6.5E-01
Mtr.20220.1.S1_at	2.94	3.85	2.92	3.63	2.91	2.83	3.24	0.53	3.12	0.44	0.97	-0.051	7.9E-01
Mtr.33475.1.S1_s_at	8.60	9.30	8.26	8.83	9.86	8.81	8.72	0.53	9.17	0.60	1.05	0.073	3.9E-01
Mtr.3999.1.S1_at	2.67	3.21	3.73	2.62	2.89	2.68	3.20	0.53	2.73	0.14	0.85	-0.230	2.1E-01
Mtr.39381.1.S1_at	6.77	6.80	7.71	6.76	7.37	6.84	7.09	0.53	6.99	0.33	0.99	-0.021	8.0E-01
Mtr.18320.1.S1_at	3.15	4.05	3.11	3.15	3.11	3.25	3.44	0.53	3.17	0.07	0.92	-0.117	4.4E-01
Mtr.41881.1.S1_at	9.73	8.79	9.69	9.70	9.77	9.51	9.40	0.53	9.66	0.14	1.03	0.039	4.6E-01
Mtr.3019.1.S1_at	7.54	6.52	7.30	7.27	7.76	7.27	7.12	0.53	7.43	0.28	1.04	0.062	4.2E-01
Mtr.8504.1.S1_at	13.75	12.69	13.13	13.17	12.87	13.23	13.19	0.53	13.09	0.19	0.99	-0.011	7.7E-01
Mtr.41930.1.S1_at	13.01	12.26	11.98	12.42	12.28	12.48	12.42	0.53	12.39	0.10	1.00	-0.002	9.5E-01
Mtr.36141.1.S1_at	6.31	5.70	5.24	5.61	5.41	5.68	5.75	0.53	5.57	0.14	0.97	-0.047	5.9E-01
Mtr.39622.1.S1_at	4.73	4.36	5.41	4.73	4.73	4.73	4.84	0.53	4.73	0.00	0.98	-0.032	7.5E-01
Mtr.49038.1.S1_s_at	9.57	10.43	10.55	10.10	10.63	9.81	10.18	0.53	10.18	0.42	1.00	0.000	1.0E+00
Mtr.29719.1.S1_at	6.42	5.65	6.68	5.92	6.32	6.31	6.25	0.53	6.19	0.23	0.99	-0.015	8.5E-01
Mtr.23575.1.S1_x_at	12.75	12.97	11.96	12.59	13.35	12.93	12.56	0.53	12.96	0.38	1.03	0.044	3.6E-01
Mtr.5642.1.S1_at	2.96	2.56	3.61	3.01	2.99	2.95	3.04	0.53	2.98	0.03	0.98	-0.027	8.6E-01
Mtr.8439.1.S1_at	5.24	4.30	4.34	5.47	5.20	5.23	4.62	0.53	5.30	0.15	1.15	0.197	1.0E-01
Mtr.1622.1.S1_at	6.51	5.47	5.80	5.77	5.71	5.88	5.93	0.53	5.79	0.08	0.98	-0.035	6.7E-01
Mtr.35493.1.S1_at	2.52	3.42	2.48	2.48	2.48	2.45	2.81	0.53	2.47	0.01	0.88	-0.185	3.3E-01
Mtr.40495.1.S1_at	13.11	14.03	13.11	12.84	13.10	12.98	13.41	0.53	12.97	0.13	0.97	-0.048	2.3E-01
Mtr.37375.1.S1_at	10.82	11.01	10.01	9.79	9.87	9.78	10.61	0.53	9.81	0.05	0.92	-0.113	6.0E-02
Mtr.813.1.S1_at	2.93	3.82	2.87	2.85	2.83	2.79	3.21	0.53	2.82	0.03	0.88	-0.185	2.8E-01
Mtr.6153.1.S1_at	8.40	9.44	9.12	8.80	9.00	8.83	8.98	0.53	8.88	0.11	0.99	-0.017	7.6E-01
Mtr.38276.1.S1_at	8.95	8.14	9.14	8.73	8.98	8.79	8.74	0.53	8.83	0.13	1.01	0.015	7.9E-01
Mtr.38780.1.S1_at	5.88	5.82	6.77	6.92	6.98	7.16	6.15	0.53	7.02	0.12	1.14	0.190	5.2E-02
Mtr.9800.1.S1_at	8.37	7.36	8.17	8.52	8.35	8.53	7.97	0.53	8.47	0.10	1.06	0.088	1.8E-01
Mtr.37792.1.S1_at	12.12	11.09	11.83	11.69	11.49	11.57	11.68	0.53	11.59	0.10	0.99	-0.012	7.7E-01
Mtr.45397.1.S1_at	5.06	4.24	5.23	4.97	5.51	5.42	4.84	0.53	5.30	0.29	1.09	0.130	2.6E-01
Mtr.29729.1.S1_at	4.27	3.31	4.19	4.14	4.77	4.16	3.92	0.53	4.36	0.36	1.11	0.151	3.1E-01
Mtr.26815.1.S1_at	2.92	3.81	2.88	2.86	2.87	2.86	3.20	0.53	2.86	0.01	0.89	-0.162	3.3E-01
Mtr.45154.1.S1_at	9.63	8.62	9.40	9.30	9.32	9.23	9.21	0.53	9.28	0.05	1.01	0.010	8.4E-01
Mtr.46633.1.S1_at	5.93	4.87	5.47	5.58	5.23	5.10	5.42	0.53	5.30	0.25	0.98	-0.032	7.5E-01
Mtr.48137.1.S1_at	2.53	3.43	2.50	2.60	2.47	2.46	2.82	0.53	2.51	0.08	0.89	-0.167	3.8E-01
Mtr.7522.1.S1_s_at	10.09	9.11	9.95	10.00	10.13	10.09	9.72	0.53	10.07	0.07	1.04	0.052	3.2E-01
Mtr.43992.1.S1_at	7.43	6.40	6.69	7.08	6.93	6.71	6.84	0.53	6.91	0.18	1.01	0.015	8.4E-01
Mtr.12714.1.S1_at	12.92	11.88	12.60	12.71	12.67	12.60	12.47	0.53	12.66	0.06	1.02	0.022	5.7E-01
Mtr.41340.1.S1_at	8.50	7.62	8.57	8.41	8.26	8.50	8.23	0.53	8.39	0.12	1.02	0.028	6.3E-01
Mtr.12303.1.S1_at	8.37	9.43	8.96	10.87	11.24	11.04	8.92	0.53	11.05	0.19	1.24	0.309	2.8E-03
Mtr.37012.1.S1_at	2.66	3.55	2.60	2.59	2.61	2.76	2.94	0.53	2.65	0.09	0.90	-0.147	4.1E-01
Mtr.43890.1.S1_at	10.26	10.96	11.30	10.69	10.88	10.66	10.84	0.53	10.75	0.12	0.99	-0.013	7.8E-01
Mtr.37102.1.S1_at	3.10	3.95	2.98	3.42	2.95	2.91	3.34	0.53	3.09	0.29	0.93	-0.111	5.1E-01
Mtr.48714.1.S1_s_at	10.06	9.05	9.83	10.60	10.29	10.58	9.65	0.53	10.49	0.17	1.09	0.121	5.9E-02
Mtr.5108.1.S1_at	6.80	5.88	6.79	6.78	6.78	6.87	6.49	0.53	6.81	0.05	1.05	0.069	3.6E-01
Mtr.19153.1.S1_at	8.26	9.22	9.14	8.56	8.95	8.44	8.87	0.53	8.65	0.27	0.97	-0.037	5.5E-01
Mtr.45521.1.S1_at	7.66	6.84	7.84	7.52	7.07	7.17	7.45	0.53	7.25	0.23	0.97	-0.038	5.9E-01
Mtr.6693.1.S1_s_at	7.19	6.59	7.65	7.08	6.83	6.91	7.14	0.53	6.94	0.12	0.97	-0.042	5.5E-01
Mtr.18451.1.S1_x_at	3.13	3.76	2.70	3.46	3.03	3.02	3.20	0.53	3.17	0.25	0.99	-0.011	9.5E-01
Mtr.32348.1.S1_at	4.18	3.28	4.22	5.40	4.42	4.80	3.90	0.53	4.87	0.49	1.25	0.323	7.9E-02
Mtr.45080.1.S1_at	8.96	8.09	9.05	9.25	9.24	9.26	8.70	0.53	9.25	0.01	1.06	0.089	1.5E-01
Mtr.45139.1.S1_at	10.52	9.63	9.58	9.86	9.73	10.04	9.91	0.53	9.88	0.16	1.00	-0.004	9.3E-01
Mtr.27178.1.S1_at	3.07	2.85	3.86	3.03	3.31	2.98	3.26	0.53	3.11	0.18	0.95	-0.069	6.6E-01
Mtr.37670.1.S1_at	7.62	6.61	7.39	7.49	7.51	7.53	7.21	0.53	7.51	0.02	1.04	0.059	3.8E-01
Mtr.14972.1.S1_at	9.83	8.81	9.56	10.07	9.77	9.90	9.40	0.53	9.91	0.15	1.05	0.077	1.8E-01
Mtr.18536.1.S1_at	11.64	10.58	11.16	11.29	11.19	11.32	11.13	0.53	11.26	0.07	1.01	0.017	6.8E-01
Mtr.28631.1.S1_at	7.55	6.56	7.39	8.34	9.08	8.80	7.16	0.53	8.74	0.37	1.22	0.287	1.4E-02
Mtr.14499.1.S1_at	4.28	5.14	5.24	3.39	3.33	3.24	4.89	0.53	3.32	0.07	0.68	-0.558	7.1E-03
Mtr.48539.1.S1_at	3.10	3.57	2.51	2.57	2.57	2.57	3.06	0.53	2.57	0.00	0.84	-0.253	1.8E-01
Mtr.16295.1.S1_at	2.71	3.43	2.40	2.40	2.40	2.40	2.85	0.53	2.40	0.00	0.84	-0.247	2.2E-01
Mtr.39120.1.S1_s_at	7.67	7.44	8.45	8.33	8.55	8.23	7.85	0.53	8.37	0.17	1.07	0.092	1.8E-01
Mtr.5347.1.S1_at	6.78	6.48	7.51	7.23	7.35	7.31	6.92	0.53	7.30	0.06	1.05	0.075	3.0E-01
Mtr.43001.1.S1_at	9.69	8.80	9.74	9.57	9.50	9.47	9.41	0.53	9.52	0.05	1.01	0.016	7.5E-01
Mtr.39459.1.S1_at	4.02	3.37	4.42	3.51	4.36	3.84	3.93	0.53	3.90	0.43	0.99	-0.012	9.4E-01
Mtr.51092.1.S1_at	3.27	4.23	3.36	3.31	3.20	3.33	3.62	0.53	3.28	0.07	0.91	-0.143	3.3E-01
Mtr.32384.1.S1_at	4.49	3.46	4.21	4.16	4.05	4.15	4.05	0.53	4.12	0.06	1.02	0.024	8.3E-01
Mtr.27128.1.S1_at	5.36	4.32	4.64	5.28	5.32	5.24	4.77	0.53	5.28	0.04	1.11	0.145	1.8E-01
Mtr.23640.1.S1_at	2.38	2.53	3.36	2.35	2.35	2.37	2.75	0.53	2.36	0.01	0.86	-0.224	2.6E-01
Mtr.42431.1.S1_at	7.55	6.54	7.32	7.47	7.62	7.47	7.14	0.53	7.52	0.09	1.05	0.075	2.8E-01
Mtr.13434.1.S1_at	9.73	8.67	9.26	9.10	9.26	9.30	9.22	0.53	9.22	0.11	1.00	0.000	1.0E+00
Mtr.20496.1.S1_at	9.53	10.15	10.59	10.27	10.17	10.00	10.09	0.53	10.15	0.14	1.01	0.008	8.7E-01
Mtr.42518.1.S1_at	7.28	6.50	7.51	7.95	8.39	8.18	7.10	0.53	8.17	0.22	1.15	0.204	3.1E-02
Mtr.16353.1.S1_at	4.29	5.31	4.57	6.83	7.86	7.23	4.73	0.53	7.31	0.52	1.55	0.629	3.8E-03
Mtr.183.1.S1_at	4.02	3.00	3.77	3.79	3.76	3.75	3.60	0.53	3.77	0.02	1.05	0.066	6.1E-01
Mtr.20925.1.S1_at	8.96	8.32	9.37	9.05	8.99	9.10	8.89	0.53	9.04	0.05	1.02	0.026	6.3E-01
Mtr.11137.1.S1_at	8.38	7.48	8.40	8.66	8.60	8.64	8.09	0.53	8.63	0.03	1.07	0.094	1.5E-01
Mtr.5825.1.S1_at	5.30	4.60	5.63	5.17	5.40	5.38	5.17	0.53	5.32	0.13	1.03	0.039	6.7E-01
Mtr.3434.1.S1_at	8.99	8.05	8.93	8.63	9.19	8.98	8.66	0.53	8.93	0.28	1.03	0.045	4.7E-01
Mtr.24536.1.S1_at	4.09	3.07	3.83	3.83	4.29	3.32	3.66	0.53	3.81	0.49	1.04	0.057	7.4E-01
Mtr.5904.1.S1_at	6.23	5.82	6.87	6.88	6.63	6.54	6.31	0.53	6.68	0.18	1.06	0.084	3.0E-01
Mtr.37493.1.S1_at	11.22	12.18	11.33	12.25	12.62	12.53	11.58	0.53	12.46	0.19	1.08	0.106	5.3E-02
Mtr.2439.1.S1_at	11.49	10.78	10.45	10.97	10.83	11.01	10.91	0.53	10.94	0.09	1.00	0.004	9.4E-01
Mtr.34770.1.S1_at	8.20	7.17	7.89	8.04	8.12	8.10	7.75	0.53	8.09	0.05	1.04	0.060	3.4E-01
Mtr.30984.1.S1_at	7.73	6.79	7.68	7.39	7.83	7.10	7.40	0.53	7.44	0.37	1.01	0.009	9.1E-01
Mtr.43771.1.S1_at	4.05	3.91	4.89	4.34	4.24	4.20	4.28	0.53	4.26	0.07	1.00	-0.007	9.5E-01
Mtr.37870.1.S1_at	11.44	10.46	10.60	10.80	10.80	10.83	10.83	0.53	10.81	0.02	1.00	-0.003	9.5E-01
Mtr.12777.1.S1_at	9.55	8.77	9.78	9.67	9.84	9.48	9.37	0.53	9.66	0.18	1.03	0.045	4.1E-01
Mtr.20240.1.S1_at	8.78	7.92	8.88	9.03	9.15	9.17	8.53	0.53	9.12	0.08	1.07	0.097	1.3E-01
Mtr.5026.1.S1_at	5.89	6.17	5.15	5.79	5.84	6.00	5.74	0.53	5.87	0.11	1.02	0.035	6.8E-01
Mtr.27840.1.S1_at	5.80	4.82	5.64	5.61	5.61	5.60	5.42	0.53	5.61	0.01	1.03	0.049	5.7E-01
Mtr.20033.1.S1_at	4.48	5.11	5.53	4.92	5.04	5.26	5.04	0.53	5.08	0.17	1.01	0.011	9.1E-01
Mtr.9578.1.S1_at	9.01	8.22	9.22	8.97	8.74	8.40	8.82	0.53	8.70	0.29	0.99	-0.019	7.6E-01
Mtr.29363.1.S1_at	5.39	6.42	5.70	5.38	5.38	5.41	5.84	0.53	5.39	0.02	0.92	-0.115	2.2E-01
Mtr.13161.1.S1_at	8.93	7.88	8.35	8.78	8.78	9.05	8.39	0.53	8.87	0.16	1.06	0.081	2.0E-01
Mtr.39472.1.S1_at	5.67	4.95	4.64	5.46	5.42	5.96	5.09	0.53	5.61	0.30	1.10	0.142	2.1E-01
Mtr.2862.1.S1_at	8.05	7.80	8.81	8.37	8.11	8.32	8.22	0.53	8.27	0.13	1.01	0.008	9.0E-01
Mtr.42941.1.S1_at	9.64	8.70	8.75	8.15	7.91	8.20	9.03	0.53	8.08	0.16	0.90	-0.159	4.1E-02
Mtr.33405.1.S1_x_at	5.84	4.85	5.05	5.12	4.72	4.99	5.25	0.53	4.94	0.21	0.94	-0.087	4.0E-01
Mtr.43791.1.S1_at	8.81	7.76	8.22	8.51	8.66	8.52	8.26	0.53	8.56	0.08	1.04	0.051	3.9E-01
Mtr.26989.1.S1_at	8.18	7.98	8.98	8.45	8.39	7.96	8.38	0.53	8.27	0.27	0.99	-0.020	7.5E-01
Mtr.29235.1.S1_at	3.58	2.67	3.57	4.81	3.56	3.57	3.28	0.53	3.98	0.72	1.22	0.281	2.4E-01
Mtr.38669.1.S1_at	8.41	8.13	9.15	8.94	8.93	8.81	8.56	0.53	8.89	0.07	1.04	0.054	3.4E-01
Mtr.3942.1.S1_at	7.25	7.06	8.05	8.07	7.39	7.21	7.45	0.53	7.56	0.46	1.01	0.020	8.1E-01
Mtr.32636.1.S1_at	10.57	9.99	11.05	10.77	10.79	10.61	10.54	0.53	10.73	0.10	1.02	0.025	5.8E-01
Mtr.36799.1.S1_at	9.46	8.66	9.65	9.49	9.51	9.30	9.25	0.53	9.43	0.11	1.02	0.027	6.0E-01
Mtr.1595.1.S1_at	8.24	7.41	8.39	8.21	8.35	8.22	8.02	0.53	8.26	0.07	1.03	0.043	4.7E-01
Mtr.37676.1.S1_at	10.35	9.32	9.64	9.98	10.18	10.16	9.77	0.53	10.11	0.11	1.03	0.050	3.3E-01
Mtr.17753.1.S1_at	7.52	6.67	7.63	7.77	7.88	7.61	7.27	0.53	7.75	0.13	1.07	0.092	2.0E-01
Mtr.20169.1.S1_at	2.77	3.68	2.76	2.76	2.76	2.75	3.07	0.53	2.75	0.00	0.90	-0.157	3.6E-01
Mtr.6261.1.S1_at	3.70	3.16	4.21	3.64	3.70	3.59	3.69	0.53	3.64	0.06	0.99	-0.018	8.9E-01
Mtr.4263.1.S1_at	4.21	3.57	4.61	3.86	4.51	4.07	4.13	0.53	4.15	0.33	1.00	0.007	9.6E-01

Mtr.30098.1.S1_at	5.52	4.65	5.59	5.05	5.90	5.55	5.25	0.53	5.50	0.43	1.05	0.067	5.6E-01
Mtr.42080.1.S1_at	6.41	5.48	6.36	6.68	6.68	6.78	6.08	0.53	6.72	0.06	1.10	0.143	1.1E-01
Mtr.39793.1.S1_s_at	7.31	6.41	7.33	7.02	7.40	7.72	7.02	0.53	7.38	0.35	1.05	0.072	3.8E-01
Mtr.10696.1.S1_at	10.61	9.57	9.93	10.11	10.48	10.46	10.04	0.53	10.35	0.21	1.03	0.044	3.9E-01
Mtr.5421.1.S1_s_at	9.78	8.82	9.68	9.70	9.77	9.77	9.43	0.53	9.75	0.04	1.03	0.049	3.5E-01
Mtr.41194.1.S1_at	8.08	7.06	7.78	8.15	7.94	8.17	7.64	0.53	8.09	0.13	1.06	0.082	2.3E-01
Mtr.2055.1.S1_at	8.79	7.92	8.86	8.96	9.08	9.18	8.52	0.53	9.07	0.11	1.06	0.090	1.5E-01
Mtr.43748.1.S1_s_at	7.06	6.03	6.74	6.52	6.44	6.57	6.61	0.53	6.51	0.07	0.99	-0.021	7.7E-01
Mtr.32345.1.S1_at	6.67	6.11	7.16	7.01	7.24	7.39	6.64	0.53	7.21	0.19	1.09	0.119	1.5E-01
Mtr.13702.1.S1_at	10.16	9.14	9.88	9.95	9.92	9.92	9.73	0.53	9.93	0.02	1.02	0.030	5.4E-01
Mtr.16110.1.S1_at	4.05	5.10	4.56	4.55	4.67	4.39	4.57	0.53	4.54	0.14	0.99	-0.010	9.2E-01
Mtr.37355.1.S1_at	12.38	11.47	12.39	12.22	12.09	12.09	12.08	0.53	12.13	0.07	1.00	0.006	8.8E-01
Mtr.47077.1.S1_at	9.34	8.84	9.89	9.49	9.55	9.37	9.36	0.53	9.47	0.09	1.01	0.017	7.3E-01
Mtr.42200.1.S1_s_at	9.09	8.06	8.78	8.83	8.74	8.76	8.65	0.53	8.78	0.04	1.02	0.022	6.9E-01
Mtr.33819.1.S1_at	8.70	8.03	9.06	8.75	8.69	8.57	8.60	0.53	8.67	0.09	1.01	0.012	8.2E-01
Mtr.35327.1.S1_at	7.13	6.27	7.23	7.11	7.29	7.29	6.87	0.53	7.23	0.11	1.05	0.073	3.1E-01
Mtr.27983.1.S1_at	5.71	4.84	4.76	4.69	4.64	4.66	5.10	0.53	4.66	0.02	0.91	-0.129	2.3E-01
Mtr.38001.1.S1_at	10.04	11.08	10.50	10.08	10.19	10.45	10.54	0.53	10.24	0.19	0.97	-0.042	4.0E-01
Mtr.15528.1.S1_x_at	3.09	3.98	3.05	3.24	2.97	2.95	3.37	0.53	3.05	0.16	0.91	-0.143	3.7E-01
Mtr.40229.1.S1_at	6.63	6.29	7.32	6.42	7.02	6.66	6.75	0.53	6.70	0.30	0.99	-0.009	9.1E-01
Mtr.6867.1.S1_at	9.56	8.63	9.52	9.53	9.24	9.40	9.24	0.53	9.39	0.15	1.02	0.024	6.5E-01
Mtr.11265.1.S1_at	4.88	4.73	5.71	4.87	4.92	5.47	5.11	0.53	5.09	0.33	1.00	-0.005	9.6E-01
Mtr.20803.1.S1_at	9.75	8.86	8.81	9.23	9.12	9.25	9.14	0.53	9.20	0.07	1.01	0.009	8.6E-01
Mtr.13261.1.S1_s_at	5.20	4.20	4.98	4.87	4.98	5.19	4.80	0.53	5.01	0.16	1.05	0.064	5.3E-01
Mtr.4971.1.S1_at	6.29	5.25	5.90	6.34	7.02	6.18	5.81	0.53	6.52	0.45	1.12	0.164	1.5E-01
Mtr.17489.1.S1_at	14.07	13.02	13.52	13.38	13.04	13.44	13.54	0.53	13.28	0.22	0.98	-0.027	4.8E-01
Mtr.28152.1.S1_at	4.91	3.90	4.66	4.90	5.03	4.61	4.49	0.52	4.85	0.21	1.08	0.110	3.4E-01
Mtr.21812.1.S1_at	11.23	10.20	10.53	11.04	10.50	11.02	10.65	0.52	10.85	0.31	1.02	0.027	6.0E-01
Mtr.46881.1.S1_at	9.22	8.36	9.31	8.91	9.09	9.12	8.96	0.52	9.04	0.11	1.01	0.012	8.2E-01
Mtr.5328.1.S1_at	4.74	3.86	4.80	4.72	4.72	4.70	4.46	0.52	4.71	0.01	1.06	0.078	4.6E-01
Mtr.5955.1.S1_at	9.90	9.11	8.90	9.87	10.20	10.32	9.30	0.52	10.13	0.23	1.09	0.123	6.7E-02
Mtr.35146.1.S1_at	5.68	4.66	5.41	5.90	5.19	5.45	5.25	0.52	5.52	0.36	1.05	0.071	5.1E-01
Mtr.30167.1.S1_at	2.94	3.96	3.68	3.94	4.59	4.63	3.53	0.52	4.39	0.39	1.24	0.316	8.3E-02
Mtr.33397.1.S1_at	7.92	8.38	7.34	7.88	8.09	8.08	7.88	0.52	8.02	0.12	1.02	0.024	6.9E-01
Mtr.32555.1.S1_s_at	7.96	6.96	7.19	7.46	7.60	7.38	7.37	0.52	7.48	0.11	1.02	0.022	7.4E-01
Mtr.49328.1.S1_at	2.68	3.58	2.67	10.07	9.93	10.10	2.97	0.52	10.04	0.09	3.38	1.755	2.1E-05
Mtr.33103.1.S1_at	5.06	4.01	4.49	4.34	4.05	4.54	4.52	0.52	4.31	0.25	0.95	-0.070	5.6E-01
Mtr.11340.1.S1_at	11.38	10.33	10.81	10.90	10.94	11.07	10.84	0.52	10.97	0.09	1.01	0.018	6.9E-01
Mtr.16824.1.S1_at	9.10	8.10	8.86	8.96	9.07	8.93	8.69	0.52	8.99	0.08	1.03	0.049	3.9E-01
Mtr.6613.1.S1_at	6.89	7.62	7.91	7.66	7.73	7.26	7.47	0.52	7.55	0.25	1.01	0.015	8.3E-01
Mtr.31697.1.S1_at	7.88	8.13	8.88	8.31	8.17	8.18	8.30	0.52	8.22	0.08	0.99	-0.014	8.1E-01
Mtr.39045.1.S1_at	6.98	6.76	7.76	7.42	7.43	7.00	7.17	0.52	7.28	0.24	1.02	0.023	7.5E-01
Mtr.38494.1.S1_at	8.05	7.01	7.66	7.69	8.00	7.64	7.57	0.52	7.78	0.19	1.03	0.039	5.6E-01
Mtr.32147.1.S1_at	2.42	3.29	2.35	2.34	2.34	2.34	2.69	0.52	2.34	0.00	0.87	-0.201	3.1E-01
Mtr.38775.1.S1_at	4.05	5.09	4.48	4.80	5.39	4.79	4.54	0.52	4.99	0.34	1.10	0.137	2.8E-01
Mtr.19934.1.S1_at	14.49	13.92	13.45	14.08	13.86	14.11	13.96	0.52	14.02	0.14	1.00	0.007	8.5E-01
Mtr.11358.1.S1_at	8.98	8.02	8.87	8.73	8.71	8.37	8.63	0.52	8.60	0.20	1.00	-0.004	9.4E-01
Mtr.35209.1.S1_at	5.99	6.76	7.00	6.10	6.61	5.04	6.58	0.52	5.91	0.80	0.90	-0.154	2.9E-01
Mtr.51005.1.S1_at	9.69	9.09	10.13	9.49	9.80	9.44	9.64	0.52	9.58	0.19	0.99	-0.009	8.6E-01
Mtr.11690.1.S1_at	7.76	6.77	7.56	7.97	7.94	8.13	7.36	0.52	8.01	0.10	1.09	0.122	1.0E-01
Mtr.34276.1.S1_at	8.74	8.13	9.17	8.91	8.84	8.85	8.68	0.52	8.87	0.03	1.02	0.030	5.8E-01
Mtr.16851.1.S1_at	8.07	7.13	8.00	7.78	8.16	8.16	7.73	0.52	8.03	0.22	1.04	0.055	4.1E-01
Mtr.35771.1.S1_at	4.27	3.42	3.31	3.33	3.27	3.26	3.67	0.52	3.29	0.04	0.90	-0.158	2.8E-01
Mtr.36964.1.S1_at	11.03	9.99	10.48	10.63	10.78	10.40	10.50	0.52	10.60	0.19	1.01	0.014	7.7E-01
Mtr.19170.1.S1_at	8.69	9.24	9.73	9.13	9.09	8.97	9.22	0.52	9.06	0.08	0.98	-0.025	6.3E-01
Mtr.360.1.S1_at	3.58	2.70	3.62	3.94	3.19	3.53	3.30	0.52	3.55	0.38	1.08	0.107	5.3E-01
Mtr.29706.1.S1_at	9.25	9.16	10.11	9.67	9.90	9.53	9.51	0.52	9.70	0.19	1.02	0.029	5.8E-01
Mtr.32934.1.S1_s_at	8.10	7.30	8.29	7.96	8.08	8.07	7.90	0.52	8.04	0.07	1.02	0.025	6.7E-01
Mtr.10043.1.S1_at	2.95	2.63	3.65	3.10	3.58	3.56	3.08	0.52	3.41	0.27	1.11	0.149	3.8E-01
Mtr.17513.1.S1_at	10.79	9.76	10.13	10.37	10.50	10.50	10.23	0.52	10.46	0.07	1.02	0.032	4.9E-01
Mtr.43806.1.S1_at	9.19	8.37	9.34	8.77	8.82	8.37	8.97	0.52	8.65	0.25	0.97	-0.051	4.0E-01
Mtr.17823.1.S1_at	5.96	5.31	6.34	5.80	6.22	5.70	5.87	0.52	5.91	0.28	1.01	0.009	9.2E-01
Mtr.47988.1.S1_at	8.91	8.26	9.30	9.12	9.41	9.08	8.82	0.52	9.21	0.18	1.04	0.061	3.0E-01
Mtr.12597.1.S1_at	7.67	8.02	8.70	9.76	10.26	10.15	8.13	0.52	10.06	0.26	1.24	0.307	4.6E-03
Mtr.37467.1.S1_at	8.09	7.10	7.89	8.07	8.71	8.21	7.69	0.52	8.33	0.34	1.08	0.114	1.5E-01
Mtr.20922.1.S1_at	4.66	3.63	3.98	3.50	3.58	4.04	4.09	0.52	3.71	0.29	0.91	-0.141	3.3E-01
Mtr.13256.1.S1_at	7.01	6.12	6.08	6.08	6.08	6.08	6.40	0.52	6.08	0.00	0.95	-0.075	3.4E-01
Mtr.43598.1.S1_at	6.94	6.70	7.70	7.56	7.50	7.21	7.11	0.52	7.42	0.18	1.04	0.063	3.8E-01
Mtr.5393.1.S1_at	7.21	6.17	6.81	6.77	6.82	6.81	6.73	0.52	6.80	0.03	1.01	0.015	8.2E-01
Mtr.24299.1.S1_s_at	4.60	5.21	4.17	4.58	4.77	4.60	4.66	0.52	4.65	0.10	1.00	-0.003	9.7E-01
Mtr.11467.1.S1_at	8.78	7.74	8.35	8.15	8.29	8.15	8.29	0.52	8.19	0.08	0.99	-0.017	7.7E-01
Mtr.35676.1.S1_at	8.70	8.75	9.63	8.95	9.02	8.80	9.02	0.52	8.92	0.11	0.99	-0.016	7.6E-01
Mtr.40290.1.S1_at	4.86	4.02	3.90	3.19	4.58	3.98	4.26	0.52	3.91	0.70	0.92	-0.122	5.3E-01
Mtr.16693.1.S1_at	9.23	9.74	10.28	11.34	11.80	11.89	9.75	0.52	11.68	0.30	1.20	0.260	5.1E-03
Mtr.28792.1.S1_at	7.34	7.34	8.24	7.83	8.01	7.80	7.64	0.52	7.88	0.11	1.03	0.045	4.8E-01
Mtr.38642.1.S1_at	9.91	8.97	9.83	9.62	9.77	9.57	9.57	0.52	9.65	0.11	1.01	0.012	8.0E-01
Mtr.37330.1.S1_at	7.95	7.78	6.97	7.69	7.23	7.33	7.57	0.52	7.42	0.24	0.98	-0.028	6.8E-01
Mtr.11981.1.S1_at	7.98	7.20	8.20	7.98	8.05	7.78	7.79	0.52	7.94	0.14	1.02	0.026	6.7E-01
Mtr.42424.1.S1_at	7.28	6.38	7.30	7.31	7.71	7.29	6.99	0.52	7.44	0.24	1.06	0.090	2.5E-01
Mtr.10238.1.S1_at	8.82	7.91	8.81	8.49	8.65	8.48	8.51	0.52	8.54	0.10	1.00	0.004	9.4E-01
Mtr.2529.1.S1_at	2.49	3.38	2.46	2.44	2.45	2.41	2.78	0.52	2.43	0.02	0.88	-0.191	3.2E-01
Mtr.44663.1.S1_s_at	8.88	8.07	9.04	9.11	8.77	8.91	8.66	0.52	8.93	0.17	1.03	0.044	4.5E-01
Mtr.8818.1.S1_at	10.69	9.68	10.42	10.44	10.40	10.42	10.27	0.52	10.42	0.02	1.02	0.022	6.4E-01
Mtr.24391.1.S1_at	11.82	10.81	11.54	11.41	11.67	11.64	11.39	0.52	11.57	0.15	1.02	0.023	5.9E-01
Mtr.22499.1.S1_s_at	3.00	3.00	3.90	3.61	3.19	3.57	3.30	0.52	3.46	0.23	1.05	0.067	6.6E-01
Mtr.9043.1.S1_at	11.04	10.15	10.11	10.00	9.96	9.84	10.43	0.52	9.93	0.08	0.95	-0.071	1.8E-01
Mtr.10314.1.S1_at	11.64	11.54	12.49	12.24	12.22	11.93	11.89	0.52	12.13	0.17	1.02	0.029	5.0E-01
Mtr.51054.1.S1_at	9.36	8.34	8.66	8.74	8.77	8.60	8.79	0.52	8.71	0.09	0.99	-0.013	8.1E-01
Mtr.26962.1.S1_at	5.08	4.58	5.62	5.23	5.19	4.95	5.10	0.52	5.12	0.15	1.01	0.008	9.3E-01
Mtr.46559.1.S1_at	2.64	3.53	2.61	2.60	2.58	2.69	2.92	0.52	2.62	0.06	0.90	-0.157	3.8E-01
Mtr.35363.1.S1_at	9.42	8.44	9.25	9.11	9.46	9.22	9.04	0.52	9.26	0.18	1.02	0.035	5.2E-01
Mtr.18891.1.S1_at	9.32	8.28	8.84	9.20	9.07	9.03	8.81	0.52	9.10	0.09	1.03	0.046	4.0E-01
Mtr.587.1.S1_at	4.02	3.11	4.01	5.24	5.83	5.55	3.72	0.52	5.54	0.29	1.49	0.576	6.2E-03
Mtr.45603.1.S1_s_at	9.02	9.37	10.05	9.07	9.96	8.88	9.48	0.52	9.30	0.57	0.98	-0.027	7.2E-01
Mtr.7605.1.S1_at	10.05	9.01	9.64	9.33	9.38	9.29	9.57	0.52	9.33	0.04	0.98	-0.036	4.8E-01
Mtr.41958.1.S1_at	5.97	5.78	6.76	5.73	5.75	5.75	6.17	0.52	5.74	0.01	0.93	-0.104	2.3E-01
Mtr.35843.1.S1_at	10.58	9.63	10.48	10.49	10.67	10.44	10.23	0.52	10.53	0.12	1.03	0.041	3.9E-01
Mtr.43127.1.S1_at	8.03	7.06	7.87	8.44	8.95	8.80	7.65	0.52	8.73	0.26	1.14	0.190	3.3E-02
Mtr.33756.1.S1_at	6.94	6.03	6.93	7.13	7.16	7.15	6.64	0.52	7.15	0.02	1.08	0.107	1.7E-01
Mtr.11055.1.S1_at	9.23	8.19	8.79	8.16	7.78	7.98	8.73	0.52	7.97	0.19	0.91	-0.132	7.6E-02
Mtr.50630.1.S1_x_at	2.95	2.08	2.02	5.26	5.39	4.61	2.35	0.52	5.09	0.42	2.16	1.113	2.1E-03
Mtr.41810.1.S1_at	7.09	6.19	7.09	6.27	6.27	6.10	6.79	0.52	6.21	0.10	0.91	-0.128	1.3E-01
Mtr.13346.1.S1_at	7.64	6.69	7.52	7.61	7.31	7.64	7.28	0.52	7.52	0.18	1.03	0.047	5.0E-01
Mtr.33834.1.S1_at	3.82	3.01	3.98	3.81	3.81	3.80	3.60	0.52	3.81	0.00	1.06	0.080	5.3E-01
Mtr.27196.1.S1_at	9.78	8.90	9.82	9.78	9.84	9.73	9.50	0.52	9.78	0.06	1.03	0.042	4.1E-01
Mtr.42016.1.S1_s_at	5.52	4.55	5.36	5.28	5.52	5.19	5.14	0.52	5.33	0.17	1.04	0.052	5.8E-01
Mtr.51687.1.S1_at	9.38	9.99	10.42	10.04	10.08	9.92	9.93	0.52	10.01	0.08	1.01	0.012	8.0E-01
Mtr.8315.1.S1_at	2.29	3.19	2.28	2.28	2.28	2.27	2.59	0.52	2.28	0.00	0.88	-0.185	3.6E-01
Mtr.4922.1.S1_at	2.17	3.06	2.16	2.15	2.15	2.14	2.46	0.52	2.15	0.00	0.87	-0.197	3.5E-01
Mtr.45791.1.S1_at	2.53	3.43	2.52	2.50	2.49	2.51	2.83	0.52	2.50	0.01	0.88	-0.177	3.4E-01
Mtr.35477.1.S1_at	5.54	4.50	5.01	4.30	5.20	4.85	5.01	0.52	4.78	0.45	0.95	-0.068	5.9E-01
Mtr.27410.1.S1_at	4.97	4.31	5.34	4.95	4.96	5.00	4.88	0.52	4.97	0.03	1.02	0.027	7.7E-01
Mtr.19267.1.S1_at	10.55	10.28	11.28	10.65	10.47	10.48	10.70	0.52	10.54	0.10	0.98	-0.023	6.1E-01
Mtr.52132.1.S1_x_at	3.39	2.35	2.91	2.93	3.05	3.00	2.88	0.52	2.99	0.06	1.04	0.053	7.4E-01
Mtr.8701.1.S1_at	11.41	12.07	12.44	12.04	12.85	12.56	11.98	0.52	12.48	0.41	1.04	0.060	2.6E-01
Mtr.35603.1.S1_x_at	3.48	2.46	3.17	3.85	3.60	4.01	3.04	0.52	3.82	0.21	1.26	0.329	7.3E-02
Mtr.31511.1.S1_at	7.86	6.83	7.49	7.55	7.49	7.44	7.39	0.52	7.49	0.06	1.01	0.020	7.6E-01
Mtr.17896.1.S1_at	6.25	5.61	6.64	5.54	5.88	5.88	6.17	0.52	5.77	0.19	0.93	-0.097	2.8E-01
Mtr.4077.1.S1_at	9.11	9.31	10.10	10.10	10.40	9.89	9.51	0.52	10.13	0.25	1.07	0.091	1.4E-01
Mtr.650.1.S1_s_at	7.54	8.21	8.57	8.47	8.32	7.82	8.11	0.52	8.20	0.34	1.01	0.017	8.0E-01
Mtr.558.1.S1_at	9.60	8.64	9.45	9.65	9.44	9.45	9.23	0.52	9.51	0.12	1.03	0.043	4.1E-01
Mtr.30409.1.S1_at	7.72	6.97	7.97	8.00	8.01	7.82	7.55	0.52	7.94	0.11	1.05	0.072	2.7E-01
Mtr.45595.1.S1_s_at	8.87	8.76	9.71	9.06	9.51	8.99	9.11	0.52	9.19	0.28	1.01	0.012	8.4E-01
Mtr.28563.1.S1_at	7.81	6.98	6.85	6.77	7.25	7.45	7.21	0.52	7.16	0.35	0.99	-0.012	8.8E-01
Mtr.42794.1.S1_at	9.23	8.41	9.38	9.15	9.06	8.82	9.00	0.52	9.01	0.17	1.00	0.001	9.8E-01
Mtr.28765.1.S1_at	5.33	5.05	6.06	5.64	5.70	5.64	5.48	0.52	5.66	0.03	1.03	0.046	5.8E-01
Mtr.14336.1.S1_at	9.51	9.49	10.40	9.90	10.11	10.19	9.80	0.52	10.07	0.15	1.03	0.039	4.4E-01
Mtr.20121.1.S1_s_at	9.12	8.30	9.26	9.13	9.23	9.13	8.89	0.52	9.16	0.06	1.03	0.044	4.2E-01
Mtr.14319.1.S1_at	11.01	11.48	12.05	11.34	11.40	11.43	11.51	0.52	11.39	0.05	0.99	-0.015	7.2E-01
Mtr.8795.1.S1_at	11.83	10.91	11.79	11.77	11.91	11.75	11.51	0.52	11.81	0.09	1.03	0.037	3.8E-01
Mtr.7165.1.S1_at	3.27	2.35	3.23	3.55	3.24	3.23	2.95	0.52	3.34	0.18	1.13	0.178	2.9E-01

Mtr.33067.1.S1_at	3.46	3.66	4.45	3.51	3.46	3.40	3.86	0.52	3.45	0.05	0.90	-0.159	2.5E-01
Mtr.51994.1.S1_at	2.94	3.81	2.89	3.01	2.92	2.85	3.21	0.52	2.93	0.08	0.91	-0.135	4.0E-01
Mtr.31328.1.S1 at	10.63	9.98	11.01	11.35	11.37	11.18	10.54	0.52	11.30	0.10	1.07	0.100	6.9E-02
Mtr.35187.1.S1 at	3.07	2.16	3.06	3.19	3.10	3.06	2.76	0.52	3.12	0.07	1.13	0.174	3.1E-01
Mtr.14902.1.S1 at	9.26	8.36	9.26	9.28	9.29	9.34	8.96	0.52	9.30	0.03	1.04	0.054	3.2E-01
Mtr.38207.1.S1_at	11.12	10.12	10.89	10.88	10.89	10.86	10.71	0.52	10.88	0.01	1.02	0.022	6.1E-01
Mtr.47408.1.S1_at	2.93	3.79	2.86	2.88	2.76	2.81	3.19	0.52	2.82	0.06	0.88	-0.181	2.8E-01
Mtr.42825.1.S1 s at	7.09	7.50	8.13	7.12	7.45	7.20	7.57	0.52	7.26	0.17	0.96	-0.062	3.7E-01
Mtr.29288.1.S1 x at	7.28	6.33	6.44	7.18	7.87	7.59	6.69	0.52	7.55	0.35	1.13	0.174	7.6E-02
Mtr.8884.1.S1 at	13.75	12.73	13.38	13.80	13.42	13.69	13.29	0.52	13.64	0.19	1.03	0.038	3.3E-01
Mtr.41203.1.S1 at	10.47	9.57	10.46	10.29	10.44	10.33	10.17	0.52	10.35	0.07	1.02	0.026	5.7E-01
Mtr.9742.1.S1_at	5.94	5.07	6.00	6.08	5.95	5.89	5.67	0.52	5.97	0.10	1.05	0.075	3.8E-01
Mtr.20335.1.S1_at	6.29	5.29	5.56	5.73	5.68	5.67	5.71	0.52	5.69	0.04	1.00	-0.005	9.5E-01
Mtr.34933.1.S1_at	3.89	3.90	4.79	4.71	4.83	4.76	4.19	0.52	4.77	0.06	1.14	0.185	1.3E-01
Mtr.13744.1.S1 at	7.59	6.70	7.60	7.19	7.54	7.01	7.29	0.52	7.25	0.27	0.99	-0.009	9.0E-01
Mtr.35882.1.S1 at	8.75	7.92	8.88	8.82	8.85	8.63	8.52	0.52	8.77	0.12	1.03	0.042	4.6E-01
Mtr.6143.1.S1 at	4.09	3.15	4.00	4.01	4.07	4.08	3.75	0.52	4.05	0.04	1.08	0.113	3.7E-01
Mtr.50620.1.S1_at	8.82	8.28	9.31	8.88	8.93	8.60	8.80	0.52	8.80	0.18	1.00	0.000	1.0E+00
Mtr.10951.1.S1_at	11.57	10.54	11.11	11.34	11.28	11.37	11.07	0.52	11.33	0.04	1.02	0.033	4.4E-01
Mtr.13947.1.S1_at	8.11	7.08	7.45	7.87	7.52	7.87	7.55	0.52	7.75	0.20	1.03	0.039	5.6E-01
Mtr.47483.1.S1 s at	9.54	8.76	9.75	9.40	9.64	9.30	9.35	0.52	9.45	0.17	1.01	0.015	7.8E-01
Mtr.34738.1.S1 s at	10.06	9.48	10.51	10.29	9.98	10.25	10.02	0.52	10.17	0.17	1.02	0.022	6.5E-01
Mtr.6964.1.S1 at	8.68	7.68	8.41	8.57	8.32	8.44	8.26	0.52	8.45	0.13	1.02	0.032	5.8E-01
Mtr.8617.1.S1_at	11.72	10.71	11.42	11.08	11.42	11.28	11.28	0.52	11.26	0.17	1.00	-0.003	9.4E-01
Mtr.40756.1.S1_at	10.91	9.87	10.46	10.77	10.66	10.69	10.42	0.52	10.71	0.06	1.03	0.040	3.9E-01
Mtr.28958.1.S1_at	6.93	6.18	7.18	7.22	7.52	7.16	6.76	0.52	7.30	0.19	1.08	0.111	1.7E-01
Mtr.8996.1.S1 at	7.99	8.32	9.01	8.47	8.53	8.36	8.44	0.52	8.45	0.09	1.00	0.002	9.7E-01
Mtr.40228.1.S1 at	11.98	10.99	11.75	11.78	11.53	11.64	11.58	0.52	11.65	0.13	1.01	0.009	8.2E-01
Mtr.617.1.S1 at	3.27	3.72	2.69	2.66	2.64	2.62	3.22	0.52	2.64	0.02	0.82	-0.288	1.2E-01
Mtr.13421.1.S1_at	9.48	9.11	10.14	9.70	9.63	9.41	9.58	0.52	9.58	0.15	1.00	0.000	9.9E-01
Mtr.28075.1.S1_at	7.04	6.09	6.94	6.87	7.00	6.72	6.69	0.52	6.87	0.14	1.03	0.037	6.1E-01
Mtr.31581.1.S1_at	8.68	7.95	8.96	8.75	9.06	8.66	8.53	0.52	8.82	0.21	1.03	0.049	4.2E-01
Mtr.38655.1.S1 at	9.61	8.93	9.95	9.58	9.62	9.60	9.50	0.52	9.60	0.02	1.01	0.015	7.5E-01
Mtr.28275.1.S1 s at	8.76	7.89	7.84	7.96	8.73	8.25	8.16	0.52	8.31	0.39	1.02	0.026	7.1E-01
Mtr.26556.1.S1 at	8.08	7.40	8.42	8.51	8.98	8.43	7.97	0.52	8.64	0.30	1.08	0.116	1.2E-01
Mtr.38537.1.S1 at	11.02	10.24	10.04	10.04	9.98	10.02	10.43	0.52	10.01	0.03	0.96	-0.059	2.3E-01
Mtr.40166.1.S1_s_at	11.49	10.48	10.80	11.18	10.93	11.42	10.93	0.52	11.18	0.25	1.02	0.033	4.9E-01
Mtr.13102.1.S1 at	7.68	6.75	7.61	7.74	7.72	7.45	7.35	0.52	7.64	0.16	1.04	0.056	4.0E-01
Mtr.41831.1.S1 at	8.29	7.36	8.21	8.35	8.21	8.39	7.95	0.52	8.32	0.09	1.05	0.065	3.0E-01
Mtr.9953.1.S1 at	3.66	3.11	4.14	3.69	4.62	4.05	3.64	0.52	4.12	0.47	1.13	0.181	3.0E-01
Mtr.27947.1.S1 at	8.81	8.56	9.55	8.92	8.88	8.75	8.97	0.52	8.85	0.09	0.99	-0.020	7.0E-01
Mtr.49791.1.S1_x_at	2.87	3.75	2.84	2.82	2.83	2.77	3.16	0.52	2.80	0.03	0.89	-0.170	3.1E-01
Mtr.49516.1.S1_at	11.75	10.82	11.68	11.44	11.53	11.16	11.41	0.52	11.38	0.19	1.00	-0.005	9.2E-01
Mtr.7487.1.S1 s at	4.70	3.68	4.06	4.46	4.71	4.39	4.15	0.52	4.52	0.17	1.09	0.124	3.0E-01
Mtr.8427.1.S1 at	3.05	3.36	2.35	2.33	2.87	2.32	2.92	0.52	2.51	0.31	0.86	-0.217	3.1E-01
Mtr.30266.1.S1 at	6.66	6.17	7.20	6.52	7.55	6.81	6.68	0.52	6.96	0.53	1.04	0.060	5.4E-01
Mtr.871.1.S1 at	3.30	4.32	3.66	3.64	3.21	3.89	3.76	0.52	3.58	0.34	0.95	-0.070	6.4E-01
Mtr.6910.1.S1_at	8.20	7.44	8.43	8.07	8.07	8.15	8.02	0.52	8.10	0.04	1.01	0.013	8.2E-01
Mtr.9323.1.S1_at	6.62	6.17	7.20	6.76	6.62	6.60	6.66	0.52	6.66	0.09	1.00	-0.001	9.9E-01
Mtr.2334.1.S1 at	5.39	4.36	4.84	5.43	5.24	4.76	4.86	0.52	5.14	0.34	1.06	0.081	4.8E-01
Mtr.27640.1.S1 at	9.14	8.38	9.37	9.32	9.21	9.02	8.96	0.52	9.18	0.15	1.02	0.035	5.2E-01
Mtr.21046.1.S1 at	4.42	3.39	4.02	4.28	4.24	3.98	3.94	0.52	4.17	0.16	1.06	0.080	5.1E-01
Mtr.40619.1.S1 at	6.51	6.06	7.09	7.48	7.96	7.55	6.55	0.52	7.66	0.26	1.17	0.226	2.9E-02
Mtr.31660.1.S1_at	7.80	6.82	7.02	7.27	6.67	7.32	7.21	0.52	7.09	0.36	0.98	-0.026	7.5E-01
Mtr.35778.1.S1_at	2.14	3.03	2.12	2.04	2.12	2.12	2.43	0.52	2.10	0.05	0.86	-0.213	3.3E-01
Mtr.34947.1.S1_at	6.88	5.85	6.29	6.35	6.47	6.33	6.34	0.52	6.38	0.08	1.01	0.010	8.9E-01
Mtr.33581.1.S1 at	7.80	7.03	8.02	7.93	7.86	7.84	7.62	0.52	7.88	0.04	1.03	0.048	4.4E-01
Mtr.11101.1.S1 at	10.02	11.04	10.65	10.08	10.97	10.48	10.57	0.52	10.51	0.44	0.99	-0.008	8.9E-01
Mtr.41929.1.S1 at	6.78	5.81	6.59	6.54	6.44	6.54	6.39	0.52	6.51	0.06	1.02	0.026	7.2E-01
Mtr.4623.1.S1_at	10.66	9.66	10.39	10.43	10.56	10.40	10.24	0.52	10.46	0.09	1.02	0.031	5.0E-01
Mtr.29468.1.S1_at	5.52	4.80	5.81	5.77	6.35	6.10	5.38	0.52	6.07	0.30	1.13	0.175	1.1E-01
Mtr.9210.1.S1_at	8.11	7.44	8.46	8.33	8.40	8.16	8.00	0.52	8.30	0.13	1.04	0.053	3.9E-01
Mtr.8217.1.S1 at	8.52	7.90	8.92	8.41	8.69	8.77	8.45	0.52	8.62	0.19	1.02	0.030	6.1E-01
Mtr.9085.1.S1 at	11.11	10.08	10.48	10.81	10.72	10.75	10.55	0.52	10.76	0.05	1.02	0.028	5.3E-01
Mtr.23100.1.S1 at	2.05	2.94	2.04	2.03	2.03	2.04	2.34	0.52	2.03	0.00	0.87	-0.203	3.6E-01
Mtr.20669.1.S1_at	7.72	6.73	6.96	7.40	7.11	7.36	7.14	0.52	7.29	0.16	1.02	0.031	6.5E-01
Mtr.28547.1.S1_s_at	12.53	13.48	13.37	13.02	13.34	13.27	13.13	0.52	13.21	0.17	1.01	0.009	8.1E-01
Mtr.9054.1.S1_at	10.96	11.65	11.97	11.42	11.93	11.73	11.53	0.52	11.69	0.26	1.01	0.020	6.5E-01
Mtr.15968.1.S1 at	8.55	7.68	8.60	8.49	8.29	8.28	8.28	0.52	8.35	0.12	1.01	0.013	8.2E-01
Mtr.10394.1.S1 at	11.67	10.85	10.71	10.75	10.61	10.76	11.07	0.52	10.70	0.08	0.97	-0.049	2.9E-01
Mtr.8460.1.S1 at	6.24	5.23	5.57	6.44	7.12	6.81	5.68	0.52	6.79	0.34	1.20	0.258	3.5E-02
Mtr.19997.1.S1_at	2.18	3.07	2.17	2.16	2.16	2.15	2.47	0.52	2.16	0.00	0.87	-0.194	3.6E-01
Mtr.26867.1.S1_s_at	6.61	5.58	6.04	6.92	7.47	7.34	6.08	0.52	7.24	0.29	1.19	0.253	2.7E-02
Mtr.38535.1.S1 s at	7.79	7.66	8.61	8.05	7.93	7.99	8.02	0.52	7.99	0.06	1.00	-0.006	9.2E-01
Mtr.13279.1.S1 at	9.41	8.38	8.95	9.34	9.38	9.45	8.91	0.52	9.39	0.06	1.05	0.075	1.9E-01
Mtr.2863.1.S1 at	5.62	4.74	5.64	5.29	5.42	5.29	5.34	0.52	5.33	0.08	1.00	-0.001	9.9E-01
Mtr.25160.1.S1 at	4.08	3.19	4.08	4.33	4.36	4.14	3.78	0.52	4.28	0.12	1.13	0.178	1.8E-01
Mtr.26965.1.S1_at	9.72	8.75	8.91	8.82	9.39	9.15	9.13	0.52	9.12	0.29	1.00	-0.001	9.9E-01
Mtr.28079.1.S1_at	3.36	3.78	4.39	3.79	4.44	3.92	3.84	0.52	4.05	0.34	1.05	0.077	5.9E-01
Mtr.2644.1.S1_at	5.95	6.51	6.98	6.54	6.50	6.53	6.48	0.52	6.52	0.02	1.01	0.009	9.0E-01
Mtr.44925.1.S1 at	11.59	10.59	11.30	11.44	11.68	11.52	11.16	0.52	11.55	0.12	1.03	0.049	2.8E-01
Mtr.37201.1.S1 at	8.96	7.93	8.46	8.70	8.71	8.73	8.45	0.52	8.71	0.02	1.03	0.044	4.3E-01
Mtr.30764.1.S1 at	10.81	9.80	10.50	10.60	10.65	10.70	10.37	0.52	10.65	0.05	1.03	0.038	4.0E-01
Mtr.43204.1.S1_at	9.69	8.70	9.45	10.07	10.35	10.09	9.28	0.52	10.17	0.16	1.10	0.132	4.6E-02
Mtr.11907.1.S1_at	7.81	7.28	8.31	8.02	8.27	8.22	7.80	0.52	8.17	0.13	1.05	0.066	3.0E-01
Mtr.4596.1.S1_at	6.98	6.02	6.83	6.85	6.83	6.80	6.61	0.52	6.83	0.03	1.03	0.047	5.1E-01
Mtr.8665.1.S1 at	11.22	10.37	11.30	10.99	11.04	10.89	10.96	0.52	10.97	0.08	1.00	0.001	9.8E-01
Mtr.26942.1.S1 at	7.78	7.61	8.58	8.08	7.95	7.61	7.99	0.52	7.88	0.24	0.99	-0.019	7.7E-01
Mtr.27058.1.S1 at	8.04	8.96	8.89	8.58	8.69	8.60	8.63	0.52	8.62	0.06	1.00	-0.001	9.8E-01
Mtr.6787.1.S1_at	5.58	5.86	4.86	5.69	6.32	6.19	5.43	0.52	6.07	0.34	1.12	0.160	1.5E-01
Mtr.39008.1.S1_s_at	8.98	7.97	8.32	8.85	8.96	9.10	8.43	0.52	8.97	0.12	1.06	0.090	1.5E-01
Mtr.24197.1.S1_at	2.44	3.32	2.42	2.40	2.38	2.39	2.73	0.52	2.39	0.01	0.88	-0.189	3.2E-01
Mtr.30710.1.S1 at	5.65	6.62	6.45	5.69	5.68	5.65	6.24	0.52	5.68	0.02	0.91	-0.137	1.3E-01
Mtr.25201.1.S1 at	2.96	3.81	2.89	2.82	2.91	3.28	3.22	0.52	3.00	0.24	0.93	-0.100	5.5E-01
Mtr.50990.1.S1 at	2.04	2.92	2.02	2.24	2.02	2.02	2.33	0.52	2.09	0.13	0.90	-0.155	4.8E-01
Mtr.31545.1.S1_at	5.37	6.25	5.35	5.33	5.35	5.36	5.66	0.52	5.35	0.01	0.94	-0.082	3.5E-01
Mtr.8517.1.S1_at	7.64	7.43	6.66	5.40	6.76	6.71	7.24	0.52	6.29	0.77	0.87	-0.203	1.5E-01
Mtr.13985.1.S1_at	4.87	4.99	5.82	5.21	5.06	4.48	5.23	0.51	4.92	0.38	0.94	-0.088	4.5E-01
Mtr.30628.1.S1 at	2.18	3.07	2.17	2.16	2.16	2.16	2.47	0.51	2.16	0.00	0.88	-0.192	3.6E-01
Mtr.50371.1.S1 s at	11.26	10.26	10.96	10.95	11.09	11.00	10.83	0.51	11.01	0.07	1.02	0.024	5.7E-01
Mtr.44777.1.S1 at	7.68	6.69	7.44	7.78	7.86	7.98	7.27	0.51	7.87	0.10	1.08	0.114	1.2E-01
Mtr.49069.1.S1 s at	7.30	8.30	8.00	7.40	7.59	7.43	7.87	0.51	7.47	0.10	0.95	-0.074	2.6E-01
Mtr.21568.1.S1_at	5.70	5.02	6.03	6.02	5.96	5.60	5.58	0.51	5.86	0.23	1.05	0.070	4.4E-01
Mtr.40685.1.S1 at	10.08	9.08	9.38	9.53	9.52	9.59	9.51	0.51	9.55	0.04	1.00	0.006	9.1E-01
Mtr.5949.1.S1 at	5.82	4.82	5.14	5.25	5.76	5.68	5.26	0.51	5.56	0.28	1.06	0.081	4.2E-01
Mtr.836.1.S1 at	6.70	6.68	7.58	6.43	7.31	6.62	6.99	0.51	6.79	0.46	0.97	-0.042	6.4E-01
Mtr.7689.1.S1 x at	2.87	3.74	2.83	2.82	2.82	2.81	3.15	0.51	2.82	0.01	0.89	-0.161	3.3E-01
Mtr.44318.1.S1_at	9.61	8.59	9.26	9.26	9.39	9.39	9.15	0.51	9.35	0.08	1.02	0.030	5.5E-01
Mtr.10808.1.S1_at	11.64	10.64	10.93	11.43	11.51	11.60	11.07	0.51	11.51	0.09	1.04	0.057	2.1E-01
Mtr.5243.1.S1 at	9.59	8.71	9.61	9.46	9.67	9.43	9.31	0.51	9.52	0.13	1.02	0.033	5.2E-01
Mtr.13534.1.S1 at	3.91	3.03	3.94	4.57	3.67	4.14	3.63	0.51	4.13	0.45	1.14	0.187	2.7E-01
Mtr.36942.1.S1 at	9.32	8.34	9.11	9.11	9.24	9.05	8.92	0.51	9.13	0.10	1.02	0.034	5.2E-01
Mtr.29154.1.S1 at	8.49	7.86	8.88	8.84	8.86	8.68	8.41	0.51	8.80	0.10	1.05	0.065	2.7E-01
Mtr.34428.1.S1_s_at	9.63	9.39	10.37	9.92	9.58	9.61	9.80	0.51	9.70	0.18	0.99	-0.014	7.8E-01
Mtr.29464.1.S1_at	8.62	7.59	8.05	8.69	8.51	8.61	8.08	0.51	8.60	0.09	1.06	0.089	1.6E-01
Mtr.1631.1.S1_s_at	7.34	6.89	7.92	7.79	7.74	7.64	7.38	0.51	7.73	0.07	1.05	0.065	3.2E-01
Mtr.24633.1.S1 at	8.95	8.90	9.81	9.30	9.18	9.19	9.22	0.51	9.22	0.07	1.00	0.000	1.0E+00
Mtr.39833.1.S1 at	9.28	8.33	9.16	9.08	9.20	9.14	8.92	0.51	9.14	0.06	1.02	0.035	5.1E-01
Mtr.26523.1.S1 at	3.08	3.27	4.05	3.06	3.06	3.06	3.47	0.51	3.06	0.00	0.88	-0.178	2.5E-01
Mtr.51000.1.S1_at	2.19	3.08	2.18	2.18	2.17	2.17	2.49	0.51	2.17	0.00	0.87	-0.193	3.5E-01
Mtr.47692.1.S1_at	6.27	5.87	6.89	6.72	6.28	6.26	6.34	0.51	6.42	0.26	1.01	0.017	8.3E-01
Mtr.44352.1.S1_at	9.49	8.48	9.14	9.37	9.38	9.43	9.04	0.51	9.39	0.04	1.04	0.055	3.0E-01
Mtr.16164.1.S1 at	9.97	9.06	9.92	9.92	9.92	9.92	9.65	0.51	9.92	0.00	1.03	0.040	4.1E-01
Mtr.42486.1.S1 at	9.69	9.50	10.47	10.06	10.38	10.08	9.89	0.51	10.17	0.18	1.03	0.041	4.1E-01
Mtr.41877.1.S1 at	9.47	9.18	10.18	9.66	9.89	9.62	9.61	0.51	9.72	0.14	1.01	0.017	7.3E-01
Mtr.30459.1.S1_at	3.44	4.11	4.45	3.41	3.80	3.83	4.00	0.51	3.68	0.23	0.92	-0.120	3.8E-01
Mtr.28330.1.S1_at	10.82	9.83	10.55	10.37	10.44	10.46	10.40	0.51	10.43	0.05	1.00	0.004	9.3E-01
Mtr.38923.1.S1_at	9.38	10.31	10.23	9.90	10.32	9.84	9.97	0.51	10.02	0.26	1.01	0.007	8.9E-01
Mtr.26883.1.S1 at	10.21	11.17	11.02	10.08	10.81	10.23	10.80	0.51	10.37	0.39	0.96	-0.059	3.1E-01
Mtr.41530.1.S1 s at	2.87	2.89	3.77	2.85	2.95	2.85	3.18	0.51	2.89	0.06	0.91	-0.138	3.9E-01
Mtr.44303.1.S1 s at	8.37	7.49	8.39	8.58	8.33	8.58	8.09	0.51	8.50	0.14	1.05	0.072	2.5E-01

Mtr.38487.1.S1_at	8.32	7.87	8.89	9.04	9.53	9.33	8.36	0.51	9.30	0.25	1.11	0.154	4.6E-02
Mtr.20180.1.S1_at	8.38	8.29	9.22	8.52	8.47	8.69	8.63	0.51	8.56	0.11	0.99	-0.011	8.3E-01
Mtr.19562.1.S1_at	3.94	3.13	2.99	2.96	2.92	2.77	3.35	0.51	2.89	0.10	0.86	-0.217	2.0E-01
Mtr.10068.1.S1_at	8.28	7.32	8.12	8.19	8.18	7.87	7.90	0.51	8.08	0.18	1.02	0.032	6.1E-01
Mtr.16193.1.S1_s_at	9.62	8.61	9.25	9.51	9.44	9.44	9.16	0.51	9.46	0.04	1.03	0.047	3.6E-01
Mtr.40346.1.S1_at	9.07	8.89	9.86	9.18	9.29	9.09	9.27	0.51	9.19	0.10	0.99	-0.013	7.9E-01
Mtr.3438.1.S1_at	8.83	7.83	8.52	8.36	8.42	8.33	8.40	0.51	8.37	0.05	1.00	-0.004	9.4E-01
Mtr.41472.1.S1_at	8.44	7.71	8.70	8.23	8.40	8.38	8.29	0.51	8.34	0.09	1.01	0.009	8.7E-01
Mtr.8133.1.S1_at	6.51	5.53	6.27	6.46	6.08	6.35	6.10	0.51	6.30	0.20	1.03	0.045	5.7E-01
Mtr.3480.1.S1_at	3.83	2.84	3.11	3.22	3.63	3.43	3.26	0.51	3.43	0.21	1.05	0.072	6.3E-01
Mtr.45484.1.S1_at	3.89	2.94	3.07	2.91	2.91	3.31	3.30	0.51	3.04	0.23	0.92	-0.117	4.7E-01
Mtr.13687.1.S1_at	7.81	7.05	8.02	7.57	7.93	7.69	7.63	0.51	7.73	0.18	1.01	0.020	7.6E-01
Mtr.46710.1.S1_at	2.29	3.17	2.28	2.27	2.27	2.28	2.58	0.51	2.27	0.00	0.88	-0.182	3.6E-01
Mtr.28620.1.S1_at	8.16	7.44	8.44	8.26	8.15	8.17	8.01	0.51	8.19	0.06	1.02	0.032	5.8E-01
Mtr.6373.1.S1_at	7.48	6.57	6.61	7.22	7.19	7.67	6.89	0.51	7.36	0.27	1.07	0.095	2.3E-01
Mtr.900.1.S1_at	6.72	5.80	6.65	7.01	7.11	6.65	6.39	0.51	6.92	0.24	1.08	0.115	1.8E-01
Mtr.14334.1.S1_at	10.55	10.35	11.32	10.86	10.91	10.56	10.74	0.51	10.78	0.19	1.00	0.005	9.2E-01
Mtr.35576.1.S1_at	7.58	6.68	7.56	7.24	7.86	7.60	7.27	0.51	7.57	0.31	1.04	0.057	4.5E-01
Mtr.19604.1.S1_at	2.66	3.54	2.65	2.65	2.60	2.62	2.95	0.51	2.62	0.02	0.89	-0.169	3.3E-01
Mtr.32632.1.S1_at	6.48	5.62	6.53	6.64	6.51	6.65	6.21	0.51	6.60	0.08	1.06	0.088	2.6E-01
Mtr.43051.1.S1_at	9.30	8.33	9.09	9.01	9.30	8.92	8.91	0.51	9.08	0.20	1.02	0.028	6.1E-01
Mtr.29840.1.S1_at	3.47	4.31	3.38	3.44	3.33	3.51	3.72	0.51	3.43	0.09	0.92	-0.117	3.9E-01
Mtr.41038.1.S1_at	9.74	8.73	9.05	9.57	9.48	9.77	9.17	0.51	9.60	0.15	1.05	0.066	2.3E-01
Mtr.48777.1.S1_at	3.13	2.28	2.22	2.21	2.21	2.21	2.54	0.51	2.21	0.00	0.87	-0.203	3.2E-01
Mtr.13229.1.S1_at	9.67	8.65	9.22	9.06	8.87	9.26	9.18	0.51	9.06	0.20	0.99	-0.019	7.3E-01
Mtr.42474.1.S1_at	8.00	7.21	8.17	7.83	7.91	7.87	7.79	0.51	7.87	0.04	1.01	0.014	8.2E-01
Mtr.33799.1.S1_at	6.46	5.91	6.94	6.15	6.54	6.65	6.44	0.51	6.45	0.26	1.00	0.002	9.8E-01
Mtr.11061.1.S1_at	8.91	7.91	8.62	8.94	8.83	8.87	8.48	0.51	8.88	0.05	1.05	0.066	2.5E-01
Mtr.20928.1.S1_at	9.36	10.36	10.05	9.58	9.77	9.57	9.93	0.51	9.64	0.11	0.97	-0.042	4.0E-01
Mtr.2093.1.S1_at	4.26	3.25	3.91	4.55	4.11	4.29	3.81	0.51	4.32	0.22	1.13	0.182	1.9E-01
Mtr.38599.1.S1_at	10.02	10.11	10.95	10.17	10.14	10.24	10.36	0.51	10.18	0.05	0.98	-0.025	5.8E-01
Mtr.48097.1.S1_at	8.61	7.84	8.81	8.76	8.91	8.88	8.42	0.51	8.85	0.08	1.05	0.071	2.3E-01
Mtr.49042.1.S1_at	3.65	2.74	2.79	3.16	2.69	2.88	3.06	0.51	2.91	0.24	0.95	-0.073	6.7E-01
Mtr.23569.1.S1_s_at	2.90	3.81	2.95	3.00	2.94	2.94	3.22	0.51	2.96	0.03	0.92	-0.119	4.4E-01
Mtr.12802.1.S1_at	9.72	8.79	8.89	9.20	9.05	9.13	9.13	0.51	9.13	0.07	1.00	-0.001	9.9E-01
Mtr.4188.1.S1_at	3.42	4.22	3.26	3.30	3.30	3.23	3.63	0.51	3.28	0.04	0.90	-0.147	3.0E-01
Mtr.28386.1.S1_at	2.46	3.34	2.45	2.44	2.39	2.44	2.75	0.51	2.42	0.03	0.88	-0.183	3.3E-01
Mtr.10507.1.S1_at	2.71	3.58	2.68	2.68	2.68	2.63	2.99	0.51	2.66	0.03	0.89	-0.167	3.3E-01
Mtr.1417.1.S1_x_at	2.86	3.81	3.67	3.10	2.78	2.83	3.45	0.51	2.90	0.17	0.84	-0.248	1.6E-01
Mtr.9614.1.S1_at	8.90	8.50	9.52	8.86	8.91	8.61	8.97	0.51	8.79	0.16	0.98	-0.029	5.9E-01
Mtr.32396.1.S1_at	6.06	5.58	6.60	6.09	6.20	5.78	6.08	0.51	6.03	0.22	0.99	-0.013	8.7E-01
Mtr.20719.1.S1_at	4.81	3.84	4.62	5.50	6.09	5.27	4.42	0.51	5.62	0.42	1.27	0.345	3.5E-02
Mtr.11249.1.S1_s_at	7.89	8.69	7.73	7.43	7.61	7.39	8.10	0.51	7.48	0.11	0.92	-0.116	1.1E-01
Mtr.47439.1.S1_at	4.04	3.61	4.63	5.34	5.50	4.87	4.10	0.51	5.23	0.32	1.28	0.354	3.1E-02
Mtr.14574.1.S1_at	10.30	9.34	9.53	9.77	9.51	9.80	9.73	0.51	9.70	0.16	1.00	-0.004	9.3E-01
Mtr.2650.1.S1_at	8.54	7.58	8.35	8.37	8.35	8.38	8.16	0.51	8.37	0.01	1.03	0.037	5.1E-01
Mtr.11102.1.S1_at	8.86	8.01	8.93	8.64	8.80	8.57	8.60	0.51	8.67	0.12	1.01	0.011	8.3E-01
Mtr.39366.1.S1_at	9.02	8.07	8.87	8.99	8.96	9.10	8.65	0.51	9.01	0.07	1.04	0.059	2.9E-01
Mtr.9154.1.S1_at	10.57	9.55	10.01	10.03	9.95	9.98	10.04	0.51	9.99	0.04	0.99	-0.008	8.6E-01
Mtr.35793.1.S1_at	9.31	8.46	9.38	9.27	9.20	9.22	9.05	0.51	9.23	0.04	1.02	0.029	5.7E-01
Mtr.13727.1.S1_at	8.19	7.32	8.22	8.80	8.56	7.92	7.91	0.51	8.43	0.45	1.07	0.092	2.6E-01
Mtr.34782.1.S1_s_at	9.84	8.82	9.39	9.56	9.51	9.84	9.35	0.51	9.64	0.18	1.03	0.044	4.1E-01
Mtr.46400.1.S1_at	2.88	3.81	2.98	3.14	2.86	3.03	3.22	0.51	3.01	0.14	0.94	-0.096	5.3E-01
Mtr.51131.1.S1_at	2.27	3.14	2.25	2.24	2.23	2.24	2.55	0.51	2.24	0.01	0.88	-0.189	3.5E-01
Mtr.21628.1.S1_at	10.69	10.11	9.67	9.99	9.72	9.99	10.15	0.51	9.90	0.15	0.98	-0.036	4.6E-01
Mtr.10505.1.S1_at	8.93	8.51	9.53	9.36	9.37	8.85	8.99	0.51	9.19	0.29	1.02	0.032	5.8E-01
Mtr.29436.1.S1_at	6.12	5.66	6.68	6.36	6.02	6.46	6.15	0.51	6.28	0.23	1.02	0.029	7.1E-01
Mtr.2498.1.S1_at	7.32	6.41	7.28	6.78	6.60	6.78	7.00	0.51	6.72	0.10	0.96	-0.059	4.0E-01
Mtr.26012.1.S1_at	4.09	3.58	4.60	3.80	3.56	3.84	4.09	0.51	3.73	0.15	0.91	-0.133	3.1E-01
Mtr.32231.1.S1_at	3.79	4.79	4.11	3.75	3.75	3.42	4.23	0.51	3.64	0.19	0.86	-0.217	1.3E-01
Mtr.37643.1.S1_at	7.65	7.26	8.27	7.98	8.40	8.02	7.73	0.51	8.13	0.24	1.05	0.074	2.8E-01
Mtr.20930.1.S1_at	9.11	8.09	8.61	8.66	9.03	8.59	8.60	0.51	8.76	0.24	1.02	0.026	6.6E-01
Mtr.10061.1.S1_at	7.92	7.18	6.94	7.42	7.44	7.59	7.35	0.51	7.48	0.09	1.02	0.027	6.7E-01
Mtr.34617.1.S1_at	7.60	6.72	7.62	7.56	7.64	7.55	7.31	0.51	7.58	0.05	1.04	0.052	4.2E-01
Mtr.39924.1.S1_at	11.22	10.59	11.60	11.05	11.40	11.01	11.14	0.51	11.15	0.22	1.00	0.002	9.7E-01
Mtr.7024.1.S1_x_at	4.82	3.95	4.85	4.99	4.73	4.82	4.54	0.51	4.85	0.13	1.07	0.093	3.8E-01
Mtr.1453.1.S1_at	10.39	9.51	10.39	9.97	10.08	9.77	10.10	0.51	9.94	0.16	0.98	-0.023	6.3E-01
Mtr.4966.1.S1_at	8.02	8.93	8.89	8.03	8.08	8.02	8.61	0.51	8.05	0.03	0.93	-0.098	1.3E-01
Mtr.45187.1.S1_at	4.69	4.41	5.40	4.64	4.64	4.51	4.83	0.51	4.59	0.08	0.95	-0.073	4.7E-01
Mtr.9925.1.S1_at	7.23	6.24	6.95	7.24	7.26	7.55	6.81	0.51	7.35	0.17	1.08	0.111	1.6E-01
Mtr.18422.1.S1_at	9.51	10.07	9.05	9.11	9.45	9.37	9.54	0.51	9.31	0.18	0.98	-0.036	5.0E-01
Mtr.13598.1.S1_at	10.62	9.61	10.23	10.34	10.20	10.23	10.15	0.51	10.26	0.07	1.01	0.015	7.4E-01
Mtr.35095.1.S1_at	5.87	5.00	4.97	5.43	5.34	5.64	5.28	0.51	5.47	0.15	1.04	0.051	5.7E-01
Mtr.24986.1.S1_at	2.60	3.48	2.60	2.56	2.56	2.54	2.90	0.51	2.55	0.01	0.88	-0.182	3.1E-01
Mtr.8988.1.S1_s_at	8.56	7.69	8.59	8.56	8.55	8.63	8.28	0.51	8.58	0.04	1.04	0.051	3.7E-01
Mtr.35955.1.S1_at	8.03	7.06	7.80	7.92	7.86	7.96	7.63	0.51	7.92	0.05	1.04	0.053	3.9E-01
Mtr.51358.1.S1_x_at	7.28	7.64	8.29	7.41	7.70	7.41	7.74	0.51	7.51	0.17	0.97	-0.044	5.0E-01
Mtr.33813.1.S1_at	9.84	8.90	9.70	9.90	9.72	10.14	9.48	0.51	9.92	0.21	1.05	0.066	2.4E-01
Mtr.42369.1.S1_at	7.35	6.74	7.75	7.04	6.67	6.63	7.28	0.51	6.78	0.22	0.93	-0.103	1.9E-01
Mtr.17540.1.S1_at	3.71	4.56	3.65	3.85	4.07	4.27	3.97	0.51	4.06	0.21	1.02	0.033	7.8E-01
Mtr.5992.1.S1_at	8.75	7.88	8.77	8.42	8.83	8.34	8.47	0.51	8.53	0.26	1.01	0.011	8.6E-01
Mtr.41965.1.S1_at	10.38	9.51	10.41	10.08	10.11	10.14	10.10	0.51	10.11	0.03	1.00	0.001	9.8E-01
Mtr.51544.1.S1_x_at	2.91	3.73	2.81	2.89	2.89	2.89	3.15	0.51	2.89	0.00	0.92	-0.123	4.3E-01
Mtr.2677.1.S1_at	10.36	11.02	11.36	10.65	10.78	10.65	10.92	0.51	10.69	0.08	0.98	-0.030	5.0E-01
Mtr.32528.1.S1_s_at	6.40	7.31	6.46	6.90	7.05	6.81	6.73	0.51	6.92	0.12	1.03	0.042	5.5E-01
Mtr.6137.1.S1_at	8.46	8.42	9.32	8.92	8.80	8.82	8.73	0.51	8.85	0.07	1.01	0.019	7.2E-01
Mtr.33753.1.S1_at	6.35	5.49	6.38	6.66	6.37	6.37	6.07	0.51	6.47	0.17	1.06	0.090	2.8E-01
Mtr.25417.1.S1_at	7.86	7.53	8.53	8.46	8.51	8.07	7.97	0.51	8.35	0.24	1.05	0.066	3.2E-01
Mtr.26615.1.S1_at	5.91	5.64	6.62	6.15	6.35	6.25	6.06	0.51	6.25	0.10	1.03	0.045	5.6E-01
Mtr.38296.1.S1_at	9.49	9.68	10.45	9.85	10.26	9.80	9.87	0.51	9.97	0.25	1.01	0.014	7.9E-01
Mtr.38610.1.S1_at	10.83	9.82	10.38	10.40	10.48	10.52	10.35	0.51	10.47	0.06	1.01	0.017	7.0E-01
Mtr.41581.1.S1_at	12.08	12.01	12.92	12.17	12.35	12.21	12.34	0.51	12.24	0.09	0.99	-0.011	7.7E-01
Mtr.32712.1.S1_at	10.40	10.58	9.62	9.47	9.27	9.66	10.20	0.51	9.47	0.20	0.93	-0.108	8.0E-02
Mtr.26114.1.S1_at	7.15	7.08	7.99	6.98	7.65	6.93	7.41	0.51	7.19	0.40	0.97	-0.043	5.9E-01
Mtr.5503.1.S1_at	8.93	7.94	8.23	8.61	8.43	8.64	8.36	0.51	8.56	0.11	1.02	0.034	5.5E-01
Mtr.44707.1.S1_s_at	8.37	7.75	8.76	8.62	8.50	8.27	8.29	0.51	8.46	0.18	1.02	0.030	6.1E-01
Mtr.21324.1.S1_at	5.41	6.19	5.23	5.28	5.07	5.54	5.61	0.51	5.29	0.24	0.94	-0.084	3.8E-01
Mtr.13927.1.S1_at	9.46	8.52	9.32	9.46	9.26	9.36	9.10	0.51	9.36	0.10	1.03	0.040	4.4E-01
Mtr.19605.1.S1_at	7.94	7.02	7.85	6.92	6.98	6.94	7.60	0.51	6.95	0.03	0.91	-0.129	9.2E-02
Mtr.5200.1.S1_at	6.92	6.12	5.97	6.36	6.65	6.60	6.34	0.51	6.54	0.16	1.03	0.045	5.5E-01
Mtr.49500.1.S1_at	8.49	8.48	9.36	8.68	8.66	8.42	8.78	0.51	8.58	0.15	0.98	-0.032	5.6E-01
Mtr.7211.1.S1_at	2.19	3.06	2.18	2.17	2.17	2.16	2.48	0.51	2.17	0.00	0.87	-0.194	3.5E-01
Mtr.3641.1.S1_at	5.64	4.80	4.73	4.85	5.16	4.99	5.06	0.51	5.00	0.15	0.99	-0.016	8.6E-01
Mtr.9526.1.S1_at	7.41	6.44	7.19	7.54	7.65	7.72	7.02	0.51	7.64	0.09	1.09	0.123	1.0E-01
Mtr.47803.1.S1_at	2.42	3.31	2.44	2.44	2.44	2.43	2.73	0.51	2.44	0.00	0.89	-0.162	3.8E-01
Mtr.51710.1.S1_at	1.98	2.86	1.98	1.97	1.97	1.97	2.27	0.51	1.97	0.00	0.87	-0.207	3.6E-01
Mtr.21125.1.S1_at	7.30	6.94	7.94	7.83	7.64	7.33	7.39	0.51	7.60	0.25	1.03	0.040	5.6E-01
Mtr.45101.1.S1_at	10.60	9.89	10.88	10.71	10.89	10.68	10.45	0.51	10.76	0.12	1.03	0.042	3.7E-01
Mtr.27275.1.S1_at	2.88	2.93	3.78	2.79	2.87	2.77	3.20	0.51	2.81	0.05	0.88	-0.187	2.6E-01
Mtr.13686.1.S1_at	6.74	6.24	7.26	7.30	7.30	7.10	6.74	0.51	7.23	0.11	1.07	0.100	1.8E-01
Mtr.45708.1.S1_s_at	9.19	8.25	9.05	9.14	9.10	9.12	8.83	0.51	9.12	0.02	1.03	0.046	3.8E-01
Mtr.27105.1.S1_at	8.96	7.95	8.39	8.62	8.58	8.60	8.43	0.51	8.60	0.02	1.02	0.028	6.0E-01
Mtr.11073.1.S1_at	11.17	10.29	10.28	10.01	10.20	10.01	10.58	0.51	10.07	0.11	0.95	-0.071	1.7E-01
Mtr.40622.1.S1_s_at	9.50	8.49	9.05	9.35	9.23	9.43	9.02	0.51	9.34	0.10	1.04	0.050	3.4E-01
Mtr.14938.1.S1_s_at	2.89	3.75	2.85	2.84	2.83	2.79	3.16	0.51	2.82	0.02	0.89	-0.165	3.1E-01
Mtr.35098.1.S1_at	9.02	8.03	8.70	8.62	8.77	8.92	8.58	0.51	8.77	0.15	1.02	0.031	5.7E-01
Mtr.28461.1.S1_at	7.76	6.83	7.66	7.51	7.58	7.59	7.42	0.51	7.56	0.04	1.02	0.028	6.5E-01
Mtr.20686.1.S1_at	6.88	7.82	7.69	7.32	7.36	7.38	7.46	0.51	7.35	0.03	0.98	-0.022	7.2E-01
Mtr.35006.1.S1_at	6.35	5.81	6.83	6.26	6.45	7.08	6.33	0.51	6.60	0.43	1.04	0.059	5.3E-01
Mtr.37695.1.S1_at	8.04	7.25	8.20	8.30	8.50	8.15	7.83	0.51	8.32	0.18	1.06	0.087	1.9E-01
Mtr.29208.1.S1_at	7.77	6.77	7.44	7.03	7.18	7.14	7.33	0.51	7.12	0.08	0.97	-0.042	5.2E-01
Mtr.24034.1.S1_s_at	7.50	7.50	8.38	7.13	7.48	7.16	7.80	0.51	7.26	0.19	0.93	-0.103	1.6E-01
Mtr.76.1.S1_at	8.40	7.40	7.76	8.03	8.00	8.17	7.86	0.51	8.07	0.09	1.03	0.039	5.1E-01
Mtr.9276.1.S1_at	8.19	7.35	8.27	8.20	8.38	8.28	7.94	0.51	8.29	0.09	1.04	0.062	3.1E-01
Mtr.43375.1.S1_at	9.92	9.19	10.16	9.79	9.88	9.70	9.76	0.51	9.79	0.09	1.00	0.005	9.1E-01
Mtr.32148.1.S1_x_at	5.90	4.94	5.72	5.49	5.20	5.67	5.52	0.51	5.46	0.24	0.99	-0.017	8.5E-01
Mtr.48309.1.S1_at	5.21	5.77	6.22	5.77	6.13	5.86	5.73	0.51	5.92	0.19	1.03	0.046	5.9E-01
Mtr.22843.1.S1_at	2.66	3.52	2.64	2.62	2.63	2.58	2.94	0.51	2.61	0.03	0.89	-0.172	3.2E-01
Mtr.9978.1.S1_at	12.14	11.14	11.79	11.78	11.78	11.69	11.69	0.51	11.75	0.05	1.01	0.008	8.4E-01
Mtr.42457.1.S1_at	10.30	9.29	9.72	9.78	9.51	9.60	9.77	0.51	9.63	0.14	0.99	-0.021	6.7E-01
Mtr.31101.1.S1_at	5.98	5.15	6.07	6.42	6.41	6.41	5.73	0.51	6.41	0.01	1.12	0.162	8.0E-02

Mtr.13823.1.S1_at	9.45	8.50	9.29	9.31	9.60	9.43	9.08	0.51	9.45	0.15	1.04	0.057	3.0E-01
Mtr.30781.1.S1_at	8.59	7.61	8.33	8.28	8.75	8.41	8.17	0.51	8.48	0.24	1.04	0.053	4.0E-01
Mtr.7450.1.S1 at	8.51	8.60	9.43	8.68	8.71	8.65	8.85	0.51	8.68	0.03	0.98	-0.028	5.9E-01
Mtr.27395.1.S1 at	7.86	6.85	7.39	7.55	7.61	7.15	7.37	0.51	7.44	0.25	1.01	0.013	8.4E-01
Mtr.22592.1.S1 at	6.55	6.05	5.54	5.92	6.29	6.19	6.05	0.51	6.13	0.19	1.01	0.021	7.9E-01
Mtr.32349.1.S1_s_at	8.43	8.06	9.06	8.39	8.75	8.05	8.52	0.51	8.40	0.35	0.99	-0.021	7.5E-01
Mtr.51429.1.S1_s_at	3.97	3.17	3.03	2.67	2.92	2.91	3.39	0.51	2.83	0.14	0.84	-0.259	1.4E-01
Mtr.49856.1.S1 s at	4.51	3.68	4.59	4.99	4.77	4.87	4.26	0.51	4.88	0.11	1.14	0.195	1.1E-01
Mtr.28591.1.S1 at	5.71	5.14	6.15	6.64	6.77	6.20	5.67	0.51	6.54	0.30	1.15	0.206	6.2E-02
Mtr.6563.1.S1 at	9.07	8.06	8.61	8.56	8.53	8.45	8.58	0.51	8.52	0.06	0.99	-0.011	8.3E-01
Mtr.291.1.S1 at	3.78	3.71	4.62	3.82	3.80	3.80	4.04	0.51	3.81	0.01	0.94	-0.085	4.8E-01
Mtr.9778.1.S1_at	4.34	3.70	4.70	4.33	4.50	4.33	4.25	0.51	4.39	0.10	1.03	0.047	6.6E-01
Mtr.13767.1.S1_at	8.99	8.08	8.93	8.75	8.78	8.75	8.66	0.51	8.76	0.01	1.01	0.016	7.6E-01
Mtr.51538.1.S1_at	6.08	7.08	6.49	6.93	7.22	6.89	6.55	0.51	7.01	0.18	1.07	0.099	2.1E-01
Mtr.22184.1.S1 at	9.39	8.43	9.19	9.25	9.25	9.23	9.00	0.51	9.25	0.01	1.03	0.039	4.5E-01
Mtr.50996.1.S1 at	8.50	7.52	8.24	8.29	8.26	8.15	8.09	0.51	8.23	0.07	1.02	0.026	6.5E-01
Mtr.21864.1.S1 at	3.02	3.88	2.98	2.97	2.95	2.95	3.29	0.51	2.95	0.01	0.90	-0.157	3.1E-01
Mtr.21823.1.S1_at	10.66	9.79	10.68	10.53	10.75	10.51	10.38	0.51	10.60	0.13	1.02	0.030	5.1E-01
Mtr.1912.1.S1_at	3.28	2.56	3.53	3.37	2.92	2.46	3.12	0.51	2.92	0.46	0.93	-0.098	6.3E-01
Mtr.12039.1.S1_at	11.86	11.08	10.90	11.21	11.07	11.36	11.28	0.51	11.22	0.15	0.99	-0.008	8.5E-01
Mtr.11899.1.S1 at	8.17	7.22	7.39	7.78	7.98	8.01	7.60	0.51	7.92	0.12	1.04	0.061	3.4E-01
Mtr.35521.1.S1 at	9.45	8.44	9.00	8.57	8.90	9.10	8.96	0.51	8.86	0.27	0.99	-0.017	7.6E-01
Mtr.31679.1.S1 at	7.90	7.26	8.25	7.91	7.84	7.83	7.80	0.51	7.86	0.04	1.01	0.010	8.6E-01
Mtr.46845.1.S1_at	10.72	9.71	10.17	10.13	10.61	10.29	10.20	0.51	10.34	0.25	1.01	0.020	6.8E-01
Mtr.42984.1.S1_at	9.17	8.80	9.80	9.57	10.09	9.65	9.25	0.51	9.77	0.28	1.06	0.078	2.0E-01
Mtr.12838.1.S1_at	9.25	8.33	9.15	8.36	8.49	8.21	8.91	0.51	8.35	0.14	0.94	-0.093	1.4E-01
Mtr.52278.1.S1 at	2.11	2.99	2.11	2.11	2.11	2.10	2.40	0.51	2.11	0.00	0.88	-0.190	3.7E-01
Mtr.12.1.S1 at	10.92	9.95	10.19	10.24	10.33	10.41	10.35	0.51	10.33	0.09	1.00	-0.004	9.4E-01
Mtr.1871.1.S1 at	6.89	6.00	6.02	6.07	6.07	6.43	6.30	0.51	6.19	0.21	0.98	-0.026	7.4E-01
Mtr.31658.1.S1_s_at	11.93	10.98	11.76	11.64	11.49	11.68	11.56	0.51	11.61	0.10	1.00	0.006	8.9E-01
Mtr.37314.1.S1_at	12.27	11.74	11.25	11.96	11.93	12.09	11.75	0.51	11.99	0.08	1.02	0.029	4.7E-01
Mtr.4182.1.S1_at	7.20	8.20	7.78	7.92	8.45	7.93	7.73	0.51	8.10	0.30	1.05	0.068	3.3E-01
Mtr.27963.1.S1 at	8.31	7.31	7.70	7.56	7.60	7.59	7.77	0.51	7.58	0.02	0.98	-0.036	5.5E-01
Mtr.21315.1.S1 at	6.11	5.52	6.52	6.15	6.13	6.02	6.05	0.51	6.10	0.07	1.01	0.011	8.9E-01
Mtr.2773.1.S1 at	2.70	3.57	2.69	2.68	2.68	2.64	2.98	0.51	2.67	0.03	0.89	-0.162	3.4E-01
Mtr.18237.1.S1 at	9.39	9.16	10.13	9.23	9.28	8.85	9.56	0.51	9.12	0.23	0.95	-0.068	2.4E-01
Mtr.44485.1.S1_at	8.82	9.26	9.83	9.41	9.40	9.28	9.30	0.51	9.37	0.07	1.01	0.010	8.4E-01
Mtr.1266.1.S1 at	5.87	5.04	4.96	6.21	6.95	6.38	5.29	0.51	6.51	0.39	1.23	0.300	2.9E-02
Mtr.32474.1.S1 at	2.48	2.45	3.34	2.40	2.40	2.39	2.75	0.51	2.40	0.01	0.87	-0.201	2.9E-01
Mtr.22670.1.S1 at	6.91	6.52	7.52	7.15	7.18	6.72	6.98	0.51	7.01	0.26	1.00	0.006	9.3E-01
Mtr.33095.1.S1 at	8.22	7.63	7.22	8.07	7.59	7.87	7.69	0.51	7.84	0.24	1.02	0.029	6.6E-01
Mtr.11716.1.S1_at	10.27	9.33	9.48	9.75	9.90	9.79	9.70	0.51	9.81	0.08	1.01	0.017	7.2E-01
Mtr.41328.1.S1_at	6.03	5.14	6.00	6.01	6.11	6.01	5.72	0.51	6.05	0.06	1.06	0.080	3.3E-01
Mtr.11163.1.S1 at	8.67	7.65	8.15	7.85	8.67	8.22	8.16	0.51	8.25	0.41	1.01	0.016	8.2E-01
Mtr.33369.1.S1 at	3.17	2.31	3.20	2.83	3.35	2.87	2.89	0.51	3.02	0.29	1.04	0.061	7.3E-01
Mtr.37155.1.S1 at	7.07	6.69	7.69	8.61	8.71	8.24	7.15	0.51	8.52	0.25	1.19	0.253	1.3E-02
Mtr.6454.1.S1 at	2.05	2.92	2.04	2.03	2.03	2.03	2.33	0.51	2.03	0.00	0.87	-0.201	3.6E-01
Mtr.13607.1.S1_at	10.30	9.41	10.27	10.31	10.42	10.41	9.99	0.51	10.38	0.06	1.04	0.054	2.6E-01
Mtr.12882.1.S1_at	9.75	8.99	9.94	9.81	9.88	9.77	9.56	0.51	9.82	0.06	1.03	0.039	4.2E-01
Mtr.21933.1.S1 at	3.17	3.94	2.98	2.97	2.87	2.94	3.37	0.51	2.93	0.05	0.87	-0.201	2.1E-01
Mtr.34597.1.S1 s at	9.61	8.72	9.58	9.46	9.24	9.34	9.31	0.51	9.35	0.11	1.00	0.006	9.0E-01
Mtr.20453.1.S1 at	11.21	10.24	10.96	10.95	10.88	10.92	10.81	0.51	10.92	0.04	1.01	0.015	7.1E-01
Mtr.29419.1.S1 at	5.82	5.11	6.08	6.11	5.81	5.81	5.67	0.51	5.91	0.17	1.04	0.060	4.8E-01
Mtr.3109.1.S1_at	6.54	5.74	6.67	6.61	6.61	6.67	6.32	0.51	6.63	0.04	1.05	0.070	3.4E-01
Mtr.6116.1.S1_at	8.43	7.98	8.99	8.34	8.37	8.50	8.46	0.50	8.41	0.08	0.99	-0.010	8.5E-01
Mtr.18735.1.S1_at	8.66	7.67	8.33	8.69	8.66	8.73	8.22	0.50	8.69	0.04	1.06	0.080	1.8E-01
Mtr.11826.1.S1 at	8.33	7.34	7.67	7.94	7.78	7.72	7.78	0.50	7.81	0.11	1.00	0.006	9.2E-01
Mtr.23679.1.S1 x at	2.73	1.87	2.75	2.72	2.78	2.96	2.45	0.50	2.82	0.12	1.15	0.202	2.9E-01
Mtr.11861.1.S1 at	10.79	9.78	10.28	10.77	10.63	10.78	10.28	0.50	10.72	0.08	1.04	0.061	2.1E-01
Mtr.33371.1.S1_at	3.10	3.94	3.04	3.02	2.94	3.14	3.36	0.50	3.03	0.10	0.90	-0.149	3.3E-01
Mtr.21009.1.S1_x_at	4.20	5.04	4.14	4.08	3.96	4.36	4.46	0.50	4.14	0.21	0.93	-0.110	3.6E-01
Mtr.794.1.S1_at	2.67	3.54	2.66	2.65	2.65	2.65	2.95	0.50	2.65	0.00	0.90	-0.157	3.5E-01
Mtr.32962.1.S1 at	7.57	6.72	7.62	7.62	7.33	7.64	7.30	0.50	7.53	0.17	1.03	0.044	5.0E-01
Mtr.20012.1.S1 at	2.91	3.77	2.88	2.86	2.80	2.83	3.19	0.50	2.83	0.03	0.89	-0.172	2.9E-01
Mtr.39659.1.S1 at	9.46	8.73	9.70	9.48	9.49	9.27	9.30	0.50	9.41	0.12	1.01	0.018	7.1E-01
Mtr.14442.1.S1_at	8.17	7.33	8.24	8.45	8.34	8.09	7.92	0.50	8.29	0.19	1.05	0.067	2.9E-01
Mtr.17966.1.S1_at	8.16	7.17	7.52	7.46	7.73	7.71	7.62	0.50	7.64	0.15	1.00	0.003	9.5E-01
Mtr.14047.1.S1_s_at	8.93	9.62	9.92	9.48	10.01	9.55	9.49	0.50	9.68	0.29	1.02	0.028	6.1E-01
Mtr.51291.1.S1 at	12.37	11.49	11.50	12.00	12.30	12.26	11.79	0.50	12.19	0.16	1.03	0.048	2.6E-01
Mtr.35438.1.S1 at	6.02	5.03	5.35	5.85	6.26	5.77	5.47	0.50	5.96	0.27	1.09	0.124	2.1E-01
Mtr.49293.1.S1 at	9.42	8.59	9.51	9.61	9.65	9.65	9.17	0.50	9.64	0.02	1.05	0.072	1.8E-01
Mtr.13351.1.S1_at	12.21	11.20	11.64	11.78	11.63	11.70	11.68	0.50	11.70	0.08	1.00	0.002	9.5E-01
Mtr.44043.1.S1_at	5.77	4.88	5.73	5.36	5.87	5.26	5.46	0.50	5.50	0.33	1.01	0.010	9.2E-01
Mtr.34767.1.S1 at	6.26	5.43	6.35	5.58	5.38	5.27	6.01	0.50	5.41	0.16	0.90	-0.153	1.2E-01
Mtr.16487.1.S1 s at	9.69	10.63	9.85	9.52	9.77	9.85	10.06	0.50	9.71	0.17	0.97	-0.051	3.2E-01
Mtr.26916.1.S1 at	3.93	3.97	3.08	3.92	3.91	3.91	3.66	0.50	3.91	0.00	1.07	0.097	4.3E-01
Mtr.18481.1.S1 at	13.80	12.84	13.57	13.38	13.34	13.49	13.40	0.50	13.40	0.08	1.00	0.000	1.0E+00
Mtr.10622.1.S1_at	12.46	11.48	12.18	12.34	12.08	12.13	12.04	0.50	12.18	0.14	1.01	0.017	6.7E-01
Mtr.40564.1.S1_at	9.92	9.16	10.11	9.58	9.23	9.65	9.73	0.50	9.49	0.22	0.98	-0.036	4.9E-01
Mtr.7235.1.S1_at	7.78	6.79	7.14	7.87	7.71	7.81	7.24	0.50	7.79	0.08	1.08	0.107	1.3E-01
Mtr.9217.1.S1 at	8.52	7.73	8.67	8.57	8.35	8.28	8.31	0.50	8.40	0.15	1.01	0.016	7.8E-01
Mtr.40869.1.S1 at	10.53	9.72	10.65	10.53	10.53	10.38	10.30	0.50	10.48	0.09	1.02	0.025	5.8E-01
Mtr.11983.1.S1 at	6.51	5.79	5.55	5.96	6.49	6.27	5.95	0.50	6.24	0.27	1.05	0.070	4.2E-01
Mtr.26394.1.S1_at	11.25	10.25	10.88	10.85	10.79	10.90	10.79	0.50	10.85	0.06	1.00	0.007	8.6E-01
Mtr.43916.1.S1_at	10.74	9.77	10.48	10.67	10.86	10.77	10.33	0.50	10.77	0.09	1.04	0.060	2.1E-01
Mtr.18058.1.S1_at	9.16	8.44	9.41	9.24	9.45	9.29	9.00	0.50	9.33	0.11	1.04	0.051	3.4E-01
Mtr.17710.1.S1 at	10.38	9.68	10.65	10.12	9.94	9.87	10.24	0.50	9.98	0.13	0.97	-0.037	4.4E-01
Mtr.41658.1.S1 at	8.95	7.99	8.73	8.35	8.77	8.65	8.56	0.50	8.59	0.22	1.00	0.005	9.3E-01
Mtr.5096.1.S1 at	8.34	7.34	7.93	8.11	8.10	8.06	7.87	0.50	8.09	0.03	1.03	0.040	4.9E-01
Mtr.31082.1.S1_at	3.99	3.49	4.50	4.22	4.89	3.78	3.99	0.50	4.30	0.56	1.08	0.106	5.2E-01
Mtr.30262.1.S1_at	7.95	7.03	7.85	7.35	7.95	7.76	7.61	0.50	7.68	0.31	1.01	0.014	8.4E-01
Mtr.6814.1.S1_at	5.86	5.83	6.72	5.82	6.14	6.28	6.13	0.50	6.08	0.23	0.99	-0.013	8.8E-01
Mtr.44354.1.S1 at	10.72	9.74	10.42	10.63	9.41	10.40	10.29	0.50	10.15	0.65	0.99	-0.021	7.7E-01
Mtr.19483.1.S1 s at	11.32	10.45	11.33	11.64	11.27	11.37	11.03	0.50	11.43	0.19	1.04	0.051	2.7E-01
Mtr.44385.1.S1 at	5.53	5.20	6.19	5.87	5.81	5.43	5.64	0.50	5.70	0.24	1.01	0.016	8.6E-01
Mtr.50390.1.S1_at	7.83	7.12	8.09	7.98	8.01	7.87	7.68	0.50	7.95	0.07	1.04	0.050	4.1E-01
Mtr.41817.1.S1_at	6.33	6.42	7.24	6.05	6.62	6.75	6.67	0.50	6.47	0.37	0.97	-0.042	6.3E-01
Mtr.30006.1.S1_at	7.02	6.29	7.25	6.88	7.08	6.95	6.85	0.50	6.97	0.10	1.02	0.025	7.1E-01
Mtr.38357.1.S1 at	11.19	10.26	11.06	11.05	10.90	10.89	10.84	0.50	10.95	0.09	1.01	0.015	7.2E-01
Mtr.38870.1.S1 x at	10.88	11.10	11.84	11.65	11.32	11.41	11.27	0.50	11.46	0.17	1.02	0.024	5.8E-01
Mtr.49974.1.S1 at	8.36	7.80	8.81	8.56	8.31	8.33	8.32	0.50	8.40	0.14	1.01	0.014	8.1E-01
Mtr.42073.1.S1 at	8.82	7.97	8.86	8.83	9.02	8.84	8.55	0.50	8.90	0.11	1.04	0.057	3.1E-01
Mtr.33867.1.S1_at	6.96	7.39	7.96	7.65	7.46	7.42	7.44	0.50	7.51	0.12	1.01	0.014	8.2E-01
Mtr.42677.1.S1 at	8.29	7.33	8.05	8.11	7.85	8.05	7.89	0.50	8.01	0.14	1.01	0.021	7.2E-01
Mtr.22558.1.S1 s at	10.07	9.07	9.50	9.68	9.89	9.93	9.55	0.50	9.84	0.13	1.03	0.043	3.9E-01
Mtr.38910.1.S1 at	8.23	7.35	7.36	7.47	6.97	7.37	7.65	0.50	7.27	0.27	0.95	-0.073	3.1E-01
Mtr.16339.1.S1 at	3.90	3.25	4.24	3.90	3.83	3.95	3.79	0.50	3.89	0.06	1.03	0.037	7.5E-01
Mtr.35861.1.S1_at	8.14	7.14	7.71	8.03	7.96	8.20	7.66	0.50	8.06	0.12	1.05	0.073	2.5E-01
Mtr.52317.1.S1_at	7.41	6.92	7.93	7.80	7.72	7.57	7.42	0.50	7.70	0.11	1.04	0.053	4.1E-01
Mtr.28321.1.S1 s at	2.06	3.04	2.35	3.90	4.87	3.84	2.48	0.50	4.21	0.58	1.69	0.759	1.8E-02
Mtr.39250.1.S1 at	8.57	7.59	8.27	8.66	8.46	8.69	8.14	0.50	8.60	0.12	1.06	0.080	2.0E-01
Mtr.40821.1.S1 at	10.53	9.90	10.89	10.80	11.11	10.84	10.44	0.50	10.92	0.17	1.05	0.064	2.0E-01
Mtr.16236.1.S1 at	2.48	3.34	2.46	2.46	2.45	2.44	2.76	0.50	2.45	0.01	0.89	-0.172	3.5E-01
Mtr.38243.1.S1_at	7.16	6.47	7.45	7.49	7.45	7.55	7.02	0.50	7.50	0.05	1.07	0.094	1.8E-01
Mtr.4622.1.S1_at	7.65	6.69	7.43	7.78	7.22	7.60	7.26	0.50	7.53	0.29	1.04	0.054	4.6E-01
Mtr.44029.1.S1_at	9.42	8.66	9.60	9.44	9.36	9.41	9.23	0.50	9.40	0.04	1.02	0.028	5.7E-01
Mtr.35539.1.S1 at	9.68	10.22	10.68	10.17	10.18	9.90	10.19	0.50	10.09	0.16	0.99	-0.015	7.4E-01
Mtr.50714.1.S1 at	9.35	8.91	8.35	8.84	8.83	8.97	8.87	0.50	8.88	0.08	1.00	0.001	9.8E-01
Mtr.23028.1.S1 at	10.06	9.15	9.97	10.33	10.26	10.27	9.73	0.50	10.29	0.04	1.06	0.081	1.3E-01
Mtr.45768.1.S1_at	8.93	8.29	7.94	8.33	8.08	8.57	8.39	0.50	8.32	0.25	0.99	-0.011	8.6E-01
Mtr.9031.1.S1_s_at	5.79	4.82	5.55	5.12	5.82	5.47	5.39	0.50	5.47	0.35	1.02	0.022	8.2E-01
Mtr.33358.1.S1_s_at	9.04	8.21	9.12	8.89	8.79	8.87	8.79	0.50	8.85	0.06	1.01	0.010	8.4E-01
Mtr.42640.1.S1 at	9.73	8.74	9.39	9.69	9.49	9.70	9.29	0.50	9.63	0.12	1.04	0.051	3.2E-01
Mtr.45580.1.S1 at	4.87	4.77	5.69	5.52	5.24	4.36	5.11	0.50	5.04	0.61	0.99	-0.020	8.8E-01
Mtr.37299.1.S1 at	12.13	11.42	11.16	10.85	10.99	11.01	11.57	0.50	10.95	0.09	0.95	-0.079	1.0E-01
Mtr.10536.1.S1_at	4.20	3.45	4.40	2.98	3.84	4.00	4.01	0.50	3.61	0.55	0.90	-0.154	4.0E-01
Mtr.13943.1.S1_at	3.94	3.06	3.91	5.40	5.00	4.35	3.64	0.50	4.92	0.53	1.35	0.435	3.9E-02
Mtr.41770.1.S1_at	8.64	8.35	9.33	9.00	8.97	8.65	8.78	0.50	8.87	0.20	1.01	0.016	7.7E-01
Mtr.21956.1.S1 at	7.91	8.05	7.12	7.38	7.71	7.81	7.70	0.50	7.63	0.22	0.99	-0.012	8.5E-01
Mtr.37335.1.S1 at	12.27	11.36	11.43	11.91	11.30	11.78	11.69	0.50	11.66	0.32	1.00	-0.003	9.5E-01
Mtr.2686.1.S1 at	7.42	6.75	7.73	7.49	7.71	7.55	7.30	0.50	7.58	0.11	1.04	0.054	4.0E-01

Mtr.918.1.S1_at	3.34	4.21	3.33	3.29	3.24	3.22	3.63	0.50	3.25	0.04	0.90	-0.158	2.6E-01
Mtr.40092.1.S1_at	7.51	7.39	8.32	7.73	7.30	7.27	7.74	0.50	7.43	0.26	0.96	-0.058	4.0E-01
Mtr.12152.1.S1_at	8.78	8.17	9.17	8.76	8.84	8.60	8.71	0.50	8.74	0.12	1.00	0.005	9.3E-01
Mtr.38643.1.S1_s_at	11.04	10.43	10.04	10.66	10.40	10.74	10.50	0.50	10.60	0.18	1.01	0.013	7.8E-01
Mtr.9663.1.S1_at	7.98	6.98	7.56	7.82	7.44	7.81	7.51	0.50	7.69	0.21	1.02	0.035	5.9E-01
Mtr.41720.1.S1_at	6.78	6.75	7.63	7.11	7.09	7.03	7.05	0.50	7.08	0.04	1.00	0.004	9.5E-01
Mtr.8790.1.S1_at	10.47	9.68	9.54	10.82	10.84	10.73	9.90	0.50	10.80	0.06	1.09	0.126	3.7E-02
Mtr.46506.1.S1_x_at	4.26	5.03	4.09	4.23	4.13	4.22	4.46	0.50	4.19	0.06	0.94	-0.088	4.2E-01
Mtr.265.1.S1_at	5.63	6.63	5.99	6.96	6.23	6.36	6.08	0.50	6.52	0.39	1.07	0.099	3.0E-01
Mtr.2684.1.S1_s_at	8.34	7.56	8.49	8.22	8.23	8.27	8.13	0.50	8.24	0.03	1.01	0.019	7.3E-01
Mtr.36034.1.S1_at	5.33	4.47	5.35	5.31	5.39	5.31	5.05	0.50	5.34	0.04	1.06	0.080	3.8E-01
Mtr.1909.1.S1_at	5.27	4.65	5.64	7.54	8.01	7.72	5.18	0.50	7.76	0.24	1.50	0.581	1.3E-03
Mtr.27224.1.S1_at	4.73	3.77	3.98	4.59	4.56	4.60	4.16	0.50	4.58	0.02	1.10	0.140	2.2E-01
Mtr.36012.1.S1_at	6.56	5.92	6.91	6.71	6.41	6.46	6.46	0.50	6.53	0.16	1.01	0.014	8.5E-01
Mtr.35238.1.S1_at	10.16	9.23	10.02	9.98	10.04	9.84	9.80	0.50	9.96	0.10	1.02	0.022	6.3E-01
Mtr.35364.1.S1_at	8.01	7.24	8.18	8.08	8.35	8.10	7.81	0.50	8.18	0.15	1.05	0.066	2.9E-01
Mtr.35618.1.S1_at	10.06	9.06	9.63	10.05	9.93	9.95	9.58	0.50	9.98	0.07	1.04	0.058	2.5E-01
Mtr.38518.1.S1_at	10.65	9.72	10.52	10.45	10.48	10.45	10.30	0.50	10.46	0.02	1.02	0.022	6.1E-01
Mtr.8328.1.S1_at	8.20	8.35	9.13	8.73	8.62	8.48	8.56	0.50	8.61	0.13	1.01	0.009	8.7E-01
Mtr.44301.1.S1_s_at	9.31	8.57	8.36	8.87	8.81	9.11	8.74	0.50	8.93	0.16	1.02	0.030	5.8E-01
Mtr.5061.1.S1_at	7.42	7.30	6.50	7.42	7.56	7.57	7.07	0.50	7.52	0.08	1.06	0.088	2.0E-01
Mtr.11677.1.S1_at	7.97	7.03	7.20	7.60	7.44	7.68	7.40	0.50	7.57	0.12	1.02	0.033	5.9E-01
Mtr.22408.1.S1_x_at	3.31	4.09	3.15	3.56	3.56	4.08	3.52	0.50	3.73	0.30	1.06	0.086	5.6E-01
Mtr.7506.1.S1_s_at	9.28	8.27	8.75	8.92	8.87	8.79	8.77	0.50	8.86	0.06	1.01	0.015	7.6E-01
Mtr.45200.1.S1_at	9.89	9.89	10.76	10.18	10.25	9.86	10.18	0.50	10.10	0.21	0.99	-0.012	8.1E-01
Mtr.21481.1.S1_at	2.25	3.11	2.24	2.23	2.23	2.23	2.53	0.50	2.23	0.00	0.88	-0.181	3.6E-01
Mtr.7508.1.S1_at	9.71	8.82	9.66	9.84	10.25	9.76	9.40	0.50	9.95	0.26	1.06	0.082	1.7E-01
Mtr.12029.1.S1_at	7.06	7.70	6.72	6.92	6.92	6.92	7.16	0.50	6.92	0.00	0.97	-0.049	4.6E-01
Mtr.20563.1.S1_at	2.30	3.16	2.28	2.27	2.27	2.26	2.58	0.50	2.26	0.01	0.88	-0.187	3.4E-01
Mtr.1283.1.S1_at	2.92	3.74	2.83	2.89	2.79	2.80	3.16	0.50	2.83	0.06	0.89	-0.162	3.1E-01
Mtr.39240.1.S1_at	8.77	8.23	9.23	8.69	8.95	9.02	8.74	0.50	8.89	0.17	1.02	0.024	6.6E-01
Mtr.13756.1.S1_at	4.42	4.68	5.39	4.55	4.56	4.74	4.83	0.50	4.62	0.11	0.96	-0.065	5.1E-01
Mtr.6141.1.S1_at	5.21	4.38	5.28	5.04	5.33	5.20	4.95	0.50	5.19	0.15	1.05	0.067	4.8E-01
Mtr.40217.1.S1_at	10.99	11.89	11.83	11.63	11.72	11.65	11.57	0.50	11.67	0.05	1.01	0.012	7.6E-01
Mtr.43664.1.S1_at	11.71	10.72	11.35	11.04	11.11	11.19	11.26	0.50	11.11	0.08	0.99	-0.019	6.4E-01
Mtr.4914.1.S1_at	10.86	9.86	10.43	10.57	10.79	10.57	10.38	0.50	10.65	0.13	1.03	0.036	4.3E-01
Mtr.15662.1.S1_s_at	14.22	13.34	14.19	14.38	14.22	14.16	13.91	0.50	14.26	0.11	1.02	0.035	3.1E-01
Mtr.46652.1.S1_x_at	2.25	3.11	2.24	2.23	2.23	2.23	2.53	0.50	2.23	0.00	0.88	-0.181	3.6E-01
Mtr.41744.1.S1_at	11.19	10.41	10.26	10.94	10.79	11.13	10.62	0.50	10.95	0.17	1.03	0.045	3.3E-01
Mtr.9087.1.S1_at	10.90	10.29	11.28	11.49	11.54	11.39	10.82	0.50	11.47	0.07	1.06	0.084	9.0E-02
Mtr.8980.1.S1_at	8.00	7.45	8.45	8.01	8.14	8.01	7.97	0.50	8.05	0.07	1.01	0.016	7.8E-01
Mtr.48109.1.S1_at	8.80	9.12	8.14	8.60	8.17	8.45	8.69	0.50	8.40	0.22	0.97	-0.047	4.2E-01
Mtr.47244.1.S1_at	5.38	5.26	6.18	5.37	5.40	4.91	5.61	0.50	5.23	0.27	0.93	-0.102	3.1E-01
Mtr.27619.1.S1_at	6.81	5.85	6.57	6.66	6.59	6.79	6.41	0.50	6.68	0.10	1.04	0.060	4.1E-01
Mtr.28692.1.S1_at	2.26	2.31	3.15	2.36	2.24	2.26	2.57	0.50	2.29	0.06	0.89	-0.172	3.8E-01
Mtr.32566.1.S1_x_at	3.81	2.91	2.99	3.82	4.24	3.83	3.24	0.50	3.96	0.24	1.22	0.291	8.7E-02
Mtr.1513.1.S1_x_at	7.33	6.40	6.54	6.57	6.28	6.56	6.76	0.50	6.47	0.16	0.96	-0.063	4.0E-01
Mtr.40836.1.S1_at	10.71	10.48	11.43	10.89	10.77	10.57	10.87	0.50	10.74	0.16	0.99	-0.017	6.9E-01
Mtr.48613.1.S1_at	2.62	3.47	2.60	2.56	2.55	2.58	2.89	0.50	2.56	0.01	0.88	-0.176	3.1E-01
Mtr.44388.1.S1_at	8.73	7.74	8.32	8.46	8.84	8.19	8.26	0.50	8.50	0.33	1.03	0.040	5.3E-01
Mtr.25945.1.S1_at	3.83	3.34	2.83	3.60	3.34	3.57	3.34	0.50	3.50	0.14	1.05	0.071	6.0E-01
Mtr.26817.1.S1_at	3.54	2.75	2.61	2.89	3.07	3.01	2.97	0.50	2.99	0.10	1.01	0.012	9.4E-01
Mtr.12775.1.S1_at	9.18	8.59	9.58	9.30	9.49	9.27	9.12	0.50	9.35	0.12	1.03	0.037	4.7E-01
Mtr.12989.1.S1_at	9.57	8.80	9.74	9.32	9.24	9.32	9.37	0.50	9.29	0.05	0.99	-0.012	8.0E-01
Mtr.19280.1.S1_at	6.95	5.95	6.34	7.46	7.31	7.20	6.41	0.50	7.32	0.13	1.14	0.190	3.9E-02
Mtr.33659.1.S1_at	3.38	2.50	2.53	2.49	2.48	2.48	2.80	0.50	2.49	0.00	0.89	-0.172	3.4E-01
Mtr.32873.1.S1_at	9.26	8.34	9.13	9.06	9.02	8.90	8.91	0.50	8.99	0.08	1.01	0.014	7.9E-01
Mtr.22306.1.S1_at	7.13	6.72	7.72	6.77	7.18	6.89	7.19	0.50	6.95	0.21	0.97	-0.050	4.8E-01
Mtr.49990.1.S1_at	4.83	3.95	4.79	4.78	4.86	4.71	4.52	0.50	4.79	0.08	1.06	0.082	4.2E-01
Mtr.44920.1.S1_at	12.28	11.69	11.29	11.47	11.57	11.53	11.75	0.50	11.52	0.05	0.98	-0.029	4.7E-01
Mtr.31982.1.S1_at	3.01	3.98	3.30	3.46	3.35	2.71	3.43	0.50	3.17	0.41	0.93	-0.112	5.3E-01
Mtr.42662.1.S1_at	2.05	2.91	2.04	2.03	2.03	2.03	2.33	0.50	2.03	0.00	0.87	-0.199	3.6E-01
Mtr.28131.1.S1_at	5.33	4.59	5.54	5.17	5.47	4.55	5.15	0.50	5.06	0.47	0.98	-0.025	8.3E-01
Mtr.26380.1.S1_at	2.84	3.68	2.80	2.80	2.76	2.75	3.11	0.50	2.77	0.02	0.89	-0.166	3.1E-01
Mtr.40462.1.S1_at	10.10	9.14	9.38	9.79	9.66	9.77	9.54	0.50	9.74	0.07	1.02	0.029	5.4E-01
Mtr.39490.1.S1_at	4.47	4.56	5.38	5.47	5.04	4.85	4.80	0.50	5.12	0.32	1.07	0.092	4.1E-01
Mtr.43655.1.S1_at	10.75	9.78	10.04	10.67	10.35	10.74	10.19	0.50	10.59	0.21	1.04	0.055	2.7E-01
Mtr.42131.1.S1_at	9.39	10.11	10.35	9.69	9.77	9.55	9.95	0.50	9.67	0.11	0.97	-0.041	4.0E-01
Mtr.21156.1.S1_at	5.55	5.70	6.48	5.93	6.39	5.87	5.91	0.50	6.06	0.29	1.02	0.035	6.8E-01
Mtr.45286.1.S1_at	7.80	6.97	7.87	8.08	7.83	7.85	7.55	0.50	7.92	0.14	1.05	0.070	2.8E-01
Mtr.40822.1.S1_at	8.75	8.73	9.60	8.84	8.97	9.00	9.02	0.50	8.94	0.09	0.99	-0.014	7.8E-01
Mtr.19682.1.S1_at	9.45	8.53	9.33	9.23	9.15	9.34	9.11	0.50	9.24	0.10	1.01	0.021	6.7E-01
Mtr.12051.1.S1_at	8.13	7.13	7.68	7.74	7.76	7.83	7.65	0.50	7.78	0.04	1.02	0.025	6.7E-01
Mtr.51421.1.S1_at	7.67	6.70	6.99	7.12	7.44	7.12	7.12	0.50	7.23	0.18	1.01	0.021	7.5E-01
Mtr.38960.1.S1_at	9.33	8.36	9.07	8.77	8.49	8.91	8.92	0.50	8.72	0.21	0.98	-0.032	5.7E-01
Mtr.25037.1.S1_s_at	2.27	3.13	2.25	2.26	2.25	2.25	2.55	0.50	2.25	0.01	0.88	-0.180	3.6E-01
Mtr.24897.1.S1_at	8.61	7.94	8.91	8.97	9.02	8.86	8.49	0.50	8.95	0.08	1.06	0.077	1.8E-01
Mtr.32786.1.S1_at	10.58	9.85	9.63	10.32	10.11	10.47	10.02	0.50	10.30	0.18	1.03	0.040	4.1E-01
Mtr.45633.1.S1_s_at	10.92	10.58	9.94	10.84	11.46	10.02	10.48	0.50	10.78	0.72	1.03	0.041	5.9E-01
Mtr.26499.1.S1_at	6.78	6.30	7.30	7.29	7.00	6.74	6.79	0.50	7.01	0.28	1.03	0.045	5.5E-01
Mtr.13801.1.S1_at	6.26	5.85	6.84	6.03	6.70	6.25	6.32	0.50	6.33	0.34	1.00	0.002	9.8E-01
Mtr.28226.1.S1_a_at	2.70	3.54	2.66	2.65	2.64	2.62	2.97	0.50	2.63	0.01	0.89	-0.171	3.1E-01
Mtr.14548.1.S1_at	2.90	3.76	2.88	2.88	2.88	2.88	3.18	0.50	2.88	0.00	0.91	-0.143	3.6E-01
Mtr.14644.1.S1_s_at	9.05	9.87	9.96	9.81	10.04	9.62	9.63	0.50	9.82	0.21	1.02	0.029	5.6E-01
Mtr.12154.1.S1_at	8.26	8.94	9.23	9.02	8.91	8.87	8.81	0.50	8.93	0.08	1.01	0.020	7.0E-01
Mtr.12150.1.S1_at	7.84	7.29	8.29	8.04	8.03	8.16	7.81	0.50	8.08	0.07	1.03	0.048	4.1E-01
Mtr.13818.1.S1_at	3.77	2.93	3.82	3.76	3.76	4.47	3.51	0.50	4.00	0.41	1.14	0.188	2.6E-01
Mtr.6024.1.S1_at	6.28	6.41	7.20	7.15	7.33	7.06	6.63	0.50	7.18	0.14	1.08	0.116	1.4E-01
Mtr.3012.1.S1_at	8.41	7.69	8.65	8.34	8.59	8.41	8.25	0.50	8.45	0.13	1.02	0.034	5.4E-01
Mtr.14710.1.S1_at	9.40	8.47	9.24	9.13	8.97	9.25	9.04	0.50	9.11	0.14	1.01	0.012	8.2E-01
Mtr.5882.1.S1_at	6.46	5.52	6.29	6.28	6.27	6.11	6.09	0.50	6.22	0.09	1.02	0.031	6.8E-01
Mtr.51011.1.S1_at	7.99	7.26	8.21	7.87	8.05	7.96	7.82	0.50	7.96	0.09	1.02	0.025	6.6E-01
Mtr.2027.1.S1_at	8.76	7.82	8.59	8.46	8.63	8.63	8.39	0.50	8.57	0.10	1.02	0.031	5.7E-01
Mtr.22645.1.S1_s_at	2.83	3.68	2.81	2.79	2.81	2.81	3.11	0.50	2.80	0.01	0.90	-0.148	3.5E-01
Mtr.11578.1.S1_at	8.32	7.33	7.71	7.77	8.07	7.69	7.79	0.50	7.84	0.20	1.01	0.011	8.6E-01
Mtr.21370.1.S1_x_at	11.49	10.92	11.91	12.39	12.28	11.86	11.44	0.50	12.17	0.28	1.06	0.089	9.1E-02
Mtr.14251.1.S1_at	9.04	9.74	8.78	8.89	8.68	8.93	9.19	0.50	8.83	0.14	0.96	-0.057	3.0E-01
Mtr.35619.1.S1_at	9.17	9.02	9.95	9.13	9.52	9.17	9.38	0.50	9.27	0.22	0.99	-0.017	7.4E-01
Mtr.51065.1.S1_at	11.33	10.34	10.73	11.11	11.07	11.16	10.80	0.50	11.11	0.04	1.03	0.042	3.3E-01
Mtr.15021.1.S1_at	8.42	7.50	8.30	7.76	8.38	7.93	8.07	0.50	8.03	0.32	0.99	-0.008	9.0E-01
Mtr.32920.1.S1_at	8.20	7.23	7.91	7.76	8.08	7.96	7.78	0.50	7.93	0.16	1.02	0.028	6.4E-01
Mtr.14421.1.S1_at	9.83	9.52	10.49	10.04	10.16	9.82	9.95	0.50	10.01	0.17	1.01	0.009	8.5E-01
Mtr.42516.1.S1_at	8.30	7.33	7.60	8.30	8.08	8.30	7.74	0.50	8.22	0.13	1.06	0.087	1.8E-01
Mtr.6726.1.S1_at	6.38	5.41	6.08	5.90	5.90	5.86	5.96	0.50	5.89	0.02	0.99	-0.016	8.3E-01
Mtr.43665.1.S1_s_at	8.86	8.80	9.69	9.12	9.00	8.71	9.12	0.50	8.94	0.21	0.98	-0.028	6.0E-01
Mtr.40976.1.S1_at	6.25	6.90	7.22	6.83	6.92	6.72	6.79	0.50	6.82	0.10	1.00	0.007	9.2E-01
Mtr.8180.1.S1_at	2.64	3.50	2.63	2.62	2.56	2.62	2.92	0.50	2.60	0.03	0.89	-0.168	3.3E-01
Mtr.34971.1.S1_s_at	5.65	4.77	5.62	4.82	4.40	4.43	5.35	0.50	4.55	0.24	0.85	-0.233	6.7E-02
Mtr.41698.1.S1_at	10.32	9.38	9.55	9.81	9.84	9.76	9.75	0.50	9.81	0.04	1.01	0.008	8.6E-01
Mtr.9564.1.S1_at	4.99	4.49	5.48	4.69	4.62	4.30	4.99	0.50	4.54	0.21	0.91	-0.136	2.2E-01
Mtr.6129.1.S1_s_at	8.81	8.38	9.37	8.99	8.77	8.63	8.85	0.50	8.80	0.19	0.99	-0.009	8.7E-01
Mtr.1766.1.S1_at	2.88	3.73	2.86	2.99	2.83	2.82	3.15	0.50	2.88	0.10	0.91	-0.129	4.1E-01
Mtr.23808.1.S1_at	2.79	3.65	2.78	2.78	2.72	2.77	3.07	0.50	2.75	0.03	0.90	-0.158	3.3E-01
Mtr.28547.1.S1_x_at	7.35	8.26	8.17	8.16	8.29	8.14	7.93	0.50	8.20	0.08	1.03	0.048	4.1E-01
Mtr.28194.1.S1_at	7.29	6.91	6.30	7.25	7.26	7.25	6.83	0.50	7.26	0.01	1.06	0.087	2.1E-01
Mtr.35047.1.S1_at	3.97	3.47	4.47	4.06	3.91	3.95	3.97	0.50	3.97	0.08	1.00	0.001	9.9E-01
Mtr.2127.1.S1_at	6.68	5.70	6.33	7.10	6.84	6.62	6.23	0.50	6.86	0.24	1.10	0.137	1.2E-01
Mtr.34798.1.S1_at	10.99	10.27	10.04	10.64	10.68	10.86	10.43	0.50	10.72	0.12	1.03	0.040	3.8E-01
Mtr.21720.1.S1_s_at	3.16	4.00	3.12	3.13	3.09	3.11	3.43	0.50	3.11	0.02	0.91	-0.140	3.3E-01
Mtr.35696.1.S1_at	4.11	3.12	3.60	3.86	3.79	3.86	3.61	0.50	3.84	0.04	1.06	0.087	4.8E-01
Mtr.29406.1.S1_at	7.01	6.09	6.88	6.81	6.84	6.78	6.66	0.50	6.81	0.03	1.02	0.032	6.3E-01
Mtr.44126.1.S1_at	9.28	8.35	9.12	8.98	9.00	8.88	8.92	0.50	8.96	0.07	1.00	0.006	9.0E-01
Mtr.36998.1.S1_at	2.83	3.66	2.78	2.77	2.76	2.74	3.09	0.50	2.76	0.01	0.89	-0.165	3.1E-01
Mtr.49963.1.S1_at	9.98	10.90	10.76	10.30	10.33	10.36	10.54	0.50	10.33	0.03	0.98	-0.029	5.0E-01
Mtr.37183.1.S1_at	4.50	3.54	4.22	5.05	6.26	5.30	4.09	0.50	5.54	0.64	1.36	0.438	3.6E-02
Mtr.2071.1.S1_at	7.50	6.61	6.66	7.11	7.03	7.57	6.92	0.50	7.23	0.29	1.04	0.063	4.0E-01
Mtr.37960.1.S1_at	10.46	9.64	10.54	10.42	10.57	10.39	10.21	0.50	10.46	0.10	1.02	0.034	4.5E-01
Mtr.39556.1.S1_at	7.72	7.60	8.51	8.13	8.34	7.74	7.94	0.50	8.07	0.30	1.02	0.023	7.2E-01
Mtr.42821.1.S1_at	7.91	6.98	7.75	8.08	7.90	7.90	7.55	0.50	7.96	0.10	1.05	0.077	2.3E-01
Mtr.41091.1.S1_at	9.99	9.00	9.58	9.75	9.70	9.78	9.52	0.50	9.74	0.04	1.02	0.033	4.8E-01
Mtr.44052.1.S1_at	10.56	9.59	9.92	10.37	10.11	10.43	10.02	0.50	10.30	0.17	1.03	0.039	4.1E-01

Mtr.5518.1.S1_at	2.92	2.94	3.79	2.87	2.82	2.88	3.22	0.50	2.86	0.03	0.89	-0.171	2.8E-01
Mtr.26798.1.S1_at	8.84	8.02	8.92	8.80	9.01	8.65	8.59	0.50	8.82	0.18	1.03	0.038	5.0E-01
Mtr.39575.1.S1_at	4.81	5.77	5.08	5.51	5.55	5.39	5.22	0.50	5.49	0.08	1.05	0.071	4.1E-01
Mtr.27974.1.S1_at	3.66	2.68	3.06	3.74	3.81	3.98	3.13	0.50	3.85	0.12	1.23	0.295	7.4E-02
Mtr.25599.1.S1_at	8.14	8.29	9.07	8.45	8.34	8.38	8.50	0.50	8.39	0.05	0.99	-0.019	7.2E-01
Mtr.7084.1.S1_at	8.05	7.06	7.67	8.35	8.64	8.45	7.59	0.50	8.48	0.15	1.12	0.159	4.1E-02
Mtr.36888.1.S1_at	3.66	3.33	4.31	3.27	4.12	3.21	3.77	0.50	3.54	0.51	0.94	-0.091	6.0E-01
Mtr.41070.1.S1_at	11.83	10.90	11.67	11.59	11.52	11.61	11.47	0.50	11.57	0.04	1.01	0.013	7.3E-01
Mtr.643.1.S1_s_at	7.72	7.09	8.06	7.37	6.62	7.14	7.62	0.50	7.04	0.38	0.92	-0.114	1.8E-01
Mtr.29065.1.S1_at	8.27	7.54	8.49	8.30	8.15	8.04	8.10	0.50	8.16	0.13	1.01	0.011	8.5E-01
Mtr.33460.1.S1_at	7.97	6.98	7.50	8.14	7.94	8.00	7.49	0.50	8.03	0.10	1.07	0.101	1.4E-01
Mtr.44746.1.S1_at	12.16	11.36	11.26	11.48	11.50	11.68	11.59	0.50	11.55	0.11	1.00	-0.005	9.0E-01
Mtr.27623.1.S1_x_at	8.66	8.16	9.15	8.69	9.31	8.46	8.66	0.50	8.82	0.44	1.02	0.027	6.9E-01
Mtr.4115.1.S1_at	2.49	2.39	3.29	3.76	3.88	3.05	2.72	0.50	3.56	0.45	1.31	0.386	9.6E-02
Mtr.12614.1.S1_at	10.88	10.05	10.94	11.02	11.30	11.02	10.62	0.50	11.11	0.16	1.05	0.065	1.8E-01
Mtr.20471.1.S1_at	7.49	6.58	6.68	6.60	6.54	6.48	6.92	0.50	6.54	0.06	0.95	-0.081	2.6E-01
Mtr.35501.1.S1_at	9.47	8.57	9.38	9.34	9.35	9.45	9.14	0.50	9.38	0.06	1.03	0.037	4.6E-01
Mtr.33654.1.S1_at	8.46	7.75	8.70	8.39	8.51	8.50	8.30	0.50	8.47	0.06	1.02	0.028	6.0E-01
Mtr.36871.1.S1_at	4.47	3.60	3.62	3.63	3.57	4.13	3.90	0.50	3.78	0.31	0.97	-0.046	7.3E-01
Mtr.44702.1.S1_at	7.79	6.85	7.58	7.47	7.37	7.68	7.41	0.50	7.51	0.16	1.01	0.019	7.6E-01
Mtr.8179.1.S1_at	3.10	3.99	3.92	3.13	3.27	3.57	3.67	0.50	3.32	0.23	0.91	-0.143	3.3E-01
Mtr.13608.1.S1_at	6.78	5.89	6.72	6.74	6.73	7.09	6.46	0.50	6.85	0.21	1.06	0.084	2.8E-01
Mtr.6983.1.S1_at	7.88	6.91	7.58	7.69	7.54	7.73	7.46	0.50	7.65	0.10	1.03	0.037	5.5E-01
Mtr.36094.1.S1_at	2.08	2.93	2.07	2.06	2.06	2.06	2.36	0.50	2.06	0.00	0.87	-0.194	3.6E-01
Mtr.40753.1.S1_s_at	9.55	8.57	9.19	8.93	9.07	8.98	9.10	0.50	8.99	0.07	0.99	-0.017	7.3E-01
Mtr.38856.1.S1_s_at	9.12	8.50	9.47	9.18	9.01	9.19	9.03	0.50	9.13	0.10	1.01	0.015	7.6E-01
Mtr.34126.1.S1_at	7.77	7.19	8.17	8.91	8.94	8.94	7.71	0.50	8.93	0.02	1.16	0.212	1.3E-02
Mtr.23151.1.S1_at	8.55	7.94	8.92	7.82	7.50	7.69	8.47	0.50	7.67	0.16	0.91	-0.143	5.7E-02
Mtr.40458.1.S1_at	12.23	11.32	11.44	11.51	11.52	11.59	11.66	0.50	11.54	0.04	0.99	-0.015	7.0E-01
Mtr.27726.1.S1_at	8.46	9.23	8.30	9.05	9.03	9.18	8.66	0.50	9.09	0.08	1.05	0.069	2.2E-01
Mtr.6878.1.S1_at	3.78	2.94	2.91	3.30	2.88	2.87	3.21	0.50	3.02	0.24	0.94	-0.089	5.8E-01
Mtr.35014.1.S1_at	6.66	5.85	6.76	6.74	6.62	6.82	6.42	0.50	6.73	0.10	1.05	0.067	3.5E-01
Mtr.32679.1.S1_at	9.78	9.17	10.15	9.83	10.08	9.46	9.70	0.50	9.79	0.32	1.01	0.013	8.1E-01
Mtr.101.1.S1_at	2.93	3.76	2.89	2.87	2.75	2.74	3.19	0.50	2.79	0.07	0.87	-0.196	2.3E-01
Mtr.13671.1.S1_s_at	9.39	8.49	9.31	9.13	9.21	9.13	9.07	0.50	9.16	0.05	1.01	0.015	7.6E-01
Mtr.28769.1.S1_at	8.25	7.50	8.43	8.31	8.70	8.22	8.06	0.50	8.41	0.25	1.04	0.061	3.4E-01
Mtr.32251.1.S1_s_at	8.16	7.30	8.16	8.25	8.53	8.31	7.87	0.50	8.36	0.15	1.06	0.087	1.8E-01
Mtr.6060.1.S1_at	7.96	7.18	8.09	8.30	8.02	8.13	7.74	0.49	8.15	0.14	1.05	0.074	2.4E-01
Mtr.44953.1.S1_at	8.64	7.65	8.13	8.76	8.68	8.82	8.14	0.49	8.76	0.07	1.08	0.105	1.0E-01
Mtr.3398.1.S1_at	3.44	2.65	3.55	3.58	4.07	3.58	3.21	0.49	3.74	0.28	1.16	0.219	1.8E-01
Mtr.42142.1.S1_at	7.96	7.21	8.15	7.84	7.83	7.95	7.77	0.49	7.87	0.07	1.01	0.018	7.5E-01
Mtr.41005.1.S1_at	10.50	9.51	10.04	10.20	10.10	10.25	10.02	0.49	10.18	0.08	1.02	0.024	6.0E-01
Mtr.35678.1.S1_at	11.94	11.15	12.07	11.91	11.97	11.83	11.72	0.49	11.91	0.07	1.02	0.023	5.5E-01
Mtr.9363.1.S1_s_at	4.26	3.40	4.25	4.87	4.27	4.64	3.97	0.49	4.59	0.30	1.16	0.210	1.4E-01
Mtr.9853.1.S1_at	5.89	5.60	6.57	6.17	6.15	6.06	6.02	0.49	6.13	0.06	1.02	0.025	7.4E-01
Mtr.33895.1.S1_at	6.73	6.05	7.01	6.86	6.99	6.72	6.60	0.49	6.86	0.14	1.04	0.056	4.3E-01
Mtr.40820.1.S1_at	8.66	7.77	7.85	9.08	9.79	9.13	8.09	0.49	9.33	0.40	1.15	0.206	2.8E-02
Mtr.43902.1.S1_s_at	9.65	8.74	9.53	9.51	9.20	9.39	9.30	0.49	9.36	0.16	1.01	0.009	8.5E-01
Mtr.47513.1.S1_at	3.09	3.93	3.07	3.03	2.94	3.43	3.36	0.49	3.13	0.26	0.93	-0.102	5.2E-01
Mtr.49029.1.S1_at	4.95	5.08	5.87	6.17	6.57	6.62	5.30	0.49	6.45	0.25	1.22	0.284	2.2E-02
Mtr.4966.1.S1_s_at	8.41	8.89	9.40	8.21	8.42	8.36	8.90	0.49	8.33	0.11	0.94	-0.095	1.2E-01
Mtr.13701.1.S1_at	8.08	7.19	8.00	8.05	8.19	7.77	7.76	0.49	8.01	0.21	1.03	0.046	4.7E-01
Mtr.2633.1.S1_at	4.22	4.14	3.32	8.26	8.39	8.28	3.89	0.49	8.31	0.07	2.13	1.094	1.1E-04
Mtr.1494.1.S1_at	9.85	9.01	8.98	9.73	9.81	9.98	9.28	0.49	9.84	0.12	1.06	0.084	1.3E-01
Mtr.3097.1.S1_at	7.97	7.00	7.30	7.59	7.70	7.69	7.42	0.49	7.66	0.06	1.03	0.045	4.6E-01
Mtr.38034.1.S1_at	13.41	12.45	12.74	12.69	12.57	12.75	12.87	0.49	12.67	0.09	0.98	-0.022	5.4E-01
Mtr.23454.1.S1_at	2.86	3.70	2.84	2.82	2.82	2.81	3.13	0.49	2.82	0.01	0.90	-0.152	3.3E-01
Mtr.25949.1.S1_at	2.40	3.24	2.37	2.36	2.36	2.36	2.67	0.49	2.36	0.00	0.88	-0.180	3.3E-01
Mtr.14232.1.S1_at	2.27	3.12	2.25	2.24	2.24	2.24	2.54	0.49	2.24	0.00	0.88	-0.182	3.5E-01
Mtr.38510.1.S1_at	8.75	7.85	8.66	8.39	8.40	8.12	8.42	0.49	8.31	0.16	0.99	-0.020	7.2E-01
Mtr.2477.1.S1_at	3.42	2.55	3.39	3.82	3.38	3.92	3.12	0.49	3.71	0.29	1.19	0.251	1.5E-01
Mtr.34449.1.S1_at	4.19	3.90	4.87	4.79	5.26	4.83	4.32	0.49	4.96	0.26	1.15	0.199	1.2E-01
Mtr.32317.1.S1_s_at	5.99	5.05	5.80	5.11	5.61	5.14	5.61	0.49	5.29	0.28	0.94	-0.087	3.7E-01
Mtr.1839.1.S1_at	9.90	9.23	10.19	10.12	10.19	10.01	9.77	0.49	10.11	0.09	1.03	0.048	3.2E-01
Mtr.33342.1.S1_at	12.85	11.86	12.33	13.90	13.77	13.76	12.35	0.49	13.81	0.08	1.12	0.162	7.1E-03
Mtr.7829.1.S1_at	2.28	3.12	2.26	2.28	2.28	2.48	2.55	0.49	2.35	0.11	0.92	-0.121	5.2E-01
Mtr.342.1.S1_at	6.16	5.51	6.48	6.53	6.55	6.51	6.05	0.49	6.53	0.02	1.08	0.110	1.7E-01
Mtr.19522.1.S1_at	8.91	8.05	8.90	8.99	8.93	8.78	8.62	0.49	8.90	0.11	1.03	0.046	3.9E-01
Mtr.24172.1.S1_at	2.99	3.84	2.98	2.89	2.97	2.96	3.27	0.49	2.94	0.04	0.90	-0.151	3.2E-01
Mtr.16642.1.S1_at	3.70	4.35	3.38	3.62	3.60	3.52	3.81	0.49	3.58	0.05	0.94	-0.090	4.7E-01
Mtr.44993.1.S1_at	7.52	6.76	7.68	8.20	8.04	8.00	7.32	0.49	8.08	0.11	1.10	0.142	6.0E-02
Mtr.45522.1.S1_at	9.45	8.51	9.26	8.54	8.43	8.47	9.07	0.49	8.48	0.06	0.93	-0.097	1.1E-01
Mtr.34600.1.S1_at	5.25	4.36	4.45	4.23	4.52	4.52	4.69	0.49	4.43	0.17	0.94	-0.083	4.3E-01
Mtr.42977.1.S1_at	8.44	7.47	8.10	8.14	7.77	8.07	8.00	0.49	7.99	0.20	1.00	-0.002	9.8E-01
Mtr.31095.1.S1_at	8.55	7.76	8.66	8.61	8.38	8.61	8.32	0.49	8.54	0.13	1.03	0.037	5.1E-01
Mtr.32850.1.S1_at	7.78	6.96	7.84	7.98	7.51	8.20	7.53	0.49	7.90	0.35	1.05	0.068	3.6E-01
Mtr.11813.1.S1_s_at	12.01	11.07	11.29	11.50	11.54	11.60	11.46	0.49	11.55	0.05	1.01	0.012	7.6E-01
Mtr.10265.1.S1_s_at	10.49	9.72	9.56	9.72	9.61	9.81	9.92	0.49	9.71	0.10	0.98	-0.031	5.1E-01
Mtr.9465.1.S1_at	7.64	6.65	7.18	7.09	7.23	7.01	7.16	0.49	7.11	0.11	0.99	-0.009	8.8E-01
Mtr.18078.1.S1_at	7.44	6.52	7.27	7.27	7.27	7.35	7.08	0.49	7.30	0.05	1.03	0.044	4.9E-01
Mtr.32146.1.S1_at	2.38	3.23	2.37	2.66	2.34	2.36	2.66	0.49	2.45	0.18	0.92	-0.115	5.4E-01
Mtr.6098.1.S1_at	3.52	2.66	3.51	3.24	3.82	4.20	3.23	0.49	3.76	0.48	1.16	0.217	2.6E-01
Mtr.2017.1.S1_at	4.18	3.20	3.76	3.05	3.11	3.05	3.72	0.49	3.07	0.04	0.83	-0.274	8.7E-02
Mtr.44022.1.S1_at	3.51	3.52	4.37	3.51	3.50	3.51	3.80	0.49	3.51	0.00	0.92	-0.117	3.6E-01
Mtr.44887.1.S1_at	9.81	8.83	9.42	9.41	9.56	9.44	9.35	0.49	9.47	0.08	1.01	0.018	7.1E-01
Mtr.4424.1.S1_at	2.67	1.84	1.80	1.80	1.80	1.80	2.10	0.49	1.80	0.00	0.86	-0.225	3.5E-01
Mtr.51303.1.S1_at	4.47	3.77	3.52	4.40	3.73	3.86	3.92	0.49	4.00	0.35	1.02	0.028	8.4E-01
Mtr.79.1.S1_at	9.12	9.99	9.95	9.11	9.17	9.17	9.69	0.49	9.15	0.03	0.94	-0.082	1.3E-01
Mtr.13746.1.S1_at	2.55	3.38	2.50	2.50	2.49	2.50	2.81	0.49	2.50	0.01	0.89	-0.171	3.3E-01
Mtr.30510.1.S1_at	4.35	5.21	5.19	5.34	5.44	5.15	4.92	0.49	5.31	0.15	1.08	0.110	2.6E-01
Mtr.36525.1.S1_at	7.41	6.81	7.79	7.91	8.24	7.95	7.34	0.49	8.04	0.18	1.10	0.131	8.2E-02
Mtr.23284.1.S1_at	7.01	6.04	6.71	8.15	8.71	8.58	6.59	0.49	8.48	0.29	1.29	0.364	4.6E-03
Mtr.22433.1.S1_at	6.87	7.46	7.85	6.90	7.41	7.02	7.40	0.49	7.11	0.27	0.96	-0.057	4.3E-01
Mtr.31008.1.S1_at	5.77	4.94	4.89	4.88	4.84	4.70	5.20	0.49	4.81	0.10	0.92	-0.114	2.5E-01
Mtr.8491.1.S1_at	3.31	2.46	3.31	3.30	3.80	3.30	3.02	0.49	3.47	0.29	1.15	0.197	2.5E-01
Mtr.13412.1.S1_at	4.29	4.86	3.87	8.81	8.75	8.70	4.34	0.49	8.75	0.05	2.02	1.012	1.0E-04
Mtr.11071.1.S1_at	4.22	3.26	3.56	3.84	3.63	3.84	3.68	0.49	3.77	0.12	1.02	0.035	7.8E-01
Mtr.38628.1.S1_at	8.51	8.69	9.44	8.96	9.06	8.95	8.88	0.49	8.99	0.07	1.01	0.018	7.2E-01
Mtr.19316.1.S1_at	2.51	3.35	2.50	2.50	2.49	2.49	2.79	0.49	2.49	0.00	0.90	-0.159	3.6E-01
Mtr.5711.1.S1_at	9.04	8.11	8.85	9.07	8.97	9.17	8.67	0.49	9.07	0.10	1.05	0.065	2.4E-01
Mtr.49268.1.S1_at	3.36	4.20	3.34	3.35	3.27	3.25	3.64	0.49	3.29	0.05	0.90	-0.145	2.9E-01
Mtr.32008.1.S1_at	8.81	7.93	8.75	8.15	8.40	8.16	8.50	0.49	8.24	0.14	0.97	-0.045	4.3E-01
Mtr.11349.1.S1_at	3.46	3.52	2.64	4.74	5.38	4.51	3.21	0.49	4.88	0.45	1.52	0.603	1.2E-02
Mtr.42563.1.S1_at	8.00	7.96	8.83	8.31	8.25	8.28	8.27	0.49	8.28	0.03	1.00	0.003	9.6E-01
Mtr.11787.1.S1_at	7.90	7.12	8.03	7.60	7.87	7.65	7.68	0.49	7.71	0.15	1.00	0.004	9.5E-01
Mtr.33441.1.S1_s_at	9.00	8.30	8.05	8.88	8.96	8.99	8.45	0.49	8.94	0.06	1.06	0.082	1.6E-01
Mtr.6055.1.S1_at	8.38	7.92	8.90	8.48	8.34	8.50	8.40	0.49	8.44	0.09	1.00	0.007	8.9E-01
Mtr.25702.1.S1_at	2.55	3.41	2.56	2.54	2.54	2.53	2.84	0.49	2.53	0.01	0.89	-0.165	3.4E-01
Mtr.38363.1.S1_at	10.96	10.01	10.26	10.49	10.26	10.56	10.41	0.49	10.43	0.15	1.00	0.003	9.4E-01
Mtr.2283.1.S1_at	5.47	4.49	5.08	5.08	5.07	5.06	5.02	0.49	5.07	0.01	1.01	0.014	8.7E-01
Mtr.45975.1.S1_at	7.90	6.92	7.35	6.77	6.66	6.79	7.39	0.49	6.74	0.07	0.91	-0.133	8.6E-02
Mtr.27314.1.S1_at	4.86	5.69	4.83	4.82	4.69	4.81	5.13	0.49	4.77	0.08	0.93	-0.103	2.9E-01
Mtr.11086.1.S1_at	12.54	11.60	11.81	12.11	11.96	12.20	11.98	0.49	12.09	0.12	1.01	0.012	7.4E-01
Mtr.11454.1.S1_at	8.54	8.83	9.50	7.98	8.34	7.90	8.96	0.49	8.08	0.24	0.90	-0.149	4.9E-02
Mtr.13783.1.S1_at	7.92	8.82	8.04	8.15	8.66	8.13	8.26	0.49	8.31	0.30	1.01	0.009	8.8E-01
Mtr.4829.1.S1_s_at	7.73	7.22	8.21	8.04	8.08	7.70	7.72	0.49	7.94	0.21	1.03	0.040	5.2E-01
Mtr.26767.1.S1_s_at	3.08	3.93	3.07	3.02	3.23	2.96	3.36	0.49	3.07	0.14	0.91	-0.131	3.8E-01
Mtr.12741.1.S1_at	9.30	8.63	9.59	9.20	9.19	9.22	9.17	0.49	9.20	0.01	1.00	0.005	9.1E-01
Mtr.43730.1.S1_at	12.94	11.96	12.37	12.52	12.67	12.54	12.42	0.49	12.58	0.08	1.01	0.018	6.1E-01
Mtr.35272.1.S1_at	3.27	4.07	3.17	3.15	3.14	3.15	3.50	0.49	3.15	0.00	0.90	-0.154	2.8E-01
Mtr.50733.1.S1_at	4.66	4.60	5.48	6.32	6.60	6.92	4.91	0.49	6.61	0.30	1.35	0.428	7.0E-03
Mtr.31947.1.S1_s_at	6.09	5.13	5.79	6.43	8.67	8.09	5.67	0.49	7.73	1.17	1.36	0.447	4.8E-02
Mtr.28068.1.S1_at	7.82	7.44	8.42	8.15	8.12	8.12	7.89	0.49	8.13	0.01	1.03	0.043	4.5E-01
Mtr.35565.1.S1_at	7.20	6.36	6.35	6.34	6.34	6.34	6.64	0.49	6.34	0.00	0.96	-0.065	3.6E-01
Mtr.49987.1.S1_at	10.67	9.71	10.00	10.32	9.91	10.24	10.13	0.49	10.16	0.22	1.00	0.004	9.4E-01
Mtr.42805.1.S1_at	4.83	4.84	5.69	4.81	4.77	4.81	5.12	0.49	4.79	0.03	0.94	-0.094	3.2E-01
Mtr.20113.1.S1_at	3.56	4.38	3.51	3.48	3.42	3.37	3.82	0.49	3.43	0.06	0.90	-0.156	2.4E-01
Mtr.2062.1.S1_at	9.97	8.99	9.47	9.51	9.45	9.40	9.48	0.49	9.46	0.06	1.00	-0.003	9.5E-01
Mtr.448.1.S1_at	2.81	3.65	2.78	2.78	2.73	2.76	3.08	0.49	2.76	0.02	0.90	-0.159	3.2E-01
Mtr.34213.1.S1_at	7.26	6.29	6.92	6.88	7.59	7.18	6.82	0.49	7.22	0.36	1.06	0.080	3.3E-01
Mtr.39179.1.S1_at	8.83	9.31	9.81	9.41	9.96	9.62	9.32	0.49	9.66	0.28	1.04	0.053	3.5E-01

Mtr.34846.1.S1_at	5.87	6.69	6.74	6.55	6.55	6.58	6.43	0.49	6.56	0.01	1.02	0.029	6.7E-01
Mtr.38432.1.S1_at	10.01	9.03	9.48	8.94	8.58	8.60	9.51	0.49	8.71	0.20	0.92	-0.126	6.0E-02
Mtr.11149.1.S1_at	9.11	8.45	9.40	9.53	9.57	9.56	8.99	0.49	9.55	0.02	1.06	0.088	1.2E-01
Mtr.31219.1.S1_at	8.66	8.69	9.53	8.97	9.48	9.10	8.96	0.49	9.18	0.26	1.02	0.035	5.3E-01
Mtr.43316.1.S1_at	10.57	9.94	10.90	10.58	10.49	10.47	10.47	0.49	10.51	0.06	1.00	0.006	8.9E-01
Mtr.44476.1.S1_at	8.55	7.71	7.68	7.73	8.35	7.87	7.98	0.49	7.98	0.33	1.00	0.000	1.0E+00
Mtr.2166.1.S1_at	8.06	7.40	8.36	7.97	7.84	7.76	7.94	0.49	7.86	0.11	0.99	-0.014	8.0E-01
Mtr.14371.1.S1_at	2.52	3.41	2.62	2.57	2.49	2.56	2.85	0.49	2.54	0.04	0.89	-0.166	3.4E-01
Mtr.11053.1.S1_at	10.82	10.00	9.94	10.49	11.11	10.90	10.25	0.49	10.83	0.32	1.06	0.080	1.6E-01
Mtr.33330.1.S1_at	7.87	7.47	8.45	7.98	8.00	8.02	7.93	0.49	8.00	0.02	1.01	0.012	8.2E-01
Mtr.1097.1.S1_at	9.49	8.70	9.60	9.71	9.73	9.65	9.26	0.49	9.70	0.04	1.05	0.066	2.0E-01
Mtr.33315.1.S1_at	7.73	6.98	7.91	7.76	8.05	7.87	7.54	0.49	7.89	0.15	1.05	0.065	3.0E-01
Mtr.41089.1.S1_at	11.44	11.21	12.15	11.59	11.66	11.59	11.60	0.49	11.61	0.04	1.00	0.001	9.7E-01
Mtr.30353.1.S1_at	1.86	1.89	2.73	1.85	1.85	1.85	2.16	0.49	1.85	0.00	0.85	-0.226	3.3E-01
Mtr.50829.1.S1_at	9.14	8.65	9.63	9.44	9.36	9.32	9.14	0.49	9.37	0.06	1.03	0.036	4.6E-01
Mtr.5267.1.S1_at	5.59	4.69	5.49	5.57	5.63	5.74	5.26	0.49	5.65	0.09	1.07	0.104	2.4E-01
Mtr.40907.1.S1_at	2.98	3.81	2.94	2.91	2.88	2.87	3.24	0.49	2.89	0.03	0.89	-0.169	2.7E-01
Mtr.260.1.S1_at	2.79	3.63	2.77	2.75	2.69	2.98	3.06	0.49	2.81	0.16	0.92	-0.124	4.4E-01
Mtr.35775.1.S1_at	8.29	7.37	8.12	8.09	8.12	7.71	7.93	0.49	7.97	0.23	1.01	0.008	9.0E-01
Mtr.20277.1.S1_at	5.98	6.63	6.94	6.83	6.00	6.01	6.51	0.49	6.28	0.48	0.96	-0.053	5.9E-01
Mtr.40028.1.S1_at	12.81	12.21	11.84	12.81	13.09	13.07	12.29	0.49	12.99	0.16	1.06	0.080	7.7E-02
Mtr.27042.1.S1_at	8.31	7.33	7.86	8.19	8.11	8.15	7.83	0.49	8.15	0.04	1.04	0.058	3.2E-01
Mtr.13812.1.S1_at	9.63	8.77	9.61	9.50	9.54	9.53	9.34	0.49	9.53	0.02	1.02	0.029	5.5E-01
Mtr.41241.1.S1_at	8.55	7.75	8.65	8.77	8.73	8.73	8.32	0.49	8.75	0.02	1.05	0.072	2.1E-01
Mtr.31664.1.S1_at	7.03	6.59	7.57	7.52	7.23	7.11	7.06	0.49	7.29	0.21	1.03	0.046	5.0E-01
Mtr.22367.1.S1_at	2.93	3.73	2.83	2.82	2.82	2.82	3.16	0.49	2.82	0.00	0.89	-0.166	2.9E-01
Mtr.7744.1.S1_at	7.40	6.73	7.68	7.53	7.92	7.40	7.27	0.49	7.62	0.27	1.05	0.067	3.5E-01
Mtr.6589.1.S1_at	5.40	4.50	4.61	5.14	5.23	4.83	4.84	0.49	5.07	0.21	1.05	0.067	5.0E-01
Mtr.39908.1.S1_at	7.69	6.71	7.18	7.19	7.45	7.04	7.19	0.49	7.23	0.21	1.00	0.007	9.2E-01
Mtr.13476.1.S1_at	8.41	7.57	8.43	8.24	8.42	8.32	8.14	0.49	8.33	0.09	1.02	0.033	5.5E-01
Mtr.31464.1.S1_at	9.42	9.29	10.19	9.54	9.74	9.46	9.63	0.49	9.58	0.14	0.99	-0.008	8.6E-01
Mtr.14228.1.S1_at	2.80	3.37	3.77	4.20	4.87	4.57	3.31	0.49	4.55	0.33	1.37	0.456	2.3E-02
Mtr.39111.1.S1_at	9.06	8.11	8.80	8.70	8.74	8.78	8.66	0.49	8.74	0.04	1.01	0.013	7.9E-01
Mtr.47159.1.S1_s_at	7.01	6.09	6.85	7.29	7.42	7.16	6.65	0.49	7.29	0.13	1.10	0.133	9.3E-02
Mtr.30113.1.S1_at	6.84	6.48	7.45	6.74	7.23	6.81	6.92	0.49	6.93	0.26	1.00	0.000	1.0E+00
Mtr.43705.1.S1_at	11.47	10.54	10.75	11.07	10.98	11.10	10.92	0.49	11.05	0.06	1.01	0.017	6.7E-01
Mtr.4460.1.S1_at	8.21	8.59	9.19	8.86	9.14	8.71	8.66	0.49	8.90	0.22	1.03	0.039	4.8E-01
Mtr.44636.1.S1_at	7.44	6.57	7.38	7.58	7.55	7.51	7.13	0.49	7.55	0.03	1.06	0.082	2.1E-01
Mtr.44249.1.S1_at	8.50	7.67	8.54	8.21	8.39	8.31	8.24	0.49	8.30	0.09	1.01	0.012	8.3E-01
Mtr.30250.1.S1_at	3.27	4.03	3.11	3.16	3.11	3.06	3.47	0.49	3.11	0.05	0.90	-0.158	2.7E-01
Mtr.42724.1.S1_at	8.16	7.25	8.03	8.29	8.01	8.25	7.81	0.49	8.18	0.15	1.05	0.068	2.8E-01
Mtr.41783.1.S1_at	8.93	8.21	9.14	8.78	8.96	8.75	8.76	0.49	8.83	0.12	1.01	0.011	8.2E-01
Mtr.39439.1.S1_at	6.58	5.83	6.75	6.29	6.77	6.55	6.39	0.49	6.54	0.24	1.02	0.034	6.6E-01
Mtr.34634.1.S1_at	10.34	9.47	10.30	10.35	10.36	10.61	10.04	0.49	10.44	0.15	1.04	0.056	2.5E-01
Mtr.37448.1.S1_at	11.40	11.48	12.29	11.52	11.43	11.39	11.72	0.49	11.45	0.07	0.98	-0.035	3.8E-01
Mtr.7873.1.S1_at	11.55	10.65	10.76	11.16	11.30	11.13	10.99	0.49	11.20	0.09	1.02	0.027	5.1E-01
Mtr.28181.1.S1_at	6.06	5.20	6.03	5.85	6.12	5.84	5.76	0.49	5.94	0.16	1.03	0.044	5.8E-01
Mtr.24090.1.S1_at	9.98	10.35	10.95	9.89	10.01	10.01	10.43	0.49	9.97	0.07	0.96	-0.064	1.9E-01
Mtr.33026.1.S1_at	8.35	7.38	7.91	8.19	8.14	8.15	7.88	0.49	8.16	0.03	1.04	0.050	3.8E-01
Mtr.28295.1.S1_at	7.39	6.41	6.85	6.90	7.44	7.13	6.88	0.49	7.16	0.27	1.04	0.056	4.4E-01
Mtr.43257.1.S1_at	12.27	12.08	11.34	11.83	11.74	11.79	11.90	0.49	11.79	0.04	0.99	-0.014	7.1E-01
Mtr.9714.1.S1_at	8.09	7.12	7.65	7.77	7.84	7.86	7.62	0.49	7.82	0.04	1.03	0.038	5.1E-01
Mtr.23754.1.S1_at	8.61	7.71	8.51	8.46	8.52	8.45	8.27	0.49	8.48	0.04	1.02	0.035	5.1E-01
Mtr.43869.1.S1_at	8.71	7.84	8.65	8.75	8.53	8.76	8.40	0.49	8.68	0.13	1.03	0.048	3.9E-01
Mtr.25627.1.S1_at	2.43	3.27	2.42	2.40	2.40	2.39	2.71	0.49	2.40	0.01	0.89	-0.176	3.3E-01
Mtr.22801.1.S1_at	3.75	4.02	4.70	3.95	3.95	3.95	4.16	0.49	3.95	0.00	0.95	-0.073	5.1E-01
Mtr.8526.1.S1_at	9.13	8.27	8.29	9.34	9.51	9.45	8.56	0.49	9.44	0.09	1.10	0.140	3.8E-02
Mtr.45537.1.S1_at	6.93	6.17	6.01	6.21	6.42	6.24	6.37	0.49	6.29	0.12	0.99	-0.018	8.0E-01
Mtr.40819.1.S1_at	11.61	11.29	10.65	11.13	11.11	11.27	11.18	0.49	11.17	0.08	1.00	-0.001	9.7E-01
Mtr.46423.1.S1_s_at	5.73	5.21	6.19	5.51	5.75	5.07	5.71	0.49	5.44	0.35	0.95	-0.070	4.8E-01
Mtr.10136.1.S1_at	7.99	7.14	7.99	7.64	7.74	7.53	7.71	0.49	7.64	0.10	0.99	-0.013	8.2E-01
Mtr.3098.1.S1_s_at	9.37	8.49	9.30	9.48	9.38	9.23	9.05	0.49	9.37	0.12	1.03	0.049	3.4E-01
Mtr.250.1.S1_at	10.17	9.30	10.13	9.73	9.65	9.65	9.87	0.49	9.67	0.05	0.98	-0.028	5.3E-01
Mtr.22964.1.S1_at	2.71	3.54	2.67	2.65	2.62	2.63	2.97	0.49	2.64	0.01	0.89	-0.174	3.0E-01
Mtr.30722.1.S1_at	10.49	9.63	10.47	10.43	10.72	10.56	10.20	0.49	10.57	0.14	1.04	0.052	2.8E-01
Mtr.32658.1.S1_at	8.23	7.44	8.34	8.21	7.87	8.06	8.00	0.49	8.04	0.17	1.00	0.007	9.0E-01
Mtr.13006.1.S1_at	9.62	8.79	9.66	9.46	9.55	9.23	9.36	0.49	9.41	0.17	1.01	0.009	8.6E-01
Mtr.30718.1.S1_at	3.11	3.91	3.03	3.08	2.98	3.28	3.35	0.49	3.11	0.16	0.93	-0.108	4.6E-01
Mtr.19268.1.S1_at	11.06	11.07	11.91	11.63	11.69	11.56	11.35	0.49	11.63	0.06	1.02	0.035	3.8E-01
Mtr.40900.1.S1_at	3.82	3.53	2.87	3.46	3.38	3.46	3.41	0.49	3.44	0.05	1.01	0.013	9.2E-01
Mtr.47709.1.S1_at	7.53	6.93	7.89	7.66	7.66	7.21	7.45	0.49	7.51	0.26	1.01	0.011	8.7E-01
Mtr.19873.1.S1_at	2.88	3.71	2.84	2.82	2.81	2.81	3.14	0.49	2.82	0.00	0.90	-0.159	3.1E-01
Mtr.6888.1.S1_at	8.35	7.48	8.29	8.30	8.27	8.49	8.04	0.49	8.36	0.12	1.04	0.056	3.3E-01
Mtr.29414.1.S1_at	3.30	2.45	3.30	3.42	3.29	3.29	3.02	0.49	3.33	0.08	1.11	0.145	3.3E-01
Mtr.43942.1.S1_s_at	10.44	11.20	11.35	11.25	11.34	11.00	11.00	0.49	11.20	0.18	1.02	0.026	5.4E-01
Mtr.12234.1.S1_s_at	8.83	8.01	8.88	8.99	8.94	8.98	8.57	0.49	8.97	0.02	1.05	0.065	2.3E-01
Mtr.2731.1.S1_at	6.51	5.67	6.52	6.36	6.50	6.34	6.23	0.49	6.40	0.09	1.03	0.039	5.8E-01
Mtr.9967.1.S1_at	7.29	7.18	8.08	7.15	7.28	6.86	7.52	0.49	7.09	0.22	0.94	-0.084	2.4E-01
Mtr.42762.1.S1_at	7.35	6.81	7.78	7.42	7.46	7.52	7.31	0.49	7.46	0.05	1.02	0.029	6.3E-01
Mtr.2463.1.S1_s_at	7.89	6.96	7.17	8.28	8.06	8.43	7.34	0.49	8.26	0.19	1.12	0.169	3.9E-02
Mtr.50300.1.S1_s_at	9.85	8.88	9.29	9.65	9.72	9.65	9.34	0.49	9.67	0.04	1.04	0.050	3.1E-01
Mtr.24000.1.S1_at	8.49	7.78	8.72	8.59	8.64	8.64	8.33	0.49	8.62	0.03	1.04	0.050	3.6E-01
Mtr.10006.1.S1_at	10.08	9.24	9.24	9.76	9.62	9.88	9.52	0.49	9.76	0.13	1.02	0.035	4.6E-01
Mtr.40331.1.S1_at	12.16	11.19	11.72	11.85	11.86	12.12	11.69	0.49	11.94	0.15	1.02	0.030	4.5E-01
Mtr.35028.1.S1_at	8.08	7.11	7.50	7.69	7.82	8.17	7.56	0.49	7.89	0.25	1.04	0.062	3.6E-01
Mtr.31755.1.S1_at	8.04	7.43	8.39	8.10	8.21	8.13	7.95	0.49	8.15	0.05	1.02	0.035	5.3E-01
Mtr.38770.1.S1_at	3.75	2.90	3.74	3.74	3.74	3.86	3.46	0.49	3.78	0.07	1.09	0.126	3.3E-01
Mtr.12718.1.S1_at	10.53	11.14	11.50	11.13	11.12	11.08	11.06	0.49	11.11	0.03	1.00	0.007	8.5E-01
Mtr.29956.1.S1_at	5.73	4.76	5.25	5.14	4.87	4.71	5.25	0.49	4.91	0.22	0.93	-0.098	3.3E-01
Mtr.36994.1.S1_s_at	2.57	2.80	3.51	2.53	2.60	2.74	2.96	0.49	2.62	0.11	0.89	-0.174	3.1E-01
Mtr.10099.1.S1_s_at	6.31	5.40	6.16	6.46	6.18	6.64	5.96	0.49	6.43	0.23	1.08	0.109	2.1E-01
Mtr.7281.1.S1_at	8.89	8.02	8.84	8.46	8.57	8.41	8.58	0.49	8.48	0.08	0.99	-0.017	7.4E-01
Mtr.49772.1.S1_at	7.52	6.62	7.40	7.64	7.54	7.47	7.18	0.49	7.55	0.08	1.05	0.073	2.6E-01
Mtr.50010.1.S1_s_at	4.71	3.87	4.72	4.69	5.61	4.61	4.43	0.49	4.97	0.56	1.12	0.165	2.8E-01
Mtr.43176.1.S1_at	7.34	7.22	6.44	7.18	6.91	6.90	7.00	0.49	7.00	0.16	1.00	0.000	1.0E+00
Mtr.28860.1.S1_at	2.85	3.69	2.84	2.83	2.83	2.82	3.12	0.49	2.83	0.00	0.90	-0.145	3.5E-01
Mtr.27497.1.S1_at	5.65	4.80	5.64	5.68	5.63	5.63	5.36	0.49	5.65	0.03	1.05	0.075	3.7E-01
Mtr.49547.1.S1_at	8.02	7.85	8.77	8.38	8.25	8.36	8.22	0.49	8.33	0.07	1.01	0.020	7.1E-01
Mtr.31091.1.S1_at	8.05	7.20	8.03	8.03	8.29	8.09	7.76	0.49	8.14	0.14	1.05	0.068	2.7E-01
Mtr.7269.1.S1_at	9.11	8.41	9.34	9.11	9.16	9.03	8.95	0.49	9.10	0.06	1.02	0.024	6.3E-01
Mtr.46844.1.S1_at	3.47	2.82	2.52	2.50	2.49	2.47	2.94	0.49	2.48	0.01	0.85	-0.241	1.8E-01
Mtr.26803.1.S1_at	7.71	7.77	8.58	7.60	8.32	7.72	8.02	0.49	7.88	0.39	0.98	-0.026	7.1E-01
Mtr.9622.1.S1_at	2.18	3.02	2.17	2.16	2.16	2.16	2.45	0.49	2.16	0.00	0.88	-0.182	3.6E-01
Mtr.1600.1.S1_s_at	3.99	4.13	4.90	3.88	4.18	3.55	4.34	0.49	3.87	0.31	0.89	-0.166	2.3E-01
Mtr.31764.1.S1_at	8.90	8.74	9.65	8.84	8.99	8.73	9.10	0.49	8.85	0.13	0.97	-0.039	4.5E-01
Mtr.12339.1.S1_at	12.77	12.08	11.84	12.04	11.88	12.17	12.23	0.49	12.03	0.14	0.98	-0.024	5.3E-01
Mtr.35651.1.S1_at	6.99	6.23	7.13	7.17	7.19	6.86	6.78	0.49	7.07	0.19	1.04	0.060	3.9E-01
Mtr.31264.1.S1_at	4.05	3.15	3.91	3.34	3.45	3.63	3.70	0.49	3.47	0.15	0.94	-0.093	4.8E-01
Mtr.12270.1.S1_at	7.96	7.28	8.22	8.01	8.08	8.00	7.82	0.49	8.03	0.04	1.03	0.038	5.0E-01
Mtr.21268.1.S1_x_at	2.98	3.82	2.97	2.94	2.89	2.93	3.26	0.49	2.92	0.03	0.90	-0.158	3.0E-01
Mtr.34967.1.S1_at	9.75	9.04	8.82	9.08	9.14	9.15	9.20	0.49	9.12	0.04	0.99	-0.013	7.8E-01
Mtr.12057.1.S1_at	9.03	8.58	8.06	8.56	8.75	8.62	8.56	0.49	8.64	0.09	1.01	0.015	7.8E-01
Mtr.29147.1.S1_at	6.52	5.74	6.63	6.44	6.41	6.35	6.30	0.49	6.40	0.05	1.02	0.023	7.3E-01
Mtr.41637.1.S1_at	6.61	5.76	6.59	4.83	5.01	5.08	6.32	0.49	4.98	0.13	0.79	-0.345	9.9E-03
Mtr.1429.1.S1_s_at	6.43	5.46	5.94	5.78	5.92	5.70	5.94	0.49	5.80	0.11	0.98	-0.035	6.5E-01
Mtr.51554.1.S1_at	5.73	4.76	5.24	5.74	5.30	5.76	5.25	0.49	5.60	0.26	1.07	0.093	3.3E-01
Mtr.22854.1.S1_at	7.13	7.18	8.00	7.37	7.65	7.61	7.44	0.49	7.55	0.15	1.01	0.021	7.3E-01
Mtr.791.1.S1_at	9.52	8.56	9.14	9.26	9.21	9.36	9.07	0.49	9.27	0.08	1.02	0.032	5.2E-01
Mtr.44270.1.S1_at	13.32	13.34	14.17	13.55	13.62	13.33	13.61	0.49	13.50	0.15	0.99	-0.011	7.4E-01
Mtr.39870.1.S1_at	8.69	7.74	8.38	8.63	8.67	8.83	8.27	0.49	8.71	0.10	1.05	0.075	2.0E-01
Mtr.5877.1.S1_at	6.97	6.23	7.14	6.75	6.65	6.83	6.78	0.49	6.74	0.09	0.99	-0.008	9.1E-01
Mtr.19367.1.S1_at	7.29	6.60	7.53	8.51	9.19	8.59	7.14	0.49	8.76	0.37	1.23	0.295	1.0E-02
Mtr.46240.1.S1_at	3.39	4.21	3.36	3.35	3.32	3.37	3.65	0.49	3.34	0.02	0.92	-0.127	3.4E-01
Mtr.4984.1.S1_at	8.94	8.14	9.02	8.93	9.20	8.92	8.70	0.49	9.02	0.16	1.04	0.052	3.4E-01
Mtr.12293.1.S1_at	9.69	8.82	8.88	8.89	9.14	9.11	9.13	0.49	9.05	0.13	0.99	-0.013	7.9E-01
Mtr.35639.1.S1_s_at	10.90	11.58	11.84	11.49	11.51	11.29	11.44	0.49	11.43	0.12	1.00	-0.001	9.8E-01
Mtr.44537.1.S1_at	7.54	6.70	7.54	7.69	7.83	7.94	7.26	0.49	7.82	0.12	1.08	0.108	1.2E-01
Mtr.42408.1.S1_at	6.59	5.62	6.16	6.66	6.11	6.44	6.12	0.49	6.41	0.28	1.05	0.065	4.3E-01
Mtr.667.1.S1_at	3.59	3.92	2.97	3.98	3.47	3.46	3.49	0.49	3.64	0.30	1.04	0.057	6.9E-01
Mtr.22203.1.S1_s_at	2.83	3.65	2.79	2.79	2.78	2.78	3.09	0.49	2.78	0.01	0.90	-0.151	3.3E-01
Mtr.7467.1.S1_s_at	6.42	5.47	6.16	6.21	6.26	6.21	6.02	0.49	6.23	0.03	1.04	0.050	4.9E-01

Mtr.20736.1.S1_at	2.49	3.35	2.53	2.52	2.55	2.55	2.79	0.49	2.54	0.02	0.91	-0.133	4.3E-01
Mtr.17974.1.S1_x_at	6.65	6.00	6.94	7.04	7.31	7.16	6.53	0.49	7.17	0.13	1.10	0.134	9.4E-02
Mtr.27938.1.S1_s_at	8.82	8.07	8.98	9.23	9.14	9.07	8.62	0.49	9.15	0.08	1.06	0.085	1.4E-01
Mtr.13471.1.S1_at	9.69	8.75	9.44	10.01	10.30	10.15	9.29	0.49	10.15	0.14	1.09	0.128	4.2E-02
Mtr.37837.1.S1_s_at	8.79	8.09	9.02	8.62	8.69	8.79	8.63	0.49	8.70	0.08	1.01	0.011	8.2E-01
Mtr.40797.1.S1_at	10.20	9.32	10.12	9.93	10.05	10.03	9.88	0.49	10.00	0.06	1.01	0.018	6.8E-01
Mtr.31724.1.S1_at	9.64	9.24	10.20	10.07	10.09	10.26	9.69	0.49	10.14	0.11	1.05	0.065	1.9E-01
Mtr.34430.1.S1_at	9.50	10.29	9.41	9.74	9.52	9.65	9.73	0.49	9.63	0.11	0.99	-0.015	7.5E-01
Mtr.41076.1.S1_at	12.38	11.47	12.22	12.11	12.07	12.08	12.02	0.49	12.09	0.02	1.01	0.007	8.4E-01
Mtr.28783.1.S1_at	3.03	4.00	3.56	3.55	4.16	3.49	3.53	0.49	3.73	0.37	1.06	0.080	6.0E-01
Mtr.12162.1.S1_s_at	5.59	6.52	6.28	6.35	6.63	6.29	6.13	0.49	6.43	0.18	1.05	0.068	3.8E-01
Mtr.1695.1.S1_at	7.23	6.27	6.70	7.26	6.82	7.25	6.73	0.49	7.11	0.25	1.06	0.079	2.9E-01
Mtr.24503.1.S1_at	2.24	3.07	2.22	2.21	2.21	2.21	2.51	0.49	2.21	0.00	0.88	-0.183	3.5E-01
Mtr.41485.1.S1_at	7.57	7.76	8.49	7.85	7.83	7.69	7.94	0.49	7.79	0.09	0.98	-0.028	6.1E-01
Mtr.16728.1.S1_at	11.75	12.30	12.71	12.51	12.38	12.31	12.25	0.49	12.40	0.10	1.01	0.017	6.3E-01
Mtr.45150.1.S1_at	9.30	8.41	9.18	8.93	9.18	8.91	8.96	0.49	9.01	0.15	1.01	0.007	8.8E-01
Mtr.13051.1.S1_at	9.40	8.58	9.44	9.42	9.52	9.46	9.14	0.49	9.47	0.05	1.04	0.051	3.1E-01
Mtr.15669.1.S1_at	2.08	2.91	2.06	2.05	2.05	2.05	2.35	0.49	2.05	0.00	0.87	-0.194	3.5E-01
Mtr.44509.1.S1_at	10.09	11.05	10.71	10.24	10.65	10.25	10.62	0.49	10.38	0.24	0.98	-0.032	5.0E-01
Mtr.35046.1.S1_at	3.33	4.16	3.31	3.35	3.32	3.24	3.60	0.48	3.31	0.06	0.92	-0.124	3.5E-01
Mtr.13108.1.S1_at	10.23	9.26	9.76	9.90	9.80	10.02	9.75	0.48	9.91	0.11	1.02	0.023	6.1E-01
Mtr.1075.1.S1_at	3.13	3.97	3.12	3.12	3.12	3.11	3.41	0.48	3.12	0.00	0.91	-0.128	3.6E-01
Mtr.11487.1.S1_at	2.95	2.30	3.25	2.96	3.40	2.77	2.84	0.48	3.04	0.32	1.07	0.103	5.7E-01
Mtr.40532.1.S1_s_at	9.98	9.09	9.21	9.61	9.79	9.93	9.43	0.48	9.78	0.16	1.04	0.052	3.0E-01
Mtr.24506.1.S1_at	4.34	5.22	5.13	5.42	5.28	5.12	4.90	0.48	5.27	0.15	1.08	0.107	2.7E-01
Mtr.44243.1.S1_at	3.07	4.02	3.68	4.34	3.55	3.58	3.59	0.48	3.83	0.45	1.07	0.093	5.6E-01
Mtr.24695.1.S1_at	6.32	5.44	6.23	6.31	6.40	6.67	6.00	0.48	6.46	0.18	1.08	0.107	2.0E-01
Mtr.25647.1.S1_at	2.54	3.37	2.52	2.48	2.48	2.46	2.81	0.48	2.48	0.01	0.88	-0.183	3.0E-01
Mtr.14582.1.S1_at	7.29	6.40	7.18	7.10	6.92	7.11	6.96	0.48	7.05	0.11	1.01	0.018	7.8E-01
Mtr.29561.1.S1_at	11.09	10.13	10.49	10.76	10.84	10.79	10.57	0.48	10.80	0.04	1.02	0.031	4.6E-01
Mtr.38290.1.S1_at	8.16	7.53	7.21	7.92	7.82	8.12	7.64	0.48	7.95	0.16	1.04	0.059	3.4E-01
Mtr.38142.1.S1_at	10.23	11.20	10.76	10.43	10.88	10.66	10.73	0.48	10.66	0.22	0.99	-0.010	8.3E-01
Mtr.2700.1.S1_at	10.91	9.99	10.71	10.70	10.75	10.59	10.54	0.48	10.68	0.08	1.01	0.019	6.4E-01
Mtr.33585.1.S1_at	9.45	8.49	8.89	9.18	9.36	9.33	8.94	0.48	9.29	0.10	1.04	0.055	2.9E-01
Mtr.17553.1.S1_s_at	3.15	3.99	3.16	3.12	3.12	3.25	3.43	0.48	3.16	0.07	0.92	-0.118	3.9E-01
Mtr.19109.1.S1_s_at	8.42	8.03	8.99	8.66	8.71	8.55	8.48	0.48	8.64	0.09	1.02	0.028	6.0E-01
Mtr.34872.1.S1_s_at	7.74	6.87	7.66	7.70	7.66	7.79	7.42	0.48	7.72	0.07	1.04	0.056	3.6E-01
Mtr.43519.1.S1_at	11.85	10.95	11.72	11.84	11.71	11.75	11.51	0.48	11.76	0.07	1.02	0.032	4.1E-01
Mtr.5313.1.S1_s_at	7.71	7.07	8.02	7.59	7.53	7.82	7.60	0.48	7.65	0.15	1.01	0.010	8.7E-01
Mtr.10049.1.S1_at	9.39	8.64	8.49	8.81	8.86	9.02	8.84	0.48	8.90	0.11	1.01	0.010	8.5E-01
Mtr.20614.1.S1_at	2.76	3.60	2.75	2.74	2.64	2.71	3.04	0.48	2.70	0.05	0.89	-0.171	2.9E-01
Mtr.46327.1.S1_at	8.64	7.68	8.26	8.27	8.45	8.35	8.19	0.48	8.36	0.09	1.02	0.029	5.9E-01
Mtr.18129.1.S1_s_at	8.69	7.82	8.61	8.55	8.55	8.64	8.37	0.48	8.58	0.05	1.02	0.035	5.0E-01
Mtr.39465.1.S1_at	5.41	4.47	5.15	5.13	5.35	5.72	5.01	0.48	5.40	0.29	1.08	0.108	3.0E-01
Mtr.43972.1.S1_at	2.43	2.50	3.30	2.41	2.45	2.39	2.75	0.48	2.41	0.03	0.88	-0.186	3.0E-01
Mtr.47465.1.S1_at	11.85	10.89	11.45	11.06	10.83	11.07	11.40	0.48	10.99	0.13	0.96	-0.053	2.3E-01
Mtr.30651.1.S1_at	8.70	7.99	8.91	8.78	9.33	8.81	8.53	0.48	8.98	0.31	1.05	0.073	2.5E-01
Mtr.36208.1.S1_at	3.16	3.98	3.12	3.10	3.08	3.02	3.42	0.48	3.07	0.04	0.90	-0.156	2.8E-01
Mtr.48976.1.S1_s_at	7.16	6.33	7.18	7.11	7.14	7.24	6.89	0.48	7.16	0.07	1.04	0.056	3.9E-01
Mtr.34221.1.S1_at	1.84	2.67	1.83	1.83	1.83	1.83	2.12	0.48	1.83	0.00	0.86	-0.210	3.6E-01
Mtr.12555.1.S1_at	8.11	7.74	8.70	8.02	7.84	7.65	8.18	0.48	7.84	0.18	0.96	-0.063	3.1E-01
Mtr.27854.1.S1_at	9.51	8.54	8.96	8.73	8.56	9.07	9.00	0.48	8.79	0.26	0.98	-0.035	5.3E-01
Mtr.30714.1.S1_at	5.72	6.31	6.68	6.05	6.08	5.99	6.24	0.48	6.04	0.04	0.97	-0.046	5.2E-01
Mtr.27503.1.S1_at	6.15	5.51	6.46	6.22	5.92	6.07	6.04	0.48	6.07	0.15	1.01	0.007	9.2E-01
Mtr.38325.1.S1_at	11.63	10.88	10.73	11.02	11.32	11.33	11.08	0.48	11.22	0.18	1.01	0.018	6.6E-01
Mtr.44490.1.S1_at	11.66	11.12	12.09	11.96	11.98	11.71	11.62	0.48	11.88	0.15	1.02	0.032	4.2E-01
Mtr.29159.1.S1_at	7.67	7.19	8.16	8.28	7.87	7.47	7.67	0.48	7.88	0.41	1.03	0.038	6.1E-01
Mtr.10936.1.S1_at	8.92	9.14	9.84	9.22	9.27	8.80	9.30	0.48	9.10	0.26	0.98	-0.032	5.5E-01
Mtr.41933.1.S1_at	11.41	11.10	12.05	11.94	12.00	11.64	11.52	0.48	11.86	0.19	1.03	0.042	3.2E-01
Mtr.29557.1.S1_at	3.67	2.91	2.76	2.73	3.46	2.71	3.11	0.48	2.97	0.43	0.95	-0.069	7.2E-01
Mtr.42391.1.S1_at	10.41	9.55	9.59	9.93	9.75	9.97	9.85	0.48	9.88	0.11	1.00	0.005	9.1E-01
Mtr.5116.1.S1_at	8.95	8.27	9.20	9.13	8.77	9.16	8.81	0.48	9.02	0.22	1.02	0.034	5.3E-01
Mtr.6387.1.S1_at	2.38	3.20	2.35	2.35	2.34	2.33	2.64	0.48	2.34	0.01	0.89	-0.175	3.4E-01
Mtr.52358.1.S1_x_at	3.08	3.88	3.01	3.01	3.05	3.04	3.32	0.48	3.04	0.02	0.91	-0.132	3.6E-01
Mtr.34590.1.S1_at	9.39	9.22	10.13	10.02	10.07	9.91	9.58	0.48	10.00	0.08	1.04	0.062	2.1E-01
Mtr.41607.1.S1_at	8.15	7.64	8.60	8.47	8.42	8.60	8.13	0.48	8.50	0.09	1.04	0.063	2.7E-01
Mtr.19708.1.S1_at	8.96	8.00	8.59	8.54	8.79	8.54	8.52	0.48	8.62	0.15	1.01	0.018	7.4E-01
Mtr.1377.1.S1_at	3.05	3.87	3.01	2.99	2.94	2.92	3.31	0.48	2.95	0.04	0.89	-0.166	2.7E-01
Mtr.11346.1.S1_at	10.59	9.89	9.67	10.18	10.13	10.37	10.05	0.48	10.23	0.13	1.02	0.026	5.7E-01
Mtr.31061.1.S1_x_at	4.33	5.11	4.23	4.34	3.75	4.27	4.55	0.48	4.12	0.32	0.90	-0.144	2.7E-01
Mtr.13910.1.S1_x_at	4.80	3.94	4.76	4.75	4.98	4.73	4.50	0.48	4.82	0.14	1.07	0.098	3.4E-01
Mtr.14088.1.S1_x_at	2.53	2.68	3.43	2.49	2.49	2.48	2.88	0.48	2.49	0.01	0.86	-0.211	2.3E-01
Mtr.45702.1.S1_s_at	2.30	3.14	2.30	2.29	2.29	2.28	2.58	0.48	2.29	0.01	0.89	-0.173	3.6E-01
Mtr.48280.1.S1_at	10.61	9.66	10.02	10.25	10.19	10.35	10.09	0.48	10.27	0.08	1.02	0.024	5.8E-01
Mtr.49094.1.S1_at	8.11	8.84	7.93	7.66	6.93	6.60	8.30	0.48	7.06	0.54	0.85	-0.232	4.2E-02
Mtr.22271.1.S1_x_at	2.37	3.20	2.36	2.36	2.36	2.36	2.65	0.48	2.36	0.00	0.89	-0.168	3.6E-01
Mtr.32826.1.S1_at	5.36	4.47	4.60	5.05	4.58	4.56	4.81	0.48	4.73	0.28	0.98	-0.025	8.1E-01
Mtr.26810.1.S1_at	6.19	5.28	6.03	5.68	5.86	5.86	5.83	0.48	5.80	0.10	0.99	-0.008	9.1E-01
Mtr.29977.1.S1_at	6.05	5.38	5.12	5.28	5.27	5.47	5.52	0.48	5.34	0.11	0.97	-0.046	5.8E-01
Mtr.29095.1.S1_at	7.27	8.10	8.12	7.20	7.24	7.53	7.83	0.48	7.32	0.18	0.93	-0.097	1.6E-01
Mtr.36311.1.S1_at	8.04	7.11	7.79	7.95	8.11	8.12	7.65	0.48	8.06	0.10	1.05	0.076	2.2E-01
Mtr.9034.1.S1_at	10.38	10.48	11.26	10.89	11.14	10.81	10.71	0.48	10.95	0.17	1.02	0.032	4.6E-01
Mtr.8443.1.S1_at	4.13	3.56	3.16	3.89	5.18	3.34	3.62	0.48	4.14	0.94	1.14	0.193	4.4E-01
Mtr.28168.1.S1_at	4.21	5.17	4.75	4.23	5.84	4.93	4.71	0.48	5.00	0.81	1.06	0.086	6.2E-01
Mtr.35140.1.S1_at	7.39	6.43	6.97	7.31	7.22	7.59	6.93	0.48	7.37	0.19	1.06	0.089	2.2E-01
Mtr.14046.1.S1_s_at	12.18	13.06	12.28	12.45	13.00	12.51	12.51	0.48	12.65	0.30	1.01	0.017	6.8E-01
Mtr.42790.1.S1_at	5.89	5.18	6.10	6.09	5.92	6.20	5.72	0.48	6.07	0.14	1.06	0.085	3.0E-01
Mtr.37085.1.S1_at	6.80	7.68	7.59	7.82	7.84	7.74	7.36	0.48	7.80	0.05	1.06	0.084	1.9E-01
Mtr.23707.1.S1_at	2.60	3.43	2.58	2.60	2.73	2.60	2.87	0.48	2.64	0.07	0.92	-0.121	4.6E-01
Mtr.36921.1.S1_at	2.87	3.70	2.85	2.85	2.75	2.85	3.14	0.48	2.82	0.06	0.90	-0.157	3.1E-01
Mtr.6635.1.S1_s_at	12.46	11.84	11.51	11.20	11.20	11.29	11.94	0.48	11.23	0.05	0.94	-0.088	6.4E-02
Mtr.16601.1.S1_at	11.54	11.58	10.72	10.99	10.81	11.09	11.28	0.48	10.97	0.14	0.97	-0.041	3.4E-01
Mtr.34399.1.S1_at	7.18	6.68	7.65	7.58	7.62	7.71	7.17	0.48	7.64	0.07	1.07	0.091	1.7E-01
Mtr.42018.1.S1_at	8.97	8.02	8.39	8.79	8.66	8.73	8.46	0.48	8.73	0.06	1.03	0.045	3.9E-01
Mtr.35877.1.S1_at	9.87	9.61	10.54	9.81	10.14	9.73	10.00	0.48	9.89	0.22	0.99	-0.016	7.4E-01
Mtr.2302.1.S1_at	7.36	8.22	7.40	7.28	7.00	7.41	7.66	0.48	7.23	0.21	0.94	-0.083	2.3E-01
Mtr.20534.1.S1_at	2.84	3.65	2.79	2.77	2.82	2.78	3.09	0.48	2.79	0.03	0.90	-0.147	3.4E-01
Mtr.13417.1.S1_at	9.62	8.66	9.26	9.38	9.36	9.46	9.18	0.48	9.40	0.05	1.02	0.034	4.8E-01
Mtr.12750.1.S1_at	8.87	7.92	8.57	8.62	8.83	8.68	8.45	0.48	8.71	0.11	1.03	0.043	4.2E-01
Mtr.13448.1.S1_at	4.07	3.97	4.85	4.60	5.04	4.07	4.30	0.48	4.57	0.48	1.06	0.089	5.2E-01
Mtr.51664.1.S1_s_at	7.98	8.69	8.91	9.10	9.24	9.01	8.52	0.48	9.12	0.12	1.07	0.097	1.1E-01
Mtr.48104.1.S1_s_at	3.06	3.40	2.44	4.52	3.35	4.08	2.97	0.48	3.98	0.59	1.34	0.426	8.2E-02
Mtr.11300.1.S1_at	10.44	10.03	10.99	10.88	10.87	10.62	10.49	0.48	10.79	0.15	1.03	0.041	3.6E-01
Mtr.21270.1.S1_at	11.12	10.47	11.41	11.36	11.41	11.26	11.00	0.48	11.34	0.07	1.03	0.045	2.9E-01
Mtr.6633.1.S1_at	9.36	8.62	9.53	9.22	9.39	8.95	9.17	0.48	9.18	0.22	1.00	0.003	9.6E-01
Mtr.39775.1.S1_at	7.34	6.40	6.72	6.86	7.01	6.89	6.82	0.48	6.92	0.08	1.01	0.020	7.5E-01
Mtr.10940.1.S1_at	11.02	10.08	10.37	10.23	10.21	10.28	10.49	0.48	10.24	0.04	0.98	-0.035	4.2E-01
Mtr.12108.1.S1_at	3.62	3.72	4.50	4.02	3.70	3.04	3.94	0.48	3.59	0.50	0.91	-0.138	4.2E-01
Mtr.27586.1.S1_at	5.77	4.95	5.80	5.76	5.70	5.70	5.50	0.48	5.72	0.04	1.04	0.056	4.8E-01
Mtr.39642.1.S1_at	7.73	8.69	8.29	8.97	9.42	9.30	8.24	0.48	9.23	0.23	1.12	0.165	3.2E-02
Mtr.40340.1.S1_at	11.42	11.31	12.19	11.62	12.08	11.63	11.64	0.48	11.78	0.26	1.01	0.017	6.8E-01
Mtr.39038.1.S1_at	10.13	9.58	10.54	9.97	10.07	9.97	10.08	0.48	10.00	0.05	0.99	-0.011	7.9E-01
Mtr.43930.1.S1_at	11.20	11.37	12.11	11.40	11.46	11.55	11.56	0.48	11.47	0.07	0.99	-0.011	7.7E-01
Mtr.20467.1.S1_at	8.51	7.56	8.19	8.19	8.40	8.28	8.09	0.48	8.29	0.10	1.02	0.035	5.2E-01
Mtr.34474.1.S1_at	7.43	6.47	6.91	7.24	7.02	7.03	6.94	0.48	7.10	0.12	1.02	0.032	6.1E-01
Mtr.43823.1.S1_at	11.89	10.96	11.63	10.69	10.69	10.76	11.49	0.48	10.71	0.04	0.93	-0.102	4.9E-02
Mtr.39163.1.S1_at	13.42	12.58	12.60	12.96	12.65	13.12	12.87	0.48	12.91	0.24	1.00	0.005	9.0E-01
Mtr.36895.1.S1_at	4.24	3.40	4.23	4.29	4.60	3.81	3.96	0.48	4.23	0.40	1.07	0.097	4.9E-01
Mtr.39365.1.S1_at	9.05	8.84	8.13	8.90	8.97	9.42	8.67	0.48	9.10	0.28	1.05	0.068	2.6E-01
Mtr.5741.1.S1_at	3.71	4.45	3.55	3.60	3.55	3.54	3.90	0.48	3.57	0.03	0.91	-0.130	2.9E-01
Mtr.35977.1.S1_at	3.54	2.67	2.76	2.67	2.57	2.66	2.99	0.48	2.63	0.05	0.88	-0.182	2.7E-01
Mtr.4264.1.S1_at	3.75	2.91	3.73	3.44	3.62	3.57	3.46	0.48	3.54	0.09	1.02	0.032	7.9E-01
Mtr.12605.1.S1_at	10.31	9.67	9.37	10.63	10.68	10.98	9.78	0.48	10.76	0.19	1.10	0.138	3.0E-02
Mtr.24823.1.S1_at	7.87	7.52	8.47	7.54	7.61	7.06	7.95	0.48	7.40	0.30	0.93	-0.103	1.7E-01
Mtr.35160.1.S1_at	9.08	8.13	8.74	8.89	8.91	9.03	8.65	0.48	8.94	0.08	1.03	0.048	3.6E-01
Mtr.35567.1.S1_at	9.67	8.84	8.83	9.37	9.48	9.46	9.11	0.48	9.44	0.06	1.04	0.050	3.1E-01
Mtr.5832.1.S1_at	7.99	7.08	7.25	7.93	7.73	7.79	7.44	0.48	7.82	0.10	1.05	0.071	2.6E-01
Mtr.30108.1.S1_at	3.26	3.87	2.91	3.59	2.95	3.45	3.35	0.48	3.33	0.33	1.00	-0.007	9.6E-01
Mtr.16896.1.S1_at	10.98	11.93	11.60	11.13	11.45	11.18	11.51	0.48	11.25	0.17	0.98	-0.032	4.4E-01
Mtr.10572.1.S1_at	13.66	12.80	13.61	13.97	14.07	14.12	13.36	0.48	14.05	0.08	1.05	0.073	6.8E-02
Mtr.36491.1.S1_at	2.21	3.02	2.17	2.17	2.16	2.16	2.47	0.48	2.16	0.01	0.88	-0.190	3.3E-01

Mtr.20613.1.S1_at	6.63	6.56	7.42	7.03	6.96	6.73	6.87	0.48	6.91	0.16	1.01	0.008	9.0E-01
Mtr.33971.1.S1_at	2.46	3.28	2.44	2.44	2.44	2.43	2.73	0.48	2.44	0.01	0.89	-0.163	3.5E-01
Mtr.38706.1.S1_at	8.26	7.30	7.87	8.04	8.07	8.28	7.81	0.48	8.13	0.13	1.04	0.058	3.3E-01
Mtr.8064.1.S1_s_at	8.96	8.05	8.79	8.96	8.92	8.83	8.60	0.48	8.90	0.06	1.04	0.050	3.4E-01
Mtr.3100.1.S1_x_at	9.27	10.22	9.67	9.73	9.89	9.80	9.72	0.48	9.81	0.08	1.01	0.013	7.7E-01
Mtr.9441.1.S1_at	8.41	7.46	7.83	8.10	8.10	7.90	7.90	0.48	8.04	0.11	1.02	0.024	6.6E-01
Mtr.34860.1.S1_s_at	8.06	7.15	7.87	8.00	8.00	7.86	7.70	0.48	7.95	0.08	1.03	0.047	4.2E-01
Mtr.14205.1.S1_x_at	8.69	7.93	8.81	8.67	8.90	8.62	8.48	0.48	8.73	0.15	1.03	0.043	4.3E-01
Mtr.46498.1.S1_at	9.48	8.73	9.62	9.44	9.31	9.27	9.28	0.48	9.34	0.09	1.01	0.010	8.4E-01
Mtr.3179.1.S1_s_at	7.13	6.27	7.07	7.25	6.96	6.69	6.82	0.48	6.97	0.28	1.02	0.029	6.8E-01
Mtr.1200.1.S1_at	4.96	5.92	5.41	5.91	6.59	6.03	5.43	0.48	6.18	0.36	1.14	0.187	9.6E-02
Mtr.40250.1.S1_at	12.18	11.33	12.14	12.30	12.38	12.22	11.88	0.48	12.30	0.08	1.04	0.050	2.1E-01
Mtr.28007.1.S1_at	8.22	7.29	7.97	8.21	8.00	8.31	7.83	0.48	8.17	0.16	1.04	0.062	3.1E-01
Mtr.8177.1.S1_at	7.91	6.95	7.43	7.61	8.08	7.65	7.43	0.48	7.78	0.26	1.05	0.067	3.3E-01
Mtr.12202.1.S1_at	2.98	3.01	3.83	3.03	2.96	2.94	3.28	0.48	2.98	0.05	0.91	-0.139	3.4E-01
Mtr.28813.1.S1_at	2.75	3.52	2.64	2.64	2.64	2.58	2.97	0.48	2.62	0.04	0.88	-0.181	2.8E-01
Mtr.33577.1.S1_at	6.29	5.33	5.82	5.80	5.62	6.21	5.82	0.48	5.88	0.30	1.01	0.015	8.6E-01
Mtr.40094.1.S1_at	11.02	10.87	11.77	11.70	11.69	11.67	11.22	0.48	11.68	0.02	1.04	0.058	1.7E-01
Mtr.13433.1.S1_at	9.55	10.13	9.17	8.95	9.37	9.27	9.62	0.48	9.20	0.22	0.96	-0.064	2.4E-01
Mtr.17314.1.S1_at	2.79	3.71	3.02	3.01	3.04	2.91	3.17	0.48	2.99	0.07	0.94	-0.085	5.5E-01
Mtr.12315.1.S1_x_at	11.90	12.76	12.69	12.24	12.70	12.34	12.45	0.48	12.43	0.24	1.00	-0.003	9.4E-01
Mtr.32860.1.S1_s_at	4.51	4.71	5.42	4.46	5.05	3.71	4.88	0.48	4.41	0.67	0.90	-0.148	3.8E-01
Mtr.38899.1.S1_at	10.52	9.59	10.27	10.48	10.56	10.41	10.13	0.48	10.48	0.07	1.03	0.049	2.8E-01
Mtr.44556.1.S1_at	8.04	7.09	7.69	7.85	7.83	7.88	7.60	0.48	7.85	0.03	1.03	0.047	4.2E-01
Mtr.15593.1.S1_at	2.64	3.46	2.62	2.56	2.61	2.61	2.91	0.48	2.60	0.03	0.89	-0.164	3.2E-01
Mtr.33396.1.S1_s_at	7.93	7.15	8.03	8.06	8.23	8.14	7.70	0.48	8.15	0.09	1.06	0.081	1.9E-01
Mtr.42505.1.S1_at	7.92	7.14	8.01	8.03	7.97	7.88	7.69	0.48	7.96	0.07	1.03	0.050	3.9E-01
Mtr.28719.1.S1_at	6.67	6.40	7.33	7.13	7.14	7.26	6.80	0.48	7.18	0.07	1.06	0.078	2.5E-01
Mtr.38573.1.S1_at	8.39	7.50	8.26	8.18	8.04	8.18	8.05	0.48	8.13	0.08	1.01	0.015	7.8E-01
Mtr.40241.1.S1_at	14.13	13.22	13.94	13.81	13.61	13.76	13.76	0.48	13.72	0.10	1.00	-0.004	8.9E-01
Mtr.13328.1.S1_at	9.51	10.25	10.41	9.79	9.80	9.74	10.06	0.48	9.78	0.03	0.97	-0.040	3.8E-01
Mtr.42326.1.S1_at	4.81	5.01	5.73	4.73	4.68	4.95	5.18	0.48	4.79	0.14	0.92	-0.114	2.4E-01
Mtr.17995.1.S1_at	9.52	8.69	9.53	9.64	9.65	9.45	9.25	0.48	9.58	0.11	1.04	0.051	3.1E-01
Mtr.29124.1.S1_at	7.33	6.42	7.12	7.12	7.23	7.13	6.96	0.48	7.16	0.06	1.03	0.042	5.0E-01
Mtr.2619.1.S1_at	7.84	8.60	8.73	8.38	9.29	8.34	8.39	0.48	8.67	0.53	1.03	0.047	5.4E-01
Mtr.20953.1.S1_at	8.51	7.74	8.62	9.36	9.70	9.46	8.29	0.48	9.51	0.18	1.15	0.198	1.4E-02
Mtr.12003.1.S1_at	6.88	6.20	7.13	7.16	6.82	6.84	6.74	0.48	6.94	0.19	1.03	0.043	5.4E-01
Mtr.14720.1.S1_at	7.88	7.04	7.85	8.10	7.93	7.98	7.59	0.48	8.00	0.09	1.05	0.077	2.2E-01
Mtr.35410.1.S1_at	6.25	5.46	6.33	6.00	6.07	5.92	6.02	0.48	6.00	0.08	1.00	-0.004	9.5E-01
Mtr.44157.1.S1_at	10.89	9.98	10.15	10.42	10.32	10.35	10.34	0.48	10.36	0.05	1.00	0.003	9.4E-01
Mtr.18133.1.S1_x_at	2.96	3.78	2.94	2.94	2.94	2.91	3.23	0.48	2.93	0.02	0.91	-0.139	3.4E-01
Mtr.26243.1.S1_s_at	11.00	10.10	10.26	10.53	10.54	10.57	10.45	0.48	10.55	0.02	1.01	0.013	7.5E-01
Mtr.52033.1.S1_at	4.56	4.17	3.60	4.04	4.13	3.99	4.11	0.48	4.05	0.07	0.99	-0.020	8.5E-01
Mtr.49955.1.S1_at	3.24	4.04	3.18	3.14	3.11	3.10	3.49	0.48	3.12	0.02	0.89	-0.162	2.5E-01
Mtr.46550.1.S1_at	3.02	3.83	2.97	2.94	2.92	2.92	3.27	0.48	2.93	0.01	0.89	-0.162	2.8E-01
Mtr.33089.1.S1_at	6.71	5.88	6.71	6.71	6.72	7.04	6.43	0.48	6.82	0.19	1.06	0.085	2.6E-01
Mtr.11012.1.S1_at	7.09	6.14	6.47	6.10	5.86	6.05	6.57	0.48	6.01	0.13	0.91	-0.129	1.2E-01
Mtr.31556.1.S1_at	7.73	7.06	7.99	7.85	7.88	7.64	7.59	0.48	7.79	0.13	1.03	0.037	5.3E-01
Mtr.45081.1.S1_at	8.85	8.59	9.52	9.09	8.99	9.14	8.98	0.48	9.08	0.08	1.01	0.015	7.6E-01
Mtr.28788.1.S1_at	9.64	10.53	10.38	10.38	10.17	10.13	10.18	0.48	10.23	0.13	1.00	0.006	8.9E-01
Mtr.41771.1.S1_at	7.79	6.93	7.73	7.76	7.79	7.55	7.48	0.48	7.70	0.13	1.03	0.042	4.8E-01
Mtr.39615.1.S1_at	7.90	7.39	8.35	7.91	8.30	7.67	7.88	0.48	7.96	0.32	1.01	0.015	8.2E-01
Mtr.4976.1.S1_at	7.95	7.32	8.26	8.06	8.06	7.90	7.84	0.48	8.01	0.09	1.02	0.030	5.9E-01
Mtr.34896.1.S1_at	5.73	5.56	4.83	4.83	4.43	4.83	5.37	0.48	4.70	0.23	0.87	-0.193	9.4E-02
Mtr.20396.1.S1_at	10.06	9.17	9.91	9.76	9.68	9.79	9.71	0.48	9.75	0.06	1.00	0.005	9.1E-01
Mtr.40630.1.S1_at	10.94	9.98	10.49	10.46	10.45	10.53	10.47	0.48	10.48	0.04	1.00	0.001	9.8E-01
Mtr.51298.1.S1_x_at	3.12	3.96	3.14	3.10	3.05	3.06	3.41	0.48	3.07	0.03	0.90	-0.150	2.9E-01
Mtr.44067.1.S1_at	8.01	7.07	7.66	7.81	7.89	7.84	7.58	0.48	7.84	0.04	1.03	0.049	4.0E-01
Mtr.828.1.S1_at	2.81	3.63	2.78	2.76	2.76	2.75	3.08	0.48	2.75	0.00	0.90	-0.159	3.1E-01
Mtr.32938.1.S1_at	6.29	5.51	5.42	5.76	6.38	6.25	5.74	0.48	6.13	0.33	1.07	0.095	3.1E-01
Mtr.43971.1.S1_at	9.81	8.88	9.14	9.17	9.35	9.37	9.27	0.48	9.30	0.11	1.00	0.003	9.4E-01
Mtr.46283.1.S1_at	2.83	3.65	2.81	2.77	2.81	2.79	3.10	0.48	2.79	0.02	0.90	-0.152	3.3E-01
Mtr.27477.1.S1_at	8.78	7.89	8.65	8.80	8.71	8.73	8.44	0.48	8.75	0.05	1.04	0.051	3.3E-01
Mtr.3509.1.S1_at	4.33	3.60	4.51	4.08	4.08	3.73	4.15	0.48	3.96	0.20	0.96	-0.066	5.7E-01
Mtr.5950.1.S1_s_at	12.25	11.30	11.67	12.15	12.26	12.09	11.74	0.48	12.17	0.09	1.04	0.051	2.1E-01
Mtr.27854.1.S1_s_at	10.15	9.19	9.57	9.56	9.14	9.82	9.64	0.48	9.51	0.34	0.99	-0.019	7.3E-01
Mtr.31708.1.S1_at	8.94	8.46	9.42	9.07	9.15	8.87	8.94	0.48	9.03	0.15	1.01	0.015	7.7E-01
Mtr.1718.1.S1_at	4.06	3.30	3.17	3.11	3.50	3.26	3.51	0.48	3.29	0.19	0.94	-0.095	5.0E-01
Mtr.19856.1.S1_x_at	3.70	3.43	2.77	3.06	3.07	3.35	3.30	0.48	3.16	0.16	0.96	-0.060	6.7E-01
Mtr.18902.1.S1_at	2.42	3.24	2.40	2.39	2.39	2.38	2.69	0.48	2.39	0.01	0.89	-0.169	3.4E-01
Mtr.10292.1.S1_at	8.86	7.91	8.46	8.54	8.71	8.44	8.41	0.48	8.56	0.13	1.02	0.026	6.2E-01
Mtr.38460.1.S1_at	6.53	5.57	6.10	5.87	6.09	6.19	6.07	0.48	6.05	0.17	1.00	-0.004	9.6E-01
Mtr.45652.1.S1_s_at	8.28	7.62	8.56	8.36	8.46	8.37	8.15	0.48	8.39	0.06	1.03	0.042	4.3E-01
Mtr.52323.1.S1_at	9.76	8.83	9.49	9.71	9.73	9.65	9.36	0.48	9.70	0.04	1.04	0.051	2.9E-01
Mtr.10205.1.S1_at	7.25	6.31	6.91	7.27	7.23	7.15	6.82	0.48	7.22	0.06	1.06	0.081	2.3E-01
Mtr.13872.1.S1_at	8.84	7.97	8.75	9.01	8.97	9.09	8.52	0.48	9.03	0.06	1.06	0.083	1.4E-01
Mtr.11777.1.S1_at	9.52	8.63	9.38	9.33	9.42	9.26	9.18	0.48	9.34	0.08	1.02	0.024	6.1E-01
Mtr.30749.1.S1_s_at	8.04	7.08	7.61	7.42	8.62	7.89	7.58	0.48	7.98	0.60	1.05	0.075	4.2E-01
Mtr.29995.1.S1_at	3.46	3.63	4.36	3.59	3.74	3.51	3.82	0.48	3.61	0.11	0.95	-0.078	5.2E-01
Mtr.5411.1.S1_at	5.64	5.05	5.99	5.16	5.76	5.24	5.56	0.48	5.39	0.33	0.97	-0.046	6.3E-01
Mtr.6028.1.S1_at	10.39	9.50	10.24	10.36	10.08	10.46	10.04	0.48	10.30	0.20	1.03	0.036	4.4E-01
Mtr.5063.1.S1_at	5.48	5.24	6.17	5.56	5.39	5.39	5.63	0.48	5.44	0.10	0.97	-0.049	5.4E-01
Mtr.26331.1.S1_at	6.80	5.85	6.37	7.18	7.01	7.37	6.34	0.48	7.19	0.18	1.13	0.181	4.6E-02
Mtr.43054.1.S1_at	11.85	12.80	12.45	12.75	13.02	12.93	12.37	0.48	12.90	0.14	1.04	0.061	1.4E-01
Mtr.47041.1.S1_at	2.75	3.57	2.73	2.74	2.70	2.69	3.01	0.48	2.71	0.02	0.90	-0.153	3.3E-01
Mtr.44487.1.S1_at	8.43	8.23	9.14	9.74	9.84	9.68	8.60	0.48	9.75	0.08	1.13	0.181	1.5E-02
Mtr.50956.1.S1_s_at	6.31	6.92	5.98	6.13	6.05	6.07	6.40	0.48	6.08	0.04	0.95	-0.074	3.1E-01
Mtr.47690.1.S1_s_at	6.72	7.39	7.64	7.42	7.89	7.63	7.25	0.48	7.64	0.23	1.05	0.076	2.7E-01
Mtr.37850.1.S1_at	9.99	9.15	9.96	10.47	10.44	10.49	9.70	0.48	10.47	0.03	1.08	0.110	5.1E-02
Mtr.36936.1.S1_at	4.01	3.19	3.17	3.16	3.15	3.17	3.46	0.48	3.16	0.01	0.91	-0.129	3.5E-01
Mtr.35333.1.S1_at	2.37	3.18	2.34	2.34	2.31	2.33	2.63	0.48	2.32	0.02	0.88	-0.178	3.3E-01
Mtr.14453.1.S1_at	8.00	7.39	8.34	8.10	8.08	8.15	7.91	0.48	8.11	0.04	1.03	0.036	5.1E-01
Mtr.6534.1.S1_at	8.43	7.93	8.89	8.49	8.64	8.42	8.42	0.48	8.52	0.11	1.01	0.017	7.4E-01
Mtr.22160.1.S1_x_at	9.80	10.30	10.76	10.07	10.28	9.92	10.29	0.48	10.09	0.18	0.98	-0.028	5.4E-01
Mtr.5708.1.S1_at	8.37	8.20	9.10	8.43	8.14	8.01	8.56	0.48	8.19	0.22	0.96	-0.063	3.0E-01
Mtr.14142.1.S1_at	9.08	8.18	8.91	9.16	8.95	9.16	8.72	0.48	9.09	0.12	1.04	0.060	2.7E-01
Mtr.40037.1.S1_at	10.09	9.14	9.67	9.66	9.98	9.87	9.64	0.48	9.84	0.16	1.02	0.030	5.3E-01
Mtr.6661.1.S1_at	6.21	5.35	6.15	6.15	6.24	6.03	5.91	0.48	6.14	0.11	1.04	0.056	4.6E-01
Mtr.35880.1.S1_at	9.65	9.05	8.70	9.60	9.37	9.63	9.13	0.48	9.53	0.14	1.04	0.062	2.4E-01
Mtr.5753.1.S1_s_at	8.87	8.32	9.27	8.73	9.08	9.14	8.82	0.48	8.98	0.22	1.02	0.026	6.3E-01
Mtr.41790.1.S1_at	11.24	10.29	10.77	11.02	10.84	11.11	10.77	0.48	10.99	0.14	1.02	0.029	4.9E-01
Mtr.12980.1.S1_at	2.94	3.89	3.32	4.42	5.46	4.95	3.38	0.48	4.94	0.52	1.46	0.547	1.9E-02
Mtr.22785.1.S1_at	3.77	3.43	2.83	2.80	2.76	2.79	3.34	0.48	2.78	0.02	0.83	-0.264	1.1E-01
Mtr.27308.1.S1_s_at	6.52	5.76	6.64	6.55	6.42	6.61	6.31	0.48	6.53	0.10	1.03	0.050	4.8E-01
Mtr.387.1.S1_s_at	3.93	4.12	4.83	4.04	4.04	4.21	4.29	0.48	4.10	0.09	0.95	-0.067	5.2E-01
Mtr.40447.1.S1_at	9.27	9.38	10.15	9.67	9.57	9.47	9.60	0.48	9.57	0.10	1.00	-0.004	9.2E-01
Mtr.22749.1.S1_at	2.23	2.31	3.10	2.25	2.25	2.24	2.55	0.48	2.25	0.00	0.88	-0.181	3.4E-01
Mtr.44282.1.S1_s_at	10.20	9.35	10.15	9.98	9.91	9.97	9.90	0.48	9.95	0.04	1.01	0.008	8.6E-01
Mtr.7634.1.S1_at	8.68	8.34	9.28	9.18	9.33	9.02	8.77	0.48	9.18	0.16	1.05	0.066	2.3E-01
Mtr.20647.1.S1_at	6.53	6.44	5.66	5.94	5.97	6.02	6.21	0.48	5.97	0.04	0.96	-0.056	4.4E-01
Mtr.36171.1.S1_s_at	11.43	10.58	11.37	11.07	11.09	10.92	11.13	0.48	11.02	0.09	0.99	-0.013	7.4E-01
Mtr.41429.1.S1_at	9.42	9.76	10.37	9.67	9.73	9.32	9.85	0.48	9.57	0.22	0.97	-0.041	4.1E-01
Mtr.29462.1.S1_at	8.09	7.43	8.36	8.06	8.08	7.96	7.96	0.48	8.04	0.07	1.01	0.014	8.0E-01
Mtr.20148.1.S1_x_at	3.39	4.16	3.29	3.39	3.32	3.56	3.61	0.48	3.42	0.12	0.95	-0.079	5.4E-01
Mtr.35993.1.S1_x_at	3.33	4.13	3.28	3.29	3.28	3.28	3.58	0.48	3.28	0.01	0.92	-0.125	3.4E-01
Mtr.44675.1.S1_at	7.72	6.88	7.69	7.66	7.67	7.84	7.43	0.48	7.72	0.10	1.04	0.056	3.6E-01
Mtr.22838.1.S1_at	2.95	3.77	2.94	2.93	2.89	2.92	3.22	0.48	2.92	0.02	0.90	-0.144	3.3E-01
Mtr.12743.1.S1_s_at	7.99	7.05	7.39	7.55	7.58	7.60	7.48	0.48	7.58	0.02	1.01	0.019	7.3E-01
Mtr.36951.1.S1_at	6.23	5.66	6.61	5.81	6.28	6.15	6.16	0.48	6.08	0.24	0.99	-0.020	8.0E-01
Mtr.28473.1.S1_at	7.15	6.29	7.07	7.16	7.10	7.25	6.84	0.48	7.17	0.07	1.05	0.069	3.0E-01
Mtr.30830.1.S1_at	5.48	6.31	6.29	6.37	6.93	5.81	6.03	0.48	6.37	0.56	1.06	0.079	4.7E-01
Mtr.13297.1.S1_at	10.45	9.55	10.29	10.19	10.34	10.31	10.10	0.48	10.28	0.08	1.02	0.026	5.5E-01
Mtr.21337.1.S1_at	3.46	4.08	3.14	3.19	3.09	3.11	3.56	0.48	3.13	0.05	0.88	-0.185	2.0E-01
Mtr.26954.1.S1_at	4.40	4.54	3.65	4.41	4.37	4.35	4.19	0.48	4.37	0.03	1.04	0.061	5.5E-01
Mtr.27753.1.S1_at	10.13	11.03	10.86	11.35	10.88	11.30	10.68	0.48	11.18	0.26	1.05	0.066	1.8E-01
Mtr.1335.1.S1_at	2.54	3.35	2.51	2.50	2.50	2.49	2.80	0.48	2.50	0.01	0.89	-0.165	3.3E-01
Mtr.7930.1.S1_at	3.41	2.61	2.57	2.58	2.52	2.52	2.86	0.48	2.54	0.03	0.89	-0.173	3.1E-01
Mtr.23077.1.S1_at	3.15	3.97	3.14	3.14	3.13	3.59	3.42	0.48	3.29	0.26	0.96	-0.056	7.0E-01
Mtr.48536.1.S1_at	3.17	3.95	3.09	3.09	3.01	2.99	3.41	0.48	3.03	0.05	0.89	-0.168	2.5E-01
Mtr.50577.1.S1_at	8.73	7.96	8.84	8.58	8.58	8.65	8.51	0.48	8.60	0.04	1.01	0.015	7.6E-01
Mtr.28273.1.S1_at	9.26	8.31	8.88	9.22	9.13	9.31	8.81	0.48	9.22	0.09	1.05	0.065	2.2E-01

Mtr.45438.1.S1_at	9.32	8.64	9.55	9.22	9.01	9.21	9.17	0.48	9.15	0.12	1.00	-0.004	9.4E-01
Mtr.42641.1.S1_at	8.43	7.63	8.47	8.57	8.40	8.49	8.18	0.48	8.49	0.08	1.04	0.054	3.3E-01
Mtr.27470.1.S1_at	2.98	2.02	2.54	2.56	2.56	2.56	2.51	0.48	2.56	0.00	1.02	0.026	8.8E-01
Mtr.41804.1.S1_at	6.41	6.53	7.29	6.81	6.94	7.01	6.75	0.48	6.92	0.10	1.03	0.036	5.7E-01
Mtr.38048.1.S1_s_at	10.16	9.22	9.57	9.75	9.79	9.98	9.65	0.48	9.84	0.13	1.02	0.028	5.5E-01
Mtr.11206.1.S1_at	9.94	8.99	9.53	9.57	9.74	9.69	9.48	0.48	9.66	0.08	1.02	0.027	5.5E-01
Mtr.43097.1.S1_at	12.54	12.32	11.63	12.20	12.24	12.23	12.16	0.48	12.22	0.02	1.00	0.007	8.4E-01
Mtr.31967.1.S1_at	6.25	5.33	6.02	6.22	6.16	6.04	5.86	0.48	6.14	0.09	1.05	0.066	3.8E-01
Mtr.40321.1.S1_at	12.00	11.17	11.18	11.63	11.52	11.56	11.45	0.48	11.57	0.06	1.01	0.015	6.9E-01
Mtr.48196.1.S1_at	11.14	10.24	10.40	10.70	10.83	10.74	10.60	0.48	10.76	0.07	1.02	0.022	5.9E-01
Mtr.1656.1.S1_at	3.72	2.86	3.66	2.95	2.97	2.95	3.41	0.48	2.96	0.01	0.87	-0.206	1.7E-01
Mtr.6704.1.S1_at	4.20	3.46	3.31	3.26	3.26	3.35	3.65	0.48	3.29	0.05	0.90	-0.151	2.6E-01
Mtr.39487.1.S1_s_at	2.84	3.78	3.15	3.45	3.11	3.13	3.26	0.48	3.23	0.19	0.99	-0.009	9.5E-01
Mtr.25202.1.S1_x_at	2.22	3.04	2.21	2.20	2.20	2.19	2.49	0.48	2.20	0.01	0.88	-0.180	3.5E-01
Mtr.15461.1.S1_at	7.94	8.20	8.86	9.56	9.66	9.75	8.33	0.48	9.66	0.09	1.16	0.213	9.1E-03
Mtr.26219.1.S1_at	8.41	7.69	8.59	8.70	8.61	8.31	8.23	0.48	8.54	0.20	1.04	0.053	3.6E-01
Mtr.8249.1.S1_at	10.29	9.35	9.90	9.84	9.93	9.82	9.85	0.48	9.86	0.06	1.00	0.002	9.5E-01
Mtr.33672.1.S1_s_at	8.30	7.39	8.07	7.94	7.90	7.71	7.92	0.48	7.85	0.12	0.99	-0.013	8.2E-01
Mtr.31563.1.S1_at	7.29	7.17	8.05	7.13	7.01	7.40	7.50	0.48	7.18	0.20	0.96	-0.063	3.4E-01
Mtr.23295.1.S1_at	2.47	3.27	2.43	2.44	2.53	2.41	2.72	0.48	2.46	0.06	0.90	-0.147	3.9E-01
Mtr.29211.1.S1_at	3.84	3.05	3.91	3.90	3.97	3.88	3.60	0.48	3.92	0.05	1.09	0.122	3.1E-01
Mtr.8588.1.S1_at	11.62	11.40	12.31	11.97	11.73	11.72	11.78	0.48	11.81	0.14	1.00	0.004	9.2E-01
Mtr.27579.1.S1_at	8.31	7.39	8.08	8.23	8.38	8.48	7.93	0.48	8.37	0.13	1.06	0.078	2.0E-01
Mtr.11896.1.S1_at	8.67	8.15	9.10	8.68	8.71	8.62	8.64	0.48	8.67	0.05	1.00	0.006	9.1E-01
Mtr.5371.1.S1_at	6.25	5.44	6.27	6.87	5.93	6.21	5.99	0.48	6.34	0.48	1.06	0.082	4.2E-01
Mtr.15748.1.S1_at	6.99	6.14	6.93	6.82	7.17	6.79	6.68	0.48	6.92	0.21	1.04	0.051	4.7E-01
Mtr.24658.1.S1_x_at	3.06	3.83	2.96	3.11	3.38	2.97	3.28	0.48	3.15	0.21	0.96	-0.058	6.9E-01
Mtr.32115.1.S1_at	10.26	11.04	11.13	10.79	11.07	10.82	10.81	0.48	10.89	0.15	1.01	0.010	8.0E-01
Mtr.35993.1.S1_at	2.56	3.33	2.45	2.45	2.42	2.44	2.78	0.48	2.44	0.01	0.88	-0.191	2.8E-01
Mtr.34384.1.S1_at	4.04	3.09	3.53	3.82	3.97	4.38	3.55	0.48	4.05	0.29	1.14	0.190	2.0E-01
Mtr.11712.1.S1_at	3.84	2.96	3.73	3.62	3.95	3.73	3.51	0.48	3.76	0.17	1.07	0.101	4.3E-01
Mtr.13837.1.S1_at	8.05	7.10	7.52	7.81	7.70	7.83	7.56	0.48	7.78	0.07	1.03	0.042	4.7E-01
Mtr.25477.1.S1_at	4.69	5.51	4.68	4.69	4.71	4.62	4.96	0.48	4.67	0.05	0.94	-0.087	3.5E-01
Mtr.22700.1.S1_at	2.34	3.16	2.33	2.32	2.32	2.31	2.61	0.48	2.32	0.00	0.89	-0.172	3.4E-01
Mtr.13684.1.S1_at	5.91	5.39	6.33	6.29	6.11	6.31	5.88	0.48	6.24	0.11	1.06	0.086	2.7E-01
Mtr.13564.1.S1_at	9.31	8.45	9.22	8.91	8.78	8.50	8.99	0.48	8.73	0.21	0.97	-0.043	4.3E-01
Mtr.45885.1.S1_at	14.03	13.08	13.60	13.88	13.74	13.98	13.57	0.48	13.87	0.12	1.02	0.031	3.5E-01
Mtr.41868.1.S1_at	8.06	7.63	8.58	8.19	8.28	8.28	8.09	0.48	8.25	0.05	1.02	0.029	5.9E-01
Mtr.1532.1.S1_at	11.20	12.10	11.90	12.56	12.81	12.52	11.73	0.47	12.63	0.16	1.08	0.106	3.6E-02
Mtr.9431.1.S1_s_at	9.20	8.27	8.91	9.10	8.72	8.98	8.79	0.47	8.93	0.20	1.02	0.022	6.7E-01
Mtr.10932.1.S1_at	7.03	6.53	7.48	6.68	7.10	6.51	7.01	0.47	6.76	0.30	0.96	-0.052	4.9E-01
Mtr.9062.1.S1_at	10.45	9.55	10.26	10.23	10.16	10.14	10.09	0.47	10.18	0.04	1.01	0.013	7.6E-01
Mtr.35294.1.S1_at	5.59	4.70	5.44	5.08	6.01	5.33	5.24	0.47	5.47	0.48	1.04	0.061	5.9E-01
Mtr.19430.1.S1_at	3.27	2.51	2.40	2.49	3.09	2.42	2.72	0.47	2.67	0.37	0.98	-0.031	8.8E-01
Mtr.45475.1.S1_at	5.67	5.03	5.95	5.72	5.93	5.73	5.55	0.47	5.80	0.12	1.04	0.063	4.3E-01
Mtr.16086.1.S1_at	9.87	10.50	10.80	10.27	10.48	10.20	10.39	0.47	10.31	0.14	0.99	-0.010	8.1E-01
Mtr.26881.1.S1_s_at	2.94	3.73	2.88	2.85	2.81	2.79	3.19	0.47	2.82	0.03	0.88	-0.178	2.5E-01
Mtr.39562.1.S1_at	11.79	10.85	11.19	11.42	11.83	11.59	11.27	0.47	11.61	0.21	1.03	0.043	3.2E-01
Mtr.41379.1.S1_at	9.59	8.67	9.32	9.22	9.39	9.15	9.20	0.47	9.25	0.12	1.01	0.009	8.4E-01
Mtr.44300.1.S1_at	10.61	9.94	9.69	10.24	10.29	10.29	10.08	0.47	10.27	0.03	1.02	0.027	5.2E-01
Mtr.1845.1.S1_at	9.32	8.38	8.83	8.97	8.83	9.08	8.84	0.47	8.96	0.12	1.01	0.019	7.0E-01
Mtr.13544.1.S1_at	8.22	7.42	8.27	8.32	8.73	8.27	7.97	0.47	8.44	0.25	1.06	0.082	2.1E-01
Mtr.7403.1.S1_at	7.23	8.13	7.94	7.77	8.82	8.43	7.77	0.47	8.34	0.53	1.07	0.102	2.4E-01
Mtr.6728.1.S1_at	9.25	8.42	9.24	9.19	9.17	9.13	8.97	0.47	9.16	0.03	1.02	0.031	5.2E-01
Mtr.24036.1.S1_x_at	3.97	4.79	3.97	4.02	3.82	3.75	4.24	0.47	3.86	0.14	0.91	-0.135	2.5E-01
Mtr.6349.1.S1_at	8.46	7.60	7.69	8.32	8.28	8.49	7.92	0.47	8.36	0.11	1.06	0.079	1.9E-01
Mtr.40159.1.S1_at	9.57	8.62	9.07	9.02	9.07	9.03	9.09	0.47	9.04	0.03	0.99	-0.008	8.7E-01
Mtr.18373.1.S1_s_at	8.38	7.55	8.37	8.31	8.32	8.31	8.10	0.47	8.31	0.01	1.03	0.038	4.7E-01
Mtr.39777.1.S1_at	10.84	10.41	11.36	11.13	11.02	11.01	10.87	0.47	11.05	0.06	1.02	0.024	5.4E-01
Mtr.12708.1.S1_at	9.82	8.91	9.61	9.92	10.00	10.17	9.45	0.47	10.03	0.13	1.06	0.086	1.1E-01
Mtr.8235.1.S1_at	10.59	9.67	9.92	10.10	9.97	10.09	10.06	0.47	10.05	0.08	1.00	-0.001	9.8E-01
Mtr.12463.1.S1_at	13.95	13.03	13.70	13.85	13.77	13.93	13.56	0.47	13.85	0.08	1.02	0.030	3.6E-01
Mtr.31595.1.S1_at	7.65	7.11	8.05	7.55	8.42	7.72	7.60	0.47	7.90	0.46	1.04	0.055	4.8E-01
Mtr.4657.1.S1_at	3.21	3.99	3.13	3.15	3.13	3.22	3.44	0.47	3.17	0.05	0.92	-0.120	3.7E-01
Mtr.37883.1.S1_at	6.46	6.57	7.33	6.56	7.02	6.45	6.79	0.47	6.68	0.30	0.98	-0.024	7.5E-01
Mtr.42220.1.S1_at	7.92	7.16	8.03	8.00	8.07	8.07	7.70	0.47	8.04	0.04	1.04	0.063	2.8E-01
Mtr.32462.1.S1_at	8.30	8.68	9.25	9.15	9.27	8.98	8.74	0.47	9.13	0.15	1.04	0.063	2.4E-01
Mtr.9613.1.S1_at	10.28	9.50	9.42	9.67	9.81	9.94	9.73	0.47	9.80	0.14	1.01	0.010	8.2E-01
Mtr.34395.1.S1_at	9.13	8.20	8.79	8.81	8.80	8.53	8.71	0.47	8.71	0.16	1.00	0.001	9.9E-01
Mtr.44592.1.S1_at	4.24	3.40	4.21	4.31	4.16	4.19	3.95	0.47	4.22	0.08	1.07	0.095	3.9E-01
Mtr.22020.1.S1_at	6.69	7.53	7.49	7.61	7.54	7.68	7.24	0.47	7.61	0.07	1.05	0.072	2.5E-01
Mtr.35682.1.S1_at	11.05	10.29	11.15	10.86	10.81	10.88	10.83	0.47	10.85	0.04	1.00	0.003	9.4E-01
Mtr.2105.1.S1_at	11.58	10.94	11.86	11.70	12.05	11.72	11.46	0.47	11.82	0.20	1.03	0.045	2.8E-01
Mtr.45004.1.S1_at	12.10	11.15	11.62	11.72	11.59	11.68	11.62	0.47	11.66	0.07	1.00	0.005	8.9E-01
Mtr.14772.1.S1_at	8.76	7.83	8.47	8.65	8.48	8.63	8.35	0.47	8.59	0.09	1.03	0.040	4.5E-01
Mtr.29410.1.S1_at	8.08	7.32	8.19	8.30	8.58	8.17	7.86	0.47	8.35	0.21	1.06	0.088	1.7E-01
Mtr.45394.1.S1_at	10.95	10.84	10.08	11.05	10.91	11.25	10.62	0.47	11.07	0.17	1.04	0.060	2.0E-01
Mtr.15021.1.S1_x_at	8.21	7.54	8.45	8.03	8.26	7.96	8.07	0.47	8.08	0.16	1.00	0.003	9.6E-01
Mtr.29783.1.S1_at	2.68	3.48	2.64	2.64	2.63	2.56	2.93	0.47	2.61	0.04	0.89	-0.168	3.1E-01
Mtr.7374.1.S1_at	9.34	8.92	9.86	9.63	9.56	9.54	9.37	0.47	9.58	0.05	1.02	0.032	4.9E-01
Mtr.21822.1.S1_at	9.98	10.02	10.82	10.78	10.87	10.92	10.27	0.47	10.86	0.07	1.06	0.080	1.0E-01
Mtr.31481.1.S1_at	3.28	4.07	3.23	3.24	3.23	3.17	3.53	0.47	3.21	0.04	0.91	-0.135	3.2E-01
Mtr.16193.1.S1_at	9.30	8.39	9.05	9.11	9.20	8.94	8.91	0.47	9.08	0.13	1.02	0.027	5.8E-01
Mtr.40946.1.S1_at	3.33	2.99	2.40	2.63	2.63	2.59	2.91	0.47	2.62	0.03	0.90	-0.151	3.5E-01
Mtr.41642.1.S1_s_at	7.76	6.90	7.67	7.64	7.70	7.90	7.44	0.47	7.75	0.14	1.04	0.058	3.5E-01
Mtr.23670.1.S1_at	2.47	3.29	2.46	2.45	2.46	2.44	2.74	0.47	2.45	0.01	0.89	-0.163	3.5E-01
Mtr.2013.1.S1_s_at	10.01	9.07	9.66	9.92	10.11	10.09	9.58	0.47	10.04	0.10	1.05	0.068	1.7E-01
Mtr.39123.1.S1_at	9.47	8.92	9.86	9.48	9.60	9.30	9.41	0.47	9.46	0.15	1.00	0.007	8.9E-01
Mtr.1101.1.S1_at	6.91	6.19	7.08	6.84	6.88	6.82	6.73	0.47	6.85	0.03	1.02	0.026	6.8E-01
Mtr.38757.1.S1_at	10.85	10.07	10.00	10.04	9.59	10.08	10.31	0.47	9.90	0.27	0.96	-0.058	2.7E-01
Mtr.12776.1.S1_at	5.59	6.41	5.58	5.60	5.58	5.61	5.86	0.47	5.59	0.01	0.95	-0.067	3.8E-01
Mtr.38353.1.S1_at	9.49	8.64	9.43	10.71	11.38	11.17	9.19	0.47	11.09	0.34	1.21	0.271	4.9E-03
Mtr.19985.1.S1_at	11.98	11.51	11.03	11.61	11.49	11.74	11.51	0.47	11.62	0.13	1.01	0.014	7.2E-01
Mtr.22703.1.S1_at	2.29	3.10	2.28	2.27	2.26	2.27	2.56	0.47	2.27	0.01	0.89	-0.172	3.5E-01
Mtr.52053.1.S1_at	8.87	8.71	9.60	8.95	8.94	8.96	9.06	0.47	8.95	0.01	0.99	-0.018	7.1E-01
Mtr.50667.1.S1_at	2.93	3.73	2.88	3.30	2.88	2.99	3.18	0.47	3.05	0.22	0.96	-0.057	7.0E-01
Mtr.7545.1.S1_at	12.05	11.44	11.11	11.72	11.78	11.89	11.53	0.47	11.80	0.08	1.02	0.032	4.0E-01
Mtr.44689.1.S1_at	9.68	8.84	9.65	9.72	9.66	9.68	9.39	0.47	9.68	0.03	1.03	0.045	3.4E-01
Mtr.33833.1.S1_at	9.70	8.78	9.44	9.56	9.64	9.65	9.31	0.47	9.62	0.05	1.03	0.047	3.2E-01
Mtr.51564.1.S1_at	8.15	8.62	9.09	8.72	8.55	8.63	8.62	0.47	8.63	0.08	1.00	0.002	9.6E-01
Mtr.13273.1.S1_at	8.24	9.13	8.42	9.06	9.47	9.27	8.60	0.47	9.27	0.21	1.08	0.108	8.8E-02
Mtr.367.1.S1_at	4.89	4.08	4.91	5.04	4.79	4.95	4.63	0.47	4.92	0.13	1.06	0.090	3.5E-01
Mtr.13087.1.S1_at	9.54	8.61	8.94	9.20	9.37	9.39	9.03	0.47	9.32	0.10	1.03	0.046	3.6E-01
Mtr.374.1.S1_at	12.53	11.60	12.21	12.35	12.26	12.44	12.11	0.47	12.35	0.09	1.02	0.028	4.4E-01
Mtr.7633.1.S1_at	11.05	10.40	11.33	11.23	11.25	11.07	10.93	0.47	11.18	0.10	1.02	0.033	4.1E-01
Mtr.30974.1.S1_at	5.18	4.29	5.02	4.97	5.02	5.01	4.83	0.47	5.00	0.03	1.03	0.049	5.8E-01
Mtr.7788.1.S1_at	5.33	5.88	4.94	5.01	5.14	5.22	5.39	0.47	5.12	0.10	0.95	-0.072	4.0E-01
Mtr.40993.1.S1_at	10.97	10.03	10.52	10.53	10.41	10.43	10.51	0.47	10.46	0.07	1.00	-0.007	8.7E-01
Mtr.50352.1.S1_x_at	4.65	5.45	4.60	4.63	4.31	4.63	4.90	0.47	4.53	0.18	0.92	-0.115	2.7E-01
Mtr.17910.1.S1_at	8.29	8.82	9.23	8.68	9.05	8.86	8.78	0.47	8.86	0.19	1.01	0.013	8.0E-01
Mtr.50159.1.S1_x_at	3.34	4.07	3.19	3.30	3.61	3.45	3.53	0.47	3.45	0.15	0.98	-0.034	7.9E-01
Mtr.49527.1.S1_at	8.59	9.27	8.36	8.91	9.94	8.88	8.74	0.47	9.24	0.60	1.06	0.081	3.2E-01
Mtr.48451.1.S1_at	2.74	3.56	2.74	2.70	2.71	2.67	3.01	0.47	2.69	0.02	0.89	-0.164	3.0E-01
Mtr.6138.1.S1_at	3.95	4.76	3.93	3.86	3.92	3.91	4.21	0.47	3.89	0.03	0.92	-0.114	3.1E-01
Mtr.16.1.S1_s_at	8.94	9.86	9.59	9.39	9.57	9.52	9.46	0.47	9.49	0.09	1.00	0.004	9.2E-01
Mtr.8357.1.S1_s_at	7.18	7.53	8.11	7.58	7.66	7.59	7.61	0.47	7.61	0.04	1.00	0.001	9.9E-01
Mtr.11820.1.S1_at	4.07	4.11	4.91	4.78	4.74	4.70	4.36	0.47	4.74	0.04	1.09	0.120	2.4E-01
Mtr.35699.1.S1_at	9.02	8.08	8.59	8.71	8.82	8.74	8.56	0.47	8.75	0.06	1.02	0.032	5.3E-01
Mtr.17004.1.S1_at	3.37	4.13	3.27	3.25	3.26	4.18	3.59	0.47	3.56	0.53	0.99	-0.010	9.5E-01
Mtr.39228.1.S1_at	6.99	6.72	7.64	7.07	6.95	7.27	7.11	0.47	7.10	0.16	1.00	-0.004	9.5E-01
Mtr.51353.1.S1_at	2.67	3.47	2.64	2.62	2.61	2.64	2.93	0.47	2.62	0.01	0.90	-0.158	3.3E-01
Mtr.38703.1.S1_at	8.25	8.25	9.07	8.60	8.94	8.60	8.52	0.47	8.71	0.19	1.02	0.032	5.5E-01
Mtr.1629.1.S1_at	4.27	3.53	3.40	3.27	3.40	3.31	3.73	0.47	3.33	0.07	0.89	-0.166	2.2E-01
Mtr.14143.1.S1_at	8.54	8.23	9.16	8.75	8.76	8.58	8.64	0.47	8.70	0.10	1.01	0.010	8.5E-01
Mtr.8323.1.S1_at	9.40	8.54	9.30	9.08	9.19	9.11	9.08	0.47	9.13	0.05	1.01	0.008	8.6E-01
Mtr.8447.1.S1_s_at	8.43	7.62	8.45	8.43	8.30	8.22	8.17	0.47	8.32	0.11	1.02	0.027	6.1E-01
Mtr.33162.1.S1_at	8.27	8.30	9.10	8.08	8.16	8.06	8.56	0.47	8.10	0.05	0.95	-0.079	1.7E-01
Mtr.24077.1.S1_at	2.65	3.47	2.66	2.64	2.64	2.63	2.93	0.47	2.64	0.01	0.90	-0.152	3.4E-01
Mtr.35158.1.S1_at	7.28	6.46	7.28	7.24	7.24	7.53	7.01	0.47	7.34	0.16	1.05	0.066	3.2E-01
Mtr.31451.1.S1_at	2.99	3.80	2.98	2.91	2.92	2.93	3.26	0.47	2.92	0.01	0.90	-0.157	2.9E-01
Mtr.27160.1.S1_at	2.36	3.29	2.70	3.29	4.87	4.22	2.78	0.47	4.12	0.80	1.48	0.568	6.6E-02

Mtr.2658.1.S1_at	6.89	6.11	6.04	6.07	6.14	6.06	6.35	0.47	6.09	0.04	0.96	-0.060	4.0E-01
Mtr.42814.1.S1_at	9.63	8.68	9.15	9.23	9.36	9.54	9.15	0.47	9.38	0.16	1.02	0.035	4.8E-01
Mtr.27523.1.S1_at	4.01	3.14	3.88	3.88	3.76	4.18	3.68	0.47	3.94	0.21	1.07	0.098	4.3E-01
Mtr.24015.1.S1_at	2.92	3.64	2.76	2.73	2.72	2.98	3.11	0.47	2.81	0.15	0.91	-0.144	3.6E-01
Mtr.36067.1.S1_at	3.43	4.22	3.38	3.35	3.23	3.34	3.68	0.47	3.31	0.07	0.90	-0.153	2.5E-01
Mtr.38279.1.S1_at	9.11	10.05	9.60	9.43	9.38	9.55	9.59	0.47	9.45	0.09	0.99	-0.020	6.5E-01
Mtr.7046.1.S1_at	5.42	4.67	5.54	5.14	5.32	5.38	5.21	0.47	5.28	0.12	1.01	0.019	8.2E-01
Mtr.50781.1.S1_at	2.88	3.70	2.88	2.81	2.79	2.88	3.15	0.47	2.83	0.05	0.90	-0.157	3.0E-01
Mtr.22679.1.S1_s_at	2.57	3.38	2.54	2.53	2.53	2.50	2.83	0.47	2.52	0.02	0.89	-0.167	3.2E-01
Mtr.2172.1.S1_at	3.70	4.37	3.46	3.66	3.66	3.73	3.85	0.47	3.68	0.04	0.96	-0.062	5.9E-01
Mtr.10106.1.S1_at	5.24	4.88	5.81	5.21	5.20	5.54	5.31	0.47	5.32	0.19	1.00	0.002	9.9E-01
Mtr.35310.1.S1_at	7.83	7.09	7.96	8.18	8.09	7.84	7.63	0.47	8.04	0.17	1.05	0.075	2.3E-01
Mtr.35375.1.S1_s_at	9.57	8.63	9.14	9.25	9.21	9.26	9.11	0.47	9.24	0.02	1.01	0.020	6.7E-01
Mtr.13539.1.S1_at	2.42	2.81	3.36	3.14	2.79	3.32	2.86	0.47	3.08	0.27	1.08	0.106	5.3E-01
Mtr.1317.1.S1_at	3.56	2.64	3.28	3.34	3.22	3.28	3.16	0.47	3.28	0.06	1.04	0.052	7.0E-01
Mtr.26507.1.S1_at	9.22	8.29	8.90	9.41	9.21	9.28	8.80	0.47	9.30	0.10	1.06	0.079	1.5E-01
Mtr.32638.1.S1_s_at	8.01	7.82	7.11	7.73	6.89	7.47	7.65	0.47	7.36	0.43	0.96	-0.055	4.8E-01
Mtr.3772.1.S1_at	6.64	6.26	7.20	6.26	6.79	6.58	6.70	0.47	6.54	0.26	0.98	-0.034	6.5E-01
Mtr.20755.1.S1_at	9.72	8.83	9.55	9.57	9.50	9.47	9.37	0.47	9.51	0.05	1.02	0.022	6.3E-01
Mtr.23583.1.S1_at	2.76	3.53	2.68	2.69	2.58	2.62	2.99	0.47	2.63	0.05	0.88	-0.185	2.6E-01
Mtr.10115.1.S1_at	7.93	7.22	8.12	8.26	7.98	8.24	7.76	0.47	8.16	0.15	1.05	0.073	2.3E-01
Mtr.31301.1.S1_s_at	9.93	9.77	10.65	10.32	10.31	10.12	10.12	0.47	10.25	0.11	1.01	0.019	6.6E-01
Mtr.31918.1.S1_at	10.02	9.32	10.22	9.76	9.97	9.86	9.86	0.47	9.86	0.10	1.00	0.001	9.8E-01
Mtr.2711.1.S1_at	7.51	6.58	6.90	6.93	7.11	6.98	7.00	0.47	7.00	0.09	1.00	0.002	9.8E-01
Mtr.13923.1.S1_at	3.11	2.33	2.27	2.28	2.28	2.27	2.57	0.47	2.28	0.01	0.89	-0.176	3.4E-01
Mtr.38927.1.S1_at	9.69	8.82	8.94	9.55	9.38	9.53	9.15	0.47	9.49	0.10	1.04	0.052	2.9E-01
Mtr.39747.1.S1_at	7.21	7.66	8.16	7.68	7.66	7.66	7.68	0.47	7.67	0.01	1.00	-0.002	9.7E-01
Mtr.48617.1.S1_at	7.10	6.20	6.89	7.01	6.83	6.96	6.73	0.47	6.93	0.09	1.03	0.043	5.1E-01
Mtr.33754.1.S1_at	6.90	7.55	6.63	6.95	6.88	6.93	7.03	0.47	6.92	0.03	0.98	-0.023	7.1E-01
Mtr.27607.1.S1_at	7.33	6.80	7.74	8.09	7.98	7.84	7.29	0.47	7.97	0.13	1.09	0.129	7.3E-02
Mtr.17090.1.S1_at	4.98	4.74	5.65	4.98	5.57	5.63	5.12	0.47	5.40	0.36	1.05	0.075	4.7E-01
Mtr.31284.1.S1_at	5.27	4.53	5.41	5.40	5.40	5.40	5.07	0.47	5.40	0.00	1.07	0.091	2.9E-01
Mtr.13264.1.S1_at	10.43	9.85	10.78	10.54	10.72	10.28	10.36	0.47	10.52	0.22	1.02	0.022	6.2E-01
Mtr.15650.1.S1_at	9.47	8.93	9.87	9.47	9.44	9.22	9.43	0.47	9.38	0.14	0.99	-0.007	8.7E-01
Mtr.23054.1.S1_at	3.14	3.95	3.14	3.08	3.12	3.10	3.41	0.47	3.10	0.02	0.91	-0.138	3.2E-01
Mtr.42489.1.S1_at	11.44	10.84	11.76	11.57	11.63	11.59	11.35	0.47	11.59	0.03	1.02	0.031	4.2E-01
Mtr.1791.1.S1_at	6.24	5.94	6.86	6.23	5.91	6.28	6.35	0.47	6.14	0.20	0.97	-0.047	5.3E-01
Mtr.41186.1.S1_at	8.25	7.44	8.26	8.32	8.14	8.24	7.98	0.47	8.23	0.09	1.03	0.045	4.2E-01
Mtr.39901.1.S1_at	9.01	8.12	8.83	9.22	9.11	9.22	8.65	0.47	9.18	0.06	1.06	0.086	1.3E-01
Mtr.44623.1.S1_at	2.48	3.28	2.44	2.44	2.43	2.42	2.73	0.47	2.43	0.01	0.89	-0.170	3.3E-01
Mtr.35171.1.S1_at	7.51	6.70	7.51	7.49	7.19	7.76	7.24	0.47	7.48	0.29	1.03	0.048	4.9E-01
Mtr.5257.1.S1_at	6.02	5.25	6.10	6.16	5.99	6.01	5.79	0.47	6.05	0.09	1.05	0.065	3.9E-01
Mtr.44582.1.S1_at	11.14	10.27	11.02	10.95	11.05	10.86	10.81	0.47	10.95	0.09	1.01	0.019	6.3E-01
Mtr.25026.1.S1_at	3.49	4.30	3.48	3.39	3.35	3.43	3.76	0.47	3.39	0.04	0.90	-0.148	2.5E-01
Mtr.44749.1.S1_at	6.90	6.15	7.02	7.11	7.10	7.02	6.69	0.47	7.08	0.05	1.06	0.081	2.3E-01
Mtr.10007.1.S1_at	10.17	9.55	10.47	10.29	10.30	10.34	10.06	0.47	10.31	0.03	1.02	0.035	4.1E-01
Mtr.50316.1.S1_at	8.93	8.15	8.99	8.84	8.86	8.89	8.69	0.47	8.86	0.03	1.02	0.029	5.5E-01
Mtr.42490.1.S1_at	7.95	7.27	8.17	8.23	7.99	8.09	7.79	0.47	8.10	0.12	1.04	0.056	3.3E-01
Mtr.51460.1.S1_at	4.23	5.00	4.14	4.12	3.97	3.88	4.45	0.47	3.99	0.12	0.90	-0.160	1.7E-01
Mtr.44901.1.S1_at	5.19	4.42	5.28	5.12	5.55	5.21	4.96	0.47	5.29	0.22	1.07	0.093	3.3E-01
Mtr.45362.1.S1_at	8.80	8.03	8.88	8.65	8.44	8.68	8.57	0.47	8.59	0.13	1.00	0.004	9.4E-01
Mtr.1174.1.S1_at	12.41	13.27	12.52	12.76	12.96	12.92	12.73	0.47	12.88	0.10	1.01	0.016	6.3E-01
Mtr.19804.1.S1_at	7.56	6.83	7.70	7.58	7.69	7.53	7.36	0.47	7.60	0.08	1.03	0.046	4.4E-01
Mtr.41464.1.S1_at	8.04	7.31	8.18	8.13	8.09	8.27	7.84	0.47	8.16	0.09	1.04	0.058	3.1E-01
Mtr.46275.1.S1_at	3.22	2.84	3.77	3.47	3.37	3.36	3.28	0.47	3.40	0.06	1.04	0.054	6.7E-01
Mtr.29163.1.S1_at	6.22	5.79	6.73	6.16	6.19	6.10	6.24	0.47	6.15	0.05	0.98	-0.022	7.5E-01
Mtr.24853.1.S1_s_at	7.95	7.08	7.83	7.64	7.72	7.77	7.62	0.47	7.71	0.07	1.01	0.017	7.6E-01
Mtr.15401.1.S1_at	2.80	3.59	2.75	2.73	2.69	2.79	3.04	0.47	2.74	0.05	0.90	-0.154	3.2E-01
Mtr.7202.1.S1_s_at	8.30	7.42	8.15	8.23	8.13	8.04	7.95	0.47	8.13	0.09	1.02	0.032	5.6E-01
Mtr.32991.1.S1_at	8.36	9.25	8.56	9.35	9.22	9.51	8.72	0.47	9.36	0.15	1.07	0.102	8.8E-02
Mtr.49497.1.S1_at	8.60	8.44	9.32	8.63	8.80	8.59	8.78	0.47	8.67	0.11	0.99	-0.019	7.0E-01
Mtr.3171.1.S1_at	7.99	7.16	7.97	7.54	7.72	7.34	7.71	0.47	7.53	0.19	0.98	-0.033	5.9E-01
Mtr.15870.1.S1_at	10.71	10.05	10.96	10.95	11.02	10.91	10.58	0.47	10.96	0.05	1.04	0.051	2.3E-01
Mtr.43069.1.S1_at	7.12	6.30	7.11	6.87	7.27	6.92	6.84	0.47	7.02	0.22	1.03	0.038	5.8E-01
Mtr.26338.1.S1_at	9.55	8.99	9.93	9.73	9.60	9.64	9.49	0.47	9.66	0.06	1.02	0.025	5.8E-01
Mtr.33436.1.S1_at	3.88	2.94	3.33	3.28	3.33	3.48	3.38	0.47	3.36	0.10	0.99	-0.009	9.5E-01
Mtr.6856.1.S1_at	8.24	7.35	8.07	8.95	9.19	9.12	7.89	0.47	9.09	0.13	1.15	0.204	1.3E-02
Mtr.13859.1.S1_at	7.35	6.45	7.13	7.64	7.27	7.73	6.97	0.47	7.55	0.24	1.08	0.114	1.3E-01
Mtr.32067.1.S1_s_at	5.80	5.89	6.66	5.85	6.02	5.78	6.12	0.47	5.88	0.12	0.96	-0.057	4.5E-01
Mtr.15743.1.S1_at	7.93	7.22	8.10	8.03	7.92	7.97	7.75	0.47	7.97	0.05	1.03	0.041	4.6E-01
Mtr.7241.1.S1_s_at	7.47	7.43	8.26	8.43	8.94	8.13	7.72	0.47	8.50	0.41	1.10	0.140	9.5E-02
Mtr.46111.1.S1_at	8.60	8.20	9.13	9.00	8.67	8.60	8.64	0.47	8.76	0.21	1.01	0.019	7.1E-01
Mtr.46035.1.S1_at	9.79	8.87	9.53	9.58	9.43	9.42	9.40	0.47	9.48	0.09	1.01	0.013	7.7E-01
Mtr.28680.1.S1_at	9.26	9.48	10.16	9.78	9.78	9.60	9.63	0.47	9.72	0.11	1.01	0.013	7.7E-01
Mtr.29685.1.S1_at	4.39	3.48	4.10	4.12	4.25	4.39	3.99	0.47	4.26	0.14	1.07	0.092	4.0E-01
Mtr.45082.1.S1_at	8.87	7.93	8.48	8.61	8.53	8.40	8.43	0.47	8.51	0.10	1.01	0.015	7.7E-01
Mtr.23565.1.S1_at	2.86	3.65	2.82	2.80	2.75	2.77	3.11	0.47	2.77	0.03	0.89	-0.167	2.8E-01
Mtr.29247.1.S1_s_at	6.64	5.76	6.49	6.45	6.63	6.58	6.30	0.47	6.55	0.10	1.04	0.057	4.1E-01
Mtr.4702.1.S1_s_at	10.99	10.13	10.23	10.46	10.53	10.44	10.45	0.47	10.48	0.05	1.00	0.004	9.2E-01
Mtr.38895.1.S1_at	11.50	10.74	11.60	11.21	11.01	11.12	11.28	0.47	11.11	0.10	0.98	-0.022	5.7E-01
Mtr.38094.1.S1_at	11.70	10.81	11.02	10.87	10.90	10.92	11.18	0.47	10.90	0.03	0.97	-0.037	3.6E-01
Mtr.7242.1.S1_at	12.74	13.22	13.68	13.10	13.28	12.95	13.21	0.47	13.11	0.16	0.99	-0.011	7.4E-01
Mtr.5265.1.S1_at	6.57	5.84	6.71	6.57	6.90	6.50	6.37	0.47	6.66	0.22	1.04	0.063	4.0E-01
Mtr.10184.1.S1_at	9.35	8.45	9.13	9.28	9.34	9.18	8.98	0.47	9.27	0.08	1.03	0.046	3.5E-01
Mtr.39570.1.S1_at	9.42	8.52	9.20	9.39	9.23	9.22	9.05	0.47	9.28	0.10	1.03	0.037	4.5E-01
Mtr.20843.1.S1_at	7.75	6.93	7.74	7.96	7.72	7.88	7.47	0.47	7.86	0.12	1.05	0.072	2.5E-01
Mtr.7541.1.S1_at	9.64	8.74	9.44	9.45	9.51	9.39	9.27	0.47	9.45	0.06	1.02	0.027	5.6E-01
Mtr.11815.1.S1_at	8.25	7.34	7.97	8.31	8.57	8.23	7.85	0.47	8.37	0.18	1.07	0.092	1.5E-01
Mtr.1663.1.S1_at	4.92	4.11	4.92	4.81	4.99	5.14	4.65	0.47	4.98	0.17	1.07	0.099	3.2E-01
Mtr.12649.1.S1_at	10.44	9.56	10.28	10.15	10.15	10.23	10.09	0.47	10.17	0.05	1.01	0.011	7.8E-01
Mtr.32210.1.S1_at	3.49	3.90	2.96	3.49	3.22	3.66	3.45	0.47	3.46	0.22	1.00	0.002	9.9E-01
Mtr.41923.1.S1_at	8.14	8.52	9.08	8.55	8.74	8.42	8.58	0.47	8.57	0.16	1.00	-0.002	9.7E-01
Mtr.49779.1.S1_at	10.95	11.82	11.68	11.55	11.64	11.52	11.48	0.47	11.57	0.06	1.01	0.011	7.7E-01
Mtr.31957.1.S1_at	3.43	4.24	3.42	3.42	3.25	3.41	3.70	0.47	3.36	0.09	0.91	-0.138	2.9E-01
Mtr.22635.1.S1_at	8.39	7.72	8.63	8.75	8.65	8.35	8.25	0.47	8.58	0.21	1.04	0.058	3.2E-01
Mtr.5658.1.S1_at	7.98	7.17	7.99	7.86	7.95	8.03	7.71	0.47	7.95	0.08	1.03	0.043	4.4E-01
Mtr.11520.1.S1_s_at	5.37	4.46	5.13	5.10	5.20	5.16	4.99	0.47	5.16	0.05	1.03	0.048	5.7E-01
Mtr.37624.1.S1_at	2.57	3.32	2.46	2.52	2.51	2.51	2.79	0.47	2.51	0.01	0.90	-0.149	3.7E-01
Mtr.45444.1.S1_at	3.85	3.94	3.09	3.88	3.87	4.65	3.63	0.47	4.13	0.45	1.14	0.188	2.5E-01
Mtr.50425.1.S1_s_at	3.32	4.08	3.24	3.22	3.07	3.07	3.55	0.47	3.12	0.09	0.88	-0.183	2.0E-01
Mtr.5643.1.S1_s_at	7.65	7.04	7.96	7.65	7.64	7.48	7.55	0.47	7.59	0.10	1.01	0.008	8.8E-01
Mtr.38476.1.S1_at	9.86	10.56	10.75	9.80	9.91	9.99	10.39	0.47	9.90	0.10	0.95	-0.069	1.5E-01
Mtr.43959.1.S1_at	12.34	11.78	12.71	12.56	12.40	12.38	12.27	0.47	12.45	0.10	1.01	0.020	5.7E-01
Mtr.42225.1.S1_at	6.49	6.63	7.36	6.61	6.61	6.59	6.83	0.47	6.60	0.01	0.97	-0.049	4.5E-01
Mtr.41961.1.S1_at	12.25	11.31	11.80	11.83	12.00	11.90	11.79	0.47	11.91	0.08	1.01	0.015	6.8E-01
Mtr.24886.1.S1_at	10.37	9.47	10.15	10.24	10.24	10.24	10.00	0.47	10.24	0.00	1.02	0.034	4.2E-01
Mtr.21843.1.S1_at	2.74	3.54	2.71	2.70	2.65	2.69	3.00	0.47	2.68	0.02	0.89	-0.163	3.0E-01
Mtr.31874.1.S1_at	2.79	3.55	2.70	2.67	2.65	2.64	3.01	0.47	2.66	0.02	0.88	-0.183	2.6E-01
Mtr.38956.1.S1_at	8.52	8.13	9.07	8.98	9.03	9.09	8.57	0.47	9.03	0.06	1.05	0.075	1.7E-01
Mtr.8981.1.S1_at	9.30	9.63	8.71	8.72	9.15	8.83	9.21	0.47	8.90	0.22	0.97	-0.050	3.5E-01
Mtr.33851.1.S1_at	5.68	5.82	6.55	5.96	6.29	6.16	6.02	0.47	6.14	0.17	1.02	0.029	7.0E-01
Mtr.39999.1.S1_at	6.26	5.38	5.54	6.40	6.61	6.72	5.73	0.47	6.58	0.16	1.15	0.200	4.1E-02
Mtr.26010.1.S1_at	6.80	6.40	7.34	6.81	7.34	6.55	6.85	0.47	6.90	0.40	1.01	0.011	8.9E-01
Mtr.27333.1.S1_at	2.99	2.19	3.01	2.94	2.93	3.00	2.73	0.47	2.96	0.04	1.09	0.118	4.4E-01
Mtr.37728.1.S1_s_at	11.24	10.30	10.82	11.10	10.92	11.22	10.78	0.47	11.08	0.15	1.03	0.039	3.6E-01
Mtr.41586.1.S1_at	10.61	10.95	11.54	10.60	10.46	10.63	11.03	0.47	10.56	0.09	0.96	-0.063	1.6E-01
Mtr.37937.1.S1_at	11.74	11.05	10.85	11.85	11.95	12.05	11.22	0.47	11.95	0.10	1.07	0.091	5.7E-02
Mtr.32781.1.S1_at	4.28	3.49	4.32	4.27	4.25	4.22	4.03	0.47	4.25	0.03	1.05	0.075	4.7E-01
Mtr.31273.1.S1_at	12.26	11.40	12.15	12.10	11.93	12.04	11.94	0.47	12.02	0.09	1.01	0.010	7.7E-01
Mtr.5240.1.S1_at	8.17	8.01	7.29	7.74	7.74	7.79	7.82	0.47	7.76	0.03	0.99	-0.013	8.1E-01
Mtr.24459.1.S1_a_at	2.95	2.21	3.07	2.86	3.72	2.94	2.74	0.47	3.17	0.47	1.16	0.210	3.3E-01
Mtr.17362.1.S1_at	4.53	4.09	5.03	4.59	5.05	4.83	4.55	0.47	4.82	0.23	1.06	0.084	4.1E-01
Mtr.45095.1.S1_at	8.38	8.03	8.96	8.84	8.84	8.65	8.46	0.47	8.78	0.11	1.04	0.054	3.1E-01
Mtr.42636.1.S1_at	9.08	8.37	9.26	8.98	8.86	9.09	8.90	0.47	8.97	0.12	1.01	0.011	8.1E-01
Mtr.14271.1.S1_at	8.49	9.07	9.41	9.81	10.45	10.36	8.99	0.47	10.21	0.34	1.14	0.183	2.2E-02
Mtr.29752.1.S1_at	2.27	3.07	2.26	2.25	2.25	2.25	2.53	0.47	2.25	0.00	0.89	-0.170	3.6E-01
Mtr.35716.1.S1_at	5.93	5.02	5.29	5.24	5.30	5.36	5.41	0.47	5.30	0.06	0.98	-0.030	7.1E-01
Mtr.13632.1.S1_at	10.74	10.19	9.81	10.52	10.48	10.67	10.24	0.47	10.56	0.10	1.03	0.043	3.2E-01
Mtr.6538.1.S1_at	3.17	3.44	4.08	4.16	3.44	3.39	3.56	0.47	3.67	0.43	1.03	0.041	8.0E-01
Mtr.24713.1.S1_at	10.97	11.90	11.53	11.21	11.10	11.14	11.47	0.47	11.15	0.06	0.97	-0.041	3.1E-01
Mtr.6224.1.S1_at	2.21	3.01	2.19	2.19	2.19	2.18	2.47	0.47	2.19	0.00	0.89	-0.175	3.6E-01

Mtr.40997.1.S1_s_at	10.34	11.13	11.16	11.07	11.19	10.80	10.88	0.47	11.02	0.20	1.01	0.019	6.5E-01
Mtr.33586.1.S1_at	8.68	7.86	7.87	8.33	8.36	8.49	8.14	0.47	8.40	0.09	1.03	0.046	4.0E-01
Mtr.1981.1.S1_x_at	2.73	3.52	2.70	2.69	2.69	2.64	2.98	0.47	2.67	0.03	0.90	-0.160	3.1E-01
Mtr.13936.1.S1_at	7.92	7.07	7.83	7.82	7.87	7.93	7.61	0.47	7.87	0.06	1.03	0.050	3.8E-01
Mtr.40555.1.S1_at	12.94	12.01	12.37	12.67	12.25	12.76	12.44	0.47	12.56	0.27	1.01	0.014	7.2E-01
Mtr.9886.1.S1_at	9.17	8.39	9.23	8.91	8.98	8.93	8.93	0.47	8.94	0.04	1.00	0.001	9.8E-01
Mtr.11338.1.S1_at	8.98	8.09	8.26	8.62	8.84	8.88	8.44	0.47	8.78	0.14	1.04	0.056	3.0E-01
Mtr.2056.1.S1_at	6.66	6.92	7.57	7.34	7.19	7.00	7.05	0.47	7.17	0.17	1.02	0.025	6.9E-01
Mtr.9435.1.S1_at	8.71	7.81	8.49	8.54	8.53	8.53	8.34	0.47	8.53	0.01	1.02	0.034	5.0E-01
Mtr.42233.1.S1_at	4.01	3.36	4.27	4.08	3.95	4.00	3.88	0.47	4.01	0.06	1.03	0.049	6.5E-01
Mtr.5886.1.S1_s_at	8.01	8.23	8.91	8.33	8.46	8.37	8.38	0.47	8.39	0.06	1.00	0.001	9.9E-01
Mtr.52327.1.S1_at	3.15	3.95	3.12	3.13	3.05	3.04	3.41	0.47	3.07	0.05	0.90	-0.151	2.8E-01
Mtr.41860.1.S1_at	4.15	4.80	3.89	4.25	4.21	4.39	4.28	0.47	4.29	0.09	1.00	0.002	9.9E-01
Mtr.49033.1.S1_s_at	9.08	10.02	9.57	9.26	9.44	9.20	9.56	0.47	9.30	0.13	0.97	-0.039	4.1E-01
Mtr.27815.1.S1_at	9.49	8.55	9.04	9.11	9.19	9.21	9.03	0.47	9.17	0.06	1.02	0.023	6.3E-01
Mtr.42558.1.S1_at	6.68	5.75	6.14	6.39	6.31	5.91	6.19	0.47	6.20	0.25	1.00	0.003	9.7E-01
Mtr.5270.1.S1_at	8.59	7.73	7.84	8.41	8.43	8.49	8.05	0.47	8.44	0.04	1.05	0.068	2.3E-01
Mtr.30779.1.S1_s_at	10.75	11.24	11.68	11.11	10.92	10.93	11.22	0.47	10.99	0.10	0.98	-0.031	4.4E-01
Mtr.17150.1.S1_at	2.70	3.50	2.69	2.69	2.54	2.68	2.97	0.47	2.64	0.09	0.89	-0.170	3.0E-01
Mtr.37245.1.S1_s_at	13.84	12.95	13.16	13.45	12.95	13.45	13.32	0.47	13.28	0.29	1.00	-0.004	9.2E-01
Mtr.5213.1.S1_at	8.55	7.65	7.89	7.83	8.09	8.10	8.03	0.47	8.00	0.15	1.00	-0.005	9.3E-01
Mtr.4230.1.S1_at	2.85	3.64	2.81	2.79	2.77	2.76	3.10	0.47	2.78	0.01	0.90	-0.158	3.0E-01
Mtr.43115.1.S1_at	12.32	11.42	11.66	12.01	12.33	12.16	11.80	0.47	12.16	0.16	1.03	0.044	2.7E-01
Mtr.36196.1.S1_at	3.19	2.29	2.93	2.72	3.18	2.63	2.80	0.47	2.84	0.30	1.01	0.020	9.1E-01
Mtr.35657.1.S1_at	5.23	5.31	6.07	5.94	5.46	5.40	5.54	0.47	5.60	0.30	1.01	0.017	8.5E-01
Mtr.46675.1.S1_at	2.77	3.58	2.77	2.75	2.72	2.77	3.04	0.47	2.75	0.02	0.90	-0.147	3.4E-01
Mtr.27993.1.S1_s_at	10.25	9.38	10.13	9.91	9.95	9.99	9.92	0.47	9.95	0.04	1.00	0.004	9.2E-01
Mtr.42550.1.S1_at	3.61	2.88	2.74	2.71	2.74	2.74	3.08	0.47	2.73	0.02	0.89	-0.172	2.7E-01
Mtr.1148.1.S1_s_at	8.39	7.59	8.42	8.51	8.51	8.71	8.13	0.47	8.58	0.11	1.05	0.077	1.9E-01
Mtr.25780.1.S1_at	4.25	5.02	4.18	4.15	3.98	4.07	4.48	0.47	4.06	0.09	0.91	-0.142	2.0E-01
Mtr.13725.1.S1_at	8.82	7.96	8.70	8.52	8.64	8.47	8.50	0.47	8.54	0.09	1.01	0.008	8.7E-01
Mtr.26100.1.S1_at	3.21	3.98	3.15	3.12	3.08	3.08	3.44	0.47	3.10	0.02	0.90	-0.154	2.7E-01
Mtr.39148.1.S1_at	7.91	6.99	7.62	7.41	7.30	7.29	7.51	0.47	7.33	0.07	0.98	-0.034	5.6E-01
Mtr.9219.1.S1_at	9.35	8.63	9.50	9.42	9.40	9.22	9.16	0.47	9.35	0.11	1.02	0.029	5.4E-01
Mtr.44500.1.S1_at	4.78	4.67	5.53	4.94	5.73	5.88	5.00	0.47	5.52	0.50	1.10	0.143	2.6E-01
Mtr.28425.1.S1_at	3.82	3.01	3.81	4.14	4.22	4.30	3.55	0.47	4.22	0.08	1.19	0.250	7.0E-02
Mtr.21769.1.S1_s_at	6.67	6.48	7.36	7.59	7.41	7.56	6.84	0.47	7.52	0.10	1.10	0.137	6.8E-02
Mtr.43557.1.S1_x_at	5.82	5.49	6.41	6.73	7.87	7.10	5.91	0.47	7.23	0.58	1.22	0.293	3.7E-02
Mtr.50290.1.S1_s_at	10.08	9.15	9.64	11.26	11.38	11.16	9.62	0.47	11.27	0.11	1.17	0.228	4.0E-03
Mtr.17141.1.S1_at	7.11	6.44	7.34	7.20	7.32	7.14	6.96	0.47	7.22	0.09	1.04	0.052	4.0E-01
Mtr.1587.1.S1_at	7.37	7.84	6.91	7.20	7.39	7.36	7.37	0.47	7.32	0.10	0.99	-0.011	8.5E-01
Mtr.38171.1.S1_at	11.40	10.53	11.25	11.74	12.01	12.00	11.06	0.47	11.92	0.15	1.08	0.108	3.9E-02
Mtr.10116.1.S1_at	10.61	9.69	10.27	10.27	10.14	10.06	10.19	0.47	10.16	0.11	1.00	-0.005	9.1E-01
Mtr.18998.1.S1_at	2.66	3.45	2.63	2.62	2.60	2.62	2.91	0.47	2.61	0.02	0.90	-0.157	3.3E-01
Mtr.5767.1.S1_at	9.00	8.10	8.78	8.58	8.44	8.85	8.63	0.47	8.62	0.21	1.00	0.000	9.9E-01
Mtr.29336.1.S1_x_at	4.20	3.48	3.33	3.32	3.32	4.06	3.67	0.47	3.57	0.43	0.97	-0.041	7.9E-01
Mtr.1660.1.S1_at	3.40	2.58	3.37	3.41	3.37	3.37	3.11	0.47	3.38	0.02	1.09	0.118	3.8E-01
Mtr.43206.1.S1_at	13.65	12.73	13.30	13.50	13.58	13.61	13.22	0.47	13.56	0.06	1.03	0.037	2.8E-01
Mtr.46921.1.S1_at	8.39	7.56	8.35	8.21	8.30	8.20	8.10	0.47	8.24	0.06	1.02	0.024	6.4E-01
Mtr.12356.1.S1_at	12.17	11.24	11.80	11.80	11.75	11.70	11.73	0.47	11.75	0.05	1.00	0.002	9.6E-01
Mtr.5551.1.S1_at	7.94	7.31	8.22	7.99	8.13	8.21	7.82	0.47	8.11	0.11	1.04	0.052	3.6E-01
Mtr.36272.1.S1_at	2.26	3.06	2.25	2.25	2.24	2.24	2.53	0.47	2.24	0.00	0.89	-0.171	3.5E-01
Mtr.13058.1.S1_at	12.09	11.20	11.89	11.90	11.83	11.88	11.73	0.47	11.87	0.03	1.01	0.018	6.2E-01
Mtr.29276.1.S1_s_at	4.52	3.68	4.45	4.98	4.45	5.29	4.22	0.47	4.91	0.42	1.16	0.218	1.3E-01
Mtr.21725.1.S1_at	2.59	3.39	2.57	2.55	2.56	2.54	2.85	0.47	2.55	0.01	0.89	-0.161	3.3E-01
Mtr.45748.1.S1_at	2.26	3.06	2.24	2.24	2.23	2.24	2.52	0.47	2.24	0.00	0.89	-0.171	3.5E-01
Mtr.42050.1.S1_at	8.28	7.51	8.35	8.49	8.48	8.25	8.05	0.47	8.41	0.14	1.04	0.063	2.7E-01
Mtr.9618.1.S1_at	9.20	8.30	8.95	9.12	8.94	9.17	8.81	0.47	9.07	0.12	1.03	0.042	4.0E-01
Mtr.34483.1.S1_s_at	11.42	10.49	10.94	11.12	10.96	11.05	10.95	0.47	11.04	0.08	1.01	0.012	7.5E-01
Mtr.33348.1.S1_at	6.32	5.53	6.35	6.25	6.36	6.34	6.06	0.47	6.32	0.06	1.04	0.060	4.0E-01
Mtr.46218.1.S1_at	2.77	3.65	2.96	3.09	2.88	2.92	3.13	0.47	2.96	0.11	0.95	-0.077	5.9E-01
Mtr.36359.1.S1_at	3.60	2.68	3.27	3.27	3.27	3.26	3.18	0.47	3.27	0.01	1.03	0.037	7.7E-01
Mtr.43726.1.S1_at	7.69	7.28	8.20	7.94	7.99	7.54	7.72	0.47	7.83	0.25	1.01	0.019	7.5E-01
Mtr.26964.1.S1_a_at	2.72	3.51	2.70	2.69	2.67	2.67	2.98	0.47	2.68	0.01	0.90	-0.150	3.3E-01
Mtr.45889.1.S1_at	10.17	9.27	9.50	9.83	9.85	9.92	9.65	0.47	9.87	0.05	1.02	0.032	4.6E-01
Mtr.12943.1.S1_at	4.09	3.36	4.22	3.64	4.10	3.72	3.89	0.47	3.82	0.25	0.98	-0.025	8.4E-01
Mtr.34426.1.S1_at	7.89	8.49	8.81	8.22	8.77	8.37	8.40	0.47	8.46	0.28	1.01	0.010	8.7E-01
Mtr.42383.1.S1_at	3.09	2.27	3.07	3.03	3.59	2.98	2.81	0.47	3.20	0.34	1.14	0.188	3.1E-01
Mtr.19536.1.S1_at	8.15	7.25	7.89	8.21	8.30	8.28	7.76	0.46	8.26	0.05	1.06	0.090	1.4E-01
Mtr.15304.1.S1_s_at	9.34	8.95	9.88	9.44	9.64	9.46	9.39	0.46	9.51	0.11	1.01	0.019	6.8E-01
Mtr.38974.1.S1_at	6.93	6.75	7.63	7.14	7.04	6.96	7.10	0.46	7.05	0.09	0.99	-0.012	8.4E-01
Mtr.13624.1.S1_at	10.74	10.74	11.55	10.90	11.33	11.01	11.01	0.46	11.08	0.22	1.01	0.009	8.3E-01
Mtr.15192.1.S1_at	2.64	3.42	2.59	2.62	2.62	2.58	2.88	0.46	2.61	0.02	0.90	-0.144	3.6E-01
Mtr.28778.1.S1_at	2.74	3.50	2.66	2.64	2.64	2.59	2.97	0.46	2.62	0.03	0.88	-0.177	2.7E-01
Mtr.43845.1.S1_at	10.80	9.94	10.05	10.49	10.71	10.66	10.26	0.46	10.62	0.11	1.03	0.049	2.7E-01
Mtr.15636.1.S1_at	2.64	3.10	3.57	2.64	2.67	2.67	3.10	0.46	2.66	0.02	0.86	-0.222	1.7E-01
Mtr.52237.1.S1_at	9.73	9.17	10.09	9.74	9.80	9.59	9.67	0.46	9.71	0.11	1.00	0.006	8.8E-01
Mtr.13231.1.S1_s_at	7.36	6.69	7.58	7.64	7.61	7.59	7.21	0.46	7.61	0.02	1.06	0.078	2.1E-01
Mtr.34586.1.S1_at	2.61	3.40	2.58	2.58	2.55	2.63	2.86	0.46	2.59	0.04	0.90	-0.144	3.7E-01
Mtr.1808.1.S1_at	7.34	6.93	7.86	7.60	7.73	7.37	7.38	0.46	7.56	0.18	1.03	0.037	5.5E-01
Mtr.41530.1.S1_at	3.42	2.92	3.85	3.15	3.85	2.95	3.39	0.46	3.32	0.47	0.98	-0.033	8.5E-01
Mtr.18483.1.S1_at	11.09	11.65	12.01	11.74	11.62	11.72	11.58	0.46	11.69	0.06	1.01	0.014	7.0E-01
Mtr.42966.1.S1_at	15.11	14.28	14.33	14.61	14.41	14.63	14.57	0.46	14.55	0.12	1.00	-0.002	9.4E-01
Mtr.28675.1.S1_at	4.69	4.20	5.13	4.60	4.80	4.55	4.67	0.46	4.65	0.13	1.00	-0.006	9.5E-01
Mtr.12925.1.S1_at	10.70	10.11	9.79	10.57	10.47	10.72	10.20	0.46	10.58	0.13	1.04	0.054	2.4E-01
Mtr.5714.1.S1_at	11.83	10.95	11.63	11.80	11.36	11.56	11.47	0.46	11.57	0.22	1.01	0.013	7.4E-01
Mtr.47101.1.S1_at	2.92	3.68	2.84	2.85	2.82	2.82	3.15	0.46	2.83	0.02	0.90	-0.153	3.0E-01
Mtr.37517.1.S1_at	8.92	8.32	9.24	8.75	8.99	8.85	8.83	0.46	8.86	0.12	1.00	0.006	9.0E-01
Mtr.30717.1.S1_at	2.68	2.84	3.55	2.67	2.62	2.61	3.02	0.46	2.63	0.03	0.87	-0.198	2.2E-01
Mtr.38496.1.S1_at	6.08	5.60	6.52	6.10	6.53	6.37	6.07	0.46	6.33	0.22	1.04	0.062	4.2E-01
Mtr.28852.1.S1_at	6.65	5.97	6.86	6.63	6.70	6.76	6.49	0.46	6.70	0.06	1.03	0.044	5.0E-01
Mtr.47309.1.S1_x_at	2.46	3.26	2.44	2.44	2.43	2.41	2.72	0.46	2.43	0.02	0.89	-0.164	3.4E-01
Mtr.32395.1.S1_s_at	9.71	10.50	10.52	10.21	10.51	10.61	10.24	0.46	10.44	0.21	1.02	0.028	5.3E-01
Mtr.44445.1.S1_at	5.06	5.26	5.94	7.46	8.36	7.80	5.42	0.46	7.87	0.45	1.45	0.539	2.8E-03
Mtr.42224.1.S1_at	7.09	6.17	6.74	7.04	6.99	7.10	6.67	0.46	7.04	0.06	1.06	0.079	2.4E-01
Mtr.5906.1.S1_at	3.54	2.83	2.67	3.60	2.62	2.65	3.01	0.46	2.96	0.55	0.98	-0.028	9.0E-01
Mtr.6976.1.S1_at	1.98	2.78	2.78	2.19	4.04	2.57	2.51	0.46	2.93	0.98	1.17	0.221	5.4E-01
Mtr.2975.1.S1_at	3.90	4.76	4.64	5.71	5.36	4.66	4.44	0.46	5.25	0.53	1.18	0.242	1.2E-01
Mtr.32775.1.S1_at	8.42	7.68	7.57	7.55	7.47	7.70	7.89	0.46	7.57	0.12	0.96	-0.059	3.2E-01
Mtr.45590.1.S1_at	7.98	7.06	7.43	7.78	7.67	7.72	7.49	0.46	7.72	0.05	1.03	0.044	4.4E-01
Mtr.13883.1.S1_at	8.48	7.59	8.26	8.69	8.38	8.57	8.11	0.46	8.55	0.15	1.05	0.075	2.0E-01
Mtr.9973.1.S1_at	5.92	4.99	5.37	5.72	5.49	5.76	5.43	0.46	5.66	0.15	1.04	0.059	4.6E-01
Mtr.32371.1.S1_s_at	8.61	7.77	8.53	8.75	8.81	8.56	8.31	0.46	8.71	0.14	1.05	0.068	2.2E-01
Mtr.18923.1.S1_at	6.94	7.51	6.59	6.92	6.76	6.65	7.01	0.46	6.77	0.14	0.97	-0.050	4.4E-01
Mtr.14393.1.S1_at	3.20	3.97	3.14	3.13	3.11	3.09	3.44	0.46	3.11	0.02	0.90	-0.144	2.9E-01
Mtr.35664.1.S1_at	8.28	9.13	8.38	8.52	8.41	8.43	8.60	0.46	8.45	0.06	0.98	-0.024	6.2E-01
Mtr.40069.1.S1_x_at	14.80	14.39	13.88	14.98	14.77	15.08	14.36	0.46	14.94	0.16	1.04	0.058	1.1E-01
Mtr.779.1.S1_at	12.44	12.43	13.24	12.86	12.80	12.61	12.70	0.46	12.76	0.13	1.00	0.007	8.5E-01
Mtr.50987.1.S1_at	2.39	3.19	2.38	2.37	2.37	2.37	2.65	0.46	2.37	0.00	0.89	-0.161	3.5E-01
Mtr.14697.1.S1_at	9.77	8.89	9.56	9.55	9.59	9.58	9.41	0.46	9.58	0.02	1.02	0.026	5.6E-01
Mtr.33088.1.S1_at	6.80	5.88	6.40	6.39	6.33	6.36	6.36	0.46	6.36	0.03	1.00	0.000	1.0E+00
Mtr.45476.1.S1_at	8.49	7.96	8.88	8.24	8.22	8.15	8.44	0.46	8.21	0.05	0.97	-0.041	4.3E-01
Mtr.7915.1.S1_at	3.21	2.94	3.84	2.68	3.14	3.26	3.33	0.46	3.03	0.31	0.91	-0.139	4.0E-01
Mtr.51779.1.S1_at	6.51	5.64	6.36	6.55	6.59	6.35	6.17	0.46	6.50	0.13	1.05	0.075	3.0E-01
Mtr.50235.1.S1_at	4.35	4.24	3.50	3.49	3.39	3.47	4.03	0.46	3.45	0.05	0.85	-0.227	9.5E-02
Mtr.20442.1.S1_at	2.16	2.96	2.16	2.15	2.15	2.15	2.43	0.46	2.15	0.00	0.89	-0.174	3.6E-01
Mtr.11373.1.S1_at	10.81	10.27	11.19	10.62	10.94	10.43	10.76	0.46	10.66	0.26	0.99	-0.013	7.7E-01
Mtr.11751.1.S1_s_at	6.84	7.24	7.77	7.55	7.55	7.47	7.28	0.46	7.52	0.04	1.03	0.047	4.2E-01
Mtr.38781.1.S1_at	9.28	8.51	9.33	9.26	9.10	9.23	9.04	0.46	9.20	0.08	1.02	0.025	6.0E-01
Mtr.6832.1.S1_at	8.60	7.95	8.85	8.60	8.71	8.65	8.47	0.46	8.65	0.05	1.02	0.031	5.3E-01
Mtr.15546.1.S1_at	8.92	8.02	8.66	8.57	8.41	8.36	8.53	0.46	8.45	0.11	0.99	-0.015	7.7E-01
Mtr.43317.1.S1_s_at	9.83	9.26	10.17	9.89	9.76	9.58	9.75	0.46	9.74	0.15	1.00	-0.001	9.8E-01
Mtr.14505.1.S1_x_at	2.99	3.78	2.97	2.96	2.89	2.88	3.24	0.46	2.91	0.04	0.90	-0.156	2.8E-01
Mtr.11216.1.S1_at	8.01	7.77	8.66	8.17	7.98	8.18	8.15	0.46	8.11	0.11	1.00	-0.007	9.0E-01
Mtr.39414.1.S1_at	8.78	8.10	8.98	8.84	8.85	8.70	8.62	0.46	8.80	0.08	1.02	0.029	5.5E-01
Mtr.6967.1.S1_at	6.84	6.13	7.00	6.81	6.96	6.81	6.66	0.46	6.86	0.09	1.03	0.043	5.0E-01
Mtr.11081.1.S1_at	9.78	8.86	9.23	9.27	9.00	9.31	9.29	0.46	9.19	0.17	0.99	-0.015	7.5E-01
Mtr.50117.1.S1_x_at	2.91	3.70	2.89	2.89	2.89	2.77	3.17	0.46	2.85	0.07	0.90	-0.151	3.1E-01
Mtr.14133.1.S1_s_at	7.82	8.38	8.73	7.76	8.40	7.88	8.31	0.46	8.02	0.34	0.96	-0.052	4.3E-01
Mtr.29680.1.S1_at	2.46	3.18	2.33	5.59	5.63	6.16	2.66	0.46	5.79	0.32	2.18	1.124	6.4E-04
Mtr.49045.1.S1_s_at	6.06	5.14	5.64	6.34	6.46	5.96	5.61	0.46	6.25	0.26	1.11	0.155	1.1E-01

Mtr.32568.1.S1_s_at	10.02	9.43	10.34	10.44	10.43	10.66	9.93	0.46	10.51	0.13	1.06	0.082	1.0E-01
Mtr.36602.1.S1_at	2.71	3.49	2.67	2.66	2.68	2.68	2.96	0.46	2.68	0.01	0.91	-0.143	3.5E-01
Mtr.50016.1.S1 at	5.72	5.04	5.92	5.39	5.37	4.89	5.56	0.46	5.22	0.28	0.94	-0.092	3.4E-01
Mtr.13292.1.S1 at	11.14	10.21	10.69	10.75	10.82	10.95	10.68	0.46	10.84	0.10	1.01	0.021	5.9E-01
Mtr.35510.1.S1 at	12.62	11.70	12.09	12.35	12.15	12.26	12.14	0.46	12.25	0.10	1.01	0.014	6.9E-01
Mtr.44143.1.S1_at	8.86	8.15	8.00	8.81	8.32	8.69	8.34	0.46	8.61	0.26	1.03	0.046	4.2E-01
Mtr.20106.1.S1_at	8.47	7.78	8.66	8.53	8.50	8.40	8.30	0.46	8.48	0.07	1.02	0.030	5.5E-01
Mtr.5333.1.S1 at	8.55	8.00	8.91	8.51	8.41	8.30	8.49	0.46	8.41	0.10	0.99	-0.014	7.8E-01
Mtr.11305.1.S1 s at	6.17	5.44	6.29	6.16	6.29	6.16	5.97	0.46	6.21	0.07	1.04	0.057	4.2E-01
Mtr.27081.1.S1 at	4.62	3.73	3.99	4.10	4.53	4.46	4.11	0.46	4.36	0.23	1.06	0.084	4.5E-01
Mtr.41735.1.S1 at	4.82	4.03	4.02	3.99	4.88	4.51	4.29	0.46	4.46	0.45	1.04	0.055	6.8E-01
Mtr.3548.1.S1_at	8.28	7.42	8.13	8.27	8.18	8.32	7.94	0.46	8.26	0.07	1.04	0.056	3.1E-01
Mtr.6950.1.S1_at	9.09	8.41	8.20	8.72	8.98	8.88	8.57	0.46	8.86	0.13	1.03	0.048	3.5E-01
Mtr.14239.1.S1_at	9.02	8.59	9.51	9.19	9.30	9.26	9.04	0.46	9.25	0.06	1.02	0.033	4.9E-01
Mtr.7933.1.S1 at	2.94	3.71	2.89	2.86	2.83	2.85	3.18	0.46	2.85	0.02	0.90	-0.158	2.8E-01
Mtr.48153.1.S1 at	8.67	9.43	9.51	9.99	10.33	9.74	9.20	0.46	10.02	0.30	1.09	0.123	6.1E-02
Mtr.35034.1.S1 x at	3.29	4.10	3.30	3.22	3.28	3.27	3.56	0.46	3.26	0.03	0.91	-0.129	3.2E-01
Mtr.8755.1.S1_at	10.75	9.93	10.72	10.66	10.69	10.72	10.47	0.46	10.69	0.03	1.02	0.030	4.5E-01
Mtr.14370.1.S1_at	3.64	3.14	4.06	3.88	4.85	4.32	3.61	0.46	4.35	0.48	1.20	0.268	1.3E-01
Mtr.16025.1.S1_at	3.90	3.06	3.81	3.89	4.06	3.80	3.59	0.46	3.92	0.13	1.09	0.126	3.0E-01
Mtr.31846.1.S1 at	7.72	8.64	8.23	7.94	7.87	7.92	8.19	0.46	7.91	0.04	0.97	-0.051	3.5E-01
Mtr.42151.1.S1 at	7.97	7.40	8.31	8.19	8.03	8.00	7.90	0.46	8.07	0.11	1.02	0.032	5.5E-01
Mtr.5284.1.S1 at	9.70	8.78	9.15	9.52	9.39	9.74	9.21	0.46	9.55	0.17	1.04	0.052	3.0E-01
Mtr.29345.1.S1_at	11.13	10.28	11.02	11.18	10.93	11.04	10.81	0.46	11.05	0.12	1.02	0.031	4.4E-01
Mtr.42783.1.S1_at	8.56	7.88	8.76	8.74	8.49	8.57	8.40	0.46	8.60	0.13	1.02	0.034	5.1E-01
Mtr.24333.1.S1_at	9.24	8.47	9.30	9.16	9.07	9.12	9.00	0.46	9.12	0.05	1.01	0.018	6.9E-01
Mtr.1751.1.S1 s at	6.51	6.84	7.42	7.02	7.44	7.21	6.92	0.46	7.23	0.21	1.04	0.062	3.6E-01
Mtr.41786.1.S1 at	8.04	8.26	8.93	7.96	8.25	7.91	8.41	0.46	8.04	0.18	0.96	-0.065	2.7E-01
Mtr.13009.1.S1 s at	8.03	7.48	8.40	8.08	8.10	8.24	7.97	0.46	8.14	0.09	1.02	0.031	5.6E-01
Mtr.39419.1.S1_at	9.27	8.45	9.23	9.41	9.50	9.66	8.98	0.46	9.52	0.13	1.06	0.084	1.2E-01
Mtr.51078.1.S1_at	2.72	3.52	2.72	2.71	2.71	2.70	2.99	0.46	2.70	0.00	0.91	-0.143	3.5E-01
Mtr.51975.1.S1_at	2.79	3.58	2.78	2.77	2.77	2.74	3.05	0.46	2.76	0.02	0.90	-0.146	3.3E-01
Mtr.28549.1.S1 at	8.67	7.77	8.40	8.52	8.33	8.56	8.28	0.46	8.47	0.12	1.02	0.033	5.2E-01
Mtr.7832.1.S1 at	10.94	10.03	10.63	10.43	10.13	10.53	10.53	0.46	10.36	0.20	0.98	-0.024	5.9E-01
Mtr.4987.1.S1 at	4.05	3.18	3.36	3.28	3.33	3.60	3.53	0.46	3.41	0.17	0.96	-0.052	6.8E-01
Mtr.30802.1.S1 at	4.06	3.25	4.03	4.03	4.01	4.02	3.78	0.46	4.02	0.01	1.06	0.090	4.1E-01
Mtr.31493.1.S1_at	2.79	3.62	2.86	2.85	2.86	2.86	3.09	0.46	2.85	0.01	0.92	-0.115	4.3E-01
Mtr.41954.1.S1 at	6.68	6.29	5.76	6.22	6.42	6.35	6.25	0.46	6.33	0.10	1.01	0.020	7.7E-01
Mtr.40598.1.S1 at	10.30	9.78	10.70	10.17	10.38	9.99	10.26	0.46	10.18	0.19	0.99	-0.011	7.9E-01
Mtr.42232.1.S1 at	10.83	10.01	10.77	10.67	10.53	10.53	10.54	0.46	10.58	0.08	1.00	0.006	8.9E-01
Mtr.35186.1.S1 at	10.99	10.25	11.10	11.24	11.09	11.01	10.78	0.46	11.11	0.11	1.03	0.044	2.9E-01
Mtr.14835.1.S1_x_at	2.72	3.50	2.69	2.68	2.64	2.67	2.97	0.46	2.66	0.02	0.90	-0.158	3.1E-01
Mtr.22300.1.S1_at	3.34	4.08	3.23	3.20	3.18	3.17	3.55	0.46	3.18	0.01	0.90	-0.157	2.4E-01
Mtr.44699.1.S1 at	6.99	6.36	7.26	6.74	6.83	6.50	6.87	0.46	6.69	0.17	0.97	-0.038	5.6E-01
Mtr.27588.1.S1 s at	9.67	8.83	9.57	9.62	9.43	9.71	9.36	0.46	9.58	0.14	1.02	0.035	4.6E-01
Mtr.44526.1.S1 at	9.44	8.55	9.18	9.09	9.16	9.15	9.06	0.46	9.13	0.04	1.01	0.012	7.9E-01
Mtr.40921.1.S1 at	7.94	7.19	8.03	7.80	7.51	7.61	7.72	0.46	7.64	0.15	0.99	-0.014	8.0E-01
Mtr.7326.1.S1_at	9.08	8.23	8.96	9.05	8.92	9.03	8.76	0.46	9.00	0.07	1.03	0.039	4.2E-01
Mtr.320.1.S1_at	8.13	9.05	8.62	8.38	8.78	8.49	8.60	0.46	8.55	0.21	0.99	-0.009	8.6E-01
Mtr.40237.1.S1 at	3.32	2.60	2.47	2.45	2.45	2.44	2.80	0.46	2.45	0.01	0.87	-0.194	2.6E-01
Mtr.39320.1.S1 at	8.97	8.10	8.81	8.78	9.08	8.71	8.62	0.46	8.86	0.19	1.03	0.038	4.7E-01
Mtr.12786.1.S1 at	4.13	3.83	4.73	4.82	5.04	4.97	4.23	0.46	4.94	0.11	1.17	0.224	6.0E-02
Mtr.31003.1.S1 at	3.19	3.96	3.14	3.13	3.10	3.21	3.43	0.46	3.15	0.06	0.92	-0.126	3.4E-01
Mtr.33039.1.S1_s_at	8.97	8.22	9.05	9.01	8.91	9.03	8.75	0.46	8.98	0.07	1.03	0.038	4.3E-01
Mtr.13582.1.S1_at	9.18	9.02	9.88	9.25	9.41	9.32	9.36	0.46	9.33	0.08	1.00	-0.005	9.1E-01
Mtr.15360.1.S1_at	3.01	3.77	2.95	2.96	2.86	2.93	3.24	0.46	2.92	0.05	0.90	-0.151	2.9E-01
Mtr.26160.1.S1 at	7.88	7.98	8.72	8.04	8.29	7.85	8.19	0.46	8.06	0.22	0.98	-0.024	6.7E-01
Mtr.42555.1.S1 at	9.46	8.98	9.90	9.80	9.77	9.89	9.45	0.46	9.82	0.06	1.04	0.056	2.4E-01
Mtr.12724.1.S1 at	11.77	11.22	10.85	10.54	10.40	10.82	11.28	0.46	10.59	0.21	0.94	-0.092	7.6E-02
Mtr.27553.1.S1_at	8.15	7.50	8.39	8.31	8.35	8.25	8.01	0.46	8.31	0.05	1.04	0.052	3.4E-01
Mtr.37242.1.S1_x_at	5.61	4.72	4.98	5.29	4.98	5.04	5.10	0.46	5.10	0.16	1.00	0.000	1.0E+00
Mtr.36224.1.S1_at	9.16	8.41	9.24	9.26	9.15	9.12	8.93	0.46	9.17	0.07	1.03	0.038	4.2E-01
Mtr.48991.1.S1 s at	5.90	6.82	6.45	7.07	6.82	6.79	6.39	0.46	6.89	0.16	1.08	0.109	1.5E-01
Mtr.31253.1.S1 at	8.03	7.67	8.58	8.20	8.01	8.48	8.09	0.46	8.23	0.23	1.02	0.024	6.7E-01
Mtr.39073.1.S1 at	12.30	11.43	11.59	11.98	11.79	12.17	11.78	0.46	11.98	0.19	1.02	0.025	5.1E-01
Mtr.28403.1.S1_s_at	3.43	4.21	3.41	3.38	3.29	3.49	3.68	0.46	3.39	0.10	0.92	-0.121	3.4E-01
Mtr.29405.1.S1_at	7.46	6.66	7.46	7.61	7.59	7.60	7.20	0.46	7.60	0.01	1.06	0.079	2.0E-01
Mtr.39544.1.S1_at	6.65	6.02	6.91	6.24	6.62	6.82	6.53	0.46	6.56	0.30	1.01	0.008	9.2E-01
Mtr.30195.1.S1 at	4.12	4.91	4.11	4.11	4.11	4.11	4.38	0.46	4.11	0.00	0.94	-0.093	3.6E-01
Mtr.32877.1.S1 at	10.11	10.39	11.01	10.94	11.19	10.74	10.51	0.46	10.96	0.22	1.04	0.061	2.0E-01
Mtr.44936.1.S1 at	9.10	8.27	9.04	9.15	9.19	9.21	8.80	0.46	9.18	0.03	1.04	0.061	2.3E-01
Mtr.41912.1.S1_at	9.21	8.36	9.09	9.47	9.26	9.51	8.89	0.46	9.41	0.13	1.06	0.083	1.3E-01
Mtr.30528.1.S1_at	2.88	3.79	3.21	2.80	2.80	2.74	3.29	0.46	2.78	0.04	0.84	-0.244	1.3E-01
Mtr.43455.1.S1 at	11.50	10.70	10.71	11.35	11.09	11.36	10.97	0.46	11.27	0.15	1.03	0.039	3.4E-01
Mtr.50839.1.S1 at	9.79	10.65	10.50	10.10	10.23	10.04	10.32	0.46	10.12	0.09	0.98	-0.027	5.2E-01
Mtr.43384.1.S1 at	12.12	11.25	11.93	12.09	12.01	12.06	11.77	0.46	12.06	0.04	1.02	0.035	3.4E-01
Mtr.740.1.S1 at	2.97	3.72	2.88	2.89	2.84	2.85	3.19	0.46	2.86	0.03	0.90	-0.157	2.8E-01
Mtr.35937.1.S1_at	9.32	8.40	8.94	8.95	8.52	8.94	8.89	0.46	8.80	0.24	0.99	-0.014	7.9E-01
Mtr.35660.1.S1_at	3.03	2.92	3.77	6.71	8.05	7.02	3.24	0.46	7.26	0.70	2.24	1.164	1.1E-03
Mtr.49117.1.S1_at	2.80	3.59	2.79	2.88	2.60	2.78	3.06	0.46	2.76	0.14	0.90	-0.151	3.4E-01
Mtr.12281.1.S1 s at	9.91	9.03	9.72	9.33	9.59	9.58	9.55	0.46	9.50	0.15	0.99	-0.008	8.6E-01
Mtr.2638.1.S1 at	7.13	7.00	7.85	7.77	8.16	7.65	7.33	0.46	7.86	0.27	1.07	0.101	1.6E-01
Mtr.31743.1.S1 s at	12.48	11.66	12.43	12.22	12.52	12.28	12.19	0.46	12.34	0.16	1.01	0.017	6.3E-01
Mtr.48195.1.S1_s_at	10.06	9.15	9.69	9.91	9.84	9.77	9.63	0.46	9.84	0.07	1.02	0.031	4.8E-01
Mtr.20642.1.S1_at	10.39	9.48	9.84	10.12	10.39	9.93	9.91	0.46	10.14	0.23	1.02	0.034	4.7E-01
Mtr.1501.1.S1_s_at	8.78	7.87	8.45	8.32	8.33	8.08	8.37	0.46	8.24	0.14	0.98	-0.023	6.6E-01
Mtr.43978.1.S1 at	8.85	8.38	9.29	8.84	8.85	8.92	8.84	0.46	8.87	0.04	1.00	0.005	9.1E-01
Mtr.17866.1.S1 at	2.95	3.74	2.93	2.90	2.86	3.03	3.21	0.46	2.93	0.09	0.91	-0.131	3.6E-01
Mtr.10807.1.S1 at	7.70	6.86	7.59	7.93	8.18	7.73	7.38	0.46	7.95	0.22	1.08	0.106	1.3E-01
Mtr.50170.1.S1_at	2.63	3.41	2.60	2.57	3.03	2.62	2.88	0.46	2.74	0.25	0.95	-0.071	6.7E-01
Mtr.1950.1.S1_at	9.02	8.11	8.46	9.20	8.77	8.91	8.53	0.46	8.96	0.22	1.05	0.070	2.2E-01
Mtr.15976.1.S1_at	7.82	7.33	8.25	7.79	7.79	7.70	7.80	0.46	7.76	0.05	0.99	-0.008	8.9E-01
Mtr.6877.1.S1 at	2.97	3.77	2.98	2.95	2.96	2.94	3.24	0.46	2.95	0.01	0.91	-0.133	3.4E-01
Mtr.3528.1.S1 at	3.91	4.75	4.01	3.88	3.91	3.90	4.22	0.46	3.89	0.01	0.92	-0.116	2.9E-01
Mtr.27894.1.S1 at	7.46	6.57	7.21	7.46	7.53	7.57	7.08	0.46	7.52	0.06	1.06	0.087	1.7E-01
Mtr.33156.1.S1_at	8.22	7.35	7.53	8.09	7.98	8.18	7.70	0.46	8.09	0.10	1.05	0.070	2.3E-01
Mtr.44222.1.S1_s_at	9.84	9.01	9.76	9.79	9.99	9.74	9.53	0.46	9.84	0.13	1.03	0.045	3.3E-01
Mtr.5580.1.S1_s_at	9.20	8.28	8.71	8.74	8.83	9.22	8.73	0.46	8.93	0.25	1.02	0.032	5.5E-01
Mtr.15518.1.S1 at	7.80	6.95	7.68	7.81	7.32	7.62	7.48	0.46	7.58	0.25	1.01	0.020	7.5E-01
Mtr.38568.1.S1 at	8.82	9.58	9.65	10.79	10.93	10.35	9.35	0.46	10.69	0.30	1.14	0.193	1.3E-02
Mtr.35992.1.S1 at	10.93	11.09	10.23	9.77	9.86	9.83	10.75	0.46	9.82	0.04	0.91	-0.130	2.5E-02
Mtr.25367.1.S1 at	3.16	3.91	3.08	3.33	3.05	3.04	3.38	0.46	3.14	0.17	0.93	-0.107	4.4E-01
Mtr.2662.1.S1_at	10.70	11.42	11.56	11.37	12.02	11.54	11.23	0.46	11.64	0.34	1.04	0.052	2.8E-01
Mtr.2478.1.S1 at	7.24	6.74	7.65	7.59	7.43	7.65	7.21	0.46	7.55	0.11	1.05	0.067	2.8E-01
Mtr.11517.1.S1 at	7.92	7.35	8.25	8.13	7.79	7.71	7.84	0.46	7.88	0.22	1.00	0.007	9.0E-01
Mtr.12816.1.S1 at	11.48	12.38	11.79	11.62	11.95	11.62	11.88	0.46	11.73	0.19	0.99	-0.019	6.2E-01
Mtr.17504.1.S1 at	8.48	7.66	8.42	8.47	8.40	8.49	8.19	0.46	8.45	0.05	1.03	0.046	3.7E-01
Mtr.486.1.S1_at	3.29	4.06	3.24	3.23	3.20	3.18	3.53	0.46	3.20	0.02	0.91	-0.141	2.8E-01
Mtr.47769.1.S1_s_at	5.23	4.42	5.20	6.07	5.58	4.96	4.95	0.46	5.54	0.56	1.12	0.162	2.3E-01
Mtr.12615.1.S1 at	13.20	12.60	12.30	12.46	12.53	12.55	12.70	0.46	12.51	0.05	0.99	-0.021	5.3E-01
Mtr.29242.1.S1 at	6.50	7.02	6.10	6.59	6.27	6.32	6.54	0.46	6.39	0.17	0.98	-0.034	6.2E-01
Mtr.46848.1.S1 at	3.17	3.91	3.06	3.08	2.98	3.04	3.38	0.46	3.03	0.05	0.90	-0.157	2.6E-01
Mtr.7060.1.S1 at	7.86	7.80	8.62	8.36	8.30	8.40	8.10	0.46	8.35	0.05	1.03	0.045	3.9E-01
Mtr.24679.1.S1_at	3.03	3.80	2.99	2.97	2.89	2.95	3.27	0.46	2.93	0.04	0.90	-0.158	2.7E-01
Mtr.17590.1.S1_at	2.99	3.77	2.96	2.94	2.83	2.91	3.24	0.46	2.89	0.06	0.89	-0.164	2.6E-01
Mtr.39331.1.S1_at	7.47	6.75	7.60	7.33	8.00	7.43	7.27	0.46	7.59	0.36	1.04	0.061	4.0E-01
Mtr.8772.1.S1 at	7.92	7.84	8.67	7.99	8.00	7.96	8.14	0.46	7.99	0.02	0.98	-0.028	5.9E-01
Mtr.48055.1.S1 at	2.59	3.21	3.49	2.52	2.55	2.54	3.10	0.46	2.54	0.02	0.82	-0.289	1.0E-01
Mtr.10204.1.S1 at	9.49	8.61	9.28	9.16	9.15	9.06	9.13	0.46	9.12	0.05	1.00	0.000	1.0E+00
Mtr.39635.1.S1_at	10.52	9.61	10.10	10.09	10.10	10.10	10.08	0.46	10.10	0.00	1.00	0.003	9.4E-01
Mtr.11590.1.S1_at	9.37	8.55	9.32	9.56	9.29	9.51	9.08	0.46	9.45	0.15	1.04	0.058	2.5E-01
Mtr.7572.1.S1_at	3.06	2.94	3.79	4.32	3.95	3.77	3.26	0.46	4.02	0.28	1.23	0.300	7.2E-02
Mtr.3260.1.S1 at	7.63	6.81	7.57	7.60	7.38	7.60	7.33	0.46	7.52	0.13	1.03	0.037	5.2E-01
Mtr.42681.1.S1 at	8.06	7.16	7.48	7.52	7.99	7.91	7.57	0.46	7.81	0.25	1.03	0.045	4.7E-01
Mtr.1756.1.S1 at	9.05	8.19	8.89	8.70	8.57	8.65	8.71	0.46	8.64	0.06	0.99	-0.012	8.1E-01
Mtr.15700.1.S1_at	2.17	2.95	2.15	2.15	2.15	2.15	2.42	0.46	2.15	0.00	0.89	-0.173	3.6E-01
Mtr.16686.1.S1_at	7.86	7.27	8.17	7.92	7.77	7.73	7.77	0.46	7.81	0.10	1.00	0.007	9.0E-01
Mtr.7394.1.S1_at	8.50	7.59	8.09	8.28	8.43	8.19	8.06	0.46	8.30	0.12	1.03	0.042	4.4E-01
Mtr.44788.1.S1 at	6.35	7.26	6.92	6.46	6.52	6.42	6.84	0.46	6.47	0.05	0.95	-0.081	2.3E-01
Mtr.36303.1.S1 at	2.84	2.08	2.02	2.02	2.02	2.02	2.31	0.46	2.02	0.00	0.87	-0.195	3.3E-01
Mtr.51571.1.S1 s at	12.52	11.71	11.73	12.08	11.99	12.23	11.99	0.46	12.10	0.13	1.01	0.013	7.1E-01

Mtr.22050.1.S1_at	9.93	9.04	9.64	9.84	9.39	9.83	9.54	0.46	9.69	0.26	1.02	0.023	6.4E-01
Mtr.42855.1.S1_s_at	12.09	12.39	12.99	12.31	12.34	12.15	12.49	0.46	12.27	0.10	0.98	-0.026	4.6E-01
Mtr.2576.1.S1_at	8.35	7.44	8.00	8.60	8.35	8.70	7.93	0.46	8.55	0.18	1.08	0.108	9.5E-02
Mtr.24102.1.S1_at	2.44	3.17	2.33	2.32	2.31	2.32	2.65	0.46	2.32	0.00	0.88	-0.191	2.8E-01
Mtr.49255.1.S1_at	2.73	3.48	2.66	2.62	2.61	2.60	2.95	0.46	2.61	0.01	0.88	-0.178	2.6E-01
Mtr.39643.1.S1_at	10.73	11.63	11.28	10.90	10.89	11.00	11.21	0.46	10.93	0.06	0.97	-0.037	3.5E-01
Mtr.32318.1.S1_s_at	4.41	5.32	4.95	4.49	4.10	4.48	4.89	0.46	4.36	0.22	0.89	-0.167	1.4E-01
Mtr.10502.1.S1_s_at	13.67	13.70	12.89	13.55	13.35	13.63	13.42	0.46	13.51	0.15	1.01	0.010	7.5E-01
Mtr.20464.1.S1_x_at	10.47	9.63	10.38	10.49	10.14	10.59	10.16	0.46	10.41	0.24	1.02	0.035	4.5E-01
Mtr.5720.1.S1_at	7.95	7.14	7.91	8.02	7.83	8.12	7.67	0.46	7.99	0.15	1.04	0.060	3.0E-01
Mtr.44217.1.S1_at	10.80	10.14	11.01	11.00	11.07	10.67	10.65	0.46	10.91	0.21	1.02	0.035	4.2E-01
Mtr.24308.1.S1_x_at	4.03	4.15	3.30	4.11	4.05	3.93	3.83	0.46	4.03	0.09	1.05	0.075	4.9E-01
Mtr.45715.1.S1_at	9.53	9.04	8.62	8.95	9.19	9.34	9.06	0.46	9.16	0.20	1.01	0.015	7.5E-01
Mtr.25275.1.S1_at	2.74	3.53	2.72	2.71	2.69	2.69	3.00	0.46	2.70	0.01	0.90	-0.152	3.2E-01
Mtr.51787.1.S1_at	6.64	7.18	7.54	6.87	7.26	6.95	7.12	0.46	7.03	0.21	0.99	-0.019	7.6E-01
Mtr.43304.1.S1_at	7.71	7.76	8.52	7.49	7.51	7.34	8.00	0.46	7.44	0.09	0.93	-0.103	1.1E-01
Mtr.11450.1.S1_at	6.96	6.39	7.30	6.99	7.37	7.10	6.88	0.46	7.15	0.19	1.04	0.055	4.0E-01
Mtr.32666.1.S1_at	8.85	8.02	8.09	8.41	8.06	8.41	8.32	0.46	8.29	0.20	1.00	-0.005	9.2E-01
Mtr.30709.1.S1_at	7.26	6.42	7.15	7.07	6.90	6.99	6.94	0.46	6.99	0.08	1.01	0.010	8.7E-01
Mtr.13240.1.S1_at	9.21	8.46	9.29	9.16	9.11	9.04	8.98	0.46	9.10	0.06	1.01	0.019	6.8E-01
Mtr.26229.1.S1_at	10.96	10.28	11.15	10.89	10.89	10.90	10.80	0.46	10.89	0.01	1.01	0.013	7.3E-01
Mtr.45713.1.S1_at	5.87	5.09	5.89	5.85	5.85	5.83	5.62	0.46	5.84	0.01	1.04	0.056	4.4E-01
Mtr.6712.1.S1_at	9.78	8.87	9.45	9.54	9.52	9.38	9.36	0.46	9.48	0.09	1.01	0.018	6.9E-01
Mtr.34837.1.S1_at	8.88	7.98	8.52	8.92	8.72	8.87	8.46	0.46	8.84	0.10	1.04	0.063	2.4E-01
Mtr.31117.1.S1_at	3.37	2.47	3.06	3.17	3.21	3.12	2.97	0.46	3.17	0.05	1.07	0.095	4.9E-01
Mtr.20805.1.S1_x_at	3.08	3.84	3.02	3.03	3.01	3.53	3.31	0.46	3.19	0.29	0.96	-0.054	7.2E-01
Mtr.20739.1.S1_s_at	9.68	8.88	8.90	9.36	9.30	9.52	9.15	0.46	9.40	0.12	1.03	0.038	4.2E-01
Mtr.7284.1.S1_at	5.52	5.96	5.04	5.74	5.84	6.10	5.51	0.46	5.89	0.19	1.07	0.097	2.5E-01
Mtr.49687.1.S1_x_at	10.27	9.62	9.39	9.45	9.45	9.46	9.76	0.46	9.45	0.01	0.97	-0.046	3.1E-01
Mtr.14014.1.S1_at	12.40	13.17	13.20	13.25	13.59	13.24	12.92	0.46	13.36	0.20	1.03	0.048	2.0E-01
Mtr.33368.1.S1_at	7.13	6.39	7.22	7.01	6.99	6.98	6.91	0.46	6.99	0.01	1.01	0.017	7.7E-01
Mtr.11671.1.S1_at	9.31	8.52	9.31	9.25	9.31	9.18	9.05	0.46	9.25	0.07	1.02	0.032	4.9E-01
Mtr.11749.1.S1_at	8.14	7.33	8.09	7.98	8.14	8.23	7.85	0.46	8.12	0.13	1.03	0.047	3.9E-01
Mtr.13409.1.S1_at	8.65	9.45	9.43	9.16	9.30	9.07	9.18	0.46	9.18	0.11	1.00	0.000	1.0E+00
Mtr.41677.1.S1_at	9.55	8.92	9.81	9.58	9.59	9.35	9.43	0.46	9.51	0.13	1.01	0.012	7.9E-01
Mtr.9240.1.S1_at	9.38	10.13	10.20	9.68	9.91	9.57	9.90	0.46	9.72	0.18	0.98	-0.027	5.5E-01
Mtr.39010.1.S1_at	8.48	7.70	7.68	7.90	7.72	7.98	7.95	0.46	7.87	0.13	0.99	-0.016	7.7E-01
Mtr.11758.1.S1_at	6.65	7.20	7.56	6.74	6.95	6.67	7.13	0.46	6.79	0.15	0.95	-0.072	2.8E-01
Mtr.52359.1.S1_at	9.75	8.86	9.16	9.21	9.35	9.36	9.26	0.46	9.31	0.08	1.01	0.008	8.6E-01
Mtr.14359.1.S1_s_at	5.14	4.40	4.31	4.63	4.43	3.77	4.62	0.46	4.28	0.45	0.93	-0.109	4.1E-01
Mtr.45291.1.S1_at	12.90	12.01	12.31	12.62	12.56	12.65	12.41	0.46	12.61	0.04	1.02	0.023	4.9E-01
Mtr.33040.1.S1_at	8.98	8.07	8.54	8.51	8.45	8.85	8.53	0.46	8.60	0.21	1.01	0.012	8.2E-01
Mtr.30208.1.S1_at	3.46	4.28	3.51	3.42	3.01	3.39	3.75	0.46	3.27	0.23	0.87	-0.197	1.8E-01
Mtr.30474.1.S1_s_at	7.36	7.82	8.28	7.72	7.62	7.58	7.82	0.46	7.64	0.07	0.98	-0.034	5.3E-01
Mtr.7707.1.S1_at	5.67	4.89	5.70	5.88	5.83	5.71	5.42	0.46	5.81	0.09	1.07	0.100	2.2E-01
Mtr.50544.1.S1_at	2.87	3.63	2.81	2.79	2.75	2.78	3.10	0.46	2.77	0.02	0.89	-0.161	2.8E-01
Mtr.44522.1.S1_at	9.36	8.48	9.12	9.25	9.21	9.45	8.99	0.46	9.31	0.13	1.04	0.050	3.1E-01
Mtr.43886.1.S1_at	10.24	9.33	9.70	9.77	9.54	9.70	9.76	0.46	9.67	0.12	0.99	-0.013	7.7E-01
Mtr.9098.1.S1_at	9.60	8.78	9.55	9.50	9.78	9.58	9.31	0.46	9.62	0.15	1.03	0.047	3.2E-01
Mtr.21314.1.S1_at	4.40	5.15	4.33	4.30	4.19	4.34	4.63	0.46	4.28	0.08	0.92	-0.114	2.6E-01
Mtr.13360.1.S1_at	10.57	9.71	10.42	10.78	10.82	10.79	10.23	0.46	10.79	0.02	1.05	0.077	1.0E-01
Mtr.17093.1.S1_at	5.99	5.26	6.10	5.85	5.95	5.53	5.78	0.46	5.78	0.22	1.00	-0.001	9.9E-01
Mtr.12385.1.S1_at	12.70	11.79	12.22	12.20	12.18	12.18	12.23	0.46	12.19	0.01	1.00	-0.006	8.7E-01
Mtr.50814.1.S1_at	10.79	9.93	10.63	10.56	10.17	10.42	10.45	0.46	10.38	0.20	0.99	-0.009	8.3E-01
Mtr.17495.1.S1_s_at	10.98	10.11	10.31	10.69	10.76	10.96	10.47	0.46	10.80	0.14	1.03	0.046	2.9E-01
Mtr.13024.1.S1_at	5.71	6.15	5.24	5.56	5.72	5.52	5.70	0.46	5.60	0.11	0.98	-0.025	7.3E-01
Mtr.48217.1.S1_at	2.28	2.31	3.09	2.28	2.28	2.28	2.56	0.46	2.28	0.00	0.89	-0.170	3.4E-01
Mtr.7750.1.S1_at	2.51	3.29	2.50	2.49	2.47	2.46	2.77	0.46	2.47	0.01	0.89	-0.161	3.3E-01
Mtr.42326.1.S1_s_at	7.00	6.15	6.85	6.25	6.44	6.24	6.66	0.46	6.31	0.11	0.95	-0.080	2.6E-01
Mtr.39971.1.S1_at	5.45	6.08	5.20	5.42	5.30	5.21	5.58	0.46	5.31	0.11	0.95	-0.072	3.7E-01
Mtr.174.1.S1_at	9.95	10.86	10.33	9.49	9.95	9.76	10.38	0.46	9.73	0.23	0.94	-0.093	9.2E-02
Mtr.6920.1.S1_s_at	7.93	7.27	8.15	7.92	8.09	7.91	7.78	0.46	7.97	0.10	1.02	0.035	5.2E-01
Mtr.592.1.S1_at	7.93	7.02	7.54	7.61	7.81	7.68	7.50	0.46	7.70	0.10	1.03	0.038	5.0E-01
Mtr.38687.1.S1_at	9.97	10.00	10.77	10.57	10.57	10.44	10.25	0.46	10.53	0.08	1.03	0.039	3.6E-01
Mtr.11873.1.S1_at	10.81	9.92	10.54	10.53	10.56	10.59	10.42	0.46	10.56	0.03	1.01	0.019	6.3E-01
Mtr.26442.1.S1_at	2.98	3.72	2.89	2.88	2.86	2.80	3.20	0.46	2.85	0.04	0.89	-0.167	2.6E-01
Mtr.41462.1.S1_at	9.49	8.59	9.14	9.35	9.41	9.40	9.07	0.46	9.39	0.03	1.03	0.049	3.0E-01
Mtr.5180.1.S1_at	7.55	6.66	7.28	7.45	7.21	7.39	7.17	0.46	7.35	0.12	1.03	0.036	5.4E-01
Mtr.23813.1.S1_at	2.70	2.43	3.32	2.69	2.69	2.68	2.82	0.46	2.69	0.00	0.95	-0.069	6.4E-01
Mtr.33660.1.S1_s_at	9.53	8.65	9.28	9.42	9.32	9.34	9.16	0.46	9.36	0.06	1.02	0.032	4.8E-01
Mtr.15273.1.S1_at	2.65	2.80	3.50	2.64	2.64	2.63	2.98	0.46	2.64	0.00	0.88	-0.178	2.6E-01
Mtr.34114.1.S1_s_at	14.37	15.27	14.70	15.00	14.86	14.99	14.78	0.46	14.95	0.08	1.01	0.016	5.6E-01
Mtr.12255.1.S1_at	3.90	3.96	3.14	3.85	4.71	4.33	3.67	0.46	4.30	0.43	1.17	0.229	1.6E-01
Mtr.7057.1.S1_s_at	8.77	7.96	8.74	8.70	8.80	8.80	8.49	0.46	8.77	0.06	1.03	0.047	3.5E-01
Mtr.47281.1.S1_at	8.99	8.09	8.70	8.76	8.79	8.71	8.59	0.46	8.75	0.04	1.02	0.027	5.7E-01
Mtr.30024.1.S1_at	10.17	11.03	10.86	10.55	10.58	10.39	10.69	0.46	10.51	0.10	0.98	-0.024	5.5E-01
Mtr.17848.1.S1_at	2.58	3.36	2.56	2.51	2.48	2.51	2.83	0.46	2.50	0.02	0.88	-0.182	2.7E-01
Mtr.23446.1.S1_at	7.44	6.60	6.71	6.41	6.54	6.36	6.92	0.45	6.43	0.09	0.93	-0.104	1.5E-01
Mtr.38480.1.S1_at	10.16	10.67	11.06	10.61	10.25	10.56	10.63	0.45	10.47	0.20	0.99	-0.021	6.2E-01
Mtr.38122.1.S1_at	11.32	11.15	12.01	11.55	12.33	11.62	11.49	0.45	11.83	0.43	1.03	0.042	4.0E-01
Mtr.4039.1.S1_at	9.14	8.42	9.26	9.10	9.16	9.08	8.94	0.45	9.12	0.04	1.02	0.028	5.5E-01
Mtr.36398.1.S1_at	3.59	4.30	3.45	3.41	3.61	3.39	3.78	0.45	3.47	0.12	0.92	-0.123	3.2E-01
Mtr.30128.1.S1_at	8.51	7.61	8.20	7.97	8.11	8.19	8.11	0.45	8.09	0.11	1.00	-0.003	9.5E-01
Mtr.40798.1.S1_at	9.49	9.33	10.18	9.94	10.02	9.63	9.67	0.45	9.87	0.20	1.02	0.029	5.3E-01
Mtr.42659.1.S1_at	9.04	8.76	9.65	9.55	9.55	9.87	9.15	0.45	9.66	0.19	1.06	0.078	1.5E-01
Mtr.32939.1.S1_at	3.60	2.98	2.71	2.53	2.84	2.83	3.10	0.45	2.73	0.18	0.88	-0.181	2.6E-01
Mtr.5728.1.S1_at	11.40	12.30	11.95	11.27	11.18	11.38	11.89	0.45	11.27	0.10	0.95	-0.076	8.5E-02
Mtr.20310.1.S1_at	2.92	3.67	2.84	2.81	2.76	2.81	3.14	0.45	2.79	0.03	0.89	-0.171	2.5E-01
Mtr.10048.1.S1_at	9.59	8.79	8.81	9.11	9.08	9.09	9.06	0.45	9.09	0.02	1.00	0.005	9.1E-01
Mtr.10954.1.S1_at	10.26	10.49	9.61	9.47	9.93	9.87	10.12	0.45	9.76	0.25	0.96	-0.053	2.9E-01
Mtr.35054.1.S1_x_at	2.05	2.83	2.04	2.04	2.03	2.03	2.31	0.45	2.04	0.00	0.88	-0.182	3.6E-01
Mtr.31306.1.S1_at	7.49	6.74	7.56	7.66	7.90	8.00	7.26	0.45	7.85	0.17	1.08	0.113	1.0E-01
Mtr.19904.1.S1_at	7.67	7.07	7.96	7.82	7.70	7.77	7.56	0.45	7.76	0.06	1.03	0.038	4.9E-01
Mtr.45317.1.S1_at	8.80	8.08	8.92	8.76	8.82	8.67	8.60	0.45	8.75	0.07	1.02	0.024	6.1E-01
Mtr.13501.1.S1_at	8.93	8.31	9.20	8.88	8.89	8.48	8.81	0.45	8.75	0.23	0.99	-0.010	8.5E-01
Mtr.45096.1.S1_at	8.74	7.83	8.33	8.63	8.74	8.69	8.30	0.45	8.69	0.05	1.05	0.066	2.2E-01
Mtr.2464.1.S1_at	6.29	7.20	6.67	6.50	6.59	6.37	6.72	0.45	6.49	0.11	0.97	-0.050	4.4E-01
Mtr.6757.1.S1_at	5.23	4.48	4.41	4.43	4.16	4.43	4.71	0.45	4.34	0.15	0.92	-0.118	2.5E-01
Mtr.21022.1.S1_at	5.49	4.61	4.83	5.42	5.61	5.21	4.98	0.45	5.42	0.20	1.09	0.122	2.0E-01
Mtr.30983.1.S1_at	2.58	3.36	2.57	2.56	2.57	2.53	2.84	0.45	2.55	0.02	0.90	-0.152	3.4E-01
Mtr.5465.1.S1_at	8.18	7.32	8.01	8.16	8.22	8.33	7.84	0.45	8.24	0.09	1.05	0.072	2.1E-01
Mtr.34198.1.S1_s_at	5.87	5.56	6.46	5.78	6.07	6.01	5.96	0.45	5.95	0.15	1.00	-0.003	9.7E-01
Mtr.32964.1.S1_s_at	3.55	2.74	3.50	4.41	4.37	4.30	3.26	0.45	4.36	0.06	1.34	0.419	1.4E-02
Mtr.35595.1.S1_at	5.29	4.38	4.75	4.31	4.84	4.75	4.81	0.45	4.63	0.28	0.96	-0.053	6.0E-01
Mtr.37515.1.S1_at	10.64	11.16	11.55	11.07	10.93	10.70	11.12	0.45	10.90	0.19	0.98	-0.028	5.0E-01
Mtr.2.1.S1_at	2.38	1.83	2.73	2.79	2.36	2.17	2.31	0.45	2.44	0.32	1.06	0.079	7.1E-01
Mtr.30871.1.S1_at	6.16	5.60	6.50	6.24	6.01	6.09	6.09	0.45	6.11	0.12	1.00	0.006	9.3E-01
Mtr.51543.1.S1_s_at	9.90	9.58	9.00	9.46	9.58	9.63	9.49	0.45	9.55	0.09	1.01	0.010	8.2E-01
Mtr.49450.1.S1_at	4.98	4.30	4.12	4.12	4.11	4.11	4.47	0.45	4.11	0.01	0.92	-0.120	2.5E-01
Mtr.11595.1.S1_at	13.01	13.70	12.84	13.15	13.47	13.29	13.19	0.45	13.30	0.16	1.01	0.013	7.0E-01
Mtr.51015.1.S1_at	9.59	9.04	9.94	9.59	9.59	9.35	9.52	0.45	9.51	0.14	1.00	-0.002	9.7E-01
Mtr.27211.1.S1_at	2.59	3.35	2.54	2.54	2.53	2.51	2.83	0.45	2.53	0.01	0.89	-0.163	3.1E-01
Mtr.51345.1.S1_at	3.33	2.43	2.89	2.91	2.33	2.33	2.88	0.45	2.52	0.33	0.88	-0.191	3.3E-01
Mtr.34442.1.S1_at	6.63	5.80	6.53	6.83	6.61	6.66	6.32	0.45	6.70	0.11	1.06	0.085	2.3E-01
Mtr.51646.1.S1_at	12.51	11.76	12.57	12.80	12.82	12.67	12.28	0.45	12.76	0.08	1.04	0.056	1.4E-01
Mtr.21338.1.S1_at	2.26	3.04	2.25	2.25	2.25	2.25	2.52	0.45	2.25	0.00	0.89	-0.165	3.6E-01
Mtr.45218.1.S1_at	6.15	5.33	6.07	5.91	5.66	5.91	5.85	0.45	5.83	0.14	1.00	-0.005	9.4E-01
Mtr.18112.1.S1_at	2.78	3.54	2.74	2.72	2.69	2.70	3.02	0.45	2.70	0.01	0.89	-0.160	2.9E-01
Mtr.38230.1.S1_at	11.02	10.22	10.26	10.55	10.42	10.59	10.50	0.45	10.52	0.09	1.00	0.002	9.5E-01
Mtr.27434.1.S1_at	6.66	5.91	5.84	5.86	6.19	5.88	6.14	0.45	5.98	0.18	0.97	-0.039	5.9E-01
Mtr.7817.1.S1_at	2.11	2.89	2.10	2.10	2.10	2.09	2.37	0.45	2.10	0.00	0.89	-0.174	3.6E-01
Mtr.2174.1.S1_at	8.33	8.03	7.44	8.12	7.83	8.09	7.93	0.45	8.01	0.16	1.01	0.015	7.9E-01
Mtr.23025.1.S1_at	4.98	4.07	4.58	4.53	4.73	4.60	4.54	0.45	4.62	0.10	1.02	0.023	8.0E-01
Mtr.45898.1.S1_at	3.53	2.77	2.73	2.73	2.70	2.73	3.01	0.45	2.72	0.02	0.90	-0.147	3.3E-01
Mtr.10985.1.S1_at	9.60	9.17	8.70	9.05	9.37	9.23	9.16	0.45	9.21	0.16	1.01	0.009	8.5E-01
Mtr.34784.1.S1_s_at	9.97	9.15	9.23	9.68	9.54	9.60	9.45	0.45	9.61	0.07	1.02	0.023	5.9E-01
Mtr.38011.1.S1_s_at	8.81	9.51	9.66	10.01	10.33	10.04	9.32	0.45	10.12	0.18	1.09	0.119	4.6E-02
Mtr.11315.1.S1_at	9.28	8.38	8.83	8.84	9.14	8.87	8.83	0.45	8.95	0.16	1.01	0.019	6.9E-01
Mtr.23860.1.S1_s_at	8.09	7.62	8.52	8.11	8.31	8.12	8.08	0.45	8.18	0.11	1.01	0.019	7.2E-01
Mtr.48702.1.S1_at	6.23	5.51	6.34	5.96	6.14	6.10	6.03	0.45	6.06	0.09	1.01	0.009	8.9E-01
Mtr.41740.1.S1_at	8.62	7.75	8.43	8.64	8.28	8.53	8.27	0.45	8.48	0.18	1.03	0.037	4.9E-01

Mtr.9598.1.S1_at	11.29	10.96	11.86	11.35	11.37	11.35	11.37	0.45	11.36	0.01	1.00	-0.002	9.6E-01
Mtr.20921.1.S1_at	8.81	7.98	8.70	8.88	8.71	8.95	8.50	0.45	8.85	0.12	1.04	0.059	2.6E-01
Mtr.43702.1.S1_s_at	6.49	5.70	6.47	6.10	6.02	6.02	6.22	0.45	6.05	0.05	0.97	-0.041	5.4E-01
Mtr.1621.1.S1_at	4.50	5.24	4.42	4.30	4.34	4.33	4.72	0.45	4.32	0.02	0.91	-0.128	2.0E-01
Mtr.51453.1.S1_at	9.15	8.55	9.44	9.17	9.10	9.06	9.05	0.45	9.11	0.06	1.01	0.010	8.3E-01
Mtr.5457.1.S1_at	7.70	6.85	7.00	7.55	7.47	7.74	7.18	0.45	7.59	0.14	1.06	0.079	2.1E-01
Mtr.33573.1.S1_at	9.74	9.08	8.88	9.73	9.59	9.89	9.23	0.45	9.74	0.15	1.05	0.077	1.4E-01
Mtr.77.1.S1_at	5.64	5.25	6.15	5.46	5.36	5.32	5.68	0.45	5.38	0.07	0.95	-0.077	3.3E-01
Mtr.10543.1.S1_at	10.99	10.44	11.34	11.42	11.58	11.55	10.92	0.45	11.52	0.08	1.05	0.076	8.9E-02
Mtr.13156.1.S1_at	10.28	9.42	10.11	10.21	10.17	10.15	9.94	0.45	10.18	0.03	1.02	0.035	4.1E-01
Mtr.27372.1.S1_at	4.78	5.42	5.66	5.43	5.74	5.40	5.29	0.45	5.52	0.19	1.04	0.063	4.5E-01
Mtr.639.1.S1_at	4.49	4.23	5.11	5.44	4.79	4.93	4.61	0.45	5.05	0.34	1.10	0.132	2.5E-01
Mtr.8920.1.S1_at	8.57	7.99	8.88	8.71	8.54	8.70	8.48	0.45	8.65	0.10	1.02	0.028	5.6E-01
Mtr.33141.1.S1_s_at	7.01	6.43	7.33	7.11	6.88	6.97	6.92	0.45	6.99	0.12	1.01	0.014	8.1E-01
Mtr.10833.1.S1_a_at	7.75	8.65	8.27	8.07	8.43	8.09	8.22	0.45	8.19	0.20	1.00	-0.005	9.2E-01
Mtr.42141.1.S1_at	12.33	13.18	12.47	12.40	11.83	12.46	12.66	0.45	12.23	0.34	0.97	-0.050	2.6E-01
Mtr.35405.1.S1_at	8.16	7.37	8.15	8.08	8.24	7.70	7.90	0.45	8.01	0.28	1.01	0.020	7.3E-01
Mtr.2195.1.S1_at	9.53	8.68	9.37	9.54	9.55	9.38	9.19	0.45	9.49	0.10	1.03	0.046	3.3E-01
Mtr.5684.1.S1_at	8.09	7.19	7.75	7.86	7.93	8.24	7.68	0.45	8.01	0.20	1.04	0.061	3.1E-01
Mtr.13457.1.S1_at	5.38	4.87	5.77	3.98	5.09	4.83	5.34	0.45	4.63	0.58	0.87	-0.205	1.7E-01
Mtr.30520.1.S1_at	2.96	3.27	3.85	2.79	2.71	2.79	3.36	0.45	2.76	0.05	0.82	-0.282	8.5E-02
Mtr.16453.1.S1_at	2.71	3.46	2.66	2.64	2.61	2.65	2.94	0.45	2.63	0.02	0.89	-0.160	3.0E-01
Mtr.35106.1.S1_at	7.03	6.26	7.06	7.12	6.87	6.97	6.79	0.45	6.99	0.13	1.03	0.042	5.0E-01
Mtr.30151.1.S1_at	2.28	3.05	2.26	2.25	2.25	2.24	2.53	0.45	2.25	0.00	0.89	-0.169	3.5E-01
Mtr.31259.1.S1_s_at	13.06	12.28	12.28	12.44	12.80	12.53	12.54	0.45	12.59	0.19	1.00	0.005	8.8E-01
Mtr.19002.1.S1_at	3.11	3.85	3.03	2.99	2.91	2.91	3.33	0.45	2.94	0.05	0.88	-0.179	2.1E-01
Mtr.32827.1.S1_s_at	8.35	7.55	8.31	8.19	8.22	8.42	8.07	0.45	8.28	0.13	1.03	0.036	4.9E-01
Mtr.48941.1.S1_at	2.98	3.73	2.92	3.28	2.84	2.92	3.21	0.45	3.02	0.23	0.94	-0.089	5.5E-01
Mtr.16116.1.S1_at	2.50	3.27	2.48	2.47	2.44	2.46	2.75	0.45	2.46	0.01	0.89	-0.161	3.3E-01
Mtr.28928.1.S1_at	2.02	2.79	2.01	2.00	2.00	2.00	2.27	0.45	2.00	0.00	0.88	-0.183	3.6E-01
Mtr.21539.1.S1_at	9.94	9.09	9.78	9.71	9.69	9.47	9.60	0.45	9.62	0.13	1.00	0.003	9.4E-01
Mtr.22957.1.S1_at	2.41	3.18	2.39	2.39	2.38	2.38	2.66	0.45	2.38	0.00	0.90	-0.158	3.5E-01
Mtr.44447.1.S1_at	8.61	8.04	8.94	8.29	8.28	8.22	8.53	0.45	8.26	0.04	0.97	-0.046	3.6E-01
Mtr.5689.1.S1_at	8.44	7.57	8.19	8.22	8.01	8.34	8.06	0.45	8.19	0.17	1.02	0.022	6.7E-01
Mtr.9861.1.S1_at	7.57	8.15	8.46	7.83	7.74	7.75	8.06	0.45	7.77	0.05	0.96	-0.052	3.4E-01
Mtr.42185.1.S1_at	8.41	7.56	8.24	7.91	7.97	8.08	8.07	0.45	7.99	0.08	0.99	-0.016	7.6E-01
Mtr.20307.1.S1_x_at	2.57	3.34	2.56	2.55	2.55	2.56	2.82	0.45	2.55	0.01	0.90	-0.145	3.6E-01
Mtr.39339.1.S1_at	5.59	4.73	5.40	5.79	5.66	5.71	5.24	0.45	5.72	0.06	1.09	0.126	1.4E-01
Mtr.27805.1.S1_at	5.02	4.51	5.41	4.94	4.94	4.98	4.98	0.45	4.95	0.02	0.99	-0.008	9.2E-01
Mtr.42065.1.S1_at	3.25	3.33	4.07	3.30	3.57	3.36	3.55	0.45	3.41	0.14	0.96	-0.058	6.4E-01
Mtr.44459.1.S1_at	9.37	8.47	8.85	9.02	8.75	8.92	8.90	0.45	8.90	0.14	1.00	0.000	1.0E+00
Mtr.11363.1.S1_at	6.82	7.59	7.60	7.29	7.73	7.04	7.34	0.45	7.35	0.35	1.00	0.003	9.7E-01
Mtr.13914.1.S1_at	9.06	8.28	9.07	8.86	8.80	8.88	8.80	0.45	8.85	0.04	1.01	0.007	8.7E-01
Mtr.41592.1.S1_at	12.68	13.46	12.67	12.62	13.05	12.77	12.94	0.45	12.81	0.22	0.99	-0.014	6.9E-01
Mtr.8474.1.S1_at	9.70	9.30	8.80	9.98	9.98	10.09	9.27	0.45	10.02	0.06	1.08	0.112	4.6E-02
Mtr.730.1.S1_s_at	6.03	5.36	6.21	6.32	6.06	6.08	5.87	0.45	6.15	0.14	1.05	0.069	3.5E-01
Mtr.40441.1.S1_at	12.17	11.37	11.41	11.97	11.94	11.93	11.65	0.45	11.95	0.02	1.03	0.036	3.2E-01
Mtr.30234.1.S1_at	3.08	3.76	2.91	2.91	2.88	2.90	3.25	0.45	2.90	0.01	0.89	-0.165	2.5E-01
Mtr.10494.1.S1_x_at	12.49	11.65	12.34	12.55	12.34	12.69	12.16	0.45	12.53	0.18	1.03	0.043	2.6E-01
Mtr.32989.1.S1_at	3.98	3.10	3.39	3.61	4.49	4.49	3.49	0.45	4.19	0.51	1.20	0.264	1.5E-01
Mtr.6884.1.S1_s_at	9.47	8.60	8.83	9.21	9.32	9.25	8.97	0.45	9.26	0.06	1.03	0.047	3.2E-01
Mtr.42400.1.S1_s_at	8.11	7.44	8.29	8.06	8.08	8.27	7.95	0.45	8.14	0.12	1.02	0.034	5.2E-01
Mtr.21990.1.S1_at	3.07	3.68	2.80	2.79	2.79	2.75	3.18	0.45	2.78	0.02	0.87	-0.194	2.0E-01
Mtr.11361.1.S1_at	10.11	9.23	9.86	9.67	9.54	9.74	9.73	0.45	9.65	0.10	0.99	-0.012	7.7E-01
Mtr.26713.1.S1_s_at	9.53	8.66	9.29	9.58	9.23	9.40	9.16	0.45	9.40	0.17	1.03	0.038	4.3E-01
Mtr.43985.1.S1_at	9.67	9.95	10.55	10.07	9.96	9.91	10.06	0.45	9.98	0.08	0.99	-0.011	7.8E-01
Mtr.38075.1.S1_at	9.72	8.86	9.54	9.83	9.55	9.71	9.37	0.45	9.70	0.14	1.03	0.049	3.0E-01
Mtr.31570.1.S1_at	4.30	3.68	4.56	4.77	5.43	5.42	4.18	0.45	5.21	0.38	1.25	0.317	3.9E-02
Mtr.20028.1.S1_x_at	3.16	3.90	3.08	3.12	3.08	3.08	3.38	0.45	3.09	0.02	0.92	-0.128	3.3E-01
Mtr.25636.1.S1_at	3.01	3.79	3.01	2.96	2.91	3.00	3.27	0.45	2.96	0.05	0.90	-0.145	3.0E-01
Mtr.37451.1.S1_at	8.70	8.04	8.90	8.67	8.91	8.63	8.54	0.45	8.74	0.15	1.02	0.032	5.2E-01
Mtr.6043.1.S1_at	3.77	3.00	3.80	4.02	4.20	3.44	3.52	0.45	3.89	0.39	1.10	0.142	3.5E-01
Mtr.41265.1.S1_at	9.95	9.24	10.08	9.54	9.20	9.42	9.76	0.45	9.39	0.17	0.96	-0.056	2.5E-01
Mtr.24476.1.S1_at	11.36	10.61	11.41	11.60	11.66	11.67	11.12	0.45	11.64	0.04	1.05	0.066	1.2E-01
Mtr.7384.1.S1_s_at	10.82	9.99	10.69	10.56	10.75	10.71	10.50	0.45	10.67	0.10	1.02	0.024	5.5E-01
Mtr.13877.1.S1_at	9.00	8.10	8.52	9.21	8.76	9.04	8.54	0.45	9.00	0.23	1.05	0.076	1.9E-01
Mtr.50863.1.S1_s_at	8.48	7.75	8.56	8.39	8.39	8.28	8.26	0.45	8.35	0.06	1.01	0.015	7.5E-01
Mtr.45637.1.S1_s_at	9.18	9.95	9.96	9.66	10.17	9.65	9.70	0.45	9.83	0.30	1.01	0.019	7.1E-01
Mtr.44608.1.S1_at	8.64	9.10	9.54	9.00	9.49	8.95	9.09	0.45	9.15	0.30	1.01	0.008	8.7E-01
Mtr.39431.1.S1_at	11.52	10.64	11.24	11.47	11.17	11.55	11.13	0.45	11.40	0.20	1.02	0.033	4.1E-01
Mtr.14198.1.S1_s_at	2.51	3.27	2.48	2.49	2.49	2.48	2.75	0.45	2.48	0.00	0.90	-0.148	3.6E-01
Mtr.30985.1.S1_at	2.58	3.35	2.56	2.57	2.56	2.51	2.83	0.45	2.55	0.03	0.90	-0.152	3.4E-01
Mtr.40587.1.S1_at	11.00	10.14	10.79	10.72	10.64	10.75	10.65	0.45	10.71	0.06	1.01	0.008	8.2E-01
Mtr.13770.1.S1_at	6.49	5.72	6.50	6.63	6.48	6.62	6.24	0.45	6.58	0.08	1.05	0.076	2.7E-01
Mtr.27257.1.S1_at	5.03	4.20	4.92	4.91	4.89	5.04	4.72	0.45	4.94	0.08	1.05	0.068	4.4E-01
Mtr.22266.1.S1_at	4.15	4.93	4.14	4.14	4.14	4.17	4.41	0.45	4.15	0.02	0.94	-0.087	3.7E-01
Mtr.13765.1.S1_at	11.34	10.68	10.48	10.60	10.88	10.68	10.84	0.45	10.72	0.14	0.99	-0.016	6.9E-01
Mtr.37235.1.S1_at	2.85	3.60	2.80	2.78	2.74	2.73	3.08	0.45	2.75	0.03	0.89	-0.164	2.7E-01
Mtr.7621.1.S1_at	2.62	3.38	2.60	2.59	2.54	2.58	2.87	0.45	2.57	0.03	0.90	-0.158	3.2E-01
Mtr.9665.1.S1_at	8.18	7.81	8.70	8.48	8.53	8.59	8.23	0.45	8.53	0.06	1.04	0.052	3.1E-01
Mtr.38287.1.S1_at	2.59	3.35	2.56	2.71	2.53	2.52	2.83	0.45	2.59	0.11	0.91	-0.129	4.2E-01
Mtr.12899.1.S1_s_at	7.86	7.29	8.17	8.08	8.07	8.35	7.77	0.45	8.17	0.16	1.05	0.071	2.3E-01
Mtr.17043.1.S1_at	6.91	7.08	7.76	6.49	6.66	7.21	7.25	0.45	6.79	0.38	0.94	-0.095	2.5E-01
Mtr.21313.1.S1_at	2.56	2.61	3.36	2.79	2.75	2.74	2.84	0.45	2.76	0.03	0.97	-0.041	7.7E-01
Mtr.51612.1.S1_at	3.02	3.79	3.00	3.02	2.91	2.97	3.27	0.45	2.97	0.06	0.91	-0.139	3.1E-01
Mtr.48649.1.S1_at	12.03	12.87	12.17	11.49	11.84	11.49	12.36	0.45	11.61	0.20	0.94	-0.091	5.7E-02
Mtr.6708.1.S1_at	8.88	8.02	8.66	8.58	8.77	8.63	8.52	0.45	8.66	0.10	1.02	0.024	6.2E-01
Mtr.14845.1.S1_s_at	7.92	8.13	8.78	8.18	8.28	8.17	8.28	0.45	8.21	0.06	0.99	-0.012	8.1E-01
Mtr.28849.1.S1_at	9.37	8.49	9.09	9.07	9.19	8.99	8.98	0.45	9.09	0.10	1.01	0.017	7.1E-01
Mtr.34748.1.S1_at	3.85	2.96	3.50	2.83	3.18	3.15	3.44	0.45	3.05	0.20	0.89	-0.171	2.5E-01
Mtr.10905.1.S1_at	10.19	10.82	11.06	10.25	9.92	10.42	10.69	0.45	10.20	0.25	0.95	-0.068	1.7E-01
Mtr.39885.1.S1_at	7.24	6.44	7.18	6.96	7.11	7.18	6.95	0.45	7.09	0.11	1.02	0.027	6.5E-01
Mtr.13643.1.S1_at	8.60	7.87	8.68	7.88	8.28	8.00	8.38	0.45	8.05	0.20	0.96	-0.058	3.1E-01
Mtr.40167.1.S1_s_at	13.43	12.54	12.99	13.05	12.87	13.09	12.98	0.45	13.00	0.11	1.00	0.002	9.5E-01
Mtr.697.1.S1_s_at	2.56	3.32	2.52	2.51	2.49	2.53	2.80	0.45	2.51	0.02	0.90	-0.157	3.3E-01
Mtr.14874.1.S1_at	3.17	3.94	3.16	3.13	3.08	3.07	3.42	0.45	3.09	0.03	0.90	-0.147	2.7E-01
Mtr.4271.1.S1_at	5.00	4.20	4.95	5.06	5.57	4.97	4.72	0.45	5.20	0.32	1.10	0.140	2.1E-01
Mtr.26979.1.S1_at	8.80	7.93	8.55	8.66	8.56	8.51	8.43	0.45	8.58	0.08	1.02	0.026	6.0E-01
Mtr.18470.1.S1_at	8.37	7.68	8.53	8.74	8.67	8.50	8.19	0.45	8.64	0.12	1.05	0.076	1.7E-01
Mtr.18053.1.S1_at	9.12	8.49	9.35	9.21	9.40	9.28	8.99	0.45	9.30	0.10	1.03	0.049	3.1E-01
Mtr.43958.1.S1_at	10.76	9.88	10.50	10.72	10.70	10.81	10.38	0.45	10.74	0.06	1.03	0.050	2.4E-01
Mtr.43423.1.S1_at	9.77	8.88	9.39	9.54	9.20	9.50	9.35	0.45	9.41	0.19	1.01	0.010	8.2E-01
Mtr.42127.1.S1_at	2.27	2.12	2.96	2.27	2.50	2.27	2.45	0.45	2.35	0.14	0.96	-0.061	7.3E-01
Mtr.35114.1.S1_at	9.21	8.43	8.45	9.00	9.00	8.99	8.70	0.45	9.00	0.01	1.03	0.049	3.1E-01
Mtr.46406.1.S1_at	8.14	7.83	8.71	7.86	8.09	7.79	8.23	0.45	7.91	0.16	0.96	-0.056	3.2E-01
Mtr.9234.1.S1_at	8.45	7.85	8.73	8.50	8.73	8.56	8.34	0.45	8.60	0.12	1.03	0.043	4.0E-01
Mtr.39499.1.S1_at	5.53	4.74	4.78	4.60	4.61	4.68	5.02	0.45	4.63	0.04	0.92	-0.115	2.1E-01
Mtr.28644.1.S1_at	4.70	3.89	4.63	4.25	4.05	3.84	4.41	0.45	4.05	0.20	0.92	-0.124	2.7E-01
Mtr.5568.1.S1_at	4.02	4.49	3.60	4.54	4.05	4.09	4.04	0.45	4.22	0.27	1.05	0.065	5.7E-01
Mtr.9624.1.S1_at	5.56	4.99	5.87	5.55	5.55	5.55	5.47	0.45	5.55	0.00	1.01	0.020	7.8E-01
Mtr.47279.1.S1_at	4.63	5.38	4.58	4.57	4.11	4.50	4.86	0.45	4.40	0.25	0.90	-0.145	1.9E-01
Mtr.44285.1.S1_at	8.31	7.59	8.42	8.41	8.38	8.34	8.11	0.45	8.38	0.04	1.03	0.047	3.6E-01
Mtr.8868.1.S1_at	6.95	6.17	6.93	6.74	6.80	6.66	6.68	0.45	6.73	0.07	1.01	0.011	8.5E-01
Mtr.6154.1.S1_s_at	10.06	9.50	9.18	9.42	9.40	9.46	9.58	0.45	9.43	0.03	0.98	-0.023	5.9E-01
Mtr.36385.1.S1_at	8.23	8.31	9.04	8.40	9.16	8.55	8.53	0.45	8.70	0.40	1.02	0.029	6.4E-01
Mtr.31154.1.S1_at	8.51	8.29	9.15	8.73	8.86	8.74	8.65	0.45	8.77	0.07	1.01	0.020	6.6E-01
Mtr.42064.1.S1_at	7.17	6.64	6.28	6.98	6.74	6.58	6.70	0.45	6.77	0.20	1.01	0.015	8.2E-01
Mtr.18116.1.S1_at	8.23	7.95	8.83	8.01	8.36	8.03	8.34	0.45	8.13	0.20	0.98	-0.035	5.1E-01
Mtr.33359.1.S1_at	3.24	2.69	3.58	4.77	5.54	4.48	3.17	0.45	4.93	0.54	1.55	0.637	1.2E-02
Mtr.51785.1.S1_at	9.42	9.69	10.29	10.17	10.16	9.96	9.80	0.45	10.09	0.12	1.03	0.042	3.4E-01
Mtr.26459.1.S1_at	10.11	10.81	10.94	11.96	12.50	12.23	10.62	0.45	12.23	0.27	1.15	0.204	5.9E-03
Mtr.28442.1.S1_at	3.97	3.08	3.43	3.24	3.64	3.21	3.50	0.45	3.36	0.24	0.96	-0.055	6.8E-01
Mtr.46212.1.S1_at	2.49	3.25	2.47	2.47	2.46	2.46	2.74	0.45	2.46	0.01	0.90	-0.152	3.5E-01
Mtr.49684.1.S1_at	3.33	4.09	3.31	3.32	3.28	3.32	3.58	0.45	3.31	0.02	0.93	-0.112	3.6E-01
Mtr.42202.1.S1_at	6.12	6.73	6.99	8.32	8.52	7.88	6.61	0.45	8.24	0.33	1.25	0.317	7.2E-03
Mtr.14147.1.S1_s_at	8.31	9.16	8.50	8.70	9.14	8.95	8.65	0.45	8.93	0.22	1.03	0.045	4.0E-01
Mtr.13200.1.S1_at	9.65	10.38	9.57	9.58	9.79	9.62	9.87	0.45	9.66	0.11	0.98	-0.030	4.9E-01
Mtr.44571.1.S1_at	8.89	8.05	8.74	8.77	8.78	8.66	8.56	0.45	8.74	0.06	1.02	0.029	5.4E-01
Mtr.34451.1.S1_at	7.75	7.10	7.95	7.96	7.66	7.91	7.60	0.45	7.85	0.16	1.03	0.046	4.2E-01
Mtr.13983.1.S1_at	10.55	10.52	11.31	11.54	10.90	11.13	10.79	0.45	11.19	0.32	1.04	0.053	2.8E-01
Mtr.2549.1.S1_at	5.77	4.88	5.40	5.58	5.40	5.61	5.35	0.45	5.53	0.11	1.03	0.049	5.3E-01

Mtr.37572.1.S1_at	7.91	7.43	8.33	7.92	8.21	7.81	7.89	0.45	7.98	0.21	1.01	0.016	7.7E-01
Mtr.52116.1.S1_at	10.58	10.41	11.25	10.73	10.40	10.45	10.75	0.45	10.53	0.18	0.98	-0.030	4.7E-01
Mtr.43640.1.S1_at	7.98	7.45	8.33	8.39	8.76	8.44	7.92	0.45	8.53	0.20	1.08	0.107	9.7E-02
Mtr.45112.1.S1_at	4.73	4.06	4.90	5.33	5.89	5.07	4.56	0.45	5.43	0.42	1.19	0.251	7.0E-02
Mtr.9089.1.S1_at	9.64	8.84	9.58	9.42	9.50	9.38	9.35	0.45	9.43	0.06	1.01	0.013	7.7E-01
Mtr.39158.1.S1_at	7.81	8.56	8.61	9.65	9.88	9.61	8.33	0.45	9.71	0.15	1.17	0.222	7.0E-03
Mtr.25889.1.S1_at	7.69	6.84	7.01	7.83	8.34	7.83	7.18	0.45	8.00	0.30	1.11	0.156	5.7E-02
Mtr.8298.1.S1_at	10.62	11.20	11.50	11.29	12.09	11.03	11.11	0.45	11.47	0.55	1.03	0.046	4.2E-01
Mtr.20174.1.S1_at	11.89	11.19	12.02	11.93	11.87	11.91	11.70	0.45	11.90	0.03	1.02	0.025	4.8E-01
Mtr.32967.1.S1_at	4.72	4.12	4.99	4.75	4.71	4.64	4.61	0.45	4.70	0.05	1.02	0.029	7.4E-01
Mtr.15765.1.S1_at	10.85	10.24	9.98	10.24	10.08	10.28	10.36	0.45	10.20	0.10	0.99	-0.022	5.9E-01
Mtr.43918.1.S1_at	12.31	12.44	11.61	11.74	11.41	11.73	12.12	0.45	11.63	0.19	0.96	-0.060	1.5E-01
Mtr.41052.1.S1_at	10.14	9.36	10.12	10.16	9.76	10.07	9.88	0.45	10.00	0.21	1.01	0.017	7.0E-01
Mtr.20298.1.S1_at	11.01	10.20	10.29	10.43	10.28	10.49	10.50	0.45	10.40	0.11	0.99	-0.014	7.2E-01
Mtr.3750.1.S1_at	2.72	1.95	2.72	2.71	2.71	2.73	2.46	0.45	2.72	0.01	1.10	0.143	3.7E-01
Mtr.6987.1.S1_at	8.52	8.11	9.01	8.95	8.71	8.79	8.55	0.45	8.82	0.12	1.03	0.045	3.7E-01
Mtr.50077.1.S1_at	2.48	3.25	2.48	2.47	2.47	2.46	2.74	0.45	2.46	0.00	0.90	-0.151	3.5E-01
Mtr.26653.1.S1_s_at	9.40	8.69	9.52	9.38	9.42	9.24	9.20	0.45	9.35	0.09	1.02	0.022	6.2E-01
Mtr.9235.1.S1_s_at	8.54	7.75	8.50	8.63	8.54	8.51	8.26	0.45	8.56	0.06	1.04	0.051	3.1E-01
Mtr.9204.1.S1_at	10.36	10.51	11.19	10.71	10.74	10.62	10.69	0.45	10.69	0.06	1.00	0.000	1.0E+00
Mtr.45115.1.S1_at	4.37	4.68	5.25	5.42	5.30	4.85	4.77	0.45	5.19	0.30	1.09	0.122	2.5E-01
Mtr.25876.1.S1_s_at	4.03	3.83	4.68	3.89	4.76	4.11	4.18	0.45	4.26	0.46	1.02	0.025	8.5E-01
Mtr.52339.1.S1_x_at	2.72	3.48	2.69	2.66	2.63	2.66	2.96	0.45	2.65	0.02	0.89	-0.161	2.9E-01
Mtr.22688.1.S1_at	8.56	7.87	8.70	8.19	8.18	8.05	8.38	0.45	8.14	0.08	0.97	-0.042	4.1E-01
Mtr.712.1.S1_at	8.00	7.45	8.33	7.80	8.03	7.88	7.93	0.45	7.90	0.11	1.00	-0.004	9.3E-01
Mtr.3439.1.S1_at	7.49	6.60	6.97	7.12	7.10	7.21	7.02	0.45	7.15	0.06	1.02	0.026	6.5E-01
Mtr.9655.1.S1_at	9.67	8.90	9.67	9.77	9.59	9.56	9.41	0.45	9.64	0.12	1.02	0.035	4.4E-01
Mtr.3046.1.S1_at	9.05	8.42	9.28	9.04	9.13	8.96	8.92	0.45	9.04	0.09	1.01	0.020	6.6E-01
Mtr.13729.1.S1_at	8.96	8.20	8.98	9.00	8.91	9.04	8.71	0.45	8.99	0.07	1.03	0.044	3.6E-01
Mtr.7688.1.S1_at	2.54	3.30	2.51	2.51	2.51	2.51	2.78	0.45	2.51	0.00	0.90	-0.149	3.5E-01
Mtr.27238.1.S1_at	8.03	7.28	8.07	7.98	8.13	7.81	7.79	0.45	7.97	0.16	1.02	0.033	5.5E-01
Mtr.9303.1.S1_at	8.74	7.87	8.49	8.70	8.64	8.49	8.37	0.45	8.61	0.11	1.03	0.041	4.1E-01
Mtr.17885.1.S1_x_at	2.69	3.45	2.66	2.63	2.67	2.62	2.93	0.45	2.64	0.03	0.90	-0.150	3.2E-01
Mtr.18080.1.S1_at	3.36	4.12	3.33	3.31	3.26	3.67	3.60	0.45	3.41	0.22	0.95	-0.079	5.4E-01
Mtr.5486.1.S1_at	10.03	9.14	9.53	8.87	8.66	8.91	9.57	0.45	8.81	0.14	0.92	-0.119	4.8E-02
Mtr.39917.1.S1_at	7.96	7.51	8.40	8.42	8.80	8.27	7.95	0.45	8.50	0.27	1.07	0.096	1.4E-01
Mtr.23519.1.S1_at	5.11	5.41	5.99	5.11	5.43	5.11	5.50	0.45	5.22	0.18	0.95	-0.077	3.6E-01
Mtr.2709.1.S1_at	11.03	10.17	10.40	10.83	10.89	11.03	10.53	0.45	10.92	0.10	1.04	0.052	2.2E-01
Mtr.35924.1.S1_at	8.47	7.64	8.34	8.26	8.49	8.22	8.15	0.45	8.33	0.14	1.02	0.031	5.5E-01
Mtr.1875.1.S1_s_at	8.17	7.42	8.21	7.99	8.08	8.13	7.93	0.45	8.07	0.07	1.02	0.025	6.2E-01
Mtr.41383.1.S1_at	8.34	7.86	8.75	8.40	8.36	8.28	8.31	0.45	8.34	0.06	1.00	0.005	9.1E-01
Mtr.4763.1.S1_at	9.11	9.36	9.97	10.65	10.88	10.71	9.48	0.45	10.75	0.12	1.13	0.181	8.8E-03
Mtr.20603.1.S1_at	8.82	8.46	9.35	8.89	8.78	8.87	8.88	0.45	8.85	0.06	1.00	-0.005	9.1E-01
Mtr.38856.1.S1_at	8.85	8.39	9.28	8.96	8.92	8.99	8.84	0.45	8.96	0.04	1.01	0.019	6.8E-01
Mtr.45358.1.S1_at	7.12	7.76	7.98	7.37	7.31	7.29	7.62	0.45	7.32	0.04	0.96	-0.057	3.2E-01
Mtr.4362.1.S1_at	9.85	8.97	9.30	9.77	9.47	9.66	9.37	0.45	9.63	0.15	1.03	0.040	3.9E-01
Mtr.11817.1.S1_at	2.95	3.00	3.75	3.70	4.03	3.69	3.23	0.45	3.81	0.19	1.18	0.237	1.1E-01
Mtr.29174.1.S1_at	10.47	11.01	11.36	10.96	11.12	10.82	10.95	0.45	10.97	0.15	1.00	0.003	9.4E-01
Mtr.23198.1.S1_x_at	3.08	3.86	3.09	3.05	3.06	3.05	3.34	0.45	3.06	0.01	0.91	-0.130	3.3E-01
Mtr.17636.1.S1_at	4.75	4.00	3.96	4.14	3.79	4.76	4.24	0.45	4.23	0.49	1.00	-0.002	9.9E-01
Mtr.14061.1.S1_s_at	9.46	10.22	10.24	10.14	10.27	9.99	9.97	0.45	10.13	0.14	1.02	0.023	5.9E-01
Mtr.46304.1.S1_at	3.92	3.03	3.52	3.51	3.89	3.33	3.49	0.45	3.58	0.28	1.02	0.035	7.9E-01
Mtr.18884.1.S1_s_at	9.16	8.33	9.02	8.90	9.00	8.84	8.84	0.45	8.91	0.08	1.01	0.012	8.0E-01
Mtr.38999.1.S1_at	10.62	9.89	10.69	10.74	10.52	10.54	10.40	0.45	10.60	0.12	1.02	0.027	5.0E-01
Mtr.8773.1.S1_s_at	6.65	6.31	7.19	6.63	6.93	6.55	6.72	0.45	6.70	0.20	1.00	-0.003	9.7E-01
Mtr.27202.1.S1_at	2.64	2.72	3.45	2.90	3.72	2.27	2.93	0.45	2.96	0.73	1.01	0.013	9.6E-01
Mtr.6800.1.S1_at	6.50	5.83	6.68	6.46	6.41	6.46	6.34	0.45	6.44	0.03	1.02	0.024	7.0E-01
Mtr.45912.1.S1_at	11.22	11.43	10.58	11.40	11.56	11.58	11.08	0.45	11.51	0.10	1.04	0.056	1.7E-01
Mtr.51358.1.S1_at	6.79	6.87	7.60	6.59	6.98	6.61	7.09	0.45	6.73	0.22	0.95	-0.075	2.8E-01
Mtr.3841.1.S1_at	4.47	4.72	5.34	5.03	5.03	5.27	4.84	0.45	5.11	0.14	1.06	0.078	3.7E-01
Mtr.42954.1.S1_at	7.93	7.06	7.66	7.76	7.66	7.85	7.55	0.45	7.76	0.09	1.03	0.039	4.7E-01
Mtr.26083.1.S1_x_at	5.17	5.55	6.06	5.55	5.81	5.39	5.59	0.45	5.58	0.21	1.00	-0.003	9.7E-01
Mtr.26072.1.S1_s_at	9.16	10.05	9.68	9.53	9.48	9.39	9.63	0.45	9.47	0.07	0.98	-0.025	5.6E-01
Mtr.26016.1.S1_at	2.85	3.60	2.81	2.83	2.77	2.77	3.08	0.45	2.79	0.03	0.90	-0.144	3.2E-01
Mtr.15964.1.S1_s_at	2.64	3.41	2.65	2.62	2.61	2.62	2.90	0.44	2.62	0.00	0.90	-0.146	3.4E-01
Mtr.2994.1.S1_s_at	13.84	13.07	13.83	14.02	14.04	14.12	13.58	0.44	14.06	0.05	1.04	0.050	1.4E-01
Mtr.23280.1.S1_at	13.17	13.79	14.03	13.30	13.32	13.47	13.67	0.44	13.36	0.10	0.98	-0.032	3.2E-01
Mtr.25976.1.S1_s_at	9.55	8.76	9.51	9.34	9.26	9.50	9.28	0.44	9.37	0.12	1.01	0.014	7.5E-01
Mtr.13689.1.S1_at	8.32	7.68	8.53	8.52	8.36	8.65	8.18	0.44	8.51	0.15	1.04	0.057	2.9E-01
Mtr.1844.1.S1_s_at	8.69	7.81	8.14	8.53	8.47	8.41	8.21	0.44	8.47	0.06	1.03	0.045	3.8E-01
Mtr.47566.1.S1_x_at	3.18	3.93	3.15	3.16	3.15	3.19	3.42	0.44	3.17	0.02	0.93	-0.111	3.8E-01
Mtr.39197.1.S1_at	9.36	10.01	10.21	10.20	10.13	9.88	9.86	0.44	10.07	0.17	1.02	0.031	4.8E-01
Mtr.38545.1.S1_at	12.01	11.41	12.28	12.03	12.32	11.88	11.90	0.44	12.08	0.22	1.01	0.021	5.7E-01
Mtr.14613.1.S1_at	10.23	9.44	10.19	10.25	10.27	10.04	9.95	0.44	10.19	0.13	1.02	0.034	4.3E-01
Mtr.13835.1.S1_at	7.40	6.65	7.44	6.82	6.76	7.08	7.16	0.44	6.89	0.17	0.96	-0.056	3.7E-01
Mtr.47843.1.S1_at	8.89	8.45	9.34	9.43	9.58	9.24	8.90	0.44	9.42	0.17	1.06	0.082	1.3E-01
Mtr.8271.1.S1_at	6.07	5.57	5.18	4.90	5.54	5.73	5.61	0.44	5.39	0.43	0.96	-0.056	5.8E-01
Mtr.25602.1.S1_x_at	3.20	3.96	3.18	3.14	3.18	3.13	3.45	0.44	3.15	0.03	0.91	-0.129	3.2E-01
Mtr.35385.1.S1_at	2.98	3.74	2.96	2.93	2.90	2.89	3.23	0.44	2.90	0.02	0.90	-0.152	2.8E-01
Mtr.41337.1.S1_at	10.71	9.83	10.16	10.26	10.31	10.28	10.23	0.44	10.28	0.02	1.00	0.007	8.6E-01
Mtr.39964.1.S1_s_at	3.29	2.52	3.28	3.62	3.97	3.26	3.03	0.44	3.62	0.36	1.19	0.256	1.5E-01
Mtr.48282.1.S1_at	2.64	3.40	2.62	2.62	2.62	2.61	2.89	0.44	2.61	0.00	0.91	-0.142	3.5E-01
Mtr.31497.1.S1_at	9.13	8.35	9.11	8.84	8.94	9.01	8.86	0.44	8.93	0.09	1.01	0.011	8.1E-01
Mtr.18928.1.S1_at	10.37	9.57	10.30	10.25	10.45	10.33	10.08	0.44	10.34	0.10	1.03	0.037	3.7E-01
Mtr.32707.1.S1_at	6.24	6.54	7.12	6.58	6.17	6.04	6.63	0.44	6.27	0.28	0.94	-0.082	2.9E-01
Mtr.45635.1.S1_s_at	12.25	12.05	12.90	12.58	12.61	12.39	12.40	0.44	12.52	0.12	1.01	0.014	6.7E-01
Mtr.38983.1.S1_at	11.25	10.45	10.51	11.00	10.89	11.11	10.74	0.44	11.00	0.11	1.02	0.035	3.8E-01
Mtr.43363.1.S1_at	10.01	10.79	10.76	12.39	12.99	12.57	10.52	0.44	12.65	0.30	1.20	0.266	2.4E-03
Mtr.25494.1.S1_at	2.63	2.62	1.85	2.51	2.51	2.51	2.37	0.44	2.51	0.00	1.06	0.086	6.0E-01
Mtr.49832.1.S1_x_at	2.71	3.47	2.70	2.69	2.95	2.69	2.96	0.44	2.78	0.15	0.94	-0.092	5.4E-01
Mtr.15607.1.S1_x_at	3.36	4.03	3.19	3.31	3.16	3.30	3.53	0.44	3.26	0.09	0.92	-0.115	3.6E-01
Mtr.7637.1.S1_at	11.72	10.83	11.28	11.38	11.31	11.42	11.28	0.44	11.37	0.05	1.01	0.012	7.3E-01
Mtr.50405.1.S1_x_at	2.47	3.23	2.45	2.44	2.38	2.44	2.71	0.44	2.42	0.03	0.89	-0.165	3.2E-01
Mtr.13175.1.S1_at	9.33	8.55	9.31	9.30	9.17	9.05	9.06	0.44	9.18	0.13	1.01	0.018	6.9E-01
Mtr.39337.1.S1_at	8.90	8.60	9.48	9.18	9.02	9.04	8.99	0.44	9.08	0.09	1.01	0.014	7.6E-01
Mtr.9956.1.S1_at	8.51	7.62	8.08	8.42	8.27	8.23	8.07	0.44	8.31	0.10	1.03	0.042	4.1E-01
Mtr.33230.1.S1_at	2.59	3.35	2.57	2.56	2.56	2.55	2.84	0.44	2.56	0.01	0.90	-0.149	3.4E-01
Mtr.36726.1.S1_at	1.95	2.72	1.94	1.94	1.94	1.94	2.20	0.44	1.94	0.00	0.88	-0.185	3.6E-01
Mtr.37731.1.S1_at	8.60	8.52	9.33	8.64	8.52	7.98	8.82	0.44	8.38	0.35	0.95	-0.073	2.6E-01
Mtr.6933.1.S1_at	9.37	8.86	8.48	8.86	8.65	8.98	8.90	0.44	8.83	0.17	0.99	-0.012	8.0E-01
Mtr.904.1.S1_s_at	8.26	7.45	8.16	8.13	7.90	7.96	7.96	0.44	8.00	0.12	1.01	0.007	8.9E-01
Mtr.15675.1.S1_x_at	7.90	7.07	7.74	7.74	7.80	7.92	7.57	0.44	7.82	0.09	1.03	0.047	3.9E-01
Mtr.49412.1.S1_at	9.60	10.45	10.25	9.66	9.95	9.54	10.10	0.44	9.72	0.21	0.96	-0.056	2.5E-01
Mtr.41725.1.S1_at	7.98	7.14	7.33	8.11	7.60	7.67	7.48	0.44	7.79	0.28	1.04	0.058	3.7E-01
Mtr.35656.1.S1_at	9.48	8.60	9.11	9.07	8.97	9.04	9.06	0.44	9.02	0.05	1.00	-0.006	8.9E-01
Mtr.43908.1.S1_at	12.97	12.53	12.08	12.60	12.34	12.58	12.53	0.44	12.51	0.14	1.00	-0.002	9.5E-01
Mtr.575.1.S1_at	9.51	10.37	10.15	9.74	10.11	9.78	10.01	0.44	9.88	0.21	0.99	-0.020	6.6E-01
Mtr.28992.1.S1_at	2.07	2.83	2.06	2.06	2.05	2.84	2.32	0.44	2.32	0.45	1.00	-0.003	9.9E-01
Mtr.38066.1.S1_at	8.74	8.37	9.25	8.72	8.85	8.67	8.78	0.44	8.75	0.09	1.00	-0.006	9.0E-01
Mtr.20893.1.S1_s_at	9.98	10.10	10.80	10.63	10.59	10.52	10.29	0.44	10.58	0.06	1.03	0.040	3.3E-01
Mtr.2469.1.S1_at	8.27	8.00	8.87	8.41	8.49	8.41	8.38	0.44	8.44	0.05	1.01	0.009	8.4E-01
Mtr.22999.1.S1_at	9.58	10.02	10.47	10.07	10.16	10.15	10.02	0.44	10.13	0.05	1.01	0.015	7.0E-01
Mtr.10338.1.S1_s_at	10.84	9.97	10.25	10.30	10.30	10.61	10.35	0.44	10.40	0.18	1.00	0.007	8.7E-01
Mtr.9916.1.S1_at	9.72	8.94	9.69	9.70	9.71	9.65	9.45	0.44	9.69	0.03	1.03	0.036	4.1E-01
Mtr.17765.1.S1_at	6.53	5.65	6.15	5.58	5.54	5.57	6.11	0.44	5.56	0.02	0.91	-0.135	1.0E-01
Mtr.38634.1.S1_at	8.90	8.01	8.42	8.66	8.70	8.84	8.44	0.44	8.73	0.09	1.03	0.049	3.3E-01
Mtr.16268.1.S1_at	2.45	3.23	2.47	2.43	2.43	2.43	2.72	0.44	2.43	0.00	0.89	-0.160	3.3E-01
Mtr.45865.1.S1_at	8.53	8.72	9.37	8.67	8.69	8.82	8.87	0.44	8.73	0.08	0.98	-0.024	6.0E-01
Mtr.11536.1.S1_at	11.42	10.55	10.87	11.30	11.38	11.36	10.95	0.44	11.35	0.04	1.04	0.052	1.9E-01
Mtr.48502.1.S1_at	2.54	3.30	2.52	2.47	2.43	2.52	2.78	0.44	2.47	0.05	0.89	-0.171	2.9E-01
Mtr.9702.1.S1_at	11.51	10.68	10.84	11.23	11.09	11.32	11.01	0.44	11.21	0.12	1.02	0.027	4.8E-01
Mtr.38478.1.S1_at	10.32	9.57	9.54	9.96	9.54	10.00	9.81	0.44	9.83	0.25	1.00	0.003	9.5E-01
Mtr.17909.1.S1_at	11.62	10.78	11.44	11.39	11.35	11.34	11.28	0.44	11.36	0.03	1.01	0.010	7.7E-01
Mtr.7711.1.S1_at	5.07	4.58	4.18	6.27	5.27	5.45	4.61	0.44	5.66	0.53	1.23	0.297	5.8E-02
Mtr.25197.1.S1_at	2.20	2.97	2.19	2.19	2.18	2.19	2.45	0.44	2.19	0.00	0.89	-0.167	3.5E-01
Mtr.13406.1.S1_at	6.70	5.90	6.63	6.90	6.95	6.84	6.41	0.44	6.89	0.06	1.08	0.105	1.3E-01
Mtr.47572.1.S1_s_at	12.37	11.51	12.13	12.28	12.09	12.19	12.01	0.44	12.19	0.09	1.02	0.022	5.3E-01
Mtr.49372.1.S1_at	9.07	8.26	8.34	9.07	9.08	8.97	8.56	0.44	9.04	0.06	1.06	0.080	1.3E-01
Mtr.43670.1.S1_at	8.63	7.88	8.66	8.69	8.74	8.85	8.39	0.44	8.76	0.08	1.04	0.062	2.3E-01
Mtr.15424.1.S1_at	2.80	2.92	3.62	2.62	2.79	2.66	3.12	0.44	2.69	0.09	0.86	-0.213	1.8E-01
Mtr.27779.1.S1_at	2.97	3.71	2.91	3.05	2.90	2.89	3.20	0.44	2.94	0.09	0.92	-0.119	3.9E-01

Mtr.2958.1.S1_at	10.19	9.33	9.96	9.94	9.84	9.89	9.83	0.44	9.89	0.05	1.01	0.009	8.2E-01
Mtr.48402.1.S1_at	2.81	3.54	2.75	2.73	2.69	2.68	3.03	0.44	2.70	0.03	0.89	-0.169	2.6E-01
Mtr.51822.1.S1_at	7.40	7.07	7.95	7.91	7.49	7.59	7.47	0.44	7.66	0.22	1.03	0.037	5.4E-01
Mtr.36985.1.S1_at	3.05	3.07	3.83	3.03	2.97	2.98	3.31	0.44	2.99	0.03	0.90	-0.149	2.7E-01
Mtr.38347.1.S1_at	12.02	11.99	11.24	11.52	11.51	11.58	11.75	0.44	11.54	0.04	0.98	-0.026	4.6E-01
Mtr.41672.1.S1_at	8.77	7.94	8.62	8.54	8.62	8.64	8.44	0.44	8.60	0.05	1.02	0.027	5.7E-01
Mtr.16781.1.S1_at	2.52	3.40	2.94	3.45	3.94	3.30	2.95	0.44	3.56	0.33	1.21	0.271	1.3E-01
Mtr.31753.1.S1_a_at	2.39	3.15	2.38	2.38	2.38	2.37	2.64	0.44	2.38	0.00	0.90	-0.153	3.6E-01
Mtr.32912.1.S1_at	6.57	5.80	6.57	6.54	6.91	6.55	6.31	0.44	6.67	0.21	1.06	0.078	2.8E-01
Mtr.52011.1.S1_s_at	2.93	2.72	3.57	3.35	3.41	3.09	3.07	0.44	3.28	0.17	1.07	0.096	4.8E-01
Mtr.13630.1.S1_at	10.39	9.71	9.57	9.91	10.03	9.92	9.89	0.44	9.95	0.06	1.01	0.009	8.2E-01
Mtr.8906.1.S1_at	10.43	9.54	10.03	10.04	10.02	10.10	10.00	0.44	10.05	0.04	1.01	0.007	8.5E-01
Mtr.29069.1.S1_at	4.77	4.60	5.44	4.66	5.07	5.03	4.94	0.44	4.92	0.22	1.00	-0.006	9.5E-01
Mtr.3681.1.S1_at	9.92	9.39	10.27	9.78	10.02	9.63	9.86	0.44	9.81	0.19	0.99	-0.008	8.6E-01
Mtr.31548.1.S1_at	9.58	8.77	9.49	9.34	9.60	9.49	9.28	0.44	9.48	0.13	1.02	0.030	5.0E-01
Mtr.7325.1.S1_at	9.40	9.80	10.28	9.76	9.82	9.57	9.83	0.44	9.72	0.13	0.99	-0.016	7.1E-01
Mtr.32375.1.S1_at	8.26	7.39	7.95	8.09	8.35	8.25	7.86	0.44	8.23	0.13	1.05	0.065	2.4E-01
Mtr.3214.1.S1_at	10.16	10.98	10.85	10.17	10.55	10.08	10.66	0.44	10.27	0.25	0.96	-0.055	2.5E-01
Mtr.2280.1.S1_at	9.28	8.39	8.86	8.82	8.77	8.89	8.84	0.44	8.82	0.06	1.00	-0.003	9.5E-01
Mtr.33706.1.S1_at	6.99	6.45	7.33	6.97	7.23	6.97	6.92	0.44	7.06	0.15	1.02	0.028	6.4E-01
Mtr.33532.1.S1_at	7.58	6.78	7.52	7.73	7.61	7.63	7.29	0.44	7.65	0.07	1.05	0.070	2.3E-01
Mtr.37350.1.S1_at	11.83	12.37	12.70	12.47	12.55	12.53	12.30	0.44	12.52	0.04	1.02	0.025	4.4E-01
Mtr.40731.1.S1_at	3.22	3.96	3.17	3.47	3.08	3.18	3.45	0.44	3.24	0.20	0.94	-0.090	5.0E-01
Mtr.9461.1.S1_at	9.58	8.74	8.91	9.18	9.25	9.33	9.08	0.44	9.25	0.08	1.02	0.027	5.4E-01
Mtr.2025.1.S1_at	7.52	6.68	7.33	7.54	7.62	7.50	7.18	0.44	7.55	0.06	1.05	0.073	2.2E-01
Mtr.23655.1.S1_s_at	2.85	3.59	2.80	2.73	2.73	2.72	3.08	0.44	2.73	0.01	0.89	-0.175	2.4E-01
Mtr.27529.1.S1_at	9.05	9.52	9.93	9.66	9.55	9.08	9.50	0.44	9.43	0.31	0.99	-0.011	8.3E-01
Mtr.37781.1.S1_at	12.66	13.41	13.44	13.03	12.98	13.10	13.17	0.44	13.04	0.06	0.99	-0.015	6.3E-01
Mtr.45563.1.S1_at	10.01	9.23	9.26	9.39	9.64	9.74	9.50	0.44	9.59	0.18	1.01	0.014	7.6E-01
Mtr.38747.1.S1_s_at	11.18	10.31	10.61	10.88	10.46	10.78	10.70	0.44	10.71	0.22	1.00	0.001	9.9E-01
Mtr.30684.1.S1_s_at	9.84	9.04	9.78	9.81	9.94	9.88	9.55	0.44	9.88	0.06	1.03	0.048	2.8E-01
Mtr.30917.1.S1_at	2.88	3.64	2.86	2.85	2.80	2.90	3.13	0.44	2.85	0.05	0.91	-0.132	3.5E-01
Mtr.18295.1.S1_at	8.52	8.14	9.02	8.74	8.73	8.73	8.56	0.44	8.73	0.01	1.02	0.029	5.3E-01
Mtr.15809.1.S1_at	7.95	7.62	8.50	8.15	8.42	8.06	8.02	0.44	8.21	0.19	1.02	0.033	5.4E-01
Mtr.44332.1.S1_at	9.56	8.76	9.48	9.24	9.16	9.10	9.27	0.44	9.16	0.07	0.99	-0.016	7.1E-01
Mtr.45482.1.S1_at	8.84	8.66	9.50	9.07	9.13	9.02	9.00	0.44	9.07	0.05	1.01	0.012	7.9E-01
Mtr.2605.1.S1_at	9.07	8.30	8.30	8.59	8.50	8.76	8.56	0.44	8.62	0.13	1.01	0.010	8.3E-01
Mtr.5489.1.S1_at	8.28	7.86	8.74	8.43	8.37	8.19	8.29	0.44	8.33	0.13	1.00	0.006	9.1E-01
Mtr.40298.1.S1_at	12.89	12.06	12.21	12.32	12.47	12.39	12.39	0.44	12.40	0.08	1.00	0.001	9.8E-01
Mtr.11521.1.S1_at	8.31	7.43	7.79	8.18	8.13	8.29	7.84	0.44	8.20	0.08	1.05	0.064	2.4E-01
Mtr.38711.1.S1_at	9.82	9.75	9.02	9.35	9.60	9.38	9.53	0.44	9.44	0.14	0.99	-0.013	7.6E-01
Mtr.17319.1.S1_at	8.55	8.43	9.25	9.04	9.09	8.95	8.74	0.44	9.03	0.07	1.03	0.046	3.3E-01
Mtr.3047.1.S1_at	7.53	6.88	6.69	6.89	6.99	6.73	7.03	0.44	6.87	0.13	0.98	-0.033	5.9E-01
Mtr.40053.1.S1_at	13.47	14.15	13.31	13.75	13.78	13.68	13.64	0.44	13.74	0.05	1.01	0.010	7.3E-01
Mtr.10121.1.S1_at	7.68	6.82	7.41	7.44	7.46	7.44	7.30	0.44	7.45	0.01	1.02	0.029	6.0E-01
Mtr.32888.1.S1_at	7.07	7.77	6.96	7.03	7.06	7.33	7.27	0.44	7.14	0.17	0.98	-0.025	6.7E-01
Mtr.3369.1.S1_at	3.68	3.85	4.52	3.72	3.65	3.42	4.02	0.44	3.60	0.16	0.89	-0.161	1.9E-01
Mtr.4776.1.S1_at	7.27	6.55	7.36	7.56	7.26	7.26	7.06	0.44	7.36	0.18	1.04	0.060	3.4E-01
Mtr.34418.1.S1_at	6.45	6.45	5.69	5.65	5.93	5.84	6.20	0.44	5.81	0.14	0.94	-0.094	2.2E-01
Mtr.7435.1.S1_at	8.98	8.23	9.00	9.22	8.98	9.06	8.73	0.44	9.09	0.12	1.04	0.057	2.5E-01
Mtr.37368.1.S1_at	10.06	9.39	9.22	8.61	8.82	8.98	9.56	0.44	8.80	0.19	0.92	-0.119	5.3E-02
Mtr.29607.1.S1_at	9.38	8.81	9.67	9.34	9.12	9.17	9.28	0.44	9.21	0.12	0.99	-0.012	7.9E-01
Mtr.20835.1.S1_at	2.35	3.11	2.34	2.34	2.33	2.34	2.60	0.44	2.34	0.00	0.90	-0.153	3.6E-01
Mtr.2602.1.S1_at	9.41	8.55	9.17	9.36	9.15	9.40	9.04	0.44	9.30	0.13	1.03	0.041	3.9E-01
Mtr.19090.1.S1_at	3.92	3.17	3.94	3.96	4.55	3.76	3.68	0.44	4.09	0.41	1.11	0.154	3.0E-01
Mtr.40990.1.S1_s_at	3.68	3.10	3.96	3.83	3.82	4.29	3.58	0.44	3.98	0.27	1.11	0.152	2.5E-01
Mtr.24437.1.S1_at	2.69	3.42	2.64	2.63	2.62	2.61	2.92	0.44	2.62	0.01	0.90	-0.155	3.1E-01
Mtr.8070.1.S1_at	3.10	3.84	3.06	3.05	2.82	3.05	3.33	0.44	2.97	0.13	0.89	-0.165	2.5E-01
Mtr.41359.1.S1_at	8.72	7.96	8.72	8.71	8.99	8.88	8.46	0.44	8.86	0.14	1.05	0.066	2.1E-01
Mtr.48218.1.S1_at	2.65	3.41	2.63	2.61	2.60	2.58	2.90	0.44	2.60	0.02	0.90	-0.158	3.0E-01
Mtr.12299.1.S1_at	9.69	8.87	9.54	9.61	9.62	9.56	9.37	0.44	9.60	0.03	1.02	0.035	4.2E-01
Mtr.14756.1.S1_at	5.44	4.99	5.87	5.17	5.69	5.23	5.43	0.44	5.37	0.29	0.99	-0.017	8.4E-01
Mtr.22084.1.S1_at	3.04	2.60	3.48	3.02	2.89	3.35	3.04	0.44	3.09	0.24	1.02	0.022	8.8E-01
Mtr.23047.1.S1_at	7.78	7.07	7.88	7.80	7.72	7.80	7.57	0.44	7.77	0.05	1.03	0.037	4.8E-01
Mtr.18842.1.S1_at	9.83	8.95	9.37	9.48	9.40	9.56	9.38	0.44	9.48	0.08	1.01	0.015	7.3E-01
Mtr.33055.1.S1_at	6.05	5.87	6.70	6.15	6.12	6.41	6.21	0.44	6.23	0.16	1.00	0.005	9.4E-01
Mtr.47942.1.S1_at	10.83	10.82	11.59	11.37	10.99	10.96	11.08	0.44	11.11	0.23	1.00	0.004	9.2E-01
Mtr.38653.1.S1_at	10.55	9.79	10.55	10.61	10.49	10.43	10.29	0.44	10.51	0.09	1.02	0.030	4.6E-01
Mtr.15962.1.S1_at	4.85	5.62	4.88	4.92	4.84	4.95	5.12	0.44	4.90	0.06	0.96	-0.061	4.5E-01
Mtr.4436.1.S1_at	7.91	7.72	8.56	8.26	8.24	8.11	8.06	0.44	8.20	0.08	1.02	0.025	6.1E-01
Mtr.31738.1.S1_at	10.43	11.18	10.41	9.87	10.39	9.95	10.67	0.44	10.07	0.28	0.94	-0.083	1.2E-01
Mtr.44955.1.S1_at	5.74	5.01	5.80	5.80	5.82	5.78	5.51	0.44	5.80	0.02	1.05	0.073	3.2E-01
Mtr.8792.1.S1_s_at	8.95	8.07	8.45	8.13	8.30	8.18	8.49	0.44	8.20	0.09	0.97	-0.049	3.4E-01
Mtr.32782.1.S1_at	6.11	5.46	6.30	5.87	5.79	5.92	5.96	0.44	5.86	0.07	0.98	-0.023	7.3E-01
Mtr.42393.1.S1_at	6.71	6.29	7.17	6.83	6.45	6.23	6.72	0.44	6.50	0.30	0.97	-0.047	5.2E-01
Mtr.42781.1.S1_at	8.52	7.72	8.44	8.71	8.52	8.61	8.23	0.44	8.61	0.10	1.05	0.065	2.2E-01
Mtr.37132.1.S1_at	4.29	3.52	4.28	4.25	4.33	4.26	4.03	0.44	4.28	0.04	1.06	0.087	3.8E-01
Mtr.4338.1.S1_at	7.92	8.58	8.76	8.00	7.79	7.79	8.42	0.44	7.86	0.12	0.93	-0.099	1.0E-01
Mtr.6092.1.S1_at	5.21	6.00	5.96	5.77	6.01	6.12	5.72	0.44	5.97	0.18	1.04	0.060	4.3E-01
Mtr.40374.1.S1_x_at	2.68	3.43	2.65	2.63	2.66	2.65	2.92	0.44	2.64	0.02	0.91	-0.143	3.4E-01
Mtr.17982.1.S1_s_at	5.69	4.99	4.87	6.30	6.31	5.43	5.18	0.44	6.01	0.51	1.16	0.214	9.9E-02
Mtr.1349.1.S1_at	2.80	3.57	3.56	3.28	3.26	3.24	3.31	0.44	3.26	0.02	0.98	-0.023	8.5E-01
Mtr.42418.1.S1_at	10.80	9.92	10.40	10.48	10.57	10.46	10.37	0.44	10.50	0.06	1.01	0.018	6.4E-01
Mtr.12225.1.S1_at	12.09	12.44	12.97	12.37	12.33	12.09	12.50	0.44	12.27	0.15	0.98	-0.028	4.3E-01
Mtr.32341.1.S1_s_at	9.86	9.06	9.79	9.85	9.74	9.86	9.57	0.44	9.81	0.07	1.03	0.036	4.0E-01
Mtr.32412.1.S1_s_at	8.03	7.33	8.14	7.84	7.86	8.01	7.83	0.44	7.90	0.09	1.01	0.013	8.0E-01
Mtr.15646.1.S1_at	13.32	12.65	13.48	13.86	14.37	13.90	13.15	0.44	14.05	0.28	1.07	0.095	4.1E-02
Mtr.41983.1.S1_at	12.03	11.22	11.33	11.70	11.91	12.03	11.53	0.44	11.88	0.17	1.03	0.043	2.7E-01
Mtr.52289.1.S1_at	8.95	8.17	8.91	8.92	8.84	8.94	8.67	0.44	8.90	0.05	1.03	0.037	4.3E-01
Mtr.23233.1.S1_at	2.59	3.34	2.57	2.59	2.58	2.56	2.83	0.44	2.58	0.02	0.91	-0.137	3.7E-01
Mtr.43023.1.S1_at	14.98	14.29	14.16	14.45	14.05	14.48	14.47	0.44	14.33	0.24	0.99	-0.015	6.4E-01
Mtr.48409.1.S1_at	2.97	3.72	2.95	2.95	2.91	2.95	3.22	0.44	2.94	0.02	0.91	-0.130	3.4E-01
Mtr.27972.1.S1_at	9.59	8.71	9.20	8.85	8.76	8.89	9.17	0.44	8.83	0.07	0.96	-0.053	2.6E-01
Mtr.33806.1.S1_at	7.27	6.68	7.54	7.13	7.40	7.47	7.16	0.44	7.33	0.18	1.02	0.035	5.6E-01
Mtr.46990.1.S1_at	2.16	2.91	2.14	2.14	2.14	2.14	2.40	0.44	2.14	0.00	0.89	-0.167	3.6E-01
Mtr.5542.1.S1_at	7.95	7.08	7.59	8.13	7.82	7.62	7.54	0.44	7.86	0.26	1.04	0.060	3.3E-01
Mtr.19140.1.S1_at	2.63	3.38	2.61	2.60	2.55	2.58	2.87	0.44	2.58	0.03	0.90	-0.156	3.1E-01
Mtr.9740.1.S1_at	6.17	5.43	6.21	6.11	6.68	6.48	5.94	0.44	6.42	0.29	1.08	0.114	1.8E-01
Mtr.28616.1.S1_at	7.56	6.71	7.34	7.33	7.21	7.27	7.20	0.44	7.27	0.06	1.01	0.014	8.0E-01
Mtr.39101.1.S1_at	9.21	8.96	9.81	9.33	9.63	9.43	9.33	0.44	9.46	0.15	1.01	0.021	6.3E-01
Mtr.11753.1.S1_at	7.70	7.27	8.15	7.68	7.43	7.87	7.70	0.44	7.66	0.22	0.99	-0.008	8.9E-01
Mtr.18746.1.S1_s_at	8.56	7.90	8.73	8.34	8.72	8.56	8.40	0.44	8.54	0.19	1.02	0.024	6.4E-01
Mtr.17248.1.S1_at	8.88	8.03	8.65	8.60	8.22	8.52	8.52	0.44	8.45	0.20	0.99	-0.012	8.1E-01
Mtr.11626.1.S1_at	9.80	9.01	9.74	9.51	9.69	9.50	9.52	0.44	9.56	0.11	1.00	0.007	8.7E-01
Mtr.10665.1.S1_at	10.97	11.48	11.84	11.18	11.54	11.21	11.43	0.44	11.31	0.20	0.99	-0.016	6.8E-01
Mtr.16734.1.S1_at	7.97	7.37	8.22	8.17	8.21	8.32	7.85	0.44	8.23	0.08	1.05	0.069	2.1E-01
Mtr.19210.1.S1_at	3.57	4.31	3.54	3.62	3.50	3.53	3.80	0.44	3.55	0.06	0.93	-0.099	3.8E-01
Mtr.5827.1.S1_at	7.95	7.28	8.10	8.18	8.12	8.22	7.78	0.44	8.17	0.05	1.05	0.072	1.9E-01
Mtr.31513.1.S1_at	3.64	2.87	3.61	3.60	3.60	3.59	3.37	0.44	3.60	0.01	1.07	0.094	4.2E-01
Mtr.23843.1.S1_at	11.21	10.49	10.41	10.85	10.94	10.97	10.71	0.44	10.92	0.06	1.02	0.029	4.5E-01
Mtr.4183.1.S1_at	2.86	3.61	2.83	2.84	2.84	2.85	3.10	0.44	2.84	0.00	0.92	-0.124	3.7E-01
Mtr.39302.1.S1_at	8.30	7.70	8.55	8.09	8.31	7.85	8.18	0.44	8.08	0.23	0.99	-0.018	7.5E-01
Mtr.8330.1.S1_at	5.10	5.02	5.81	5.08	5.14	5.07	5.31	0.44	5.10	0.04	0.96	-0.059	4.5E-01
Mtr.35926.1.S1_at	6.93	6.05	6.48	6.50	6.94	6.99	6.49	0.44	6.81	0.27	1.05	0.070	3.4E-01
Mtr.37780.1.S1_at	10.44	10.41	11.18	10.36	10.40	9.94	10.68	0.44	10.23	0.26	0.96	-0.061	2.1E-01
Mtr.45994.1.S1_s_at	3.00	2.15	2.75	4.72	4.07	3.48	2.63	0.44	4.09	0.62	1.55	0.634	2.9E-02
Mtr.46994.1.S1_s_at	11.08	10.69	11.56	11.36	11.19	11.27	11.11	0.44	11.27	0.08	1.01	0.021	5.6E-01
Mtr.21273.1.S1_at	7.65	7.32	8.18	7.33	7.48	7.47	7.72	0.44	7.42	0.09	0.96	-0.056	3.2E-01
Mtr.6008.1.S1_at	7.98	7.11	7.48	7.96	8.27	8.28	7.52	0.44	8.17	0.18	1.09	0.119	7.7E-02
Mtr.22403.1.S1_at	2.24	2.99	2.23	2.23	2.23	2.21	2.49	0.44	2.22	0.01	0.89	-0.161	3.6E-01
Mtr.33232.1.S1_at	8.80	8.46	9.33	9.02	9.37	8.95	8.86	0.44	9.11	0.23	1.03	0.040	4.3E-01
Mtr.22818.1.S1_at	8.32	7.46	8.05	10.02	10.27	9.95	7.94	0.44	10.08	0.17	1.27	0.344	1.4E-03
Mtr.122.1.S1_at	3.59	4.34	3.58	3.57	3.47	3.58	3.83	0.44	3.54	0.06	0.92	-0.115	3.1E-01
Mtr.29158.1.S1_at	8.65	8.54	9.35	8.74	8.80	8.63	8.85	0.44	8.72	0.09	0.99	-0.020	6.6E-01
Mtr.48783.1.S1_at	2.86	3.62	2.87	2.83	2.79	2.77	3.12	0.44	2.80	0.03	0.90	-0.156	2.8E-01
Mtr.1914.1.S1_at	2.51	2.54	3.28	2.45	2.46	2.45	2.78	0.44	2.45	0.01	0.88	-0.181	2.7E-01
Mtr.23638.1.S1_at	10.31	9.75	10.62	10.16	10.31	10.09	10.23	0.44	10.19	0.11	1.00	-0.005	8.9E-01
Mtr.19651.1.S1_at	7.19	6.49	7.30	6.87	7.30	6.81	7.00	0.44	6.99	0.27	1.00	-0.001	9.8E-01
Mtr.9102.1.S1_at	8.25	7.53	8.32	8.02	8.10	8.16	8.03	0.44	8.09	0.07	1.01	0.011	8.2E-01
Mtr.48598.1.S1_x_at	3.19	3.86	3.04	3.18	3.01	3.07	3.36	0.44	3.09	0.09	0.92	-0.123	3.5E-01
Mtr.627.1.S1_at	2.36	3.11	2.34	2.35	2.45	2.29	2.61	0.44	2.37	0.08	0.91	-0.140	4.0E-01

Mtr.6982.1.S1_at	5.97	5.47	6.34	6.43	6.35	6.37	5.93	0.44	6.38	0.04	1.08	0.107	1.5E-01
Mtr.2864.1.S1_at	7.45	8.33	7.85	7.43	8.06	7.55	7.88	0.44	7.68	0.34	0.98	-0.036	5.7E-01
Mtr.47800.1.S1_at	2.83	3.57	2.79	2.79	2.79	3.09	3.07	0.44	2.89	0.18	0.94	-0.085	5.6E-01
Mtr.12842.1.S1_at	3.75	2.88	3.18	3.44	3.17	2.77	3.27	0.44	3.13	0.33	0.96	-0.064	6.8E-01
Mtr.40474.1.S1_at	9.34	8.78	9.64	9.55	9.79	9.34	9.25	0.44	9.56	0.22	1.03	0.047	3.4E-01
Mtr.8953.1.S1_s_at	9.32	8.94	8.45	8.87	8.77	8.93	8.90	0.44	8.86	0.08	0.99	-0.007	8.7E-01
Mtr.46038.1.S1_at	2.69	3.43	2.66	2.64	2.62	2.63	2.93	0.44	2.63	0.01	0.90	-0.155	3.0E-01
Mtr.33336.1.S1_at	8.07	7.31	7.31	7.47	7.41	7.56	7.57	0.44	7.48	0.07	0.99	-0.016	7.6E-01
Mtr.34410.1.S1_at	6.43	6.54	7.23	7.03	6.66	6.63	6.73	0.44	6.77	0.22	1.01	0.008	9.0E-01
Mtr.45557.1.S1_at	9.25	8.38	8.74	9.37	9.14	9.38	8.79	0.44	9.30	0.13	1.06	0.081	1.3E-01
Mtr.44127.1.S1_at	6.22	5.82	6.69	6.36	6.69	6.41	6.24	0.44	6.49	0.18	1.04	0.056	4.2E-01
Mtr.38593.1.S1_at	10.32	9.56	10.32	10.21	9.94	10.09	10.07	0.44	10.08	0.14	1.00	0.002	9.7E-01
Mtr.45408.1.S1_at	7.75	6.94	7.65	7.70	7.54	7.65	7.45	0.44	7.63	0.09	1.02	0.035	5.2E-01
Mtr.10131.1.S1_at	6.09	6.50	6.96	6.34	6.38	6.40	6.52	0.44	6.37	0.03	0.98	-0.032	6.1E-01
Mtr.26623.1.S1_at	2.27	3.02	2.25	2.25	2.24	2.25	2.51	0.44	2.25	0.01	0.89	-0.161	3.5E-01
Mtr.41065.1.S1_at	7.98	8.67	8.80	8.65	8.55	8.50	8.48	0.44	8.57	0.08	1.01	0.014	7.6E-01
Mtr.33224.1.S1_s_at	8.14	7.35	8.07	8.16	8.32	8.20	7.85	0.44	8.23	0.08	1.05	0.068	2.2E-01
Mtr.52344.1.S1_at	9.17	8.30	8.66	8.69	8.65	8.41	8.71	0.44	8.58	0.15	0.99	-0.021	6.6E-01
Mtr.42253.1.S1_at	9.64	9.36	10.22	9.90	9.80	10.03	9.74	0.44	9.91	0.12	1.02	0.024	5.6E-01
Mtr.33006.1.S1_a_at	5.26	5.57	6.12	5.55	5.67	5.73	5.65	0.44	5.65	0.09	1.00	0.000	1.0E+00
Mtr.20120.1.S1_at	12.40	13.14	13.17	12.71	12.87	12.94	12.90	0.44	12.84	0.12	1.00	-0.006	8.4E-01
Mtr.42367.1.S1_at	7.61	7.26	8.13	8.34	8.28	8.31	7.66	0.44	8.31	0.03	1.08	0.116	6.4E-02
Mtr.44133.1.S1_at	10.16	9.29	9.80	10.04	9.94	9.98	9.75	0.44	9.99	0.05	1.02	0.035	4.0E-01
Mtr.17380.1.S1_at	2.80	3.56	2.82	2.74	2.74	2.71	3.06	0.44	2.73	0.02	0.89	-0.165	2.6E-01
Mtr.37210.1.S1_at	13.84	13.03	13.15	13.50	12.84	13.45	13.34	0.44	13.27	0.37	0.99	-0.008	8.3E-01
Mtr.20185.1.S1_x_at	13.58	12.70	13.18	13.19	12.78	13.17	13.15	0.44	13.05	0.23	0.99	-0.011	7.4E-01
Mtr.47086.1.S1_at	9.21	8.86	8.34	8.78	8.48	8.90	8.80	0.44	8.72	0.21	0.99	-0.013	7.9E-01
Mtr.24151.1.S1_at	3.15	3.89	3.12	3.10	3.09	3.08	3.39	0.44	3.09	0.01	0.91	-0.134	3.0E-01
Mtr.34750.1.S1_at	8.92	8.39	9.26	8.85	8.80	8.84	8.86	0.44	8.83	0.02	1.00	-0.005	9.2E-01
Mtr.9187.1.S1_at	6.03	6.06	6.80	6.00	5.81	5.91	6.30	0.44	5.91	0.09	0.94	-0.093	2.0E-01
Mtr.35859.1.S1_at	8.36	7.80	8.67	8.58	8.43	8.45	8.28	0.44	8.49	0.08	1.03	0.036	4.6E-01
Mtr.1629.1.S1_s_at	8.93	8.14	8.87	8.97	8.92	8.81	8.65	0.44	8.90	0.08	1.03	0.042	3.8E-01
Mtr.33411.1.S1_at	8.16	7.30	7.90	7.89	7.80	8.02	7.79	0.44	7.90	0.11	1.02	0.022	6.7E-01
Mtr.23406.1.S1_at	8.86	8.56	9.42	8.90	9.27	9.20	8.95	0.44	9.13	0.20	1.02	0.029	5.5E-01
Mtr.12659.1.S1_at	9.45	8.84	9.69	9.63	9.49	9.48	9.33	0.44	9.53	0.08	1.02	0.032	4.6E-01
Mtr.46874.1.S1_at	7.68	7.26	8.14	7.92	7.55	7.59	7.70	0.44	7.69	0.20	1.00	-0.002	9.8E-01
Mtr.38737.1.S1_at	10.05	9.41	9.22	9.63	9.36	9.48	9.56	0.44	9.49	0.14	0.99	-0.010	8.1E-01
Mtr.21244.1.S1_at	5.97	5.09	5.55	6.00	5.84	5.84	5.54	0.44	5.90	0.09	1.06	0.091	2.4E-01
Mtr.35412.1.S1_at	9.59	9.08	9.94	9.52	9.65	9.34	9.53	0.44	9.50	0.15	1.00	-0.005	9.2E-01
Mtr.26054.1.S1_at	3.46	2.59	2.99	3.06	2.78	3.12	3.01	0.44	2.98	0.18	0.99	-0.014	9.2E-01
Mtr.5672.1.S1_at	10.08	9.21	9.63	9.61	9.81	9.65	9.64	0.44	9.69	0.11	1.01	0.008	8.5E-01
Mtr.899.1.S1_at	3.03	3.78	3.02	3.01	3.01	2.98	3.28	0.44	3.00	0.02	0.92	-0.126	3.4E-01
Mtr.7389.1.S1_at	7.98	7.40	8.26	8.00	7.86	8.03	7.88	0.44	7.96	0.09	1.01	0.016	7.6E-01
Mtr.10514.1.S1_at	10.18	9.32	9.60	9.82	9.79	9.84	9.70	0.44	9.82	0.02	1.01	0.017	6.7E-01
Mtr.40530.1.S1_at	5.05	5.36	4.49	5.65	5.50	5.14	4.97	0.44	5.43	0.26	1.09	0.129	1.9E-01
Mtr.41908.1.S1_at	9.00	8.79	9.63	9.27	8.96	9.03	9.14	0.44	9.09	0.16	0.99	-0.009	8.5E-01
Mtr.39523.1.S1_at	8.85	8.18	9.00	8.92	9.04	8.80	8.68	0.44	8.92	0.12	1.03	0.039	4.1E-01
Mtr.4969.1.S1_at	7.41	6.78	7.62	7.51	7.78	7.62	7.27	0.44	7.64	0.14	1.05	0.072	2.3E-01
Mtr.20763.1.S1_at	2.38	3.13	2.36	2.34	2.33	2.58	2.62	0.44	2.42	0.14	0.92	-0.118	4.8E-01
Mtr.17938.1.S1_at	6.48	6.51	7.25	6.37	6.45	6.47	6.74	0.44	6.43	0.05	0.95	-0.068	2.9E-01
Mtr.19968.1.S1_x_at	4.41	5.11	4.30	4.34	4.39	4.42	4.61	0.44	4.38	0.04	0.95	-0.072	4.3E-01
Mtr.45460.1.S1_at	10.00	10.87	10.48	10.07	10.36	10.02	10.45	0.44	10.15	0.19	0.97	-0.042	3.3E-01
Mtr.20749.1.S1_at	3.03	3.76	2.97	2.92	3.02	3.01	3.25	0.44	2.98	0.05	0.92	-0.126	3.5E-01
Mtr.31520.1.S1_at	5.41	4.68	4.63	5.10	4.63	5.40	4.90	0.44	5.04	0.39	1.03	0.041	7.0E-01
Mtr.25654.1.S1_at	2.83	2.69	3.51	7.81	7.94	7.42	3.01	0.44	7.72	0.27	2.57	1.360	9.1E-05
Mtr.42359.1.S1_at	11.24	10.37	10.88	10.88	10.83	10.88	10.83	0.44	10.86	0.03	1.00	0.004	9.1E-01
Mtr.11567.1.S1_s_at	3.43	4.24	4.12	4.17	4.11	4.45	3.93	0.44	4.25	0.18	1.08	0.111	3.1E-01
Mtr.51178.1.S1_at	6.16	6.74	5.89	5.89	5.79	5.89	6.27	0.44	5.86	0.06	0.93	-0.097	1.8E-01
Mtr.684.1.S1_at	8.50	9.25	9.27	9.73	10.14	9.80	9.01	0.44	9.89	0.22	1.10	0.135	3.5E-02
Mtr.6721.1.S1_at	4.42	3.60	4.27	4.32	4.34	4.18	4.10	0.44	4.28	0.09	1.05	0.065	5.0E-01
Mtr.10681.1.S1_at	11.46	11.71	12.31	11.38	11.97	11.10	11.83	0.44	11.49	0.45	0.97	-0.042	4.0E-01
Mtr.40028.1.S1_s_at	13.03	12.44	12.18	12.96	13.36	13.18	12.55	0.44	13.17	0.20	1.05	0.069	8.9E-02
Mtr.38327.1.S1_at	8.69	8.26	9.14	8.99	9.03	8.84	8.70	0.44	8.95	0.10	1.03	0.042	3.8E-01
Mtr.29952.1.S1_at	11.09	10.27	10.95	10.84	10.97	10.77	10.77	0.44	10.86	0.10	1.01	0.012	7.4E-01
Mtr.42913.1.S1_at	9.90	9.04	9.35	9.56	9.88	9.70	9.43	0.44	9.71	0.16	1.03	0.043	3.5E-01
Mtr.7509.1.S1_at	3.79	3.14	2.96	3.06	3.82	2.91	3.30	0.44	3.26	0.49	0.99	-0.015	9.3E-01
Mtr.4354.1.S1_at	8.83	8.04	8.75	8.88	8.65	8.64	8.54	0.44	8.72	0.14	1.02	0.031	5.3E-01
Mtr.6149.1.S1_at	5.82	5.00	5.14	6.12	5.88	5.78	5.32	0.44	5.93	0.18	1.11	0.156	8.9E-02
Mtr.31037.1.S1_at	5.56	5.12	4.69	5.08	5.08	5.08	5.12	0.44	5.08	0.00	0.99	-0.011	8.8E-01
Mtr.51342.1.S1_at	2.58	3.32	2.55	2.53	2.52	2.52	2.81	0.44	2.52	0.01	0.90	-0.156	3.2E-01
Mtr.33848.1.S1_at	9.09	8.38	9.17	9.09	9.21	9.21	8.88	0.44	9.17	0.07	1.03	0.047	3.2E-01
Mtr.4108.1.S1_at	2.19	3.00	2.32	2.15	2.18	2.18	2.51	0.44	2.17	0.02	0.87	-0.209	2.5E-01
Mtr.3074.1.S1_s_at	9.47	8.60	8.98	8.91	8.81	8.89	9.02	0.44	8.87	0.05	0.98	-0.023	6.0E-01
Mtr.10067.1.S1_at	4.70	3.94	4.70	4.63	4.66	4.66	4.45	0.44	4.65	0.02	1.05	0.064	4.7E-01
Mtr.40189.1.S1_at	11.04	11.79	11.79	11.70	12.18	11.70	11.54	0.44	11.86	0.27	1.03	0.040	3.4E-01
Mtr.47659.1.S1_at	2.45	3.19	2.43	2.38	2.37	2.50	2.69	0.44	2.42	0.07	0.90	-0.156	3.4E-01
Mtr.16551.1.S1_s_at	7.62	7.00	7.84	7.79	7.72	7.72	7.49	0.44	7.74	0.04	1.03	0.049	3.6E-01
Mtr.29540.1.S1_at	9.84	8.98	9.50	9.49	9.58	9.41	9.44	0.44	9.50	0.09	1.01	0.008	8.4E-01
Mtr.33721.1.S1_at	9.15	8.29	8.62	8.80	8.51	8.79	8.69	0.44	8.70	0.17	1.00	0.002	9.7E-01
Mtr.42348.1.S1_at	10.14	9.49	10.32	9.83	9.98	9.87	9.98	0.44	9.89	0.08	0.99	-0.013	7.4E-01
Mtr.40887.1.S1_at	10.60	9.87	10.64	10.30	10.47	10.19	10.37	0.44	10.32	0.14	1.00	-0.007	8.5E-01
Mtr.44762.1.S1_s_at	8.00	8.61	8.84	8.34	8.69	8.49	8.48	0.44	8.51	0.17	1.00	0.005	9.2E-01
Mtr.25496.1.S1_at	9.47	8.78	9.58	9.47	9.60	9.57	9.28	0.44	9.55	0.07	1.03	0.042	3.5E-01
Mtr.19236.1.S1_at	9.32	9.28	10.06	9.60	9.63	9.42	9.55	0.44	9.55	0.12	1.00	-0.001	9.9E-01
Mtr.18952.1.S1_at	3.96	3.30	3.13	3.36	3.08	3.90	3.46	0.44	3.45	0.41	1.00	-0.006	9.7E-01
Mtr.44941.1.S1_s_at	8.47	7.68	7.75	7.79	7.70	7.80	7.97	0.44	7.76	0.05	0.97	-0.038	4.6E-01
Mtr.19077.1.S1_x_at	3.03	3.74	2.95	2.92	2.89	2.85	3.24	0.44	2.89	0.03	0.89	-0.165	2.4E-01
Mtr.31650.1.S1_at	8.50	8.25	9.10	8.58	8.76	8.42	8.62	0.44	8.59	0.17	1.00	-0.006	9.1E-01
Mtr.2296.1.S1_at	4.25	5.01	5.00	4.10	4.49	4.61	4.75	0.44	4.40	0.27	0.93	-0.111	3.0E-01
Mtr.30700.1.S1_at	3.66	2.87	2.95	2.94	2.94	2.55	3.16	0.44	2.81	0.22	0.89	-0.168	2.9E-01
Mtr.47943.1.S1_s_at	10.19	10.63	11.06	10.57	10.59	10.44	10.63	0.44	10.53	0.08	0.99	-0.013	7.3E-01
Mtr.9040.1.S1_at	10.38	9.67	9.58	9.96	10.22	9.94	9.88	0.44	10.04	0.16	1.02	0.024	5.7E-01
Mtr.33177.1.S1_at	7.60	6.82	7.54	7.74	7.74	7.86	7.32	0.44	7.78	0.07	1.06	0.088	1.4E-01
Mtr.18655.1.S1_at	5.52	4.76	5.51	5.39	5.44	5.31	5.27	0.44	5.38	0.07	1.02	0.031	6.7E-01
Mtr.37877.1.S1_at	10.44	9.71	10.49	10.45	10.53	10.29	10.22	0.44	10.42	0.12	1.02	0.029	4.7E-01
Mtr.51392.1.S1_at	8.49	8.08	8.95	8.32	8.30	8.42	8.50	0.44	8.35	0.07	0.98	-0.027	5.7E-01
Mtr.10603.1.S1_at	13.29	14.16	13.80	13.45	13.56	13.30	13.75	0.44	13.44	0.13	0.98	-0.033	3.0E-01
Mtr.17874.1.S1_at	2.96	3.70	2.94	2.96	2.87	2.95	3.20	0.44	2.93	0.05	0.91	-0.129	3.4E-01
Mtr.31750.1.S1_at	4.98	4.11	4.52	4.71	4.43	4.01	4.54	0.43	4.38	0.35	0.97	-0.050	6.6E-01
Mtr.23430.1.S1_at	4.09	3.41	3.28	3.19	3.14	3.12	3.59	0.43	3.15	0.04	0.88	-0.190	1.5E-01
Mtr.42267.1.S1_at	8.77	8.40	9.26	8.75	9.00	8.86	8.81	0.43	8.87	0.13	1.01	0.010	8.3E-01
Mtr.52112.1.S1_at	4.75	4.23	5.09	4.85	5.70	5.27	4.69	0.43	5.27	0.42	1.12	0.169	1.7E-01
Mtr.48277.1.S1_at	10.09	9.28	9.41	9.79	9.63	9.74	9.59	0.43	9.72	0.08	1.01	0.019	6.5E-01
Mtr.13924.1.S1_at	5.69	4.84	5.10	5.31	5.10	5.25	5.21	0.43	5.22	0.11	1.00	0.002	9.8E-01
Mtr.8946.1.S1_at	8.91	8.08	8.71	8.51	8.89	8.63	8.57	0.43	8.68	0.19	1.01	0.019	7.0E-01
Mtr.11047.1.S1_at	9.67	8.81	9.34	9.30	9.39	9.45	9.27	0.43	9.38	0.08	1.01	0.017	7.0E-01
Mtr.13335.1.S1_at	9.13	8.35	9.08	9.10	8.96	9.10	8.85	0.43	9.05	0.08	1.02	0.032	4.8E-01
Mtr.7630.1.S1_at	10.12	9.25	9.72	9.88	9.83	9.76	9.70	0.43	9.82	0.06	1.01	0.019	6.4E-01
Mtr.46524.1.S1_at	3.25	3.26	4.01	3.23	3.23	3.23	3.50	0.43	3.23	0.00	0.92	-0.118	3.4E-01
Mtr.28981.1.S1_at	4.13	5.00	4.56	5.42	6.08	5.15	4.56	0.43	5.55	0.48	1.22	0.282	5.8E-02
Mtr.11515.1.S1_at	9.29	9.92	9.09	9.44	9.09	9.23	9.43	0.43	9.25	0.17	0.98	-0.028	5.4E-01
Mtr.11639.1.S1_at	11.17	10.37	11.07	11.20	11.18	11.26	10.87	0.43	11.21	0.04	1.03	0.045	2.4E-01
Mtr.19971.1.S1_at	7.80	7.11	7.91	7.71	7.88	7.70	7.61	0.43	7.76	0.10	1.02	0.030	5.7E-01
Mtr.18235.1.S1_at	2.12	2.87	2.11	2.11	2.10	2.10	2.36	0.43	2.10	0.00	0.89	-0.168	3.6E-01
Mtr.33676.1.S1_at	8.77	9.62	9.32	9.34	9.36	9.39	9.24	0.43	9.36	0.03	1.01	0.019	6.5E-01
Mtr.43799.1.S1_at	8.13	7.32	8.00	8.21	8.34	8.39	7.82	0.43	8.31	0.09	1.06	0.089	1.3E-01
Mtr.42560.1.S1_at	6.37	5.60	6.32	5.80	5.52	5.23	6.10	0.43	5.52	0.29	0.91	-0.144	1.3E-01
Mtr.24216.1.S1_x_at	2.79	3.52	2.75	2.74	2.69	2.69	3.02	0.43	2.71	0.03	0.90	-0.157	2.8E-01
Mtr.47313.1.S1_at	2.40	3.16	2.41	2.37	2.36	2.39	2.66	0.43	2.37	0.01	0.89	-0.162	3.2E-01
Mtr.26893.1.S1_at	10.86	10.07	10.77	10.92	10.71	10.88	10.57	0.43	10.83	0.11	1.03	0.036	3.6E-01
Mtr.3483.1.S1_at	3.46	3.91	3.04	3.15	3.07	3.26	3.47	0.43	3.16	0.09	0.91	-0.135	2.9E-01
Mtr.19387.1.S1_at	2.60	3.33	2.55	2.55	2.54	2.54	2.83	0.43	2.54	0.01	0.90	-0.153	3.2E-01
Mtr.51743.1.S1_at	2.39	3.13	2.38	2.37	2.37	2.37	2.63	0.43	2.37	0.00	0.90	-0.153	3.5E-01
Mtr.46587.1.S1_at	3.19	3.90	3.12	3.07	3.00	2.99	3.40	0.43	3.02	0.05	0.89	-0.174	2.0E-01
Mtr.13109.1.S1_at	8.68	8.06	8.89	8.74	8.94	8.67	8.54	0.43	8.78	0.14	1.03	0.040	4.2E-01
Mtr.26822.1.S1_at	9.07	8.92	9.74	9.48	9.61	9.19	9.24	0.43	9.43	0.21	1.02	0.028	5.5E-01
Mtr.37909.1.S1_at	10.78	11.59	11.45	11.07	11.29	10.87	11.27	0.43	11.08	0.21	0.98	-0.025	5.2E-01
Mtr.5260.1.S1_at	10.92	10.63	11.48	11.17	11.03	11.21	11.01	0.43	11.14	0.09	1.01	0.017	6.4E-01
Mtr.31547.1.S1_s_at	9.64	9.17	10.04	9.73	9.72	9.74	9.61	0.43	9.73	0.01	1.01	0.017	6.7E-01
Mtr.2073.1.S1_at	3.24	3.53	2.67	2.65	2.64	2.75	3.15	0.43	2.68	0.06	0.85	-0.232	1.4E-01
Mtr.13811.1.S1_at	6.39	6.04	6.90	6.58	6.62	6.25	6.45	0.43	6.48	0.21	1.01	0.009	9.0E-01

Mtr.11629.1.S1_at	3.97	4.14	3.32	4.07	3.93	3.93	3.81	0.43	3.98	0.08	1.04	0.062	5.5E-01
Mtr.48737.1.S1_at	4.21	3.57	4.40	4.45	4.25	4.04	4.06	0.43	4.25	0.21	1.05	0.066	5.3E-01
Mtr.17664.1.S1_at	8.07	7.22	7.79	7.67	7.60	7.60	7.69	0.43	7.62	0.04	0.99	-0.013	8.0E-01
Mtr.31886.1.S1_at	4.35	4.14	3.51	4.06	3.78	4.74	4.00	0.43	4.20	0.49	1.05	0.070	6.3E-01
Mtr.46798.1.S1_s_at	6.63	6.11	6.98	5.32	4.65	4.57	6.57	0.43	4.85	0.42	0.74	-0.439	7.6E-03
Mtr.20692.1.S1_at	3.67	2.80	3.30	3.86	3.80	3.66	3.26	0.43	3.77	0.10	1.16	0.213	1.1E-01
Mtr.17749.1.S1_at	2.61	3.35	2.58	2.56	2.55	2.53	2.85	0.43	2.55	0.01	0.89	-0.161	3.0E-01
Mtr.24297.1.S1_at	2.70	3.43	2.66	2.65	2.62	2.61	2.93	0.43	2.63	0.02	0.90	-0.157	3.0E-01
Mtr.15352.1.S1_at	7.03	6.33	7.12	7.55	7.29	7.39	6.83	0.43	7.41	0.13	1.09	0.118	9.0E-02
Mtr.4445.1.S1_at	10.28	10.07	10.91	10.56	10.54	10.55	10.42	0.43	10.55	0.01	1.01	0.018	6.2E-01
Mtr.24438.1.S1_at	3.15	3.88	3.11	3.08	3.06	3.08	3.38	0.43	3.07	0.01	0.91	-0.136	2.9E-01
Mtr.25749.1.S1_at	9.97	9.34	10.17	10.44	10.36	10.23	9.83	0.43	10.34	0.11	1.05	0.074	1.2E-01
Mtr.37933.1.S1_s_at	11.23	10.39	10.96	10.86	10.79	10.95	10.86	0.43	10.87	0.08	1.00	0.001	9.9E-01
Mtr.30679.1.S1_at	3.10	3.95	3.68	3.35	3.11	3.33	3.58	0.43	3.26	0.13	0.91	-0.134	2.9E-01
Mtr.20771.1.S1_at	10.54	9.82	10.61	10.60	10.40	10.57	10.32	0.43	10.52	0.11	1.02	0.028	4.8E-01
Mtr.38530.1.S1_at	7.31	6.46	7.02	7.09	7.51	7.14	6.93	0.43	7.25	0.23	1.05	0.064	3.3E-01
Mtr.596.1.S1_at	3.26	3.90	3.07	3.06	2.94	3.04	3.41	0.43	3.01	0.06	0.88	-0.179	1.9E-01
Mtr.36030.1.S1_at	3.35	2.80	3.66	2.58	4.09	2.67	3.27	0.43	3.11	0.85	0.95	-0.070	7.9E-01
Mtr.5712.1.S1_at	2.73	3.48	2.73	2.76	3.16	2.72	2.98	0.43	2.88	0.25	0.97	-0.048	7.5E-01
Mtr.4399.1.S1_at	5.57	5.09	5.96	5.64	6.28	5.98	5.54	0.43	5.97	0.32	1.08	0.107	2.4E-01
Mtr.14125.1.S1_at	10.77	10.16	11.00	10.78	10.90	10.71	10.64	0.43	10.79	0.09	1.01	0.020	5.8E-01
Mtr.5512.1.S1_at	9.64	9.13	8.78	9.11	9.13	9.21	9.18	0.43	9.15	0.05	1.00	-0.005	9.1E-01
Mtr.5753.1.S1_at	5.11	5.39	5.96	5.24	5.06	4.53	5.48	0.43	4.94	0.37	0.90	-0.149	1.8E-01
Mtr.23999.1.S1_at	8.69	8.11	8.96	9.02	9.19	8.95	8.59	0.43	9.05	0.12	1.05	0.076	1.5E-01
Mtr.29293.1.S1_at	5.63	5.21	6.08	5.82	5.97	5.69	5.64	0.43	5.82	0.14	1.03	0.047	5.2E-01
Mtr.42135.1.S1_at	7.18	6.38	7.06	6.89	7.06	7.06	6.87	0.43	7.00	0.09	1.02	0.027	6.4E-01
Mtr.25154.1.S1_s_at	9.24	8.75	8.37	9.12	8.81	9.21	8.79	0.43	9.05	0.21	1.03	0.042	4.0E-01
Mtr.7352.1.S1_at	11.08	10.26	10.90	11.14	11.07	11.17	10.75	0.43	11.13	0.05	1.04	0.050	2.1E-01
Mtr.2181.1.S1_at	6.26	5.40	5.77	5.09	5.36	5.14	5.81	0.43	5.20	0.14	0.89	-0.161	8.1E-02
Mtr.5495.1.S1_s_at	7.68	6.96	7.75	7.53	7.47	7.20	7.46	0.43	7.40	0.18	0.99	-0.012	8.3E-01
Mtr.27772.1.S1_s_at	10.97	10.12	10.71	10.75	10.84	10.95	10.60	0.43	10.85	0.10	1.02	0.033	3.9E-01
Mtr.14538.1.S1_at	9.95	9.18	9.91	9.85	9.83	9.81	9.68	0.43	9.83	0.02	1.02	0.022	5.9E-01
Mtr.34403.1.S1_at	7.69	8.51	7.85	7.71	7.62	7.74	8.02	0.43	7.69	0.06	0.96	-0.061	2.6E-01
Mtr.4491.1.S1_at	9.33	8.97	9.83	9.07	9.34	9.09	9.38	0.43	9.17	0.15	0.98	-0.033	4.7E-01
Mtr.31612.1.S1_at	6.94	7.02	7.73	7.15	7.30	7.26	7.23	0.43	7.23	0.08	1.00	0.001	9.9E-01
Mtr.32598.1.S1_at	2.91	3.64	2.87	2.86	2.84	2.85	3.14	0.43	2.85	0.01	0.91	-0.139	3.1E-01
Mtr.5053.1.S1_at	10.40	11.26	10.72	10.49	10.72	10.76	10.79	0.43	10.66	0.15	0.99	-0.018	6.3E-01
Mtr.9317.1.S1_at	9.71	9.00	9.78	9.93	9.89	9.90	9.50	0.43	9.90	0.02	1.04	0.061	1.8E-01
Mtr.14954.1.S1_at	2.25	2.99	2.24	2.23	2.23	2.23	2.49	0.43	2.23	0.00	0.89	-0.161	3.5E-01
Mtr.11527.1.S1_at	7.66	6.89	7.63	7.81	7.58	7.75	7.39	0.43	7.71	0.12	1.04	0.061	2.8E-01
Mtr.38265.1.S1_s_at	9.11	8.38	9.15	9.06	8.97	9.03	8.88	0.43	9.02	0.05	1.02	0.022	6.2E-01
Mtr.8095.1.S1_at	2.71	3.52	2.85	2.74	2.58	2.71	3.03	0.43	2.67	0.09	0.88	-0.178	2.4E-01
Mtr.1535.1.S1_at	6.98	6.18	6.87	7.08	6.87	6.88	6.68	0.43	6.94	0.12	1.04	0.056	3.6E-01
Mtr.13387.1.S1_at	8.70	7.91	8.61	8.52	8.61	8.29	8.41	0.43	8.47	0.17	1.01	0.011	8.3E-01
Mtr.39998.1.S1_at	9.09	8.25	8.82	8.82	8.29	8.68	8.72	0.43	8.60	0.27	0.99	-0.021	7.0E-01
Mtr.8939.1.S1_at	7.50	6.78	7.55	7.54	7.53	7.26	7.27	0.43	7.44	0.16	1.02	0.033	5.6E-01
Mtr.7927.1.S1_at	7.58	6.81	7.53	7.65	7.79	7.51	7.31	0.43	7.65	0.14	1.05	0.066	2.6E-01
Mtr.47601.1.S1_at	2.23	2.97	2.21	2.22	2.22	2.22	2.47	0.43	2.22	0.00	0.90	-0.152	3.8E-01
Mtr.4071.1.S1_at	7.47	6.88	7.73	7.62	7.69	7.54	7.36	0.43	7.62	0.07	1.03	0.049	3.7E-01
Mtr.2963.1.S1_at	7.02	6.36	7.18	6.69	7.11	6.96	6.85	0.43	6.92	0.21	1.01	0.014	8.2E-01
Mtr.233.1.S1_at	5.92	5.20	5.15	5.13	4.80	4.85	5.43	0.43	4.93	0.17	0.91	-0.139	1.4E-01
Mtr.42616.1.S1_at	6.88	6.29	7.13	7.07	7.56	7.48	6.76	0.43	7.37	0.26	1.09	0.124	1.1E-01
Mtr.12908.1.S1_at	8.91	8.07	8.33	8.64	8.68	8.76	8.44	0.43	8.70	0.06	1.03	0.043	3.6E-01
Mtr.43557.1.S1_at	5.80	5.52	6.37	7.11	7.87	7.23	5.90	0.43	7.40	0.41	1.26	0.328	1.2E-02
Mtr.44075.1.S1_at	9.79	8.95	9.56	9.64	9.84	9.81	9.43	0.43	9.76	0.11	1.03	0.049	2.7E-01
Mtr.32958.1.S1_at	5.26	5.60	6.12	5.54	6.23	5.48	5.66	0.43	5.75	0.42	1.02	0.022	8.1E-01
Mtr.4765.1.S1_s_at	2.86	3.56	2.77	2.72	2.74	2.69	3.06	0.43	2.72	0.03	0.89	-0.171	2.4E-01
Mtr.43798.1.S1_at	9.78	8.98	9.68	9.50	9.38	9.76	9.48	0.43	9.55	0.19	1.01	0.010	8.2E-01
Mtr.45261.1.S1_at	11.06	10.28	10.99	10.99	11.05	10.93	10.78	0.43	10.99	0.06	1.02	0.028	4.5E-01
Mtr.38896.1.S1_at	8.79	8.27	9.13	8.96	8.88	8.93	8.73	0.43	8.92	0.04	1.02	0.032	4.8E-01
Mtr.18609.1.S1_at	3.80	2.99	3.14	3.60	3.52	3.44	3.31	0.43	3.52	0.08	1.06	0.088	4.6E-01
Mtr.41140.1.S1_at	10.36	10.41	11.13	10.74	11.22	10.77	10.63	0.43	10.91	0.27	1.03	0.037	4.0E-01
Mtr.40469.1.S1_at	12.06	11.20	11.71	11.78	11.72	11.75	11.66	0.43	11.75	0.03	1.01	0.012	7.2E-01
Mtr.9970.1.S1_at	9.27	8.47	9.15	9.36	9.71	9.41	8.96	0.43	9.49	0.19	1.06	0.083	1.2E-01
Mtr.42524.1.S1_at	9.42	8.87	9.72	9.46	9.53	9.50	9.34	0.43	9.50	0.04	1.02	0.025	5.6E-01
Mtr.46313.1.S1_at	2.19	2.93	2.18	2.17	2.17	2.17	2.43	0.43	2.17	0.00	0.89	-0.164	3.5E-01
Mtr.1306.1.S1_at	3.41	4.16	3.42	3.39	3.12	3.40	3.66	0.43	3.30	0.16	0.90	-0.150	2.5E-01
Mtr.14024.1.S1_at	10.04	9.18	9.64	9.74	9.58	9.97	9.62	0.43	9.76	0.20	1.01	0.021	6.3E-01
Mtr.45295.1.S1_s_at	10.10	10.34	10.94	10.54	10.54	10.44	10.46	0.43	10.51	0.06	1.00	0.006	8.7E-01
Mtr.29256.1.S1_at	4.11	3.34	4.07	4.05	4.05	4.02	3.84	0.43	4.04	0.02	1.05	0.074	4.6E-01
Mtr.44005.1.S1_at	12.15	12.26	12.95	12.33	12.10	12.34	12.46	0.43	12.26	0.14	0.98	-0.023	4.9E-01
Mtr.22171.1.S1_at	8.94	8.09	8.42	8.84	8.85	8.84	8.48	0.43	8.84	0.01	1.04	0.060	2.2E-01
Mtr.6794.1.S1_at	5.68	5.77	6.47	5.86	6.36	5.39	5.97	0.43	5.87	0.49	0.98	-0.026	7.9E-01
Mtr.42683.1.S1_at	11.58	10.76	10.94	11.33	10.96	11.47	11.09	0.43	11.25	0.26	1.01	0.021	6.1E-01
Mtr.12391.1.S1_at	3.21	2.49	2.44	2.40	2.42	3.04	2.71	0.43	2.62	0.37	0.97	-0.050	7.9E-01
Mtr.9666.1.S1_at	7.17	6.47	6.39	7.20	6.83	7.15	6.68	0.43	7.06	0.20	1.06	0.080	2.4E-01
Mtr.42456.1.S1_at	10.32	10.52	11.14	10.92	11.06	10.60	10.66	0.43	10.86	0.24	1.02	0.027	5.2E-01
Mtr.39969.1.S1_at	5.76	5.70	6.47	5.72	5.66	5.69	5.98	0.43	5.69	0.03	0.95	-0.072	3.1E-01
Mtr.47523.1.S1_at	2.75	3.48	2.71	2.71	2.70	2.71	2.98	0.43	2.70	0.01	0.91	-0.140	3.3E-01
Mtr.30368.1.S1_at	4.38	3.69	4.49	4.04	4.84	4.21	4.19	0.43	4.36	0.42	1.04	0.059	6.4E-01
Mtr.35724.1.S1_at	6.73	6.04	6.83	6.71	7.30	6.70	6.53	0.43	6.90	0.34	1.06	0.079	3.1E-01
Mtr.44878.1.S1_at	9.28	8.44	9.03	9.28	9.08	9.11	8.91	0.43	9.16	0.11	1.03	0.039	3.9E-01
Mtr.52213.1.S1_a_at	3.77	3.70	2.99	3.60	3.75	3.51	3.49	0.43	3.62	0.12	1.04	0.054	6.3E-01
Mtr.41774.1.S1_at	6.51	6.34	7.16	6.79	6.87	6.62	6.67	0.43	6.76	0.13	1.01	0.019	7.5E-01
Mtr.42717.1.S1_at	7.95	7.45	8.31	8.05	8.18	8.05	7.90	0.43	8.09	0.07	1.02	0.034	4.9E-01
Mtr.21515.1.S1_at	11.65	10.79	11.29	10.90	11.20	11.01	11.25	0.43	11.04	0.15	0.98	-0.027	4.7E-01
Mtr.17558.1.S1_s_at	10.04	9.23	9.90	10.09	9.99	10.15	9.72	0.43	10.07	0.08	1.04	0.051	2.4E-01
Mtr.9037.1.S1_at	10.98	10.56	10.11	10.83	10.93	10.92	10.55	0.43	10.89	0.06	1.03	0.046	2.4E-01
Mtr.52113.1.S1_at	3.83	3.83	3.09	3.79	3.34	4.10	3.58	0.43	3.74	0.38	1.04	0.062	6.6E-01
Mtr.18575.1.S1_at	4.60	3.94	4.74	4.92	4.80	4.76	4.43	0.43	4.83	0.08	1.09	0.125	1.9E-01
Mtr.5470.1.S1_x_at	3.82	4.29	3.43	3.74	3.63	3.72	3.84	0.43	3.70	0.06	0.96	-0.057	5.9E-01
Mtr.21035.1.S1_at	12.16	12.82	12.98	12.63	12.83	12.44	12.65	0.43	12.64	0.20	1.00	-0.002	9.6E-01
Mtr.31625.1.S1_at	7.98	7.48	7.12	7.25	7.17	7.19	7.53	0.43	7.20	0.04	0.96	-0.063	2.7E-01
Mtr.50456.1.S1_at	2.50	3.24	2.48	2.49	2.46	2.48	2.74	0.43	2.47	0.01	0.90	-0.146	3.5E-01
Mtr.1600.1.S1_at	2.86	3.70	3.09	3.08	3.08	3.70	3.21	0.43	3.29	0.36	1.02	0.032	8.3E-01
Mtr.38741.1.S1_at	9.94	9.09	9.66	9.64	9.73	9.56	9.56	0.43	9.64	0.08	1.01	0.012	7.7E-01
Mtr.3299.1.S1_at	3.06	3.78	3.01	3.03	2.99	3.26	3.29	0.43	3.09	0.15	0.94	-0.088	5.0E-01
Mtr.44817.1.S1_at	8.22	9.01	8.92	8.48	8.88	8.42	8.72	0.43	8.59	0.25	0.99	-0.021	6.8E-01
Mtr.20627.1.S1_x_at	2.84	3.62	2.91	2.92	2.84	2.89	3.12	0.43	2.88	0.04	0.92	-0.114	4.0E-01
Mtr.37975.1.S1_at	10.48	9.92	10.77	10.56	10.72	10.25	10.39	0.43	10.51	0.24	1.01	0.016	7.1E-01
Mtr.8227.1.S1_at	10.21	9.43	10.14	9.67	9.95	9.87	9.93	0.43	9.83	0.14	0.99	-0.014	7.3E-01
Mtr.320.1.S1_x_at	7.92	8.74	8.54	8.21	8.54	8.35	8.40	0.43	8.37	0.17	1.00	-0.005	9.1E-01
Mtr.32728.1.S1_s_at	7.11	6.49	7.32	7.01	7.18	6.57	6.97	0.43	6.92	0.32	0.99	-0.011	8.7E-01
Mtr.46435.1.S1_at	13.32	13.93	14.16	13.61	13.51	13.52	13.80	0.43	13.55	0.06	0.98	-0.027	3.7E-01
Mtr.44491.1.S1_at	8.24	7.57	8.38	8.22	8.13	8.22	8.06	0.43	8.19	0.05	1.02	0.023	6.4E-01
Mtr.27639.1.S1_at	7.46	6.63	7.25	7.84	8.43	7.98	7.12	0.43	8.08	0.31	1.14	0.184	3.4E-02
Mtr.25825.1.S1_at	2.74	3.47	2.71	2.72	2.69	2.68	2.97	0.43	2.70	0.02	0.91	-0.140	3.3E-01
Mtr.31104.1.S1_at	7.48	6.74	7.50	7.29	7.29	7.28	7.24	0.43	7.29	0.00	1.01	0.009	8.6E-01
Mtr.49114.1.S1_at	4.26	3.47	3.58	3.50	3.65	3.16	3.77	0.43	3.43	0.25	0.91	-0.134	3.1E-01
Mtr.41865.1.S1_at	6.34	7.15	6.50	6.77	6.88	6.68	6.66	0.43	6.78	0.10	1.02	0.024	6.8E-01
Mtr.45332.1.S1_at	11.64	10.81	11.04	11.23	11.26	11.26	11.16	0.43	11.25	0.02	1.01	0.011	7.5E-01
Mtr.28258.1.S1_at	3.10	3.82	3.05	3.02	2.94	2.93	3.32	0.43	2.96	0.05	0.89	-0.165	2.2E-01
Mtr.11670.1.S1_s_at	3.08	2.27	2.41	2.49	2.28	2.22	2.59	0.43	2.33	0.14	0.90	-0.149	3.9E-01
Mtr.34127.1.S1_at	3.93	3.38	3.09	3.21	3.41	3.12	3.47	0.43	3.25	0.15	0.94	-0.094	4.5E-01
Mtr.24490.1.S1_at	13.34	13.72	14.20	13.84	14.26	13.68	13.75	0.43	13.93	0.30	1.01	0.018	5.9E-01
Mtr.1020.1.S1_at	2.35	2.37	3.11	2.39	2.16	2.35	2.61	0.43	2.30	0.12	0.88	-0.183	2.9E-01
Mtr.39425.1.S1_s_at	4.14	4.15	4.89	4.38	4.37	5.08	4.40	0.43	4.61	0.41	1.05	0.068	5.7E-01
Mtr.14980.1.S1_s_at	3.67	4.40	3.64	3.63	3.74	3.57	3.90	0.43	3.65	0.08	0.93	-0.098	3.7E-01
Mtr.42828.1.S1_at	9.67	8.84	9.04	9.20	9.24	9.36	9.19	0.43	9.27	0.08	1.01	0.013	7.6E-01
Mtr.34948.1.S1_at	8.04	7.68	7.18	7.44	7.57	7.46	7.63	0.43	7.49	0.07	0.98	-0.027	6.0E-01
Mtr.48808.1.S1_x_at	1.81	2.55	1.80	1.80	1.79	1.80	2.05	0.43	1.80	0.00	0.88	-0.192	3.6E-01
Mtr.5631.1.S1_s_at	6.31	5.47	6.03	6.18	6.05	6.08	5.94	0.43	6.10	0.07	1.03	0.040	5.4E-01
Mtr.18288.1.S1_x_at	2.53	3.26	2.50	2.48	2.56	2.46	2.76	0.43	2.50	0.05	0.90	-0.144	3.5E-01
Mtr.38301.1.S1_at	9.33	9.75	10.19	9.84	9.98	9.86	9.76	0.43	9.89	0.08	1.01	0.020	6.2E-01
Mtr.42535.1.S1_at	8.06	7.21	7.52	8.19	7.94	8.11	7.60	0.43	8.08	0.13	1.06	0.089	1.4E-01
Mtr.4387.1.S1_at	7.31	6.48	7.10	7.44	7.23	7.20	6.96	0.43	7.29	0.13	1.05	0.067	2.7E-01
Mtr.20493.1.S1_at	3.47	3.80	4.32	3.41	3.30	3.69	3.86	0.43	3.47	0.20	0.90	-0.155	2.2E-01
Mtr.44192.1.S1_a_at	11.29	10.44	10.98	10.91	11.16	10.90	10.90	0.43	10.99	0.15	1.01	0.012	7.5E-01
Mtr.19911.1.S1_at	3.49	2.64	2.99	2.49	2.49	2.49	3.04	0.43	2.49	0.01	0.82	-0.287	9.2E-02
Mtr.13705.1.S1_at	8.58	9.31	9.34	8.96	8.90	9.02	9.08	0.43	8.96	0.06	0.99	-0.019	6.6E-01
Mtr.44961.1.S1_at	9.62	8.79	9.43	9.36	9.31	9.35	9.28	0.43	9.34	0.02	1.01	0.010	8.2E-01

Mtr.1612.1.S1_at	3.01	3.75	3.01	2.99	2.96	2.96	3.26	0.43	2.97	0.02	0.91	-0.134	3.1E-01
Mtr.10163.1.S1_at	9.20	8.60	9.43	9.12	9.16	9.10	9.08	0.43	9.13	0.03	1.01	0.008	8.5E-01
Mtr.44176.1.S1_at	7.25	7.05	7.88	7.47	7.40	7.17	7.39	0.43	7.35	0.16	0.99	-0.009	8.8E-01
Mtr.43749.1.S1_s_at	8.16	7.44	8.20	7.54	7.68	7.67	7.93	0.43	7.63	0.08	0.96	-0.057	2.9E-01
Mtr.17970.1.S1_at	3.12	3.81	3.02	3.00	2.98	2.99	3.31	0.43	2.99	0.01	0.90	-0.148	2.6E-01
Mtr.14489.1.S1_at	7.82	7.92	7.13	7.98	7.44	7.78	7.62	0.43	7.73	0.27	1.01	0.021	7.2E-01
Mtr.24258.1.S1_at	8.27	7.41	7.91	7.38	7.58	7.35	7.86	0.43	7.44	0.13	0.95	-0.080	1.8E-01
Mtr.42235.1.S1_at	10.26	11.10	10.82	10.31	10.70	10.46	10.73	0.43	10.49	0.20	0.98	-0.033	4.3E-01
Mtr.6111.1.S1_s_at	7.72	6.90	7.54	7.64	7.48	7.65	7.39	0.43	7.59	0.09	1.03	0.039	4.6E-01
Mtr.33288.1.S1_s_at	7.90	7.06	7.30	7.60	7.68	7.68	7.42	0.43	7.65	0.04	1.03	0.045	4.0E-01
Mtr.39094.1.S1_at	6.95	6.37	7.21	7.66	8.28	7.86	6.84	0.43	7.93	0.31	1.16	0.213	2.4E-02
Mtr.4056.1.S1_at	11.78	10.96	11.59	11.62	11.62	11.62	11.44	0.43	11.62	0.00	1.02	0.022	5.2E-01
Mtr.4555.1.S1_at	3.51	2.90	2.68	2.94	2.59	2.57	3.03	0.43	2.70	0.21	0.89	-0.165	3.0E-01
Mtr.40599.1.S1_at	10.92	10.08	10.65	10.66	10.64	10.53	10.55	0.43	10.61	0.07	1.01	0.009	8.1E-01
Mtr.12355.1.S1_at	13.95	13.26	14.04	13.75	13.84	13.67	13.75	0.43	13.75	0.09	1.00	0.000	1.0E+00
Mtr.11158.1.S1_at	6.60	5.93	6.73	6.54	6.31	6.46	6.42	0.43	6.44	0.12	1.00	0.004	9.4E-01
Mtr.39248.1.S1_at	8.68	8.11	8.95	8.47	8.60	8.55	8.58	0.43	8.54	0.06	1.00	-0.007	8.7E-01
Mtr.13601.1.S1_at	8.41	8.07	8.92	8.52	8.68	8.41	8.47	0.43	8.54	0.13	1.01	0.012	7.9E-01
Mtr.31199.1.S1_s_at	12.42	12.10	11.57	11.85	11.90	12.05	12.03	0.43	11.93	0.10	0.99	-0.011	7.3E-01
Mtr.31243.1.S1_at	8.58	7.86	8.63	8.73	8.73	8.69	8.36	0.43	8.72	0.03	1.04	0.061	2.2E-01
Mtr.13477.1.S1_at	9.61	9.05	9.89	9.68	9.67	9.62	9.51	0.43	9.66	0.03	1.01	0.021	6.0E-01
Mtr.42943.1.S1_at	9.66	8.88	8.96	9.29	9.45	9.45	9.17	0.43	9.40	0.10	1.03	0.036	4.1E-01
Mtr.8223.1.S1_at	2.61	3.31	2.53	2.60	2.60	2.61	2.82	0.43	2.60	0.01	0.92	-0.117	4.3E-01
Mtr.39438.1.S1_s_at	8.61	8.08	8.93	9.08	9.26	9.22	8.54	0.43	9.19	0.10	1.08	0.106	6.3E-02
Mtr.29443.1.S1_s_at	10.02	10.61	10.85	10.40	10.39	10.54	10.49	0.43	10.44	0.09	1.00	-0.007	8.6E-01
Mtr.3442.1.S1_at	7.64	6.79	7.33	7.15	7.07	6.97	7.26	0.43	7.06	0.09	0.97	-0.039	4.9E-01
Mtr.727.1.S1_at	8.08	7.60	7.22	7.51	7.75	8.18	7.64	0.43	7.81	0.34	1.02	0.033	6.1E-01
Mtr.6930.1.S1_at	8.00	7.19	7.34	7.85	7.80	8.16	7.51	0.43	7.94	0.19	1.06	0.079	1.9E-01
Mtr.42824.1.S1_at	3.64	3.22	2.78	3.22	2.97	2.97	3.21	0.43	3.05	0.15	0.95	-0.072	5.8E-01
Mtr.40508.1.S1_at	10.47	10.40	11.17	10.62	10.72	10.40	10.68	0.43	10.58	0.16	0.99	-0.014	7.3E-01
Mtr.26801.1.S1_a_at	8.53	9.07	9.38	8.86	8.98	8.64	9.00	0.43	8.83	0.17	0.98	-0.027	5.6E-01
Mtr.42927.1.S1_at	9.44	10.04	10.27	9.92	10.10	9.95	9.91	0.43	9.99	0.10	1.01	0.012	7.7E-01
Mtr.20875.1.S1_at	3.02	2.31	2.25	2.25	2.24	2.25	2.53	0.43	2.24	0.01	0.89	-0.172	3.2E-01
Mtr.772.1.S1_at	3.34	4.14	3.47	3.44	3.39	3.39	3.65	0.43	3.41	0.03	0.93	-0.098	3.9E-01
Mtr.1530.1.S1_at	4.72	3.98	4.73	4.35	4.21	4.31	4.48	0.43	4.29	0.07	0.96	-0.062	5.0E-01
Mtr.37207.1.S1_at	9.51	8.70	9.34	9.38	9.36	9.56	9.18	0.43	9.43	0.11	1.03	0.039	3.8E-01
Mtr.11439.1.S1_s_at	7.93	7.13	7.81	7.86	7.80	7.91	7.62	0.43	7.86	0.05	1.03	0.044	4.0E-01
Mtr.15850.1.S1_at	14.18	14.49	15.03	14.66	14.75	14.30	14.57	0.43	14.57	0.24	1.00	0.000	9.9E-01
Mtr.48019.1.S1_at	2.43	3.14	2.36	2.34	2.37	2.87	2.65	0.43	2.53	0.30	0.96	-0.065	7.2E-01
Mtr.32870.1.S1_at	7.55	7.45	8.24	8.13	8.40	8.00	7.75	0.43	8.17	0.20	1.05	0.077	2.0E-01
Mtr.52259.1.S1_s_at	10.53	9.69	9.96	10.23	10.11	10.19	10.06	0.43	10.17	0.06	1.01	0.016	6.7E-01
Mtr.42837.1.S1_at	9.98	9.91	10.69	10.01	10.02	9.91	10.19	0.43	9.98	0.06	0.98	-0.031	4.4E-01
Mtr.6279.1.S1_at	7.93	7.45	8.30	8.35	8.33	8.17	7.89	0.43	8.28	0.10	1.05	0.069	2.0E-01
Mtr.35638.1.S1_at	7.91	8.72	8.56	7.90	8.32	8.00	8.40	0.43	8.08	0.22	0.96	-0.057	3.1E-01
Mtr.20646.1.S1_s_at	9.88	9.27	10.10	9.74	9.47	9.76	9.75	0.43	9.66	0.16	0.99	-0.013	7.5E-01
Mtr.17539.1.S1_x_at	2.63	3.36	2.60	2.59	2.55	2.56	2.86	0.43	2.57	0.02	0.90	-0.156	3.0E-01
Mtr.8466.1.S1_at	14.67	13.88	13.97	13.95	13.69	13.88	14.17	0.43	13.84	0.13	0.98	-0.034	2.7E-01
Mtr.17268.1.S1_at	11.44	12.30	11.90	11.72	11.84	11.60	11.88	0.43	11.72	0.12	0.99	-0.019	5.7E-01
Mtr.36133.1.S1_at	6.11	6.55	5.69	5.84	5.86	6.03	6.12	0.43	5.91	0.11	0.97	-0.050	4.6E-01
Mtr.14440.1.S1_at	10.13	9.31	9.94	10.02	9.79	9.84	9.80	0.43	9.88	0.13	1.01	0.013	7.6E-01
Mtr.5307.1.S1_at	2.68	3.39	2.63	2.62	2.63	2.59	2.90	0.43	2.61	0.02	0.90	-0.151	3.1E-01
Mtr.34491.1.S1_at	8.75	7.95	8.61	8.22	8.40	8.33	8.44	0.43	8.32	0.09	0.99	-0.021	6.6E-01
Mtr.20780.1.S1_at	8.16	7.34	7.94	8.01	8.06	8.03	7.81	0.43	8.04	0.02	1.03	0.041	4.2E-01
Mtr.9885.1.S1_at	9.65	9.03	9.86	9.24	9.65	9.02	9.51	0.43	9.31	0.32	0.98	-0.032	5.4E-01
Mtr.32455.1.S1_s_at	5.29	4.61	5.40	5.63	5.08	5.03	5.10	0.43	5.25	0.34	1.03	0.042	6.5E-01
Mtr.39484.1.S1_at	9.29	8.68	9.51	9.22	9.36	9.12	9.16	0.43	9.23	0.12	1.01	0.011	7.9E-01
Mtr.46528.1.S1_at	7.02	7.83	7.16	7.59	6.80	7.26	7.34	0.43	7.22	0.40	0.98	-0.024	7.4E-01
Mtr.10889.1.S1_at	8.27	7.83	8.69	8.23	8.29	8.22	8.26	0.43	8.25	0.04	1.00	-0.003	9.5E-01
Mtr.45500.1.S1_at	7.81	7.03	7.74	7.59	7.87	7.65	7.53	0.43	7.70	0.15	1.02	0.033	5.4E-01
Mtr.28618.1.S1_at	5.89	5.66	6.49	6.38	6.10	5.79	6.01	0.43	6.09	0.30	1.01	0.018	8.1E-01
Mtr.11702.1.S1_at	8.00	7.28	8.05	8.11	7.96	8.01	7.77	0.43	8.03	0.07	1.03	0.046	3.7E-01
Mtr.44561.1.S1_at	9.53	8.79	9.54	9.44	9.64	9.53	9.29	0.43	9.53	0.10	1.03	0.038	3.9E-01
Mtr.34772.1.S1_at	8.38	7.55	8.15	8.26	7.95	7.75	8.03	0.43	7.99	0.26	0.99	-0.008	8.9E-01
Mtr.2710.1.S1_at	8.62	8.10	8.95	8.53	8.35	8.57	8.56	0.43	8.48	0.12	0.99	-0.012	7.9E-01
Mtr.14166.1.S1_at	7.56	6.92	7.74	7.54	7.32	7.14	7.40	0.43	7.33	0.20	0.99	-0.014	8.0E-01
Mtr.45741.1.S1_at	8.57	9.42	9.13	9.28	9.49	9.55	9.04	0.43	9.44	0.14	1.04	0.062	2.0E-01
Mtr.33863.1.S1_at	8.94	8.54	9.40	9.09	9.19	8.82	8.96	0.43	9.03	0.19	1.01	0.012	8.0E-01
Mtr.12317.1.S1_at	10.97	11.17	11.79	11.31	11.58	11.40	11.31	0.43	11.43	0.14	1.01	0.015	6.7E-01
Mtr.9216.1.S1_at	9.76	8.98	9.69	9.68	9.67	9.68	9.48	0.43	9.68	0.01	1.02	0.030	4.6E-01
Mtr.27687.1.S1_at	9.19	9.83	9.02	9.27	9.27	9.28	9.35	0.43	9.27	0.01	0.99	-0.011	7.8E-01
Mtr.9036.1.S1_at	10.11	9.26	9.63	9.83	9.75	9.87	9.67	0.43	9.82	0.06	1.02	0.022	5.9E-01
Mtr.7010.1.S1_at	3.81	3.51	4.36	4.71	4.02	4.22	3.89	0.43	4.32	0.35	1.11	0.149	2.6E-01
Mtr.38816.1.S1_at	11.47	10.61	11.08	11.22	11.09	11.07	11.05	0.43	11.13	0.08	1.01	0.010	7.8E-01
Mtr.7459.1.S1_at	9.80	8.96	9.52	9.93	10.09	10.04	9.43	0.43	10.02	0.08	1.06	0.088	7.8E-02
Mtr.31712.1.S1_at	9.53	9.30	10.13	9.53	9.74	9.65	9.66	0.43	9.64	0.11	1.00	-0.002	9.6E-01
Mtr.7843.1.S1_at	3.70	4.44	3.69	3.38	3.69	3.42	3.94	0.43	3.50	0.17	0.89	-0.173	1.7E-01
Mtr.11255.1.S1_s_at	7.59	7.02	7.86	7.50	7.53	7.67	7.49	0.43	7.57	0.09	1.01	0.014	7.9E-01
Mtr.39502.1.S1_at	9.90	9.06	9.60	9.61	9.38	9.61	9.52	0.43	9.53	0.13	1.00	0.002	9.7E-01
Mtr.1652.1.S1_at	2.52	2.36	3.17	2.55	2.51	2.48	2.68	0.43	2.51	0.04	0.94	-0.095	5.3E-01
Mtr.25230.1.S1_at	2.51	3.24	2.49	2.48	2.47	2.46	2.75	0.43	2.47	0.01	0.90	-0.154	3.2E-01
Mtr.13238.1.S1_at	9.08	9.82	9.81	9.23	9.42	9.19	9.57	0.43	9.28	0.12	0.97	-0.044	3.2E-01
Mtr.40240.1.S1_s_at	9.48	10.24	9.52	9.08	9.71	9.44	9.75	0.43	9.41	0.31	0.97	-0.051	3.3E-01
Mtr.40277.1.S1_at	12.68	11.83	12.21	12.27	12.16	12.51	12.24	0.43	12.31	0.18	1.01	0.009	7.9E-01
Mtr.3101.1.S1_at	9.21	8.44	8.51	8.44	8.45	8.58	8.72	0.43	8.49	0.08	0.97	-0.038	4.1E-01
Mtr.39077.1.S1_at	9.68	9.61	10.38	10.19	10.31	10.21	9.89	0.43	10.24	0.06	1.04	0.050	2.3E-01
Mtr.18762.1.S1_at	4.02	3.45	3.18	3.31	3.39	3.47	3.55	0.43	3.39	0.08	0.96	-0.065	5.7E-01
Mtr.20370.1.S1_at	3.09	3.80	3.03	3.01	2.93	2.95	3.31	0.43	2.96	0.04	0.90	-0.158	2.4E-01
Mtr.25065.1.S1_s_at	9.37	8.54	9.13	9.19	9.02	9.20	9.02	0.43	9.14	0.10	1.01	0.020	6.5E-01
Mtr.15806.1.S1_s_at	2.86	3.56	2.80	2.77	2.71	2.75	3.07	0.43	2.74	0.03	0.89	-0.164	2.5E-01
Mtr.12595.1.S1_at	10.28	9.99	10.83	10.17	10.47	10.18	10.37	0.43	10.28	0.17	0.99	-0.013	7.4E-01
Mtr.26904.1.S1_at	3.21	3.05	3.85	3.17	4.22	3.84	3.37	0.43	3.75	0.53	1.11	0.152	4.0E-01
Mtr.48708.1.S1_x_at	3.32	4.00	3.21	3.19	3.16	3.36	3.51	0.43	3.24	0.11	0.92	-0.118	3.4E-01
Mtr.2493.1.S1_at	4.24	3.43	3.62	3.68	3.22	3.24	3.76	0.43	3.38	0.26	0.90	-0.155	2.6E-01
Mtr.42199.1.S1_at	11.83	11.74	12.52	12.21	12.28	11.97	12.03	0.43	12.15	0.16	1.01	0.015	6.6E-01
Mtr.34603.1.S1_s_at	11.89	11.24	12.04	11.79	12.04	11.88	11.72	0.43	11.91	0.13	1.02	0.023	5.1E-01
Mtr.35723.1.S1_at	9.68	8.89	9.58	9.63	9.79	9.53	9.38	0.43	9.65	0.13	1.03	0.041	3.6E-01
Mtr.20986.1.S1_s_at	2.21	2.94	2.20	2.20	2.19	2.19	2.45	0.43	2.19	0.00	0.90	-0.159	3.6E-01
Mtr.39742.1.S1_at	7.10	7.32	7.92	7.36	7.42	7.09	7.45	0.43	7.29	0.18	0.98	-0.030	5.9E-01
Mtr.1100.1.S1_at	2.54	3.25	2.49	2.48	2.41	2.52	2.76	0.43	2.47	0.06	0.90	-0.158	3.1E-01
Mtr.7296.1.S1_at	6.99	6.58	7.43	7.21	7.34	6.91	7.00	0.43	7.15	0.22	1.02	0.031	6.2E-01
Mtr.23129.1.S1_at	6.55	5.72	6.29	6.35	6.27	6.24	6.19	0.43	6.29	0.06	1.02	0.023	7.1E-01
Mtr.5823.1.S1_a_at	10.45	9.63	9.83	10.22	9.87	10.46	9.97	0.43	10.18	0.30	1.02	0.031	5.2E-01
Mtr.18927.1.S1_at	2.88	3.60	2.84	2.82	2.83	2.80	3.11	0.43	2.82	0.01	0.91	-0.141	3.1E-01
Mtr.42150.1.S1_at	8.12	8.67	8.96	8.71	8.63	8.58	8.58	0.43	8.64	0.07	1.01	0.009	8.4E-01
Mtr.35052.1.S1_at	7.45	6.60	7.01	7.56	7.47	7.70	7.02	0.43	7.58	0.11	1.08	0.110	9.4E-02
Mtr.11491.1.S1_at	10.18	9.68	10.52	10.25	10.36	10.22	10.13	0.43	10.28	0.07	1.02	0.022	5.8E-01
Mtr.42613.1.S1_at	9.26	10.09	9.52	9.33	9.19	9.41	9.62	0.43	9.31	0.11	0.97	-0.047	2.9E-01
Mtr.27519.1.S1_at	3.43	2.72	2.66	2.66	2.41	2.66	2.94	0.43	2.57	0.15	0.88	-0.189	2.4E-01
Mtr.44583.1.S1_at	9.50	8.65	9.17	9.62	9.94	9.66	9.11	0.43	9.74	0.18	1.07	0.097	7.7E-02
Mtr.2535.1.S1_at	10.04	9.22	9.81	10.08	9.78	9.79	9.69	0.43	9.88	0.17	1.02	0.029	5.0E-01
Mtr.20731.1.S1_at	8.82	8.10	8.86	8.66	8.73	8.53	8.59	0.43	8.64	0.10	1.01	0.008	8.7E-01
Mtr.6110.1.S1_s_at	10.37	9.52	9.99	9.97	10.24	10.12	9.96	0.43	10.11	0.14	1.02	0.022	5.9E-01
Mtr.4488.1.S1_at	10.22	9.37	9.89	9.69	9.70	9.58	9.83	0.43	9.66	0.07	0.98	-0.025	5.3E-01
Mtr.39772.1.S1_at	10.50	11.26	10.55	10.44	11.13	10.70	10.77	0.43	10.76	0.35	1.00	-0.002	9.7E-01
Mtr.17513.1.S1_s_at	12.25	11.47	11.55	11.92	11.89	12.02	11.76	0.43	11.94	0.07	1.02	0.023	4.9E-01
Mtr.8912.1.S1_at	2.16	2.90	2.16	2.15	2.14	2.13	2.41	0.43	2.14	0.01	0.89	-0.169	3.4E-01
Mtr.19255.1.S1_at	10.37	9.60	10.31	10.30	10.47	10.35	10.09	0.43	10.37	0.09	1.03	0.039	3.3E-01
Mtr.36181.1.S1_at	7.85	7.61	8.43	7.81	8.23	7.54	7.96	0.43	7.86	0.35	0.99	-0.019	7.6E-01
Mtr.15886.1.S1_at	12.01	11.20	11.84	11.90	11.89	11.76	11.69	0.43	11.85	0.08	1.01	0.020	5.5E-01
Mtr.47864.1.S1_s_at	10.46	9.63	9.87	9.97	10.10	9.89	9.99	0.43	9.99	0.11	1.00	0.000	1.0E+00
Mtr.35604.1.S1_at	10.22	9.47	10.19	10.19	10.31	10.12	9.96	0.43	10.21	0.10	1.02	0.035	3.8E-01
Mtr.44028.1.S1_at	13.87	13.21	13.08	13.29	13.28	13.40	13.39	0.43	13.32	0.07	1.00	-0.007	8.1E-01
Mtr.13880.1.S1_at	10.86	10.21	10.06	10.47	10.72	10.59	10.38	0.43	10.59	0.13	1.02	0.030	4.5E-01
Mtr.41917.1.S1_at	9.57	8.83	9.57	9.74	9.68	9.81	9.33	0.43	9.74	0.07	1.04	0.063	1.7E-01
Mtr.30047.1.S1_at	5.95	5.36	6.18	5.46	6.28	5.53	5.83	0.43	5.76	0.46	0.99	-0.018	8.5E-01
Mtr.33536.1.S1_at	9.12	8.33	9.00	8.95	8.85	8.92	8.82	0.43	8.91	0.05	1.01	0.015	7.3E-01
Mtr.29299.1.S1_at	3.50	3.52	4.25	3.12	3.94	3.30	3.76	0.43	3.45	0.43	0.92	-0.121	4.4E-01
Mtr.51698.1.S1_a_at	8.98	8.30	8.20	8.92	8.83	9.26	8.50	0.43	9.00	0.22	1.06	0.083	1.4E-01
Mtr.20185.1.S1_at	13.41	12.57	13.12	13.03	12.68	13.05	13.03	0.43	12.92	0.21	0.99	-0.013	7.0E-01
Mtr.45593.1.S1_at	11.13	11.76	11.94	11.43	11.54	11.37	11.61	0.43	11.45	0.09	0.99	-0.020	5.6E-01
Mtr.31240.1.S1_s_at	8.10	7.28	7.90	7.80	8.55	8.05	7.76	0.43	8.13	0.38	1.05	0.068	3.2E-01

Mtr.32344.1.S1_at	9.12	8.36	9.08	9.25	9.12	9.09	8.85	0.43	9.15	0.08	1.03	0.048	3.0E-01
Mtr.45000.1.S1_at	9.85	10.21	9.37	10.06	10.41	10.13	9.81	0.43	10.20	0.19	1.04	0.056	2.2E-01
Mtr.789.1.S1_at	8.68	7.85	8.09	8.38	8.47	8.50	8.21	0.43	8.45	0.06	1.03	0.042	3.8E-01
Mtr.47992.1.S1_at	9.47	8.63	9.17	9.27	9.22	9.27	9.09	0.43	9.26	0.03	1.02	0.026	5.4E-01
Mtr.25059.1.S1_at	10.81	11.00	11.62	11.49	11.41	11.38	11.14	0.43	11.43	0.06	1.03	0.036	3.1E-01
Mtr.3102.1.S1_at	7.22	6.52	7.28	7.20	7.13	7.20	7.00	0.43	7.18	0.04	1.02	0.036	5.2E-01
Mtr.48534.1.S1_at	11.53	10.69	11.21	11.21	11.24	11.51	11.15	0.43	11.32	0.17	1.02	0.022	5.5E-01
Mtr.29878.1.S1_at	7.95	8.00	8.71	7.92	8.06	7.86	8.22	0.43	7.95	0.10	0.97	-0.049	3.3E-01
Mtr.42055.1.S1_at	7.65	8.08	7.23	7.62	7.63	7.59	7.66	0.43	7.61	0.02	0.99	-0.008	8.6E-01
Mtr.47272.1.S1_at	8.91	9.62	9.68	9.22	8.99	8.96	9.40	0.43	9.06	0.14	0.96	-0.055	2.5E-01
Mtr.15772.1.S1_at	13.63	13.22	12.78	13.02	13.34	13.28	13.21	0.43	13.21	0.17	1.00	0.000	9.9E-01
Mtr.46426.1.S1_s_at	9.54	8.95	8.71	8.42	8.65	8.82	9.07	0.43	8.63	0.20	0.95	-0.071	1.9E-01
Mtr.10795.1.S1_at	8.15	7.73	8.58	8.41	8.70	8.24	8.16	0.43	8.45	0.23	1.04	0.051	3.5E-01
Mtr.39288.1.S1_at	9.23	8.38	8.73	9.10	9.03	8.83	8.78	0.43	8.99	0.14	1.02	0.034	4.6E-01
Mtr.16345.1.S1_s_at	9.70	10.27	10.53	10.33	10.16	10.17	10.16	0.43	10.22	0.09	1.01	0.008	8.3E-01
Mtr.10891.1.S1_at	9.99	10.57	10.82	10.45	10.60	10.39	10.46	0.42	10.48	0.11	1.00	0.003	9.4E-01
Mtr.39164.1.S1_at	8.49	8.45	9.21	8.36	8.60	8.38	8.72	0.42	8.44	0.14	0.97	-0.046	3.5E-01
Mtr.34440.1.S1_at	9.59	10.03	10.44	10.09	9.98	10.04	10.02	0.42	10.04	0.05	1.00	0.002	9.5E-01
Mtr.34131.1.S1_at	3.81	4.59	4.49	5.66	5.25	5.24	4.30	0.42	5.38	0.24	1.25	0.326	1.8E-02
Mtr.13856.1.S1_at	10.76	10.50	11.33	10.93	11.10	10.88	10.87	0.42	10.97	0.12	1.01	0.014	7.1E-01
Mtr.7071.1.S1_at	9.42	8.63	9.29	9.30	8.94	9.26	9.11	0.42	9.16	0.20	1.01	0.008	8.6E-01
Mtr.47284.1.S1_at	9.24	8.40	8.81	9.16	9.19	9.16	8.82	0.42	9.17	0.02	1.04	0.057	2.2E-01
Mtr.5726.1.S1_at	4.95	4.12	4.70	4.74	4.94	4.71	4.59	0.42	4.80	0.12	1.05	0.065	4.6E-01
Mtr.11041.1.S1_at	7.46	7.06	7.90	7.28	7.21	7.19	7.47	0.42	7.23	0.05	0.97	-0.049	3.7E-01
Mtr.6922.1.S1_at	11.05	11.75	11.82	10.88	11.01	10.93	11.54	0.42	10.94	0.07	0.95	-0.077	7.3E-02
Mtr.24057.1.S1_at	2.38	3.10	2.36	2.36	2.35	2.35	2.61	0.42	2.35	0.00	0.90	-0.151	3.5E-01
Mtr.12537.1.S1_at	9.93	9.42	10.27	9.79	10.06	9.87	9.87	0.42	9.91	0.14	1.00	0.005	9.0E-01
Mtr.36903.1.S1_at	7.45	6.71	7.44	7.36	7.66	7.03	7.20	0.42	7.35	0.31	1.02	0.029	6.5E-01
Mtr.37918.1.S1_at	14.93	14.12	14.33	14.52	14.43	14.50	14.46	0.42	14.48	0.05	1.00	0.002	9.3E-01
Mtr.40689.1.S1_at	10.94	10.44	11.28	10.63	10.94	10.64	10.89	0.42	10.74	0.18	0.99	-0.020	6.1E-01
Mtr.32308.1.S1_at	9.71	8.86	9.25	8.77	8.59	8.64	9.28	0.42	8.67	0.10	0.93	-0.098	7.3E-02
Mtr.22030.1.S1_at	7.64	6.83	7.00	7.43	7.22	7.67	7.16	0.42	7.44	0.22	1.04	0.056	3.6E-01
Mtr.23686.1.S1_at	8.95	8.47	9.31	9.31	9.21	9.07	8.91	0.42	9.20	0.12	1.03	0.046	3.2E-01
Mtr.13236.1.S1_at	9.97	9.31	10.10	10.10	10.09	9.89	9.79	0.42	10.03	0.12	1.02	0.034	4.1E-01
Mtr.36982.1.S1_at	3.28	2.48	2.63	2.48	2.62	2.62	2.80	0.42	2.58	0.08	0.92	-0.118	4.3E-01
Mtr.42374.1.S1_at	6.07	5.52	6.36	5.61	6.08	5.93	5.98	0.42	5.87	0.24	0.98	-0.026	7.2E-01
Mtr.15791.1.S1_at	3.08	3.87	3.22	5.27	3.72	5.00	3.39	0.42	4.67	0.83	1.38	0.460	7.7E-02
Mtr.12901.1.S1_at	9.57	8.75	9.34	9.32	9.25	9.33	9.22	0.42	9.30	0.04	1.01	0.012	7.6E-01
Mtr.26963.1.S1_at	8.13	7.31	7.92	7.99	8.04	7.90	7.79	0.42	7.98	0.07	1.02	0.035	4.8E-01
Mtr.2659.1.S1_at	7.88	7.24	8.05	8.37	8.27	8.22	7.72	0.42	8.29	0.08	1.07	0.102	8.6E-02
Mtr.29380.1.S1_at	10.08	9.26	9.49	10.02	10.31	10.17	9.61	0.42	10.17	0.15	1.06	0.081	9.8E-02
Mtr.22292.1.S1_at	3.02	3.75	3.02	3.00	2.92	2.99	3.26	0.42	2.97	0.04	0.91	-0.135	3.0E-01
Mtr.10441.1.S1_at	5.42	4.66	5.35	5.24	5.33	5.07	5.14	0.42	5.22	0.13	1.01	0.020	7.9E-01
Mtr.4869.1.S1_at	7.90	7.41	8.26	7.95	8.35	7.90	7.85	0.42	8.07	0.25	1.03	0.039	4.9E-01
Mtr.5179.1.S1_at	8.66	7.82	8.36	8.69	8.79	8.69	8.28	0.42	8.72	0.06	1.05	0.075	1.5E-01
Mtr.42317.1.S1_at	10.13	9.29	9.64	9.87	10.09	10.02	9.69	0.42	9.99	0.12	1.03	0.045	2.9E-01
Mtr.34739.1.S1_at	7.34	6.69	7.48	7.47	7.30	7.36	7.17	0.42	7.38	0.09	1.03	0.041	4.5E-01
Mtr.38977.1.S1_at	7.53	6.87	7.66	7.75	7.49	7.91	7.35	0.42	7.72	0.22	1.05	0.070	2.6E-01
Mtr.11543.1.S1_at	8.73	8.09	7.93	8.57	8.63	8.39	8.25	0.42	8.53	0.12	1.03	0.048	3.3E-01
Mtr.44443.1.S1_at	8.93	8.09	8.63	8.76	8.88	8.60	8.55	0.42	8.75	0.14	1.02	0.032	4.9E-01
Mtr.43513.1.S1_at	9.78	10.59	10.40	11.26	11.81	11.51	10.26	0.42	11.53	0.28	1.12	0.169	1.2E-02
Mtr.17680.1.S1_x_at	3.20	3.91	3.15	3.19	3.07	3.19	3.42	0.42	3.15	0.07	0.92	-0.119	3.4E-01
Mtr.20076.1.S1_at	2.97	3.68	2.93	2.95	2.93	2.86	3.19	0.42	2.91	0.05	0.91	-0.133	3.2E-01
Mtr.28493.1.S1_at	4.39	5.03	5.20	5.04	5.00	5.09	4.87	0.42	5.04	0.05	1.03	0.048	5.4E-01
Mtr.31557.1.S1_at	7.51	8.10	8.33	7.98	8.14	7.82	7.98	0.42	7.98	0.16	1.00	0.000	1.0E+00
Mtr.25490.1.S1_at	9.76	9.16	9.97	9.71	9.62	9.63	9.63	0.42	9.65	0.05	1.00	0.004	9.3E-01
Mtr.43937.1.S1_at	12.21	12.61	13.05	12.54	12.85	12.82	12.62	0.42	12.74	0.17	1.01	0.013	6.8E-01
Mtr.51147.1.S1_at	2.20	2.93	2.19	2.19	2.19	2.19	2.44	0.42	2.19	0.00	0.90	-0.156	3.6E-01
Mtr.5089.1.S1_at	7.81	7.14	7.93	7.73	8.47	7.82	7.63	0.42	8.01	0.40	1.05	0.069	3.3E-01
Mtr.41050.1.S1_at	9.53	9.34	10.15	9.99	10.06	9.62	9.67	0.42	9.89	0.24	1.02	0.032	4.8E-01
Mtr.16647.1.S1_at	3.10	3.80	3.04	3.02	2.95	3.11	3.32	0.42	3.02	0.08	0.91	-0.134	3.0E-01
Mtr.44474.1.S1_at	8.87	8.91	9.62	9.09	9.27	9.10	9.14	0.42	9.15	0.10	1.00	0.003	9.5E-01
Mtr.44977.1.S1_at	11.79	11.76	12.51	12.11	12.14	11.96	12.02	0.42	12.07	0.10	1.00	0.006	8.5E-01
Mtr.10219.1.S1_at	7.57	7.27	6.73	7.23	7.04	7.25	7.19	0.42	7.18	0.11	1.00	-0.003	9.6E-01
Mtr.25168.1.S1_at	4.85	4.14	4.89	5.12	5.53	4.83	4.63	0.42	5.16	0.35	1.11	0.157	1.7E-01
Mtr.40276.1.S1_at	6.62	6.27	7.11	8.00	8.16	8.42	6.67	0.42	8.20	0.21	1.23	0.298	5.0E-03
Mtr.5351.1.S1_at	2.70	3.42	2.67	2.67	2.63	2.87	2.93	0.42	2.72	0.13	0.93	-0.106	4.6E-01
Mtr.29054.1.S1_at	3.38	2.58	3.21	3.05	3.05	3.04	3.05	0.42	3.05	0.00	1.00	-0.004	9.7E-01
Mtr.45179.1.S1_at	10.53	9.73	10.38	10.06	9.89	9.93	10.21	0.42	9.96	0.09	0.98	-0.036	3.7E-01
Mtr.11082.1.S1_at	9.41	9.85	10.26	10.09	9.96	9.88	9.84	0.42	9.98	0.10	1.01	0.020	6.2E-01
Mtr.32327.1.S1_at	8.98	8.14	8.47	9.02	8.94	8.98	8.53	0.42	8.98	0.04	1.05	0.075	1.4E-01
Mtr.28074.1.S1_s_at	7.04	6.70	7.54	7.56	7.17	7.14	7.09	0.42	7.29	0.24	1.03	0.039	5.2E-01
Mtr.15738.1.S1_at	6.83	6.00	6.26	6.67	6.74	6.79	6.36	0.42	6.73	0.06	1.06	0.081	2.1E-01
Mtr.44656.1.S1_at	9.74	8.93	9.55	9.58	9.59	9.58	9.41	0.42	9.58	0.00	1.02	0.027	5.0E-01
Mtr.13314.1.S1_at	11.02	10.17	10.63	10.68	10.70	10.71	10.61	0.42	10.69	0.02	1.01	0.012	7.4E-01
Mtr.1439.1.S1_at	9.00	8.16	8.62	8.57	8.31	8.40	8.59	0.42	8.42	0.13	0.98	-0.029	5.5E-01
Mtr.38946.1.S1_at	7.57	6.74	7.28	7.63	7.33	7.45	7.20	0.42	7.47	0.15	1.04	0.053	3.5E-01
Mtr.10754.1.S1_at	8.75	8.09	8.88	8.48	8.74	8.70	8.57	0.42	8.64	0.14	1.01	0.011	8.1E-01
Mtr.44257.1.S1_s_at	8.93	8.09	8.40	8.26	7.65	7.81	8.48	0.42	7.91	0.32	0.93	-0.100	1.4E-01
Mtr.24067.1.S1_s_at	6.87	6.13	6.15	6.26	6.31	6.26	6.39	0.42	6.28	0.03	0.98	-0.025	6.8E-01
Mtr.48041.1.S1_at	2.87	3.58	2.83	2.82	2.79	2.76	3.09	0.42	2.79	0.03	0.90	-0.149	2.8E-01
Mtr.38306.1.S1_at	8.72	8.00	8.74	8.86	8.91	8.84	8.49	0.42	8.87	0.03	1.05	0.064	1.9E-01
Mtr.4364.1.S1_at	11.15	11.78	11.95	11.52	11.61	11.37	11.62	0.42	11.50	0.12	0.99	-0.016	6.5E-01
Mtr.28047.1.S1_at	2.97	3.63	2.85	3.60	4.20	3.89	3.15	0.42	3.90	0.30	1.24	0.308	6.7E-02
Mtr.19668.1.S1_at	5.25	4.50	5.20	5.95	5.64	5.64	4.98	0.42	5.75	0.18	1.15	0.205	4.5E-02
Mtr.29023.1.S1_at	4.52	3.79	4.52	4.49	4.63	4.60	4.28	0.42	4.57	0.07	1.07	0.096	3.0E-01
Mtr.41508.1.S1_at	6.60	6.36	7.18	6.85	6.71	6.43	6.71	0.42	6.66	0.21	0.99	-0.011	8.6E-01
Mtr.26378.1.S1_at	11.78	12.03	12.61	12.14	12.27	12.08	12.14	0.42	12.17	0.10	1.00	0.003	9.2E-01
Mtr.34977.1.S1_at	7.04	6.30	6.32	6.87	7.03	7.18	6.55	0.42	7.03	0.16	1.07	0.100	1.4E-01
Mtr.4938.1.S1_at	9.90	10.06	10.70	10.37	10.31	10.12	10.22	0.42	10.27	0.13	1.00	0.007	8.6E-01
Mtr.10567.1.S1_at	2.64	3.33	2.57	2.55	2.56	2.53	2.85	0.42	2.55	0.02	0.90	-0.159	2.9E-01
Mtr.10438.1.S1_at	15.14	14.32	14.58	14.53	15.12	14.97	14.68	0.42	14.87	0.31	1.01	0.019	5.6E-01
Mtr.9929.1.S1_at	8.72	9.28	8.46	8.71	8.56	8.85	8.82	0.42	8.71	0.15	0.99	-0.019	6.8E-01
Mtr.42619.1.S1_at	9.89	9.07	9.31	9.36	9.23	9.33	9.42	0.42	9.31	0.07	0.99	-0.017	6.7E-01
Mtr.7778.1.S1_at	3.17	3.86	3.10	3.08	3.02	3.04	3.38	0.42	3.05	0.03	0.90	-0.149	2.5E-01
Mtr.52223.1.S1_x_at	2.85	3.58	2.84	2.84	2.84	2.84	3.09	0.42	2.84	0.00	0.92	-0.123	3.6E-01
Mtr.43291.1.S1_at	10.37	9.64	9.63	9.59	9.78	9.81	9.88	0.42	9.73	0.12	0.98	-0.023	5.8E-01
Mtr.1782.1.S1_at	8.17	7.36	7.96	8.07	8.06	8.20	7.83	0.42	8.11	0.08	1.04	0.051	3.1E-01
Mtr.22369.1.S1_at	2.86	3.58	2.85	2.84	2.81	2.80	3.10	0.42	2.81	0.02	0.91	-0.137	3.1E-01
Mtr.28775.1.S1_at	8.44	8.09	8.93	8.85	8.71	8.55	8.48	0.42	8.70	0.15	1.03	0.037	4.4E-01
Mtr.9617.1.S1_at	8.85	9.21	8.37	8.08	8.82	8.70	8.81	0.42	8.53	0.39	0.97	-0.046	4.5E-01
Mtr.13894.1.S1_at	12.16	11.35	11.95	12.06	12.28	12.10	11.82	0.42	12.15	0.12	1.03	0.039	2.7E-01
Mtr.17816.1.S1_s_at	10.47	10.77	11.31	10.66	10.49	10.59	10.85	0.42	10.58	0.09	0.98	-0.037	3.4E-01
Mtr.18517.1.S1_at	6.74	7.58	7.26	6.41	6.78	6.34	7.19	0.42	6.51	0.24	0.90	-0.144	7.1E-02
Mtr.43264.1.S1_at	7.80	7.19	8.00	8.05	8.04	8.14	7.66	0.42	8.08	0.06	1.05	0.076	1.7E-01
Mtr.34572.1.S1_at	2.15	2.87	2.13	2.13	2.14	2.14	2.38	0.42	2.14	0.01	0.90	-0.157	3.7E-01
Mtr.10348.1.S1_at	8.93	8.19	8.20	7.89	7.84	7.83	8.44	0.42	7.86	0.03	0.93	-0.103	7.5E-02
Mtr.45192.1.S1_at	8.22	7.46	8.16	7.74	8.06	7.73	7.95	0.42	7.85	0.19	0.99	-0.019	7.1E-01
Mtr.41320.1.S1_at	5.69	5.45	6.27	5.75	6.03	6.01	5.80	0.42	5.93	0.16	1.02	0.031	6.5E-01
Mtr.40910.1.S1_at	10.60	9.80	10.41	10.35	10.40	10.48	10.27	0.42	10.41	0.07	1.01	0.019	6.0E-01
Mtr.40480.1.S1_at	10.87	10.07	10.69	10.74	10.86	10.74	10.54	0.42	10.78	0.07	1.02	0.032	4.0E-01
Mtr.30706.1.S1_at	10.54	9.75	9.90	9.93	10.00	9.86	10.06	0.42	9.93	0.07	0.99	-0.019	6.2E-01
Mtr.21549.1.S1_at	2.39	3.11	2.38	2.37	2.36	2.37	2.63	0.42	2.37	0.01	0.90	-0.151	3.4E-01
Mtr.20660.1.S1_at	2.17	2.89	2.16	2.15	2.15	2.14	2.41	0.42	2.15	0.00	0.89	-0.163	3.5E-01
Mtr.17857.1.S1_at	3.07	3.80	3.06	3.02	3.02	3.05	3.31	0.42	3.03	0.02	0.92	-0.127	3.2E-01
Mtr.44641.1.S1_at	4.27	4.75	5.11	6.78	7.35	7.08	4.71	0.42	7.07	0.29	1.50	0.586	1.3E-03
Mtr.22218.1.S1_x_at	2.54	3.25	2.49	2.52	2.50	2.47	2.76	0.42	2.50	0.02	0.90	-0.145	3.4E-01
Mtr.18570.1.S1_at	3.41	3.80	4.25	3.74	3.69	3.96	3.82	0.42	3.80	0.15	0.99	-0.008	9.4E-01
Mtr.24389.1.S1_at	3.04	3.75	3.01	2.99	2.92	2.91	3.27	0.42	2.94	0.04	0.90	-0.151	2.5E-01
Mtr.30109.1.S1_at	3.19	2.58	2.38	2.38	3.10	2.39	2.72	0.42	2.62	0.41	0.97	-0.050	8.0E-01
Mtr.45554.1.S1_at	8.07	7.46	8.26	7.94	8.14	7.81	7.93	0.42	7.96	0.17	1.00	0.007	9.0E-01
Mtr.37336.1.S1_at	10.83	10.99	11.62	11.13	11.11	11.00	11.15	0.42	11.08	0.07	0.99	-0.009	8.0E-01
Mtr.13197.1.S1_at	8.12	7.28	7.64	8.51	7.98	8.24	7.68	0.42	8.24	0.27	1.07	0.101	1.3E-01
Mtr.21969.1.S1_at	2.98	3.71	2.99	2.96	2.91	2.90	3.23	0.42	2.92	0.03	0.90	-0.144	2.8E-01
Mtr.45517.1.S1_at	5.33	5.23	4.55	5.35	5.55	5.86	5.04	0.42	5.58	0.26	1.11	0.148	1.3E-01
Mtr.46904.1.S1_x_at	3.45	4.29	3.93	3.32	3.43	2.82	3.89	0.42	3.19	0.32	0.82	-0.285	8.5E-02
Mtr.8921.1.S1_at	10.05	9.40	10.19	10.17	10.03	10.21	9.88	0.42	10.14	0.10	1.03	0.036	3.7E-01
Mtr.39829.1.S1_at	8.87	8.24	9.03	8.59	8.57	8.53	8.71	0.42	8.56	0.03	0.98	-0.026	5.6E-01
Mtr.51670.1.S1_at	11.92	11.23	11.16	11.43	11.56	11.51	11.44	0.42	11.50	0.07	1.01	0.008	8.1E-01
Mtr.8486.1.S1_at	8.58	7.75	8.25	8.83	8.64	8.75	8.19	0.42	8.74	0.09	1.07	0.094	9.2E-02
Mtr.44191.1.S1_at	10.55	9.89	10.67	10.38	10.65	10.37	10.37	0.42	10.47	0.16	1.01	0.013	7.3E-01

Mtr.1696.1.S1_at	2.75	3.47	2.74	2.73	2.71	2.69	2.99	0.42	2.71	0.02	0.91	-0.142	3.1E-01
Mtr.39371.1.S1_at	8.12	7.97	8.76	9.28	9.25	9.26	8.28	0.42	9.26	0.01	1.12	0.161	1.6E-02
Mtr.32872.1.S1_at	3.40	2.87	2.57	2.57	2.58	2.56	2.95	0.42	2.57	0.01	0.87	-0.196	2.0E-01
Mtr.26564.1.S1_at	2.71	3.43	2.70	3.07	2.56	2.68	2.95	0.42	2.77	0.27	0.94	-0.090	5.7E-01
Mtr.3689.1.S1_at	6.86	6.31	7.14	6.68	7.24	6.91	6.77	0.42	6.94	0.28	1.03	0.036	5.9E-01
Mtr.41405.1.S1_at	12.42	11.70	11.68	11.83	12.14	12.29	11.93	0.42	12.09	0.24	1.01	0.019	6.1E-01
Mtr.2938.1.S1_at	2.40	3.12	2.38	2.38	2.38	2.38	2.63	0.42	2.38	0.00	0.90	-0.145	3.6E-01
Mtr.18179.1.S1_at	6.29	6.90	6.10	6.32	6.78	6.51	6.43	0.42	6.53	0.23	1.02	0.023	7.2E-01
Mtr.40735.1.S1_at	8.70	7.98	8.70	8.81	8.85	9.04	8.46	0.42	8.90	0.12	1.05	0.073	1.6E-01
Mtr.20469.1.S1_at	2.94	3.65	2.90	2.89	2.89	2.89	3.17	0.42	2.89	0.00	0.91	-0.130	3.2E-01
Mtr.18791.1.S1_at	3.00	3.71	2.98	2.97	2.96	3.01	3.23	0.42	2.98	0.03	0.92	-0.115	3.7E-01
Mtr.12865.1.S1_at	9.66	8.98	9.74	9.40	9.81	9.37	9.46	0.42	9.52	0.25	1.01	0.010	8.3E-01
Mtr.19396.1.S1_at	5.39	4.58	5.20	5.15	5.17	5.21	5.06	0.42	5.18	0.03	1.02	0.034	6.5E-01
Mtr.14493.1.S1_x_at	4.84	5.65	5.45	5.67	5.39	5.22	5.32	0.42	5.43	0.23	1.02	0.030	7.1E-01
Mtr.4471.1.S1_at	10.30	9.57	10.29	10.13	10.14	10.15	10.06	0.42	10.14	0.01	1.01	0.012	7.4E-01
Mtr.9304.1.S1_at	10.44	9.60	10.05	10.65	10.58	10.64	10.03	0.42	10.63	0.04	1.06	0.083	7.1E-02
Mtr.38356.1.S1_s_at	5.49	4.66	4.98	5.59	5.39	5.32	5.04	0.42	5.43	0.14	1.08	0.107	2.0E-01
Mtr.33527.1.S1_at	8.52	8.13	8.97	8.78	8.74	8.75	8.54	0.42	8.76	0.02	1.03	0.036	4.3E-01
Mtr.33382.1.S1_at	7.50	6.69	7.31	7.51	7.65	7.83	7.17	0.42	7.66	0.17	1.07	0.097	1.3E-01
Mtr.11407.1.S1_at	10.01	9.46	10.29	9.90	9.83	9.94	9.92	0.42	9.89	0.06	1.00	-0.004	9.1E-01
Mtr.45668.1.S1_at	8.35	7.51	7.90	8.11	7.86	7.95	7.92	0.42	7.97	0.13	1.01	0.010	8.3E-01
Mtr.40069.1.S1_at	14.75	14.39	13.91	14.96	14.74	14.99	14.35	0.42	14.89	0.14	1.04	0.054	9.9E-02
Mtr.40747.1.S1_at	11.75	10.92	11.39	11.57	11.64	11.68	11.35	0.42	11.63	0.05	1.02	0.035	3.2E-01
Mtr.20587.1.S1_at	2.83	3.54	2.81	2.79	2.72	2.79	3.06	0.42	2.77	0.04	0.91	-0.143	3.0E-01
Mtr.45011.1.S1_at	8.81	8.03	8.68	8.72	8.76	8.72	8.51	0.42	8.73	0.02	1.03	0.038	4.0E-01
Mtr.44882.1.S1_at	4.19	3.45	3.49	3.61	3.36	3.49	3.71	0.42	3.49	0.12	0.94	-0.089	4.3E-01
Mtr.24914.1.S1_at	8.21	7.86	8.69	8.47	8.34	8.49	8.25	0.42	8.44	0.08	1.02	0.031	5.0E-01
Mtr.2001.1.S1_s_at	8.53	7.88	8.67	8.70	8.49	8.33	8.36	0.42	8.51	0.19	1.02	0.024	6.2E-01
Mtr.36931.1.S1_at	8.89	8.18	8.93	8.98	9.10	9.06	8.67	0.42	9.05	0.06	1.04	0.062	2.0E-01
Mtr.4391.1.S1_at	2.53	2.54	3.26	2.52	2.64	2.51	2.77	0.42	2.56	0.07	0.92	-0.118	4.3E-01
Mtr.3068.1.S1_at	8.90	8.14	8.85	8.48	8.80	8.82	8.63	0.42	8.70	0.19	1.01	0.012	8.0E-01
Mtr.50257.1.S1_at	8.16	7.54	8.34	8.18	8.06	7.89	8.02	0.42	8.05	0.15	1.00	0.006	9.1E-01
Mtr.29352.1.S1_at	7.50	6.67	7.14	7.31	7.40	7.10	7.10	0.42	7.27	0.15	1.02	0.033	5.6E-01
Mtr.51983.1.S1_s_at	2.91	3.58	2.81	2.86	2.88	2.89	3.10	0.42	2.88	0.02	0.93	-0.107	4.1E-01
Mtr.32758.1.S1_at	2.90	3.59	2.83	2.82	2.78	2.80	3.11	0.42	2.80	0.02	0.90	-0.151	2.7E-01
Mtr.41522.1.S1_at	10.14	9.31	9.78	10.04	9.76	9.96	9.74	0.42	9.92	0.14	1.02	0.026	5.3E-01
Mtr.38808.1.S1_at	4.67	5.51	5.07	4.97	4.99	4.97	5.09	0.42	4.98	0.01	0.98	-0.031	6.8E-01
Mtr.15764.1.S1_at	3.00	3.75	3.05	3.13	2.87	3.23	3.26	0.42	3.08	0.19	0.94	-0.086	5.2E-01
Mtr.39055.1.S1_at	8.14	7.31	7.86	8.50	9.14	8.64	7.77	0.42	8.76	0.34	1.13	0.173	3.4E-02
Mtr.40079.1.S1_at	11.17	10.34	10.67	10.52	10.66	10.43	10.73	0.42	10.54	0.12	0.98	-0.026	4.9E-01
Mtr.30921.1.S1_at	2.71	3.41	2.66	2.64	2.58	2.59	2.92	0.42	2.60	0.03	0.89	-0.167	2.6E-01
Mtr.27624.1.S1_at	8.24	7.74	7.41	7.70	7.71	8.08	7.80	0.42	7.83	0.22	1.00	0.007	9.0E-01
Mtr.1749.1.S1_s_at	7.05	6.54	7.37	7.02	7.02	6.92	6.99	0.42	6.99	0.06	1.00	0.001	9.9E-01
Mtr.27601.1.S1_at	4.63	3.96	4.73	4.57	4.66	4.50	4.44	0.42	4.58	0.08	1.03	0.043	6.2E-01
Mtr.10370.1.S1_at	10.91	10.93	11.65	11.84	11.97	11.81	11.16	0.42	11.87	0.09	1.06	0.089	4.5E-02
Mtr.24887.1.S1_s_at	5.45	5.84	5.00	5.76	5.50	5.64	5.43	0.42	5.63	0.13	1.04	0.052	4.7E-01
Mtr.28741.1.S1_at	8.40	7.91	8.74	8.43	8.35	8.34	8.35	0.42	8.37	0.05	1.00	0.004	9.3E-01
Mtr.9529.1.S1_at	11.03	10.19	10.64	10.79	11.09	10.99	10.62	0.42	10.96	0.15	1.03	0.045	2.6E-01
Mtr.6536.1.S1_at	7.25	8.08	7.52	7.16	6.99	6.91	7.62	0.42	7.02	0.13	0.92	-0.117	7.9E-02
Mtr.50853.1.S1_s_at	9.76	8.96	9.58	9.95	9.97	10.21	9.44	0.42	10.04	0.14	1.06	0.090	7.6E-02
Mtr.39758.1.S1_at	9.53	8.88	9.66	9.58	9.53	9.63	9.35	0.42	9.58	0.05	1.02	0.034	4.1E-01
Mtr.10081.1.S1_at	4.82	4.08	4.79	4.77	4.74	5.51	4.56	0.42	5.01	0.43	1.10	0.135	2.7E-01
Mtr.1520.1.S1_at	2.71	2.42	3.24	2.32	3.04	2.64	2.79	0.42	2.67	0.36	0.96	-0.064	7.2E-01
Mtr.42196.1.S1_at	8.30	7.50	8.10	8.23	8.25	8.31	7.97	0.42	8.26	0.04	1.04	0.053	2.9E-01
Mtr.6277.1.S1_at	3.10	3.79	3.03	3.00	2.98	2.93	3.31	0.42	2.97	0.04	0.90	-0.156	2.4E-01
Mtr.6506.1.S1_at	4.35	3.62	4.35	3.92	4.12	4.13	4.11	0.42	4.06	0.12	0.99	-0.017	8.6E-01
Mtr.32740.1.S1_at	13.07	13.59	13.90	13.41	13.18	13.43	13.52	0.42	13.34	0.14	0.99	-0.019	5.2E-01
Mtr.27412.1.S1_at	9.01	8.19	8.72	8.90	8.62	8.60	8.64	0.42	8.71	0.17	1.01	0.011	8.1E-01
Mtr.3346.1.S1_at	5.96	5.80	6.59	5.55	5.96	5.88	6.11	0.42	5.80	0.22	0.95	-0.077	3.1E-01
Mtr.13755.1.S1_at	8.71	7.90	8.50	8.79	8.51	8.54	8.37	0.42	8.61	0.16	1.03	0.041	4.0E-01
Mtr.31706.1.S1_s_at	3.24	3.91	3.14	3.13	3.46	3.75	3.43	0.42	3.45	0.31	1.00	0.006	9.7E-01
Mtr.23986.1.S1_at	2.47	3.22	2.52	2.44	2.47	2.44	2.74	0.42	2.45	0.02	0.89	-0.161	3.0E-01
Mtr.8297.1.S1_at	7.91	7.35	8.16	6.60	8.64	7.99	7.81	0.42	7.75	1.04	0.99	-0.011	9.3E-01
Mtr.7344.1.S1_at	11.27	10.60	10.51	9.95	10.09	9.98	10.79	0.42	10.01	0.08	0.93	-0.109	3.3E-02
Mtr.5839.1.S1_at	8.18	7.51	8.28	7.97	7.97	7.98	7.99	0.42	7.97	0.01	1.00	-0.003	9.5E-01
Mtr.6109.1.S1_at	9.66	9.57	10.34	9.80	10.12	10.01	9.86	0.42	9.98	0.16	1.01	0.017	6.7E-01
Mtr.44016.1.S1_at	10.41	9.58	10.09	9.66	9.80	9.72	10.03	0.42	9.72	0.07	0.97	-0.044	2.9E-01
Mtr.1268.1.S1_at	8.39	8.23	9.02	8.73	8.61	8.50	8.55	0.42	8.62	0.11	1.01	0.011	8.1E-01
Mtr.42175.1.S1_at	9.42	9.01	9.84	9.49	9.64	9.47	9.43	0.42	9.53	0.10	1.01	0.016	6.9E-01
Mtr.10378.1.S1_at	3.31	4.14	3.63	3.24	3.29	3.29	3.69	0.42	3.27	0.03	0.89	-0.173	1.6E-01
Mtr.8622.1.S1_at	11.58	10.80	11.45	11.41	11.56	11.48	11.28	0.42	11.48	0.07	1.02	0.026	4.5E-01
Mtr.1450.1.S1_at	5.79	5.72	5.03	5.75	6.14	5.64	5.52	0.42	5.84	0.26	1.06	0.083	3.2E-01
Mtr.13093.1.S1_at	8.15	7.56	8.37	8.06	8.26	8.09	8.02	0.42	8.14	0.11	1.01	0.020	6.7E-01
Mtr.50462.1.S1_at	12.11	12.05	12.80	12.46	12.40	12.20	12.32	0.42	12.35	0.13	1.00	0.004	9.0E-01
Mtr.43252.1.S1_s_at	5.02	4.55	5.39	6.44	6.39	5.77	4.99	0.42	6.20	0.37	1.24	0.314	2.0E-02
Mtr.38767.1.S1_at	10.91	10.11	10.29	10.90	10.69	11.00	10.44	0.42	10.87	0.16	1.04	0.058	1.8E-01
Mtr.28022.1.S1_x_at	2.62	3.34	2.61	2.61	2.65	2.59	2.86	0.42	2.61	0.03	0.92	-0.128	3.7E-01
Mtr.45607.1.S1_s_at	13.54	14.18	14.33	13.84	14.49	13.99	14.02	0.42	14.11	0.34	1.01	0.010	7.8E-01
Mtr.50943.1.S1_s_at	8.66	7.83	8.29	8.47	8.18	8.38	8.26	0.42	8.34	0.15	1.01	0.015	7.6E-01
Mtr.16541.1.S1_at	2.59	3.31	2.58	2.57	2.57	2.56	2.82	0.42	2.57	0.00	0.91	-0.137	3.5E-01
Mtr.9798.1.S1_s_at	12.06	11.31	11.36	11.59	11.45	11.58	11.58	0.42	11.54	0.08	1.00	-0.004	8.9E-01
Mtr.24271.1.S1_x_at	2.96	3.65	2.91	2.92	2.85	2.84	3.17	0.42	2.87	0.04	0.90	-0.146	2.8E-01
Mtr.45090.1.S1_at	3.16	3.98	3.44	3.03	3.11	3.01	3.52	0.42	3.05	0.05	0.86	-0.209	1.2E-01
Mtr.32132.1.S1_at	3.94	3.87	4.62	5.28	5.76	4.85	4.14	0.42	5.30	0.45	1.28	0.355	3.2E-02
Mtr.42323.1.S1_at	8.50	8.32	9.12	8.81	8.86	8.55	8.65	0.42	8.74	0.16	1.01	0.016	7.3E-01
Mtr.6567.1.S1_at	7.37	6.67	7.42	7.16	7.38	7.65	7.15	0.42	7.40	0.25	1.03	0.048	4.3E-01
Mtr.44317.1.S1_at	7.71	7.32	8.15	7.51	7.55	7.35	7.73	0.42	7.47	0.11	0.97	-0.049	3.6E-01
Mtr.3117.1.S1_at	4.13	3.45	3.37	3.58	3.74	4.02	3.65	0.42	3.78	0.22	1.04	0.052	6.5E-01
Mtr.4884.1.S1_at	11.12	10.47	11.26	10.92	11.15	10.90	10.95	0.42	10.99	0.14	1.00	0.005	8.8E-01
Mtr.1818.1.S1_at	2.26	2.98	2.25	2.23	2.25	2.22	2.50	0.42	2.23	0.01	0.89	-0.160	3.4E-01
Mtr.3805.1.S1_at	6.47	5.74	6.46	6.13	5.96	6.11	6.22	0.42	6.07	0.09	0.98	-0.036	5.7E-01
Mtr.11874.1.S1_at	10.84	10.00	10.37	10.51	10.51	10.55	10.40	0.42	10.52	0.02	1.01	0.016	6.6E-01
Mtr.8652.1.S1_at	13.54	12.71	13.19	13.35	13.25	13.39	13.15	0.42	13.33	0.07	1.01	0.020	4.9E-01
Mtr.15298.1.S1_s_at	9.91	9.07	9.56	9.79	9.64	9.68	9.51	0.42	9.70	0.08	1.02	0.028	4.8E-01
Mtr.44756.1.S1_at	7.41	7.54	8.19	7.64	7.51	7.33	7.71	0.42	7.49	0.16	0.97	-0.041	4.4E-01
Mtr.11187.1.S1_at	9.21	8.63	9.45	9.08	9.45	8.96	9.10	0.42	9.16	0.26	1.01	0.010	8.3E-01
Mtr.4590.1.S1_at	5.33	4.57	4.64	4.69	5.01	4.68	4.85	0.42	4.79	0.19	0.99	-0.017	8.4E-01
Mtr.28289.1.S1_at	5.04	4.28	4.95	4.91	4.77	4.91	4.76	0.42	4.86	0.08	1.02	0.032	6.9E-01
Mtr.9163.1.S1_at	7.42	7.69	8.24	7.84	7.77	7.62	7.78	0.42	7.74	0.11	1.00	-0.007	8.9E-01
Mtr.42141.1.S1_s_at	13.84	14.56	13.83	13.74	13.41	13.86	14.08	0.42	13.67	0.23	0.97	-0.042	2.1E-01
Mtr.48748.1.S1_at	2.27	2.99	2.26	2.26	2.26	2.25	2.50	0.42	2.25	0.01	0.90	-0.151	3.6E-01
Mtr.34633.1.S1_at	10.82	10.08	10.80	10.75	10.46	10.73	10.57	0.42	10.65	0.16	1.01	0.011	7.8E-01
Mtr.43185.1.S1_at	9.85	9.04	9.61	9.31	9.50	9.26	9.50	0.42	9.36	0.13	0.98	-0.022	6.0E-01
Mtr.39558.1.S1_at	9.13	8.96	9.75	9.72	9.57	9.43	9.28	0.42	9.58	0.15	1.03	0.045	3.1E-01
Mtr.2132.1.S1_at	7.98	7.15	7.52	7.73	7.32	7.76	7.55	0.42	7.60	0.25	1.01	0.010	8.5E-01
Mtr.41357.1.S1_at	11.12	10.47	10.34	10.55	10.55	10.53	10.65	0.42	10.54	0.01	0.99	-0.014	7.0E-01
Mtr.32677.1.S1_at	8.09	8.77	8.01	8.07	8.02	8.43	8.29	0.42	8.17	0.23	0.99	-0.021	6.9E-01
Mtr.28751.1.S1_at	6.21	5.87	6.70	6.91	6.41	6.47	6.26	0.42	6.60	0.27	1.05	0.076	3.1E-01
Mtr.18290.1.S1_at	3.06	2.33	3.05	3.20	3.04	3.05	2.81	0.42	3.10	0.09	1.10	0.140	3.1E-01
Mtr.7406.1.S1_at	10.52	9.84	10.59	10.40	10.31	10.36	10.32	0.42	10.35	0.04	1.00	0.005	8.9E-01
Mtr.17078.1.S1_at	6.09	5.27	5.83	5.49	5.60	5.67	5.73	0.42	5.59	0.09	0.97	-0.037	5.9E-01
Mtr.9533.1.S1_at	9.46	8.67	9.29	9.29	9.53	9.25	9.14	0.42	9.35	0.15	1.02	0.033	4.5E-01
Mtr.43690.1.S1_s_at	9.32	8.48	8.94	9.21	9.13	9.27	8.91	0.42	9.20	0.07	1.03	0.046	3.0E-01
Mtr.8412.1.S1_at	2.22	2.89	2.12	2.12	2.11	2.12	2.41	0.42	2.12	0.00	0.88	-0.188	2.9E-01
Mtr.13827.1.S1_at	8.51	7.69	8.23	8.50	8.33	8.52	8.14	0.42	8.45	0.10	1.04	0.053	2.9E-01
Mtr.8932.1.S1_at	9.86	9.15	9.89	9.84	9.73	9.70	9.64	0.42	9.76	0.07	1.01	0.019	6.4E-01
Mtr.43850.1.S1_at	10.92	10.61	10.10	9.84	9.47	10.13	10.54	0.42	9.81	0.33	0.93	-0.104	7.7E-02
Mtr.24086.1.S1_at	2.63	3.33	2.59	2.58	2.57	2.58	2.85	0.42	2.58	0.00	0.90	-0.144	3.2E-01
Mtr.43825.1.S1_at	10.14	9.76	10.59	10.27	10.58	10.32	10.17	0.42	10.39	0.16	1.02	0.031	4.4E-01
Mtr.18152.1.S1_at	9.04	8.31	9.03	9.26	8.95	9.00	8.79	0.42	9.07	0.17	1.03	0.045	3.5E-01
Mtr.43565.1.S1_at	8.02	8.45	7.62	6.66	5.84	5.65	8.03	0.42	6.05	0.54	0.75	-0.409	7.4E-03
Mtr.25877.1.S1_s_at	8.82	7.99	8.49	8.61	8.77	8.76	8.43	0.42	8.71	0.09	1.03	0.047	3.2E-01
Mtr.17610.1.S1_at	6.65	5.83	6.39	6.11	6.06	6.43	6.29	0.42	6.20	0.20	0.99	-0.020	7.6E-01
Mtr.29781.1.S1_at	5.83	5.99	5.20	6.18	5.65	5.68	5.67	0.42	5.84	0.30	1.03	0.041	6.1E-01
Mtr.43693.1.S1_at	10.78	9.96	10.51	10.60	10.42	10.46	10.42	0.42	10.49	0.09	1.01	0.011	7.7E-01
Mtr.10092.1.S1_at	10.86	10.06	10.67	10.72	10.83	10.60	10.53	0.42	10.72	0.12	1.02	0.025	4.9E-01
Mtr.35556.1.S1_at	3.14	3.77	2.98	3.08	2.94	3.04	3.30	0.42	3.02	0.07	0.92	-0.126	3.2E-01
Mtr.17284.1.S1_at	7.57	6.85	6.83	7.60	8.41	8.05	7.09	0.42	8.02	0.41	1.13	0.179	4.9E-02
Mtr.38693.1.S1_at	3.29	2.52	2.62	2.62	3.26	2.50	2.81	0.42	2.79	0.41	0.99	-0.008	9.7E-01
Mtr.30063.1.S1_at	10.29	9.55	10.25	9.97	10.14	9.82	10.03	0.42	9.98	0.16	0.99	-0.007	8.5E-01
Mtr.46945.1.S1_at	8.69	8.85	9.48	9.11	9.38	9.13	9.01	0.42	9.21	0.15	1.02	0.031	4.8E-01

Mtr.34462.1.S1_at	3.97	3.28	3.22	3.93	4.61	4.18	3.49	0.42	4.24	0.35	1.21	0.280	7.5E-02
Mtr.32320.1.S1_s_at	7.45	6.63	7.16	7.21	7.15	7.20	7.08	0.42	7.18	0.03	1.01	0.021	7.0E-01
Mtr.43512.1.S1_at	8.63	7.90	8.62	8.61	9.07	8.76	8.38	0.42	8.82	0.23	1.05	0.073	1.9E-01
Mtr.31391.1.S1_at	3.11	3.80	3.06	3.00	3.07	3.02	3.32	0.42	3.03	0.04	0.91	-0.133	2.9E-01
Mtr.2614.1.S1_at	6.56	6.65	7.32	6.55	6.54	5.48	6.84	0.42	6.19	0.62	0.90	-0.145	2.0E-01
Mtr.28498.1.S1_at	8.93	8.10	8.56	8.26	8.73	8.71	8.53	0.42	8.57	0.27	1.00	0.005	9.2E-01
Mtr.11438.1.S1_at	9.82	10.10	10.64	10.31	10.42	10.18	10.19	0.42	10.30	0.12	1.01	0.016	6.8E-01
Mtr.4350.1.S1_at	10.82	11.57	11.52	11.04	11.32	11.13	11.30	0.42	11.16	0.14	0.99	-0.018	6.1E-01
Mtr.26626.1.S1_s_at	10.24	9.41	9.85	9.81	9.76	9.89	9.83	0.42	9.82	0.07	1.00	-0.002	9.6E-01
Mtr.13760.1.S1_at	10.90	11.50	11.70	11.58	11.57	11.45	11.37	0.42	11.53	0.07	1.01	0.021	5.4E-01
Mtr.27608.1.S1_s_at	3.58	2.85	3.57	4.03	3.84	3.11	3.33	0.42	3.66	0.49	1.10	0.135	4.3E-01
Mtr.21219.1.S1_at	3.39	4.12	3.41	3.51	3.65	3.60	3.64	0.42	3.58	0.07	0.98	-0.024	8.2E-01
Mtr.19084.1.S1_at	4.53	4.59	5.28	4.32	4.75	4.67	4.80	0.42	4.58	0.23	0.95	-0.067	4.7E-01
Mtr.51429.1.S1_at	2.94	3.77	3.41	2.25	2.70	2.93	3.38	0.42	2.63	0.34	0.78	-0.361	7.5E-02
Mtr.20557.1.S1_x_at	2.67	3.36	2.61	2.58	2.61	2.60	2.88	0.42	2.60	0.01	0.90	-0.149	3.0E-01
Mtr.8659.1.S1_at	5.04	5.54	5.87	4.61	4.67	5.20	5.49	0.42	4.83	0.33	0.88	-0.184	9.8E-02
Mtr.4442.1.S1_at	5.78	5.10	5.03	5.16	5.87	5.06	5.30	0.42	5.37	0.44	1.01	0.017	8.7E-01
Mtr.35708.1.S1_at	11.00	10.22	10.87	10.80	10.89	10.69	10.70	0.42	10.79	0.10	1.01	0.013	7.2E-01
Mtr.1079.1.S1_at	2.53	3.25	2.52	2.51	2.51	2.50	2.77	0.42	2.51	0.00	0.91	-0.142	3.4E-01
Mtr.9802.1.S1_at	11.52	10.70	11.26	11.37	11.21	11.39	11.16	0.42	11.32	0.10	1.01	0.021	5.5E-01
Mtr.13318.1.S1_at	9.79	9.22	8.98	9.69	9.62	9.81	9.33	0.42	9.70	0.09	1.04	0.057	2.0E-01
Mtr.42601.1.S1_at	10.34	9.69	10.47	10.31	10.44	10.08	10.17	0.42	10.28	0.18	1.01	0.015	7.0E-01
Mtr.22977.1.S1_s_at	3.03	3.73	2.99	2.97	2.94	2.98	3.25	0.42	2.96	0.02	0.91	-0.132	3.1E-01
Mtr.17520.1.S1_s_at	9.26	8.73	9.55	9.27	9.25	9.22	9.18	0.42	9.25	0.02	1.01	0.011	7.9E-01
Mtr.41590.1.S1_at	10.64	9.89	10.59	10.38	10.11	10.32	10.37	0.42	10.27	0.14	0.99	-0.014	7.1E-01
Mtr.39387.1.S1_s_at	7.99	7.63	8.46	8.04	8.00	8.00	8.03	0.42	8.01	0.02	1.00	-0.002	9.6E-01
Mtr.40568.1.S1_s_at	9.35	8.67	9.43	9.13	9.28	9.19	9.15	0.42	9.20	0.08	1.01	0.008	8.5E-01
Mtr.44051.1.S1_at	9.77	10.61	10.16	10.19	10.28	10.12	10.18	0.42	10.20	0.08	1.00	0.002	9.5E-01
Mtr.43323.1.S1_at	13.11	13.41	13.93	13.30	13.45	13.46	13.48	0.42	13.41	0.09	0.99	-0.008	7.6E-01
Mtr.16673.1.S1_at	3.09	3.79	3.04	3.02	2.98	3.01	3.31	0.42	3.00	0.02	0.91	-0.139	2.8E-01
Mtr.47450.1.S1_at	6.45	6.70	7.26	6.89	6.90	6.85	6.80	0.42	6.88	0.03	1.01	0.016	7.7E-01
Mtr.47552.1.S1_at	2.77	3.46	2.71	2.69	2.63	2.65	2.98	0.42	2.66	0.03	0.89	-0.166	2.5E-01
Mtr.38675.1.S1_s_at	10.50	9.89	10.69	10.43	10.69	10.47	10.36	0.42	10.53	0.14	1.02	0.024	5.3E-01
Mtr.10673.1.S1_s_at	11.77	12.09	12.60	12.10	12.32	12.02	12.15	0.42	12.15	0.16	1.00	0.000	9.9E-01
Mtr.14894.1.S1_at	8.90	8.37	9.19	9.09	9.29	9.23	8.82	0.42	9.20	0.10	1.04	0.062	1.9E-01
Mtr.24770.1.S1_at	6.63	6.05	6.85	6.70	6.91	6.56	6.51	0.42	6.72	0.17	1.03	0.046	4.6E-01
Mtr.6700.1.S1_at	8.44	7.61	8.06	8.07	8.31	7.98	8.03	0.42	8.12	0.17	1.01	0.016	7.5E-01
Mtr.46486.1.S1_at	10.31	9.78	10.60	10.31	10.49	10.32	10.23	0.42	10.37	0.10	1.01	0.020	5.9E-01
Mtr.41900.1.S1_at	3.12	2.42	2.38	2.34	2.37	2.58	2.64	0.42	2.43	0.13	0.92	-0.118	4.6E-01
Mtr.6882.1.S1_at	8.81	7.98	8.48	8.79	8.54	8.92	8.42	0.42	8.75	0.19	1.04	0.055	2.8E-01
Mtr.21995.1.S1_at	11.01	10.21	10.42	10.78	11.04	10.79	10.55	0.42	10.87	0.15	1.03	0.044	2.7E-01
Mtr.42875.1.S1_s_at	2.62	3.33	2.61	2.61	2.60	2.60	2.85	0.42	2.60	0.00	0.91	-0.132	3.6E-01
Mtr.43253.1.S1_at	14.14	13.47	14.23	13.74	13.28	13.38	13.95	0.42	13.47	0.24	0.97	-0.050	1.6E-01
Mtr.3453.1.S1_s_at	5.26	4.50	4.58	4.60	4.78	4.96	4.78	0.42	4.78	0.18	1.00	0.000	1.0E+00
Mtr.25596.1.S1_at	2.57	3.27	2.54	2.53	2.52	2.54	2.79	0.42	2.53	0.01	0.91	-0.144	3.3E-01
Mtr.16760.1.S1_at	7.89	7.05	7.49	7.91	7.66	7.92	7.48	0.42	7.83	0.15	1.05	0.066	2.4E-01
Mtr.12019.1.S1_at	6.06	5.52	5.24	5.80	5.43	5.89	5.60	0.42	5.71	0.25	1.02	0.026	7.3E-01
Mtr.10342.1.S1_at	11.70	12.02	12.53	11.80	12.13	12.02	12.08	0.42	11.98	0.16	0.99	-0.012	7.1E-01
Mtr.45443.1.S1_at	8.34	7.62	8.34	8.08	8.15	8.16	8.10	0.42	8.13	0.04	1.00	0.005	9.2E-01
Mtr.495.1.S1_at	6.37	5.67	6.40	6.32	6.00	6.78	6.14	0.42	6.37	0.39	1.04	0.052	5.3E-01
Mtr.41858.1.S1_at	7.08	6.29	6.90	7.07	7.06	6.86	6.75	0.42	7.00	0.12	1.04	0.051	3.9E-01
Mtr.34758.1.S1_at	6.81	6.08	6.80	6.80	6.84	6.80	6.56	0.42	6.81	0.02	1.04	0.054	3.6E-01
Mtr.13212.1.S1_at	11.76	10.93	11.41	11.43	10.94	11.54	11.37	0.42	11.30	0.32	0.99	-0.009	8.4E-01
Mtr.46219.1.S1_s_at	7.20	7.37	7.99	7.22	7.49	7.14	7.52	0.42	7.29	0.18	0.97	-0.045	4.3E-01
Mtr.6435.1.S1_at	9.84	9.53	10.36	10.09	10.13	9.98	9.91	0.42	10.07	0.08	1.02	0.023	5.5E-01
Mtr.40928.1.S1_at	11.13	10.37	10.47	10.40	10.84	10.31	10.66	0.42	10.52	0.28	0.99	-0.019	6.5E-01
Mtr.21837.1.S1_at	7.27	7.76	8.09	8.08	8.05	7.88	7.71	0.42	8.00	0.11	1.04	0.055	3.0E-01
Mtr.44908.1.S1_at	3.05	2.30	2.98	3.41	4.62	3.13	2.78	0.42	3.72	0.79	1.34	0.422	1.4E-01
Mtr.1308.1.S1_at	2.86	3.57	2.84	2.82	2.81	2.75	3.09	0.42	2.79	0.04	0.90	-0.146	2.8E-01
Mtr.15552.1.S1_at	12.23	12.48	13.04	12.72	12.77	12.70	12.58	0.42	12.73	0.04	1.01	0.017	5.7E-01
Mtr.28487.1.S1_s_at	9.54	8.73	9.31	9.07	9.01	9.02	9.19	0.42	9.04	0.03	0.98	-0.025	5.5E-01
Mtr.15226.1.S1_at	13.42	12.59	12.96	12.99	12.98	13.12	12.99	0.42	13.03	0.08	1.00	0.005	8.7E-01
Mtr.38189.1.S1_at	9.40	8.57	8.96	9.23	9.34	9.23	8.98	0.42	9.27	0.06	1.03	0.045	3.0E-01
Mtr.1159.1.S1_at	3.30	3.99	3.23	3.28	3.28	3.23	3.51	0.42	3.26	0.03	0.93	-0.104	3.7E-01
Mtr.12004.1.S1_at	8.90	8.08	8.53	8.57	8.59	8.65	8.50	0.41	8.60	0.04	1.01	0.017	7.0E-01
Mtr.11175.1.S1_at	9.97	10.20	10.77	10.28	10.24	10.04	10.31	0.41	10.19	0.13	0.99	-0.018	6.4E-01
Mtr.32383.1.S1_s_at	8.84	8.15	8.89	8.78	8.92	8.93	8.63	0.41	8.87	0.08	1.03	0.041	3.7E-01
Mtr.14399.1.S1_at	12.94	13.69	13.02	13.08	13.53	13.29	13.22	0.41	13.30	0.22	1.01	0.009	7.7E-01
Mtr.42217.1.S1_at	6.90	6.85	7.60	7.46	7.59	7.46	7.12	0.41	7.50	0.07	1.05	0.076	1.9E-01
Mtr.33188.1.S1_at	8.25	7.42	7.78	8.17	8.17	8.10	7.82	0.41	8.15	0.04	1.04	0.059	2.4E-01
Mtr.12181.1.S1_at	7.44	6.62	7.12	7.29	7.31	7.24	7.06	0.41	7.28	0.04	1.03	0.044	4.1E-01
Mtr.19915.1.S1_at	3.12	3.81	3.07	3.04	3.32	2.96	3.33	0.41	3.11	0.19	0.93	-0.102	4.4E-01
Mtr.44044.1.S1_s_at	8.94	8.19	8.86	8.88	8.76	8.96	8.66	0.41	8.87	0.10	1.02	0.034	4.5E-01
Mtr.15543.1.S1_at	9.66	8.83	9.27	9.35	9.36	9.28	9.25	0.41	9.33	0.04	1.01	0.012	7.6E-01
Mtr.45329.1.S1_at	11.11	10.28	10.71	10.72	10.56	10.43	10.70	0.41	10.57	0.14	0.99	-0.018	6.3E-01
Mtr.26254.1.S1_at	10.71	9.88	10.26	10.50	10.13	10.32	10.29	0.41	10.32	0.18	1.00	0.004	9.1E-01
Mtr.34826.1.S1_at	8.86	8.26	9.05	9.01	9.15	9.02	8.72	0.41	9.06	0.08	1.04	0.055	2.4E-01
Mtr.6647.1.S1_s_at	12.36	11.72	12.50	12.25	12.64	12.35	12.20	0.41	12.41	0.20	1.02	0.025	4.6E-01
Mtr.990.1.S1_at	3.02	3.70	2.96	2.96	2.94	2.88	3.23	0.41	2.92	0.04	0.91	-0.141	2.8E-01
Mtr.26618.1.S1_s_at	10.81	9.99	10.27	10.45	10.51	10.47	10.36	0.41	10.48	0.03	1.01	0.017	6.4E-01
Mtr.8929.1.S1_x_at	7.20	7.21	6.49	7.06	7.34	7.28	6.97	0.41	7.23	0.15	1.04	0.053	3.7E-01
Mtr.42416.1.S1_at	10.43	10.08	10.91	10.55	10.45	10.52	10.47	0.41	10.51	0.05	1.00	0.005	8.8E-01
Mtr.15860.1.S1_s_at	2.78	3.47	2.74	2.73	2.73	2.73	3.00	0.41	2.73	0.00	0.91	-0.134	3.3E-01
Mtr.27612.1.S1_at	2.57	3.29	2.57	2.62	2.57	2.52	2.81	0.41	2.57	0.05	0.91	-0.129	3.8E-01
Mtr.7207.1.S1_at	11.26	10.84	10.43	10.89	11.19	10.91	10.85	0.41	11.00	0.16	1.01	0.020	5.9E-01
Mtr.20299.1.S1_at	10.07	9.26	9.81	9.93	9.72	9.92	9.71	0.41	9.86	0.12	1.01	0.021	5.9E-01
Mtr.4438.1.S1_x_at	5.72	4.99	5.69	5.98	5.52	5.92	5.46	0.41	5.81	0.25	1.06	0.088	2.9E-01
Mtr.25491.1.S1_at	9.75	9.06	9.79	9.74	9.64	9.71	9.53	0.41	9.70	0.05	1.02	0.025	5.3E-01
Mtr.32691.1.S1_at	9.74	10.48	10.43	9.66	9.85	9.63	10.22	0.41	9.71	0.12	0.95	-0.073	1.1E-01
Mtr.42644.1.S1_s_at	10.10	9.31	9.92	9.95	9.84	9.73	9.77	0.41	9.84	0.11	1.01	0.010	8.1E-01
Mtr.20794.1.S1_at	6.72	7.38	6.62	7.31	8.12	7.36	6.91	0.41	7.59	0.45	1.10	0.137	1.2E-01
Mtr.3665.1.S1_at	5.02	4.34	5.09	5.05	5.32	5.08	4.82	0.41	5.15	0.15	1.07	0.097	2.6E-01
Mtr.28371.1.S1_at	9.78	8.98	9.58	10.07	9.93	9.98	9.45	0.41	9.99	0.07	1.06	0.081	8.9E-02
Mtr.34452.1.S1_at	2.87	2.81	3.56	3.82	3.04	2.60	3.08	0.41	3.15	0.61	1.02	0.035	8.7E-01
Mtr.40222.1.S1_s_at	13.90	13.08	13.43	13.52	13.45	13.54	13.47	0.41	13.50	0.05	1.00	0.004	8.9E-01
Mtr.11618.1.S1_at	11.78	11.12	11.88	11.85	11.63	11.72	11.59	0.41	11.73	0.11	1.01	0.017	6.1E-01
Mtr.34930.1.S1_at	8.44	7.71	7.73	8.21	8.18	8.37	7.96	0.41	8.25	0.10	1.04	0.052	3.0E-01
Mtr.11683.1.S1_at	11.22	10.39	10.76	10.77	10.80	10.82	10.79	0.41	10.79	0.03	1.00	0.000	9.9E-01
Mtr.18010.1.S1_at	11.90	11.07	11.52	11.69	11.65	11.53	11.50	0.41	11.62	0.08	1.01	0.015	6.4E-01
Mtr.15891.1.S1_at	8.24	7.86	8.68	8.14	8.14	8.17	8.26	0.41	8.15	0.02	0.99	-0.020	6.6E-01
Mtr.25291.1.S1_at	9.48	8.74	9.43	9.38	9.52	9.34	9.22	0.41	9.41	0.09	1.02	0.030	4.8E-01
Mtr.44355.1.S1_at	10.01	9.18	9.63	9.84	9.78	9.74	9.61	0.41	9.79	0.05	1.02	0.027	5.0E-01
Mtr.24413.1.S1_x_at	3.44	4.11	3.35	3.33	3.16	3.16	3.63	0.41	3.22	0.10	0.88	-0.176	1.6E-01
Mtr.10928.1.S1_s_at	7.73	8.14	8.56	9.79	10.19	10.21	8.14	0.41	10.06	0.24	1.24	0.306	2.2E-03
Mtr.14428.1.S1_at	6.89	6.10	6.27	5.91	5.47	5.93	6.42	0.41	5.77	0.26	0.90	-0.155	8.2E-02
Mtr.5922.1.S1_at	3.38	3.76	2.93	4.02	3.98	3.64	3.35	0.41	3.88	0.21	1.16	0.211	1.2E-01
Mtr.7097.1.S1_at	9.42	8.70	9.42	9.61	9.49	9.60	9.18	0.41	9.57	0.07	1.04	0.060	1.9E-01
Mtr.37681.1.S1_at	2.40	3.10	2.37	2.37	2.36	2.37	2.62	0.41	2.37	0.00	0.90	-0.148	3.4E-01
Mtr.10671.1.S1_at	12.04	11.25	11.43	11.54	11.61	11.54	11.57	0.41	11.56	0.04	1.00	-0.001	9.7E-01
Mtr.14826.1.S1_at	3.80	3.15	3.03	3.17	3.14	3.13	3.32	0.41	3.15	0.02	0.95	-0.079	5.0E-01
Mtr.37737.1.S1_at	11.53	10.82	11.53	11.75	11.75	11.54	11.29	0.41	11.68	0.12	1.03	0.049	1.9E-01
Mtr.32397.1.S1_s_at	8.59	7.99	8.78	8.53	8.70	8.82	8.45	0.41	8.68	0.15	1.03	0.039	4.1E-01
Mtr.13275.1.S1_at	7.26	6.46	6.68	5.86	6.23	5.88	6.80	0.41	5.99	0.21	0.88	-0.183	3.9E-02
Mtr.38092.1.S1_at	11.45	10.77	10.70	11.33	11.21	11.54	10.97	0.41	11.36	0.17	1.04	0.050	2.1E-01
Mtr.39252.1.S1_at	8.40	7.76	8.54	8.38	8.73	8.25	8.23	0.41	8.46	0.25	1.03	0.038	4.7E-01
Mtr.29836.1.S1_at	6.64	5.84	6.42	6.47	6.37	6.31	6.30	0.41	6.38	0.08	1.01	0.019	7.5E-01
Mtr.8219.1.S1_at	8.89	8.14	8.81	8.45	8.36	8.20	8.61	0.41	8.34	0.13	0.97	-0.047	3.3E-01
Mtr.9732.1.S1_at	8.59	7.81	7.95	8.14	7.80	7.91	8.12	0.41	7.95	0.17	0.98	-0.030	5.5E-01
Mtr.32007.1.S1_at	9.02	8.30	9.02	9.06	9.31	8.90	8.78	0.41	9.09	0.20	1.04	0.050	3.1E-01
Mtr.32816.1.S1_at	7.54	8.06	8.36	8.26	8.00	8.00	7.99	0.41	8.09	0.15	1.01	0.018	7.2E-01
Mtr.40062.1.S1_at	10.49	9.69	10.24	10.26	10.00	10.13	10.14	0.41	10.13	0.13	1.00	-0.001	9.8E-01
Mtr.19929.1.S1_at	2.71	3.39	2.65	2.64	2.63	2.63	2.92	0.41	2.64	0.01	0.90	-0.145	3.1E-01
Mtr.37744.1.S1_at	10.52	9.81	9.79	9.95	9.68	9.85	10.04	0.41	9.83	0.13	0.98	-0.031	4.5E-01
Mtr.11658.1.S1_at	9.97	9.84	10.61	10.21	10.25	9.94	10.14	0.41	10.13	0.17	1.00	-0.001	9.8E-01
Mtr.47227.1.S1_s_at	9.56	8.73	9.15	9.35	9.17	9.25	9.15	0.41	9.26	0.09	1.01	0.018	6.7E-01
Mtr.12538.1.S1_at	10.91	10.09	10.53	10.43	10.42	10.60	10.51	0.41	10.49	0.10	1.00	-0.003	9.2E-01
Mtr.37921.1.S1_at	10.83	10.00	10.49	10.68	10.75	10.64	10.44	0.41	10.69	0.06	1.02	0.034	3.6E-01
Mtr.30720.1.S1_at	8.87	8.46	9.28	8.81	8.92	8.71	8.87	0.41	8.81	0.10	0.99	-0.010	8.2E-01
Mtr.28232.1.S1_s_at	9.72	9.20	10.01	9.66	9.36	9.54	9.65	0.41	9.52	0.15	0.99	-0.019	6.4E-01
Mtr.40420.1.S1_at	13.36	14.15	13.97	13.99	14.15	14.14	13.83	0.41	14.09	0.09	1.02	0.027	3.4E-01
Mtr.30101.1.S1_at	3.85	3.15	3.12	3.12	3.03	3.03	3.37	0.41	3.06	0.05	0.91	-0.140	2.6E-01

Mtr.31746.1.S1_at	8.24	8.83	9.04	8.43	8.49	8.46	8.70	0.41	8.46	0.03	0.97	-0.041	3.7E-01
Mtr.24198.1.S1_s_at	9.63	9.32	10.14	10.95	10.99	11.41	9.70	0.41	11.12	0.25	1.15	0.197	7.0E-03
Mtr.12988.1.S1_at	5.44	4.78	5.54	6.84	6.24	6.10	5.25	0.41	6.40	0.39	1.22	0.285	2.5E-02
Mtr.44981.1.S1_at	3.11	2.75	3.57	2.90	3.95	2.79	3.14	0.41	3.22	0.64	1.02	0.034	8.7E-01
Mtr.11441.1.S1_at	8.24	7.91	8.73	8.55	8.55	8.50	8.29	0.41	8.53	0.03	1.03	0.042	3.7E-01
Mtr.32859.1.S1_s_at	11.12	10.67	10.30	10.90	10.71	11.09	10.70	0.41	10.90	0.19	1.02	0.027	4.9E-01
Mtr.40453.1.S1_at	11.18	10.40	11.01	11.04	11.09	10.94	10.86	0.41	11.02	0.07	1.01	0.021	5.5E-01
Mtr.35562.1.S1_at	10.56	10.14	9.73	10.34	10.55	10.23	10.14	0.41	10.37	0.16	1.02	0.033	4.2E-01
Mtr.25620.1.S1_at	11.42	10.59	11.01	10.93	11.05	11.08	11.01	0.41	11.02	0.08	1.00	0.002	9.6E-01
Mtr.21065.1.S1_at	2.42	3.14	2.43	2.40	2.38	2.39	2.67	0.41	2.39	0.01	0.90	-0.157	3.1E-01
Mtr.10581.1.S1_at	9.42	9.11	9.93	9.78	9.98	9.77	9.49	0.41	9.84	0.12	1.04	0.053	2.3E-01
Mtr.25597.1.S1_at	2.76	3.46	2.74	2.75	2.75	2.72	2.99	0.41	2.74	0.01	0.92	-0.126	3.6E-01
Mtr.11529.1.S1_at	3.62	3.18	4.01	3.26	3.08	3.10	3.61	0.41	3.15	0.10	0.87	-0.197	1.3E-01
Mtr.40165.1.S1_at	9.17	9.75	9.96	9.59	9.90	9.31	9.63	0.41	9.60	0.29	1.00	-0.004	9.3E-01
Mtr.5867.1.S1_at	10.18	10.21	9.48	10.06	9.99	10.14	9.96	0.41	10.07	0.08	1.01	0.016	6.8E-01
Mtr.21793.1.S1_at	7.44	6.96	7.78	7.16	7.41	7.54	7.39	0.41	7.37	0.19	1.00	-0.004	9.4E-01
Mtr.48475.1.S1_at	4.49	4.48	5.20	5.09	4.67	5.05	4.72	0.41	4.94	0.24	1.05	0.064	4.8E-01
Mtr.46810.1.S1_at	5.35	4.67	4.60	6.66	7.47	7.76	4.88	0.41	7.29	0.57	1.50	0.581	4.0E-03
Mtr.47782.1.S1_at	7.44	6.71	7.41	7.29	7.35	7.42	7.19	0.41	7.35	0.07	1.02	0.033	5.3E-01
Mtr.49360.1.S1_at	3.65	2.90	3.57	3.65	4.03	3.80	3.37	0.41	3.83	0.19	1.14	0.184	1.6E-01
Mtr.32249.1.S1_at	10.57	9.97	10.76	10.89	10.51	10.70	10.43	0.41	10.70	0.19	1.03	0.037	3.6E-01
Mtr.9523.1.S1_at	6.68	5.86	6.19	6.88	7.19	7.49	6.24	0.41	7.19	0.30	1.15	0.203	3.3E-02
Mtr.12052.1.S1_at	4.88	4.72	5.50	5.49	5.52	5.64	5.03	0.41	5.55	0.08	1.10	0.141	1.0E-01
Mtr.30762.1.S1_at	9.99	9.26	9.96	9.93	9.73	9.95	9.73	0.41	9.87	0.12	1.01	0.019	6.2E-01
Mtr.34450.1.S1_at	10.90	11.64	11.57	11.05	10.95	11.04	11.37	0.41	11.01	0.06	0.97	-0.046	2.1E-01
Mtr.50453.1.S1_at	9.18	8.72	8.35	8.81	8.77	9.02	8.75	0.41	8.86	0.13	1.01	0.019	6.7E-01
Mtr.1220.1.S1_at	3.14	3.80	3.04	3.02	2.97	2.96	3.33	0.41	2.98	0.03	0.90	-0.156	2.3E-01
Mtr.42243.1.S1_at	11.08	11.81	11.79	11.43	11.56	11.47	11.56	0.41	11.49	0.07	0.99	-0.009	7.7E-01
Mtr.31700.1.S1_at	8.70	8.03	8.78	8.69	8.84	8.49	8.50	0.41	8.67	0.18	1.02	0.029	5.4E-01
Mtr.43491.1.S1_at	9.56	9.35	10.15	9.73	9.59	9.57	9.69	0.41	9.63	0.09	0.99	-0.008	8.3E-01
Mtr.36375.1.S1_at	4.48	4.19	3.67	3.75	3.66	3.66	4.11	0.41	3.69	0.05	0.90	-0.156	1.5E-01
Mtr.23653.1.S1_at	10.73	10.03	10.76	10.76	10.84	10.78	10.51	0.41	10.79	0.04	1.03	0.039	3.0E-01
Mtr.32040.1.S1_at	6.73	7.40	7.48	6.54	7.11	6.61	7.20	0.41	6.75	0.31	0.94	-0.093	2.1E-01
Mtr.7355.1.S1_at	8.87	9.69	9.37	9.22	9.36	8.96	9.31	0.41	9.18	0.20	0.99	-0.020	6.5E-01
Mtr.6730.1.S1_at	6.37	6.39	7.09	6.45	6.58	6.40	6.62	0.41	6.48	0.09	0.98	-0.030	6.1E-01
Mtr.44853.1.S1_at	8.49	7.67	8.00	8.08	7.85	8.15	8.05	0.41	8.02	0.16	1.00	-0.005	9.2E-01
Mtr.10747.1.S1_at	12.15	11.45	11.42	11.89	11.88	11.97	11.67	0.41	11.91	0.05	1.02	0.029	3.7E-01
Mtr.48572.1.S1_at	10.49	9.94	9.69	9.88	9.76	10.00	10.04	0.41	9.88	0.12	0.98	-0.023	5.6E-01
Mtr.2537.1.S1_a_at	11.06	11.72	11.82	11.60	11.47	11.56	11.53	0.41	11.54	0.06	1.00	0.001	9.7E-01
Mtr.26255.1.S1_at	7.67	6.86	7.14	7.34	7.21	7.45	7.23	0.41	7.33	0.12	1.02	0.022	6.8E-01
Mtr.38663.1.S1_at	9.58	9.39	10.18	9.98	9.92	9.74	9.72	0.41	9.88	0.12	1.02	0.024	5.5E-01
Mtr.10240.1.S1_at	8.80	8.34	9.16	8.80	8.95	8.85	8.76	0.41	8.87	0.08	1.01	0.017	7.0E-01
Mtr.39398.1.S1_at	9.74	9.22	10.03	9.88	9.86	9.91	9.66	0.41	9.88	0.02	1.02	0.033	4.1E-01
Mtr.51595.1.S1_at	10.63	9.84	10.44	10.32	10.00	9.97	10.31	0.41	10.10	0.19	0.98	-0.030	4.7E-01
Mtr.3484.1.S1_at	2.88	3.56	2.83	2.79	2.79	2.74	3.09	0.41	2.78	0.03	0.90	-0.154	2.6E-01
Mtr.5062.1.S1_at	8.39	7.77	8.55	8.62	8.41	8.56	8.24	0.41	8.53	0.11	1.04	0.051	3.0E-01
Mtr.50377.1.S1_at	4.48	4.26	3.68	4.35	4.37	4.38	4.14	0.41	4.37	0.01	1.05	0.077	4.0E-01
Mtr.46008.1.S1_at	9.29	8.49	9.04	9.22	9.08	9.29	8.94	0.41	9.20	0.11	1.03	0.041	3.5E-01
Mtr.37156.1.S1_at	9.03	8.36	9.11	8.57	8.76	8.66	8.83	0.41	8.67	0.10	0.98	-0.028	5.3E-01
Mtr.42312.1.S1_at	10.41	9.59	9.97	10.00	9.98	10.04	9.99	0.41	10.01	0.03	1.00	0.002	9.5E-01
Mtr.24038.1.S1_x_at	2.78	3.49	2.76	2.75	2.75	2.74	3.01	0.41	2.75	0.01	0.91	-0.132	3.3E-01
Mtr.11809.1.S1_at	8.23	8.47	9.03	8.38	8.80	8.37	8.58	0.41	8.52	0.24	0.99	-0.011	8.3E-01
Mtr.13321.1.S1_at	5.22	4.78	4.40	4.74	5.49	4.63	4.80	0.41	4.96	0.47	1.03	0.046	6.9E-01
Mtr.11974.1.S1_at	9.37	8.64	9.33	9.27	9.27	9.20	9.11	0.41	9.24	0.04	1.01	0.020	6.2E-01
Mtr.8419.1.S1_at	10.67	10.19	11.01	10.63	10.64	10.51	10.62	0.41	10.59	0.07	1.00	-0.004	9.1E-01
Mtr.39442.1.S1_at	8.66	8.24	9.06	8.76	8.77	8.77	8.65	0.41	8.77	0.01	1.01	0.019	6.5E-01
Mtr.12288.1.S1_at	12.89	12.07	12.50	12.30	12.11	12.36	12.48	0.41	12.26	0.13	0.98	-0.027	4.1E-01
Mtr.41978.1.S1_at	10.14	9.40	10.08	9.96	9.98	9.86	9.88	0.41	9.93	0.06	1.01	0.009	8.2E-01
Mtr.12139.1.S1_at	7.05	6.44	7.22	7.23	7.20	7.07	6.90	0.41	7.16	0.09	1.04	0.054	3.4E-01
Mtr.38043.1.S1_at	10.08	10.85	10.20	9.91	9.84	10.17	10.38	0.41	9.97	0.17	0.96	-0.057	1.9E-01
Mtr.31824.1.S1_at	9.07	9.85	9.67	9.14	9.43	9.38	9.53	0.41	9.31	0.16	0.98	-0.033	4.5E-01
Mtr.40481.1.S1_at	11.47	11.25	10.67	10.93	11.12	11.00	11.13	0.41	11.02	0.10	0.99	-0.015	6.7E-01
Mtr.4884.1.S1_s_at	9.87	9.19	9.92	9.78	9.64	9.72	9.66	0.41	9.71	0.07	1.01	0.008	8.4E-01
Mtr.19764.1.S1_at	8.92	8.33	9.13	8.96	9.10	9.04	8.79	0.41	9.03	0.07	1.03	0.039	3.7E-01
Mtr.38837.1.S1_s_at	8.57	9.28	8.57	8.76	8.69	8.45	8.80	0.41	8.63	0.16	0.98	-0.028	5.4E-01
Mtr.13497.1.S1_at	9.71	8.90	9.39	9.17	9.27	9.34	9.33	0.41	9.26	0.08	0.99	-0.011	7.8E-01
Mtr.15845.1.S1_at	4.58	4.63	5.32	4.56	4.57	4.64	4.84	0.41	4.59	0.04	0.95	-0.078	3.5E-01
Mtr.15331.1.S1_s_at	9.06	8.44	9.22	9.70	9.76	9.76	8.90	0.41	9.74	0.03	1.09	0.130	2.5E-02
Mtr.3682.1.S1_at	2.69	3.40	2.68	2.72	2.64	2.67	2.92	0.41	2.68	0.04	0.92	-0.126	3.6E-01
Mtr.39695.1.S1_at	10.36	9.63	10.32	10.57	10.34	10.42	10.10	0.41	10.45	0.11	1.03	0.048	2.4E-01
Mtr.44374.1.S1_at	7.82	7.68	8.45	7.98	8.52	8.21	7.98	0.41	8.24	0.27	1.03	0.045	4.2E-01
Mtr.40272.1.S1_at	11.55	11.24	12.05	12.18	12.65	12.34	11.61	0.41	12.39	0.24	1.07	0.094	4.7E-02
Mtr.12913.1.S1_at	8.54	7.96	8.76	8.83	8.68	8.57	8.42	0.41	8.69	0.13	1.03	0.046	3.4E-01
Mtr.19647.1.S1_at	3.44	2.78	2.69	2.70	3.57	2.66	2.97	0.41	2.98	0.51	1.00	0.004	9.8E-01
Mtr.5.1.S1_x_at	7.37	7.22	7.99	7.47	7.42	7.49	7.53	0.41	7.46	0.04	0.99	-0.013	7.9E-01
Mtr.40618.1.S1_at	10.15	9.80	10.62	10.18	10.29	10.16	10.19	0.41	10.21	0.07	1.00	0.003	9.4E-01
Mtr.27961.1.S1_s_at	9.18	9.82	9.05	9.08	9.67	9.43	9.35	0.41	9.39	0.30	1.00	0.007	8.8E-01
Mtr.41210.1.S1_at	11.18	10.42	11.07	11.19	11.08	10.74	10.89	0.41	11.00	0.24	1.01	0.015	7.0E-01
Mtr.13973.1.S1_s_at	9.72	9.44	10.25	9.45	9.86	9.65	9.81	0.41	9.65	0.21	0.98	-0.023	5.9E-01
Mtr.15661.1.S1_at	9.94	9.72	10.51	9.91	10.04	10.04	10.06	0.41	10.00	0.07	0.99	-0.009	8.1E-01
Mtr.36874.1.S1_at	2.52	3.23	2.51	2.50	2.50	2.45	2.75	0.41	2.48	0.03	0.90	-0.147	3.2E-01
Mtr.5326.1.S1_at	7.90	7.40	8.22	7.96	8.33	7.96	7.84	0.41	8.08	0.22	1.03	0.044	4.2E-01
Mtr.24670.1.S1_at	3.80	2.98	3.34	3.47	3.57	3.47	3.37	0.41	3.50	0.06	1.04	0.054	6.2E-01
Mtr.26179.1.S1_at	2.51	3.21	2.48	2.48	2.47	2.47	2.74	0.41	2.47	0.00	0.90	-0.145	3.3E-01
Mtr.37892.1.S1_at	9.93	10.39	10.75	10.40	10.45	10.26	10.35	0.41	10.37	0.10	1.00	0.002	9.5E-01
Mtr.4705.1.S1_at	7.43	8.14	8.14	7.71	8.04	7.68	7.90	0.41	7.81	0.20	0.99	-0.017	7.4E-01
Mtr.20952.1.S1_at	2.83	3.54	2.82	2.70	2.71	2.60	3.06	0.41	2.67	0.06	0.87	-0.196	1.8E-01
Mtr.10984.1.S1_at	9.68	8.87	9.37	9.59	9.90	9.81	9.30	0.41	9.77	0.16	1.05	0.070	1.4E-01
Mtr.44676.1.S1_at	8.39	7.67	8.38	8.39	8.41	8.52	8.15	0.41	8.44	0.07	1.04	0.051	2.9E-01
Mtr.17518.1.S1_at	2.81	3.47	2.72	2.70	2.67	2.70	3.00	0.41	2.69	0.02	0.90	-0.157	2.6E-01
Mtr.25046.1.S1_at	2.70	3.40	2.68	2.67	2.67	2.63	2.93	0.41	2.66	0.02	0.91	-0.138	3.2E-01
Mtr.12156.1.S1_at	8.27	7.55	7.56	7.97	7.87	7.78	7.79	0.41	7.88	0.10	1.01	0.015	7.6E-01
Mtr.29846.1.S1_at	5.63	5.86	6.43	5.13	5.46	5.06	5.97	0.41	5.22	0.22	0.87	-0.195	4.8E-02
Mtr.23976.1.S1_at	2.57	3.25	2.51	2.50	2.51	2.51	2.78	0.41	2.51	0.01	0.90	-0.148	3.2E-01
Mtr.10830.1.S1_at	8.72	9.48	8.85	8.19	8.10	7.99	9.02	0.41	8.09	0.10	0.90	-0.156	1.9E-02
Mtr.4313.1.S1_x_at	3.10	3.77	3.03	3.14	2.93	2.97	3.30	0.41	3.01	0.11	0.91	-0.130	3.1E-01
Mtr.31734.1.S1_at	10.56	11.02	11.38	11.03	11.16	10.81	10.99	0.41	11.00	0.18	1.00	0.002	9.7E-01
Mtr.49431.1.S1_at	4.04	4.79	4.70	4.11	4.28	4.32	4.51	0.41	4.24	0.11	0.94	-0.091	3.2E-01
Mtr.20256.1.S1_at	2.89	3.59	2.88	2.86	2.83	2.87	3.12	0.41	2.85	0.02	0.91	-0.128	3.3E-01
Mtr.9930.1.S1_at	9.46	8.67	8.89	9.20	9.13	9.15	9.01	0.41	9.16	0.04	1.02	0.025	5.5E-01
Mtr.39494.1.S1_at	2.15	2.86	2.15	2.16	2.13	2.18	2.39	0.41	2.16	0.02	0.90	-0.147	3.8E-01
Mtr.47187.1.S1_at	10.43	9.61	9.96	10.01	9.88	9.94	10.00	0.41	9.94	0.07	0.99	-0.008	8.3E-01
Mtr.41633.1.S1_at	8.03	8.36	8.85	8.47	8.71	8.38	8.41	0.41	8.52	0.17	1.01	0.018	7.0E-01
Mtr.40620.1.S1_at	3.49	3.04	3.86	2.73	2.78	2.73	3.46	0.41	2.75	0.03	0.79	-0.332	4.0E-02
Mtr.29285.1.S1_at	8.28	7.56	8.26	7.67	7.88	7.87	8.03	0.41	7.81	0.12	0.97	-0.042	4.0E-01
Mtr.18402.1.S1_at	7.07	6.29	6.90	6.83	6.79	7.04	6.75	0.41	6.88	0.13	1.02	0.028	6.2E-01
Mtr.21893.1.S1_s_at	5.99	5.46	6.26	6.34	6.31	6.08	5.90	0.41	6.24	0.14	1.06	0.081	2.5E-01
Mtr.45441.1.S1_at	9.41	8.75	9.50	9.68	9.58	9.52	9.22	0.41	9.59	0.08	1.04	0.057	2.0E-01
Mtr.40153.1.S1_at	9.49	8.67	9.15	9.41	9.53	9.61	9.10	0.41	9.52	0.10	1.05	0.064	1.7E-01
Mtr.39309.1.S1_at	7.09	7.12	7.81	7.28	7.14	6.91	7.34	0.41	7.11	0.19	0.97	-0.046	4.3E-01
Mtr.31501.1.S1_at	3.51	3.06	3.88	3.33	3.33	3.33	3.48	0.41	3.33	0.00	0.96	-0.064	5.6E-01
Mtr.44321.1.S1_at	7.89	7.22	7.96	7.82	7.79	7.90	7.69	0.41	7.84	0.05	1.02	0.028	5.7E-01
Mtr.35759.1.S1_at	7.56	6.80	6.93	7.26	7.61	7.57	7.10	0.41	7.48	0.19	1.05	0.076	2.1E-01
Mtr.43504.1.S1_at	12.57	11.75	12.17	12.42	12.79	12.58	12.16	0.41	12.60	0.18	1.04	0.051	1.7E-01
Mtr.11046.1.S1_at	7.80	7.18	7.95	7.67	7.25	7.18	7.64	0.41	7.37	0.26	0.96	-0.054	3.8E-01
Mtr.162.1.S1_at	3.03	3.70	2.96	2.96	2.94	2.94	3.23	0.41	2.95	0.01	0.91	-0.132	3.0E-01
Mtr.44922.1.S1_at	8.53	9.26	9.21	9.48	9.15	9.30	9.00	0.41	9.31	0.17	1.03	0.049	2.9E-01
Mtr.15314.1.S1_at	2.55	3.19	2.44	2.50	2.50	2.48	2.73	0.41	2.49	0.01	0.91	-0.130	3.8E-01
Mtr.32188.1.S1_x_at	5.59	4.79	5.30	5.27	5.14	5.29	5.23	0.41	5.23	0.09	1.00	0.002	9.8E-01
Mtr.49427.1.S1_at	7.87	8.04	8.65	7.90	7.94	7.68	8.19	0.41	7.84	0.14	0.96	-0.062	2.4E-01
Mtr.43746.1.S1_at	11.25	12.05	11.50	11.41	11.26	11.38	11.60	0.41	11.35	0.08	0.98	-0.032	3.5E-01
Mtr.25174.1.S1_at	9.40	10.14	10.07	9.72	9.84	9.86	9.87	0.41	9.81	0.08	0.99	-0.009	8.1E-01
Mtr.6744.1.S1_at	2.85	3.01	3.63	2.81	2.90	2.86	3.16	0.41	2.85	0.04	0.90	-0.150	2.6E-01
Mtr.5785.1.S1_s_at	10.56	9.92	9.80	10.45	10.41	10.82	10.09	0.41	10.56	0.23	1.05	0.066	1.6E-01
Mtr.6315.1.S1_at	8.99	8.22	8.85	9.17	8.91	9.11	8.69	0.41	9.06	0.13	1.04	0.061	2.0E-01
Mtr.26765.1.S1_at	3.58	4.28	3.56	3.55	3.54	3.83	3.81	0.41	3.64	0.16	0.96	-0.064	5.5E-01
Mtr.45164.1.S1_at	9.46	8.68	9.28	9.11	9.22	9.35	9.14	0.41	9.23	0.12	1.01	0.014	7.3E-01
Mtr.15902.1.S1_at	2.72	3.43	2.71	2.71	2.70	2.66	2.95	0.41	2.69	0.02	0.91	-0.135	3.3E-01
Mtr.43134.1.S1_at	12.48	11.68	11.92	11.90	11.74	11.81	12.03	0.41	11.81	0.08	0.98	-0.026	4.3E-01
Mtr.10142.1.S1_at	6.53	5.84	6.57	6.52	6.51	6.50	6.31	0.41	6.51	0.01	1.03	0.044	4.6E-01
Mtr.37569.1.S1_at	11.07	10.40	11.15	10.71	10.80	10.70	10.87	0.41	10.74	0.05	0.99	-0.018	6.0E-01

Mtr.29526.1.S1_at	8.73	7.93	8.50	8.29	8.51	8.28	8.39	0.41	8.36	0.13	1.00	-0.005	9.2E-01
Mtr.27831.1.S1_at	7.90	7.08	7.54	7.92	7.77	7.94	7.51	0.41	7.88	0.09	1.05	0.069	2.0E-01
Mtr.44807.1.S1_at	8.47	7.71	7.83	8.45	8.26	8.51	8.00	0.41	8.40	0.13	1.05	0.070	1.8E-01
Mtr.38165.1.S1_at	11.85	11.06	11.63	11.84	11.91	11.73	11.51	0.41	11.83	0.09	1.03	0.039	2.6E-01
Mtr.39272.1.S1_at	9.43	8.90	8.63	9.18	9.15	9.24	8.99	0.41	9.19	0.04	1.02	0.032	4.4E-01
Mtr.4062.1.S1_at	8.74	8.29	9.10	8.58	8.77	8.60	8.71	0.41	8.65	0.10	0.99	-0.010	8.2E-01
Mtr.16151.1.S1_at	5.98	5.18	5.74	6.25	6.43	6.06	5.63	0.41	6.25	0.19	1.11	0.150	7.6E-02
Mtr.48955.1.S1_at	4.44	3.76	3.72	5.31	4.58	5.47	3.97	0.41	5.12	0.47	1.29	0.366	3.3E-02
Mtr.27010.1.S1_at	2.60	3.29	2.56	2.55	2.55	2.54	2.81	0.41	2.55	0.00	0.90	-0.144	3.2E-01
Mtr.45028.1.S1_s_at	7.73	8.36	8.49	8.23	8.43	8.31	8.19	0.41	8.33	0.10	1.02	0.023	6.1E-01
Mtr.14446.1.S1_at	7.81	7.25	8.05	7.82	7.89	7.86	7.70	0.41	7.86	0.04	1.02	0.028	5.5E-01
Mtr.11838.1.S1_at	7.71	7.80	8.46	8.05	8.06	7.84	7.99	0.41	7.98	0.13	1.00	-0.001	9.8E-01
Mtr.31711.1.S1_at	7.44	6.81	7.57	7.48	7.49	7.10	7.28	0.41	7.35	0.22	1.01	0.016	7.8E-01
Mtr.4435.1.S1_at	9.37	8.58	9.16	8.98	8.90	9.16	9.04	0.41	9.01	0.13	1.00	-0.004	9.3E-01
Mtr.49067.1.S1_at	8.19	7.55	8.31	8.36	8.29	8.27	8.02	0.41	8.31	0.05	1.04	0.052	2.8E-01
Mtr.38444.1.S1_at	11.00	10.38	10.22	10.26	10.51	10.39	10.53	0.41	10.39	0.13	0.99	-0.020	6.0E-01
Mtr.44780.1.S1_at	12.42	13.11	13.13	12.76	12.67	12.67	12.89	0.41	12.70	0.05	0.99	-0.021	4.7E-01
Mtr.37587.1.S1_at	14.07	13.44	13.31	13.72	13.57	13.74	13.61	0.41	13.68	0.09	1.01	0.008	7.8E-01
Mtr.21108.1.S1_at	2.33	3.03	2.31	2.30	2.30	2.28	2.55	0.41	2.29	0.01	0.90	-0.155	3.3E-01
Mtr.31543.1.S1_at	6.96	6.29	7.04	7.11	7.05	7.21	6.76	0.41	7.12	0.08	1.05	0.074	2.1E-01
Mtr.4696.1.S1_s_at	9.17	8.36	8.89	8.69	8.95	8.70	8.81	0.41	8.78	0.15	1.00	-0.004	9.2E-01
Mtr.4894.1.S1_at	2.40	3.10	2.38	2.37	2.37	2.37	2.63	0.41	2.37	0.00	0.90	-0.151	3.3E-01
Mtr.18800.1.S1_at	2.66	3.35	2.62	2.61	2.59	2.59	2.88	0.41	2.60	0.01	0.90	-0.147	3.0E-01
Mtr.4948.1.S1_at	4.39	3.61	3.81	3.80	4.62	3.75	3.94	0.41	4.06	0.49	1.03	0.043	7.6E-01
Mtr.24414.1.S1_x_at	2.95	3.65	2.93	2.92	2.91	2.90	3.18	0.41	2.91	0.01	0.91	-0.128	3.1E-01
Mtr.27467.1.S1_s_at	3.11	3.81	3.09	3.72	3.06	4.01	3.33	0.41	3.60	0.49	1.08	0.109	5.1E-01
Mtr.9599.1.S1_at	9.44	9.34	10.09	9.69	9.69	9.39	9.62	0.41	9.59	0.17	1.00	-0.006	8.9E-01
Mtr.44651.1.S1_at	9.40	8.84	9.64	9.31	9.35	9.39	9.29	0.41	9.35	0.04	1.01	0.009	8.2E-01
Mtr.39292.1.S1_at	2.16	2.95	2.38	2.14	2.13	2.15	2.50	0.41	2.14	0.01	0.86	-0.224	2.0E-01
Mtr.1366.1.S1_at	2.19	2.89	2.18	2.17	2.17	2.17	2.42	0.41	2.17	0.00	0.90	-0.157	3.5E-01
Mtr.20920.1.S1_at	10.73	9.95	10.55	10.66	10.62	10.69	10.41	0.41	10.66	0.03	1.02	0.034	3.5E-01
Mtr.2792.1.S1_x_at	11.73	11.23	12.04	11.83	11.64	11.87	11.67	0.41	11.78	0.12	1.01	0.014	6.7E-01
Mtr.383.1.S1_at	9.56	9.94	10.38	9.87	9.81	9.72	9.96	0.41	9.80	0.08	0.98	-0.023	5.5E-01
Mtr.21553.1.S1_at	2.44	3.14	2.43	2.41	2.41	2.39	2.67	0.41	2.40	0.01	0.90	-0.153	3.2E-01
Mtr.23337.1.S1_at	7.46	6.67	6.90	6.79	6.72	6.83	7.01	0.41	6.78	0.05	0.97	-0.048	3.9E-01
Mtr.28745.1.S1_s_at	8.43	7.83	8.61	8.49	8.75	8.49	8.29	0.41	8.58	0.15	1.03	0.048	3.2E-01
Mtr.39397.1.S1_at	9.00	8.19	8.69	8.72	8.80	8.81	8.63	0.41	8.78	0.05	1.02	0.025	5.6E-01
Mtr.11729.1.S1_at	9.58	9.18	9.99	9.64	9.60	9.53	9.58	0.41	9.59	0.06	1.00	0.002	9.7E-01
Mtr.29448.1.S1_at	8.95	8.43	9.23	8.88	8.86	8.79	8.87	0.41	8.84	0.05	1.00	-0.004	9.2E-01
Mtr.28110.1.S1_at	4.74	4.03	4.73	4.74	4.73	5.06	4.50	0.41	4.84	0.19	1.08	0.107	2.5E-01
Mtr.31258.1.S1_at	7.82	7.09	7.77	7.84	7.75	7.97	7.56	0.41	7.85	0.11	1.04	0.055	3.0E-01
Mtr.38408.1.S1_at	10.59	10.00	10.79	10.52	10.54	10.42	10.46	0.41	10.49	0.06	1.00	0.005	8.9E-01
Mtr.21815.1.S1_at	7.22	7.17	7.90	7.24	7.37	7.27	7.43	0.41	7.29	0.07	0.98	-0.028	5.8E-01
Mtr.46187.1.S1_at	10.28	9.53	10.18	10.05	10.00	10.11	9.99	0.41	10.05	0.06	1.01	0.009	8.1E-01
Mtr.49855.1.S1_at	2.90	3.58	2.85	2.83	2.82	2.85	3.11	0.41	2.83	0.01	0.91	-0.134	3.1E-01
Mtr.37957.1.S1_at	11.97	11.67	12.47	12.29	12.31	12.11	12.04	0.41	12.24	0.11	1.02	0.024	4.6E-01
Mtr.25868.1.S1_s_at	9.48	9.28	10.07	9.64	9.80	9.92	9.61	0.41	9.79	0.14	1.02	0.027	5.1E-01
Mtr.1033.1.S1_s_at	11.92	11.17	11.83	11.76	11.56	11.39	11.64	0.41	11.57	0.18	0.99	-0.009	7.9E-01
Mtr.32391.1.S1_at	8.24	9.04	8.74	8.59	8.46	8.52	8.67	0.41	8.52	0.07	0.98	-0.025	5.6E-01
Mtr.51877.1.S1_at	9.29	8.75	9.55	9.20	9.18	9.13	9.19	0.41	9.17	0.04	1.00	-0.004	9.3E-01
Mtr.19014.1.S1_at	7.50	6.83	7.57	7.94	8.42	8.09	7.30	0.41	8.15	0.24	1.12	0.158	3.7E-02
Mtr.51388.1.S1_at	3.09	3.76	3.02	2.99	2.96	2.95	3.29	0.41	2.97	0.02	0.90	-0.149	2.4E-01
Mtr.27648.1.S1_at	7.72	8.19	8.53	8.17	8.85	8.18	8.15	0.41	8.40	0.39	1.03	0.044	4.8E-01
Mtr.27328.1.S1_at	6.14	5.51	6.26	6.16	5.98	6.18	5.97	0.41	6.11	0.11	1.02	0.032	6.1E-01
Mtr.20787.1.S1_at	8.58	7.88	8.59	8.67	8.48	8.73	8.35	0.41	8.63	0.13	1.03	0.046	3.3E-01
Mtr.13511.1.S1_at	9.64	10.45	9.96	9.94	9.94	9.99	10.02	0.41	9.96	0.03	0.99	-0.009	8.1E-01
Mtr.4307.1.S1_at	3.29	2.53	2.65	2.43	2.36	2.40	2.82	0.41	2.40	0.03	0.85	-0.233	1.5E-01
Mtr.14601.1.S1_s_at	3.44	3.72	4.25	3.67	4.80	3.47	3.80	0.41	3.98	0.72	1.05	0.066	7.3E-01
Mtr.33704.1.S1_at	5.24	4.61	5.37	5.23	5.23	5.10	5.07	0.41	5.19	0.07	1.02	0.032	6.6E-01
Mtr.27024.1.S1_at	3.78	3.08	3.78	3.80	3.91	3.72	3.55	0.41	3.81	0.10	1.07	0.103	3.4E-01
Mtr.7372.1.S1_at	7.86	7.10	7.74	7.76	7.80	7.96	7.57	0.41	7.84	0.10	1.04	0.051	3.2E-01
Mtr.43540.1.S1_at	11.32	10.54	11.12	11.15	11.08	11.16	10.99	0.41	11.13	0.04	1.01	0.018	5.9E-01
Mtr.219.1.S1_at	3.06	3.77	3.06	3.12	3.07	3.04	3.30	0.41	3.08	0.04	0.93	-0.098	4.1E-01
Mtr.51279.1.S1_s_at	2.47	3.19	2.50	2.46	2.36	2.46	2.72	0.41	2.43	0.06	0.89	-0.163	2.9E-01
Mtr.23072.1.S1_s_at	9.04	8.60	9.41	9.14	9.00	9.00	9.01	0.41	9.05	0.08	1.00	0.005	9.0E-01
Mtr.39001.1.S1_at	6.49	5.76	6.45	6.48	6.59	6.83	6.23	0.41	6.63	0.18	1.06	0.089	2.0E-01
Mtr.7563.1.S1_at	5.94	5.14	5.66	5.72	5.54	5.42	5.58	0.41	5.56	0.15	1.00	-0.005	9.4E-01
Mtr.32124.1.S1_at	9.14	8.33	8.66	8.01	7.51	7.91	8.71	0.41	7.81	0.27	0.90	-0.156	3.3E-02
Mtr.4037.1.S1_at	12.34	13.05	13.03	12.28	12.19	12.40	12.81	0.41	12.29	0.10	0.96	-0.059	1.0E-01
Mtr.35075.1.S1_s_at	7.31	6.78	7.58	7.38	7.51	7.31	7.22	0.41	7.40	0.10	1.03	0.036	5.0E-01
Mtr.16969.1.S1_at	7.00	6.79	7.58	6.73	7.09	7.06	7.13	0.41	6.96	0.20	0.98	-0.034	5.6E-01
Mtr.47607.1.S1_at	11.96	11.20	11.83	11.98	11.97	12.05	11.66	0.41	12.00	0.04	1.03	0.041	2.3E-01
Mtr.11755.1.S1_at	9.60	8.79	9.17	9.27	9.21	9.16	9.19	0.41	9.21	0.05	1.00	0.004	9.2E-01
Mtr.30950.1.S1_at	10.15	9.53	9.39	9.50	9.42	9.59	9.69	0.41	9.50	0.09	0.98	-0.028	4.8E-01
Mtr.40259.1.S1_s_at	9.55	8.98	8.77	9.30	9.27	9.64	9.10	0.41	9.41	0.20	1.03	0.048	3.1E-01
Mtr.13920.1.S1_at	8.89	8.10	8.36	8.81	8.39	8.88	8.45	0.41	8.70	0.26	1.03	0.042	4.3E-01
Mtr.11244.1.S1_at	2.87	2.18	2.16	2.13	2.78	2.39	2.40	0.41	2.43	0.33	1.01	0.018	9.3E-01
Mtr.8077.1.S1_at	2.56	3.27	2.56	2.53	2.52	3.35	2.80	0.41	2.80	0.47	1.00	0.002	9.9E-01
Mtr.47629.1.S1_at	6.22	6.00	6.79	6.41	6.36	6.34	6.34	0.41	6.37	0.04	1.00	0.007	9.1E-01
Mtr.28622.1.S1_at	8.79	8.13	8.87	8.65	8.74	8.79	8.60	0.41	8.73	0.07	1.02	0.022	6.1E-01
Mtr.9458.1.S1_at	8.35	7.85	8.65	8.47	8.39	8.36	8.28	0.41	8.41	0.06	1.02	0.022	6.2E-01
Mtr.41371.1.S1_s_at	9.20	8.45	8.56	8.94	8.81	9.02	8.74	0.41	8.92	0.11	1.02	0.030	4.9E-01
Mtr.35231.1.S1_s_at	3.32	3.64	2.84	3.70	5.08	4.50	3.27	0.41	4.43	0.69	1.35	0.438	6.6E-02
Mtr.44627.1.S1_at	11.62	10.86	10.99	11.39	11.37	11.37	11.16	0.41	11.38	0.01	1.02	0.028	4.1E-01
Mtr.21400.1.S1_at	8.29	7.52	8.13	8.37	8.50	8.33	7.98	0.41	8.40	0.09	1.05	0.074	1.6E-01
Mtr.38210.1.S1_at	10.42	9.63	9.87	10.18	10.31	10.32	9.97	0.41	10.27	0.08	1.03	0.042	2.8E-01
Mtr.52193.1.S1_at	9.79	9.82	10.51	9.90	9.74	9.73	10.04	0.41	9.79	0.10	0.98	-0.036	3.6E-01
Mtr.47932.1.S1_at	9.24	8.52	9.22	9.25	9.37	9.23	8.99	0.41	9.28	0.08	1.03	0.046	2.9E-01
Mtr.523.1.S1_at	2.90	3.57	2.84	2.81	2.81	2.74	3.10	0.41	2.79	0.04	0.90	-0.154	2.5E-01
Mtr.12971.1.S1_at	10.05	9.41	10.16	10.08	10.08	9.94	9.88	0.41	10.03	0.08	1.02	0.022	5.6E-01
Mtr.29852.1.S1_at	9.55	8.75	9.27	9.64	9.72	9.44	9.19	0.41	9.60	0.14	1.05	0.064	1.7E-01
Mtr.22707.1.S1_at	3.00	3.70	2.98	2.96	2.93	2.98	3.23	0.41	2.96	0.02	0.92	-0.126	3.1E-01
Mtr.30197.1.S1_at	11.37	10.94	10.56	11.09	11.38	11.38	10.96	0.41	11.28	0.16	1.03	0.043	2.6E-01
Mtr.6451.1.S1_at	2.41	3.10	2.39	2.38	2.38	2.38	2.63	0.41	2.38	0.00	0.90	-0.145	3.4E-01
Mtr.43114.1.S1_at	12.68	12.35	11.87	12.22	12.52	12.54	12.30	0.41	12.43	0.18	1.01	0.014	6.6E-01
Mtr.12191.1.S1_at	8.24	8.54	9.04	8.47	9.04	8.75	8.60	0.41	8.75	0.28	1.02	0.025	6.3E-01
Mtr.50404.1.S1_at	5.60	5.16	5.97	5.66	5.45	5.22	5.57	0.41	5.45	0.22	0.98	-0.034	6.6E-01
Mtr.12543.1.S1_at	12.78	11.97	12.42	12.51	12.03	12.38	12.39	0.41	12.31	0.24	0.99	-0.010	7.7E-01
Mtr.14260.1.S1_at	2.72	2.75	3.44	2.71	2.71	2.71	2.97	0.41	2.71	0.00	0.91	-0.133	3.3E-01
Mtr.5374.1.S1_at	2.61	3.30	2.59	2.59	2.57	2.54	2.83	0.41	2.57	0.02	0.91	-0.142	3.2E-01
Mtr.280.1.S1_at	11.46	10.66	11.00	11.13	11.16	11.21	11.04	0.41	11.17	0.04	1.01	0.016	6.2E-01
Mtr.11272.1.S1_at	9.32	10.04	9.36	9.62	9.09	9.29	9.57	0.41	9.33	0.27	0.97	-0.037	4.3E-01
Mtr.12033.1.S1_at	7.72	8.52	8.27	8.14	7.83	8.10	8.17	0.41	8.02	0.17	0.98	-0.026	5.9E-01
Mtr.44058.1.S1_at	6.03	6.03	6.73	5.98	5.90	6.03	6.26	0.41	5.97	0.06	0.95	-0.069	2.8E-01
Mtr.19233.1.S1_at	9.01	8.63	9.44	9.15	9.05	8.84	9.02	0.41	9.01	0.16	1.00	-0.001	9.8E-01
Mtr.19436.1.S1_at	2.94	3.60	2.86	2.95	2.81	2.80	3.13	0.41	2.85	0.08	0.91	-0.135	3.1E-01
Mtr.22080.1.S1_at	2.42	3.11	2.40	2.39	2.39	2.40	2.64	0.41	2.39	0.01	0.91	-0.144	3.4E-01
Mtr.37808.1.S1_at	7.04	7.10	7.77	7.03	6.92	6.91	7.30	0.41	6.95	0.07	0.95	-0.071	2.1E-01
Mtr.46440.1.S1_at	2.74	3.55	3.11	3.07	3.06	3.09	3.13	0.41	3.07	0.02	0.98	-0.027	8.2E-01
Mtr.12126.1.S1_at	9.05	8.24	8.58	8.92	8.63	8.88	8.63	0.41	8.81	0.16	1.02	0.030	5.1E-01
Mtr.33717.1.S1_s_at	10.27	10.87	11.04	10.66	10.95	10.93	10.72	0.41	10.85	0.16	1.01	0.016	6.6E-01
Mtr.7442.1.S1_at	11.70	11.00	11.71	11.66	11.34	11.43	11.47	0.41	11.48	0.16	1.00	0.001	9.8E-01
Mtr.43231.1.S1_s_at	13.22	12.56	13.31	13.56	13.68	13.66	13.03	0.41	13.63	0.07	1.05	0.065	6.4E-02
Mtr.40238.1.S1_at	13.93	13.12	13.52	13.67	13.50	13.72	13.53	0.41	13.63	0.11	1.01	0.011	6.9E-01
Mtr.50132.1.S1_s_at	9.21	8.57	9.33	9.17	9.07	9.22	9.04	0.41	9.15	0.08	1.01	0.018	6.5E-01
Mtr.20190.1.S1_at	8.08	7.46	8.22	8.24	8.11	8.12	7.92	0.41	8.16	0.07	1.03	0.043	3.7E-01
Mtr.45694.1.S1_s_at	2.60	3.29	2.57	2.57	2.55	2.55	2.82	0.41	2.56	0.01	0.91	-0.141	3.2E-01
Mtr.28835.1.S1_at	8.76	8.31	9.11	8.93	9.05	8.99	8.73	0.41	8.99	0.06	1.03	0.043	3.3E-01
Mtr.43131.1.S1_at	11.37	12.18	11.83	11.68	12.06	11.75	11.79	0.41	11.83	0.20	1.00	0.005	8.8E-01
Mtr.37572.1.S1_s_at	8.85	8.35	9.15	9.16	9.05	8.99	8.78	0.41	9.07	0.09	1.03	0.046	3.0E-01
Mtr.17949.1.S1_at	7.98	8.14	8.75	8.34	8.47	8.28	8.29	0.41	8.36	0.09	1.01	0.013	7.8E-01
Mtr.31706.1.S1_at	5.55	6.20	5.46	5.24	5.33	5.79	5.74	0.41	5.45	0.30	0.95	-0.074	3.8E-01
Mtr.23734.1.S1_at	6.53	6.36	7.13	6.11	6.01	5.89	6.68	0.41	6.01	0.11	0.90	-0.153	5.1E-02
Mtr.2379.1.S1_at	9.93	9.22	9.90	9.60	9.67	9.53	9.68	0.41	9.60	0.07	0.99	-0.013	7.4E-01
Mtr.43507.1.S1_at	12.55	13.36	12.92	12.96	13.27	13.22	12.94	0.41	13.15	0.17	1.02	0.023	4.5E-01
Mtr.39709.1.S1_at	5.98	5.40	5.20	5.68	5.76	5.70	5.53	0.41	5.71	0.04	1.03	0.047	4.8E-01
Mtr.27102.1.S1_at	8.00	7.19	7.67	8.11	8.13	8.23	7.62	0.41	8.16	0.06	1.07	0.098	8.5E-02
Mtr.13937.1.S1_at	8.01	7.46	8.25	8.15	8.03	8.02	7.91	0.41	8.07	0.08	1.02	0.029	5.4E-01
Mtr.27670.1.S1_at	9.69	8.89	9.18	9.84	9.47	10.08	9.25	0.41	9.80	0.31	1.06	0.083	1.4E-01
Mtr.7950.1.S1_at	10.11	9.33	9.91	10.04	10.13	10.02	9.79	0.41	10.06	0.06	1.03	0.040	3.1E-01
Mtr.9004.1.S1_at	11.05	10.74	11.55	11.43	11.29	11.17	11.11	0.41	11.30	0.13	1.02	0.024	5.0E-01

Mtr.11570.1.S1_at	4.02	3.89	4.65	4.51	4.25	4.27	4.19	0.41	4.34	0.14	1.04	0.053	5.6E-01
Mtr.40090.1.S1_at	9.50	9.07	9.88	9.60	9.81	9.65	9.48	0.41	9.69	0.11	1.02	0.030	4.5E-01
Mtr.46922.1.S1_at	4.62	5.06	5.43	4.70	4.86	4.20	5.04	0.41	4.59	0.35	0.91	-0.135	2.2E-01
Mtr.32810.1.S1_x_at	3.06	3.21	3.83	3.17	3.21	3.18	3.36	0.41	3.19	0.02	0.95	-0.079	4.9E-01
Mtr.35728.1.S1_at	4.61	3.89	3.92	4.04	4.04	4.18	4.14	0.41	4.09	0.08	0.99	-0.018	8.4E-01
Mtr.18559.1.S1_at	6.29	5.60	6.31	6.32	6.32	6.31	6.06	0.41	6.32	0.01	1.04	0.059	3.4E-01
Mtr.49258.1.S1_at	8.92	8.14	8.35	9.02	8.91	9.04	8.47	0.40	8.99	0.07	1.06	0.086	9.4E-02
Mtr.39805.1.S1_at	8.01	8.18	8.78	8.36	8.40	8.35	8.32	0.40	8.37	0.03	1.01	0.008	8.6E-01
Mtr.640.1.S1_s_at	8.12	7.34	7.91	8.11	7.99	8.09	7.79	0.40	8.06	0.07	1.04	0.050	3.1E-01
Mtr.15118.1.S1_x_at	6.33	7.04	6.36	6.08	6.32	6.30	6.58	0.40	6.24	0.13	0.95	-0.077	2.4E-01
Mtr.35185.1.S1_at	7.43	7.29	6.67	7.54	8.04	7.57	7.13	0.40	7.72	0.28	1.08	0.115	1.1E-01
Mtr.34901.1.S1_s_at	11.49	10.71	10.90	10.93	10.97	11.19	11.03	0.40	11.03	0.14	1.00	0.000	9.9E-01
Mtr.43183.1.S1_at	13.36	12.79	13.57	13.46	13.40	13.51	13.24	0.40	13.46	0.05	1.02	0.023	4.1E-01
Mtr.2725.1.S1_at	3.44	3.02	3.83	3.46	3.80	3.47	3.43	0.40	3.58	0.20	1.04	0.061	6.0E-01
Mtr.46396.1.S1_s_at	12.23	11.69	12.48	12.36	12.21	12.26	12.13	0.40	12.28	0.07	1.01	0.017	5.7E-01
Mtr.10213.1.S1_at	7.79	8.58	8.02	7.51	7.23	7.65	8.13	0.40	7.47	0.21	0.92	-0.123	6.5E-02
Mtr.6484.1.S1_at	14.11	13.38	13.45	13.66	13.44	13.63	13.65	0.40	13.58	0.12	0.99	-0.007	7.9E-01
Mtr.9975.1.S1_at	8.35	8.26	9.00	8.85	8.95	8.84	8.53	0.40	8.88	0.06	1.04	0.057	2.2E-01
Mtr.37856.1.S1_at	9.84	9.09	9.72	9.82	9.64	9.77	9.55	0.40	9.74	0.09	1.02	0.029	4.7E-01
Mtr.33025.1.S1_s_at	10.63	9.84	10.09	10.25	10.15	10.30	10.19	0.40	10.23	0.08	1.00	0.006	8.7E-01
Mtr.15618.1.S1_at	9.55	10.36	10.01	9.76	9.93	9.79	9.97	0.40	9.82	0.09	0.98	-0.022	5.6E-01
Mtr.41650.1.S1_at	9.60	8.96	9.71	9.80	9.62	9.56	9.43	0.40	9.66	0.12	1.02	0.035	4.0E-01
Mtr.21074.1.S1_at	2.22	2.91	2.21	2.21	2.21	2.21	2.45	0.40	2.21	0.00	0.90	-0.148	3.6E-01
Mtr.16874.1.S1_at	7.60	7.83	8.38	7.31	7.49	7.67	7.94	0.40	7.49	0.18	0.94	-0.084	1.5E-01
Mtr.40597.1.S1_at	10.81	10.00	10.47	10.66	10.89	10.70	10.43	0.40	10.75	0.12	1.03	0.044	2.5E-01
Mtr.28117.1.S1_at	5.27	5.23	5.95	5.79	5.70	6.38	5.48	0.40	5.95	0.37	1.09	0.119	2.1E-01
Mtr.25425.1.S1_at	1.92	2.62	1.91	1.91	1.91	1.91	2.15	0.40	1.91	0.00	0.89	-0.171	3.6E-01
Mtr.17074.1.S1_at	9.61	8.98	9.73	9.41	8.92	9.08	9.44	0.40	9.14	0.25	0.97	-0.047	3.3E-01
Mtr.49435.1.S1_at	2.79	3.48	2.76	2.72	2.72	2.72	3.01	0.40	2.72	0.00	0.90	-0.145	2.8E-01
Mtr.6360.1.S1_at	5.38	5.02	4.58	5.59	5.55	5.43	4.99	0.40	5.52	0.09	1.11	0.145	9.1E-02
Mtr.37262.1.S1_at	10.55	9.76	10.31	10.38	10.28	10.21	10.21	0.40	10.29	0.09	1.01	0.012	7.4E-01
Mtr.24464.1.S1_at	8.64	7.97	8.70	8.79	9.30	9.09	8.44	0.40	9.06	0.26	1.07	0.103	8.6E-02
Mtr.14118.1.S1_at	8.65	8.17	8.98	8.70	8.87	8.88	8.60	0.40	8.82	0.10	1.03	0.036	4.2E-01
Mtr.35418.1.S1_at	11.19	10.42	10.59	11.07	11.27	11.36	10.73	0.40	11.23	0.15	1.05	0.065	1.2E-01
Mtr.39522.1.S1_s_at	8.35	7.59	8.19	8.43	8.48	8.34	8.04	0.40	8.42	0.07	1.05	0.065	1.9E-01
Mtr.18669.1.S1_at	3.70	4.27	3.49	3.65	3.68	3.74	3.82	0.40	3.69	0.04	0.97	-0.050	6.1E-01
Mtr.35299.1.S1_at	8.82	9.22	9.63	9.76	10.23	9.40	9.22	0.40	9.79	0.41	1.06	0.087	1.6E-01
Mtr.44829.1.S1_at	4.41	3.79	4.54	4.39	4.39	4.81	4.25	0.40	4.53	0.24	1.07	0.093	3.5E-01
Mtr.33151.1.S1_at	9.92	10.61	10.63	10.59	10.99	10.73	10.39	0.40	10.77	0.20	1.04	0.052	2.2E-01
Mtr.40915.1.S1_at	10.21	9.45	10.05	10.10	10.13	10.19	9.91	0.40	10.14	0.04	1.02	0.034	3.8E-01
Mtr.43891.1.S1_at	7.80	7.68	8.43	8.26	8.19	8.19	7.97	0.40	8.21	0.04	1.03	0.043	3.6E-01
Mtr.32473.1.S1_at	5.94	5.62	6.42	6.54	6.21	6.61	6.00	0.40	6.46	0.21	1.08	0.107	1.6E-01
Mtr.35557.1.S1_s_at	12.42	13.13	13.11	12.38	11.94	12.34	12.88	0.40	12.22	0.24	0.95	-0.077	7.0E-02
Mtr.45860.1.S1_x_at	3.49	2.79	3.48	3.71	3.69	3.46	3.25	0.40	3.62	0.14	1.11	0.154	2.1E-01
Mtr.985.1.S1_at	10.01	10.76	10.65	10.65	10.80	10.82	10.48	0.40	10.76	0.10	1.03	0.038	3.1E-01
Mtr.36059.1.S1_at	3.06	3.76	3.06	3.07	3.02	3.05	3.30	0.40	3.05	0.03	0.92	-0.114	3.4E-01
Mtr.7958.1.S1_at	11.90	11.68	12.47	12.24	12.61	12.10	12.02	0.40	12.32	0.26	1.03	0.036	3.4E-01
Mtr.18608.1.S1_at	8.73	8.15	8.92	8.86	8.59	8.68	8.60	0.40	8.71	0.14	1.01	0.019	6.7E-01
Mtr.20726.1.S1_at	9.51	8.70	9.06	9.31	9.27	9.28	9.09	0.40	9.29	0.02	1.02	0.031	4.5E-01
Mtr.33431.1.S1_at	8.01	7.20	7.55	7.83	7.81	7.97	7.59	0.40	7.87	0.09	1.04	0.053	3.0E-01
Mtr.38801.1.S1_at	12.19	11.43	11.58	11.90	11.99	11.88	11.73	0.40	11.92	0.06	1.02	0.023	4.6E-01
Mtr.43270.1.S1_at	12.43	11.62	12.08	11.98	12.01	12.15	12.04	0.40	12.04	0.09	1.00	0.000	1.0E+00
Mtr.28790.1.S1_at	9.76	9.96	9.18	9.21	9.38	9.36	9.64	0.40	9.32	0.09	0.97	-0.048	2.5E-01
Mtr.7663.1.S1_at	3.64	3.23	2.83	2.83	2.69	2.82	3.23	0.40	2.78	0.08	0.86	-0.217	1.3E-01
Mtr.40759.1.S1_at	9.61	9.05	9.83	9.38	9.33	9.21	9.50	0.40	9.31	0.09	0.98	-0.029	4.7E-01
Mtr.37168.1.S1_at	11.12	11.84	11.80	11.74	11.75	11.58	11.59	0.40	11.69	0.09	1.01	0.013	6.9E-01
Mtr.42890.1.S1_at	8.38	7.58	7.88	8.05	7.58	7.75	7.95	0.40	7.79	0.23	0.98	-0.028	6.1E-01
Mtr.18297.1.S1_at	8.99	8.34	9.07	8.98	8.99	8.72	8.80	0.40	8.90	0.15	1.01	0.016	7.2E-01
Mtr.32260.1.S1_at	6.44	7.12	6.41	7.27	8.31	7.23	6.66	0.40	7.60	0.61	1.14	0.192	8.9E-02
Mtr.28671.1.S1_at	9.71	9.69	10.40	10.01	9.88	9.73	9.94	0.40	9.87	0.14	0.99	-0.009	8.1E-01
Mtr.8221.1.S1_at	2.73	3.39	2.67	2.68	2.65	2.67	2.93	0.40	2.67	0.01	0.91	-0.135	3.3E-01
Mtr.46858.1.S1_at	2.70	3.38	2.67	2.69	2.68	2.69	2.92	0.40	2.69	0.01	0.92	-0.118	3.8E-01
Mtr.44516.1.S1_at	11.39	10.61	11.18	11.33	11.54	11.46	11.06	0.40	11.44	0.11	1.03	0.049	1.9E-01
Mtr.43457.1.S1_s_at	9.63	9.10	9.89	9.70	9.58	9.60	9.54	0.40	9.63	0.06	1.01	0.013	7.3E-01
Mtr.35495.1.S1_at	10.78	9.99	10.50	10.32	10.46	10.29	10.42	0.40	10.36	0.09	0.99	-0.009	7.9E-01
Mtr.21498.1.S1_at	10.33	10.92	11.10	9.97	10.08	10.02	10.78	0.40	10.03	0.06	0.93	-0.105	3.2E-02
Mtr.5588.1.S1_at	7.91	7.36	8.15	8.04	8.02	8.03	7.81	0.40	8.03	0.01	1.03	0.041	3.9E-01
Mtr.30842.1.S1_at	2.23	2.92	2.22	2.21	2.21	2.21	2.46	0.40	2.21	0.00	0.90	-0.149	3.6E-01
Mtr.21709.1.S1_at	14.15	13.44	13.47	13.85	13.65	13.80	13.69	0.40	13.77	0.10	1.01	0.009	7.5E-01
Mtr.8848.1.S1_at	10.00	10.81	10.45	10.03	10.27	10.30	10.42	0.40	10.20	0.15	0.98	-0.031	4.3E-01
Mtr.31060.1.S1_at	8.27	9.07	8.58	8.36	8.59	8.26	8.64	0.40	8.41	0.17	0.97	-0.040	4.1E-01
Mtr.42012.1.S1_at	8.44	9.24	8.76	8.70	8.80	8.72	8.81	0.40	8.74	0.05	0.99	-0.012	7.7E-01
Mtr.1843.1.S1_at	3.46	2.81	3.55	3.44	3.71	3.44	3.27	0.40	3.53	0.16	1.08	0.109	3.6E-01
Mtr.41255.1.S1_at	13.69	13.02	12.98	13.21	13.33	13.23	13.23	0.40	13.26	0.06	1.00	0.003	9.1E-01
Mtr.6533.1.S1_at	5.92	5.16	5.79	4.83	4.78	4.83	5.62	0.40	4.81	0.03	0.86	-0.224	2.6E-02
Mtr.28638.1.S1_at	7.85	7.18	7.12	8.06	7.78	8.09	7.38	0.40	7.98	0.17	1.08	0.112	7.7E-02
Mtr.15717.1.S1_at	12.47	11.66	12.11	12.20	11.94	11.96	12.08	0.40	12.03	0.14	1.00	-0.006	8.5E-01
Mtr.24064.1.S1_at	2.42	3.11	2.40	2.39	2.39	2.38	2.64	0.40	2.39	0.01	0.90	-0.148	3.3E-01
Mtr.45084.1.S1_at	6.54	5.82	6.50	6.41	6.64	6.45	6.29	0.40	6.50	0.13	1.03	0.048	4.3E-01
Mtr.28477.1.S1_at	9.75	10.49	9.83	9.92	10.27	10.03	10.02	0.40	10.07	0.18	1.01	0.007	8.5E-01
Mtr.31486.1.S1_at	6.10	6.22	6.85	6.17	6.26	6.16	6.39	0.40	6.20	0.05	0.97	-0.044	4.6E-01
Mtr.1751.1.S1_at	7.02	7.04	7.73	7.21	7.61	7.21	7.26	0.40	7.34	0.23	1.01	0.016	7.8E-01
Mtr.34942.1.S1_at	7.03	6.32	7.00	7.00	6.98	6.84	6.78	0.40	6.94	0.09	1.02	0.034	5.4E-01
Mtr.12674.1.S1_at	11.19	10.75	10.38	11.02	11.22	11.20	10.77	0.40	11.15	0.11	1.03	0.049	2.0E-01
Mtr.49410.1.S1_at	3.71	3.09	2.95	2.92	3.14	2.98	3.25	0.40	3.01	0.11	0.93	-0.109	3.8E-01
Mtr.15010.1.S1_s_at	9.44	9.12	8.64	8.81	8.97	9.08	9.07	0.40	8.95	0.14	0.99	-0.018	6.8E-01
Mtr.18580.1.S1_at	2.78	3.47	2.76	2.75	2.74	2.73	3.01	0.40	2.74	0.01	0.91	-0.133	3.2E-01
Mtr.40768.1.S1_at	9.04	8.62	9.42	9.08	9.17	8.90	9.02	0.40	9.05	0.14	1.00	0.004	9.2E-01
Mtr.37333.1.S1_at	11.90	12.47	12.68	12.34	12.41	12.22	12.35	0.40	12.32	0.10	1.00	-0.003	9.1E-01
Mtr.17409.1.S1_s_at	9.79	9.03	9.19	9.41	9.59	9.48	9.34	0.40	9.49	0.09	1.02	0.023	5.6E-01
Mtr.29161.1.S1_at	8.64	8.10	8.89	7.95	8.02	8.01	8.55	0.40	7.99	0.04	0.93	-0.097	7.6E-02
Mtr.12926.1.S1_at	13.10	12.30	12.80	12.81	12.89	12.82	12.73	0.40	12.84	0.05	1.01	0.012	6.7E-01
Mtr.39691.1.S1_at	7.63	7.65	8.34	8.10	7.96	8.12	7.87	0.40	8.06	0.09	1.02	0.034	4.7E-01
Mtr.13050.1.S1_at	11.53	12.19	12.26	11.84	11.96	12.03	11.99	0.40	11.94	0.09	1.00	-0.006	8.5E-01
Mtr.18036.1.S1_s_at	10.14	9.34	9.62	9.92	9.85	9.82	9.70	0.40	9.86	0.06	1.02	0.024	5.2E-01
Mtr.41691.1.S1_at	7.62	8.41	7.87	7.38	7.57	7.39	7.97	0.40	7.44	0.11	0.93	-0.098	9.4E-02
Mtr.36813.1.S1_at	8.77	8.41	9.21	9.11	8.69	8.72	8.79	0.40	8.84	0.23	1.01	0.008	8.7E-01
Mtr.17703.1.S1_at	11.04	10.24	10.58	10.69	10.62	10.63	10.62	0.40	10.65	0.04	1.00	0.004	9.0E-01
Mtr.1221.1.S1_at	2.37	3.05	2.34	2.33	2.32	2.33	2.58	0.40	2.33	0.01	0.90	-0.151	3.3E-01
Mtr.23495.1.S1_s_at	8.12	8.79	8.84	8.40	8.36	8.56	8.58	0.40	8.44	0.11	0.98	-0.024	5.8E-01
Mtr.11576.1.S1_at	6.14	5.42	6.09	5.96	5.66	5.50	5.88	0.40	5.71	0.23	0.97	-0.044	5.5E-01
Mtr.22610.1.S1_at	6.20	5.40	5.88	5.97	5.95	5.94	5.83	0.40	5.95	0.01	1.02	0.031	6.1E-01
Mtr.18401.1.S1_at	2.17	2.86	2.16	2.16	2.15	2.17	2.40	0.40	2.16	0.01	0.90	-0.152	3.6E-01
Mtr.27863.1.S1_at	9.77	8.98	9.22	9.65	9.53	9.53	9.32	0.40	9.57	0.07	1.03	0.038	3.5E-01
Mtr.12115.1.S1_s_at	8.98	8.21	8.78	8.77	8.64	8.73	8.66	0.40	8.71	0.06	1.01	0.010	8.2E-01
Mtr.15119.1.S1_s_at	2.97	3.64	2.91	2.89	2.84	2.90	3.17	0.40	2.88	0.03	0.91	-0.141	2.7E-01
Mtr.229.1.S1_at	5.31	4.70	5.47	5.23	5.19	4.99	5.16	0.40	5.13	0.13	1.00	-0.007	9.3E-01
Mtr.1780.1.S1_at	11.29	10.49	10.97	11.19	11.03	11.25	10.92	0.40	11.16	0.11	1.02	0.031	3.8E-01
Mtr.3077.1.S1_at	5.23	5.27	5.94	5.04	5.37	5.07	5.48	0.40	5.16	0.19	0.94	-0.086	2.8E-01
Mtr.41171.1.S1_at	8.60	8.17	8.97	8.79	8.73	8.72	8.58	0.40	8.75	0.04	1.02	0.028	5.2E-01
Mtr.17485.1.S1_at	2.57	3.26	2.55	2.55	2.52	2.55	2.79	0.40	2.54	0.01	0.91	-0.137	3.4E-01
Mtr.45774.1.S1_x_at	2.29	2.98	2.28	2.29	2.27	2.27	2.52	0.40	2.27	0.01	0.90	-0.147	3.5E-01
Mtr.17501.1.S1_at	9.41	8.82	9.59	9.53	9.44	9.57	9.28	0.40	9.51	0.07	1.03	0.037	3.7E-01
Mtr.9574.1.S1_at	8.05	7.28	7.86	7.97	7.67	7.85	7.73	0.40	7.83	0.15	1.01	0.019	7.1E-01
Mtr.36041.1.S1_at	9.14	8.34	8.82	9.08	9.04	9.30	8.77	0.40	9.14	0.14	1.04	0.060	2.0E-01
Mtr.15956.1.S1_at	7.70	7.08	7.83	7.57	7.51	7.42	7.54	0.40	7.50	0.08	1.00	-0.007	8.8E-01
Mtr.3869.1.S1_at	10.41	11.09	11.12	10.55	10.71	10.60	10.87	0.40	10.62	0.08	0.98	-0.034	3.4E-01
Mtr.15286.1.S1_at	3.63	4.32	3.62	3.62	3.17	3.61	3.86	0.40	3.47	0.25	0.90	-0.153	2.3E-01
Mtr.41225.1.S1_at	9.14	9.78	9.88	9.64	9.60	9.55	9.60	0.40	9.60	0.05	1.00	-0.001	9.9E-01
Mtr.28731.1.S1_at	3.47	3.24	4.02	3.91	3.59	3.95	3.58	0.40	3.82	0.19	1.07	0.094	4.0E-01
Mtr.22830.1.S1_at	4.41	5.12	5.07	4.86	4.86	4.87	4.87	0.40	4.86	0.01	1.00	-0.001	9.8E-01
Mtr.14451.1.S1_s_at	9.83	9.14	9.85	9.74	9.60	9.75	9.61	0.40	9.70	0.08	1.01	0.013	7.3E-01
Mtr.45461.1.S1_at	8.97	8.43	9.22	8.81	8.92	8.91	8.87	0.40	8.88	0.06	1.00	0.001	9.8E-01
Mtr.27638.1.S1_at	7.48	8.26	8.01	7.63	8.09	7.51	7.92	0.40	7.74	0.30	0.98	-0.032	5.9E-01
Mtr.4772.1.S1_at	9.20	9.97	9.81	8.98	9.54	9.31	9.66	0.40	9.28	0.28	0.96	-0.058	2.5E-01
Mtr.47907.1.S1_at	2.99	3.73	3.62	4.04	4.13	3.29	3.45	0.40	3.82	0.46	1.11	0.149	3.5E-01
Mtr.13677.1.S1_at	9.29	8.70	9.46	9.42	9.65	9.57	9.15	0.40	9.55	0.12	1.04	0.061	1.8E-01
Mtr.20167.1.S1_at	8.32	7.70	8.44	8.28	8.37	8.25	8.15	0.40	8.30	0.06	1.02	0.025	5.7E-01
Mtr.8666.1.S1_s_at	9.77	10.56	10.04	10.63	10.73	10.37	10.13	0.40	10.58	0.19	1.04	0.063	1.5E-01
Mtr.8358.1.S1_at	6.06	5.68	6.48	5.60	7.42	6.14	6.07	0.40	6.39	0.93	1.05	0.073	6.2E-01
Mtr.33907.1.S1_at	3.36	2.77	2.59	2.66	2.66	2.65	2.91	0.40	2.65	0.01	0.91	-0.131	3.4E-01

Mtr.8531.1.S1_at	13.83	13.03	13.48	13.64	13.38	13.67	13.45	0.40	13.56	0.16	1.01	0.012	6.6E-01
Mtr.2858.1.S1_at	7.72	7.52	6.95	6.95	7.45	7.41	7.40	0.40	7.27	0.28	0.98	-0.025	6.7E-01
Mtr.14638.1.S1_s_at	9.20	8.40	8.86	9.09	8.67	9.04	8.82	0.40	8.93	0.23	1.01	0.018	7.0E-01
Mtr.49509.1.S1_at	9.88	9.22	9.94	9.89	9.85	9.74	9.68	0.40	9.83	0.08	1.02	0.022	5.6E-01
Mtr.40911.1.S1_s_at	14.66	14.04	14.79	14.75	14.59	14.68	14.49	0.40	14.67	0.08	1.01	0.018	4.9E-01
Mtr.47222.1.S1_at	7.40	7.13	6.61	7.10	7.02	6.90	7.04	0.40	7.01	0.10	0.99	-0.008	8.8E-01
Mtr.36064.1.S1_at	3.08	3.31	2.53	2.66	2.52	2.47	2.98	0.40	2.55	0.10	0.86	-0.221	1.5E-01
Mtr.49103.1.S1_s_at	10.35	10.73	11.15	10.51	10.54	10.53	10.74	0.40	10.53	0.02	0.98	-0.030	4.0E-01
Mtr.3034.1.S1_at	8.50	7.79	8.45	8.44	8.38	8.75	8.25	0.40	8.52	0.20	1.03	0.048	3.4E-01
Mtr.25842.1.S1_at	7.29	7.58	8.08	7.57	7.65	7.32	7.65	0.40	7.52	0.17	0.98	-0.026	6.2E-01
Mtr.26988.1.S1_at	8.69	7.90	8.17	8.66	9.08	8.75	8.25	0.40	8.83	0.22	1.07	0.098	9.3E-02
Mtr.27281.1.S1_at	2.94	3.62	2.92	2.90	2.85	2.94	3.16	0.40	2.90	0.04	0.92	-0.125	3.2E-01
Mtr.31457.1.S1_at	8.32	7.55	8.15	8.41	8.43	8.39	8.01	0.40	8.41	0.02	1.05	0.071	1.6E-01
Mtr.47441.1.S1_at	7.13	6.51	7.26	7.62	7.43	7.63	6.97	0.40	7.56	0.11	1.08	0.117	7.1E-02
Mtr.22656.1.S1_at	3.02	3.69	2.98	2.97	2.91	2.91	3.23	0.40	2.93	0.03	0.91	-0.141	2.6E-01
Mtr.10404.1.S1_at	9.25	8.46	8.95	8.88	8.82	9.03	8.89	0.40	8.91	0.11	1.00	0.004	9.2E-01
Mtr.11280.1.S1_at	3.82	3.40	3.02	3.29	3.42	3.36	3.41	0.40	3.36	0.06	0.98	-0.023	8.3E-01
Mtr.39923.1.S1_at	10.68	9.96	10.63	10.60	10.41	10.25	10.42	0.40	10.42	0.18	1.00	0.000	9.9E-01
Mtr.17491.1.S1_at	9.35	8.67	9.38	9.46	9.17	9.21	9.14	0.40	9.28	0.16	1.02	0.023	5.9E-01
Mtr.32194.1.S1_at	7.06	6.81	7.59	7.84	8.10	7.42	7.15	0.40	7.78	0.34	1.09	0.122	1.1E-01
Mtr.31693.1.S1_at	9.12	8.96	9.72	8.86	8.44	9.05	9.27	0.40	8.78	0.32	0.95	-0.077	1.8E-01
Mtr.50613.1.S1_at	2.82	3.51	2.81	2.81	2.79	2.81	3.05	0.40	2.80	0.01	0.92	-0.122	3.4E-01
Mtr.14769.1.S1_at	12.40	12.96	13.17	12.65	12.66	12.55	12.84	0.40	12.62	0.06	0.98	-0.025	3.9E-01
Mtr.48752.1.S1_at	2.68	3.36	2.66	2.64	2.71	2.60	2.90	0.40	2.65	0.06	0.91	-0.131	3.4E-01
Mtr.28479.1.S1_at	6.84	7.25	7.64	8.17	8.39	8.19	7.25	0.40	8.25	0.12	1.14	0.187	1.4E-02
Mtr.19348.1.S1_x_at	8.87	8.22	8.14	8.08	8.43	8.35	8.41	0.40	8.28	0.18	0.98	-0.022	6.5E-01
Mtr.15539.1.S1_at	8.89	8.95	9.61	9.03	9.36	9.15	9.15	0.40	9.18	0.16	1.00	0.005	9.1E-01
Mtr.3498.1.S1_at	2.09	2.78	2.08	2.08	2.08	2.08	2.32	0.40	2.08	0.00	0.90	-0.156	3.6E-01
Mtr.51592.1.S1_at	2.53	3.22	2.52	2.52	2.52	2.52	2.76	0.40	2.52	0.00	0.91	-0.130	3.6E-01
Mtr.34048.1.S1_at	2.70	3.38	2.67	2.66	2.66	2.65	2.92	0.40	2.66	0.01	0.91	-0.136	3.2E-01
Mtr.17715.1.S1_x_at	2.50	3.19	2.49	2.54	2.45	2.47	2.72	0.40	2.49	0.05	0.91	-0.129	3.7E-01
Mtr.15172.1.S1_at	8.02	7.22	7.58	8.07	8.14	8.32	7.61	0.40	8.17	0.13	1.07	0.103	8.0E-02
Mtr.20315.1.S1_at	10.17	10.18	10.87	10.65	10.67	10.84	10.40	0.40	10.72	0.10	1.03	0.044	2.5E-01
Mtr.27234.1.S1_at	1.99	2.67	1.97	1.97	1.97	1.97	2.21	0.40	1.97	0.00	0.89	-0.165	3.6E-01
Mtr.23716.1.S1_s_at	2.97	3.63	2.90	2.90	2.90	2.89	3.16	0.40	2.90	0.01	0.92	-0.127	3.1E-01
Mtr.1891.1.S1_at	6.99	7.25	7.77	7.22	7.30	6.94	7.34	0.40	7.15	0.19	0.98	-0.036	5.2E-01
Mtr.26950.1.S1_at	8.61	7.82	8.08	8.54	8.57	8.44	8.17	0.40	8.52	0.07	1.04	0.060	2.1E-01
Mtr.3865.1.S1_at	2.81	3.48	2.77	2.76	2.76	3.02	3.02	0.40	2.85	0.15	0.94	-0.086	5.2E-01
Mtr.9787.1.S1_at	8.99	8.85	9.60	9.16	8.96	9.17	9.15	0.40	9.10	0.12	0.99	-0.008	8.5E-01
Mtr.39681.1.S1_at	9.77	9.02	9.63	9.73	9.57	9.64	9.47	0.40	9.64	0.08	1.02	0.026	5.1E-01
Mtr.9535.1.S1_at	4.66	5.19	5.44	5.09	5.09	5.09	5.09	0.40	5.09	0.00	1.00	-0.001	9.9E-01
Mtr.5721.1.S1_s_at	9.06	9.80	9.70	9.18	9.33	9.37	9.52	0.40	9.29	0.10	0.98	-0.034	4.0E-01
Mtr.6158.1.S1_at	5.91	5.97	6.63	6.05	6.85	5.94	6.17	0.40	6.28	0.49	1.02	0.026	7.7E-01
Mtr.22341.1.S1_at	5.18	4.52	4.46	4.42	4.39	4.61	4.72	0.40	4.47	0.12	0.95	-0.078	3.6E-01
Mtr.46661.1.S1_x_at	4.06	3.69	3.26	3.69	3.08	3.31	3.67	0.40	3.36	0.31	0.92	-0.126	3.5E-01
Mtr.16859.1.S1_at	11.95	12.30	12.75	13.32	13.83	13.61	12.33	0.40	13.59	0.26	1.10	0.140	1.0E-02
Mtr.41507.1.S1_at	9.46	8.87	9.64	9.59	9.63	9.60	9.32	0.40	9.61	0.02	1.03	0.043	2.9E-01
Mtr.11453.1.S1_at	8.00	7.38	8.13	8.03	7.72	7.91	7.84	0.40	7.89	0.15	1.01	0.009	8.5E-01
Mtr.30473.1.S1_at	3.59	2.79	3.13	5.39	6.20	5.67	3.17	0.40	5.75	0.41	1.81	0.860	1.4E-03
Mtr.1508.1.S1_at	7.84	7.04	7.53	7.80	8.09	7.53	7.47	0.40	7.81	0.28	1.05	0.064	3.0E-01
Mtr.11510.1.S1_at	11.48	11.25	10.70	10.97	10.20	11.04	11.14	0.40	10.74	0.47	0.96	-0.054	3.2E-01
Mtr.9434.1.S1_at	6.12	5.41	6.09	6.09	6.32	6.11	5.87	0.40	6.17	0.13	1.05	0.072	2.8E-01
Mtr.9632.1.S1_at	9.70	8.97	9.61	9.50	9.57	9.44	9.43	0.40	9.50	0.06	1.01	0.011	7.6E-01
Mtr.37753.1.S1_at	6.10	5.48	5.35	6.17	7.00	6.47	5.65	0.40	6.55	0.42	1.16	0.214	5.5E-02
Mtr.41684.1.S1_at	6.51	5.73	5.96	5.80	6.25	6.31	6.06	0.40	6.12	0.28	1.01	0.013	8.5E-01
Mtr.21117.1.S1_at	3.12	3.78	3.06	2.98	3.06	2.97	3.32	0.40	3.00	0.05	0.90	-0.147	2.4E-01
Mtr.44397.1.S1_at	8.02	7.30	7.97	7.80	8.07	7.92	7.76	0.40	7.93	0.14	1.02	0.031	5.3E-01
Mtr.32537.1.S1_at	7.21	7.98	7.78	7.71	8.19	8.02	7.66	0.40	7.97	0.24	1.04	0.058	3.1E-01
Mtr.14191.1.S1_at	11.53	10.77	11.38	11.20	11.23	11.35	11.23	0.40	11.26	0.08	1.00	0.004	8.9E-01
Mtr.32429.1.S1_at	5.50	4.75	5.35	5.60	5.74	5.77	5.20	0.40	5.70	0.09	1.10	0.132	1.0E-01
Mtr.42589.1.S1_s_at	7.67	7.14	7.93	7.60	7.67	7.27	7.58	0.40	7.51	0.21	0.99	-0.013	8.1E-01
Mtr.45839.1.S1_at	10.91	11.20	11.70	11.37	11.27	11.29	11.27	0.40	11.31	0.05	1.00	0.005	8.7E-01
Mtr.4865.1.S1_at	7.72	7.87	8.47	8.34	8.02	7.67	8.02	0.40	8.01	0.34	1.00	-0.001	9.9E-01
Mtr.33844.1.S1_s_at	10.12	9.45	10.16	10.19	10.21	10.18	9.91	0.40	10.19	0.01	1.03	0.040	2.9E-01
Mtr.27827.1.S1_at	8.25	8.08	8.84	8.53	8.11	8.30	8.39	0.40	8.31	0.21	0.99	-0.013	7.9E-01
Mtr.2720.1.S1_at	9.14	8.93	9.70	9.54	9.52	9.36	9.26	0.40	9.47	0.10	1.02	0.034	4.1E-01
Mtr.10471.1.S1_at	6.26	5.81	6.60	6.01	6.67	6.50	6.22	0.40	6.39	0.34	1.03	0.039	6.1E-01
Mtr.2685.1.S1_at	8.59	9.07	9.39	9.28	9.14	9.03	9.02	0.40	9.15	0.12	1.01	0.021	6.2E-01
Mtr.49980.1.S1_at	3.22	3.95	3.86	2.93	3.17	3.00	3.67	0.40	3.03	0.12	0.83	-0.276	5.7E-02
Mtr.4646.1.S1_at	9.67	9.13	9.91	9.60	9.48	9.68	9.57	0.40	9.59	0.10	1.00	0.002	9.5E-01
Mtr.27073.1.S1_at	7.25	6.56	7.26	7.35	7.32	7.32	7.03	0.40	7.33	0.02	1.04	0.061	2.6E-01
Mtr.13757.1.S1_at	12.36	12.12	11.58	11.95	11.56	12.09	12.02	0.40	11.87	0.27	0.99	-0.019	6.1E-01
Mtr.31914.1.S1_at	12.24	13.03	12.66	12.27	12.51	12.52	12.64	0.40	12.43	0.14	0.98	-0.024	4.3E-01
Mtr.28071.1.S1_at	7.12	6.36	6.95	6.71	6.98	6.98	6.81	0.40	6.89	0.15	1.01	0.017	7.6E-01
Mtr.39413.1.S1_at	7.77	7.41	8.20	7.90	7.88	7.83	7.79	0.40	7.87	0.04	1.01	0.014	7.7E-01
Mtr.38397.1.S1_at	11.67	10.89	11.13	11.44	11.23	11.43	11.23	0.40	11.37	0.12	1.01	0.018	5.9E-01
Mtr.41670.1.S1_at	10.79	10.06	10.14	10.63	10.96	10.61	10.33	0.40	10.73	0.20	1.04	0.055	1.9E-01
Mtr.39505.1.S1_at	8.33	9.02	9.02	8.29	8.85	8.44	8.79	0.40	8.53	0.29	0.97	-0.044	4.1E-01
Mtr.42118.1.S1_at	7.57	7.54	8.25	7.83	7.76	8.09	7.79	0.40	7.89	0.17	1.01	0.020	6.9E-01
Mtr.25090.1.S1_at	2.40	3.08	2.37	2.35	2.32	2.35	2.62	0.40	2.34	0.02	0.89	-0.162	2.9E-01
Mtr.9604.1.S1_at	8.56	7.97	8.74	8.91	8.75	8.83	8.42	0.40	8.83	0.08	1.05	0.068	1.6E-01
Mtr.52072.1.S1_at	7.42	6.79	6.68	8.19	9.13	7.56	6.96	0.40	8.29	0.79	1.19	0.252	6.0E-02
Mtr.13773.1.S1_at	11.44	10.66	11.15	11.35	11.43	11.26	11.08	0.40	11.34	0.08	1.02	0.033	3.3E-01
Mtr.2191.1.S1_at	8.89	8.14	8.27	8.43	8.38	8.60	8.43	0.40	8.47	0.12	1.00	0.006	9.0E-01
Mtr.13216.1.S1_at	8.63	8.01	8.75	8.23	8.47	8.54	8.46	0.40	8.41	0.16	0.99	-0.008	8.6E-01
Mtr.15694.1.S1_at	6.46	5.67	6.20	6.74	6.91	6.56	6.11	0.40	6.74	0.18	1.10	0.141	6.8E-02
Mtr.16505.1.S1_at	13.45	12.70	13.30	13.45	13.41	13.46	13.15	0.40	13.44	0.03	1.02	0.031	2.8E-01
Mtr.19255.1.S1_x_at	10.61	9.86	10.47	10.48	10.63	10.32	10.31	0.40	10.47	0.15	1.02	0.023	5.5E-01
Mtr.13665.1.S1_at	7.07	7.74	7.04	6.95	7.05	7.22	7.28	0.40	7.07	0.14	0.97	-0.042	4.4E-01
Mtr.44866.1.S1_at	9.06	8.83	9.61	9.37	9.45	9.34	9.16	0.40	9.39	0.06	1.02	0.035	3.9E-01
Mtr.38620.1.S1_at	12.16	11.36	11.70	11.81	11.49	11.95	11.74	0.40	11.75	0.24	1.00	0.002	9.6E-01
Mtr.30481.1.S1_at	9.17	9.04	9.79	9.31	9.73	9.07	9.33	0.40	9.37	0.33	1.00	0.005	9.1E-01
Mtr.41155.1.S1_at	8.20	7.98	8.75	8.34	8.54	8.42	8.31	0.40	8.43	0.10	1.01	0.021	6.3E-01
Mtr.52198.1.S1_at	8.94	8.18	8.36	7.98	7.40	7.94	8.49	0.40	7.77	0.32	0.92	-0.127	7.3E-02
Mtr.15657.1.S1_at	7.38	8.15	7.95	7.30	7.46	7.48	7.83	0.40	7.41	0.10	0.95	-0.079	1.5E-01
Mtr.394.1.S1_s_at	10.51	9.88	9.78	10.04	9.63	9.80	10.06	0.40	9.82	0.20	0.98	-0.034	4.2E-01
Mtr.10789.1.S1_at	10.68	9.99	10.00	10.17	9.98	10.26	10.22	0.40	10.14	0.14	0.99	-0.013	7.4E-01
Mtr.9009.1.S1_at	8.51	7.85	8.56	8.19	8.45	8.42	8.31	0.40	8.35	0.14	1.01	0.008	8.6E-01
Mtr.39284.1.S1_at	7.31	6.51	6.92	7.19	7.31	7.25	6.91	0.40	7.25	0.06	1.05	0.069	2.2E-01
Mtr.45083.1.S1_at	7.68	7.85	8.44	7.99	7.87	7.69	7.99	0.40	7.85	0.15	0.98	-0.025	6.1E-01
Mtr.270.1.S1_at	3.08	3.77	3.07	3.27	2.67	2.94	3.31	0.40	2.96	0.30	0.90	-0.159	3.0E-01
Mtr.13403.1.S1_at	9.10	8.53	9.30	9.31	9.37	9.09	8.98	0.40	9.25	0.15	1.03	0.044	3.2E-01
Mtr.26522.1.S1_at	4.47	3.83	4.56	4.55	4.25	4.28	4.29	0.40	4.36	0.16	1.02	0.024	7.9E-01
Mtr.17310.1.S1_x_at	2.75	3.41	2.70	2.68	2.66	2.80	2.95	0.40	2.71	0.08	0.92	-0.122	3.6E-01
Mtr.32678.1.S1_s_at	2.72	3.44	2.78	3.24	3.65	2.73	2.98	0.40	3.21	0.46	1.08	0.105	5.6E-01
Mtr.51596.1.S1_at	6.89	7.11	7.66	7.11	7.57	7.09	7.22	0.40	7.26	0.27	1.01	0.008	9.0E-01
Mtr.23638.1.S1_s_at	11.24	10.76	11.55	11.08	11.03	11.04	11.18	0.40	11.05	0.03	0.99	-0.017	6.0E-01
Mtr.14773.1.S1_at	7.39	7.38	8.08	7.89	8.10	7.90	7.62	0.40	7.96	0.12	1.05	0.064	2.2E-01
Mtr.51684.1.S1_at	3.89	4.54	3.82	3.79	3.75	3.77	4.08	0.40	3.77	0.02	0.92	-0.114	2.5E-01
Mtr.27005.1.S1_at	2.19	2.88	2.19	2.19	2.17	2.18	2.42	0.40	2.18	0.01	0.90	-0.150	3.6E-01
Mtr.16648.1.S1_at	3.19	3.84	3.11	3.09	3.03	3.02	3.38	0.40	3.05	0.04	0.90	-0.149	2.2E-01
Mtr.44213.1.S1_at	8.55	8.56	9.24	8.87	8.92	8.90	8.79	0.40	8.90	0.02	1.01	0.019	6.5E-01
Mtr.1543.1.S1_at	3.27	2.57	3.26	3.50	3.10	3.43	3.03	0.40	3.34	0.21	1.10	0.140	3.0E-01
Mtr.33470.1.S1_at	7.33	7.16	7.92	7.35	7.18	7.69	7.47	0.40	7.40	0.26	0.99	-0.013	8.2E-01
Mtr.15251.1.S1_at	4.24	3.45	3.76	3.78	3.92	3.75	3.82	0.40	3.82	0.09	1.00	0.001	1.0E+00
Mtr.31594.1.S1_at	8.70	7.98	8.64	8.57	8.59	8.81	8.44	0.40	8.66	0.14	1.03	0.037	4.2E-01
Mtr.29548.1.S1_at	2.86	3.54	2.84	2.82	2.78	2.76	3.08	0.40	2.79	0.03	0.90	-0.144	2.7E-01
Mtr.32116.1.S1_x_at	3.01	3.64	2.90	2.92	2.89	2.87	3.18	0.40	2.90	0.03	0.91	-0.135	2.8E-01
Mtr.28370.1.S1_at	6.59	6.04	6.82	6.57	7.02	6.73	6.48	0.40	6.77	0.23	1.04	0.063	3.3E-01
Mtr.6225.1.S1_at	3.40	2.80	2.65	2.64	2.66	2.61	2.95	0.40	2.64	0.03	0.89	-0.163	2.4E-01
Mtr.11062.1.S1_at	10.00	9.48	10.27	9.93	9.90	9.82	9.92	0.40	9.89	0.06	1.00	-0.005	9.0E-01
Mtr.12179.1.S1_at	8.18	8.56	8.98	8.62	8.69	8.45	8.57	0.40	8.59	0.12	1.00	0.002	9.6E-01
Mtr.17426.1.S1_at	10.73	9.96	10.49	10.58	10.58	10.58	10.39	0.40	10.58	0.00	1.02	0.026	4.5E-01
Mtr.41517.1.S1_at	8.68	8.23	9.02	8.60	8.63	8.84	8.64	0.40	8.69	0.13	1.01	0.008	8.5E-01
Mtr.39198.1.S1_at	8.88	8.15	8.80	9.05	9.16	9.12	8.61	0.40	9.11	0.06	1.06	0.081	9.9E-02
Mtr.16745.1.S1_at	2.69	3.35	2.64	2.64	2.64	2.61	2.90	0.40	2.63	0.02	0.91	-0.141	3.1E-01
Mtr.32768.1.S1_s_at	8.92	8.16	8.74	8.89	8.96	9.02	8.61	0.40	8.96	0.06	1.04	0.057	2.1E-01
Mtr.27006.1.S1_at	9.39	8.60	9.08	9.38	9.43	9.14	9.02	0.40	9.32	0.16	1.03	0.047	3.0E-01
Mtr.48590.1.S1_at	6.12	5.64	6.43	6.33	6.52	6.17	6.06	0.40	6.34	0.18	1.05	0.064	3.4E-01
Mtr.51350.1.S1_at	2.72	3.39	2.69	2.69	2.60	2.68	2.93	0.40	2.65	0.05	0.90	-0.144	2.9E-01
Mtr.25043.1.S1_at	12.42	12.18	12.96	12.13	11.87	11.78	12.52	0.40	11.93	0.18	0.95	-0.070	7.8E-02

Mtr.25789.1.S1_at	7.40	8.19	7.74	7.70	7.72	7.68	7.78	0.40	7.70	0.02	0.99	-0.014	7.6E-01
Mtr.32118.1.S1_x_at	8.19	8.89	8.87	8.37	8.32	8.27	8.65	0.40	8.32	0.05	0.96	-0.057	2.2E-01
Mtr.40503.1.S1_s_at	10.58	9.88	9.90	11.20	11.92	11.43	10.12	0.40	11.52	0.37	1.14	0.186	1.1E-02
Mtr.50492.1.S1_s_at	8.71	9.14	9.51	9.46	9.46	9.20	9.12	0.40	9.37	0.15	1.03	0.040	3.6E-01
Mtr.42779.1.S1_at	10.75	10.30	11.09	10.80	10.72	10.78	10.71	0.40	10.77	0.04	1.01	0.007	8.2E-01
Mtr.7405.1.S1_at	8.01	7.27	7.90	8.21	7.96	7.89	7.73	0.40	8.02	0.17	1.04	0.053	3.1E-01
Mtr.31396.1.S1_at	2.89	2.29	3.04	3.96	4.02	2.99	2.74	0.40	3.66	0.58	1.33	0.416	8.6E-02
Mtr.38463.1.S1_at	8.85	8.69	9.45	8.84	8.84	8.87	9.00	0.40	8.85	0.02	0.98	-0.024	5.6E-01
Mtr.22008.1.S1_at	2.69	3.36	2.66	2.65	2.64	2.61	2.90	0.40	2.63	0.02	0.91	-0.140	3.1E-01
Mtr.49000.1.S1_s_at	10.29	10.27	10.97	11.03	10.87	10.75	10.51	0.40	10.88	0.14	1.04	0.050	2.0E-01
Mtr.35523.1.S1_at	5.12	5.92	5.58	4.89	5.43	5.50	5.54	0.40	5.27	0.33	0.95	-0.071	4.2E-01
Mtr.19135.1.S1_at	9.62	8.83	9.31	9.34	9.38	9.22	9.25	0.40	9.31	0.08	1.01	0.009	8.1E-01
Mtr.1434.1.S1_at	9.39	9.74	10.18	9.82	10.28	10.03	9.77	0.40	10.04	0.23	1.03	0.039	3.6E-01
Mtr.12929.1.S1_at	12.03	11.24	11.76	11.77	11.67	11.66	11.67	0.40	11.70	0.06	1.00	0.003	9.1E-01
Mtr.37646.1.S1_s_at	10.86	10.08	10.33	10.55	10.30	10.77	10.42	0.40	10.54	0.24	1.01	0.016	6.9E-01
Mtr.38625.1.S1_at	9.06	8.31	8.91	8.70	9.05	8.81	8.76	0.40	8.86	0.18	1.01	0.016	7.3E-01
Mtr.26433.1.S1_at	2.94	3.61	2.90	2.88	2.78	2.80	3.15	0.40	2.82	0.05	0.90	-0.158	2.3E-01
Mtr.12493.1.S1_at	8.21	7.76	8.55	8.28	8.10	8.03	8.17	0.40	8.13	0.13	1.00	-0.006	8.9E-01
Mtr.46531.1.S1_at	2.83	3.51	2.81	2.80	2.79	2.73	3.05	0.40	2.77	0.04	0.91	-0.136	3.0E-01
Mtr.36866.1.S1_at	3.13	2.44	3.12	2.40	3.10	3.09	2.90	0.40	2.86	0.40	0.99	-0.017	9.2E-01
Mtr.13820.1.S1_at	10.30	10.67	11.10	10.32	10.33	10.17	10.69	0.40	10.28	0.09	0.96	-0.057	1.5E-01
Mtr.29965.1.S1_at	2.08	2.76	2.07	2.07	2.07	2.07	2.31	0.40	2.07	0.00	0.90	-0.155	3.6E-01
Mtr.13023.1.S1_at	10.12	10.83	10.79	10.60	10.71	10.62	10.58	0.40	10.65	0.06	1.01	0.009	8.0E-01
Mtr.45917.1.S1_at	11.92	11.14	11.40	11.61	11.60	11.64	11.49	0.40	11.62	0.02	1.01	0.016	6.1E-01
Mtr.2499.1.S1_at	7.21	8.01	7.59	7.69	7.62	7.51	7.60	0.40	7.61	0.09	1.00	0.001	9.9E-01
Mtr.49188.1.S1_at	3.05	3.07	3.75	3.04	3.01	3.06	3.29	0.40	3.04	0.03	0.92	-0.116	3.3E-01
Mtr.51188.1.S1_at	10.03	10.03	10.71	10.16	9.86	9.91	10.26	0.40	9.98	0.16	0.97	-0.040	3.2E-01
Mtr.7401.1.S1_at	10.98	10.28	10.95	11.02	11.19	10.99	10.73	0.40	11.07	0.11	1.03	0.044	2.3E-01
Mtr.50458.1.S1_at	8.51	8.89	9.30	8.80	9.17	8.90	8.90	0.40	8.96	0.19	1.01	0.010	8.3E-01
Mtr.42104.1.S1_at	8.72	8.11	8.85	8.70	8.73	8.71	8.56	0.40	8.71	0.01	1.02	0.025	5.5E-01
Mtr.12905.1.S1_at	9.34	8.69	9.41	9.12	9.19	9.08	9.15	0.40	9.13	0.06	1.00	-0.003	9.3E-01
Mtr.37410.1.S1_at	10.83	10.05	10.54	10.57	10.72	10.65	10.48	0.40	10.64	0.07	1.02	0.023	5.1E-01
Mtr.18021.1.S1_at	3.18	2.53	2.46	2.62	2.97	2.49	2.72	0.40	2.69	0.25	0.99	-0.015	9.2E-01
Mtr.24980.1.S1_at	3.94	4.06	4.68	4.04	3.97	3.86	4.23	0.40	3.96	0.09	0.94	-0.096	3.1E-01
Mtr.38033.1.S1_at	10.34	9.84	9.55	9.82	9.98	9.85	9.91	0.40	9.88	0.09	1.00	-0.004	9.2E-01
Mtr.33271.1.S1_at	6.12	6.91	6.45	6.79	6.86	6.96	6.49	0.40	6.87	0.09	1.06	0.082	1.8E-01
Mtr.45014.1.S1_at	8.50	7.83	8.53	8.54	8.54	8.53	8.29	0.40	8.54	0.00	1.03	0.043	3.3E-01
Mtr.20226.1.S1_at	4.24	3.61	3.51	3.49	3.48	3.49	3.79	0.40	3.49	0.00	0.92	-0.120	2.6E-01
Mtr.10770.1.S1_at	10.26	9.47	9.83	9.79	9.93	9.79	9.85	0.40	9.83	0.08	1.00	-0.002	9.5E-01
Mtr.43285.1.S1_at	10.65	11.30	11.36	11.28	11.56	11.27	11.10	0.40	11.37	0.17	1.02	0.034	3.4E-01
Mtr.43138.1.S1_at	8.67	7.91	8.48	8.52	8.68	8.55	8.35	0.40	8.58	0.09	1.03	0.039	3.8E-01
Mtr.50912.1.S1_at	2.60	3.30	2.63	2.60	2.63	2.68	2.85	0.40	2.64	0.04	0.93	-0.110	4.2E-01
Mtr.22947.1.S1_at	4.33	3.93	3.54	3.55	4.49	3.59	3.93	0.40	3.88	0.53	0.99	-0.021	8.9E-01
Mtr.2713.1.S1_at	7.95	7.59	8.38	8.07	8.37	7.73	7.97	0.40	8.06	0.32	1.01	0.015	7.9E-01
Mtr.49873.1.S1_at	3.21	2.99	3.76	3.78	3.97	3.49	3.32	0.40	3.75	0.25	1.13	0.175	1.9E-01
Mtr.52138.1.S1_at	2.84	3.52	2.83	2.90	2.80	2.76	3.06	0.40	2.82	0.07	0.92	-0.118	3.6E-01
Mtr.52083.1.S1_at	2.73	3.41	2.71	2.66	2.70	2.69	2.95	0.40	2.68	0.02	0.91	-0.137	3.1E-01
Mtr.44064.1.S1_at	9.55	8.93	9.67	9.74	9.43	9.65	9.38	0.40	9.61	0.16	1.02	0.034	4.2E-01
Mtr.29270.1.S1_at	10.93	10.14	10.52	10.74	10.81	10.73	10.53	0.40	10.76	0.04	1.02	0.032	3.7E-01
Mtr.31474.1.S1_at	8.54	9.33	9.02	8.65	8.54	8.65	8.96	0.40	8.61	0.06	0.96	-0.057	2.1E-01
Mtr.44376.1.S1_at	10.25	10.67	11.04	10.41	10.30	10.21	10.65	0.40	10.31	0.10	0.97	-0.048	2.2E-01
Mtr.43402.1.S1_at	10.36	11.08	11.00	10.68	10.67	10.72	10.81	0.40	10.69	0.02	0.99	-0.017	6.1E-01
Mtr.24467.1.S1_s_at	9.61	8.87	9.48	9.51	9.48	9.58	9.32	0.40	9.52	0.06	1.02	0.031	4.3E-01
Mtr.32844.1.S1_at	9.02	8.23	8.56	9.11	8.87	9.06	8.60	0.40	9.01	0.13	1.05	0.067	1.6E-01
Mtr.35196.1.S1_s_at	9.50	9.24	10.02	10.10	10.12	10.06	9.58	0.40	10.09	0.03	1.05	0.075	9.1E-02
Mtr.31529.1.S1_at	5.54	5.42	6.15	5.23	5.46	5.45	5.70	0.40	5.38	0.13	0.94	-0.084	2.5E-01
Mtr.20699.1.S1_at	3.76	3.82	3.10	3.23	2.94	3.11	3.56	0.40	3.09	0.15	0.87	-0.203	1.3E-01
Mtr.3154.1.S1_at	5.28	5.63	6.07	5.89	6.08	5.55	5.66	0.40	5.84	0.27	1.03	0.046	5.5E-01
Mtr.2430.1.S1_at	2.72	3.38	2.66	2.64	2.58	2.61	2.92	0.40	2.61	0.03	0.89	-0.161	2.5E-01
Mtr.25652.1.S1_at	2.77	2.82	2.11	3.02	3.88	2.73	2.57	0.40	3.21	0.59	1.25	0.323	1.9E-01
Mtr.43382.1.S1_at	12.86	13.55	12.87	12.52	12.59	12.57	13.09	0.40	12.56	0.04	0.96	-0.060	8.2E-02
Mtr.27806.1.S1_at	9.10	8.44	9.15	8.87	9.08	8.92	8.90	0.40	8.96	0.11	1.01	0.010	8.0E-01
Mtr.25573.1.S1_s_at	12.21	12.95	12.84	12.47	12.59	12.55	12.67	0.40	12.54	0.06	0.99	-0.015	6.1E-01
Mtr.1933.1.S1_at	8.45	7.83	8.57	8.55	8.48	8.17	8.29	0.40	8.40	0.20	1.01	0.020	6.8E-01
Mtr.13157.1.S1_at	10.87	10.08	10.52	10.40	10.62	10.42	10.49	0.40	10.48	0.13	1.00	-0.002	9.6E-01
Mtr.26887.1.S1_x_at	11.19	10.43	11.00	11.13	11.01	10.83	10.87	0.40	10.99	0.15	1.01	0.015	6.6E-01
Mtr.19546.1.S1_x_at	3.38	4.07	3.38	3.50	3.37	3.37	3.61	0.40	3.41	0.07	0.95	-0.081	4.4E-01
Mtr.37728.1.S1_at	11.94	11.15	11.58	11.74	11.71	11.71	11.55	0.40	11.72	0.02	1.01	0.020	5.1E-01
Mtr.14428.1.S1_x_at	7.05	6.40	6.34	6.17	5.54	6.25	6.59	0.40	5.99	0.39	0.91	-0.139	1.3E-01
Mtr.30981.1.S1_at	4.23	3.58	3.52	3.52	3.39	3.52	3.78	0.40	3.47	0.08	0.92	-0.121	2.6E-01
Mtr.5367.1.S1_at	2.68	2.47	3.24	2.67	2.88	2.58	2.80	0.40	2.71	0.15	0.97	-0.045	7.4E-01
Mtr.33213.1.S1_at	10.96	10.17	10.54	10.67	10.81	10.76	10.56	0.40	10.75	0.07	1.02	0.026	4.6E-01
Mtr.32561.1.S1_at	8.52	8.61	9.25	8.59	8.77	8.65	8.79	0.40	8.67	0.09	0.99	-0.020	6.3E-01
Mtr.42340.1.S1_at	8.61	9.11	9.39	9.83	9.35	9.33	9.04	0.40	9.50	0.28	1.05	0.072	1.7E-01
Mtr.5073.1.S1_at	5.48	4.83	5.54	5.54	5.51	5.58	5.28	0.40	5.55	0.04	1.05	0.070	3.2E-01
Mtr.12109.1.S1_at	10.74	10.03	10.70	10.63	10.66	10.55	10.49	0.40	10.61	0.05	1.01	0.017	6.2E-01
Mtr.50001.1.S1_s_at	9.39	8.63	9.20	9.60	9.41	9.38	9.07	0.40	9.47	0.12	1.04	0.062	1.7E-01
Mtr.33091.1.S1_at	6.01	5.38	5.28	5.40	5.61	5.61	5.56	0.40	5.54	0.12	1.00	-0.004	9.6E-01
Mtr.9966.1.S1_at	8.96	8.22	8.84	8.74	8.60	8.78	8.67	0.40	8.71	0.09	1.00	0.005	9.0E-01
Mtr.21955.1.S1_at	3.17	3.84	3.14	3.23	3.13	2.86	3.38	0.40	3.07	0.19	0.91	-0.138	2.9E-01
Mtr.35822.1.S1_s_at	8.52	7.76	8.31	8.38	8.24	8.31	8.20	0.40	8.31	0.07	1.01	0.020	6.5E-01
Mtr.5388.1.S1_at	8.54	7.77	8.33	8.11	8.14	7.69	8.21	0.40	7.98	0.25	0.97	-0.042	4.3E-01
Mtr.51383.1.S1_x_at	2.97	3.64	2.94	2.93	2.92	2.92	3.18	0.40	2.92	0.01	0.92	-0.123	3.2E-01
Mtr.24424.1.S1_s_at	8.88	8.48	9.27	9.06	8.98	8.97	8.88	0.40	9.00	0.05	1.01	0.020	6.2E-01
Mtr.48864.1.S1_at	11.41	10.62	10.99	10.82	10.83	10.96	11.01	0.40	10.87	0.08	0.99	-0.018	5.9E-01
Mtr.22296.1.S1_at	2.88	3.54	2.84	2.84	2.83	2.93	3.09	0.40	2.86	0.05	0.93	-0.109	3.8E-01
Mtr.33354.1.S1_at	11.65	10.86	11.20	11.43	11.34	11.57	11.24	0.40	11.45	0.12	1.02	0.027	4.3E-01
Mtr.4011.1.S1_at	4.27	3.57	4.25	3.88	4.57	4.46	4.03	0.40	4.30	0.37	1.07	0.095	4.3E-01
Mtr.43359.1.S1_at	11.51	12.18	12.20	11.65	11.94	11.67	11.96	0.40	11.75	0.16	0.98	-0.025	4.5E-01
Mtr.46625.1.S1_at	3.43	3.98	3.22	3.20	3.16	3.15	3.54	0.40	3.17	0.03	0.89	-0.162	1.8E-01
Mtr.4732.1.S1_at	9.74	9.42	10.21	9.48	9.92	9.31	9.79	0.40	9.57	0.31	0.98	-0.033	4.9E-01
Mtr.5192.1.S1_at	5.05	4.98	4.33	5.29	5.13	5.09	4.79	0.40	5.17	0.11	1.08	0.112	1.8E-01
Mtr.17320.1.S1_at	8.30	7.93	8.72	8.44	8.57	8.25	8.32	0.40	8.42	0.16	1.01	0.018	6.9E-01
Mtr.24837.1.S1_at	2.60	3.26	2.56	2.53	2.52	2.51	2.81	0.40	2.52	0.01	0.90	-0.155	2.8E-01
Mtr.17143.1.S1_at	2.99	3.65	2.95	2.92	2.90	2.89	3.20	0.40	2.90	0.02	0.91	-0.138	2.7E-01
Mtr.20094.1.S1_x_at	5.10	4.34	4.93	4.88	4.90	4.90	4.79	0.40	4.89	0.01	1.02	0.031	6.7E-01
Mtr.49653.1.S1_at	7.50	8.08	8.25	8.04	7.72	7.58	7.94	0.40	7.78	0.23	0.98	-0.030	5.8E-01
Mtr.8583.1.S1_at	7.05	6.26	6.63	7.11	6.90	6.50	6.65	0.40	6.84	0.31	1.03	0.041	5.5E-01
Mtr.31138.1.S1_at	5.46	4.76	4.78	6.18	5.54	5.57	5.00	0.40	5.76	0.36	1.15	0.205	6.9E-02
Mtr.43262.1.S1_at	2.16	2.84	2.15	2.15	2.14	2.09	2.38	0.40	2.13	0.03	0.89	-0.166	3.2E-01
Mtr.5067.1.S1_at	9.38	8.85	9.62	9.29	9.37	9.15	9.28	0.40	9.27	0.11	1.00	-0.002	9.6E-01
Mtr.13065.1.S1_at	8.50	7.72	8.02	8.21	8.51	8.36	8.08	0.40	8.36	0.15	1.04	0.050	3.1E-01
Mtr.11136.1.S1_at	10.23	10.60	11.02	10.72	10.75	10.62	10.61	0.39	10.70	0.07	1.01	0.011	7.4E-01
Mtr.6729.1.S1_at	6.40	5.82	6.58	6.11	5.73	5.73	6.27	0.39	5.85	0.22	0.93	-0.098	1.9E-01
Mtr.30171.1.S1_at	2.57	3.25	2.56	2.54	2.52	2.53	2.79	0.39	2.53	0.01	0.91	-0.143	3.1E-01
Mtr.19201.1.S1_s_at	5.25	4.57	5.25	5.62	5.16	5.28	5.02	0.39	5.36	0.24	1.07	0.092	2.8E-01
Mtr.38527.1.S1_at	11.06	10.28	10.76	10.85	10.89	10.85	10.70	0.39	10.86	0.03	1.02	0.022	5.1E-01
Mtr.1267.1.S1_at	8.64	8.06	8.81	8.56	8.72	8.53	8.50	0.39	8.60	0.10	1.01	0.016	7.0E-01
Mtr.7088.1.S1_s_at	10.49	9.81	10.48	10.43	10.31	10.33	10.26	0.39	10.36	0.06	1.01	0.014	6.9E-01
Mtr.35818.1.S1_at	9.60	8.84	9.04	10.13	10.46	10.18	9.16	0.39	10.26	0.18	1.12	0.164	1.2E-02
Mtr.4888.1.S1_at	2.35	3.02	2.32	2.34	2.33	2.30	2.56	0.39	2.32	0.02	0.91	-0.143	3.5E-01
Mtr.19845.1.S1_at	9.01	8.23	8.59	8.82	8.99	8.90	8.61	0.39	8.90	0.08	1.03	0.048	2.8E-01
Mtr.13592.1.S1_at	9.31	8.54	9.08	8.91	8.62	8.76	8.98	0.39	8.76	0.15	0.98	-0.035	4.3E-01
Mtr.45813.1.S1_at	8.23	7.50	8.12	8.14	7.98	7.81	7.95	0.39	7.98	0.16	1.00	0.005	9.1E-01
Mtr.28167.1.S1_s_at	10.00	10.73	10.63	10.53	10.62	10.37	10.45	0.39	10.51	0.13	1.00	0.007	8.4E-01
Mtr.42795.1.S1_at	10.96	11.09	10.35	10.85	10.57	10.89	10.80	0.39	10.77	0.17	1.00	-0.004	9.1E-01
Mtr.25866.1.S1_at	8.60	7.81	8.26	8.17	8.05	8.15	8.23	0.39	8.12	0.07	0.99	-0.018	6.8E-01
Mtr.163.1.S1_at	3.86	4.23	3.44	3.39	3.35	3.40	3.84	0.39	3.38	0.03	0.88	-0.186	1.1E-01
Mtr.49349.1.S1_at	12.27	11.49	11.81	12.32	12.17	12.28	11.86	0.39	12.25	0.07	1.03	0.048	1.6E-01
Mtr.14281.1.S1_at	11.19	11.62	11.98	11.32	11.32	11.57	11.60	0.39	11.40	0.15	0.98	-0.025	4.6E-01
Mtr.10517.1.S1_at	12.71	13.50	13.09	12.81	13.15	13.11	13.10	0.39	13.03	0.19	0.99	-0.008	7.9E-01
Mtr.32512.1.S1_at	7.51	6.76	7.34	7.45	7.34	7.49	7.20	0.39	7.43	0.08	1.03	0.044	3.9E-01
Mtr.11857.1.S1_x_at	3.21	3.64	3.99	3.71	3.26	3.30	3.61	0.39	3.42	0.25	0.95	-0.078	5.2E-01
Mtr.29715.1.S1_at	6.97	7.62	6.91	6.91	6.97	6.94	7.17	0.39	6.94	0.03	0.97	-0.046	3.8E-01
Mtr.38258.1.S1_at	9.03	8.36	8.33	8.51	8.51	8.49	8.57	0.39	8.50	0.01	0.99	-0.012	7.7E-01
Mtr.10202.1.S1_at	8.79	8.03	8.24	8.56	8.33	8.52	8.36	0.39	8.47	0.12	1.01	0.020	6.6E-01
Mtr.15508.1.S1_at	3.58	4.06	3.27	3.77	3.72	3.77	3.64	0.39	3.76	0.03	1.03	0.047	6.3E-01
Mtr.14187.1.S1_at	5.84	5.13	5.79	5.20	5.90	6.09	5.59	0.39	5.73	0.47	1.03	0.037	7.1E-01
Mtr.29830.1.S1_at	7.39	6.66	7.29	7.43	7.31	7.46	7.12	0.39	7.40	0.08	1.04	0.057	2.9E-01
Mtr.7497.1.S1_at	6.77	6.05	6.69	6.94	6.78	6.96	6.50	0.39	6.90	0.10	1.06	0.085	1.7E-01
Mtr.48439.1.S1_at	9.09	8.97	9.71	9.42	9.44	9.18	9.26	0.39	9.34	0.15	1.01	0.014	7.3E-01

Mtr.28499.1.S1_at	3.12	3.24	3.85	3.32	3.25	3.53	3.40	0.39	3.37	0.14	0.99	-0.017	8.8E-01
Mtr.34623.1.S1_at	9.84	9.11	9.75	9.24	9.30	9.28	9.57	0.39	9.28	0.03	0.97	-0.044	2.7E-01
Mtr.38433.1.S1_at	11.47	10.72	10.90	11.08	11.00	11.06	11.03	0.39	11.05	0.05	1.00	0.002	9.5E-01
Mtr.8459.1.S1_at	12.19	11.45	12.07	11.90	12.12	12.12	11.90	0.39	12.05	0.13	1.01	0.017	5.8E-01
Mtr.16355.1.S1_at	4.78	5.44	4.74	4.61	4.55	4.93	4.98	0.39	4.70	0.20	0.94	-0.085	3.2E-01
Mtr.27795.1.S1_at	8.13	7.34	7.81	8.12	7.96	7.89	7.76	0.39	7.99	0.12	1.03	0.042	3.9E-01
Mtr.4594.1.S1_at	8.36	7.73	8.45	8.26	8.36	8.13	8.18	0.39	8.25	0.11	1.01	0.012	7.9E-01
Mtr.5385.1.S1_at	2.14	2.50	2.92	2.85	3.40	4.06	2.52	0.39	3.44	0.61	1.36	0.448	9.3E-02
Mtr.11721.1.S1_at	8.68	8.05	8.77	8.66	8.52	8.45	8.50	0.39	8.54	0.11	1.01	0.008	8.5E-01
Mtr.43789.1.S1_at	9.14	8.45	9.12	9.06	9.11	9.29	8.90	0.39	9.15	0.12	1.03	0.040	3.5E-01
Mtr.13613.1.S1_s_at	10.02	9.24	9.52	9.75	9.65	9.80	9.59	0.39	9.73	0.08	1.01	0.021	5.7E-01
Mtr.8428.1.S1_at	14.46	13.79	14.50	14.72	14.69	14.72	14.25	0.39	14.71	0.01	1.03	0.046	1.1E-01
Mtr.19007.1.S1_at	8.96	9.04	8.33	8.95	9.41	9.09	8.78	0.39	9.15	0.24	1.04	0.060	2.3E-01
Mtr.42606.1.S1_at	11.54	12.12	12.29	11.55	11.14	11.44	11.99	0.39	11.38	0.22	0.95	-0.075	7.8E-02
Mtr.42748.1.S1_s_at	9.84	9.05	9.49	9.67	9.57	9.59	9.46	0.39	9.61	0.05	1.02	0.023	5.5E-01
Mtr.40205.1.S1_at	12.20	11.50	11.54	11.56	12.03	11.71	11.75	0.39	11.77	0.24	1.00	0.002	9.5E-01
Mtr.2724.1.S1_at	2.72	3.40	2.72	2.70	2.68	2.70	2.94	0.39	2.69	0.01	0.92	-0.128	3.3E-01
Mtr.35162.1.S1_at	9.01	8.36	9.07	9.19	9.25	9.16	8.81	0.39	9.20	0.04	1.04	0.062	1.7E-01
Mtr.42070.1.S1_at	9.20	8.55	9.26	9.35	9.29	9.14	9.00	0.39	9.26	0.11	1.03	0.041	3.4E-01
Mtr.41292.1.S1_at	9.29	8.86	9.65	9.60	9.68	9.46	9.27	0.39	9.58	0.11	1.03	0.048	2.6E-01
Mtr.13180.1.S1_at	10.95	10.19	10.75	10.86	10.85	10.84	10.63	0.39	10.85	0.01	1.02	0.030	3.9E-01
Mtr.22975.1.S1_at	2.36	3.03	2.34	2.33	2.31	2.32	2.57	0.39	2.32	0.01	0.90	-0.149	3.3E-01
Mtr.50521.1.S1_s_at	2.84	3.52	2.84	2.80	2.80	2.74	3.07	0.39	2.78	0.03	0.91	-0.141	2.8E-01
Mtr.35259.1.S1_at	4.38	3.62	4.20	3.97	4.54	4.06	4.07	0.39	4.19	0.31	1.03	0.042	7.0E-01
Mtr.17795.1.S1_x_at	12.30	12.94	13.01	12.60	12.74	12.56	12.75	0.39	12.63	0.10	0.99	-0.013	6.5E-01
Mtr.25463.1.S1_at	2.49	3.16	2.47	2.47	2.45	2.46	2.71	0.39	2.46	0.01	0.91	-0.137	3.4E-01
Mtr.15233.1.S1_s_at	2.83	3.49	2.78	2.78	2.77	2.77	3.03	0.39	2.77	0.00	0.91	-0.130	3.1E-01
Mtr.7155.1.S1_at	9.45	8.73	9.37	9.57	9.57	9.60	9.18	0.39	9.58	0.02	1.04	0.061	1.6E-01
Mtr.45622.1.S1_s_at	10.31	10.63	11.09	10.59	11.18	10.47	10.68	0.39	10.74	0.38	1.01	0.009	8.4E-01
Mtr.19369.1.S1_at	9.68	9.63	10.34	9.40	9.08	9.22	9.88	0.39	9.23	0.16	0.93	-0.098	5.7E-02
Mtr.5600.1.S1_at	9.28	8.54	9.15	9.32	9.25	9.13	8.99	0.39	9.24	0.10	1.03	0.039	3.5E-01
Mtr.17106.1.S1_at	2.26	2.92	2.22	2.22	2.21	2.21	2.46	0.39	2.21	0.00	0.90	-0.156	3.3E-01
Mtr.23328.1.S1_at	2.43	3.10	2.41	2.41	2.40	2.39	2.65	0.39	2.40	0.01	0.91	-0.141	3.4E-01
Mtr.3010.1.S1_at	3.92	4.68	4.13	3.93	3.59	3.92	4.24	0.39	3.81	0.20	0.90	-0.155	1.6E-01
Mtr.32985.1.S1_at	8.07	7.59	8.37	8.49	8.17	8.27	8.01	0.39	8.31	0.17	1.04	0.052	2.9E-01
Mtr.500.1.S1_at	8.71	8.18	8.94	8.74	8.77	8.74	8.61	0.39	8.75	0.01	1.02	0.023	5.7E-01
Mtr.39175.1.S1_at	6.59	5.83	6.37	6.52	6.43	6.46	6.26	0.39	6.47	0.04	1.03	0.047	4.1E-01
Mtr.50715.1.S1_at	6.38	7.07	6.39	6.37	6.45	6.52	6.61	0.39	6.45	0.07	0.98	-0.036	5.1E-01
Mtr.29061.1.S1_at	8.92	8.13	8.53	8.95	8.86	8.73	8.53	0.39	8.84	0.11	1.04	0.053	2.5E-01
Mtr.40611.1.S1_at	13.84	13.40	14.18	14.25	13.86	14.27	13.81	0.39	14.13	0.23	1.02	0.033	2.9E-01
Mtr.48120.1.S1_at	5.41	4.89	5.66	4.86	5.09	5.09	5.32	0.39	5.01	0.13	0.94	-0.085	2.7E-01
Mtr.38591.1.S1_at	9.47	10.25	9.94	10.00	10.17	9.89	9.89	0.39	10.02	0.14	1.01	0.019	6.2E-01
Mtr.44624.1.S1_at	4.77	5.45	4.77	4.74	4.83	4.71	5.00	0.39	4.76	0.06	0.95	-0.070	3.6E-01
Mtr.4972.1.S1_at	10.11	10.88	10.60	10.11	10.51	10.33	10.53	0.39	10.32	0.20	0.98	-0.029	4.5E-01
Mtr.9291.1.S1_s_at	10.89	10.17	10.26	10.26	10.47	10.26	10.44	0.39	10.33	0.12	0.99	-0.015	6.7E-01
Mtr.10412.1.S1_at	12.34	12.25	12.97	12.46	12.24	12.21	12.52	0.39	12.30	0.14	0.98	-0.025	4.2E-01
Mtr.29762.1.S1_at	6.09	5.66	6.44	6.08	6.32	5.97	6.06	0.39	6.12	0.18	1.01	0.014	8.2E-01
Mtr.19627.1.S1_at	9.25	8.46	8.86	8.87	8.79	9.02	8.86	0.39	8.89	0.12	1.00	0.006	8.8E-01
Mtr.33102.1.S1_at	7.69	7.11	6.95	7.36	7.27	7.17	7.25	0.39	7.27	0.09	1.00	0.004	9.4E-01
Mtr.18117.1.S1_at	6.46	6.02	6.80	6.84	6.50	6.49	6.42	0.39	6.61	0.20	1.03	0.041	5.0E-01
Mtr.20779.1.S1_at	2.86	3.53	2.85	2.84	2.82	2.84	3.08	0.39	2.83	0.01	0.92	-0.119	3.4E-01
Mtr.777.1.S1_s_at	2.59	3.24	2.55	2.53	2.53	2.52	2.79	0.39	2.53	0.01	0.90	-0.145	3.0E-01
Mtr.39477.1.S1_at	7.95	8.68	8.07	8.08	7.94	8.04	8.23	0.39	8.02	0.07	0.97	-0.037	4.1E-01
Mtr.47153.1.S1_at	11.15	10.39	10.94	11.09	10.82	11.08	10.83	0.39	11.00	0.15	1.02	0.023	5.2E-01
Mtr.25106.1.S1_x_at	2.77	3.42	2.73	2.71	2.71	2.64	2.97	0.39	2.69	0.04	0.90	-0.147	2.7E-01
Mtr.26972.1.S1_at	3.49	2.88	2.76	2.71	2.71	2.74	3.04	0.39	2.72	0.02	0.89	-0.163	2.3E-01
Mtr.31822.1.S1_at	6.93	6.84	7.56	7.56	7.68	7.64	7.11	0.39	7.63	0.06	1.07	0.102	8.6E-02
Mtr.17531.1.S1_at	5.04	4.32	4.95	5.55	4.95	5.08	4.77	0.39	5.19	0.31	1.09	0.123	2.2E-01
Mtr.28799.1.S1_at	11.75	11.03	11.66	11.76	11.70	11.82	11.48	0.39	11.76	0.06	1.02	0.035	2.9E-01
Mtr.29716.1.S1_at	9.30	8.53	9.05	8.88	8.91	9.10	8.96	0.39	8.96	0.12	1.00	0.000	9.9E-01
Mtr.20851.1.S1_at	2.61	3.29	2.62	2.60	2.59	2.59	2.84	0.39	2.59	0.01	0.91	-0.131	3.4E-01
Mtr.9390.1.S1_at	8.52	8.14	8.93	8.51	8.60	8.32	8.53	0.39	8.48	0.14	0.99	-0.009	8.3E-01
Mtr.36383.1.S1_at	5.25	4.51	4.66	4.66	4.66	4.66	4.81	0.39	4.66	0.00	0.97	-0.044	5.6E-01
Mtr.39791.1.S1_at	2.76	2.24	3.01	3.27	3.90	2.97	2.67	0.39	3.38	0.48	1.27	0.341	1.2E-01
Mtr.11234.1.S1_at	8.55	7.77	8.12	8.22	8.32	8.42	8.15	0.39	8.32	0.10	1.02	0.030	5.0E-01
Mtr.13737.1.S1_at	8.64	8.01	8.73	8.71	8.74	8.68	8.46	0.39	8.71	0.03	1.03	0.041	3.4E-01
Mtr.37326.1.S1_at	10.54	10.75	11.30	10.68	10.74	10.50	10.86	0.39	10.64	0.12	0.98	-0.030	4.0E-01
Mtr.33442.1.S1_at	4.67	4.56	5.28	4.58	4.58	4.50	4.84	0.39	4.55	0.05	0.94	-0.087	2.8E-01
Mtr.14079.1.S1_at	2.90	3.64	3.49	2.83	2.65	2.82	3.35	0.39	2.77	0.10	0.83	-0.274	6.9E-02
Mtr.7467.1.S1_at	3.58	4.36	3.91	4.10	4.69	4.20	3.95	0.39	4.33	0.32	1.10	0.132	2.6E-01
Mtr.6549.1.S1_at	8.99	8.21	8.70	8.61	8.70	8.60	8.63	0.39	8.64	0.06	1.00	0.001	9.8E-01
Mtr.43492.1.S1_at	10.93	11.31	11.71	10.75	10.87	10.79	11.32	0.39	10.80	0.06	0.95	-0.067	8.8E-02
Mtr.29714.1.S1_at	5.98	5.34	5.27	5.13	5.30	5.34	5.53	0.39	5.26	0.11	0.95	-0.073	3.1E-01
Mtr.49233.1.S1_at	2.20	2.88	2.19	2.19	2.19	2.19	2.42	0.39	2.19	0.00	0.90	-0.147	3.6E-01
Mtr.26491.1.S1_at	11.31	11.56	12.08	11.67	11.79	11.65	11.65	0.39	11.70	0.07	1.00	0.006	8.4E-01
Mtr.7115.1.S1_s_at	7.49	6.96	7.73	7.75	8.60	8.67	7.40	0.39	8.34	0.51	1.13	0.174	6.3E-02
Mtr.19097.1.S1_at	9.25	8.60	9.31	10.46	10.95	10.79	9.05	0.39	10.73	0.25	1.19	0.245	3.3E-03
Mtr.2470.1.S1_at	5.82	5.05	5.54	5.48	5.36	5.73	5.47	0.39	5.53	0.19	1.01	0.015	8.3E-01
Mtr.9595.1.S1_at	12.64	13.26	12.54	12.49	12.63	12.57	12.81	0.39	12.56	0.07	0.98	-0.028	3.4E-01
Mtr.52102.1.S1_at	13.10	12.33	12.59	12.84	12.77	12.89	12.68	0.39	12.83	0.06	1.01	0.018	5.3E-01
Mtr.7479.1.S1_at	8.17	7.54	8.26	7.76	7.69	7.63	7.99	0.39	7.70	0.07	0.96	-0.054	2.7E-01
Mtr.32736.1.S1_at	8.58	7.96	8.68	8.61	8.65	8.65	8.41	0.39	8.64	0.02	1.03	0.039	3.7E-01
Mtr.9189.1.S1_at	10.76	10.20	10.95	10.56	10.63	10.61	10.64	0.39	10.60	0.04	1.00	-0.006	8.7E-01
Mtr.45037.1.S1_at	12.43	12.25	11.68	12.24	11.89	12.31	12.12	0.39	12.15	0.23	1.00	0.003	9.3E-01
Mtr.19146.1.S1_at	2.75	3.40	2.70	2.69	2.67	2.69	2.95	0.39	2.68	0.01	0.91	-0.137	3.0E-01
Mtr.43700.1.S1_at	9.55	8.96	9.71	9.42	9.50	9.52	9.41	0.39	9.48	0.05	1.01	0.011	7.6E-01
Mtr.19162.1.S1_at	8.56	7.81	8.02	8.02	7.88	7.96	8.13	0.39	7.95	0.07	0.98	-0.032	4.8E-01
Mtr.22687.1.S1_at	5.80	5.31	6.08	6.41	5.80	5.72	5.73	0.39	5.97	0.38	1.04	0.060	4.8E-01
Mtr.1918.1.S1_at	10.35	10.07	10.84	10.46	10.44	10.45	10.42	0.39	10.45	0.01	1.00	0.004	9.0E-01
Mtr.29043.1.S1_at	2.75	3.40	2.71	2.70	2.66	2.96	2.95	0.39	2.77	0.16	0.94	-0.091	5.0E-01
Mtr.34701.1.S1_at	7.92	7.43	8.21	7.91	7.93	7.89	7.85	0.39	7.91	0.02	1.01	0.010	8.2E-01
Mtr.12730.1.S1_at	11.89	11.26	11.18	11.43	11.38	11.51	11.44	0.39	11.44	0.06	1.00	-0.001	9.9E-01
Mtr.5307.1.S1_s_at	11.05	11.70	11.01	11.33	11.34	11.54	11.25	0.39	11.40	0.12	1.01	0.019	5.6E-01
Mtr.28574.1.S1_at	2.79	2.13	2.10	2.10	2.09	2.09	2.34	0.39	2.09	0.00	0.89	-0.161	3.4E-01
Mtr.31067.1.S1_at	2.47	2.41	3.12	2.36	2.36	2.36	2.67	0.39	2.36	0.00	0.88	-0.177	2.5E-01
Mtr.46668.1.S1_s_at	8.59	8.49	7.87	8.65	8.12	8.44	8.31	0.39	8.40	0.27	1.01	0.015	7.6E-01
Mtr.48884.1.S1_at	7.07	6.45	6.35	6.40	6.36	6.36	6.62	0.39	6.38	0.02	0.96	-0.055	3.3E-01
Mtr.10079.1.S1_at	10.92	10.23	10.89	10.46	10.59	10.53	10.68	0.39	10.53	0.07	0.99	-0.021	5.4E-01
Mtr.42370.1.S1_s_at	8.74	7.98	8.52	8.44	8.63	8.72	8.42	0.39	8.60	0.14	1.02	0.031	4.9E-01
Mtr.44325.1.S1_at	8.20	8.63	8.98	8.67	8.85	8.67	8.60	0.39	8.73	0.10	1.02	0.022	6.1E-01
Mtr.23911.1.S1_at	3.03	3.69	3.00	2.97	2.98	3.30	3.24	0.39	3.08	0.19	0.95	-0.072	5.6E-01
Mtr.29103.1.S1_at	6.31	5.53	5.86	5.77	5.81	5.91	5.90	0.39	5.83	0.07	0.99	-0.018	7.7E-01
Mtr.33495.1.S1_at	3.01	2.44	3.18	3.23	2.85	2.93	2.88	0.39	3.00	0.20	1.04	0.062	6.5E-01
Mtr.9232.1.S1_at	10.65	11.35	11.31	12.08	12.54	12.21	11.10	0.39	12.28	0.24	1.11	0.145	1.1E-02
Mtr.41573.1.S1_at	10.88	10.11	10.64	10.77	10.83	10.78	10.54	0.39	10.79	0.03	1.02	0.034	3.3E-01
Mtr.40846.1.S1_at	10.94	10.17	10.69	11.07	11.11	11.21	10.60	0.39	11.13	0.07	1.05	0.071	8.1E-02
Mtr.46288.1.S1_at	7.65	7.87	8.41	8.13	8.43	7.91	7.98	0.39	8.15	0.26	1.02	0.032	5.5E-01
Mtr.50343.1.S1_s_at	8.75	8.07	8.74	8.75	8.84	8.47	8.52	0.39	8.68	0.19	1.02	0.028	5.5E-01
Mtr.49019.1.S1_at	12.21	11.57	11.49	11.40	11.75	11.81	11.76	0.39	11.65	0.22	0.99	-0.013	7.0E-01
Mtr.31602.1.S1_at	7.26	6.56	7.22	7.42	7.01	6.84	7.01	0.39	7.09	0.30	1.01	0.016	8.0E-01
Mtr.18374.1.S1_x_at	3.35	4.12	3.82	3.75	3.66	3.64	3.76	0.39	3.68	0.06	0.98	-0.031	7.4E-01
Mtr.51790.1.S1_at	2.94	3.59	2.89	2.91	2.87	2.89	3.14	0.39	2.89	0.02	0.92	-0.120	3.3E-01
Mtr.20304.1.S1_at	12.55	11.77	12.11	12.32	12.37	12.44	12.14	0.39	12.38	0.06	1.02	0.028	3.5E-01
Mtr.49599.1.S1_at	2.96	3.62	2.93	2.89	2.83	2.82	3.17	0.39	2.85	0.04	0.90	-0.155	2.3E-01
Mtr.2876.1.S1_at	7.85	7.08	7.55	7.46	7.85	7.92	7.49	0.39	7.75	0.25	1.03	0.048	4.0E-01
Mtr.30064.1.S1_at	3.03	3.70	3.02	3.02	3.02	2.89	3.25	0.39	2.98	0.07	0.92	-0.128	3.0E-01
Mtr.7300.1.S1_at	8.29	8.76	9.06	8.53	8.73	8.55	8.70	0.39	8.60	0.11	0.99	-0.017	6.9E-01
Mtr.15078.1.S1_at	2.58	3.24	2.55	2.53	2.54	2.54	2.79	0.39	2.54	0.01	0.91	-0.137	3.3E-01
Mtr.28442.1.S1_s_at	9.66	8.89	9.41	9.36	9.41	9.48	9.32	0.39	9.42	0.06	1.01	0.015	7.0E-01
Mtr.40400.1.S1_at	4.61	3.87	4.03	4.29	4.44	3.89	4.17	0.39	4.21	0.28	1.01	0.013	9.0E-01
Mtr.33426.1.S1_at	6.95	7.45	7.72	6.36	6.67	5.86	7.37	0.39	6.30	0.41	0.85	-0.228	3.0E-02
Mtr.35870.1.S1_at	6.59	6.67	7.30	6.59	6.38	6.58	6.85	0.39	6.52	0.12	0.95	-0.072	2.3E-01
Mtr.39648.1.S1_s_at	9.22	8.56	9.26	9.20	9.22	9.28	9.01	0.39	9.23	0.04	1.02	0.034	3.9E-01
Mtr.10016.1.S1_at	8.83	8.21	8.93	8.89	8.87	8.77	8.66	0.39	8.84	0.07	1.02	0.030	4.7E-01
Mtr.45582.1.S1_at	10.82	10.15	10.13	10.47	10.59	10.55	10.37	0.39	10.53	0.06	1.02	0.023	5.0E-01
Mtr.41598.1.S1_at	10.38	10.78	11.16	10.73	10.76	10.42	10.77	0.39	10.64	0.19	0.99	-0.018	6.2E-01
Mtr.46960.1.S1_at	3.20	3.89	3.23	3.49	3.21	3.20	3.44	0.39	3.30	0.16	0.96	-0.059	6.0E-01
Mtr.32384.1.S1_s_at	12.19	11.69	11.43	11.39	11.34	11.54	11.77	0.39	11.42	0.10	0.97	-0.043	2.1E-01
Mtr.37440.1.S1_at	12.58	12.86	12.09	11.53	11.03	11.38	12.51	0.39	11.31	0.26	0.90	-0.145	1.2E-02
Mtr.47863.1.S1_s_at	9.78	9.01	9.46	9.64	9.70	9.65	9.42	0.39	9.66	0.03	1.03	0.037	3.3E-01
Mtr.11760.1.S1_at	8.35	8.70	7.92	8.69	9.29	8.99	8.33	0.39	8.99	0.30	1.08	0.111	7.9E-02

Mtr.48366.1.S1_at	8.92	9.02	9.64	9.08	9.08	9.07	9.19	0.39	9.08	0.01	0.99	-0.018	6.4E-01
Mtr.37559.1.S1_at	13.37	12.78	13.51	13.61	13.66	13.46	13.22	0.39	13.58	0.11	1.03	0.039	2.0E-01
Mtr.31974.1.S1_at	7.46	6.85	7.58	7.70	7.67	7.56	7.30	0.39	7.64	0.07	1.05	0.066	2.1E-01
Mtr.38178.1.S1_at	9.03	8.31	8.94	8.78	8.92	8.79	8.76	0.39	8.83	0.08	1.01	0.011	7.8E-01
Mtr.1592.1.S1_at	6.59	6.53	7.24	6.71	6.89	6.70	6.79	0.39	6.77	0.10	1.00	-0.004	9.4E-01
Mtr.20338.1.S1_x_at	3.09	3.72	3.01	2.98	2.91	2.98	3.27	0.39	2.96	0.04	0.90	-0.145	2.4E-01
Mtr.30578.1.S1_at	4.18	3.57	3.46	3.58	3.43	3.42	3.74	0.39	3.48	0.09	0.93	-0.104	3.2E-01
Mtr.30052.1.S1_at	8.29	7.86	8.64	7.85	8.56	8.45	8.26	0.39	8.29	0.38	1.00	0.004	9.4E-01
Mtr.6133.1.S1_at	10.36	9.65	9.73	9.93	9.95	9.86	9.91	0.39	9.91	0.05	1.00	0.000	1.0E+00
Mtr.20432.1.S1_x_at	2.06	2.73	2.05	2.05	2.05	2.05	2.28	0.39	2.05	0.00	0.90	-0.153	3.7E-01
Mtr.41556.1.S1_at	6.37	5.92	5.59	6.08	6.28	6.11	5.96	0.39	6.16	0.11	1.03	0.047	4.5E-01
Mtr.2722.1.S1_at	9.63	8.91	9.02	9.39	9.52	9.45	9.19	0.39	9.45	0.06	1.03	0.041	3.1E-01
Mtr.25743.1.S1_at	5.18	5.73	4.97	4.94	5.64	5.00	5.29	0.39	5.19	0.39	0.98	-0.028	7.7E-01
Mtr.9756.1.S1_at	6.24	5.62	6.34	6.70	6.49	6.56	6.07	0.39	6.58	0.11	1.09	0.118	9.1E-02
Mtr.42292.1.S1_at	9.49	8.95	9.71	9.33	9.35	9.39	9.38	0.39	9.36	0.03	1.00	-0.004	9.2E-01
Mtr.37993.1.S1_at	9.25	9.73	10.02	9.54	9.77	9.58	9.66	0.39	9.63	0.12	1.00	-0.005	8.9E-01
Mtr.3777.1.S1_at	5.50	5.02	5.79	5.20	5.58	5.56	5.44	0.39	5.44	0.22	1.00	0.002	9.8E-01
Mtr.44751.1.S1_at	9.39	8.62	9.05	9.10	9.15	9.12	9.02	0.39	9.12	0.03	1.01	0.016	6.7E-01
Mtr.30993.1.S1_at	3.63	3.06	2.88	3.18	4.34	3.22	3.19	0.39	3.58	0.66	1.12	0.166	4.3E-01
Mtr.26032.1.S1_at	8.65	9.40	9.21	8.77	8.98	8.69	9.08	0.39	8.81	0.15	0.97	-0.044	3.3E-01
Mtr.11635.1.S1_at	8.64	7.86	8.29	8.48	8.38	8.43	8.26	0.39	8.43	0.05	1.02	0.029	5.0E-01
Mtr.8558.1.S1_at	14.39	13.74	13.69	13.74	13.97	13.80	13.94	0.39	13.83	0.12	0.99	-0.011	6.8E-01
Mtr.11036.1.S1_at	9.61	8.83	9.30	9.20	9.33	9.28	9.24	0.39	9.27	0.06	1.00	0.004	9.1E-01
Mtr.39884.1.S1_at	9.56	8.95	8.83	8.99	8.96	8.93	9.11	0.39	8.96	0.03	0.98	-0.024	5.4E-01
Mtr.9277.1.S1_at	10.41	9.66	9.86	10.04	9.95	10.14	9.98	0.39	10.04	0.09	1.01	0.009	7.9E-01
Mtr.46686.1.S1_at	2.79	3.44	2.74	2.74	2.59	2.69	2.99	0.39	2.67	0.08	0.89	-0.162	2.4E-01
Mtr.51959.1.S1_at	4.80	4.06	4.21	3.99	4.61	4.16	4.35	0.39	4.25	0.32	0.98	-0.034	7.4E-01
Mtr.24675.1.S1_at	3.06	3.70	2.99	2.98	2.88	2.96	3.25	0.39	2.94	0.05	0.90	-0.145	2.4E-01
Mtr.8780.1.S1_s_at	8.98	8.20	8.59	8.67	8.84	8.71	8.59	0.39	8.74	0.09	1.02	0.024	5.6E-01
Mtr.42015.1.S1_at	10.17	9.40	9.90	9.84	9.78	9.99	9.83	0.39	9.87	0.11	1.00	0.007	8.6E-01
Mtr.16700.1.S1_at	2.65	3.33	2.66	2.63	2.58	2.61	2.88	0.39	2.61	0.03	0.90	-0.144	2.9E-01
Mtr.44171.1.S1_at	10.24	10.23	10.91	10.23	10.23	10.07	10.46	0.39	10.18	0.09	0.97	-0.039	2.9E-01
Mtr.33191.1.S1_at	4.21	4.91	4.86	4.87	5.45	5.86	4.66	0.39	5.39	0.50	1.16	0.210	1.2E-01
Mtr.32594.1.S1_at	9.20	8.51	9.16	9.39	9.56	9.32	8.96	0.39	9.42	0.12	1.05	0.073	1.2E-01
Mtr.5246.1.S1_at	4.06	3.51	4.25	4.01	4.00	4.01	3.94	0.39	4.01	0.01	1.02	0.025	7.8E-01
Mtr.1085.1.S1_at	8.68	8.42	9.18	8.94	8.74	8.75	8.76	0.39	8.81	0.11	1.01	0.008	8.5E-01
Mtr.19716.1.S1_at	7.34	6.56	7.02	7.11	7.17	6.95	6.97	0.39	7.08	0.11	1.01	0.021	6.8E-01
Mtr.18766.1.S1_at	8.33	8.94	9.06	8.91	8.97	8.79	8.78	0.39	8.89	0.09	1.01	0.018	6.5E-01
Mtr.35075.1.S1_at	4.37	5.08	5.00	5.28	5.01	4.96	4.82	0.39	5.08	0.17	1.05	0.077	3.4E-01
Mtr.1623.1.S1_at	5.64	6.03	6.42	5.99	6.89	6.27	6.03	0.39	6.38	0.46	1.06	0.082	3.7E-01
Mtr.4392.1.S1_at	4.17	4.36	4.92	5.67	5.68	4.91	4.48	0.39	5.42	0.44	1.21	0.275	5.0E-02
Mtr.22060.1.S1_at	11.69	10.94	11.50	11.44	11.46	11.39	11.37	0.39	11.43	0.03	1.00	0.007	8.2E-01
Mtr.5244.1.S1_at	7.11	7.67	6.93	7.09	6.89	7.05	7.23	0.39	7.01	0.10	0.97	-0.045	3.9E-01
Mtr.8770.1.S1_at	10.31	9.61	10.25	10.17	10.12	10.21	10.05	0.39	10.17	0.04	1.01	0.016	6.4E-01
Mtr.45217.1.S1_at	2.83	3.24	3.61	3.59	4.51	3.59	3.23	0.39	3.90	0.53	1.21	0.273	1.5E-01
Mtr.44635.1.S1_at	9.94	9.20	9.77	9.68	10.00	9.69	9.64	0.39	9.79	0.18	1.02	0.023	5.7E-01
Mtr.8761.1.S1_at	12.15	12.57	12.93	12.41	12.62	12.34	12.55	0.39	12.46	0.15	0.99	-0.011	7.2E-01
Mtr.42899.1.S1_a_at	7.57	6.82	7.02	8.33	8.36	8.38	7.14	0.39	8.36	0.02	1.17	0.227	5.6E-03
Mtr.38133.1.S1_at	10.97	10.22	10.75	10.76	10.82	10.87	10.65	0.39	10.81	0.05	1.02	0.023	5.0E-01
Mtr.30421.1.S1_at	9.89	9.12	9.57	9.70	9.85	9.82	9.53	0.39	9.79	0.08	1.03	0.039	3.2E-01
Mtr.12441.1.S1_at	10.89	10.13	10.64	11.12	11.45	11.18	10.55	0.39	11.25	0.18	1.07	0.093	4.7E-02
Mtr.38192.1.S1_at	9.82	9.22	9.94	9.65	9.96	9.70	9.66	0.39	9.77	0.16	1.01	0.016	6.9E-01
Mtr.44375.1.S1_at	9.61	9.94	10.39	9.92	10.10	9.84	9.98	0.39	9.95	0.14	1.00	-0.004	9.0E-01
Mtr.32422.1.S1_at	7.86	7.11	7.67	7.94	7.81	7.72	7.55	0.39	7.82	0.11	1.04	0.051	3.1E-01
Mtr.31304.1.S1_s_at	7.37	6.76	7.48	7.35	7.48	7.17	7.20	0.39	7.34	0.16	1.02	0.027	6.1E-01
Mtr.2278.1.S1_at	8.46	7.69	8.17	8.14	8.31	8.03	8.10	0.39	8.16	0.14	1.01	0.010	8.3E-01
Mtr.1723.1.S1_at	6.17	5.40	5.89	5.92	6.27	6.27	5.82	0.39	6.15	0.20	1.06	0.080	2.6E-01
Mtr.23048.1.S1_at	5.61	4.93	5.60	5.59	5.73	5.55	5.38	0.39	5.62	0.10	1.04	0.063	3.6E-01
Mtr.24113.1.S1_at	2.99	3.64	2.94	2.94	2.83	2.82	3.19	0.39	2.86	0.07	0.90	-0.157	2.2E-01
Mtr.16537.1.S1_at	8.43	7.68	7.89	8.19	8.15	8.19	8.00	0.39	8.18	0.02	1.02	0.032	4.7E-01
Mtr.43269.1.S1_at	10.43	10.06	10.84	10.55	10.54	10.43	10.44	0.39	10.51	0.07	1.01	0.009	7.9E-01
Mtr.1837.1.S1_at	6.09	6.29	6.84	6.91	6.72	6.51	6.41	0.39	6.71	0.20	1.05	0.067	2.9E-01
Mtr.50426.1.S1_at	4.91	4.15	4.38	4.14	3.98	4.11	4.48	0.39	4.08	0.08	0.91	-0.135	1.6E-01
Mtr.41267.1.S1_at	11.45	11.45	12.12	11.70	12.00	11.65	11.67	0.39	11.78	0.19	1.01	0.013	7.0E-01
Mtr.49938.1.S1_at	2.86	3.52	2.84	2.82	2.80	2.79	3.07	0.39	2.80	0.02	0.91	-0.133	2.9E-01
Mtr.46337.1.S1_at	9.72	8.97	9.51	9.59	9.71	9.61	9.40	0.39	9.64	0.07	1.03	0.036	3.6E-01
Mtr.46087.1.S1_at	9.78	9.24	10.00	9.71	9.75	9.75	9.68	0.39	9.74	0.02	1.01	0.009	8.0E-01
Mtr.23742.1.S1_x_at	2.22	2.89	2.21	2.21	2.20	2.21	2.44	0.39	2.21	0.00	0.90	-0.145	3.6E-01
Mtr.46391.1.S1_at	4.23	4.81	4.08	4.52	4.10	4.07	4.37	0.39	4.23	0.25	0.97	-0.048	6.2E-01
Mtr.12656.1.S1_at	10.62	9.84	10.18	10.35	10.12	10.35	10.21	0.39	10.27	0.13	1.01	0.009	8.1E-01
Mtr.49279.1.S1_at	8.33	9.04	8.41	8.49	8.37	8.44	8.59	0.39	8.43	0.06	0.98	-0.027	5.2E-01
Mtr.23392.1.S1_x_at	13.15	12.50	12.46	12.69	12.49	12.76	12.71	0.39	12.65	0.14	1.00	-0.007	8.2E-01
Mtr.26456.1.S1_at	9.17	8.65	9.41	9.28	9.12	9.16	9.08	0.39	9.19	0.08	1.01	0.017	6.6E-01
Mtr.36710.1.S1_at	2.91	3.58	2.90	2.88	2.88	2.86	3.13	0.39	2.87	0.01	0.92	-0.124	3.2E-01
Mtr.2126.1.S1_at	8.13	7.36	7.70	7.79	7.88	7.80	7.73	0.39	7.83	0.05	1.01	0.018	6.9E-01
Mtr.50538.1.S1_at	3.02	3.67	2.99	2.96	2.93	2.92	3.23	0.39	2.94	0.02	0.91	-0.135	2.7E-01
Mtr.37614.1.S1_at	12.13	11.38	11.93	11.91	12.27	11.95	11.81	0.39	12.04	0.19	1.02	0.028	4.1E-01
Mtr.20954.1.S1_at	2.69	3.35	2.67	3.26	2.84	4.02	2.90	0.39	3.37	0.60	1.16	0.218	3.1E-01
Mtr.743.1.S1_at	10.48	9.90	10.63	10.66	10.47	10.55	10.34	0.39	10.56	0.09	1.02	0.031	3.9E-01
Mtr.9213.1.S1_at	9.69	8.91	9.30	9.57	9.48	9.58	9.30	0.39	9.54	0.05	1.03	0.037	3.5E-01
Mtr.44775.1.S1_at	9.11	8.36	8.91	9.20	8.87	9.02	8.79	0.39	9.03	0.17	1.03	0.038	3.9E-01
Mtr.25158.1.S1_at	2.76	3.44	2.77	2.74	2.73	2.66	2.99	0.39	2.71	0.04	0.91	-0.141	2.8E-01
Mtr.43828.1.S1_at	5.40	4.69	5.31	5.45	5.86	5.98	5.13	0.39	5.76	0.28	1.12	0.168	8.3E-02
Mtr.43566.1.S1_at	9.59	9.04	9.78	9.47	9.79	9.57	9.47	0.39	9.61	0.16	1.01	0.021	6.0E-01
Mtr.34675.1.S1_at	2.72	3.38	2.69	2.68	2.64	2.64	2.93	0.39	2.65	0.02	0.91	-0.144	2.8E-01
Mtr.36324.1.S1_at	3.05	3.75	3.11	4.05	2.95	3.30	3.30	0.39	3.43	0.56	1.04	0.055	7.6E-01
Mtr.52212.1.S1_s_at	4.22	3.70	3.46	3.58	3.38	3.45	3.79	0.39	3.47	0.10	0.91	-0.129	2.3E-01
Mtr.40981.1.S1_at	11.40	10.71	11.37	11.45	11.25	11.16	11.16	0.39	11.29	0.15	1.01	0.016	6.2E-01
Mtr.3470.1.S1_at	6.08	5.30	5.67	5.54	6.26	5.88	5.68	0.39	5.89	0.36	1.04	0.053	5.3E-01
Mtr.5741.1.S1_s_at	5.43	5.73	6.20	5.92	5.78	5.84	5.79	0.39	5.85	0.08	1.01	0.014	8.2E-01
Mtr.50971.1.S1_at	2.89	3.55	2.87	2.84	2.86	2.78	3.10	0.39	2.83	0.04	0.91	-0.133	2.9E-01
Mtr.48894.1.S1_x_at	2.58	2.60	3.26	2.57	2.57	2.58	2.81	0.39	2.57	0.00	0.91	-0.129	3.4E-01
Mtr.5444.1.S1_s_at	6.11	5.81	5.34	4.78	5.72	5.98	5.75	0.39	5.49	0.63	0.95	-0.067	5.8E-01
Mtr.7077.1.S1_at	10.69	9.96	10.10	10.66	10.26	10.53	10.25	0.39	10.48	0.21	1.02	0.032	4.2E-01
Mtr.7044.1.S1_at	3.04	2.46	2.31	2.30	2.30	2.29	2.60	0.39	2.30	0.01	0.88	-0.181	2.4E-01
Mtr.6169.1.S1_at	4.27	5.04	4.72	4.48	4.51	4.47	4.68	0.39	4.49	0.02	0.96	-0.060	4.5E-01
Mtr.7274.1.S1_at	11.35	10.60	11.15	11.14	11.06	11.18	11.04	0.39	11.13	0.06	1.01	0.012	7.1E-01
Mtr.33649.1.S1_at	2.70	3.36	2.67	2.62	2.60	2.63	2.91	0.39	2.62	0.01	0.90	-0.153	2.6E-01
Mtr.45964.1.S1_at	2.81	3.45	2.76	2.77	2.77	2.76	3.01	0.39	2.77	0.01	0.92	-0.118	3.5E-01
Mtr.42263.1.S1_s_at	9.29	8.59	9.22	8.90	8.66	9.13	9.04	0.39	8.90	0.24	0.98	-0.022	6.3E-01
Mtr.41420.1.S1_s_at	7.84	7.30	8.05	8.46	9.23	8.57	7.73	0.39	8.76	0.41	1.13	0.179	3.5E-02
Mtr.33393.1.S1_at	7.42	6.83	7.56	7.53	7.57	7.78	7.27	0.39	7.63	0.13	1.05	0.069	2.1E-01
Mtr.16966.1.S1_x_at	2.77	3.43	2.74	2.72	2.71	2.66	2.98	0.39	2.69	0.03	0.90	-0.147	2.7E-01
Mtr.21729.1.S1_at	3.05	3.69	2.99	3.00	2.90	3.01	3.24	0.39	2.97	0.06	0.92	-0.126	3.0E-01
Mtr.26540.1.S1_at	2.38	2.55	3.12	2.34	2.33	2.33	2.68	0.39	2.34	0.01	0.87	-0.199	2.0E-01
Mtr.33606.1.S1_at	6.17	5.51	6.20	6.27	6.15	6.44	5.96	0.39	6.29	0.14	1.05	0.077	2.4E-01
Mtr.27055.1.S1_s_at	8.81	8.10	8.72	8.64	8.58	8.72	8.54	0.39	8.65	0.07	1.01	0.017	6.8E-01
Mtr.5102.1.S1_at	11.27	10.52	10.72	10.91	10.80	10.95	10.84	0.39	10.89	0.08	1.00	0.006	8.5E-01
Mtr.46091.1.S1_at	3.16	3.82	3.14	3.12	2.94	3.11	3.37	0.39	3.06	0.10	0.91	-0.141	2.5E-01
Mtr.11679.1.S1_at	6.50	6.63	7.23	7.14	7.19	7.36	6.79	0.39	7.23	0.12	1.07	0.092	1.3E-01
Mtr.33384.1.S1_at	6.66	6.96	7.42	6.80	6.67	6.74	7.01	0.39	6.74	0.06	0.96	-0.058	2.9E-01
Mtr.47245.1.S1_s_at	7.13	6.36	6.78	7.09	7.06	6.93	6.75	0.39	7.03	0.08	1.04	0.057	3.0E-01
Mtr.3230.1.S1_at	11.64	10.88	11.13	11.34	11.38	11.36	11.22	0.39	11.36	0.02	1.01	0.019	5.5E-01
Mtr.7825.1.S1_at	2.98	3.60	2.89	2.88	2.86	2.83	3.16	0.39	2.86	0.03	0.91	-0.144	2.5E-01
Mtr.19525.1.S1_at	3.05	3.69	3.00	3.00	2.90	3.05	3.25	0.39	2.98	0.07	0.92	-0.123	3.1E-01
Mtr.15777.1.S1_at	10.00	9.23	9.63	9.96	10.03	9.79	9.62	0.39	9.92	0.13	1.03	0.045	2.6E-01
Mtr.43980.1.S1_at	9.19	8.81	9.59	9.32	9.06	9.10	9.20	0.39	9.16	0.14	1.00	-0.005	8.9E-01
Mtr.32772.1.S1_at	7.58	8.25	7.59	7.47	7.31	7.52	7.81	0.39	7.43	0.11	0.95	-0.071	1.8E-01
Mtr.774.1.S1_at	9.28	8.50	8.85	8.91	8.96	8.94	8.88	0.39	8.94	0.03	1.01	0.010	8.0E-01
Mtr.39783.1.S1_at	8.09	7.33	7.82	8.31	7.88	8.08	7.75	0.39	8.09	0.21	1.04	0.063	2.5E-01
Mtr.13035.1.S1_at	5.33	4.73	5.45	5.43	5.16	5.43	5.17	0.39	5.34	0.16	1.03	0.046	5.3E-01
Mtr.32398.1.S1_at	7.86	7.17	7.81	8.05	8.13	8.13	7.61	0.39	8.10	0.05	1.06	0.090	9.6E-02
Mtr.25068.1.S1_at	6.41	6.20	6.95	6.73	7.21	6.50	6.52	0.39	6.81	0.36	1.04	0.063	3.9E-01
Mtr.50436.1.S1_at	2.84	3.50	2.83	2.80	2.74	2.79	3.06	0.39	2.78	0.03	0.91	-0.137	2.8E-01
Mtr.38656.1.S1_at	3.26	2.60	2.58	2.57	2.58	2.57	2.81	0.39	2.57	0.01	0.91	-0.130	3.4E-01
Mtr.33328.1.S1_at	8.52	7.82	7.89	8.72	8.40	8.65	8.08	0.39	8.59	0.17	1.06	0.089	1.0E-01
Mtr.15261.1.S1_s_at	13.01	12.90	12.29	12.65	12.27	12.67	12.73	0.39	12.53	0.22	0.98	-0.023	4.8E-01
Mtr.28410.1.S1_at	8.68	8.11	8.85	8.71	8.44	8.82	8.55	0.39	8.66	0.20	1.01	0.018	6.8E-01
Mtr.47672.1.S1_at	4.67	5.31	4.62	4.60	4.58	4.49	4.87	0.39	4.56	0.06	0.94	-0.095	2.4E-01
Mtr.44466.1.S1_at	10.70	9.93	10.34	10.54	10.38	10.43	10.32	0.39	10.45	0.08	1.01	0.018	6.0E-01
Mtr.45035.1.S1_at	6.71	6.79	7.41	6.94	7.12	6.63	6.97	0.39	6.90	0.25	0.99	-0.015	8.0E-01

Mtr.35878.1.S1_at	8.97	8.60	9.37	8.76	8.90	8.79	8.98	0.39	8.82	0.08	0.98	-0.026	5.1E-01
Mtr.25178.1.S1_at	7.17	7.94	7.51	7.15	7.29	7.28	7.54	0.39	7.24	0.08	0.96	-0.059	2.5E-01
Mtr.12986.1.S1_at	9.94	9.45	10.22	10.05	10.04	9.96	9.87	0.39	10.01	0.05	1.01	0.021	5.5E-01
Mtr.14827.1.S1_s_at	7.79	7.31	7.03	7.31	7.24	7.69	7.38	0.39	7.41	0.24	1.00	0.007	9.0E-01
Mtr.4756.1.S1_at	3.15	3.76	3.04	3.17	2.97	2.97	3.32	0.39	3.04	0.11	0.91	-0.128	2.9E-01
Mtr.1675.1.S1_at	7.87	7.34	8.10	8.69	8.45	8.44	7.77	0.39	8.53	0.14	1.10	0.134	3.3E-02
Mtr.44272.1.S1_at	8.61	8.62	9.28	8.91	8.99	8.85	8.84	0.39	8.92	0.07	1.01	0.013	7.4E-01
Mtr.22162.1.S1_x_at	4.14	3.80	3.37	3.70	3.70	4.13	3.77	0.39	3.84	0.25	1.02	0.027	8.0E-01
Mtr.32551.1.S1_s_at	9.28	8.56	9.16	9.13	9.18	9.19	9.00	0.39	9.16	0.03	1.02	0.026	5.0E-01
Mtr.11113.1.S1_at	8.79	8.12	8.79	8.75	8.85	8.93	8.57	0.39	8.84	0.09	1.03	0.045	3.0E-01
Mtr.5682.1.S1_s_at	10.66	9.95	10.57	10.60	10.52	10.52	10.40	0.39	10.55	0.04	1.01	0.021	5.4E-01
Mtr.48129.1.S1_x_at	2.76	3.42	2.74	2.71	2.71	2.67	2.97	0.39	2.70	0.02	0.91	-0.141	2.8E-01
Mtr.37832.1.S1_at	9.07	8.33	8.87	8.41	8.33	8.32	8.76	0.39	8.36	0.05	0.95	-0.068	1.5E-01
Mtr.43862.1.S1_at	12.61	11.84	12.19	12.49	11.96	12.54	12.21	0.39	12.33	0.32	1.01	0.013	7.2E-01
Mtr.38202.1.S1_at	9.42	8.71	9.32	9.50	9.47	9.67	9.15	0.39	9.55	0.11	1.04	0.062	1.6E-01
Mtr.31730.1.S1_at	8.10	7.72	8.49	8.75	8.51	8.47	8.10	0.39	8.58	0.15	1.06	0.082	1.2E-01
Mtr.51836.1.S1_at	2.23	2.89	2.22	2.23	2.21	2.21	2.45	0.39	2.21	0.01	0.90	-0.145	3.5E-01
Mtr.41951.1.S1_at	9.64	8.90	9.47	9.55	9.49	9.56	9.34	0.39	9.53	0.04	1.02	0.030	4.3E-01
Mtr.2483.1.S1_at	8.07	7.70	8.47	8.12	8.23	8.37	8.08	0.39	8.24	0.13	1.02	0.028	5.3E-01
Mtr.12457.1.S1_s_at	11.36	10.59	10.89	11.08	10.84	11.22	10.95	0.39	11.05	0.19	1.01	0.013	7.1E-01
Mtr.8880.1.S1_at	8.94	9.64	9.57	10.61	11.32	11.07	9.38	0.39	11.00	0.36	1.17	0.230	6.0E-03
Mtr.15975.1.S1_s_at	8.48	9.21	9.05	8.86	8.79	9.16	8.91	0.39	8.94	0.20	1.00	0.004	9.3E-01
Mtr.18806.1.S1_at	7.75	6.98	7.42	7.58	7.69	7.52	7.38	0.39	7.60	0.09	1.03	0.042	3.9E-01
Mtr.49940.1.S1_at	7.53	7.03	7.79	7.76	7.92	7.75	7.45	0.39	7.81	0.10	1.05	0.068	1.9E-01
Mtr.28834.1.S1_at	6.48	6.34	7.07	7.05	7.37	6.89	6.63	0.39	7.11	0.25	1.07	0.100	1.5E-01
Mtr.22121.1.S1_at	2.76	3.40	2.71	2.68	2.65	2.64	2.96	0.39	2.66	0.02	0.90	-0.154	2.5E-01
Mtr.50022.1.S1_at	2.96	3.62	2.95	3.20	2.87	2.85	3.18	0.39	2.97	0.20	0.94	-0.095	4.6E-01
Mtr.31312.1.S1_at	11.28	10.75	10.53	11.02	11.04	11.19	10.85	0.39	11.08	0.09	1.02	0.031	3.7E-01
Mtr.32484.1.S1_at	6.89	6.12	6.48	6.98	6.62	6.69	6.50	0.39	6.76	0.19	1.04	0.058	3.4E-01
Mtr.22238.1.S1_at	12.51	12.21	11.75	12.42	11.44	12.15	12.16	0.39	12.00	0.50	0.99	-0.018	6.9E-01
Mtr.6336.1.S1_at	3.84	3.17	3.83	4.17	3.83	3.83	3.62	0.39	3.94	0.20	1.09	0.125	2.6E-01
Mtr.7840.1.S1_at	6.54	7.19	7.22	6.74	7.19	6.67	6.98	0.39	6.87	0.28	0.98	-0.023	7.0E-01
Mtr.42940.1.S1_at	11.48	10.71	11.10	11.11	11.31	11.25	11.10	0.39	11.22	0.10	1.01	0.016	6.2E-01
Mtr.10272.1.S1_at	10.09	10.79	10.72	10.50	10.65	10.66	10.54	0.39	10.60	0.09	1.01	0.009	7.9E-01
Mtr.22897.1.S1_at	8.83	8.97	9.56	9.16	9.65	9.17	9.12	0.39	9.33	0.28	1.02	0.033	4.9E-01
Mtr.8370.1.S1_at	2.91	3.57	2.89	2.86	2.84	2.83	3.12	0.39	2.85	0.02	0.91	-0.134	2.8E-01
Mtr.29210.1.S1_at	9.25	8.79	8.49	9.13	8.98	9.20	8.85	0.39	9.11	0.11	1.03	0.042	3.2E-01
Mtr.47343.1.S1_s_at	8.99	9.18	9.73	9.08	9.13	9.09	9.30	0.39	9.10	0.03	0.98	-0.032	4.2E-01
Mtr.18888.1.S1_at	2.90	3.54	2.84	2.84	2.79	2.85	3.10	0.39	2.83	0.03	0.91	-0.130	3.0E-01
Mtr.38418.1.S1_at	8.61	7.88	8.45	8.44	8.42	8.36	8.31	0.39	8.41	0.04	1.01	0.017	6.8E-01
Mtr.14751.1.S1_at	10.94	10.18	10.66	10.64	10.50	10.72	10.59	0.39	10.62	0.11	1.00	0.004	9.0E-01
Mtr.34490.1.S1_at	4.58	4.24	5.01	4.75	4.60	4.37	4.61	0.39	4.58	0.19	0.99	-0.011	9.0E-01
Mtr.51848.1.S1_x_at	2.63	3.29	2.61	2.57	2.57	2.50	2.84	0.39	2.55	0.04	0.90	-0.159	2.6E-01
Mtr.42009.1.S1_at	9.20	8.49	9.11	9.11	9.22	9.04	8.93	0.39	9.12	0.09	1.02	0.030	4.6E-01
Mtr.45561.1.S1_at	9.38	8.63	9.17	9.13	8.95	8.90	9.06	0.39	8.99	0.12	0.99	-0.011	7.9E-01
Mtr.18138.1.S1_at	2.96	3.61	2.93	2.92	2.87	3.18	3.17	0.39	2.99	0.17	0.94	-0.085	5.0E-01
Mtr.23850.1.S1_at	11.64	10.87	11.23	11.24	11.07	11.12	11.24	0.39	11.14	0.09	0.99	-0.013	6.7E-01
Mtr.45587.1.S1_at	9.70	8.95	9.47	9.88	9.91	9.75	9.37	0.38	9.84	0.09	1.05	0.071	1.1E-01
Mtr.48476.1.S1_at	7.84	7.67	8.40	8.33	8.43	8.19	7.97	0.38	8.32	0.12	1.04	0.062	2.1E-01
Mtr.45285.1.S1_at	7.51	6.74	7.12	7.32	6.94	7.11	7.12	0.38	7.12	0.19	1.00	0.001	9.9E-01
Mtr.24159.1.S1_at	2.62	3.28	2.60	2.56	2.57	2.56	2.83	0.38	2.56	0.01	0.91	-0.144	2.9E-01
Mtr.48105.1.S1_at	8.60	9.34	9.14	9.20	9.12	8.88	9.03	0.38	9.07	0.17	1.00	0.006	8.8E-01
Mtr.13201.1.S1_at	12.94	12.24	12.86	11.82	11.47	11.84	12.68	0.38	11.71	0.21	0.92	-0.115	1.9E-02
Mtr.25966.1.S1_at	3.27	3.28	2.61	3.49	3.17	3.48	3.05	0.38	3.38	0.18	1.11	0.147	2.5E-01
Mtr.22782.1.S1_at	2.65	3.32	2.65	2.62	2.60	2.61	2.87	0.38	2.61	0.01	0.91	-0.139	3.0E-01
Mtr.19655.1.S1_s_at	9.66	8.89	9.25	9.33	9.27	9.45	9.27	0.38	9.35	0.09	1.01	0.012	7.4E-01
Mtr.37111.1.S1_at	5.17	4.49	5.15	5.10	5.10	5.09	4.94	0.38	5.10	0.01	1.03	0.045	5.2E-01
Mtr.13400.1.S1_at	8.65	7.93	8.50	8.83	8.58	8.64	8.36	0.38	8.68	0.13	1.04	0.054	2.5E-01
Mtr.15992.1.S1_at	4.43	5.13	5.06	6.00	6.58	6.74	4.88	0.38	6.44	0.39	1.32	0.402	7.8E-03
Mtr.27650.1.S1_at	3.85	3.90	4.54	4.19	4.29	4.16	4.10	0.38	4.21	0.07	1.03	0.040	6.3E-01
Mtr.2883.1.S1_at	8.29	7.53	7.81	8.22	8.20	8.29	7.87	0.38	8.24	0.04	1.05	0.065	1.8E-01
Mtr.37927.1.S1_at	12.74	12.79	13.43	13.00	12.98	12.81	12.99	0.38	12.93	0.10	1.00	-0.007	8.1E-01
Mtr.1979.1.S1_at	4.57	4.15	3.80	3.70	3.74	4.00	4.17	0.38	3.81	0.16	0.91	-0.130	2.1E-01
Mtr.22638.1.S1_at	2.93	3.60	2.93	2.94	2.94	2.94	3.15	0.38	2.94	0.00	0.93	-0.099	4.0E-01
Mtr.11332.1.S1_at	9.51	9.05	9.81	9.33	9.31	9.09	9.46	0.38	9.24	0.13	0.98	-0.033	4.2E-01
Mtr.30739.1.S1_s_at	10.23	9.59	9.55	9.55	9.53	9.61	9.79	0.38	9.56	0.04	0.98	-0.034	3.6E-01
Mtr.28717.1.S1_at	9.89	9.25	9.21	9.41	9.24	9.49	9.45	0.38	9.38	0.13	0.99	-0.010	7.8E-01
Mtr.37708.1.S1_at	7.46	8.06	8.17	8.78	9.48	9.41	7.90	0.38	9.22	0.38	1.17	0.224	1.3E-02
Mtr.11840.1.S1_at	8.51	7.80	8.41	8.63	9.01	8.75	8.24	0.38	8.80	0.19	1.07	0.094	8.9E-02
Mtr.16873.1.S1_s_at	2.75	3.41	2.73	2.74	2.73	2.71	2.96	0.38	2.73	0.02	0.92	-0.118	3.5E-01
Mtr.43410.1.S1_at	9.05	8.28	8.71	8.75	9.49	9.16	8.68	0.38	9.13	0.37	1.05	0.074	2.2E-01
Mtr.41135.1.S1_at	9.46	8.72	9.28	9.43	9.51	9.42	9.15	0.38	9.45	0.05	1.03	0.046	2.6E-01
Mtr.41873.1.S1_at	9.12	8.44	9.09	9.36	9.15	9.09	8.88	0.38	9.20	0.14	1.04	0.051	2.5E-01
Mtr.41146.1.S1_at	8.58	8.61	9.26	8.78	8.76	8.45	8.82	0.38	8.66	0.18	0.98	-0.025	5.7E-01
Mtr.24534.1.S1_at	6.87	6.60	7.36	8.60	8.54	8.54	6.94	0.38	8.56	0.04	1.23	0.302	1.9E-03
Mtr.23144.1.S1_x_at	4.46	5.09	4.39	4.33	4.26	4.33	4.64	0.38	4.31	0.04	0.93	-0.108	2.1E-01
Mtr.15013.1.S1_s_at	2.70	3.34	2.66	2.65	2.63	2.61	2.90	0.38	2.63	0.02	0.91	-0.142	2.9E-01
Mtr.5334.1.S1_at	6.00	5.27	5.85	5.94	5.95	5.97	5.71	0.38	5.95	0.01	1.04	0.060	3.4E-01
Mtr.43551.1.S1_at	10.68	10.20	10.96	10.58	10.79	10.49	10.62	0.38	10.62	0.15	1.00	0.001	9.8E-01
Mtr.11693.1.S1_s_at	5.96	5.86	5.25	5.81	6.40	6.45	5.69	0.38	6.22	0.36	1.09	0.128	1.5E-01
Mtr.15849.1.S1_at	11.63	11.80	12.36	11.92	11.85	11.52	11.93	0.38	11.76	0.21	0.99	-0.020	5.5E-01
Mtr.9757.1.S1_at	10.73	10.02	10.65	10.57	10.59	10.59	10.47	0.38	10.58	0.01	1.01	0.016	6.3E-01
Mtr.5312.1.S1_s_at	10.29	9.58	10.19	10.19	10.07	10.26	10.02	0.38	10.18	0.10	1.02	0.023	5.3E-01
Mtr.52054.1.S1_at	8.24	8.02	8.77	8.32	8.28	8.20	8.34	0.38	8.27	0.06	0.99	-0.014	7.4E-01
Mtr.11227.1.S1_at	10.43	9.67	9.96	9.99	10.00	10.03	10.02	0.38	10.01	0.02	1.00	-0.002	9.6E-01
Mtr.39245.1.S1_at	8.26	7.51	8.03	7.99	8.05	7.84	7.93	0.38	7.96	0.11	1.00	0.005	9.2E-01
Mtr.4048.1.S1_at	12.28	11.57	11.68	11.79	12.08	11.84	11.84	0.38	11.91	0.15	1.01	0.008	8.0E-01
Mtr.9591.1.S1_at	6.66	6.95	7.42	7.07	7.47	7.09	7.01	0.38	7.21	0.22	1.03	0.042	4.7E-01
Mtr.2068.1.S1_at	6.41	5.76	6.44	6.73	7.10	6.28	6.20	0.38	6.70	0.41	1.08	0.112	2.0E-01
Mtr.38754.1.S1_at	8.65	8.20	8.97	8.96	8.84	8.87	8.60	0.38	8.89	0.07	1.03	0.047	2.7E-01
Mtr.12068.1.S1_at	8.13	7.77	8.54	8.39	8.38	8.36	8.15	0.38	8.38	0.02	1.03	0.040	3.6E-01
Mtr.26162.1.S1_s_at	9.56	10.26	10.19	10.25	10.18	10.14	10.01	0.38	10.19	0.05	1.02	0.026	4.6E-01
Mtr.41674.1.S1_at	8.21	7.54	8.20	8.19	8.63	8.19	7.98	0.38	8.34	0.25	1.04	0.063	2.5E-01
Mtr.38913.1.S1_at	8.79	8.19	8.08	8.40	8.16	8.50	8.36	0.38	8.35	0.17	1.00	-0.001	9.9E-01
Mtr.19406.1.S1_at	2.84	3.47	2.78	2.76	2.76	2.75	3.03	0.38	2.76	0.01	0.91	-0.135	2.9E-01
Mtr.44054.1.S1_at	8.74	8.06	8.10	8.59	8.63	8.67	8.30	0.38	8.63	0.04	1.04	0.056	2.1E-01
Mtr.26889.1.S1_at	8.35	7.74	8.45	8.39	8.50	8.25	8.18	0.38	8.38	0.13	1.02	0.035	4.4E-01
Mtr.10929.1.S1_at	8.16	7.72	8.48	8.19	8.32	8.25	8.12	0.38	8.25	0.07	1.02	0.024	5.8E-01
Mtr.11866.1.S1_at	6.99	6.54	6.23	6.89	6.53	6.88	6.58	0.38	6.77	0.21	1.03	0.040	5.1E-01
Mtr.22003.1.S1_x_at	3.43	2.66	3.10	3.41	4.25	3.93	3.06	0.38	3.86	0.42	1.26	0.333	7.3E-02
Mtr.50288.1.S1_at	6.89	6.27	6.97	6.87	6.37	6.42	6.71	0.38	6.55	0.27	0.98	-0.034	5.9E-01
Mtr.44359.1.S1_at	9.46	8.87	9.59	9.49	9.73	9.38	9.31	0.38	9.54	0.18	1.02	0.035	4.0E-01
Mtr.25658.1.S1_at	10.63	10.05	10.77	10.67	10.63	10.50	10.48	0.38	10.60	0.09	1.01	0.016	6.2E-01
Mtr.8998.1.S1_at	10.81	11.52	11.42	10.96	11.32	11.19	11.25	0.38	11.16	0.18	0.99	-0.012	7.2E-01
Mtr.43030.1.S1_at	8.36	7.61	8.16	7.89	8.15	8.25	8.04	0.38	8.10	0.19	1.01	0.010	8.3E-01
Mtr.2844.1.S1_at	3.29	2.68	2.58	3.14	2.57	2.66	2.85	0.38	2.79	0.31	0.98	-0.029	8.5E-01
Mtr.1906.1.S1_at	8.11	7.87	8.62	8.16	8.42	8.23	8.20	0.38	8.27	0.13	1.01	0.012	7.8E-01
Mtr.52025.1.S1_at	9.00	9.32	9.77	9.58	9.51	9.37	9.36	0.38	9.49	0.11	1.01	0.019	6.1E-01
Mtr.29774.1.S1_at	2.48	3.14	2.46	2.45	2.44	2.44	2.69	0.38	2.44	0.00	0.91	-0.141	3.2E-01
Mtr.3478.1.S1_at	4.49	4.80	5.25	5.03	5.97	5.29	4.85	0.38	5.43	0.49	1.12	0.164	1.8E-01
Mtr.25504.1.S1_at	2.97	3.61	2.93	2.95	2.94	2.95	3.17	0.38	2.95	0.01	0.93	-0.105	3.7E-01
Mtr.46012.1.S1_x_at	8.51	7.97	8.71	8.71	9.29	8.95	8.40	0.38	8.99	0.29	1.07	0.098	1.0E-01
Mtr.25774.1.S1_x_at	5.78	5.75	5.10	5.58	5.66	5.84	5.55	0.38	5.69	0.13	1.03	0.038	5.6E-01
Mtr.16941.1.S1_at	10.31	9.55	10.02	9.88	9.90	9.85	9.96	0.38	9.88	0.02	0.99	-0.012	7.3E-01
Mtr.6041.1.S1_at	4.74	5.49	5.23	6.11	5.61	5.59	5.15	0.38	5.77	0.30	1.12	0.164	9.0E-02
Mtr.23335.1.S1_at	9.62	8.86	9.32	9.27	9.32	9.36	9.27	0.38	9.32	0.05	1.01	0.008	8.2E-01
Mtr.22234.1.S1_x_at	3.07	3.70	3.01	2.98	2.92	2.91	3.26	0.38	2.94	0.04	0.90	-0.150	2.2E-01
Mtr.50108.1.S1_s_at	3.12	3.75	3.06	3.03	3.07	3.06	3.31	0.38	3.05	0.02	0.92	-0.116	3.1E-01
Mtr.42426.1.S1_at	3.47	2.87	2.75	2.88	2.82	2.81	3.03	0.38	2.84	0.04	0.94	-0.096	4.3E-01
Mtr.31917.1.S1_at	10.00	10.69	10.63	10.13	10.39	10.02	10.44	0.38	10.18	0.19	0.98	-0.036	3.6E-01
Mtr.41513.1.S1_at	9.15	9.25	9.86	9.44	9.31	9.38	9.42	0.38	9.38	0.06	1.00	-0.006	8.6E-01
Mtr.1312.1.S1_at	3.80	3.32	4.08	3.78	3.80	3.87	3.73	0.38	3.82	0.05	1.02	0.032	7.3E-01
Mtr.20223.1.S1_at	8.97	8.45	9.19	9.54	9.55	9.38	8.87	0.38	9.49	0.10	1.07	0.098	5.3E-02
Mtr.2114.1.S1_at	9.35	10.12	9.80	9.47	9.33	9.35	9.76	0.38	9.38	0.08	0.96	-0.057	1.7E-01
Mtr.44129.1.S1_at	4.07	3.33	3.89	3.58	3.57	3.43	3.76	0.38	3.52	0.08	0.94	-0.096	3.5E-01
Mtr.35582.1.S1_at	8.57	7.89	8.54	8.45	8.45	8.27	8.33	0.38	8.39	0.11	1.01	0.009	8.3E-01
Mtr.7082.1.S1_at	7.85	7.22	7.91	8.05	8.18	8.03	7.66	0.38	8.09	0.08	1.06	0.078	1.3E-01
Mtr.11880.1.S1_at	3.40	4.06	3.39	3.39	3.04	3.38	3.62	0.38	3.27	0.20	0.90	-0.145	2.4E-01
Mtr.25055.1.S1_at	8.05	7.56	8.32	8.05	8.09	8.16	7.98	0.38	8.10	0.05	1.02	0.022	6.2E-01
Mtr.27113.1.S1_s_at	10.22	9.57	10.24	10.17	10.13	10.05	10.01	0.38	10.12	0.06	1.01	0.015	6.6E-01
Mtr.48985.1.S1_at	12.63	11.87	12.33	12.58	12.49	12.56	12.28	0.38	12.54	0.05	1.02	0.031	3.0E-01

Mtr.41797.1.S1_at	9.13	8.42	8.52	9.00	8.75	9.13	8.69	0.38	8.96	0.19	1.03	0.044	3.4E-01
Mtr.18592.1.S1_at	8.50	8.99	8.24	8.84	9.16	8.93	8.58	0.38	8.98	0.16	1.05	0.066	1.7E-01
Mtr.46809.1.S1_at	2.83	3.49	2.82	2.79	2.76	2.75	3.05	0.38	2.77	0.02	0.91	-0.139	2.8E-01
Mtr.26277.1.S1_at	10.56	9.81	10.08	10.23	10.13	10.21	10.15	0.38	10.19	0.05	1.00	0.005	8.7E-01
Mtr.18994.1.S1_at	6.49	5.81	6.45	6.88	6.79	6.67	6.25	0.38	6.78	0.11	1.08	0.117	8.2E-02
Mtr.35015.1.S1_s_at	9.07	8.36	8.46	8.56	9.01	8.88	8.63	0.38	8.82	0.23	1.02	0.030	5.2E-01
Mtr.13347.1.S1_at	9.79	9.11	9.14	9.35	9.33	9.61	9.35	0.38	9.43	0.16	1.01	0.012	7.5E-01
Mtr.20403.1.S1_at	2.84	3.48	2.80	2.82	2.76	2.84	3.04	0.38	2.81	0.04	0.92	-0.115	3.5E-01
Mtr.4000.1.S1_at	2.47	3.13	2.46	2.44	2.45	2.43	2.69	0.38	2.44	0.01	0.91	-0.138	3.3E-01
Mtr.26484.1.S1_at	2.87	3.45	2.73	2.70	2.74	2.72	3.02	0.38	2.72	0.02	0.90	-0.150	2.5E-01
Mtr.13637.1.S1_at	8.25	7.69	8.42	8.59	8.32	8.42	8.12	0.38	8.44	0.14	1.04	0.056	2.4E-01
Mtr.39275.1.S1_at	7.37	7.03	7.79	7.32	7.89	7.80	7.40	0.38	7.67	0.31	1.04	0.052	3.9E-01
Mtr.37374.1.S1_at	14.25	13.80	13.49	13.65	13.98	13.89	13.85	0.38	13.84	0.17	1.00	-0.001	9.8E-01
Mtr.11661.1.S1_at	9.55	10.08	10.30	9.75	9.82	9.87	9.98	0.38	9.82	0.06	0.98	-0.023	5.1E-01
Mtr.31455.1.S1_at	8.77	9.20	8.44	8.52	8.82	8.66	8.80	0.38	8.67	0.15	0.98	-0.022	6.0E-01
Mtr.37955.1.S1_at	4.04	3.29	3.81	5.29	4.86	4.86	3.71	0.38	5.00	0.25	1.35	0.431	8.0E-03
Mtr.49673.1.S1_at	2.69	3.31	2.62	2.61	2.61	3.19	2.87	0.38	2.80	0.34	0.98	-0.036	8.2E-01
Mtr.11648.1.S1_at	6.87	6.43	6.10	6.43	6.55	6.45	6.47	0.38	6.47	0.07	1.00	0.002	9.7E-01
Mtr.42512.1.S1_at	8.25	7.70	8.44	8.14	8.35	8.09	8.13	0.38	8.19	0.14	1.01	0.012	7.9E-01
Mtr.10200.1.S1_at	9.04	8.28	8.67	8.94	8.96	8.91	8.66	0.38	8.94	0.03	1.03	0.045	2.8E-01
Mtr.12758.1.S1_at	7.82	7.06	7.43	7.57	7.36	7.39	7.44	0.38	7.44	0.11	1.00	0.001	9.8E-01
Mtr.43514.1.S1_s_at	9.07	9.83	9.49	10.57	10.96	10.68	9.46	0.38	10.74	0.20	1.13	0.182	6.9E-03
Mtr.11881.1.S1_at	10.26	9.50	9.82	9.96	10.05	10.20	9.86	0.38	10.07	0.12	1.02	0.030	4.2E-01
Mtr.49436.1.S1_at	9.75	9.25	10.00	10.00	9.85	9.73	9.67	0.38	9.86	0.13	1.02	0.029	4.5E-01
Mtr.19118.1.S1_at	3.33	3.56	4.08	4.01	3.34	3.27	3.66	0.38	3.54	0.41	0.97	-0.047	7.4E-01
Mtr.43226.1.S1_at	12.86	13.32	13.62	13.14	13.08	13.08	13.26	0.38	13.10	0.04	0.99	-0.018	5.0E-01
Mtr.3692.1.S1_at	3.06	2.35	2.93	2.91	2.82	3.47	2.78	0.38	3.07	0.35	1.10	0.141	3.9E-01
Mtr.24475.1.S1_at	8.48	7.93	8.67	8.45	8.53	8.38	8.36	0.38	8.45	0.07	1.01	0.016	7.1E-01
Mtr.22002.1.S1_at	7.71	7.59	8.31	8.06	7.84	7.83	7.87	0.38	7.91	0.13	1.00	0.007	8.8E-01
Mtr.15223.1.S1_s_at	3.09	2.50	2.37	2.37	2.37	2.36	2.65	0.38	2.36	0.00	0.89	-0.166	2.6E-01
Mtr.41626.1.S1_at	4.11	3.43	4.07	4.22	3.89	4.05	3.87	0.38	4.05	0.16	1.05	0.067	4.9E-01
Mtr.16232.1.S1_at	9.87	9.68	10.42	10.21	10.05	10.09	9.99	0.38	10.12	0.08	1.01	0.018	6.0E-01
Mtr.9310.1.S1_at	2.93	3.17	3.68	3.46	2.65	3.01	3.26	0.38	3.04	0.41	0.93	-0.099	5.4E-01
Mtr.11771.1.S1_at	10.76	11.39	11.45	10.71	10.86	10.81	11.20	0.38	10.79	0.07	0.96	-0.053	1.4E-01
Mtr.9268.1.S1_at	10.50	10.45	11.13	10.46	10.80	10.56	10.69	0.38	10.61	0.18	0.99	-0.012	7.4E-01
Mtr.24607.1.S1_at	2.64	3.29	2.61	2.63	2.57	2.63	2.85	0.38	2.61	0.03	0.92	-0.126	3.4E-01
Mtr.7766.1.S1_at	2.68	3.35	2.69	2.66	2.67	2.66	2.91	0.38	2.66	0.00	0.92	-0.126	3.3E-01
Mtr.382.1.S1_at	11.34	11.87	12.08	11.62	11.67	11.60	11.77	0.38	11.63	0.03	0.99	-0.017	5.8E-01
Mtr.25838.1.S1_at	12.09	11.91	11.36	11.71	11.99	11.78	11.79	0.38	11.83	0.15	1.00	0.005	8.7E-01
Mtr.36131.1.S1_s_at	10.70	9.99	10.57	10.21	10.05	10.16	10.42	0.38	10.14	0.08	0.97	-0.039	2.8E-01
Mtr.46420.1.S1_at	3.66	4.29	3.60	3.56	3.51	3.49	3.85	0.38	3.52	0.04	0.91	-0.128	2.1E-01
Mtr.40571.1.S1_at	10.90	10.31	11.02	10.87	10.77	10.82	10.75	0.38	10.82	0.05	1.01	0.010	7.6E-01
Mtr.9166.1.S1_at	2.60	3.23	2.55	2.65	2.54	2.54	2.79	0.38	2.58	0.06	0.92	-0.118	3.8E-01
Mtr.44706.1.S1_at	8.02	8.75	8.56	8.29	8.51	8.27	8.45	0.38	8.35	0.13	0.99	-0.016	7.2E-01
Mtr.12374.1.S1_at	11.91	11.91	12.57	12.05	11.97	11.85	12.13	0.38	11.96	0.10	0.99	-0.021	4.9E-01
Mtr.8801.1.S1_at	6.37	6.17	6.91	6.90	7.09	6.91	6.48	0.38	6.97	0.11	1.07	0.104	1.0E-01
Mtr.28338.1.S1_at	3.11	3.76	3.10	3.09	3.08	3.08	3.32	0.38	3.08	0.00	0.93	-0.109	3.4E-01
Mtr.15346.1.S1_at	3.91	3.96	4.60	4.20	3.84	3.94	4.16	0.38	3.99	0.18	0.96	-0.057	5.4E-01
Mtr.35806.1.S1_at	7.95	7.20	7.65	8.94	9.35	8.99	7.60	0.38	9.09	0.22	1.20	0.259	4.2E-03
Mtr.16250.1.S1_x_at	3.02	3.64	2.95	2.94	2.91	2.86	3.20	0.38	2.90	0.04	0.91	-0.142	2.5E-01
Mtr.38799.1.S1_s_at	8.93	8.81	8.22	8.52	8.34	8.65	8.65	0.38	8.50	0.16	0.98	-0.025	5.7E-01
Mtr.45724.1.S1_at	3.56	3.62	4.25	3.55	3.55	3.55	3.81	0.38	3.55	0.00	0.93	-0.102	3.0E-01
Mtr.43417.1.S1_at	11.17	10.41	10.71	10.90	10.55	10.79	10.76	0.38	10.75	0.18	1.00	-0.002	9.5E-01
Mtr.48829.1.S1_at	13.75	13.96	13.22	13.49	13.68	13.75	13.64	0.38	13.64	0.14	1.00	0.000	9.9E-01
Mtr.47633.1.S1_at	2.56	3.22	2.56	2.54	2.53	2.52	2.78	0.38	2.53	0.01	0.91	-0.135	3.2E-01
Mtr.43720.1.S1_at	11.08	10.34	10.87	10.95	11.06	10.92	10.77	0.38	10.98	0.08	1.02	0.028	4.0E-01
Mtr.11728.1.S1_at	10.24	9.48	9.93	9.99	10.22	9.80	9.88	0.38	10.00	0.21	1.01	0.017	6.7E-01
Mtr.43779.1.S1_at	11.62	10.90	11.47	11.36	11.18	11.29	11.33	0.38	11.28	0.09	1.00	-0.007	8.3E-01
Mtr.38570.1.S1_at	9.51	8.75	9.10	9.29	9.40	9.29	9.12	0.38	9.33	0.06	1.02	0.032	4.1E-01
Mtr.14808.1.S1_at	11.39	11.64	12.14	11.60	11.79	11.74	11.72	0.38	11.71	0.10	1.00	-0.002	9.6E-01
Mtr.46286.1.S1_at	11.08	10.50	10.36	11.12	11.04	11.36	10.65	0.38	11.17	0.17	1.05	0.069	9.6E-02
Mtr.15385.1.S1_at	7.86	7.57	8.32	8.04	8.46	8.09	7.92	0.38	8.20	0.23	1.04	0.050	3.3E-01
Mtr.15610.1.S1_at	2.71	3.35	2.68	2.67	2.60	2.61	2.91	0.38	2.63	0.04	0.90	-0.146	2.7E-01
Mtr.41687.1.S1_s_at	9.45	8.79	9.46	9.24	9.28	9.44	9.23	0.38	9.32	0.11	1.01	0.013	7.3E-01
Mtr.27397.1.S1_at	7.75	7.42	8.18	7.70	7.72	7.59	7.79	0.38	7.67	0.07	0.98	-0.022	6.2E-01
Mtr.34235.1.S1_at	2.45	3.10	2.44	2.43	2.42	2.43	2.66	0.38	2.43	0.01	0.91	-0.134	3.4E-01
Mtr.38044.1.S1_at	10.89	10.44	11.20	10.94	11.08	10.93	10.84	0.38	10.99	0.08	1.01	0.019	5.6E-01
Mtr.42664.1.S1_at	6.86	6.96	7.56	7.25	7.25	7.57	7.13	0.38	7.36	0.19	1.03	0.046	4.0E-01
Mtr.13813.1.S1_s_at	8.94	8.29	8.96	8.94	8.81	8.92	8.73	0.38	8.89	0.07	1.02	0.027	5.0E-01
Mtr.16783.1.S1_at	10.00	9.26	9.48	9.63	9.74	9.62	9.58	0.38	9.66	0.07	1.01	0.012	7.4E-01
Mtr.33724.1.S1_at	4.81	4.05	4.48	4.28	4.60	4.12	4.45	0.38	4.33	0.24	0.98	-0.036	6.9E-01
Mtr.35080.1.S1_at	11.09	11.43	10.67	11.53	11.71	11.39	11.06	0.38	11.54	0.16	1.04	0.061	1.1E-01
Mtr.12101.1.S1_at	9.12	8.51	9.20	9.11	9.11	9.13	8.94	0.38	9.12	0.01	1.02	0.027	4.8E-01
Mtr.21919.1.S1_at	2.63	3.26	2.58	2.58	2.57	2.57	2.83	0.38	2.57	0.01	0.91	-0.135	3.1E-01
Mtr.21809.1.S1_at	13.73	13.13	13.03	13.27	13.46	13.11	13.30	0.38	13.28	0.18	1.00	-0.002	9.4E-01
Mtr.47761.1.S1_at	9.07	9.37	9.83	8.52	9.30	8.51	9.42	0.38	8.78	0.45	0.93	-0.102	1.3E-01
Mtr.5470.1.S1_at	4.14	4.71	3.99	3.97	3.81	3.80	4.28	0.38	3.86	0.09	0.90	-0.149	1.4E-01
Mtr.37531.1.S1_at	12.98	12.36	13.05	12.82	12.97	12.93	12.80	0.38	12.91	0.08	1.01	0.012	6.5E-01
Mtr.44059.1.S1_at	9.46	9.81	10.22	9.92	10.08	9.71	9.83	0.38	9.90	0.19	1.01	0.010	7.9E-01
Mtr.34607.1.S1_at	9.24	8.68	9.41	9.46	9.41	9.31	9.11	0.38	9.39	0.07	1.03	0.044	2.8E-01
Mtr.35941.1.S1_at	8.31	8.07	8.81	8.49	8.54	8.35	8.40	0.38	8.46	0.10	1.01	0.011	7.9E-01
Mtr.41177.1.S1_at	11.08	11.83	11.36	11.04	11.23	11.08	11.42	0.38	11.12	0.10	0.97	-0.039	2.5E-01
Mtr.42565.1.S1_s_at	8.03	7.90	8.62	8.21	8.35	8.31	8.19	0.38	8.29	0.07	1.01	0.018	6.7E-01
Mtr.32578.1.S1_at	8.67	8.10	8.81	8.68	8.95	8.76	8.53	0.38	8.80	0.14	1.03	0.045	3.1E-01
Mtr.8842.1.S1_at	11.69	10.98	11.58	11.68	11.54	11.58	11.42	0.38	11.60	0.07	1.02	0.023	4.6E-01
Mtr.47554.1.S1_x_at	2.81	3.47	2.81	2.78	2.72	2.75	3.03	0.38	2.75	0.03	0.91	-0.140	2.7E-01
Mtr.32033.1.S1_x_at	3.40	4.02	3.33	3.26	3.20	3.19	3.58	0.38	3.22	0.04	0.90	-0.155	1.7E-01
Mtr.37999.1.S1_at	10.35	9.85	10.60	10.48	10.49	10.32	10.27	0.38	10.43	0.10	1.02	0.023	5.1E-01
Mtr.10943.1.S1_at	9.75	9.17	9.04	9.83	10.24	10.18	9.32	0.38	10.08	0.22	1.08	0.113	4.0E-02
Mtr.15288.1.S1_at	2.67	3.31	2.63	2.59	2.58	2.56	2.87	0.38	2.58	0.02	0.90	-0.154	2.6E-01
Mtr.7797.1.S1_at	5.85	5.12	5.30	5.16	5.41	5.67	5.42	0.38	5.41	0.26	1.00	-0.002	9.7E-01
Mtr.6845.1.S1_at	7.42	7.22	7.96	8.10	8.03	8.12	7.53	0.38	8.08	0.05	1.07	0.102	6.8E-02
Mtr.34846.1.S1_s_at	6.80	6.24	6.96	6.78	6.45	6.96	6.67	0.38	6.73	0.26	1.01	0.014	8.2E-01
Mtr.10834.1.S1_at	4.12	4.65	3.91	4.08	4.43	4.01	4.23	0.38	4.17	0.23	0.99	-0.020	8.3E-01
Mtr.49944.1.S1_x_at	5.66	4.98	5.61	5.59	5.59	5.56	5.42	0.38	5.58	0.02	1.03	0.043	4.9E-01
Mtr.13897.1.S1_at	8.57	8.53	9.21	8.77	8.81	8.77	8.77	0.38	8.78	0.02	1.00	0.003	9.5E-01
Mtr.52351.1.S1_x_at	5.20	4.44	4.85	5.52	5.19	5.53	4.83	0.38	5.41	0.19	1.12	0.165	7.7E-02
Mtr.10186.1.S1_at	9.93	9.56	10.32	9.83	9.98	9.58	9.93	0.38	9.80	0.20	0.99	-0.020	6.1E-01
Mtr.38877.1.S1_at	7.01	6.72	7.47	6.75	7.32	6.54	7.07	0.38	6.87	0.40	0.97	-0.041	5.7E-01
Mtr.12760.1.S1_at	4.89	4.54	5.30	5.47	5.36	5.36	4.91	0.38	5.40	0.06	1.10	0.136	9.5E-02
Mtr.8927.1.S1_s_at	9.61	9.10	9.84	9.45	9.72	9.81	9.52	0.38	9.66	0.18	1.01	0.021	6.0E-01
Mtr.24251.1.S1_s_at	10.62	10.39	11.14	10.93	10.86	10.71	10.72	0.38	10.83	0.11	1.01	0.015	6.4E-01
Mtr.44907.1.S1_s_at	9.79	9.26	10.00	9.75	9.74	9.74	9.68	0.38	9.74	0.01	1.01	0.009	7.9E-01
Mtr.38804.1.S1_at	9.54	9.00	8.81	9.14	9.37	9.38	9.11	0.38	9.30	0.13	1.02	0.029	4.7E-01
Mtr.8398.1.S1_at	4.17	3.51	3.52	3.50	5.07	3.73	3.73	0.38	4.10	0.85	1.10	0.136	5.3E-01
Mtr.20600.1.S1_at	2.90	3.58	2.95	3.14	2.89	2.84	3.15	0.38	2.96	0.16	0.94	-0.090	4.7E-01
Mtr.7892.1.S1_at	2.94	3.59	2.92	2.91	2.91	2.92	3.15	0.38	2.92	0.00	0.93	-0.111	3.5E-01
Mtr.2249.1.S1_at	8.35	7.59	7.96	8.25	8.51	8.56	7.96	0.38	8.44	0.17	1.06	0.084	1.2E-01
Mtr.11147.1.S1_at	10.06	10.75	10.68	10.18	10.50	10.03	10.50	0.38	10.23	0.24	0.97	-0.037	3.7E-01
Mtr.32286.1.S1_at	8.66	8.56	9.26	8.89	8.97	8.96	8.83	0.38	8.94	0.04	1.01	0.018	6.5E-01
Mtr.31892.1.S1_at	8.63	8.41	9.15	8.75	8.85	8.58	8.73	0.38	8.73	0.14	1.00	-0.001	9.8E-01
Mtr.12823.1.S1_at	10.41	10.30	11.01	10.70	10.73	10.58	10.57	0.38	10.67	0.08	1.01	0.014	6.8E-01
Mtr.3443.1.S1_at	8.50	7.75	8.24	8.18	8.22	8.26	8.16	0.38	8.22	0.04	1.01	0.010	8.1E-01
Mtr.10859.1.S1_at	11.71	12.15	12.47	12.16	12.11	12.09	12.11	0.38	12.12	0.04	1.00	0.001	9.8E-01
Mtr.3573.1.S1_at	4.15	4.91	4.51	4.19	4.34	4.26	4.52	0.38	4.26	0.07	0.94	-0.085	3.1E-01
Mtr.19932.1.S1_s_at	2.97	3.50	3.71	2.88	4.57	4.61	3.39	0.38	4.02	0.99	1.18	0.243	3.7E-01
Mtr.39206.1.S1_at	8.68	8.90	8.16	8.71	8.58	8.85	8.58	0.38	8.72	0.13	1.02	0.023	5.9E-01
Mtr.30765.1.S1_at	9.72	9.09	9.03	9.50	8.75	9.45	9.28	0.38	9.23	0.42	0.99	-0.007	8.9E-01
Mtr.1612.1.S1_s_at	4.98	5.63	4.97	4.94	4.97	4.96	5.20	0.38	4.96	0.02	0.95	-0.068	3.3E-01
Mtr.42122.1.S1_at	10.54	10.23	10.98	10.67	10.76	10.63	10.58	0.38	10.69	0.07	1.01	0.014	6.6E-01
Mtr.11549.1.S1_at	8.93	8.17	8.52	8.77	8.57	8.71	8.54	0.38	8.68	0.10	1.02	0.023	5.7E-01
Mtr.38045.1.S1_at	9.92	10.38	10.67	11.40	12.12	11.72	10.33	0.38	11.75	0.36	1.14	0.186	9.2E-03
Mtr.6822.1.S1_at	6.17	5.72	6.47	6.81	6.55	6.42	6.12	0.38	6.59	0.20	1.08	0.108	1.3E-01
Mtr.39692.1.S1_at	8.54	7.95	8.65	8.48	8.38	8.69	8.38	0.38	8.52	0.16	1.02	0.023	6.0E-01
Mtr.32176.1.S1_at	8.10	8.47	8.86	8.50	8.62	8.32	8.47	0.38	8.48	0.15	1.00	0.001	9.8E-01
Mtr.24849.1.S1_at	3.15	3.80	3.13	3.13	3.13	3.13	3.36	0.38	3.13	0.00	0.93	-0.102	3.5E-01
Mtr.38672.1.S1_at	2.59	2.64	3.27	2.58	2.58	2.57	2.84	0.38	2.58	0.00	0.91	-0.139	3.0E-01
Mtr.2208.1.S1_at	3.20	3.82	3.13	3.10	3.09	3.02	3.38	0.38	3.07	0.05	0.91	-0.139	2.3E-01
Mtr.27673.1.S1_at	10.99	10.59	11.35	10.95	10.89	10.56	10.98	0.38	10.80	0.21	0.98	-0.023	5.2E-01
Mtr.28058.1.S1_at	3.00	2.31	2.93	2.45	3.24	3.25	2.75	0.38	2.98	0.46	1.09	0.119	5.3E-01
Mtr.50350.1.S1_at	9.27	8.51	8.98	9.24	9.26	9.19	8.92	0.38	9.23	0.03	1.03	0.049	2.3E-01

Mtr.44927.1.S1_at	8.40	7.70	8.30	8.28	8.51	7.82	8.14	0.38	8.20	0.35	1.01	0.012	8.3E-01
Mtr.36216.1.S1_at	9.57	9.17	9.93	9.67	9.86	9.54	9.55	0.38	9.69	0.16	1.01	0.020	6.0E-01
Mtr.1672.1.S1_at	2.39	2.44	3.07	2.32	2.36	2.36	2.63	0.38	2.35	0.02	0.89	-0.165	2.6E-01
Mtr.35494.1.S1_at	8.72	8.74	9.38	9.04	9.10	9.09	8.95	0.38	9.07	0.03	1.01	0.020	5.9E-01
Mtr.43254.1.S1_at	13.16	12.45	12.56	12.80	12.97	12.95	12.72	0.38	12.91	0.09	1.01	0.021	4.6E-01
Mtr.7719.1.S1_at	13.92	13.22	13.32	13.72	13.66	13.79	13.49	0.38	13.72	0.06	1.02	0.025	3.5E-01
Mtr.6623.1.S1_at	2.33	2.98	2.32	2.31	2.31	2.31	2.54	0.38	2.31	0.00	0.91	-0.137	3.5E-01
Mtr.44521.1.S1_at	8.40	8.58	9.13	8.83	9.16	8.47	8.71	0.38	8.82	0.35	1.01	0.019	7.2E-01
Mtr.47125.1.S1_at	2.56	3.20	2.53	2.51	2.50	2.48	2.76	0.38	2.50	0.02	0.90	-0.146	2.9E-01
Mtr.21595.1.S1_at	9.72	9.00	9.57	9.37	9.52	9.39	9.43	0.38	9.43	0.08	1.00	-0.001	9.8E-01
Mtr.12754.1.S1_at	9.26	8.72	9.45	9.37	9.28	9.29	9.14	0.38	9.31	0.05	1.02	0.027	4.8E-01
Mtr.37992.1.S1_s_at	10.51	10.95	11.26	10.72	10.90	10.58	10.91	0.38	10.73	0.16	0.98	-0.023	5.1E-01
Mtr.27071.1.S1_at	3.80	4.43	3.75	3.79	3.99	3.77	3.99	0.38	3.85	0.12	0.96	-0.052	5.7E-01
Mtr.30601.1.S1_at	3.46	2.80	3.45	3.43	3.43	3.44	3.24	0.38	3.44	0.01	1.06	0.086	4.1E-01
Mtr.28710.1.S1_at	2.82	2.77	3.45	3.56	3.32	2.80	3.01	0.38	3.23	0.39	1.07	0.098	5.4E-01
Mtr.32469.1.S1_at	5.53	4.77	5.16	4.95	5.10	5.54	5.15	0.38	5.20	0.30	1.01	0.013	8.8E-01
Mtr.33558.1.S1_at	8.58	8.18	8.93	8.67	8.47	8.42	8.57	0.38	8.52	0.13	0.99	-0.008	8.5E-01
Mtr.10868.1.S1_at	12.81	12.88	12.19	12.18	11.95	12.16	12.63	0.38	12.10	0.13	0.96	-0.062	8.4E-02
Mtr.3889.1.S1_at	9.57	8.91	9.57	9.49	10.22	9.74	9.35	0.38	9.82	0.37	1.05	0.070	2.0E-01
Mtr.43178.1.S1_at	8.54	8.05	8.79	8.67	8.80	8.69	8.46	0.38	8.72	0.07	1.03	0.044	3.0E-01
Mtr.32515.1.S1_s_at	5.82	6.47	5.81	5.84	5.79	5.72	6.03	0.38	5.78	0.06	0.96	-0.061	3.2E-01
Mtr.42211.1.S1_at	10.94	10.98	11.61	11.18	11.01	11.09	11.18	0.38	11.09	0.09	0.99	-0.011	7.3E-01
Mtr.46063.1.S1_at	2.49	3.14	2.48	2.47	2.47	2.46	2.70	0.38	2.47	0.01	0.91	-0.131	3.4E-01
Mtr.18824.1.S1_at	6.16	5.70	6.45	6.18	6.21	6.15	6.10	0.38	6.18	0.03	1.01	0.018	7.4E-01
Mtr.41319.1.S1_s_at	9.30	8.55	8.86	9.25	9.18	9.02	8.90	0.38	9.15	0.12	1.03	0.040	3.4E-01
Mtr.1106.1.S1_at	9.62	9.27	10.03	9.84	9.90	9.66	9.64	0.38	9.80	0.12	1.02	0.024	5.2E-01
Mtr.42440.1.S1_a_at	10.63	9.88	10.17	10.39	10.41	10.44	10.22	0.38	10.41	0.02	1.02	0.026	4.4E-01
Mtr.10364.1.S1_at	3.02	3.65	2.97	2.94	2.93	2.88	3.21	0.38	2.91	0.03	0.91	-0.139	2.5E-01
Mtr.39956.1.S1_at	9.25	9.94	9.87	9.52	9.70	9.64	9.69	0.38	9.62	0.09	0.99	-0.010	7.8E-01
Mtr.23020.1.S1_at	9.34	8.69	9.36	9.30	9.11	9.14	9.13	0.38	9.18	0.10	1.01	0.009	8.2E-01
Mtr.3247.1.S1_at	10.25	9.49	9.86	9.92	10.11	9.92	9.87	0.38	9.98	0.11	1.01	0.017	6.4E-01
Mtr.10157.1.S1_at	9.10	8.45	9.11	9.11	8.81	8.97	8.89	0.38	8.96	0.15	1.01	0.012	7.6E-01
Mtr.46975.1.S1_at	6.84	7.05	7.58	6.66	6.82	6.74	7.16	0.38	6.74	0.08	0.94	-0.086	1.4E-01
Mtr.9882.1.S1_at	9.15	9.20	9.83	9.57	9.54	9.59	9.39	0.38	9.57	0.03	1.02	0.027	4.7E-01
Mtr.13431.1.S1_at	8.81	9.29	9.56	9.28	9.11	9.33	9.22	0.38	9.24	0.12	1.00	0.002	9.5E-01
Mtr.22811.1.S1_at	2.44	2.55	3.14	2.42	2.41	2.42	2.71	0.38	2.42	0.01	0.89	-0.167	2.5E-01
Mtr.17639.1.S1_at	8.33	7.87	8.62	8.35	8.47	8.42	8.28	0.38	8.42	0.06	1.02	0.024	5.7E-01
Mtr.49238.1.S1_x_at	5.83	6.58	6.30	5.70	6.47	5.56	6.24	0.38	5.91	0.49	0.95	-0.078	4.1E-01
Mtr.43609.1.S1_at	8.38	8.73	9.14	8.07	8.25	8.40	8.75	0.38	8.24	0.16	0.94	-0.087	9.8E-02
Mtr.17619.1.S1_at	2.46	3.10	2.42	2.42	2.42	2.41	2.66	0.38	2.41	0.01	0.91	-0.141	3.2E-01
Mtr.22352.1.S1_at	2.59	3.25	2.59	2.57	2.52	2.54	2.81	0.38	2.54	0.02	0.90	-0.144	2.9E-01
Mtr.39896.1.S1_at	9.90	9.26	9.93	9.94	9.48	9.75	9.70	0.38	9.72	0.23	1.00	0.004	9.2E-01
Mtr.41772.1.S1_at	13.12	12.38	12.59	12.93	13.07	12.93	12.70	0.38	12.98	0.08	1.02	0.031	2.8E-01
Mtr.34612.1.S1_at	8.66	8.26	9.02	8.76	8.71	8.67	8.65	0.38	8.71	0.05	1.01	0.011	7.8E-01
Mtr.51416.1.S1_at	8.36	8.90	9.10	8.68	8.69	8.54	8.79	0.38	8.64	0.09	0.98	-0.025	5.4E-01
Mtr.20243.1.S1_s_at	7.70	7.08	7.01	7.50	7.47	7.55	7.26	0.38	7.51	0.04	1.03	0.047	3.3E-01
Mtr.26288.1.S1_s_at	9.34	8.84	9.59	9.37	9.13	9.23	9.26	0.38	9.24	0.12	1.00	-0.002	9.5E-01
Mtr.28844.1.S1_at	7.66	6.90	7.27	7.60	7.49	7.54	7.28	0.38	7.54	0.06	1.04	0.052	2.9E-01
Mtr.22813.1.S1_at	2.13	2.78	2.12	2.11	2.11	2.11	2.34	0.38	2.11	0.00	0.90	-0.149	3.5E-01
Mtr.8664.1.S1_at	9.94	9.25	9.87	9.48	9.92	9.62	9.69	0.38	9.67	0.22	1.00	-0.002	9.6E-01
Mtr.43271.1.S1_at	7.26	6.59	6.62	6.48	6.74	6.72	6.83	0.38	6.65	0.14	0.97	-0.038	4.9E-01
Mtr.17735.1.S1_at	5.95	6.63	6.01	5.93	5.96	5.92	6.20	0.38	5.93	0.02	0.96	-0.063	3.0E-01
Mtr.12045.1.S1_at	10.73	10.13	10.83	10.56	10.58	10.60	10.57	0.38	10.58	0.02	1.00	0.002	9.4E-01
Mtr.8215.1.S1_s_at	9.70	9.04	9.05	9.21	9.06	9.11	9.26	0.38	9.13	0.08	0.99	-0.021	5.8E-01
Mtr.49521.1.S1_x_at	2.93	3.59	2.95	2.91	2.90	2.89	3.15	0.38	2.90	0.01	0.92	-0.123	3.0E-01
Mtr.12934.1.S1_at	11.39	11.90	12.13	12.09	12.16	11.89	11.81	0.38	12.05	0.14	1.02	0.029	3.6E-01
Mtr.12726.1.S1_at	10.07	9.39	10.01	9.96	10.09	9.96	9.83	0.38	10.01	0.08	1.02	0.026	4.6E-01
Mtr.47753.1.S1_at	2.96	3.57	2.89	2.88	2.86	2.86	3.14	0.38	2.87	0.01	0.91	-0.132	2.8E-01
Mtr.41647.1.S1_at	7.67	7.01	7.65	7.76	7.75	7.79	7.44	0.38	7.77	0.02	1.04	0.062	2.1E-01
Mtr.16634.1.S1_s_at	3.21	2.57	2.54	2.53	2.43	2.53	2.77	0.38	2.50	0.06	0.90	-0.150	2.8E-01
Mtr.51495.1.S1_x_at	3.04	3.67	2.99	2.97	2.94	3.00	3.23	0.38	2.97	0.03	0.92	-0.122	3.0E-01
Mtr.34706.1.S1_s_at	9.79	9.24	9.96	9.74	9.64	9.70	9.67	0.38	9.69	0.05	1.00	0.004	9.1E-01
Mtr.35256.1.S1_at	7.48	6.93	7.65	7.35	7.82	7.42	7.35	0.38	7.53	0.25	1.02	0.035	5.3E-01
Mtr.41154.1.S1_s_at	9.90	9.20	9.79	9.76	9.72	9.74	9.63	0.38	9.74	0.02	1.01	0.016	6.5E-01
Mtr.5871.1.S1_at	5.75	5.00	5.41	5.48	5.42	5.41	5.39	0.38	5.44	0.04	1.01	0.014	8.2E-01
Mtr.9965.1.S1_at	8.07	7.34	7.54	8.28	7.84	8.24	7.65	0.38	8.12	0.25	1.06	0.085	1.5E-01
Mtr.33697.1.S1_s_at	8.77	9.05	9.52	9.24	9.02	9.19	9.12	0.38	9.15	0.11	1.00	0.005	8.9E-01
Mtr.8851.1.S1_at	12.69	12.60	12.00	12.49	12.88	12.66	12.43	0.38	12.68	0.20	1.02	0.029	3.7E-01
Mtr.26913.1.S1_at	6.00	5.32	5.94	5.87	5.97	5.94	5.75	0.38	5.93	0.05	1.03	0.043	4.7E-01
Mtr.14749.1.S1_at	2.07	2.72	2.06	2.06	2.06	2.08	2.29	0.38	2.06	0.01	0.90	-0.147	3.7E-01
Mtr.34680.1.S1_at	10.37	9.66	9.79	10.08	9.94	9.99	9.94	0.38	10.00	0.07	1.01	0.009	7.9E-01
Mtr.27490.1.S1_s_at	11.62	11.23	11.99	11.73	11.68	11.61	11.62	0.38	11.67	0.06	1.00	0.007	8.1E-01
Mtr.10760.1.S1_at	11.19	10.44	10.89	11.10	11.28	11.39	10.84	0.38	11.26	0.15	1.04	0.054	1.5E-01
Mtr.51661.1.S1_at	2.21	2.80	2.09	2.14	2.09	2.44	2.37	0.38	2.22	0.19	0.94	-0.092	5.8E-01
Mtr.35346.1.S1_at	7.88	7.25	7.92	7.88	7.88	7.88	7.68	0.38	7.88	0.00	1.03	0.036	4.2E-01
Mtr.22138.1.S1_at	2.35	2.99	2.33	2.33	2.32	2.31	2.56	0.38	2.32	0.01	0.91	-0.141	3.4E-01
Mtr.32246.1.S1_at	3.36	4.01	3.35	3.41	3.26	3.45	3.57	0.38	3.37	0.10	0.94	-0.084	4.2E-01
Mtr.13117.1.S1_at	8.98	8.48	9.22	9.54	9.72	9.50	8.89	0.38	9.58	0.12	1.08	0.108	3.9E-02
Mtr.42003.1.S1_at	8.84	8.45	9.21	9.06	8.69	8.73	8.84	0.38	8.83	0.20	1.00	-0.001	9.8E-01
Mtr.4717.1.S1_at	7.04	6.96	7.65	6.69	7.58	7.29	7.22	0.38	7.18	0.45	1.00	-0.007	9.3E-01
Mtr.3451.1.S1_s_at	3.36	3.74	2.99	3.00	2.97	2.95	3.36	0.38	2.98	0.02	0.88	-0.178	1.5E-01
Mtr.39520.1.S1_at	11.36	12.12	11.76	11.30	11.51	11.58	11.75	0.38	11.46	0.15	0.98	-0.035	2.9E-01
Mtr.40258.1.S1_at	11.18	10.58	10.48	11.00	10.80	11.16	10.74	0.38	10.99	0.18	1.02	0.032	3.7E-01
Mtr.31919.1.S1_at	8.35	7.64	8.21	8.20	8.26	8.07	8.06	0.38	8.18	0.09	1.01	0.020	6.4E-01
Mtr.5460.1.S1_at	10.32	11.07	10.74	10.40	10.31	10.41	10.71	0.38	10.37	0.05	0.97	-0.046	2.0E-01
Mtr.45044.1.S1_at	6.97	7.71	7.21	7.17	7.32	7.21	7.30	0.38	7.23	0.08	0.99	-0.013	7.9E-01
Mtr.23146.1.S1_at	12.18	12.92	12.66	12.60	12.50	12.54	12.59	0.38	12.54	0.05	1.00	-0.005	8.5E-01
Mtr.3374.1.S1_at	3.96	3.35	4.03	3.36	4.01	3.42	3.78	0.38	3.60	0.36	0.95	-0.072	5.7E-01
Mtr.31298.1.S1_at	11.26	10.51	10.89	10.93	11.06	10.87	10.89	0.38	10.95	0.10	1.01	0.009	7.8E-01
Mtr.37554.1.S1_at	13.27	13.65	14.02	13.90	13.86	13.80	13.64	0.38	13.85	0.05	1.02	0.022	3.9E-01
Mtr.11350.1.S1_at	9.94	9.20	9.72	9.70	10.00	9.63	9.62	0.38	9.78	0.20	1.02	0.024	5.5E-01
Mtr.6147.1.S1_at	8.76	8.61	9.32	8.91	8.85	8.86	8.89	0.38	8.87	0.03	1.00	-0.004	9.3E-01
Mtr.38724.1.S1_at	7.55	7.00	7.72	7.47	7.72	7.45	7.42	0.38	7.55	0.15	1.02	0.025	6.2E-01
Mtr.41899.1.S1_at	9.32	8.57	8.99	9.87	9.81	9.71	8.96	0.38	9.80	0.08	1.09	0.129	1.9E-02
Mtr.31208.1.S1_at	4.23	3.59	4.25	4.00	3.37	4.41	4.02	0.38	3.93	0.53	0.98	-0.035	8.1E-01
Mtr.38484.1.S1_at	9.85	9.10	9.53	9.71	9.78	9.74	9.49	0.38	9.74	0.03	1.03	0.037	3.2E-01
Mtr.34876.1.S1_at	2.54	3.18	2.51	2.51	2.50	2.47	2.74	0.38	2.49	0.02	0.91	-0.137	3.2E-01
Mtr.22269.1.S1_at	7.35	7.78	7.03	6.88	6.76	7.00	7.39	0.38	6.88	0.12	0.93	-0.102	9.1E-02
Mtr.50752.1.S1_at	10.59	9.91	10.53	10.05	10.14	10.05	10.34	0.38	10.08	0.05	0.97	-0.038	2.9E-01
Mtr.44914.1.S1_at	7.90	7.22	7.84	7.97	7.94	8.23	7.65	0.38	8.04	0.16	1.05	0.072	1.7E-01
Mtr.34566.1.S1_at	9.14	8.77	9.52	9.39	9.50	9.09	9.14	0.38	9.33	0.22	1.02	0.029	5.0E-01
Mtr.10702.1.S1_at	10.40	9.69	9.84	10.12	10.21	10.19	9.97	0.38	10.17	0.05	1.02	0.028	4.2E-01
Mtr.966.1.S1_at	2.98	2.41	2.27	3.19	2.28	2.54	2.55	0.38	2.67	0.47	1.05	0.065	7.5E-01
Mtr.42147.1.S1_at	7.62	6.87	7.21	7.92	8.29	8.30	7.23	0.38	8.17	0.22	1.13	0.175	2.0E-02
Mtr.43590.1.S1_at	10.01	9.33	9.94	9.95	9.86	10.03	9.76	0.38	9.95	0.08	1.02	0.027	4.5E-01
Mtr.46005.1.S1_at	2.29	2.93	2.27	2.26	2.26	2.26	2.50	0.38	2.26	0.00	0.91	-0.144	3.4E-01
Mtr.39627.1.S1_at	11.22	11.73	11.95	11.44	11.57	11.45	11.63	0.38	11.48	0.07	0.99	-0.019	5.4E-01
Mtr.38993.1.S1_at	10.78	10.19	10.09	10.15	10.28	10.07	10.35	0.38	10.17	0.10	0.98	-0.026	4.5E-01
Mtr.15579.1.S1_x_at	2.96	3.57	2.88	2.87	2.85	2.81	3.14	0.38	2.84	0.03	0.91	-0.142	2.5E-01
Mtr.33360.1.S1_at	10.35	11.07	10.89	11.11	11.06	11.19	10.77	0.38	11.12	0.07	1.03	0.046	1.9E-01
Mtr.27077.1.S1_at	5.07	5.20	5.78	5.27	5.17	5.12	5.35	0.38	5.19	0.08	0.97	-0.045	5.0E-01
Mtr.37855.1.S1_at	8.78	8.20	8.08	8.92	9.05	9.33	8.35	0.38	9.10	0.21	1.09	0.124	3.9E-02
Mtr.15773.1.S1_at	9.61	8.91	9.50	9.41	9.53	9.47	9.34	0.38	9.47	0.06	1.01	0.020	5.8E-01
Mtr.40709.1.S1_at	11.52	11.10	10.77	10.95	11.17	11.04	11.13	0.38	11.05	0.11	0.99	-0.010	7.4E-01
Mtr.19438.1.S1_x_at	3.04	3.66	2.99	3.14	2.89	2.96	3.23	0.38	2.99	0.13	0.93	-0.109	3.6E-01
Mtr.49624.1.S1_at	6.36	6.89	6.16	6.05	6.16	5.49	6.47	0.38	5.90	0.36	0.91	-0.134	1.3E-01
Mtr.43203.1.S1_at	10.92	10.95	11.59	11.57	11.72	11.76	11.15	0.38	11.68	0.10	1.05	0.067	7.9E-02
Mtr.31744.1.S1_at	9.15	8.43	8.61	8.23	8.39	8.29	8.73	0.38	8.30	0.08	0.95	-0.073	1.3E-01
Mtr.34578.1.S1_at	2.55	3.19	2.54	2.54	2.53	2.51	2.76	0.38	2.52	0.01	0.91	-0.129	3.4E-01
Mtr.11139.1.S1_at	9.91	9.18	9.38	9.57	9.70	9.63	9.49	0.38	9.64	0.06	1.02	0.022	5.4E-01
Mtr.13719.1.S1_at	9.15	8.94	9.67	9.43	9.31	9.26	9.25	0.38	9.33	0.09	1.01	0.012	7.4E-01
Mtr.43801.1.S1_at	9.41	8.93	9.67	9.46	9.43	9.26	9.34	0.38	9.38	0.11	1.00	0.007	8.5E-01
Mtr.29660.1.S1_at	4.33	4.08	4.82	3.80	4.31	4.29	4.41	0.38	4.13	0.29	0.94	-0.093	3.7E-01
Mtr.27870.1.S1_at	2.38	2.37	3.03	2.30	2.30	2.30	2.59	0.38	2.30	0.00	0.89	-0.173	2.5E-01
Mtr.8587.1.S1_at	2.88	3.51	3.54	3.53	4.04	3.82	3.31	0.38	3.80	0.25	1.15	0.197	1.4E-01
Mtr.34538.1.S1_at	4.73	4.82	5.42	4.76	4.71	4.75	4.99	0.38	4.74	0.02	0.95	-0.074	3.2E-01
Mtr.9464.1.S1_at	7.60	7.37	8.10	8.02	7.94	8.24	7.69	0.38	8.07	0.15	1.05	0.069	1.8E-01
Mtr.35154.1.S1_at	5.34	4.82	4.60	5.42	4.78	5.31	4.92	0.38	5.17	0.34	1.05	0.072	4.4E-01
Mtr.27757.1.S1_at	4.81	4.08	4.29	4.36	4.40	4.31	4.40	0.38	4.36	0.05	0.99	-0.014	8.6E-01
Mtr.34887.1.S1_at	3.94	3.20	3.64	3.77	3.85	3.77	3.59	0.38	3.80	0.05	1.06	0.080	4.0E-01
Mtr.2467.1.S1_at	5.66	6.28	5.60	6.60	5.79	6.57	5.84	0.38	6.32	0.46	1.08	0.113	2.4E-01

Mtr.3174.1.S1_x_at	5.49	6.05	5.34	5.50	5.99	5.20	5.63	0.38	5.56	0.40	0.99	-0.017	8.5E-01
Mtr.680.1.S1_at	3.40	2.65	2.93	2.75	3.57	2.60	2.99	0.38	2.97	0.52	0.99	-0.008	9.7E-01
Mtr.38213.1.S1_at	9.28	8.60	8.66	9.04	9.06	9.16	8.85	0.38	9.09	0.06	1.03	0.039	3.3E-01
Mtr.13061.1.S1_at	9.83	9.32	10.05	9.77	9.71	9.80	9.73	0.38	9.76	0.04	1.00	0.005	8.9E-01
Mtr.1014.1.S1_at	7.25	7.59	8.00	7.45	7.62	7.53	7.61	0.38	7.53	0.08	0.99	-0.015	7.4E-01
Mtr.36336.1.S1_at	2.07	2.72	2.07	2.06	2.06	2.06	2.28	0.38	2.06	0.00	0.90	-0.149	3.6E-01
Mtr.48645.1.S1_at	6.13	6.79	6.78	6.41	7.09	6.33	6.57	0.38	6.61	0.42	1.01	0.009	9.0E-01
Mtr.13178.1.S1_at	8.19	7.58	8.26	8.18	8.17	8.11	8.01	0.38	8.15	0.04	1.02	0.026	5.4E-01
Mtr.27994.1.S1_at	8.95	8.24	8.38	9.02	9.00	8.75	8.52	0.38	8.93	0.15	1.05	0.067	1.6E-01
Mtr.21852.1.S1_at	9.63	8.94	9.53	10.00	9.76	9.85	9.37	0.38	9.87	0.12	1.05	0.076	9.2E-02
Mtr.47935.1.S1_at	9.08	9.69	9.77	9.12	9.34	8.63	9.52	0.38	9.03	0.36	0.95	-0.075	1.8E-01
Mtr.5917.1.S1_at	9.01	8.31	8.43	8.83	8.70	9.02	8.59	0.38	8.85	0.16	1.03	0.043	3.3E-01
Mtr.3768.1.S1_s_at	9.72	8.98	9.21	9.24	9.28	9.48	9.30	0.38	9.33	0.12	1.00	0.005	9.0E-01
Mtr.13081.1.S1_at	7.53	6.83	6.96	7.61	7.36	7.66	7.11	0.38	7.55	0.16	1.06	0.086	1.4E-01
Mtr.34940.1.S1_at	4.00	3.33	3.38	3.36	3.80	3.51	3.57	0.38	3.56	0.22	1.00	-0.005	9.6E-01
Mtr.35032.1.S1_at	7.36	6.62	7.10	7.46	7.08	7.26	7.03	0.38	7.26	0.19	1.03	0.048	3.9E-01
Mtr.14805.1.S1_at	7.45	7.51	8.13	7.53	7.44	7.82	7.70	0.38	7.60	0.20	0.99	-0.018	7.1E-01
Mtr.24511.1.S1_at	3.57	3.12	2.82	2.77	3.57	3.05	3.17	0.38	3.13	0.40	0.99	-0.019	9.0E-01
Mtr.48399.1.S1_at	4.92	4.83	5.52	5.15	5.35	5.12	5.09	0.38	5.20	0.12	1.02	0.032	6.5E-01
Mtr.44619.1.S1_at	8.16	8.29	8.87	8.62	8.95	8.53	8.44	0.38	8.70	0.22	1.03	0.044	3.6E-01
Mtr.5777.1.S1_at	9.02	8.42	9.11	9.10	9.20	9.11	8.85	0.38	9.14	0.05	1.03	0.046	2.6E-01
Mtr.40472.1.S1_at	12.16	12.62	12.90	12.54	12.67	12.59	12.56	0.38	12.60	0.06	1.00	0.004	8.7E-01
Mtr.47356.1.S1_at	2.90	3.53	2.86	2.83	2.85	2.85	3.10	0.38	2.84	0.01	0.92	-0.124	3.0E-01
Mtr.39495.1.S1_at	9.03	8.42	9.10	9.17	9.12	9.21	8.85	0.38	9.17	0.05	1.04	0.051	2.2E-01
Mtr.11253.1.S1_at	7.82	7.49	8.24	8.20	8.58	8.34	7.85	0.38	8.37	0.19	1.07	0.093	9.9E-02
Mtr.37298.1.S1_at	13.77	13.40	13.02	13.06	13.08	13.18	13.39	0.38	13.10	0.06	0.98	-0.032	2.6E-01
Mtr.40580.1.S1_at	11.09	11.17	11.77	11.24	11.16	11.09	11.34	0.38	11.16	0.08	0.98	-0.023	4.6E-01
Mtr.11006.1.S1_at	9.12	8.81	9.56	9.22	9.26	9.37	9.16	0.38	9.29	0.08	1.01	0.019	6.1E-01
Mtr.27472.1.S1_s_at	9.12	9.67	9.84	9.57	9.51	9.49	9.54	0.37	9.52	0.04	1.00	-0.003	9.4E-01
Mtr.32701.1.S1_at	11.08	10.56	11.29	10.67	10.11	10.72	10.98	0.37	10.50	0.34	0.96	-0.065	1.7E-01
Mtr.46258.1.S1_at	10.58	9.88	10.47	10.45	10.46	10.58	10.31	0.37	10.49	0.07	1.02	0.025	4.6E-01
Mtr.11481.1.S1_at	9.21	8.50	9.08	8.89	8.76	8.89	8.93	0.37	8.85	0.08	0.99	-0.014	7.2E-01
Mtr.17100.1.S1_s_at	9.48	8.75	9.26	9.36	9.29	9.13	9.16	0.37	9.26	0.12	1.01	0.016	6.8E-01
Mtr.8537.1.S1_s_at	11.48	10.73	11.10	11.36	11.30	11.35	11.11	0.37	11.34	0.03	1.02	0.030	3.5E-01
Mtr.24434.1.S1_at	8.20	8.61	8.95	8.87	8.87	8.74	8.59	0.37	8.83	0.08	1.03	0.040	3.4E-01
Mtr.11687.1.S1_at	7.96	7.54	8.29	8.26	8.18	8.10	7.93	0.37	8.18	0.08	1.03	0.045	3.2E-01
Mtr.41789.1.S1_at	8.79	8.20	8.90	9.11	8.75	8.98	8.63	0.37	8.95	0.18	1.04	0.052	2.6E-01
Mtr.32545.1.S1_at	4.31	3.64	4.26	4.12	4.52	4.46	4.07	0.37	4.37	0.22	1.07	0.103	2.9E-01
Mtr.40221.1.S1_at	13.94	13.19	13.63	13.68	13.61	13.55	13.59	0.37	13.61	0.06	1.00	0.003	9.0E-01
Mtr.44753.1.S1_at	4.76	4.13	4.80	4.58	4.65	4.41	4.56	0.37	4.55	0.12	1.00	-0.005	9.5E-01
Mtr.10636.1.S1_at	13.40	12.70	13.27	13.05	13.29	13.25	13.12	0.37	13.20	0.13	1.01	0.008	7.7E-01
Mtr.11236.1.S1_at	7.70	7.03	7.07	6.70	7.18	7.45	7.27	0.37	7.11	0.38	0.98	-0.032	6.3E-01
Mtr.5559.1.S1_at	3.72	3.10	3.05	3.11	3.01	3.09	3.29	0.37	3.07	0.05	0.93	-0.098	3.8E-01
Mtr.45584.1.S1_at	7.42	6.69	6.91	7.11	7.19	7.37	7.01	0.37	7.22	0.13	1.03	0.044	4.0E-01
Mtr.45976.1.S1_at	11.92	11.28	11.27	11.69	11.87	11.74	11.49	0.37	11.77	0.09	1.02	0.034	2.8E-01
Mtr.3384.1.S1_at	3.05	2.31	2.79	2.87	2.71	2.91	2.72	0.37	2.83	0.11	1.04	0.056	6.6E-01
Mtr.46463.1.S1_at	2.46	3.10	2.45	2.44	2.44	2.44	2.67	0.37	2.44	0.00	0.91	-0.128	3.5E-01
Mtr.35530.1.S1_at	8.60	8.23	8.98	8.22	8.52	8.20	8.61	0.37	8.31	0.18	0.97	-0.051	2.8E-01
Mtr.30716.1.S1_at	3.86	4.45	3.76	4.24	3.86	4.08	4.02	0.37	4.06	0.19	1.01	0.015	8.7E-01
Mtr.30926.1.S1_at	3.41	4.05	3.39	3.19	3.45	3.63	3.62	0.37	3.42	0.22	0.95	-0.080	4.8E-01
Mtr.22734.1.S1_at	11.73	11.47	12.21	11.98	12.06	12.11	11.81	0.37	12.05	0.06	1.02	0.029	3.3E-01
Mtr.33098.1.S1_at	7.40	6.68	7.22	7.38	7.37	7.34	7.10	0.37	7.36	0.02	1.04	0.052	3.0E-01
Mtr.35864.1.S1_at	10.81	11.39	11.51	11.12	11.19	11.16	11.24	0.37	11.16	0.03	0.99	-0.010	7.3E-01
Mtr.5962.1.S1_s_at	12.13	12.06	12.74	12.30	12.27	12.20	12.31	0.37	12.26	0.05	1.00	-0.006	8.2E-01
Mtr.44926.1.S1_at	9.43	8.93	9.66	9.80	9.60	9.60	9.34	0.37	9.67	0.12	1.04	0.050	2.2E-01
Mtr.30184.1.S1_at	3.42	2.77	3.42	3.37	3.63	3.69	3.20	0.37	3.57	0.17	1.11	0.154	2.0E-01
Mtr.14445.1.S1_at	2.84	2.94	3.54	2.74	2.99	2.74	3.11	0.37	2.82	0.15	0.91	-0.138	2.9E-01
Mtr.42055.1.S1_s_at	7.03	6.38	7.02	7.13	7.12	7.10	6.81	0.37	7.12	0.02	1.05	0.064	2.3E-01
Mtr.35252.1.S1_at	11.01	10.29	10.83	10.56	10.36	10.51	10.71	0.37	10.47	0.10	0.98	-0.032	3.5E-01
Mtr.43788.1.S1_at	10.89	10.14	10.55	10.64	10.69	10.89	10.53	0.37	10.74	0.13	1.02	0.029	4.1E-01
Mtr.8900.1.S1_at	8.95	8.70	9.44	8.82	9.06	8.71	9.03	0.37	8.86	0.18	0.98	-0.027	5.3E-01
Mtr.47033.1.S1_at	2.58	3.22	2.57	2.68	2.61	2.60	2.79	0.37	2.63	0.04	0.94	-0.086	5.0E-01
Mtr.10070.1.S1_at	9.30	8.55	8.93	9.11	8.99	8.92	8.93	0.37	9.01	0.10	1.01	0.013	7.3E-01
Mtr.40353.1.S1_at	13.78	14.29	14.51	13.96	13.89	13.94	14.19	0.37	13.93	0.03	0.98	-0.027	3.0E-01
Mtr.4602.1.S1_at	9.31	9.56	10.05	9.49	9.53	9.49	9.64	0.37	9.50	0.02	0.99	-0.021	5.6E-01
Mtr.11862.1.S1_at	8.55	8.16	8.91	8.90	8.69	8.75	8.54	0.37	8.78	0.11	1.03	0.041	3.4E-01
Mtr.5584.1.S1_at	8.85	8.42	9.17	8.92	8.84	9.05	8.81	0.37	8.94	0.11	1.01	0.021	6.0E-01
Mtr.46497.1.S1_at	8.47	8.22	8.96	8.73	8.94	8.74	8.55	0.37	8.80	0.12	1.03	0.042	3.3E-01
Mtr.33415.1.S1_at	6.84	7.59	7.23	8.57	8.67	8.67	7.22	0.37	8.64	0.06	1.20	0.259	2.9E-03
Mtr.17250.1.S1_at	9.25	8.58	9.22	9.32	9.25	9.20	9.02	0.37	9.26	0.06	1.03	0.038	3.3E-01
Mtr.3076.1.S1_s_at	5.57	4.90	5.53	4.63	5.57	5.31	5.33	0.37	5.17	0.48	0.97	-0.045	6.7E-01
Mtr.41083.1.S1_at	10.37	9.63	10.12	10.10	10.03	10.32	10.04	0.37	10.15	0.15	1.01	0.016	6.6E-01
Mtr.33839.1.S1_at	7.95	8.52	8.66	8.55	8.55	8.43	8.38	0.37	8.51	0.07	1.02	0.023	5.8E-01
Mtr.16578.1.S1_x_at	2.89	3.64	3.20	2.85	2.85	2.90	3.24	0.37	2.87	0.03	0.88	-0.177	1.6E-01
Mtr.40677.1.S1_at	10.91	10.17	10.45	10.72	10.80	10.72	10.51	0.37	10.75	0.05	1.02	0.032	3.4E-01
Mtr.37512.1.S1_at	12.54	11.79	12.19	12.20	12.23	12.21	12.17	0.37	12.21	0.01	1.00	0.005	8.6E-01
Mtr.21594.1.S1_at	10.79	10.05	10.52	10.32	10.15	10.31	10.45	0.37	10.26	0.10	0.98	-0.027	4.4E-01
Mtr.14519.1.S1_a_at	9.88	9.42	10.16	9.82	9.87	9.59	9.82	0.37	9.76	0.15	0.99	-0.009	8.1E-01
Mtr.13465.1.S1_at	3.38	4.01	3.35	3.32	3.23	3.34	3.58	0.37	3.30	0.06	0.92	-0.118	2.7E-01
Mtr.50228.1.S1_s_at	2.18	2.82	2.17	2.17	2.17	2.16	2.39	0.37	2.17	0.01	0.91	-0.142	3.6E-01
Mtr.38395.1.S1_at	9.33	9.50	10.05	9.85	9.87	9.51	9.63	0.37	9.74	0.20	1.01	0.017	6.6E-01
Mtr.2371.1.S1_at	10.11	9.42	9.52	10.10	9.89	10.08	9.68	0.37	10.02	0.12	1.04	0.050	2.1E-01
Mtr.10862.1.S1_at	12.42	11.70	12.23	12.07	11.95	11.86	12.11	0.37	11.96	0.10	0.99	-0.019	5.3E-01
Mtr.37529.1.S1_at	11.26	10.53	10.79	10.90	10.52	10.70	10.86	0.37	10.71	0.19	0.99	-0.021	5.6E-01
Mtr.20850.1.S1_at	10.17	9.42	9.71	9.89	9.91	9.97	9.77	0.37	9.92	0.04	1.02	0.023	5.2E-01
Mtr.38021.1.S1_at	10.08	9.98	10.67	10.16	10.12	10.05	10.24	0.37	10.11	0.06	0.99	-0.019	5.8E-01
Mtr.38095.1.S1_at	9.84	9.78	10.46	11.29	11.57	11.54	10.03	0.37	11.47	0.16	1.14	0.194	3.5E-03
Mtr.32023.1.S1_at	2.89	3.53	2.87	2.87	2.78	2.84	3.10	0.37	2.83	0.05	0.91	-0.130	2.9E-01
Mtr.44361.1.S1_at	11.28	11.17	11.86	11.45	11.49	11.45	11.44	0.37	11.46	0.02	1.00	0.003	9.1E-01
Mtr.20056.1.S1_at	8.36	7.92	8.67	8.41	8.42	8.42	8.32	0.37	8.42	0.01	1.01	0.017	6.7E-01
Mtr.39872.1.S1_at	8.19	7.51	8.13	8.18	8.11	7.94	7.94	0.37	8.08	0.12	1.02	0.024	5.9E-01
Mtr.50532.1.S1_s_at	4.28	3.54	3.85	3.76	3.69	3.61	3.89	0.37	3.69	0.08	0.95	-0.079	4.0E-01
Mtr.32489.1.S1_at	10.82	10.17	10.82	10.51	10.65	10.53	10.61	0.37	10.56	0.07	1.00	-0.006	8.5E-01
Mtr.47185.1.S1_s_at	9.03	8.40	9.07	9.36	9.36	9.17	8.83	0.37	9.30	0.11	1.05	0.074	1.1E-01
Mtr.32622.1.S1_at	7.67	8.03	8.42	8.03	8.41	7.96	8.04	0.37	8.13	0.25	1.01	0.016	7.4E-01
Mtr.37990.1.S1_s_at	11.44	10.84	10.75	11.16	11.05	11.18	11.01	0.37	11.13	0.07	1.01	0.016	6.1E-01
Mtr.50974.1.S1_at	6.45	6.54	7.14	6.74	6.40	6.50	6.71	0.37	6.55	0.17	0.98	-0.036	5.3E-01
Mtr.6769.1.S1_s_at	8.53	9.21	8.60	7.85	8.09	8.18	8.78	0.37	8.04	0.17	0.92	-0.126	3.6E-02
Mtr.41999.1.S1_at	9.47	9.04	9.78	9.53	9.56	9.51	9.43	0.37	9.53	0.03	1.01	0.015	6.7E-01
Mtr.44544.1.S1_at	7.42	7.14	6.68	7.12	7.10	7.54	7.08	0.37	7.25	0.25	1.02	0.035	5.4E-01
Mtr.24756.1.S1_at	3.29	3.89	3.21	3.28	3.15	3.14	3.46	0.37	3.19	0.08	0.92	-0.117	2.9E-01
Mtr.13195.1.S1_at	11.14	10.43	10.98	11.45	11.45	11.49	10.85	0.37	11.46	0.02	1.06	0.079	4.7E-02
Mtr.45732.1.S1_at	6.52	6.67	7.23	6.64	7.07	6.68	6.81	0.37	6.80	0.24	1.00	-0.002	9.7E-01
Mtr.4866.1.S1_at	8.87	8.34	9.06	8.77	8.93	8.77	8.76	0.37	8.82	0.09	1.01	0.011	7.8E-01
Mtr.34943.1.S1_at	4.20	3.58	4.26	4.10	4.93	4.37	4.01	0.37	4.46	0.43	1.11	0.154	2.4E-01
Mtr.15403.1.S1_at	5.81	5.54	5.07	5.87	5.99	5.82	5.48	0.37	5.89	0.09	1.08	0.106	1.3E-01
Mtr.26643.1.S1_at	3.11	3.73	3.06	3.03	2.95	2.94	3.30	0.37	2.97	0.05	0.90	-0.151	2.1E-01
Mtr.11392.1.S1_at	4.12	3.41	3.95	3.95	3.99	4.24	3.83	0.37	4.06	0.16	1.06	0.086	3.7E-01
Mtr.7702.1.S1_x_at	3.03	3.64	2.97	2.93	2.88	2.95	3.21	0.37	2.92	0.04	0.91	-0.137	2.5E-01
Mtr.45921.1.S1_at	7.98	7.85	8.55	8.27	8.34	8.25	8.13	0.37	8.29	0.05	1.02	0.028	5.0E-01
Mtr.11562.1.S1_at	9.49	8.83	9.46	9.19	9.44	9.40	9.26	0.37	9.34	0.13	1.01	0.012	7.5E-01
Mtr.20588.1.S1_at	8.36	8.52	9.07	9.95	9.93	10.09	8.65	0.37	9.99	0.09	1.15	0.207	3.8E-03
Mtr.38394.1.S1_at	9.55	10.08	10.27	9.74	9.97	9.84	9.97	0.37	9.85	0.11	0.99	-0.017	6.3E-01
Mtr.46346.1.S1_at	2.26	2.90	2.26	2.24	2.22	2.22	2.47	0.37	2.22	0.01	0.90	-0.152	3.1E-01
Mtr.11261.1.S1_at	7.36	6.93	7.67	7.46	7.31	7.38	7.32	0.37	7.38	0.08	1.01	0.013	7.9E-01
Mtr.8853.1.S1_at	10.15	10.23	10.83	10.63	10.58	10.48	10.40	0.37	10.56	0.08	1.02	0.022	5.1E-01
Mtr.11298.1.S1_at	9.52	8.82	9.38	9.51	9.52	9.54	9.24	0.37	9.52	0.02	1.03	0.044	2.6E-01
Mtr.13323.1.S1_at	9.55	8.99	9.70	9.47	9.47	9.55	9.41	0.37	9.49	0.04	1.01	0.013	7.2E-01
Mtr.20354.1.S1_at	3.21	3.81	3.13	3.21	3.06	3.09	3.38	0.37	3.12	0.08	0.92	-0.117	3.0E-01
Mtr.10656.1.S1_at	12.83	12.16	12.22	12.55	12.32	12.35	12.40	0.37	12.41	0.13	1.00	0.000	1.0E+00
Mtr.22663.1.S1_at	3.37	3.96	3.28	3.25	3.17	3.22	3.54	0.37	3.21	0.04	0.91	-0.140	2.1E-01
Mtr.50574.1.S1_at	11.54	12.28	11.81	11.50	11.64	11.41	11.88	0.37	11.52	0.12	0.97	-0.044	1.9E-01
Mtr.17285.1.S1_s_at	3.33	3.98	3.33	3.80	3.76	3.62	3.55	0.37	3.73	0.09	1.05	0.072	4.6E-01
Mtr.49342.1.S1_x_at	2.94	3.55	2.87	2.86	2.80	2.79	3.12	0.37	2.82	0.04	0.90	-0.147	2.4E-01
Mtr.38417.1.S1_at	8.40	7.66	7.99	7.97	8.01	8.08	8.02	0.37	8.02	0.06	1.00	0.001	9.8E-01
Mtr.9473.1.S1_at	6.37	5.74	6.40	6.51	6.48	6.63	6.17	0.37	6.54	0.08	1.06	0.084	1.7E-01
Mtr.29693.1.S1_at	3.01	3.63	2.95	2.93	2.91	2.97	3.20	0.37	2.94	0.03	0.92	-0.123	2.9E-01
Mtr.35866.1.S1_at	3.53	3.75	3.02	3.15	3.11	2.92	3.43	0.37	3.06	0.12	0.89	-0.165	1.8E-01
Mtr.45946.1.S1_at	2.95	3.55	2.87	2.85	2.87	2.86	3.13	0.37	2.86	0.01	0.91	-0.129	2.8E-01
Mtr.17612.1.S1_x_at	11.55	10.82	11.33	11.37	11.39	11.53	11.23	0.37	11.43	0.09	1.02	0.025	4.3E-01
Mtr.23157.1.S1_at	2.96	3.60	2.95	2.95	2.95	3.01	3.17	0.37	2.97	0.04	0.94	-0.094	4.1E-01

Mtr.17186.1.S1_at	3.11	3.74	3.07	3.06	2.95	2.89	3.31	0.37	2.97	0.09	0.90	-0.157	2.0E-01
Mtr.28737.1.S1_at	2.50	2.57	3.18	2.49	2.48	2.48	2.75	0.37	2.48	0.01	0.90	-0.146	2.9E-01
Mtr.28482.1.S1_s_at	9.42	8.72	9.28	8.91	8.61	8.66	9.14	0.37	8.72	0.16	0.95	-0.068	1.5E-01
Mtr.32349.1.S1_at	8.05	8.39	8.80	8.06	8.35	7.82	8.41	0.37	8.08	0.27	0.96	-0.058	2.8E-01
Mtr.20523.1.S1_at	7.42	7.41	6.77	7.75	7.52	7.88	7.20	0.37	7.71	0.18	1.07	0.099	1.0E-01
Mtr.51412.1.S1_at	10.37	9.68	9.78	10.17	10.08	10.10	9.94	0.37	10.12	0.04	1.02	0.025	4.7E-01
Mtr.7422.1.S1_at	10.85	11.33	11.58	11.20	11.37	11.12	11.25	0.37	11.23	0.13	1.00	-0.002	9.4E-01
Mtr.4355.1.S1_at	3.57	3.78	4.30	3.70	3.71	3.71	3.88	0.37	3.71	0.00	0.95	-0.068	4.5E-01
Mtr.7642.1.S1_at	2.96	3.60	2.95	2.91	2.90	2.82	3.17	0.37	2.88	0.05	0.91	-0.139	2.5E-01
Mtr.41561.1.S1_at	13.11	13.06	13.73	13.48	13.58	13.35	13.30	0.37	13.47	0.12	1.01	0.019	4.9E-01
Mtr.29596.1.S1_at	2.98	3.61	2.95	2.94	2.86	2.80	3.18	0.37	2.87	0.07	0.90	-0.146	2.3E-01
Mtr.18344.1.S1_at	7.97	7.31	7.34	7.68	7.84	7.34	7.54	0.37	7.62	0.26	1.01	0.015	7.7E-01
Mtr.16848.1.S1_at	6.80	6.88	7.48	8.23	8.06	8.31	7.06	0.37	8.20	0.13	1.16	0.217	7.2E-03
Mtr.28243.1.S1_at	5.37	4.63	5.01	5.38	5.19	5.09	5.01	0.37	5.22	0.15	1.04	0.061	4.0E-01
Mtr.16473.1.S1_at	2.19	2.84	2.20	2.17	2.16	2.18	2.41	0.37	2.17	0.01	0.90	-0.151	3.3E-01
Mtr.10178.1.S1_at	9.65	9.02	9.68	9.65	9.69	9.63	9.45	0.37	9.65	0.03	1.02	0.031	4.0E-01
Mtr.3023.1.S1_at	11.78	12.33	12.49	12.76	12.60	12.41	12.20	0.37	12.59	0.17	1.03	0.045	1.8E-01
Mtr.31666.1.S1_at	9.17	8.83	8.42	8.58	8.10	8.46	8.81	0.37	8.38	0.25	0.95	-0.072	1.7E-01
Mtr.24198.1.S1_at	3.15	3.77	3.10	3.12	3.49	3.02	3.34	0.37	3.21	0.24	0.96	-0.058	6.4E-01
Mtr.46690.1.S1_at	6.57	5.90	6.52	6.53	6.50	6.62	6.33	0.37	6.55	0.06	1.03	0.049	3.8E-01
Mtr.25036.1.S1_at	3.77	3.14	3.80	3.62	3.93	3.51	3.57	0.37	3.69	0.22	1.03	0.046	6.7E-01
Mtr.50423.1.S1_x_at	3.05	3.75	3.17	3.23	3.11	3.20	3.33	0.37	3.18	0.07	0.96	-0.065	5.4E-01
Mtr.23496.1.S1_at	6.46	6.94	6.21	6.73	6.82	6.60	6.54	0.37	6.71	0.11	1.03	0.039	4.7E-01
Mtr.34163.1.S1_at	4.07	3.32	3.66	3.63	3.64	3.64	3.68	0.37	3.64	0.01	0.99	-0.018	8.4E-01
Mtr.45357.1.S1_at	3.74	3.72	4.37	4.02	4.72	3.97	3.95	0.37	4.24	0.42	1.07	0.103	4.2E-01
Mtr.10071.1.S1_s_at	8.32	8.16	8.87	7.86	6.98	7.82	8.45	0.37	7.55	0.50	0.89	-0.162	6.7E-02
Mtr.28594.1.S1_at	4.46	3.82	4.46	4.46	4.45	4.46	4.25	0.37	4.46	0.00	1.05	0.070	3.8E-01
Mtr.44587.1.S1_at	10.51	10.10	9.77	9.89	10.27	9.99	10.13	0.37	10.05	0.20	0.99	-0.011	7.7E-01
Mtr.13674.1.S1_at	8.95	9.45	9.68	9.20	9.43	9.18	9.36	0.37	9.27	0.14	0.99	-0.014	7.2E-01
Mtr.33028.1.S1_at	2.07	2.70	2.06	2.05	2.05	2.05	2.28	0.37	2.05	0.00	0.90	-0.148	3.6E-01
Mtr.51851.1.S1_x_at	2.30	2.92	2.26	2.26	2.04	2.23	2.49	0.37	2.18	0.12	0.87	-0.196	2.3E-01
Mtr.9075.1.S1_s_at	8.75	8.25	8.98	8.79	8.68	8.76	8.66	0.37	8.74	0.06	1.01	0.014	7.2E-01
Mtr.30771.1.S1_at	10.12	9.41	9.96	9.79	10.02	9.88	9.83	0.37	9.90	0.12	1.01	0.010	7.8E-01
Mtr.30361.1.S1_at	3.22	2.60	2.56	2.54	2.76	2.56	2.79	0.37	2.62	0.12	0.94	-0.092	4.9E-01
Mtr.32171.1.S1_at	10.34	9.61	9.87	9.96	9.79	10.25	9.94	0.37	10.00	0.24	1.01	0.009	8.2E-01
Mtr.27940.1.S1_at	7.87	8.53	8.51	8.34	8.45	8.46	8.30	0.37	8.41	0.07	1.01	0.019	6.4E-01
Mtr.50243.1.S1_at	2.71	3.34	2.68	2.71	2.70	2.69	2.91	0.37	2.70	0.01	0.93	-0.109	3.8E-01
Mtr.32379.1.S1_at	9.23	8.61	9.27	9.02	9.22	8.94	9.04	0.37	9.06	0.15	1.00	0.004	9.1E-01
Mtr.9746.1.S1_at	11.76	11.94	12.47	12.24	12.09	12.18	12.06	0.37	12.17	0.07	1.01	0.013	6.3E-01
Mtr.38067.1.S1_at	9.64	9.40	8.91	9.50	9.45	9.65	9.32	0.37	9.53	0.10	1.02	0.033	3.9E-01
Mtr.9959.1.S1_at	7.41	7.69	8.14	7.38	7.60	7.62	7.75	0.37	7.53	0.13	0.97	-0.040	4.0E-01
Mtr.43683.1.S1_at	11.61	11.24	10.86	11.00	11.02	11.08	11.24	0.37	11.03	0.05	0.98	-0.027	4.0E-01
Mtr.47877.1.S1_at	6.26	5.98	6.71	6.68	7.11	6.59	6.32	0.37	6.79	0.28	1.07	0.104	1.5E-01
Mtr.45049.1.S1_at	8.00	7.69	8.43	8.28	8.46	8.45	8.04	0.37	8.40	0.10	1.04	0.062	1.9E-01
Mtr.3351.1.S1_at	2.36	2.99	2.34	2.34	2.33	2.33	2.56	0.37	2.33	0.00	0.91	-0.135	3.4E-01
Mtr.17747.1.S1_at	2.90	3.51	2.84	2.78	2.77	2.75	3.08	0.37	2.77	0.01	0.90	-0.155	2.2E-01
Mtr.18068.1.S1_at	7.03	6.48	7.19	7.06	7.10	7.16	6.90	0.37	7.11	0.05	1.03	0.043	3.9E-01
Mtr.7115.1.S1_at	9.77	10.35	9.66	9.68	10.52	10.62	9.93	0.37	10.27	0.52	1.03	0.049	4.0E-01
Mtr.2936.1.S1_at	9.76	9.25	9.97	9.67	9.81	9.56	9.66	0.37	9.68	0.12	1.00	0.003	9.3E-01
Mtr.13111.1.S1_at	6.61	7.34	6.85	7.20	7.94	7.45	6.93	0.37	7.53	0.38	1.09	0.120	1.2E-01
Mtr.44025.1.S1_at	8.41	7.80	8.48	8.47	8.57	8.19	8.23	0.37	8.41	0.20	1.02	0.031	5.0E-01
Mtr.19362.1.S1_at	9.50	8.81	9.39	9.41	9.33	9.27	9.23	0.37	9.34	0.07	1.01	0.017	6.5E-01
Mtr.48814.1.S1_x_at	2.93	3.57	2.92	2.91	2.91	2.93	3.14	0.37	2.91	0.01	0.93	-0.108	3.5E-01
Mtr.33801.1.S1_at	3.66	4.30	3.65	3.40	3.64	3.64	3.87	0.37	3.56	0.14	0.92	-0.120	2.5E-01
Mtr.27761.1.S1_at	9.21	9.30	9.90	9.47	9.37	9.46	9.47	0.37	9.44	0.06	1.00	-0.005	8.9E-01
Mtr.9863.1.S1_at	3.13	3.73	3.05	2.98	3.08	3.08	3.31	0.37	3.05	0.06	0.92	-0.116	3.0E-01
Mtr.40837.1.S1_at	10.86	10.87	11.51	10.90	11.35	11.02	11.08	0.37	11.09	0.23	1.00	0.001	9.8E-01
Mtr.33840.1.S1_at	3.66	3.27	4.02	3.59	3.79	3.85	3.65	0.37	3.74	0.14	1.02	0.035	7.1E-01
Mtr.4729.1.S1_at	3.01	3.60	2.92	2.94	2.91	2.89	3.18	0.37	2.91	0.02	0.92	-0.126	2.8E-01
Mtr.37739.1.S1_at	7.37	6.64	6.87	6.76	7.11	6.95	6.96	0.37	6.94	0.17	1.00	-0.004	9.4E-01
Mtr.29360.1.S1_at	9.98	9.53	10.26	10.19	10.11	10.08	9.92	0.37	10.13	0.06	1.02	0.030	4.0E-01
Mtr.34101.1.S1_s_at	10.23	9.56	9.61	10.02	9.98	10.12	9.80	0.37	10.04	0.07	1.02	0.035	3.3E-01
Mtr.12665.1.S1_at	11.45	11.03	11.77	11.78	11.73	11.50	11.42	0.37	11.67	0.15	1.02	0.031	3.4E-01
Mtr.6781.1.S1_at	6.55	5.82	6.27	6.63	6.56	6.69	6.21	0.37	6.63	0.07	1.07	0.093	1.3E-01
Mtr.989.1.S1_x_at	3.29	3.94	3.30	3.27	2.95	3.27	3.51	0.37	3.17	0.19	0.90	-0.150	2.2E-01
Mtr.49454.1.S1_x_at	4.35	3.62	4.11	4.08	5.03	4.42	4.03	0.37	4.51	0.48	1.12	0.162	2.4E-01
Mtr.35742.1.S1_at	4.86	4.23	4.89	4.96	5.24	4.87	4.66	0.37	5.02	0.19	1.08	0.108	2.1E-01
Mtr.14753.1.S1_at	2.59	3.24	2.59	2.57	2.57	2.55	2.81	0.37	2.56	0.02	0.91	-0.131	3.2E-01
Mtr.20488.1.S1_at	8.87	8.13	8.52	8.71	8.59	8.73	8.51	0.37	8.67	0.07	1.02	0.028	4.8E-01
Mtr.27510.1.S1_at	5.91	5.36	5.20	5.09	5.85	5.29	5.49	0.37	5.41	0.39	0.98	-0.022	8.0E-01
Mtr.13455.1.S1_at	9.19	8.72	8.45	8.71	8.93	9.20	8.79	0.37	8.95	0.25	1.02	0.026	5.7E-01
Mtr.7400.1.S1_at	7.83	7.15	7.74	8.01	8.21	8.02	7.57	0.37	8.08	0.11	1.07	0.093	8.8E-02
Mtr.39180.1.S1_at	10.33	9.60	10.06	10.14	10.16	10.25	10.00	0.37	10.18	0.06	1.02	0.026	4.5E-01
Mtr.11059.1.S1_at	10.02	9.28	9.59	9.68	9.76	9.66	9.63	0.37	9.70	0.05	1.01	0.010	7.7E-01
Mtr.13309.1.S1_at	4.74	4.59	4.03	3.67	3.83	3.86	4.45	0.37	3.79	0.10	0.85	-0.233	4.0E-02
Mtr.48100.1.S1_at	6.27	6.52	7.00	6.97	8.14	7.54	6.60	0.37	7.55	0.59	1.14	0.194	7.6E-02
Mtr.48045.1.S1_at	10.73	10.10	10.09	10.34	10.74	10.44	10.31	0.37	10.50	0.21	1.02	0.027	4.7E-01
Mtr.2443.1.S1_at	10.17	10.55	10.91	10.35	10.61	10.37	10.55	0.37	10.44	0.15	0.99	-0.014	6.8E-01
Mtr.21791.1.S1_at	4.35	4.15	3.63	4.02	4.02	4.01	4.04	0.37	4.01	0.00	0.99	-0.010	9.0E-01
Mtr.36054.1.S1_x_at	4.10	3.53	3.40	3.42	3.69	3.39	3.68	0.37	3.50	0.17	0.95	-0.071	4.9E-01
Mtr.6649.1.S1_at	9.83	9.28	9.98	9.73	10.12	9.78	9.70	0.37	9.88	0.21	1.02	0.027	5.0E-01
Mtr.24175.1.S1_at	2.39	3.03	2.38	2.38	2.38	2.37	2.60	0.37	2.37	0.00	0.91	-0.131	3.5E-01
Mtr.5462.1.S1_at	2.68	2.83	3.39	2.63	2.68	2.63	2.97	0.37	2.65	0.03	0.89	-0.162	2.2E-01
Mtr.44195.1.S1_at	9.51	8.77	9.08	9.09	9.33	9.29	9.12	0.37	9.23	0.13	1.01	0.018	6.4E-01
Mtr.1157.1.S1_s_at	8.01	7.57	7.27	8.22	8.43	8.27	7.61	0.37	8.31	0.11	1.09	0.125	3.6E-02
Mtr.29357.1.S1_at	9.13	8.40	8.87	9.11	9.05	9.09	8.80	0.37	9.08	0.03	1.03	0.045	2.6E-01
Mtr.39930.1.S1_at	8.84	8.30	9.01	8.74	8.68	8.75	8.72	0.37	8.72	0.04	1.00	0.001	9.7E-01
Mtr.2948.1.S1_at	2.10	2.74	2.09	2.09	2.09	2.08	2.31	0.37	2.09	0.00	0.90	-0.147	3.5E-01
Mtr.41480.1.S1_at	10.65	11.39	11.08	10.70	10.77	10.98	11.04	0.37	10.82	0.14	0.98	-0.030	3.8E-01
Mtr.26132.1.S1_s_at	3.61	4.25	3.60	3.74	3.60	3.59	3.82	0.37	3.64	0.09	0.95	-0.068	4.7E-01
Mtr.2063.1.S1_at	9.39	8.83	9.53	9.55	9.40	9.36	9.25	0.37	9.44	0.10	1.02	0.029	4.4E-01
Mtr.47087.1.S1_at	9.33	8.63	8.77	8.89	8.65	8.91	8.91	0.37	8.82	0.14	0.99	-0.015	7.0E-01
Mtr.12184.1.S1_at	10.53	10.24	10.98	10.69	10.57	10.58	10.58	0.37	10.61	0.07	1.00	0.004	9.0E-01
Mtr.37899.1.S1_at	10.53	11.26	10.79	10.82	10.94	10.99	10.86	0.37	10.91	0.09	1.00	0.007	8.2E-01
Mtr.52341.1.S1_at	9.74	9.56	10.27	9.91	10.07	9.93	9.85	0.37	9.97	0.08	1.01	0.017	6.3E-01
Mtr.8589.1.S1_at	12.80	12.10	12.66	12.61	12.61	12.65	12.52	0.37	12.62	0.02	1.01	0.012	6.6E-01
Mtr.38351.1.S1_at	10.16	9.54	10.20	9.99	10.28	9.99	9.97	0.37	10.09	0.17	1.01	0.018	6.3E-01
Mtr.26822.1.S1_s_at	9.66	9.37	10.10	9.89	10.18	9.48	9.71	0.37	9.85	0.35	1.01	0.020	6.6E-01
Mtr.39632.1.S1_at	10.23	9.50	9.75	10.05	9.96	10.01	9.83	0.37	10.01	0.05	1.02	0.026	4.5E-01
Mtr.15141.1.S1_at	13.43	12.77	13.39	13.26	13.37	13.28	13.20	0.37	13.30	0.06	1.01	0.012	6.5E-01
Mtr.50848.1.S1_at	4.01	4.75	4.40	4.20	4.20	4.19	4.38	0.37	4.19	0.01	0.96	-0.064	4.3E-01
Mtr.5969.1.S1_at	8.95	8.45	9.17	9.03	9.04	9.09	8.85	0.37	9.05	0.03	1.02	0.032	4.1E-01
Mtr.44562.1.S1_at	8.48	7.79	8.38	8.25	8.34	8.21	8.22	0.37	8.27	0.07	1.01	0.009	8.3E-01
Mtr.35434.1.S1_at	3.89	3.28	3.94	6.62	7.51	5.85	3.71	0.37	6.66	0.83	1.80	0.846	4.9E-03
Mtr.7000.1.S1_at	8.69	8.37	9.11	8.98	8.71	9.01	8.72	0.37	8.90	0.16	1.02	0.029	4.9E-01
Mtr.40072.1.S1_at	8.01	8.07	8.68	8.42	8.28	8.43	8.26	0.37	8.38	0.08	1.01	0.021	6.1E-01
Mtr.898.1.S1_at	9.82	10.51	9.94	10.00	9.74	10.03	10.09	0.37	9.92	0.16	0.98	-0.024	5.1E-01
Mtr.44836.1.S1_at	8.37	8.39	9.02	8.44	8.63	8.15	8.59	0.37	8.41	0.24	0.98	-0.031	5.1E-01
Mtr.26887.1.S1_at	11.05	10.33	10.84	10.89	10.85	10.55	10.74	0.37	10.76	0.18	1.00	0.003	9.3E-01
Mtr.25396.1.S1_at	11.68	11.45	12.17	11.87	11.84	11.79	11.77	0.37	11.83	0.04	1.01	0.008	7.7E-01
Mtr.740.1.S1_s_at	8.94	8.82	9.52	9.07	9.18	9.10	9.09	0.37	9.12	0.06	1.00	0.004	9.2E-01
Mtr.38364.1.S1_at	11.32	10.63	11.20	11.17	11.02	11.06	11.05	0.37	11.08	0.08	1.00	0.004	8.9E-01
Mtr.44333.1.S1_s_at	10.14	10.15	10.78	11.17	11.21	11.08	10.35	0.37	11.15	0.07	1.08	0.107	2.1E-02
Mtr.13234.1.S1_at	4.51	3.96	4.66	4.12	4.11	3.88	4.38	0.37	4.04	0.14	0.92	-0.117	2.1E-01
Mtr.29237.1.S1_at	2.59	3.31	3.09	3.20	4.40	4.30	2.99	0.37	3.96	0.66	1.32	0.405	9.1E-02
Mtr.7566.1.S1_at	7.65	7.09	7.79	7.68	7.65	7.41	7.51	0.37	7.58	0.15	1.01	0.013	7.9E-01
Mtr.20388.1.S1_s_at	2.95	3.58	2.93	2.91	2.92	2.91	3.16	0.37	2.91	0.01	0.92	-0.116	3.2E-01
Mtr.41345.1.S1_at	9.38	8.72	8.77	8.80	8.91	8.82	8.96	0.37	8.85	0.06	0.99	-0.018	6.4E-01
Mtr.10349.1.S1_at	7.87	7.17	7.30	7.25	7.25	7.29	7.45	0.37	7.26	0.02	0.98	-0.036	4.4E-01
Mtr.1119.1.S1_at	3.26	3.87	3.21	3.19	3.06	3.04	3.45	0.37	3.10	0.08	0.90	-0.155	1.8E-01
Mtr.15291.1.S1_at	2.89	3.50	2.84	2.84	2.83	2.80	3.08	0.37	2.83	0.02	0.92	-0.123	3.1E-01
Mtr.11456.1.S1_s_at	9.53	8.83	9.37	9.47	9.29	9.59	9.24	0.37	9.45	0.15	1.02	0.032	4.2E-01
Mtr.15832.1.S1_at	2.96	3.59	2.95	2.95	2.93	2.92	3.17	0.37	2.93	0.01	0.93	-0.111	3.3E-01
Mtr.31465.1.S1_at	9.03	8.50	9.21	9.27	9.34	9.10	8.91	0.37	9.24	0.13	1.04	0.052	2.2E-01
Mtr.11894.1.S1_at	9.07	8.53	9.23	8.89	8.70	8.69	8.94	0.37	8.76	0.12	0.98	-0.030	4.5E-01
Mtr.17890.1.S1_at	3.01	3.60	2.92	2.92	2.89	2.87	3.17	0.37	2.89	0.02	0.91	-0.135	2.6E-01
Mtr.18027.1.S1_at	11.15	10.47	10.56	10.31	10.48	10.39	10.73	0.37	10.39	0.09	0.97	-0.046	2.0E-01
Mtr.27445.1.S1_at	7.03	6.71	7.45	7.22	7.44	7.05	7.06	0.37	7.24	0.20	1.02	0.035	5.2E-01
Mtr.5019.1.S1_x_at	2.61	3.23	2.57	2.58	2.57	2.52	2.80	0.37	2.56	0.03	0.91	-0.132	3.2E-01
Mtr.12739.1.S1_at	13.10	12.36	12.71	12.75	12.87	12.81	12.72	0.37	12.81	0.06	1.01	0.010	6.9E-01
Mtr.33746.1.S1_s_at	9.82	9.09	9.37	9.66	9.36	9.76	9.43	0.37	9.59	0.21	1.02	0.025	5.3E-01

Mtr.22255.1.S1_s_at	7.86	7.20	7.24	7.46	7.66	7.54	7.43	0.37	7.55	0.10	1.02	0.023	6.2E-01
Mtr.28000.1.S1_at	3.04	2.43	2.37	3.29	2.37	2.36	2.61	0.37	2.68	0.53	1.02	0.033	8.8E-01
Mtr.14149.1.S1_at	4.00	3.40	3.33	3.33	3.69	3.39	3.58	0.37	3.47	0.19	0.97	-0.044	6.8E-01
Mtr.49015.1.S1_at	11.02	10.30	10.82	10.89	10.56	10.66	10.71	0.37	10.71	0.17	1.00	-0.001	9.8E-01
Mtr.43892.1.S1_at	8.60	8.62	9.25	8.66	9.03	8.45	8.82	0.37	8.72	0.29	0.99	-0.018	7.2E-01
Mtr.41711.1.S1_at	9.76	9.06	9.59	9.55	9.67	9.30	9.47	0.37	9.51	0.19	1.00	0.006	8.8E-01
Mtr.23803.1.S1_at	8.57	7.90	8.50	8.55	8.42	8.33	8.33	0.37	8.43	0.11	1.01	0.018	6.6E-01
Mtr.47929.1.S1_at	3.14	3.75	3.09	3.07	3.05	2.96	3.33	0.37	3.02	0.06	0.91	-0.138	2.3E-01
Mtr.50947.1.S1_s_at	8.39	8.04	8.78	8.60	8.52	8.27	8.40	0.37	8.46	0.17	1.01	0.010	8.1E-01
Mtr.9753.1.S1_at	10.20	9.63	10.32	9.98	10.02	10.09	10.05	0.37	10.03	0.05	1.00	-0.003	9.3E-01
Mtr.50008.1.S1_at	2.11	2.75	2.11	2.10	2.10	2.10	2.32	0.37	2.10	0.00	0.90	-0.145	3.6E-01
Mtr.38265.1.S1_at	3.65	3.00	3.63	3.62	3.65	3.63	3.43	0.37	3.63	0.01	1.06	0.083	3.9E-01
Mtr.24098.1.S1_at	3.01	3.63	2.97	3.00	2.90	2.91	3.20	0.37	2.94	0.06	0.92	-0.125	2.9E-01
Mtr.44664.1.S1_at	9.46	8.92	9.63	10.08	10.56	10.38	9.34	0.37	10.34	0.24	1.11	0.147	1.7E-02
Mtr.6749.1.S1_at	9.41	9.10	9.84	9.56	9.57	9.55	9.45	0.37	9.56	0.01	1.01	0.017	6.2E-01
Mtr.9741.1.S1_s_at	10.04	10.56	10.75	10.44	10.69	10.37	10.45	0.37	10.50	0.17	1.00	0.007	8.5E-01
Mtr.44905.1.S1_at	8.63	8.24	8.98	8.66	8.72	8.57	8.62	0.37	8.65	0.08	1.00	0.005	8.9E-01
Mtr.37533.1.S1_at	4.07	4.14	4.74	4.07	4.75	4.05	4.32	0.37	4.29	0.40	0.99	-0.009	9.4E-01
Mtr.43287.1.S1_at	10.53	10.55	11.18	10.67	10.80	10.59	10.75	0.37	10.69	0.11	0.99	-0.009	7.7E-01
Mtr.17607.1.S1_at	14.59	13.85	14.26	14.52	14.50	14.52	14.23	0.37	14.51	0.01	1.02	0.028	2.6E-01
Mtr.48380.1.S1_at	2.53	3.24	2.70	2.52	2.52	2.51	2.82	0.37	2.52	0.01	0.89	-0.165	2.3E-01
Mtr.14708.1.S1_at	10.32	9.59	10.09	9.97	9.87	9.94	10.00	0.37	9.93	0.05	0.99	-0.011	7.5E-01
Mtr.6642.1.S1_at	11.28	10.55	10.93	10.97	10.99	10.96	10.92	0.37	10.97	0.02	1.00	0.007	8.2E-01
Mtr.19930.1.S1_at	2.13	2.76	2.11	2.11	2.11	2.11	2.33	0.37	2.11	0.00	0.90	-0.146	3.5E-01
Mtr.14008.1.S1_s_at	8.97	8.54	9.27	9.18	9.11	8.99	8.93	0.37	9.09	0.10	1.02	0.027	4.9E-01
Mtr.46338.1.S1_s_at	2.97	3.60	2.94	2.92	2.64	3.08	3.17	0.37	2.88	0.22	0.91	-0.140	3.0E-01
Mtr.23083.1.S1_at	8.93	8.40	9.12	9.14	8.99	8.98	8.82	0.37	9.04	0.09	1.02	0.035	3.8E-01
Mtr.37412.1.S1_at	12.32	11.69	12.34	12.24	12.07	12.23	12.12	0.37	12.18	0.10	1.01	0.007	7.9E-01
Mtr.39826.1.S1_at	10.15	9.58	9.47	9.49	9.45	9.54	9.73	0.37	9.49	0.05	0.98	-0.036	3.3E-01
Mtr.29227.1.S1_at	9.87	10.58	10.41	10.29	10.52	10.48	10.29	0.37	10.43	0.12	1.01	0.020	5.6E-01
Mtr.45721.1.S1_at	9.53	8.79	9.16	9.49	9.40	9.56	9.16	0.37	9.48	0.08	1.04	0.050	2.1E-01
Mtr.11695.1.S1_at	8.91	8.65	9.38	9.26	9.11	8.98	8.98	0.37	9.12	0.14	1.02	0.022	5.8E-01
Mtr.33023.1.S1_at	9.73	9.00	9.44	9.74	9.66	9.70	9.39	0.37	9.70	0.04	1.03	0.047	2.2E-01
Mtr.25077.1.S1_at	9.82	10.14	10.56	9.96	9.99	9.93	10.17	0.37	9.96	0.03	0.98	-0.031	3.7E-01
Mtr.50716.1.S1_s_at	12.16	11.49	11.56	11.84	11.49	11.75	11.74	0.37	11.69	0.18	1.00	-0.005	8.6E-01
Mtr.13277.1.S1_at	10.26	9.53	9.94	9.95	9.93	9.89	9.91	0.37	9.92	0.03	1.00	0.002	9.5E-01
Mtr.10245.1.S1_at	10.14	9.41	9.89	10.10	10.10	10.08	9.81	0.37	10.10	0.01	1.03	0.041	2.6E-01
Mtr.9194.1.S1_s_at	8.48	7.96	8.67	8.79	8.68	8.67	8.37	0.37	8.71	0.07	1.04	0.058	1.9E-01
Mtr.18386.1.S1_at	7.13	6.80	7.53	7.17	7.18	7.19	7.15	0.37	7.18	0.01	1.00	0.005	9.1E-01
Mtr.49092.1.S1_at	6.98	6.96	7.61	7.35	6.93	7.36	7.18	0.37	7.21	0.25	1.00	0.006	9.2E-01
Mtr.32329.1.S1_s_at	9.23	8.78	9.52	9.43	9.63	9.12	9.18	0.37	9.39	0.26	1.02	0.033	4.6E-01
Mtr.21362.1.S1_s_at	9.99	9.82	9.29	9.42	9.54	9.63	9.70	0.37	9.53	0.11	0.98	-0.025	4.9E-01
Mtr.28320.1.S1_at	6.44	6.14	6.88	7.01	7.24	6.69	6.49	0.37	6.98	0.28	1.08	0.106	1.4E-01
Mtr.7133.1.S1_at	10.14	9.54	10.21	10.21	9.81	10.05	9.97	0.37	10.02	0.20	1.01	0.008	8.3E-01
Mtr.3261.1.S1_at	10.39	9.67	9.91	9.93	10.05	9.97	9.99	0.37	9.99	0.06	1.00	-0.001	9.8E-01
Mtr.10360.1.S1_s_at	10.13	9.48	10.11	10.38	10.40	10.31	9.90	0.37	10.36	0.05	1.05	0.065	9.9E-02
Mtr.31779.1.S1_at	2.45	2.49	3.11	2.42	2.47	2.42	2.69	0.37	2.44	0.03	0.91	-0.139	3.1E-01
Mtr.18980.1.S1_at	7.51	6.94	7.63	7.80	7.54	7.74	7.36	0.37	7.69	0.13	1.04	0.063	2.2E-01
Mtr.44946.1.S1_at	9.60	9.04	9.74	9.72	9.72	9.68	9.46	0.37	9.70	0.02	1.03	0.037	3.1E-01
Mtr.1076.1.S1_at	11.07	10.88	11.59	11.53	11.56	11.30	11.18	0.37	11.47	0.14	1.03	0.036	2.8E-01
Mtr.24465.1.S1_x_at	5.29	5.19	4.61	4.67	5.24	5.07	5.03	0.37	5.00	0.29	0.99	-0.010	9.1E-01
Mtr.4224.1.S1_at	6.59	6.89	7.32	6.13	7.17	6.52	6.93	0.37	6.60	0.52	0.95	-0.070	4.2E-01
Mtr.20265.1.S1_at	13.56	12.90	13.51	13.61	13.51	13.52	13.33	0.37	13.55	0.05	1.02	0.024	3.6E-01
Mtr.16230.1.S1_at	10.21	9.54	10.13	9.83	9.91	9.93	9.96	0.37	9.89	0.05	0.99	-0.011	7.5E-01
Mtr.7365.1.S1_at	4.29	3.61	4.21	4.35	4.14	4.14	4.04	0.37	4.21	0.12	1.04	0.061	4.8E-01
Mtr.34988.1.S1_s_at	12.51	12.14	11.78	12.00	11.66	12.20	12.14	0.37	11.95	0.27	0.98	-0.023	5.1E-01
Mtr.9199.1.S1_at	10.38	9.65	9.98	10.05	10.09	10.00	10.01	0.37	10.05	0.05	1.00	0.006	8.6E-01
Mtr.24641.1.S1_at	2.94	3.57	2.92	2.90	2.94	2.87	3.14	0.37	2.91	0.04	0.93	-0.112	3.3E-01
Mtr.418.1.S1_at	2.93	3.56	2.92	2.91	2.85	2.91	3.14	0.37	2.89	0.04	0.92	-0.118	3.1E-01
Mtr.45544.1.S1_s_at	6.71	5.98	6.35	5.90	6.59	6.96	6.35	0.37	6.48	0.54	1.02	0.031	7.3E-01
Mtr.11713.1.S1_at	9.61	8.88	9.34	9.37	9.36	9.45	9.28	0.37	9.39	0.05	1.01	0.018	6.2E-01
Mtr.44394.1.S1_at	10.56	9.83	10.13	10.53	10.32	10.45	10.17	0.37	10.43	0.11	1.03	0.036	3.1E-01
Mtr.39271.1.S1_at	3.08	3.79	3.61	3.59	4.30	4.20	3.49	0.37	4.03	0.38	1.15	0.207	1.5E-01
Mtr.45407.1.S1_at	7.78	7.31	7.05	7.66	7.50	7.62	7.38	0.37	7.59	0.09	1.03	0.041	3.9E-01
Mtr.19285.1.S1_s_at	8.64	7.97	8.57	8.76	8.66	8.77	8.39	0.37	8.73	0.06	1.04	0.057	1.9E-01
Mtr.34673.1.S1_at	2.37	3.00	2.35	2.46	2.34	2.33	2.57	0.37	2.38	0.07	0.92	-0.114	4.2E-01
Mtr.32370.1.S1_at	5.52	4.95	5.65	5.71	5.81	5.91	5.37	0.37	5.81	0.10	1.08	0.113	1.2E-01
Mtr.11368.1.S1_at	2.64	3.26	2.61	2.59	2.59	2.62	2.84	0.37	2.60	0.02	0.92	-0.125	3.3E-01
Mtr.37248.1.S1_at	14.67	14.27	13.93	14.20	14.17	14.26	14.29	0.37	14.21	0.04	0.99	-0.008	7.4E-01
Mtr.11134.1.S1_at	11.77	11.05	11.32	11.42	11.60	11.34	11.38	0.37	11.45	0.13	1.01	0.009	7.6E-01
Mtr.4449.1.S1_at	6.96	6.50	7.22	6.97	7.01	7.15	6.89	0.37	7.04	0.10	1.02	0.031	5.3E-01
Mtr.17524.1.S1_x_at	2.37	3.00	2.36	2.36	2.34	2.35	2.58	0.37	2.35	0.01	0.91	-0.133	3.5E-01
Mtr.20425.1.S1_at	11.03	10.31	10.77	10.62	10.87	11.07	10.71	0.37	10.85	0.22	1.01	0.020	5.9E-01
Mtr.45033.1.S1_at	11.00	10.38	11.03	10.66	10.80	10.61	10.81	0.37	10.69	0.10	0.99	-0.016	6.2E-01
Mtr.14518.1.S1_at	11.26	11.77	11.98	11.42	11.47	11.45	11.67	0.37	11.44	0.02	0.98	-0.028	3.5E-01
Mtr.13330.1.S1_at	8.03	8.61	8.70	8.47	8.58	8.35	8.45	0.37	8.47	0.11	1.00	0.003	9.3E-01
Mtr.1145.1.S1_at	2.66	3.28	2.64	2.65	2.71	2.59	2.86	0.37	2.65	0.06	0.93	-0.108	3.9E-01
Mtr.5693.1.S1_at	11.10	10.37	10.74	10.98	10.72	10.94	10.74	0.37	10.88	0.14	1.01	0.019	5.6E-01
Mtr.21144.1.S1_at	8.19	7.53	8.15	8.27	8.23	8.03	7.96	0.37	8.18	0.13	1.03	0.039	3.9E-01
Mtr.1081.1.S1_at	8.60	7.87	8.31	8.37	8.58	8.43	8.26	0.37	8.46	0.11	1.02	0.035	4.2E-01
Mtr.42360.1.S1_at	7.46	7.90	7.17	8.12	8.34	8.13	7.51	0.37	8.20	0.12	1.09	0.126	3.7E-02
Mtr.44501.1.S1_at	12.20	11.71	11.47	11.55	11.42	11.57	11.79	0.37	11.52	0.08	0.98	-0.034	2.7E-01
Mtr.39176.1.S1_at	10.84	10.11	10.55	10.56	10.51	10.58	10.50	0.37	10.55	0.03	1.00	0.007	8.3E-01
Mtr.29196.1.S1_at	2.12	2.76	2.13	2.13	2.13	2.13	2.34	0.37	2.13	0.00	0.91	-0.136	3.8E-01
Mtr.51989.1.S1_s_at	6.48	5.75	6.13	7.02	7.17	6.95	6.12	0.37	7.04	0.11	1.15	0.203	1.4E-02
Mtr.36174.1.S1_at	2.23	2.86	2.22	2.21	2.21	2.20	2.44	0.37	2.21	0.00	0.91	-0.141	3.4E-01
Mtr.45305.1.S1_at	2.73	2.62	3.30	3.06	2.65	2.67	2.89	0.37	2.79	0.23	0.97	-0.047	7.3E-01
Mtr.35306.1.S1_at	7.23	7.26	7.88	7.36	7.75	7.34	7.46	0.37	7.48	0.23	1.00	0.005	9.2E-01
Mtr.48287.1.S1_s_at	10.38	9.65	10.11	10.13	10.03	10.15	10.04	0.37	10.10	0.06	1.01	0.008	8.0E-01
Mtr.52178.1.S1_at	7.37	7.64	8.09	7.67	7.77	7.39	7.70	0.37	7.61	0.20	0.99	-0.017	7.2E-01
Mtr.957.1.S1_at	9.43	8.95	9.67	9.55	9.85	9.66	9.35	0.37	9.69	0.15	1.04	0.051	2.2E-01
Mtr.30835.1.S1_at	1.98	2.61	1.97	1.96	1.96	1.96	2.18	0.37	1.96	0.00	0.90	-0.155	3.5E-01
Mtr.30603.1.S1_at	2.08	2.71	2.07	2.10	2.07	2.06	2.29	0.37	2.07	0.02	0.91	-0.142	3.7E-01
Mtr.22427.1.S1_at	2.63	3.25	2.60	2.61	2.61	2.56	2.83	0.37	2.59	0.03	0.92	-0.126	3.3E-01
Mtr.12219.1.S1_at	11.85	11.16	11.73	11.57	11.65	11.59	11.58	0.37	11.60	0.04	1.00	0.003	9.1E-01
Mtr.31215.1.S1_s_at	3.18	2.65	2.48	2.68	2.82	2.51	2.77	0.37	2.67	0.16	0.96	-0.052	6.9E-01
Mtr.45193.1.S1_at	8.38	8.29	7.70	8.16	8.19	8.14	8.12	0.37	8.16	0.02	1.00	0.007	8.6E-01
Mtr.43176.1.S1_s_at	12.41	11.72	11.85	12.02	11.73	12.00	11.99	0.37	11.92	0.16	0.99	-0.009	7.7E-01
Mtr.36379.1.S1_s_at	9.64	9.08	9.76	9.41	9.62	9.46	9.49	0.37	9.50	0.11	1.00	0.001	9.9E-01
Mtr.46824.1.S1_at	8.88	9.15	9.60	9.37	9.26	8.95	9.21	0.37	9.19	0.22	1.00	-0.003	9.5E-01
Mtr.27956.1.S1_s_at	8.92	8.19	8.62	8.81	8.75	8.87	8.58	0.37	8.81	0.06	1.03	0.038	3.4E-01
Mtr.43123.1.S1_at	12.08	11.47	12.12	11.94	11.60	11.79	11.89	0.37	11.78	0.17	0.99	-0.013	6.6E-01
Mtr.44323.1.S1_at	10.45	9.87	10.55	10.55	10.40	10.41	10.29	0.37	10.45	0.08	1.02	0.022	5.0E-01
Mtr.38470.1.S1_s_at	7.78	7.10	7.68	7.78	7.75	7.83	7.52	0.37	7.79	0.04	1.04	0.050	2.8E-01
Mtr.39466.1.S1_at	6.24	6.28	6.89	6.40	6.55	6.33	6.47	0.37	6.43	0.12	0.99	-0.010	8.6E-01
Mtr.37865.1.S1_at	9.64	9.06	9.73	9.52	9.46	9.52	9.48	0.37	9.50	0.03	1.00	0.003	9.3E-01
Mtr.10883.1.S1_at	10.59	10.31	11.03	10.77	10.97	10.66	10.64	0.37	10.80	0.16	1.01	0.021	5.4E-01
Mtr.45196.1.S1_at	9.57	9.28	10.01	9.55	9.77	9.55	9.62	0.37	9.62	0.13	1.00	0.000	9.9E-01
Mtr.28940.1.S1_s_at	6.41	7.03	7.06	6.86	6.85	7.02	6.83	0.37	6.91	0.10	1.01	0.016	7.5E-01
Mtr.39839.1.S1_s_at	6.02	6.42	6.75	6.39	6.37	6.38	6.39	0.37	6.38	0.01	1.00	-0.003	9.5E-01
Mtr.43741.1.S1_at	8.72	8.16	8.85	8.64	8.73	8.45	8.58	0.37	8.61	0.14	1.00	0.005	9.0E-01
Mtr.44413.1.S1_at	11.34	10.62	10.86	11.06	11.10	10.91	10.94	0.37	11.02	0.10	1.01	0.011	7.2E-01
Mtr.44012.1.S1_at	9.31	9.22	9.89	9.36	9.28	9.40	9.47	0.37	9.34	0.06	0.99	-0.020	5.8E-01
Mtr.24903.1.S1_at	2.93	3.55	2.89	2.88	2.87	2.87	3.12	0.37	2.87	0.01	0.92	-0.121	3.0E-01
Mtr.41287.1.S1_at	8.22	7.65	8.33	7.86	8.05	7.92	8.07	0.37	7.94	0.09	0.98	-0.022	6.0E-01
Mtr.30809.1.S1_at	3.72	3.36	2.98	2.99	3.12	2.97	3.35	0.37	3.03	0.08	0.90	-0.146	2.1E-01
Mtr.1633.1.S1_x_at	3.88	4.48	3.82	3.78	3.33	3.80	4.06	0.37	3.64	0.27	0.90	-0.158	1.8E-01
Mtr.41514.1.S1_at	11.00	10.28	10.54	11.06	10.72	11.12	10.60	0.37	10.97	0.22	1.03	0.049	2.1E-01
Mtr.27451.1.S1_at	6.32	6.16	5.62	6.17	6.72	6.22	6.03	0.37	6.37	0.30	1.06	0.079	2.8E-01
Mtr.43263.1.S1_at	12.11	11.38	11.78	11.78	12.09	11.91	11.76	0.37	11.93	0.16	1.01	0.021	5.0E-01
Mtr.15105.1.S1_at	10.15	9.63	10.34	10.22	10.02	10.08	10.04	0.37	10.11	0.10	1.01	0.010	7.7E-01
Mtr.22932.1.S1_at	7.24	7.41	7.94	7.49	7.64	7.25	7.53	0.37	7.46	0.19	0.99	-0.013	7.9E-01
Mtr.15432.1.S1_at	2.87	3.49	2.84	2.82	2.79	2.78	3.07	0.37	2.80	0.02	0.91	-0.134	2.7E-01
Mtr.2303.1.S1_at	10.58	9.86	10.34	10.52	10.55	10.53	10.26	0.37	10.53	0.02	1.03	0.037	2.7E-01
Mtr.27409.1.S1_at	5.14	4.50	5.13	5.13	5.13	5.13	4.93	0.37	5.13	0.00	1.04	0.058	3.9E-01
Mtr.33544.1.S1_at	11.89	11.55	12.28	12.09	12.06	12.06	11.91	0.37	12.07	0.02	1.01	0.020	4.8E-01
Mtr.26323.1.S1_at	7.81	8.51	8.36	8.24	8.72	8.04	8.23	0.37	8.34	0.35	1.01	0.019	7.3E-01
Mtr.6896.1.S1_at	6.88	6.47	7.20	6.54	6.31	6.72	6.85	0.37	6.52	0.21	0.95	-0.070	2.5E-01
Mtr.11186.1.S1_at	2.93	2.31	2.28	2.27	2.28	2.17	2.50	0.37	2.24	0.06	0.89	-0.160	2.9E-01
Mtr.38997.1.S1_at	7.84	7.18	7.80	7.77	8.04	7.99	7.61	0.37	7.93	0.14	1.04	0.061	2.2E-01
Mtr.31134.1.S1_at	12.42	12.82	13.15	13.00	13.01	12.74	12.79	0.37	12.92	0.16	1.01	0.014	6.2E-01

Mtr.33228.1.S1_x_at	3.64	3.00	3.63	3.62	4.48	3.59	3.43	0.37	3.90	0.50	1.14	0.185	2.6E-01
Mtr.4318.1.S1_s_at	9.37	8.67	9.20	9.28	9.27	9.19	9.08	0.37	9.25	0.05	1.02	0.026	4.8E-01
Mtr.8754.1.S1_at	10.13	10.18	10.79	10.37	10.46	10.24	10.37	0.37	10.35	0.11	1.00	-0.002	9.6E-01
Mtr.45161.1.S1_at	5.35	5.05	5.78	5.47	5.52	5.59	5.39	0.37	5.53	0.06	1.03	0.036	5.5E-01
Mtr.39704.1.S1_x_at	3.08	3.69	3.04	3.06	3.06	2.99	3.27	0.37	3.04	0.04	0.93	-0.106	3.4E-01
Mtr.27653.1.S1_s_at	5.90	5.27	5.92	5.80	5.87	5.87	5.70	0.37	5.85	0.04	1.03	0.038	5.1E-01
Mtr.30232.1.S1_at	5.62	5.76	6.31	5.92	6.21	6.14	5.90	0.37	6.09	0.15	1.03	0.047	4.4E-01
Mtr.50467.1.S1_at	8.71	7.98	8.42	7.59	6.94	7.36	8.37	0.37	7.30	0.33	0.87	-0.198	1.9E-02
Mtr.23790.1.S1_at	3.08	3.71	3.07	3.07	2.92	3.03	3.28	0.37	3.00	0.08	0.91	-0.129	2.6E-01
Mtr.46603.1.S1_at	2.64	3.27	2.63	2.60	2.60	2.63	2.85	0.37	2.61	0.02	0.92	-0.125	3.3E-01
Mtr.42197.1.S1_at	11.07	11.41	11.80	11.33	11.34	11.32	11.42	0.37	11.33	0.01	0.99	-0.013	6.6E-01
Mtr.971.1.S1_at	10.93	10.25	10.83	10.70	10.68	10.78	10.67	0.37	10.72	0.05	1.00	0.007	8.2E-01
Mtr.8644.1.S1_x_at	2.77	3.39	2.74	2.73	2.68	2.68	2.97	0.37	2.69	0.03	0.91	-0.139	2.7E-01
Mtr.2145.1.S1_at	8.32	7.87	8.60	8.32	8.34	8.24	8.26	0.37	8.30	0.05	1.00	0.007	8.6E-01
Mtr.38419.1.S1_at	10.53	9.88	10.48	10.47	10.58	10.54	10.30	0.37	10.53	0.05	1.02	0.032	3.4E-01
Mtr.23724.1.S1_s_at	3.16	3.76	3.10	3.04	3.01	3.01	3.34	0.37	3.02	0.02	0.90	-0.145	2.0E-01
Mtr.39749.1.S1_at	6.70	6.34	5.97	6.26	6.25	5.89	6.33	0.37	6.13	0.22	0.97	-0.047	4.6E-01
Mtr.34687.1.S1_s_at	9.35	9.49	10.04	9.71	9.86	9.56	9.63	0.37	9.71	0.15	1.01	0.013	7.3E-01
Mtr.39253.1.S1_at	9.47	8.77	8.95	9.51	9.50	9.31	9.06	0.37	9.44	0.11	1.04	0.059	1.6E-01
Mtr.3223.1.S1_at	5.07	5.75	5.17	4.52	5.05	4.97	5.33	0.37	4.85	0.29	0.91	-0.136	1.5E-01
Mtr.21294.1.S1_s_at	2.53	3.17	2.53	2.51	2.53	2.50	2.74	0.37	2.51	0.02	0.92	-0.128	3.3E-01
Mtr.37692.1.S1_at	3.92	3.20	3.64	3.64	4.58	3.88	3.59	0.37	4.03	0.49	1.12	0.169	2.8E-01
Mtr.41703.1.S1_at	10.53	9.86	9.94	10.15	9.84	10.17	10.11	0.37	10.05	0.18	0.99	-0.008	8.2E-01
Mtr.4944.1.S1_at	3.48	3.99	3.28	4.00	3.50	3.40	3.59	0.37	3.63	0.32	1.01	0.018	8.8E-01
Mtr.46168.1.S1_at	2.79	3.41	2.78	2.70	2.77	2.77	2.99	0.37	2.74	0.04	0.92	-0.125	3.1E-01
Mtr.45068.1.S1_at	9.59	8.92	9.50	9.22	9.12	9.17	9.34	0.37	9.17	0.05	0.98	-0.026	4.8E-01
Mtr.2956.1.S1_at	9.82	9.10	9.53	9.72	9.62	9.70	9.48	0.37	9.68	0.06	1.02	0.029	4.1E-01
Mtr.37439.1.S1_at	12.45	11.74	11.95	12.16	12.16	12.21	12.05	0.37	12.18	0.03	1.01	0.016	5.7E-01
Mtr.39421.1.S1_at	7.90	7.19	7.41	7.96	7.91	7.74	7.50	0.37	7.87	0.11	1.05	0.069	1.7E-01
Mtr.37049.1.S1_at	7.18	6.88	7.60	7.34	7.74	7.35	7.22	0.37	7.48	0.23	1.04	0.050	3.6E-01
Mtr.48866.1.S1_at	9.67	9.13	8.97	7.49	6.73	7.70	9.25	0.37	7.31	0.51	0.79	-0.341	5.8E-03
Mtr.17954.1.S1_s_at	9.00	8.35	8.96	9.15	9.21	9.25	8.77	0.36	9.20	0.05	1.05	0.069	1.1E-01
Mtr.35312.1.S1_at	10.56	10.87	11.29	10.60	10.68	10.56	10.90	0.36	10.62	0.06	0.97	-0.039	2.5E-01
Mtr.9421.1.S1_at	9.34	9.02	9.75	9.58	9.61	9.32	9.37	0.36	9.50	0.16	1.01	0.020	6.0E-01
Mtr.40041.1.S1_at	14.57	13.92	13.95	14.62	14.47	14.67	14.14	0.36	14.59	0.11	1.03	0.044	1.1E-01
Mtr.13267.1.S1_at	10.82	11.28	11.54	11.23	11.29	11.33	11.21	0.36	11.28	0.05	1.01	0.009	7.5E-01
Mtr.9843.1.S1_at	9.65	8.92	9.23	9.42	9.13	9.44	9.27	0.36	9.33	0.18	1.01	0.010	8.0E-01
Mtr.2064.1.S1_at	6.48	5.76	6.03	5.73	5.39	5.61	6.09	0.36	5.58	0.17	0.92	-0.128	9.2E-02
Mtr.48503.1.S1_at	10.31	10.88	10.99	10.63	10.81	10.76	10.73	0.36	10.73	0.09	1.00	0.001	9.8E-01
Mtr.34173.1.S1_at	9.32	9.92	9.26	9.53	9.17	9.68	9.50	0.36	9.46	0.26	1.00	-0.006	8.9E-01
Mtr.32033.1.S1_at	3.31	3.90	3.24	3.26	3.18	3.25	3.48	0.36	3.23	0.04	0.93	-0.109	3.0E-01
Mtr.41830.1.S1_at	10.50	9.81	9.96	10.42	10.47	10.64	10.09	0.36	10.51	0.12	1.04	0.059	1.3E-01
Mtr.40920.1.S1_at	5.77	5.04	5.43	5.55	5.83	5.37	5.41	0.36	5.58	0.23	1.03	0.044	5.4E-01
Mtr.26620.1.S1_at	8.74	8.02	8.27	8.51	8.61	8.77	8.34	0.36	8.63	0.13	1.03	0.049	2.6E-01
Mtr.45795.1.S1_at	10.85	11.44	11.51	11.16	11.29	10.90	11.27	0.36	11.11	0.19	0.99	-0.020	5.5E-01
Mtr.46731.1.S1_at	4.62	5.08	5.34	4.86	5.20	4.77	5.02	0.36	4.94	0.23	0.99	-0.021	7.8E-01
Mtr.40952.1.S1_at	9.28	8.73	9.42	9.32	9.35	9.32	9.14	0.36	9.33	0.01	1.02	0.029	4.2E-01
Mtr.12671.1.S1_at	9.23	8.87	9.60	9.29	9.51	9.29	9.23	0.36	9.36	0.13	1.01	0.020	5.9E-01
Mtr.35848.1.S1_s_at	9.64	9.02	9.66	9.63	9.67	9.56	9.44	0.36	9.62	0.05	1.02	0.027	4.4E-01
Mtr.52040.1.S1_at	10.84	10.89	11.49	11.43	11.19	10.95	11.07	0.36	11.19	0.24	1.01	0.015	6.6E-01
Mtr.34495.1.S1_s_at	9.12	8.42	8.92	9.05	8.93	9.21	8.82	0.36	9.07	0.14	1.03	0.040	3.4E-01
Mtr.10421.1.S1_at	13.59	13.00	13.66	13.61	13.70	13.69	13.42	0.36	13.67	0.05	1.02	0.026	3.1E-01
Mtr.45437.1.S1_at	8.31	8.81	8.10	8.64	8.49	8.60	8.41	0.36	8.58	0.08	1.02	0.028	4.8E-01
Mtr.37487.1.S1_s_at	10.28	9.55	9.94	9.94	9.97	10.01	9.92	0.36	9.97	0.04	1.01	0.007	8.2E-01
Mtr.31782.1.S1_at	2.71	3.33	2.69	2.69	2.69	2.69	2.91	0.36	2.69	0.00	0.92	-0.114	3.5E-01
Mtr.13893.1.S1_at	2.99	2.36	2.99	3.16	2.97	2.98	2.78	0.36	3.04	0.11	1.09	0.128	3.1E-01
Mtr.42216.1.S1_at	10.10	9.54	10.22	9.95	10.19	9.79	9.95	0.36	9.98	0.20	1.00	0.004	9.1E-01
Mtr.18298.1.S1_at	11.64	11.19	10.92	11.28	11.28	11.55	11.25	0.36	11.37	0.15	1.01	0.015	6.3E-01
Mtr.27719.1.S1_at	9.08	8.40	8.97	9.05	9.11	9.21	8.82	0.36	9.12	0.09	1.03	0.049	2.3E-01
Mtr.3803.1.S1_at	6.19	5.99	6.70	6.54	7.05	6.63	6.29	0.36	6.74	0.27	1.07	0.099	1.6E-01
Mtr.31063.1.S1_at	7.76	7.06	7.60	7.73	8.06	7.66	7.47	0.36	7.82	0.22	1.05	0.065	2.3E-01
Mtr.11718.1.S1_at	6.23	5.61	5.59	5.59	5.59	5.35	5.81	0.36	5.51	0.14	0.95	-0.077	2.5E-01
Mtr.35853.1.S1_at	7.04	6.68	7.40	7.18	7.07	7.13	7.04	0.36	7.13	0.05	1.01	0.017	7.1E-01
Mtr.49621.1.S1_at	2.80	3.42	2.78	2.78	2.78	2.78	3.00	0.36	2.78	0.00	0.93	-0.109	3.6E-01
Mtr.12096.1.S1_at	5.44	5.52	6.11	5.95	6.12	5.53	5.69	0.36	5.86	0.31	1.03	0.044	5.6E-01
Mtr.39962.1.S1_at	10.42	9.71	10.18	10.25	10.25	10.24	10.10	0.36	10.25	0.00	1.01	0.020	5.4E-01
Mtr.13317.1.S1_at	11.39	10.74	11.34	11.21	11.26	11.34	11.16	0.36	11.27	0.06	1.01	0.015	6.2E-01
Mtr.8288.1.S1_at	2.43	3.05	2.42	2.42	2.40	2.39	2.63	0.36	2.40	0.02	0.91	-0.132	3.3E-01
Mtr.13209.1.S1_at	8.46	8.09	7.73	8.45	8.43	8.49	8.09	0.36	8.46	0.03	1.05	0.064	1.6E-01
Mtr.19614.1.S1_at	9.23	8.51	8.99	9.06	9.01	9.05	8.91	0.36	9.04	0.03	1.01	0.021	5.7E-01
Mtr.9883.1.S1_at	7.18	7.15	7.80	7.51	7.50	7.50	7.38	0.36	7.50	0.00	1.02	0.024	5.9E-01
Mtr.22897.1.S1_s_at	9.18	9.35	9.88	9.45	9.81	9.51	9.47	0.36	9.59	0.19	1.01	0.019	6.2E-01
Mtr.49353.1.S1_at	10.72	10.02	10.52	10.66	10.65	10.56	10.42	0.36	10.62	0.06	1.02	0.027	4.0E-01
Mtr.37275.1.S1_at	13.37	13.54	12.84	13.33	13.32	13.40	13.25	0.36	13.35	0.04	1.01	0.010	6.7E-01
Mtr.47539.1.S1_at	2.07	2.70	2.06	2.06	2.06	2.05	2.28	0.36	2.06	0.00	0.90	-0.147	3.5E-01
Mtr.36379.1.S1_at	3.64	3.85	4.35	3.55	3.49	3.60	3.95	0.36	3.55	0.05	0.90	-0.154	1.3E-01
Mtr.5991.1.S1_at	3.22	3.89	3.30	3.16	3.09	3.08	3.47	0.36	3.11	0.04	0.90	-0.158	1.6E-01
Mtr.52202.1.S1_s_at	8.55	8.53	9.17	8.67	8.86	8.63	8.75	0.36	8.72	0.12	1.00	-0.005	9.0E-01
Mtr.1804.1.S1_at	9.03	8.59	9.31	9.01	9.16	8.93	8.97	0.36	9.03	0.11	1.01	0.010	8.0E-01
Mtr.22620.1.S1_at	2.60	3.21	2.56	2.55	2.55	2.53	2.79	0.36	2.54	0.01	0.91	-0.133	3.1E-01
Mtr.27633.1.S1_at	7.20	6.94	7.66	7.27	7.07	7.07	7.27	0.36	7.14	0.11	0.98	-0.026	5.9E-01
Mtr.32085.1.S1_at	10.62	9.91	10.12	9.95	9.25	9.65	10.22	0.36	9.61	0.35	0.94	-0.087	1.1E-01
Mtr.27352.1.S1_at	8.75	8.04	8.54	8.71	8.65	8.87	8.44	0.36	8.74	0.11	1.04	0.050	2.5E-01
Mtr.6892.1.S1_at	5.70	5.03	5.12	5.22	5.18	5.27	5.28	0.36	5.22	0.04	0.99	-0.017	7.9E-01
Mtr.28604.1.S1_at	1.91	1.95	2.56	3.58	4.85	3.10	2.14	0.36	3.84	0.90	1.80	0.845	3.9E-02
Mtr.51322.1.S1_at	2.39	3.02	2.38	2.38	2.36	2.40	2.60	0.36	2.38	0.02	0.92	-0.127	3.6E-01
Mtr.33158.1.S1_at	7.30	7.49	8.00	6.86	7.15	7.22	7.60	0.36	7.08	0.19	0.93	-0.103	9.3E-02
Mtr.709.1.S1_at	2.11	2.73	2.10	2.11	2.10	2.14	2.31	0.36	2.11	0.02	0.91	-0.131	4.0E-01
Mtr.40087.1.S1_at	10.16	10.47	10.89	10.55	10.53	10.50	10.51	0.36	10.53	0.03	1.00	0.002	9.4E-01
Mtr.26321.1.S1_at	2.90	3.34	2.62	2.74	2.71	2.58	2.95	0.36	2.68	0.09	0.91	-0.142	2.7E-01
Mtr.10865.1.S1_at	9.51	8.78	9.21	9.33	9.37	9.38	9.17	0.36	9.36	0.03	1.02	0.031	4.0E-01
Mtr.31901.1.S1_at	10.96	10.26	10.78	10.78	10.72	10.81	10.67	0.36	10.77	0.04	1.01	0.014	6.6E-01
Mtr.33440.1.S1_at	5.85	5.91	6.51	5.93	5.75	5.83	6.09	0.36	5.84	0.09	0.96	-0.061	3.1E-01
Mtr.4585.1.S1_s_at	9.67	10.15	10.39	10.04	9.99	9.75	10.07	0.36	9.93	0.15	0.99	-0.020	5.7E-01
Mtr.19830.1.S1_at	3.38	3.84	3.13	3.37	3.10	3.68	3.45	0.36	3.38	0.29	0.98	-0.028	8.2E-01
Mtr.43685.1.S1_at	10.06	9.45	9.42	9.70	9.82	9.59	9.64	0.36	9.70	0.12	1.01	0.009	8.0E-01
Mtr.30644.1.S1_at	6.02	6.27	6.73	5.99	6.78	6.51	6.34	0.36	6.43	0.40	1.01	0.020	7.9E-01
Mtr.46184.1.S1_at	10.20	9.55	10.15	9.98	9.82	10.01	9.97	0.36	9.93	0.10	1.00	-0.005	8.9E-01
Mtr.18814.1.S1_at	11.59	10.89	11.07	11.19	11.08	11.13	11.18	0.36	11.13	0.06	1.00	-0.007	8.2E-01
Mtr.6988.1.S1_at	9.06	8.74	8.33	8.94	8.77	8.86	8.71	0.36	8.86	0.08	1.02	0.024	5.3E-01
Mtr.43775.1.S1_at	2.25	2.29	2.90	2.22	2.14	2.25	2.48	0.36	2.20	0.06	0.89	-0.170	2.7E-01
Mtr.42925.1.S1_s_at	7.15	6.55	7.21	6.88	6.40	6.48	6.97	0.36	6.59	0.26	0.94	-0.082	2.1E-01
Mtr.18786.1.S1_at	6.42	6.51	7.09	7.17	6.90	6.97	6.68	0.36	7.01	0.14	1.05	0.071	2.1E-01
Mtr.25267.1.S1_at	2.57	2.39	3.09	2.29	2.34	2.36	2.68	0.36	2.33	0.04	0.87	-0.205	1.7E-01
Mtr.3732.1.S1_s_at	9.60	9.68	10.26	9.81	9.77	9.76	9.85	0.36	9.78	0.03	0.99	-0.010	7.6E-01
Mtr.17621.1.S1_x_at	13.90	13.18	13.46	13.73	13.38	13.78	13.51	0.36	13.63	0.21	1.01	0.012	6.6E-01
Mtr.6125.1.S1_s_at	11.76	12.46	12.29	12.04	12.07	12.04	12.17	0.36	12.05	0.02	0.99	-0.014	6.0E-01
Mtr.37225.1.S1_at	11.90	12.22	12.62	12.36	12.51	12.35	12.25	0.36	12.41	0.09	1.01	0.019	4.9E-01
Mtr.16797.1.S1_s_at	12.61	13.03	13.33	13.07	13.09	12.93	12.99	0.36	13.03	0.09	1.00	0.004	8.6E-01
Mtr.31090.1.S1_at	9.17	8.45	8.75	9.04	9.26	9.11	8.79	0.36	9.14	0.11	1.04	0.056	1.9E-01
Mtr.6760.1.S1_at	5.51	4.79	5.09	5.01	5.59	5.02	5.13	0.36	5.21	0.33	1.01	0.021	8.1E-01
Mtr.39202.1.S1_at	2.76	3.38	2.75	2.74	2.70	2.83	2.96	0.36	2.76	0.06	0.93	-0.103	3.9E-01
Mtr.31360.1.S1_at	9.89	10.61	10.33	9.92	10.08	10.02	10.27	0.36	10.01	0.08	0.97	-0.038	2.8E-01
Mtr.6166.1.S1_at	8.88	8.20	8.76	9.02	9.09	9.07	8.61	0.36	9.06	0.03	1.05	0.073	1.0E-01
Mtr.15862.1.S1_at	2.49	3.09	2.44	2.40	2.39	2.43	2.67	0.36	2.41	0.02	0.90	-0.152	2.7E-01
Mtr.39428.1.S1_at	9.00	8.31	8.45	8.44	8.60	8.81	8.59	0.36	8.62	0.18	1.00	0.005	9.0E-01
Mtr.11142.1.S1_at	9.28	8.55	8.91	8.87	8.94	9.03	8.92	0.36	8.95	0.08	1.00	0.005	8.9E-01
Mtr.1856.1.S1_at	9.61	8.90	9.36	9.43	9.54	9.47	9.29	0.36	9.48	0.06	1.02	0.030	4.2E-01
Mtr.20245.1.S1_at	7.74	7.24	7.95	7.81	7.99	7.53	7.65	0.36	7.77	0.23	1.02	0.024	6.3E-01
Mtr.30502.1.S1_at	10.33	9.65	9.75	9.83	9.84	9.96	9.91	0.36	9.87	0.07	1.00	-0.005	8.7E-01
Mtr.35088.1.S1_at	7.68	7.07	7.04	7.54	7.66	7.72	7.27	0.36	7.64	0.09	1.05	0.073	1.6E-01
Mtr.47846.1.S1_at	3.01	3.63	2.98	2.96	2.96	2.94	3.21	0.36	2.95	0.01	0.92	-0.122	2.8E-01
Mtr.40908.1.S1_at	9.25	9.29	9.90	9.41	9.35	9.21	9.48	0.36	9.32	0.10	0.98	-0.024	5.1E-01
Mtr.43415.1.S1_s_at	11.17	10.61	10.49	10.88	10.84	11.12	10.75	0.36	10.95	0.15	1.02	0.026	4.4E-01
Mtr.3244.1.S1_x_at	2.11	2.73	2.10	2.09	2.09	2.09	2.32	0.36	2.09	0.00	0.90	-0.145	3.5E-01
Mtr.14102.1.S1_at	7.90	7.35	8.03	8.15	7.69	8.05	7.76	0.36	7.96	0.24	1.03	0.037	4.7E-01
Mtr.37557.1.S1_at	11.21	10.49	10.83	11.42	11.14	11.25	10.84	0.36	11.27	0.14	1.04	0.056	1.3E-01
Mtr.51257.1.S1_x_at	3.00	3.64	3.02	2.97	2.89	2.95	3.22	0.36	2.94	0.04	0.91	-0.132	2.5E-01
Mtr.10618.1.S1_x_at	8.99	8.29	8.81	8.63	8.57	8.61	8.70	0.36	8.60	0.03	0.99	-0.016	6.7E-01
Mtr.15047.1.S1_s_at	2.67	3.29	2.66	2.65	2.65	2.64	2.87	0.36	2.65	0.00	0.92	-0.119	3.4E-01
Mtr.9110.1.S1_at	12.91	12.19	12.59	12.60	12.61	12.68	12.56	0.36	12.63	0.05	1.01	0.008	7.7E-01

Mtr.12599.1.S1_at	11.40	10.68	11.10	11.18	11.18	11.26	11.06	0.36	11.21	0.05	1.01	0.019	5.3E-01
Mtr.49359.1.S1_at	2.84	3.43	2.77	2.75	2.71	2.73	3.01	0.36	2.73	0.02	0.91	-0.142	2.5E-01
Mtr.9994.1.S1_at	5.73	5.01	5.37	5.72	6.04	5.58	5.37	0.36	5.78	0.23	1.08	0.106	1.7E-01
Mtr.14254.1.S1_at	5.01	4.43	5.10	5.16	5.28	5.21	4.85	0.36	5.22	0.06	1.08	0.106	1.6E-01
Mtr.35927.1.S1_at	4.27	4.04	4.75	4.99	6.55	5.87	4.36	0.36	5.80	0.78	1.33	0.413	4.4E-02
Mtr.29122.1.S1_at	7.13	6.61	7.30	7.17	7.32	7.39	7.01	0.36	7.29	0.11	1.04	0.057	2.7E-01
Mtr.27418.1.S1_at	4.60	3.90	4.40	4.30	4.31	4.31	4.30	0.36	4.31	0.01	1.00	0.001	9.9E-01
Mtr.41854.1.S1_s_at	5.04	5.52	5.75	4.95	5.24	5.22	5.43	0.36	5.14	0.16	0.95	-0.081	2.6E-01
Mtr.49949.1.S1_at	3.24	3.85	3.20	3.20	3.19	3.13	3.43	0.36	3.17	0.04	0.92	-0.113	2.9E-01
Mtr.34192.1.S1_at	2.01	2.63	1.99	2.00	2.00	2.00	2.21	0.36	2.00	0.00	0.90	-0.147	3.6E-01
Mtr.3854.1.S1_at	3.66	4.26	3.61	3.61	3.62	3.62	3.84	0.36	3.62	0.00	0.94	-0.088	3.4E-01
Mtr.52228.1.S1_at	2.98	3.57	2.92	2.91	2.87	2.85	3.15	0.36	2.88	0.03	0.91	-0.134	2.5E-01
Mtr.32557.1.S1_at	2.75	2.08	2.19	2.20	2.30	2.20	2.34	0.36	2.24	0.06	0.95	-0.067	6.4E-01
Mtr.30147.1.S1_at	2.54	3.14	2.49	2.48	2.44	2.46	2.72	0.36	2.46	0.02	0.90	-0.147	2.8E-01
Mtr.36683.1.S1_at	4.41	5.03	4.39	4.35	4.37	4.56	4.61	0.36	4.42	0.12	0.96	-0.059	4.5E-01
Mtr.37489.1.S1_at	9.04	8.39	8.43	8.79	8.96	8.79	8.62	0.36	8.85	0.10	1.03	0.038	3.5E-01
Mtr.19533.1.S1_at	3.43	3.79	3.06	3.24	3.09	3.15	3.43	0.36	3.16	0.07	0.92	-0.116	2.8E-01
Mtr.13932.1.S1_at	4.54	4.06	4.77	4.98	4.68	5.32	4.46	0.36	4.99	0.32	1.12	0.163	1.3E-01
Mtr.39899.1.S1_at	9.75	9.42	10.14	10.26	10.38	10.24	9.77	0.36	10.29	0.08	1.05	0.075	7.2E-02
Mtr.9384.1.S1_at	8.38	7.91	8.62	8.50	8.59	8.50	8.30	0.36	8.53	0.05	1.03	0.038	3.5E-01
Mtr.48584.1.S1_at	3.10	2.57	2.41	2.41	2.40	2.40	2.70	0.36	2.40	0.01	0.89	-0.166	2.3E-01
Mtr.5390.1.S1_at	8.19	7.48	7.70	7.83	7.61	7.68	7.79	0.36	7.71	0.11	0.99	-0.015	7.3E-01
Mtr.8676.1.S1_s_at	9.54	8.83	9.06	9.43	9.77	9.75	9.14	0.36	9.65	0.19	1.06	0.078	9.9E-02
Mtr.50600.1.S1_s_at	2.65	3.26	2.62	2.59	2.59	2.56	2.84	0.36	2.58	0.02	0.91	-0.138	2.8E-01
Mtr.12392.1.S1_at	7.40	7.82	8.13	9.74	10.22	9.72	7.78	0.36	9.90	0.28	1.27	0.346	1.4E-03
Mtr.11580.1.S1_at	11.91	11.20	11.46	11.48	11.30	11.34	11.52	0.36	11.37	0.09	0.99	-0.019	5.3E-01
Mtr.3288.1.S1_at	6.77	6.44	7.17	7.22	7.12	6.94	6.79	0.36	7.09	0.14	1.04	0.062	2.5E-01
Mtr.39501.1.S1_at	10.42	9.88	9.73	10.29	10.20	10.43	10.01	0.36	10.31	0.12	1.03	0.042	2.5E-01
Mtr.6513.1.S1_at	6.72	7.24	7.42	6.81	7.39	6.99	7.13	0.36	7.07	0.29	0.99	-0.013	8.3E-01
Mtr.34877.1.S1_at	3.35	2.78	2.68	2.74	2.69	2.65	2.94	0.36	2.69	0.05	0.92	-0.125	3.1E-01
Mtr.11395.1.S1_at	9.96	9.83	10.51	10.07	10.30	9.94	10.10	0.36	10.10	0.18	1.00	0.000	1.0E+00
Mtr.11182.1.S1_at	12.64	11.98	12.55	12.62	12.47	12.48	12.39	0.36	12.52	0.08	1.01	0.015	5.7E-01
Mtr.27134.1.S1_at	10.38	9.66	9.96	10.03	10.05	10.16	10.00	0.36	10.08	0.07	1.01	0.012	7.2E-01
Mtr.22772.1.S1_at	2.11	2.73	2.09	2.09	2.09	2.09	2.31	0.36	2.09	0.00	0.90	-0.144	3.5E-01
Mtr.34964.1.S1_s_at	8.61	7.89	8.15	8.84	8.87	8.92	8.22	0.36	8.88	0.04	1.08	0.112	3.5E-02
Mtr.42939.1.S1_at	14.80	14.32	15.03	15.23	15.21	15.07	14.72	0.36	15.17	0.09	1.03	0.044	1.0E-01
Mtr.43340.1.S1_at	8.64	9.33	8.81	8.60	8.94	8.89	8.93	0.36	8.81	0.19	0.99	-0.019	6.4E-01
Mtr.29585.1.S1_at	4.25	3.63	3.61	3.52	3.44	3.45	3.83	0.36	3.47	0.04	0.91	-0.142	1.6E-01
Mtr.44610.1.S1_at	4.27	3.99	4.71	4.61	4.58	4.68	4.32	0.36	4.63	0.05	1.07	0.097	2.3E-01
Mtr.38335.1.S1_s_at	8.92	8.27	8.87	8.81	8.94	9.01	8.69	0.36	8.92	0.10	1.03	0.038	3.5E-01
Mtr.29368.1.S1_at	7.76	7.04	7.43	7.87	8.58	8.49	7.41	0.36	8.31	0.39	1.12	0.166	4.2E-02
Mtr.51719.1.S1_at	7.82	7.85	8.46	7.82	7.88	7.68	8.04	0.36	7.79	0.10	0.97	-0.046	3.1E-01
Mtr.6789.1.S1_s_at	6.37	5.65	6.02	6.18	6.36	6.12	6.01	0.36	6.22	0.12	1.03	0.049	4.0E-01
Mtr.17416.1.S1_at	9.79	9.19	9.84	9.92	9.75	9.86	9.60	0.36	9.84	0.09	1.02	0.035	3.3E-01
Mtr.30005.1.S1_at	2.82	2.16	2.75	2.80	2.79	2.55	2.58	0.36	2.71	0.14	1.05	0.072	5.9E-01
Mtr.41729.1.S1_at	8.94	8.52	9.24	9.34	9.42	9.28	8.90	0.36	9.35	0.07	1.05	0.071	1.0E-01
Mtr.51070.1.S1_at	2.87	3.47	2.83	2.80	2.75	2.73	3.05	0.36	2.76	0.03	0.90	-0.146	2.3E-01
Mtr.21184.1.S1_at	10.02	9.30	9.59	9.75	9.75	9.96	9.63	0.36	9.82	0.12	1.02	0.028	4.5E-01
Mtr.13629.1.S1_at	8.69	8.10	8.03	7.57	7.50	7.69	8.27	0.36	7.59	0.10	0.92	-0.124	3.4E-02
Mtr.29376.1.S1_at	8.60	7.88	8.24	9.26	9.81	9.43	8.24	0.36	9.50	0.28	1.15	0.205	8.9E-03
Mtr.14576.1.S1_at	2.84	3.45	2.82	2.81	2.74	2.81	3.03	0.36	2.79	0.04	0.92	-0.122	3.1E-01
Mtr.43997.1.S1_at	9.05	8.50	9.19	8.99	9.19	9.03	8.91	0.36	9.07	0.10	1.02	0.026	5.0E-01
Mtr.24971.1.S1_at	2.85	3.46	2.82	2.80	2.78	2.77	3.04	0.36	2.78	0.02	0.91	-0.129	2.8E-01
Mtr.47512.1.S1_at	8.01	7.66	8.39	7.96	8.12	8.05	8.02	0.36	8.04	0.08	1.00	0.004	9.2E-01
Mtr.32956.1.S1_at	5.49	6.20	5.70	5.66	5.62	5.84	5.80	0.36	5.71	0.12	0.98	-0.023	7.0E-01
Mtr.42768.1.S1_at	9.62	10.35	9.95	9.94	10.15	9.88	9.97	0.36	9.99	0.14	1.00	0.003	9.4E-01
Mtr.41348.1.S1_at	9.89	9.19	9.70	9.70	9.59	9.79	9.59	0.36	9.69	0.10	1.01	0.015	6.6E-01
Mtr.35175.1.S1_at	7.65	6.98	7.57	7.80	7.54	7.40	7.40	0.36	7.58	0.21	1.02	0.034	5.0E-01
Mtr.21561.1.S1_s_at	3.39	3.91	3.22	3.18	3.21	3.20	3.50	0.36	3.20	0.02	0.91	-0.132	2.2E-01
Mtr.18111.1.S1_at	8.15	8.28	8.83	8.45	8.47	8.37	8.42	0.36	8.43	0.05	1.00	0.002	9.7E-01
Mtr.8434.1.S1_at	2.69	3.30	2.66	2.64	2.62	2.65	2.88	0.36	2.64	0.01	0.91	-0.130	3.0E-01
Mtr.27289.1.S1_at	5.93	5.30	5.30	5.70	5.83	5.59	5.51	0.36	5.71	0.12	1.04	0.051	4.2E-01
Mtr.30156.1.S1_at	11.87	11.17	11.67	11.74	11.40	11.78	11.57	0.36	11.64	0.21	1.01	0.009	7.8E-01
Mtr.36079.1.S1_at	9.32	10.04	9.75	9.36	9.76	9.24	9.70	0.36	9.45	0.27	0.97	-0.038	3.9E-01
Mtr.28756.1.S1_at	7.93	7.23	7.76	8.00	7.90	7.99	7.64	0.36	7.96	0.06	1.04	0.059	2.0E-01
Mtr.6553.1.S1_s_at	7.00	6.29	6.50	6.42	6.19	6.39	6.60	0.36	6.33	0.12	0.96	-0.059	3.0E-01
Mtr.31553.1.S1_at	2.86	3.49	2.88	2.80	2.78	2.83	3.08	0.36	2.80	0.02	0.91	-0.135	2.6E-01
Mtr.39877.1.S1_at	7.93	7.62	8.34	8.27	8.23	8.11	7.96	0.36	8.20	0.08	1.03	0.044	3.2E-01
Mtr.41621.1.S1_at	8.34	7.70	8.31	8.13	8.04	8.12	8.12	0.36	8.10	0.05	1.00	-0.003	9.4E-01
Mtr.29645.1.S1_at	2.75	3.36	2.71	2.70	2.65	2.68	2.94	0.36	2.68	0.02	0.91	-0.136	2.8E-01
Mtr.44211.1.S1_at	9.77	9.05	9.40	10.95	11.64	11.48	9.41	0.36	11.36	0.36	1.21	0.272	2.7E-03
Mtr.38351.1.S1_s_at	8.67	8.06	8.71	8.64	8.72	8.66	8.48	0.36	8.67	0.04	1.02	0.032	4.2E-01
Mtr.52275.1.S1_at	5.77	5.11	5.18	4.95	5.11	5.20	5.35	0.36	5.09	0.13	0.95	-0.072	3.0E-01
Mtr.32324.1.S1_at	7.99	8.58	7.91	7.51	8.13	7.53	8.16	0.36	7.72	0.35	0.95	-0.080	2.0E-01
Mtr.32056.1.S1_at	3.09	2.44	2.49	2.37	2.79	2.38	2.67	0.36	2.51	0.24	0.94	-0.089	5.6E-01
Mtr.31164.1.S1_at	6.66	6.14	6.83	6.84	7.19	6.81	6.54	0.36	6.94	0.21	1.06	0.086	1.7E-01
Mtr.31380.1.S1_at	2.83	3.40	2.73	2.90	2.78	2.97	2.99	0.36	2.88	0.10	0.96	-0.052	6.5E-01
Mtr.35630.1.S1_at	3.31	3.88	3.22	3.36	3.20	3.25	3.47	0.36	3.27	0.08	0.94	-0.084	4.1E-01
Mtr.8215.1.S1_at	2.54	3.26	2.86	2.51	2.49	2.47	2.89	0.36	2.49	0.02	0.86	-0.213	1.3E-01
Mtr.35329.1.S1_s_at	9.25	8.65	9.30	9.28	9.19	9.38	9.07	0.36	9.28	0.09	1.02	0.034	3.7E-01
Mtr.44605.1.S1_at	9.05	9.43	9.77	9.30	9.56	9.35	9.42	0.36	9.40	0.14	1.00	-0.002	9.5E-01
Mtr.39423.1.S1_at	12.40	11.77	12.40	12.42	12.54	12.53	12.19	0.36	12.50	0.06	1.02	0.036	2.2E-01
Mtr.1846.1.S1_at	9.05	9.58	8.89	8.98	8.81	8.94	9.17	0.36	8.91	0.09	0.97	-0.042	2.9E-01
Mtr.23387.1.S1_at	12.17	12.02	11.49	12.34	12.32	12.55	11.89	0.36	12.40	0.13	1.04	0.060	8.4E-02
Mtr.13288.1.S1_at	5.58	4.94	4.97	5.02	5.01	5.16	5.16	0.36	5.06	0.09	0.98	-0.029	6.6E-01
Mtr.20788.1.S1_at	2.19	2.81	2.18	2.17	2.17	2.17	2.39	0.36	2.17	0.00	0.91	-0.140	3.5E-01
Mtr.29370.1.S1_at	4.04	4.47	3.75	3.84	3.79	4.05	4.09	0.36	3.89	0.14	0.95	-0.071	4.3E-01
Mtr.32778.1.S1_at	8.39	7.71	8.26	8.33	8.34	8.33	8.12	0.36	8.33	0.01	1.03	0.037	3.7E-01
Mtr.10031.1.S1_at	9.22	9.26	9.87	9.52	9.70	9.27	9.45	0.36	9.50	0.21	1.01	0.007	8.5E-01
Mtr.15048.1.S1_s_at	8.69	8.33	9.05	8.63	8.64	8.58	8.69	0.36	8.62	0.03	0.99	-0.013	7.3E-01
Mtr.12464.1.S1_s_at	12.53	11.82	12.08	12.13	11.93	12.21	12.14	0.36	12.09	0.14	1.00	-0.007	8.2E-01
Mtr.39039.1.S1_at	11.97	12.57	12.61	12.14	12.26	12.22	12.39	0.36	12.21	0.06	0.99	-0.021	4.5E-01
Mtr.18699.1.S1_at	3.82	3.18	3.79	3.89	3.70	3.41	3.60	0.36	3.67	0.24	1.02	0.027	8.0E-01
Mtr.35569.1.S1_at	10.84	11.17	11.56	11.21	11.34	11.36	11.19	0.36	11.30	0.08	1.01	0.015	6.2E-01
Mtr.23768.1.S1_at	2.48	3.09	2.46	2.45	2.45	2.45	2.68	0.36	2.45	0.00	0.92	-0.127	3.4E-01
Mtr.49076.1.S1_at	5.17	4.46	4.68	4.68	4.88	4.82	4.77	0.36	4.79	0.10	1.00	0.007	9.2E-01
Mtr.15392.1.S1_at	13.80	13.12	13.26	13.36	13.44	13.49	13.40	0.36	13.43	0.07	1.00	0.004	8.8E-01
Mtr.50065.1.S1_at	2.86	3.43	2.77	2.80	2.85	2.76	3.02	0.36	2.80	0.04	0.93	-0.109	3.5E-01
Mtr.14474.1.S1_at	6.57	7.22	7.16	7.43	7.47	7.56	6.98	0.36	7.49	0.07	1.07	0.101	7.6E-02
Mtr.28018.1.S1_at	9.20	9.92	9.56	9.59	9.59	9.50	9.56	0.36	9.56	0.05	1.00	0.000	9.9E-01
Mtr.10424.1.S1_at	11.13	10.70	11.42	12.22	12.52	12.42	11.08	0.36	12.38	0.15	1.12	0.160	4.6E-03
Mtr.4244.1.S1_at	3.89	3.24	3.30	3.14	4.81	4.41	3.48	0.36	4.12	0.87	1.18	0.244	3.0E-01
Mtr.2992.1.S1_at	5.65	5.03	5.67	6.17	6.31	6.25	5.45	0.36	6.24	0.07	1.15	0.196	2.0E-02
Mtr.45556.1.S1_at	9.61	8.91	9.11	9.30	9.11	9.37	9.21	0.36	9.26	0.14	1.01	0.008	8.3E-01
Mtr.41201.1.S1_at	9.52	9.72	10.22	9.83	9.94	9.72	9.82	0.36	9.83	0.11	1.00	0.001	9.7E-01
Mtr.44412.1.S1_at	11.34	11.75	12.06	11.22	11.36	11.49	11.72	0.36	11.36	0.13	0.97	-0.045	1.8E-01
Mtr.31449.1.S1_x_at	3.17	3.74	3.08	3.07	3.02	3.07	3.33	0.36	3.05	0.03	0.92	-0.128	2.5E-01
Mtr.1397.1.S1_at	10.07	9.57	10.26	10.01	9.93	9.70	9.97	0.36	9.88	0.16	0.99	-0.013	7.2E-01
Mtr.14362.1.S1_at	3.77	3.85	4.43	4.28	3.66	3.63	4.02	0.36	3.86	0.37	0.96	-0.058	6.2E-01
Mtr.22209.1.S1_at	3.60	4.08	3.37	3.49	3.48	3.48	3.69	0.36	3.49	0.00	0.95	-0.081	3.9E-01
Mtr.37546.1.S1_at	2.91	3.52	2.88	2.88	2.88	2.91	3.10	0.36	2.89	0.01	0.93	-0.102	3.7E-01
Mtr.8433.1.S1_s_at	14.75	14.23	14.05	14.34	14.53	14.35	14.34	0.36	14.41	0.11	1.00	0.006	7.8E-01
Mtr.42212.1.S1_at	7.32	6.86	7.57	7.60	7.47	7.64	7.25	0.36	7.57	0.09	1.04	0.062	2.1E-01
Mtr.11853.1.S1_at	8.43	7.76	8.33	8.41	8.44	8.18	8.17	0.36	8.34	0.14	1.02	0.030	4.9E-01
Mtr.52069.1.S1_at	3.02	3.61	2.95	2.93	2.89	2.85	3.19	0.36	2.89	0.04	0.91	-0.143	2.2E-01
Mtr.20491.1.S1_at	6.21	6.67	5.96	6.17	6.46	6.46	6.28	0.36	6.37	0.17	1.01	0.019	7.3E-01
Mtr.48575.1.S1_at	11.55	12.25	12.05	11.70	11.70	11.62	11.95	0.36	11.67	0.05	0.98	-0.034	2.6E-01
Mtr.35150.1.S1_at	6.17	5.55	6.18	6.36	6.41	6.37	5.97	0.36	6.38	0.03	1.07	0.097	1.2E-01
Mtr.35802.1.S1_at	11.34	11.53	12.04	12.03	12.25	12.16	11.64	0.36	12.15	0.12	1.04	0.062	8.0E-02
Mtr.39471.1.S1_at	8.60	9.01	9.32	9.15	9.20	8.87	8.98	0.36	9.08	0.18	1.01	0.016	6.9E-01
Mtr.19025.1.S1_s_at	7.03	6.31	6.71	6.82	6.82	6.90	6.68	0.36	6.85	0.04	1.02	0.035	4.8E-01
Mtr.22459.1.S1_at	11.45	10.75	11.27	11.02	11.16	11.14	11.16	0.36	11.11	0.07	1.00	-0.007	8.2E-01
Mtr.23926.1.S1_at	6.68	6.01	6.58	6.64	6.40	6.62	6.42	0.36	6.55	0.14	1.02	0.029	5.9E-01
Mtr.38516.1.S1_at	10.71	10.10	10.73	10.57	10.47	10.55	10.51	0.36	10.53	0.05	1.00	0.003	9.3E-01
Mtr.16863.1.S1_at	7.88	7.16	7.54	8.08	7.29	8.06	7.53	0.36	7.81	0.45	1.04	0.054	4.4E-01
Mtr.2429.1.S1_at	2.29	2.91	2.27	2.27	2.27	2.26	2.49	0.36	2.26	0.01	0.91	-0.138	3.4E-01
Mtr.50616.1.S1_at	9.62	10.26	10.22	9.92	10.01	9.99	10.03	0.36	9.97	0.04	0.99	-0.008	8.0E-01
Mtr.43678.1.S1_at	10.17	10.30	10.85	10.13	9.84	10.06	10.44	0.36	10.01	0.15	0.96	-0.061	1.3E-01
Mtr.11193.1.S1_at	10.11	9.43	9.98	10.11	10.13	9.96	9.84	0.36	10.07	0.09	1.02	0.033	3.5E-01
Mtr.39902.1.S1_at	6.51	6.53	7.14	6.82	6.87	6.65	6.73	0.36	6.78	0.12	1.01	0.012	8.1E-01
Mtr.23357.1.S1_at	8.87	8.22	8.81	8.94	9.25	9.08	8.64	0.36	9.09	0.16	1.05	0.074	1.2E-01

Mtr.48541.1.S1_at	2.89	3.49	2.84	2.83	2.80	2.75	3.07	0.36	2.79	0.04	0.91	-0.138	2.5E-01
Mtr.34341.1.S1_at	3.30	3.32	3.93	3.91	4.87	3.93	3.51	0.36	4.24	0.55	1.21	0.270	1.3E-01
Mtr.31603.1.S1_at	10.55	9.91	10.51	10.29	10.59	10.34	10.32	0.36	10.41	0.16	1.01	0.012	7.3E-01
Mtr.38863.1.S1_at	10.64	10.86	11.34	11.02	11.04	10.89	10.95	0.36	10.98	0.08	1.00	0.004	8.8E-01
Mtr.10492.1.S1_at	7.81	8.39	7.74	7.77	7.47	7.94	7.98	0.36	7.73	0.24	0.97	-0.046	3.7E-01
Mtr.14736.1.S1_s_at	7.63	8.15	8.31	7.82	7.70	7.68	8.03	0.36	7.74	0.08	0.96	-0.054	2.4E-01
Mtr.4302.1.S1_x_at	9.53	10.11	10.19	9.58	9.51	9.50	9.94	0.36	9.53	0.05	0.96	-0.062	1.2E-01
Mtr.49515.1.S1_at	8.95	8.26	8.78	8.69	8.82	8.65	8.66	0.36	8.72	0.08	1.01	0.010	7.9E-01
Mtr.16750.1.S1_at	5.14	5.75	5.11	5.12	5.12	5.12	5.34	0.36	5.12	0.00	0.96	-0.059	3.6E-01
Mtr.9109.1.S1_at	9.04	8.33	8.56	8.76	8.78	8.85	8.64	0.36	8.80	0.05	1.02	0.026	5.0E-01
Mtr.31621.1.S1_at	7.54	7.12	7.84	7.48	7.76	7.85	7.50	0.36	7.70	0.19	1.03	0.037	4.5E-01
Mtr.31183.1.S1_at	3.71	4.13	3.42	3.27	4.02	3.53	3.75	0.36	3.61	0.38	0.96	-0.057	6.5E-01
Mtr.46721.1.S1_s_at	8.12	7.91	8.61	8.10	8.43	7.97	8.21	0.36	8.17	0.23	0.99	-0.008	8.6E-01
Mtr.10071.1.S1_at	11.08	10.63	11.34	10.10	9.68	10.20	11.01	0.36	9.99	0.28	0.91	-0.141	1.8E-02
Mtr.47410.1.S1_at	3.69	4.31	3.68	3.60	3.60	3.68	3.89	0.36	3.63	0.04	0.93	-0.103	2.7E-01
Mtr.29767.1.S1_at	3.63	4.31	3.77	3.61	3.37	3.67	3.90	0.36	3.55	0.16	0.91	-0.137	1.9E-01
Mtr.5683.1.S1_at	5.10	4.65	5.36	5.09	5.12	5.03	5.03	0.36	5.08	0.04	1.01	0.014	8.3E-01
Mtr.49442.1.S1_at	6.09	5.46	6.09	6.26	6.51	6.07	5.88	0.36	6.28	0.22	1.07	0.095	1.8E-01
Mtr.43159.1.S1_at	12.82	12.19	12.80	12.78	12.98	12.97	12.61	0.36	12.91	0.11	1.02	0.034	2.4E-01
Mtr.35305.1.S1_at	6.10	6.02	6.68	5.99	6.37	5.95	6.27	0.36	6.10	0.24	0.97	-0.038	5.5E-01
Mtr.16697.1.S1_at	7.90	7.74	8.42	7.98	7.93	8.01	8.02	0.36	7.97	0.04	0.99	-0.008	8.3E-01
Mtr.9398.1.S1_at	8.45	7.76	8.26	8.50	8.30	8.52	8.16	0.36	8.44	0.12	1.03	0.048	2.7E-01
Mtr.22015.1.S1_at	7.43	7.05	7.76	7.38	7.35	7.38	7.41	0.36	7.37	0.02	0.99	-0.009	8.4E-01
Mtr.42725.1.S1_at	10.82	10.27	10.14	10.74	10.61	10.69	10.41	0.36	10.68	0.06	1.03	0.037	2.7E-01
Mtr.13066.1.S1_at	9.62	10.34	10.04	9.82	9.95	9.77	10.00	0.36	9.85	0.10	0.98	-0.022	5.1E-01
Mtr.19327.1.S1_s_at	8.00	7.32	7.87	8.00	7.93	7.96	7.73	0.36	7.97	0.04	1.03	0.044	3.2E-01
Mtr.49342.1.S1_at	2.93	3.53	2.89	2.88	2.89	2.77	3.12	0.36	2.85	0.06	0.91	-0.131	2.7E-01
Mtr.42694.1.S1_at	7.69	8.21	8.38	7.94	8.16	8.37	8.10	0.36	8.16	0.22	1.01	0.011	8.2E-01
Mtr.49829.1.S1_at	6.95	6.25	6.49	7.36	7.38	7.32	6.56	0.36	7.36	0.03	1.12	0.164	1.9E-02
Mtr.26923.1.S1_at	9.18	9.13	9.77	9.29	9.53	9.15	9.36	0.36	9.32	0.19	1.00	-0.006	8.8E-01
Mtr.47859.1.S1_at	2.92	3.55	2.92	2.89	2.90	2.82	3.13	0.36	2.87	0.04	0.92	-0.125	2.8E-01
Mtr.51633.1.S1_at	10.25	9.54	9.96	9.90	9.99	9.88	9.92	0.36	9.92	0.06	1.00	0.001	9.8E-01
Mtr.2370.1.S1_at	4.10	3.47	4.07	3.55	3.53	3.27	3.88	0.36	3.45	0.15	0.89	-0.170	1.3E-01
Mtr.25738.1.S1_s_at	2.61	3.22	2.59	2.57	2.55	2.55	2.81	0.36	2.56	0.01	0.91	-0.134	2.9E-01
Mtr.37785.1.S1_at	12.49	12.27	12.98	12.70	12.83	12.60	12.58	0.36	12.71	0.12	1.01	0.015	5.9E-01
Mtr.26959.1.S1_at	7.55	7.04	7.72	7.70	7.87	7.70	7.44	0.36	7.76	0.09	1.04	0.061	2.1E-01
Mtr.17112.1.S1_at	4.05	3.54	4.23	3.68	4.70	3.93	3.94	0.36	4.11	0.53	1.04	0.059	6.8E-01
Mtr.32552.1.S1_at	10.30	9.62	9.77	9.97	9.73	9.93	9.90	0.36	9.88	0.13	1.00	-0.003	9.2E-01
Mtr.42472.1.S1_at	6.65	7.32	7.20	7.70	8.04	8.24	7.06	0.36	8.00	0.27	1.13	0.180	2.3E-02
Mtr.43365.1.S1_at	11.33	10.62	11.01	10.98	10.97	11.04	10.99	0.36	11.00	0.04	1.00	0.001	9.6E-01
Mtr.11399.1.S1_at	11.88	11.43	11.17	11.73	11.47	11.73	11.49	0.36	11.64	0.15	1.01	0.019	5.3E-01
Mtr.49316.1.S1_x_at	3.17	3.77	3.14	3.09	3.02	3.11	3.36	0.36	3.07	0.05	0.91	-0.129	2.4E-01
Mtr.10062.1.S1_at	7.94	8.01	8.59	8.11	8.34	8.06	8.18	0.36	8.17	0.15	1.00	-0.001	9.7E-01
Mtr.21317.1.S1_at	11.33	10.62	10.88	11.06	10.97	11.10	10.94	0.36	11.04	0.06	1.01	0.013	6.5E-01
Mtr.22081.1.S1_at	3.35	3.96	3.33	3.33	3.37	3.29	3.55	0.36	3.33	0.04	0.94	-0.092	3.5E-01
Mtr.40761.1.S1_at	9.13	9.51	9.84	9.47	9.31	9.09	9.49	0.36	9.29	0.19	0.98	-0.031	4.4E-01
Mtr.10840.1.S1_at	11.94	11.22	11.55	11.86	11.92	11.92	11.57	0.36	11.90	0.04	1.03	0.040	1.9E-01
Mtr.7531.1.S1_at	9.01	8.76	9.46	9.01	9.28	9.02	9.08	0.36	9.10	0.15	1.00	0.005	9.0E-01
Mtr.44709.1.S1_at	3.20	3.89	3.38	3.34	3.36	3.37	3.49	0.36	3.36	0.01	0.96	-0.056	5.6E-01
Mtr.17422.1.S1_at	5.17	4.71	5.41	5.77	5.21	5.48	5.10	0.36	5.49	0.28	1.08	0.107	2.1E-01
Mtr.50064.1.S1_s_at	9.52	9.06	9.77	9.48	9.56	9.47	9.45	0.36	9.50	0.05	1.01	0.008	8.2E-01
Mtr.20187.1.S1_x_at	14.74	14.03	14.42	14.35	14.23	14.31	14.40	0.36	14.30	0.06	0.99	-0.010	6.6E-01
Mtr.771.1.S1_at	14.29	13.59	13.81	14.04	13.73	14.03	13.90	0.36	13.93	0.18	1.00	0.003	8.9E-01
Mtr.9868.1.S1_at	9.42	8.92	9.61	9.46	9.41	9.27	9.31	0.36	9.38	0.10	1.01	0.010	7.7E-01
Mtr.8756.1.S1_at	10.12	10.84	10.46	10.20	10.59	10.38	10.47	0.36	10.39	0.19	0.99	-0.012	7.4E-01
Mtr.13283.1.S1_at	9.06	9.36	9.77	9.16	9.76	9.34	9.40	0.36	9.42	0.30	1.00	0.004	9.3E-01
Mtr.40974.1.S1_at	12.11	12.25	12.79	12.66	12.64	12.53	12.38	0.36	12.61	0.07	1.02	0.026	3.4E-01
Mtr.44767.1.S1_at	9.00	8.34	8.91	8.85	8.85	8.98	8.75	0.36	8.89	0.08	1.02	0.024	5.3E-01
Mtr.14354.1.S1_at	10.34	9.68	9.78	10.10	10.71	10.49	9.93	0.36	10.44	0.31	1.05	0.071	1.4E-01
Mtr.32031.1.S1_s_at	10.33	9.77	10.43	10.46	10.34	10.43	10.18	0.36	10.41	0.06	1.02	0.033	3.3E-01
Mtr.18572.1.S1_at	12.19	11.47	11.83	12.02	11.83	11.84	11.83	0.36	11.90	0.11	1.01	0.008	7.8E-01
Mtr.33149.1.S1_at	9.55	8.84	9.24	9.42	9.44	9.28	9.21	0.36	9.38	0.09	1.02	0.026	4.7E-01
Mtr.4375.1.S1_at	11.34	10.84	10.65	11.83	11.96	11.80	10.94	0.36	11.86	0.09	1.08	0.117	1.2E-02
Mtr.13110.1.S1_at	8.68	7.98	8.49	8.49	8.37	8.30	8.38	0.36	8.39	0.09	1.00	0.001	9.9E-01
Mtr.20564.1.S1_at	8.37	7.74	8.36	8.40	8.28	8.36	8.16	0.36	8.35	0.06	1.02	0.034	4.1E-01
Mtr.39719.1.S1_at	9.30	8.89	9.60	9.29	9.29	9.11	9.26	0.36	9.23	0.11	1.00	-0.005	8.9E-01
Mtr.15258.1.S1_at	12.27	12.90	12.88	12.43	12.54	12.39	12.69	0.36	12.45	0.08	0.98	-0.027	3.3E-01
Mtr.3661.1.S1_at	10.00	9.43	10.09	10.01	10.10	9.67	9.84	0.36	9.93	0.22	1.01	0.012	7.5E-01
Mtr.1957.1.S1_at	2.88	3.12	3.59	3.06	3.40	2.97	3.20	0.36	3.14	0.23	0.98	-0.026	8.3E-01
Mtr.11867.1.S1_at	8.61	7.93	8.07	8.06	8.12	8.02	8.20	0.36	8.06	0.05	0.98	-0.024	5.4E-01
Mtr.43001.1.S1_s_at	11.65	11.05	11.70	11.61	11.60	11.37	11.47	0.36	11.52	0.14	1.01	0.007	8.0E-01
Mtr.25763.1.S1_s_at	2.44	3.05	2.42	2.41	2.41	2.39	2.64	0.36	2.41	0.01	0.91	-0.133	3.2E-01
Mtr.9491.1.S1_s_at	8.70	8.16	8.83	8.80	8.50	8.70	8.56	0.36	8.67	0.15	1.01	0.017	6.7E-01
Mtr.49014.1.S1_at	10.93	10.72	11.42	11.11	11.00	11.09	11.02	0.36	11.06	0.06	1.00	0.006	8.5E-01
Mtr.40499.1.S1_at	7.81	7.15	7.72	7.46	7.50	7.53	7.56	0.36	7.50	0.04	0.99	-0.012	7.7E-01
Mtr.5407.1.S1_at	3.31	2.73	2.66	2.66	2.66	2.66	2.90	0.36	2.66	0.00	0.92	-0.125	3.1E-01
Mtr.41679.1.S1_at	9.05	8.34	8.77	8.78	8.89	8.79	8.72	0.36	8.82	0.06	1.01	0.017	6.4E-01
Mtr.10152.1.S1_at	7.96	7.46	8.16	8.16	7.93	7.95	7.86	0.36	8.02	0.13	1.02	0.028	5.3E-01
Mtr.33172.1.S1_at	8.17	7.45	7.79	8.14	8.23	8.09	7.80	0.36	8.15	0.07	1.04	0.063	1.7E-01
Mtr.4577.1.S1_at	10.93	10.26	10.80	10.71	10.88	10.65	10.66	0.36	10.75	0.12	1.01	0.012	7.2E-01
Mtr.14512.1.S1_x_at	2.94	3.55	2.93	2.93	2.95	2.83	3.14	0.36	2.90	0.07	0.92	-0.114	3.2E-01
Mtr.49249.1.S1_s_at	4.25	4.38	4.92	4.43	4.95	4.32	4.52	0.36	4.57	0.33	1.01	0.016	8.7E-01
Mtr.27308.1.S1_at	2.79	3.37	2.71	2.69	2.64	2.63	2.96	0.36	2.65	0.03	0.90	-0.157	2.1E-01
Mtr.36665.1.S1_at	2.08	2.70	2.08	2.08	2.08	2.08	2.29	0.36	2.08	0.00	0.91	-0.139	3.7E-01
Mtr.30408.1.S1_at	8.89	9.54	8.96	8.87	8.87	8.80	9.13	0.36	8.85	0.04	0.97	-0.045	2.5E-01
Mtr.36914.1.S1_at	2.56	3.17	2.55	2.54	2.54	2.53	2.76	0.36	2.54	0.01	0.92	-0.120	3.5E-01
Mtr.37383.1.S1_at	13.33	12.62	13.07	13.08	13.26	13.19	13.01	0.36	13.18	0.09	1.01	0.019	4.6E-01
Mtr.34191.1.S1_at	11.90	11.22	11.39	11.51	11.56	11.68	11.50	0.36	11.58	0.09	1.01	0.010	7.2E-01
Mtr.39644.1.S1_at	8.12	7.48	8.07	7.83	8.14	7.67	7.89	0.36	7.88	0.24	1.00	-0.001	9.8E-01
Mtr.37445.1.S1_at	14.01	13.39	13.39	13.70	13.52	13.74	13.60	0.36	13.65	0.11	1.00	0.006	8.0E-01
Mtr.42727.1.S1_at	10.06	9.35	9.62	9.77	9.59	9.80	9.68	0.36	9.72	0.11	1.00	0.006	8.6E-01
Mtr.10907.1.S1_at	10.05	9.50	9.38	9.87	9.58	9.91	9.64	0.36	9.79	0.18	1.02	0.022	5.6E-01
Mtr.42162.1.S1_at	8.49	8.20	8.91	8.76	8.77	8.60	8.53	0.36	8.71	0.09	1.02	0.030	4.5E-01
Mtr.44421.1.S1_at	10.30	9.68	10.30	10.13	10.12	10.22	10.09	0.36	10.15	0.05	1.01	0.009	7.8E-01
Mtr.48831.1.S1_at	7.19	7.78	7.83	8.53	9.26	8.59	7.60	0.36	8.79	0.40	1.16	0.210	1.9E-02
Mtr.39469.1.S1_at	7.29	6.58	6.98	7.14	7.32	7.08	6.95	0.36	7.18	0.13	1.03	0.047	3.5E-01
Mtr.7367.1.S1_at	2.79	3.13	3.50	3.65	4.62	4.78	3.14	0.36	4.35	0.61	1.38	0.469	4.2E-02
Mtr.12410.1.S1_at	10.59	9.88	10.28	10.26	10.05	10.31	10.25	0.36	10.21	0.14	1.00	-0.006	8.5E-01
Mtr.52088.1.S1_at	10.40	10.77	11.12	10.70	10.77	10.81	10.76	0.36	10.76	0.05	1.00	-0.001	9.8E-01
Mtr.8877.1.S1_at	13.46	14.09	14.07	13.58	13.79	13.60	13.87	0.36	13.66	0.11	0.98	-0.023	3.8E-01
Mtr.39020.1.S1_at	8.82	8.20	8.81	9.06	9.05	9.06	8.61	0.36	9.06	0.01	1.05	0.073	9.6E-02
Mtr.8353.1.S1_at	3.20	2.64	2.54	2.54	2.54	2.98	2.79	0.36	2.68	0.26	0.96	-0.058	6.8E-01
Mtr.31287.1.S1_at	10.33	9.63	10.10	10.18	10.24	10.15	10.02	0.36	10.19	0.05	1.02	0.025	4.5E-01
Mtr.49520.1.S1_at	3.35	2.68	3.23	3.23	3.57	3.11	3.09	0.36	3.30	0.24	1.07	0.098	4.3E-01
Mtr.26218.1.S1_s_at	11.01	10.68	10.29	10.43	10.60	10.65	10.66	0.36	10.56	0.11	0.99	-0.013	6.8E-01
Mtr.27704.1.S1_at	9.53	9.29	10.00	9.73	9.69	9.62	9.61	0.36	9.68	0.05	1.01	0.011	7.4E-01
Mtr.14072.1.S1_at	10.95	10.52	11.23	11.08	10.93	10.82	10.90	0.36	10.95	0.13	1.00	0.006	8.5E-01
Mtr.23127.1.S1_at	6.30	5.59	6.02	5.71	6.35	5.74	5.97	0.36	5.94	0.36	0.99	-0.008	9.1E-01
Mtr.43943.1.S1_at	9.14	8.43	8.80	9.11	9.07	9.26	8.79	0.36	9.15	0.10	1.04	0.057	1.7E-01
Mtr.26450.1.S1_at	2.68	3.27	2.64	2.72	2.56	2.95	2.86	0.36	2.74	0.20	0.96	-0.063	6.3E-01
Mtr.8959.1.S1_at	1.97	2.66	2.46	2.37	3.25	2.85	2.37	0.36	2.82	0.44	1.19	0.255	2.3E-01
Mtr.18272.1.S1_at	5.53	5.80	6.24	5.96	6.14	5.78	5.86	0.36	5.96	0.18	1.02	0.025	6.8E-01
Mtr.18381.1.S1_at	2.81	3.39	2.74	2.80	2.73	3.15	2.98	0.36	2.89	0.22	0.97	-0.044	7.3E-01
Mtr.49178.1.S1_at	2.96	3.57	2.94	2.91	3.05	2.84	3.16	0.36	2.93	0.10	0.93	-0.106	3.6E-01
Mtr.45481.1.S1_at	9.52	9.03	8.83	9.25	9.29	9.42	9.13	0.36	9.32	0.09	1.02	0.031	4.1E-01
Mtr.49096.1.S1_at	3.21	2.68	2.53	2.51	2.54	2.49	2.81	0.36	2.51	0.02	0.90	-0.159	2.3E-01
Mtr.8036.1.S1_at	2.55	3.16	2.53	2.52	2.52	2.51	2.75	0.36	2.52	0.01	0.92	-0.127	3.2E-01
Mtr.17635.1.S1_at	3.20	3.76	3.10	3.16	3.15	3.14	3.35	0.36	3.15	0.01	0.94	-0.089	3.9E-01
Mtr.49584.1.S1_at	2.85	3.46	2.83	2.80	2.74	2.83	3.04	0.36	2.79	0.05	0.92	-0.125	2.9E-01
Mtr.49588.1.S1_at	2.63	3.24	2.61	2.60	2.59	2.56	2.82	0.36	2.58	0.02	0.91	-0.128	3.1E-01
Mtr.27243.1.S1_at	4.63	3.92	4.36	3.39	3.92	4.07	4.30	0.36	3.79	0.36	0.88	-0.182	1.5E-01
Mtr.42974.1.S1_at	10.03	9.59	10.30	10.06	10.21	10.16	9.97	0.36	10.15	0.07	1.02	0.025	4.6E-01
Mtr.40922.1.S1_at	8.71	8.97	9.42	9.09	9.07	8.98	9.04	0.36	9.05	0.06	1.00	0.002	9.5E-01
Mtr.11199.1.S1_at	9.07	8.43	9.03	8.93	8.81	8.76	8.84	0.36	8.84	0.09	1.00	-0.001	9.8E-01
Mtr.15666.1.S1_at	4.12	3.47	3.53	3.25	3.14	3.15	3.71	0.36	3.18	0.06	0.86	-0.221	6.5E-02
Mtr.19557.1.S1_at	2.81	3.41	2.77	2.74	2.77	2.74	3.00	0.36	2.75	0.02	0.92	-0.125	3.0E-01
Mtr.42463.1.S1_at	10.63	10.05	10.71	10.48	10.72	10.49	10.46	0.36	10.56	0.14	1.01	0.014	6.7E-01
Mtr.14189.1.S1_s_at	9.79	9.13	9.22	9.76	9.68	9.85	9.38	0.36	9.76	0.08	1.04	0.058	1.4E-01
Mtr.5209.1.S1_at	3.30	2.69	3.32	3.92	4.06	3.99	3.10	0.36	3.99	0.07	1.28	0.361	1.4E-02
Mtr.43878.1.S1_at	5.77	5.39	6.11	6.57	7.71	6.14	5.76	0.36	6.80	0.81	1.18	0.241	1.1E-01
Mtr.19669.1.S1_at	2.60	2.63	3.23	2.59	2.58	2.59	2.82	0.36	2.59	0.00	0.92	-0.126	3.2E-01

Mtr.30356.1.S1_at	2.92	2.29	2.89	2.64	2.96	2.83	2.70	0.36	2.81	0.16	1.04	0.059	6.5E-01
Mtr.2896.1.S1_at	4.01	4.09	4.67	4.10	4.11	3.85	4.26	0.36	4.02	0.15	0.94	-0.082	3.5E-01
Mtr.28566.1.S1_at	4.28	3.69	4.34	4.34	5.25	4.64	4.11	0.36	4.75	0.46	1.16	0.209	1.3E-01
Mtr.25301.1.S1_at	5.79	5.42	5.08	6.58	5.66	5.94	5.43	0.36	6.06	0.47	1.12	0.157	1.4E-01
Mtr.35770.1.S1_at	10.38	10.14	10.85	10.34	10.28	10.41	10.46	0.36	10.34	0.07	0.99	-0.016	6.2E-01
Mtr.4790.1.S1_at	10.90	10.24	10.34	10.72	10.59	10.62	10.49	0.36	10.64	0.07	1.01	0.020	5.2E-01
Mtr.45270.1.S1_at	10.67	10.82	11.35	11.30	12.08	11.53	10.94	0.36	11.63	0.40	1.06	0.088	9.1E-02
Mtr.46251.1.S1_x_at	4.05	3.88	3.37	3.32	3.23	3.29	3.77	0.36	3.28	0.05	0.87	-0.201	7.8E-02
Mtr.22414.1.S1_s_at	6.34	5.77	6.42	6.62	6.92	6.99	6.18	0.36	6.84	0.20	1.11	0.148	4.7E-02
Mtr.5468.1.S1_at	2.63	2.32	3.04	2.80	2.89	2.99	2.66	0.36	2.90	0.09	1.09	0.120	3.4E-01
Mtr.32063.1.S1_at	7.27	7.36	7.92	8.15	7.74	7.66	7.51	0.36	7.85	0.26	1.04	0.062	2.6E-01
Mtr.18924.1.S1_at	4.76	4.09	4.63	4.51	4.41	4.46	4.49	0.36	4.46	0.05	0.99	-0.011	8.8E-01
Mtr.35393.1.S1_at	2.81	3.40	2.76	2.74	2.72	2.73	2.99	0.36	2.73	0.01	0.91	-0.132	2.7E-01
Mtr.40746.1.S1_at	10.64	10.28	10.99	10.96	10.92	11.02	10.63	0.36	10.97	0.05	1.03	0.044	1.9E-01
Mtr.27696.1.S1_at	3.91	4.17	4.62	3.88	3.90	3.83	4.23	0.36	3.87	0.03	0.91	-0.130	1.5E-01
Mtr.13939.1.S1_at	9.50	9.64	10.18	9.64	9.84	9.44	9.78	0.36	9.64	0.20	0.99	-0.020	5.9E-01
Mtr.18273.1.S1_at	2.63	3.24	2.62	2.60	2.58	3.47	2.83	0.36	2.88	0.50	1.02	0.026	8.9E-01
Mtr.18660.1.S1_at	2.83	3.48	2.90	2.89	2.86	2.85	3.07	0.36	2.87	0.02	0.93	-0.098	3.9E-01
Mtr.44954.1.S1_s_at	4.85	4.22	4.25	3.55	4.36	4.27	4.44	0.36	4.06	0.44	0.91	-0.129	3.1E-01
Mtr.14491.1.S1_x_at	2.83	3.43	2.80	2.78	2.74	2.74	3.02	0.36	2.75	0.02	0.91	-0.133	2.7E-01
Mtr.35018.1.S1_at	2.04	2.65	2.03	2.03	2.03	2.02	2.24	0.36	2.03	0.00	0.90	-0.147	3.5E-01
Mtr.31988.1.S1_at	3.34	3.84	3.15	3.43	3.01	3.01	3.44	0.36	3.15	0.24	0.91	-0.129	3.0E-01
Mtr.9934.1.S1_at	11.74	12.26	12.42	12.14	12.12	12.09	12.14	0.36	12.12	0.03	1.00	-0.002	9.3E-01
Mtr.9393.1.S1_at	10.27	9.58	9.76	9.82	9.96	10.05	9.87	0.36	9.94	0.12	1.01	0.011	7.5E-01
Mtr.27745.1.S1_at	10.68	10.57	11.23	10.63	10.69	10.80	10.83	0.36	10.71	0.09	0.99	-0.016	6.0E-01
Mtr.45427.1.S1_at	6.95	7.66	7.38	7.36	7.52	7.40	7.33	0.36	7.43	0.08	1.01	0.019	6.7E-01
Mtr.18069.1.S1_x_at	8.02	7.34	7.86	7.88	7.89	7.92	7.74	0.36	7.90	0.03	1.02	0.029	4.9E-01
Mtr.33708.1.S1_at	2.44	3.05	2.42	2.42	2.39	2.37	2.63	0.36	2.40	0.03	0.91	-0.136	3.2E-01
Mtr.4585.1.S1_at	9.40	9.86	10.10	9.70	9.81	9.44	9.79	0.36	9.65	0.19	0.99	-0.020	5.9E-01
Mtr.1913.1.S1_at	2.81	3.37	2.71	3.03	2.89	2.89	2.96	0.36	2.94	0.08	0.99	-0.012	9.1E-01
Mtr.10276.1.S1_at	7.36	7.32	7.96	7.03	7.67	7.74	7.55	0.36	7.48	0.39	0.99	-0.013	8.3E-01
Mtr.46812.1.S1_at	10.10	10.40	10.81	10.50	10.43	10.40	10.43	0.36	10.44	0.05	1.00	0.001	9.6E-01
Mtr.35057.1.S1_at	11.83	11.84	11.22	11.35	10.43	11.26	11.63	0.36	11.01	0.51	0.95	-0.078	1.6E-01
Mtr.38236.1.S1_at	9.42	8.77	9.34	9.31	9.31	9.35	9.18	0.36	9.32	0.03	1.02	0.022	5.2E-01
Mtr.29126.1.S1_at	7.56	7.56	8.17	7.95	7.97	7.97	7.76	0.36	7.96	0.01	1.03	0.037	3.9E-01
Mtr.10982.1.S1_at	2.53	3.13	2.50	2.48	2.47	2.47	2.72	0.36	2.47	0.01	0.91	-0.137	3.0E-01
Mtr.20182.1.S1_at	10.70	9.99	10.33	10.28	10.28	10.38	10.34	0.36	10.31	0.06	1.00	-0.004	8.9E-01
Mtr.25311.1.S1_at	10.59	9.88	10.18	10.40	10.17	10.43	10.22	0.36	10.33	0.14	1.01	0.016	6.3E-01
Mtr.38641.1.S1_at	9.57	8.86	9.17	9.29	9.28	9.40	9.20	0.36	9.32	0.07	1.01	0.019	5.9E-01
Mtr.42719.1.S1_at	8.17	8.42	8.87	8.80	8.58	8.60	8.49	0.36	8.66	0.12	1.02	0.029	4.8E-01
Mtr.22342.1.S1_at	6.52	7.03	6.35	6.51	6.46	6.95	6.64	0.36	6.64	0.27	1.00	0.002	9.8E-01
Mtr.41558.1.S1_at	14.10	13.56	13.43	13.61	13.48	13.41	13.70	0.36	13.50	0.10	0.99	-0.021	4.1E-01
Mtr.38467.1.S1_at	10.77	10.30	10.99	10.69	10.71	10.71	10.69	0.36	10.71	0.01	1.00	0.003	9.3E-01
Mtr.49646.1.S1_at	3.22	3.91	3.42	3.25	3.05	3.24	3.52	0.36	3.18	0.11	0.90	-0.145	1.9E-01
Mtr.29143.1.S1_s_at	8.44	8.73	9.15	8.70	9.17	8.86	8.78	0.36	8.91	0.24	1.02	0.022	6.2E-01
Mtr.46004.1.S1_at	2.28	2.33	2.92	2.25	2.23	2.73	2.51	0.36	2.40	0.28	0.96	-0.061	7.1E-01
Mtr.2163.1.S1_s_at	7.57	7.36	8.05	7.50	7.53	7.80	7.66	0.36	7.61	0.17	0.99	-0.010	8.3E-01
Mtr.43932.1.S1_at	9.44	8.73	9.13	9.22	9.17	9.10	9.10	0.36	9.16	0.06	1.01	0.009	7.9E-01
Mtr.45124.1.S1_at	10.25	10.81	10.92	9.79	9.66	9.92	10.66	0.36	9.79	0.13	0.92	-0.123	1.7E-02
Mtr.42589.1.S1_at	8.07	7.81	8.51	8.18	8.34	7.65	8.13	0.36	8.06	0.36	0.99	-0.013	8.1E-01
Mtr.9661.1.S1_at	2.88	2.49	3.20	3.20	3.41	4.45	2.86	0.36	3.68	0.67	1.29	0.366	1.3E-01
Mtr.11824.1.S1_at	10.32	9.62	9.86	10.27	10.16	10.28	9.93	0.36	10.24	0.07	1.03	0.044	2.2E-01
Mtr.4310.1.S1_at	2.29	2.89	2.27	2.23	2.26	2.27	2.48	0.36	2.25	0.02	0.91	-0.140	3.3E-01
Mtr.14347.1.S1_at	10.13	9.57	9.48	10.01	10.29	10.37	9.73	0.36	10.22	0.19	1.05	0.072	9.9E-02
Mtr.19048.1.S1_at	2.67	3.23	2.57	2.57	2.55	2.53	2.82	0.36	2.55	0.02	0.90	-0.145	2.6E-01
Mtr.27134.1.S1_s_at	10.13	9.43	9.73	9.89	9.73	9.98	9.76	0.36	9.87	0.13	1.01	0.015	6.6E-01
Mtr.9357.1.S1_at	4.61	3.96	4.05	4.10	4.05	3.97	4.21	0.36	4.04	0.06	0.96	-0.059	4.6E-01
Mtr.18075.1.S1_at	3.58	2.99	2.94	2.94	2.90	3.30	3.17	0.36	3.04	0.22	0.96	-0.058	6.3E-01
Mtr.21000.1.S1_at	14.66	14.90	14.20	14.65	14.60	14.71	14.59	0.36	14.65	0.05	1.00	0.006	7.7E-01
Mtr.42452.1.S1_at	7.10	6.40	6.70	6.99	7.04	6.98	6.73	0.36	7.00	0.04	1.04	0.057	2.6E-01
Mtr.3011.1.S1_at	8.54	7.85	8.34	8.37	8.37	8.39	8.25	0.36	8.38	0.01	1.02	0.023	5.6E-01
Mtr.7313.1.S1_at	9.76	10.18	10.46	10.11	10.24	10.07	10.13	0.36	10.14	0.09	1.00	0.001	9.8E-01
Mtr.33861.1.S1_at	9.78	10.45	10.32	9.67	10.02	10.00	10.18	0.36	9.90	0.20	0.97	-0.041	2.9E-01
Mtr.49486.1.S1_at	2.69	2.88	3.37	2.64	2.62	2.81	2.98	0.36	2.69	0.11	0.90	-0.147	2.5E-01
Mtr.42265.1.S1_at	10.87	11.19	11.58	11.10	11.00	11.04	11.22	0.36	11.05	0.05	0.98	-0.022	4.6E-01
Mtr.17406.1.S1_at	4.06	3.50	3.40	3.39	3.39	3.37	3.65	0.36	3.39	0.01	0.93	-0.110	2.6E-01
Mtr.45586.1.S1_at	7.96	7.53	8.23	7.71	7.83	7.71	7.91	0.36	7.75	0.07	0.98	-0.029	5.0E-01
Mtr.14189.1.S1_at	8.63	8.15	7.94	8.40	8.40	8.50	8.24	0.36	8.43	0.06	1.02	0.033	4.1E-01
Mtr.44473.1.S1_at	4.68	3.97	4.26	3.95	4.18	3.93	4.30	0.36	4.02	0.14	0.93	-0.097	2.7E-01
Mtr.8850.1.S1_at	10.41	10.39	11.01	10.54	10.62	10.50	10.60	0.36	10.55	0.06	1.00	-0.007	8.2E-01
Mtr.26348.1.S1_at	7.14	6.57	7.22	7.62	7.27	7.56	6.98	0.36	7.48	0.19	1.07	0.101	9.5E-02
Mtr.22366.1.S1_at	5.13	4.43	4.68	4.89	4.67	4.87	4.75	0.36	4.81	0.12	1.01	0.019	7.8E-01
Mtr.43729.1.S1_at	9.96	9.25	9.64	9.79	9.85	9.70	9.61	0.36	9.78	0.07	1.02	0.025	4.6E-01
Mtr.25176.1.S1_s_at	10.72	10.67	11.31	11.09	10.89	10.99	10.90	0.36	10.99	0.10	1.01	0.012	6.9E-01
Mtr.35689.1.S1_at	12.09	12.50	12.79	12.45	12.33	12.37	12.46	0.36	12.38	0.06	0.99	-0.009	7.3E-01
Mtr.15242.1.S1_at	2.74	3.32	2.67	2.67	2.64	2.69	2.91	0.36	2.67	0.02	0.92	-0.126	3.0E-01
Mtr.32052.1.S1_at	2.39	2.45	3.04	2.38	2.40	2.38	2.63	0.36	2.39	0.01	0.91	-0.139	3.0E-01
Mtr.148.1.S1_s_at	7.11	6.83	6.41	6.79	7.09	7.14	6.78	0.36	7.00	0.19	1.03	0.046	3.9E-01
Mtr.10962.1.S1_at	11.08	10.46	11.06	11.14	11.02	10.96	10.87	0.36	11.04	0.09	1.02	0.023	4.6E-01
Mtr.202.1.S1_at	10.36	9.72	10.30	10.26	10.24	10.29	10.13	0.36	10.26	0.02	1.01	0.020	5.4E-01
Mtr.40464.1.S1_at	12.06	12.77	12.38	12.18	12.37	12.26	12.40	0.35	12.27	0.09	0.99	-0.015	5.7E-01
Mtr.33740.1.S1_at	3.58	2.92	3.48	3.41	3.41	3.48	3.33	0.35	3.43	0.04	1.03	0.046	6.3E-01
Mtr.27429.1.S1_at	8.74	8.32	9.02	8.50	8.25	8.60	8.69	0.35	8.45	0.18	0.97	-0.041	3.5E-01
Mtr.34627.1.S1_at	9.11	8.41	8.86	9.12	8.93	9.04	8.79	0.35	9.03	0.10	1.03	0.039	3.2E-01
Mtr.44717.1.S1_at	10.36	9.78	10.42	10.49	10.38	10.30	10.19	0.35	10.39	0.10	1.02	0.029	3.9E-01
Mtr.11844.1.S1_at	9.58	8.95	9.54	10.49	10.60	10.16	9.36	0.35	10.41	0.23	1.11	0.155	1.2E-02
Mtr.35769.1.S1_at	9.01	9.42	9.72	9.21	9.49	9.20	9.39	0.35	9.30	0.17	0.99	-0.013	7.3E-01
Mtr.42503.1.S1_at	5.46	4.79	5.33	4.55	4.85	4.52	5.19	0.35	4.64	0.18	0.89	-0.162	7.5E-02
Mtr.12361.1.S1_at	6.59	6.67	6.02	8.21	6.62	7.46	6.43	0.35	7.43	0.80	1.16	0.210	1.2E-01
Mtr.22583.1.S1_s_at	11.37	11.78	12.08	11.66	11.66	11.73	11.75	0.35	11.68	0.04	0.99	-0.008	7.8E-01
Mtr.38558.1.S1_at	9.00	8.30	8.55	8.73	8.72	9.08	8.62	0.35	8.84	0.20	1.03	0.038	3.9E-01
Mtr.7894.1.S1_at	12.25	11.60	11.67	11.82	11.95	11.68	11.84	0.35	11.82	0.14	1.00	-0.003	9.3E-01
Mtr.4390.1.S1_at	2.62	2.57	3.21	2.61	2.50	2.61	2.80	0.35	2.58	0.06	0.92	-0.120	3.4E-01
Mtr.41250.1.S1_at	8.22	7.52	7.78	7.85	7.88	7.68	7.84	0.35	7.80	0.11	1.00	-0.007	8.7E-01
Mtr.39658.1.S1_at	6.77	6.18	6.81	7.04	7.05	6.96	6.59	0.35	7.02	0.05	1.07	0.091	1.1E-01
Mtr.397.1.S1_at	3.51	4.11	3.47	3.46	3.45	3.56	3.70	0.35	3.49	0.06	0.94	-0.084	3.7E-01
Mtr.34926.1.S1_s_at	7.86	7.30	7.96	7.90	8.16	8.19	7.71	0.35	8.09	0.16	1.05	0.069	1.7E-01
Mtr.52155.1.S1_at	11.38	10.98	11.69	11.43	11.51	11.51	11.35	0.35	11.48	0.04	1.01	0.017	5.5E-01
Mtr.876.1.S1_s_at	9.95	9.47	10.16	9.78	9.92	9.55	9.86	0.35	9.75	0.19	0.99	-0.016	6.6E-01
Mtr.30174.1.S1_at	2.93	3.53	2.91	2.89	2.87	2.86	3.12	0.35	2.87	0.02	0.92	-0.121	2.9E-01
Mtr.6946.1.S1_at	8.28	8.06	8.75	8.84	8.72	8.54	8.37	0.35	8.70	0.15	1.04	0.057	2.1E-01
Mtr.38135.1.S1_at	10.79	10.13	10.69	10.73	10.70	10.68	10.54	0.35	10.70	0.02	1.02	0.022	4.7E-01
Mtr.22683.1.S1_s_at	6.97	6.98	7.59	6.95	6.87	6.96	7.18	0.35	6.93	0.05	0.96	-0.052	2.9E-01
Mtr.13422.1.S1_at	8.01	7.99	8.61	9.01	8.30	8.73	8.20	0.35	8.68	0.36	1.06	0.081	1.8E-01
Mtr.51283.1.S1_at	2.73	3.33	2.70	2.70	2.62	2.64	2.92	0.35	2.65	0.04	0.91	-0.139	2.6E-01
Mtr.12606.1.S1_at	12.45	12.87	13.16	12.58	12.84	12.52	12.83	0.35	12.65	0.17	0.99	-0.020	4.8E-01
Mtr.2165.1.S1_at	4.68	4.08	4.05	4.03	4.37	4.01	4.27	0.35	4.14	0.20	0.97	-0.046	6.0E-01
Mtr.30872.1.S1_at	9.24	8.68	9.33	9.20	9.38	8.93	9.08	0.35	9.17	0.23	1.01	0.014	7.4E-01
Mtr.35852.1.S1_at	10.69	11.37	11.20	10.71	10.58	10.81	11.09	0.35	10.70	0.11	0.97	-0.051	1.5E-01
Mtr.7511.1.S1_at	5.14	5.35	5.83	5.67	5.02	5.36	5.44	0.35	5.35	0.33	0.98	-0.023	7.7E-01
Mtr.39223.1.S1_at	9.38	8.80	9.44	9.25	9.47	9.42	9.21	0.35	9.38	0.12	1.02	0.027	4.7E-01
Mtr.10884.1.S1_at	10.65	10.62	11.25	10.89	10.93	10.83	10.84	0.35	10.88	0.05	1.00	0.006	8.4E-01
Mtr.50587.1.S1_at	4.26	4.93	4.42	5.97	5.71	5.08	4.54	0.35	5.59	0.46	1.23	0.301	3.5E-02
Mtr.34854.1.S1_s_at	7.61	7.05	7.70	7.82	7.73	7.82	7.45	0.35	7.79	0.05	1.05	0.064	1.8E-01
Mtr.11691.1.S1_at	11.51	11.38	12.05	11.84	11.71	11.47	11.65	0.35	11.67	0.19	1.00	0.003	9.2E-01
Mtr.38012.1.S1_at	8.36	7.66	7.97	8.19	8.22	8.22	8.00	0.35	8.21	0.02	1.03	0.039	3.5E-01
Mtr.43684.1.S1_at	9.51	8.87	8.93	9.46	9.33	9.69	9.10	0.35	9.49	0.18	1.04	0.061	1.6E-01
Mtr.45373.1.S1_at	10.86	10.95	10.30	10.49	10.32	10.50	10.70	0.35	10.44	0.10	0.98	-0.036	2.8E-01
Mtr.2726.1.S1_x_at	2.99	3.58	2.93	2.91	2.89	2.85	3.17	0.35	2.88	0.03	0.91	-0.137	2.4E-01
Mtr.16799.1.S1_at	3.69	3.07	3.68	4.14	3.68	3.73	3.48	0.35	3.85	0.25	1.11	0.145	2.2E-01
Mtr.8522.1.S1_at	9.70	10.27	9.62	9.96	10.10	10.25	9.86	0.35	10.10	0.15	1.02	0.035	3.4E-01
Mtr.28683.1.S1_at	2.30	2.39	2.95	2.29	2.29	2.28	2.55	0.35	2.29	0.01	0.90	-0.156	2.7E-01
Mtr.48200.1.S1_at	8.08	7.58	8.27	8.14	8.21	8.21	7.98	0.35	8.19	0.04	1.03	0.037	3.7E-01
Mtr.8436.1.S1_at	10.39	9.99	10.70	11.47	11.47	11.83	10.36	0.35	11.59	0.21	1.12	0.161	6.7E-03
Mtr.18329.1.S1_at	2.67	3.26	2.63	2.64	2.61	2.64	2.85	0.35	2.63	0.02	0.92	-0.116	3.4E-01
Mtr.14326.1.S1_s_at	9.12	8.91	9.60	9.56	9.47	9.21	9.21	0.35	9.42	0.18	1.02	0.032	4.2E-01
Mtr.5960.1.S1_s_at	8.74	8.25	8.94	8.54	8.44	8.55	8.65	0.35	8.51	0.06	0.98	-0.023	5.4E-01
Mtr.38020.1.S1_at	8.86	8.24	8.83	8.86	8.97	8.60	8.64	0.35	8.81	0.19	1.02	0.027	5.2E-01
Mtr.17229.1.S1_at	9.63	9.06	9.70	9.77	9.66	9.54	9.46	0.35	9.66	0.12	1.02	0.029	4.2E-01
Mtr.42319.1.S1_at	7.38	7.28	6.72	7.42	7.22	7.24	7.13	0.35	7.30	0.11	1.02	0.034	4.8E-01
Mtr.42907.1.S1_at	3.39	2.99	2.69	3.69	4.50	3.81	3.03	0.35	4.00	0.44	1.32	0.402	4.0E-02

Mtr.10396.1.S1_at	8.62	8.02	8.65	10.52	10.66	10.57	8.43	0.35	10.58	0.07	1.26	0.328	4.9E-04
Mtr.38301.1.S1_s_at	10.52	10.98	11.21	11.04	11.06	11.06	10.91	0.35	11.06	0.01	1.01	0.020	5.1E-01
Mtr.430.1.S1_at	2.97	3.57	2.95	3.27	2.96	2.94	3.16	0.35	3.05	0.18	0.97	-0.051	6.6E-01
Mtr.16081.1.S1_at	3.50	4.09	3.45	3.37	3.30	3.38	3.68	0.35	3.35	0.04	0.91	-0.135	1.9E-01
Mtr.49525.1.S1_at	7.80	8.07	8.50	8.12	7.94	7.69	8.12	0.35	7.91	0.22	0.97	-0.037	4.4E-01
Mtr.38296.1.S1_s_at	10.55	11.00	11.24	11.02	11.19	10.80	10.93	0.35	11.01	0.19	1.01	0.010	7.6E-01
Mtr.1297.1.S1_x_at	3.26	3.84	3.20	3.14	3.12	3.13	3.43	0.35	3.13	0.01	0.91	-0.135	2.1E-01
Mtr.38014.1.S1_at	8.05	7.58	8.27	8.02	7.98	8.00	7.97	0.35	8.00	0.02	1.00	0.006	8.8E-01
Mtr.10475.1.S1_at	13.40	13.19	13.88	13.43	13.39	13.26	13.49	0.35	13.36	0.09	0.99	-0.014	5.6E-01
Mtr.10287.1.S1_at	9.77	9.09	9.62	9.50	9.58	9.52	9.49	0.35	9.53	0.04	1.00	0.006	8.6E-01
Mtr.22044.1.S1_at	2.81	3.40	2.76	2.92	2.78	2.84	2.99	0.35	2.85	0.07	0.95	-0.070	5.3E-01
Mtr.38261.1.S1_at	9.04	8.48	8.39	8.76	8.75	8.95	8.64	0.35	8.82	0.11	1.02	0.030	4.4E-01
Mtr.39220.1.S1_at	5.98	5.87	6.53	6.28	5.31	5.70	6.12	0.35	5.76	0.49	0.94	-0.088	3.5E-01
Mtr.1429.1.S1_x_at	3.13	3.72	3.08	3.07	3.07	2.92	3.31	0.35	3.02	0.09	0.91	-0.131	2.4E-01
Mtr.43629.1.S1_s_at	9.46	8.75	9.06	9.04	9.29	9.24	9.09	0.35	9.19	0.13	1.01	0.016	6.7E-01
Mtr.2676.1.S1_at	10.91	10.24	10.76	10.75	10.34	10.60	10.64	0.35	10.56	0.21	0.99	-0.010	7.7E-01
Mtr.11448.1.S1_at	9.18	8.48	8.88	9.24	9.21	9.40	8.84	0.35	9.28	0.10	1.05	0.069	1.1E-01
Mtr.13381.1.S1_at	8.23	8.01	8.70	8.53	8.51	8.37	8.31	0.35	8.47	0.09	1.02	0.027	4.9E-01
Mtr.40064.1.S1_at	10.33	10.71	11.03	10.77	10.80	10.86	10.69	0.35	10.81	0.05	1.01	0.016	5.8E-01
Mtr.36581.1.S1_at	6.46	5.83	6.41	6.33	6.82	6.36	6.23	0.35	6.50	0.27	1.04	0.061	3.6E-01
Mtr.40077.1.S1_s_at	9.68	10.32	10.27	9.82	9.83	9.92	10.09	0.35	9.85	0.05	0.98	-0.034	3.2E-01
Mtr.15332.1.S1_at	10.40	9.69	10.12	9.79	10.13	10.13	10.07	0.35	10.01	0.20	0.99	-0.008	8.2E-01
Mtr.47243.1.S1_at	8.69	8.24	8.94	8.62	8.75	8.32	8.62	0.35	8.56	0.22	0.99	-0.010	8.2E-01
Mtr.45513.1.S1_at	7.28	7.98	7.60	7.37	7.65	7.51	7.62	0.35	7.51	0.14	0.99	-0.021	6.4E-01
Mtr.48249.1.S1_at	2.61	2.74	3.27	2.59	2.56	2.54	2.87	0.35	2.56	0.03	0.89	-0.164	2.1E-01
Mtr.24170.1.S1_s_at	7.27	7.75	7.96	7.82	7.84	7.68	7.66	0.35	7.78	0.09	1.02	0.022	6.0E-01
Mtr.20901.1.S1_s_at	11.48	11.46	10.86	11.31	11.21	11.34	11.27	0.35	11.29	0.07	1.00	0.002	9.4E-01
Mtr.13296.1.S1_at	9.70	9.99	10.41	9.70	10.03	9.69	10.04	0.35	9.81	0.20	0.98	-0.033	3.8E-01
Mtr.16050.1.S1_at	8.23	8.92	8.72	8.67	8.51	8.66	8.63	0.35	8.61	0.09	1.00	-0.002	9.5E-01
Mtr.38323.1.S1_at	7.82	7.37	8.07	7.64	7.80	7.89	7.75	0.35	7.78	0.13	1.00	0.004	9.3E-01
Mtr.35355.1.S1_at	9.92	9.75	10.43	9.97	10.26	9.90	10.03	0.35	10.04	0.19	1.00	0.002	9.6E-01
Mtr.21241.1.S1_at	12.89	13.53	13.47	13.31	13.33	13.32	13.30	0.35	13.32	0.01	1.00	0.002	9.2E-01
Mtr.543.1.S1_at	2.82	3.41	2.78	2.78	2.78	2.73	3.00	0.35	2.77	0.03	0.92	-0.119	3.1E-01
Mtr.409.1.S1_at	7.32	6.72	6.70	7.05	7.36	6.86	6.91	0.35	7.09	0.25	1.03	0.036	5.2E-01
Mtr.51452.1.S1_x_at	2.54	3.15	2.53	2.53	2.53	2.51	2.74	0.35	2.52	0.01	0.92	-0.121	3.4E-01
Mtr.40277.1.S1_x_at	13.05	12.35	12.66	12.75	12.54	12.92	12.69	0.35	12.74	0.19	1.00	0.006	8.4E-01
Mtr.32406.1.S1_at	7.19	7.09	7.75	7.65	7.70	7.60	7.34	0.35	7.65	0.05	1.04	0.059	2.1E-01
Mtr.35726.1.S1_at	11.89	11.43	12.13	12.03	11.88	12.00	11.81	0.35	11.97	0.08	1.01	0.018	5.1E-01
Mtr.51396.1.S1_at	4.28	3.58	3.93	3.94	3.87	3.97	3.93	0.35	3.93	0.05	1.00	0.000	1.0E+00
Mtr.35893.1.S1_at	13.36	12.66	12.89	13.10	13.12	13.13	12.97	0.35	13.12	0.01	1.01	0.016	5.2E-01
Mtr.16806.1.S1_at	12.55	11.87	12.37	12.51	12.39	12.59	12.26	0.35	12.50	0.10	1.02	0.027	3.3E-01
Mtr.39282.1.S1_at	8.74	9.10	9.44	9.00	9.21	8.90	9.09	0.35	9.04	0.16	0.99	-0.009	8.2E-01
Mtr.49335.1.S1_at	2.45	3.07	2.46	2.46	2.44	2.49	2.66	0.35	2.46	0.03	0.93	-0.109	4.0E-01
Mtr.13075.1.S1_at	10.71	10.03	10.51	10.57	10.41	10.58	10.42	0.35	10.52	0.10	1.01	0.014	6.5E-01
Mtr.27970.1.S1_at	9.23	8.68	9.34	9.08	9.40	8.98	9.09	0.35	9.15	0.22	1.01	0.010	8.0E-01
Mtr.5372.1.S1_at	9.74	10.05	10.44	10.06	10.25	9.93	10.07	0.35	10.08	0.16	1.00	0.001	9.7E-01
Mtr.1874.1.S1_s_at	10.76	10.07	10.29	10.20	10.27	10.32	10.38	0.35	10.26	0.06	0.99	-0.016	6.1E-01
Mtr.43785.1.S1_at	10.13	9.56	10.20	10.07	10.21	9.97	9.96	0.35	10.08	0.12	1.01	0.017	6.1E-01
Mtr.5816.1.S1_at	3.33	2.73	2.70	2.56	2.54	3.05	2.92	0.35	2.72	0.29	0.93	-0.103	4.9E-01
Mtr.39112.1.S1_at	8.11	8.78	8.24	8.20	8.57	8.24	8.38	0.35	8.34	0.20	1.00	-0.007	8.8E-01
Mtr.36571.1.S1_at	7.86	7.21	7.77	7.85	7.68	7.82	7.62	0.35	7.78	0.09	1.02	0.031	4.7E-01
Mtr.13714.1.S1_at	12.66	13.01	12.31	12.53	12.69	12.52	12.66	0.35	12.58	0.09	0.99	-0.009	7.2E-01
Mtr.33876.1.S1_at	9.58	9.26	9.96	9.78	9.72	9.60	9.60	0.35	9.70	0.09	1.01	0.015	6.6E-01
Mtr.11710.1.S1_at	8.02	7.85	8.53	7.46	7.12	7.20	8.13	0.35	7.26	0.18	0.89	-0.164	1.9E-02
Mtr.1203.1.S1_at	9.42	9.63	10.11	11.34	11.99	11.68	9.72	0.35	11.67	0.33	1.20	0.264	2.2E-03
Mtr.20763.1.S1_s_at	4.02	3.95	4.59	4.15	4.38	4.28	4.19	0.35	4.27	0.11	1.02	0.027	7.3E-01
Mtr.6167.1.S1_at	11.33	11.77	12.03	12.01	11.88	11.79	11.71	0.35	11.89	0.11	1.02	0.022	4.4E-01
Mtr.10254.1.S1_at	8.81	8.84	9.44	8.20	8.10	8.44	9.03	0.35	8.25	0.17	0.91	-0.131	2.6E-02
Mtr.22990.1.S1_at	11.91	11.98	12.55	12.22	12.13	12.07	12.15	0.35	12.14	0.08	1.00	-0.001	9.8E-01
Mtr.46547.1.S1_x_at	3.36	3.82	3.13	3.20	3.08	3.13	3.44	0.35	3.14	0.06	0.91	-0.131	2.2E-01
Mtr.1073.1.S1_at	7.67	7.24	7.94	7.25	7.25	6.88	7.62	0.35	7.13	0.21	0.94	-0.096	1.1E-01
Mtr.35788.1.S1_at	10.73	10.03	10.27	10.47	10.44	10.41	10.34	0.35	10.44	0.03	1.01	0.014	6.5E-01
Mtr.16347.1.S1_at	2.88	2.76	3.42	2.93	2.87	3.08	3.02	0.35	2.96	0.11	0.98	-0.030	7.8E-01
Mtr.15165.1.S1_at	2.46	3.07	2.45	2.45	2.43	2.44	2.66	0.35	2.44	0.01	0.92	-0.126	3.4E-01
Mtr.5114.1.S1_s_at	3.86	4.50	4.44	4.63	4.38	4.59	4.26	0.35	4.53	0.13	1.06	0.088	2.8E-01
Mtr.19635.1.S1_at	3.04	3.65	3.03	3.02	2.97	2.92	3.24	0.35	2.97	0.05	0.92	-0.126	2.6E-01
Mtr.18630.1.S1_at	10.88	10.42	10.19	10.11	9.92	10.14	10.50	0.35	10.05	0.12	0.96	-0.063	1.1E-01
Mtr.35267.1.S1_at	4.70	4.00	4.36	4.35	4.52	4.34	4.35	0.35	4.40	0.10	1.01	0.017	8.2E-01
Mtr.8670.1.S1_at	13.13	12.60	13.26	13.19	12.96	13.16	13.00	0.35	13.10	0.13	1.01	0.012	6.5E-01
Mtr.11560.1.S1_s_at	8.79	8.13	8.24	8.33	8.50	8.47	8.38	0.35	8.43	0.09	1.01	0.008	8.3E-01
Mtr.47621.1.S1_at	2.90	3.47	2.83	2.82	2.75	2.73	3.06	0.35	2.77	0.05	0.90	-0.146	2.2E-01
Mtr.10439.1.S1_at	11.96	11.27	11.70	5.24	5.21	5.09	11.64	0.35	5.18	0.08	0.44	-1.169	6.4E-06
Mtr.13125.1.S1_at	8.81	8.53	9.23	8.82	8.87	8.86	8.86	0.35	8.85	0.03	1.00	0.000	9.9E-01
Mtr.35487.1.S1_at	2.82	2.24	2.18	2.65	2.93	2.85	2.41	0.35	2.81	0.15	1.17	0.221	1.4E-01
Mtr.42599.1.S1_at	9.09	8.43	8.96	9.07	8.96	9.22	8.82	0.35	9.09	0.13	1.03	0.042	3.0E-01
Mtr.45473.1.S1_at	9.67	8.97	9.37	9.46	9.51	9.54	9.34	0.35	9.50	0.04	1.02	0.025	4.6E-01
Mtr.41969.1.S1_at	9.38	8.85	9.52	9.37	9.50	9.26	9.25	0.35	9.38	0.12	1.01	0.020	5.7E-01
Mtr.6140.1.S1_s_at	6.25	5.67	6.31	6.74	6.36	6.32	6.08	0.35	6.47	0.23	1.07	0.091	1.8E-01
Mtr.42872.1.S1_at	15.24	14.63	14.63	14.96	15.12	15.10	14.83	0.35	15.06	0.08	1.02	0.022	3.4E-01
Mtr.42617.1.S1_at	11.30	11.52	11.99	11.73	11.58	11.60	11.60	0.35	11.64	0.08	1.00	0.004	8.8E-01
Mtr.9350.1.S1_at	10.90	10.28	10.88	10.76	10.89	10.77	10.69	0.35	10.81	0.07	1.01	0.016	5.9E-01
Mtr.29437.1.S1_at	3.23	3.82	3.19	3.16	3.14	3.11	3.41	0.35	3.14	0.02	0.92	-0.121	2.5E-01
Mtr.33261.1.S1_at	8.66	8.04	8.65	8.50	8.73	8.69	8.45	0.35	8.64	0.12	1.02	0.033	4.2E-01
Mtr.634.1.S1_at	8.42	8.01	8.71	8.70	8.97	8.80	8.38	0.35	8.83	0.14	1.05	0.075	1.1E-01
Mtr.50972.1.S1_s_at	9.18	8.56	9.17	9.30	9.06	9.13	8.97	0.35	9.16	0.13	1.02	0.031	4.2E-01
Mtr.48607.1.S1_at	2.26	2.33	2.90	2.25	2.21	2.24	2.50	0.35	2.23	0.02	0.89	-0.161	2.7E-01
Mtr.34588.1.S1_s_at	11.63	11.57	12.21	12.01	12.00	11.86	11.80	0.35	11.96	0.09	1.01	0.019	5.0E-01
Mtr.7018.1.S1_at	4.83	4.47	5.18	4.21	4.63	4.55	4.83	0.35	4.47	0.22	0.93	-0.112	2.1E-01
Mtr.41016.1.S1_at	8.31	7.94	8.65	8.65	8.47	8.66	8.30	0.35	8.59	0.11	1.04	0.050	2.4E-01
Mtr.4428.1.S1_at	2.99	3.57	2.94	2.92	2.88	2.89	3.17	0.35	2.90	0.02	0.91	-0.130	2.5E-01
Mtr.4416.1.S1_s_at	2.95	3.53	2.90	2.90	2.90	2.87	3.13	0.35	2.89	0.02	0.92	-0.113	3.1E-01
Mtr.36904.1.S1_at	10.09	10.75	10.64	10.17	10.51	10.20	10.49	0.35	10.29	0.19	0.98	-0.028	4.4E-01
Mtr.1390.1.S1_at	3.00	3.58	2.94	2.92	2.86	2.92	3.18	0.35	2.90	0.03	0.91	-0.131	2.5E-01
Mtr.1156.1.S1_at	13.99	13.36	13.40	13.77	13.81	14.01	13.58	0.35	13.86	0.13	1.02	0.030	2.6E-01
Mtr.31541.1.S1_at	6.31	5.79	6.46	6.87	6.85	6.44	6.19	0.35	6.72	0.24	1.09	0.119	9.7E-02
Mtr.48133.1.S1_s_at	7.94	7.69	8.38	8.18	8.08	7.97	8.00	0.35	8.07	0.10	1.01	0.013	7.6E-01
Mtr.33498.1.S1_at	9.80	9.45	9.10	9.75	9.68	9.92	9.45	0.35	9.79	0.12	1.04	0.051	1.9E-01
Mtr.14124.1.S1_at	4.11	4.77	4.22	4.16	3.81	4.16	4.37	0.35	4.05	0.20	0.93	-0.110	2.4E-01
Mtr.12637.1.S1_at	10.34	9.66	10.15	9.93	9.94	9.85	10.05	0.35	9.91	0.05	0.99	-0.021	5.2E-01
Mtr.34233.1.S1_at	2.08	2.68	2.07	2.07	2.07	2.07	2.28	0.35	2.07	0.00	0.91	-0.139	3.6E-01
Mtr.17580.1.S1_at	2.90	3.46	2.81	3.06	2.76	2.74	3.06	0.35	2.85	0.18	0.93	-0.099	4.2E-01
Mtr.47276.1.S1_at	3.51	4.06	3.41	3.44	3.38	3.29	3.66	0.35	3.37	0.07	0.92	-0.119	2.4E-01
Mtr.11267.1.S1_at	12.11	11.74	11.41	11.99	11.97	12.13	11.76	0.35	12.03	0.09	1.02	0.033	2.6E-01
Mtr.28486.1.S1_at	8.33	8.89	8.98	8.61	8.86	8.47	8.73	0.35	8.64	0.20	0.99	-0.014	7.3E-01
Mtr.16342.1.S1_at	9.35	8.65	8.96	9.00	8.97	9.03	8.99	0.35	9.00	0.03	1.00	0.002	9.5E-01
Mtr.35792.1.S1_at	5.42	5.79	6.12	5.46	5.36	5.57	5.78	0.35	5.46	0.10	0.95	-0.081	2.1E-01
Mtr.38380.1.S1_at	7.95	7.46	8.14	8.44	8.86	8.63	7.85	0.35	8.64	0.21	1.10	0.139	2.8E-02
Mtr.26139.1.S1_at	3.46	3.06	2.76	2.82	2.75	2.78	3.09	0.35	2.78	0.03	0.90	-0.154	2.0E-01
Mtr.50197.1.S1_at	8.75	8.64	9.29	8.98	8.96	8.93	8.89	0.35	8.96	0.02	1.01	0.011	7.6E-01
Mtr.1051.1.S1_at	2.98	3.56	2.92	2.88	2.84	2.86	3.15	0.35	2.86	0.02	0.91	-0.140	2.2E-01
Mtr.9038.1.S1_at	11.98	11.33	11.88	11.61	11.93	11.70	11.73	0.35	11.74	0.16	1.00	0.002	9.6E-01
Mtr.762.1.S1_at	13.67	13.08	13.04	12.97	12.76	13.05	13.26	0.35	12.92	0.15	0.97	-0.037	2.0E-01
Mtr.9101.1.S1_at	12.00	11.35	11.43	11.50	11.55	11.66	11.59	0.35	11.57	0.08	1.00	-0.003	9.0E-01
Mtr.51916.1.S1_at	10.99	10.30	10.71	10.62	10.60	10.71	10.67	0.35	10.64	0.06	1.00	-0.003	9.1E-01
Mtr.51139.1.S1_at	2.93	2.62	3.32	2.92	2.92	2.92	2.96	0.35	2.92	0.00	0.99	-0.020	8.5E-01
Mtr.6520.1.S1_at	9.88	9.20	9.41	9.61	9.41	9.54	9.50	0.35	9.52	0.10	1.00	0.003	9.2E-01
Mtr.44727.1.S1_at	9.81	9.94	10.47	10.01	10.31	9.72	10.08	0.35	10.01	0.29	0.99	-0.009	8.3E-01
Mtr.1693.1.S1_at	3.24	3.85	3.24	3.21	3.10	3.20	3.45	0.35	3.17	0.06	0.92	-0.122	2.5E-01
Mtr.10047.1.S1_at	3.83	3.37	3.14	5.06	4.49	3.37	3.44	0.35	4.31	0.86	1.25	0.322	1.8E-01
Mtr.35698.1.S1_at	3.31	3.91	3.29	8.98	9.36	9.10	3.50	0.35	9.15	0.19	2.61	1.384	1.7E-05
Mtr.37481.1.S1_at	8.23	7.95	8.65	8.61	8.33	8.37	8.28	0.35	8.44	0.15	1.02	0.027	5.1E-01
Mtr.51501.1.S1_at	11.89	11.20	11.44	12.02	12.01	12.10	11.51	0.35	12.04	0.05	1.05	0.065	6.1E-02
Mtr.12701.1.S1_at	9.74	9.57	10.25	9.80	9.93	9.65	9.86	0.35	9.80	0.14	0.99	-0.009	8.0E-01
Mtr.10329.1.S1_at	2.58	3.18	2.57	2.56	2.54	2.53	2.78	0.35	2.54	0.01	0.92	-0.126	3.2E-01
Mtr.37613.1.S1_at	11.99	11.30	11.73	11.64	11.77	11.71	11.67	0.35	11.71	0.07	1.00	0.004	8.7E-01
Mtr.41569.1.S1_at	10.70	10.45	11.14	10.80	10.62	10.66	10.76	0.35	10.69	0.09	0.99	-0.010	7.5E-01
Mtr.10496.1.S1_at	9.20	8.58	9.18	9.13	9.14	9.02	8.98	0.35	9.10	0.07	1.01	0.018	6.2E-01
Mtr.11538.1.S1_at	10.27	10.18	10.83	10.68	11.02	10.77	10.43	0.35	10.82	0.17	1.04	0.054	1.5E-01
Mtr.49679.1.S1_at	2.80	3.39	2.77	2.77	2.74	2.71	2.99	0.35	2.74	0.03	0.92	-0.125	2.9E-01
Mtr.26431.1.S1_at	2.84	3.44	2.83	2.83	2.74	2.80	3.04	0.35	2.79	0.05	0.92	-0.122	3.0E-01
Mtr.2559.1.S1_at	8.68	8.04	8.60	8.85	8.78	8.83	8.44	0.35	8.82	0.04	1.05	0.064	1.3E-01

Mtr.46462.1.S1_at	8.51	7.84	8.00	8.40	8.58	8.43	8.12	0.35	8.47	0.10	1.04	0.061	1.7E-01
Mtr.50305.1.S1_at	9.74	9.56	10.24	9.53	8.92	9.24	9.85	0.35	9.23	0.30	0.94	-0.093	8.3E-02
Mtr.10377.1.S1_at	11.66	12.31	12.19	11.84	12.10	11.88	12.05	0.35	11.94	0.10	0.99	-0.014	6.3E-01
Mtr.27222.1.S1_at	9.21	8.53	8.73	9.10	9.10	9.27	8.82	0.35	9.16	0.10	1.04	0.053	1.9E-01
Mtr.25225.1.S1_at	2.66	3.24	2.61	2.61	2.60	2.60	2.84	0.35	2.60	0.01	0.92	-0.123	3.2E-01
Mtr.38882.1.S1_s_at	9.22	8.64	9.28	9.25	9.17	9.32	9.05	0.35	9.25	0.07	1.02	0.031	3.9E-01
Mtr.52094.1.S1_at	6.84	7.41	7.47	7.15	7.69	7.28	7.24	0.35	7.37	0.28	1.02	0.026	6.3E-01
Mtr.24346.1.S1_at	2.80	3.40	2.78	2.85	2.74	2.77	2.99	0.35	2.79	0.06	0.93	-0.102	3.8E-01
Mtr.18525.1.S1_at	9.10	8.48	9.09	9.19	9.14	9.07	8.89	0.35	9.14	0.06	1.03	0.039	3.0E-01
Mtr.42411.1.S1_at	9.09	8.41	8.91	8.86	9.21	9.13	8.81	0.35	9.07	0.18	1.03	0.042	3.1E-01
Mtr.39322.1.S1_at	10.89	11.58	11.11	10.72	10.77	10.94	11.19	0.35	10.81	0.12	0.97	-0.051	1.5E-01
Mtr.32122.1.S1_at	7.75	7.50	7.06	7.38	7.42	7.78	7.44	0.35	7.53	0.22	1.01	0.017	7.2E-01
Mtr.42153.1.S1_at	8.68	7.99	8.24	8.48	8.39	8.37	8.31	0.35	8.41	0.06	1.01	0.018	6.3E-01
Mtr.43489.1.S1_at	11.87	12.16	12.57	12.32	12.52	12.23	12.20	0.35	12.36	0.15	1.01	0.018	5.2E-01
Mtr.14305.1.S1_s_at	2.94	3.53	2.91	2.89	2.83	2.82	3.13	0.35	2.85	0.04	0.91	-0.135	2.4E-01
Mtr.5186.1.S1_at	9.43	8.95	8.75	9.45	9.16	9.41	9.04	0.35	9.34	0.16	1.03	0.047	2.5E-01
Mtr.14909.1.S1_s_at	9.34	8.66	9.15	9.28	9.11	9.16	9.05	0.35	9.18	0.09	1.01	0.021	5.6E-01
Mtr.5406.1.S1_s_at	8.58	8.21	8.91	8.60	8.71	8.61	8.56	0.35	8.64	0.06	1.01	0.013	7.3E-01
Mtr.40750.1.S1_at	11.22	10.86	11.56	11.48	11.28	11.34	11.22	0.35	11.37	0.10	1.01	0.019	5.2E-01
Mtr.8733.1.S1_at	13.12	12.42	12.85	12.78	12.74	12.89	12.79	0.35	12.80	0.08	1.00	0.001	9.7E-01
Mtr.32923.1.S1_at	2.12	2.71	2.10	2.09	2.10	2.09	2.31	0.35	2.09	0.00	0.91	-0.143	3.4E-01
Mtr.44236.1.S1_at	8.98	9.15	9.65	9.05	9.43	9.00	9.26	0.35	9.16	0.24	0.99	-0.016	7.0E-01
Mtr.5699.1.S1_at	9.42	8.84	9.48	9.41	9.25	9.37	9.25	0.35	9.34	0.08	1.01	0.015	6.6E-01
Mtr.43964.1.S1_at	11.78	11.08	11.44	11.42	11.58	11.48	11.44	0.35	11.49	0.08	1.00	0.007	8.0E-01
Mtr.5059.1.S1_at	2.97	3.52	2.87	2.83	2.84	2.89	3.12	0.35	2.85	0.03	0.91	-0.128	2.6E-01
Mtr.33306.1.S1_at	7.60	6.92	7.40	7.60	7.79	7.73	7.30	0.35	7.71	0.10	1.06	0.078	1.3E-01
Mtr.15880.1.S1_at	11.91	11.25	11.79	11.47	11.48	11.46	11.65	0.35	11.47	0.01	0.98	-0.023	4.2E-01
Mtr.1047.1.S1_at	2.74	3.34	2.72	2.70	2.71	2.70	2.93	0.35	2.70	0.00	0.92	-0.119	3.1E-01
Mtr.33282.1.S1_at	2.91	2.30	2.91	3.43	2.90	3.16	2.71	0.35	3.16	0.26	1.17	0.224	1.5E-01
Mtr.44330.1.S1_at	2.77	2.65	3.30	2.65	3.03	2.96	2.91	0.35	2.88	0.20	0.99	-0.011	9.3E-01
Mtr.39507.1.S1_at	9.48	8.88	9.49	9.65	9.82	9.62	9.29	0.35	9.70	0.11	1.04	0.063	1.2E-01
Mtr.36212.1.S1_at	2.89	3.47	2.84	2.83	2.82	2.78	3.07	0.35	2.81	0.03	0.92	-0.128	2.7E-01
Mtr.13645.1.S1_at	8.47	7.79	8.01	8.42	8.24	8.53	8.09	0.35	8.39	0.15	1.04	0.053	2.4E-01
Mtr.3208.1.S1_at	10.43	10.36	11.00	10.21	10.65	10.37	10.59	0.35	10.41	0.22	0.98	-0.025	4.8E-01
Mtr.7117.1.S1_at	5.87	5.26	5.86	6.03	6.10	5.63	5.67	0.35	5.92	0.25	1.05	0.064	3.6E-01
Mtr.9307.1.S1_at	11.11	10.81	10.41	10.87	10.72	11.06	10.78	0.35	10.88	0.17	1.01	0.014	6.6E-01
Mtr.52145.1.S1_at	10.65	9.99	10.53	10.39	10.33	10.37	10.39	0.35	10.36	0.03	1.00	-0.005	8.8E-01
Mtr.33071.1.S1_at	3.08	3.00	3.64	2.95	3.15	2.94	3.24	0.35	3.02	0.12	0.93	-0.104	3.5E-01
Mtr.13251.1.S1_at	9.37	8.97	9.67	9.46	9.52	9.56	9.34	0.35	9.51	0.05	1.02	0.027	4.4E-01
Mtr.47458.1.S1_at	2.44	3.04	2.42	2.42	2.33	2.39	2.63	0.35	2.38	0.04	0.90	-0.144	2.9E-01
Mtr.17206.1.S1_at	3.65	4.05	3.35	3.35	3.06	3.33	3.68	0.35	3.25	0.17	0.88	-0.180	1.3E-01
Mtr.51013.1.S1_s_at	2.92	3.50	2.86	2.84	2.83	2.77	3.09	0.35	2.81	0.04	0.91	-0.136	2.4E-01
Mtr.7205.1.S1_at	2.93	3.52	2.90	2.88	2.89	2.87	3.12	0.35	2.88	0.01	0.92	-0.113	3.1E-01
Mtr.51685.1.S1_s_at	2.71	2.77	3.34	3.35	4.03	2.61	2.94	0.35	3.33	0.71	1.13	0.179	4.4E-01
Mtr.8654.1.S1_at	10.19	9.49	9.88	9.73	11.09	10.55	9.85	0.35	10.45	0.69	1.06	0.086	2.5E-01
Mtr.13227.1.S1_s_at	8.02	8.11	8.66	8.17	8.17	8.17	8.26	0.35	8.17	0.00	0.99	-0.017	6.6E-01
Mtr.44306.1.S1_at	3.12	3.61	3.79	5.75	6.19	5.95	3.51	0.35	5.97	0.22	1.70	0.767	5.0E-04
Mtr.38817.1.S1_at	7.27	7.77	7.94	7.66	7.91	7.49	7.66	0.35	7.69	0.21	1.00	0.005	9.2E-01
Mtr.10502.1.S1_at	13.01	12.51	12.33	12.79	12.88	13.06	12.62	0.35	12.91	0.13	1.02	0.033	2.4E-01
Mtr.21633.1.S1_at	8.80	8.32	9.00	8.92	9.17	8.99	8.71	0.35	9.03	0.13	1.04	0.052	2.1E-01
Mtr.44320.1.S1_at	8.80	9.15	9.50	8.80	8.95	8.93	9.15	0.35	8.89	0.08	0.97	-0.042	2.8E-01
Mtr.45351.1.S1_s_at	3.43	2.88	2.78	3.18	2.87	3.06	3.03	0.35	3.04	0.15	1.00	0.003	9.8E-01
Mtr.35193.1.S1_s_at	6.11	5.53	6.16	6.28	6.32	6.42	5.94	0.35	6.34	0.07	1.07	0.095	1.2E-01
Mtr.18409.1.S1_s_at	3.19	3.71	3.05	2.98	3.12	3.10	3.32	0.35	3.07	0.08	0.92	-0.114	2.9E-01
Mtr.47538.1.S1_at	2.94	2.67	2.25	2.82	2.41	3.12	2.62	0.35	2.78	0.36	1.06	0.088	6.0E-01
Mtr.45058.1.S1_at	9.09	9.08	9.69	9.23	9.35	9.27	9.28	0.35	9.28	0.06	1.00	0.000	9.9E-01
Mtr.11954.1.S1_at	7.72	7.20	7.87	7.58	7.77	7.69	7.60	0.35	7.68	0.09	1.01	0.016	7.1E-01
Mtr.41487.1.S1_at	9.89	9.66	10.35	10.00	10.10	9.93	9.97	0.35	10.01	0.09	1.00	0.006	8.5E-01
Mtr.21609.1.S1_at	7.14	7.07	7.70	7.37	7.46	7.24	7.30	0.35	7.36	0.11	1.01	0.011	8.1E-01
Mtr.15581.1.S1_x_at	2.36	2.96	2.35	2.33	2.32	2.34	2.55	0.35	2.33	0.01	0.91	-0.132	3.3E-01
Mtr.23153.1.S1_at	3.16	3.39	3.85	3.07	3.09	3.44	3.47	0.35	3.20	0.20	0.92	-0.115	3.2E-01
Mtr.20199.1.S1_at	5.08	4.66	5.36	5.44	5.14	5.09	5.03	0.35	5.22	0.19	1.04	0.053	4.5E-01
Mtr.47121.1.S1_at	2.25	2.90	2.36	2.35	2.35	2.36	2.50	0.35	2.35	0.00	0.94	-0.087	5.1E-01
Mtr.4718.1.S1_at	7.63	7.54	8.19	7.98	7.73	7.97	7.79	0.35	7.89	0.14	1.01	0.020	6.4E-01
Mtr.3423.1.S1_at	10.50	9.88	9.92	10.13	9.87	10.08	10.10	0.35	10.03	0.14	0.99	-0.010	7.6E-01
Mtr.39653.1.S1_at	8.72	8.03	8.47	8.54	8.65	8.43	8.41	0.35	8.54	0.11	1.02	0.023	5.6E-01
Mtr.9712.1.S1_at	11.21	10.88	11.58	11.21	11.27	11.24	11.22	0.35	11.24	0.03	1.00	0.002	9.4E-01
Mtr.52114.1.S1_at	7.90	7.48	8.18	8.23	8.61	7.89	7.85	0.35	8.24	0.36	1.05	0.070	2.5E-01
Mtr.8759.1.S1_at	11.28	10.60	11.04	11.01	10.91	11.12	10.97	0.35	11.01	0.11	1.00	0.005	8.5E-01
Mtr.50617.1.S1_at	4.02	4.61	3.99	4.00	3.99	3.98	4.21	0.35	3.99	0.01	0.95	-0.077	3.4E-01
Mtr.5115.1.S1_s_at	8.34	8.12	8.80	8.59	8.32	8.26	8.42	0.35	8.39	0.17	1.00	-0.005	9.1E-01
Mtr.4836.1.S1_at	9.72	9.02	9.43	9.34	9.51	9.39	9.39	0.35	9.42	0.09	1.00	0.004	9.1E-01
Mtr.38175.1.S1_at	10.77	10.94	11.44	10.97	10.98	10.80	11.05	0.35	10.92	0.10	0.99	-0.017	5.6E-01
Mtr.18600.1.S1_at	4.05	3.99	3.42	3.35	3.35	3.48	3.82	0.35	3.40	0.08	0.89	-0.170	1.1E-01
Mtr.41870.1.S1_at	10.50	9.83	10.00	10.37	10.35	10.43	10.11	0.35	10.38	0.04	1.03	0.038	2.5E-01
Mtr.50722.1.S1_at	2.28	2.34	2.91	2.26	2.23	2.26	2.51	0.35	2.25	0.02	0.90	-0.157	2.7E-01
Mtr.43451.1.S1_at	13.30	12.64	12.78	12.98	13.08	13.03	12.90	0.35	13.03	0.05	1.01	0.014	5.7E-01
Mtr.25906.1.S1_s_at	9.38	8.69	9.16	9.27	9.26	9.15	9.08	0.35	9.23	0.06	1.02	0.024	5.0E-01
Mtr.44639.1.S1_at	9.13	8.45	8.66	8.96	8.99	9.14	8.75	0.35	9.03	0.10	1.03	0.046	2.5E-01
Mtr.45347.1.S1_at	10.17	9.65	10.31	10.40	10.30	10.22	10.04	0.35	10.31	0.09	1.03	0.037	2.7E-01
Mtr.5127.1.S1_at	8.29	7.60	8.03	8.38	8.53	8.53	7.97	0.35	8.48	0.09	1.06	0.090	6.9E-02
Mtr.10132.1.S1_at	8.85	8.52	9.21	8.84	8.93	8.87	8.86	0.35	8.88	0.05	1.00	0.003	9.3E-01
Mtr.51307.1.S1_at	3.68	4.01	4.38	3.66	3.63	3.62	4.02	0.35	3.64	0.02	0.90	-0.145	1.3E-01
Mtr.16726.1.S1_at	9.41	8.76	9.31	9.26	9.23	9.24	9.16	0.35	9.24	0.01	1.01	0.013	7.0E-01
Mtr.24061.1.S1_at	7.78	8.43	8.33	7.88	8.11	8.02	8.18	0.35	8.00	0.12	0.98	-0.032	4.4E-01
Mtr.2363.1.S1_at	7.89	8.17	8.59	8.15	8.16	8.04	8.22	0.35	8.12	0.06	0.99	-0.017	6.5E-01
Mtr.18463.1.S1_s_at	11.39	10.75	11.31	11.35	11.24	11.35	11.15	0.35	11.31	0.06	1.01	0.021	4.7E-01
Mtr.23891.1.S1_at	2.52	3.11	2.50	2.48	2.46	2.45	2.71	0.35	2.46	0.01	0.91	-0.138	2.9E-01
Mtr.33093.1.S1_at	11.63	10.98	11.10	11.62	11.62	11.80	11.24	0.35	11.68	0.10	1.04	0.056	1.0E-01
Mtr.10520.1.S1_s_at	8.49	7.81	8.29	8.66	8.36	8.42	8.20	0.35	8.48	0.16	1.03	0.049	2.7E-01
Mtr.45833.1.S1_s_at	9.21	8.58	9.15	9.14	9.24	9.46	8.98	0.35	9.28	0.16	1.03	0.047	2.5E-01
Mtr.32854.1.S1_at	7.54	6.87	7.04	7.53	7.54	7.31	7.15	0.35	7.46	0.13	1.04	0.062	2.2E-01
Mtr.39226.1.S1_at	7.01	6.34	6.84	6.97	6.89	6.88	6.73	0.35	6.91	0.05	1.03	0.038	4.2E-01
Mtr.11049.1.S1_at	8.73	8.50	9.18	9.27	9.63	9.28	8.80	0.35	9.40	0.21	1.07	0.094	6.4E-02
Mtr.9640.1.S1_at	9.22	8.65	9.28	9.94	9.46	9.91	9.05	0.35	9.77	0.27	1.08	0.111	4.6E-02
Mtr.47032.1.S1_at	10.86	11.10	11.55	11.19	11.11	11.04	11.17	0.35	11.11	0.08	0.99	-0.007	8.0E-01
Mtr.47943.1.S1_at	12.68	13.04	13.37	12.90	13.02	12.77	13.03	0.35	12.90	0.13	0.99	-0.015	5.6E-01
Mtr.29521.1.S1_at	9.09	8.40	8.85	9.51	9.58	9.26	8.78	0.35	9.45	0.17	1.08	0.106	4.0E-02
Mtr.332.1.S1_at	8.55	8.66	8.01	8.04	7.90	8.21	8.40	0.35	8.05	0.16	0.96	-0.062	1.9E-01
Mtr.44559.1.S1_at	11.35	11.86	11.19	11.07	11.02	10.92	11.47	0.35	11.00	0.08	0.96	-0.060	8.6E-02
Mtr.51386.1.S1_at	7.87	7.27	7.88	7.82	7.94	7.72	7.67	0.35	7.83	0.11	1.02	0.029	5.0E-01
Mtr.12892.1.S1_at	11.20	10.53	11.02	11.07	11.06	11.00	10.92	0.35	11.04	0.03	1.01	0.016	5.7E-01
Mtr.8530.1.S1_x_at	10.38	9.69	10.05	9.71	9.81	9.89	10.04	0.35	9.80	0.09	0.98	-0.034	3.2E-01
Mtr.48356.1.S1_at	7.73	7.11	7.70	7.99	7.76	7.79	7.52	0.35	7.85	0.13	1.04	0.062	2.0E-01
Mtr.28318.1.S1_at	3.49	3.72	4.17	3.87	4.04	3.77	3.79	0.35	3.90	0.14	1.03	0.039	6.6E-01
Mtr.45772.1.S1_at	9.97	9.29	9.75	9.79	9.39	9.54	9.67	0.35	9.57	0.20	0.99	-0.015	7.0E-01
Mtr.16020.1.S1_at	10.61	10.12	10.80	10.60	10.65	10.54	10.51	0.35	10.59	0.05	1.01	0.012	7.0E-01
Mtr.50796.1.S1_at	9.97	9.40	10.02	10.04	9.96	9.88	9.80	0.35	9.96	0.08	1.02	0.024	4.7E-01
Mtr.20955.1.S1_at	5.28	4.64	5.18	5.18	5.18	5.18	5.03	0.35	5.18	0.00	1.03	0.041	5.1E-01
Mtr.27498.1.S1_s_at	10.01	9.35	9.48	9.99	9.79	10.04	9.62	0.35	9.94	0.14	1.03	0.048	2.0E-01
Mtr.41552.1.S1_at	10.06	9.67	10.36	10.29	10.41	10.24	10.03	0.35	10.31	0.09	1.03	0.040	2.5E-01
Mtr.43866.1.S1_at	9.77	9.11	9.64	9.60	9.68	9.55	9.51	0.35	9.61	0.07	1.01	0.016	6.4E-01
Mtr.26473.1.S1_at	7.25	6.59	7.12	7.09	6.99	6.97	6.99	0.35	7.02	0.07	1.00	0.006	9.0E-01
Mtr.12589.1.S1_at	12.71	12.04	12.22	12.53	12.36	12.54	12.32	0.35	12.48	0.10	1.01	0.018	5.1E-01
Mtr.6067.1.S1_at	6.82	6.14	6.60	6.68	6.58	7.03	6.52	0.35	6.76	0.23	1.04	0.053	3.7E-01
Mtr.5675.1.S1_s_at	9.02	9.36	9.72	9.37	9.46	9.46	9.36	0.35	9.43	0.05	1.01	0.010	7.7E-01
Mtr.39903.1.S1_s_at	8.23	7.60	8.16	8.23	8.08	7.86	8.00	0.35	8.06	0.19	1.01	0.011	7.9E-01
Mtr.13549.1.S1_at	4.83	4.24	4.86	4.96	4.84	5.15	4.64	0.35	4.98	0.15	1.07	0.102	2.0E-01
Mtr.216.1.S1_at	7.21	6.91	7.60	7.25	7.27	7.30	7.24	0.35	7.27	0.03	1.00	0.007	8.7E-01
Mtr.9143.1.S1_at	12.08	11.39	11.83	11.93	11.63	11.79	11.77	0.35	11.78	0.15	1.00	0.002	9.5E-01
Mtr.25705.1.S1_at	2.49	3.11	2.53	2.48	2.47	2.46	2.71	0.35	2.47	0.01	0.91	-0.134	3.0E-01
Mtr.19392.1.S1_at	8.44	7.74	8.12	8.35	8.05	8.02	8.10	0.35	8.14	0.18	1.00	0.007	8.8E-01
Mtr.14469.1.S1_at	2.29	1.95	2.65	2.29	2.04	2.06	2.30	0.35	2.13	0.14	0.93	-0.107	4.9E-01
Mtr.39675.1.S1_at	2.99	3.57	2.95	2.93	2.85	2.90	3.17	0.35	2.89	0.04	0.91	-0.132	2.4E-01
Mtr.45415.1.S1_a_at	10.91	10.36	11.00	10.82	11.25	10.68	10.76	0.35	10.92	0.29	1.02	0.022	5.7E-01
Mtr.1487.1.S1_at	2.21	2.81	2.20	2.20	2.20	2.20	2.41	0.35	2.20	0.00	0.91	-0.131	3.6E-01
Mtr.13113.1.S1_at	9.10	8.42	8.90	8.91	8.92	8.92	8.81	0.35	8.92	0.00	1.01	0.017	6.3E-01
Mtr.14327.1.S1_at	10.47	9.83	10.38	10.43	10.55	10.47	10.23	0.35	10.48	0.06	1.03	0.036	2.8E-01
Mtr.32377.1.S1_at	6.92	6.26	6.76	7.17	6.66	7.15	6.65	0.35	6.99	0.28	1.05	0.074	2.5E-01
Mtr.30413.1.S1_at	8.14	7.66	8.33	7.89	7.78	7.47	8.04	0.35	7.72	0.22	0.96	-0.060	2.4E-01
Mtr.23228.1.S1_at	15.14	14.49	14.62	15.34	15.32	15.26	14.75	0.35	15.31	0.04	1.04	0.053	5.1E-02

Mtr.45731.1.S1_at	10.00	9.41	10.02	9.81	9.79	9.91	9.81	0.35	9.84	0.06	1.00	0.004	9.0E-01
Mtr.5354.1.S1_at	8.90	8.90	9.50	8.91	9.05	9.08	9.10	0.35	9.01	0.09	0.99	-0.014	6.9E-01
Mtr.43926.1.S1_at	11.23	10.68	11.32	11.31	11.26	11.23	11.08	0.35	11.27	0.04	1.02	0.025	4.0E-01
Mtr.1578.1.S1_at	7.69	7.12	7.75	7.73	7.74	7.97	7.52	0.35	7.81	0.13	1.04	0.055	2.4E-01
Mtr.23398.1.S1_at	2.82	2.84	3.43	2.77	2.77	2.79	3.03	0.35	2.78	0.01	0.92	-0.124	2.8E-01
Mtr.15909.1.S1_at	2.94	3.53	2.92	2.92	2.92	2.94	3.13	0.35	2.92	0.01	0.93	-0.099	3.6E-01
Mtr.35266.1.S1_at	8.93	8.40	9.06	8.82	8.82	8.85	8.80	0.35	8.83	0.02	1.00	0.005	8.8E-01
Mtr.51286.1.S1_at	3.70	3.12	3.08	3.07	3.04	3.07	3.30	0.35	3.06	0.02	0.93	-0.110	2.9E-01
Mtr.12712.1.S1_at	2.87	3.54	3.03	3.27	4.65	3.25	3.15	0.35	3.72	0.80	1.18	0.243	3.2E-01
Mtr.16993.1.S1_at	10.02	9.48	10.13	9.80	9.94	9.83	9.88	0.35	9.86	0.08	1.00	-0.003	9.3E-01
Mtr.35631.1.S1_at	9.38	8.82	8.76	8.42	8.50	8.69	8.99	0.35	8.53	0.14	0.95	-0.074	1.1E-01
Mtr.1269.1.S1_at	3.14	3.79	3.68	3.67	3.67	3.67	3.54	0.35	3.67	0.00	1.04	0.054	5.4E-01
Mtr.14858.1.S1_at	10.19	10.60	10.88	10.17	10.25	10.16	10.56	0.35	10.19	0.05	0.97	-0.051	1.5E-01
Mtr.38901.1.S1_at	10.78	10.11	10.60	10.51	10.60	10.50	10.49	0.35	10.54	0.05	1.00	0.006	8.4E-01
Mtr.33334.1.S1_at	11.23	11.92	11.50	11.60	11.81	11.76	11.55	0.35	11.73	0.11	1.02	0.022	4.4E-01
Mtr.24785.1.S1_s_at	4.31	3.74	3.69	3.65	4.20	3.64	3.91	0.35	3.83	0.32	0.98	-0.031	7.8E-01
Mtr.31798.1.S1_at	9.00	8.53	9.21	9.13	9.06	8.93	8.91	0.35	9.04	0.10	1.01	0.020	5.8E-01
Mtr.12090.1.S1_at	10.24	9.81	10.50	10.24	10.22	10.16	10.18	0.35	10.21	0.04	1.00	0.003	9.1E-01
Mtr.749.1.S1_x_at	2.99	3.56	2.93	2.86	2.84	2.86	3.16	0.35	2.85	0.01	0.90	-0.145	2.1E-01
Mtr.41103.1.S1_at	10.55	10.22	10.92	10.76	10.63	10.82	10.56	0.35	10.74	0.10	1.02	0.023	4.5E-01
Mtr.44484.1.S1_at	7.20	6.51	6.76	7.06	7.32	7.20	6.82	0.35	7.19	0.13	1.05	0.077	1.6E-01
Mtr.5115.1.S1_at	7.05	6.77	7.46	7.07	7.08	7.07	7.10	0.35	7.07	0.01	1.00	-0.006	9.0E-01
Mtr.39561.1.S1_at	9.41	8.87	9.52	9.31	9.28	9.26	9.27	0.35	9.28	0.02	1.00	0.003	9.3E-01
Mtr.12131.1.S1_at	8.04	7.57	8.24	8.35	8.10	8.16	7.95	0.35	8.20	0.13	1.03	0.045	3.0E-01
Mtr.46223.1.S1_s_at	2.65	2.78	3.30	3.37	3.82	3.94	2.91	0.35	3.71	0.30	1.27	0.350	3.9E-02
Mtr.49394.1.S1_at	2.44	3.03	2.42	2.42	2.41	2.40	2.63	0.35	2.41	0.01	0.92	-0.127	3.3E-01
Mtr.48438.1.S1_at	2.33	2.93	2.33	2.32	2.31	2.31	2.53	0.35	2.31	0.01	0.91	-0.129	3.4E-01
Mtr.12043.1.S1_at	8.62	9.27	9.14	9.16	9.50	9.11	9.01	0.35	9.25	0.21	1.03	0.038	3.6E-01
Mtr.45583.1.S1_at	9.90	9.21	9.55	9.62	9.67	9.78	9.55	0.35	9.69	0.08	1.01	0.021	5.3E-01
Mtr.17858.1.S1_at	8.22	7.72	8.39	8.27	8.42	8.34	8.11	0.35	8.34	0.08	1.03	0.040	3.2E-01
Mtr.6032.1.S1_at	5.86	5.85	6.46	5.79	5.34	5.19	6.06	0.35	5.44	0.31	0.90	-0.155	8.3E-02
Mtr.40105.1.S1_at	9.84	10.06	10.52	10.06	10.08	9.73	10.14	0.35	9.96	0.20	0.98	-0.026	4.7E-01
Mtr.38285.1.S1_at	8.67	8.21	8.89	8.63	8.77	8.69	8.59	0.35	8.70	0.07	1.01	0.018	6.2E-01
Mtr.24577.1.S1_at	3.07	3.62	2.98	2.99	2.95	3.56	3.22	0.35	3.17	0.34	0.98	-0.026	8.5E-01
Mtr.40444.1.S1_at	9.77	10.46	10.04	9.46	9.72	9.75	10.09	0.35	9.64	0.16	0.96	-0.065	1.1E-01
Mtr.23741.1.S1_at	8.90	8.21	8.49	8.57	8.52	8.63	8.53	0.35	8.57	0.05	1.01	0.008	8.4E-01
Mtr.13909.1.S1_at	7.12	6.42	6.78	6.47	6.50	6.52	6.77	0.35	6.50	0.03	0.96	-0.060	2.4E-01
Mtr.12322.1.S1_at	14.28	14.07	13.60	13.88	13.90	13.96	13.98	0.35	13.91	0.04	0.99	-0.007	7.4E-01
Mtr.23050.1.S1_at	8.88	8.97	9.52	9.30	9.07	9.28	9.12	0.35	9.22	0.13	1.01	0.015	6.8E-01
Mtr.38061.1.S1_at	2.17	2.76	2.15	2.14	2.15	2.14	2.36	0.35	2.14	0.01	0.91	-0.140	3.4E-01
Mtr.16080.1.S1_at	3.17	2.63	2.53	2.51	2.54	2.53	2.78	0.35	2.53	0.01	0.91	-0.137	2.8E-01
Mtr.35902.1.S1_at	11.36	12.01	11.90	11.29	11.78	11.53	11.76	0.35	11.53	0.24	0.98	-0.028	4.1E-01
Mtr.12171.1.S1_at	9.55	9.45	10.09	9.00	8.81	9.09	9.70	0.35	8.97	0.14	0.93	-0.112	2.9E-02
Mtr.20225.1.S1_at	9.86	9.17	9.43	9.57	9.65	9.58	9.49	0.35	9.60	0.04	1.01	0.017	6.0E-01
Mtr.16767.1.S1_at	8.02	7.35	7.84	8.05	7.90	7.98	7.74	0.35	7.98	0.07	1.03	0.044	3.1E-01
Mtr.41104.1.S1_at	11.85	12.40	12.50	12.01	12.29	12.04	12.25	0.35	12.11	0.15	0.99	-0.016	5.6E-01
Mtr.23698.1.S1_at	3.81	3.25	3.18	3.18	3.18	3.17	3.41	0.35	3.18	0.00	0.93	-0.103	3.0E-01
Mtr.34816.1.S1_s_at	9.18	9.36	8.69	8.75	8.70	8.89	9.08	0.35	8.78	0.10	0.97	-0.049	2.2E-01
Mtr.19861.1.S1_at	2.78	3.35	2.73	2.73	2.70	2.69	2.95	0.35	2.71	0.02	0.92	-0.126	2.8E-01
Mtr.8561.1.S1_at	10.92	11.40	11.60	11.37	11.37	11.21	11.31	0.35	11.32	0.09	1.00	0.001	9.6E-01
Mtr.5898.1.S1_s_at	7.65	7.14	7.79	7.57	7.92	7.82	7.53	0.35	7.77	0.18	1.03	0.046	3.4E-01
Mtr.5259.1.S1_at	8.78	8.68	9.32	8.81	8.67	8.73	8.93	0.35	8.74	0.07	0.98	-0.031	4.0E-01
Mtr.33931.1.S1_at	11.16	10.87	10.47	11.31	11.16	11.10	10.83	0.35	11.19	0.11	1.03	0.047	1.6E-01
Mtr.27017.1.S1_at	3.20	3.76	3.13	3.15	3.15	3.03	3.37	0.35	3.11	0.07	0.92	-0.115	2.7E-01
Mtr.17498.1.S1_x_at	2.67	3.26	2.65	2.63	2.60	2.59	2.86	0.35	2.61	0.02	0.91	-0.132	2.8E-01
Mtr.1222.1.S1_at	2.63	3.22	2.60	2.60	2.57	2.55	2.82	0.35	2.57	0.02	0.91	-0.132	2.9E-01
Mtr.5880.1.S1_at	3.52	4.10	3.48	4.41	3.75	3.72	3.70	0.35	3.96	0.39	1.07	0.098	4.3E-01
Mtr.23568.1.S1_s_at	3.77	3.09	3.56	3.61	3.57	4.08	3.47	0.35	3.75	0.29	1.08	0.113	3.4E-01
Mtr.19897.1.S1_x_at	3.48	4.03	3.40	3.36	3.46	3.26	3.64	0.35	3.36	0.10	0.92	-0.113	2.6E-01
Mtr.45210.1.S1_at	3.36	2.70	3.21	3.04	3.37	2.98	3.09	0.35	3.13	0.21	1.01	0.018	8.7E-01
Mtr.42610.1.S1_at	9.79	9.30	9.12	9.68	9.65	9.99	9.40	0.35	9.78	0.19	1.04	0.056	1.7E-01
Mtr.4965.1.S1_s_at	9.70	9.09	9.67	9.51	9.68	9.76	9.49	0.35	9.65	0.13	1.02	0.024	4.9E-01
Mtr.20271.1.S1_at	2.48	3.07	2.45	2.44	2.42	2.43	2.67	0.35	2.43	0.01	0.91	-0.135	3.0E-01
Mtr.18017.1.S1_at	11.43	11.49	12.06	11.93	12.01	11.91	11.66	0.35	11.95	0.06	1.02	0.036	2.2E-01
Mtr.26703.1.S1_s_at	9.19	8.96	9.64	9.56	9.11	9.40	9.27	0.35	9.36	0.23	1.01	0.014	7.2E-01
Mtr.14172.1.S1_at	6.43	7.01	7.05	6.67	6.36	6.70	6.83	0.35	6.57	0.19	0.96	-0.055	3.2E-01
Mtr.5168.1.S1_at	8.82	9.05	9.50	8.79	8.92	8.83	9.13	0.35	8.85	0.07	0.97	-0.045	2.4E-01
Mtr.52101.1.S1_at	9.63	8.97	9.50	9.45	9.53	9.45	9.36	0.35	9.48	0.05	1.01	0.017	6.1E-01
Mtr.32518.1.S1_at	4.13	3.46	3.94	4.12	3.93	4.44	3.84	0.35	4.16	0.26	1.08	0.116	2.6E-01
Mtr.4682.1.S1_s_at	10.42	11.11	10.70	10.55	10.74	10.76	10.74	0.35	10.68	0.12	0.99	-0.008	7.9E-01
Mtr.46560.1.S1_at	10.70	10.08	10.66	10.80	10.69	10.72	10.48	0.35	10.74	0.05	1.02	0.035	2.7E-01
Mtr.47993.1.S1_s_at	7.28	7.76	7.96	7.72	7.73	7.56	7.67	0.35	7.67	0.09	1.00	0.001	9.7E-01
Mtr.34201.1.S1_at	7.27	6.61	7.12	7.32	7.25	7.09	7.00	0.35	7.22	0.12	1.03	0.044	3.6E-01
Mtr.43915.1.S1_at	11.40	10.78	10.84	10.81	10.78	10.76	11.01	0.35	10.79	0.02	0.98	-0.029	3.3E-01
Mtr.41409.1.S1_at	9.76	9.61	10.27	10.06	10.25	9.99	9.88	0.35	10.10	0.13	1.02	0.031	3.7E-01
Mtr.28596.1.S1_at	4.70	5.37	4.89	4.89	4.87	4.76	4.99	0.35	4.84	0.07	0.97	-0.044	5.0E-01
Mtr.26240.1.S1_at	9.29	9.85	9.21	9.50	9.29	9.36	9.45	0.35	9.39	0.11	0.99	-0.010	7.8E-01
Mtr.20316.1.S1_at	10.90	10.80	11.44	11.22	11.30	11.35	11.05	0.35	11.29	0.06	1.02	0.031	3.0E-01
Mtr.15539.1.S1_s_at	8.09	8.49	8.78	8.29	8.44	8.35	8.45	0.35	8.36	0.07	0.99	-0.016	6.7E-01
Mtr.2435.1.S1_s_at	2.89	3.47	2.86	2.84	2.81	2.81	3.08	0.35	2.82	0.02	0.92	-0.125	2.7E-01
Mtr.25507.1.S1_at	3.03	3.61	2.99	2.95	2.94	2.95	3.21	0.35	2.95	0.01	0.92	-0.122	2.6E-01
Mtr.32590.1.S1_at	3.11	2.50	3.10	3.04	3.11	3.51	2.90	0.35	3.22	0.25	1.11	0.147	2.8E-01
Mtr.12135.1.S1_at	7.70	7.39	8.08	8.20	8.05	7.66	7.73	0.35	7.97	0.28	1.03	0.045	4.0E-01
Mtr.29177.1.S1_at	10.29	9.64	10.18	10.19	10.04	10.08	10.04	0.35	10.10	0.08	1.01	0.010	7.6E-01
Mtr.38056.1.S1_at	11.16	10.49	10.96	10.74	10.91	10.73	10.87	0.35	10.79	0.10	0.99	-0.010	7.3E-01
Mtr.19029.1.S1_at	6.91	6.96	6.34	7.24	7.47	7.22	6.74	0.35	7.31	0.14	1.09	0.118	5.6E-02
Mtr.33463.1.S1_s_at	6.92	6.48	7.16	6.23	6.09	6.02	6.85	0.35	6.11	0.11	0.89	-0.166	2.4E-02
Mtr.9141.1.S1_at	9.71	9.12	9.73	9.81	10.10	9.76	9.52	0.35	9.89	0.19	1.04	0.055	1.8E-01
Mtr.51883.1.S1_x_at	6.26	5.65	6.24	6.34	7.24	6.24	6.05	0.35	6.60	0.55	1.09	0.126	2.2E-01
Mtr.27406.1.S1_at	8.93	8.63	9.32	8.94	8.95	8.63	8.96	0.35	8.84	0.18	0.99	-0.020	6.2E-01
Mtr.44773.1.S1_s_at	7.84	7.26	7.87	7.84	7.80	7.85	7.66	0.35	7.83	0.03	1.02	0.032	4.4E-01
Mtr.23187.1.S1_at	8.22	7.55	8.04	8.08	8.01	7.96	7.94	0.35	8.02	0.06	1.01	0.015	7.1E-01
Mtr.32806.1.S1_at	9.09	8.95	9.61	9.33	9.41	9.41	9.22	0.35	9.38	0.04	1.02	0.026	4.5E-01
Mtr.5143.1.S1_s_at	12.41	12.93	13.07	12.75	13.15	12.83	12.81	0.35	12.91	0.21	1.01	0.012	6.8E-01
Mtr.29278.1.S1_at	5.04	4.50	5.14	5.04	5.13	5.28	4.89	0.35	5.15	0.12	1.05	0.073	2.9E-01
Mtr.46092.1.S1_at	2.73	3.34	2.75	2.71	2.72	2.71	2.94	0.35	2.71	0.01	0.92	-0.116	3.2E-01
Mtr.32003.1.S1_at	3.12	2.61	2.46	2.45	2.44	2.45	2.73	0.35	2.45	0.00	0.90	-0.159	2.3E-01
Mtr.37141.1.S1_at	8.44	7.79	8.33	8.00	8.49	8.13	8.18	0.35	8.20	0.25	1.00	0.003	9.4E-01
Mtr.2103.1.S1_at	11.29	10.63	10.77	10.75	10.39	10.87	10.90	0.35	10.67	0.25	0.98	-0.030	4.1E-01
Mtr.31854.1.S1_at	11.91	11.25	11.77	11.62	11.74	11.56	11.64	0.35	11.64	0.09	1.00	0.000	9.9E-01
Mtr.43429.1.S1_at	8.83	8.18	8.72	8.64	8.72	8.45	8.58	0.35	8.60	0.14	1.00	0.004	9.1E-01
Mtr.13147.1.S1_at	9.59	10.12	10.24	9.91	10.12	9.66	9.99	0.35	9.90	0.23	0.99	-0.013	7.3E-01
Mtr.37082.1.S1_at	2.40	2.99	2.38	2.61	2.37	2.33	2.59	0.35	2.44	0.16	0.94	-0.088	5.2E-01
Mtr.13334.1.S1_at	7.70	7.03	7.23	7.17	7.24	7.40	7.32	0.35	7.27	0.12	0.99	-0.011	8.1E-01
Mtr.25896.1.S1_at	3.15	3.71	3.08	3.09	3.03	3.03	3.31	0.35	3.05	0.03	0.92	-0.120	2.6E-01
Mtr.50627.1.S1_at	11.60	10.98	11.03	11.50	11.36	11.44	11.20	0.35	11.43	0.07	1.02	0.030	3.1E-01
Mtr.43881.1.S1_at	4.94	4.44	4.28	4.31	4.40	4.50	4.56	0.35	4.40	0.10	0.97	-0.049	5.0E-01
Mtr.42633.1.S1_at	12.03	11.36	11.83	11.82	11.74	11.73	11.74	0.35	11.76	0.05	1.00	0.003	9.2E-01
Mtr.3610.1.S1_at	7.35	7.29	7.91	7.85	7.82	7.69	7.52	0.35	7.79	0.08	1.04	0.051	2.5E-01
Mtr.4715.1.S1_at	6.91	6.36	6.99	6.81	6.86	6.96	6.76	0.35	6.88	0.08	1.02	0.025	5.9E-01
Mtr.51349.1.S1_at	2.84	3.39	2.75	2.77	2.74	2.70	2.99	0.35	2.74	0.03	0.92	-0.128	2.7E-01
Mtr.20541.1.S1_at	9.02	9.30	9.71	9.42	9.34	9.25	9.35	0.35	9.34	0.09	1.00	-0.001	9.8E-01
Mtr.27823.1.S1_at	6.29	5.64	6.18	6.17	6.29	6.38	6.04	0.34	6.28	0.10	1.04	0.057	3.1E-01
Mtr.16455.1.S1_x_at	2.97	3.55	2.94	3.52	2.74	2.92	3.15	0.34	3.06	0.41	0.97	-0.043	7.8E-01
Mtr.48789.1.S1_at	11.01	10.42	10.41	10.15	9.91	10.23	10.61	0.34	10.10	0.17	0.95	-0.072	8.1E-02
Mtr.50342.1.S1_at	5.20	4.52	4.94	4.96	5.00	4.94	4.89	0.34	4.97	0.03	1.02	0.024	7.1E-01
Mtr.17109.1.S1_s_at	12.17	11.54	12.10	12.05	11.97	12.08	11.93	0.34	12.04	0.05	1.01	0.012	6.5E-01
Mtr.13481.1.S1_at	4.43	3.82	4.40	4.39	4.99	4.37	4.22	0.34	4.58	0.35	1.09	0.120	2.7E-01
Mtr.51475.1.S1_at	3.41	2.81	3.40	3.40	3.40	3.93	3.21	0.34	3.58	0.31	1.11	0.157	2.4E-01
Mtr.3519.1.S1_at	6.79	6.11	6.57	7.24	7.48	7.27	6.49	0.34	7.33	0.13	1.13	0.175	1.7E-02
Mtr.12577.1.S1_at	8.96	8.51	9.18	8.92	9.11	8.90	8.88	0.34	8.98	0.12	1.01	0.015	6.8E-01
Mtr.20043.1.S1_s_at	2.87	3.45	2.83	2.79	2.75	2.78	3.05	0.34	2.77	0.02	0.91	-0.136	2.4E-01
Mtr.4246.1.S1_at	8.36	7.78	8.39	8.34	8.48	8.33	8.17	0.34	8.38	0.08	1.03	0.036	3.6E-01
Mtr.7559.1.S1_at	10.18	10.15	10.77	10.51	10.68	10.20	10.37	0.34	10.46	0.24	1.01	0.013	7.2E-01
Mtr.6366.1.S1_s_at	8.68	8.76	8.12	8.77	8.75	9.20	8.52	0.34	8.91	0.26	1.05	0.064	1.9E-01
Mtr.82.1.S1_at	10.50	11.10	11.10	10.72	10.68	10.79	10.90	0.34	10.73	0.05	0.98	-0.023	4.5E-01
Mtr.4419.1.S1_at	9.66	9.82	10.32	10.17	10.48	10.29	9.93	0.34	10.31	0.15	1.04	0.054	1.6E-01
Mtr.33216.1.S1_at	7.98	7.58	8.26	7.96	8.15	8.11	7.94	0.34	8.07	0.10	1.02	0.024	5.6E-01
Mtr.41563.1.S1_s_at	8.21	7.78	8.46	8.51	8.22	8.39	8.15	0.34	8.37	0.15	1.03	0.039	3.6E-01
Mtr.1592.1.S1_s_at	10.38	10.47	11.02	10.52	10.81	10.59	10.62	0.34	10.64	0.15	1.00	0.002	9.5E-01
Mtr.40942.1.S1_at	3.90	3.23	3.70	5.73	5.75	5.51	3.61	0.34	5.66	0.13	1.57	0.649	6.5E-04

Mtr.6367.1.S1_at	7.09	6.43	6.59	6.88	6.49	6.60	6.70	0.34	6.65	0.20	0.99	-0.010	8.5E-01
Mtr.35593.1.S1_at	3.10	3.68	3.06	3.04	2.93	3.01	3.28	0.34	3.00	0.06	0.91	-0.130	2.3E-01
Mtr.16998.1.S1_at	7.03	7.17	7.68	7.15	7.29	7.21	7.29	0.34	7.22	0.07	0.99	-0.015	7.2E-01
Mtr.11699.1.S1_at	8.80	8.11	8.46	8.53	8.44	8.61	8.46	0.34	8.52	0.08	1.01	0.011	7.7E-01
Mtr.45695.1.S1_s_at	3.09	3.57	2.91	3.03	3.05	3.04	3.19	0.34	3.04	0.01	0.95	-0.068	5.0E-01
Mtr.9455.1.S1_at	8.95	8.53	9.21	9.19	9.04	9.13	8.90	0.34	9.12	0.08	1.02	0.036	3.4E-01
Mtr.28933.1.S1_at	9.20	9.05	8.54	8.97	8.90	9.12	8.93	0.34	9.00	0.12	1.01	0.010	7.8E-01
Mtr.8926.1.S1_at	13.14	13.77	13.70	13.31	13.47	13.19	13.54	0.34	13.32	0.14	0.98	-0.024	3.6E-01
Mtr.41560.1.S1_at	10.21	10.79	10.81	10.24	10.53	10.15	10.60	0.34	10.31	0.20	0.97	-0.041	2.7E-01
Mtr.37505.1.S1_at	13.92	13.23	13.57	13.60	13.59	13.63	13.57	0.34	13.61	0.02	1.00	0.004	8.7E-01
Mtr.5549.1.S1_at	9.10	9.74	9.20	9.26	9.29	9.20	9.34	0.34	9.25	0.05	0.99	-0.014	6.8E-01
Mtr.12384.1.S1_at	12.05	12.73	12.46	12.27	12.68	12.31	12.41	0.34	12.42	0.23	1.00	0.001	9.8E-01
Mtr.24942.1.S1_s_at	4.51	3.91	4.49	5.03	4.97	4.14	4.30	0.34	4.71	0.50	1.10	0.132	3.0E-01
Mtr.32368.1.S1_at	8.71	9.39	8.95	8.57	8.98	8.64	9.01	0.34	8.73	0.22	0.97	-0.046	3.0E-01
Mtr.2666.1.S1_at	5.91	5.22	5.53	6.06	6.03	6.12	5.55	0.34	6.07	0.05	1.09	0.128	6.3E-02
Mtr.45795.1.S1_x_at	10.81	11.34	11.46	11.00	11.20	10.97	11.21	0.34	11.06	0.12	0.99	-0.019	5.2E-01
Mtr.44395.1.S1_at	4.74	5.31	5.36	5.19	5.21	5.27	5.14	0.34	5.22	0.04	1.02	0.024	6.9E-01
Mtr.27104.1.S1_at	9.66	9.00	9.48	9.46	9.57	9.34	9.38	0.34	9.46	0.11	1.01	0.012	7.2E-01
Mtr.2290.1.S1_at	3.92	3.31	3.90	3.88	3.90	4.07	3.71	0.34	3.95	0.11	1.07	0.091	3.1E-01
Mtr.35889.1.S1_at	2.97	2.33	2.44	3.43	3.08	2.96	2.58	0.34	3.16	0.24	1.22	0.290	7.7E-02
Mtr.27585.1.S1_at	11.20	11.51	11.88	11.34	11.39	11.25	11.53	0.34	11.33	0.07	0.98	-0.026	3.7E-01
Mtr.27093.1.S1_at	9.70	9.37	10.05	9.79	9.88	9.73	9.71	0.34	9.80	0.08	1.01	0.014	6.7E-01
Mtr.37656.1.S1_at	10.48	11.07	10.46	10.03	10.34	10.31	10.67	0.34	10.23	0.17	0.96	-0.062	1.2E-01
Mtr.23725.1.S1_at	9.48	9.06	9.74	9.46	9.48	9.60	9.43	0.34	9.51	0.08	1.01	0.013	7.0E-01
Mtr.9021.1.S1_at	8.40	8.15	7.72	8.18	8.25	8.28	8.09	0.34	8.24	0.05	1.02	0.026	5.1E-01
Mtr.34790.1.S1_at	2.51	3.10	2.50	2.49	2.46	2.45	2.71	0.34	2.47	0.02	0.91	-0.132	3.0E-01
Mtr.35152.1.S1_at	8.96	8.66	9.35	9.06	9.19	8.98	8.99	0.34	9.08	0.11	1.01	0.014	6.9E-01
Mtr.32821.1.S1_at	8.12	7.96	8.62	8.44	8.29	7.90	8.23	0.34	8.21	0.28	1.00	-0.004	9.4E-01
Mtr.41074.1.S1_at	7.13	6.83	7.51	7.85	7.77	7.93	7.15	0.34	7.85	0.08	1.10	0.133	2.7E-02
Mtr.34155.1.S1_x_at	3.17	3.58	2.90	4.44	3.88	3.57	3.21	0.34	3.96	0.44	1.23	0.302	8.0E-02
Mtr.28544.1.S1_at	9.02	8.36	8.84	9.06	8.94	8.91	8.74	0.34	8.97	0.08	1.03	0.037	3.3E-01
Mtr.7317.1.S1_at	8.18	7.79	8.47	9.23	9.19	9.11	8.15	0.34	9.18	0.06	1.13	0.172	7.0E-03
Mtr.5965.1.S1_at	7.72	7.25	7.93	7.67	7.75	7.57	7.63	0.34	7.66	0.09	1.00	0.006	8.8E-01
Mtr.18747.1.S1_at	10.81	10.38	10.14	10.41	10.36	10.46	10.44	0.34	10.41	0.05	1.00	-0.005	8.6E-01
Mtr.25927.1.S1_at	2.57	3.15	2.55	2.49	2.49	2.48	2.76	0.34	2.49	0.01	0.90	-0.148	2.5E-01
Mtr.5184.1.S1_at	8.71	8.13	8.11	8.80	8.71	8.78	8.32	0.34	8.76	0.05	1.05	0.075	9.0E-02
Mtr.8345.1.S1_at	5.54	5.01	5.66	5.50	5.79	5.52	5.40	0.34	5.60	0.16	1.04	0.053	4.1E-01
Mtr.32831.1.S1_at	9.54	8.91	9.47	9.53	9.32	9.50	9.31	0.34	9.45	0.11	1.02	0.022	5.4E-01
Mtr.5655.1.S1_s_at	4.23	3.63	4.23	4.11	4.12	4.12	4.03	0.34	4.12	0.01	1.02	0.030	6.9E-01
Mtr.29708.1.S1_at	7.97	8.52	7.89	7.78	8.10	7.89	8.13	0.34	7.92	0.16	0.97	-0.038	3.9E-01
Mtr.19642.1.S1_at	9.15	8.51	9.05	9.20	9.18	9.23	8.90	0.34	9.21	0.03	1.03	0.048	2.0E-01
Mtr.31316.1.S1_at	10.58	9.91	10.40	10.35	10.21	10.40	10.30	0.34	10.32	0.10	1.00	0.003	9.1E-01
Mtr.27428.1.S1_s_at	9.33	9.76	9.09	9.63	9.22	9.66	9.39	0.34	9.50	0.24	1.01	0.017	6.8E-01
Mtr.12236.1.S1_at	11.19	11.22	11.80	11.35	11.33	11.20	11.40	0.34	11.30	0.08	0.99	-0.014	6.2E-01
Mtr.45546.1.S1_at	9.56	10.15	10.17	9.77	9.91	9.69	9.96	0.34	9.79	0.11	0.98	-0.025	4.5E-01
Mtr.44531.1.S1_s_at	8.24	8.02	8.69	8.26	8.18	8.21	8.32	0.34	8.22	0.04	0.99	-0.017	6.4E-01
Mtr.27168.1.S1_at	7.18	6.50	6.96	7.16	7.20	7.22	6.88	0.34	7.20	0.03	1.05	0.065	1.9E-01
Mtr.37480.1.S1_at	12.07	12.36	12.75	12.45	12.39	12.43	12.39	0.34	12.42	0.03	1.00	0.004	8.9E-01
Mtr.50923.1.S1_at	3.00	3.56	2.94	2.97	2.94	2.97	3.17	0.34	2.96	0.02	0.93	-0.098	3.5E-01
Mtr.1770.1.S1_at	7.05	6.37	6.83	6.76	6.88	7.03	6.75	0.34	6.89	0.14	1.02	0.029	5.6E-01
Mtr.50368.1.S1_s_at	9.27	8.75	9.39	9.40	9.16	9.27	9.14	0.34	9.28	0.12	1.02	0.022	5.4E-01
Mtr.8092.1.S1_at	2.26	2.85	2.25	2.25	2.09	2.25	2.46	0.34	2.20	0.09	0.90	-0.159	2.8E-01
Mtr.10419.1.S1_at	12.69	12.16	12.81	12.60	12.58	12.62	12.55	0.34	12.60	0.02	1.00	0.006	8.2E-01
Mtr.46867.1.S1_at	3.22	3.77	3.14	3.14	3.09	3.16	3.37	0.34	3.13	0.03	0.93	-0.109	2.9E-01
Mtr.33129.1.S1_at	5.15	4.62	4.50	5.32	5.89	5.89	4.76	0.34	5.70	0.33	1.20	0.261	2.7E-02
Mtr.39841.1.S1_at	6.23	5.75	6.42	6.72	6.85	6.62	6.13	0.34	6.73	0.12	1.10	0.134	4.7E-02
Mtr.40861.1.S1_at	5.92	5.32	5.33	5.33	5.30	5.31	5.52	0.34	5.31	0.01	0.96	-0.055	3.5E-01
Mtr.24254.1.S1_at	2.22	2.81	2.22	2.22	2.21	2.21	2.42	0.34	2.21	0.00	0.92	-0.127	3.6E-01
Mtr.19602.1.S1_at	3.24	3.85	3.27	3.24	3.22	3.22	3.45	0.34	3.23	0.01	0.94	-0.097	3.2E-01
Mtr.38852.1.S1_at	8.35	7.94	8.62	8.36	8.47	8.14	8.30	0.34	8.32	0.17	1.00	0.004	9.2E-01
Mtr.1877.1.S1_at	9.97	9.44	10.09	9.90	9.90	9.75	9.83	0.34	9.85	0.09	1.00	0.003	9.3E-01
Mtr.5069.1.S1_at	7.28	6.64	6.74	6.89	7.06	7.13	6.89	0.34	7.02	0.12	1.02	0.029	5.5E-01
Mtr.32602.1.S1_at	9.43	9.50	8.87	9.17	9.03	9.30	9.27	0.34	9.16	0.13	0.99	-0.016	6.5E-01
Mtr.12822.1.S1_at	9.58	9.82	9.14	9.36	10.38	9.60	9.51	0.34	9.78	0.54	1.03	0.039	5.1E-01
Mtr.25542.1.S1_s_at	2.28	2.86	2.26	2.25	2.26	2.26	2.47	0.34	2.25	0.00	0.91	-0.130	3.4E-01
Mtr.39307.1.S1_at	4.60	4.29	4.97	4.95	5.19	4.83	4.62	0.34	4.99	0.18	1.08	0.111	1.7E-01
Mtr.39200.1.S1_at	10.38	9.72	10.19	10.23	10.20	10.02	10.10	0.34	10.15	0.11	1.01	0.008	8.1E-01
Mtr.16017.1.S1_at	9.95	9.69	10.37	10.20	10.03	10.20	10.01	0.34	10.14	0.10	1.01	0.020	5.4E-01
Mtr.21708.1.S1_s_at	9.38	9.75	9.06	9.32	9.27	9.39	9.40	0.34	9.33	0.06	0.99	-0.011	7.5E-01
Mtr.4053.1.S1_at	9.77	9.22	9.14	9.16	9.30	9.49	9.38	0.34	9.32	0.16	0.99	-0.009	8.0E-01
Mtr.31810.1.S1_at	8.85	8.31	8.95	8.60	8.89	8.62	8.70	0.34	8.71	0.16	1.00	0.000	9.9E-01
Mtr.9581.1.S1_at	12.32	11.67	11.80	12.09	12.11	11.93	11.93	0.34	12.04	0.10	1.01	0.014	6.1E-01
Mtr.13550.1.S1_at	7.51	7.00	7.65	7.18	7.56	7.10	7.39	0.34	7.28	0.24	0.99	-0.020	6.9E-01
Mtr.21904.1.S1_at	5.38	5.33	5.95	5.39	5.80	5.44	5.55	0.34	5.54	0.22	1.00	-0.004	9.6E-01
Mtr.29613.1.S1_at	10.86	10.33	10.97	11.28	11.12	10.71	10.72	0.34	11.04	0.29	1.03	0.042	2.9E-01
Mtr.12907.1.S1_at	7.87	7.56	8.25	8.19	8.29	8.17	7.89	0.34	8.22	0.06	1.04	0.058	1.8E-01
Mtr.31394.1.S1_s_at	10.69	10.84	11.35	11.17	11.25	11.34	10.96	0.34	11.25	0.08	1.03	0.038	2.2E-01
Mtr.17868.1.S1_at	9.06	8.86	9.53	10.22	10.55	10.26	9.15	0.34	10.34	0.18	1.13	0.177	5.8E-03
Mtr.47338.1.S1_x_at	3.03	3.62	3.03	3.01	2.98	2.97	3.23	0.34	2.99	0.02	0.92	-0.113	2.9E-01
Mtr.27868.1.S1_at	5.75	5.06	5.34	6.45	6.43	6.15	5.38	0.34	6.34	0.17	1.18	0.237	1.2E-02
Mtr.38492.1.S1_at	8.52	7.93	8.53	8.69	8.61	8.59	8.32	0.34	8.63	0.05	1.04	0.052	2.0E-01
Mtr.38618.1.S1_at	2.87	2.30	2.92	2.92	3.13	2.99	2.70	0.34	3.01	0.11	1.12	0.159	2.0E-01
Mtr.1275.1.S1_at	2.29	2.87	2.27	2.28	2.26	2.28	2.48	0.34	2.27	0.01	0.92	-0.125	3.6E-01
Mtr.18.1.S1_at	12.11	12.07	11.50	11.27	11.55	11.60	11.90	0.34	11.47	0.18	0.96	-0.052	1.3E-01
Mtr.16359.1.S1_x_at	2.99	3.55	2.93	2.90	2.87	2.85	3.16	0.34	2.87	0.03	0.91	-0.135	2.3E-01
Mtr.12965.1.S1_at	3.27	3.86	3.27	3.25	2.97	3.24	3.47	0.34	3.15	0.16	0.91	-0.136	2.3E-01
Mtr.5772.1.S1_s_at	9.93	9.39	10.03	9.93	10.08	9.99	9.78	0.34	10.00	0.08	1.02	0.031	3.5E-01
Mtr.8940.1.S1_s_at	9.46	8.79	9.00	8.98	8.89	9.03	9.08	0.34	8.97	0.08	0.99	-0.018	6.1E-01
Mtr.35244.1.S1_at	9.74	9.51	10.19	10.01	10.10	9.96	9.81	0.34	10.02	0.07	1.02	0.030	3.6E-01
Mtr.40713.1.S1_at	10.23	9.74	10.39	10.10	10.21	10.13	10.12	0.34	10.15	0.05	1.00	0.004	9.0E-01
Mtr.18178.1.S1_at	9.54	10.11	10.15	10.24	10.83	10.73	9.93	0.34	10.60	0.32	1.07	0.094	6.8E-02
Mtr.37991.1.S1_at	13.12	12.50	13.05	12.88	12.86	12.72	12.89	0.34	12.82	0.09	0.99	-0.008	7.5E-01
Mtr.631.1.S1_at	2.94	3.52	2.92	2.89	2.90	2.89	3.12	0.34	2.89	0.01	0.93	-0.111	3.1E-01
Mtr.6197.1.S1_s_at	9.87	10.11	9.43	10.45	9.53	10.27	9.80	0.34	10.08	0.49	1.03	0.041	4.6E-01
Mtr.49455.1.S1_s_at	8.98	8.30	8.69	8.81	8.89	8.62	8.66	0.34	8.77	0.14	1.01	0.019	6.1E-01
Mtr.31671.1.S1_s_at	2.56	3.11	2.48	2.47	2.45	2.46	2.72	0.34	2.46	0.01	0.91	-0.142	2.7E-01
Mtr.40831.1.S1_at	12.09	12.45	12.78	12.41	12.32	12.44	12.44	0.34	12.39	0.06	1.00	-0.005	8.3E-01
Mtr.46126.1.S1_at	2.45	3.03	2.43	2.44	2.44	2.37	2.64	0.34	2.41	0.04	0.92	-0.127	3.2E-01
Mtr.47571.1.S1_at	8.98	9.14	9.63	9.71	9.54	9.55	9.25	0.34	9.60	0.09	1.04	0.054	1.6E-01
Mtr.38499.1.S1_at	11.34	10.72	11.28	11.32	11.37	11.33	11.11	0.34	11.34	0.03	1.02	0.029	3.2E-01
Mtr.9295.1.S1_at	9.26	9.71	9.94	9.53	9.52	9.40	9.64	0.34	9.48	0.07	0.98	-0.023	4.9E-01
Mtr.31500.1.S1_at	6.02	5.39	5.94	5.88	5.25	6.18	5.78	0.34	5.77	0.48	1.00	-0.003	9.7E-01
Mtr.31769.1.S1_s_at	9.50	8.86	9.40	9.44	9.42	9.38	9.25	0.34	9.41	0.03	1.02	0.025	4.6E-01
Mtr.42149.1.S1_at	9.02	9.45	9.70	9.68	9.70	9.52	9.39	0.34	9.63	0.09	1.03	0.037	3.0E-01
Mtr.40308.1.S1_at	10.38	9.73	10.23	10.00	9.98	10.04	10.11	0.34	10.01	0.03	0.99	-0.015	6.3E-01
Mtr.12067.1.S1_at	8.92	8.88	9.49	9.02	8.89	9.04	9.10	0.34	8.98	0.08	0.99	-0.018	6.0E-01
Mtr.52100.1.S1_at	7.52	6.98	7.62	7.39	7.67	7.23	7.37	0.34	7.43	0.22	1.01	0.011	8.2E-01
Mtr.38973.1.S1_at	7.27	7.21	7.83	7.32	7.61	7.51	7.44	0.34	7.48	0.14	1.01	0.008	8.5E-01
Mtr.33703.1.S1_at	2.58	3.17	2.57	2.55	2.53	2.51	2.77	0.34	2.53	0.02	0.91	-0.131	2.9E-01
Mtr.3840.1.S1_at	2.50	3.08	2.47	2.46	2.46	2.45	2.68	0.34	2.46	0.00	0.92	-0.126	3.2E-01
Mtr.9206.1.S1_s_at	8.84	8.16	8.54	7.98	7.57	8.01	8.51	0.34	7.85	0.24	0.92	-0.117	5.2E-02
Mtr.40652.1.S1_at	10.47	9.79	10.23	10.32	10.47	10.24	10.16	0.34	10.34	0.12	1.02	0.025	4.4E-01
Mtr.38508.1.S1_at	8.27	7.90	7.58	7.92	7.98	8.19	7.92	0.34	8.03	0.14	1.01	0.020	6.3E-01
Mtr.19872.1.S1_at	10.07	9.41	9.90	9.91	9.95	9.99	9.79	0.34	9.95	0.04	1.02	0.023	4.7E-01
Mtr.7550.1.S1_at	9.38	8.72	9.22	9.34	9.20	9.07	9.11	0.34	9.20	0.14	1.01	0.015	6.8E-01
Mtr.26844.1.S1_at	2.47	3.03	2.41	2.33	2.34	2.42	2.64	0.34	2.36	0.05	0.90	-0.159	2.4E-01
Mtr.37412.1.S1_s_at	13.48	12.88	13.47	13.40	13.16	13.37	13.28	0.34	13.31	0.13	1.00	0.004	8.8E-01
Mtr.20607.1.S1_at	2.59	3.17	2.56	2.55	2.54	2.53	2.78	0.34	2.54	0.01	0.91	-0.129	2.9E-01
Mtr.37206.1.S1_at	10.28	10.33	9.71	10.39	10.24	10.35	10.10	0.34	10.33	0.07	1.02	0.031	3.3E-01
Mtr.1083.1.S1_at	9.88	9.35	9.99	9.65	9.84	9.67	9.74	0.34	9.72	0.10	1.00	-0.002	9.4E-01
Mtr.39530.1.S1_s_at	9.15	8.84	9.52	9.59	9.91	9.78	9.17	0.34	9.76	0.16	1.06	0.089	5.5E-02
Mtr.31083.1.S1_at	8.78	8.13	8.65	8.96	8.74	8.87	8.52	0.34	8.86	0.11	1.04	0.056	1.8E-01
Mtr.35752.1.S1_at	7.17	6.68	7.34	7.56	7.86	7.59	7.07	0.34	7.67	0.16	1.09	0.119	5.1E-02
Mtr.9798.1.S1_at	13.14	12.47	12.68	12.72	12.74	12.71	12.76	0.34	12.72	0.02	1.00	-0.005	8.5E-01
Mtr.49302.1.S1_at	2.52	3.11	2.52	2.50	2.49	2.49	2.72	0.34	2.49	0.01	0.92	-0.124	3.2E-01
Mtr.44704.1.S1_at	9.99	9.55	10.22	10.03	10.22	10.05	9.92	0.34	10.10	0.11	1.02	0.026	4.4E-01
Mtr.14545.1.S1_at	10.24	9.60	10.13	9.92	9.99	9.88	9.99	0.34	9.93	0.06	0.99	-0.009	7.8E-01
Mtr.1826.1.S1_at	8.81	8.23	8.83	8.98	8.98	8.91	8.62	0.34	8.96	0.04	1.04	0.055	1.7E-01
Mtr.11302.1.S1_at	8.67	8.96	9.35	8.88	8.98	8.77	8.99	0.34	8.87	0.10	0.99	-0.019	5.9E-01
Mtr.42887.1.S1_at	10.64	11.07	11.32	11.14	10.98	10.99	11.01	0.34	11.04	0.09	1.00	0.003	9.0E-01
Mtr.15650.1.S1_s_at	9.23	9.13	9.76	9.20	9.37	9.13	9.37	0.34	9.23	0.12	0.98	-0.022	5.3E-01

Mtr.40791.1.S1_at	9.04	8.60	9.27	9.03	8.69	8.92	8.97	0.34	8.88	0.17	0.99	-0.015	7.0E-01
Mtr.15547.1.S1_at	10.59	9.94	10.07	10.34	10.33	10.23	10.20	0.34	10.30	0.06	1.01	0.014	6.4E-01
Mtr.27943.1.S1_at	10.10	10.35	10.78	10.45	10.36	10.41	10.41	0.34	10.41	0.05	1.00	-0.001	9.8E-01
Mtr.33621.1.S1_at	8.41	8.37	8.98	8.57	8.60	8.65	8.59	0.34	8.61	0.04	1.00	0.003	9.3E-01
Mtr.22119.1.S1_s_at	4.27	3.66	4.22	4.20	4.17	4.16	4.05	0.34	4.18	0.02	1.03	0.044	5.6E-01
Mtr.22279.1.S1_at	4.61	4.69	5.23	5.18	5.22	4.69	4.84	0.34	5.03	0.30	1.04	0.055	5.1E-01
Mtr.50938.1.S1_at	8.82	8.35	9.01	8.91	8.95	8.87	8.73	0.34	8.91	0.04	1.02	0.030	4.0E-01
Mtr.36087.1.S1_at	9.32	9.94	9.38	9.14	8.84	9.28	9.55	0.34	9.09	0.22	0.95	-0.071	1.2E-01
Mtr.30158.1.S1_s_at	10.01	9.58	10.26	9.89	9.90	9.96	9.95	0.34	9.92	0.04	1.00	-0.004	8.9E-01
Mtr.4994.1.S1_at	11.31	10.74	11.35	10.98	11.15	11.05	11.13	0.34	11.06	0.09	0.99	-0.010	7.2E-01
Mtr.50126.1.S1_at	9.07	8.63	9.30	9.13	8.99	9.13	9.00	0.34	9.08	0.08	1.01	0.013	7.1E-01
Mtr.2125.1.S1_at	2.60	3.18	2.57	2.57	2.57	2.58	2.78	0.34	2.57	0.01	0.92	-0.114	3.4E-01
Mtr.29167.1.S1_s_at	8.03	7.86	8.52	8.33	7.93	8.17	8.14	0.34	8.14	0.20	1.00	0.001	9.9E-01
Mtr.1568.1.S1_at	10.38	10.88	11.04	10.60	10.39	10.44	10.77	0.34	10.48	0.11	0.97	-0.039	2.4E-01
Mtr.37771.1.S1_at	11.46	10.80	11.29	11.40	11.50	11.46	11.18	0.34	11.45	0.05	1.02	0.035	2.4E-01
Mtr.10560.1.S1_at	11.41	10.93	11.59	11.26	11.25	11.15	11.31	0.34	11.22	0.06	0.99	-0.011	6.8E-01
Mtr.6792.1.S1_at	2.60	2.89	3.28	2.39	2.56	2.46	2.92	0.34	2.47	0.08	0.85	-0.243	8.9E-02
Mtr.27511.1.S1_at	5.27	4.89	5.57	5.91	6.32	6.30	5.24	0.34	6.18	0.23	1.18	0.236	1.7E-02
Mtr.33200.1.S1_at	2.19	1.90	2.58	2.58	2.34	2.01	2.23	0.34	2.31	0.28	1.04	0.055	7.5E-01
Mtr.17195.1.S1_at	9.36	8.76	9.35	9.30	9.26	9.40	9.16	0.34	9.32	0.07	1.02	0.025	4.7E-01
Mtr.21327.1.S1_at	8.43	7.99	8.66	8.20	8.79	8.62	8.36	0.34	8.54	0.30	1.02	0.030	5.4E-01
Mtr.34420.1.S1_at	9.10	8.46	8.99	9.19	8.96	9.18	8.85	0.34	9.11	0.13	1.03	0.042	2.8E-01
Mtr.41060.1.S1_at	14.24	13.58	13.77	13.84	13.56	13.79	13.86	0.34	13.73	0.15	0.99	-0.014	5.7E-01
Mtr.12694.1.S1_at	11.51	10.86	11.01	11.27	11.49	11.51	11.12	0.34	11.42	0.13	1.03	0.038	2.3E-01
Mtr.48563.1.S1_at	4.97	4.83	4.32	4.92	5.07	4.68	4.71	0.34	4.89	0.20	1.04	0.055	4.7E-01
Mtr.46514.1.S1_s_at	9.35	9.93	9.96	9.59	9.57	9.42	9.75	0.34	9.53	0.10	0.98	-0.033	3.5E-01
Mtr.38504.1.S1_at	7.63	6.95	7.23	7.84	7.51	7.73	7.27	0.34	7.69	0.17	1.06	0.082	1.2E-01
Mtr.44365.1.S1_s_at	10.95	11.27	11.63	11.49	11.64	11.24	11.28	0.34	11.46	0.20	1.02	0.022	4.9E-01
Mtr.40971.1.S1_at	10.33	9.67	10.12	10.14	10.16	10.07	10.04	0.34	10.12	0.05	1.01	0.012	7.0E-01
Mtr.25643.1.S1_at	2.29	2.87	2.28	2.28	2.32	2.28	2.48	0.34	2.29	0.02	0.93	-0.112	4.0E-01
Mtr.13971.1.S1_at	7.14	6.63	7.27	7.21	7.97	7.12	7.01	0.34	7.43	0.47	1.06	0.084	2.8E-01
Mtr.16231.1.S1_s_at	9.09	8.43	8.89	9.01	8.69	9.01	8.80	0.34	8.90	0.18	1.01	0.016	6.8E-01
Mtr.5863.1.S1_at	11.60	10.96	11.07	11.11	10.90	11.20	11.21	0.34	11.07	0.15	0.99	-0.019	5.4E-01
Mtr.48510.1.S1_at	10.00	9.84	10.49	10.54	10.65	10.37	10.11	0.34	10.52	0.14	1.04	0.057	1.3E-01
Mtr.31567.1.S1_at	8.91	8.48	9.15	8.80	9.05	8.92	8.84	0.34	8.92	0.13	1.01	0.013	7.3E-01
Mtr.43865.1.S1_at	9.66	9.25	8.99	9.92	9.96	9.92	9.30	0.34	9.94	0.02	1.07	0.095	3.2E-02
Mtr.38565.1.S1_at	9.93	9.26	9.48	9.46	10.08	9.54	9.55	0.34	9.70	0.34	1.01	0.021	6.3E-01
Mtr.31806.1.S1_at	3.04	3.60	2.99	2.98	2.86	2.87	3.21	0.34	2.90	0.06	0.90	-0.145	2.0E-01
Mtr.20684.1.S1_at	10.77	10.44	11.12	10.65	10.69	10.68	10.78	0.34	10.67	0.02	0.99	-0.014	6.2E-01
Mtr.2819.1.S1_at	8.41	7.80	8.37	8.36	8.38	8.22	8.19	0.34	8.32	0.09	1.02	0.022	5.6E-01
Mtr.42157.1.S1_at	10.15	9.78	10.46	10.11	10.17	10.24	10.13	0.34	10.18	0.07	1.00	0.006	8.3E-01
Mtr.15180.1.S1_at	13.10	12.42	12.86	12.85	12.74	12.87	12.79	0.34	12.82	0.07	1.00	0.003	9.1E-01
Mtr.5210.1.S1_at	8.03	8.70	8.44	8.43	8.66	8.26	8.39	0.34	8.45	0.20	1.01	0.010	8.1E-01
Mtr.3680.1.S1_at	2.41	3.00	2.40	2.40	2.37	2.37	2.60	0.34	2.38	0.02	0.91	-0.129	3.2E-01
Mtr.32032.1.S1_at	3.57	4.13	3.51	3.53	3.53	3.52	3.73	0.34	3.53	0.01	0.94	-0.082	3.5E-01
Mtr.47337.1.S1_at	2.89	3.45	2.84	2.82	2.76	2.76	3.06	0.34	2.78	0.03	0.91	-0.138	2.3E-01
Mtr.2654.1.S1_at	4.23	3.58	3.73	4.11	4.04	4.57	3.85	0.34	4.24	0.29	1.10	0.140	2.0E-01
Mtr.4624.1.S1_at	8.82	8.28	8.90	8.80	8.61	8.81	8.67	0.34	8.74	0.11	1.01	0.012	7.4E-01
Mtr.43390.1.S1_at	10.39	11.07	10.77	11.77	12.12	12.09	10.75	0.34	11.99	0.20	1.12	0.158	5.4E-03
Mtr.38619.1.S1_at	7.72	7.73	8.32	7.78	7.75	7.77	7.93	0.34	7.77	0.02	0.98	-0.030	4.6E-01
Mtr.25171.1.S1_at	2.76	3.33	2.73	2.80	2.87	2.70	2.94	0.34	2.79	0.08	0.95	-0.073	5.1E-01
Mtr.30254.1.S1_at	9.71	9.37	10.06	9.74	9.79	9.75	9.71	0.34	9.76	0.03	1.00	0.007	8.2E-01
Mtr.40606.1.S1_at	10.84	10.24	10.81	10.63	10.71	10.65	10.63	0.34	10.66	0.04	1.00	0.005	8.6E-01
Mtr.47904.1.S1_at	8.65	8.07	8.67	8.81	8.75	8.78	8.46	0.34	8.78	0.03	1.04	0.053	1.9E-01
Mtr.7539.1.S1_at	10.09	10.02	10.64	10.31	10.23	10.23	10.25	0.34	10.26	0.04	1.00	0.001	9.8E-01
Mtr.9469.1.S1_at	9.66	9.78	10.30	9.70	9.89	9.71	9.91	0.34	9.76	0.11	0.99	-0.022	5.1E-01
Mtr.12415.1.S1_at	13.46	13.85	13.17	13.33	13.51	13.54	13.49	0.34	13.46	0.11	1.00	-0.003	8.8E-01
Mtr.43372.1.S1_at	10.42	9.75	10.00	10.36	10.42	10.47	10.06	0.34	10.41	0.05	1.04	0.050	1.5E-01
Mtr.9177.1.S1_at	11.63	11.92	12.31	12.13	12.17	12.07	11.95	0.34	12.12	0.05	1.01	0.020	4.4E-01
Mtr.36154.1.S1_s_at	8.51	8.40	9.04	8.82	8.76	8.76	8.65	0.34	8.78	0.03	1.01	0.021	5.5E-01
Mtr.33915.1.S1_at	6.56	6.53	5.95	5.94	5.93	6.24	6.34	0.34	6.04	0.18	0.95	-0.071	2.4E-01
Mtr.44588.1.S1_at	4.21	4.14	4.76	4.69	5.00	4.23	4.37	0.34	4.64	0.39	1.06	0.088	4.1E-01
Mtr.6701.1.S1_at	10.37	9.83	10.46	10.55	10.45	10.36	10.22	0.34	10.45	0.10	1.02	0.032	3.2E-01
Mtr.44745.1.S1_at	8.82	8.22	8.80	8.66	8.38	8.46	8.61	0.34	8.50	0.15	0.99	-0.019	6.3E-01
Mtr.36153.1.S1_s_at	8.89	8.86	9.46	9.32	9.41	8.95	9.07	0.34	9.23	0.24	1.02	0.025	5.5E-01
Mtr.41914.1.S1_at	6.78	6.11	6.50	6.64	6.36	6.67	6.46	0.34	6.56	0.17	1.01	0.020	7.0E-01
Mtr.11334.1.S1_s_at	9.68	9.41	9.00	9.51	9.55	9.72	9.36	0.34	9.59	0.11	1.02	0.035	3.3E-01
Mtr.43136.1.S1_at	3.10	3.69	3.09	2.98	3.18	3.40	3.29	0.34	3.19	0.21	0.97	-0.048	6.6E-01
Mtr.35558.1.S1_at	2.91	3.49	2.90	2.84	2.83	2.89	3.10	0.34	2.85	0.03	0.92	-0.121	2.7E-01
Mtr.44396.1.S1_at	10.48	10.30	10.95	9.31	8.33	9.18	10.58	0.34	8.94	0.53	0.85	-0.243	1.1E-02
Mtr.9855.1.S1_at	9.12	8.82	9.49	9.38	9.13	9.15	9.14	0.34	9.22	0.14	1.01	0.012	7.4E-01
Mtr.10226.1.S1_at	8.74	8.09	8.61	8.78	9.19	8.83	8.48	0.34	8.94	0.22	1.05	0.076	1.2E-01
Mtr.27044.1.S1_at	6.79	6.60	6.13	6.21	6.05	6.22	6.51	0.34	6.16	0.09	0.95	-0.080	1.6E-01
Mtr.43018.1.S1_at	13.71	13.41	13.03	13.24	13.33	13.23	13.38	0.34	13.27	0.06	0.99	-0.013	5.9E-01
Mtr.39822.1.S1_at	10.48	9.86	9.93	10.08	10.46	10.23	10.09	0.34	10.26	0.19	1.02	0.024	5.0E-01
Mtr.48641.1.S1_at	3.97	3.65	4.33	3.93	4.05	4.01	3.98	0.34	3.99	0.06	1.00	0.004	9.6E-01
Mtr.6960.1.S1_at	7.41	7.03	7.70	7.37	7.62	7.54	7.38	0.34	7.51	0.13	1.02	0.026	5.6E-01
Mtr.34820.1.S1_at	9.86	9.19	9.61	9.73	9.69	9.76	9.56	0.34	9.72	0.04	1.02	0.025	4.4E-01
Mtr.42402.1.S1_at	10.49	9.82	10.26	10.35	10.59	10.47	10.19	0.34	10.47	0.12	1.03	0.039	2.5E-01
Mtr.29661.1.S1_at	6.81	6.81	6.22	7.57	7.44	7.44	6.61	0.34	7.48	0.07	1.13	0.178	1.2E-02
Mtr.7444.1.S1_s_at	9.43	8.78	9.26	9.27	9.39	9.15	9.15	0.34	9.27	0.12	1.01	0.018	6.1E-01
Mtr.38915.1.S1_at	9.29	8.61	8.91	9.29	9.03	9.20	8.94	0.34	9.17	0.13	1.03	0.038	3.2E-01
Mtr.43548.1.S1_at	10.85	10.21	10.70	10.73	10.79	10.62	10.59	0.34	10.71	0.09	1.01	0.017	5.7E-01
Mtr.28112.1.S1_at	12.46	11.79	12.03	12.20	12.22	12.31	12.09	0.34	12.24	0.06	1.01	0.018	4.9E-01
Mtr.30748.1.S1_at	7.70	7.02	7.41	7.20	7.36	7.36	7.38	0.34	7.30	0.09	0.99	-0.014	7.4E-01
Mtr.51220.1.S1_at	3.06	3.62	3.02	3.02	3.01	3.01	3.23	0.34	3.01	0.01	0.93	-0.103	3.2E-01
Mtr.51334.1.S1_at	2.98	3.54	2.93	2.94	2.94	2.93	3.15	0.34	2.94	0.01	0.93	-0.102	3.3E-01
Mtr.31799.1.S1_s_at	7.91	8.08	8.57	8.40	8.53	8.45	8.19	0.34	8.46	0.07	1.03	0.048	2.4E-01
Mtr.27206.1.S1_at	6.05	6.65	6.07	7.35	7.43	7.21	6.26	0.34	7.33	0.11	1.17	0.229	6.4E-03
Mtr.252.1.S1_at	2.51	2.95	2.29	2.47	2.46	2.47	2.59	0.34	2.47	0.00	0.95	-0.068	5.7E-01
Mtr.32358.1.S1_at	8.85	8.33	8.22	8.83	8.65	8.72	8.46	0.34	8.73	0.09	1.03	0.045	2.6E-01
Mtr.13063.1.S1_at	9.49	8.84	9.33	9.13	9.21	9.08	9.22	0.34	9.14	0.07	0.99	-0.012	7.1E-01
Mtr.38934.1.S1_at	10.19	9.59	10.16	10.28	10.09	10.09	9.98	0.34	10.15	0.11	1.02	0.025	4.4E-01
Mtr.28077.1.S1_at	8.60	8.27	7.92	8.36	8.75	8.83	8.27	0.34	8.65	0.25	1.05	0.065	1.9E-01
Mtr.32501.1.S1_s_at	8.09	7.62	8.28	8.33	8.19	8.29	8.00	0.34	8.27	0.07	1.03	0.049	2.4E-01
Mtr.36986.1.S1_s_at	9.28	9.75	9.93	9.52	10.29	9.58	9.65	0.34	9.80	0.43	1.02	0.022	6.7E-01
Mtr.43124.1.S1_at	13.80	13.33	13.14	13.46	13.46	13.49	13.42	0.34	13.47	0.02	1.00	0.005	8.3E-01
Mtr.42670.1.S1_s_at	8.61	8.21	8.89	8.58	8.62	8.59	8.57	0.34	8.60	0.02	1.00	0.006	8.8E-01
Mtr.38875.1.S1_at	10.84	10.59	11.26	10.87	10.91	10.77	10.90	0.34	10.85	0.08	1.00	-0.006	8.2E-01
Mtr.35628.1.S1_at	9.55	9.06	8.90	9.69	10.00	9.89	9.17	0.34	9.86	0.16	1.08	0.105	3.3E-02
Mtr.707.1.S1_s_at	7.33	6.80	7.44	7.32	7.44	7.32	7.19	0.34	7.36	0.07	1.02	0.034	4.4E-01
Mtr.1822.1.S1_at	2.42	2.56	3.06	2.60	2.65	2.51	2.68	0.34	2.59	0.07	0.96	-0.052	6.6E-01
Mtr.44112.1.S1_at	7.39	6.99	7.66	7.36	7.35	7.47	7.35	0.34	7.39	0.07	1.01	0.009	8.3E-01
Mtr.498.1.S1_s_at	6.67	6.18	6.83	6.85	6.97	6.63	6.56	0.34	6.82	0.17	1.04	0.056	3.0E-01
Mtr.19944.1.S1_at	9.38	8.81	9.41	9.23	9.23	9.10	9.20	0.34	9.19	0.08	1.00	-0.001	9.7E-01
Mtr.51765.1.S1_x_at	2.62	3.18	2.57	2.57	2.55	2.58	2.79	0.34	2.56	0.01	0.92	-0.122	3.1E-01
Mtr.43597.1.S1_at	9.60	9.00	9.02	9.14	8.97	9.20	9.21	0.34	9.11	0.12	0.99	-0.016	6.5E-01
Mtr.36038.1.S1_at	3.07	3.58	2.93	2.88	2.85	2.84	3.19	0.34	2.86	0.02	0.90	-0.160	1.6E-01
Mtr.33744.1.S1_s_at	6.47	5.80	6.24	5.97	6.11	6.07	6.17	0.34	6.05	0.07	0.98	-0.028	5.9E-01
Mtr.2639.1.S1_at	5.54	6.06	5.43	5.27	5.97	5.44	5.68	0.34	5.56	0.37	0.98	-0.030	7.0E-01
Mtr.18854.1.S1_x_at	3.20	3.78	3.18	3.18	3.04	3.22	3.39	0.34	3.15	0.10	0.93	-0.106	3.0E-01
Mtr.40187.1.S1_at	10.39	10.05	10.73	10.29	10.54	10.44	10.39	0.34	10.42	0.12	1.00	0.004	8.9E-01
Mtr.2915.1.S1_at	8.40	7.94	8.61	8.51	8.53	8.70	8.32	0.34	8.58	0.11	1.03	0.044	2.7E-01
Mtr.9527.1.S1_at	9.02	8.90	9.54	9.09	8.94	9.01	9.16	0.34	9.01	0.07	0.98	-0.023	5.2E-01
Mtr.15684.1.S1_at	2.97	3.52	2.90	3.01	2.89	2.87	3.13	0.34	2.92	0.08	0.93	-0.099	3.6E-01
Mtr.38958.1.S1_at	9.87	9.25	9.80	9.63	9.79	9.65	9.64	0.34	9.69	0.09	1.00	0.007	8.2E-01
Mtr.42399.1.S1_at	8.96	8.54	9.21	9.30	9.19	9.06	8.90	0.34	9.19	0.12	1.03	0.045	2.5E-01
Mtr.31825.1.S1_at	2.87	3.44	2.83	2.83	2.85	2.76	3.04	0.34	2.81	0.05	0.92	-0.114	3.1E-01
Mtr.41869.1.S1_at	8.41	7.75	7.93	7.99	8.11	7.99	8.03	0.34	8.03	0.07	1.00	0.000	1.0E+00
Mtr.20492.1.S1_at	7.21	6.55	7.01	6.91	7.06	7.29	6.92	0.34	7.08	0.19	1.02	0.033	5.1E-01
Mtr.42972.1.S1_at	14.12	13.77	13.44	13.91	13.73	13.89	13.78	0.34	13.84	0.10	1.00	0.007	7.7E-01
Mtr.33771.1.S1_s_at	9.50	8.88	9.42	9.48	9.46	9.66	9.26	0.34	9.53	0.11	1.03	0.041	2.7E-01
Mtr.13032.1.S1_at	9.47	9.76	10.14	9.81	9.77	9.70	9.79	0.34	9.76	0.05	1.00	-0.005	8.8E-01
Mtr.50429.1.S1_x_at	3.66	3.70	3.09	3.13	2.99	3.25	3.48	0.34	3.12	0.13	0.90	-0.157	1.6E-01
Mtr.3182.1.S1_at	3.06	3.64	3.04	3.00	3.03	2.85	3.25	0.34	2.96	0.10	0.91	-0.135	2.3E-01
Mtr.13988.1.S1_at	4.07	4.66	4.65	4.16	4.35	4.13	4.46	0.34	4.21	0.12	0.95	-0.081	3.0E-01
Mtr.1688.1.S1_at	7.61	7.15	7.80	7.38	7.77	7.88	7.52	0.34	7.68	0.27	1.02	0.030	5.6E-01
Mtr.37310.1.S1_s_at	11.37	12.04	11.79	11.40	11.65	11.52	11.73	0.34	11.52	0.12	0.98	-0.026	3.7E-01
Mtr.47105.1.S1_at	2.56	3.13	2.54	2.53	2.49	2.49	2.74	0.34	2.51	0.02	0.91	-0.130	3.0E-01
Mtr.32493.1.S1_at	7.46	6.84	7.39	7.30	7.52	7.64	7.23	0.34	7.48	0.17	1.03	0.049	3.1E-01
Mtr.7260.1.S1_at	11.69	11.18	11.05	10.96	11.12	11.18	11.30	0.34	11.08	0.11	0.98	-0.028	3.5E-01

Mtr.41120.1.S1_at	10.77	10.18	10.75	10.84	10.78	10.78	10.57	0.34	10.80	0.03	1.02	0.032	2.9E-01
Mtr.41511.1.S1_at	10.41	9.77	9.90	10.30	10.16	10.35	10.03	0.34	10.27	0.10	1.02	0.034	3.0E-01
Mtr.40941.1.S1_at	9.52	8.88	9.38	9.45	9.61	9.58	9.26	0.34	9.55	0.09	1.03	0.044	2.3E-01
Mtr.45066.1.S1_at	10.10	9.47	9.99	9.97	9.90	10.14	9.86	0.34	10.00	0.12	1.01	0.021	5.2E-01
Mtr.26771.1.S1_s_at	7.96	7.29	7.57	8.12	8.37	8.03	7.61	0.34	8.17	0.17	1.07	0.104	6.1E-02
Mtr.38906.1.S1_at	8.83	9.50	9.12	9.07	8.83	9.03	9.15	0.34	8.98	0.13	0.98	-0.027	4.6E-01
Mtr.26642.1.S1_at	3.13	3.68	3.07	2.84	3.11	3.10	3.29	0.34	3.01	0.15	0.91	-0.128	2.6E-01
Mtr.2015.1.S1_at	10.05	9.42	9.96	9.60	9.85	9.75	9.81	0.34	9.73	0.13	0.99	-0.011	7.3E-01
Mtr.48699.1.S1_at	2.84	3.38	3.47	2.77	2.92	2.88	3.23	0.34	2.86	0.08	0.88	-0.177	1.4E-01
Mtr.47453.1.S1_at	8.65	8.21	7.99	8.43	8.67	8.75	8.28	0.34	8.62	0.17	1.04	0.057	2.0E-01
Mtr.10996.1.S1_at	8.44	8.00	8.67	8.45	8.70	8.25	8.37	0.34	8.47	0.22	1.01	0.016	7.0E-01
Mtr.9548.1.S1_at	9.32	8.73	9.30	9.28	9.17	9.33	9.12	0.34	9.26	0.08	1.02	0.022	5.2E-01
Mtr.22181.1.S1_at	2.97	3.57	2.99	2.96	2.91	2.88	3.18	0.34	2.91	0.04	0.92	-0.124	2.5E-01
Mtr.2442.1.S1_at	2.73	2.06	2.30	2.40	2.39	2.67	2.36	0.34	2.49	0.16	1.05	0.073	6.0E-01
Mtr.35966.1.S1_at	8.74	8.19	8.80	8.42	8.90	8.50	8.58	0.34	8.61	0.26	1.00	0.005	9.0E-01
Mtr.14080.1.S1_x_at	2.88	3.44	2.84	2.81	2.81	2.79	3.05	0.34	2.80	0.02	0.92	-0.124	2.7E-01
Mtr.22280.1.S1_s_at	2.93	3.51	2.93	2.91	2.86	2.82	3.12	0.34	2.86	0.05	0.92	-0.125	2.6E-01
Mtr.47179.1.S1_at	2.70	3.23	2.59	2.59	2.51	2.59	2.84	0.34	2.56	0.05	0.90	-0.148	2.3E-01
Mtr.32560.1.S1_at	7.79	7.16	7.67	7.72	7.66	7.86	7.54	0.34	7.75	0.10	1.03	0.040	3.6E-01
Mtr.25531.1.S1_at	9.31	8.92	9.60	9.22	9.38	9.33	9.27	0.34	9.31	0.08	1.00	0.005	8.7E-01
Mtr.39900.1.S1_at	1.86	2.44	1.85	1.85	1.85	1.85	2.05	0.34	1.85	0.00	0.90	-0.151	3.5E-01
Mtr.24051.1.S1_at	4.63	5.21	4.62	4.62	4.62	4.61	4.82	0.34	4.62	0.00	0.96	-0.062	3.6E-01
Mtr.28753.1.S1_at	7.54	7.72	8.19	8.19	8.29	8.26	7.82	0.34	8.25	0.06	1.05	0.077	9.6E-02
Mtr.13011.1.S1_at	9.28	8.80	9.46	9.36	9.34	9.38	9.18	0.34	9.36	0.02	1.02	0.029	4.0E-01
Mtr.9412.1.S1_at	9.75	10.30	10.36	10.19	10.16	10.16	10.14	0.34	10.17	0.02	1.00	0.004	8.8E-01
Mtr.1055.1.S1_x_at	2.63	3.20	2.61	2.58	2.58	2.56	2.81	0.34	2.57	0.01	0.91	-0.130	2.8E-01
Mtr.29927.1.S1_at	3.77	4.36	3.77	3.77	3.71	3.76	3.97	0.34	3.75	0.03	0.94	-0.083	3.2E-01
Mtr.5481.1.S1_s_at	8.01	7.80	8.46	8.45	8.17	8.28	8.09	0.34	8.30	0.14	1.03	0.037	3.8E-01
Mtr.47926.1.S1_at	2.65	3.21	2.60	2.57	2.53	2.53	2.82	0.34	2.54	0.02	0.90	-0.149	2.3E-01
Mtr.34593.1.S1_s_at	12.73	12.25	12.90	12.91	12.89	12.91	12.63	0.34	12.90	0.01	1.02	0.031	2.4E-01
Mtr.13130.1.S1_at	8.86	8.31	8.91	8.83	8.83	8.83	8.69	0.34	8.83	0.00	1.02	0.023	5.2E-01
Mtr.14903.1.S1_at	2.43	3.01	2.41	2.41	2.38	2.40	2.62	0.34	2.40	0.01	0.92	-0.127	3.2E-01
Mtr.48971.1.S1_at	8.99	9.25	9.66	9.39	9.34	9.25	9.30	0.34	9.32	0.07	1.00	0.004	9.0E-01
Mtr.9367.1.S1_at	8.79	8.21	8.80	8.84	8.76	8.90	8.60	0.34	8.83	0.07	1.03	0.038	3.1E-01
Mtr.51232.1.S1_at	12.81	12.18	12.70	12.66	12.64	12.73	12.56	0.34	12.68	0.05	1.01	0.013	5.9E-01
Mtr.21204.1.S1_x_at	7.65	6.99	7.47	7.48	7.33	7.48	7.37	0.34	7.43	0.09	1.01	0.011	7.9E-01
Mtr.39809.1.S1_s_at	4.19	3.60	3.62	3.46	3.61	4.12	3.81	0.34	3.73	0.35	0.98	-0.029	8.0E-01
Mtr.7078.1.S1_at	7.46	6.97	7.62	7.38	7.51	7.55	7.35	0.34	7.48	0.09	1.02	0.025	5.5E-01
Mtr.38040.1.S1_at	9.91	9.85	10.46	10.35	10.60	10.18	10.07	0.34	10.37	0.21	1.03	0.043	2.6E-01
Mtr.27075.1.S1_at	10.82	10.14	10.49	11.16	10.85	11.33	10.48	0.34	11.11	0.24	1.06	0.085	5.8E-02
Mtr.6010.1.S1_at	9.55	9.41	10.06	9.70	9.76	9.81	9.67	0.34	9.76	0.06	1.01	0.012	6.9E-01
Mtr.25375.1.S1_at	8.22	7.93	8.60	8.26	8.37	8.44	8.25	0.34	8.36	0.09	1.01	0.018	6.3E-01
Mtr.18253.1.S1_at	7.99	8.67	8.34	8.17	8.23	8.37	8.33	0.34	8.26	0.10	0.99	-0.014	7.2E-01
Mtr.51879.1.S1_x_at	2.91	3.45	2.83	2.83	2.77	2.79	3.07	0.34	2.80	0.03	0.91	-0.133	2.4E-01
Mtr.11659.1.S1_at	7.71	7.08	7.61	7.74	7.68	7.54	7.47	0.34	7.65	0.10	1.02	0.035	4.1E-01
Mtr.21781.1.S1_at	3.20	3.77	3.18	3.18	3.15	3.18	3.39	0.34	3.17	0.02	0.94	-0.095	3.3E-01
Mtr.11366.1.S1_at	9.12	8.59	9.22	9.12	9.30	9.01	8.97	0.34	9.14	0.15	1.02	0.027	4.7E-01
Mtr.16909.1.S1_s_at	11.92	11.50	12.16	12.23	12.18	12.30	11.86	0.34	12.24	0.06	1.03	0.045	1.3E-01
Mtr.51171.1.S1_at	4.03	3.97	4.58	3.67	4.02	4.28	4.19	0.34	3.99	0.30	0.95	-0.072	4.8E-01
Mtr.30740.1.S1_at	8.13	7.71	8.38	8.27	8.28	7.94	8.07	0.34	8.16	0.19	1.01	0.017	7.0E-01
Mtr.11476.1.S1_at	9.20	8.80	9.47	8.90	9.00	9.02	9.16	0.34	8.97	0.06	0.98	-0.030	3.9E-01
Mtr.41281.1.S1_s_at	6.81	6.72	7.34	6.93	6.95	7.17	6.96	0.34	7.02	0.13	1.01	0.012	7.9E-01
Mtr.5122.1.S1_at	5.38	5.41	5.98	5.60	6.31	5.48	5.59	0.34	5.80	0.45	1.04	0.052	5.6E-01
Mtr.13097.1.S1_at	9.92	9.78	9.28	9.91	9.88	10.14	9.66	0.34	9.97	0.14	1.03	0.046	2.1E-01
Mtr.12967.1.S1_at	6.89	6.24	6.73	6.73	6.64	6.87	6.62	0.34	6.75	0.12	1.02	0.027	5.8E-01
Mtr.27832.1.S1_at	11.63	11.02	11.56	11.55	11.68	11.64	11.40	0.34	11.62	0.07	1.02	0.028	3.3E-01
Mtr.15532.1.S1_at	8.97	8.38	8.96	8.96	8.88	8.81	8.77	0.34	8.88	0.07	1.01	0.018	6.1E-01
Mtr.39662.1.S1_at	8.66	8.37	9.04	8.87	8.59	8.66	8.69	0.34	8.71	0.15	1.00	0.002	9.5E-01
Mtr.32047.1.S1_at	7.35	6.71	7.20	7.14	7.16	6.89	7.08	0.34	7.06	0.15	1.00	-0.004	9.3E-01
Mtr.42572.1.S1_at	6.20	5.97	6.64	7.05	6.80	6.55	6.27	0.34	6.80	0.25	1.08	0.117	9.3E-02
Mtr.23120.1.S1_s_at	9.12	8.45	8.73	8.94	8.91	9.02	8.77	0.34	8.95	0.06	1.02	0.030	4.0E-01
Mtr.36439.1.S1_at	6.69	7.22	7.32	7.16	7.57	7.39	7.08	0.34	7.37	0.20	1.04	0.059	2.6E-01
Mtr.50826.1.S1_at	11.42	11.01	11.67	11.54	11.44	11.49	11.37	0.34	11.49	0.05	1.01	0.016	5.6E-01
Mtr.15922.1.S1_at	9.23	9.52	9.90	9.26	9.40	9.35	9.55	0.34	9.34	0.07	0.98	-0.032	3.5E-01
Mtr.49074.1.S1_at	2.80	3.39	2.80	2.76	2.74	2.72	3.00	0.34	2.74	0.02	0.91	-0.130	2.6E-01
Mtr.42504.1.S1_at	9.77	10.19	10.44	10.24	10.32	10.18	10.13	0.34	10.24	0.07	1.01	0.016	6.0E-01
Mtr.13060.1.S1_at	8.52	7.86	8.08	7.70	7.77	8.12	8.15	0.34	7.87	0.23	0.96	-0.052	2.9E-01
Mtr.15177.1.S1_at	2.68	3.24	2.64	2.63	2.71	2.62	2.85	0.34	2.65	0.05	0.93	-0.107	3.6E-01
Mtr.42344.1.S1_at	9.59	9.32	9.99	9.12	9.47	9.31	9.63	0.34	9.30	0.18	0.97	-0.050	2.1E-01
Mtr.11475.1.S1_at	9.99	9.36	9.88	10.12	10.03	10.13	9.74	0.34	10.09	0.05	1.04	0.051	1.5E-01
Mtr.15603.1.S1_at	10.08	9.49	10.07	10.01	10.09	10.04	9.88	0.34	10.05	0.04	1.02	0.024	4.4E-01
Mtr.22724.1.S1_at	3.17	3.71	3.09	3.06	3.01	2.99	3.32	0.34	3.02	0.03	0.91	-0.137	2.0E-01
Mtr.43230.1.S1_at	13.66	13.14	13.78	13.91	13.96	13.88	13.53	0.34	13.92	0.04	1.03	0.041	1.2E-01
Mtr.25137.1.S1_at	2.65	3.22	2.63	2.63	2.62	2.59	2.83	0.34	2.61	0.02	0.92	-0.119	3.1E-01
Mtr.44823.1.S1_at	10.39	11.05	10.83	10.83	10.90	10.81	10.76	0.34	10.85	0.05	1.01	0.012	6.7E-01
Mtr.12458.1.S1_at	12.23	11.56	11.86	12.07	11.98	12.11	11.89	0.34	12.05	0.07	1.01	0.020	4.6E-01
Mtr.37427.1.S1_at	4.00	3.41	3.99	3.99	3.99	4.20	3.80	0.34	4.06	0.13	1.07	0.096	2.8E-01
Mtr.39562.1.S1_s_at	10.26	9.61	9.77	10.12	10.25	10.38	9.88	0.34	10.25	0.13	1.04	0.053	1.5E-01
Mtr.45166.1.S1_at	2.53	3.10	2.51	2.50	2.50	2.49	2.71	0.34	2.50	0.00	0.92	-0.119	3.3E-01
Mtr.28787.1.S1_at	5.27	5.47	5.93	5.38	5.62	5.37	5.56	0.34	5.46	0.14	0.98	-0.026	6.6E-01
Mtr.34819.1.S1_at	2.11	2.69	2.10	2.10	2.09	2.09	2.30	0.34	2.09	0.00	0.91	-0.135	3.5E-01
Mtr.30051.1.S1_at	4.21	3.76	4.42	3.82	5.23	4.23	4.13	0.34	4.43	0.73	1.07	0.099	5.6E-01
Mtr.44584.1.S1_at	9.49	8.84	9.29	9.01	8.83	8.79	9.21	0.34	8.87	0.12	0.96	-0.053	1.8E-01
Mtr.38408.1.S1_s_at	11.18	10.70	11.35	11.14	11.12	11.16	11.08	0.34	11.14	0.02	1.01	0.008	7.6E-01
Mtr.12764.1.S1_at	11.51	10.90	10.95	11.53	11.27	11.47	11.12	0.34	11.42	0.14	1.03	0.039	2.2E-01
Mtr.41743.1.S1_at	9.99	9.64	10.31	10.07	10.04	10.05	9.98	0.34	10.05	0.02	1.01	0.011	7.2E-01
Mtr.15963.1.S1_at	6.61	5.99	6.51	6.17	5.91	6.16	6.37	0.34	6.08	0.15	0.95	-0.067	2.4E-01
Mtr.45144.1.S1_at	10.84	10.17	10.60	10.76	10.76	10.60	10.54	0.34	10.71	0.09	1.02	0.023	4.5E-01
Mtr.29139.1.S1_at	3.71	3.53	4.18	3.65	3.85	3.66	3.80	0.34	3.72	0.12	0.98	-0.032	7.0E-01
Mtr.14016.1.S1_at	2.52	2.51	3.10	2.42	2.63	2.48	2.71	0.34	2.51	0.11	0.93	-0.111	3.8E-01
Mtr.4247.1.S1_at	2.45	2.53	3.07	2.42	2.42	2.44	2.68	0.34	2.43	0.01	0.90	-0.146	2.5E-01
Mtr.40905.1.S1_s_at	10.48	10.05	10.72	10.53	10.53	10.58	10.42	0.34	10.55	0.03	1.01	0.018	5.5E-01
Mtr.33296.1.S1_s_at	7.93	7.31	7.41	7.67	7.93	7.71	7.55	0.34	7.77	0.14	1.03	0.042	3.5E-01
Mtr.40485.1.S1_at	9.69	9.03	9.26	9.35	9.44	9.14	9.33	0.34	9.31	0.15	1.00	-0.002	9.4E-01
Mtr.3818.1.S1_at	9.09	8.56	9.18	8.84	9.08	9.06	8.94	0.34	8.99	0.13	1.01	0.008	8.3E-01
Mtr.21977.1.S1_at	11.02	11.16	11.66	11.43	11.32	11.32	11.28	0.34	11.35	0.06	1.01	0.009	7.3E-01
Mtr.41196.1.S1_at	9.61	9.05	9.65	9.54	9.69	9.56	9.44	0.34	9.60	0.08	1.02	0.024	4.7E-01
Mtr.38684.1.S1_at	10.60	10.31	10.98	10.72	10.69	10.46	10.63	0.34	10.62	0.14	1.00	-0.001	9.7E-01
Mtr.8400.1.S1_at	7.93	7.27	7.73	7.92	7.75	7.88	7.64	0.34	7.85	0.09	1.03	0.039	3.6E-01
Mtr.38633.1.S1_at	9.68	9.12	9.73	9.33	9.41	9.51	9.51	0.34	9.41	0.09	0.99	-0.015	6.6E-01
Mtr.28569.1.S1_at	5.33	5.99	5.55	5.57	5.98	5.46	5.62	0.34	5.67	0.28	1.01	0.012	8.6E-01
Mtr.22354.1.S1_at	10.96	11.30	10.63	11.04	10.90	10.96	10.96	0.34	10.97	0.07	1.00	0.001	9.7E-01
Mtr.48710.1.S1_at	6.88	7.13	7.54	7.17	7.25	7.17	7.18	0.34	7.20	0.05	1.00	0.003	9.4E-01
Mtr.18262.1.S1_at	3.37	2.87	2.73	2.67	2.67	2.66	2.99	0.34	2.67	0.00	0.89	-0.165	1.7E-01
Mtr.5942.1.S1_s_at	10.93	10.35	10.35	10.65	10.26	10.58	10.55	0.34	10.50	0.21	1.00	-0.006	8.5E-01
Mtr.39044.1.S1_at	8.12	7.45	7.73	8.03	8.16	8.07	7.77	0.34	8.08	0.07	1.04	0.057	1.9E-01
Mtr.35540.1.S1_at	2.37	2.41	2.97	2.38	2.37	2.37	2.59	0.34	2.37	0.01	0.92	-0.125	3.3E-01
Mtr.31412.1.S1_s_at	2.69	3.22	2.59	2.58	2.56	2.58	2.83	0.34	2.57	0.01	0.91	-0.141	2.5E-01
Mtr.24033.1.S1_at	5.51	5.55	6.11	5.94	5.85	5.47	5.72	0.34	5.75	0.25	1.01	0.008	9.0E-01
Mtr.32711.1.S1_at	2.22	2.25	2.82	2.20	2.21	2.21	2.43	0.34	2.21	0.00	0.91	-0.139	3.1E-01
Mtr.8990.1.S1_at	11.57	12.00	11.34	12.57	12.61	12.71	11.64	0.34	12.63	0.07	1.09	0.118	7.4E-03
Mtr.39290.1.S1_at	8.75	8.09	8.36	8.35	8.15	8.19	8.40	0.34	8.23	0.10	0.98	-0.029	4.5E-01
Mtr.7948.1.S1_at	7.76	7.16	7.72	8.08	8.37	8.10	7.55	0.34	8.18	0.16	1.08	0.117	4.2E-02
Mtr.22413.1.S1_at	2.84	3.39	2.79	2.78	2.74	2.79	3.01	0.34	2.77	0.03	0.92	-0.120	2.9E-01
Mtr.50272.1.S1_at	12.62	12.06	12.66	12.59	12.61	12.58	12.45	0.34	12.59	0.02	1.01	0.017	4.9E-01
Mtr.44586.1.S1_at	2.79	3.35	2.75	2.75	2.75	2.74	2.96	0.34	2.75	0.01	0.93	-0.109	3.3E-01
Mtr.51996.1.S1_at	10.23	10.82	10.81	10.97	11.06	10.94	10.62	0.34	10.99	0.06	1.04	0.050	1.3E-01
Mtr.28666.1.S1_at	10.67	10.00	10.32	10.59	10.57	10.68	10.33	0.34	10.61	0.06	1.03	0.039	2.2E-01
Mtr.9823.1.S1_at	8.67	8.35	9.02	9.14	8.87	8.81	8.68	0.34	8.94	0.18	1.03	0.043	3.0E-01
Mtr.30670.1.S1_a_at	3.89	3.94	4.50	3.89	3.88	4.41	4.11	0.34	4.06	0.30	0.99	-0.018	8.6E-01
Mtr.220.1.S1_at	2.22	2.79	2.20	2.19	2.19	2.18	2.40	0.34	2.19	0.01	0.91	-0.133	3.4E-01
Mtr.6561.1.S1_at	8.87	9.52	9.35	8.84	9.30	8.89	9.25	0.34	9.01	0.25	0.97	-0.037	3.8E-01
Mtr.32689.1.S1_at	5.06	5.70	5.19	5.19	5.17	5.47	5.32	0.34	5.28	0.16	0.99	-0.011	8.6E-01
Mtr.36582.1.S1_at	3.87	3.39	4.04	3.82	3.77	3.76	3.77	0.34	3.78	0.03	1.00	0.005	9.5E-01
Mtr.17401.1.S1_at	13.01	13.53	13.63	13.43	13.67	13.43	13.39	0.34	13.51	0.14	1.01	0.013	5.9E-01
Mtr.12137.1.S1_at	2.50	3.08	2.49	2.49	2.36	2.49	2.69	0.34	2.45	0.07	0.91	-0.137	2.9E-01
Mtr.3059.1.S1_at	9.19	8.62	9.21	9.17	9.28	9.24	9.01	0.34	9.23	0.06	1.02	0.035	3.2E-01
Mtr.16207.1.S1_at	11.83	12.39	11.80	11.51	11.65	11.93	12.01	0.34	11.70	0.21	0.97	-0.037	2.5E-01
Mtr.42133.1.S1_at	7.17	7.08	7.70	7.34	7.39	7.34	7.32	0.34	7.36	0.03	1.01	0.008	8.5E-01
Mtr.50830.1.S1_s_at	9.34	8.69	9.15	9.05	8.78	9.10	9.06	0.34	8.98	0.17	0.99	-0.013	7.2E-01
Mtr.38925.1.S1_s_at	9.04	8.45	9.02	8.86	8.97	8.91	8.84	0.34	8.91	0.06	1.01	0.012	7.2E-01

Mtr.7653.1.S1_at	9.65	8.99	9.24	9.53	9.75	9.69	9.29	0.34	9.65	0.11	1.04	0.055	1.5E-01
Mtr.25930.1.S1_x_at	3.32	2.81	2.69	2.68	2.66	2.67	2.94	0.34	2.67	0.01	0.91	-0.141	2.3E-01
Mtr.1901.1.S1_at	2.89	3.46	2.86	2.84	2.80	2.79	3.07	0.34	2.81	0.03	0.92	-0.128	2.5E-01
Mtr.32405.1.S1_s_at	10.68	10.53	11.17	10.83	10.64	10.77	10.80	0.34	10.74	0.10	1.00	-0.007	8.1E-01
Mtr.50214.1.S1_s_at	7.16	7.35	7.82	7.68	7.35	7.34	7.44	0.34	7.46	0.19	1.00	0.003	9.5E-01
Mtr.23622.1.S1_at	3.37	3.12	2.71	2.69	2.68	2.69	3.06	0.34	2.69	0.00	0.88	-0.188	1.2E-01
Mtr.32587.1.S1_at	10.30	10.77	10.95	10.54	10.80	10.58	10.67	0.34	10.64	0.14	1.00	-0.005	8.8E-01
Mtr.8944.1.S1_at	11.36	10.69	11.09	11.23	11.07	11.29	11.05	0.34	11.19	0.11	1.01	0.019	5.2E-01
Mtr.37193.1.S1_at	8.20	7.68	8.31	8.24	8.21	8.13	8.06	0.34	8.19	0.06	1.02	0.023	5.4E-01
Mtr.41412.1.S1_at	7.81	7.39	8.05	7.85	7.82	7.97	7.75	0.34	7.88	0.08	1.02	0.024	5.6E-01
Mtr.43999.1.S1_at	9.30	9.89	9.86	10.00	10.19	9.94	9.68	0.34	10.04	0.13	1.04	0.053	1.6E-01
Mtr.13891.1.S1_at	6.98	6.31	6.67	6.85	6.87	7.08	6.65	0.34	6.94	0.13	1.04	0.060	2.5E-01
Mtr.43197.1.S1_at	8.45	8.10	8.77	8.00	8.28	8.07	8.44	0.34	8.12	0.15	0.96	-0.057	2.0E-01
Mtr.11632.1.S1_at	7.91	7.47	8.13	8.16	8.08	8.10	7.84	0.34	8.11	0.04	1.04	0.050	2.3E-01
Mtr.3266.1.S1_at	4.79	4.41	5.08	4.87	5.79	5.14	4.76	0.34	5.27	0.47	1.11	0.145	2.1E-01
Mtr.40996.1.S1_at	8.29	8.07	8.73	8.23	8.34	8.27	8.37	0.34	8.28	0.06	0.99	-0.015	6.8E-01
Mtr.29268.1.S1_x_at	5.88	5.40	5.24	5.20	6.17	5.66	5.51	0.34	5.68	0.49	1.03	0.044	6.4E-01
Mtr.3145.1.S1_at	7.05	6.39	6.83	6.73	7.27	6.95	6.76	0.34	6.98	0.27	1.03	0.048	4.1E-01
Mtr.13560.1.S1_at	7.63	7.85	8.29	8.03	8.01	7.79	7.92	0.34	7.94	0.13	1.00	0.004	9.3E-01
Mtr.30756.1.S1_s_at	9.21	9.22	8.64	8.79	9.12	9.17	9.02	0.34	9.03	0.21	1.00	0.001	9.8E-01
Mtr.9498.1.S1_at	8.91	8.26	8.69	8.90	8.99	9.09	8.62	0.34	8.99	0.10	1.04	0.061	1.4E-01
Mtr.22582.1.S1_at	9.99	9.38	9.45	9.57	9.36	9.50	9.61	0.33	9.47	0.11	0.99	-0.021	5.4E-01
Mtr.40635.1.S1_at	11.25	10.58	10.90	11.02	11.06	11.01	10.91	0.33	11.03	0.03	1.01	0.016	5.7E-01
Mtr.21316.1.S1_at	11.44	10.83	10.90	11.07	11.04	11.09	11.06	0.33	11.07	0.02	1.00	0.001	9.6E-01
Mtr.18594.1.S1_s_at	4.95	4.53	4.29	5.05	4.25	4.51	4.59	0.33	4.60	0.41	1.00	0.004	9.7E-01
Mtr.10531.1.S1_at	12.59	11.94	12.14	12.33	12.32	12.33	12.22	0.33	12.33	0.01	1.01	0.012	6.2E-01
Mtr.23673.1.S1_at	7.21	7.66	7.87	7.76	7.70	7.60	7.58	0.33	7.69	0.08	1.01	0.020	6.2E-01
Mtr.18303.1.S1_at	5.50	5.53	4.94	4.80	4.97	4.84	5.32	0.33	4.87	0.09	0.92	-0.127	8.8E-02
Mtr.43428.1.S1_at	8.83	8.16	8.43	8.66	8.52	8.61	8.47	0.33	8.60	0.07	1.01	0.021	5.6E-01
Mtr.15877.1.S1_at	8.41	7.92	8.56	7.83	7.69	7.84	8.30	0.33	7.79	0.08	0.94	-0.092	6.2E-02
Mtr.6657.1.S1_at	3.35	2.70	3.14	3.03	3.01	3.09	3.06	0.33	3.04	0.04	0.99	-0.009	9.3E-01
Mtr.23387.1.S1_x_at	12.19	11.96	11.53	12.36	12.40	12.50	11.89	0.33	12.42	0.07	1.04	0.063	5.5E-02
Mtr.2480.1.S1_at	6.37	7.02	6.53	7.35	7.56	7.35	6.64	0.33	7.42	0.12	1.12	0.160	1.9E-02
Mtr.42214.1.S1_at	10.02	9.46	10.06	10.12	10.00	9.98	9.85	0.33	10.03	0.08	1.02	0.026	4.1E-01
Mtr.12535.1.S1_at	12.50	12.21	12.88	12.47	12.44	12.36	12.53	0.33	12.43	0.06	0.99	-0.012	6.2E-01
Mtr.32221.1.S1_at	11.12	10.49	10.98	10.99	11.06	10.94	10.86	0.33	11.00	0.06	1.01	0.017	5.4E-01
Mtr.43125.1.S1_at	10.88	10.96	11.50	11.33	11.42	11.25	11.11	0.33	11.33	0.09	1.02	0.028	3.3E-01
Mtr.11278.1.S1_at	11.62	12.19	12.22	11.45	11.76	11.94	12.01	0.33	11.72	0.25	0.98	-0.035	2.9E-01
Mtr.15025.1.S1_s_at	2.62	3.19	2.59	2.62	2.33	2.60	2.80	0.33	2.51	0.16	0.90	-0.156	2.5E-01
Mtr.7958.1.S1_s_at	10.42	10.43	11.00	10.92	11.22	10.86	10.62	0.33	11.00	0.19	1.04	0.051	1.6E-01
Mtr.13169.1.S1_at	9.63	10.25	10.16	9.91	10.03	9.92	10.02	0.33	9.95	0.06	0.99	-0.009	7.7E-01
Mtr.21504.1.S1_a_at	2.38	2.95	2.36	2.35	2.35	2.34	2.56	0.33	2.35	0.00	0.92	-0.127	3.2E-01
Mtr.50867.1.S1_at	6.26	6.27	6.84	6.99	6.84	7.01	6.45	0.33	6.95	0.09	1.08	0.107	6.8E-02
Mtr.26400.1.S1_at	3.25	3.84	3.26	3.26	2.97	3.30	3.45	0.33	3.18	0.18	0.92	-0.119	2.8E-01
Mtr.34961.1.S1_at	9.69	9.02	9.29	9.60	9.48	9.72	9.33	0.33	9.60	0.12	1.03	0.041	2.6E-01
Mtr.29651.1.S1_at	6.72	6.99	7.39	7.29	7.03	7.34	7.03	0.33	7.22	0.17	1.03	0.038	4.4E-01
Mtr.1832.1.S1_at	4.89	4.32	4.31	4.73	4.81	5.39	4.51	0.33	4.98	0.36	1.10	0.143	1.7E-01
Mtr.32167.1.S1_at	7.36	6.82	7.44	7.29	7.23	7.30	7.21	0.33	7.27	0.04	1.01	0.012	7.6E-01
Mtr.35812.1.S1_at	8.98	8.41	8.38	8.57	8.66	8.57	8.59	0.33	8.60	0.06	1.00	0.002	9.6E-01
Mtr.43731.1.S1_at	9.34	9.13	8.69	9.35	9.07	9.34	9.05	0.33	9.25	0.16	1.02	0.031	4.1E-01
Mtr.35248.1.S1_at	11.30	11.75	11.95	11.70	11.82	11.64	11.67	0.33	11.72	0.09	1.00	0.007	7.9E-01
Mtr.10772.1.S1_at	10.68	10.57	11.20	10.80	10.86	10.89	10.82	0.33	10.85	0.04	1.00	0.004	8.8E-01
Mtr.26319.1.S1_at	6.95	6.91	6.36	7.53	7.86	7.63	6.74	0.33	7.67	0.17	1.14	0.187	1.3E-02
Mtr.45273.1.S1_at	7.64	7.30	7.97	7.81	7.90	7.74	7.64	0.33	7.82	0.08	1.02	0.034	4.1E-01
Mtr.20997.1.S1_s_at	8.25	7.72	8.33	8.30	8.24	8.24	8.10	0.33	8.26	0.04	1.02	0.029	4.5E-01
Mtr.52021.1.S1_at	8.78	8.25	8.87	8.90	8.76	8.65	8.63	0.33	8.77	0.13	1.02	0.022	5.5E-01
Mtr.10756.1.S1_at	10.92	10.44	11.09	10.96	10.82	10.81	10.82	0.33	10.86	0.08	1.00	0.006	8.4E-01
Mtr.20214.1.S1_at	8.93	8.26	8.58	8.69	8.61	8.57	8.59	0.33	8.62	0.06	1.00	0.006	8.7E-01
Mtr.45415.1.S1_at	9.03	8.42	8.96	8.98	9.34	8.90	8.80	0.33	9.07	0.23	1.03	0.044	3.1E-01
Mtr.6917.1.S1_at	5.37	5.13	5.79	5.29	5.45	5.15	5.43	0.33	5.30	0.15	0.98	-0.036	5.6E-01
Mtr.26195.1.S1_at	9.61	9.01	9.57	9.60	9.58	9.57	9.40	0.33	9.58	0.01	1.02	0.028	3.9E-01
Mtr.43118.1.S1_s_at	11.03	10.36	10.64	10.59	10.36	10.71	10.67	0.33	10.56	0.18	0.99	-0.016	6.2E-01
Mtr.52283.1.S1_at	5.11	5.49	4.82	5.10	5.33	5.11	5.14	0.33	5.18	0.13	1.01	0.011	8.6E-01
Mtr.43556.1.S1_s_at	11.14	10.55	11.12	11.04	11.01	10.94	10.93	0.33	11.00	0.05	1.01	0.008	7.6E-01
Mtr.8216.1.S1_at	10.32	10.35	10.92	10.16	10.54	10.23	10.53	0.33	10.31	0.20	0.98	-0.030	3.9E-01
Mtr.11546.1.S1_at	7.48	6.82	7.06	7.09	7.27	7.23	7.12	0.33	7.20	0.10	1.01	0.015	7.3E-01
Mtr.39435.1.S1_s_at	7.73	7.13	7.68	7.79	7.68	7.68	7.51	0.33	7.72	0.07	1.03	0.039	3.6E-01
Mtr.4885.1.S1_at	2.93	3.50	2.92	2.91	2.91	2.88	3.11	0.33	2.90	0.02	0.93	-0.103	3.3E-01
Mtr.25951.1.S1_at	2.80	2.92	3.43	2.73	2.76	2.67	3.05	0.33	2.72	0.05	0.89	-0.165	1.7E-01
Mtr.9447.1.S1_at	9.67	9.24	9.90	9.47	9.63	9.59	9.60	0.33	9.56	0.08	1.00	-0.006	8.5E-01
Mtr.11772.1.S1_at	11.78	11.19	11.22	11.42	11.54	11.37	11.40	0.33	11.44	0.09	1.00	0.005	8.4E-01
Mtr.25917.1.S1_at	2.92	3.38	2.74	2.72	2.68	2.67	3.01	0.33	2.69	0.02	0.89	-0.163	1.7E-01
Mtr.35675.1.S1_at	7.58	7.16	7.81	7.63	7.73	7.33	7.52	0.33	7.56	0.21	1.01	0.009	8.5E-01
Mtr.2951.1.S1_at	8.50	8.74	9.16	8.82	8.74	8.60	8.80	0.33	8.72	0.11	0.99	-0.013	7.2E-01
Mtr.31031.1.S1_at	2.68	3.24	2.64	2.63	2.59	2.62	2.85	0.33	2.61	0.02	0.92	-0.128	2.8E-01
Mtr.16624.1.S1_at	2.53	3.09	2.51	2.49	2.48	2.49	2.71	0.33	2.49	0.01	0.92	-0.123	3.1E-01
Mtr.17895.1.S1_at	11.39	12.04	11.87	11.42	11.68	11.38	11.76	0.33	11.49	0.16	0.98	-0.034	2.7E-01
Mtr.3018.1.S1_at	2.59	2.62	3.18	2.54	2.54	2.54	2.80	0.33	2.54	0.00	0.91	-0.138	2.6E-01
Mtr.31584.1.S1_at	7.39	6.87	7.48	7.68	7.20	7.55	7.25	0.33	7.48	0.25	1.03	0.045	3.9E-01
Mtr.27709.1.S1_at	7.87	7.25	7.33	7.83	7.66	7.97	7.48	0.33	7.82	0.15	1.04	0.063	1.9E-01
Mtr.31715.1.S1_at	10.04	10.03	10.61	10.25	10.23	10.23	10.23	0.33	10.24	0.01	1.00	0.001	9.6E-01
Mtr.28827.1.S1_at	8.69	8.41	9.08	8.99	8.93	8.82	8.73	0.33	8.91	0.08	1.02	0.030	4.0E-01
Mtr.27478.1.S1_at	7.17	6.64	7.25	7.12	7.44	7.36	7.02	0.33	7.31	0.17	1.04	0.059	2.5E-01
Mtr.15689.1.S1_at	3.79	4.32	3.70	3.67	3.62	3.80	3.94	0.33	3.70	0.10	0.94	-0.090	3.0E-01
Mtr.35230.1.S1_at	10.02	9.55	10.20	10.32	10.16	10.26	9.92	0.33	10.24	0.08	1.03	0.046	1.8E-01
Mtr.38112.1.S1_at	11.48	10.84	11.33	11.36	11.37	11.37	11.22	0.33	11.37	0.00	1.01	0.019	4.7E-01
Mtr.8814.1.S1_at	12.17	11.51	11.93	11.97	12.07	11.92	11.87	0.33	11.98	0.08	1.01	0.014	6.0E-01
Mtr.36196.1.S1_s_at	9.63	9.01	9.52	9.48	9.46	9.52	9.39	0.33	9.49	0.03	1.01	0.015	6.3E-01
Mtr.30622.1.S1_at	3.20	3.78	3.21	3.20	3.17	3.22	3.40	0.33	3.19	0.03	0.94	-0.089	3.5E-01
Mtr.42152.1.S1_at	8.26	8.40	8.90	8.49	8.54	8.53	8.52	0.33	8.52	0.03	1.00	0.000	1.0E+00
Mtr.7588.1.S1_at	10.54	9.89	10.10	10.47	10.41	10.46	10.18	0.33	10.45	0.03	1.03	0.038	2.3E-01
Mtr.42158.1.S1_at	7.34	6.83	7.45	7.35	7.38	7.53	7.20	0.33	7.42	0.10	1.03	0.042	3.4E-01
Mtr.26568.1.S1_at	2.96	3.51	2.91	2.88	2.84	2.81	3.13	0.33	2.84	0.03	0.91	-0.137	2.2E-01
Mtr.41458.1.S1_at	10.08	9.42	9.81	9.76	9.63	9.84	9.77	0.33	9.74	0.11	1.00	-0.004	9.0E-01
Mtr.13284.1.S1_at	9.53	8.90	9.41	9.50	9.14	9.35	9.28	0.33	9.33	0.18	1.01	0.008	8.3E-01
Mtr.29535.1.S1_at	11.56	10.95	11.03	11.57	11.68	11.55	11.18	0.33	11.60	0.07	1.04	0.053	1.0E-01
Mtr.36728.1.S1_at	2.70	3.27	2.69	2.68	2.68	2.68	2.89	0.33	2.68	0.00	0.93	-0.107	3.4E-01
Mtr.16614.1.S1_at	2.86	3.41	2.82	2.79	2.75	2.76	3.03	0.33	2.77	0.02	0.91	-0.131	2.4E-01
Mtr.40872.1.S1_at	9.86	9.31	9.92	9.70	9.79	9.65	9.70	0.33	9.71	0.07	1.00	0.002	9.5E-01
Mtr.31127.1.S1_s_at	6.58	6.08	6.72	6.85	6.72	6.72	6.46	0.33	6.76	0.07	1.05	0.066	2.0E-01
Mtr.37882.1.S1_at	2.96	3.59	3.09	3.74	4.02	3.69	3.21	0.33	3.82	0.18	1.19	0.249	5.0E-02
Mtr.657.1.S1_at	4.09	4.52	3.86	4.62	4.51	4.90	4.16	0.33	4.67	0.20	1.12	0.169	8.2E-02
Mtr.45304.1.S1_at	9.43	8.88	9.48	9.49	9.40	9.49	9.27	0.33	9.46	0.05	1.02	0.030	3.8E-01
Mtr.13792.1.S1_at	5.69	6.35	6.08	6.21	6.26	5.82	6.04	0.33	6.09	0.24	1.01	0.012	8.4E-01
Mtr.34822.1.S1_at	5.73	5.75	6.31	6.47	6.55	6.63	5.93	0.33	6.55	0.08	1.10	0.144	3.5E-02
Mtr.41118.1.S1_s_at	8.96	8.48	9.12	9.14	9.04	9.31	8.86	0.33	9.16	0.14	1.03	0.050	2.1E-01
Mtr.9587.1.S1_at	12.22	11.57	11.78	11.82	11.94	11.74	11.86	0.33	11.84	0.10	1.00	-0.003	9.2E-01
Mtr.10492.1.S1_x_at	12.41	12.85	12.19	12.26	12.15	12.46	12.48	0.33	12.29	0.16	0.98	-0.022	4.2E-01
Mtr.2307.1.S1_at	6.11	5.61	6.23	6.67	6.54	6.59	5.99	0.33	6.60	0.07	1.10	0.141	3.5E-02
Mtr.44962.1.S1_at	10.07	9.61	10.26	10.26	10.13	10.14	9.98	0.33	10.18	0.07	1.02	0.028	3.8E-01
Mtr.39961.1.S1_at	4.77	5.02	5.43	4.91	4.85	4.71	5.07	0.33	4.83	0.10	0.95	-0.072	2.9E-01
Mtr.38139.1.S1_s_at	9.24	8.64	8.71	9.21	9.25	9.20	8.86	0.33	9.22	0.03	1.04	0.057	1.4E-01
Mtr.46545.1.S1_at	10.35	9.76	10.33	10.17	10.24	10.15	10.15	0.33	10.19	0.05	1.00	0.006	8.5E-01
Mtr.48893.1.S1_at	3.23	3.82	3.26	3.24	3.11	3.29	3.44	0.33	3.21	0.09	0.93	-0.098	3.2E-01
Mtr.6631.1.S1_s_at	10.53	10.03	10.66	10.61	11.00	10.79	10.41	0.33	10.80	0.20	1.04	0.054	1.5E-01
Mtr.51973.1.S1_at	9.15	8.50	8.71	9.09	8.87	9.08	8.79	0.33	9.01	0.12	1.03	0.036	3.4E-01
Mtr.18168.1.S1_at	12.45	11.93	11.83	12.14	12.00	12.25	12.07	0.33	12.13	0.12	1.00	0.007	7.9E-01
Mtr.40330.1.S1_at	13.24	12.69	12.65	12.67	12.62	12.71	12.86	0.33	12.66	0.04	0.98	-0.022	3.7E-01
Mtr.33502.1.S1_s_at	8.78	8.68	9.30	9.17	8.95	9.01	8.92	0.33	9.04	0.11	1.01	0.019	5.8E-01
Mtr.9456.1.S1_at	10.02	9.83	10.48	10.16	10.21	10.10	10.11	0.33	10.16	0.06	1.00	0.006	8.3E-01
Mtr.15071.1.S1_at	7.53	7.21	7.88	7.42	7.58	7.39	7.54	0.33	7.46	0.10	0.99	-0.015	7.2E-01
Mtr.49371.1.S1_x_at	2.98	3.58	3.02	2.92	2.85	2.91	3.19	0.33	2.89	0.03	0.91	-0.142	2.0E-01
Mtr.45572.1.S1_at	3.70	3.40	3.04	3.04	2.99	3.36	3.38	0.33	3.13	0.20	0.93	-0.109	3.3E-01
Mtr.31443.1.S1_at	2.55	3.11	2.52	2.51	2.52	2.47	2.73	0.33	2.50	0.03	0.92	-0.126	3.0E-01
Mtr.8800.1.S1_at	10.58	9.93	10.41	10.53	10.46	10.42	10.31	0.33	10.47	0.05	1.02	0.023	4.4E-01
Mtr.44515.1.S1_at	9.62	9.86	10.27	9.96	10.01	9.85	9.92	0.33	9.94	0.08	1.00	0.003	9.2E-01
Mtr.8645.1.S1_at	9.47	9.03	9.69	10.00	10.34	10.15	9.40	0.33	10.16	0.17	1.08	0.113	2.4E-02
Mtr.28761.1.S1_at	8.45	7.94	8.57	8.59	8.39	8.59	8.32	0.33	8.52	0.11	1.02	0.034	3.8E-01
Mtr.44184.1.S1_at	10.71	10.22	10.86	10.55	10.58	10.59	10.60	0.33	10.57	0.02	1.00	-0.003	9.1E-01
Mtr.45775.1.S1_at	2.50	3.07	2.48	2.46	2.45	2.45	2.68	0.33	2.46	0.00	0.91	-0.128	3.0E-01
Mtr.1931.1.S1_at	11.44	10.80	10.97	11.09	11.21	11.13	11.07	0.33	11.14	0.06	1.01	0.010	7.2E-01

Mtr.12572.1.S1_at	12.20	11.59	12.12	11.94	11.73	11.92	11.97	0.33	11.86	0.12	0.99	-0.013	6.3E-01
Mtr.15228.1.S1_at	11.21	11.51	11.87	11.80	12.06	11.59	11.53	0.33	11.82	0.24	1.02	0.036	2.9E-01
Mtr.2817.1.S1_at	2.68	2.27	2.93	2.64	2.64	2.61	2.62	0.33	2.63	0.02	1.00	0.001	9.9E-01
Mtr.40701.1.S1_at	9.10	8.65	9.30	9.43	9.24	9.17	9.02	0.33	9.28	0.13	1.03	0.042	2.7E-01
Mtr.47959.1.S1_at	3.61	3.52	3.00	5.98	6.04	5.86	3.38	0.33	5.96	0.09	1.76	0.819	2.0E-04
Mtr.11063.1.S1_at	10.49	9.83	10.20	10.39	10.38	10.32	10.18	0.33	10.36	0.04	1.02	0.026	3.9E-01
Mtr.51958.1.S1_x_at	2.20	2.78	2.21	2.19	2.16	2.19	2.40	0.33	2.18	0.02	0.91	-0.138	3.2E-01
Mtr.46176.1.S1_at	13.40	12.75	13.19	13.26	13.22	13.19	13.11	0.33	13.22	0.03	1.01	0.012	6.0E-01
Mtr.39817.1.S1_at	10.32	9.88	10.54	10.28	10.37	10.26	10.25	0.33	10.31	0.06	1.01	0.008	7.8E-01
Mtr.44907.1.S1_at	9.97	9.45	10.06	9.88	9.92	9.83	9.83	0.33	9.88	0.05	1.01	0.007	8.1E-01
Mtr.47854.1.S1_at	10.17	9.62	10.22	9.74	9.75	9.77	10.01	0.33	9.75	0.01	0.97	-0.037	2.6E-01
Mtr.21538.1.S1_at	8.90	8.28	8.81	8.77	8.65	8.67	8.66	0.33	8.70	0.06	1.00	0.006	8.7E-01
Mtr.39650.1.S1_at	9.43	8.85	9.42	9.60	9.54	9.48	9.23	0.33	9.54	0.06	1.03	0.048	1.9E-01
Mtr.11773.1.S1_s_at	12.40	11.75	12.20	12.23	12.49	12.47	12.11	0.33	12.40	0.14	1.02	0.033	2.5E-01
Mtr.25524.1.S1_at	2.36	1.80	2.39	2.40	3.19	2.34	2.19	0.33	2.64	0.47	1.21	0.274	2.4E-01
Mtr.30796.1.S1_at	8.07	7.40	7.71	7.41	8.30	7.68	7.73	0.33	7.80	0.46	1.01	0.013	8.4E-01
Mtr.10476.1.S1_at	12.87	13.34	13.52	13.35	13.42	13.39	13.24	0.33	13.38	0.03	1.01	0.015	5.0E-01
Mtr.21621.1.S1_at	3.64	4.20	3.60	3.66	3.55	3.61	3.82	0.33	3.61	0.06	0.95	-0.081	3.5E-01
Mtr.36624.1.S1_at	3.24	2.60	3.08	3.24	3.18	3.54	2.97	0.33	3.32	0.19	1.12	0.158	2.0E-01
Mtr.39624.1.S1_at	5.91	6.18	6.57	6.52	6.77	6.22	6.22	0.33	6.50	0.27	1.04	0.062	3.3E-01
Mtr.18521.1.S1_at	10.84	10.34	10.21	10.55	11.45	10.95	10.47	0.33	10.98	0.45	1.05	0.070	1.9E-01
Mtr.29841.1.S1_at	8.11	7.53	8.11	8.11	8.16	7.99	7.91	0.33	8.09	0.09	1.02	0.031	4.3E-01
Mtr.33198.1.S1_at	3.42	2.83	3.39	4.38	4.24	4.21	3.21	0.33	4.28	0.09	1.33	0.412	5.9E-03
Mtr.43924.1.S1_at	9.60	8.94	9.33	9.57	9.53	9.63	9.29	0.33	9.58	0.05	1.03	0.044	2.1E-01
Mtr.14129.1.S1_at	9.93	9.94	10.51	10.08	10.22	10.07	10.13	0.33	10.12	0.08	1.00	-0.001	9.9E-01
Mtr.49786.1.S1_x_at	3.12	3.66	3.06	3.03	2.96	4.22	3.28	0.33	3.40	0.71	1.04	0.053	8.0E-01
Mtr.36381.1.S1_at	8.97	8.39	8.96	8.78	8.91	8.76	8.78	0.33	8.82	0.08	1.00	0.007	8.4E-01
Mtr.31958.1.S1_at	2.46	3.03	2.45	2.44	2.44	2.43	2.65	0.33	2.44	0.00	0.92	-0.118	3.4E-01
Mtr.29413.1.S1_at	8.67	8.04	8.16	8.62	8.43	8.98	8.29	0.33	8.67	0.28	1.05	0.065	2.0E-01
Mtr.49890.1.S1_at	3.03	3.58	2.99	2.96	2.96	2.96	3.20	0.33	2.96	0.00	0.92	-0.113	2.8E-01
Mtr.18410.1.S1_at	12.75	13.25	13.38	13.25	13.22	13.26	13.13	0.33	13.24	0.02	1.01	0.013	5.7E-01
Mtr.26446.1.S1_at	8.32	7.80	8.41	8.09	8.26	7.94	8.18	0.33	8.10	0.16	0.99	-0.014	7.2E-01
Mtr.49053.1.S1_s_at	9.17	8.53	9.01	8.91	8.82	9.02	8.90	0.33	8.92	0.10	1.00	0.002	9.4E-01
Mtr.12779.1.S1_at	7.05	7.40	7.71	7.37	7.44	6.95	7.39	0.33	7.25	0.26	0.98	-0.027	6.1E-01
Mtr.12737.1.S1_at	10.04	10.12	10.65	10.47	10.52	10.46	10.27	0.33	10.48	0.04	1.02	0.030	3.3E-01
Mtr.50490.1.S1_at	2.93	2.82	3.44	2.87	2.87	2.86	3.06	0.33	2.87	0.01	0.94	-0.096	3.6E-01
Mtr.49251.1.S1_at	10.69	10.23	10.88	10.63	10.29	10.53	10.60	0.33	10.48	0.17	0.99	-0.017	6.0E-01
Mtr.39988.1.S1_at	9.44	8.88	9.47	9.38	9.25	9.06	9.26	0.33	9.23	0.16	1.00	-0.005	8.8E-01
Mtr.43698.1.S1_s_at	3.24	2.96	2.58	2.56	2.55	2.53	2.93	0.33	2.55	0.01	0.87	-0.201	1.2E-01
Mtr.26048.1.S1_at	10.03	9.83	10.47	10.16	10.01	9.93	10.11	0.33	10.03	0.12	0.99	-0.011	7.3E-01
Mtr.38720.1.S1_at	12.28	11.63	11.84	11.93	11.93	11.98	11.91	0.33	11.95	0.03	1.00	0.004	8.8E-01
Mtr.18820.1.S1_at	3.09	3.34	3.75	3.08	3.07	4.01	3.39	0.33	3.39	0.54	1.00	-0.002	9.9E-01
Mtr.8567.1.S1_at	13.27	13.75	13.11	13.31	13.09	13.22	13.38	0.33	13.20	0.11	0.99	-0.019	4.4E-01
Mtr.27386.1.S1_at	4.49	4.52	5.08	5.45	5.44	4.64	4.70	0.33	5.18	0.46	1.10	0.140	2.2E-01
Mtr.15637.1.S1_at	2.93	3.48	2.89	2.84	2.93	2.77	3.10	0.33	2.85	0.08	0.92	-0.122	2.7E-01
Mtr.28523.1.S1_at	9.90	10.17	9.51	8.27	8.66	8.47	9.86	0.33	8.47	0.19	0.86	-0.220	3.3E-03
Mtr.24155.1.S1_s_at	2.97	3.52	2.92	2.89	2.91	2.82	3.14	0.33	2.87	0.05	0.92	-0.126	2.5E-01
Mtr.13500.1.S1_at	8.78	8.88	9.40	9.12	8.81	8.97	9.02	0.33	8.97	0.15	0.99	-0.008	8.2E-01
Mtr.7135.1.S1_at	13.04	13.19	13.68	13.53	13.38	13.09	13.30	0.33	13.33	0.22	1.00	0.003	9.0E-01
Mtr.45617.1.S1_s_at	12.29	12.63	12.96	12.67	13.18	12.72	12.63	0.33	12.86	0.28	1.02	0.026	4.1E-01
Mtr.23071.1.S1_at	5.04	5.62	5.06	5.04	5.03	4.92	5.24	0.33	5.00	0.06	0.95	-0.068	2.8E-01
Mtr.43568.1.S1_at	12.56	11.91	12.11	12.51	12.60	12.63	12.19	0.33	12.58	0.06	1.03	0.045	1.2E-01
Mtr.37169.1.S1_at	6.53	5.87	6.12	6.12	6.04	6.12	6.17	0.33	6.10	0.05	0.99	-0.018	7.1E-01
Mtr.9539.1.S1_at	9.77	9.77	10.35	10.01	9.87	9.87	9.96	0.33	9.92	0.08	1.00	-0.007	8.3E-01
Mtr.25667.1.S1_s_at	9.14	8.48	8.82	8.97	8.89	9.16	8.81	0.33	9.01	0.14	1.02	0.032	4.0E-01
Mtr.46500.1.S1_at	3.00	3.54	2.94	2.93	2.63	3.06	3.16	0.33	2.87	0.22	0.91	-0.136	2.8E-01
Mtr.40784.1.S1_at	11.49	10.84	11.05	10.85	10.97	10.82	11.13	0.33	10.88	0.08	0.98	-0.033	2.8E-01
Mtr.35653.1.S1_at	9.03	9.42	9.69	9.36	9.53	9.47	9.38	0.33	9.45	0.09	1.01	0.011	7.4E-01
Mtr.23512.1.S1_at	2.76	3.31	2.71	2.69	2.70	2.69	2.93	0.33	2.69	0.00	0.92	-0.120	2.9E-01
Mtr.29755.1.S1_at	5.89	6.52	6.39	5.18	6.02	5.94	6.27	0.33	5.71	0.46	0.91	-0.134	1.7E-01
Mtr.23091.1.S1_at	2.67	3.25	2.67	2.63	2.63	2.62	2.86	0.33	2.63	0.01	0.92	-0.124	2.9E-01
Mtr.6465.1.S1_at	2.55	3.10	2.51	2.51	2.49	2.48	2.72	0.33	2.49	0.01	0.92	-0.126	3.0E-01
Mtr.28939.1.S1_s_at	9.35	8.98	8.69	9.10	9.15	9.33	9.00	0.33	9.19	0.12	1.02	0.030	4.0E-01
Mtr.13172.1.S1_at	9.05	8.41	8.60	8.72	8.72	8.78	8.69	0.33	8.74	0.04	1.01	0.009	8.0E-01
Mtr.2107.1.S1_at	6.62	7.17	6.59	6.57	6.25	6.63	6.79	0.33	6.48	0.20	0.95	-0.068	2.4E-01
Mtr.32735.1.S1_at	5.73	5.73	5.15	6.16	6.89	6.33	5.54	0.33	6.46	0.38	1.17	0.222	3.4E-02
Mtr.18274.1.S1_at	4.94	4.74	4.30	4.56	4.51	4.65	4.66	0.33	4.57	0.07	0.98	-0.028	6.7E-01
Mtr.10426.1.S1_at	13.35	13.76	14.01	13.72	13.73	13.69	13.70	0.33	13.71	0.02	1.00	0.001	9.6E-01
Mtr.18046.1.S1_x_at	3.02	3.55	2.95	2.94	2.87	2.87	3.17	0.33	2.89	0.04	0.91	-0.134	2.2E-01
Mtr.50496.1.S1_at	9.54	9.06	9.69	9.61	9.59	9.66	9.43	0.33	9.62	0.04	1.02	0.029	3.7E-01
Mtr.26361.1.S1_at	8.27	8.41	8.90	8.86	8.89	8.70	8.53	0.33	8.81	0.10	1.03	0.048	2.2E-01
Mtr.50678.1.S1_at	8.77	8.35	9.00	8.76	8.65	8.71	8.71	0.33	8.70	0.05	1.00	0.000	9.9E-01
Mtr.32359.1.S1_s_at	10.16	10.02	10.65	10.32	10.27	10.33	10.27	0.33	10.31	0.03	1.00	0.005	8.7E-01
Mtr.25696.1.S1_at	2.33	2.89	2.31	2.30	2.24	2.30	2.51	0.33	2.28	0.03	0.91	-0.138	3.0E-01
Mtr.3108.1.S1_at	3.47	2.82	3.25	3.25	3.21	3.72	3.18	0.33	3.39	0.29	1.07	0.095	4.4E-01
Mtr.37583.1.S1_at	12.24	12.66	12.90	12.41	12.56	12.31	12.60	0.33	12.42	0.12	0.99	-0.020	4.4E-01
Mtr.5607.1.S1_at	2.95	3.49	2.89	2.97	2.82	2.93	3.11	0.33	2.91	0.08	0.93	-0.097	3.6E-01
Mtr.28236.1.S1_s_at	8.78	8.42	9.08	9.04	9.05	8.95	8.76	0.33	9.02	0.05	1.03	0.042	2.6E-01
Mtr.40203.1.S1_at	13.13	12.60	13.20	13.21	13.37	13.14	12.98	0.33	13.24	0.12	1.02	0.029	2.7E-01
Mtr.12888.1.S1_at	9.35	8.80	9.41	9.48	9.59	9.26	9.19	0.33	9.45	0.17	1.03	0.040	2.9E-01
Mtr.37009.1.S1_at	9.36	9.00	9.67	9.45	9.61	9.28	9.34	0.33	9.45	0.17	1.01	0.016	6.5E-01
Mtr.2436.1.S1_at	6.07	5.94	6.57	6.23	6.33	6.11	6.19	0.33	6.22	0.11	1.00	0.007	8.9E-01
Mtr.39597.1.S1_at	9.56	9.08	9.71	9.58	9.62	9.51	9.45	0.33	9.57	0.05	1.01	0.018	5.7E-01
Mtr.12372.1.S1_a_at	11.00	10.33	10.67	10.06	10.25	10.19	10.67	0.33	10.17	0.10	0.95	-0.069	6.7E-02
Mtr.17499.1.S1_at	7.96	7.70	8.36	8.31	8.54	8.10	8.01	0.33	8.32	0.22	1.04	0.055	2.5E-01
Mtr.45516.1.S1_at	9.44	8.84	9.38	9.43	9.53	9.51	9.22	0.33	9.49	0.05	1.03	0.041	2.4E-01
Mtr.38424.1.S1_at	10.30	9.77	10.38	10.16	10.49	10.33	10.15	0.33	10.33	0.17	1.02	0.025	4.6E-01
Mtr.45299.1.S1_at	8.57	8.10	8.74	8.53	8.55	8.59	8.47	0.33	8.56	0.03	1.01	0.015	6.7E-01
Mtr.13442.1.S1_at	10.48	9.82	10.20	9.81	10.18	9.87	10.17	0.33	9.95	0.20	0.98	-0.031	3.9E-01
Mtr.14617.1.S1_at	9.85	9.53	10.19	9.82	9.74	9.80	9.86	0.33	9.78	0.04	0.99	-0.011	7.2E-01
Mtr.43473.1.S1_at	9.72	9.06	9.44	9.36	9.62	9.57	9.41	0.33	9.52	0.14	1.01	0.017	6.2E-01
Mtr.45316.1.S1_at	11.11	10.46	10.88	11.06	10.91	11.07	10.82	0.33	11.01	0.09	1.02	0.026	3.8E-01
Mtr.10180.1.S1_at	4.79	4.87	5.40	4.83	4.77	4.66	5.02	0.33	4.76	0.09	0.95	-0.078	2.5E-01
Mtr.4612.1.S1_at	12.99	12.48	13.10	12.65	12.63	12.48	12.86	0.33	12.59	0.10	0.98	-0.031	2.5E-01
Mtr.45794.1.S1_at	3.29	2.76	2.68	2.67	3.06	2.66	2.91	0.33	2.80	0.23	0.96	-0.056	6.6E-01
Mtr.8919.1.S1_at	10.97	10.32	10.55	10.71	10.74	10.88	10.61	0.33	10.78	0.09	1.02	0.022	4.6E-01
Mtr.41769.1.S1_at	9.85	9.57	10.23	9.95	10.04	9.90	9.88	0.33	9.96	0.07	1.01	0.011	7.1E-01
Mtr.19081.1.S1_at	10.56	10.22	10.88	10.75	10.76	10.69	10.55	0.33	10.73	0.04	1.02	0.024	4.0E-01
Mtr.41987.1.S1_at	9.18	8.73	9.38	9.25	9.24	9.03	9.10	0.33	9.17	0.12	1.01	0.012	7.3E-01
Mtr.13426.1.S1_at	10.54	10.08	10.72	10.64	10.81	10.54	10.45	0.33	10.66	0.13	1.02	0.030	3.5E-01
Mtr.8936.1.S1_at	10.91	10.25	10.48	10.77	10.79	10.79	10.55	0.33	10.78	0.01	1.02	0.032	2.9E-01
Mtr.26761.1.S1_at	2.92	3.47	2.87	2.84	2.79	2.78	3.09	0.33	2.81	0.03	0.91	-0.136	2.2E-01
Mtr.42237.1.S1_at	9.57	9.97	10.23	9.90	9.98	9.83	9.92	0.33	9.90	0.08	1.00	-0.003	9.3E-01
Mtr.43500.1.S1_at	6.40	5.76	6.21	5.94	6.08	6.08	6.12	0.33	6.03	0.08	0.99	-0.021	6.7E-01
Mtr.13170.1.S1_at	9.06	9.23	9.70	9.34	9.42	9.44	9.33	0.33	9.40	0.05	1.01	0.011	7.3E-01
Mtr.13715.1.S1_at	7.84	8.38	7.77	7.91	8.21	8.12	8.00	0.33	8.08	0.15	1.01	0.015	7.0E-01
Mtr.15656.1.S1_at	3.82	3.35	3.18	2.72	2.72	3.26	3.45	0.33	2.90	0.31	0.84	-0.253	1.0E-01
Mtr.52296.1.S1_x_at	5.87	5.24	5.72	5.66	5.72	5.94	5.61	0.33	5.77	0.15	1.03	0.042	4.7E-01
Mtr.49320.1.S1_s_at	7.11	6.46	6.67	6.83	6.77	6.76	6.75	0.33	6.79	0.04	1.01	0.008	8.5E-01
Mtr.23657.1.S1_at	4.87	4.38	4.24	5.22	4.93	4.44	4.50	0.33	4.86	0.40	1.08	0.113	2.9E-01
Mtr.47412.1.S1_at	7.97	8.16	7.51	8.81	8.52	8.79	7.88	0.33	8.70	0.16	1.10	0.144	1.8E-02
Mtr.44073.1.S1_at	9.57	9.17	8.91	9.11	9.04	9.25	9.22	0.33	9.14	0.11	0.99	-0.013	7.0E-01
Mtr.12748.1.S1_at	14.75	14.90	14.27	14.63	14.68	14.69	14.64	0.33	14.67	0.03	1.00	0.002	9.1E-01
Mtr.21260.1.S1_at	3.77	3.52	3.11	3.33	3.39	3.38	3.47	0.33	3.37	0.03	0.97	-0.042	6.3E-01
Mtr.52168.1.S1_at	2.98	3.19	3.63	3.06	2.85	2.87	3.27	0.33	2.93	0.11	0.90	-0.158	1.7E-01
Mtr.7421.1.S1_at	8.03	7.39	7.57	8.17	7.79	8.08	7.66	0.33	8.01	0.20	1.05	0.064	1.9E-01
Mtr.34924.1.S1_at	9.16	8.53	9.01	9.29	9.07	9.33	8.90	0.33	9.23	0.14	1.04	0.052	1.9E-01
Mtr.42365.1.S1_at	10.04	9.40	9.83	9.86	9.91	9.83	9.76	0.33	9.87	0.04	1.01	0.016	5.9E-01
Mtr.38557.1.S1_at	11.27	10.61	10.96	11.08	10.82	11.07	10.95	0.33	10.99	0.15	1.00	0.006	8.5E-01
Mtr.35952.1.S1_at	9.40	8.89	9.50	9.19	9.50	9.31	9.26	0.33	9.33	0.16	1.01	0.011	7.5E-01
Mtr.30698.1.S1_at	9.43	9.85	9.20	8.49	8.75	8.70	9.49	0.33	8.64	0.14	0.91	-0.135	1.5E-02
Mtr.26370.1.S1_at	13.35	13.79	13.99	13.72	13.70	13.60	13.71	0.33	13.67	0.07	1.00	-0.004	8.6E-01
Mtr.38050.1.S1_at	11.31	10.65	11.05	10.97	10.95	11.03	11.00	0.33	10.98	0.04	1.00	-0.002	9.3E-01
Mtr.14872.1.S1_at	3.08	2.60	2.45	2.50	2.50	2.50	2.71	0.33	2.50	0.00	0.92	-0.115	3.4E-01
Mtr.35758.1.S1_at	8.67	8.22	8.87	8.70	8.98	8.74	8.59	0.33	8.81	0.15	1.03	0.036	3.6E-01
Mtr.49490.1.S1_x_at	2.11	2.77	2.43	2.41	2.41	2.40	2.44	0.33	2.41	0.00	0.99	-0.019	8.8E-01
Mtr.2550.1.S1_at	3.53	2.88	3.33	3.82	3.28	3.48	3.25	0.33	3.52	0.27	1.09	0.118	3.3E-01
Mtr.35341.1.S1_at	9.10	9.54	9.74	9.35	9.49	9.28	9.46	0.33	9.38	0.11	0.99	-0.013	7.0E-01
Mtr.41753.1.S1_at	10.10	9.45	9.85	9.88	9.89	9.84	9.80	0.33	9.87	0.03	1.01	0.011	7.3E-01
Mtr.4656.1.S1_at	10.59	9.98	10.50	10.71	10.68	10.46	10.36	0.33	10.62	0.14	1.02	0.035	2.8E-01
Mtr.15979.1.S1_s_at	3.09	3.64	3.05	3.07	3.17	3.07	3.26	0.33	3.11	0.06	0.95	-0.072	4.6E-01

Mtr.51364.1.S1_at	2.50	3.07	2.49	2.54	2.47	2.46	2.69	0.33	2.49	0.04	0.93	-0.109	3.7E-01
Mtr.10444.1.S1_at	13.69	13.57	13.07	13.44	13.57	13.53	13.44	0.33	13.51	0.07	1.01	0.008	7.3E-01
Mtr.34830.1.S1_at	4.49	4.45	5.04	4.46	4.95	4.47	4.66	0.33	4.63	0.28	0.99	-0.010	9.0E-01
Mtr.35791.1.S1_s_at	9.82	9.38	10.02	10.01	10.27	10.08	9.74	0.33	10.12	0.14	1.04	0.055	1.4E-01
Mtr.15514.1.S1_at	2.21	2.78	2.21	2.21	2.20	2.20	2.40	0.33	2.20	0.00	0.92	-0.127	3.5E-01
Mtr.5301.1.S1_at	7.90	7.25	7.54	7.79	7.63	8.04	7.56	0.33	7.82	0.21	1.03	0.048	3.2E-01
Mtr.38523.1.S1_at	9.94	9.34	9.87	9.86	9.85	9.83	9.72	0.33	9.85	0.01	1.01	0.019	5.3E-01
Mtr.43882.1.S1_at	7.89	7.40	8.02	7.88	8.19	8.08	7.77	0.33	8.05	0.16	1.04	0.051	2.6E-01
Mtr.27521.1.S1_s_at	8.02	7.66	8.32	8.34	8.16	8.35	8.00	0.33	8.29	0.11	1.04	0.050	2.3E-01
Mtr.43399.1.S1_at	11.56	11.02	11.61	11.62	11.67	11.54	11.40	0.33	11.61	0.06	1.02	0.027	3.3E-01
Mtr.12323.1.S1_at	11.57	10.98	11.03	11.30	11.39	11.29	11.19	0.33	11.33	0.06	1.01	0.017	5.2E-01
Mtr.18022.1.S1_at	2.66	3.22	2.63	2.62	2.60	2.62	2.84	0.33	2.61	0.01	0.92	-0.118	3.1E-01
Mtr.9178.1.S1_s_at	9.46	8.82	9.25	9.37	9.38	9.64	9.18	0.33	9.46	0.15	1.03	0.044	2.4E-01
Mtr.12628.1.S1_at	2.95	3.50	2.92	2.90	2.88	2.87	3.12	0.33	2.88	0.01	0.92	-0.116	2.7E-01
Mtr.12012.1.S1_at	9.85	9.45	10.11	9.91	9.93	9.78	9.80	0.33	9.87	0.08	1.01	0.010	7.5E-01
Mtr.41562.1.S1_at	4.28	3.70	4.27	4.39	4.27	4.38	4.08	0.33	4.34	0.07	1.06	0.089	2.5E-01
Mtr.14245.1.S1_at	7.11	6.50	7.03	6.95	7.13	6.87	6.88	0.33	6.98	0.13	1.01	0.021	6.5E-01
Mtr.32697.1.S1_at	6.35	6.02	6.68	6.49	6.43	6.87	6.35	0.33	6.60	0.24	1.04	0.054	3.6E-01
Mtr.41438.1.S1_at	8.98	8.65	9.31	9.15	9.53	9.31	8.98	0.33	9.33	0.19	1.04	0.055	1.8E-01
Mtr.24156.1.S1_at	4.51	5.15	4.70	4.34	4.58	4.51	4.79	0.33	4.48	0.12	0.94	-0.096	2.0E-01
Mtr.39053.1.S1_at	9.61	9.14	9.78	9.67	9.73	9.65	9.51	0.33	9.68	0.04	1.02	0.026	4.2E-01
Mtr.39620.1.S1_at	7.73	7.10	7.59	7.65	7.58	7.56	7.47	0.33	7.60	0.05	1.02	0.025	5.4E-01
Mtr.26677.1.S1_at	2.86	3.43	2.85	2.86	2.89	2.64	3.05	0.33	2.80	0.14	0.92	-0.125	2.9E-01
Mtr.28449.1.S1_x_at	3.93	3.28	3.56	3.54	3.53	3.53	3.59	0.33	3.53	0.01	0.98	-0.023	7.8E-01
Mtr.43338.1.S1_at	8.81	8.24	8.24	8.61	8.16	8.51	8.43	0.33	8.43	0.24	1.00	-0.001	9.9E-01
Mtr.30731.1.S1_at	5.24	5.06	5.70	5.08	5.64	5.21	5.33	0.33	5.31	0.29	1.00	-0.007	9.3E-01
Mtr.31449.1.S1_s_at	3.63	3.11	3.02	3.01	3.01	3.39	3.25	0.33	3.13	0.22	0.96	-0.053	6.3E-01
Mtr.38640.1.S1_at	9.58	9.53	10.12	9.75	9.77	9.72	9.74	0.33	9.75	0.03	1.00	0.001	9.8E-01
Mtr.9381.1.S1_at	12.13	11.51	11.63	11.57	11.45	11.59	11.76	0.33	11.54	0.07	0.98	-0.028	3.2E-01
Mtr.23865.1.S1_at	13.25	12.95	12.59	13.50	13.46	13.64	12.93	0.33	13.53	0.10	1.05	0.065	3.9E-02
Mtr.17137.1.S1_at	2.79	3.33	2.73	2.71	2.71	2.70	2.95	0.33	2.71	0.01	0.92	-0.125	2.7E-01
Mtr.37902.1.S1_at	5.04	4.42	4.53	4.79	4.63	4.84	4.66	0.33	4.75	0.11	1.02	0.027	6.8E-01
Mtr.29574.1.S1_at	4.61	5.23	5.11	5.58	6.05	6.16	4.99	0.33	5.93	0.31	1.19	0.249	2.3E-02
Mtr.4588.1.S1_at	4.84	4.28	4.87	4.38	5.44	4.85	4.66	0.33	4.89	0.53	1.05	0.068	5.6E-01
Mtr.20237.1.S1_at	8.73	8.34	8.08	8.40	8.39	8.46	8.39	0.33	8.42	0.04	1.00	0.005	8.8E-01
Mtr.8464.1.S1_s_at	13.16	12.55	12.64	12.73	12.72	12.87	12.78	0.33	12.77	0.08	1.00	-0.001	9.8E-01
Mtr.45016.1.S1_at	2.15	1.61	2.21	2.14	2.14	2.14	1.99	0.33	2.14	0.00	1.08	0.105	4.7E-01
Mtr.31767.1.S1_at	7.33	6.77	7.35	7.35	7.35	7.38	7.15	0.33	7.36	0.02	1.03	0.042	3.3E-01
Mtr.37600.1.S1_s_at	4.45	4.21	3.80	4.41	4.64	5.27	4.15	0.33	4.77	0.44	1.15	0.200	1.2E-01
Mtr.32812.1.S1_s_at	14.74	14.39	14.08	14.27	14.25	14.15	14.40	0.33	14.22	0.06	0.99	-0.018	4.1E-01
Mtr.9902.1.S1_at	7.39	6.79	7.32	7.42	7.36	7.44	7.17	0.33	7.41	0.04	1.03	0.048	2.7E-01
Mtr.50130.1.S1_at	3.33	3.00	2.67	2.65	2.64	2.62	3.00	0.33	2.64	0.02	0.88	-0.188	1.3E-01
Mtr.40870.1.S1_at	11.41	10.87	11.46	11.24	11.18	11.28	11.25	0.33	11.23	0.05	1.00	-0.001	9.6E-01
Mtr.44380.1.S1_at	11.34	10.68	11.09	11.23	11.25	11.22	11.04	0.33	11.23	0.02	1.02	0.025	3.6E-01
Mtr.44778.1.S1_at	4.38	3.87	3.76	4.04	3.59	3.89	4.00	0.33	3.84	0.23	0.96	-0.060	5.2E-01
Mtr.4860.1.S1_at	7.51	6.95	7.53	7.50	7.50	7.53	7.33	0.33	7.51	0.02	1.02	0.035	4.0E-01
Mtr.3206.1.S1_at	9.95	9.61	10.27	10.07	10.14	10.08	9.94	0.33	10.10	0.04	1.02	0.022	4.6E-01
Mtr.22551.1.S1_s_at	2.49	2.98	2.35	2.48	2.38	2.35	2.61	0.33	2.41	0.07	0.92	-0.115	3.6E-01
Mtr.47758.1.S1_at	3.24	2.71	2.64	2.66	2.61	2.66	2.87	0.33	2.64	0.03	0.92	-0.118	3.0E-01
Mtr.25096.1.S1_s_at	10.56	9.98	9.99	10.32	10.07	10.33	10.18	0.33	10.24	0.15	1.01	0.009	7.7E-01
Mtr.41956.1.S1_at	8.25	8.84	8.29	8.06	8.45	8.52	8.46	0.33	8.34	0.25	0.99	-0.020	6.5E-01
Mtr.14301.1.S1_s_at	9.46	8.95	9.55	9.39	9.34	9.33	9.32	0.33	9.35	0.03	1.00	0.005	8.8E-01
Mtr.44757.1.S1_at	10.87	10.40	11.03	10.60	10.53	10.57	10.77	0.33	10.57	0.03	0.98	-0.027	3.5E-01
Mtr.44602.1.S1_at	11.57	12.16	12.11	11.82	11.93	11.65	11.94	0.33	11.80	0.14	0.99	-0.017	5.3E-01
Mtr.4764.1.S1_at	2.29	2.85	2.27	2.26	2.25	2.26	2.47	0.33	2.26	0.01	0.91	-0.130	3.2E-01
Mtr.26355.1.S1_s_at	8.35	7.88	8.52	8.63	8.32	8.50	8.25	0.33	8.49	0.15	1.03	0.040	3.3E-01
Mtr.8598.1.S1_at	13.03	12.38	12.80	12.99	12.81	12.93	12.74	0.33	12.91	0.09	1.01	0.020	4.3E-01
Mtr.11834.1.S1_at	8.09	7.58	8.20	8.23	8.02	8.02	7.96	0.33	8.09	0.12	1.02	0.024	5.5E-01
Mtr.11362.1.S1_at	9.86	9.93	9.33	10.13	10.66	10.07	9.71	0.33	10.29	0.32	1.06	0.084	9.5E-02
Mtr.40136.1.S1_at	14.09	14.07	13.51	14.09	13.81	14.00	13.89	0.33	13.97	0.14	1.01	0.008	7.3E-01
Mtr.7747.1.S1_at	2.33	2.45	2.95	2.39	2.38	2.35	2.58	0.33	2.38	0.02	0.92	-0.118	3.5E-01
Mtr.9133.1.S1_at	10.59	10.03	10.61	10.43	10.29	10.43	10.41	0.33	10.39	0.08	1.00	-0.003	9.1E-01
Mtr.4248.1.S1_at	3.03	3.33	2.68	2.66	2.63	2.64	3.01	0.33	2.64	0.01	0.88	-0.190	1.2E-01
Mtr.12987.1.S1_at	9.53	8.88	9.14	9.38	9.41	9.13	9.18	0.33	9.31	0.15	1.01	0.019	5.9E-01
Mtr.41307.1.S1_at	9.72	9.06	9.35	9.49	9.39	9.72	9.37	0.33	9.53	0.17	1.02	0.024	5.0E-01
Mtr.19419.1.S1_s_at	9.41	9.21	9.85	9.90	9.70	9.65	9.49	0.33	9.75	0.13	1.03	0.039	2.7E-01
Mtr.45696.1.S1_at	2.48	3.02	2.42	2.41	2.36	2.41	2.64	0.33	2.39	0.03	0.91	-0.142	2.6E-01
Mtr.13590.1.S1_at	9.85	9.46	10.12	9.70	9.84	9.75	9.81	0.33	9.76	0.07	1.00	-0.007	8.2E-01
Mtr.47304.1.S1_at	3.56	3.92	3.27	3.39	3.36	3.42	3.58	0.33	3.39	0.03	0.95	-0.079	3.7E-01
Mtr.48132.1.S1_at	3.66	3.24	3.01	2.99	2.97	2.99	3.30	0.33	2.99	0.01	0.90	-0.146	1.7E-01
Mtr.7414.1.S1_at	6.46	6.17	6.82	6.55	7.08	6.56	6.48	0.33	6.73	0.30	1.04	0.054	3.9E-01
Mtr.9386.1.S1_at	12.84	12.45	13.11	12.83	13.04	12.71	12.80	0.33	12.86	0.17	1.00	0.007	7.9E-01
Mtr.45.1.S1_at	2.96	3.51	2.93	2.92	2.85	2.85	3.14	0.33	2.87	0.04	0.92	-0.126	2.4E-01
Mtr.37407.1.S1_at	3.61	2.96	3.36	3.36	4.20	3.41	3.31	0.33	3.66	0.47	1.10	0.142	3.6E-01
Mtr.27483.1.S1_at	8.23	7.61	8.09	7.99	8.27	8.21	7.98	0.33	8.16	0.15	1.02	0.032	4.4E-01
Mtr.31349.1.S1_at	3.26	2.61	2.87	2.86	2.86	2.67	2.91	0.33	2.80	0.11	0.96	-0.058	5.9E-01
Mtr.37347.1.S1_s_at	10.82	10.31	10.93	10.76	10.89	10.66	10.69	0.33	10.77	0.12	1.01	0.011	7.0E-01
Mtr.15709.1.S1_x_at	2.89	3.44	2.86	2.85	2.84	2.83	3.06	0.33	2.84	0.01	0.93	-0.108	3.1E-01
Mtr.2971.1.S1_at	2.35	2.41	2.94	2.35	2.34	2.34	2.56	0.33	2.34	0.01	0.91	-0.130	3.1E-01
Mtr.15423.1.S1_at	5.58	5.18	5.83	6.52	6.38	6.29	5.53	0.33	6.39	0.12	1.16	0.209	1.3E-02
Mtr.25421.1.S1_at	9.45	9.42	10.00	9.73	9.75	9.74	9.63	0.33	9.74	0.01	1.01	0.017	5.7E-01
Mtr.47224.1.S1_at	9.40	8.97	9.62	9.56	9.51	9.49	9.33	0.33	9.52	0.04	1.02	0.029	3.7E-01
Mtr.7460.1.S1_at	8.67	8.02	8.41	8.45	8.48	8.58	8.37	0.33	8.50	0.07	1.02	0.023	5.2E-01
Mtr.1862.1.S1_at	11.21	11.42	10.77	11.01	10.94	11.21	11.13	0.33	11.05	0.14	0.99	-0.011	7.1E-01
Mtr.43220.1.S1_at	10.26	10.24	10.82	10.41	10.78	10.30	10.44	0.33	10.50	0.25	1.01	0.008	8.3E-01
Mtr.12853.1.S1_at	9.75	9.74	10.31	10.11	10.21	10.24	9.94	0.33	10.19	0.07	1.03	0.036	2.6E-01
Mtr.36243.1.S1_at	3.22	3.57	2.91	2.94	2.87	2.87	3.23	0.33	2.89	0.04	0.90	-0.160	1.5E-01
Mtr.13558.1.S1_at	12.85	12.59	13.24	13.05	13.03	12.87	12.89	0.33	12.98	0.10	1.01	0.010	6.6E-01
Mtr.6705.1.S1_at	3.53	2.87	3.26	3.09	3.50	3.24	3.22	0.33	3.28	0.21	1.02	0.027	8.0E-01
Mtr.10558.1.S1_at	9.34	9.14	8.70	9.10	9.01	9.36	9.06	0.33	9.16	0.18	1.01	0.016	6.7E-01
Mtr.2912.1.S1_at	2.65	3.21	2.63	2.61	2.62	2.62	2.83	0.33	2.62	0.01	0.93	-0.111	3.3E-01
Mtr.12975.1.S1_at	9.43	8.78	9.17	9.19	9.12	9.23	9.13	0.33	9.18	0.05	1.01	0.008	7.9E-01
Mtr.49132.1.S1_at	9.74	9.36	10.01	9.58	9.54	9.48	9.71	0.33	9.53	0.05	0.98	-0.026	4.2E-01
Mtr.43361.1.S1_at	11.77	12.09	12.43	12.25	12.10	12.20	12.10	0.33	12.18	0.08	1.01	0.010	6.8E-01
Mtr.3579.1.S1_at	2.46	2.43	3.01	2.45	2.40	2.45	2.64	0.33	2.43	0.03	0.92	-0.114	3.5E-01
Mtr.33510.1.S1_at	7.48	7.05	7.69	7.47	7.52	7.34	7.41	0.33	7.44	0.09	1.00	0.007	8.7E-01
Mtr.20344.1.S1_at	8.85	8.77	9.37	9.17	8.99	9.09	9.00	0.33	9.08	0.09	1.01	0.014	6.8E-01
Mtr.2194.1.S1_at	3.55	3.04	2.94	3.10	3.24	3.09	3.18	0.33	3.15	0.08	0.99	-0.014	8.8E-01
Mtr.31635.1.S1_at	3.88	3.24	3.68	3.71	3.73	3.71	3.60	0.33	3.72	0.01	1.03	0.047	5.6E-01
Mtr.40416.1.S1_at	12.44	11.90	12.49	12.30	12.20	12.36	12.28	0.33	12.28	0.08	1.00	0.001	9.7E-01
Mtr.24985.1.S1_at	12.04	11.39	11.67	11.58	11.41	11.46	11.70	0.33	11.48	0.09	0.98	-0.027	3.3E-01
Mtr.40002.1.S1_at	9.74	9.09	9.39	9.51	9.67	9.51	9.41	0.33	9.56	0.09	1.02	0.024	4.7E-01
Mtr.29415.1.S1_at	7.83	7.34	7.96	8.17	8.14	8.07	7.71	0.33	8.13	0.05	1.05	0.076	9.5E-02
AFFX-Mtr-ubq11-3_x_at	11.44	10.79	11.14	11.14	11.16	11.35	11.12	0.33	11.21	0.11	1.01	0.012	6.8E-01
Mtr.43790.1.S1_at	10.84	10.88	11.43	10.72	10.85	10.68	11.05	0.33	10.75	0.09	0.97	-0.039	2.0E-01
Mtr.28708.1.S1_at	8.67	9.31	9.13	8.87	8.62	8.78	9.04	0.33	8.76	0.12	0.97	-0.045	2.4E-01
Mtr.20866.1.S1_at	7.83	7.35	7.98	7.91	7.78	7.86	7.72	0.33	7.85	0.07	1.02	0.024	5.4E-01
Mtr.17853.1.S1_at	8.88	8.35	8.95	8.84	9.05	8.90	8.73	0.33	8.93	0.11	1.02	0.033	3.7E-01
Mtr.30721.1.S1_at	8.54	7.89	8.23	8.24	8.19	8.03	8.22	0.33	8.15	0.11	0.99	-0.012	7.5E-01
Mtr.4566.1.S1_at	8.44	8.76	9.09	8.38	8.68	8.46	8.76	0.33	8.51	0.15	0.97	-0.043	2.8E-01
Mtr.42709.1.S1_at	3.98	3.40	3.95	4.11	3.95	3.95	3.77	0.33	4.00	0.09	1.06	0.085	3.1E-01
Mtr.27611.1.S1_at	6.68	6.52	7.15	7.50	7.27	7.12	6.78	0.33	7.29	0.19	1.08	0.105	8.0E-02
Mtr.41325.1.S1_at	10.27	9.69	10.25	10.24	10.24	9.96	10.07	0.33	10.15	0.16	1.01	0.011	7.3E-01
Mtr.15246.1.S1_at	2.99	3.55	2.98	2.96	2.98	3.14	3.17	0.33	3.03	0.10	0.95	-0.067	5.1E-01
Mtr.38590.1.S1_at	10.79	10.99	11.43	11.22	11.27	11.23	11.07	0.33	11.24	0.02	1.01	0.021	4.3E-01
Mtr.27603.1.S1_at	9.63	8.99	9.44	9.44	9.51	9.43	9.36	0.33	9.46	0.04	1.01	0.016	6.2E-01
Mtr.28831.1.S1_at	5.01	4.37	4.82	4.75	4.83	4.72	4.73	0.33	4.77	0.06	1.01	0.010	8.7E-01
Mtr.11584.1.S1_at	2.37	2.93	2.35	2.35	2.33	2.34	2.55	0.33	2.34	0.01	0.92	-0.124	3.3E-01
Mtr.20421.1.S1_s_at	2.93	3.47	2.88	2.84	2.81	2.81	3.09	0.33	2.82	0.02	0.91	-0.134	2.2E-01
Mtr.32119.1.S1_at	5.04	4.51	4.44	4.72	4.59	4.73	4.66	0.33	4.68	0.08	1.00	0.006	9.3E-01
Mtr.18463.1.S1_at	11.32	10.72	11.26	11.17	11.17	11.33	11.10	0.33	11.22	0.09	1.01	0.016	5.6E-01
Mtr.28417.1.S1_at	4.23	3.58	3.95	4.45	4.17	3.86	3.92	0.33	4.16	0.30	1.06	0.085	4.0E-01
Mtr.31623.1.S1_at	8.36	7.71	7.95	8.20	7.90	8.16	8.01	0.33	8.08	0.16	1.01	0.014	7.3E-01
Mtr.17614.1.S1_at	2.73	3.27	2.69	2.68	2.66	2.65	2.89	0.33	2.66	0.01	0.92	-0.121	2.9E-01
Mtr.6978.1.S1_at	8.21	7.56	7.98	8.18	8.14	8.10	7.92	0.33	8.14	0.04	1.03	0.040	3.1E-01
Mtr.39426.1.S1_at	9.60	9.05	9.64	9.67	9.84	9.79	9.43	0.33	9.77	0.09	1.04	0.050	1.6E-01
Mtr.12766.1.S1_at	10.79	10.28	10.89	10.62	10.59	10.72	10.65	0.33	10.64	0.07	1.00	-0.001	9.6E-01
Mtr.34720.1.S1_at	2.40	2.49	3.00	3.15	2.38	3.26	2.63	0.33	2.93	0.48	1.11	0.156	4.2E-01
Mtr.18038.1.S1_at	4.06	4.05	4.62	4.42	4.39	4.43	4.24	0.33	4.41	0.02	1.04	0.056	4.2E-01
Mtr.33903.1.S1_s_at	10.82	10.66	11.29	10.77	10.76	10.66	10.92	0.33	10.73	0.06	0.98	-0.025	3.8E-01
Mtr.41228.1.S1_at	11.48	10.84	11.28	11.37	11.33	11.37	11.20	0.33	11.36	0.03	1.01	0.020	4.5E-01

Mtr.46553.1.S1_s_at	9.98	9.35	9.81	9.91	9.67	9.63	9.71	0.33	9.74	0.15	1.00	0.004	9.0E-01
Mtr.30371.1.S1_at	4.41	4.56	5.03	3.44	5.02	4.27	4.67	0.33	4.24	0.79	0.91	-0.137	4.4E-01
Mtr.18429.1.S1_at	9.93	10.47	10.52	10.37	10.48	10.31	10.31	0.33	10.39	0.09	1.01	0.011	7.1E-01
Mtr.41554.1.S1_at	8.89	8.36	8.30	8.81	8.91	9.16	8.52	0.33	8.96	0.18	1.05	0.073	1.1E-01
Mtr.35458.1.S1_at	9.66	9.26	9.91	9.68	9.58	9.73	9.61	0.33	9.66	0.08	1.01	0.007	8.1E-01
Mtr.34841.1.S1_s_at	8.92	8.30	8.80	8.85	8.77	8.86	8.67	0.33	8.83	0.05	1.02	0.025	4.6E-01
Mtr.42670.1.S1_at	9.79	9.55	10.19	9.88	9.88	9.92	9.84	0.33	9.89	0.03	1.01	0.007	8.0E-01
Mtr.45466.1.S1_at	9.49	9.73	10.14	9.76	9.64	9.80	9.79	0.33	9.73	0.08	0.99	-0.008	8.0E-01
Mtr.6458.1.S1_at	2.75	2.82	3.35	3.69	3.66	3.32	2.97	0.33	3.56	0.21	1.20	0.260	5.8E-02
Mtr.12861.1.S1_at	9.57	9.10	9.73	9.50	9.57	9.57	9.47	0.33	9.55	0.04	1.01	0.012	6.9E-01
Mtr.37974.1.S1_at	10.76	11.04	11.41	11.03	10.99	10.91	11.07	0.33	10.98	0.06	0.99	-0.012	6.5E-01
Mtr.13043.1.S1_s_at	7.95	7.30	7.69	7.68	7.68	7.84	7.65	0.33	7.74	0.09	1.01	0.017	6.8E-01
Mtr.33620.1.S1_at	8.21	7.66	8.24	8.38	8.22	8.33	8.04	0.33	8.31	0.09	1.03	0.048	2.4E-01
Mtr.19779.1.S1_at	8.37	8.47	8.98	8.87	8.83	8.70	8.61	0.33	8.80	0.09	1.02	0.032	3.8E-01
Mtr.51640.1.S1_at	2.85	3.42	2.85	2.85	2.84	2.82	3.04	0.33	2.84	0.02	0.93	-0.098	3.5E-01
Mtr.47188.1.S1_at	2.43	2.99	2.42	2.41	2.41	2.41	2.61	0.33	2.41	0.00	0.92	-0.116	3.4E-01
Mtr.39552.1.S1_at	11.03	10.56	11.18	10.86	11.04	10.83	10.92	0.33	10.91	0.11	1.00	-0.002	9.5E-01
Mtr.33066.1.S1_at	2.68	2.85	3.31	2.67	2.60	2.66	2.94	0.33	2.64	0.04	0.90	-0.156	1.9E-01
Mtr.3588.1.S1_at	4.01	4.58	4.01	3.99	4.43	4.01	4.20	0.33	4.14	0.25	0.99	-0.020	8.2E-01
Mtr.16534.1.S1_at	3.20	3.51	2.86	2.83	3.48	3.13	3.19	0.33	3.15	0.33	0.99	-0.020	8.8E-01
Mtr.22312.1.S1_at	9.51	9.14	9.79	9.73	9.65	9.70	9.48	0.33	9.69	0.04	1.02	0.032	3.2E-01
Mtr.21937.1.S1_at	2.74	3.28	2.70	2.68	2.68	2.67	2.90	0.33	2.68	0.00	0.92	-0.117	3.0E-01
Mtr.18250.1.S1_at	8.69	8.19	8.80	8.68	8.87	8.72	8.56	0.33	8.75	0.10	1.02	0.032	3.8E-01
Mtr.17310.1.S1_at	2.93	3.45	2.84	2.82	2.80	2.78	3.07	0.33	2.80	0.02	0.91	-0.135	2.2E-01
Mtr.40654.1.S1_at	10.52	10.45	11.05	10.59	10.50	10.61	10.67	0.33	10.57	0.06	0.99	-0.014	6.1E-01
Mtr.32732.1.S1_at	7.58	7.76	8.21	8.02	8.37	8.00	7.85	0.33	8.13	0.21	1.04	0.051	2.8E-01
Mtr.10407.1.S1_at	10.15	10.34	9.70	9.87	9.94	10.18	10.06	0.33	10.00	0.16	0.99	-0.009	7.8E-01
Mtr.17782.1.S1_at	4.12	3.61	4.23	4.29	4.26	4.59	3.99	0.33	4.38	0.18	1.10	0.135	1.4E-01
Mtr.41997.1.S1_at	4.28	4.14	3.66	4.68	4.09	3.47	4.03	0.33	4.08	0.60	1.01	0.019	9.0E-01
Mtr.4653.1.S1_at	8.50	7.99	8.60	9.28	8.59	8.78	8.36	0.33	8.88	0.36	1.06	0.087	1.3E-01
Mtr.27793.1.S1_s_at	8.64	8.30	8.95	8.64	8.83	8.76	8.63	0.33	8.74	0.10	1.01	0.019	6.0E-01
Mtr.17570.1.S1_at	8.17	8.10	8.70	8.43	8.53	8.69	8.32	0.33	8.55	0.13	1.03	0.039	3.2E-01
Mtr.16906.1.S1_s_at	2.49	3.02	2.43	2.42	2.42	2.41	2.65	0.33	2.42	0.01	0.91	-0.131	2.9E-01
Mtr.25118.1.S1_s_at	10.77	10.12	10.46	10.66	10.60	10.38	10.45	0.33	10.55	0.15	1.01	0.014	6.5E-01
Mtr.44806.1.S1_at	11.05	10.46	11.00	10.92	10.96	10.93	10.84	0.33	10.94	0.02	1.01	0.013	6.3E-01
Mtr.3603.1.S1_at	5.83	5.68	6.30	6.29	7.49	6.85	5.93	0.33	6.88	0.60	1.16	0.213	7.5E-02
Mtr.30959.1.S1_at	2.80	3.41	2.89	2.78	2.76	2.71	3.03	0.33	2.75	0.03	0.91	-0.141	2.1E-01
Mtr.35055.1.S1_at	9.88	9.23	9.59	10.01	9.73	9.89	9.57	0.33	9.88	0.14	1.03	0.045	2.1E-01
Mtr.4604.1.S1_at	9.72	9.22	9.83	9.84	9.85	9.68	9.59	0.33	9.79	0.10	1.02	0.031	3.5E-01
Mtr.12085.1.S1_at	6.78	6.19	6.23	6.52	6.44	6.81	6.40	0.33	6.59	0.19	1.03	0.042	4.3E-01
Mtr.1736.1.S1_at	2.66	2.12	2.08	2.07	2.07	2.07	2.29	0.33	2.07	0.00	0.91	-0.142	3.2E-01
Mtr.16929.1.S1_at	8.15	7.75	8.39	8.34	8.47	8.21	8.10	0.33	8.34	0.13	1.03	0.042	3.0E-01
Mtr.22041.1.S1_at	2.79	3.39	3.31	2.78	2.78	2.78	3.16	0.33	2.78	0.00	0.88	-0.187	1.1E-01
Mtr.39889.1.S1_at	9.10	9.37	9.75	9.19	9.44	9.08	9.41	0.33	9.24	0.18	0.98	-0.026	4.7E-01
Mtr.29057.1.S1_at	6.77	6.21	6.79	6.66	6.65	6.69	6.59	0.33	6.67	0.02	1.01	0.017	7.0E-01
Mtr.15854.1.S1_x_at	2.55	3.11	2.53	2.53	2.52	2.53	2.73	0.33	2.53	0.00	0.93	-0.110	3.5E-01
Mtr.50348.1.S1_s_at	8.01	8.39	8.65	8.04	8.29	8.03	8.35	0.33	8.12	0.15	0.97	-0.040	3.4E-01
Mtr.38131.1.S1_at	10.59	10.18	9.94	10.29	10.21	9.98	10.24	0.33	10.16	0.16	0.99	-0.011	7.4E-01
Mtr.33523.1.S1_at	2.74	2.32	2.97	3.43	2.49	2.73	2.68	0.33	2.88	0.49	1.08	0.108	5.7E-01
Mtr.24688.1.S1_at	4.00	4.48	3.86	4.19	3.96	3.95	4.11	0.33	4.03	0.14	0.98	-0.029	7.0E-01
Mtr.42921.1.S1_at	11.20	11.54	11.85	11.66	11.16	11.32	11.53	0.33	11.38	0.26	0.99	-0.019	5.7E-01
Mtr.45030.1.S1_at	11.16	10.53	11.00	10.88	10.95	10.91	10.89	0.33	10.91	0.03	1.00	0.003	9.2E-01
Mtr.23409.1.S1_at	12.71	13.01	13.36	13.10	13.13	12.98	13.03	0.33	13.07	0.08	1.00	0.005	8.4E-01
Mtr.6421.1.S1_at	2.07	2.68	2.16	2.16	2.15	2.22	2.30	0.33	2.18	0.04	0.94	-0.083	5.3E-01
Mtr.1.1.S1_s_at	11.11	10.50	10.99	10.97	10.90	11.01	10.86	0.33	10.96	0.05	1.01	0.012	6.5E-01
Mtr.45372.1.S1_at	12.01	12.38	12.66	12.27	12.45	12.25	12.35	0.33	12.32	0.11	1.00	-0.003	8.9E-01
Mtr.33290.1.S1_at	9.43	8.85	9.39	9.25	9.69	9.22	9.22	0.33	9.39	0.26	1.02	0.026	5.3E-01
Mtr.1102.1.S1_at	6.47	6.65	7.10	6.46	6.46	6.43	6.74	0.33	6.45	0.01	0.96	-0.064	2.0E-01
Mtr.51413.1.S1_at	3.26	3.16	3.77	5.03	5.48	4.37	3.40	0.33	4.96	0.56	1.46	0.546	1.4E-02
Mtr.39511.1.S1_at	12.65	12.13	12.73	12.84	12.93	12.71	12.50	0.33	12.83	0.11	1.03	0.037	1.8E-01
Mtr.11976.1.S1_at	11.20	10.54	10.88	10.88	10.90	10.82	10.87	0.33	10.87	0.04	1.00	-0.001	9.8E-01
Mtr.42644.1.S1_at	10.54	9.88	10.20	10.24	10.34	10.08	10.21	0.33	10.22	0.13	1.00	0.002	9.6E-01
Mtr.28745.1.S1_at	8.89	8.36	8.95	8.76	8.79	8.75	8.73	0.33	8.77	0.02	1.00	0.006	8.6E-01
Mtr.37516.1.S1_at	10.71	11.00	11.36	11.00	11.19	10.91	11.02	0.33	11.04	0.14	1.00	0.001	9.6E-01
Mtr.25796.1.S1_at	3.21	3.72	3.12	3.18	3.06	3.11	3.35	0.33	3.12	0.06	0.93	-0.105	2.9E-01
Mtr.24390.1.S1_at	2.70	3.25	2.66	2.63	2.62	2.63	2.87	0.33	2.63	0.01	0.91	-0.129	2.6E-01
Mtr.13479.1.S1_at	2.47	2.52	3.06	2.43	3.29	2.42	2.68	0.33	2.71	0.50	1.01	0.015	9.4E-01
Mtr.13388.1.S1_s_at	8.56	8.47	9.08	8.52	8.63	8.04	8.70	0.33	8.40	0.31	0.96	-0.052	3.1E-01
Mtr.36448.1.S1_at	10.63	9.99	10.43	10.49	10.59	10.37	10.35	0.33	10.48	0.11	1.01	0.018	5.6E-01
Mtr.45982.1.S1_at	5.61	5.34	4.96	5.30	5.31	5.32	5.30	0.33	5.31	0.01	1.00	0.002	9.7E-01
Mtr.51141.1.S1_at	2.53	3.08	2.50	2.47	2.47	2.45	2.71	0.33	2.46	0.01	0.91	-0.135	2.7E-01
Mtr.39891.1.S1_at	4.84	4.20	4.64	4.23	4.04	4.31	4.56	0.33	4.19	0.14	0.92	-0.122	1.4E-01
Mtr.605.1.S1_s_at	2.94	3.50	2.92	2.92	2.89	2.83	3.12	0.33	2.88	0.05	0.92	-0.116	2.7E-01
Mtr.31107.1.S1_at	9.52	10.06	10.11	9.90	10.10	9.96	9.89	0.33	9.99	0.10	1.01	0.013	6.6E-01
Mtr.48843.1.S1_at	10.73	10.11	10.59	10.46	10.41	10.50	10.48	0.33	10.45	0.04	1.00	-0.003	9.0E-01
Mtr.35118.1.S1_at	3.50	3.91	3.26	3.53	3.44	3.48	3.56	0.33	3.48	0.05	0.98	-0.030	7.2E-01
Mtr.4045.1.S1_at	11.16	11.33	11.79	11.48	11.58	11.34	11.43	0.33	11.47	0.12	1.00	0.005	8.5E-01
Mtr.1817.1.S1_at	8.85	8.33	8.93	8.88	8.95	8.87	8.70	0.33	8.90	0.04	1.02	0.032	3.6E-01
Mtr.44891.1.S1_at	10.14	10.52	10.79	10.63	10.94	10.49	10.48	0.33	10.69	0.23	1.02	0.028	4.2E-01
Mtr.3069.1.S1_s_at	2.77	3.32	2.75	2.69	2.67	2.66	2.94	0.33	2.68	0.02	0.91	-0.138	2.3E-01
Mtr.37626.1.S1_at	12.78	13.17	13.42	12.99	13.22	13.13	13.12	0.33	13.11	0.11	1.00	-0.001	9.6E-01
Mtr.21057.1.S1_at	8.60	9.23	9.06	8.67	8.86	8.71	8.97	0.33	8.75	0.10	0.98	-0.035	3.4E-01
Mtr.43625.1.S1_at	10.91	11.39	11.52	11.13	11.37	11.01	11.27	0.33	11.17	0.19	0.99	-0.013	6.6E-01
Mtr.42378.1.S1_at	9.46	8.92	9.51	9.21	9.31	9.35	9.30	0.33	9.29	0.07	1.00	-0.001	9.8E-01
Mtr.39367.1.S1_at	8.97	8.56	9.20	9.19	9.25	8.95	8.91	0.33	9.13	0.16	1.03	0.036	3.4E-01
Mtr.31763.1.S1_at	7.42	7.09	7.74	7.39	7.33	6.98	7.42	0.33	7.23	0.22	0.98	-0.036	4.6E-01
Mtr.4298.1.S1_at	7.26	7.29	7.84	7.08	7.41	7.25	7.46	0.33	7.25	0.17	0.97	-0.043	3.6E-01
Mtr.14042.1.S1_s_at	10.80	11.36	11.36	11.41	11.89	11.25	11.17	0.33	11.51	0.33	1.03	0.044	2.7E-01
Mtr.5778.1.S1_at	6.64	5.99	6.35	6.91	6.69	6.69	6.33	0.33	6.76	0.13	1.07	0.096	9.8E-02
Mtr.34845.1.S1_at	5.29	5.42	5.91	5.49	5.78	5.41	5.54	0.33	5.56	0.19	1.00	0.004	9.4E-01
Mtr.31777.1.S1_at	2.18	2.73	2.17	2.16	2.16	2.16	2.36	0.33	2.16	0.00	0.92	-0.126	3.5E-01
Mtr.43482.1.S1_at	5.07	4.62	5.26	4.70	4.48	4.11	4.98	0.33	4.43	0.30	0.89	-0.170	9.5E-02
Mtr.35733.1.S1_at	9.47	9.01	9.64	9.63	9.41	9.34	9.37	0.33	9.46	0.15	1.01	0.013	6.9E-01
Mtr.30770.1.S1_at	12.67	12.03	12.42	6.91	7.20	6.88	12.38	0.33	6.99	0.18	0.57	-0.823	1.5E-05
Mtr.49652.1.S1_at	9.71	9.16	9.73	9.60	9.61	9.60	9.53	0.33	9.60	0.01	1.01	0.011	7.2E-01
Mtr.11357.1.S1_at	8.28	7.65	8.10	8.29	8.26	8.21	8.01	0.33	8.25	0.04	1.03	0.043	2.7E-01
Mtr.31209.1.S1_at	2.86	2.88	3.43	2.84	2.84	2.84	3.05	0.33	2.84	0.00	0.93	-0.105	3.2E-01
Mtr.16547.1.S1_at	3.94	3.92	3.36	3.88	3.81	4.79	3.74	0.32	4.16	0.55	1.11	0.153	3.2E-01
Mtr.47484.1.S1_x_at	3.02	3.56	2.98	2.95	2.90	2.95	3.19	0.32	2.93	0.03	0.92	-0.120	2.5E-01
Mtr.6654.1.S1_at	7.63	7.74	8.24	7.97	8.23	7.84	7.87	0.32	8.01	0.20	1.02	0.026	5.4E-01
Mtr.4159.1.S1_at	2.88	2.40	2.26	2.26	2.25	2.26	2.51	0.32	2.25	0.00	0.90	-0.155	2.4E-01
Mtr.41361.1.S1_at	9.46	8.85	8.96	9.05	9.08	9.13	9.09	0.32	9.09	0.04	1.00	0.000	9.9E-01
Mtr.10201.1.S1_at	5.85	6.35	5.75	5.54	5.94	5.94	5.98	0.32	5.81	0.23	0.97	-0.043	4.9E-01
Mtr.34621.1.S1_at	8.23	8.14	8.74	8.64	8.87	8.52	8.37	0.32	8.68	0.18	1.04	0.052	2.3E-01
Mtr.11598.1.S1_at	10.34	9.69	10.05	10.09	9.99	10.03	10.03	0.32	10.03	0.05	1.00	0.000	9.9E-01
Mtr.18586.1.S1_at	10.91	10.64	11.29	10.90	10.64	10.81	10.95	0.32	10.78	0.13	0.99	-0.022	4.7E-01
Mtr.49572.1.S1_s_at	14.95	14.31	14.54	14.68	14.26	14.73	14.60	0.32	14.55	0.26	1.00	-0.004	8.6E-01
Mtr.13621.1.S1_s_at	6.90	6.30	6.81	6.79	6.97	6.60	6.67	0.32	6.79	0.19	1.02	0.025	6.2E-01
Mtr.30969.1.S1_at	2.38	2.93	2.36	2.35	2.35	2.34	2.56	0.32	2.35	0.01	0.92	-0.124	3.2E-01
Mtr.9362.1.S1_at	11.17	10.52	10.87	11.07	11.16	11.12	10.85	0.32	11.11	0.05	1.02	0.034	2.4E-01
Mtr.1711.1.S1_at	4.44	3.79	4.09	4.43	4.94	4.44	4.11	0.32	4.60	0.29	1.12	0.165	1.2E-01
Mtr.14625.1.S1_at	12.30	12.80	12.91	12.70	13.05	12.54	12.67	0.32	12.76	0.26	1.01	0.011	7.1E-01
Mtr.20093.1.S1_at	4.28	3.65	4.12	4.12	4.12	4.46	4.02	0.32	4.23	0.20	1.05	0.074	3.9E-01
Mtr.43677.1.S1_at	10.48	10.40	11.00	10.59	10.68	10.41	10.63	0.32	10.56	0.13	0.99	-0.010	7.4E-01
Mtr.13247.1.S1_at	11.82	11.24	11.27	11.48	11.23	11.30	11.44	0.32	11.34	0.13	0.99	-0.013	6.3E-01
Mtr.43033.1.S1_at	12.02	11.38	11.60	11.76	11.89	11.82	11.67	0.32	11.82	0.07	1.01	0.019	4.6E-01
Mtr.42465.1.S1_at	7.09	6.88	7.51	7.32	7.57	7.71	7.16	0.32	7.53	0.20	1.05	0.073	1.6E-01
Mtr.8879.1.S1_x_at	2.96	2.43	3.02	2.85	2.90	2.84	2.81	0.32	2.86	0.03	1.02	0.029	7.8E-01
Mtr.51817.1.S1_s_at	3.13	2.96	2.50	3.32	3.06	3.46	2.86	0.32	3.28	0.20	1.15	0.196	1.3E-01
Mtr.9643.1.S1_at	8.26	7.74	8.33	8.30	8.31	8.27	8.11	0.32	8.29	0.02	1.02	0.032	3.8E-01
Mtr.40944.1.S1_at	11.12	10.48	10.72	10.98	10.87	10.95	10.77	0.32	10.93	0.05	1.01	0.021	4.5E-01
Mtr.38406.1.S1_at	8.16	7.54	7.70	8.06	8.00	8.07	7.80	0.32	8.05	0.04	1.03	0.045	2.6E-01
Mtr.11173.1.S1_at	11.59	11.24	11.89	11.79	11.55	11.65	11.57	0.32	11.66	0.12	1.01	0.012	6.6E-01
Mtr.5887.1.S1_at	12.28	11.76	12.36	11.90	11.72	11.68	12.13	0.32	11.77	0.12	0.97	-0.045	1.4E-01
Mtr.4189.1.S1_at	3.86	4.08	4.50	3.91	4.99	3.86	4.14	0.32	4.25	0.64	1.03	0.037	8.1E-01
Mtr.32170.1.S1_s_at	13.49	13.06	13.69	13.67	13.87	13.78	13.41	0.32	13.77	0.10	1.03	0.038	1.4E-01
Mtr.48342.1.S1_at	2.71	3.26	2.68	2.69	2.69	2.67	2.88	0.32	2.68	0.01	0.93	-0.103	3.5E-01
Mtr.2941.1.S1_at	11.08	10.44	10.88	10.84	10.59	10.59	10.80	0.32	10.67	0.15	0.99	-0.017	5.7E-01
Mtr.40723.1.S1_at	9.33	9.05	9.70	9.43	9.37	9.29	9.36	0.32	9.36	0.07	1.00	0.000	1.0E+00
Mtr.45117.1.S1_at	9.83	10.40	10.39	9.44	9.84	10.00	10.21	0.32	9.76	0.29	0.96	-0.065	1.5E-01
Mtr.40078.1.S1_x_at	9.67	9.31	9.02	9.91	9.62	9.91	9.34	0.32	9.81	0.17	1.05	0.072	8.6E-02

Mtr.5573.1.S1_at	4.86	4.41	5.04	4.81	4.96	4.66	4.77	0.32	4.81	0.15	1.01	0.013	8.5E-01
Mtr.31300.1.S1_at	5.72	5.20	5.12	5.25	5.26	5.42	5.35	0.32	5.31	0.09	0.99	-0.010	8.6E-01
Mtr.5534.1.S1_at	2.71	2.15	2.70	3.33	4.82	3.30	2.52	0.32	3.82	0.87	1.51	0.599	7.2E-02
Mtr.46036.1.S1_at	3.20	3.73	3.14	3.53	3.17	3.12	3.36	0.32	3.27	0.23	0.98	-0.036	7.4E-01
Mtr.12898.1.S1_at	7.92	7.46	8.08	7.75	7.96	7.82	7.82	0.32	7.84	0.10	1.00	0.005	9.0E-01
Mtr.49254.1.S1_at	9.86	9.22	9.62	9.91	9.83	9.75	9.56	0.32	9.83	0.08	1.03	0.040	2.4E-01
Mtr.10126.1.S1_at	2.55	2.46	3.06	2.39	2.31	2.34	2.69	0.32	2.35	0.04	0.87	-0.199	1.4E-01
Mtr.29535.1.S1_x_at	11.21	10.61	10.69	11.21	11.23	11.25	10.84	0.32	11.23	0.02	1.04	0.051	1.0E-01
Mtr.25998.1.S1_s_at	8.36	8.58	9.00	8.80	8.80	8.72	8.65	0.32	8.77	0.04	1.01	0.021	5.4E-01
Mtr.16526.1.S1_at	7.20	6.64	7.19	7.19	7.43	7.19	7.01	0.32	7.27	0.14	1.04	0.052	2.7E-01
Mtr.31503.1.S1_at	10.40	10.97	10.95	10.65	10.64	10.66	10.77	0.32	10.65	0.01	0.99	-0.017	5.5E-01
Mtr.30938.1.S1_at	2.32	2.87	2.30	2.30	2.28	2.29	2.50	0.32	2.29	0.01	0.92	-0.125	3.3E-01
Mtr.19751.1.S1_s_at	3.12	3.66	3.08	3.07	3.05	2.94	3.29	0.32	3.02	0.07	0.92	-0.121	2.4E-01
Mtr.43576.1.S1_at	9.68	9.03	9.41	9.69	9.46	9.58	9.38	0.32	9.58	0.11	1.02	0.031	3.7E-01
Mtr.11935.1.S1_at	9.08	8.44	8.87	8.97	8.88	8.88	8.79	0.32	8.91	0.05	1.01	0.019	5.7E-01
Mtr.38376.1.S1_at	10.69	10.13	10.13	10.28	10.20	10.34	10.32	0.32	10.27	0.07	1.00	-0.006	8.3E-01
Mtr.41926.1.S1_at	10.27	10.83	10.27	10.88	10.82	10.93	10.46	0.32	10.88	0.06	1.04	0.057	8.9E-02
Mtr.6761.1.S1_at	7.71	7.31	7.95	7.61	7.93	7.62	7.66	0.32	7.72	0.18	1.01	0.013	7.7E-01
Mtr.33160.1.S1_at	7.56	6.94	7.41	7.41	7.41	7.43	7.30	0.32	7.42	0.01	1.02	0.022	5.8E-01
Mtr.5876.1.S1_at	7.46	6.92	7.50	7.62	7.51	7.98	7.29	0.32	7.71	0.25	1.06	0.079	1.5E-01
Mtr.10366.1.S1_at	3.38	2.79	2.86	2.77	3.05	3.19	3.01	0.32	3.00	0.22	1.00	-0.003	9.8E-01
Mtr.17493.1.S1_at	4.54	4.40	5.02	5.21	5.12	4.83	4.65	0.32	5.05	0.20	1.09	0.118	1.4E-01
Mtr.5791.1.S1_at	9.52	9.18	9.83	9.48	9.69	9.80	9.51	0.32	9.66	0.16	1.02	0.023	5.1E-01
Mtr.13286.1.S1_at	9.85	10.30	10.48	10.26	10.37	10.18	10.21	0.32	10.27	0.10	1.01	0.009	7.7E-01
Mtr.10153.1.S1_at	5.99	5.41	5.96	5.51	6.48	6.07	5.79	0.32	6.02	0.48	1.04	0.057	5.2E-01
Mtr.29044.1.S1_at	8.10	7.65	8.27	8.47	8.23	8.25	8.01	0.32	8.31	0.13	1.04	0.054	2.0E-01
Mtr.39217.1.S1_at	11.12	10.50	10.99	11.04	10.67	11.00	10.87	0.32	10.90	0.20	1.00	0.005	8.8E-01
Mtr.44238.1.S1_at	8.55	8.55	7.99	8.58	8.73	8.67	8.37	0.32	8.66	0.08	1.03	0.049	2.1E-01
Mtr.9339.1.S1_at	10.10	10.03	10.62	10.05	10.14	9.91	10.25	0.32	10.03	0.12	0.98	-0.030	3.4E-01
Mtr.27778.1.S1_at	9.75	9.30	9.92	9.92	9.88	9.90	9.66	0.32	9.90	0.02	1.03	0.036	2.6E-01
Mtr.40567.1.S1_at	10.77	10.30	10.91	10.78	10.71	10.65	10.66	0.32	10.72	0.06	1.01	0.008	7.8E-01
Mtr.19730.1.S1_s_at	10.15	9.53	10.00	10.27	10.03	10.19	9.89	0.32	10.16	0.12	1.03	0.039	2.4E-01
Mtr.11462.1.S1_s_at	8.80	8.21	8.75	8.79	8.55	8.68	8.59	0.32	8.68	0.12	1.01	0.015	6.8E-01
Mtr.48023.1.S1_s_at	13.33	13.03	12.69	12.94	12.55	12.98	13.02	0.32	12.82	0.24	0.99	-0.022	4.5E-01
Mtr.26026.1.S1_at	10.07	9.48	10.00	9.85	9.71	9.78	9.85	0.32	9.78	0.07	0.99	-0.010	7.5E-01
Mtr.12366.1.S1_s_at	13.29	12.66	13.11	13.45	13.39	13.54	13.02	0.32	13.46	0.08	1.03	0.048	8.4E-02
Mtr.44197.1.S1_at	8.48	7.85	8.03	8.30	8.35	8.31	8.12	0.32	8.32	0.03	1.02	0.034	3.6E-01
Mtr.31267.1.S1_at	8.51	7.98	7.91	8.54	8.16	8.51	8.14	0.32	8.40	0.21	1.03	0.046	3.0E-01
Mtr.39572.1.S1_at	10.96	10.33	10.76	10.71	10.88	10.73	10.68	0.32	10.78	0.09	1.01	0.013	6.6E-01
Mtr.24648.1.S1_at	9.74	9.18	9.73	9.67	9.88	9.74	9.55	0.32	9.76	0.11	1.02	0.032	3.4E-01
Mtr.33793.1.S1_at	10.70	11.02	10.38	10.58	10.99	10.57	10.70	0.32	10.71	0.24	1.00	0.002	9.6E-01
Mtr.39915.1.S1_at	10.31	10.36	10.89	10.47	10.42	10.20	10.52	0.32	10.36	0.14	0.99	-0.022	4.8E-01
Mtr.38454.1.S1_at	7.72	7.20	7.79	7.96	7.88	7.81	7.57	0.32	7.88	0.07	1.04	0.058	1.8E-01
Mtr.42910.1.S1_s_at	11.09	10.62	11.24	11.10	10.96	10.97	10.99	0.32	11.01	0.08	1.00	0.004	8.9E-01
Mtr.39117.1.S1_at	7.87	7.34	7.92	7.92	7.97	7.99	7.71	0.32	7.96	0.03	1.03	0.046	2.5E-01
Mtr.22202.1.S1_x_at	2.37	2.92	2.36	2.36	2.32	2.34	2.55	0.32	2.34	0.02	0.92	-0.124	3.2E-01
Mtr.38122.1.S1_s_at	12.68	12.61	13.20	12.69	13.36	12.84	12.83	0.32	12.97	0.35	1.01	0.015	6.5E-01
Mtr.31834.1.S1_at	2.84	3.39	2.81	2.79	2.79	2.80	3.01	0.32	2.79	0.01	0.93	-0.109	3.0E-01
Mtr.28492.1.S1_at	12.87	12.25	12.41	13.05	12.81	13.05	12.51	0.32	12.97	0.14	1.04	0.052	8.5E-02
Mtr.12864.1.S1_at	2.60	3.15	2.58	2.56	2.55	2.55	2.77	0.32	2.55	0.00	0.92	-0.119	3.1E-01
Mtr.10681.1.S1_s_at	10.74	11.00	11.38	10.55	11.07	10.41	11.04	0.32	10.68	0.35	0.97	-0.048	2.6E-01
Mtr.7403.1.S1_s_at	7.64	8.22	8.18	8.19	9.27	8.85	8.01	0.32	8.77	0.54	1.10	0.131	1.1E-01
Mtr.14728.1.S1_x_at	3.11	3.64	3.06	3.06	3.05	2.98	3.27	0.32	3.03	0.04	0.93	-0.109	2.8E-01
Mtr.3578.1.S1_at	3.05	3.59	3.02	2.96	3.00	2.98	3.22	0.32	2.98	0.02	0.93	-0.111	2.7E-01
Mtr.13166.1.S1_at	8.73	8.81	9.33	8.92	8.83	8.79	8.96	0.32	8.85	0.07	0.99	-0.018	5.9E-01
Mtr.42084.1.S1_at	7.75	7.43	8.08	7.75	7.95	7.81	7.75	0.32	7.84	0.10	1.01	0.015	7.0E-01
Mtr.50870.1.S1_at	12.30	11.66	11.94	11.96	12.06	12.04	11.96	0.32	12.02	0.05	1.00	0.007	7.8E-01
Mtr.50797.1.S1_at	8.30	8.14	8.76	8.42	8.47	8.62	8.40	0.32	8.50	0.11	1.01	0.017	6.4E-01
Mtr.42612.1.S1_at	3.35	3.54	2.91	3.27	3.16	3.27	3.27	0.32	3.23	0.07	0.99	-0.014	8.8E-01
Mtr.10094.1.S1_at	3.65	3.57	4.16	3.63	3.43	3.39	3.79	0.32	3.48	0.13	0.92	-0.122	2.0E-01
Mtr.36284.1.S1_at	9.39	8.75	9.12	9.33	9.51	9.14	9.09	0.32	9.33	0.19	1.03	0.038	3.3E-01
Mtr.44140.1.S1_at	8.76	8.12	8.51	8.62	8.50	8.61	8.46	0.32	8.58	0.06	1.01	0.020	5.7E-01
Mtr.39750.1.S1_at	10.71	10.95	11.35	10.78	10.84	10.70	11.00	0.32	10.78	0.07	0.98	-0.030	3.0E-01
Mtr.20235.1.S1_at	10.28	10.66	10.93	10.68	10.65	10.64	10.62	0.32	10.66	0.02	1.00	0.005	8.6E-01
Mtr.36913.1.S1_at	10.58	10.94	11.23	10.92	10.99	10.85	10.92	0.32	10.92	0.07	1.00	0.000	9.9E-01
Mtr.48901.1.S1_at	9.44	9.33	9.94	9.29	9.17	9.34	9.57	0.32	9.27	0.09	0.97	-0.046	2.0E-01
Mtr.38954.1.S1_at	12.62	12.10	12.69	12.86	12.72	12.63	12.47	0.32	12.74	0.12	1.02	0.031	2.5E-01
Mtr.12995.1.S1_at	11.05	10.43	10.89	10.78	10.85	10.83	10.79	0.32	10.82	0.04	1.00	0.004	8.8E-01
Mtr.43727.1.S1_s_at	4.08	3.44	3.69	3.85	3.69	3.67	3.74	0.32	3.73	0.10	1.00	-0.002	9.8E-01
Mtr.40072.1.S1_s_at	9.77	10.08	10.42	10.28	10.17	10.26	10.09	0.32	10.24	0.06	1.01	0.020	4.9E-01
Mtr.43379.1.S1_at	13.14	12.62	12.55	12.91	13.24	13.15	12.77	0.32	13.10	0.17	1.03	0.037	1.9E-01
Mtr.810.1.S1_at	6.08	5.45	5.66	5.53	5.27	4.93	5.73	0.32	5.24	0.30	0.92	-0.128	1.3E-01
Mtr.38794.1.S1_at	8.28	8.72	8.91	8.74	8.53	8.69	8.64	0.32	8.65	0.11	1.00	0.003	9.4E-01
Mtr.27659.1.S1_at	8.31	7.97	8.62	8.21	8.53	8.10	8.30	0.32	8.28	0.22	1.00	-0.003	9.3E-01
Mtr.42109.1.S1_at	10.64	9.99	10.32	10.38	10.39	10.34	10.32	0.32	10.37	0.03	1.01	0.008	7.8E-01
Mtr.15103.1.S1_at	7.16	7.04	7.65	7.71	7.89	7.53	7.29	0.32	7.71	0.18	1.06	0.081	1.2E-01
Mtr.35351.1.S1_at	9.67	9.04	9.47	9.41	9.43	9.48	9.39	0.32	9.44	0.04	1.00	0.007	8.2E-01
Mtr.22489.1.S1_at	3.32	3.73	3.09	3.38	3.58	3.30	3.38	0.32	3.42	0.15	1.01	0.016	8.6E-01
Mtr.952.1.S1_at	2.68	3.23	2.66	2.66	2.66	2.62	2.86	0.32	2.65	0.03	0.93	-0.108	3.3E-01
Mtr.34619.1.S1_at	9.01	9.09	9.60	9.07	8.96	9.11	9.23	0.32	9.05	0.07	0.98	-0.029	3.9E-01
Mtr.11344.1.S1_s_at	9.47	8.87	9.37	9.65	9.72	9.92	9.24	0.32	9.76	0.14	1.06	0.080	6.1E-02
Mtr.12639.1.S1_at	12.61	12.52	13.12	12.92	12.74	12.84	12.75	0.32	12.83	0.09	1.01	0.009	7.0E-01
Mtr.33772.1.S1_at	10.39	10.11	9.74	9.46	9.54	9.69	10.08	0.32	9.56	0.12	0.95	-0.076	5.9E-02
Mtr.39941.1.S1_at	9.54	10.17	9.97	9.51	9.66	9.36	9.89	0.32	9.51	0.15	0.96	-0.057	1.4E-01
Mtr.1033.1.S1_at	12.38	11.79	12.32	12.16	12.12	11.97	12.17	0.32	12.09	0.10	0.99	-0.010	7.0E-01
Mtr.39834.1.S1_at	9.02	9.23	9.65	9.35	9.63	9.29	9.30	0.32	9.42	0.18	1.01	0.019	6.0E-01
Mtr.38778.1.S1_at	11.29	11.41	11.90	11.76	11.82	11.64	11.53	0.32	11.74	0.09	1.02	0.026	3.4E-01
Mtr.10773.1.S1_at	10.56	10.08	10.70	10.44	10.66	10.47	10.45	0.32	10.52	0.12	1.01	0.010	7.2E-01
Mtr.13661.1.S1_at	7.98	7.78	8.41	8.29	8.23	8.28	8.06	0.32	8.27	0.03	1.03	0.037	3.3E-01
Mtr.6958.1.S1_at	7.59	6.99	7.49	7.17	7.87	7.67	7.36	0.32	7.57	0.36	1.03	0.041	4.9E-01
Mtr.27359.1.S1_at	5.29	5.10	5.73	5.56	5.46	5.73	5.37	0.32	5.58	0.14	1.04	0.055	3.6E-01
Mtr.41625.1.S1_s_at	10.76	10.66	11.26	10.84	10.93	10.61	10.89	0.32	10.79	0.16	0.99	-0.013	6.6E-01
Mtr.18183.1.S1_at	4.05	3.71	4.35	4.06	4.01	4.38	4.04	0.32	4.15	0.20	1.03	0.039	6.4E-01
Mtr.47306.1.S1_x_at	2.72	3.28	2.71	2.71	2.71	2.70	2.90	0.32	2.71	0.00	0.93	-0.101	3.5E-01
Mtr.40383.1.S1_at	13.00	12.49	12.40	12.92	13.08	13.05	12.63	0.32	13.01	0.08	1.03	0.043	1.1E-01
Mtr.22554.1.S1_at	3.58	4.11	3.53	3.51	3.46	3.79	3.74	0.32	3.59	0.18	0.96	-0.061	5.1E-01
Mtr.9821.1.S1_at	3.83	4.35	3.76	4.26	4.71	3.59	3.98	0.32	4.19	0.56	1.05	0.074	6.1E-01
Mtr.1411.1.S1_at	10.08	10.69	10.58	10.11	10.28	10.19	10.45	0.32	10.19	0.09	0.98	-0.036	2.5E-01
Mtr.26579.1.S1_s_at	3.21	3.75	3.17	3.17	3.17	3.16	3.38	0.32	3.17	0.00	0.94	-0.092	3.3E-01
Mtr.43626.1.S1_at	10.15	10.34	10.78	10.23	10.49	10.32	10.43	0.32	10.35	0.13	0.99	-0.011	7.3E-01
Mtr.2072.1.S1_s_at	2.72	2.12	2.63	2.60	2.91	2.46	2.49	0.32	2.66	0.23	1.07	0.093	5.1E-01
Mtr.33167.1.S1_at	9.34	8.84	9.45	9.20	9.56	9.24	9.21	0.32	9.33	0.20	1.01	0.019	6.1E-01
Mtr.5.1.S1_at	8.20	8.63	8.83	8.37	8.27	8.36	8.55	0.32	8.33	0.05	0.97	-0.037	3.2E-01
Mtr.24627.1.S1_at	9.79	9.37	10.00	9.87	9.98	9.68	9.72	0.32	9.84	0.15	1.01	0.019	5.7E-01
Mtr.43028.1.S1_s_at	13.56	12.92	13.33	13.72	13.71	13.64	13.27	0.32	13.69	0.05	1.03	0.045	8.8E-02
Mtr.30247.1.S1_at	2.94	3.42	2.81	2.84	2.79	2.83	3.06	0.32	2.82	0.02	0.92	-0.115	2.8E-01
Mtr.13914.1.S1_s_at	8.52	7.95	8.50	8.40	8.38	8.49	8.32	0.32	8.43	0.06	1.01	0.018	6.1E-01
Mtr.47201.1.S1_at	2.82	3.36	2.78	2.78	2.91	2.71	2.99	0.32	2.80	0.10	0.94	-0.094	3.9E-01
Mtr.4650.1.S1_s_at	3.07	3.61	3.04	3.01	2.94	3.01	3.24	0.32	2.99	0.04	0.92	-0.119	2.4E-01
Mtr.52137.1.S1_at	2.82	3.36	2.78	2.76	2.73	2.72	2.99	0.32	2.74	0.02	0.92	-0.125	2.5E-01
Mtr.3530.1.S1_at	9.10	9.61	9.69	9.39	9.56	9.31	9.47	0.32	9.42	0.13	0.99	-0.007	8.2E-01
Mtr.34723.1.S1_at	4.57	4.62	5.15	4.56	4.50	4.43	4.78	0.32	4.50	0.07	0.94	-0.088	2.1E-01
Mtr.5974.1.S1_at	4.84	4.84	5.40	4.82	4.83	4.81	5.02	0.32	4.82	0.01	0.96	-0.060	3.3E-01
Mtr.43874.1.S1_s_at	10.20	9.55	9.88	9.81	9.98	10.02	9.88	0.32	9.94	0.11	1.01	0.009	7.7E-01
Mtr.2434.1.S1_s_at	9.46	9.95	10.06	9.60	9.92	9.79	9.82	0.32	9.77	0.16	0.99	-0.008	8.1E-01
Mtr.25498.1.S1_at	9.00	8.70	9.34	9.11	9.17	9.12	9.01	0.32	9.13	0.03	1.01	0.019	5.5E-01
Mtr.7636.1.S1_at	6.43	7.05	6.57	6.77	6.98	6.92	6.69	0.32	6.89	0.11	1.03	0.043	3.6E-01
Mtr.27366.1.S1_at	9.99	9.36	9.80	9.70	9.77	9.77	9.72	0.32	9.74	0.04	1.00	0.004	9.0E-01
Mtr.38474.1.S1_at	3.06	2.74	2.42	3.27	5.60	3.42	2.74	0.32	4.09	1.31	1.49	0.580	1.6E-01
Mtr.4712.1.S1_s_at	8.87	9.43	9.41	8.78	9.25	9.01	9.24	0.32	9.01	0.24	0.98	-0.036	3.8E-01
Mtr.37184.1.S1_at	8.67	8.07	8.18	8.76	8.61	8.87	8.31	0.32	8.75	0.13	1.05	0.074	9.5E-02
Mtr.38029.1.S1_at	10.06	10.09	10.63	10.02	10.11	9.86	10.26	0.32	10.00	0.13	0.97	-0.037	2.6E-01
Mtr.2386.1.S1_at	11.41	10.86	10.84	10.08	10.10	9.92	11.04	0.32	10.03	0.10	0.91	-0.138	6.6E-03
Mtr.29253.1.S1_at	7.46	7.02	7.65	7.72	7.74	7.56	7.38	0.32	7.67	0.10	1.04	0.056	2.1E-01
Mtr.18831.1.S1_at	4.93	4.98	5.51	5.07	5.81	5.23	5.14	0.32	5.37	0.39	1.04	0.063	4.8E-01
Mtr.41384.1.S1_s_at	10.42	10.26	9.80	10.42	10.49	10.67	10.16	0.32	10.53	0.13	1.04	0.051	1.4E-01
Mtr.10973.1.S1_at	10.84	10.21	10.67	10.54	10.66	10.60	10.57	0.32	10.60	0.06	1.00	0.004	9.0E-01
Mtr.18691.1.S1_s_at	11.04	11.14	11.64	12.12	12.07	11.94	11.27	0.32	12.04	0.09	1.07	0.095	1.6E-02
Mtr.1414.1.S1_at	8.41	8.85	9.04	8.37	8.83	8.23	8.77	0.32	8.48	0.31	0.97	-0.049	3.3E-01
Mtr.5117.1.S1_s_at	4.05	3.41	3.64	3.48	4.28	3.64	3.70	0.32	3.80	0.42	1.03	0.039	7.6E-01
Mtr.4958.1.S1_at	8.99	9.29	9.64	9.62	9.73	9.36	9.31	0.32	9.57	0.19	1.03	0.040	2.9E-01

Mtr.7143.1.S1_x_at	3.06	3.70	3.41	3.69	3.43	3.76	3.39	0.32	3.63	0.18	1.07	0.097	3.3E-01
Mtr.1735.1.S1_at	11.15	10.57	10.61	11.11	10.93	11.19	10.78	0.32	11.08	0.13	1.03	0.040	2.1E-01
Mtr.38060.1.S1_at	8.90	8.36	8.93	8.90	8.93	8.73	8.73	0.32	8.85	0.11	1.01	0.020	5.7E-01
Mtr.17788.1.S1_at	8.57	9.22	8.93	10.96	11.83	11.50	8.91	0.32	11.43	0.44	1.28	0.360	1.3E-03
Mtr.20939.1.S1_at	2.57	2.87	2.23	2.73	2.55	2.55	2.56	0.32	2.61	0.11	1.02	0.031	7.9E-01
Mtr.27236.1.S1_at	7.94	7.69	8.32	8.14	8.12	7.94	7.98	0.32	8.07	0.11	1.01	0.015	6.9E-01
Mtr.36255.1.S1_at	8.34	8.15	8.77	8.50	8.97	8.85	8.42	0.32	8.77	0.25	1.04	0.059	2.1E-01
Mtr.44601.1.S1_at	5.17	5.36	5.80	5.37	5.33	5.71	5.44	0.32	5.47	0.21	1.00	0.007	9.1E-01
Mtr.50993.1.S1_x_at	3.63	4.25	4.07	3.64	3.50	3.42	3.98	0.32	3.52	0.11	0.88	-0.179	7.7E-02
Mtr.40356.1.S1_at	11.75	12.22	12.37	12.03	12.34	11.99	12.12	0.32	12.12	0.19	1.00	0.001	9.8E-01
Mtr.21534.1.S1_at	2.80	3.33	2.75	2.72	2.67	2.70	2.96	0.32	2.70	0.02	0.91	-0.133	2.4E-01
Mtr.12022.1.S1_s_at	9.38	9.01	9.65	9.35	9.18	9.12	9.34	0.32	9.22	0.12	0.99	-0.020	5.6E-01
Mtr.27883.1.S1_at	2.70	3.24	2.67	2.46	2.74	2.68	2.87	0.32	2.63	0.15	0.92	-0.125	3.1E-01
Mtr.25499.1.S1_s_at	10.25	9.63	9.80	9.92	9.74	9.93	9.90	0.32	9.86	0.11	1.00	-0.005	8.8E-01
Mtr.45520.1.S1_at	9.45	8.96	8.84	8.91	8.81	9.05	9.08	0.32	8.92	0.12	0.98	-0.025	4.7E-01
Mtr.5477.1.S1_at	10.32	9.82	9.73	10.00	10.07	10.31	9.96	0.32	10.13	0.16	1.02	0.025	4.6E-01
Mtr.33688.1.S1_at	4.15	4.00	4.62	4.83	4.63	4.40	4.25	0.32	4.62	0.21	1.09	0.119	1.8E-01
Mtr.5849.1.S1_s_at	2.82	2.20	2.65	2.65	2.89	2.68	2.56	0.32	2.74	0.13	1.07	0.098	4.2E-01
Mtr.14977.1.S1_at	8.10	7.46	7.84	7.65	7.70	7.41	7.80	0.32	7.59	0.15	0.97	-0.040	3.6E-01
Mtr.9553.1.S1_at	8.81	8.18	8.57	8.77	8.66	8.83	8.52	0.32	8.75	0.09	1.03	0.039	2.9E-01
Mtr.14770.1.S1_at	10.03	9.73	10.37	10.25	9.97	10.11	10.04	0.32	10.11	0.14	1.01	0.010	7.5E-01
Mtr.37837.1.S1_at	7.77	7.48	8.12	7.62	7.74	8.16	7.79	0.32	7.84	0.29	1.01	0.010	8.4E-01
Mtr.34778.1.S1_x_at	4.43	5.07	4.67	4.67	5.03	4.76	4.73	0.32	4.82	0.19	1.02	0.029	6.8E-01
Mtr.7544.1.S1_at	9.28	8.98	9.62	9.37	9.47	9.22	9.29	0.32	9.35	0.13	1.01	0.009	7.8E-01
Mtr.5070.1.S1_at	6.99	6.97	7.54	7.22	7.08	7.20	7.17	0.32	7.17	0.07	1.00	0.000	1.0E+00
Mtr.766.1.S1_at	5.05	4.48	5.02	5.24	5.75	5.18	4.85	0.32	5.39	0.31	1.11	0.151	1.1E-01
Mtr.25930.1.S1_at	3.34	2.85	2.73	2.70	2.68	2.67	2.97	0.32	2.68	0.02	0.90	-0.148	1.9E-01
Mtr.10410.1.S1_at	6.27	5.67	6.16	5.83	5.74	5.81	6.03	0.32	5.79	0.05	0.96	-0.059	2.7E-01
Mtr.37866.1.S1_at	10.30	9.88	10.51	10.30	10.45	10.28	10.23	0.32	10.34	0.09	1.01	0.016	6.0E-01
Mtr.39920.1.S1_at	9.96	9.59	10.23	9.99	10.01	10.01	9.93	0.32	10.00	0.01	1.01	0.011	7.1E-01
Mtr.32478.1.S1_at	2.74	2.76	3.31	2.70	2.72	2.66	2.94	0.32	2.69	0.03	0.92	-0.125	2.6E-01
Mtr.20864.1.S1_at	7.54	7.06	7.67	7.35	7.44	7.45	7.43	0.32	7.41	0.05	1.00	-0.002	9.5E-01
Mtr.13655.1.S1_at	9.20	9.53	9.84	9.51	9.55	9.32	9.53	0.32	9.46	0.12	0.99	-0.010	7.6E-01
Mtr.13635.1.S1_at	9.71	9.24	9.86	9.90	9.59	9.61	9.60	0.32	9.70	0.18	1.01	0.015	6.7E-01
Mtr.11585.1.S1_at	9.25	8.61	8.89	9.08	8.94	9.10	8.91	0.32	9.04	0.09	1.01	0.020	5.5E-01
Mtr.39660.1.S1_at	9.10	8.56	9.12	9.13	9.08	9.21	8.93	0.32	9.14	0.07	1.02	0.035	3.2E-01
Mtr.37452.1.S1_at	10.37	10.06	10.70	10.33	10.89	10.36	10.38	0.32	10.53	0.32	1.01	0.021	6.0E-01
Mtr.43694.1.S1_at	10.71	10.31	10.94	10.64	10.72	10.57	10.65	0.32	10.65	0.07	1.00	-0.001	9.8E-01
Mtr.39003.1.S1_at	3.03	2.51	2.44	2.44	2.44	2.43	2.66	0.32	2.43	0.00	0.91	-0.130	2.8E-01
Mtr.15549.1.S1_at	2.86	2.34	2.27	2.68	2.53	2.53	2.49	0.32	2.58	0.08	1.04	0.051	6.6E-01
Mtr.15877.1.S1_s_at	9.82	9.28	9.85	9.32	9.40	9.45	9.65	0.32	9.39	0.07	0.97	-0.039	2.4E-01
Mtr.28174.1.S1_at	2.98	3.52	2.95	2.94	2.91	2.88	3.15	0.32	2.91	0.03	0.92	-0.115	2.6E-01
Mtr.11382.1.S1_s_at	8.52	8.31	7.89	8.19	8.25	8.47	8.24	0.32	8.30	0.15	1.01	0.012	7.6E-01
Mtr.27888.1.S1_s_at	11.44	10.83	10.97	11.42	11.76	11.58	11.08	0.32	11.59	0.17	1.05	0.065	7.3E-02
Mtr.27978.1.S1_at	9.65	9.17	9.78	9.96	9.92	10.02	9.53	0.32	9.97	0.05	1.05	0.064	8.2E-02
Mtr.37264.1.S1_at	9.63	8.99	9.38	9.46	9.60	9.50	9.33	0.32	9.52	0.07	1.02	0.029	3.8E-01
Mtr.33908.1.S1_at	7.58	6.94	7.24	7.64	8.52	7.80	7.25	0.32	7.98	0.47	1.10	0.139	8.9E-02
Mtr.41764.1.S1_s_at	11.90	12.40	12.50	12.10	12.27	12.02	12.27	0.32	12.13	0.13	0.99	-0.016	5.2E-01
Mtr.9794.1.S1_at	6.61	6.46	7.07	6.97	7.27	7.07	6.71	0.32	7.11	0.16	1.06	0.082	1.3E-01
Mtr.39174.1.S1_at	9.79	9.15	9.48	9.71	9.55	9.75	9.48	0.32	9.67	0.10	1.02	0.030	3.7E-01
Mtr.20822.1.S1_at	3.18	2.74	3.36	3.36	3.20	3.04	3.09	0.32	3.20	0.16	1.03	0.048	6.4E-01
Mtr.12911.1.S1_s_at	8.63	8.13	8.73	8.71	8.50	8.51	8.50	0.32	8.57	0.12	1.01	0.012	7.3E-01
Mtr.24949.1.S1_at	2.66	3.18	2.60	2.57	2.79	2.58	2.81	0.32	2.65	0.13	0.94	-0.087	4.6E-01
Mtr.16504.1.S1_at	10.83	11.24	11.46	10.91	10.85	10.87	11.17	0.32	10.88	0.03	0.97	-0.039	1.9E-01
Mtr.27628.1.S1_at	6.74	7.33	6.82	7.06	7.72	6.94	6.96	0.32	7.24	0.42	1.04	0.056	4.2E-01
Mtr.17597.1.S1_x_at	2.93	3.44	2.85	2.86	2.80	2.78	3.07	0.32	2.81	0.04	0.92	-0.126	2.4E-01
Mtr.41805.1.S1_at	8.02	7.41	7.90	7.70	7.65	7.69	7.78	0.32	7.68	0.03	0.99	-0.018	6.4E-01
Mtr.9639.1.S1_at	8.50	8.02	8.62	8.47	8.49	8.58	8.38	0.32	8.51	0.06	1.02	0.022	5.2E-01
Mtr.42244.1.S1_at	9.62	9.95	10.26	10.11	10.21	9.98	9.94	0.32	10.10	0.12	1.02	0.023	4.5E-01
Mtr.22415.1.S1_at	3.51	3.46	2.94	2.91	2.92	2.89	3.31	0.32	2.91	0.01	0.88	-0.185	9.8E-02
Mtr.25668.1.S1_at	2.59	2.08	2.01	2.03	2.03	2.03	2.23	0.32	2.03	0.00	0.91	-0.134	3.4E-01
Mtr.8662.1.S1_at	11.68	11.09	11.60	11.62	11.43	11.57	11.46	0.32	11.54	0.10	1.01	0.010	6.9E-01
Mtr.27855.1.S1_at	6.54	6.35	6.98	6.72	6.53	6.78	6.62	0.32	6.68	0.13	1.01	0.011	8.0E-01
Mtr.35388.1.S1_at	2.95	3.49	2.93	3.02	2.85	2.83	3.12	0.32	2.90	0.10	0.93	-0.107	3.2E-01
Mtr.4440.1.S1_at	11.26	11.04	11.67	11.26	11.28	11.13	11.32	0.32	11.22	0.08	0.99	-0.012	6.4E-01
Mtr.14136.1.S1_at	10.55	9.99	10.53	10.62	10.53	10.47	10.36	0.32	10.54	0.07	1.02	0.025	3.9E-01
Mtr.23612.1.S1_s_at	9.54	9.43	10.03	9.65	9.61	9.50	9.66	0.32	9.59	0.08	0.99	-0.012	7.1E-01
Mtr.34463.1.S1_at	2.46	3.01	2.45	2.43	2.43	2.50	2.64	0.32	2.45	0.04	0.93	-0.110	3.6E-01
Mtr.29550.1.S1_s_at	11.14	10.52	10.95	11.19	11.08	11.26	10.87	0.32	11.18	0.09	1.03	0.041	1.8E-01
Mtr.14097.1.S1_at	4.26	4.31	4.84	4.22	4.29	4.21	4.47	0.32	4.24	0.04	0.95	-0.075	2.9E-01
Mtr.11150.1.S1_at	10.67	10.72	11.25	10.96	10.92	10.75	10.88	0.32	10.88	0.11	1.00	0.000	1.0E+00
Mtr.22919.1.S1_x_at	11.79	12.20	12.41	12.04	12.07	11.96	12.13	0.32	12.02	0.05	0.99	-0.013	5.9E-01
Mtr.43580.1.S1_at	11.50	10.98	10.92	10.20	9.89	10.12	11.14	0.32	10.07	0.16	0.90	-0.145	6.8E-03
Mtr.15247.1.S1_at	2.63	3.18	2.62	2.61	2.61	2.62	2.81	0.32	2.61	0.00	0.93	-0.104	3.5E-01
Mtr.11434.1.S1_at	11.87	11.24	11.63	11.85	11.88	11.81	11.58	0.32	11.85	0.04	1.02	0.033	2.2E-01
Mtr.36800.1.S1_at	8.56	9.20	8.93	8.60	9.50	9.00	8.89	0.32	9.03	0.45	1.02	0.022	6.8E-01
Mtr.38807.1.S1_at	6.06	6.58	6.00	6.03	5.79	6.48	6.21	0.32	6.10	0.35	0.98	-0.026	7.0E-01
Mtr.46984.1.S1_at	2.33	2.88	2.32	2.31	2.31	2.31	2.51	0.32	2.31	0.00	0.92	-0.116	3.5E-01
Mtr.17303.1.S1_at	5.78	5.53	6.17	5.82	5.85	5.77	5.83	0.32	5.81	0.04	1.00	-0.003	9.5E-01
Mtr.24836.1.S1_at	2.92	3.31	2.67	2.65	2.88	2.60	2.97	0.32	2.71	0.15	0.91	-0.130	2.8E-01
Mtr.1513.1.S1_at	7.48	7.17	6.85	7.04	6.91	7.27	7.17	0.32	7.07	0.18	0.99	-0.019	6.8E-01
Mtr.25611.1.S1_at	12.98	12.35	12.58	12.64	12.04	12.69	12.64	0.32	12.45	0.36	0.99	-0.021	5.5E-01
Mtr.18482.1.S1_at	13.05	12.74	13.38	13.19	13.13	13.10	13.06	0.32	13.14	0.05	1.01	0.009	6.9E-01
Mtr.13134.1.S1_at	8.31	7.68	7.92	8.52	8.38	8.06	7.97	0.32	8.32	0.24	1.04	0.062	2.0E-01
Mtr.44679.1.S1_at	8.81	8.55	9.19	9.16	9.11	9.33	8.85	0.32	9.20	0.11	1.04	0.056	1.5E-01
Mtr.4955.1.S1_at	8.66	8.30	8.94	8.44	8.54	8.49	8.63	0.32	8.49	0.05	0.98	-0.024	4.8E-01
Mtr.27354.1.S1_s_at	10.97	10.33	10.66	10.76	10.78	10.89	10.65	0.32	10.81	0.07	1.01	0.021	4.5E-01
Mtr.23491.1.S1_at	3.27	3.32	2.74	3.55	3.13	3.27	3.11	0.32	3.32	0.22	1.07	0.091	4.2E-01
Mtr.48873.1.S1_s_at	10.40	10.25	10.86	10.71	10.55	10.73	10.50	0.32	10.66	0.10	1.01	0.021	4.6E-01
Mtr.43099.1.S1_at	13.61	13.00	13.46	13.65	13.61	13.61	13.35	0.32	13.62	0.02	1.02	0.029	2.2E-01
Mtr.24531.1.S1_s_at	8.91	8.88	9.45	9.21	9.07	9.17	9.08	0.32	9.15	0.07	1.01	0.011	7.3E-01
Mtr.34704.1.S1_at	10.41	9.99	10.62	10.48	10.50	10.49	10.34	0.32	10.49	0.01	1.01	0.021	4.6E-01
Mtr.40467.1.S1_at	3.11	2.65	2.50	2.49	2.49	2.48	2.75	0.32	2.49	0.01	0.90	-0.144	2.3E-01
Mtr.33074.1.S1_at	7.52	7.42	6.92	7.47	7.07	7.48	7.29	0.32	7.34	0.23	1.01	0.010	8.3E-01
Mtr.27279.1.S1_at	8.47	7.98	7.87	8.45	8.23	8.34	8.11	0.32	8.34	0.11	1.03	0.041	3.0E-01
Mtr.13181.1.S1_at	8.53	8.29	8.92	8.63	8.75	8.36	8.58	0.32	8.58	0.20	1.00	0.000	1.0E+00
Mtr.11360.1.S1_at	5.47	5.01	4.86	5.55	5.70	5.79	5.11	0.32	5.68	0.12	1.11	0.152	4.5E-02
Mtr.35840.1.S1_at	8.30	7.79	8.38	8.40	8.30	8.47	8.16	0.32	8.39	0.09	1.03	0.041	2.9E-01
Mtr.43086.1.S1_at	12.88	13.31	13.51	13.20	13.22	13.25	13.23	0.32	13.22	0.03	1.00	-0.001	9.7E-01
Mtr.9783.1.S1_at	11.69	11.19	11.79	11.71	11.78	11.69	11.56	0.32	11.73	0.05	1.01	0.021	4.2E-01
Mtr.45428.1.S1_at	10.87	10.84	11.41	11.17	11.10	11.19	11.04	0.32	11.15	0.05	1.01	0.014	5.9E-01
Mtr.37711.1.S1_at	11.77	11.17	11.64	11.59	11.59	11.67	11.53	0.32	11.62	0.05	1.01	0.011	6.6E-01
Mtr.5516.1.S1_at	3.17	2.70	2.57	2.31	2.62	3.30	2.81	0.32	2.74	0.51	0.97	-0.037	8.5E-01
Mtr.49107.1.S1_at	9.59	9.08	9.67	9.41	9.38	9.48	9.45	0.32	9.42	0.05	1.00	-0.004	9.1E-01
Mtr.32511.1.S1_at	4.99	4.51	5.12	5.04	4.42	4.94	4.87	0.32	4.80	0.33	0.99	-0.021	8.0E-01
Mtr.36867.1.S1_at	2.56	2.76	3.19	2.66	2.52	2.69	2.84	0.32	2.62	0.09	0.92	-0.114	3.2E-01
Mtr.37853.1.S1_s_at	10.00	9.48	10.07	10.10	10.06	9.98	9.85	0.32	10.05	0.06	1.02	0.028	3.6E-01
Mtr.39392.1.S1_at	10.70	10.13	10.67	10.72	10.63	10.62	10.50	0.32	10.66	0.05	1.01	0.021	4.5E-01
Mtr.25118.1.S1_at	11.94	11.34	11.46	11.85	11.81	11.71	11.58	0.32	11.79	0.07	1.02	0.026	3.3E-01
Mtr.13257.1.S1_at	9.60	8.99	9.45	9.74	9.59	9.64	9.35	0.32	9.66	0.08	1.03	0.047	1.8E-01
Mtr.27584.1.S1_at	7.64	7.89	8.27	7.87	7.96	7.85	7.93	0.32	7.89	0.06	0.99	-0.007	8.4E-01
Mtr.45121.1.S1_at	5.28	4.76	5.34	5.45	5.16	5.59	5.12	0.32	5.40	0.22	1.05	0.076	2.9E-01
Mtr.10359.1.S1_at	11.13	10.68	11.29	11.18	11.12	11.13	11.03	0.32	11.14	0.03	1.01	0.014	5.9E-01
Mtr.38839.1.S1_at	3.20	2.57	2.85	2.85	2.84	2.86	2.87	0.32	2.85	0.01	0.99	-0.010	9.2E-01
Mtr.20045.1.S1_at	2.90	3.43	2.85	2.89	2.87	2.91	3.06	0.32	2.89	0.02	0.94	-0.084	4.0E-01
Mtr.49190.1.S1_at	8.40	7.92	7.80	8.00	8.11	8.22	8.04	0.32	8.11	0.11	1.01	0.013	7.3E-01
Mtr.48188.1.S1_at	2.93	3.45	2.86	2.85	2.86	2.85	3.08	0.32	2.85	0.00	0.93	-0.111	2.8E-01
Mtr.40818.1.S1_at	9.82	9.24	9.77	10.06	9.76	9.90	9.61	0.32	9.91	0.15	1.03	0.044	2.2E-01
Mtr.19165.1.S1_at	9.70	9.11	9.60	9.44	9.23	9.30	9.47	0.32	9.32	0.10	0.98	-0.022	4.9E-01
Mtr.21854.1.S1_s_at	2.94	3.46	2.89	2.86	2.81	2.80	3.10	0.32	2.82	0.03	0.91	-0.134	2.1E-01
Mtr.45159.1.S1_at	9.62	9.17	9.00	9.60	10.12	10.11	9.26	0.32	9.94	0.30	1.07	0.102	5.5E-02
Mtr.14196.1.S1_s_at	10.79	10.15	10.51	10.61	10.52	10.74	10.48	0.32	10.62	0.11	1.01	0.019	5.2E-01
Mtr.9466.1.S1_at	11.82	11.31	11.89	11.81	11.83	11.92	11.67	0.32	11.85	0.06	1.02	0.022	3.9E-01
Mtr.8669.1.S1_at	14.01	14.58	14.06	13.99	14.22	14.11	14.22	0.32	14.11	0.12	0.99	-0.011	6.0E-01
Mtr.15252.1.S1_at	5.18	5.79	5.35	5.71	5.79	5.98	5.44	0.32	5.83	0.14	1.07	0.099	1.3E-01
Mtr.13953.1.S1_at	5.48	5.04	4.86	4.66	5.27	5.25	5.13	0.32	5.06	0.35	0.99	-0.019	8.1E-01
Mtr.6350.1.S1_at	7.41	6.92	7.51	7.42	7.42	7.42	7.28	0.32	7.42	0.00	1.02	0.027	5.0E-01
Mtr.42603.1.S1_at	6.05	5.48	6.01	6.02	6.19	5.92	5.84	0.32	6.05	0.14	1.03	0.049	3.7E-01
Mtr.21449.1.S1_at	2.14	2.10	2.67	2.74	3.93	2.67	2.30	0.32	3.11	0.71	1.35	0.434	1.5E-01
Mtr.21016.1.S1_at	6.17	6.15	5.61	5.91	6.09	5.66	5.98	0.32	5.88	0.22	0.98	-0.024	6.9E-01
Mtr.39890.1.S1_at	12.26	11.86	12.49	11.80	11.50	11.55	12.20	0.32	11.62	0.16	0.95	-0.071	4.7E-02

Mtr.30517.1.S1_at	8.28	7.71	7.74	6.99	7.79	7.49	7.91	0.32	7.43	0.40	0.94	-0.091	1.8E-01
Mtr.50215.1.S1_at	3.86	3.26	3.37	3.35	3.29	3.34	3.50	0.32	3.32	0.03	0.95	-0.074	4.0E-01
Mtr.13972.1.S1_at	9.07	8.52	9.07	8.92	8.89	8.69	8.88	0.32	8.83	0.13	0.99	-0.009	8.0E-01
Mtr.25361.1.S1_at	3.14	3.69	3.13	3.13	3.03	3.31	3.32	0.32	3.16	0.14	0.95	-0.074	4.6E-01
Mtr.4567.1.S1_at	8.35	7.75	8.24	8.07	8.49	7.72	8.11	0.32	8.09	0.38	1.00	-0.003	9.5E-01
Mtr.35972.1.S1_at	2.24	1.96	2.60	2.24	2.24	2.35	2.27	0.32	2.28	0.07	1.00	0.005	9.7E-01
Mtr.43870.1.S1_at	3.43	3.01	2.81	3.02	3.04	2.73	3.08	0.32	2.93	0.17	0.95	-0.074	5.0E-01
Mtr.41205.1.S1_at	10.41	9.77	10.10	10.66	10.52	10.90	10.10	0.32	10.69	0.19	1.06	0.083	5.0E-02
Mtr.38594.1.S1_at	8.17	8.09	8.68	8.26	8.32	8.16	8.31	0.32	8.24	0.08	0.99	-0.012	7.4E-01
Mtr.33161.1.S1_s_at	10.27	9.66	10.14	10.02	9.92	10.13	10.02	0.32	10.02	0.10	1.00	0.000	1.0E+00
Mtr.4368.1.S1_at	4.50	3.90	4.38	4.67	5.20	5.24	4.26	0.32	5.03	0.32	1.18	0.241	4.1E-02
Mtr.24809.1.S1_at	2.64	3.18	2.62	2.64	2.62	2.62	2.81	0.32	2.62	0.01	0.93	-0.100	3.6E-01
Mtr.26503.1.S1_s_at	2.99	3.49	2.90	2.89	2.89	2.93	3.13	0.32	2.90	0.03	0.93	-0.108	2.9E-01
Mtr.13287.1.S1_at	9.16	8.57	9.07	8.99	9.08	8.96	8.93	0.32	9.01	0.06	1.01	0.013	7.0E-01
Mtr.5813.1.S1_at	7.21	7.63	7.00	7.05	7.21	6.97	7.28	0.32	7.08	0.12	0.97	-0.040	3.7E-01
Mtr.13884.1.S1_at	11.19	11.46	11.83	11.32	11.24	11.31	11.49	0.32	11.29	0.04	0.98	-0.025	3.4E-01
Mtr.24268.1.S1_s_at	9.94	9.42	10.01	10.06	10.12	10.00	9.79	0.32	10.06	0.06	1.03	0.039	2.2E-01
Mtr.37091.1.S1_at	11.84	12.20	12.47	12.21	12.15	12.35	12.17	0.32	12.24	0.10	1.01	0.008	7.6E-01
Mtr.33207.1.S1_at	7.61	6.98	7.36	7.93	7.85	7.95	7.32	0.32	7.91	0.05	1.08	0.112	3.3E-02
Mtr.41575.1.S1_at	3.62	3.92	3.28	3.99	4.20	3.70	3.61	0.32	3.96	0.25	1.10	0.137	2.0E-01
Mtr.7219.1.S1_at	4.47	4.00	4.62	4.62	4.87	4.62	4.36	0.32	4.70	0.14	1.08	0.107	1.7E-01
Mtr.39046.1.S1_at	9.68	9.57	10.17	10.18	10.06	10.06	9.81	0.32	10.10	0.07	1.03	0.043	1.9E-01
Mtr.38150.1.S1_at	9.92	9.34	9.86	9.77	9.78	9.68	9.71	0.32	9.74	0.06	1.00	0.006	8.5E-01
Mtr.40511.1.S1_at	12.47	11.83	12.12	12.28	12.24	12.25	12.14	0.32	12.26	0.02	1.01	0.014	5.6E-01
Mtr.14730.1.S1_at	2.66	3.21	2.65	2.65	2.65	2.62	2.84	0.32	2.64	0.02	0.93	-0.107	3.3E-01
Mtr.29119.1.S1_at	6.39	5.89	6.48	5.51	6.19	5.56	6.25	0.32	5.75	0.38	0.92	-0.121	1.5E-01
Mtr.44228.1.S1_at	9.70	9.07	9.37	9.76	9.68	9.94	9.38	0.32	9.79	0.13	1.04	0.063	1.1E-01
Mtr.16476.1.S1_at	3.43	2.91	2.85	2.70	3.00	2.79	3.07	0.32	2.83	0.15	0.92	-0.115	3.1E-01
Mtr.5290.1.S1_at	8.84	8.21	8.49	8.84	9.03	9.03	8.51	0.32	8.96	0.11	1.05	0.074	8.2E-02
Mtr.4973.1.S1_s_at	11.10	10.48	10.89	10.71	10.92	10.87	10.82	0.32	10.83	0.11	1.00	0.001	9.6E-01
Mtr.42660.1.S1_at	8.04	7.41	7.80	7.53	7.95	7.75	7.75	0.32	7.74	0.21	1.00	-0.002	9.6E-01
Mtr.11726.1.S1_at	2.82	3.45	3.12	3.13	3.55	3.10	3.13	0.32	3.26	0.25	1.04	0.059	6.1E-01
Mtr.27506.1.S1_at	9.41	8.88	9.45	9.65	9.67	9.55	9.25	0.32	9.62	0.07	1.04	0.057	1.2E-01
Mtr.20683.1.S1_at	2.62	3.16	2.60	2.61	2.57	2.59	2.79	0.32	2.59	0.02	0.93	-0.108	3.3E-01
Mtr.39100.1.S1_at	11.07	10.50	11.02	11.07	10.87	11.08	10.86	0.32	11.01	0.12	1.01	0.019	5.0E-01
Mtr.50247.1.S1_at	9.99	10.31	9.67	9.84	9.57	9.88	9.99	0.32	9.76	0.17	0.98	-0.034	3.3E-01
Mtr.14476.1.S1_at	3.95	4.13	3.51	3.67	3.67	3.32	3.86	0.32	3.55	0.20	0.92	-0.120	2.3E-01
Mtr.1762.1.S1_at	9.69	9.09	9.59	9.75	9.55	9.51	9.46	0.32	9.60	0.13	1.02	0.022	5.0E-01
Mtr.14546.1.S1_at	8.49	7.88	8.35	8.46	8.12	8.44	8.24	0.32	8.34	0.19	1.01	0.017	6.7E-01
Mtr.17509.1.S1_at	8.40	8.86	8.25	8.56	7.73	8.19	8.50	0.32	8.16	0.42	0.96	-0.059	3.2E-01
Mtr.26786.1.S1_at	2.54	3.08	2.52	2.50	2.47	2.48	2.71	0.32	2.48	0.01	0.92	-0.128	2.8E-01
Mtr.40953.1.S1_at	12.26	11.63	12.01	12.00	12.02	12.04	11.97	0.32	12.02	0.02	1.00	0.007	7.8E-01
Mtr.9022.1.S1_s_at	9.96	9.39	9.42	9.71	9.68	9.95	9.59	0.32	9.78	0.15	1.02	0.028	4.0E-01
Mtr.43982.1.S1_at	8.63	8.37	9.01	8.66	8.84	8.72	8.67	0.32	8.74	0.09	1.01	0.011	7.4E-01
Mtr.8798.1.S1_at	9.71	9.21	9.80	9.80	9.97	9.74	9.58	0.32	9.84	0.12	1.03	0.039	2.5E-01
Mtr.15896.1.S1_at	3.65	3.61	3.08	2.91	3.04	2.94	3.45	0.32	2.97	0.07	0.86	-0.218	6.2E-02
Mtr.36788.1.S1_at	8.70	8.17	8.74	8.78	9.03	8.75	8.54	0.32	8.85	0.15	1.04	0.052	2.0E-01
Mtr.12435.1.S1_s_at	11.57	10.93	11.26	11.51	11.52	11.55	11.25	0.32	11.53	0.02	1.02	0.035	2.1E-01
Mtr.46008.1.S1_s_at	10.34	9.71	10.08	10.08	10.15	10.16	10.05	0.32	10.13	0.04	1.01	0.012	6.7E-01
Mtr.39054.1.S1_s_at	7.59	6.96	7.28	7.42	7.25	7.50	7.28	0.32	7.39	0.13	1.02	0.022	6.0E-01
Mtr.30037.1.S1_at	6.18	6.07	6.67	6.76	7.11	6.73	6.31	0.32	6.87	0.21	1.09	0.122	6.5E-02
Mtr.6638.1.S1_at	9.22	8.64	9.14	9.03	9.03	8.98	9.00	0.32	9.01	0.03	1.00	0.002	9.5E-01
Mtr.5835.1.S1_at	10.78	11.24	11.39	11.17	11.15	11.10	11.14	0.32	11.14	0.04	1.00	0.001	9.8E-01
Mtr.50172.1.S1_at	8.43	7.90	8.46	8.17	8.18	8.19	8.26	0.32	8.18	0.01	0.99	-0.014	6.8E-01
Mtr.48435.1.S1_at	2.48	3.02	2.46	2.94	2.51	2.70	2.66	0.32	2.71	0.22	1.02	0.032	8.0E-01
Mtr.35572.1.S1_at	9.77	9.16	9.63	9.57	9.60	9.55	9.52	0.32	9.57	0.02	1.01	0.008	7.9E-01
Mtr.15912.1.S1_s_at	10.86	10.39	11.00	10.87	10.73	10.76	10.75	0.32	10.79	0.07	1.00	0.005	8.5E-01
Mtr.8746.1.S1_at	12.14	11.54	12.01	11.94	11.85	11.95	11.90	0.32	11.91	0.05	1.00	0.002	9.5E-01
Mtr.28764.1.S1_at	8.87	8.80	9.38	9.32	9.33	9.24	9.01	0.32	9.29	0.05	1.03	0.044	2.0E-01
Mtr.43950.1.S1_at	11.71	11.18	11.14	11.24	11.21	11.23	11.34	0.32	11.22	0.01	0.99	-0.015	5.6E-01
Mtr.7062.1.S1_at	2.99	2.55	2.38	2.41	2.39	2.31	2.64	0.32	2.37	0.05	0.90	-0.155	2.2E-01
Mtr.43302.1.S1_at	8.74	9.17	9.36	8.95	9.18	8.77	9.09	0.32	8.96	0.20	0.99	-0.020	6.0E-01
Mtr.15519.1.S1_at	9.20	8.57	8.84	8.94	8.97	8.94	8.87	0.32	8.95	0.02	1.01	0.013	6.9E-01
Mtr.37729.1.S1_at	11.46	10.82	11.12	11.04	11.18	11.21	11.13	0.32	11.14	0.09	1.00	0.001	9.7E-01
Mtr.25998.1.S1_at	3.05	3.64	3.14	3.00	2.96	2.95	3.28	0.32	2.97	0.03	0.91	-0.142	1.7E-01
Mtr.11545.1.S1_at	8.59	8.42	9.04	8.22	7.99	8.24	8.68	0.32	8.15	0.14	0.94	-0.092	5.6E-02
Mtr.31915.1.S1_at	4.57	5.09	4.52	4.65	4.43	4.55	4.73	0.32	4.54	0.11	0.96	-0.058	3.9E-01
Mtr.30739.1.S1_at	7.73	7.73	7.18	7.31	7.34	7.21	7.55	0.32	7.29	0.07	0.97	-0.051	2.4E-01
Mtr.28240.1.S1_at	6.89	6.31	6.82	6.91	7.17	6.92	6.68	0.32	7.00	0.14	1.05	0.069	1.8E-01
Mtr.39907.1.S1_at	8.21	8.84	8.48	8.40	8.58	8.22	8.51	0.32	8.40	0.18	0.99	-0.018	6.3E-01
Mtr.34611.1.S1_at	9.23	8.61	8.81	8.76	8.83	8.70	8.88	0.32	8.76	0.07	0.99	-0.019	5.6E-01
Mtr.16385.1.S1_s_at	6.02	5.40	5.83	6.41	6.78	6.33	5.75	0.32	6.50	0.24	1.13	0.179	3.0E-02
Mtr.6180.1.S1_s_at	11.60	10.97	11.36	11.42	11.43	11.45	11.31	0.32	11.44	0.01	1.01	0.015	5.4E-01
Mtr.13712.1.S1_at	8.54	8.39	9.00	8.65	8.70	8.65	8.64	0.32	8.67	0.03	1.00	0.004	8.9E-01
Mtr.9045.1.S1_at	5.56	5.54	6.10	5.78	6.02	5.85	5.74	0.32	5.88	0.12	1.03	0.036	5.0E-01
Mtr.28316.1.S1_at	3.45	4.04	3.55	3.70	3.36	3.40	3.68	0.32	3.49	0.19	0.95	-0.079	4.1E-01
Mtr.27135.1.S1_at	7.86	7.34	7.92	7.90	7.93	8.10	7.71	0.32	7.98	0.11	1.03	0.049	2.4E-01
Mtr.9484.1.S1_at	11.21	11.35	11.82	11.51	11.55	11.40	11.46	0.32	11.49	0.08	1.00	0.003	9.0E-01
Mtr.5199.1.S1_s_at	7.58	7.10	7.70	7.78	7.97	8.05	7.46	0.32	7.93	0.14	1.06	0.088	7.9E-02
Mtr.13339.1.S1_at	9.98	9.96	10.52	10.09	10.30	10.09	10.15	0.32	10.16	0.12	1.00	0.002	9.5E-01
Mtr.29987.1.S1_at	7.78	8.04	8.41	8.14	8.22	7.97	8.07	0.32	8.11	0.13	1.00	0.007	8.6E-01
Mtr.39458.1.S1_at	11.42	10.79	11.03	11.25	11.13	11.16	11.08	0.32	11.18	0.06	1.01	0.014	6.0E-01
Mtr.24907.1.S1_at	9.84	9.49	10.13	9.98	9.88	9.74	9.82	0.32	9.87	0.12	1.00	0.007	8.3E-01
Mtr.21738.1.S1_at	9.21	8.58	8.98	9.11	8.95	9.10	8.92	0.32	9.06	0.09	1.02	0.022	5.2E-01
Mtr.40409.1.S1_at	9.66	10.00	10.29	9.98	9.96	9.83	9.98	0.32	9.93	0.08	0.99	-0.008	7.9E-01
Mtr.27247.1.S1_at	5.63	5.20	5.01	5.52	6.40	5.41	5.28	0.32	5.78	0.55	1.09	0.129	2.5E-01
Mtr.19030.1.S1_at	4.93	4.30	4.54	4.51	4.67	4.19	4.59	0.32	4.46	0.25	0.97	-0.042	6.0E-01
Mtr.19266.1.S1_at	9.58	9.13	9.74	9.48	9.46	9.43	9.48	0.32	9.45	0.03	1.00	-0.004	8.9E-01
Mtr.21987.1.S1_at	3.06	3.58	3.00	2.96	2.92	2.97	3.21	0.32	2.95	0.03	0.92	-0.123	2.3E-01
Mtr.50605.1.S1_at	3.28	2.89	2.65	2.66	2.64	2.66	2.94	0.32	2.65	0.01	0.90	-0.148	1.9E-01
Mtr.25468.1.S1_at	2.86	3.40	2.85	2.83	2.81	2.83	3.04	0.32	2.83	0.01	0.93	-0.104	3.1E-01
Mtr.7251.1.S1_s_at	7.26	6.73	7.29	7.18	7.40	6.96	7.09	0.32	7.18	0.22	1.01	0.018	7.2E-01
Mtr.33146.1.S1_at	2.62	3.12	3.20	3.54	3.87	3.26	2.98	0.32	3.56	0.31	1.19	0.256	8.6E-02
Mtr.39882.1.S1_at	7.00	6.74	6.37	6.71	7.21	7.11	6.71	0.32	7.01	0.26	1.05	0.064	2.7E-01
Mtr.48713.1.S1_at	8.66	9.18	8.60	8.79	9.61	9.37	8.81	0.32	9.26	0.42	1.05	0.071	2.2E-01
Mtr.10097.1.S1_at	7.86	7.75	7.27	7.80	7.81	7.93	7.63	0.32	7.84	0.07	1.03	0.041	3.1E-01
Mtr.2044.1.S1_at	8.69	8.10	8.59	8.66	8.54	8.64	8.46	0.32	8.61	0.07	1.02	0.026	4.6E-01
Mtr.20696.1.S1_at	10.24	9.67	10.21	10.17	10.36	10.37	10.04	0.32	10.30	0.11	1.03	0.037	2.5E-01
Mtr.17014.1.S1_at	3.09	3.56	2.95	2.95	2.89	2.92	3.20	0.32	2.92	0.03	0.91	-0.131	2.1E-01
Mtr.26634.1.S1_at	2.34	2.89	2.33	2.33	2.33	2.32	2.52	0.32	2.32	0.00	0.92	-0.117	3.4E-01
Mtr.6547.1.S1_s_at	7.64	7.02	7.40	7.38	7.79	7.34	7.35	0.32	7.50	0.25	1.02	0.029	5.5E-01
Mtr.10557.1.S1_at	12.64	12.21	12.83	12.70	12.81	12.81	12.56	0.32	12.77	0.06	1.02	0.024	3.2E-01
Mtr.9090.1.S1_at	11.49	10.86	11.18	11.24	11.21	11.19	11.18	0.32	11.21	0.03	1.00	0.005	8.5E-01
Mtr.43511.1.S1_at	9.04	8.82	9.44	9.21	9.18	9.25	9.10	0.32	9.21	0.03	1.01	0.017	5.8E-01
Mtr.2989.1.S1_s_at	7.04	6.48	7.02	7.06	6.90	7.20	6.84	0.32	7.05	0.15	1.03	0.043	3.7E-01
Mtr.44026.1.S1_at	10.39	9.76	10.13	10.09	10.09	10.08	10.09	0.32	10.08	0.00	1.00	-0.001	9.7E-01
Mtr.12445.1.S1_at	11.49	10.95	11.51	11.08	10.86	10.92	11.31	0.32	10.95	0.11	0.97	-0.046	1.4E-01
Mtr.19167.1.S1_at	2.76	3.30	2.74	2.71	2.71	2.74	2.93	0.32	2.72	0.01	0.93	-0.108	3.1E-01
Mtr.12323.1.S1_x_at	12.04	11.45	11.56	11.74	11.80	11.79	11.68	0.32	11.78	0.03	1.01	0.012	6.3E-01
Mtr.11711.1.S1_at	3.79	3.17	3.60	3.60	3.60	3.59	3.52	0.32	3.60	0.00	1.02	0.031	6.9E-01
Mtr.44932.1.S1_s_at	8.49	7.90	8.41	8.32	8.40	8.36	8.27	0.32	8.36	0.04	1.01	0.016	6.3E-01
Mtr.50144.1.S1_at	7.81	7.30	7.87	7.96	7.86	7.82	7.66	0.32	7.88	0.07	1.03	0.041	3.1E-01
Mtr.4741.1.S1_at	7.39	7.47	7.98	8.20	7.99	8.06	7.62	0.32	8.09	0.11	1.06	0.086	7.1E-02
Mtr.34082.1.S1_at	7.14	7.01	7.61	6.86	7.15	7.09	7.25	0.32	7.03	0.15	0.97	-0.044	3.4E-01
Mtr.38013.1.S1_at	9.44	9.19	9.81	9.48	9.33	9.34	9.48	0.32	9.38	0.08	0.99	-0.014	6.4E-01
Mtr.26161.1.S1_at	8.17	7.84	8.47	8.09	8.10	8.14	8.16	0.32	8.11	0.02	0.99	-0.009	8.1E-01
Mtr.40199.1.S1_at	15.17	14.59	14.66	14.76	14.61	14.68	14.80	0.32	14.68	0.07	0.99	-0.012	5.5E-01
Mtr.38259.1.S1_at	10.43	9.84	10.34	10.38	10.20	10.26	10.21	0.32	10.28	0.09	1.01	0.010	7.1E-01
Mtr.27771.1.S1_at	3.12	2.68	3.29	3.05	3.02	3.01	3.03	0.32	3.03	0.02	1.00	-0.002	9.9E-01
Mtr.24889.1.S1_at	7.59	7.91	8.22	8.11	8.14	7.78	7.91	0.32	8.01	0.20	1.01	0.019	6.6E-01
Mtr.10619.1.S1_at	13.12	12.58	12.56	12.72	12.40	12.74	12.76	0.32	12.62	0.19	0.99	-0.015	5.7E-01
Mtr.21901.1.S1_at	2.73	3.25	2.69	2.69	2.66	2.65	2.89	0.32	2.66	0.02	0.92	-0.118	2.8E-01
Mtr.19632.1.S1_at	2.54	3.06	2.49	2.46	2.47	2.47	2.69	0.32	2.47	0.00	0.92	-0.128	2.8E-01
Mtr.39361.1.S1_at	10.72	10.47	11.10	10.59	10.90	10.53	10.76	0.32	10.67	0.20	0.99	-0.012	6.9E-01
Mtr.507.1.S1_at	2.23	2.77	2.22	2.21	2.21	2.21	2.41	0.32	2.21	0.00	0.92	-0.121	3.5E-01
Mtr.38113.1.S1_at	9.83	9.32	9.90	9.72	9.83	9.67	9.68	0.32	9.74	0.08	1.01	0.008	7.8E-01
Mtr.42889.1.S1_s_at	10.79	11.40	10.95	11.25	11.17	11.41	11.05	0.32	11.28	0.12	1.02	0.030	3.0E-01
Mtr.40178.1.S1_at	12.07	12.69	12.51	13.21	13.49	13.41	12.42	0.32	13.37	0.14	1.08	0.106	9.0E-03
Mtr.10344.1.S1_at	13.15	12.53	12.92	13.11	13.06	13.11	12.87	0.32	13.10	0.03	1.02	0.025	2.8E-01
Mtr.10768.1.S1_at	11.42	10.80	11.02	11.19	11.18	11.16	11.08	0.32	11.18	0.01	1.01	0.013	6.2E-01
Mtr.41833.1.S1_at	10.19	9.57	9.99	10.13	9.98	10.14	9.91	0.32	10.08	0.09	1.02	0.024	4.3E-01

Mtr.34751.1.S1_at	9.57	9.10	9.70	9.72	9.75	9.55	9.45	0.32	9.67	0.11	1.02	0.033	3.2E-01
Mtr.32539.1.S1_at	3.01	2.52	3.10	2.99	3.55	3.00	2.88	0.32	3.18	0.32	1.11	0.144	3.1E-01
Mtr.44353.1.S1_s_at	6.39	6.41	6.95	6.72	6.57	6.57	6.58	0.32	6.62	0.09	1.01	0.008	8.6E-01
Mtr.13453.1.S1_at	9.92	9.35	9.40	8.37	8.63	8.46	9.56	0.32	8.49	0.13	0.89	-0.172	5.6E-03
Mtr.3847.1.S1_at	9.73	9.50	10.12	10.20	10.19	10.08	9.78	0.32	10.16	0.07	1.04	0.054	1.1E-01
Mtr.48398.1.S1_at	9.92	9.66	10.29	10.01	10.03	9.99	9.96	0.32	10.01	0.02	1.01	0.008	7.8E-01
Mtr.14311.1.S1_at	3.51	3.24	3.87	3.25	3.17	3.20	3.54	0.32	3.20	0.04	0.91	-0.143	1.4E-01
Mtr.29176.1.S1_at	8.76	9.25	9.35	8.72	8.70	8.58	9.12	0.32	8.67	0.08	0.95	-0.073	7.3E-02
Mtr.21340.1.S1_at	2.52	3.06	2.50	2.48	2.48	2.48	2.69	0.32	2.48	0.00	0.92	-0.117	3.2E-01
Mtr.11444.1.S1_at	8.68	8.36	9.00	8.36	8.49	8.71	8.68	0.32	8.52	0.18	0.98	-0.026	4.9E-01
Mtr.35970.1.S1_at	2.48	3.02	2.47	2.46	2.46	2.46	2.66	0.32	2.46	0.00	0.93	-0.111	3.4E-01
Mtr.47590.1.S1_s_at	3.76	3.31	3.91	3.75	3.81	3.66	3.66	0.32	3.74	0.08	1.02	0.030	7.1E-01
Mtr.44311.1.S1_s_at	9.43	9.36	9.94	9.80	9.64	9.83	9.58	0.32	9.75	0.10	1.02	0.026	4.1E-01
Mtr.42386.1.S1_at	8.84	8.25	8.73	9.06	8.54	8.82	8.60	0.32	8.81	0.26	1.02	0.034	4.3E-01
Mtr.27098.1.S1_at	9.92	9.40	9.97	9.86	9.90	9.68	9.76	0.32	9.81	0.12	1.00	0.007	8.2E-01
Mtr.40972.1.S1_at	9.93	9.63	10.26	10.16	10.34	10.17	9.94	0.32	10.23	0.10	1.03	0.040	2.1E-01
Mtr.27479.1.S1_at	3.25	2.64	3.08	3.08	3.08	3.53	2.99	0.32	3.23	0.26	1.08	0.111	3.7E-01
Mtr.49409.1.S1_at	9.48	10.10	9.90	9.67	9.66	9.60	9.83	0.32	9.64	0.04	0.98	-0.027	3.8E-01
Mtr.27592.1.S1_at	8.04	7.56	8.16	8.13	8.11	8.14	7.92	0.32	8.12	0.02	1.03	0.037	3.3E-01
Mtr.2712.1.S1_at	8.55	8.56	9.10	8.79	8.71	8.57	8.74	0.32	8.69	0.11	0.99	-0.008	8.1E-01
Mtr.688.1.S1_at	2.77	3.32	2.77	2.76	2.76	2.76	2.95	0.32	2.76	0.00	0.93	-0.097	3.5E-01
Mtr.34838.1.S1_at	10.31	9.73	10.23	10.32	10.14	10.18	10.09	0.32	10.21	0.10	1.01	0.017	5.6E-01
Mtr.34833.1.S1_s_at	9.18	8.55	8.86	9.02	9.07	9.19	8.87	0.32	9.09	0.09	1.03	0.036	3.0E-01
Mtr.16410.1.S1_at	2.45	2.54	3.03	2.42	2.42	2.42	2.67	0.32	2.42	0.00	0.91	-0.143	2.4E-01
Mtr.48279.1.S1_at	3.14	3.74	3.26	3.02	3.01	3.14	3.38	0.32	3.06	0.07	0.90	-0.145	1.6E-01
Mtr.20001.1.S1_at	2.21	2.28	2.79	2.19	2.19	2.18	2.43	0.32	2.18	0.00	0.90	-0.152	2.5E-01
Mtr.46614.1.S1_x_at	3.03	3.50	2.90	3.00	2.84	2.83	3.14	0.32	2.89	0.10	0.92	-0.121	2.6E-01
Mtr.38943.1.S1_at	4.56	3.98	4.50	4.61	4.45	4.94	4.35	0.32	4.67	0.25	1.07	0.104	2.4E-01
Mtr.13750.1.S1_at	8.58	8.72	9.18	8.52	8.75	8.66	8.83	0.32	8.64	0.12	0.98	-0.030	4.0E-01
Mtr.43208.1.S1_at	11.50	11.10	11.72	11.18	10.85	11.11	11.44	0.32	11.05	0.17	0.97	-0.050	1.3E-01
Mtr.48789.1.S1_s_at	10.33	9.76	9.81	9.52	9.38	9.51	9.97	0.32	9.47	0.08	0.95	-0.074	5.7E-02
Mtr.49375.1.S1_at	9.88	9.39	9.98	9.96	9.88	9.88	9.75	0.32	9.91	0.05	1.02	0.023	4.5E-01
Mtr.1582.1.S1_at	10.21	9.90	10.53	10.34	10.41	10.34	10.21	0.32	10.36	0.04	1.01	0.021	4.7E-01
Mtr.7331.1.S1_at	12.46	12.53	13.04	12.85	12.99	12.80	12.68	0.32	12.88	0.10	1.02	0.023	3.4E-01
Mtr.35142.1.S1_at	12.65	12.07	12.57	12.47	12.44	12.52	12.43	0.32	12.48	0.04	1.00	0.005	8.2E-01
Mtr.43620.1.S1_at	10.75	11.14	11.38	10.88	11.14	10.72	11.09	0.32	10.91	0.21	0.98	-0.023	4.6E-01
Mtr.26906.1.S1_at	10.07	10.53	10.68	10.25	10.60	10.12	10.43	0.32	10.32	0.25	0.99	-0.014	6.8E-01
Mtr.8857.1.S1_at	11.04	11.42	11.66	11.16	11.05	11.23	11.37	0.32	11.14	0.09	0.98	-0.029	2.9E-01
Mtr.47155.1.S1_at	2.53	3.07	2.52	2.51	2.51	2.51	2.70	0.32	2.51	0.00	0.93	-0.106	3.5E-01
Mtr.34972.1.S1_at	8.89	8.37	8.32	8.94	8.93	8.96	8.53	0.32	8.94	0.02	1.05	0.069	8.5E-02
Mtr.47316.1.S1_x_at	3.07	3.56	2.98	2.93	2.89	2.88	3.20	0.32	2.90	0.03	0.90	-0.144	1.7E-01
Mtr.39106.1.S1_at	10.35	10.27	10.85	10.50	10.52	10.38	10.49	0.32	10.47	0.08	1.00	-0.003	9.2E-01
Mtr.5036.1.S1_at	5.85	5.58	6.20	6.32	6.91	6.53	5.87	0.32	6.59	0.30	1.12	0.165	4.8E-02
Mtr.4742.1.S1_s_at	2.59	3.13	2.57	2.58	2.61	2.55	2.76	0.32	2.58	0.03	0.93	-0.100	3.7E-01
Mtr.45405.1.S1_at	4.10	3.47	3.71	3.70	3.44	3.91	3.76	0.32	3.68	0.24	0.98	-0.030	7.6E-01
Mtr.13825.1.S1_at	6.01	5.83	6.44	6.61	6.30	6.48	6.10	0.32	6.47	0.15	1.06	0.085	1.4E-01
Mtr.20707.1.S1_at	5.11	4.83	4.48	4.45	4.79	5.09	4.80	0.32	4.77	0.32	0.99	-0.009	9.1E-01
Mtr.50931.1.S1_s_at	3.52	3.48	4.05	4.98	3.98	3.51	3.68	0.32	4.16	0.75	1.13	0.174	3.7E-01
Mtr.28832.1.S1_at	5.94	5.46	6.05	6.09	6.02	5.98	5.81	0.31	6.03	0.05	1.04	0.053	3.1E-01
Mtr.3196.1.S1_at	2.36	2.91	2.36	2.36	2.34	2.35	2.55	0.31	2.35	0.01	0.92	-0.114	3.5E-01
Mtr.47291.1.S1_at	9.57	8.96	9.41	9.46	9.43	9.44	9.31	0.31	9.44	0.01	1.01	0.020	5.1E-01
Mtr.50315.1.S1_at	10.10	9.54	10.08	9.93	9.99	9.93	9.91	0.31	9.95	0.04	1.00	0.006	8.2E-01
Mtr.377.1.S1_s_at	11.41	11.54	12.01	11.83	11.81	11.71	11.65	0.31	11.78	0.06	1.01	0.016	5.2E-01
Mtr.48306.1.S1_at	8.68	8.15	8.12	9.37	9.90	9.63	8.32	0.31	9.63	0.26	1.16	0.212	5.2E-03
Mtr.33029.1.S1_s_at	12.02	11.80	11.40	11.53	11.08	11.42	11.74	0.31	11.35	0.23	0.97	-0.049	1.6E-01
Mtr.9182.1.S1_at	10.28	9.66	10.07	10.10	10.11	10.14	10.00	0.31	10.11	0.02	1.01	0.016	5.8E-01
Mtr.8233.1.S1_at	11.15	11.19	11.71	11.19	11.47	11.10	11.35	0.31	11.26	0.19	0.99	-0.012	6.8E-01
Mtr.3531.1.S1_at	3.29	3.83	3.27	3.27	3.26	3.26	3.46	0.31	3.26	0.01	0.94	-0.087	3.3E-01
Mtr.51659.1.S1_at	7.17	7.58	6.96	7.34	7.81	7.69	7.24	0.31	7.62	0.25	1.05	0.073	1.8E-01
Mtr.45953.1.S1_at	2.73	3.26	2.71	2.68	2.83	2.63	2.90	0.31	2.71	0.10	0.93	-0.098	3.8E-01
Mtr.5806.1.S1_s_at	3.55	3.69	4.15	4.15	4.90	4.91	3.79	0.31	4.65	0.44	1.23	0.294	5.1E-02
Mtr.28133.1.S1_at	11.93	11.40	11.36	11.73	11.61	11.85	11.56	0.31	11.73	0.12	1.01	0.021	4.4E-01
Mtr.10810.1.S1_at	12.04	12.46	12.65	12.29	12.39	12.29	12.39	0.31	12.32	0.06	0.99	-0.007	7.5E-01
Mtr.28509.1.S1_at	8.28	7.65	8.01	8.15	8.40	8.13	7.98	0.31	8.23	0.15	1.03	0.044	2.9E-01
Mtr.34959.1.S1_at	7.37	7.71	7.08	7.36	7.09	7.39	7.39	0.31	7.28	0.17	0.99	-0.021	6.4E-01
Mtr.33453.1.S1_at	6.11	5.51	5.96	5.32	5.47	4.71	5.86	0.31	5.17	0.41	0.88	-0.182	8.0E-02
Mtr.2149.1.S1_at	8.92	8.70	8.30	8.26	8.28	8.39	8.64	0.31	8.31	0.07	0.96	-0.056	1.5E-01
Mtr.3406.1.S1_at	7.47	6.97	7.55	7.43	7.66	7.63	7.33	0.31	7.58	0.12	1.03	0.047	2.8E-01
Mtr.13155.1.S1_at	9.18	8.58	9.06	9.08	9.09	9.10	8.94	0.31	9.09	0.01	1.02	0.023	4.7E-01
Mtr.38024.1.S1_at	10.10	10.29	10.71	10.34	10.36	10.25	10.37	0.31	10.32	0.06	1.00	-0.007	8.0E-01
Mtr.51095.1.S1_at	2.55	3.08	2.52	2.49	2.49	2.48	2.72	0.31	2.49	0.01	0.91	-0.129	2.7E-01
Mtr.50437.1.S1_s_at	8.75	8.32	8.93	8.72	8.65	8.78	8.66	0.31	8.72	0.06	1.01	0.009	7.9E-01
Mtr.41390.1.S1_at	9.23	9.23	9.78	9.07	9.70	9.17	9.41	0.31	9.31	0.34	0.99	-0.015	7.3E-01
Mtr.44813.1.S1_at	12.45	13.05	12.90	12.41	12.67	12.47	12.80	0.31	12.52	0.14	0.98	-0.032	2.3E-01
Mtr.35315.1.S1_at	9.24	8.69	9.22	9.19	9.36	8.95	9.05	0.31	9.16	0.21	1.01	0.018	6.3E-01
Mtr.45369.1.S1_at	11.13	10.72	10.51	11.00	10.84	11.10	10.79	0.31	10.98	0.13	1.02	0.026	3.8E-01
Mtr.31732.1.S1_at	9.00	9.61	9.44	9.66	10.23	9.73	9.35	0.31	9.87	0.31	1.06	0.078	1.1E-01
Mtr.6532.1.S1_s_at	9.76	9.42	10.05	9.82	9.75	10.00	9.74	0.31	9.86	0.13	1.01	0.017	5.8E-01
Mtr.35472.1.S1_at	9.72	9.17	9.19	9.21	9.35	9.22	9.36	0.31	9.26	0.08	0.99	-0.016	6.2E-01
Mtr.4095.1.S1_at	10.76	10.27	10.86	10.31	10.56	10.84	10.63	0.31	10.57	0.27	0.99	-0.007	8.3E-01
Mtr.3660.1.S1_at	2.80	3.34	2.79	2.78	2.78	2.70	2.97	0.31	2.75	0.05	0.93	-0.111	3.0E-01
Mtr.15213.1.S1_at	10.00	10.05	10.57	10.16	9.94	10.13	10.21	0.31	10.08	0.12	0.99	-0.018	5.4E-01
Mtr.44275.1.S1_s_at	10.64	11.25	11.07	10.75	10.81	10.88	10.99	0.31	10.81	0.07	0.98	-0.024	3.9E-01
Mtr.28234.1.S1_at	8.94	8.32	8.53	9.53	10.29	10.41	8.59	0.31	10.08	0.47	1.17	0.230	1.1E-02
Mtr.42287.1.S1_at	3.70	3.74	4.27	3.79	3.69	3.98	3.90	0.31	3.82	0.15	0.98	-0.031	7.0E-01
Mtr.51677.1.S1_at	3.89	3.83	3.32	4.41	3.77	3.95	3.68	0.31	4.04	0.33	1.10	0.137	2.4E-01
Mtr.41490.1.S1_at	10.05	9.63	10.25	9.98	10.04	10.02	9.98	0.31	10.01	0.03	1.00	0.005	8.5E-01
Mtr.46721.1.S1_at	9.49	9.38	9.97	9.86	10.07	9.44	9.61	0.31	9.79	0.32	1.02	0.027	5.3E-01
Mtr.1603.1.S1_at	2.34	2.88	2.33	2.32	2.31	2.31	2.52	0.31	2.32	0.00	0.92	-0.120	3.3E-01
Mtr.44206.1.S1_at	10.26	9.64	10.06	10.08	9.96	10.13	9.98	0.31	10.06	0.09	1.01	0.010	7.2E-01
Mtr.36457.1.S1_at	10.31	9.69	9.89	9.99	9.57	9.72	9.96	0.31	9.76	0.21	0.98	-0.030	4.1E-01
Mtr.51136.1.S1_s_at	9.24	8.66	9.18	9.11	9.18	9.11	9.03	0.31	9.13	0.04	1.01	0.017	6.0E-01
Mtr.41077.1.S1_at	12.99	12.44	12.99	12.97	13.03	12.91	12.80	0.31	12.97	0.06	1.01	0.018	4.2E-01
Mtr.43246.1.S1_s_at	11.47	10.85	11.05	11.19	10.91	11.34	11.12	0.31	11.15	0.22	1.00	0.003	9.2E-01
Mtr.13306.1.S1_at	10.50	10.06	9.89	10.11	10.30	10.10	10.15	0.31	10.17	0.11	1.00	0.003	9.3E-01
Mtr.50465.1.S1_at	2.51	3.05	2.50	2.50	2.48	2.47	2.69	0.31	2.48	0.02	0.92	-0.114	3.2E-01
Mtr.10753.1.S1_at	12.53	12.98	13.14	12.32	12.45	12.37	12.88	0.31	12.38	0.07	0.96	-0.057	5.3E-02
Mtr.20187.1.S1_at	14.83	14.21	14.50	14.50	14.33	14.41	14.51	0.31	14.41	0.09	0.99	-0.010	6.3E-01
Mtr.11703.1.S1_at	9.67	9.69	10.23	10.02	10.28	9.84	9.86	0.31	10.05	0.22	1.02	0.027	4.5E-01
Mtr.29337.1.S1_at	8.18	7.59	8.07	8.13	8.06	8.02	7.95	0.31	8.07	0.06	1.02	0.022	5.4E-01
Mtr.2944.1.S1_at	8.24	8.33	8.83	8.41	8.51	8.14	8.47	0.31	8.36	0.19	0.99	-0.019	6.3E-01
Mtr.17625.1.S1_at	12.05	11.47	11.97	12.08	12.24	12.23	11.83	0.31	12.18	0.09	1.03	0.043	1.3E-01
Mtr.521.1.S1_x_at	3.41	3.99	3.49	3.47	3.54	3.71	3.63	0.31	3.57	0.13	0.98	-0.023	7.9E-01
Mtr.38477.1.S1_at	9.47	8.85	9.26	9.47	9.30	9.43	9.19	0.31	9.40	0.09	1.02	0.032	3.4E-01
Mtr.27187.1.S1_at	3.39	3.43	2.87	3.35	3.93	3.83	3.23	0.31	3.70	0.31	1.15	0.198	1.3E-01
Mtr.6751.1.S1_at	6.73	6.80	7.30	7.13	7.13	7.12	6.94	0.31	7.13	0.00	1.03	0.038	3.6E-01
Mtr.10339.1.S1_at	7.03	6.80	7.42	7.24	7.34	6.79	7.08	0.31	7.12	0.30	1.01	0.009	8.7E-01
Mtr.29524.1.S1_at	2.83	3.34	2.77	2.75	2.71	2.70	2.98	0.31	2.72	0.03	0.91	-0.131	2.3E-01
Mtr.4129.1.S1_at	3.93	3.47	3.33	3.31	3.38	3.30	3.58	0.31	3.33	0.05	0.93	-0.103	2.5E-01
Mtr.29118.1.S1_at	3.26	3.85	3.73	3.05	3.64	2.95	3.61	0.31	3.21	0.37	0.89	-0.168	2.3E-01
Mtr.38867.1.S1_at	9.46	9.07	8.84	8.78	9.07	9.22	9.12	0.31	9.02	0.23	0.99	-0.016	6.7E-01
Mtr.28252.1.S1_at	9.67	9.19	9.78	9.58	9.46	9.59	9.55	0.31	9.55	0.07	1.00	0.000	1.0E+00
Mtr.23973.1.S1_at	2.66	3.19	2.63	2.61	2.59	2.58	2.83	0.31	2.59	0.02	0.92	-0.125	2.7E-01
Mtr.49967.1.S1_at	9.32	8.86	8.73	9.30	9.12	9.16	8.97	0.31	9.20	0.10	1.03	0.036	3.0E-01
Mtr.13531.1.S1_at	10.42	9.81	9.97	10.11	10.07	10.10	10.07	0.31	10.10	0.02	1.00	0.004	8.8E-01
Mtr.38545.1.S1_s_at	11.78	11.40	12.02	12.06	11.96	11.88	11.73	0.31	11.97	0.09	1.02	0.029	2.8E-01
Mtr.17083.1.S1_at	12.29	11.66	11.93	11.95	12.19	12.10	11.96	0.31	12.08	0.12	1.01	0.014	5.7E-01
Mtr.37988.1.S1_at	11.69	11.14	11.15	10.97	11.16	11.15	11.32	0.31	11.09	0.11	0.98	-0.030	2.9E-01
Mtr.44200.1.S1_at	12.60	12.00	12.47	12.30	12.19	12.34	12.36	0.31	12.28	0.08	0.99	-0.009	7.0E-01
Mtr.2268.1.S1_at	2.49	2.52	3.04	2.48	2.48	2.29	2.68	0.31	2.42	0.11	0.90	-0.150	2.4E-01
Mtr.5641.1.S1_at	3.04	2.51	2.48	2.48	2.53	2.48	2.68	0.31	2.50	0.03	0.93	-0.100	3.8E-01
Mtr.45036.1.S1_at	9.12	8.57	9.11	9.18	9.14	9.09	8.93	0.31	9.14	0.05	1.02	0.032	3.3E-01
Mtr.51699.1.S1_at	10.03	9.40	9.76	9.98	9.92	10.01	9.73	0.31	9.97	0.05	1.02	0.035	2.7E-01
Mtr.25691.1.S1_s_at	8.42	7.80	8.18	8.09	7.92	8.00	8.13	0.31	8.00	0.08	0.98	-0.023	5.3E-01
Mtr.43391.1.S1_at	10.22	9.72	9.64	10.27	10.45	10.23	9.86	0.31	10.32	0.11	1.05	0.066	7.5E-02
Mtr.8173.1.S1_at	3.19	3.73	3.18	3.18	3.17	3.16	3.37	0.31	3.17	0.01	0.94	-0.088	3.3E-01
Mtr.39354.1.S1_at	9.37	8.90	8.78	9.22	9.26	9.41	9.02	0.31	9.30	0.10	1.03	0.045	2.1E-01
Mtr.24215.1.S1_at	2.82	3.35	2.79	2.79	2.78	2.79	2.99	0.31	2.79	0.01	0.93	-0.098	3.4E-01
Mtr.15782.1.S1_at	3.04	3.54	2.96	3.24	2.88	2.91	3.18	0.31	3.01	0.20	0.95	-0.079	4.7E-01
Mtr.6522.1.S1_at	9.69	9.11	9.59	9.60	9.53	9.67	9.46	0.31	9.60	0.07	1.01	0.020	5.1E-01
Mtr.37842.1.S1_at	10.23	9.70	10.26	10.01	10.13	9.97	10.07	0.31	10.04	0.08	1.00	-0.004	8.8E-01

Mtr.3348.1.S1_at	7.71	8.32	8.16	7.86	7.84	8.18	8.06	0.31	7.96	0.19	0.99	-0.018	6.5E-01
Mtr.29418.1.S1_at	12.49	11.90	12.02	11.91	11.74	11.88	12.14	0.31	11.84	0.09	0.98	-0.035	1.9E-01
Mtr.45237.1.S1_at	7.09	7.13	7.65	7.11	7.46	7.07	7.29	0.31	7.21	0.21	0.99	-0.015	7.4E-01
Mtr.50610.1.S1_at	3.74	3.24	3.81	3.58	3.58	3.59	3.60	0.31	3.58	0.01	1.00	-0.005	9.5E-01
Mtr.39406.1.S1_at	2.16	2.69	2.14	2.29	2.15	2.09	2.33	0.31	2.18	0.10	0.94	-0.097	4.7E-01
Mtr.32969.1.S1_at	8.79	8.33	8.92	8.54	8.38	8.22	8.68	0.31	8.38	0.16	0.97	-0.051	2.1E-01
Mtr.6087.1.S1_at	6.36	6.48	6.96	6.26	6.61	6.04	6.60	0.31	6.30	0.29	0.95	-0.067	2.9E-01
Mtr.32577.1.S1_at	6.06	5.52	6.07	6.08	6.07	6.16	5.88	0.31	6.10	0.05	1.04	0.053	3.0E-01
Mtr.43743.1.S1_at	11.05	11.15	11.64	11.33	11.16	11.20	11.28	0.31	11.23	0.09	1.00	-0.006	8.0E-01
Mtr.32459.1.S1_at	7.24	6.67	6.73	6.94	7.06	7.05	6.88	0.31	7.02	0.07	1.02	0.027	5.1E-01
Mtr.5005.1.S1_at	10.48	11.03	10.50	10.35	10.47	10.52	10.67	0.31	10.45	0.09	0.98	-0.030	3.1E-01
Mtr.11727.1.S1_at	9.11	8.90	9.51	9.54	9.46	9.45	9.17	0.31	9.48	0.05	1.03	0.048	1.7E-01
Mtr.19304.1.S1_at	10.35	9.73	10.09	9.90	9.71	9.88	10.06	0.31	9.83	0.11	0.98	-0.033	3.0E-01
Mtr.9483.1.S1_at	7.22	7.03	7.64	7.20	7.21	7.15	7.30	0.31	7.19	0.03	0.98	-0.022	5.7E-01
Mtr.18381.1.S1_x_at	2.77	3.28	2.71	2.71	2.71	2.62	2.92	0.31	2.68	0.05	0.92	-0.125	2.6E-01
Mtr.7586.1.S1_at	2.97	3.46	2.88	2.87	2.84	2.83	3.11	0.31	2.85	0.02	0.92	-0.126	2.3E-01
Mtr.4579.1.S1_at	10.30	10.55	10.92	10.56	10.60	10.34	10.59	0.31	10.50	0.14	0.99	-0.012	6.8E-01
Mtr.33505.1.S1_at	2.67	2.31	2.94	2.55	3.32	2.67	2.64	0.31	2.85	0.42	1.08	0.110	5.3E-01
Mtr.50056.1.S1_at	2.88	3.40	2.85	2.83	2.81	2.81	3.04	0.31	2.82	0.01	0.93	-0.109	2.9E-01
Mtr.6772.1.S1_at	10.18	9.56	9.86	10.03	10.02	9.96	9.87	0.31	10.00	0.04	1.01	0.020	5.0E-01
Mtr.32509.1.S1_s_at	9.29	8.73	9.26	9.25	9.17	9.34	9.09	0.31	9.26	0.09	1.02	0.025	4.4E-01
Mtr.48550.1.S1_at	9.54	9.03	9.60	9.82	9.75	9.62	9.39	0.31	9.73	0.10	1.04	0.051	1.5E-01
Mtr.7270.1.S1_at	4.42	4.07	4.69	5.60	5.79	5.29	4.39	0.31	5.56	0.25	1.26	0.339	7.4E-03
Mtr.7723.1.S1_at	10.01	10.56	10.54	9.77	10.15	9.60	10.37	0.31	9.84	0.28	0.95	-0.076	9.5E-02
Mtr.6775.1.S1_at	5.06	4.54	4.51	4.52	5.04	4.47	4.70	0.31	4.68	0.32	0.99	-0.008	9.2E-01
Mtr.2507.1.S1_at	11.36	10.83	11.37	11.25	11.28	11.30	11.19	0.31	11.28	0.02	1.01	0.012	6.5E-01
Mtr.42997.1.S1_at	11.33	11.41	11.91	11.61	11.78	11.31	11.55	0.31	11.57	0.24	1.00	0.002	9.5E-01
Mtr.28601.1.S1_at	9.62	9.46	9.01	9.63	9.57	9.68	9.36	0.31	9.63	0.05	1.03	0.040	2.2E-01
Mtr.1231.1.S1_s_at	7.96	7.76	8.37	8.22	8.12	8.29	8.03	0.31	8.21	0.09	1.02	0.032	3.8E-01
Mtr.50988.1.S1_at	2.66	3.20	2.65	2.64	2.64	2.60	2.84	0.31	2.63	0.03	0.93	-0.111	3.1E-01
Mtr.25130.1.S1_at	2.41	2.94	2.39	2.37	2.38	2.37	2.58	0.31	2.37	0.01	0.92	-0.122	3.1E-01
Mtr.40101.1.S1_s_at	14.66	14.96	15.28	15.01	15.15	15.03	14.97	0.31	15.06	0.08	1.01	0.009	6.3E-01
Mtr.32338.1.S1_at	8.19	7.56	7.82	8.25	8.07	8.22	7.86	0.31	8.18	0.10	1.04	0.058	1.6E-01
Mtr.11425.1.S1_at	9.51	8.98	9.54	9.53	9.65	9.58	9.34	0.31	9.59	0.06	1.03	0.038	2.5E-01
Mtr.26998.1.S1_at	9.01	8.46	8.99	8.81	8.93	8.72	8.82	0.31	8.82	0.10	1.00	0.001	9.8E-01
Mtr.25393.1.S1_at	2.57	3.08	2.51	2.51	2.51	2.49	2.72	0.31	2.50	0.01	0.92	-0.120	3.0E-01
Mtr.43501.1.S1_at	12.30	11.72	12.22	12.19	12.16	12.11	12.08	0.31	12.15	0.04	1.01	0.009	6.9E-01
Mtr.19384.1.S1_at	11.23	10.65	11.13	10.98	10.92	10.94	11.01	0.31	10.95	0.03	0.99	-0.008	7.6E-01
Mtr.49915.1.S1_s_at	12.65	12.84	13.26	12.86	12.70	12.78	12.92	0.31	12.78	0.08	0.99	-0.015	5.0E-01
Mtr.16256.1.S1_at	2.44	2.97	2.41	2.46	2.39	2.37	2.61	0.31	2.41	0.05	0.92	-0.113	3.4E-01
Mtr.2522.1.S1_at	3.84	3.84	3.30	4.71	4.47	3.91	3.66	0.31	4.37	0.41	1.19	0.253	7.8E-02
Mtr.49007.1.S1_s_at	8.39	7.77	7.98	8.17	8.33	8.47	8.05	0.31	8.32	0.15	1.03	0.048	2.4E-01
Mtr.31864.1.S1_at	4.14	4.55	3.94	4.04	4.04	4.07	4.21	0.31	4.05	0.02	0.96	-0.056	4.2E-01
Mtr.5571.1.S1_s_at	10.93	10.32	10.50	10.71	10.73	10.88	10.58	0.31	10.77	0.09	1.02	0.026	3.6E-01
Mtr.43298.1.S1_at	11.16	10.55	10.75	11.08	10.90	11.04	10.82	0.31	11.01	0.10	1.02	0.025	3.8E-01
Mtr.45325.1.S1_at	7.56	7.14	7.75	7.50	7.92	7.54	7.49	0.31	7.65	0.23	1.02	0.032	4.9E-01
Mtr.42767.1.S1_at	5.22	4.60	4.97	5.05	5.35	4.95	4.93	0.31	5.12	0.21	1.04	0.053	4.4E-01
Mtr.22376.1.S1_at	3.19	3.25	3.76	3.21	3.27	4.47	3.40	0.31	3.65	0.71	1.07	0.102	6.1E-01
Mtr.15658.1.S1_at	3.36	3.41	3.92	3.34	3.57	3.19	3.56	0.31	3.37	0.19	0.95	-0.081	4.1E-01
Mtr.3114.1.S1_s_at	8.54	8.09	8.68	8.69	8.78	8.61	8.44	0.31	8.70	0.08	1.03	0.043	2.4E-01
Mtr.9215.1.S1_at	11.63	11.26	11.88	11.55	11.55	11.40	11.59	0.31	11.50	0.08	0.99	-0.011	6.6E-01
Mtr.17107.1.S1_at	7.46	7.04	7.65	7.48	7.78	7.64	7.39	0.31	7.63	0.15	1.03	0.047	2.8E-01
Mtr.38268.1.S1_at	8.92	8.43	9.01	8.83	9.06	8.86	8.78	0.31	8.92	0.12	1.01	0.021	5.3E-01
Mtr.12884.1.S1_at	9.60	9.02	9.51	9.38	9.53	9.40	9.38	0.31	9.44	0.08	1.01	0.010	7.5E-01
Mtr.43221.1.S1_at	12.55	12.69	13.15	12.89	13.04	12.79	12.80	0.31	12.91	0.13	1.01	0.013	5.9E-01
Mtr.43607.1.S1_at	9.75	9.21	9.74	9.67	9.54	9.65	9.57	0.31	9.62	0.07	1.01	0.008	7.9E-01
Mtr.45478.1.S1_at	9.18	8.70	8.59	8.99	8.89	9.00	8.82	0.31	8.96	0.06	1.02	0.022	4.9E-01
Mtr.6366.1.S1_at	7.93	7.55	7.31	8.09	7.89	8.52	7.60	0.31	8.17	0.32	1.08	0.104	9.3E-02
Mtr.12713.1.S1_at	11.05	10.51	11.04	11.10	11.05	11.08	10.87	0.31	11.08	0.03	1.02	0.028	3.1E-01
Mtr.7989.1.S1_at	6.02	5.49	6.04	5.91	6.11	6.15	5.85	0.31	6.06	0.13	1.04	0.050	3.5E-01
Mtr.15853.1.S1_at	9.82	9.20	9.47	9.58	9.57	9.53	9.50	0.31	9.56	0.02	1.01	0.010	7.5E-01
Mtr.31108.1.S1_at	2.89	3.40	2.83	2.82	2.81	2.80	3.04	0.31	2.81	0.01	0.93	-0.112	2.8E-01
Mtr.5483.1.S1_at	8.25	8.40	7.80	7.48	7.83	7.42	8.15	0.31	7.58	0.22	0.93	-0.106	5.9E-02
Mtr.44441.1.S1_s_at	8.30	7.82	8.41	8.23	8.15	8.27	8.18	0.31	8.21	0.06	1.00	0.006	8.5E-01
Mtr.2744.1.S1_at	9.56	9.06	9.63	9.49	9.74	9.74	9.42	0.31	9.66	0.14	1.03	0.036	2.9E-01
Mtr.42110.1.S1_at	10.25	9.72	9.71	9.96	9.84	9.93	9.89	0.31	9.91	0.06	1.00	0.003	9.2E-01
Mtr.25541.1.S1_at	5.91	5.70	6.32	5.89	5.97	5.96	5.98	0.31	5.94	0.04	0.99	-0.008	8.6E-01
Mtr.33356.1.S1_s_at	11.47	10.85	11.26	11.32	11.30	11.35	11.19	0.31	11.32	0.02	1.01	0.017	5.1E-01
Mtr.49008.1.S1_x_at	6.27	5.80	6.39	5.97	5.86	5.85	6.15	0.31	5.89	0.07	0.96	-0.062	2.3E-01
Mtr.9377.1.S1_at	8.18	7.79	8.40	8.58	8.47	8.48	8.12	0.31	8.51	0.06	1.05	0.067	1.0E-01
Mtr.43244.1.S1_s_at	2.62	2.11	2.05	2.05	2.05	2.05	2.26	0.31	2.05	0.00	0.91	-0.141	3.1E-01
Mtr.12771.1.S1_at	11.44	10.81	11.14	11.25	11.17	11.32	11.13	0.31	11.24	0.07	1.01	0.015	5.7E-01
Mtr.46230.1.S1_at	6.14	6.24	6.72	6.62	6.21	5.85	6.37	0.31	6.23	0.39	0.98	-0.032	6.5E-01
Mtr.45243.1.S1_at	12.72	12.90	13.33	13.10	13.07	12.85	12.98	0.31	13.01	0.13	1.00	0.003	9.0E-01
Mtr.18538.1.S1_at	8.50	8.01	8.59	8.72	8.69	8.50	8.37	0.31	8.64	0.12	1.03	0.046	2.4E-01
Mtr.13858.1.S1_at	2.92	2.40	2.95	3.27	3.53	2.35	2.76	0.31	3.05	0.62	1.11	0.145	5.1E-01
Mtr.47822.1.S1_s_at	2.72	3.24	2.68	2.66	2.65	2.65	2.88	0.31	2.65	0.01	0.92	-0.121	2.7E-01
Mtr.13839.1.S1_at	8.60	8.06	8.07	8.48	8.03	8.50	8.24	0.31	8.34	0.27	1.01	0.016	7.1E-01
Mtr.12187.1.S1_at	9.81	9.95	10.41	10.17	10.05	9.75	10.06	0.31	9.99	0.21	0.99	-0.010	7.8E-01
Mtr.27537.1.S1_s_at	8.23	7.67	8.18	8.07	8.19	8.25	8.03	0.31	8.17	0.09	1.02	0.026	4.9E-01
Mtr.24142.1.S1_at	2.57	3.11	2.56	2.56	2.56	2.56	2.75	0.31	2.56	0.00	0.93	-0.103	3.5E-01
Mtr.32721.1.S1_at	7.86	7.97	8.45	8.20	8.06	8.03	8.09	0.31	8.10	0.09	1.00	0.001	9.8E-01
Mtr.22850.1.S1_at	5.39	5.54	5.99	5.84	5.77	5.76	5.64	0.31	5.79	0.04	1.03	0.038	4.5E-01
Mtr.45970.1.S1_at	10.62	10.82	10.21	10.76	10.58	10.63	10.55	0.31	10.66	0.10	1.01	0.014	6.1E-01
Mtr.24400.1.S1_at	2.41	2.94	2.39	2.35	2.39	2.36	2.58	0.31	2.36	0.02	0.92	-0.127	2.9E-01
Mtr.44713.1.S1_at	10.11	9.52	10.00	9.86	9.88	10.00	9.88	0.31	9.91	0.08	1.00	0.005	8.6E-01
Mtr.14030.1.S1_s_at	8.94	9.12	9.55	9.66	9.64	9.71	9.20	0.31	9.67	0.04	1.05	0.071	6.2E-02
Mtr.44424.1.S1_at	9.42	9.69	10.04	9.75	9.75	9.81	9.72	0.31	9.77	0.03	1.01	0.008	7.9E-01
Mtr.34996.1.S1_at	5.62	5.07	5.60	5.49	5.74	5.62	5.43	0.31	5.62	0.12	1.03	0.049	3.9E-01
Mtr.34862.1.S1_at	2.81	2.19	2.58	2.54	2.58	2.57	2.53	0.31	2.56	0.02	1.01	0.020	8.5E-01
Mtr.46588.1.S1_at	4.32	4.22	4.81	5.42	5.16	5.00	4.45	0.31	5.19	0.21	1.17	0.222	2.7E-02
Mtr.19678.1.S1_at	9.55	9.05	9.62	9.87	9.82	9.75	9.41	0.31	9.81	0.06	1.04	0.061	9.1E-02
Mtr.37698.1.S1_at	13.04	13.61	13.54	13.11	13.31	13.17	13.40	0.31	13.20	0.10	0.99	-0.022	3.5E-01
Mtr.11215.1.S1_at	9.86	10.17	10.48	10.39	10.06	10.05	10.17	0.31	10.16	0.20	1.00	-0.001	9.7E-01
Mtr.17696.1.S1_at	2.93	3.38	3.53	2.86	2.80	2.81	3.28	0.31	2.82	0.03	0.86	-0.218	6.3E-02
Mtr.4890.1.S1_at	9.51	8.98	9.53	9.52	9.50	9.55	9.34	0.31	9.52	0.03	1.02	0.028	3.6E-01
Mtr.8518.1.S1_s_at	11.82	12.36	11.82	11.44	12.06	11.90	12.00	0.31	11.80	0.32	0.98	-0.025	4.8E-01
Mtr.10666.1.S1_at	11.42	10.85	11.35	11.28	11.40	11.23	11.21	0.31	11.30	0.09	1.01	0.012	6.3E-01
Mtr.15503.1.S1_at	3.01	3.58	3.08	2.98	2.85	2.96	3.22	0.31	2.93	0.07	0.91	-0.137	1.9E-01
Mtr.5467.1.S1_at	8.21	7.80	7.61	8.18	8.16	8.29	7.87	0.31	8.21	0.07	1.04	0.060	1.4E-01
Mtr.3864.1.S1_at	3.66	3.17	3.75	3.75	4.22	3.83	3.53	0.31	3.93	0.25	1.12	0.158	1.5E-01
Mtr.40882.1.S1_at	11.49	10.90	11.03	10.85	10.89	10.85	11.14	0.31	10.86	0.02	0.97	-0.037	2.0E-01
Mtr.20293.1.S1_at	9.87	9.26	9.64	9.80	9.76	9.78	9.59	0.31	9.78	0.02	1.02	0.028	3.5E-01
Mtr.39139.1.S1_at	14.35	13.86	13.77	13.92	13.75	13.90	14.00	0.31	13.86	0.09	0.99	-0.014	5.0E-01
Mtr.7474.1.S1_at	3.02	3.52	2.96	3.09	3.10	3.05	3.17	0.31	3.08	0.03	0.97	-0.039	6.6E-01
Mtr.5420.1.S1_s_at	8.27	7.74	8.29	8.12	7.92	8.11	8.10	0.31	8.05	0.11	0.99	-0.009	8.1E-01
Mtr.17385.1.S1_at	9.83	9.33	9.90	9.82	9.81	9.81	9.68	0.31	9.82	0.01	1.01	0.020	5.0E-01
Mtr.36263.1.S1_at	9.72	9.11	9.51	9.49	9.62	9.47	9.45	0.31	9.53	0.08	1.01	0.012	6.9E-01
Mtr.47296.1.S1_at	2.16	2.70	2.15	2.14	2.08	2.15	2.34	0.31	2.13	0.04	0.91	-0.136	3.1E-01
Mtr.40288.1.S1_at	9.04	8.90	9.49	9.21	9.12	9.17	9.14	0.31	9.17	0.05	1.00	0.004	8.8E-01
Mtr.10695.1.S1_s_at	10.79	10.22	10.28	10.43	10.47	10.65	10.43	0.31	10.52	0.12	1.01	0.012	6.7E-01
Mtr.35141.1.S1_at	9.39	9.40	8.86	9.07	9.21	9.34	9.22	0.31	9.20	0.13	1.00	-0.002	9.6E-01
Mtr.13027.1.S1_at	9.11	8.49	8.78	8.95	9.10	9.01	8.80	0.31	9.02	0.08	1.03	0.036	2.9E-01
Mtr.7570.1.S1_at	8.48	7.86	8.20	8.76	9.67	9.38	8.18	0.31	9.27	0.46	1.13	0.181	2.7E-02
Mtr.11854.1.S1_at	10.14	9.55	9.99	10.11	10.09	9.93	9.89	0.31	10.05	0.10	1.02	0.022	4.6E-01
Mtr.39480.1.S1_at	7.94	7.61	8.23	8.03	7.92	7.92	7.93	0.31	7.96	0.06	1.00	0.006	8.6E-01
Mtr.23164.1.S1_at	2.80	3.32	2.76	2.76	2.76	2.70	2.96	0.31	2.74	0.03	0.93	-0.111	2.9E-01
Mtr.15674.1.S1_x_at	4.75	4.18	4.70	4.82	4.21	4.82	4.54	0.31	4.62	0.36	1.02	0.024	7.9E-01
Mtr.29626.1.S1_at	3.08	3.61	3.07	3.03	3.04	2.97	3.25	0.31	3.01	0.04	0.93	-0.111	2.6E-01
Mtr.50822.1.S1_at	4.95	5.50	4.96	4.91	4.78	4.92	5.14	0.31	4.87	0.08	0.95	-0.077	2.2E-01
Mtr.39672.1.S1_at	9.75	10.02	10.37	9.90	9.95	9.87	10.05	0.31	9.91	0.04	0.99	-0.021	4.8E-01
Mtr.35991.1.S1_at	9.33	8.98	9.61	9.47	9.64	9.38	9.31	0.31	9.50	0.13	1.02	0.030	3.8E-01
Mtr.32987.1.S1_at	11.21	11.02	10.60	11.01	10.91	11.10	10.95	0.31	11.00	0.10	1.01	0.008	7.7E-01
Mtr.18455.1.S1_s_at	11.43	11.07	11.69	11.43	11.41	11.40	11.40	0.31	11.42	0.01	1.00	0.002	9.3E-01
Mtr.37359.1.S1_at	9.29	9.41	9.88	9.38	9.35	9.36	9.53	0.31	9.36	0.02	0.98	-0.025	4.1E-01
Mtr.48638.1.S1_at	2.53	3.06	2.51	2.49	2.48	2.47	2.70	0.31	2.48	0.01	0.92	-0.126	2.8E-01
Mtr.39061.1.S1_s_at	8.32	7.78	8.32	8.31	8.21	8.38	8.14	0.31	8.30	0.08	1.02	0.028	4.4E-01
Mtr.13079.1.S1_s_at	8.36	7.82	8.36	8.39	8.50	8.42	8.18	0.31	8.43	0.06	1.03	0.044	2.4E-01
Mtr.17273.1.S1_s_at	11.37	11.98	11.56	10.68	11.06	10.90	11.64	0.31	10.88	0.19	0.93	-0.097	2.3E-02
Mtr.48567.1.S1_at	2.80	3.32	2.76	2.75	2.26	2.73	2.96	0.31	2.58	0.27	0.87	-0.198	1.9E-01
Mtr.40528.1.S1_s_at	6.65	6.04	6.47	6.01	6.38	6.55	6.39	0.31	6.31	0.27	0.99	-0.017	7.7E-01

Mtr.41504.1.S1_at	2.61	3.12	2.57	2.56	2.54	2.53	2.77	0.31	2.55	0.01	0.92	-0.120	2.9E-01
Mtr.42293.1.S1_at	7.34	6.75	6.87	6.99	7.06	7.13	6.99	0.31	7.06	0.07	1.01	0.015	7.0E-01
Mtr.26023.1.S1_at	3.21	3.72	3.16	3.14	3.10	3.09	3.36	0.31	3.11	0.02	0.93	-0.112	2.4E-01
Mtr.25080.1.S1_at	2.90	3.41	2.85	2.83	2.78	2.78	3.06	0.31	2.80	0.03	0.92	-0.128	2.2E-01
Mtr.39950.1.S1_at	11.15	11.27	11.74	11.74	11.63	11.56	11.39	0.31	11.65	0.09	1.02	0.032	2.4E-01
Mtr.27080.1.S1_at	8.19	7.88	8.50	8.33	8.39	8.29	8.19	0.31	8.34	0.05	1.02	0.025	4.7E-01
Mtr.13605.1.S1_at	8.18	7.70	8.29	8.22	8.60	8.46	8.06	0.31	8.43	0.19	1.05	0.065	1.5E-01
Mtr.47002.1.S1_s_at	3.50	3.39	3.97	3.46	3.52	3.47	3.62	0.31	3.48	0.03	0.96	-0.057	4.8E-01
Mtr.8856.1.S1_at	2.94	3.44	2.87	2.87	2.80	2.86	3.08	0.31	2.84	0.04	0.92	-0.117	2.5E-01
Mtr.38661.1.S1_at	7.97	8.04	8.54	8.17	7.89	8.04	8.18	0.31	8.04	0.14	0.98	-0.026	5.0E-01
Mtr.46699.1.S1_at	9.89	9.62	10.24	10.29	10.16	10.18	9.92	0.31	10.21	0.07	1.03	0.042	1.9E-01
Mtr.28299.1.S1_at	9.89	9.27	9.57	9.46	9.50	9.48	9.57	0.31	9.48	0.02	0.99	-0.014	6.2E-01
Mtr.37397.1.S1_at	8.89	9.33	9.49	9.42	9.80	9.57	9.23	0.31	9.60	0.19	1.04	0.056	1.6E-01
Mtr.3755.1.S1_at	2.36	2.89	2.35	2.33	2.32	2.34	2.53	0.31	2.33	0.01	0.92	-0.120	3.2E-01
Mtr.51148.1.S1_at	3.78	3.26	3.23	3.22	3.19	3.22	3.42	0.31	3.21	0.02	0.94	-0.092	3.0E-01
Mtr.29935.1.S1_at	3.04	3.57	3.04	3.01	3.01	3.03	3.22	0.31	3.02	0.01	0.94	-0.092	3.3E-01
Mtr.17067.1.S1_at	12.56	12.30	12.91	12.26	12.33	12.23	12.59	0.31	12.27	0.05	0.97	-0.037	1.6E-01
Mtr.43355.1.S1_at	10.89	11.39	11.47	11.37	11.35	11.30	11.25	0.31	11.34	0.04	1.01	0.012	6.3E-01
Mtr.43525.1.S1_at	9.68	9.15	9.13	9.26	9.11	9.02	9.32	0.31	9.13	0.12	0.98	-0.029	3.9E-01
Mtr.29271.1.S1_at	6.79	6.18	6.57	6.63	6.88	6.57	6.51	0.31	6.69	0.17	1.03	0.039	4.3E-01
Mtr.20877.1.S1_at	3.37	3.90	3.35	3.31	3.45	3.31	3.54	0.31	3.36	0.08	0.95	-0.077	3.7E-01
Mtr.7905.1.S1_at	2.69	3.22	2.67	2.66	2.63	2.66	2.86	0.31	2.65	0.02	0.93	-0.110	3.1E-01
Mtr.2532.1.S1_at	9.54	9.45	10.03	9.46	9.99	9.58	9.67	0.31	9.68	0.28	1.00	0.001	9.7E-01
Mtr.48945.1.S1_at	3.23	3.83	3.40	3.30	3.99	3.21	3.49	0.31	3.50	0.43	1.00	0.006	9.6E-01
Mtr.10683.1.S1_at	12.18	11.66	12.21	12.20	12.23	12.21	12.02	0.31	12.21	0.01	1.02	0.023	3.3E-01
Mtr.12862.1.S1_at	10.08	9.68	10.30	10.19	10.63	10.29	10.02	0.31	10.37	0.23	1.03	0.049	1.9E-01
Mtr.18911.1.S1_s_at	9.11	8.66	9.26	9.11	8.81	8.90	9.01	0.31	8.94	0.15	0.99	-0.011	7.4E-01
Mtr.18531.1.S1_at	3.08	3.53	2.94	2.92	2.87	2.85	3.18	0.31	2.88	0.03	0.90	-0.146	1.6E-01
Mtr.13135.1.S1_at	2.52	3.05	2.50	2.49	2.48	2.84	2.69	0.31	2.61	0.20	0.97	-0.045	7.2E-01
Mtr.18362.1.S1_at	2.84	3.38	2.83	2.83	2.82	2.84	3.02	0.31	2.83	0.01	0.94	-0.092	3.6E-01
Mtr.42765.1.S1_s_at	5.81	5.20	5.60	5.34	5.57	5.96	5.54	0.31	5.62	0.32	1.02	0.022	7.6E-01
Mtr.24453.1.S1_at	2.67	2.92	3.28	2.64	2.62	2.61	2.96	0.31	2.62	0.01	0.89	-0.174	1.4E-01
Mtr.39669.1.S1_at	7.47	6.97	7.55	7.28	7.17	7.48	7.33	0.31	7.31	0.16	1.00	-0.004	9.3E-01
Mtr.36349.1.S1_at	3.93	4.42	3.85	4.05	3.84	4.11	4.07	0.31	4.00	0.14	0.98	-0.024	7.5E-01
Mtr.40118.1.S1_at	12.19	12.05	12.65	12.55	12.67	12.63	12.30	0.31	12.62	0.06	1.03	0.037	1.5E-01
Mtr.31551.1.S1_at	10.33	10.63	10.95	10.53	10.65	10.49	10.64	0.31	10.56	0.09	0.99	-0.011	6.8E-01
Mtr.35427.1.S1_s_at	10.18	9.56	9.83	10.05	10.12	10.25	9.85	0.31	10.14	0.11	1.03	0.041	2.1E-01
Mtr.13223.1.S1_at	9.44	8.86	9.33	9.60	9.58	9.65	9.21	0.31	9.61	0.04	1.04	0.062	9.0E-02
Mtr.40365.1.S1_s_at	11.10	11.39	11.72	11.61	11.36	11.56	11.40	0.31	11.51	0.13	1.01	0.014	6.1E-01
Mtr.18873.1.S1_at	7.05	6.91	7.50	6.82	6.90	6.19	7.15	0.31	6.64	0.39	0.93	-0.108	1.5E-01
Mtr.8474.1.S1_s_at	10.71	10.54	10.11	11.16	11.10	11.21	10.45	0.31	11.16	0.06	1.07	0.094	1.8E-02
Mtr.37934.1.S1_at	11.39	10.80	11.25	11.14	11.14	11.05	11.15	0.31	11.11	0.05	1.00	-0.004	8.6E-01
Mtr.37782.1.S1_at	11.30	10.69	11.06	11.14	10.92	11.19	11.01	0.31	11.08	0.15	1.01	0.009	7.4E-01
Mtr.13969.1.S1_at	10.71	10.09	10.40	10.60	10.52	10.69	10.40	0.31	10.60	0.08	1.02	0.028	3.3E-01
Mtr.31039.1.S1_at	7.07	7.51	7.67	6.73	7.48	7.44	7.42	0.31	7.22	0.42	0.97	-0.040	5.4E-01
Mtr.45491.1.S1_at	3.13	3.09	3.65	3.28	3.51	3.08	3.29	0.31	3.29	0.22	1.00	0.000	1.0E+00
Mtr.11474.1.S1_at	9.76	9.48	9.14	9.64	9.68	9.78	9.46	0.31	9.70	0.07	1.03	0.037	2.6E-01
Mtr.10723.1.S1_at	13.03	12.82	13.43	13.83	13.96	13.87	13.10	0.31	13.89	0.07	1.06	0.085	1.2E-02
Mtr.12584.1.S1_at	9.96	10.40	10.56	10.23	10.36	10.14	10.31	0.31	10.24	0.11	0.99	-0.009	7.5E-01
Mtr.24869.1.S1_at	2.52	3.05	2.51	2.53	2.47	2.50	2.69	0.31	2.50	0.03	0.93	-0.108	3.4E-01
Mtr.47366.1.S1_at	8.41	8.20	8.81	8.43	8.55	8.51	8.47	0.31	8.50	0.06	1.00	0.004	9.0E-01
Mtr.51072.1.S1_at	2.61	3.13	2.58	2.58	2.58	2.55	2.78	0.31	2.57	0.02	0.92	-0.113	3.1E-01
Mtr.40003.1.S1_at	9.23	9.21	9.75	9.30	9.47	9.36	9.40	0.31	9.38	0.09	1.00	-0.003	9.2E-01
Mtr.12103.1.S1_at	10.36	9.82	10.36	10.22	10.12	10.33	10.18	0.31	10.22	0.11	1.00	0.006	8.4E-01
Mtr.27684.1.S1_at	8.18	7.56	7.86	7.95	8.29	8.40	7.87	0.31	8.21	0.24	1.04	0.062	2.0E-01
Mtr.13951.1.S1_at	7.83	8.34	8.39	8.06	8.20	7.70	8.19	0.31	7.99	0.26	0.98	-0.036	4.3E-01
Mtr.49405.1.S1_s_at	8.09	7.63	8.22	7.85	8.19	8.09	7.98	0.31	8.04	0.17	1.01	0.011	7.8E-01
Mtr.41232.1.S1_at	12.99	12.70	13.32	13.08	13.00	13.04	13.00	0.31	13.04	0.04	1.00	0.004	8.6E-01
Mtr.52206.1.S1_at	2.92	3.43	2.87	2.84	2.81	2.80	3.07	0.31	2.82	0.02	0.92	-0.125	2.3E-01
Mtr.39860.1.S1_at	2.35	2.25	2.83	2.34	2.34	2.32	2.48	0.31	2.33	0.01	0.94	-0.088	4.6E-01
Mtr.7525.1.S1_at	12.19	12.01	12.61	12.50	12.50	12.30	12.27	0.31	12.43	0.12	1.01	0.019	4.5E-01
Mtr.23593.1.S1_at	8.02	8.42	7.81	8.24	8.03	7.88	8.08	0.31	8.05	0.18	1.00	-0.005	8.9E-01
Mtr.8805.1.S1_at	9.99	10.61	10.25	10.28	10.40	10.23	10.28	0.31	10.30	0.09	1.00	0.002	9.3E-01
Mtr.46043.1.S1_s_at	9.90	9.28	9.60	9.90	9.85	9.73	9.59	0.31	9.83	0.09	1.02	0.035	2.7E-01
Mtr.49565.1.S1_at	11.56	10.95	11.31	11.57	11.56	11.53	11.27	0.31	11.55	0.02	1.02	0.035	2.0E-01
Mtr.20360.1.S1_at	3.37	3.05	2.75	3.01	3.01	3.01	3.06	0.31	3.01	0.00	0.98	-0.024	7.9E-01
Mtr.5913.1.S1_at	3.59	3.00	3.45	3.53	3.68	4.10	3.34	0.31	3.77	0.30	1.13	0.172	1.6E-01
Mtr.43411.1.S1_at	12.62	12.00	12.27	12.26	12.48	12.29	12.30	0.31	12.34	0.12	1.00	0.006	8.1E-01
Mtr.9934.1.S1_s_at	12.32	12.64	12.94	12.63	12.63	12.57	12.63	0.31	12.61	0.03	1.00	-0.002	9.2E-01
Mtr.9296.1.S1_at	11.00	10.82	10.40	10.95	10.77	11.06	10.74	0.31	10.93	0.15	1.02	0.025	4.0E-01
Mtr.41425.1.S1_at	9.82	9.30	9.86	9.83	9.85	9.99	9.66	0.31	9.89	0.09	1.02	0.034	2.8E-01
Mtr.35808.1.S1_at	8.42	8.37	8.93	8.31	8.44	8.39	8.57	0.31	8.38	0.07	0.98	-0.033	3.5E-01
Mtr.13786.1.S1_at	3.22	2.72	2.65	2.61	2.60	2.60	2.86	0.31	2.60	0.01	0.91	-0.136	2.2E-01
Mtr.16555.1.S1_at	4.29	4.38	3.80	4.12	4.12	4.11	4.16	0.31	4.12	0.00	0.99	-0.014	8.4E-01
Mtr.50200.1.S1_at	11.70	11.08	11.39	11.43	11.18	11.46	11.39	0.31	11.36	0.16	1.00	-0.004	8.7E-01
Mtr.43374.1.S1_at	11.09	11.47	11.71	11.46	11.54	11.34	11.42	0.31	11.44	0.10	1.00	0.002	9.2E-01
Mtr.1567.1.S1_at	10.36	10.06	9.75	10.47	10.41	10.69	10.06	0.31	10.53	0.15	1.05	0.066	7.6E-02
Mtr.44172.1.S1_at	9.28	8.73	9.25	9.28	9.30	9.25	9.09	0.31	9.28	0.03	1.02	0.030	3.5E-01
Mtr.11095.1.S1_at	9.35	8.74	9.17	9.18	9.30	9.10	9.09	0.31	9.19	0.10	1.01	0.017	6.0E-01
Mtr.52357.1.S1_s_at	11.93	11.84	11.35	11.95	11.84	11.98	11.71	0.31	11.92	0.07	1.02	0.026	3.1E-01
Mtr.8951.1.S1_at	9.70	10.31	9.97	9.95	9.91	9.91	9.99	0.31	9.92	0.02	0.99	-0.010	7.2E-01
Mtr.32171.1.S1_x_at	12.05	11.45	11.62	11.83	11.67	11.84	11.71	0.31	11.78	0.09	1.01	0.009	7.1E-01
Mtr.43312.1.S1_at	3.51	3.54	4.06	3.55	3.43	3.85	3.70	0.31	3.61	0.22	0.97	-0.037	6.9E-01
Mtr.37023.1.S1_s_at	11.16	11.60	11.00	11.26	11.47	10.96	11.25	0.31	11.23	0.25	1.00	-0.003	9.3E-01
Mtr.46125.1.S1_s_at	10.75	10.57	11.17	10.88	11.01	10.82	10.83	0.31	10.90	0.10	1.01	0.010	7.2E-01
Mtr.46471.1.S1_at	4.41	4.47	3.91	4.32	4.32	4.31	4.26	0.31	4.32	0.00	1.01	0.019	7.7E-01
Mtr.52208.1.S1_at	2.12	2.15	2.67	2.02	3.19	2.35	2.32	0.31	2.52	0.60	1.09	0.123	6.3E-01
Mtr.42624.1.S1_s_at	9.50	8.88	9.21	9.52	9.25	9.43	9.19	0.31	9.40	0.14	1.02	0.032	3.5E-01
Mtr.38106.1.S1_s_at	9.13	8.51	8.83	9.01	8.86	8.97	8.82	0.31	8.95	0.08	1.01	0.020	5.4E-01
Mtr.3591.1.S1_at	2.84	2.91	3.40	2.82	2.82	2.78	3.05	0.31	2.81	0.03	0.92	-0.119	2.5E-01
Mtr.9332.1.S1_at	10.26	10.55	10.88	10.36	10.44	10.48	10.56	0.31	10.43	0.06	0.99	-0.019	5.0E-01
Mtr.43112.1.S1_at	10.78	10.66	10.19	10.23	10.47	10.51	10.54	0.31	10.40	0.15	0.99	-0.019	5.3E-01
Mtr.32998.1.S1_s_at	9.93	9.40	9.39	9.41	9.68	9.76	9.57	0.31	9.62	0.18	1.00	0.006	8.5E-01
Mtr.28324.1.S1_at	8.80	8.43	8.18	8.74	8.52	8.73	8.47	0.31	8.66	0.12	1.02	0.032	3.8E-01
Mtr.33183.1.S1_at	11.76	11.60	12.20	11.67	11.79	11.74	11.85	0.31	11.73	0.06	0.99	-0.015	5.4E-01
Mtr.16606.1.S1_at	3.56	3.14	2.95	2.91	2.87	2.91	3.21	0.31	2.90	0.02	0.90	-0.151	1.5E-01
Mtr.9612.1.S1_at	8.14	7.55	7.99	8.00	8.02	8.08	7.89	0.31	8.03	0.05	1.02	0.025	4.8E-01
Mtr.29621.1.S1_at	3.06	3.55	2.97	2.94	2.90	2.87	3.19	0.31	2.91	0.04	0.91	-0.136	1.8E-01
Mtr.4769.1.S1_at	9.92	9.32	9.73	9.49	9.83	9.55	9.65	0.31	9.62	0.18	1.00	-0.005	8.9E-01
Mtr.35069.1.S1_at	2.82	3.34	2.79	3.20	2.70	2.69	2.98	0.31	2.86	0.29	0.96	-0.058	6.5E-01
Mtr.41453.1.S1_at	9.90	9.34	9.39	9.83	10.30	10.26	9.54	0.31	10.13	0.26	1.06	0.087	6.5E-02
Mtr.24842.1.S1_at	12.47	12.90	13.08	12.27	12.08	12.30	12.82	0.31	12.22	0.12	0.95	-0.069	3.5E-02
Mtr.3315.1.S1_at	11.06	10.74	11.35	11.11	11.31	11.03	11.05	0.31	11.15	0.14	1.01	0.013	6.4E-01
Mtr.39711.1.S1_at	9.30	8.78	9.33	9.36	9.37	9.31	9.14	0.31	9.35	0.03	1.02	0.032	3.2E-01
Mtr.9006.1.S1_at	11.23	11.75	11.79	11.20	11.22	11.26	11.59	0.31	11.23	0.03	0.97	-0.046	1.1E-01
Mtr.35932.1.S1_at	10.27	9.84	10.43	10.18	10.66	10.18	10.18	0.31	10.34	0.28	1.02	0.022	5.4E-01
Mtr.46986.1.S1_at	2.74	3.27	2.73	2.73	2.72	2.70	2.91	0.31	2.72	0.01	0.93	-0.102	3.3E-01
Mtr.35391.1.S1_s_at	8.33	7.73	8.15	8.01	8.07	8.38	8.07	0.31	8.15	0.20	1.01	0.015	7.1E-01
Mtr.19865.1.S1_at	2.13	2.66	2.12	2.11	2.11	2.11	2.30	0.31	2.11	0.00	0.92	-0.125	3.5E-01
Mtr.13581.1.S1_at	9.49	8.95	9.48	9.38	9.39	9.18	9.31	0.31	9.32	0.12	1.00	0.002	9.5E-01
Mtr.17821.1.S1_at	2.95	3.47	2.93	2.93	2.93	2.89	3.12	0.31	2.92	0.02	0.94	-0.094	3.4E-01
Mtr.40514.1.S1_at	5.72	5.11	5.52	5.44	5.61	5.47	5.45	0.31	5.51	0.09	1.01	0.016	7.6E-01
Mtr.21530.1.S1_at	8.52	7.99	8.53	8.56	8.59	8.58	8.34	0.31	8.58	0.02	1.03	0.040	2.6E-01
Mtr.50123.1.S1_at	12.82	12.62	13.22	12.99	12.79	13.03	12.89	0.31	12.94	0.13	1.00	0.005	8.2E-01
Mtr.11865.1.S1_s_at	11.03	10.44	10.60	10.73	10.60	10.91	10.69	0.31	10.74	0.15	1.00	0.007	8.1E-01
Mtr.5147.1.S1_at	8.72	8.43	9.05	9.04	8.53	9.03	8.73	0.31	8.87	0.29	1.02	0.022	6.1E-01
Mtr.30078.1.S1_at	7.16	7.09	7.66	7.22	7.55	7.21	7.30	0.31	7.33	0.19	1.00	0.005	9.2E-01
Mtr.39408.1.S1_at	9.19	9.40	9.80	9.45	9.63	9.47	9.46	0.31	9.52	0.10	1.01	0.008	7.9E-01
Mtr.44403.1.S1_at	3.82	3.49	3.21	3.53	3.40	3.86	3.51	0.31	3.60	0.24	1.03	0.037	7.1E-01
Mtr.36000.1.S1_at	3.07	3.56	2.99	2.98	2.97	2.98	3.21	0.31	2.98	0.00	0.93	-0.108	2.6E-01
Mtr.2387.1.S1_at	2.82	2.25	2.74	2.15	2.15	2.30	2.60	0.31	2.20	0.08	0.85	-0.242	9.5E-02
Mtr.6258.1.S1_at	10.70	11.17	11.29	11.11	11.27	11.08	11.05	0.31	11.15	0.10	1.01	0.013	6.2E-01
Mtr.4600.1.S1_at	9.44	8.97	9.56	9.50	9.76	9.48	9.32	0.31	9.58	0.16	1.03	0.039	2.7E-01
Mtr.49116.1.S1_at	3.23	3.74	3.18	3.18	3.16	3.15	3.38	0.31	3.16	0.01	0.94	-0.097	2.9E-01
Mtr.7408.1.S1_at	3.57	2.95	3.28	3.65	3.29	3.23	3.27	0.31	3.39	0.23	1.04	0.053	6.1E-01
Mtr.41652.1.S1_at	8.27	7.78	8.36	8.08	8.17	8.15	8.14	0.31	8.13	0.05	1.00	-0.001	9.7E-01
Mtr.4141.1.S1_at	10.34	9.73	10.14	10.21	10.22	10.31	10.07	0.31	10.25	0.05	1.02	0.025	3.9E-01
Mtr.31704.1.S1_at	9.90	9.52	10.13	9.79	9.76	9.82	9.85	0.31	9.79	0.03	0.99	-0.009	7.5E-01
Mtr.36870.1.S1_at	8.97	9.34	9.58	9.41	9.50	9.42	9.30	0.31	9.44	0.05	1.02	0.023	4.6E-01
Mtr.15053.1.S1_at	3.66	3.76	3.18	5.04	6.03	4.61	3.53	0.31	5.23	0.73	1.48	0.566	2.0E-02
Mtr.32613.1.S1_s_at	9.43	8.81	9.14	8.99	9.17	9.15	9.13	0.31	9.11	0.10	1.00	-0.003	9.2E-01

Mtr.41547.1.S1_at	9.25	8.63	8.99	9.20	9.28	9.46	8.96	0.31	9.31	0.14	1.04	0.056	1.4E-01
Mtr.41244.1.S1_at	10.66	11.00	11.28	10.55	10.73	10.77	10.98	0.31	10.68	0.12	0.97	-0.040	1.9E-01
Mtr.24166.1.S1_at	2.50	3.01	2.45	2.44	2.44	2.43	2.65	0.31	2.44	0.01	0.92	-0.123	2.9E-01
Mtr.9554.1.S1_at	12.84	13.33	13.41	13.19	13.20	13.02	13.19	0.31	13.14	0.10	1.00	-0.006	7.8E-01
Mtr.4629.1.S1_at	9.45	9.05	9.66	9.44	9.39	9.28	9.39	0.31	9.37	0.08	1.00	-0.003	9.3E-01
Mtr.24599.1.S1_at	10.04	10.25	10.65	10.31	10.37	10.21	10.32	0.31	10.29	0.08	1.00	-0.003	9.1E-01
Mtr.14752.1.S1_at	2.66	3.18	2.63	2.63	2.60	2.59	2.83	0.31	2.61	0.02	0.92	-0.115	2.9E-01
Mtr.18581.1.S1_at	8.29	7.76	8.31	8.19	8.31	8.17	8.12	0.31	8.22	0.07	1.01	0.019	5.9E-01
Mtr.11633.1.S1_at	8.65	8.12	8.11	8.50	8.47	8.66	8.30	0.31	8.54	0.10	1.03	0.042	2.6E-01
Mtr.4260.1.S1_at	10.26	10.36	10.84	10.43	10.52	10.55	10.49	0.31	10.50	0.06	1.00	0.002	9.4E-01
Mtr.30453.1.S1_at	8.73	9.34	8.93	9.39	10.17	9.29	9.00	0.31	9.62	0.48	1.07	0.096	1.4E-01
Mtr.32307.1.S1_s_at	12.55	12.01	12.03	12.30	12.15	12.35	12.19	0.31	12.27	0.10	1.01	0.009	7.1E-01
Mtr.5745.1.S1_at	9.26	9.15	9.73	9.21	9.23	9.13	9.38	0.31	9.19	0.05	0.98	-0.030	3.5E-01
Mtr.41587.1.S1_s_at	9.10	8.70	8.49	8.79	8.43	8.78	8.76	0.31	8.67	0.21	0.99	-0.016	6.8E-01
Mtr.31998.1.S1_at	10.02	9.61	10.21	9.73	9.57	9.31	9.95	0.31	9.54	0.21	0.96	-0.061	1.3E-01
Mtr.1207.1.S1_at	2.97	3.54	3.04	2.91	2.91	2.89	3.18	0.31	2.90	0.01	0.91	-0.131	2.0E-01
Mtr.45215.1.S1_at	9.99	9.39	9.83	10.14	10.13	10.06	9.74	0.31	10.11	0.04	1.04	0.054	1.1E-01
Mtr.26898.1.S1_at	9.12	8.56	9.06	9.17	9.23	9.27	8.91	0.31	9.22	0.05	1.03	0.050	1.6E-01
Mtr.22075.1.S1_at	3.20	3.73	3.18	3.18	3.18	2.92	3.37	0.31	3.09	0.15	0.92	-0.125	2.3E-01
Mtr.48842.1.S1_at	13.44	13.98	13.46	13.74	13.85	13.95	13.63	0.31	13.85	0.11	1.02	0.023	3.1E-01
Mtr.9419.1.S1_at	10.74	10.15	10.61	10.76	10.60	10.94	10.50	0.31	10.77	0.17	1.03	0.036	2.6E-01
Mtr.5246.1.S1_s_at	9.25	8.64	8.95	9.27	9.07	9.20	8.95	0.31	9.18	0.10	1.03	0.037	2.8E-01
Mtr.6310.1.S1_at	3.27	2.78	2.70	2.70	2.69	2.69	2.92	0.31	2.69	0.00	0.92	-0.116	2.7E-01
Mtr.44779.1.S1_s_at	2.63	2.90	3.25	2.87	3.11	2.64	2.93	0.31	2.87	0.24	0.98	-0.027	8.2E-01
Mtr.12318.1.S1_at	13.17	12.56	12.81	12.82	13.03	12.75	12.85	0.31	12.87	0.15	1.00	0.002	9.2E-01
Mtr.48115.1.S1_at	4.10	4.47	4.71	5.01	5.32	4.76	4.43	0.31	5.03	0.28	1.14	0.184	6.6E-02
Mtr.38945.1.S1_at	8.92	8.31	8.52	8.35	8.40	8.35	8.58	0.31	8.37	0.03	0.97	-0.037	2.9E-01
Mtr.12308.1.S1_at	11.34	11.23	11.81	11.35	11.14	11.17	11.46	0.31	11.22	0.11	0.98	-0.031	2.7E-01
Mtr.10294.1.S1_at	10.10	9.48	9.85	9.99	10.06	10.17	9.81	0.31	10.07	0.09	1.03	0.038	2.3E-01
Mtr.35786.1.S1_at	9.17	8.59	9.05	9.38	9.39	9.37	8.93	0.31	9.38	0.01	1.05	0.070	6.6E-02
Mtr.41619.1.S1_at	11.34	10.79	11.30	11.17	11.11	11.14	11.15	0.31	11.14	0.03	1.00	0.000	9.9E-01
Mtr.24513.1.S1_at	8.88	8.27	8.48	8.65	8.61	8.63	8.54	0.31	8.63	0.02	1.01	0.015	6.4E-01
Mtr.23229.1.S1_at	2.93	3.43	2.86	2.87	2.88	2.78	3.07	0.31	2.84	0.05	0.93	-0.111	2.7E-01
Mtr.32099.1.S1_at	7.97	8.06	8.54	8.32	8.44	8.24	8.19	0.31	8.33	0.10	1.02	0.025	4.9E-01
Mtr.40897.1.S1_at	12.00	11.46	11.48	11.56	11.44	11.38	11.65	0.31	11.46	0.09	0.98	-0.024	3.7E-01
Mtr.13680.1.S1_at	3.68	3.24	3.08	3.02	3.01	2.87	3.33	0.31	2.96	0.08	0.89	-0.169	1.2E-01
Mtr.13230.1.S1_at	11.32	11.83	11.87	11.75	11.72	11.80	11.67	0.31	11.76	0.04	1.01	0.011	6.5E-01
Mtr.32114.1.S1_s_at	11.21	11.08	11.67	11.48	11.66	11.48	11.32	0.31	11.54	0.10	1.02	0.027	3.1E-01
Mtr.42666.1.S1_at	10.62	10.88	11.23	11.07	11.20	11.01	10.91	0.31	11.09	0.10	1.02	0.024	3.9E-01
Mtr.38426.1.S1_at	8.08	8.19	8.66	8.22	8.39	8.00	8.31	0.31	8.20	0.20	0.99	-0.018	6.5E-01
Mtr.6964.1.S1_x_at	8.91	8.33	8.46	8.68	8.60	8.50	8.57	0.31	8.59	0.09	1.00	0.004	9.1E-01
Mtr.12636.1.S1_at	11.37	11.42	11.92	11.76	11.79	11.63	11.57	0.31	11.73	0.09	1.01	0.019	4.4E-01
Mtr.50352.1.S1_at	5.00	5.44	4.84	4.83	4.85	4.92	5.09	0.31	4.87	0.04	0.96	-0.065	2.8E-01
Mtr.25929.1.S1_at	2.69	3.22	2.68	2.68	2.68	2.65	2.86	0.31	2.67	0.02	0.93	-0.102	3.3E-01
Mtr.11031.1.S1_s_at	8.95	8.43	8.99	8.94	8.75	8.85	8.79	0.31	8.85	0.09	1.01	0.010	7.7E-01
Mtr.12119.1.S1_at	9.88	10.27	10.49	10.14	10.21	9.63	10.22	0.31	9.99	0.32	0.98	-0.032	4.3E-01
Mtr.19919.1.S1_at	2.32	2.35	2.87	2.28	2.28	2.31	2.51	0.31	2.29	0.02	0.91	-0.132	2.8E-01
Mtr.4130.1.S1_at	3.35	2.85	2.79	2.82	2.79	2.90	3.00	0.31	2.84	0.06	0.95	-0.079	4.3E-01
Mtr.47238.1.S1_at	2.22	2.74	2.21	2.46	2.21	1.99	2.39	0.31	2.22	0.24	0.93	-0.106	4.9E-01
Mtr.37742.1.S1_s_at	11.04	10.50	10.52	10.77	10.48	10.66	10.69	0.31	10.63	0.15	1.00	-0.007	8.0E-01
Mtr.46179.1.S1_at	2.50	3.03	2.48	2.48	2.45	2.45	2.67	0.31	2.46	0.02	0.92	-0.119	3.0E-01
Mtr.40839.1.S1_at	11.88	11.30	11.77	11.71	11.66	11.73	11.65	0.31	11.70	0.04	1.00	0.006	7.9E-01
Mtr.4799.1.S1_at	12.29	11.79	12.34	11.72	11.86	11.72	12.14	0.31	11.77	0.08	0.97	-0.045	1.1E-01
Mtr.1942.1.S1_at	9.32	8.75	9.24	9.11	9.34	9.08	9.10	0.31	9.18	0.14	1.01	0.012	7.2E-01
Mtr.47275.1.S1_at	2.92	3.42	2.85	2.83	2.80	2.82	3.06	0.31	2.82	0.02	0.92	-0.120	2.4E-01
Mtr.15914.1.S1_at	12.74	12.20	12.73	12.74	12.61	12.59	12.56	0.31	12.65	0.08	1.01	0.010	6.6E-01
Mtr.3341.1.S1_at	2.73	3.25	2.71	2.69	2.66	2.66	2.90	0.31	2.67	0.02	0.92	-0.119	2.7E-01
Mtr.37354.1.S1_s_at	13.60	14.13	14.14	13.78	13.79	13.70	13.96	0.31	13.76	0.05	0.99	-0.021	3.3E-01
Mtr.11064.1.S1_at	11.76	11.89	12.35	12.00	11.99	11.99	12.00	0.31	12.00	0.00	1.00	-0.001	9.8E-01
Mtr.35216.1.S1_at	9.82	10.05	10.43	10.00	9.99	9.98	10.10	0.31	9.99	0.01	0.99	-0.016	5.7E-01
Mtr.15737.1.S1_at	8.04	7.45	7.89	8.00	8.20	8.11	7.79	0.31	8.11	0.10	1.04	0.057	1.7E-01
Mtr.12509.1.S1_at	13.48	13.03	12.89	13.24	13.01	13.34	13.13	0.31	13.20	0.17	1.01	0.007	7.6E-01
Mtr.984.1.S1_at	3.09	3.63	3.10	3.07	3.04	3.06	3.27	0.31	3.06	0.02	0.93	-0.098	2.9E-01
Mtr.39621.1.S1_at	2.52	1.91	2.13	2.63	2.50	2.69	2.19	0.31	2.60	0.10	1.19	0.252	8.8E-02
Mtr.15969.1.S1_at	8.17	8.45	8.78	8.37	8.35	8.55	8.47	0.31	8.42	0.11	0.99	-0.007	8.3E-01
Mtr.10641.1.S1_at	11.53	10.95	11.06	11.42	11.30	11.44	11.18	0.31	11.38	0.07	1.02	0.026	3.2E-01
Mtr.9998.1.S1_at	11.33	11.21	11.79	11.30	11.53	11.32	11.44	0.31	11.38	0.13	0.99	-0.008	7.6E-01
Mtr.42567.1.S1_at	3.08	3.63	3.12	3.05	3.02	3.05	3.28	0.31	3.04	0.02	0.93	-0.110	2.5E-01
Mtr.8870.1.S1_at	10.69	10.08	10.47	10.16	10.55	10.27	10.42	0.31	10.33	0.20	0.99	-0.013	6.9E-01
Mtr.14041.1.S1_s_at	11.03	11.50	11.61	11.39	11.85	11.48	11.38	0.31	11.58	0.24	1.02	0.024	4.4E-01
Mtr.32984.1.S1_at	3.80	3.79	4.32	3.78	3.78	3.77	3.97	0.31	3.77	0.00	0.95	-0.073	3.3E-01
Mtr.6941.1.S1_at	2.37	2.96	2.82	5.37	4.73	4.36	2.72	0.31	4.82	0.51	1.77	0.827	3.6E-03
Mtr.48833.1.S1_s_at	2.95	2.46	2.38	2.37	2.37	2.36	2.59	0.31	2.37	0.00	0.91	-0.131	2.7E-01
Mtr.13486.1.S1_at	8.41	8.08	8.70	8.42	8.92	8.48	8.40	0.31	8.61	0.27	1.02	0.035	4.3E-01
Mtr.25719.1.S1_at	1.83	2.36	1.82	1.82	1.82	1.82	2.00	0.31	1.82	0.00	0.91	-0.140	3.6E-01
Mtr.45091.1.S1_at	3.45	3.98	3.45	3.41	4.22	3.88	3.63	0.31	3.84	0.41	1.06	0.080	5.2E-01
Mtr.39103.1.S1_at	7.60	7.66	8.16	7.86	7.80	7.56	7.81	0.31	7.74	0.16	0.99	-0.012	7.5E-01
Mtr.44748.1.S1_at	8.29	7.75	7.77	8.13	7.98	7.98	7.93	0.31	8.03	0.09	1.01	0.017	6.3E-01
Mtr.43048.1.S1_at	4.10	4.57	3.99	3.97	3.94	3.96	4.22	0.31	3.96	0.01	0.94	-0.092	2.2E-01
Mtr.44801.1.S1_at	7.61	7.83	8.21	7.72	7.66	7.47	7.88	0.31	7.62	0.13	0.97	-0.050	2.3E-01
Mtr.49422.1.S1_at	9.13	8.84	9.46	9.31	9.21	9.18	9.14	0.31	9.23	0.07	1.01	0.014	6.4E-01
Mtr.31136.1.S1_x_at	3.09	3.61	3.06	2.94	3.04	3.03	3.25	0.31	3.01	0.05	0.92	-0.114	2.4E-01
Mtr.44177.1.S1_at	11.10	10.54	11.03	11.15	11.16	11.06	10.89	0.31	11.12	0.05	1.02	0.031	2.6E-01
Mtr.12130.1.S1_at	9.08	9.45	9.69	9.37	9.36	9.27	9.41	0.31	9.33	0.06	0.99	-0.012	6.9E-01
Mtr.51705.1.S1_at	6.77	6.23	6.76	6.90	6.83	6.91	6.59	0.31	6.88	0.04	1.04	0.063	1.7E-01
Mtr.13133.1.S1_at	5.50	5.20	5.81	5.97	6.99	6.19	5.50	0.31	6.38	0.53	1.16	0.215	6.8E-02
Mtr.1669.1.S1_at	9.90	9.45	10.03	9.88	9.89	9.99	9.79	0.31	9.92	0.06	1.01	0.018	5.2E-01
Mtr.34939.1.S1_at	5.84	5.35	5.27	5.12	5.18	5.53	5.49	0.31	5.28	0.22	0.96	-0.056	3.9E-01
Mtr.28593.1.S1_at	3.59	3.12	3.69	3.58	3.63	3.58	3.47	0.31	3.60	0.03	1.04	0.053	5.1E-01
Mtr.15651.1.S1_at	3.03	3.53	2.98	2.98	2.99	2.98	3.18	0.31	2.98	0.01	0.94	-0.093	3.3E-01
Mtr.18379.1.S1_at	7.43	6.97	7.55	7.47	7.54	7.38	7.31	0.31	7.46	0.08	1.02	0.029	4.6E-01
Mtr.1176.1.S1_x_at	2.97	3.50	2.96	2.94	2.85	2.83	3.14	0.31	2.88	0.06	0.91	-0.129	2.1E-01
Mtr.33702.1.S1_s_at	10.49	9.90	10.32	10.46	10.32	10.45	10.24	0.31	10.41	0.08	1.02	0.024	4.1E-01
Mtr.9413.1.S1_at	10.23	9.63	10.05	10.06	10.16	10.10	9.97	0.31	10.11	0.05	1.01	0.019	5.0E-01
Mtr.13564.1.S1_s_at	9.68	9.16	9.71	9.30	9.00	9.08	9.52	0.31	9.13	0.15	0.96	-0.060	1.2E-01
Mtr.45733.1.S1_at	9.14	8.94	9.54	9.43	9.44	9.29	9.21	0.31	9.39	0.08	1.02	0.028	3.9E-01
Mtr.29085.1.S1_s_at	8.81	8.32	8.89	8.68	8.93	8.74	8.67	0.31	8.78	0.13	1.01	0.018	6.0E-01
Mtr.11910.1.S1_at	8.80	8.37	8.20	8.42	8.77	8.54	8.46	0.31	8.58	0.17	1.01	0.021	5.8E-01
Mtr.30504.1.S1_at	5.04	4.48	4.55	4.74	5.06	5.05	4.69	0.31	4.95	0.18	1.06	0.078	2.7E-01
Mtr.13878.1.S1_s_at	5.34	5.17	5.77	5.71	5.71	5.73	5.43	0.31	5.72	0.01	1.05	0.075	1.8E-01
Mtr.10699.1.S1_at	14.34	13.73	14.07	14.00	13.85	13.98	14.05	0.31	13.94	0.08	0.99	-0.011	6.0E-01
Mtr.36328.1.S1_at	4.01	4.20	3.60	3.92	4.07	3.92	3.94	0.31	3.97	0.09	1.01	0.011	8.7E-01
Mtr.12208.1.S1_at	12.85	12.59	12.24	12.96	12.87	13.13	12.56	0.31	12.99	0.13	1.03	0.048	9.1E-02
Mtr.39345.1.S1_at	10.18	10.44	10.79	10.54	10.48	10.41	10.47	0.31	10.48	0.06	1.00	0.001	9.8E-01
Mtr.13361.1.S1_at	9.27	8.68	9.14	9.06	9.17	9.14	9.03	0.31	9.13	0.06	1.01	0.015	6.2E-01
Mtr.6734.1.S1_s_at	10.11	9.59	10.13	9.97	9.83	9.93	9.94	0.31	9.91	0.07	1.00	-0.005	8.7E-01
Mtr.2040.1.S1_at	3.40	3.86	3.98	4.09	3.51	4.50	3.75	0.31	4.03	0.50	1.08	0.106	4.5E-01
Mtr.43600.1.S1_at	11.89	11.33	11.38	11.63	11.73	11.71	11.53	0.31	11.69	0.05	1.01	0.019	4.3E-01
Mtr.1148.1.S1_at	2.92	3.44	2.90	3.69	2.83	2.81	3.08	0.31	3.11	0.50	1.01	0.012	9.4E-01
Mtr.5907.1.S1_at	9.64	10.00	10.25	10.07	9.95	9.87	9.96	0.31	9.97	0.10	1.00	0.001	9.8E-01
Mtr.12338.1.S1_at	12.07	11.46	11.73	11.66	11.77	11.68	11.75	0.31	11.70	0.06	1.00	-0.006	7.9E-01
Mtr.31718.1.S1_at	8.57	8.50	9.06	8.48	8.66	8.88	8.71	0.31	8.68	0.20	1.00	-0.006	8.8E-01
Mtr.12903.1.S1_at	10.22	9.62	10.04	10.16	10.33	10.21	9.96	0.31	10.23	0.09	1.03	0.039	2.1E-01
Mtr.42316.1.S1_at	2.76	2.18	2.65	2.74	2.73	2.83	2.53	0.31	2.77	0.06	1.09	0.128	2.6E-01
Mtr.33696.1.S1_at	6.77	6.33	6.18	6.81	5.98	6.43	6.42	0.31	6.40	0.41	1.00	-0.005	9.5E-01
Mtr.2438.1.S1_at	4.85	4.33	4.30	3.74	4.32	4.44	4.49	0.31	4.17	0.37	0.93	-0.109	3.1E-01
Mtr.21267.1.S1_at	8.95	9.05	9.52	9.21	9.09	9.04	9.17	0.31	9.11	0.09	0.99	-0.009	7.7E-01
Mtr.20337.1.S1_at	8.46	7.99	8.56	7.84	8.30	8.03	8.34	0.31	8.06	0.23	0.97	-0.049	2.8E-01
Mtr.48704.1.S1_x_at	12.05	11.44	11.84	11.80	11.82	11.92	11.78	0.31	11.85	0.06	1.01	0.008	7.2E-01
Mtr.16071.1.S1_x_at	6.25	6.75	6.19	6.18	6.14	6.16	6.40	0.31	6.16	0.02	0.96	-0.054	2.6E-01
Mtr.28822.1.S1_s_at	4.69	4.27	4.09	4.23	4.32	4.07	4.35	0.31	4.20	0.13	0.97	-0.049	4.9E-01
Mtr.15044.1.S1_at	8.32	8.92	8.51	8.50	8.80	8.42	8.58	0.31	8.57	0.20	1.00	-0.001	9.7E-01
Mtr.11903.1.S1_at	9.46	9.48	8.94	9.58	9.46	9.76	9.29	0.31	9.60	0.15	1.03	0.047	1.9E-01
Mtr.25202.1.S1_at	2.57	3.09	2.55	2.55	2.51	2.54	2.74	0.31	2.53	0.02	0.93	-0.111	3.2E-01
Mtr.10270.1.S1_at	9.42	9.99	9.53	9.77	9.48	9.75	9.64	0.31	9.67	0.16	1.00	0.003	9.1E-01
Mtr.28065.1.S1_at	11.29	11.90	11.67	11.27	11.47	11.47	11.62	0.31	11.40	0.11	0.98	-0.027	3.1E-01
Mtr.23753.1.S1_at	4.86	4.46	5.06	4.89	4.99	4.85	4.79	0.31	4.91	0.07	1.02	0.035	5.5E-01
Mtr.46735.1.S1_x_at	4.32	4.60	3.99	4.69	6.21	5.45	4.31	0.31	5.45	0.76	1.27	0.340	7.3E-02
Mtr.3149.1.S1_at	8.85	8.32	8.85	8.89	9.23	9.02	8.67	0.31	9.05	0.17	1.04	0.061	1.4E-01
Mtr.32141.1.S1_at	2.01	2.54	2.00	2.00	2.00	2.00	2.18	0.31	2.00	0.00	0.92	-0.126	3.6E-01
Mtr.35621.1.S1_at	10.32	9.71	10.10	10.06	10.24	9.88	10.04	0.31	10.06	0.18	1.00	0.002	9.4E-01

Mtr.13163.1.S1_at	11.19	11.55	11.80	11.61	11.67	11.49	11.51	0.31	11.59	0.09	1.01	0.010	7.0E-01
Mtr.24463.1.S1_at	3.13	3.68	3.16	3.17	3.17	2.96	3.32	0.31	3.10	0.13	0.93	-0.099	3.1E-01
Mtr.33253.1.S1_at	10.87	10.38	10.31	10.67	10.67	10.87	10.52	0.31	10.74	0.11	1.02	0.029	3.2E-01
Mtr.13091.1.S1_at	11.76	11.22	11.25	11.57	11.37	11.52	11.41	0.31	11.49	0.10	1.01	0.010	7.0E-01
Mtr.37812.1.S1_at	11.53	11.25	11.86	11.64	11.48	11.52	11.55	0.31	11.55	0.08	1.00	0.000	9.8E-01
Mtr.35819.1.S1_s_at	8.88	8.41	8.99	9.16	9.05	8.95	8.76	0.31	9.05	0.10	1.03	0.047	1.9E-01
Mtr.41646.1.S1_at	9.54	8.97	9.45	9.32	9.26	9.25	9.32	0.31	9.28	0.04	1.00	-0.006	8.3E-01
Mtr.13220.1.S1_at	9.64	9.22	9.82	9.64	9.63	9.68	9.56	0.31	9.65	0.02	1.01	0.014	6.3E-01
Mtr.38904.1.S1_at	9.95	9.69	10.30	9.97	10.30	10.08	9.98	0.31	10.12	0.17	1.01	0.019	5.4E-01
Mtr.20865.1.S1_at	9.58	8.97	9.23	9.63	9.67	9.53	9.26	0.31	9.61	0.07	1.04	0.054	1.2E-01
Mtr.48420.1.S1_at	2.85	3.37	2.84	2.83	2.84	2.83	3.02	0.31	2.83	0.00	0.94	-0.092	3.5E-01
Mtr.19881.1.S1_x_at	2.76	3.27	2.72	2.74	2.65	2.73	2.92	0.31	2.71	0.05	0.93	-0.108	3.1E-01
Mtr.14862.1.S1_at	2.72	3.24	2.71	4.73	3.93	3.15	2.89	0.31	3.93	0.79	1.36	0.444	1.0E-01
Mtr.8063.1.S1_at	4.19	3.65	4.16	4.35	4.72	4.69	4.00	0.31	4.59	0.20	1.15	0.197	5.1E-02
Mtr.3204.1.S1_at	3.03	3.64	3.31	3.28	3.33	4.50	3.33	0.31	3.70	0.69	1.11	0.154	4.4E-01
Mtr.47205.1.S1_at	3.33	3.84	3.30	3.27	3.12	3.23	3.49	0.31	3.21	0.08	0.92	-0.122	2.0E-01
Mtr.11616.1.S1_at	9.82	10.42	10.19	9.96	10.26	9.93	10.14	0.31	10.05	0.18	0.99	-0.013	6.8E-01
Mtr.37537.1.S1_s_at	12.06	11.45	11.78	12.63	12.96	12.88	11.76	0.31	12.82	0.17	1.09	0.124	6.4E-03
Mtr.38647.1.S1_at	9.31	9.84	9.31	9.21	9.28	9.32	9.48	0.31	9.27	0.06	0.98	-0.033	3.0E-01
Mtr.30799.1.S1_at	3.09	2.75	3.36	3.04	4.01	3.32	3.07	0.31	3.46	0.50	1.13	0.173	3.1E-01
Mtr.42817.1.S1_at	10.65	10.10	10.59	10.64	10.55	10.66	10.45	0.31	10.62	0.05	1.02	0.023	4.0E-01
Mtr.49566.1.S1_at	2.86	3.36	2.81	2.79	2.76	2.74	3.01	0.31	2.76	0.03	0.92	-0.125	2.3E-01
Mtr.12878.1.S1_at	14.63	15.12	15.20	14.95	14.94	14.97	14.98	0.31	14.95	0.02	1.00	-0.003	8.7E-01
Mtr.43979.1.S1_at	8.98	8.38	8.57	8.76	8.68	8.85	8.64	0.31	8.76	0.08	1.01	0.020	5.4E-01
Mtr.19829.1.S1_at	9.03	8.90	9.48	9.00	9.17	9.10	9.14	0.31	9.09	0.09	0.99	-0.007	8.1E-01
Mtr.41498.1.S1_at	9.65	10.18	10.17	9.57	9.91	9.69	10.00	0.31	9.72	0.17	0.97	-0.041	2.4E-01
Mtr.46956.1.S1_at	11.59	12.04	12.17	11.78	11.94	11.62	11.93	0.31	11.78	0.16	0.99	-0.018	5.0E-01
Mtr.17803.1.S1_at	6.92	7.50	7.36	6.88	7.83	7.23	7.26	0.31	7.31	0.48	1.01	0.011	8.7E-01
Mtr.25489.1.S1_at	2.98	3.48	2.92	2.90	2.89	3.00	3.13	0.31	2.93	0.06	0.94	-0.093	3.4E-01
Mtr.43456.1.S1_at	11.14	10.67	11.24	11.16	11.18	11.07	11.02	0.31	11.13	0.06	1.01	0.016	5.4E-01
Mtr.45656.1.S1_at	9.33	8.90	8.74	9.23	9.08	9.15	8.99	0.31	9.15	0.08	1.02	0.026	4.2E-01
Mtr.43236.1.S1_at	12.46	11.88	12.33	12.67	12.71	12.71	12.22	0.31	12.70	0.02	1.04	0.055	5.5E-02
Mtr.12378.1.S1_at	12.11	11.77	11.50	11.86	11.99	11.78	11.79	0.31	11.88	0.10	1.01	0.010	6.7E-01
Mtr.20900.1.S1_at	11.50	12.00	12.05	11.88	12.17	12.23	11.85	0.31	12.09	0.19	1.02	0.029	3.0E-01
Mtr.9470.1.S1_at	9.58	8.97	9.22	9.36	9.49	9.58	9.26	0.31	9.48	0.11	1.02	0.034	3.1E-01
Mtr.37293.1.S1_x_at	10.99	10.50	10.44	11.39	11.42	11.33	10.64	0.31	11.38	0.05	1.07	0.097	1.4E-02
Mtr.26508.1.S1_at	5.05	4.64	4.45	4.58	4.93	4.82	4.71	0.31	4.78	0.18	1.01	0.019	7.7E-01
Mtr.51880.1.S1_at	2.66	3.17	2.62	2.61	3.02	2.56	2.82	0.31	2.73	0.25	0.97	-0.045	7.3E-01
Mtr.7288.1.S1_at	9.55	8.99	9.48	9.45	9.46	9.42	9.34	0.30	9.44	0.02	1.01	0.016	5.9E-01
Mtr.39069.1.S1_at	11.55	10.98	11.43	11.10	10.90	10.99	11.32	0.30	11.00	0.10	0.97	-0.042	1.6E-01
Mtr.9136.1.S1_at	9.90	10.15	10.51	10.15	10.04	10.06	10.18	0.30	10.08	0.06	0.99	-0.015	6.0E-01
Mtr.40868.1.S1_at	14.55	15.15	14.75	14.76	14.85	14.77	14.82	0.30	14.79	0.05	1.00	-0.003	8.9E-01
Mtr.3849.1.S1_at	5.83	5.33	5.27	5.09	5.08	5.03	5.48	0.30	5.07	0.03	0.93	-0.112	8.2E-02
Mtr.52188.1.S1_at	4.82	4.77	5.32	5.28	5.40	5.97	4.97	0.30	5.55	0.37	1.12	0.159	1.0E-01
Mtr.25195.1.S1_x_at	5.71	5.10	5.36	5.79	5.93	5.85	5.39	0.30	5.86	0.07	1.09	0.120	6.1E-02
Mtr.48678.1.S1_at	2.09	2.61	2.08	2.08	2.08	2.07	2.26	0.30	2.08	0.00	0.92	-0.125	3.5E-01
Mtr.42184.1.S1_at	7.98	7.72	8.33	8.35	8.27	8.38	8.01	0.30	8.33	0.06	1.04	0.058	1.4E-01
Mtr.8490.1.S1_at	10.24	10.71	10.13	10.39	10.17	10.32	10.36	0.30	10.29	0.11	0.99	-0.009	7.4E-01
Mtr.29934.1.S1_at	2.42	2.92	2.37	2.34	2.32	2.31	2.57	0.30	2.32	0.02	0.90	-0.144	2.4E-01
Mtr.9183.1.S1_at	9.04	8.44	8.68	8.85	9.14	8.83	8.72	0.30	8.94	0.17	1.03	0.036	3.4E-01
Mtr.6935.1.S1_at	8.89	8.29	8.54	8.84	8.86	8.94	8.57	0.30	8.88	0.06	1.04	0.051	1.6E-01
Mtr.27535.1.S1_at	3.16	3.69	3.17	3.13	3.04	3.13	3.34	0.30	3.10	0.05	0.93	-0.108	2.5E-01
Mtr.30930.1.S1_at	10.66	10.06	10.44	10.33	10.42	10.32	10.39	0.30	10.36	0.05	1.00	-0.004	8.8E-01
Mtr.44845.1.S1_at	10.30	10.19	10.76	10.36	10.25	10.23	10.42	0.30	10.28	0.07	0.99	-0.019	4.9E-01
Mtr.21994.1.S1_at	2.87	3.41	2.89	2.86	2.87	2.79	3.06	0.30	2.84	0.04	0.93	-0.104	3.0E-01
Mtr.43334.1.S1_at	9.52	8.94	9.38	8.76	8.99	8.79	9.28	0.30	8.85	0.12	0.95	-0.070	8.3E-02
Mtr.46118.1.S1_at	10.63	10.83	11.23	10.78	10.75	10.76	10.90	0.30	10.77	0.02	0.99	-0.017	5.0E-01
Mtr.38371.1.S1_at	8.90	8.94	8.39	8.94	8.86	9.16	8.74	0.30	8.98	0.16	1.03	0.039	2.9E-01
Mtr.19671.1.S1_at	11.39	10.95	11.53	11.40	11.34	11.47	11.29	0.30	11.40	0.06	1.01	0.014	5.6E-01
Mtr.37543.1.S1_at	10.43	9.86	10.33	10.32	10.26	10.27	10.21	0.30	10.28	0.03	1.01	0.011	6.9E-01
Mtr.47174.1.S1_at	10.63	10.59	11.14	10.87	9.42	10.80	10.79	0.30	10.36	0.82	0.96	-0.058	4.4E-01
Mtr.48022.1.S1_at	2.74	3.26	2.73	2.71	2.67	2.67	2.91	0.30	2.69	0.02	0.92	-0.115	2.7E-01
Mtr.20618.1.S1_at	10.63	10.95	10.34	10.36	10.62	10.74	10.64	0.30	10.57	0.20	0.99	-0.009	7.6E-01
Mtr.46200.1.S1_at	10.03	10.64	10.37	12.20	12.91	12.64	10.35	0.30	12.58	0.36	1.22	0.282	1.2E-03
Mtr.39451.1.S1_at	7.32	7.91	7.73	8.33	8.58	8.57	7.65	0.30	8.49	0.14	1.11	0.150	1.2E-02
Mtr.46965.1.S1_at	2.66	3.18	2.66	2.64	2.70	2.58	2.83	0.30	2.64	0.06	0.93	-0.103	3.4E-01
Mtr.600.1.S1_at	2.76	3.27	2.72	2.70	2.67	2.65	2.92	0.30	2.67	0.02	0.92	-0.125	2.4E-01
Mtr.37659.1.S1_at	12.62	12.30	12.01	12.24	12.38	12.30	12.31	0.30	12.31	0.07	1.00	0.000	9.9E-01
Mtr.43020.1.S1_at	12.51	12.93	13.10	12.90	13.08	12.94	12.84	0.30	12.97	0.09	1.01	0.014	5.2E-01
Mtr.47171.1.S1_at	2.16	2.68	2.15	2.14	2.15	2.14	2.33	0.30	2.14	0.00	0.92	-0.120	3.5E-01
Mtr.8837.1.S1_at	6.46	6.06	6.66	6.42	5.87	6.63	6.39	0.30	6.31	0.40	0.99	-0.020	7.7E-01
Mtr.2123.1.S1_at	3.20	2.77	2.61	2.60	2.59	2.58	2.86	0.30	2.59	0.01	0.91	-0.141	2.0E-01
Mtr.24654.1.S1_at	2.74	3.26	2.73	2.69	2.69	2.73	2.91	0.30	2.70	0.02	0.93	-0.105	3.1E-01
Mtr.11473.1.S1_at	10.70	10.28	10.87	10.56	10.66	10.56	10.62	0.30	10.59	0.06	1.00	-0.004	8.8E-01
Mtr.10427.1.S1_at	12.50	12.97	12.40	12.85	13.12	12.85	12.62	0.30	12.94	0.15	1.02	0.036	1.8E-01
Mtr.40211.1.S1_at	10.00	9.88	10.45	10.40	10.55	10.33	10.11	0.30	10.42	0.11	1.03	0.044	1.7E-01
Mtr.43170.1.S1_s_at	10.37	10.94	10.84	10.63	10.98	10.80	10.71	0.30	10.80	0.18	1.01	0.012	6.8E-01
Mtr.16826.1.S1_at	2.55	3.07	2.54	2.54	2.50	2.53	2.72	0.30	2.52	0.02	0.93	-0.109	3.2E-01
Mtr.49296.1.S1_at	9.96	9.37	9.81	9.65	9.65	9.66	9.71	0.30	9.66	0.01	0.99	-0.009	7.5E-01
Mtr.44461.1.S1_at	12.43	11.96	12.54	12.19	12.45	12.09	12.31	0.30	12.25	0.18	0.99	-0.007	7.7E-01
Mtr.9997.1.S1_at	9.70	10.24	10.20	9.97	10.07	9.57	10.05	0.30	9.87	0.26	0.98	-0.026	4.8E-01
Mtr.32651.1.S1_at	8.69	8.08	8.36	8.67	8.48	8.55	8.38	0.30	8.57	0.09	1.02	0.032	3.7E-01
Mtr.9050.1.S1_at	11.98	11.38	11.74	11.05	10.74	11.17	11.70	0.30	10.99	0.22	0.94	-0.091	3.0E-02
Mtr.39373.1.S1_at	8.39	7.94	8.52	8.50	8.31	8.36	8.28	0.30	8.39	0.10	1.01	0.019	5.9E-01
Mtr.49423.1.S1_at	2.38	2.88	2.33	2.33	2.31	2.35	2.53	0.30	2.33	0.02	0.92	-0.120	3.2E-01
Mtr.6928.1.S1_s_at	9.84	9.45	9.25	9.87	9.80	9.98	9.51	0.30	9.88	0.09	1.04	0.055	1.1E-01
Mtr.51458.1.S1_at	13.28	12.68	12.88	13.06	13.02	13.00	12.95	0.30	13.02	0.03	1.01	0.009	6.7E-01
Mtr.30410.1.S1_at	9.12	9.69	9.59	9.41	9.61	9.49	9.46	0.30	9.50	0.10	1.00	0.006	8.4E-01
Mtr.41395.1.S1_at	10.65	10.31	10.04	10.49	10.63	10.49	10.34	0.30	10.53	0.08	1.02	0.028	3.3E-01
Mtr.38255.1.S1_at	8.55	8.48	9.03	8.43	8.52	8.44	8.69	0.30	8.46	0.05	0.97	-0.038	2.8E-01
Mtr.19092.1.S1_at	2.79	3.31	2.76	2.74	2.75	2.71	2.96	0.30	2.73	0.02	0.92	-0.113	2.8E-01
Mtr.42440.1.S1_at	9.89	9.28	9.61	9.77	9.80	9.81	9.59	0.30	9.79	0.02	1.02	0.030	3.2E-01
Mtr.39869.1.S1_at	8.96	8.41	8.91	8.80	8.96	8.99	8.76	0.30	8.92	0.10	1.02	0.026	4.4E-01
Mtr.2594.1.S1_at	8.17	8.05	8.63	8.64	8.37	8.42	8.28	0.30	8.47	0.14	1.02	0.033	3.8E-01
Mtr.40741.1.S1_at	12.33	11.75	12.18	12.09	12.02	12.19	12.09	0.30	12.10	0.08	1.00	0.001	9.5E-01
Mtr.38921.1.S1_s_at	8.97	8.37	8.79	8.85	8.60	8.95	8.71	0.30	8.80	0.18	1.01	0.015	6.9E-01
Mtr.45701.1.S1_at	12.32	11.73	12.13	12.28	12.21	12.24	12.06	0.30	12.24	0.03	1.01	0.021	3.7E-01
Mtr.16440.1.S1_at	2.92	3.42	2.88	2.86	2.87	2.88	3.07	0.30	2.87	0.01	0.93	-0.099	3.1E-01
Mtr.37831.1.S1_at	4.42	4.36	3.86	4.58	4.71	4.77	4.21	0.30	4.69	0.10	1.11	0.154	6.1E-02
Mtr.20704.1.S1_at	9.69	9.49	10.09	9.87	10.01	9.81	9.76	0.30	9.90	0.10	1.01	0.021	4.9E-01
Mtr.43657.1.S1_at	11.77	11.17	11.41	11.61	11.41	11.64	11.45	0.30	11.55	0.13	1.01	0.013	6.1E-01
Mtr.34874.1.S1_s_at	9.44	8.99	9.57	9.53	9.55	9.48	9.33	0.30	9.52	0.04	1.02	0.028	3.6E-01
Mtr.1256.1.S1_x_at	2.54	2.04	1.99	1.98	1.98	1.98	2.19	0.30	1.98	0.00	0.90	-0.144	3.0E-01
Mtr.12017.1.S1_at	9.18	9.49	8.88	9.22	9.17	9.16	9.18	0.30	9.18	0.03	1.00	0.000	1.0E+00
Mtr.48719.1.S1_at	13.60	13.27	12.99	13.30	13.47	13.43	13.29	0.30	13.40	0.09	1.01	0.012	5.7E-01
Mtr.47690.1.S1_x_at	2.51	2.66	3.09	4.25	3.27	4.57	2.75	0.30	4.03	0.68	1.47	0.551	4.0E-02
Mtr.5101.1.S1_at	2.81	3.29	2.73	2.85	2.85	2.95	2.95	0.30	2.88	0.06	0.98	-0.030	7.5E-01
Mtr.39614.1.S1_at	10.79	10.97	11.38	10.87	10.73	10.66	11.05	0.30	10.75	0.11	0.97	-0.039	1.8E-01
Mtr.27338.1.S1_at	8.72	8.20	8.18	8.60	8.78	8.45	8.37	0.30	8.61	0.16	1.03	0.042	2.9E-01
Mtr.8692.1.S1_at	10.81	10.21	10.43	10.48	10.35	10.47	10.48	0.30	10.43	0.07	1.00	-0.006	8.1E-01
Mtr.47375.1.S1_at	5.43	4.99	4.84	5.19	5.08	5.07	5.09	0.30	5.11	0.06	1.01	0.007	8.9E-01
Mtr.47461.1.S1_at	6.73	7.05	6.44	4.96	6.65	6.52	6.74	0.30	6.05	0.94	0.90	-0.157	2.9E-01
Mtr.32128.1.S1_at	11.62	12.15	12.14	11.54	11.80	11.69	11.97	0.30	11.68	0.13	0.98	-0.036	2.0E-01
Mtr.27467.1.S1_at	2.79	3.29	2.74	2.72	2.68	2.65	2.94	0.30	2.69	0.04	0.91	-0.131	2.2E-01
Mtr.12227.1.S1_s_at	9.51	9.08	8.92	9.17	8.92	8.89	9.17	0.30	8.99	0.16	0.98	-0.029	4.1E-01
Mtr.36834.1.S1_at	3.47	3.96	3.40	3.88	3.76	3.72	3.61	0.30	3.78	0.08	1.05	0.068	3.9E-01
Mtr.22759.1.S1_x_at	3.99	4.50	3.97	3.93	3.96	3.95	4.15	0.30	3.95	0.01	0.95	-0.072	3.1E-01
Mtr.40879.1.S1_at	11.02	10.50	11.03	10.98	11.04	10.95	10.85	0.30	10.99	0.04	1.01	0.019	4.7E-01
Mtr.4383.1.S1_at	2.69	2.72	3.23	2.67	2.67	2.72	2.88	0.30	2.69	0.03	0.93	-0.102	3.3E-01
Mtr.13115.1.S1_at	11.94	11.38	11.86	11.81	11.64	11.76	11.73	0.30	11.74	0.09	1.00	0.001	9.6E-01
Mtr.38586.1.S1_at	7.83	7.75	8.31	7.95	8.02	8.09	7.96	0.30	8.02	0.07	1.01	0.010	7.8E-01
Mtr.20547.1.S1_at	9.03	8.49	9.01	8.89	8.93	8.73	8.84	0.30	8.85	0.11	1.00	0.001	9.7E-01
Mtr.48837.1.S1_s_at	11.20	10.60	10.99	11.11	11.21	11.21	10.93	0.30	11.18	0.06	1.02	0.032	2.3E-01
Mtr.34044.1.S1_at	2.51	3.04	2.51	2.48	2.47	2.46	2.69	0.30	2.47	0.01	0.92	-0.120	2.9E-01
Mtr.4687.1.S1_at	2.48	2.99	2.46	2.47	2.45	2.44	2.64	0.30	2.45	0.02	0.93	-0.110	3.3E-01
Mtr.39921.1.S1_at	8.94	8.40	8.92	8.89	8.96	8.75	8.75	0.30	8.87	0.11	1.01	0.018	5.8E-01
Mtr.48330.1.S1_at	2.91	3.42	2.87	2.89	2.89	2.81	3.07	0.30	2.87	0.05	0.93	-0.097	3.2E-01
Mtr.10663.1.S1_at	11.27	10.88	11.48	11.86	11.74	11.75	11.21	0.30	11.78	0.07	1.05	0.072	3.3E-02
Mtr.11264.1.S1_at	8.27	8.76	8.82	8.79	9.07	9.04	8.61	0.30	8.97	0.15	1.04	0.058	1.4E-01
Mtr.40254.1.S1_s_at	13.15	12.57	13.03	12.98	13.04	13.12	12.92	0.30	13.05	0.07	1.01	0.015	5.1E-01
Mtr.13841.1.S1_at	2.39	2.53	2.97	2.63	2.57	2.47	2.63	0.30	2.56	0.08	0.97	-0.038	7.2E-01

Mtr.23331.1.S1_at	8.89	8.65	9.25	8.50	8.99	8.87	8.93	0.30	8.79	0.25	0.98	-0.023	5.6E-01
Mtr.6025.1.S1_at	2.29	2.88	2.71	2.44	2.70	2.70	2.62	0.30	2.61	0.15	1.00	-0.005	9.6E-01
Mtr.45777.1.S1_at	4.88	4.44	4.29	4.27	4.11	4.27	4.54	0.30	4.22	0.09	0.93	-0.105	1.6E-01
Mtr.22795.1.S1_at	2.98	3.50	2.97	2.97	2.92	2.97	3.15	0.30	2.95	0.03	0.94	-0.094	3.2E-01
Mtr.2196.1.S1_x_at	7.25	7.20	6.70	6.85	6.99	6.84	7.05	0.30	6.89	0.08	0.98	-0.033	4.3E-01
Mtr.39007.1.S1_at	10.14	9.69	10.27	10.23	10.19	10.11	10.04	0.30	10.18	0.06	1.01	0.020	4.7E-01
Mtr.48289.1.S1_at	2.79	2.71	3.27	2.78	2.78	2.80	2.92	0.30	2.79	0.01	0.95	-0.069	4.8E-01
Mtr.43601.1.S1_at	6.64	6.68	7.19	7.19	7.06	7.07	6.84	0.30	7.10	0.07	1.04	0.055	2.1E-01
Mtr.31799.1.S1_at	8.31	8.90	8.74	8.65	8.75	8.87	8.65	0.30	8.75	0.11	1.01	0.018	6.0E-01
Mtr.47437.1.S1_at	5.23	4.65	5.10	3.47	4.55	4.64	4.99	0.30	4.22	0.65	0.84	-0.243	1.4E-01
Mtr.22702.1.S1_x_at	3.29	3.80	3.26	3.27	3.32	3.26	3.45	0.30	3.28	0.04	0.95	-0.072	3.9E-01
Mtr.11551.1.S1_at	8.43	8.31	8.89	8.54	8.84	8.51	8.54	0.30	8.63	0.19	1.01	0.015	6.9E-01
Mtr.2482.1.S1_at	8.69	8.10	8.53	8.37	8.29	8.39	8.44	0.30	8.35	0.05	0.99	-0.016	6.4E-01
Mtr.13366.1.S1_at	9.76	9.26	9.22	9.24	9.34	9.31	9.41	0.30	9.30	0.05	0.99	-0.018	5.5E-01
Mtr.33244.1.S1_at	6.74	6.13	6.41	6.30	5.77	6.10	6.43	0.30	6.05	0.27	0.94	-0.086	1.8E-01
Mtr.4.1.S1_s_at	10.95	10.35	10.59	10.82	10.83	10.96	10.63	0.30	10.87	0.08	1.02	0.032	2.6E-01
Mtr.39277.1.S1_at	3.41	2.93	2.86	5.57	6.05	5.92	3.07	0.30	5.84	0.25	1.91	0.930	2.5E-04
Mtr.32362.1.S1_at	6.36	6.53	5.94	6.34	6.42	6.35	6.28	0.30	6.37	0.04	1.01	0.021	6.3E-01
Mtr.20126.1.S1_at	7.78	7.18	7.55	7.67	7.50	7.49	7.50	0.30	7.55	0.10	1.01	0.009	8.1E-01
Mtr.50946.1.S1_s_at	6.83	6.86	7.37	7.05	7.16	7.03	7.02	0.30	7.08	0.07	1.01	0.011	7.7E-01
Mtr.10304.1.S1_at	13.94	14.23	14.55	14.29	14.24	14.16	14.24	0.30	14.23	0.06	1.00	-0.001	9.5E-01
Mtr.25232.1.S1_at	2.43	2.94	2.41	2.41	2.40	2.38	2.59	0.30	2.40	0.02	0.92	-0.115	3.2E-01
Mtr.9908.1.S1_at	6.83	6.65	7.24	7.18	7.58	7.40	6.91	0.30	7.39	0.20	1.07	0.097	8.2E-02
Mtr.49041.1.S1_at	9.29	8.93	9.54	9.41	9.43	9.38	9.25	0.30	9.41	0.03	1.02	0.024	4.3E-01
Mtr.37399.1.S1_at	13.54	13.18	12.94	13.01	13.11	13.17	13.22	0.30	13.09	0.08	0.99	-0.014	5.2E-01
Mtr.17051.1.S1_at	2.58	3.09	2.55	2.57	2.51	2.56	2.74	0.30	2.54	0.03	0.93	-0.105	3.3E-01
Mtr.14035.1.S1_s_at	8.44	8.42	8.96	8.90	9.11	8.66	8.61	0.30	8.89	0.22	1.03	0.047	2.6E-01
Mtr.27283.1.S1_at	7.78	7.18	7.43	7.67	7.67	7.84	7.46	0.30	7.73	0.10	1.04	0.051	2.2E-01
Mtr.32496.1.S1_at	3.58	3.59	4.11	4.48	3.59	3.55	3.76	0.30	3.87	0.52	1.03	0.044	7.6E-01
Mtr.15257.1.S1_s_at	12.29	11.99	12.60	12.42	12.57	12.35	12.30	0.30	12.44	0.11	1.01	0.017	4.7E-01
Mtr.37422.1.S1_at	10.95	10.38	10.48	10.98	11.25	10.98	10.61	0.30	11.07	0.16	1.04	0.062	7.8E-02
Mtr.51204.1.S1_at	10.16	9.58	10.01	9.95	9.85	9.84	9.92	0.30	9.88	0.06	1.00	-0.005	8.6E-01
Mtr.12848.1.S1_at	12.05	12.57	12.05	11.54	12.10	11.59	12.22	0.30	11.74	0.31	0.96	-0.058	1.3E-01
Mtr.13342.1.S1_at	9.75	9.15	9.44	9.83	9.60	9.51	9.45	0.30	9.65	0.16	1.02	0.030	3.7E-01
Mtr.975.1.S1_at	8.69	8.65	8.15	8.07	8.58	8.39	8.49	0.30	8.35	0.26	0.98	-0.025	5.6E-01
Mtr.20768.1.S1_at	9.07	8.48	8.92	9.02	8.99	9.05	8.82	0.30	9.02	0.03	1.02	0.032	3.2E-01
Mtr.11496.1.S1_at	8.11	7.59	8.11	8.30	8.26	8.22	7.94	0.30	8.26	0.04	1.04	0.057	1.4E-01
Mtr.41911.1.S1_s_at	10.29	9.73	10.22	10.20	10.40	10.41	10.08	0.30	10.34	0.12	1.03	0.036	2.4E-01
Mtr.38018.1.S1_at	10.60	10.17	10.01	9.90	9.51	9.59	10.26	0.30	9.67	0.20	0.94	-0.086	4.8E-02
Mtr.50841.1.S1_at	3.56	3.27	3.88	3.23	3.81	4.11	3.57	0.30	3.72	0.45	1.04	0.059	6.6E-01
Mtr.6066.1.S1_x_at	8.27	7.68	8.07	8.10	8.11	8.14	8.01	0.30	8.12	0.02	1.01	0.019	5.7E-01
Mtr.42595.1.S1_at	13.49	13.96	14.05	13.44	13.04	13.34	13.83	0.30	13.27	0.21	0.96	-0.059	5.7E-02
Mtr.14017.1.S1_at	2.19	2.73	2.23	2.27	2.18	2.18	2.38	0.30	2.21	0.05	0.93	-0.108	3.9E-01
Mtr.2939.1.S1_at	6.56	6.58	7.09	6.38	6.65	6.53	6.74	0.30	6.52	0.13	0.97	-0.048	3.1E-01
Mtr.29048.1.S1_at	6.46	6.04	6.63	6.58	6.67	6.82	6.37	0.30	6.69	0.12	1.05	0.070	1.7E-01
Mtr.43530.1.S1_at	11.83	11.23	11.45	11.64	11.65	11.70	11.50	0.30	11.66	0.03	1.01	0.020	4.1E-01
Mtr.35317.1.S1_at	8.45	8.00	7.88	8.01	7.76	8.03	8.11	0.30	7.94	0.15	0.98	-0.031	4.2E-01
Mtr.33100.1.S1_at	6.68	7.28	7.00	7.17	7.18	7.16	6.99	0.30	7.17	0.01	1.03	0.038	3.5E-01
Mtr.42924.1.S1_at	3.00	3.52	2.99	2.96	2.95	2.96	3.17	0.30	2.96	0.00	0.93	-0.100	2.9E-01
Mtr.44841.1.S1_at	8.65	8.26	8.85	8.81	9.34	8.82	8.59	0.30	8.99	0.31	1.05	0.066	1.8E-01
Mtr.51122.1.S1_at	2.89	3.33	2.76	2.71	2.80	2.72	2.99	0.30	2.74	0.05	0.92	-0.125	2.3E-01
Mtr.7070.1.S1_at	9.06	8.62	9.19	9.20	9.21	9.30	8.96	0.30	9.23	0.06	1.03	0.044	1.9E-01
Mtr.11708.1.S1_s_at	9.26	9.55	9.86	9.31	9.38	9.32	9.55	0.30	9.34	0.04	0.98	-0.033	2.8E-01
Mtr.10703.1.S1_at	6.50	5.90	6.19	6.36	6.19	6.52	6.20	0.30	6.36	0.17	1.03	0.037	4.7E-01
Mtr.39321.1.S1_at	9.30	9.26	8.76	9.80	9.35	9.45	9.10	0.30	9.53	0.23	1.05	0.067	1.2E-01
Mtr.38621.1.S1_at	9.26	9.43	9.84	9.39	9.41	9.23	9.51	0.30	9.34	0.10	0.98	-0.026	4.1E-01
Mtr.38964.1.S1_at	8.11	7.51	7.80	8.06	8.08	8.05	7.81	0.30	8.06	0.02	1.03	0.047	2.2E-01
Mtr.33307.1.S1_at	8.37	7.85	8.38	8.28	8.25	8.23	8.20	0.30	8.25	0.02	1.01	0.009	7.8E-01
Mtr.42342.1.S1_at	3.93	3.40	3.92	3.92	3.92	3.92	3.75	0.30	3.92	0.00	1.04	0.063	3.9E-01
Mtr.20497.1.S1_s_at	9.52	10.01	10.08	9.89	9.80	9.87	9.87	0.30	9.85	0.05	1.00	-0.003	9.2E-01
Mtr.26937.1.S1_at	3.12	3.64	3.11	3.09	3.11	3.11	3.29	0.30	3.10	0.01	0.94	-0.084	3.4E-01
Mtr.30539.1.S1_at	2.87	3.38	2.84	2.82	2.83	2.78	3.03	0.30	2.81	0.03	0.93	-0.108	2.8E-01
Mtr.35339.1.S1_x_at	9.02	8.42	8.72	8.34	8.28	8.30	8.72	0.30	8.31	0.03	0.95	-0.070	7.8E-02
Mtr.3513.1.S1_at	11.05	11.61	11.51	11.06	11.19	10.99	11.39	0.30	11.08	0.10	0.97	-0.040	1.7E-01
Mtr.50816.1.S1_s_at	10.21	10.24	10.75	10.30	10.30	10.44	10.40	0.30	10.35	0.08	0.99	-0.007	7.8E-01
Mtr.35489.1.S1_at	2.69	3.20	2.66	2.64	2.59	2.57	2.85	0.30	2.60	0.03	0.91	-0.132	2.3E-01
Mtr.15482.1.S1_at	8.35	8.09	8.69	8.47	8.37	8.30	8.38	0.30	8.38	0.09	1.00	0.000	9.9E-01
Mtr.47207.1.S1_at	3.34	3.12	3.72	3.26	3.29	3.30	3.39	0.30	3.28	0.02	0.97	-0.047	5.6E-01
Mtr.10170.1.S1_at	6.12	5.58	6.08	5.28	5.45	5.22	5.93	0.30	5.32	0.12	0.90	-0.157	3.1E-02
Mtr.19128.1.S1_at	2.52	3.03	2.49	2.44	2.44	2.61	2.68	0.30	2.50	0.10	0.93	-0.101	3.8E-01
Mtr.12387.1.S1_at	9.64	9.07	9.51	9.44	9.44	9.69	9.40	0.30	9.52	0.14	1.01	0.018	5.7E-01
Mtr.9650.1.S1_at	9.30	9.22	9.78	9.46	9.75	9.56	9.43	0.30	9.59	0.15	1.02	0.023	4.7E-01
Mtr.35900.1.S1_at	9.13	8.53	8.86	8.80	9.20	8.83	8.84	0.30	8.94	0.22	1.01	0.017	6.6E-01
Mtr.8667.1.S1_at	7.30	7.68	7.09	7.54	8.09	7.04	7.36	0.30	7.55	0.52	1.03	0.038	6.0E-01
Mtr.27762.1.S1_at	4.60	5.12	4.60	4.92	4.74	4.72	4.77	0.30	4.79	0.11	1.00	0.006	9.2E-01
Mtr.7118.1.S1_at	9.56	9.47	10.03	9.67	9.56	9.54	9.69	0.30	9.59	0.07	0.99	-0.015	6.2E-01
Mtr.41121.1.S1_at	10.48	11.06	10.93	10.69	10.83	10.55	10.82	0.30	10.69	0.14	0.99	-0.018	5.2E-01
Mtr.39565.1.S1_at	11.57	10.97	11.32	11.41	11.45	11.33	11.28	0.30	11.40	0.06	1.01	0.014	5.6E-01
Mtr.34852.1.S1_at	2.97	3.49	2.97	3.00	2.89	2.86	3.15	0.30	2.92	0.07	0.93	-0.108	2.7E-01
Mtr.10420.1.S1_a_at	11.82	11.25	11.70	11.59	11.63	11.67	11.59	0.30	11.63	0.04	1.00	0.005	8.4E-01
Mtr.31256.1.S1_at	10.83	10.63	11.22	10.78	10.71	10.67	10.89	0.30	10.72	0.06	0.98	-0.023	3.8E-01
Mtr.26222.1.S1_at	10.19	9.88	10.49	10.14	10.11	10.23	10.19	0.30	10.16	0.06	1.00	-0.004	8.9E-01
Mtr.12683.1.S1_at	5.58	5.50	5.02	5.58	6.67	6.26	5.37	0.30	6.17	0.55	1.15	0.201	9.0E-02
Mtr.40682.1.S1_at	12.71	12.13	12.57	12.32	12.19	12.22	12.47	0.30	12.24	0.07	0.98	-0.027	2.7E-01
Mtr.9675.1.S1_at	10.02	9.60	10.18	10.10	10.09	9.97	9.93	0.30	10.06	0.07	1.01	0.018	5.2E-01
Mtr.7451.1.S1_at	10.27	9.92	9.67	9.62	9.83	9.89	9.96	0.30	9.78	0.14	0.98	-0.025	4.2E-01
Mtr.18541.1.S1_at	2.64	3.14	2.60	2.59	2.57	2.58	2.80	0.30	2.58	0.01	0.92	-0.117	2.8E-01
Mtr.33808.1.S1_at	10.73	11.18	10.61	10.07	10.48	10.22	10.84	0.30	10.26	0.21	0.95	-0.080	5.0E-02
Mtr.11634.1.S1_at	10.07	9.67	9.48	9.89	9.51	9.70	9.74	0.30	9.70	0.19	1.00	-0.006	8.5E-01
Mtr.12082.1.S1_at	8.38	8.20	7.79	8.42	8.37	8.56	8.12	0.30	8.45	0.10	1.04	0.057	1.5E-01
Mtr.4035.1.S1_at	10.54	10.69	11.12	10.87	11.11	10.69	10.78	0.30	10.89	0.21	1.01	0.014	6.5E-01
Mtr.21836.1.S1_s_at	3.14	3.61	3.05	3.03	2.98	2.97	3.27	0.30	3.00	0.03	0.92	-0.127	1.9E-01
Mtr.4547.1.S1_at	8.05	7.45	7.73	7.12	7.27	7.30	7.74	0.30	7.23	0.09	0.93	-0.099	4.8E-02
Mtr.1938.1.S1_at	5.79	6.23	6.37	8.10	8.16	7.52	6.13	0.30	7.93	0.36	1.29	0.371	2.6E-03
Mtr.32933.1.S1_at	9.41	8.96	9.53	9.42	9.66	9.53	9.30	0.30	9.54	0.12	1.03	0.036	2.8E-01
Mtr.21711.1.S1_s_at	8.26	7.77	8.32	8.39	8.88	8.89	8.12	0.30	8.72	0.28	1.07	0.103	6.5E-02
Mtr.40428.1.S1_at	11.35	10.76	11.15	11.09	11.45	11.10	11.08	0.30	11.21	0.21	1.01	0.016	5.8E-01
Mtr.5966.1.S1_at	7.87	8.41	7.92	7.81	7.52	7.93	8.07	0.30	7.75	0.21	0.96	-0.058	2.1E-01
Mtr.50336.1.S1_s_at	3.66	3.23	3.09	3.17	2.65	3.25	3.33	0.30	3.02	0.33	0.91	-0.138	3.0E-01
Mtr.7585.1.S1_at	3.30	3.15	2.72	2.72	2.70	2.68	3.06	0.30	2.70	0.02	0.88	-0.179	1.1E-01
Mtr.48954.1.S1_at	9.92	9.32	9.59	9.57	9.64	9.76	9.61	0.30	9.66	0.10	1.01	0.007	8.0E-01
Mtr.43309.1.S1_at	10.67	10.28	10.87	10.68	10.54	10.63	10.61	0.30	10.62	0.07	1.00	0.001	9.6E-01
Mtr.5398.1.S1_at	2.75	3.26	2.73	2.71	2.70	2.67	2.91	0.30	2.70	0.02	0.93	-0.112	2.8E-01
Mtr.40811.1.S1_at	12.59	12.79	13.18	12.99	13.06	12.99	12.85	0.30	13.01	0.04	1.01	0.018	4.2E-01
Mtr.13966.1.S1_at	8.78	8.33	8.91	8.83	9.08	8.93	8.67	0.30	8.95	0.12	1.03	0.045	2.1E-01
Mtr.34729.1.S1_at	7.69	7.10	7.50	7.43	7.61	7.31	7.43	0.30	7.45	0.15	1.00	0.005	9.1E-01
Mtr.37635.1.S1_at	11.02	10.48	10.52	10.52	10.50	10.82	10.67	0.30	10.61	0.18	0.99	-0.008	7.8E-01
Mtr.15277.1.S1_at	3.19	3.68	3.14	3.11	3.07	3.07	3.34	0.30	3.08	0.02	0.92	-0.114	2.2E-01
Mtr.27747.1.S1_at	8.28	7.75	8.27	8.65	8.65	8.31	8.10	0.30	8.54	0.20	1.05	0.077	1.0E-01
Mtr.553.1.S1_at	7.86	7.69	8.27	7.80	7.81	7.96	7.94	0.30	7.86	0.09	0.99	-0.016	6.6E-01
Mtr.2893.1.S1_at	7.13	6.60	7.10	7.60	7.44	7.68	6.94	0.30	7.57	0.12	1.09	0.125	2.9E-02
Mtr.14456.1.S1_at	7.39	7.65	7.99	7.43	7.55	7.10	7.68	0.30	7.36	0.23	0.96	-0.061	2.2E-01
Mtr.34150.1.S1_at	3.31	3.77	3.20	3.31	3.30	3.30	3.43	0.30	3.30	0.00	0.96	-0.053	5.2E-01
Mtr.11343.1.S1_at	3.03	3.12	2.56	2.90	2.89	3.38	2.90	0.30	3.06	0.28	1.05	0.075	5.5E-01
Mtr.12156.1.S1_s_at	11.51	10.93	11.07	11.18	11.40	11.18	11.17	0.30	11.25	0.13	1.01	0.011	6.8E-01
Mtr.2184.1.S1_at	8.14	7.54	7.79	7.89	7.90	7.68	7.83	0.30	7.82	0.12	1.00	-0.001	9.9E-01
Mtr.43100.1.S1_at	8.13	7.53	7.80	8.06	8.21	8.03	7.82	0.30	8.10	0.10	1.04	0.051	2.0E-01
Mtr.5717.1.S1_at	7.28	6.90	6.69	6.58	6.65	6.64	6.96	0.30	6.62	0.04	0.95	-0.071	1.3E-01
Mtr.41032.1.S1_at	10.98	10.38	10.69	10.64	10.56	10.74	10.68	0.30	10.65	0.09	1.00	-0.005	8.6E-01
Mtr.24890.1.S1_at	2.85	3.36	2.84	2.82	2.81	2.80	3.02	0.30	2.81	0.01	0.93	-0.104	2.9E-01
Mtr.24618.1.S1_at	3.10	3.51	2.92	2.90	2.88	2.87	3.18	0.30	2.89	0.02	0.91	-0.139	1.7E-01
Mtr.36291.1.S1_at	2.85	2.98	3.42	2.82	2.93	3.01	3.09	0.30	2.92	0.10	0.95	-0.080	4.1E-01
Mtr.32355.1.S1_at	8.94	8.34	8.55	8.69	8.69	8.79	8.61	0.30	8.72	0.06	1.01	0.019	5.6E-01
Mtr.44494.1.S1_at	9.89	9.34	9.83	9.49	9.78	9.65	9.69	0.30	9.64	0.15	1.00	-0.007	8.2E-01
Mtr.44966.1.S1_at	3.44	3.05	2.84	2.99	4.21	3.98	3.11	0.30	3.73	0.65	1.20	0.261	2.1E-01
Mtr.8509.1.S1_at	12.13	11.66	12.22	12.16	11.94	11.88	12.00	0.30	11.99	0.14	1.00	-0.001	9.7E-01
Mtr.21014.1.S1_s_at	5.74	6.19	6.30	6.20	6.20	6.20	6.08	0.30	6.20	0.00	1.02	0.028	5.2E-01
Mtr.8412.1.S1_s_at	8.57	8.03	8.53	8.66	8.71	8.90	8.38	0.30	8.75	0.13	1.04	0.063	1.2E-01
Mtr.39437.1.S1_at	9.98	9.66	9.38	9.85	9.54	9.73	9.67	0.30	9.71	0.15	1.00	0.005	8.6E-01
Mtr.44401.1.S1_at	9.34	8.74	9.01	9.32	9.19	9.41	9.03	0.30	9.31	0.11	1.03	0.043	2.1E-01
Mtr.23418.1.S1_s_at	13.88	14.29	14.46	13.93	14.04	13.86	14.21	0.30	13.94	0.09	0.98	-0.027	2.1E-01

Mtr.46455.1.S1_at	5.42	5.93	5.96	5.80	5.87	6.08	5.77	0.30	5.92	0.15	1.03	0.036	4.9E-01
Mtr.23801.1.S1_x_at	3.38	3.85	3.29	3.28	3.24	3.26	3.51	0.30	3.26	0.02	0.93	-0.106	2.3E-01
Mtr.294.1.S1_x_at	3.33	3.26	2.78	3.21	3.22	3.20	3.13	0.30	3.21	0.01	1.03	0.039	6.5E-01
Mtr.7149.1.S1_at	8.73	8.45	9.05	9.11	9.41	9.02	8.74	0.30	9.18	0.20	1.05	0.070	1.1E-01
Mtr.6487.1.S1_at	8.50	8.11	8.71	8.58	8.73	8.80	8.44	0.30	8.70	0.11	1.03	0.044	2.3E-01
Mtr.4766.1.S1_at	9.72	9.25	9.80	9.69	9.71	9.69	9.59	0.30	9.70	0.01	1.01	0.016	5.8E-01
Mtr.36781.1.S1_at	10.35	10.07	10.67	10.50	10.52	10.43	10.37	0.30	10.48	0.05	1.01	0.016	5.4E-01
Mtr.35258.1.S1_at	11.40	10.80	11.15	11.27	11.32	11.16	11.12	0.30	11.25	0.08	1.01	0.017	5.1E-01
Mtr.15799.1.S1_at	9.87	9.48	9.28	9.99	10.10	10.15	9.54	0.30	10.08	0.08	1.06	0.080	3.9E-02
Mtr.29334.1.S1_s_at	9.25	8.67	8.81	9.00	9.16	9.32	8.91	0.30	9.16	0.16	1.03	0.040	2.7E-01
Mtr.37601.1.S1_at	12.57	11.97	12.28	12.31	12.36	12.27	12.27	0.30	12.31	0.05	1.00	0.005	8.2E-01
Mtr.40795.1.S1_at	10.25	9.86	10.45	10.37	10.44	10.30	10.19	0.30	10.37	0.07	1.02	0.026	3.6E-01
Mtr.2820.1.S1_at	4.40	3.82	4.24	4.23	4.79	4.09	4.15	0.30	4.37	0.37	1.05	0.073	4.8E-01
Mtr.9657.1.S1_at	10.49	9.90	10.29	10.36	10.44	10.35	10.23	0.30	10.38	0.05	1.02	0.022	4.3E-01
Mtr.45189.1.S1_at	6.76	6.54	7.13	7.15	7.30	7.23	6.81	0.30	7.23	0.07	1.06	0.086	8.0E-02
Mtr.39980.1.S1_at	8.89	8.43	8.33	8.64	8.56	8.26	8.55	0.30	8.48	0.20	0.99	-0.011	7.6E-01
Mtr.30869.1.S1_at	8.78	8.31	8.86	8.65	8.85	8.78	8.65	0.30	8.76	0.10	1.01	0.018	5.8E-01
Mtr.31045.1.S1_at	10.76	10.34	10.92	10.66	10.72	10.59	10.67	0.30	10.66	0.06	1.00	-0.002	9.3E-01
Mtr.6531.1.S1_at	3.07	2.66	2.48	2.46	2.45	2.45	2.74	0.30	2.45	0.01	0.90	-0.160	1.7E-01
Mtr.14109.1.S1_at	10.27	9.68	9.87	9.96	9.96	10.09	9.94	0.30	10.01	0.07	1.01	0.010	7.2E-01
Mtr.45180.1.S1_at	8.39	7.88	8.41	8.35	8.45	8.24	8.23	0.30	8.35	0.11	1.01	0.020	5.6E-01
Mtr.6706.1.S1_at	5.01	4.49	5.00	5.41	5.00	5.79	4.83	0.30	5.40	0.39	1.12	0.159	1.2E-01
Mtr.4792.1.S1_at	8.69	8.21	8.77	9.56	9.59	9.60	8.55	0.30	9.59	0.02	1.12	0.164	4.0E-03
Mtr.32402.1.S1_at	6.65	6.13	6.66	6.51	6.02	6.46	6.48	0.30	6.33	0.27	0.98	-0.034	5.6E-01
Mtr.39025.1.S1_at	9.78	9.65	10.22	9.84	9.75	9.77	9.88	0.30	9.79	0.05	0.99	-0.014	6.1E-01
Mtr.47060.1.S1_at	3.22	3.70	3.14	3.14	3.12	3.12	3.35	0.30	3.13	0.01	0.93	-0.101	2.6E-01
Mtr.36226.1.S1_at	3.59	4.05	3.48	3.50	3.41	3.66	3.71	0.30	3.52	0.12	0.95	-0.073	3.9E-01
Mtr.36418.1.S1_at	2.69	2.71	3.22	2.68	2.68	2.68	2.87	0.30	2.68	0.00	0.93	-0.100	3.3E-01
Mtr.45163.1.S1_at	9.46	9.21	9.81	9.54	9.52	9.49	9.49	0.30	9.52	0.02	1.00	0.004	8.8E-01
Mtr.19690.1.S1_at	5.54	5.53	6.05	6.78	6.87	6.35	5.70	0.30	6.67	0.28	1.17	0.226	1.5E-02
Mtr.37571.1.S1_at	8.04	7.65	8.24	8.15	8.31	8.26	7.98	0.30	8.24	0.08	1.03	0.047	2.2E-01
Mtr.7399.1.S1_at	9.85	9.29	9.38	8.59	9.04	9.04	9.50	0.30	8.89	0.26	0.94	-0.096	5.6E-02
Mtr.5247.1.S1_at	2.12	2.18	2.67	2.10	2.10	2.10	2.32	0.30	2.10	0.00	0.91	-0.144	2.7E-01
Mtr.46377.1.S1_at	2.85	3.33	2.78	2.76	2.74	2.71	2.99	0.30	2.74	0.02	0.92	-0.126	2.2E-01
Mtr.2917.1.S1_at	5.28	4.79	5.32	5.29	5.32	5.37	5.13	0.30	5.32	0.04	1.04	0.054	3.3E-01
Mtr.18818.1.S1_at	9.92	9.39	9.90	9.95	9.82	9.96	9.74	0.30	9.91	0.08	1.02	0.026	3.8E-01
Mtr.43859.1.S1_at	9.59	9.36	9.00	8.81	8.69	8.96	9.32	0.30	8.82	0.14	0.95	-0.079	5.8E-02
Mtr.34217.1.S1_at	10.18	9.83	10.42	10.50	10.39	10.49	10.14	0.30	10.46	0.06	1.03	0.044	1.5E-01
Mtr.45433.1.S1_at	8.53	8.43	9.00	8.75	8.86	8.41	8.65	0.30	8.67	0.23	1.00	0.003	9.4E-01
Mtr.36574.1.S1_at	3.10	2.73	3.33	2.92	3.16	2.94	3.05	0.30	3.01	0.13	0.99	-0.021	8.2E-01
Mtr.5128.1.S1_at	4.53	4.42	4.99	4.51	4.48	4.51	4.65	0.30	4.50	0.01	0.97	-0.047	4.4E-01
Mtr.39500.1.S1_at	10.83	10.25	10.41	10.58	10.69	10.73	10.50	0.30	10.67	0.08	1.02	0.023	4.0E-01
Mtr.3724.1.S1_at	2.20	2.29	2.76	2.18	2.92	2.23	2.42	0.30	2.45	0.42	1.01	0.017	9.3E-01
Mtr.40004.1.S1_at	10.72	10.94	11.31	11.12	11.13	10.95	10.99	0.30	11.07	0.10	1.01	0.010	7.0E-01
Mtr.39586.1.S1_at	9.53	9.89	10.13	10.10	9.92	9.87	9.85	0.30	9.96	0.12	1.01	0.017	5.8E-01
Mtr.42747.1.S1_at	7.43	6.83	7.12	7.59	7.24	7.64	7.13	0.30	7.49	0.22	1.05	0.072	1.6E-01
Mtr.95.1.S1_at	8.86	8.68	9.27	8.93	8.83	8.76	8.94	0.30	8.84	0.09	0.99	-0.015	6.3E-01
Mtr.27126.1.S1_at	9.59	9.01	9.17	9.66	9.61	9.60	9.26	0.30	9.62	0.03	1.04	0.056	1.0E-01
Mtr.18654.1.S1_at	2.85	3.35	2.82	2.78	2.80	2.79	3.01	0.30	2.79	0.01	0.93	-0.110	2.7E-01
Mtr.14634.1.S1_at	2.93	3.42	2.88	2.85	2.81	2.82	3.08	0.30	2.83	0.02	0.92	-0.123	2.2E-01
Mtr.4339.1.S1_at	7.42	7.94	7.93	7.76	7.93	7.65	7.77	0.30	7.78	0.14	1.00	0.003	9.4E-01
Mtr.45209.1.S1_at	6.53	6.51	7.04	6.22	6.10	6.74	6.69	0.30	6.36	0.34	0.95	-0.075	2.7E-01
Mtr.17550.1.S1_at	8.20	8.72	8.70	9.60	10.31	9.08	8.54	0.30	9.66	0.62	1.13	0.178	4.7E-02
Mtr.15716.1.S1_at	8.70	9.12	9.27	9.29	9.28	9.11	9.03	0.30	9.23	0.10	1.02	0.032	3.4E-01
Mtr.17347.1.S1_at	2.44	2.95	2.42	2.42	2.41	2.41	2.60	0.30	2.41	0.00	0.93	-0.110	3.3E-01
Mtr.18387.1.S1_at	9.09	8.63	9.19	8.78	9.00	8.73	8.97	0.30	8.84	0.14	0.98	-0.022	5.2E-01
Mtr.43923.1.S1_at	11.72	11.28	11.85	11.81	11.69	11.70	11.61	0.30	11.73	0.07	1.01	0.015	5.4E-01
Mtr.3674.1.S1_at	2.74	3.25	2.72	2.70	2.66	2.64	2.90	0.30	2.66	0.03	0.92	-0.124	2.4E-01
Mtr.11045.1.S1_at	8.91	9.39	9.46	8.80	8.63	8.73	9.25	0.30	8.72	0.08	0.94	-0.086	4.1E-02
Mtr.20168.1.S1_s_at	8.92	8.35	8.49	8.69	8.59	8.55	8.59	0.30	8.61	0.07	1.00	0.003	9.2E-01
Mtr.28818.1.S1_at	12.28	12.04	11.69	12.10	12.04	12.18	12.00	0.30	12.11	0.07	1.01	0.012	5.9E-01
Mtr.19571.1.S1_at	2.71	3.22	2.70	2.68	2.67	2.66	2.88	0.30	2.67	0.01	0.93	-0.106	3.1E-01
Mtr.41269.1.S1_at	12.05	11.67	12.26	12.21	11.66	12.11	12.00	0.30	11.99	0.29	1.00	0.000	9.9E-01
Mtr.27169.1.S1_at	2.96	3.45	2.91	2.89	2.81	2.87	3.10	0.30	2.86	0.04	0.92	-0.120	2.3E-01
Mtr.31668.1.S1_s_at	14.02	14.34	14.61	14.36	14.36	14.33	14.32	0.30	14.35	0.01	1.00	0.003	8.8E-01
Mtr.4611.1.S1_at	9.03	8.60	8.46	8.62	8.79	8.62	8.70	0.30	8.68	0.10	1.00	-0.003	9.1E-01
Mtr.46367.1.S1_at	11.57	11.84	12.16	11.82	11.92	11.72	11.86	0.30	11.82	0.10	1.00	-0.005	8.5E-01
Mtr.28612.1.S1_at	7.40	6.88	7.40	7.31	7.52	7.45	7.23	0.30	7.42	0.11	1.03	0.039	3.4E-01
Mtr.6104.1.S1_at	8.64	8.31	8.91	8.78	8.89	8.68	8.62	0.30	8.78	0.11	1.02	0.027	4.2E-01
Mtr.30626.1.S1_at	2.07	2.58	2.05	2.05	2.05	2.05	2.23	0.30	2.05	0.00	0.92	-0.123	3.5E-01
Mtr.19399.1.S1_at	12.78	12.24	12.72	12.85	12.91	12.88	12.58	0.30	12.88	0.03	1.02	0.034	1.6E-01
Mtr.28204.1.S1_at	10.08	9.71	10.30	10.34	10.29	10.16	10.03	0.30	10.26	0.09	1.02	0.033	2.7E-01
Mtr.34106.1.S1_at	9.71	9.16	9.64	9.37	9.52	9.52	9.51	0.30	9.47	0.08	1.00	-0.005	8.5E-01
Mtr.37710.1.S1_at	7.01	6.44	6.58	7.01	7.23	6.91	6.68	0.30	7.05	0.17	1.06	0.078	1.3E-01
Mtr.31719.1.S1_at	8.64	8.96	9.24	8.78	9.06	8.54	8.95	0.30	8.79	0.26	0.98	-0.025	5.4E-01
Mtr.9078.1.S1_at	11.74	12.28	12.23	11.82	11.68	11.79	12.08	0.30	11.76	0.07	0.97	-0.039	1.5E-01
Mtr.13709.1.S1_at	8.28	8.52	8.87	8.36	8.64	8.45	8.55	0.30	8.48	0.14	0.99	-0.013	7.2E-01
Mtr.16034.1.S1_at	9.39	9.22	9.80	9.75	9.73	9.63	9.47	0.30	9.70	0.07	1.02	0.035	2.6E-01
Mtr.3749.1.S1_at	2.03	2.57	2.09	2.09	2.09	2.09	2.23	0.30	2.09	0.00	0.94	-0.093	4.6E-01
Mtr.10026.1.S1_s_at	8.32	7.72	8.01	8.34	8.40	8.37	8.02	0.30	8.37	0.03	1.04	0.062	1.1E-01
Mtr.29506.1.S1_at	9.19	8.77	9.35	9.13	9.15	8.93	9.10	0.30	9.07	0.12	1.00	-0.005	8.7E-01
Mtr.6525.1.S1_at	5.46	4.94	5.46	5.75	5.29	5.57	5.29	0.30	5.54	0.23	1.05	0.066	3.2E-01
Mtr.5203.1.S1_at	6.70	7.22	7.21	6.53	7.50	6.85	7.04	0.30	6.96	0.49	0.99	-0.017	8.2E-01
Mtr.8592.1.S1_at	11.71	11.14	11.56	11.70	11.84	11.68	11.47	0.30	11.74	0.09	1.02	0.034	2.0E-01
Mtr.50764.1.S1_at	8.52	7.97	8.44	8.44	8.52	8.47	8.31	0.30	8.48	0.04	1.02	0.029	3.9E-01
Mtr.36902.1.S1_at	10.12	9.92	10.51	10.01	10.19	10.12	10.18	0.30	10.11	0.09	0.99	-0.011	6.9E-01
Mtr.21056.1.S1_s_at	10.61	10.01	10.30	10.21	10.41	10.34	10.30	0.30	10.32	0.10	1.00	0.002	9.3E-01
Mtr.25140.1.S1_at	3.15	3.65	3.12	3.29	3.06	2.74	3.31	0.30	3.03	0.28	0.92	-0.127	3.0E-01
Mtr.10871.1.S1_at	10.27	9.99	10.59	10.54	10.70	10.46	10.28	0.30	10.56	0.12	1.03	0.039	2.0E-01
Mtr.12081.1.S1_at	8.15	8.12	8.65	8.46	8.40	8.34	8.31	0.30	8.40	0.06	1.01	0.015	6.4E-01
Mtr.10598.1.S1_at	11.37	10.78	11.12	11.17	11.18	11.31	11.09	0.30	11.22	0.08	1.01	0.017	5.1E-01
Mtr.34205.1.S1_at	9.10	8.99	9.55	9.16	9.52	8.73	9.21	0.30	9.14	0.40	0.99	-0.012	8.0E-01
Mtr.23685.1.S1_at	8.33	7.88	8.45	8.39	8.35	8.36	8.22	0.30	8.36	0.02	1.02	0.025	4.5E-01
Mtr.14600.1.S1_at	3.41	3.77	3.17	3.28	3.60	3.15	3.45	0.30	3.34	0.23	0.97	-0.046	6.5E-01
Mtr.20775.1.S1_s_at	13.80	13.51	14.11	13.69	13.83	13.56	13.81	0.30	13.69	0.13	0.99	-0.012	5.7E-01
Mtr.1961.1.S1_at	6.17	5.81	6.41	5.97	5.95	5.58	6.13	0.30	5.83	0.22	0.95	-0.071	2.4E-01
Mtr.13793.1.S1_at	10.83	10.31	10.82	10.61	10.93	10.66	10.65	0.30	10.73	0.17	1.01	0.011	7.1E-01
Mtr.5911.1.S1_at	8.92	8.35	8.50	8.71	8.66	8.89	8.59	0.30	8.75	0.12	1.02	0.027	4.4E-01
Mtr.12541.1.S1_s_at	12.17	11.65	11.66	11.76	11.64	11.85	11.83	0.30	11.75	0.10	0.99	-0.009	7.0E-01
Mtr.16195.1.S1_at	9.93	9.36	9.81	10.25	10.45	10.34	9.70	0.30	10.35	0.10	1.07	0.094	2.3E-02
Mtr.2997.1.S1_at	8.79	8.85	9.33	9.10	9.05	9.14	8.99	0.30	9.10	0.04	1.01	0.017	5.7E-01
Mtr.43467.1.S1_at	15.00	14.86	14.43	14.65	14.89	14.73	14.77	0.30	14.76	0.12	1.00	-0.001	9.8E-01
Mtr.27917.1.S1_s_at	10.68	10.31	10.90	10.71	10.56	10.40	10.63	0.30	10.56	0.15	0.99	-0.010	7.2E-01
Mtr.35127.1.S1_s_at	10.82	10.23	10.43	10.63	10.54	10.81	10.49	0.30	10.66	0.14	1.02	0.023	4.3E-01
Mtr.12911.1.S1_at	7.59	7.12	7.67	7.74	7.66	7.29	7.46	0.30	7.56	0.24	1.01	0.020	6.7E-01
Mtr.18728.1.S1_s_at	2.49	3.01	2.49	2.49	2.48	2.48	2.66	0.30	2.48	0.01	0.93	-0.101	3.6E-01
Mtr.19431.1.S1_at	3.14	3.64	3.12	3.08	3.07	3.02	3.30	0.30	3.05	0.03	0.93	-0.111	2.3E-01
Mtr.21891.1.S1_at	12.50	13.02	13.01	12.65	12.73	12.64	12.84	0.30	12.68	0.05	0.99	-0.019	4.0E-01
Mtr.21392.1.S1_at	2.57	3.06	2.52	2.53	2.50	2.49	2.72	0.30	2.50	0.02	0.92	-0.117	2.9E-01
Mtr.29047.1.S1_at	10.60	11.07	11.15	10.71	10.66	10.73	10.94	0.30	10.70	0.04	0.98	-0.031	2.5E-01
Mtr.11013.1.S1_at	6.91	6.53	7.11	6.32	6.06	6.11	6.85	0.30	6.16	0.14	0.90	-0.152	2.3E-02
Mtr.13099.1.S1_at	11.52	10.98	11.04	11.43	11.37	11.57	11.18	0.30	11.46	0.10	1.02	0.035	2.0E-01
Mtr.18601.1.S1_at	8.81	8.25	8.73	8.45	8.54	8.55	8.60	0.30	8.51	0.06	0.99	-0.014	6.7E-01
Mtr.22693.1.S1_s_at	1.95	1.99	2.48	2.10	2.00	2.03	2.14	0.30	2.04	0.05	0.95	-0.067	6.1E-01
Mtr.44734.1.S1_at	8.70	8.14	8.24	8.40	8.40	8.69	8.36	0.30	8.49	0.17	1.02	0.023	5.3E-01
Mtr.26758.1.S1_at	2.79	3.30	2.77	2.76	2.75	2.74	2.95	0.30	2.75	0.01	0.93	-0.104	3.0E-01
Mtr.29959.1.S1_at	10.23	10.52	10.83	10.56	10.82	10.65	10.53	0.30	10.67	0.13	1.01	0.020	4.8E-01
Mtr.24106.1.S1_s_at	10.28	9.73	10.21	10.20	10.08	10.25	10.07	0.30	10.17	0.09	1.01	0.014	6.1E-01
Mtr.30757.1.S1_at	10.37	9.94	10.51	10.45	10.40	10.26	10.27	0.30	10.37	0.10	1.01	0.014	6.1E-01
[truncated: 4,096,203 more chars]
